# Lateral Variation of Crustal Lg Attenuation in Eastern North America

**DOI:** 10.1038/s41598-018-25649-5

**Published:** 2018-05-08

**Authors:** Lian-Feng Zhao, S. Mostafa Mousavi

**Affiliations:** 1 Key Laboratory of Earth and Planetary Physics, Institute of Geology and Geophysics, Chinese Academy of Sciences, Beijing, 100029 P.R. China; 2 Department of Geophysics, Stanford University, Stanford, CA 94305 USA

## Abstract

We perform *Q*_*Lg*_ tomography for the northeastern part of North America. Vertical broadband seismograms of 473 crustal earthquakes recorded by 302 stations are processed to extract the *Lg* amplitude spectra. Tomographic inversions are independently conducted at 58 discrete frequencies distributed evenly in log space between 0.1 and 20.0 Hz. This relatively large dataset with good ray coverage allows us to image lateral variation of the crustal attenuation over the region. Obtained *Q*_*Lg*_ maps at broadband and individual frequencies provide new insights into the crustal attenuation of the region and its relationship to geological structures and past tectonic activity in the area. The *Q*_*Lg*_ shows more uniform values over the older, colder, and drier Canadian Shield, in contrast to higher variations in the younger margins. Results confirm the correlation of large-scale variations with crustal geological features in the area. Existence of low-velocity anomalies, thick sediments, volcanic rocks, and thin oceanic crust are potential sources of observed anomalies. The mean *Q* values are inversely correlated with average heat flow/generation for main geological provinces.

## Introduction

The *Lg*, consisting of multiple reflected shear waves trapped within the crust, is often the most prominent seismic energy on regional seismograms. *Lg* provides a measure of depth-averaged crustal properties because it is not associated with displacements propagating along a well-defined ray path. It has relatively high-frequency content and its amplitude is not affected by the radiation pattern of the source, while it is highly sensitive to the crustal thickness and heterogeneities^1^. These characteristics make *Lg* a suitable tool to study apparent attenuation and map the crustal structures. Hence, it has been extensively used by seismologists for attenuation estimation within different tectonic regimes^2–34^.

**Figure 1:**
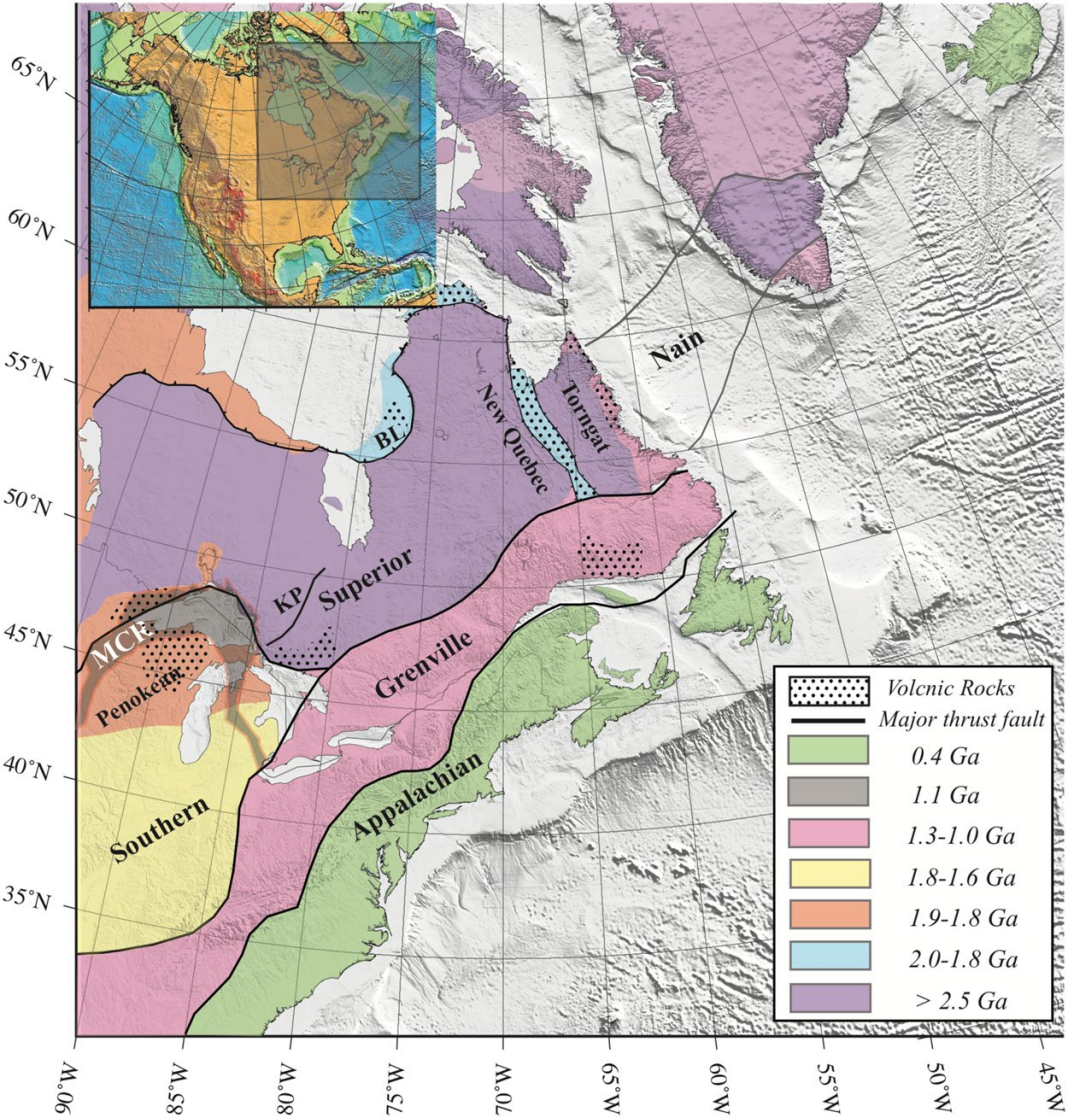
The essential geological blocks of eastern Canada. The Mid-Continent Rift (MCR) is shown by the gray shaded area. Solid black lines show boundaries of geological blocks. Abbreviations are Belcher thrust-fold belts (BL) and Kapuskasing Uplift (KP). Numbers are associated to the locations of Q anomalies in the tomography results. The figure is generated from GMT, which is an open source software available at: http://gmt.soest.hawaaii.edu.

The study area of this paper includes most of eastern Canada and adjacent areas in the northeastern United States (Fig. 1). Some large and damaging earthquakes have occurred within this area, including the M7 1929 Grand Banks and M7.3 1663 Charlevoix earthquakes. The tectonic stress field in the region has a compressive pattern with the maximum horizontal axis in NE–SW orientation^35^ due to far-field plate-tectonic forces (especially ridge push) and/or from geoid perturbations and mantle thermal anomalies^36^.

In this region, the *Lg* is the dominant seismic energy for the earthquakes with an epicentral distance greater than about two crustal thicknesses^37^. The Canadian National Data Center (CNDC) reported that *Lg* has been the main source of damage during large earthquakes in the Eastern Canada, due to the large amplitude, long durations and relatively low attenuation of *Lg* in this region.

In this study, we map spatial variation of the *Q*_*Lg*_ for eastern North America using a dataset of 473 earthquakes occurring between January 1990 and January 2017. This relatively large dataset offers an opportunity to study the variation of *Q*_*Lg*_ across the region. The sensitivity of *Q*_*Lg*_ to the crustal temperature, composition, and heterogeneities makes it a valuable tool to improve our understanding of the crustal properties underlying eastern North America. We investigate the potential correlation of *Q*_*Lg*_ variation with crustal and upper mantle heterogeneities. This is the first time that a *Q* tomography study using a relatively big dataset and wide frequency range is performed over this area. This article provides new insights into crustal attenuation and heterogeneities across the region. The result of this study can be used for earthquake monitoring, magnitude calibration, and seismic hazard assessment.

## Tectonic Overview

This region consists of the exposed part of the world’s largest craton, the North American craton, and is composed of very old (4 billion to 1 billion years old) Archean and Proterozoic rocks^38^. Geological provinces and subprovinces that originated separately as small microcontinents and remnants of ocean-floor crust were welded together by plate tectonics processes to form the craton (Fig. 1).

Eastern North America is complicated in terms of its geological history. It has experienced two complete successions of Wilson cycles – the opening of the Iapetus Ocean around 600 million years ago and the opening of the Atlantic Ocean around 200 million years ago^39^. The Superior province is the oldest building block in this region. This geological province is composed of several distinct terranes with origin dates as early as 3.8 billion years ago^40^. The Superior craton was assembled from these terranes in 2.72 to 2.68 billion years ago^41^ during the Archean era.

The Southern Province had been added to the Superior by about 1.9 billion years ago. Then the Grenville province (also called Grenville Orogenic belt) was formed because of a continental collision during the assembly of supercontinent Rodinia between 1.1 to 0.9 billion years ago. The Grenville consists predominantly of banded gneisses, highly metamorphosed sediments, and igneous rocks^42^. Rodinia started breaking up around 750 to 570 million years ago, forming Iapetus Ocean. The Iapetus started closing in 475 million years ago, forming supercontinent Pangea 40 million years later. The Appalachian is composed of highly deformed Paleozoic sediments and was formed by buckling of the outer margins of the North American craton during the closure of the Iapetus Ocean^43^.

On average, the lithosphere of the Archean portion of the craton is 200 km thick, while the Paleozoic part averages 175 km thick^44^. Yuan and Romanowicz^45^ discovered two distinct lithospheric layers under the North American craton. The top layer that corresponds to the ancient highly depleted Archean lithosphere is thicker in the oldest part of the craton and thins towards the surrounding younger Paleozoic provinces. This layer is thinnest near the Mid-Continent Rift (MCR), suggesting that the original Archean lithosphere may have been perturbed subsequently by rifting^45^. The lower layer represents a younger, less depleted, thermal boundary layer.

Other important geological features in this region are the MCR (also known as Keweenawan Rift), Kapuskasing Uplift, New Quebec, Torngat, and Penokean orogenies. MCR is a 3000-km-long failed rift system, formed when the North American craton began to split apart (about 1.1 billion years ago) during the assembly of Rodinia^46^. The MCR, a large igneous province, is a region of extensive volcanism associated with upwelling and melting of deep mantle materials^47^. The dense volcanic material is responsible for a major gravity anomaly^48^. The presence of a significant mantle plume contributing to the MCR basalts implies a thinned lithosphere underneath the rift.

The Kapuskasing Uplift is a 500-km long zone of granulite and upper-amphibolite-facies rocks that transects the central Superior province. The sedimentary-volcanic belt of New Quebec (Labrador Trough) orogeny is located at the northeast margin of the Superior province. New Quebec is an 800-km-long thrust-fold belt. This orogeny was formed because of the Early Proterozoic collision between the Archean Superior and Rae provinces, while the Torngat orogeny on the western side is the zone of intense deformation formed as the result of the collision between the Rae and Nain provinces in the early Proterozoic. The Torngat orogeny is possibly younger than 1.81 Ga^38^. The Penokean orogeny at the southern edge of the Superior is a 1.90–1.83 Ga orogeny, crossed by the younger (1.1 Ga) MCR^46,48^.

## Data and Pre-Processing

This study initially processed more than 100,000 high-gain vertical seismograms within the region recorded during the time-period January 1990 to January 2017. 1000 sec long vertical seismograms recorded on 642 stations within 1.5° to 30° epicentral distances have been automatically retrieved from IRIS data management center. Based on the catalog of events, 17-minutes long seismograms (~5 minutes before and 12 minutes after onset time) were cut from continuous data recorded by all stations in operation at the time of each event. Since the earthquake signals were not visible at all stations, we first used the STA/LTA technique^49^ to distinguish traces containing earthquake signals from those recording just noise. Next, high-quality traces with a signal to noise ration (SNR) of equal or greater than two were used for further processes. All traces have sampling rates of 40 points per seconds and higher and have been checked and corrected for gaps and spikes. After removing the trends and mean, instrument responses were deconvolved using the causal correction method^50^.

Because the seismic signal and background noise are superimposed in the recorded seismograms, studies based on the measurement of observed amplitudes at different distances, such as attenuation estimation, can suffer from varying noise levels at different stations. To address this problem, Zhao *et al*.^51^ proposed an approach to correct the amplitude of *Lg*-displacement in the frequency domain and reduce uncertainties in the *Q* estimation. However, in this study, we used time-frequency denoising techniques^52–55^ to decrease the effect of background noise. These methods are data driven and automatically remove the noise from the entire seismogram and frequency bands. These methods assume that the seismic denoising can be treated as a non-linear nonparametric regression problem and use characteristics of sparse time-frequency transform to estimate the underlying regression function (seismic signal) from noisy observations. In these algorithms, noisy data is first transferred into the time-frequency domain. Then time-frequency coefficients in a pre-signal noise section (a few tens of seconds to a few minutes before the first arrival) are used to estimate noise level and find an optimal threshold level. Here, we applied wavelet transform using Morlet mother wavelet and to further constrain the internal arrival time estimation and selection of pre-signal noise we use an algorithm proposed by Kalkan^56^. Next, time-frequency coefficients are thresholded and inverse transformed into the time domain to reconstruct denoised signals.

**Figure 2:**
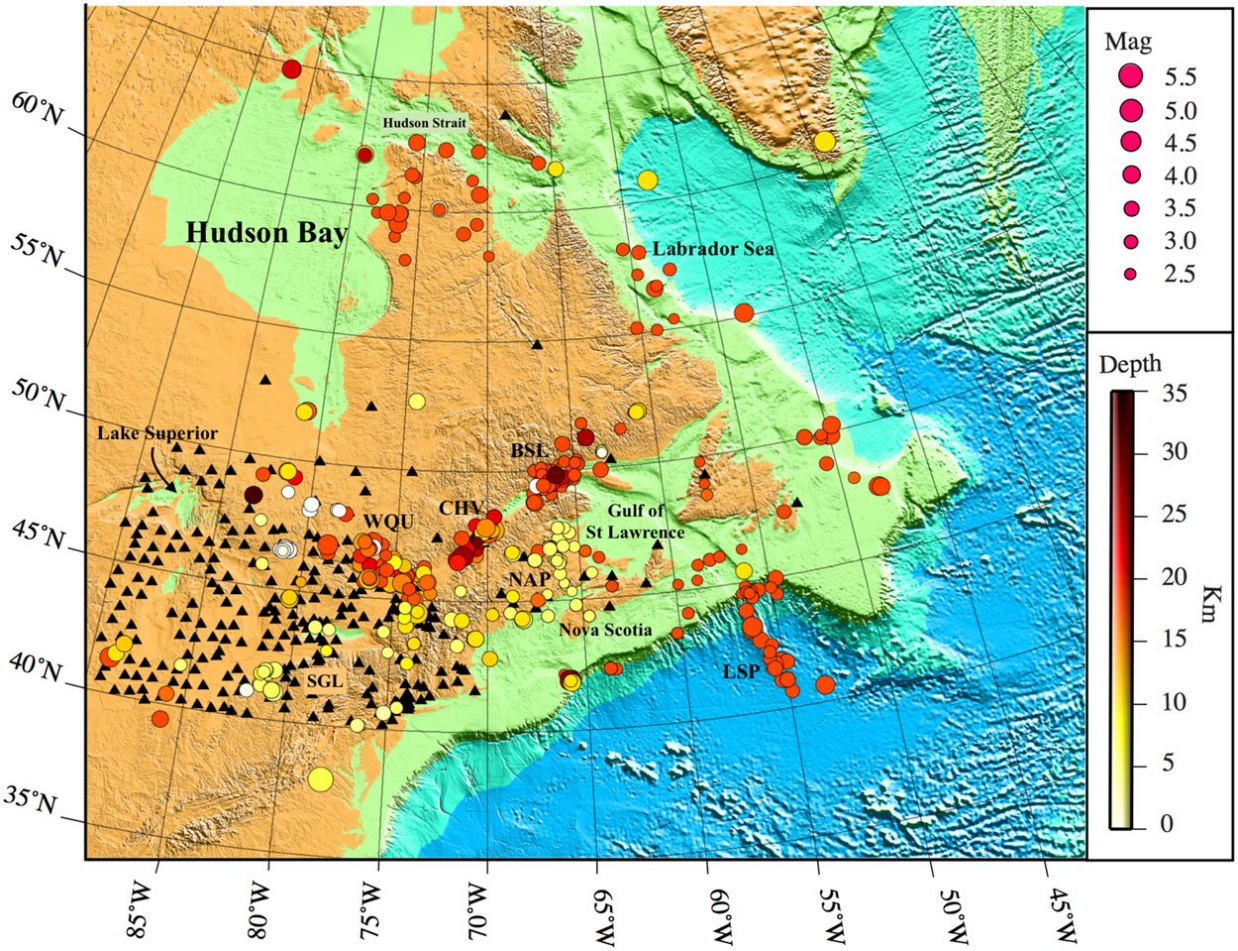
Distribution of events (circles) and stations (black triangles) used for the Q estimation. Circles are scaled based on earthquake magnitudes and color coded based on event depths. Major seismic zones in the region are marked on the map: Southern Great Lakes Seismic zone (SGL), Western Quebec Seismic Zone (WQU), Charlevoix Seismic Zone (CHV), Lower St. Lawrence seismic Zone (BSL), Northern Appalachians Seismic Zone (NAP), and Laurentian Slope Seismic Zone (LSP). The figure is generated from GMT, which is an open source software available at: http://gmt.soest.hawaaii.edu.

**Figure 3:**
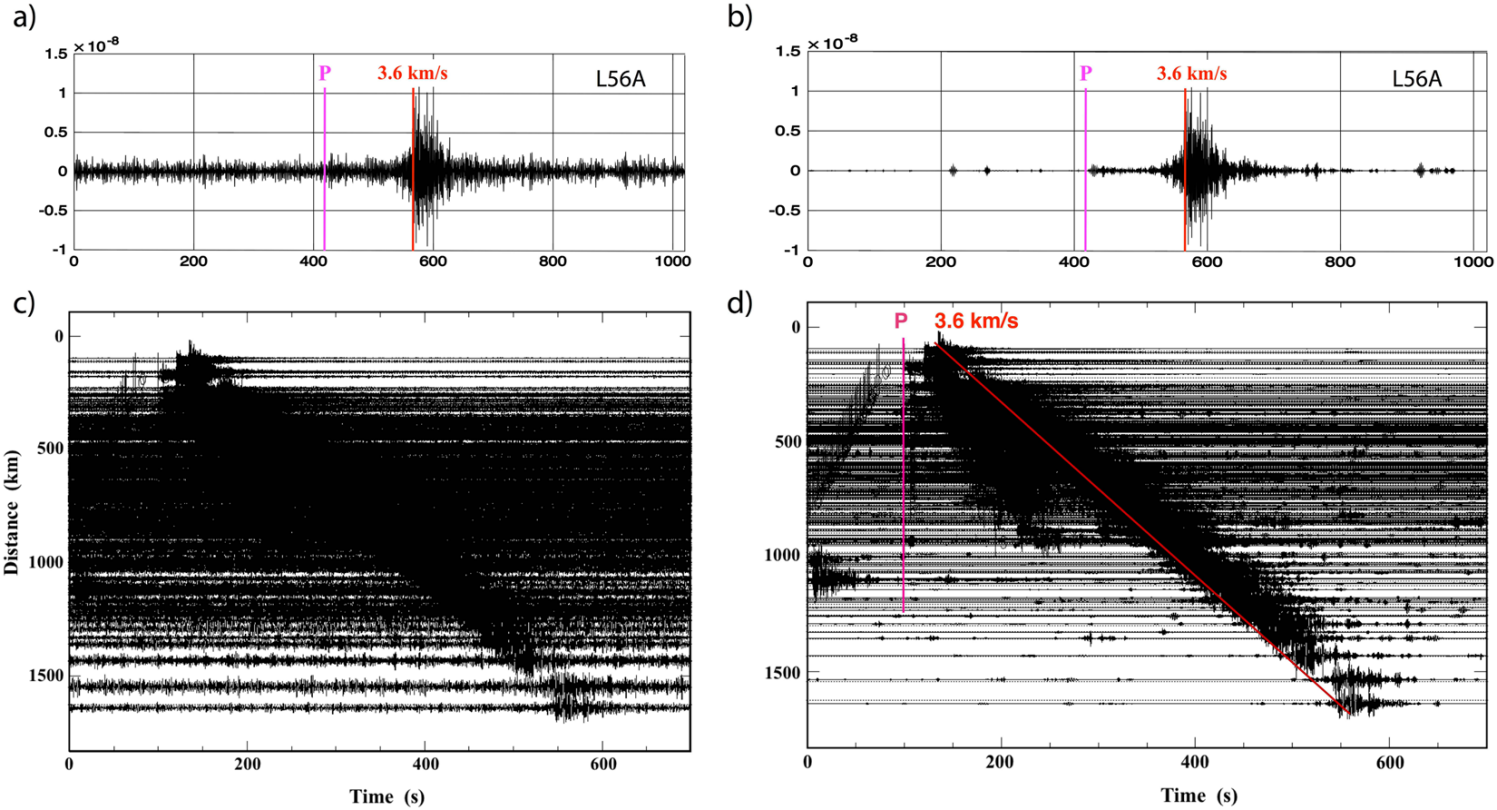
A sample seismogram (top) and associated plot record sections (bottom) from a M 3.5 event that occurred in July 2015 to the south of Nova Scotia, before (**a**) and after (**b**) the denoising. Associated record sections are presented in (**c**,**d**). P arrival and group velocity of 3.6 km/s are marked on the denoised record sections.

The final dataset used for the tomographic inversion consists of approximately 20,000 high-quality waveforms recorded from 473 events on 302 broadband stations. All earthquakes are shallower than 36 km and have magnitudes larger than 2.5. Figure 2 presents the distribution of the associated events and stations. Figure 3 shows the plot record section of seismograms from one sample event before and after denoising.

After pre-processing all waveforms, the single- and two-station amplitudes were measured at discrete frequencies between 0.1 and 20.0 Hz. A group-velocity window of 3.6–3.0 km/s is used for the *Lg* extraction. The joint inversion is performed for individual frequencies.

## Lg-wave Q Tomography

The attenuation of *Lg*-wave displacement is derived from the decay of spectral amplitude with epicentral distance (Δ), as a function of frequency *f*, based on the following relation^57^

1 $${A}_{ki}(f,{\rm{\Delta }})={G}_{ki}({\rm{\Delta }}){\rm{\Gamma }}(f,{\rm{\Delta }}){S}_{k}(f){P}_{i}(f){R}_{ki}(f),$$

in which *A*_*ki*_ is the *Lg*-displacement spectral amplitude for event *k* observed at station *i*. ${G}_{ki}({\rm{\Delta }})$ is the geometrical spreading factor, ${\rm{\Gamma }}(f,{\rm{\Delta }})$ represents the anelastic attenuation, ${S}_{k}(f)$ is the source term, ${P}_{i}(f)$ is the site amplification term, and ${R}_{ki}(f)$ is the cumulative effects of other minor factors along the propagation path and computational errors.

${G}_{ki}({\rm{\Delta }})={({{\rm{\Delta }}}_{0}{\rm{\Delta }})}^{-\gamma }$ is independent of frequency with a reference distance of ${{\rm{\Delta }}}_{0}=100\,km$^58^. The geometrical decay rate $\gamma$ is known to be 0.5 for the epicentral distance ranges used in this study $({\rm{\Delta }}\ge 1.5^\circ )$, based on observational studies in eastern Canada^59,60^.

The attenuation factor in Equation (1) is expressed by:

2 $${\rm{\Gamma }}(f,{\rm{\Delta }})=\exp (\frac{-\pi f}{\nu }\,{\int }_{k}^{i}\frac{ds}{Q(x,y,f)}),$$

where $\nu$ is the average group velocity of *Lg* (3.6 km/s)^61^, ${\int }_{k}^{i}ds$ is the integral along the great circle path from event *k* to station *i*, and $Q(x,y,f)$ is the *Lg*-wave apparent/effective quality factor. If we ignore the effects of random error term ${R}_{ki}(f)$, we can write the tomographic *Q* inversion based on Equations (1) and (2) as^62^

3 $$\begin{array}{c}\mathrm{log}\,{A}_{ki}(f,{\rm{\Delta }})+0.5\,\mathrm{log}(100{\rm{\Delta }})-\,\mathrm{log}\,{S}_{k}^{0}(f)-\,\mathrm{log}\,{{\rm{\Gamma }}}^{0}(f,{\rm{\Delta }})-\,\mathrm{log}\,{P}_{i}^{0}(f)\\ \,\,\,\,\,\,=\,\delta \,\mathrm{log}\,{S}_{k}(f)-\frac{\pi f}{\nu }{\int }_{k}^{i}\frac{\delta Q(x,y,f)}{{Q}^{0}{(x,y,f)}^{2}}ds+\delta \,\mathrm{log}\,{P}_{i}(f),\end{array}$$

Using this relation, the perturbations of the source $\mathrm{log}\,{S}_{k}(f)$, quality factor $\delta Q(x,y,f)$, and site term $\delta \mathrm{log}\,{P}_{i}(f)$ are inverted in an iterative scheme from observed spectral amplitudes, assumed geometrical spreading, and the source, *Q* values and site term from initial models or previous iterations (denoted by superscript ^0^). Following^20^ we constrain the site terms by assuming ${\sum }_{l=1}\mathrm{log}\,{P}_{l}(f)=0$.

To deal with the tradeoff between the source and attenuation terms and further constrain the model, the initial *Q*_*Lg*_ is obtained using the two-station approach^7,21,22,57^, which improves the reliability of the estimated *Q* by removing the source term from the inversion model. In this method, the interstation amplitude is calculated from observed amplitudes of one event at two stations that are approximately aligned with each other and the earthquake epicenter^29,57,62^:

4 $${A}_{lj}\approx \frac{{A}_{kj}}{{A}_{ki}}={(\frac{{{\rm{\Delta }}}_{kj}}{{{\rm{\Delta }}}_{ki}})}^{-\frac{1}{2}}exp[\frac{-\pi f}{\nu }({\int }_{l}^{i}\frac{ds}{Q(x,y,f)})](\frac{{P}_{j}}{{P}_{i}}),$$

where ${A}_{kj}$ and ${A}_{ki}$ are the spectral amplitudes recorded at stations *j* and *i* from event *k* respectively, and *l* is a reference point on the ray-path *kj* (see^63^ for more details).

Two systems of linear equations can be set up for single- and two-station data based on Equations (3) and (4) respectively:

5 $${H}_{s}={{\boldsymbol{A}}}_{{\boldsymbol{s}}}.\delta Q+{\boldsymbol{E}}.\delta U+{{\boldsymbol{F}}}_{{\boldsymbol{s}}}.\delta P,$$

and

6 $${H}_{t}={{\boldsymbol{A}}}_{{\boldsymbol{t}}}.\delta Q+{{\boldsymbol{F}}}_{{\boldsymbol{t}}}.\delta P,$$

where $\delta Q$ is a vector composed of the *Q*-perturbations, $\delta U$ is a vector composed of the source-term perturbations, Matrix ***E*** sets up the relationship between the source-perturbations and the observed *Lg*-wave amplitudes, *δP* is a vector for the site-term perturbations, Matrix ***F*** is a bridge linking the site-perturbations with the observations, ***A***_***s***_ and ***A***_***t***_ are matrices setting up the relations between *Q*-perturbations and the *Lg*-wave spectral amplitudes based on single- and two-station relations data, respectively. *H*_*s*_ and *H*_*t*_ are vectors composed of residuals between the observed and synthetic *Lg*-spectra.

Zhao *et al*.^63^ proposed a joint tomography approach by combining both dual- and single-station data to further reduce the attenuation-source tradeoffs and improve the resolution at high frequencies as:

7 $$[\begin{array}{c}{H}_{s}\\ {H}_{t}\end{array}]=[\begin{array}{c}{A}_{s}\\ {A}_{t}\end{array}].\delta Q+[\begin{array}{c}E\\ 0\end{array}].\delta U+[\begin{array}{c}{F}_{s}\\ {F}_{t}\end{array}].\delta P.$$

Based on the given assumption that the sum of all site responses is zero, we add an equation as

8a $$[0]=[1].\delta P,$$

and simultaneously controlling a relative variation of the site responses, we add:

8b $$[\varepsilon ]=[1].|\delta P|,$$

where ε is an empirical value for normalizing the site responses.

The initial *Q*_*Lg*_ is obtained by linear regression of two-station data; a unit source function is assumed as the initial source model. The LSQR algorithm with regularization, damping, and smoothing is used to solve the linear systems^16^. The damping is designed with a function of iterations as ${\lambda }_{k}={\lambda }_{0}{\alpha }^{(k-1)}$, where *λ*_0_ is the initial optimal damping coefficient, *α* is the attenuation coefficient, usually ranging from 0.5 to 0.9 and *k* is the iterative number. Here a smoothing with 5 points (a 2^nd^ order differential function) was selected for the smoothing. Perturbations *δQ* and *δU* are simultaneously inverted at individual frequencies by minimizing ${[{H}_{s}{H}_{t}0\varepsilon ]}^{T}$. This hybrid approach has been successfully applied to map spatial variation of *Q*_*Lg*_ in North China^63^, Tibetan Plateau^64^, and the Middle East^65^.

## Results

**Figure 4:**
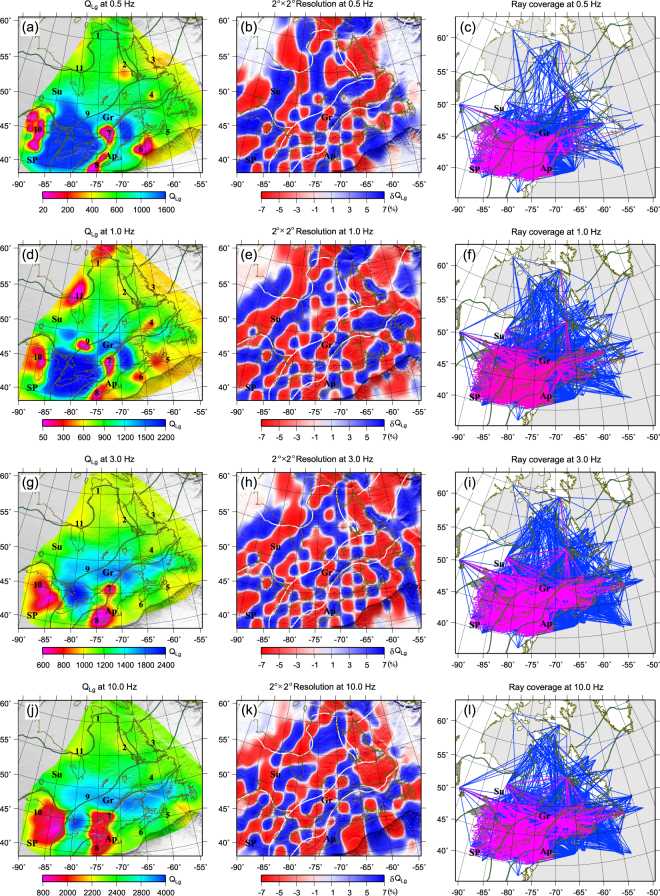
Lateral variation of *Q*_*Lg*_ (left), resolution analysis at 2° × 2°(center), and associated ray-path coverage (right) at 0.5 Hz (**a**–**c**), 1.0 Hz (**d**–**f**), 3.0 Hz (**g**–**i**), and 10.0 Hz (**j**–**l**). Note that different color scales are used for the maps. Numbers are associated to the locations of Q anomalies in the tomography results. Major geological provinces are indicated as; Superior (Su), Grenville (Gr), Appalachian (Ap), and Southern (SP). In c, f, and i, the blue lines are single-station paths, whereas the pink lines are two-station paths. The figure is generated from GMT, which is an open source software available at: http://gmt.soest.hawaaii.edu.

Using the aforementioned inversion procedure, we obtained the crustal attenuation model of the region, consisting of *Q*_*Lg*_ variations at 58 individual frequencies between 0.1 to 20 Hz. *Q*_*Lg*_ maps at three selected frequencies (0.5 Hz, 1.0 Hz, and 3.0 Hz) and associated checkerboard resolution analysis^63^ and ray coverage are presented in Fig. 4. Ray density is very high over most of the region. As can be seen from Fig. 4c,f,i, most of the area is covered by crossing ray paths providing relatively good azimuthal coverage. Among these frequencies, ray path coverage at frequencies lower than 1 Hz, is poor in western Superior Province. For the areas with better coverage of the two-station paths, the inverted Q would be independent of the input model and have certain values; whereas, for the regions covered with fewer two-station paths and more single-station paths, the uncertainty would be larger due to the possible source-Q and site-Q tradeoffs. The checkerboard resolution analysis shows that the maximum spatial resolution is higher than 2° in well-covered areas and for frequencies between 1.0 and 10.0 Hz.

From these maps, we see that *Q* values increase with frequency. The Canadian Shield (Superior province plus northern Grenville) is characterized by broad, uniformed low attenuation, while younger marginal areas that have experienced more intense deformation during past tectonic processes exhibit greater lateral variation and higher attenuation.

Low-*Q*-anomalies (high attenuation) are observed at all frequencies in the New Quebec orogeny (anomaly #2 on the map with Q_0_ ~= 600), coastal area of Labrador Sea (anomaly #3 on the map with Q_0_ ~= 550), northwest of Hudson Strait (anomaly #1 on the map with Q_0_ ~= 300), Belcher belt southeast of Hudson Bay (anomaly #11 on the map with Q_0_ ~= 100–200), Wisconsin area south of Lake Superior (anomaly #10 on the map with Q_0_ ~= 100–400), southwest of CHV (anomaly #7 on the map with Q_0_ ~= 100), southeast of KP (anomaly #9 on the map with Q_0_ ~= 100), southeast of SGL including the New York metropolitan area and Delaware Valley (anomaly #8 on the map with Q_0_ ~= 100), southeast and northeast of Nova Scotia (anomalies #6 & #5 on the map with Q_0_ ~= 300), and north of St. Lawrence Gulf (anomaly #4 on the map with Q_0_ ~= 400). In contrast, the Great Lake Basin (anomaly #13 on the map with Q_0_ ~= 2000) and NAP (including the state of Maine) (anomaly #14 on the map with Q_0_ ~= 1500), exhibit high-*Q*-values (low attenuation).

**Figure 5:**
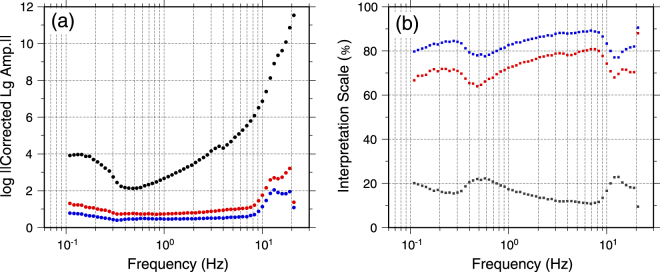
(**a**) All 58 corrected Lg amplitudes at individual frequencies, including the amplitudes corrected by the geometric spreading functions (black dots), the residuals removing both the source term and path Q (red dots), and final residuals removing the source term, Q and site response (blue dots). (**b**) The interpretation scales at 58 frequencies. Interpretation scale is the ratio of estimated amplitude to observed amplitude.

The uncertainty in the tomography can be due to two sources. First, the final residuals, which could be used for measuring how many observed Lg amplitudes were not interpreted. The second issue is whether the observations were properly spread out between the source, Q and site terms, which usually depends on the survey system with the given ray coverage, including single-station and two-station paths, as well as some constraints for source^63^, and site^20^ terms. In Fig. 5, the residuals and interpretation scales at 58 individual frequencies are provided. This plot shows that ~70% amplitudes (red dots) are interpreted by joint inversion including the source and Q, and ~80% (blue dots) can be explained if additionally considering site response. The residuals are relatively lower between 1 Hz to 10 Hz and reach ~20% which could be the random effects.

**Figure 6:**
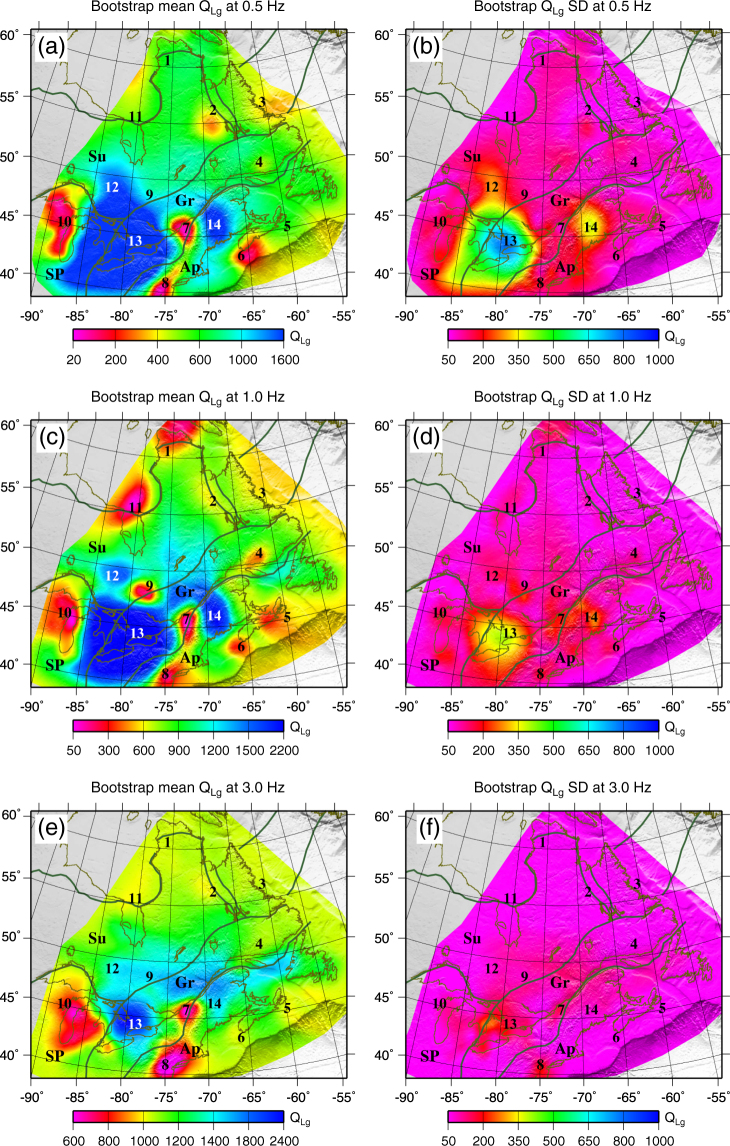
The mean Q distribution from Bootstrapping (left) and associated standard deviations (on the right). The figure is generated from GMT, which is an open source software available at: http://gmt.soest.hawaaii.edu.

We also examined the mean and standard deviation of Q for all frequencies by resampling the original dataset using the bootstrapping technique. To obtain an error estimate, we randomly selected 85% of raypaths from the total number of observations to create 100 new bootstrap datasets and then inverted each bootstrap dataset to determine 100 Q values at each point. From these 100 Q values, we calculated a standard deviation and mean value for the region at each frequency. The directly inverted Q (Fig. 4) and Bootstrap Mean Q (Fig. 6) agree very well which indicate the stability of the results. The standard deviation is larger at very low (<1.0 Hz) and high (>13.0 Hz) frequencies. These agree with previous observations in the region^61^. High uncertainties at low frequencies can be due to the presence of microseismic noise and poor signals of the small events. While high uncertainties at high frequencies can be due to the stronger attenuation of Lg at higher frequencies or/and contamination of Lg window by the Sn coda.

## Discussion

### Comparison with Previous Studies

In this section, we summarize the many studies that have been conducted to estimate crustal attenuation within this region using different approaches^3,4,11,14,25,34,37,58–61,66–71^, and compare their attenuation estimates to our results.

Singh and Herrmann^66^ conducted one of the earliest works addressing the crustal attenuation in southeast Canada, studying the coda attenuation of 250 local and near regional earthquakes with magnitudes between 3.0 and 5.0. They determined a crustal *Q*_0_ (*Q* at 1.0 Hz) value of 700 to 900. Atkinson^71^ estimated the average *Q*_*Lg*_ = 540 *f* ^0.41^ in southeastern Canada for the frequency range 0.5–10 Hz. However, Atkinson and Mereu^4^ proposed higher ${Q}_{Lg}=670\,{f}^{0.33}$ for southeastern Canada by analyzing approximately 1000 digital short-period vertical accelerograms; this is close to the estimated values in our study. Benz *et al*.^11^ estimated the frequency dependent *Q*_*Lg*_ over the frequency band of 1.5 to 14.0 Hz to be $\,{Q}_{Lg}=1052{(f/1.5)}^{0.22}$ for southeastern Canada, which is within the range of *Q* values for this region in our model. However, they did not find any significant differences in *Lg* attenuation between the central United States and the northeastern United States and southeastern Canada. Atkinson^70^ investigated the empirical attenuation of ground-motion spectral amplitudes in southeast Canada using 186 events with a magnitude range of 2.5 to 5.6 MN. She used regression analysis to determine the shape and level of attenuation of Fourier spectral amplitudes for the shear waves in both vertical and horizontal components to calculate the quality factor for the frequency range of 0.2 to 20 Hz. She estimated ${Q}_{S}=893\,{f}^{0.32}$ for frequencies greater than 1 Hz and suggested that *Q* can be better modeled over a wider frequency range with a polynomial expression. She included records from stations out to 400 km for M ≥ 3.0, out to 800 km for M ≥ 3.6, and out to 2000 km for M ≥ 4.2. Mousavi *et al*.^61^ studied the average *Q*_*Lg*_ and *Q*_*Sn*_ within the frequency range of 0.9 to 10.75 Hz for easternmost Canada. They used records of 91 events with epicentral distances of 100 to 1200 km and magnitude ranges of 2.5 ≤ M ≤ 4.7 and estimated ${Q}_{Lg}=615\,{f}^{0.35}$ for the vertical components. Moreover, they observed the *Lg* blockage and a strong tradeoff between *Lg* and *Sn* amplitude at coastal areas of Nova Scotia.

Hasegawa^37^ obtained the *Q*_*Lg*_ = 900 *f* ^0.2^ for the Canadian Shield, which is very close to estimated values for this region from this study. Hasegawa^37^ used the Fourier amplitude density of ground acceleration to estimate the attenuation of *Lg* from vertical seismograms of 54 events with epicentral distances ranging from 70 to 900 km. The spectral amplitudes were measured over the frequency band of 0.6 to 20 Hz. Shin and Herrmann^67^ used data from the 1982 Miramichi earthquake in central New Brunswick to estimate the attenuation of *Lg* for the epicentral range of 135 to 994 km in both time and frequency domains. Their measurements were limited to the frequency range of 0.5 to 15.0 Hz. The estimated *Q*_*Lg*_ for eastern Canada rises from 300 at 0.5 Hz to above 1400 at 10 Hz and is modeled by ${Q}_{Lg}=(500\,\,to\,550)\,{f}^{0.65}$ for frequencies up to 7 Hz and distances less than 600 km. Chun *et al*.^3^ measured the attenuation of *Lg* spectral amplitudes in frequencies between 0.6 to 10 Hz using velocity seismograms of 21 events with epicentral distances ranging from 90 to 867 km. Using the reversed two-station method, they calculated the *Lg* attenuation coefficient for the Grenville Province to be γ(f) = 0.0008 *f* ^0.81^.

Shi *et al*.^68^ estimated corner frequencies of small (2.2 ≤ M ≤ 3.8) earthquakes from *Lg* windows observed on vertical seismograms, then estimated *Q* for the northeastern United States by fitting the resulting source spectra to the observed spectra. Earthquake data used in their study had a magnitude range of 1.5 to 4.1 *m*_*bLg*_ and epicentral distance range of 41 to 1394 km. They measured the attenuation in frequency bands of 0.5–16 Hz and 1–30 Hz. They estimated a low *Lg* attenuation with ${Q}_{Lg}=905\,{f}^{0.40}$ in the Adirondack Mountains with exposed Precambrian Grenville basement, but found higher *Lg* attenuation in the central Appalachian Province with ${Q}_{Lg}=561-721{f}^{0.46-0.47}$. These numbers agree with the range of *Q* values at 1 Hz obtained in our study; we also determined lower *Q* for the Appalachian compared to the Grenville. Erickson *et al*.^69^ analyzed a set of horizontal recordings obtained at 12 broadband stations from 12 earthquakes (3.5 ≤ M ≤ 5.0) ranging in distance from 110 to 890 km. The $\,{Q}_{Lg}=650\,{f}^{0.36}$ was determined for the northeastern United States from time-domain amplitude measurements in a series of narrow frequency bands between 0.75 to 12 Hz.

Pasyanos^25^ provided the attenuation models of crustal and upper mantle P and S waves in the North America for a 2–4 Hz passband. The high-*Q* anomalies in the eastern Great Lakes area and the low-*Q* anomaly south of Lake Superior that we observed in our study have been mapped^25^. He also estimated lower attenuation values for the cratonic Canadian Shield, compared to the younger surrounding areas. Gallegos *et al*.^34^ performed a *Q*-tomography study using USArray data from 39 events (occurring between 2010 to 2012) using the two-station and the reverse two-station method, and provided 1.0-Hz-*Q* models for the central and eastern United States. However, since their study did not cover our study area, it is difficult to provide a comparison. Mitchell *et al*.^14^, who performed the latest attenuation study over the entire continent of North America, mapped the *Lg* coda *Q*_0_ at 1 Hz. Mitchell *et al*.^14^ obtained higher *Q*_*Lg*_ over the Canadian Shield, in agreement with our results. Their model also identified relatively high *Q* in the eastern Great Lakes area and lower *Q* south of Lake Superior, consistent with our estimates.

In summary, estimates of crustal *Q* from previous studies in this region have a frequency dependent value of 0.19 to 0.65 and *Q*_0_ varying from 525 to 1100 (Table 1), which are within the range of our results (Fig. 4). This variation in the previous results presumably comes from differences in the specific regions being analyzed, earthquake data with different magnitude and epicentral distance ranges, different frequency ranges, and/or the different methods used for *Q* estimation. The large dataset used in this study provides much higher ray path coverage, resulting in higher resolution of the model. We confirm the reliability of our results by comparing the range of our obtained *Q* values and verifying dominant anomalies with values from previous studies. In addition, finer attenuation structure obtained in this study can help improve our understanding of crustal structure of this region.

**Table 1:** Previous attenuation studies at the region.

Reference	Q(f)	Wave type	Number of events	Frequency Band (Hz)	Region
Singh & Herrmann^66^	*Q*_0_ = 700–900	Coda	250	0.5–3.5	Costal East and Northeast U.S
Hasegawa^37^	*Q*_*Lg*_ = 900 *f* ^0.20^	Lg	54	0.6–20	Canadian Shield
Shin & Herrmann^67^	*Q*_*Lg*_ = (500–550) *f* ^0^	Lg	1	0.5–7	Southeastern Canada
Chun *et al*.^3^	${Q}_{Lg}^{-1}=1100{f}^{0.19}$	Lg	21	0.6–10	Grenvill Province
Atkinson^71^	${Q}_{Lg}=540\,{f}^{0.41}$	Lg		0.5–10	southeastern Canada
Atkinson & Mereu^4^	*Q* = 670 *f* ^0.33^	Shear wave window	100	1–10	Southeastern Canada
Shi *et al*.^68^	*Q*_*Lg*_ = (561–721) *f* ^0^	Lg	8	0.5–16	Appalachian in northeast U.S
Benz, *et al*.^11^	*Q*_*Lg*_ = 1052(*f*/1.5)	Lg	—	1.5–12	Northeastern U.S
Atkinson^70^	*Q* = 893 *f* ^0.32^	Shear wave window	186	0.2–20	Southeastern Canada
Erickson, *et al*.^69^	*Q*_*Lg*_ = 650 *f* ^0.36^	Lg	12	0.5–16	Southeastern Canada
Boatwright & Seekins^59^	*Q* = 410 *f* ^0.5^	Shear & Lg	3	0.2–20	Southeastern Canada
Pasyanos^25^	*Q Tomography*	Pn, Pg, Sn, & Lg	—	2–4	North America
Atkinson & Boore^60^	*Q* = 525 *f* ^0.45^	Shear wave window	—	0.2–20	Eastern North America
Mousavi *et al*.^61^	*Q*_*Lg*_ = 615 *f* ^0.35^	Lg and Sn	91	0.9–10.75	Easternmost Canada
Gallegos *et al*.^34^	*Q Tomography*	Lg	39	1 Hz	Central and Eastern United States
Mitchell *et al*.^14^	*Q Tomography*	Lg coda	—	1 Hz	North America
This Study	*Q Tomography*	Lg	473	0.1–20	Eastnorthern North America

### Correlations with geological structures

The age, physical properties, thermal status, degree of heterogeneity, and/or crustal thickness all affect *Q*_*Lg*_. Hence, crustal attenuation can reflect large-scale crustal features and the intensity of recent tectonic activity in a region^13,72^. *Q*_*Lg*_ exhibits higher values for stable regions, in contrast to lower values in tectonically active regions. *Q*_*Lg*_ values have a direct relationship with crustal thickness^62,63^ and an inverse relationship with either strong scattering by small-scale heterogeneity or partial melting in the crust^22^. Thick unconsolidated sediments^12^ volcanic and geothermal regions^6,27^ have been associated with low-*Q*-values.

**Figure 7:**
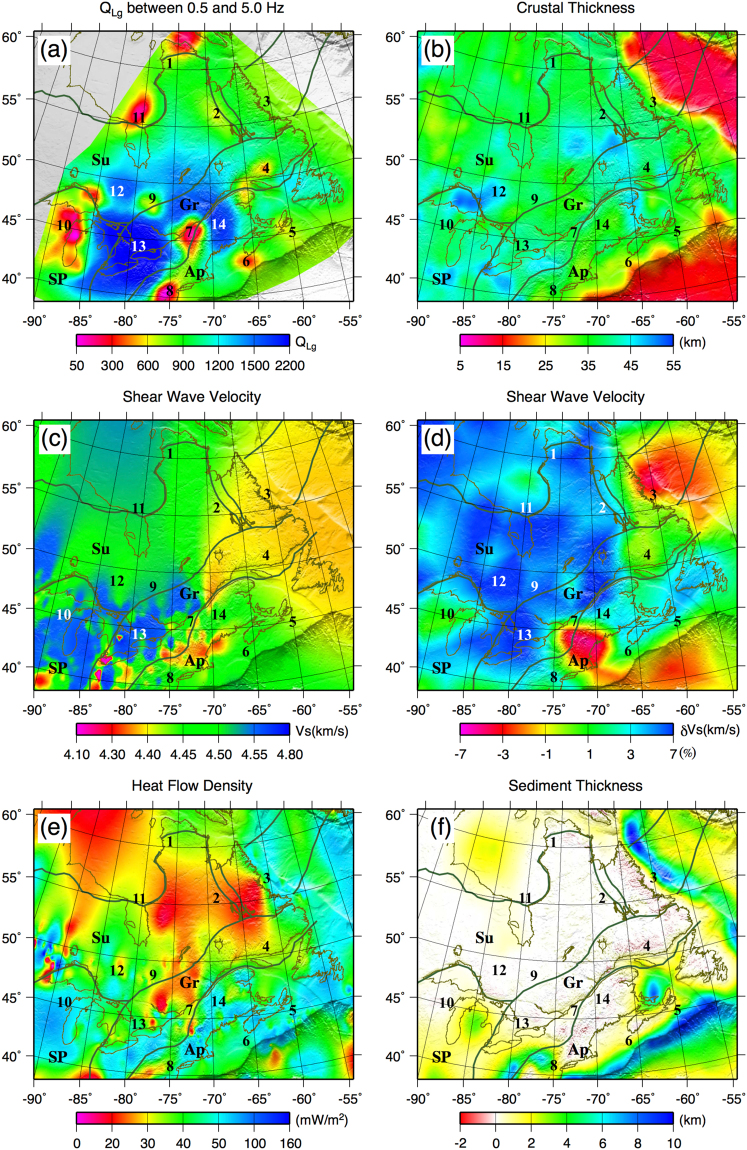
(**a**) The average *Q*_*Lg*_ in logarithmic scale between 0.5 and 5.0 Hz, (**b**) crustal thickness based on CRUST1.0 model^83^, (**c**) shear wave velocity model^78^ at 60 km depth, (**d**) dVs model^44^ at 60 km depth, (**e**) estimated heat flow density at a depth of 100 km^84^, and sediment thickness^83^, along with major geological provinces; Superior (Su), Grenville (Gr), Appalachian (Ap), and Southern (SP). The figure is generated from GMT, which is an open source software available at: http://gmt.soest.hawaaii.edu.

We present our broadband *Q*_*Lg*_ model (average *Q* values of individual frequencies between 0.5 to 5.0 Hz) in Fig. 7a. This average crustal model, which has similar features as the *Q*_*0*_ model (*Q*_*Lg*_ at 1 Hz), is used to further explore the correlation of the attenuation model with geological structures in this region. Generally, *Q*_*Lg*_ increases with the age of a region since its last major tectonic event^65^, because crustal rocks cool over time. The Canadian Shield is the exposed part of the craton that is the oldest and most stable part of North America, which explains the relatively high and uniform *Q* values observed over the Canadian Shield. Moreover, several studies showed that seismic velocities are significantly higher than average under the Canadian Shield^44^, while the surrounding Paleozoic areas have slower S wave velocity^44^ (Fig. 6d).

The Canadian Shield also has low average heat flow (Fig. 7e), indicating that the cratonic lithosphere must be thick and cold^73^. The intrinsic attenuation of shear waves is strongly correlated with the temperature of the Earth. Average crustal heat generation is estimated to be 0.6 μW/m^2^ in the Canadian Shield^74^. The New Quebec orogeny, northwest of Hudson Strait, and the Belcher belt are parts of a passive margin, composed of Proterozoic volcanic materials. Hence, relatively lower *Q* values observed at these regions correlate with a slightly younger geological setting and major thrust-faulting in these areas.

Relatively low *Q*-values observed in coastal areas of Labrador Sea and Nova Scotia can be attributed to their younger age relative to the neighboring areas, and/or *Lg*-blockage and strong attenuation of *Lg* energy in oceanic regions^27^. Mousavi *et al*.^61^ have reported *Lg*-blockage for southeastern Nova Scotia. However, these areas have relatively low shear wave velocities (Fig. 7d) and thick sedimentary layers (Fig. 7f). Mitchell and Hwang^12^ mentioned the thick unconsolidated sediments as a source of high *Lg*-attenuation.

A marked increase in heat flow has been observed in the young Appalachian Province. Shapiro *et al*.^75^ mapped high mantle heat-flow and thin lithosphere to the southwest of the CHV. Moreover, low shear wave velocities have been reported in this zone (Fig. 7d) by 76 and 44. These results can explain the observed low-*Q* anomaly to the southwest of the CHV. Volcanic rocks of Quaternary age are located north of St. Lawrence Gulf ^76^. Xie^6^ and Zor *et al*.^24^ have reported low *Q*_*Lg*_ values for volcanic and geothermal regions.

The low-*Q* anomaly south of Lake Superior, reported by both 25 and 34, is confined inside the 1.1-billion-year-old MCR, a lithospheric-scale feature. The lithosphere beneath the rift is different from the surrounding continental material because of thinning, modification, or removal of the continental lithosphere and its replacement by less-depleted mantle material^77^. The upper lithospheric layer is thinnest, with a thickness of about 50 km, at the MCR^45^ The crust in the west arm of the MCR, thicker than its surroundings, is composed of sedimentary rocks underlain by layered volcanic rocks^47^. The middle crust in this zone, associated with low shear wave velocities^44,45,77,78^ and higher temperatures^2002^, is interpreted to be influenced by the Great Meteor hotspot track^77^.

The low-*Q* anomaly at southeast of SGL (#8), including the New York metropolitan area and Delaware Valley, is close to the area of the intense low-velocity anomaly known as Northern Appalachian Anomaly (NAA)^44^. This low-velocity anomaly has been associated with the Great Meteor hotspot^79^, the combined effects of repeated rifting processes and northward extension of the hotspot-related Bermuda low-velocity channel^80^, and a small-scale asthenosphere upwelling^81^.

Anomalously high shear-wave velocity has been reported^44,78,80^ for the crustal structure of the Great Lake basin in the northwestern SGL, where dominant high-*Q* anomalies are observed (#13). The thickness of the continental lithosphere in this zone is about 115 km beneath the Michigan basin^80^. Yuan *et al*.^80^ proposed the existence of deep-rooted high-velocity blocks in this area representing the Proterozoic Gondwanian terranes of pan-African affinity, which were captured during the Rodinia formation but left behind after the opening of the Atlantic Ocean. A big portion of this high-*Q* anomaly is within the Granite-Rhyolite province (1.55–1.35 Ga)^43^ that was formed through intracratonic magmatism. 25 and 34 obtained similar high-Q values in this region.

### The frequency dependence of crustal attenuation

**Figure 8:**
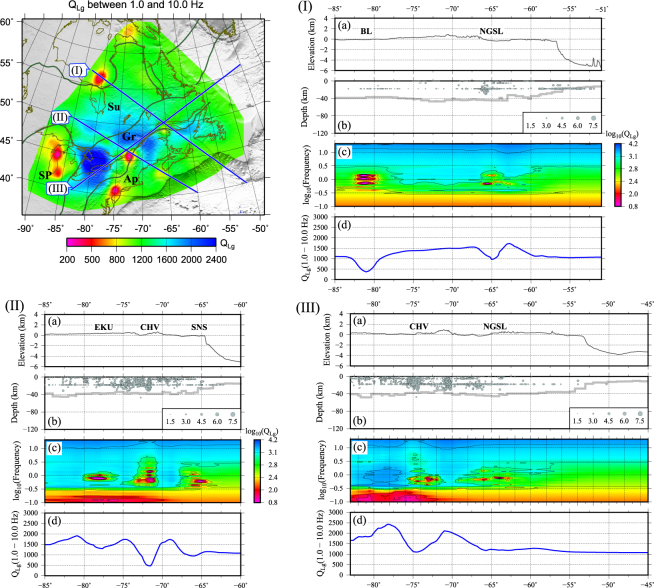
On the upper left plot, an average *Q*_*Lg*_ (1.0–10.0 Hz) map is presented showing location of three lines associated to each subplots ((I), (II), and (III)). In each subplot, from top to bottom: (**a**) Surface topography, (**b**) Moho depth based on CRUST1.0^83^ with seismicity, (**c**) *Q*_*Lg*_ versus frequency, and (**d**) average *Q*_*Lg*_. The horizontal coordinate is longitude. The figure is generated from GMT, which is an open source software available at: http://gmt.soest.hawaaii.edu.

The *Q*_*Lg*_ variations in different frequency bands sheds light on the features of crustal structure in response to varying depth ranges and wavelengths. Crustal attenuation as a function of frequency has been investigated along three lines, two almost parallel in NW-SE direction and one orthogonal to these two (Fig. 8). Two apparent low-*Q*_*Lg*_ anomalies along profile-I correspond to the low-*Q*_*Lg*_ under the Belcher belt (#11) and north of the Gulf of St. Lawrence (#4), respectively. The absorbing frequency band of the anomaly under the Belcher belt is relatively wider: 0.6–1.6 Hz, while it is narrower at 0.6–0.8 Hz under the NGSL region.

Three low-*Q*_*Lg*_ anomalies along profile-II correspond to high attenuation under the east Kapuskasing Uplift (EKU), southwest of the CHV, and southern Nova Scotia (SNS), respectively. Dominant absorbing frequencies for all anomalies are less than 1 Hz with a decreasing trend from northwest to southeast toward the Atlantic coast. Anomalies along profile-III are associated with NGSL and southwest of the CHV. The high-*Q* anomaly along this profile has a relatively wide frequency band (0.3–2.2 Hz). We also observe relatively lower *Q*_*Lg*_ within higher frequencies at the end of each profile where it crosses off-shore regions, which can be due to the high attenuation of *Lg* within oceanic crusts^61^.

### Average Q

**Figure 9:**
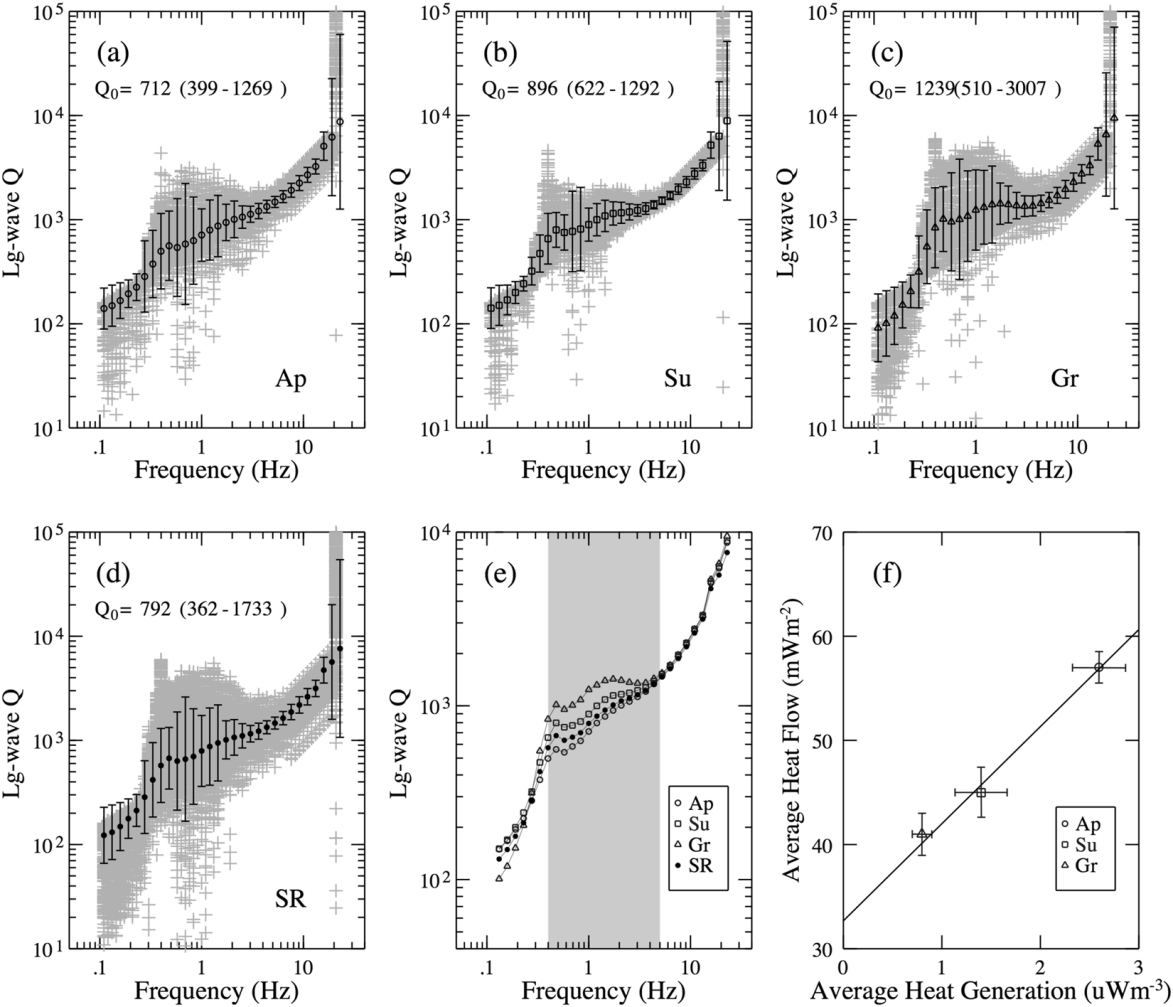
Average *Q*_*Lg*_ at 1 Hz for Appalachians (**a**), Superior (**b**), Grenville (**c**) provinces, entire area (**d**), and *Q* variation as a function of frequency for these regions (**e**). Mean heat flow and heat production for these geological provinces (**f**) average Q vs the average heat flow^73,85^.

To investigate any systematic differences between attenuation in different geological provinces, we calculated the average *Q*_*Lg*_ for major geological blocks, both at 1.0 Hz and at broadband (0.5–5.0 Hz). No clear relationship between the age and average *Q* value of geological provinces can be found (Table 2), because of significant variation in *Q* values within each province. For instance, southern Grenville has a totally different range of *Q* values compared to the northeastern Grenville. However, an inverse relationship can be seen between average *Q* and heat flow/production (Fig. 9). Mitchell *et al*.^14^ also showed that an inverse relation exists between crustal *Q*_*0*_ and upper mantle temperature for North America. Curves in Fig. 9a–e exhibit a kink at 0.5 Hz that may result from a change between crustal trapped Lg and other surface waves (fundamental and/or higher modes together). Interestingly, the lowest frequency Q does not show much regional variation. In addition, the high frequency curve upward, that might be an effect of increasing Sn coda in higher bands.

**Table 2:** Average broadband Q and Q_0_ for each geological province, and the entire study area.

Region	Age (Ga)	Average Q (0.5–5.0 Hz)			Q_0_		
		Mean Value	Lower Limit	Upper Limit	Mean Value	Lower Limit	Upper Limit
Entire Study Area	—	932	496	1753	792	362	1733
Superior	>2.5	1039	727	1484	896	622	1292
Southern + Penokean	1.9–1.6	791	317	1972	689	236	2009
Grenville	1.3–1.0	1266	662	2415	1239	510	3007
Appalachian	0.4	865	490	1531	712	399	1269

## Conclusion

High-resolution *Q*-models have been developed for eastern North America in the frequency range of 0.1 to 20.0 Hz and have been explained in the context of the tectonics and geology of the region. Our results not only agree with those from previous studies of this region, but also provide higher resolution and more reliable results due to higher azimuthal coverage of ray paths. Our *Q* estimates confirm the existence of heterogeneities in the crust related to the large-scale geological features in the area. Low-*Q* anomalies (high attenuation) are observed in New Quebec orogeny, coastal area of Labrador Sea, northwest of Hudson Strait, southeast of the Hudson Bay, south of Lake Superior, southwest of the CHV, southeast of Kapuskasing Uplift, New York metropolitan area and Delaware Valley, southeast and northeast of Nova Scotia, and north of St. Lawrence Gulf. In contrast, the Great Lake Basin and north Appalachian AP regions exhibit high-*Q* values (low attenuation). Thick sediments, volcanic rocks, and thin oceanic crust are potential sources of observed low-*Q* and low-velocity anomalies. The average *Q* of the main geological provinces is inversely correlated with average heat flow/generation.

### Data availability

Waveform data were collected from Incorporated Research Institutions for Seismology (IRIS) Data Services (http://ds.iris.edu/ds/nodes/dmc/, last accessed Jan 2017). The facilities of IRIS Data Services (DS), and specifically the IRIS DataManagement Center, were used for access to waveform, metadata, or products required in this study. The IRIS DS is funded through the National Science Foundation and specifically the GEO Directorate through the Instrumentation and Facilities Program of the National. Figures 1, 2, 4 and 6–8 were prepared using Generic Mapping Tools^82^ (version 4, http://www.soest.hawaii.edu/gmt/).

## Electronic supplementary material

supplementary information
