# Supplementary material for: Lateral Variation of Crustal Lg Attenuation in Eastern North America
Source: Sci Rep. 2018 May 8;8:7285. doi: 10.1038/s41598-018-25649-5 (PMC5940689; doi:10.1038/s41598-018-25649-5)
Supplement: Supplementary file 1 — supplementary information [file 41598_2018_25649_MOESM1_ESM.pdf]

Supplementary Information for:

Lateral Variation of Crustal Lg Attenuation in Eastern North America

By

Lian-Feng Zhao[a], and S. Mostafa Mousavi[b,*]

Running title: **Lg attenuation in eastern North America**

[a] Key Laboratory of Earth and Planetary Physics, Institute of Geology and Geophysics, Chinese Academy of Sciences, Beijing100029, PR China

[b] Department of Geophysics, Stanford University, CA 94305, USA mmousavi@stanford.edu

List of figures:

Figure S1 ------------------ data pre-processing procedure.

List of data:

RayNumber ------------------ The numbers of ray paths at individual frequencies.
Parameters ------------------ Associated frequencies for each Q and Ray file.
Q.#a ------------------ 58 lists containing Q values for different frequencies.
Columns are latitudes, longitudes, Q values, and bootstrapping STD respectively.

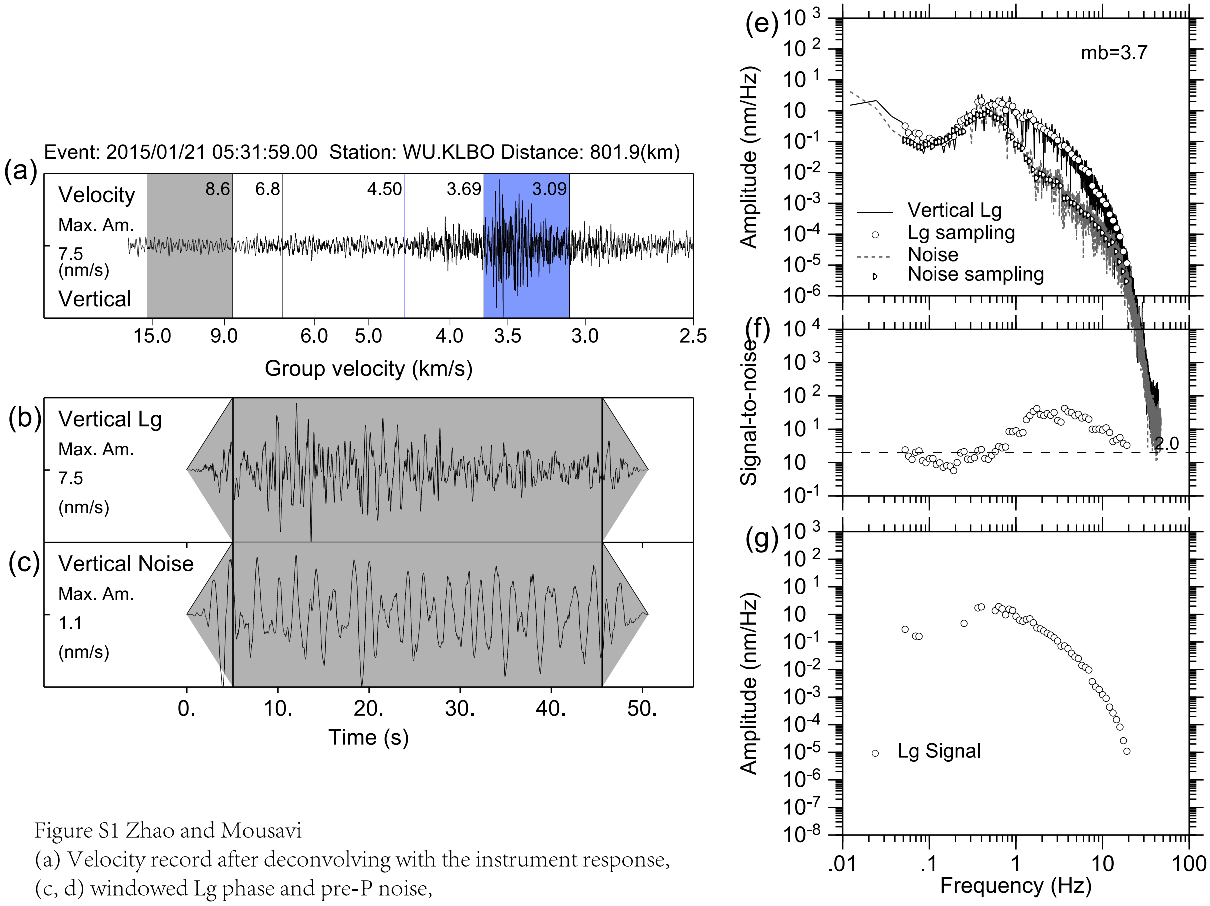

Figure S1 Zhao and Mousavi

(a) Velocity record after deconvolving with the instrument response,

(c, d) windowed Lg phase and pre-P noise,

(e) Lg-wave and noise spectra,

(f) signal-to-noise ratio, and

(g) Lg-wave spectrum. Note that the data points have been dropped where the signal-to-noise ratio is below the threshold of 2.0.

RayNumbers

freq. numbers of 1s ray, 2sray and both ray pathes.

.1097	2389	4524	6913
.1202	2291	4288	6579
.1318	2179	3993	6172
.1445	2116	3655	5771
.1585	1996	3409	5405
.1738	1923	3141	5064
.1905	1916	2998	4914
.2089	1827	2977	4804
.2291	1753	2892	4645
.2512	1681	2789	4470
.2754	1612	2793	4405
.3020	1527	2687	4214
.3311	1538	3110	4648
.3631	1639	3559	5198
.3981	1867	4356	6223
.4365	2155	4964	7119
.4786	2394	5811	8205
.5248	2745	6797	9542
.5754	3172	7642	10814
.6310	3566	8366	11932
.6918	3983	9254	13237
.7586	4277	9604	13881
.8318	4581	9923	14504
.9120	4846	10105	14951
1.0000	5151	10372	15523
1.0965	5404	10526	15930
1.2023	5693	10741	16434
1.3183	5907	10872	16779
1.4454	6114	11005	17119
1.5849	6273	11023	17296
1.7378	6445	11149	17594
1.9055	6561	11178	17739
2.0893	6710	11186	17896
2.2909	6794	11206	18000
2.5119	6865	11168	18033
2.7542	6897	11145	18042
3.0199	6931	11023	17954
3.3113	6940	10912	17852
3.6308	6922	10937	17859
3.9811	6912	10853	17765
4.3652	6863	10676	17539
4.7863	6787	10187	16974
5.2481	6685	9675	16360
5.7544	6637	9576	16213
6.3096	6546	9257	15803
6.9183	6445	8890	15335
7.5858	6320	8491	14811
8.3176	6162	8044	14206
9.1201	5978	7467	13445
10.0000	5839	7261	13100
10.9648	5762	6997	12759
12.0226	5449	5961	11410
13.1826	4617	3560	8177
14.4544	4144	2389	6533
15.8489	3799	1848	5647
17.3780	3530	1455	4985
19.0546	2527	676	3203
20.8930	2211	428	2639

Parameters

File#	Frequency	Max Q	Min Q
1	1.096500E-01	159.007800	-20.979000
2	1.202300E-01	167.506400	-31.093500
3	1.318300E-01	176.458600	-40.246310
4	1.445400E-01	185.882600	-63.999370
5	1.584900E-01	195.820000	-38.267230
6	1.737800E-01	206.284400	-93.400420
7	1.905500E-01	217.311200	391.963300
8	2.089300E-01	228.922400	254.824800
9	2.290900E-01	241.158100	279.733800
10	2.511900E-01	254.044800	167.860300
11	2.754200E-01	267.619000	287.125800
12	3.020000E-01	281.925300	-3880.775000
13	3.311300E-01	296.988700	-42548.680000
14	3.630800E-01	312.861900	9422.017000
15	3.981100E-01	329.582100	629.168200
16	4.365200E-01	347.196100	196.631000
17	4.786300E-01	365.748900	239.306300
18	5.248100E-01	385.296400	166.690100
19	5.754400E-01	405.886300	294.344700
20	6.309600E-01	427.578600	331.478200
21	6.918300E-01	450.427900	459.411900
22	7.585800E-01	474.500700	369.075600
23	8.317600E-01	499.856800	375.319900
24	9.120100E-01	526.571100	400.047900
25	1.000000	554.712500	549.782000
26	1.096480	584.357700	472.936700
27	1.202260	615.585400	545.835300
28	1.318260	648.485700	646.706400
29	1.445440	683.141000	963.095400
30	1.584890	719.648600	934.385900
31	1.737800	758.108600	1674.555000
32	1.905460	798.623500	1804.966000
33	2.089300	841.304500	1317.338000
34	2.290870	886.265100	2140.024000
35	2.511890	933.629000	1963.755000
36	2.754230	983.522900	1958.233000
37	3.019950	1036.084000	1005.666000
38	3.311310	1091.454000	1150.634000
39	3.630780	1149.784000	1003.150000
40	3.981070	1211.230000	985.651200
41	4.365160	1275.962000	1113.518000
42	4.786300	1344.151000	1107.915000
43	5.248070	1415.984000	1306.297000
44	5.754400	1491.658000	1232.457000
45	6.309570	1571.375000	1535.307000
46	6.918310	1655.352000	1485.018000
47	7.585770	1743.817000	1172.329000
48	8.317640	1837.011000	1303.744000
49	9.120110	1935.184000	1132.429000
50	10.000000	2038.604000	1856.995000
51	10.964780	2147.551000	2836.305000
52	12.022640	2262.320000	-167301.600000
53	13.182560	2383.223000	3155.193000
54	14.454400	2510.587000	2888.950000
55	15.848930	2644.757000	-77733.490000
56	17.378000	2786.098000	2728.217000
57	19.054610	2934.993000	2686.338000
58	20.892960	3091.845000	-4644.975000

Q.01a

-89.453400	32.991660	158.928700	.060671
-88.345930	33.191160	158.914400	.069782
-87.228100	33.381660	158.860800	.102486
-86.100230	33.562890	158.767700	.157854
-84.962690	33.734530	158.635300	.234040
-83.815900	33.896310	158.469500	.326559
-82.660300	34.047950	158.284600	.427918
-81.496410	34.189190	158.101800	.528270
-80.324730	34.319790	157.943400	.617271
-79.145840	34.439500	157.818800	.689090
-77.960310	34.548100	157.716900	.746828
-76.768760	34.645400	157.630300	.791405
-75.571840	34.731200	157.598100	.802265
-74.370180	34.805370	157.691500	.746729
-73.164480	34.867740	157.925500	.620309
-71.955430	34.918200	158.232500	.454681
-70.743730	34.956650	158.520700	.296575
-69.530080	34.983020	158.737200	.174564
-68.315220	34.997260	158.874500	.093139
-67.099880	34.999340	158.949700	.044760
-65.884770	34.989250	158.985600	.019181
-64.670640	34.967020	159.000400	.007249
-63.458190	34.932690	159.005700	.002375
-62.248150	34.886320	159.007300	.000657
-61.041230	34.828000	159.007800	.000163
-59.838120	34.757850	159.007800	.000029
-58.639500	34.676010	159.007800	.000000
-57.446040	34.582610	159.007800	.000000
-56.258390	34.477850	159.007800	.000000
-55.077160	34.361910	159.007800	.000000
-53.902950	34.235010	159.007800	.000000
-52.736340	34.097380	159.007800	.000000
-51.577860	33.949260	159.007800	.000000
-50.428010	33.790910	159.007800	.000000
-49.287250	33.622600	159.007800	.000000
-48.156040	33.444620	159.007800	.000000
-47.034740	33.257250	159.007800	.000000
-45.923690	33.060790	159.007800	.000000
-44.823220	32.855560	159.007800	.000000
-89.701210	33.918400	158.855400	.101229
-88.583520	34.125020	158.820400	.120000
-87.455040	34.322360	158.708400	.177859
-86.316120	34.510110	158.516900	.274234
-85.167100	34.687960	158.245200	.406260
-84.008420	34.855620	157.904900	.565619
-82.840550	35.012790	157.524800	.739147
-81.663990	35.159200	157.148100	.911140
-80.479300	35.294580	156.824600	1.063274
-79.287040	35.418700	156.579100	1.187271
-78.087840	35.531310	156.371800	1.300705
-76.882320	35.632210	156.156900	1.418560
-75.671150	35.721200	156.026500	1.489312
-74.455030	35.798110	156.193900	1.418380
-73.234660	35.862810	156.713200	1.183514
-72.010770	35.915150	157.401300	.854012
-70.784100	35.955040	158.029600	.539080
-69.555380	35.982390	158.481200	.305488
-68.325390	35.997170	158.755400	.157868
-67.094890	35.999320	158.900400	.074246
-65.864640	35.988860	158.967500	.031421
-64.635410	35.965800	158.994600	.011803
-63.407950	35.930180	159.004100	.003859

-62.183030	35.882080	159.007000	.001077
-60.961370	35.821590	159.007700	.000252
-59.743720	35.748840	159.007800	.000064
-58.530780	35.663950	159.007800	.000027
-57.323260	35.567100	159.007800	.000013
-56.121820	35.458470	159.007800	.000000
-54.927120	35.338260	159.007800	.000000
-53.739770	35.206700	159.007800	.000000
-52.560380	35.064020	159.007800	.000000
-51.389480	34.910500	159.007800	.000000
-50.227610	34.746390	159.007800	.000000
-49.075240	34.571990	159.007800	.000000
-47.932830	34.387580	159.007800	.000000
-46.800740	34.193490	159.007800	.000000
-45.679330	33.990000	159.007800	.000000
-44.568900	33.777440	159.007800	.000000
-89.960660	34.843320	158.630600	.212718
-88.832330	35.057210	158.529400	.258238
-87.692770	35.261540	158.238000	.384306
-86.542320	35.455950	157.745500	.591825
-85.381340	35.640140	157.049900	.873443
-84.210270	35.813810	156.183700	1.207039
-83.029580	35.976620	155.219800	1.564041
-81.839790	36.128310	154.257900	1.918657
-80.641480	36.268600	153.434400	2.231713
-79.435230	36.397220	152.840900	2.483092
-78.221690	36.513940	152.319400	2.749077
-77.001530	36.618520	151.619400	3.112408
-75.775430	36.710780	151.008300	3.438823
-74.544130	36.790520	151.322700	3.381656
-73.308360	36.857590	152.769900	2.856624
-72.068890	36.911860	154.737700	2.052993
-70.826490	36.953220	156.482300	1.263902
-69.581950	36.981590	157.689800	.689553
-68.336070	36.996900	158.391600	.343940
-67.089650	36.999130	158.749700	.158253
-65.843500	36.988290	158.911500	.066656
-64.598400	36.964370	158.976200	.025257
-63.355190	36.927440	158.998900	.008443
-62.114630	36.877570	159.005800	.002410
-60.877510	36.814860	159.007500	.000575
-59.644590	36.739430	159.007800	.000157
-58.416640	36.651430	159.007800	.000078
-57.194370	36.551040	159.007800	.000047
-55.978490	36.438440	159.007800	.000031
-54.769680	36.313860	159.007800	.000018
-53.568590	36.177530	159.007800	.000018
-52.375820	36.029710	159.007800	.000000
-51.191970	35.870660	159.007800	.000000
-50.017550	35.700660	159.007800	.000000
-48.853080	35.520030	159.007800	.000000
-47.698980	35.329070	159.007800	.000000
-46.555660	35.128100	159.007800	.000000
-45.423470	34.917440	159.007800	.000000
-44.302690	34.697410	159.007800	.000000
-90.232250	35.766320	158.134000	.424902
-89.092860	35.987640	157.869300	.526680
-87.941790	36.199090	157.155900	.791985
-86.779330	36.400320	155.956700	1.225396
-85.605870	36.591000	154.266100	1.805826
-84.421870	36.770790	152.186400	2.467638
-83.227800	36.939400	149.895500	3.153113
-82.024180	37.096500	147.575200	3.848004
-80.811620	37.241800	145.600300	4.454042
-79.590740	37.375040	144.332300	4.884138
-78.362190	37.495960	143.196700	5.378878
-77.126670	37.604320	141.004200	6.359147

-75.884930	37.699920	138.246500	7.538821
-74.637700	37.782550	138.588100	7.703794
-73.385770	37.852060	142.593700	6.533910
-72.129940	37.908310	148.065000	4.734938
-70.871030	37.951180	152.763800	2.869443
-69.609860	37.980580	155.859900	1.514471
-68.347290	37.996460	157.583800	.726021
-67.084140	37.998780	158.425800	.323706
-65.821270	37.987530	158.793300	.134623
-64.559530	37.962740	158.937500	.051324
-63.299760	37.924460	158.987900	.017436
-62.042780	37.872770	159.003300	.005101
-60.789440	37.807780	159.007200	.001231
-59.540510	37.729610	159.007900	.000360
-58.296810	37.638420	159.007900	.000221
-57.059080	37.534390	159.007900	.000135
-55.828080	37.417740	159.007800	.000077
-54.604500	37.288680	159.007800	.000049
-53.389020	37.147460	159.007800	.000045
-52.182290	36.994370	159.007800	.000043
-50.984880	36.829660	159.007800	.000029
-49.797380	36.653650	159.007800	.000039
-48.620270	36.466650	159.007800	.000029
-47.454020	36.268990	159.007800	.000013
-46.299010	36.060990	159.007800	.000000
-45.155610	35.843010	159.007800	.000000
-44.024070	35.615370	159.007800	.000000
-90.516530	36.687310	157.190200	.773124
-89.365660	36.916220	156.571700	.980200
-88.202590	37.134950	154.976900	1.499289
-87.027650	37.343150	152.286300	2.345856
-85.841200	37.540460	148.489700	3.452571
-84.643710	37.726540	143.916600	4.618297
-83.435650	37.901050	139.022100	5.731963
-82.217600	38.063690	133.861100	6.966345
-80.990140	38.214140	129.252200	8.127596
-79.753940	38.352110	127.294700	8.582078
-78.509670	38.477340	125.789000	9.117922
-77.258070	38.589580	119.663300	11.141620
-75.999910	38.688600	109.482200	14.776800
-74.735980	38.774210	108.363800	15.439890
-73.467090	38.846230	118.673700	12.973100
-72.194090	38.904510	133.092200	9.675606
-70.917830	38.948930	144.880700	5.864296
-69.639210	38.979390	152.219500	3.002578
-68.359090	38.995840	156.056500	1.382533
-67.078360	38.998240	157.843500	.593255
-65.797930	38.986590	158.586200	.243386
-64.518690	38.960900	158.869500	.093585
-63.241520	38.921240	158.968600	.032469
-61.967320	38.867690	158.999200	.009714
-60.696920	38.800350	159.007000	.002478
-59.431210	38.719360	159.008400	.000886
-58.170970	38.624900	159.008300	.000604
-56.917050	38.517150	159.008100	.000392
-55.670200	38.396340	159.008000	.000231
-54.431160	38.262690	159.007800	.000118
-53.200640	38.116470	159.007800	.000059
-51.979310	37.957960	159.007800	.000048
-50.767770	37.787460	159.007800	.000058
-49.566610	37.605300	159.007800	.000065
-48.376340	37.411790	159.007800	.000062
-47.197410	37.207270	159.007800	.000055
-46.030250	36.992090	159.007800	.000045
-44.875180	36.766620	159.007800	.000029
-43.732470	36.531200	159.007800	.000000
-90.814130	37.606180	155.627900	1.280331

-89.651300 37.842850 154.333800 1.661915
-88.475770 38.069030 151.069000 2.582318
-87.287830 38.284350 145.489500 4.077374
-86.087840 38.488450 137.451100 5.956678
-84.876290 38.680970 128.178500 7.599969
-83.653650 38.861550 119.047500 8.769002
-82.420500 39.029860 108.162600 10.777820
-81.177470 39.185590 98.784360 12.780520
-79.925230 39.328410 97.003300 12.810610
-78.664510 39.458070 98.651500 11.882430
-77.396060 39.574290 87.780510 14.314620
-76.120680 39.676830 65.854870 18.693560
-74.839220 39.765490 60.014650 19.512230
-73.552520 39.840080 72.816650 18.628220
-72.261480 39.900440 103.759900 16.020810
-70.967010 39.946460 130.416300 10.271160
-69.670030 39.978020 146.066100 5.159272
-68.371470 39.995060 153.700300 2.274297
-67.072270 39.997550 157.011600 .934728
-65.773400 39.985470 158.287100 .385533
-64.475770 39.958870 158.768100 .153779
-63.180340 39.917780 158.938900 .055224
-61.888020 39.862300 158.993500 .017126
-60.599740 39.792560 159.007700 .004726
-59.316390 39.708680 159.009900 .002084
-58.038840 39.610860 159.009600 .001477
-56.767930 39.499290 159.008900 .000958
-55.504470 39.374200 159.008400 .000567
-54.249260 39.235840 159.008100 .000316
-53.003000 39.084480 159.007900 .000167
-51.766410 38.920440 159.007800 .000075
-50.540120 38.744010 159.007800 .000053
-49.324710 38.555530 159.007800 .000072
-48.120720 38.355340 159.007800 .000067
-46.928620 38.143810 159.007800 .000063
-45.748810 37.921300 159.007800 .000058
-44.581620 37.688170 159.007800 .000048
-43.427300 37.444810 159.007800 .000000
-91.125630 38.522790 153.406000 1.926269
-89.950410 38.767390 150.975400 2.526552
-88.761920 39.001200 144.863700 3.895735
-87.560450 39.223820 133.911600 6.045656
-86.346370 39.434880 117.414400 8.974633
-85.120160 39.633990 99.979480 10.561270
-83.882300 39.820790 86.513400 10.175860
-82.633390 39.994930 71.679220 11.159960
-81.374080 40.156070 60.632210 11.153070
-80.105060 40.303890 59.774560 12.187180
-78.827100 40.438090 69.894160 8.545595
-77.540980 40.558400 63.610690 8.039172
-76.247540 40.664560 33.260620 11.215000
-74.947690 40.756360 23.502650 12.366890
-73.642300 40.833600 31.594250 14.821720
-72.332310 40.896110 63.357780 19.707200
-71.018690 40.943760 110.345100 14.253900
-69.702430 40.976440 138.120100 7.391032
-68.384490 40.994090 150.978500 3.151416
-67.065880 40.996670 156.169900 1.254181
-65.747600 40.984170 157.922100 .551064
-64.430650 40.956610 158.619200 .237845
-63.116020 40.914060 158.895100 .090029
-61.804690 40.856610 158.986200 .028926
-60.497620 40.784390 159.010300 .008953
-59.195770 40.697540 159.013600 .004681
-57.900040 40.596260 159.012400 .003344
-56.611330 40.480750 159.010800 .002108
-55.330490 40.351270 159.009600 .001242

-54.058330	40.208070	159.008800	.000708
-52.795620	40.051450	159.008300	.000395
-51.543080	39.881710	159.008000	.000204
-50.301390	39.699200	159.007800	.000097
-49.071130	39.504250	159.007800	.000051
-47.852850	39.297230	159.007800	.000058
-46.647030	39.078520	159.007800	.000061
-45.454070	38.848490	159.007800	.000055
-44.274280	38.607530	159.007800	.000050
-43.107920	38.356030	159.007800	.000000
-91.451740	39.437030	150.782400	2.604229
-90.263630	39.689750	146.718300	3.353642
-89.061680	39.931370	136.147600	4.952051
-87.846150	40.161480	115.899500	8.073937
-86.617400	40.379670	85.695800	11.804980
-85.375900	40.585540	58.941800	9.670514
-84.122160	40.778730	46.830190	7.100227
-82.856800	40.958850	49.313340	3.844393
-81.580460	41.125550	28.601600	6.279860
-80.293880	41.278500	32.915160	4.115539
-78.997860	41.417370	54.777120	3.943822
-77.693230	41.541890	61.862870	6.217582
-76.380870	41.651780	31.280920	5.174717
-75.061690	41.746810	15.241740	3.217311
-73.736660	41.826770	25.636620	8.699018
-72.406780	41.891490	49.974110	18.260370
-71.073040	41.940830	95.232910	16.825760
-69.736500	41.974670	131.735400	8.808047
-68.398190	41.992950	148.643400	3.828309
-67.059160	41.995620	155.203700	1.621086
-65.720480	41.982670	157.418100	.793866
-64.383220	41.954140	158.350200	.387828
-63.048400	41.910080	158.819700	.147662
-61.717090	41.850600	158.978200	.047542
-60.390290	41.775820	159.016700	.016888
-59.069010	41.685920	159.021500	.010351
-57.754210	41.581080	159.017800	.007616
-56.446830	41.461530	159.015000	.004354
-55.147770	41.327530	159.012200	.002446
-53.857880	41.179360	159.010400	.001401
-52.577970	41.017320	159.009200	.000814
-51.308780	40.841740	159.008400	.000456
-50.051000	40.652980	159.008000	.000234
-48.805250	40.451390	159.007800	.000108
-47.572090	40.237360	159.007800	.000051
-46.352010	40.011290	159.007800	.000058
-45.145370	39.773560	159.007800	.000056
-43.952510	39.524580	159.007800	.000055
-42.773630	39.264750	159.007800	.000000
-91.793200	40.348750	148.426200	3.152734
-90.591710	40.609810	142.547900	3.953388
-89.375760	40.859430	127.118400	5.859946
-88.145600	41.097210	97.405970	9.405808
-86.901570	41.322720	55.754810	9.878080
-85.644150	41.535550	21.817950	6.774298
-84.373860	41.735290	29.637610	5.419497
-83.091290	41.921560	22.667030	2.882721
-81.797160	42.093980	26.363770	6.896639
-80.492230	42.252200	31.391500	3.784701
-79.177290	42.395880	52.908840	6.651014
-77.853240	42.524730	53.764490	7.817567
-76.521000	42.638450	29.043880	4.707515
-75.181550	42.736820	19.454030	5.933766
-73.835900	42.819590	37.213700	4.676438
-72.485100	42.886590	62.199410	14.509970
-71.130210	42.937670	98.487400	14.787700
-69.772340	42.972710	130.055600	8.704010

-68.412590 42.991630 146.446800 4.421541
-67.052090 42.994400 153.581300 2.203278
-65.691960 42.980990 156.548000 1.156471
-64.333320 42.951450 158.001600 .583486
-62.977290 42.905830 158.733300 .223083
-61.624970 42.844260 158.979600 .073301
-60.277440 42.766850 159.028300 .032326
-58.935750 42.673790 159.035800 .023325
-57.600940 42.565280 159.030300 .015818
-56.273990 42.441570 159.021800 .009111
-54.955840 42.302930 159.016800 .004460
-53.647390 42.149640 159.013100 .002544
-52.349470 41.982030 159.010800 .001519
-51.062890 41.800450 159.009400 .000892
-49.788320 41.605260 159.008500 .000480
-48.526440 41.396860 159.008000 .000247
-47.277780 41.175640 159.007800 .000100
-46.042850 40.942000 159.007800 .000058
-44.822010 40.696380 159.007800 .000067
-43.615560 40.439180 159.007800 .000061
-42.423690 40.170830 159.007800 .000000
-92.150770 41.257820 147.310000 3.485076
-90.935390 41.527410 140.835800 4.346678
-89.704910 41.785250 123.718100 6.245495
-88.459540 42.030900 91.828780 9.340285
-87.199620 42.263930 47.620290 10.570640
-85.925610 42.483890 21.617270 5.769006
-84.638040 42.690380 18.742140 5.710294
-83.337520 42.882970 28.036290 6.432158
-82.024790 43.061280 27.015040 4.117589
-80.700610 43.224930 58.295280 6.397836
-79.365870 43.373580 66.240010 8.613997
-78.021450 43.506890 55.658370 6.428453
-76.668370 43.624570 14.181060 2.151704
-75.307630 43.726360 22.285470 5.742717
-73.940300 43.812040 42.243700 5.810265
-72.567500 43.881390 70.349760 10.404460
-71.190370 43.934270 103.062800 11.796200
-69.810050 43.970550 128.253400 9.080487
-68.427750 43.990140 144.469900 5.089447
-67.044650 43.993000 152.552200 2.627813
-65.661930 43.979120 156.161600 1.405517
-64.280810 43.948540 158.134400 .607414
-62.902460 43.901310 158.836300 .253996
-61.528050 43.837570 159.008600 .108097
-60.158730 43.757450 159.051700 .064031
-58.795620 43.661140 159.070600 .047136
-57.439790 43.548850 159.071900 .033490
-56.092310 43.420850 159.036900 .015198
-54.754150 43.277410 159.022700 .007602
-53.426270 43.118850 159.017000 .004385
-52.109530 42.945500 159.013400 .002717
-50.804760 42.757750 159.010900 .001624
-49.512690 42.555960 159.009300 .000897
-48.233980 42.340550 159.008400 .000456
-46.969190 42.111940 159.008000 .000196
-45.718810 41.870550 159.007800 .000085
-44.483210 41.616830 159.007800 .000077
-43.262650 41.351200 159.007800 .000066
-42.057300 41.074100 159.007800 .000000
-92.525320 42.164070 147.535600 3.616622
-91.295520 42.442420 141.164000 4.895727
-90.049940 42.708690 124.426200 7.680344
-88.788760 42.962440 93.653850 12.250380
-87.512310 43.203190 54.683580 15.573540
-86.221000 43.430500 27.194390 8.812938
-84.915410 43.643920 22.531850 4.679948

-83.596150	43.843030	21.015590	5.842265
-82.263960	44.027400	39.642610	7.127750
-80.919660	44.196660	70.624380	9.061487
-79.564150	44.350410	78.454570	9.392076
-78.198390	44.488330	54.458880	5.255031
-76.823410	44.610100	25.510030	5.573349
-75.440290	44.715440	10.687060	2.823734
-74.050180	44.804110	29.447900	3.628113
-72.654240	44.875890	62.540770	9.520075
-71.253690	44.930630	96.060030	12.603340
-69.849760	44.968180	125.296000	9.540401
-68.443720	44.988460	142.122000	5.956868
-67.036810	44.991420	151.362200	3.161775
-65.630330	44.977050	156.097400	1.488600
-64.225530	44.945390	158.230300	.666570
-62.823690	44.896510	158.834500	.308326
-61.426040	44.830540	158.899500	.186821
-60.033810	44.747610	159.012000	.098981
-58.648170	44.647940	159.098700	.068151
-57.270290	44.531750	159.080000	.043662
-55.901260	44.399320	159.044800	.021449
-54.542130	44.250930	159.024600	.011882
-53.193900	44.086940	159.020300	.007651
-51.857480	43.907680	159.016700	.004818
-50.533710	43.713560	159.013100	.002821
-49.223370	43.504980	159.010500	.001532
-47.927120	43.282370	159.008900	.000767
-46.645540	43.046150	159.008100	.000344
-45.379100	42.796800	159.007900	.000141
-44.128160	42.534760	159.007800	.000079
-42.892960	42.260480	159.007800	.000070
-41.673610	41.974410	159.007800	.000013
-92.917770	43.067330	148.809400	3.379095
-91.673000	43.354680	143.141800	4.701313
-90.411740	43.629630	128.080700	7.499164
-89.134110	43.891690	99.680070	11.497520
-87.840450	44.140390	58.357700	15.079030
-86.531140	44.375260	23.158810	13.232390
-85.206750	44.595830	23.486780	7.126283
-83.867900	44.801650	24.038610	4.605598
-82.515380	44.992280	36.471810	5.915939
-81.150000	45.167310	56.259180	7.069511
-79.772730	45.326340	59.862640	7.732142
-78.384560	45.469020	55.756910	5.715020
-76.986600	45.595010	30.559430	2.651969
-75.579970	45.704020	17.486950	3.684256
-74.165890	45.795780	30.529410	6.070580
-72.745610	45.870080	67.481270	9.863958
-71.320400	45.926740	107.208300	8.855439
-69.891600	45.965610	125.405300	8.607303
-68.460530	45.986610	139.119000	6.605011
-67.028560	45.989670	148.624900	3.923382
-65.597030	45.974800	154.330400	1.946919
-64.167300	45.942020	157.056600	.949821
-62.740720	45.891430	157.969800	.539164
-61.318600	45.823140	158.362400	.330903
-59.902260	45.737310	158.680600	.201734
-58.492950	45.634170	158.898600	.115958
-57.091900	45.513950	158.943900	.074706
-55.700260	45.376930	158.961500	.048792
-54.319140	45.223450	158.999500	.027432
-52.949590	45.053840	159.020900	.014923
-51.592580	44.868490	159.020900	.008640
-50.248970	44.667810	159.016100	.004708
-48.919560	44.452230	159.012100	.002416
-47.605040	44.222190	159.009700	.001160
-46.305980	43.978160	159.008500	.000533

-45.022840	43.720610	159.008000	.000211
-43.755960	43.450020	159.007800	.000088
-42.505550	43.166850	159.007800	.000073
-41.271660	42.871580	159.007800	.000018
-93.329110	43.967430	150.749300	2.872445
-92.068780	44.264030	146.229600	3.954295
-90.791210	44.547890	134.271600	6.181468
-89.496510	44.818510	110.176100	9.758515
-88.184940	45.075390	70.736720	14.474320
-86.856880	45.318050	29.806240	14.765830
-85.512890	45.545990	13.514580	9.726449
-84.153590	45.758730	27.407780	7.568871
-82.779790	45.955820	28.029460	4.863902
-81.392350	46.136820	26.659460	4.107601
-79.992260	46.301310	33.389850	3.923567
-78.580580	46.448910	38.177200	4.781782
-77.158460	46.579270	30.707390	6.170969
-75.727110	46.692070	33.071370	8.536216
-74.287820	46.787040	49.286510	9.257676
-72.841890	46.863950	92.399240	7.089405
-71.390720	46.922590	116.175300	6.861625
-69.935700	46.962830	128.433300	7.318302
-68.478260	46.984570	136.911900	6.821243
-67.019850	46.987740	145.202900	4.748225
-65.561920	46.972340	151.222700	2.782501
-64.105920	46.938410	154.793900	1.543854
-62.653260	46.886040	156.558800	.917409
-61.205390	46.815350	157.406900	.623636
-59.763680	46.726520	158.015400	.424681
-58.329470	46.619790	158.438000	.272300
-56.904060	46.495390	158.693700	.173182
-55.488670	46.353640	158.888100	.101269
-54.084500	46.194880	159.020800	.054956
-52.692630	46.019470	159.042700	.029078
-51.314070	45.827840	159.029700	.014949
-49.949730	45.620390	159.019500	.007640
-48.600430	45.397590	159.013800	.003460
-47.266860	45.159900	159.010500	.001564
-45.949600	44.907820	159.008800	.000687
-44.649100	44.641840	159.008100	.000284
-43.365670	44.362450	159.007800	.000093
-42.099450	44.070140	159.007800	.000073
-40.850440	43.765410	159.007800	.000018
-93.760410	44.864160	152.909100	2.260400
-92.483920	45.170280	149.705800	3.183267
-91.189420	45.463320	141.178100	5.252914
-89.876960	45.742750	123.647500	8.892336
-88.546760	46.008070	90.983440	16.084580
-87.199170	46.258760	52.090140	22.220080
-85.834720	46.494300	34.247980	19.037150
-84.454070	46.714200	37.402480	13.548010
-83.058010	46.917960	41.610990	10.568740
-81.647480	47.105130	33.589730	11.429020
-80.223460	47.275260	23.056020	13.726370
-78.787090	47.427960	30.032300	13.562950
-77.339580	47.562840	34.477510	14.970470
-75.882230	47.679570	51.829280	15.645940
-74.416370	47.777870	83.068730	12.697120
-72.943440	47.857470	114.187200	7.866329
-71.464880	47.918190	129.980100	5.533482
-69.982220	47.959850	135.835300	5.924068
-68.496960	47.982350	139.827200	5.828229
-67.010670	47.985630	144.424000	4.771624
-65.524890	47.969690	149.144700	3.275027
-64.041170	47.934570	152.641900	2.126629
-62.561040	47.880340	154.622500	1.461114
-61.086020	47.807170	155.654800	1.119663

-59.617590	47.715230	156.551700	.829094
-58.157170	47.604770	157.449200	.554177
-56.706150	47.476050	158.300900	.297623
-55.265830	47.329400	158.838300	.150289
-53.837470	47.165180	159.071400	.088621
-52.422200	46.983780	159.083100	.049003
-51.021090	46.785640	159.042300	.020305
-49.635090	46.571200	159.020800	.010102
-48.265020	46.340950	159.014300	.004618
-46.911610	46.095370	159.011100	.001770
-45.575410	45.834990	159.009100	.000761
-44.256860	45.560320	159.008200	.000311
-42.956210	45.271870	159.007800	.000117
-41.673580	44.970160	159.007800	.000066
-40.408870	44.655690	159.007800	.000013
-94.212860	45.757310	154.880600	1.634566
-92.919560	46.073230	152.851000	2.278148
-91.607470	46.375730	147.625100	3.869657
-90.276550	46.664250	136.890700	6.982748
-88.926960	46.938270	116.531200	13.482600
-87.559010	47.197250	87.102250	23.367200
-86.173240	47.440650	68.017950	26.321360
-84.770290	47.667940	66.102750	22.418820
-83.350960	47.878600	65.801290	18.671720
-81.916200	48.072160	56.368890	19.484840
-80.467090	48.248140	50.924020	20.328180
-79.004780	48.406110	53.511690	21.102410
-77.530590	48.545680	67.588140	18.813780
-76.045850	48.666500	91.370530	16.011380
-74.552020	48.768250	114.134100	12.012850
-73.050600	48.850660	131.664200	7.301241
-71.543170	48.913520	141.223400	4.551482
-70.031330	48.956650	144.818400	3.964096
-68.516710	48.979950	146.327600	3.893583
-67.000980	48.983350	148.212800	3.449851
-65.485790	48.966840	150.385900	2.816944
-63.972820	48.930470	152.464200	2.151821
-62.463700	48.874330	153.769300	1.706917
-60.960050	48.798580	154.328900	1.484811
-59.463460	48.703410	154.914000	1.290444
-57.975440	48.589080	156.155900	.913463
-56.497490	48.455880	157.716600	.449829
-55.030970	48.304140	158.700800	.176564
-53.577210	48.134270	159.050100	.096276
-52.137430	47.946660	159.093300	.048950
-50.712720	47.741790	159.050900	.019101
-49.304080	47.520120	159.024400	.008432
-47.912350	47.282170	159.014500	.003893
-46.538250	47.028450	159.011300	.001875
-45.182340	46.759510	159.009300	.000743
-43.845020	46.475880	159.008300	.000313
-42.526500	46.178100	159.007900	.000118
-41.226800	45.866700	159.007800	.000048
-39.945740	45.542200	159.007800	.000000
-94.687740	46.646660	156.417100	1.100034
-93.376940	46.972690	155.273300	1.482849
-92.046550	47.284930	152.475700	2.395232
-90.696440	47.582840	146.646800	4.349044
-89.326680	47.865840	136.134700	8.190540
-87.937550	48.133390	120.576500	14.624200
-86.529520	48.384910	105.963800	19.781850
-85.103260	48.619850	100.152900	19.071940
-83.659580	48.837660	96.414070	17.109810
-82.199460	49.037830	91.999180	16.871420
-80.724000	49.219880	89.799760	18.640280
-79.234450	49.383340	97.974820	16.814890
-77.732160	49.527770	110.540400	14.454770

-76.218590	49.652830	122.679700	12.011880
-74.695260	49.758160	133.997200	8.501764
-73.163800	49.843490	142.660000	5.674136
-71.625870	49.908580	148.047700	3.725162
-70.083210	49.953250	150.635900	2.717986
-68.537570	49.977380	151.818200	2.320674
-66.990740	49.980900	152.647300	2.036537
-65.444490	49.963800	153.688800	1.726503
-63.900620	49.926140	154.577900	1.488810
-62.360880	49.868010	155.180200	1.302596
-60.827010	49.789570	155.342300	1.210574
-59.300730	49.691040	155.524700	1.124610
-57.783640	49.572690	156.424300	.829206
-56.277320	49.434830	157.740300	.440205
-54.783270	49.277820	158.681700	.160855
-53.302860	49.102080	159.020500	.073585
-51.837360	48.908040	159.071700	.041046
-50.387950	48.696200	159.052900	.019389
-48.955640	48.467040	159.032600	.009375
-47.541310	48.221130	159.020400	.004790
-46.145660	47.959000	159.012900	.001881
-44.769230	47.681210	159.009600	.000831
-43.412380	47.388330	159.008400	.000332
-42.075270	47.080920	159.007900	.000123
-40.757810	46.759540	159.007800	.000032
-39.459720	46.424710	159.007800	.000000
-95.186440	47.531940	157.490700	.698170
-93.857410	47.868410	156.937700	.883340
-92.508000	48.190720	155.640900	1.320102
-91.137910	48.498310	152.872000	2.297899
-89.747150	48.790600	147.906300	4.135883
-88.335950	49.067010	140.957800	6.986868
-86.904710	49.326930	133.564000	9.781419
-85.454100	49.569790	128.150900	10.771860
-83.984930	49.795020	125.418200	9.953870
-82.498220	50.002060	123.049300	10.468470
-80.995120	50.190400	125.230200	10.590550
-79.476920	50.359550	130.785400	9.808662
-77.945060	50.509060	136.270200	8.628379
-76.401090	50.638520	141.621600	6.788511
-74.846650	50.747580	146.075800	5.068742
-73.283450	50.835950	149.778100	3.609298
-71.713320	50.903350	152.362600	2.601357
-70.138080	50.949630	153.598400	2.060378
-68.559640	50.974620	154.569800	1.599579
-66.979900	50.978270	155.585900	1.171500
-65.400800	50.960560	156.284900	.921933
-63.824260	50.921550	156.714300	.776103
-62.252170	50.861340	156.989200	.689109
-60.686400	50.780110	156.958100	.703236
-59.128770	50.678080	157.058600	.654069
-57.581030	50.555550	157.475700	.520330
-56.044860	50.412850	158.299200	.257251
-54.521820	50.250360	158.795800	.108708
-53.013420	50.068520	158.981800	.045895
-51.520960	49.867810	159.033200	.022625
-50.045680	49.648740	159.035500	.013652
-48.588630	49.411830	159.026700	.007919
-47.150700	49.157670	159.017400	.003756
-45.732590	48.886830	159.011800	.001746
-44.334810	48.599900	159.009400	.000755
-42.957660	48.297460	159.008300	.000301
-41.601190	47.980120	159.007900	.000119
-40.265250	47.648430	159.007800	.000024
-38.949390	47.302930	159.007800	.000000
-95.710510	48.412890	158.213900	.414657
-94.362470	48.760130	158.027700	.475558

-92.993230	49.092850	157.546200	.639396
-91.602360	49.410460	156.363500	1.076905
-90.189750	49.712350	154.233500	1.893042
-88.755540	49.997940	151.392900	2.989400
-87.300090	50.266570	148.279600	4.197454
-85.824040	50.517650	145.584100	4.880148
-84.328210	50.750570	143.740000	5.172336
-82.813610	50.964740	143.262800	5.214622
-81.281460	51.159630	144.986700	5.115430
-79.733130	51.334700	147.158300	4.736028
-78.170130	51.489480	149.364600	4.033259
-76.594090	51.623530	151.154500	3.326222
-75.006800	51.736490	152.532900	2.682839
-73.410070	51.828010	153.897300	2.102362
-71.805870	51.897850	154.966900	1.708198
-70.196170	51.945790	155.347700	1.543673
-68.582990	51.971680	155.723000	1.361726
-66.968440	51.975460	156.761700	.927229
-65.354560	51.957120	157.758400	.541413
-63.743440	51.916690	158.095900	.383377
-62.137130	51.854320	158.032200	.358321
-60.537630	51.770170	157.977200	.360870
-58.946900	51.664510	158.043400	.349024
-57.366820	51.537620	158.297100	.248368
-55.799180	51.389870	158.642600	.140823
-54.245650	51.221680	158.864600	.062661
-52.707820	51.033510	158.969800	.025789
-51.187080	50.825860	159.009500	.012072
-49.684720	50.599280	159.018400	.006848
-48.201800	50.354330	159.017200	.004215
-46.739240	50.091630	159.013200	.002360
-45.297720	49.811770	159.010400	.001186
-43.877700	49.515370	159.008900	.000558
-42.479410	49.203060	159.008100	.000232
-41.102810	48.875440	159.007800	.000093
-39.747590	48.533100	159.007800	.000029
-38.413150	48.176570	159.007800	.000000
-96.261670	49.289190	158.725100	.217027
-94.893750	49.647600	158.716100	.233043
-93.503820	49.991080	158.610700	.265523
-92.091320	50.319060	158.115300	.458192
-90.655950	50.630910	157.246200	.790429
-89.197760	50.925990	156.091600	1.240532
-87.717060	51.203660	154.833300	1.707383
-86.214420	51.463270	153.752600	2.095643
-84.690660	51.704190	152.921300	2.292626
-83.146820	51.925780	152.823300	2.385204
-81.584160	52.127480	153.521100	2.264123
-80.004110	52.308710	154.442500	2.001972
-78.408280	52.468990	155.294300	1.701435
-76.798410	52.607840	155.776500	1.455517
-75.176390	52.724850	155.883200	1.333512
-73.544200	52.819690	156.078600	1.240028
-71.903920	52.892050	156.499300	1.118669
-70.257710	52.941730	156.376800	1.183318
-68.607750	52.968570	156.318200	1.191665
-66.956280	52.972490	156.984400	.922310
-65.305560	52.953480	158.021600	.493415
-63.657820	52.911580	158.474700	.295016
-62.015270	52.846950	158.495000	.231829
-60.380080	52.759750	158.426700	.234679
-58.754360	52.650280	158.512900	.182318
-57.140120	52.518850	158.635400	.134110
-55.539290	52.365840	158.794500	.077603
-53.953660	52.191710	158.915400	.037796
-52.384890	51.996940	158.976200	.016749
-50.834480	51.782070	159.002200	.007235

-49.303730	51.547700	159.010800	.003595
-47.793770	51.294400	159.011700	.002132
-46.305490	51.022820	159.010500	.001267
-44.839570	50.733620	159.009200	.000694
-43.396390	50.427420	159.008400	.000346
-41.976090	50.104880	159.008000	.000145
-40.578500	49.766630	159.007800	.000061
-39.203150	49.413260	159.007800	.000029
-37.849210	49.045330	159.007800	.000000
-96.841810	50.160530	158.991200	.113480
-95.453030	50.530490	159.038200	.119214
-94.041510	50.885130	159.013100	.124271
-92.606450	51.223850	158.790900	.189435
-91.147380	51.546020	158.415400	.325095
-89.664200	51.850960	157.942300	.502940
-88.157130	52.138020	157.442900	.692256
-86.626690	52.406500	156.999500	.859643
-85.073680	52.655720	156.700400	.970945
-83.499180	52.885050	156.724000	1.001597
-81.904440	53.093850	157.059700	.921433
-80.290990	53.281520	157.536800	.776922
-78.660530	53.447530	157.900200	.624835
-77.014920	53.591380	157.881500	.580988
-75.356160	53.712650	157.445900	.681111
-73.686420	53.810940	157.072700	.814600
-72.007930	53.885960	157.113400	.828542
-70.322990	53.937460	156.963100	.897675
-68.634010	53.965280	156.627000	1.040772
-66.943400	53.969350	156.965300	.894987
-65.253590	53.949630	157.791300	.554197
-63.567000	53.906200	158.387300	.298619
-61.886040	53.839190	158.587600	.213141
-60.213040	53.748820	158.678300	.143782
-58.550290	53.635360	158.720600	.108516
-56.899960	53.499180	158.778600	.081383
-55.264110	53.340690	158.876800	.048407
-53.644650	53.160350	158.948500	.024242
-52.043340	52.958710	158.984700	.011202
-50.461760	52.736320	159.001200	.004802
-48.901280	52.493820	159.007500	.002066
-47.363030	52.231850	159.009000	.001044
-45.847900	51.951060	159.008900	.000622
-44.356530	51.652150	159.008400	.000361
-42.889220	51.335790	159.008000	.000193
-41.445980	51.002650	159.007800	.000083
-40.026490	50.653380	159.007800	.000034
-38.630060	50.288590	159.007800	.000032
-37.255620	49.908830	159.007800	.000000
-97.453060	51.026530	159.048300	.056982
-96.042340	51.408490	159.081600	.060809
-94.608220	51.774700	159.058800	.061362
-93.149600	52.124570	158.962800	.081983
-91.665800	52.457450	158.812700	.133164
-90.156560	52.772640	158.629300	.203694
-88.621980	53.069450	158.441000	.278047
-87.062480	53.347160	158.279100	.341364
-85.478850	53.605050	158.183900	.386957
-83.872130	53.842430	158.221100	.387025
-82.243660	54.058630	158.403100	.345476
-80.595020	54.253030	158.650800	.266793
-78.928000	54.425040	158.830400	.218566
-77.244600	54.574130	158.660300	.257176
-75.546970	54.699840	158.051900	.412258
-73.837420	54.801750	157.461700	.623471
-72.118360	54.879540	157.282100	.718123
-70.392330	54.932960	157.292400	.723913
-68.661900	54.961820	157.043500	.818916

-66.929700	54.966030	157.200900	.761846
-65.198370	54.945580	157.801400	.513462
-63.470540	54.900540	158.332700	.306642
-61.748820	54.831050	158.672500	.163599
-60.035740	54.737340	158.815100	.092456
-58.333780	54.619720	158.819700	.070753
-56.645260	54.478570	158.852500	.056580
-54.972420	54.314330	158.921400	.032772
-53.317280	54.127510	158.968000	.016487
-51.681740	53.918690	158.991100	.007791
-50.067420	53.688470	159.001800	.003416
-48.475770	53.437500	159.006300	.001384
-46.907930	53.166490	159.007800	.000555
-45.364770	52.876130	159.008100	.000279
-43.846840	52.567130	159.008000	.000166
-42.354370	52.240220	159.007800	.000078
-40.887210	51.896080	159.007800	.000042
-39.444820	51.535370	159.007800	.000034
-38.026250	51.158730	159.007800	.000034
-36.630120	50.766700	159.007800	.000000
-98.097820	51.886770	159.042300	.026758
-96.663910	52.281200	159.049500	.027168
-95.206040	52.659450	159.039000	.027564
-93.722790	53.020900	159.005400	.034316
-92.213200	53.364910	158.952700	.052628
-90.676770	53.690770	158.887700	.078852
-89.113440	53.997730	158.822000	.106717
-87.523560	54.285050	158.768400	.130027
-85.907830	54.551970	158.743800	.142250
-84.267300	54.797760	158.769700	.139640
-82.603340	55.021710	158.853500	.113978
-80.917590	55.223140	158.989800	.093647
-79.211960	55.401440	159.120200	.132011
-77.488560	55.556020	158.944500	.182250
-75.749730	55.686380	158.325400	.295327
-73.997920	55.792100	157.644700	.529512
-72.235800	55.872810	157.234700	.709704
-70.466090	55.928230	157.290900	.698350
-68.691570	55.958180	157.378600	.658007
-66.915130	55.962550	157.568800	.581207
-65.139640	55.941330	158.029400	.410513
-63.367970	55.894590	158.437100	.247086
-61.602940	55.822490	158.721500	.130030
-59.847310	55.725280	158.840400	.073116
-58.103760	55.603290	158.860500	.054629
-56.374820	55.456930	158.893000	.043176
-54.662870	55.286670	158.946500	.023844
-52.970100	55.093080	158.979200	.012025
-51.298500	54.876750	158.995100	.005745
-49.649800	54.638340	159.002700	.002583
-48.025460	54.378550	159.006000	.001070
-46.426660	54.098120	159.007400	.000405
-44.854210	53.797780	159.007800	.000159
-43.308580	53.478290	159.007800	.000075
-41.789850	53.140400	159.007800	.000078
-40.297680	52.784830	159.007800	.000075
-38.831280	52.412250	159.007800	.000082
-37.389370	52.023280	159.007800	.000077
-35.970190	51.618480	159.007800	.000000
-98.778840	52.740810	159.030200	.012835
-97.320270	53.148240	159.030500	.012516
-95.837370	53.539010	159.026700	.012332
-94.328290	53.912530	159.015600	.013753
-92.791720	54.268120	158.998000	.019310
-91.226900	54.605080	158.976600	.028195
-89.633560	54.922620	158.955500	.037874
-88.011890	55.219970	158.940300	.045700

-86.362530	55.496330	158.937600	.048900
-84.686490	55.750910	158.955800	.044752
-82.985150	55.982970	159.003600	.037380
-81.260250	56.191770	159.095600	.065381
-79.513790	56.376650	159.213800	.139413
-77.748020	56.536990	159.083200	.179393
-75.965460	56.672260	158.484300	.233296
-74.168790	56.781970	157.756900	.475877
-72.360860	56.865740	157.136100	.732939
-70.544630	56.923280	157.119800	.750293
-68.723180	56.954370	157.452100	.610722
-66.899620	56.958910	157.870100	.452917
-65.077100	56.936880	158.284300	.298524
-63.258730	56.888360	158.574600	.186756
-61.447630	56.813510	158.756100	.109525
-59.646790	56.712620	158.840100	.065115
-57.859090	56.586040	158.851000	.058818
-56.087300	56.434210	158.909000	.037612
-54.333970	56.257640	158.959700	.019436
-52.601470	56.056940	158.985200	.009540
-50.891880	55.832740	158.997500	.004542
-49.207030	55.585770	159.003500	.002075
-47.548420	55.316760	159.006100	.000893
-45.917200	55.026500	159.007200	.000354
-44.314130	54.715760	159.007600	.000135
-42.739580	54.385350	159.007800	.000100
-41.193420	54.036030	159.007800	.000078
-39.675070	53.668560	159.007800	.000073
-38.183400	53.283620	159.007800	.000073
-36.716770	52.881820	159.007800	.000069
-35.272900	52.463700	159.007800	.000000
-99.499240	53.588130	159.020000	.006210
-98.014280	54.009130	159.019600	.005920
-96.504860	54.412960	159.018100	.005559
-94.968610	54.799050	159.014600	.005440
-93.403790	55.166730	159.009000	.006507
-91.809290	55.515250	159.002400	.008987
-90.184560	55.843850	158.996400	.011921
-88.529620	56.151670	158.993300	.014163
-86.844990	56.437890	158.996400	.014667
-85.131650	56.701690	159.010900	.014529
-83.390960	56.942230	159.047600	.028262
-81.624720	57.158780	159.125800	.072005
-79.835050	57.350580	159.242700	.145380
-78.024370	57.516980	159.157300	.181510
-76.195360	57.657400	158.583700	.195207
-74.350940	57.771310	157.844100	.440012
-72.494220	57.858320	157.095400	.743404
-70.628410	57.918090	156.969200	.796768
-68.756890	57.950390	157.437900	.609019
-66.883060	57.955100	158.026800	.383099
-65.010370	57.932220	158.441500	.233335
-63.142240	57.881810	158.692000	.140007
-61.282040	57.804080	158.818100	.083661
-59.433060	57.699310	158.846800	.063021
-57.598450	57.567890	158.857100	.057284
-55.781200	57.410300	158.914500	.036206
-53.984060	57.227100	158.965300	.016989
-52.209560	57.018940	158.988700	.008081
-50.459930	56.786500	158.999200	.003767
-48.737060	56.530560	159.004100	.001725
-47.042490	56.251900	159.006200	.000764
-45.377330	55.951360	159.007200	.000314
-43.742270	55.629770	159.007500	.000138
-42.137470	55.287960	159.007800	.000110
-40.562610	54.926740	159.007800	.000081
-39.016750	54.546860	159.007800	.000075

-37.498410 54.149020 159.007800 .000073
-36.005400 53.733830 159.007800 .000072
-34.534880 53.301770 159.007800 .000000
-100.262700 54.428120 159.013700 .002963
-98.749250 54.863350 159.013300 .002758
-97.211560 55.280850 159.012600 .002509
-95.646590 55.680060 159.011500 .002221
-94.052090 56.060360 159.009800 .002132
-92.426510 56.420970 159.007900 .002523
-90.768940 56.761100 159.006400 .003219
-89.079190 57.079890 159.006600 .003882
-87.357570 57.376450 159.010500 .005276
-85.605000 57.649890 159.023300 .011633
-83.822840 57.899360 159.057400 .032247
-82.012920 58.124030 159.131800 .075117
-80.177480 58.323120 159.246800 .140857
-78.319110 58.495910 159.208300 .174251
-76.440710 58.641750 158.683000 .156797
-74.545430 58.760110 157.976700 .401929
-72.636660 58.850530 157.211100 .701201
-70.717930 58.912650 156.922500 .809698
-68.792920 58.946230 157.405300 .617485
-66.865380 58.951130 158.076500 .363898
-64.939080 58.927340 158.502400 .208054
-63.017780 58.874950 158.753600 .114696
-61.105210 58.794160 158.868100 .064868
-59.204930 58.685300 158.876200 .051641
-57.320400 58.548780 158.870000 .052564
-55.454840 58.385130 158.925500 .032129
-53.611270 58.194950 158.972000 .014534
-51.792370 57.978940 158.991900 .006724
-50.000500 57.737840 159.000700 .003089
-48.237630 57.472490 159.004700 .001414
-46.505310 57.183730 159.006500 .000632
-44.804610 56.872430 159.007200 .000281
-43.136050 56.539500 159.007600 .000119
-41.499610 56.185780 159.007800 .000105
-39.894630 55.812120 159.007800 .000078
-38.319800 55.419290 159.007800 .000074
-36.773090 55.007980 159.007800 .000073
-35.251730 54.578750 159.007800 .000070
-33.752120 54.132060 159.007800 .000000
-101.073700 55.260130 159.010300 .001341
-99.529010 55.710320 159.010100 .001225
-97.960900 56.142140 159.009800 .001100
-96.365430 56.555100 159.009300 .000922
-94.739640 56.948590 159.008800 .000780
-93.081440 57.321860 159.008200 .000755
-91.389490 57.674070 159.007900 .001044
-89.663250 58.004340 159.008500 .001997
-87.902820 58.311730 159.012100 .004678
-86.109000 58.595320 159.024000 .012234
-84.283100 58.854170 159.057700 .033468
-82.427000 59.087400 159.128800 .073075
-80.543050 59.294160 159.241100 .127916
-78.634010 59.473690 159.249700 .154498
-76.703000 59.625280 158.798400 .131179
-74.753460 59.748340 158.252600 .326383
-72.789070 59.842370 157.548700 .591312
-70.813740 59.906970 157.069200 .757523
-68.831480 59.941910 157.369600 .635369
-66.846440 59.947010 158.057900 .375099
-64.862770 59.922250 158.528500 .199881
-62.884610 59.867760 158.794400 .097528
-60.916040 59.783740 158.906600 .049324
-58.961010 59.670550 158.911700 .037638
-57.023270 59.528640 158.903200 .040146

-55.106350 59.358580 158.943700 .024926
-53.213500 59.161040 158.980100 .011087
-51.347600 58.936760 158.995700 .005124
-49.511150 58.686560 159.002300 .002348
-47.706190 58.411320 159.005400 .001094
-45.934240 58.111950 159.006800 .000499
-44.196280 57.789400 159.007400 .000223
-42.492650 57.444580 159.007600 .000104
-40.823030 57.078400 159.007800 .000103
-39.186360 56.691730 159.007800 .000068
-37.580830 56.285340 159.007800 .000074
-36.003750 55.859910 159.007800 .000073
-34.451600 55.415950 159.007800 .000071
-32.919830 54.953830 159.007800 .000000
-101.937200 56.083360 159.008700 .000567
-100.358100 56.549350 159.008600 .000505
-98.756940 56.996240 159.008400 .000435
-97.128790 57.423640 159.008300 .000363
-95.469860 57.830970 159.008000 .000316
-93.777320 58.217490 159.007800 .000386
-92.049300 58.582370 159.007600 .000790
-90.284800 58.924680 159.008000 .001790
-88.483640 59.243460 159.010900 .004323
-86.646400 59.537720 159.021600 .011495
-84.774360 59.806450 159.053000 .032098
-82.869400 60.048700 159.114800 .064015
-80.933970 60.263580 159.199600 .099789
-78.971000 60.450230 159.188200 .109719
-76.983890 60.607900 158.887400 .107003
-74.976370 60.735940 158.528200 .249740
-72.952470 60.833800 158.039200 .433843
-70.916480 60.901050 157.406600 .649261
-68.872850 60.937420 157.375100 .641174
-66.826130 60.942730 158.017300 .397322
-64.780930 60.916960 158.562900 .194025
-62.741830 60.860230 158.849900 .084942
-60.713310 60.772790 158.963500 .037009
-58.699720 60.654990 158.970000 .018285
-56.705200 60.507380 158.956300 .019387
-54.733590 60.330550 158.974000 .013142
-52.788400 60.125220 158.991700 .006596
-50.872730 59.892220 159.000200 .003233
-48.989200 59.632420 159.004200 .001566
-47.139900 59.346780 159.006100 .000750
-45.326320 59.036270 159.007000 .000346
-43.549270 58.701890 159.007500 .000171
-41.808860 58.344600 159.007700 .000103
-40.104370 57.965370 159.007800 .000084
-38.434220 57.565060 159.007800 .000074
-36.795950 57.144450 159.007800 .000073
-35.186060 56.704150 159.007800 .000073
-33.600030 56.244660 159.007800 .000069
-32.032190 55.766200 159.007800 .000000
-102.859900 56.896880 159.008100 .000221
-101.242000 57.379640 159.008000 .000177
-99.604390 57.842470 159.007900 .000144
-97.940930 58.285070 159.007800 .000121
-96.246660 58.706940 159.007700 .000164
-94.517820 59.107400 159.007400 .000307
-92.751850 59.485580 159.007200 .000669
-90.947170 59.840560 159.007300 .001490
-89.103220 60.171320 159.009600 .003642
-87.220290 60.476800 159.018500 .010196
-85.299550 60.755960 159.044300 .028740
-83.342830 61.007760 159.082600 .047720
-81.352710 61.231220 159.105000 .049199
-79.332320 61.425420 159.048300 .048798

-77.285290	61.589540	158.840100	.103612
-75.215710	61.722870	158.685500	.190124
-73.128000	61.824790	158.406600	.311278
-71.026890	61.894870	157.789600	.526091
-68.917300	61.932760	157.481200	.616473
-66.804310	61.938290	158.025900	.404727
-64.692990	61.911450	158.625100	.184198
-62.588420	61.852340	158.911900	.080239
-60.495590	61.761240	159.011500	.041212
-58.419300	61.638580	159.019500	.019032
-56.364090	61.484900	158.999600	.006562
-54.334160	61.300890	158.997400	.004497
-52.333320	61.087320	159.001000	.002907
-50.364880	60.845090	159.003900	.001646
-48.431600	60.575160	159.005800	.000883
-46.535590	60.278550	159.006800	.000461
-44.678250	59.956310	159.007300	.000226
-42.860180	59.609490	159.007600	.000116
-41.081100	59.239120	159.007800	.000105
-39.339830	58.846180	159.007800	.000071
-37.634110	58.431540	159.007800	.000073
-35.960610	57.995940	159.007800	.000073
-34.314830	57.539980	159.007800	.000069
-32.690990	57.064010	159.007800	.000074
-31.082000	56.568130	159.007800	.000000
-103.849600	57.699570	159.007800	.000079
-102.187300	58.200280	159.007800	.000059
-100.509000	58.680030	159.007800	.000065
-98.806840	59.138710	159.007800	.000095
-97.074520	59.575940	159.007500	.000140
-95.307120	59.991050	159.007400	.000282
-93.501110	60.383240	159.007000	.000557
-91.654170	60.751550	159.007000	.001176
-89.765210	61.094930	159.008700	.003016
-87.834170	61.412270	159.016000	.009244
-85.861950	61.702450	159.034200	.024664
-83.850420	61.964360	159.043600	.026872
-81.802150	62.196920	159.009100	.012557
-79.720490	62.399150	158.947900	.049110
-77.609360	62.570130	158.795100	.122289
-75.473210	62.709080	158.722700	.175089
-73.316960	62.815350	158.591200	.243136
-71.145790	62.888430	158.095700	.418097
-68.965200	62.927950	157.775200	.515694
-66.780790	62.933720	158.171500	.362060
-64.598260	62.905720	158.701600	.160567
-62.423240	62.844070	158.943200	.069785
-60.261290	62.749080	159.014400	.034534
-58.117710	62.621230	159.018800	.016020
-55.997520	62.461110	159.009000	.005319
-53.905320	62.269450	159.005800	.002002
-51.845240	62.047150	159.005600	.001112
-49.820820	61.795150	159.006200	.000714
-47.834930	61.514500	159.006900	.000427
-45.889670	61.206310	159.007300	.000255
-43.986290	60.871700	159.007500	.000138
-42.125070	60.511780	159.007700	.000110
-40.305270	60.127630	159.007800	.000084
-38.525020	59.720250	159.007800	.000073
-36.781200	59.290500	159.007800	.000082
-35.069400	58.839090	159.007800	.000079
-33.383770	58.366500	159.007800	.000072
-31.716990	57.872960	159.007800	.000061
-30.060130	57.358350	159.007800	.000000
-104.916500	58.490070	159.007800	.000020
-103.202300	59.010120	159.007800	.000026
-101.477600	59.507980	159.007800	.000083

-99.732440	59.983750	159.007800	.000115
-97.958720	60.437220	159.007500	.000148
-96.150010	60.867820	159.007400	.000256
-94.301540	61.274790	159.007000	.000443
-92.410060	61.657170	159.007000	.000889
-90.473700	62.013880	159.008400	.002498
-88.491920	62.343760	159.014500	.008527
-86.465330	62.645610	159.025000	.016588
-84.395630	62.918240	159.016600	.009053
-82.285480	63.160470	158.985100	.022443
-80.138370	63.371240	158.942200	.048420
-77.958560	63.549540	158.878100	.084243
-75.750930	63.694510	158.813600	.121898
-73.520860	63.805420	158.715400	.173906
-71.274150	63.881700	158.475100	.261833
-69.016910	63.922960	158.210500	.347869
-66.755400	63.928990	158.488000	.236199
-64.495980	63.899750	158.807400	.111440
-62.244980	63.835380	158.949500	.048204
-60.008560	63.736250	158.996600	.021070
-57.792640	63.602840	159.008400	.009307
-55.602750	63.435840	159.008900	.003933
-53.443960	63.236060	159.007900	.001533
-51.320750	63.004450	159.007400	.000588
-49.236890	62.742080	159.007400	.000294
-47.195330	62.450080	159.007400	.000189
-45.198120	62.129630	159.007600	.000117
-43.246250	61.781950	159.007700	.000099
-41.339580	61.408210	159.007800	.000082
-39.476710	61.009530	159.007800	.000081
-37.654880	60.586900	159.007800	.000070
-35.869840	60.141170	159.007800	.000070
-34.115770	59.672970	159.007800	.000059
-32.385130	59.182660	159.007800	.000055
-30.668580	58.670220	159.007800	.000013
-28.954870	58.135290	159.007800	.000000
-106.073800	59.266680	159.007800	.000000
-104.297500	59.807790	159.007800	.000000
-102.518800	60.325180	159.007800	.000058
-100.725000	60.819250	159.007800	.000114
-98.905530	61.289990	159.007600	.000114
-97.052100	61.737010	159.007400	.000205
-95.158340	62.159620	159.007200	.000321
-93.219700	62.556880	159.007100	.000610
-91.233350	62.927700	159.008100	.001874
-89.198040	63.270870	159.012500	.005729
-87.113920	63.585110	159.014700	.008030
-84.982480	63.869130	159.006600	.003679
-82.806390	64.121680	158.985500	.020770
-80.589290	64.341560	158.976200	.026687
-78.335770	64.527670	158.954400	.038867
-76.051160	64.679080	158.917500	.059529
-73.741460	64.794950	158.857700	.087707
-71.413090	64.874680	158.760900	.129779
-69.072890	64.917820	158.651700	.165174
-66.727910	64.924110	158.773700	.115020
-64.385280	64.893550	158.899500	.060599
-62.052090	64.826280	158.966600	.028266
-59.735260	64.722690	158.995500	.012886
-57.441380	64.583340	159.005800	.005937
-55.176610	64.408980	159.008200	.002665
-52.946510	64.200520	159.008300	.001121
-50.755950	63.959000	159.008000	.000432
-48.608900	63.685590	159.007800	.000157
-46.508400	63.381530	159.007800	.000106
-44.456330	63.048090	159.007800	.000083
-42.453320	62.686580	159.007800	.000077

-40.498620 62.298210 159.007800 .000075
-38.589950 61.884150 159.007800 .000061
-36.723370 61.445380 159.007800 .000022
-34.893150 60.982680 159.007800 .000013
-33.091610 60.496560 159.007800 .000022
-31.309040 59.987160 159.007800 .000013
-29.533510 59.454220 159.007800 .000000
-27.750760 58.896990 159.007800 .000000
-107.339200 60.027230 159.007800 .000000
-105.487000 60.591610 159.007800 .000000
-103.643800 61.130270 159.007800 .000026
-101.793500 61.644070 159.007800 .000071
-99.922550 62.133320 159.007700 .000110
-98.020040 62.597820 159.007500 .000164
-96.077510 63.037030 159.007400 .000267
-94.088710 63.450070 159.007200 .000379
-92.049460 63.835860 159.007600 .000871
-89.957580 64.193130 159.009400 .002561
-87.812570 64.520550 159.009900 .002026
-85.615560 64.816710 159.002900 .005388
-83.369120 65.080260 158.996900 .012123
-81.077050 65.309890 158.995600 .012067
-78.744270 65.504390 158.986200 .017998
-76.376630 65.662700 158.968500 .027725
-73.980770 65.783920 158.941600 .040744
-71.563890 65.867360 158.906300 .056416
-69.133670 65.912500 158.879100 .065936
-66.698060 65.919100 158.912100 .051128
-64.265090 65.887100 158.954400 .030736
-61.842790 65.816700 158.983700 .016367
-59.438900 65.708320 158.999200 .008123
-57.060850 65.562580 159.005800 .003850
-54.715470 65.380330 159.007900 .001769
-52.408880 65.162570 159.008200 .000785
-50.146370 64.910470 159.008100 .000319
-47.932120 64.625290 159.007800 .000131
-45.769150 64.308400 159.007800 .000045
-43.659080 63.961160 159.007800 .000018
-41.602020 63.584970 159.007800 .000013
-39.596350 63.181100 159.007800 .000013
-37.638600 62.750720 159.007800 .000013
-35.723270 62.294770 159.007800 .000000
-33.842640 61.813940 159.007800 .000000
-31.986660 61.308540 159.007800 .000000
-30.142740 60.778430 159.007800 .000000
-28.295590 60.222970 159.007800 .000000
-26.427080 59.640870 159.007800 .000000
-108.736700 60.768890 159.007800 .000000
-106.789100 61.359350 159.007800 .000000
-104.866900 61.921510 159.007800 .000026
-102.949500 62.456830 159.007800 .000074
-101.019200 62.966030 159.007800 .000115
-99.061880 63.449270 159.007500 .000148
-97.066180 63.906190 159.007400 .000218
-95.023590 64.336020 159.007300 .000290
-92.928180 64.737730 159.007400 .000441
-90.776390 65.110020 159.008100 .000861
-88.566850 65.451470 159.008200 .000575
-86.300130 65.760610 159.004300 .004397
-83.978550 66.035940 159.003600 .005939
-81.606060 66.276030 159.002100 .007199
-79.187900 66.479550 158.996200 .011069
-76.730480 66.645290 158.985500 .016883
-74.241170 66.772270 158.970500 .024223
-71.728080 66.859700 158.954000 .031672
-69.199870 66.907030 158.945300 .035104
-66.665550 66.913940 158.956500 .029135

-64.134220	66.880400	158.975400	.019657
-61.614990	66.806620	158.991300	.011545
-59.116610	66.693070	159.001100	.006124
-56.647430	66.540450	159.005900	.003022
-54.215080	66.349720	159.007700	.001411
-51.826330	66.121990	159.008100	.000640
-49.486860	65.858530	159.008100	.000274
-47.201050	65.560780	159.007900	.000119
-44.971820	65.230190	159.007800	.000056
-42.800360	64.868270	159.007800	.000013
-40.685980	64.476460	159.007800	.000000
-38.625890	64.056100	159.007800	.000000
-36.614990	63.608330	159.007800	.000000
-34.645670	63.134020	159.007800	.000000
-32.707610	62.633670	159.007800	.000000
-30.787590	62.107330	159.007800	.000000
-28.869240	61.554470	159.007800	.000000
-26.932890	60.973880	159.007800	.000000
-24.955330	60.363580	159.007800	.000000

Q.02a

-89.453400	32.991660	167.399200	.089242
-88.345930	33.191160	167.380100	.102157
-87.228100	33.381660	167.309400	.148441
-86.100230	33.562890	167.186900	.226036
-84.962690	33.734530	167.013500	.331480
-83.815900	33.896310	166.796700	.457825
-82.660300	34.047950	166.556000	.594413
-81.496410	34.189190	166.320300	.728122
-80.324730	34.319790	166.117100	.847862
-79.145840	34.439500	165.954000	.951239
-77.960310	34.548100	165.809500	1.045695
-76.768760	34.645400	165.671400	1.128786
-75.571840	34.731200	165.597900	1.166761
-74.370180	34.805370	165.695000	1.107946
-73.164480	34.867740	165.988700	.943803
-71.955430	34.918200	166.396000	.712353
-70.743730	34.956650	166.791800	.477919
-69.530080	34.983020	167.097700	.287828
-68.315220	34.997260	167.297400	.156870
-67.099880	34.999340	167.410500	.077466
-65.884770	34.989250	167.467000	.034553
-64.670640	34.967020	167.492100	.013798
-63.458190	34.932690	167.501800	.004851

-62.248150	34.886320	167.505200	.001450
-61.041230	34.828000	167.506200	.000337
-59.838120	34.757850	167.506400	.000064
-58.639500	34.676010	167.506400	.000060
-57.446040	34.582610	167.506400	.000048
-56.258390	34.477850	167.506400	.000041
-55.077160	34.361910	167.506400	.000031
-53.902950	34.235010	167.506400	.000031
-52.736340	34.097380	167.506400	.000031
-51.577860	33.949260	167.506400	.000031
-50.428010	33.790910	167.506400	.000031
-49.287250	33.622600	167.506400	.000031
-48.156040	33.444620	167.506400	.000031
-47.034740	33.257250	167.506400	.000031
-45.923690	33.060790	167.506400	.000031
-44.823220	32.855560	167.506400	.000031
-89.701210	33.918400	167.305500	.147701
-88.583520	34.125020	167.260400	.173858
-87.455040	34.322360	167.117600	.254282
-86.316120	34.510110	166.875100	.385720
-85.167100	34.687960	166.533800	.561344
-84.008420	34.855620	166.109000	.768640
-82.840550	35.012790	165.638400	.989580
-81.663990	35.159200	165.178600	1.203441
-80.479300	35.294580	164.790000	1.393517
-79.287040	35.418700	164.490600	1.559998
-78.087840	35.531310	164.208800	1.731630
-76.882320	35.632210	163.878100	1.921571
-75.671150	35.721200	163.636200	2.050784
-74.455030	35.798110	163.792200	1.982112
-73.234660	35.862810	164.413800	1.694359
-72.010770	35.915150	165.288700	1.268810
-70.784100	35.955040	166.120900	.833261
-69.555380	35.982390	166.738300	.488309
-68.325390	35.997170	167.124600	.259051
-67.094890	35.999320	167.335700	.125067
-65.864640	35.988860	167.437900	.054725
-64.635410	35.965800	167.482100	.021473
-63.407950	35.930180	167.498900	.007365
-62.183030	35.882080	167.504600	.002090
-60.961370	35.821590	167.506200	.000420
-59.743720	35.748840	167.506500	.000141
-58.530780	35.663950	167.506500	.000141
-57.323260	35.567100	167.506500	.000105
-56.121820	35.458470	167.506400	.000061
-54.927120	35.338260	167.506400	.000049
-53.739770	35.206700	167.506400	.000031
-52.560380	35.064020	167.506400	.000031
-51.389480	34.910500	167.506400	.000031
-50.227610	34.746390	167.506400	.000031
-49.075240	34.571990	167.506400	.000031
-47.932830	34.387580	167.506400	.000031
-46.800740	34.193490	167.506400	.000031
-45.679330	33.990000	167.506400	.000031
-44.568900	33.777440	167.506400	.000031
-89.960660	34.843320	167.021300	.309109
-88.832330	35.057210	166.895200	.371501
-87.692770	35.261540	166.535400	.542365
-86.542320	35.455950	165.935800	.813519
-85.381340	35.640140	165.101500	1.166829
-84.210270	35.813810	164.073300	1.572466
-83.029580	35.976620	162.938100	1.995885
-81.839790	36.128310	161.831000	2.402192
-80.641480	36.268600	160.906000	2.757315
-79.435230	36.397220	160.243600	3.066657
-78.221690	36.513940	159.564500	3.440449
-77.001530	36.618520	158.528700	3.977849

-75.775430	36.710780	157.568700	4.470507
-74.544130	36.790520	157.808000	4.402335
-73.308360	36.857590	159.458800	3.791477
-72.068890	36.911860	161.846500	2.834234
-70.826490	36.953220	164.065700	1.836480
-69.581950	36.981590	165.657200	1.050123
-68.336070	36.996900	166.611100	.543374
-67.089650	36.999130	167.113900	.257405
-65.843500	36.988290	167.351300	.111456
-64.598400	36.964370	167.452100	.043481
-63.355190	36.927440	167.490400	.014813
-62.114630	36.877570	167.503100	.004085
-60.877510	36.814860	167.506500	.000779
-59.644590	36.739430	167.507000	.000479
-58.416640	36.651430	167.506900	.000431
-57.194370	36.551040	167.506700	.000303
-55.978490	36.438440	167.506500	.000187
-54.769680	36.313860	167.506400	.000112
-53.568590	36.177530	167.506400	.000058
-52.375820	36.029710	167.506400	.000049
-51.191970	35.870660	167.506400	.000031
-50.017550	35.700660	167.506400	.000032
-48.853080	35.520030	167.506400	.000031
-47.698980	35.329070	167.506400	.000031
-46.555660	35.128100	167.506400	.000031
-45.423470	34.917440	167.506400	.000031
-44.302690	34.697410	167.506400	.000031
-90.232250	35.766320	166.401800	.617583
-89.092860	35.987640	166.079000	.754405
-87.941790	36.199090	165.221300	1.100863
-86.779330	36.400320	163.813200	1.634698
-85.605870	36.591000	161.881200	2.305272
-84.421870	36.770790	159.531600	3.043652
-83.227800	36.939400	156.946000	3.804426
-82.024180	37.096500	154.386300	4.534739
-80.811620	37.241800	152.322800	5.151335
-79.590740	37.375040	151.046500	5.618277
-78.362190	37.495960	149.622000	6.319566
-77.126670	37.604320	146.530300	7.770573
-75.884930	37.699920	142.647500	9.454988
-74.637700	37.782550	142.908900	9.422754
-73.385770	37.852060	147.236300	8.083058
-72.129940	37.908310	153.515100	6.087749
-70.871030	37.951180	159.286700	3.895858
-69.609860	37.980580	163.241700	2.179608
-68.347290	37.996460	165.510400	1.097002
-67.084140	37.998780	166.653900	.507923
-65.821270	37.987530	167.176100	.217039
-64.559530	37.962740	167.393200	.084091
-63.299760	37.924460	167.474500	.028333
-62.042780	37.872770	167.501100	.007555
-60.789440	37.807780	167.507900	.001633
-59.540510	37.729610	167.508700	.001468
-58.296810	37.638420	167.508100	.001245
-57.059080	37.534390	167.507400	.000865
-55.828080	37.417740	167.507000	.000534
-54.604500	37.288680	167.506700	.000308
-53.389020	37.147460	167.506500	.000162
-52.182290	36.994370	167.506400	.000085
-50.984880	36.829660	167.506400	.000053
-49.797380	36.653650	167.506400	.000036
-48.620270	36.466650	167.506400	.000035
-47.454020	36.268990	167.506400	.000035
-46.299010	36.060990	167.506400	.000031
-45.155610	35.843010	167.506400	.000031
-44.024070	35.615370	167.506400	.000031
-90.516530	36.687310	165.235100	1.122955

-89.365660	36.916220	164.491400	1.394112
-88.202590	37.134950	162.610900	2.041254
-87.027650	37.343150	159.565400	3.001984
-85.841200	37.540460	155.476600	4.131013
-84.643710	37.726540	150.611700	5.288625
-83.435650	37.901050	145.204400	6.496961
-82.217600	38.063690	139.799300	7.713930
-80.990140	38.214140	135.107200	8.836495
-79.753940	38.352110	133.674400	9.125773
-78.509670	38.477340	131.729000	9.976793
-77.258070	38.589580	123.432300	13.313050
-75.999910	38.688600	109.490900	18.706400
-74.735980	38.774210	109.468700	17.976180
-73.467090	38.846230	120.292300	15.102440
-72.194090	38.904510	135.682900	11.734080
-70.917830	38.948930	149.442700	7.516876
-69.639210	38.979390	158.588900	4.079919
-68.359090	38.995840	163.504800	1.991723
-67.078360	38.998240	165.858300	.897077
-65.797930	38.986590	166.884100	.378101
-64.518690	38.960900	167.299500	.145591
-63.241520	38.921240	167.452700	.048417
-61.967320	38.867690	167.501400	.012502
-60.696920	38.800350	167.513000	.003892
-59.431210	38.719360	167.513200	.003887
-58.170970	38.624900	167.511300	.003162
-56.917050	38.517150	167.509500	.002189
-55.670200	38.396340	167.508200	.001381
-54.431160	38.262690	167.507400	.000799
-53.200640	38.116470	167.506900	.000433
-51.979310	37.957960	167.506600	.000210
-50.767770	37.787460	167.506400	.000103
-49.566610	37.605300	167.506400	.000055
-48.376340	37.411790	167.506400	.000038
-47.197410	37.207270	167.506400	.000037
-46.030250	36.992090	167.506400	.000037
-44.875180	36.766620	167.506400	.000031
-43.732470	36.531200	167.506400	.000031
-90.814130	37.606180	163.311100	1.852203
-89.651300	37.842850	161.755300	2.342869
-88.475770	38.069030	157.934100	3.467449
-87.287830	38.284350	151.817400	5.057616
-86.087840	38.488450	143.881900	6.687444
-84.876290	38.680970	134.928800	7.957397
-83.653650	38.861550	124.934900	9.460578
-82.420500	39.029860	113.252600	11.835590
-81.177470	39.185590	103.869200	13.754910
-79.925230	39.328410	103.603700	12.785830
-78.664510	39.458070	106.206800	11.944760
-77.396060	39.574290	91.091020	16.790710
-76.120680	39.676830	63.442420	23.308110
-74.839220	39.765490	57.234930	22.687540
-73.552520	39.840080	72.585670	19.917830
-72.261480	39.900440	102.482600	18.880440
-70.967010	39.946460	132.210500	12.582680
-69.670030	39.978020	150.961900	6.701931
-68.371470	39.995060	160.512300	3.133624
-67.072270	39.997550	164.747500	1.371581
-65.773400	39.985470	166.491900	.578533
-64.475770	39.958870	167.182300	.223436
-63.180340	39.917780	167.434100	.073435
-61.888020	39.862300	167.511600	.019042
-60.599740	39.792560	167.526900	.009486
-59.316390	39.708680	167.524400	.009115
-58.038840	39.610860	167.519100	.007187
-56.767930	39.499290	167.514600	.005016
-55.504470	39.374200	167.511400	.003181

-54.249260	39.235840	167.509200	.001859
-53.003000	39.084480	167.507800	.001027
-51.766410	38.920440	167.507000	.000525
-50.540120	38.744010	167.506600	.000237
-49.324710	38.555530	167.506400	.000117
-48.120720	38.355340	167.506400	.000046
-46.928620	38.143810	167.506300	.000046
-45.748810	37.921300	167.506300	.000046
-44.581620	37.688170	167.506400	.000031
-43.427300	37.444810	167.506400	.000031
-91.125630	38.522790	160.570900	2.768826
-89.950410	38.767390	157.598000	3.596781
-88.761920	39.001200	150.281600	5.484890
-87.560450	39.223820	138.494400	8.012300
-86.346370	39.434880	123.667500	9.965467
-85.120160	39.633990	110.189400	9.631956
-83.882300	39.820790	94.434300	10.695330
-82.633390	39.994930	75.706180	13.346330
-81.374080	40.156070	63.000150	13.101060
-80.105060	40.303890	66.569310	11.703830
-78.827100	40.438090	81.940450	6.663530
-77.540980	40.558400	72.695590	8.165084
-76.247540	40.664560	37.467340	11.753800
-74.947690	40.756360	25.457250	11.093240
-73.642300	40.833600	29.623430	15.158340
-72.332310	40.896110	60.296920	21.074050
-71.018690	40.943760	109.382700	17.070810
-69.702430	40.976440	141.663100	9.236163
-68.384490	40.994090	157.115300	4.240488
-67.065880	40.996670	163.567500	1.837843
-65.747600	40.984170	166.053600	.799356
-64.430650	40.956610	167.073200	.311873
-63.116020	40.914060	167.440900	.100580
-61.804690	40.856610	167.546200	.029384
-60.497620	40.784390	167.559200	.021566
-59.195770	40.697540	167.548400	.019316
-57.900040	40.596260	167.535800	.014921
-56.611330	40.480750	167.526200	.010511
-55.330490	40.351270	167.518600	.006646
-54.058330	40.208070	167.513300	.003915
-52.795620	40.051450	167.510000	.002199
-51.543080	39.881710	167.508100	.001161
-50.301390	39.699200	167.507100	.000571
-49.071130	39.504250	167.506700	.000259
-47.852850	39.297230	167.506400	.000114
-46.647030	39.078520	167.506300	.000050
-45.454070	38.848490	167.506300	.000046
-44.274280	38.607530	167.506400	.000031
-43.107920	38.356030	167.506400	.000031
-91.451740	39.437030	157.350400	3.733505
-90.263630	39.689750	152.235500	5.040693
-89.061680	39.931370	139.378100	7.976211
-87.846150	40.161480	117.418100	11.866930
-86.617400	40.379670	90.129160	13.321520
-85.375900	40.585540	71.352690	7.730444
-84.122160	40.778730	56.105690	7.494810
-82.856800	40.958850	54.187390	5.289275
-81.580460	41.125550	28.592870	6.147980
-80.293880	41.278500	37.246580	5.620279
-78.997860	41.417370	68.746330	6.749280
-77.693230	41.541890	80.278560	10.500510
-76.380870	41.651780	41.089980	5.882007
-75.061690	41.746810	20.921100	4.707656
-73.736660	41.826770	28.328050	6.548686
-72.406780	41.891490	47.094860	19.642680
-71.073040	41.940830	94.706370	18.299980
-69.736500	41.974670	134.347400	10.889740

-68.398190	41.992950	153.963800	5.335314
-67.059160	41.995620	162.234500	2.449593
-65.720480	41.982670	165.555600	1.114372
-64.383220	41.954140	166.990500	.425743
-63.048400	41.910080	167.512100	.127126
-61.717090	41.850600	167.632100	.050850
-60.390290	41.775820	167.625600	.045705
-59.069010	41.685920	167.595300	.038614
-57.754210	41.581080	167.568400	.029240
-56.446830	41.461530	167.551100	.020731
-55.147770	41.327530	167.532700	.012760
-53.857880	41.179360	167.521500	.007419
-52.577970	41.017320	167.514600	.004224
-51.308780	40.841740	167.510500	.002322
-50.051000	40.652980	167.508300	.001193
-48.805250	40.451390	167.507100	.000561
-47.572090	40.237360	167.506600	.000240
-46.352010	40.011290	167.506400	.000102
-45.145370	39.773560	167.506300	.000050
-43.952510	39.524580	167.506400	.000031
-42.773630	39.264750	167.506400	.000031
-91.793200	40.348750	154.632100	4.480288
-90.591710	40.609810	147.506900	6.052182
-89.375760	40.859430	129.065500	9.532247
-88.145600	41.097210	95.391960	14.244770
-86.901570	41.322720	55.831410	12.501900
-85.644150	41.535550	28.966280	4.974376
-84.373860	41.735290	37.775810	6.197124
-83.091290	41.921560	25.704440	3.451432
-81.797160	42.093980	28.809600	7.362461
-80.492230	42.252200	33.999420	5.700780
-79.177290	42.395880	62.241100	8.100013
-77.853240	42.524730	64.460400	7.811967
-76.521000	42.638450	37.734870	7.737999
-75.181550	42.736820	23.696020	5.379939
-73.835900	42.819590	43.596950	4.428720
-72.485100	42.886590	66.107320	13.742470
-71.130210	42.937670	99.180890	17.100000
-69.772340	42.972710	132.230400	11.548440
-68.412590	42.991630	150.712600	6.654185
-67.052090	42.994400	160.183400	3.418067
-65.691960	42.980990	164.879500	1.523206
-64.333320	42.951450	167.072400	.511143
-62.977290	42.905830	167.764600	.141317
-61.624970	42.844260	167.828300	.107408
-60.277440	42.766850	167.751700	.095457
-58.935750	42.673790	167.680600	.077094
-57.600940	42.565280	167.629700	.058351
-56.273990	42.441570	167.596400	.039966
-54.955840	42.302930	167.561400	.023305
-53.647390	42.149640	167.537000	.013110
-52.349470	41.982030	167.523400	.007517
-51.062890	41.800450	167.515100	.004247
-49.788320	41.605260	167.510500	.002263
-48.526440	41.396860	167.508100	.001093
-47.277780	41.175640	167.507000	.000478
-46.042850	40.942000	167.506600	.000195
-44.822010	40.696380	167.506400	.000081
-43.615560	40.439180	167.506400	.000031
-42.423690	40.170830	167.506400	.000031
-92.150770	41.257820	153.601500	4.737792
-90.935390	41.527410	146.431500	6.055548
-89.704910	41.785250	127.879200	8.746782
-88.459540	42.030900	93.196540	12.806650
-87.199620	42.263930	48.739150	12.481140
-85.925610	42.483890	25.780370	6.684711
-84.638040	42.690380	22.271560	6.646788

-83.337520	42.882970	32.983420	7.461960
-82.024790	43.061280	28.006750	4.913031
-80.700610	43.224930	61.501070	7.161483
-79.365870	43.373580	67.878620	8.070069
-78.021450	43.506890	58.682540	8.616720
-76.668370	43.624570	15.867160	3.218991
-75.307630	43.726360	29.962260	5.662331
-73.940300	43.812040	48.966600	4.812990
-72.567500	43.881390	74.671650	11.482810
-71.190370	43.934270	100.847000	16.914190
-69.810050	43.970550	127.046700	13.955790
-68.427750	43.990140	147.802900	8.063700
-67.044650	43.993000	159.366000	3.863961
-65.661930	43.979120	165.049700	1.593741
-64.280810	43.948540	167.800000	.438819
-62.902460	43.901310	168.430700	.281699
-61.528050	43.837570	168.238800	.253059
-60.158730	43.757450	167.981000	.203592
-58.795620	43.661140	167.845600	.160874
-57.439790	43.548850	167.790700	.123335
-56.092310	43.420850	167.695600	.078908
-54.754150	43.277410	167.614600	.041514
-53.426270	43.118850	167.565900	.022137
-52.109530	42.945500	167.539300	.012842
-50.804760	42.757750	167.523500	.007336
-49.512690	42.555960	167.514500	.003959
-48.233980	42.340550	167.509900	.001950
-46.969190	42.111940	167.507700	.000879
-45.718810	41.870550	167.506800	.000361
-44.483210	41.616830	167.506500	.000136
-43.262650	41.351200	167.506400	.000049
-42.057300	41.074100	167.506400	.000031
-92.525320	42.164070	153.913400	4.841843
-91.295520	42.442420	146.685400	6.496233
-90.049940	42.708690	128.265300	10.058990
-88.788760	42.962440	96.050830	14.238960
-87.512310	43.203190	54.278740	16.460510
-86.221000	43.430500	29.299660	9.916892
-84.915410	43.643920	23.454280	5.056908
-83.596150	43.843030	21.465750	6.913791
-82.263960	44.027400	46.385930	10.234620
-80.919660	44.196660	72.958650	10.528310
-79.564150	44.350410	73.920110	10.069490
-78.198390	44.488330	53.324070	4.838583
-76.823410	44.610100	30.028760	5.697687
-75.440290	44.715440	14.057440	2.853182
-74.050180	44.804110	33.453080	4.909216
-72.654240	44.875890	52.940020	13.719310
-71.253690	44.930630	86.149270	20.217820
-69.849760	44.968180	120.621000	16.412220
-68.443720	44.988460	144.844000	9.291068
-67.036810	44.991420	158.317700	4.417130
-65.630330	44.977050	165.297400	1.633567
-64.225530	44.945390	168.464600	.569012
-62.823690	44.896510	169.084800	.523346
-61.426040	44.830540	168.699600	.419237
-60.033810	44.747610	168.321600	.320816
-58.648170	44.647940	168.119000	.248670
-57.270290	44.531750	167.961500	.175937
-55.901260	44.399320	167.829000	.109994
-54.542130	44.250930	167.702100	.063272
-53.193900	44.086940	167.615100	.036629
-51.857480	43.907680	167.565500	.021516
-50.533710	43.713560	167.536800	.012124
-49.223370	43.504980	167.520800	.006398
-47.927120	43.282370	167.512600	.003153
-46.645540	43.046150	167.508700	.001423

-45.379100	42.796800	167.507200	.000573
-44.128160	42.534760	167.506600	.000210
-42.892960	42.260480	167.506400	.000072
-41.673610	41.974410	167.506400	.000036
-92.917770	43.067330	155.443300	4.577358
-91.673000	43.354680	148.922000	6.289223
-90.411740	43.629630	132.130600	10.109460
-89.134110	43.891690	101.996400	15.761770
-87.840450	44.140390	60.670990	19.135760
-86.531140	44.375260	24.485020	16.967720
-85.206750	44.595830	24.641820	6.946797
-83.867900	44.801650	27.252520	7.595779
-82.515380	44.992280	35.507140	9.489922
-81.150000	45.167310	56.036940	9.896687
-79.772730	45.326340	51.117220	6.826919
-78.384560	45.469020	51.542090	5.417610
-76.986600	45.595010	37.044590	4.306780
-75.579970	45.704020	25.145860	4.157864
-74.165890	45.795780	29.679870	3.911376
-72.745610	45.870080	57.125580	12.176650
-71.320400	45.926740	93.307930	17.130640
-69.891600	45.965610	121.069800	15.300530
-68.460530	45.986610	140.727500	10.780050
-67.028560	45.989670	154.360200	5.953383
-65.597030	45.974800	162.834400	2.527404
-64.167300	45.942020	167.048900	.860416
-62.740720	45.891430	168.387300	.440497
-61.318600	45.823140	168.586500	.374694
-59.902260	45.737310	168.280300	.271012
-58.492950	45.634170	168.095800	.208810
-57.091900	45.513950	167.977400	.166079
-55.700260	45.376930	167.864300	.131718
-54.319140	45.223450	167.771500	.103660
-52.949590	45.053840	167.683700	.065659
-51.592580	44.868490	167.606600	.036527
-50.248970	44.667810	167.556500	.019125
-48.919560	44.452230	167.529400	.009607
-47.605040	44.222190	167.516100	.004585
-46.305980	43.978160	167.510100	.002047
-45.022840	43.720610	167.507700	.000833
-43.755960	43.450020	167.506800	.000303
-42.505550	43.166850	167.506400	.000105
-41.271660	42.871580	167.506400	.000056
-93.329110	43.967430	157.742800	3.963996
-92.068780	44.264030	152.543800	5.438722
-90.791210	44.547890	139.211900	8.722810
-89.496510	44.818510	114.365700	13.304530
-88.184940	45.075390	74.103200	18.867200
-86.856880	45.318050	31.459520	20.242040
-85.512890	45.545990	16.325820	12.606450
-84.153590	45.758730	27.923130	5.568324
-82.779790	45.955820	34.053480	4.831971
-81.392350	46.136820	28.535810	5.956078
-79.992260	46.301310	29.157250	6.298115
-78.580580	46.448910	41.232520	7.711185
-77.158460	46.579270	37.710270	6.844855
-75.727110	46.692070	39.082520	8.106048
-74.287820	46.787040	51.761770	9.536192
-72.841890	46.863950	86.277580	11.674640
-71.390720	46.922590	111.112400	12.686450
-69.935700	46.962830	125.250800	13.878460
-68.478260	46.984570	137.074400	12.249130
-67.019850	46.987740	148.877300	8.115767
-65.561920	46.972340	157.839900	4.446208
-64.105920	46.938410	163.339500	2.167794
-62.653260	46.886040	166.134500	1.042256
-61.205390	46.815350	167.174400	.608064

-59.763680	46.726520	167.494100	.429130
-58.329470	46.619790	167.622500	.324784
-56.904060	46.495390	167.883800	.271264
-55.488670	46.353640	168.080700	.264962
-54.084500	46.194880	168.102200	.224088
-52.692630	46.019470	167.870400	.130319
-51.314070	45.827840	167.679200	.061885
-49.949730	45.620390	167.582800	.028309
-48.600430	45.397590	167.539000	.013076
-47.266860	45.159900	167.519500	.005970
-45.949600	44.907820	167.511300	.002615
-44.649100	44.641840	167.508000	.001056
-43.365670	44.362450	167.506900	.000381
-42.099450	44.070140	167.506500	.000130
-40.850440	43.765410	167.506400	.000052
-93.760410	44.864160	160.247300	3.157110
-92.483920	45.170280	156.500600	4.334507
-91.189420	45.463320	146.960500	6.924776
-89.876960	45.742750	127.905000	11.321850
-88.546760	46.008070	93.599160	18.565150
-87.199170	46.258760	51.860260	25.312210
-85.834720	46.494300	31.114360	23.032280
-84.454070	46.714200	36.196120	16.563200
-83.058010	46.917960	42.421230	12.835110
-81.647480	47.105130	36.432280	14.792130
-80.223460	47.275260	25.325410	17.580420
-78.787090	47.427960	29.702430	16.030110
-77.339580	47.562840	36.377320	17.503260
-75.882230	47.679570	50.097940	17.636630
-74.416370	47.777870	82.845700	16.380570
-72.943440	47.857470	113.876700	11.333820
-71.464880	47.918190	129.467100	9.777322
-69.982220	47.959850	136.027200	10.749720
-68.496960	47.982350	140.318200	10.879750
-67.010670	47.985630	147.072900	8.664301
-65.524890	47.969690	153.933900	5.924896
-64.041170	47.934570	158.912300	3.890699
-62.561040	47.880340	161.946800	2.648993
-61.086020	47.807170	163.508800	1.970897
-59.617590	47.715230	164.697800	1.428081
-58.157170	47.604770	166.159300	.811250
-56.706150	47.476050	167.567400	.362966
-55.265830	47.329400	168.348900	.357866
-53.837470	47.165180	168.470000	.351777
-52.422200	46.983780	168.100300	.207570
-51.021090	46.785640	167.757400	.088611
-49.635090	46.571200	167.601900	.035388
-48.265020	46.340950	167.543900	.015053
-46.911610	46.095370	167.521100	.006655
-45.575410	45.834990	167.511800	.002877
-44.256860	45.560320	167.508200	.001170
-42.956210	45.271870	167.506900	.000423
-41.673580	44.970160	167.506500	.000156
-40.408870	44.655690	167.506400	.000058
-94.212860	45.757310	162.501400	2.327091
-92.919560	46.073230	160.065800	3.173845
-91.607470	46.375730	154.041700	5.060099
-90.276550	46.664250	142.035200	8.477504
-88.926960	46.938270	119.139800	15.262290
-87.559010	47.197250	85.000690	25.800770
-86.173240	47.440650	60.002440	31.824310
-84.770290	47.667940	54.188760	30.622290
-83.350960	47.878600	58.272720	26.579290
-81.916200	48.072160	56.174510	23.421760
-80.467090	48.248140	50.605910	23.521190
-79.004780	48.406110	54.566250	23.035200
-77.530590	48.545680	66.108860	21.804000

-76.045850 48.666500 90.317670 19.196720
-74.552020 48.768250 114.944200 14.635040
-73.050600 48.850660 134.011800 9.907237
-71.543170 48.913520 144.878200 6.977840
-70.031330 48.956650 148.349500 6.818724
-68.516710 48.979950 149.854600 7.042972
-67.000980 48.983350 152.649900 6.179083
-65.485790 48.966840 155.917200 4.930657
-63.972820 48.930470 158.504400 3.964959
-62.463700 48.874330 159.861300 3.435163
-60.960050 48.798580 160.518400 3.148305
-59.463460 48.703410 161.510300 2.690503
-57.975440 48.589080 163.773500 1.719749
-56.497490 48.455880 166.509300 .623699
-55.030970 48.304140 167.968800 .261631
-53.577210 48.134270 168.234300 .269974
-52.137430 47.946660 167.956600 .163499
-50.712720 47.741790 167.674700 .070382
-49.304080 47.520120 167.575000 .029434
-47.912350 47.282170 167.536100 .013264
-46.538250 47.028450 167.518700 .006035
-45.182340 46.759510 167.511000 .002697
-43.845020 46.475880 167.507900 .001102
-42.526500 46.178100 167.506800 .000406
-41.226800 45.866700 167.506400 .000135
-39.945740 45.542200 167.506400 .000049
-94.687740 46.646660 164.278100 1.607225
-93.376940 46.972690 162.865800 2.136813
-92.046550 47.284930 159.608700 3.275682
-90.696440 47.582840 152.587300 5.689411
-89.326680 47.865840 139.692900 10.339280
-87.937550 48.133390 120.094300 18.382400
-86.529520 48.384910 98.887890 27.068280
-85.103260 48.619850 88.851050 29.669090
-83.659580 48.837660 88.288310 26.228280
-82.199460 49.037830 91.783560 22.105280
-80.724000 49.219880 94.325580 19.885920
-79.234450 49.383340 99.498740 19.657860
-77.732160 49.527770 112.229400 16.761860
-76.218590 49.652830 124.155600 14.514950
-74.695260 49.758160 136.089600 11.281940
-73.163800 49.843490 145.761200 8.124990
-71.625870 49.908580 152.692200 5.656971
-70.083210 49.953250 155.898800 4.595845
-68.537570 49.977380 157.483700 4.116003
-66.990740 49.980900 159.334300 3.408690
-65.444490 49.963800 160.911700 2.843537
-63.900620 49.926140 161.965900 2.495195
-62.360880 49.868010 162.300000 2.398713
-60.827010 49.789570 162.061600 2.472987
-59.300730 49.691040 162.267500 2.346585
-57.783640 49.572690 163.970800 1.617671
-56.277320 49.434830 166.150200 .704461
-54.783270 49.277820 167.559600 .199308
-53.302860 49.102080 167.909000 .163928
-51.837360 48.908040 167.730500 .087150
-50.387950 48.696200 167.568300 .044955
-48.955640 48.467040 167.532400 .021332
-47.541310 48.221130 167.523600 .009175
-46.145660 47.959000 167.514700 .004579
-44.769230 47.681210 167.509700 .002164
-43.412380 47.388330 167.507500 .000915
-42.075270 47.080920 167.506700 .000340
-40.757810 46.759540 167.506400 .000121
-39.459720 46.424710 167.506400 .000049
-95.186440 47.531940 165.583700 1.047563
-93.857410 47.868410 164.923500 1.308791

-92.508000	48.190720	163.483600	1.820233
-91.137910	48.498310	160.142300	3.067846
-89.747150	48.790600	153.741800	5.572344
-88.335950	49.067010	144.286400	9.641380
-86.904710	49.326930	133.793700	14.532380
-85.454100	49.569790	125.691500	17.301320
-83.984930	49.795020	125.629100	15.070590
-82.498220	50.002060	126.641500	13.387150
-80.995120	50.190400	131.124500	12.195190
-79.476920	50.359550	135.202500	11.624380
-77.945060	50.509060	140.034000	10.599580
-76.401090	50.638520	145.120400	9.168223
-74.846650	50.747580	149.565100	7.644886
-73.283450	50.835950	153.879800	5.894139
-71.713320	50.903350	157.292900	4.424416
-70.138080	50.949630	159.292600	3.562419
-68.559640	50.974620	161.165100	2.812181
-66.979900	50.978270	163.034300	2.026542
-65.400800	50.960560	164.200100	1.538060
-63.824260	50.921550	164.704900	1.322730
-62.252170	50.861340	164.967900	1.217632
-60.686400	50.780110	164.730000	1.302051
-59.128770	50.678080	164.757900	1.266755
-57.581030	50.555550	165.459100	.943586
-56.044860	50.412850	166.842900	.384165
-54.521820	50.250360	167.575700	.144320
-53.013420	50.068520	167.772600	.126758
-51.520960	49.867810	167.703600	.077554
-50.045680	49.648740	167.597300	.032164
-48.588630	49.411830	167.533100	.012396
-47.150700	49.157670	167.519500	.006351
-45.732590	48.886830	167.512500	.003362
-44.334810	48.599900	167.508800	.001614
-42.957660	48.297460	167.507100	.000677
-41.601190	47.980120	167.506500	.000251
-40.265250	47.648430	167.506400	.000083
-38.949390	47.302930	167.506400	.000041
-95.710510	48.412890	166.569200	.631667
-94.362470	48.760130	166.454600	.696202
-92.993230	49.092850	166.100000	.778694
-91.602360	49.410460	164.707400	1.290737
-90.189750	49.712350	161.847600	2.431932
-88.755540	49.997940	157.933300	4.104851
-87.300090	50.266570	153.481300	6.068977
-85.824040	50.517650	150.141900	7.346136
-84.328210	50.750570	148.954400	7.025679
-82.813610	50.964740	149.496600	6.774965
-81.281460	51.159630	151.440800	6.366616
-79.733130	51.334700	153.169500	6.077977
-78.170130	51.489480	155.017000	5.606508
-76.594090	51.623530	156.646800	5.024007
-75.006800	51.736490	157.729300	4.532340
-73.410070	51.828010	159.140400	3.842436
-71.805870	51.897850	160.468000	3.187362
-70.196170	51.945790	161.389100	2.777417
-68.582990	51.971680	162.525500	2.314689
-66.968440	51.975460	164.396900	1.557160
-65.354560	51.957120	165.618800	.989520
-63.743440	51.916690	166.012900	.763456
-62.137130	51.854320	166.018400	.725817
-60.537630	51.770170	166.121900	.669623
-58.946900	51.664510	166.265200	.596662
-57.366820	51.537620	166.712300	.391223
-55.799180	51.389870	167.208200	.176775
-54.245650	51.221680	167.514500	.072794
-52.707820	51.033510	167.651200	.072917
-51.187080	50.825860	167.644200	.061265

-49.684720	50.599280	167.566800	.024242
-48.201800	50.354330	167.534100	.010668
-46.739240	50.091630	167.518400	.005169
-45.297720	49.811770	167.511200	.002498
-43.877700	49.515370	167.508100	.001150
-42.479410	49.203060	167.506900	.000467
-41.102810	48.875440	167.506400	.000175
-39.747590	48.533100	167.506400	.000065
-38.413150	48.176570	167.506400	.000031
-96.261670	49.289190	167.390000	.354194
-94.893750	49.647600	167.560700	.371538
-93.503820	49.991080	167.580200	.320100
-92.091320	50.319060	166.910900	.434261
-90.655950	50.630910	165.596000	.935701
-89.197760	50.925990	163.835800	1.672122
-87.717060	51.203660	162.057400	2.435527
-86.214420	51.463270	160.776800	2.964949
-84.690660	51.704190	160.068600	3.156832
-83.146820	51.925780	160.089500	3.180113
-81.584160	52.127480	160.747500	3.046935
-80.004110	52.308710	161.496300	2.898600
-78.408280	52.468990	162.186800	2.655363
-76.798410	52.607840	162.506800	2.470245
-75.176390	52.724850	162.234000	2.509160
-73.544200	52.819690	162.039500	2.506996
-71.903920	52.892050	162.521800	2.257265
-70.257710	52.941730	162.699600	2.178259
-68.607750	52.968570	163.232600	2.002190
-66.956280	52.972490	164.625400	1.460449
-65.305560	52.953480	165.944400	.851306
-63.657820	52.911580	166.378000	.606639
-62.015270	52.846950	166.555300	.499306
-60.380080	52.759750	166.687900	.422581
-58.754360	52.650280	166.956100	.279665
-57.140120	52.518850	167.176000	.168013
-55.539290	52.365840	167.358200	.085224
-53.953660	52.191710	167.486800	.036595
-52.384890	51.996940	167.549900	.026734
-50.834480	51.782070	167.559900	.024255
-49.303730	51.547700	167.541200	.014575
-47.793770	51.294400	167.524400	.007579
-46.305490	51.022820	167.514300	.003616
-44.839570	50.733620	167.509600	.001745
-43.396390	50.427420	167.507500	.000768
-41.976090	50.104880	167.506700	.000303
-40.578500	49.766630	167.506400	.000106
-39.203150	49.413260	167.506300	.000046
-37.849210	49.045330	167.506400	.000031
-96.841810	50.160530	167.779100	.236555
-95.453030	50.530490	167.943300	.277552
-94.041510	50.885130	167.987500	.247856
-92.606450	51.223850	167.582400	.182610
-91.147380	51.546020	166.936400	.382211
-89.664200	51.850960	166.197000	.692003
-88.157130	52.138020	165.493600	.995560
-86.626690	52.406500	164.951000	1.224384
-85.073680	52.655720	164.631300	1.359029
-83.499180	52.885050	164.591100	1.408149
-81.904440	53.093850	164.861300	1.352547
-80.290990	53.281520	165.265900	1.226414
-78.660530	53.447530	165.645600	1.052723
-77.014920	53.591380	165.542300	1.040226
-75.356160	53.712650	164.641600	1.354997
-73.686420	53.810940	163.628100	1.742518
-72.007930	53.885960	163.539400	1.767977
-70.322990	53.937460	163.617800	1.741270
-68.634010	53.965280	163.655600	1.779862

-66.943400	53.969350	164.595700	1.421837
-65.253590	53.949630	165.786400	.888346
-63.567000	53.906200	166.514300	.541388
-61.886040	53.839190	166.789500	.396384
-60.213040	53.748820	167.064200	.242410
-58.550290	53.635360	167.248000	.138009
-56.899960	53.499180	167.355000	.080193
-55.264110	53.340690	167.435700	.042130
-53.644650	53.160350	167.489700	.019346
-52.043340	52.958710	167.518400	.010864
-50.461760	52.736320	167.526100	.009410
-48.901280	52.493820	167.522200	.007015
-47.363030	52.231850	167.515500	.004109
-45.847900	51.951060	167.510900	.002225
-44.356530	51.652150	167.508300	.001081
-42.889220	51.335790	167.507000	.000459
-41.445980	51.002650	167.506600	.000175
-40.026490	50.653380	167.506400	.000063
-38.630060	50.288590	167.506400	.000048
-37.255620	49.908830	167.506400	.000031
-97.453060	51.026530	167.737500	.133230
-96.042340	51.408490	167.848100	.161277
-94.608220	51.774700	167.812200	.135672
-93.149600	52.124570	167.653300	.124556
-91.665800	52.457450	167.429100	.187728
-90.156560	52.772640	167.164300	.292778
-88.621980	53.069450	166.901300	.403321
-87.062480	53.347160	166.685300	.493992
-85.478850	53.605050	166.548600	.553948
-83.872130	53.842430	166.519700	.577628
-82.243660	54.058630	166.640300	.538220
-80.595020	54.253030	166.929200	.444530
-78.928000	54.425040	167.248100	.347680
-77.244600	54.574130	167.074200	.379910
-75.546970	54.699840	165.994900	.723754
-73.837420	54.801750	164.670400	1.251662
-72.118360	54.879540	164.182400	1.469522
-70.392330	54.932960	164.322800	1.429013
-68.661900	54.961820	164.241000	1.476922
-66.929700	54.966030	164.839700	1.256562
-65.198370	54.945580	165.846000	.812392
-63.470540	54.900540	166.616300	.471949
-61.748820	54.831050	167.058900	.255962
-60.035740	54.737340	167.272900	.133666
-58.333780	54.619720	167.375900	.073240
-56.645260	54.478570	167.434600	.040746
-54.972420	54.314330	167.472200	.021543
-53.317280	54.127510	167.496400	.010099
-51.681740	53.918690	167.509500	.004974
-50.067420	53.688470	167.513900	.003930
-48.475770	53.437500	167.513000	.003139
-46.907930	53.166490	167.510700	.002114
-45.364770	52.876130	167.508600	.001200
-43.846840	52.567130	167.507400	.000603
-42.354370	52.240220	167.506700	.000258
-40.887210	51.896080	167.506500	.000094
-39.444820	51.535370	167.506400	.000045
-38.026250	51.158730	167.506400	.000031
-36.630120	50.766700	167.506400	.000031
-98.097820	51.886770	167.636700	.062717
-96.663910	52.281200	167.661000	.067223
-95.206040	52.659450	167.651800	.064240
-93.722790	53.020900	167.610900	.070863
-92.213200	53.364910	167.546200	.096474
-90.676770	53.690770	167.470000	.134965
-89.113440	53.997730	167.394700	.175144
-87.523560	54.285050	167.340700	.210025

-85.907830	54.551970	167.314000	.232304
-84.267300	54.797760	167.322100	.237177
-82.603340	55.021710	167.398500	.218522
-80.917590	55.223140	167.598300	.210380
-79.211960	55.401440	167.917400	.299973
-77.488560	55.556020	167.817300	.331470
-75.749730	55.686380	166.791500	.411283
-73.997920	55.792100	165.352400	.947787
-72.235800	55.872810	164.470500	1.343537
-70.466090	55.928230	164.519300	1.339971
-68.691570	55.958180	164.813500	1.219199
-66.915130	55.962550	165.330000	.999351
-65.139640	55.941330	166.212200	.620942
-63.367970	55.894590	166.861500	.328337
-61.602940	55.822490	167.229100	.155535
-59.847310	55.725280	167.374200	.077773
-58.103760	55.603290	167.436400	.041181
-56.374820	55.456930	167.469800	.022235
-54.662870	55.286670	167.489300	.011493
-52.970100	55.093080	167.500800	.005319
-51.298500	54.876750	167.506900	.002349
-49.649800	54.638340	167.509100	.001627
-48.025460	54.378550	167.509100	.001387
-46.426660	54.098120	167.508200	.000988
-44.854210	53.797780	167.507400	.000584
-43.308580	53.478290	167.506800	.000286
-41.789850	53.140400	167.506600	.000131
-40.297680	52.784830	167.506400	.000062
-38.831280	52.412250	167.506400	.000031
-37.389370	52.023280	167.506400	.000031
-35.970190	51.618480	167.506400	.000031
-98.778840	52.740810	167.576400	.030665
-97.320270	53.148240	167.578700	.030676
-95.837370	53.539010	167.576300	.030734
-94.328290	53.912530	167.564000	.033647
-92.791720	54.268120	167.544000	.042500
-91.226900	54.605080	167.522700	.057337
-89.633560	54.922620	167.508400	.075588
-88.011890	55.219970	167.505100	.093061
-86.362530	55.496330	167.517800	.107568
-84.686490	55.750910	167.554500	.119309
-82.985150	55.982970	167.634800	.137834
-81.260250	56.191770	167.824100	.206486
-79.513790	56.376650	168.154800	.364644
-77.748020	56.536990	168.177900	.423956
-75.965460	56.672260	167.239300	.295021
-74.168790	56.781970	165.763900	.783020
-72.360860	56.865740	164.530300	1.332131
-70.544630	56.923280	164.413700	1.384643
-68.723180	56.954370	165.024600	1.124868
-66.899620	56.958910	165.839900	.765727
-65.077100	56.936880	166.578200	.435767
-63.258730	56.888360	167.047200	.221044
-61.447630	56.813510	167.291100	.107847
-59.646790	56.712620	167.418500	.050877
-57.859090	56.586040	167.463400	.025839
-56.087300	56.434210	167.485300	.013281
-54.333970	56.257640	167.497000	.006578
-52.601470	56.056940	167.503200	.002941
-50.891880	55.832740	167.506100	.001160
-49.207030	55.585770	167.507300	.000647
-47.548420	55.316760	167.507300	.000569
-45.917200	55.026500	167.507100	.000425
-44.314130	54.715760	167.506700	.000254
-42.739580	54.385350	167.506600	.000132
-41.193420	54.036030	167.506400	.000059
-39.675070	53.668560	167.506400	.000031

-38.183400 53.283620 167.506400 .000031
-36.716770 52.881820 167.506400 .000031
-35.272900 52.463700 167.506400 .000031
-99.499240 53.588130 167.541600 .015522
-98.014280 54.009130 167.541100 .015177
-96.504860 54.412960 167.540100 .015237
-94.968610 54.799050 167.536700 .016181
-93.403790 55.166730 167.531300 .019095
-91.809290 55.515250 167.525800 .024292
-90.184560 55.843850 167.523400 .031238
-88.529620 56.151670 167.526100 .038339
-86.844990 56.437890 167.538700 .045797
-85.131650 56.701690 167.572000 .058504
-83.390960 56.942230 167.649800 .092463
-81.624720 57.158780 167.817800 .179483
-79.835050 57.350580 168.141200 .347863
-78.024370 57.516980 168.233900 .429351
-76.195360 57.657400 167.389400 .243546
-74.350940 57.771310 165.968400 .704403
-72.494220 57.858320 164.590200 1.319663
-70.628410 57.918090 164.309500 1.440341
-68.756890 57.950390 165.114000 1.079981
-66.883060 57.955100 166.163800 .615254
-65.010370 57.932220 166.826000 .313618
-63.142240 57.881810 167.142700 .166072
-61.282040 57.804080 167.325100 .085993
-59.433060 57.699310 167.424100 .041846
-57.598450 57.567890 167.473400 .019241
-55.781200 57.410300 167.491800 .009165
-53.984060 57.227100 167.500200 .004227
-52.209560 57.018940 167.504100 .001798
-50.459930 56.786500 167.505900 .000648
-48.737060 56.530560 167.506500 .000256
-47.042490 56.251900 167.506700 .000204
-45.377330 55.951360 167.506600 .000169
-43.742270 55.629770 167.506500 .000103
-42.137470 55.287960 167.506300 .000065
-40.562610 54.926740 167.506300 .000046
-39.016750 54.546860 167.506300 .000046
-37.498410 54.149020 167.506300 .000046
-36.005400 53.733830 167.506400 .000031
-34.534880 53.301770 167.506400 .000031
-100.262700 54.428120 167.523000 .007804
-98.749250 54.863350 167.522300 .007514
-97.211560 55.280850 167.521900 .007499
-95.646590 55.680060 167.521100 .007858
-94.052090 56.060360 167.520000 .008893
-92.426510 56.420970 167.519100 .010790
-90.768940 56.761100 167.519500 .013425
-89.079190 57.079890 167.522900 .016663
-87.357570 57.376450 167.532700 .021746
-85.605000 57.649890 167.558400 .034411
-83.822840 57.899360 167.623200 .069375
-82.012920 58.124030 167.768400 .150162
-80.177480 58.323120 168.037400 .297222
-78.319110 58.495910 168.144700 .379199
-76.440710 58.641750 167.351500 .181410
-74.545430 58.760110 166.074700 .650242
-72.636660 58.850530 164.760700 1.245477
-70.717930 58.912650 164.286300 1.445731
-68.792920 58.946230 165.125800 1.068280
-66.865380 58.951130 166.291400 .554414
-64.939080 58.927340 166.933800 .261976
-63.017780 58.874950 167.209400 .134525
-61.105210 58.794160 167.346100 .073800
-59.204930 58.685300 167.427900 .038837
-57.320400 58.548780 167.474000 .017600

-55.454840 58.385130 167.493400 .007580
-53.611270 58.194950 167.501400 .003185
-51.792370 57.978940 167.504600 .001273
-50.000500 57.737840 167.505800 .000455
-48.237630 57.472490 167.506300 .000151
-46.505310 57.183730 167.506300 .000077
-44.804610 56.872430 167.506300 .000069
-43.136050 56.539500 167.506300 .000046
-41.499610 56.185780 167.506300 .000039
-39.894630 55.812120 167.506300 .000039
-38.319800 55.419290 167.506300 .000040
-36.773090 55.007980 167.506300 .000041
-35.251730 54.578750 167.506400 .000031
-33.752120 54.132060 167.506400 .000031
-101.073700 55.260130 167.513600 .003751
-99.529010 55.710320 167.513100 .003555
-97.960900 56.142140 167.512900 .003531
-96.365430 56.555100 167.512800 .003696
-94.739640 56.948590 167.512700 .004125
-93.081440 57.321860 167.512800 .004888
-91.389490 57.674070 167.513400 .006091
-89.663250 58.004340 167.515800 .008121
-87.902820 58.311730 167.523000 .012835
-86.109000 58.595320 167.543700 .025452
-84.283100 58.854170 167.599600 .058429
-82.427000 59.087400 167.724200 .128488
-80.543050 59.294160 167.947100 .248590
-78.634010 59.473690 168.033300 .306201
-76.703000 59.625280 167.301900 .142931
-74.753460 59.748340 166.301400 .561762
-72.789070 59.842370 165.180200 1.063818
-70.813740 59.906970 164.481900 1.354192
-68.831480 59.941910 165.062900 1.091362
-66.846440 59.947010 166.261100 .567442
-64.862770 59.922250 166.954700 .253593
-62.884610 59.867760 167.248900 .118711
-60.916040 59.783740 167.372200 .061894
-58.961010 59.670550 167.438300 .033186
-57.023270 59.528640 167.474700 .016423
-55.106350 59.358580 167.494300 .006783
-53.213500 59.161040 167.502100 .002604
-51.347600 58.936760 167.504800 .000978
-49.511150 58.686560 167.505800 .000350
-47.706190 58.411320 167.506200 .000133
-45.934240 58.111950 167.506300 .000046
-44.196280 57.789400 167.506300 .000031
-42.492650 57.444580 167.506300 .000038
-40.823030 57.078400 167.506300 .000038
-39.186360 56.691730 167.506300 .000035
-37.580830 56.285340 167.506300 .000036
-36.003750 55.859910 167.506300 .000039
-34.451600 55.415950 167.506400 .000031
-32.919830 54.953830 167.506400 .000031
-101.937200 56.083360 167.509200 .001681
-100.358100 56.549350 167.508900 .001554
-98.756940 56.996240 167.508900 .001533
-97.128790 57.423640 167.508800 .001606
-95.469860 57.830970 167.508900 .001795
-93.777320 58.217490 167.508800 .002165
-92.049300 58.582370 167.509100 .002907
-90.284800 58.924680 167.510300 .004621
-88.483640 59.243460 167.515300 .008945
-86.646400 59.537720 167.531400 .020115
-84.774360 59.806450 167.577600 .048619
-82.869400 60.048700 167.675900 .103429
-80.933970 60.263580 167.838100 .184968
-78.971000 60.450230 167.827600 .188437

-76.983890 60.607900 167.270200 .153870
-74.976370 60.735940 166.589000 .454263
-72.952470 60.833800 165.797200 .807088
-70.916480 60.901050 164.908600 1.177783
-68.872850 60.937420 165.018500 1.112197
-66.826130 60.942730 166.152600 .616822
-64.780930 60.916960 166.942600 .263754
-62.741830 60.860230 167.275100 .112041
-60.713310 60.772790 167.407900 .049718
-58.699720 60.654990 167.460300 .023455
-56.705200 60.507380 167.485400 .011168
-54.733590 60.330550 167.498100 .004765
-52.788400 60.125220 167.503400 .001852
-50.872730 59.892220 167.505200 .000675
-48.989200 59.632420 167.505900 .000245
-47.139900 59.346780 167.506200 .000098
-45.326320 59.036270 167.506300 .000063
-43.549270 58.701890 167.506300 .000035
-41.808860 58.344600 167.506300 .000038
-40.104370 57.965370 167.506300 .000038
-38.434220 57.565060 167.506300 .000038
-36.795950 57.144450 167.506300 .000033
-35.186060 56.704150 167.506300 .000036
-33.600030 56.244660 167.506400 .000031
-32.032190 55.766200 167.506400 .000031
-102.859900 56.896880 167.507400 .000681
-101.242000 57.379640 167.507300 .000619
-99.604390 57.842470 167.507100 .000590
-97.940930 58.285070 167.507100 .000622
-96.246660 58.706940 167.507100 .000702
-94.517820 59.107400 167.506900 .000952
-92.751850 59.485580 167.506700 .001579
-90.947170 59.840560 167.507000 .003037
-89.103220 60.171320 167.510100 .006547
-87.220290 60.476800 167.521700 .015384
-85.299550 60.755960 167.555500 .037783
-83.342830 61.007760 167.611900 .068329
-81.352710 61.231220 167.664800 .088247
-79.332320 61.425420 167.570700 .060237
-77.285290 61.589540 167.157700 .193697
-75.215710 61.722870 166.778700 .378983
-73.128000 61.824790 166.307100 .599187
-71.026890 61.894870 165.393800 .984451
-68.917300 61.932760 165.077100 1.095177
-66.804310 61.938290 166.063000 .660482
-64.692990 61.911450 166.936400 .274377
-62.588420 61.852340 167.298400 .108740
-60.495590 61.761240 167.436600 .044500
-58.419300 61.638580 167.482700 .018600
-56.364090 61.484900 167.496800 .007605
-54.334160 61.300890 167.502200 .003109
-52.333320 61.087320 167.504700 .001210
-50.364880 60.845090 167.505700 .000452
-48.431600 60.575160 167.506100 .000159
-46.535590 60.278550 167.506300 .000080
-44.678250 59.956310 167.506300 .000065
-42.860180 59.609490 167.506300 .000042
-41.081100 59.239120 167.506300 .000038
-39.339830 58.846180 167.506300 .000033
-37.634110 58.431540 167.506300 .000033
-35.960610 57.995940 167.506300 .000035
-34.314830 57.539980 167.506300 .000036
-32.690990 57.064010 167.506400 .000031
-31.082000 56.568130 167.506400 .000031
-103.849600 57.699570 167.506700 .000245
-102.187300 58.200280 167.506600 .000216
-100.509000 58.680030 167.506600 .000203

-98.806840	59.138710	167.506500	.000206
-97.074520	59.575940	167.506300	.000291
-95.307120	59.991050	167.506100	.000521
-93.501110	60.383240	167.505600	.001072
-91.654170	60.751550	167.505500	.002166
-89.765210	61.094930	167.507200	.004723
-87.834170	61.412270	167.515300	.011641
-85.861950	61.702450	167.536300	.027677
-83.850420	61.964360	167.546900	.032678
-81.802150	62.196920	167.502100	.018405
-79.720490	62.399150	167.393200	.080394
-77.609360	62.570130	167.088100	.232371
-75.473210	62.709080	166.889200	.335370
-73.316960	62.815350	166.601800	.481899
-71.145790	62.888430	165.828100	.801144
-68.965200	62.927950	165.436400	.953032
-66.780790	62.933720	166.157600	.623115
-64.598260	62.905720	166.977000	.259232
-62.423240	62.844070	167.322500	.099676
-60.261290	62.749080	167.446000	.039887
-58.117710	62.621230	167.485800	.016224
-55.997520	62.461110	167.498700	.006512
-53.905320	62.269450	167.503600	.002556
-51.845240	62.047150	167.505400	.000938
-49.820820	61.795150	167.506100	.000316
-47.834930	61.514500	167.506200	.000102
-45.889670	61.206310	167.506300	.000068
-43.986290	60.871700	167.506300	.000037
-42.125070	60.511780	167.506300	.000031
-40.305270	60.127630	167.506300	.000031
-38.525020	59.720250	167.506300	.000032
-36.781200	59.290500	167.506300	.000034
-35.069400	58.839090	167.506300	.000034
-33.383770	58.366500	167.506300	.000035
-31.716990	57.872960	167.506400	.000031
-30.060130	57.358350	167.506400	.000031
-104.916500	58.490070	167.506500	.000087
-103.202300	59.010120	167.506400	.000070
-101.477600	59.507980	167.506400	.000067
-99.732440	59.983750	167.506300	.000093
-97.958720	60.437220	167.506100	.000193
-96.150010	60.867820	167.505800	.000411
-94.301540	61.274790	167.505300	.000809
-92.410060	61.657170	167.505000	.001521
-90.473700	62.013880	167.506100	.003348
-88.491920	62.343760	167.512500	.009111
-86.465330	62.645610	167.523200	.018841
-84.395630	62.918240	167.512300	.007089
-82.285480	63.160470	167.467800	.030641
-80.138370	63.371240	167.388000	.079363
-77.958560	63.549540	167.248100	.153664
-75.750930	63.694510	167.102800	.227316
-73.520860	63.805420	166.892900	.327566
-71.274150	63.881700	166.487500	.511077
-69.016910	63.922960	166.106600	.654096
-66.755400	63.928990	166.615500	.424826
-64.495980	63.899750	167.124900	.189185
-62.244980	63.835380	167.360300	.077159
-60.008560	63.736250	167.452000	.031690
-57.792640	63.602840	167.486700	.013140
-55.602750	63.435840	167.499600	.005350
-53.443960	63.236060	167.504200	.002065
-51.320750	63.004450	167.505700	.000747
-49.236890	62.742080	167.506200	.000236
-47.195330	62.450080	167.506300	.000096
-45.198120	62.129630	167.506300	.000046
-43.246250	61.781950	167.506300	.000046

-41.339580 61.408210 167.506300 .000046
-39.476710 61.009530 167.506300 .000046
-37.654880 60.586900 167.506400 .000039
-35.869840 60.141170 167.506300 .000046
-34.115770 59.672970 167.506400 .000039
-32.385130 59.182660 167.506400 .000038
-30.668580 58.670220 167.506400 .000031
-28.954870 58.135290 167.506400 .000031
-106.073800 59.266680 167.506400 .000049
-104.297500 59.807790 167.506400 .000031
-102.518800 60.325180 167.506400 .000037
-100.725000 60.819250 167.506300 .000095
-98.905530 61.289990 167.506100 .000181
-97.052100 61.737010 167.505800 .000350
-95.158340 62.159620 167.505400 .000604
-93.219700 62.556880 167.505100 .001013
-91.233350 62.927700 167.506000 .002143
-89.198040 63.270870 167.510200 .006333
-87.113920 63.585110 167.512300 .007353
-84.982480 63.869130 167.501700 .004619
-82.806390 64.121680 167.473200 .029208
-80.589290 64.341560 167.446600 .042621
-78.335770 64.527670 167.394100 .071106
-76.051160 64.679080 167.315500 .110460
-73.741460 64.794950 167.200000 .165334
-71.413090 64.874680 167.028500 .245867
-69.072890 64.917820 166.863100 .311822
-66.727910 64.924110 167.085700 .207041
-64.385280 64.893550 167.297900 .105072
-62.052090 64.826280 167.413800 .048938
-59.735260 64.722690 167.467900 .022102
-57.441380 64.583340 167.491500 .009648
-55.176610 64.408980 167.501100 .004001
-52.946510 64.200520 167.504700 .001555
-50.755950 63.959000 167.505900 .000555
-48.608900 63.685590 167.506300 .000188
-46.508400 63.381530 167.506300 .000065
-44.456330 63.048090 167.506400 .000039
-42.453320 62.686580 167.506400 .000037
-40.498620 62.298210 167.506400 .000037
-38.589950 61.884150 167.506400 .000036
-36.723370 61.445380 167.506400 .000036
-34.893150 60.982680 167.506400 .000036
-33.091610 60.496560 167.506400 .000036
-31.309040 59.987160 167.506400 .000036
-29.533510 59.454220 167.506400 .000031
-27.750760 58.896990 167.506400 .000031
-107.339200 60.027230 167.506400 .000031
-105.487000 60.591610 167.506400 .000031
-103.643800 61.130270 167.506400 .000039
-101.793500 61.644070 167.506300 .000082
-99.922550 62.133320 167.506100 .000154
-98.020040 62.597820 167.506000 .000283
-96.077510 63.037030 167.505600 .000465
-94.088710 63.450070 167.505300 .000675
-92.049460 63.835860 167.505600 .001066
-89.957580 64.193130 167.507400 .002403
-87.812570 64.520550 167.507500 .002171
-85.615560 64.816710 167.499400 .007651
-83.369120 65.080260 167.489300 .016199
-81.077050 65.309890 167.480200 .021463
-78.744270 65.504390 167.456400 .034005
-76.376630 65.662700 167.419200 .052376
-73.980770 65.783920 167.368000 .076712
-71.563890 65.867360 167.306200 .105260
-69.133670 65.912500 167.266300 .122286
-66.698060 65.919100 167.326600 .091520

-64.265090	65.887100	167.400900	.054836
-61.842790	65.816700	167.452600	.029422
-59.438900	65.708320	167.481600	.014669
-57.060850	65.562580	167.496000	.006829
-54.715470	65.380330	167.502600	.002943
-52.408880	65.162570	167.505200	.001167
-50.146370	64.910470	167.506100	.000423
-47.932120	64.625290	167.506300	.000138
-45.769150	64.308400	167.506300	.000062
-43.659080	63.961160	167.506400	.000035
-41.602020	63.584970	167.506400	.000031
-39.596350	63.181100	167.506400	.000031
-37.638600	62.750720	167.506400	.000036
-35.723270	62.294770	167.506400	.000031
-33.842640	61.813940	167.506400	.000031
-31.986660	61.308540	167.506400	.000031
-30.142740	60.778430	167.506400	.000034
-28.295590	60.222970	167.506400	.000031
-26.427080	59.640870	167.506400	.000031
-108.736700	60.768890	167.506400	.000031
-106.789100	61.359350	167.506400	.000031
-104.866900	61.921510	167.506400	.000042
-102.949500	62.456830	167.506300	.000087
-101.019200	62.966030	167.506200	.000137
-99.061880	63.449270	167.506000	.000251
-97.066180	63.906190	167.505700	.000401
-95.023590	64.336020	167.505400	.000515
-92.928180	64.737730	167.505500	.000578
-90.776390	65.110020	167.506200	.000651
-88.566850	65.451470	167.506200	.001026
-86.300130	65.760610	167.501300	.006140
-83.978550	66.035940	167.498300	.009374
-81.606060	66.276030	167.491600	.013918
-79.187900	66.479550	167.476900	.021658
-76.730480	66.645290	167.454300	.032688
-74.241170	66.772270	167.425700	.046281
-71.728080	66.859700	167.396900	.059463
-69.199870	66.907030	167.384000	.064619
-66.665550	66.913940	167.405700	.053104
-64.134220	66.880400	167.440000	.035885
-61.614990	66.806620	167.468800	.021334
-59.116610	66.693070	167.487600	.011499
-56.647430	66.540450	167.498000	.005672
-54.215080	66.349720	167.503100	.002547
-51.826330	66.121990	167.505300	.001048
-49.486860	65.858530	167.506100	.000389
-47.201050	65.560780	167.506300	.000128
-44.971820	65.230190	167.506300	.000054
-42.800360	64.868270	167.506400	.000034
-40.685980	64.476460	167.506400	.000031
-38.625890	64.056100	167.506400	.000031
-36.614990	63.608330	167.506400	.000031
-34.645670	63.134020	167.506400	.000031
-32.707610	62.633670	167.506400	.000031
-30.787590	62.107330	167.506400	.000031
-28.869240	61.554470	167.506400	.000031
-26.932890	60.973880	167.506400	.000031
-24.955330	60.363580	167.506400	.000031

Q.03a
-89.453400 32.991660 176.414600 .066648
-88.345930 33.191160 176.405200 .077706
-87.228100 33.381660 176.369900 .117988
-86.100230 33.562890 176.307400 .186304
-84.962690 33.734530 176.216600 .280246
-83.815900 33.896310 176.101000 .394049
-82.660300 34.047950 175.971300 .518068
-81.496410 34.189190 175.844200 .640427
-80.324730 34.319790 175.734500 .751103
-79.145840 34.439500 175.644600 .847735
-77.960310 34.548100 175.558500 .936828
-76.768760 34.645400 175.467700 1.016292
-75.571840 34.731200 175.410000 1.053312
-74.370180 34.805370 175.455400 .999004
-73.164480 34.867740 175.627000 .843842
-71.955430 34.918200 175.867600 .625007
-70.743730 34.956650 176.095900 .406817
-69.530080 34.983020 176.263500 .235312
-68.315220 34.997260 176.365400 .122150
-67.099880 34.999340 176.418700 .057152
-65.884770 34.989250 176.443100 .024068
-64.670640 34.967020 176.453300 .009007
-63.458190 34.932690 176.457000 .002842
-62.248150 34.886320 176.458400 .000592
-61.041230 34.828000 176.459000 .000472
-59.838120 34.757850 176.459200 .000743
-58.639500 34.676010 176.459300 .000840
-57.446040 34.582610 176.459400 .000842
-56.258390 34.477850 176.459300 .000742
-55.077160 34.361910 176.459100 .000562
-53.902950 34.235010 176.458900 .000372
-52.736340 34.097380 176.458700 .000214
-51.577860 33.949260 176.458600 .000087
-50.428010 33.790910 176.458600 .000045
-49.287250 33.622600 176.458600 .000046
-48.156040 33.444620 176.458600 .000046
-47.034740 33.257250 176.458600 .000046
-45.923690 33.060790 176.458600 .000046
-44.823220 32.855560 176.458600 .000046
-89.701210 33.918400 176.365700 .119150
-88.583520 34.125020 176.340800 .143346
-87.455040 34.322360 176.259900 .218872
-86.316120 34.510110 176.119700 .343828
-85.167100 34.687960 175.917900 .513212
-84.008420 34.855620 175.662100 .716058
-82.840550 35.012790 175.375900 .935348
-81.663990 35.159200 175.095300 1.149967
-80.479300 35.294580 174.857800 1.341621
-79.287040 35.418700 174.672300 1.508350
-78.087840 35.531310 174.491600 1.676274
-76.882320 35.632210 174.270700 1.858570
-75.671150 35.721200 174.096100 1.978763
-74.455030 35.798110 174.177400 1.905078

-73.234660	35.862810	174.583700	1.613724
-72.010770	35.915150	175.152400	1.185525
-70.784100	35.955040	175.676800	.755466
-69.555380	35.982390	176.049700	.425201
-68.325390	35.997170	176.268700	.214872
-67.094890	35.999320	176.379400	.098164
-65.864640	35.988860	176.428900	.040464
-64.635410	35.965800	176.448700	.014803
-63.407950	35.930180	176.456000	.004493
-62.183030	35.882080	176.458600	.000928
-60.961370	35.821590	176.459500	.001002
-59.743720	35.748840	176.460000	.001415
-58.530780	35.663950	176.460300	.001566
-57.323260	35.567100	176.460300	.001527
-56.121820	35.458470	176.460100	.001331
-54.927120	35.338260	176.459700	.000995
-53.739770	35.206700	176.459300	.000620
-52.560380	35.064020	176.458900	.000314
-51.389480	34.910500	176.458700	.000126
-50.227610	34.746390	176.458600	.000045
-49.075240	34.571990	176.458600	.000046
-47.932830	34.387580	176.458600	.000046
-46.800740	34.193490	176.458600	.000046
-45.679330	33.990000	176.458600	.000046
-44.568900	33.777440	176.458600	.000046
-89.960660	34.843320	176.203200	.271621
-88.832330	35.057210	176.124300	.334057
-87.692770	35.261540	175.892900	.508284
-86.542320	35.455950	175.500000	.789226
-85.381340	35.640140	174.942100	1.162477
-84.210270	35.813810	174.241900	1.602878
-83.029580	35.976620	173.458500	2.075669
-81.839790	36.128310	172.690600	2.536850
-80.641480	36.268600	172.046500	2.942356
-79.435230	36.397220	171.580200	3.281250
-78.221690	36.513940	171.112100	3.657159
-77.001530	36.618520	170.405100	4.173845
-75.775430	36.710780	169.718500	4.611550
-74.544130	36.790520	169.872600	4.514707
-73.308360	36.857590	171.083600	3.852047
-72.068890	36.911860	172.793600	2.826595
-70.826490	36.953220	174.316100	1.779573
-69.581950	36.981590	175.363400	.979181
-68.336070	36.996900	175.960800	.483192
-67.089650	36.999130	176.255200	.216679
-65.843500	36.988290	176.383900	.088138
-64.598400	36.964370	176.434500	.031881
-63.355190	36.927440	176.452800	.009515
-62.114630	36.877570	176.459200	.002115
-60.877510	36.814860	176.461600	.002593
-59.644590	36.739430	176.462700	.003422
-58.416640	36.651430	176.463200	.003714
-57.194370	36.551040	176.463300	.003584
-55.978490	36.438440	176.462700	.003075
-54.769680	36.313860	176.461500	.002237
-53.568590	36.177530	176.460300	.001286
-52.375820	36.029710	176.459300	.000580
-51.191970	35.870660	176.458800	.000194
-50.017550	35.700660	176.458600	.000046
-48.853080	35.520030	176.458600	.000052
-47.698980	35.329070	176.458600	.000050
-46.555660	35.128100	176.458600	.000049
-45.423470	34.917440	176.458600	.000048
-44.302690	34.697410	176.458600	.000046
-90.232250	35.766320	175.806100	.582764
-89.092860	35.987640	175.580600	.729398
-87.941790	36.199090	174.966500	1.108304

-86.779330	36.400320	173.941300	1.703081
-85.605870	36.591000	172.506800	2.474270
-84.421870	36.770790	170.716900	3.371934
-83.227800	36.939400	168.715300	4.340110
-82.024180	37.096500	166.727400	5.302451
-80.811620	37.241800	165.113800	6.107101
-79.590740	37.375040	164.079500	6.711396
-78.362190	37.495960	163.013900	7.426077
-77.126670	37.604320	160.804100	8.773897
-75.884930	37.699920	158.054100	10.307710
-74.637700	37.782550	158.215900	10.145790
-73.385770	37.852060	161.725400	8.674896
-72.129940	37.908310	166.687700	6.398892
-70.871030	37.951180	170.927200	3.993762
-69.609860	37.980580	173.715500	2.153104
-68.347290	37.996460	175.244900	1.034415
-67.084140	37.998780	175.974900	.452793
-65.821270	37.987530	176.285600	.181104
-64.559530	37.962740	176.405000	.064566
-63.299760	37.924460	176.447300	.018819
-62.042780	37.872770	176.461800	.004829
-60.789440	37.807780	176.467200	.006535
-59.540510	37.729610	176.469700	.008183
-58.296810	37.638420	176.470900	.008738
-57.059080	37.534390	176.470900	.008445
-55.828080	37.417740	176.469300	.007216
-54.604500	37.288680	176.466200	.005069
-53.389020	37.147460	176.462400	.002623
-52.182290	36.994370	176.459900	.000975
-50.984880	36.829660	176.458800	.000199
-49.797380	36.653650	176.458500	.000096
-48.620270	36.466650	176.458400	.000128
-47.454020	36.268990	176.458500	.000095
-46.299010	36.060990	176.458500	.000077
-45.155610	35.843010	176.458600	.000051
-44.024070	35.615370	176.458600	.000048
-90.516530	36.687310	174.973100	1.126646
-89.365660	36.916220	174.401400	1.433997
-88.202590	37.134950	172.928100	2.179091
-87.027650	37.343150	170.508400	3.306244
-85.841200	37.540460	167.172700	4.711345
-84.643710	37.726540	163.045100	6.311951
-83.435650	37.901050	158.355500	8.138119
-82.217600	38.063690	153.659300	9.982190
-80.990140	38.214140	149.782500	11.557900
-79.753940	38.352110	148.137200	12.259790
-78.509670	38.477340	146.363300	13.199280
-77.258070	38.589580	140.301800	16.227700
-75.999910	38.688600	130.193100	20.665980
-74.735980	38.774210	129.739400	20.570030
-73.467090	38.846230	138.903500	17.215210
-72.194090	38.904510	152.598800	12.976220
-70.917830	38.948930	163.510600	8.091356
-69.639210	38.979390	170.296800	4.261686
-68.359090	38.995840	173.832200	1.986337
-67.078360	38.998240	175.446500	.843871
-65.797930	38.986590	176.109600	.330194
-64.518690	38.960900	176.356500	.116000
-63.241520	38.921240	176.442200	.032769
-61.967320	38.867690	176.471000	.010827
-60.696920	38.800350	176.481600	.015654
-59.431210	38.719360	176.486700	.018908
-58.170970	38.624900	176.489300	.020152
-56.917050	38.517150	176.489200	.019660
-55.670200	38.396340	176.485300	.016993
-54.431160	38.262690	176.477100	.011902
-53.200640	38.116470	176.466100	.004853

-51.979310	37.957960	176.460300	.001179
-50.767770	37.787460	176.458300	.000162
-49.566610	37.605300	176.458000	.000429
-48.376340	37.411790	176.458100	.000353
-47.197410	37.207270	176.458300	.000224
-46.030250	36.992090	176.458400	.000132
-44.875180	36.766620	176.458500	.000076
-43.732470	36.531200	176.458600	.000057
-90.814130	37.606180	173.466900	1.954746
-89.651300	37.842850	172.175300	2.526862
-88.475770	38.069030	168.966000	3.835536
-87.287830	38.284350	163.766500	5.706587
-86.087840	38.488450	156.802200	7.817147
-84.876290	38.680970	148.254100	10.137450
-83.653650	38.861550	138.387000	12.906290
-82.420500	39.029860	127.520800	16.592750
-81.177470	39.185590	118.871100	18.695240
-79.925230	39.328410	117.064400	18.799560
-78.664510	39.458070	118.005900	18.295580
-77.396060	39.574290	106.754600	21.891340
-76.120680	39.676830	85.206030	27.885650
-74.839220	39.765490	77.648250	26.866610
-73.552520	39.840080	93.071390	25.881720
-72.261480	39.900440	123.434300	22.437030
-70.967010	39.946460	149.407000	14.299420
-69.670030	39.978020	164.279700	7.378909
-68.371470	39.995060	171.508100	3.313106
-67.072270	39.997550	174.633200	1.358991
-65.773400	39.985470	175.859200	.525784
-64.475770	39.958870	176.297200	.182982
-63.180340	39.917780	176.446900	.050523
-61.888020	39.862300	176.497600	.025051
-60.599740	39.792560	176.516600	.036320
-59.316390	39.708680	176.526700	.043245
-58.038840	39.610860	176.532700	.046850
-56.767930	39.499290	176.533300	.047255
-55.504470	39.374200	176.525200	.041980
-54.249260	39.235840	176.504100	.027260
-53.003000	39.084480	176.470200	.007384
-51.766410	38.920440	176.458200	.000293
-50.540120	38.744010	176.456100	.001585
-49.324710	38.555530	176.456600	.001318
-48.120720	38.355340	176.457500	.000835
-46.928620	38.143810	176.458000	.000454
-45.748810	37.921300	176.458300	.000223
-44.581620	37.688170	176.458500	.000103
-43.427300	37.444810	176.458500	.000092
-91.125630	38.522790	171.158000	3.029544
-89.950410	38.767390	168.550800	3.973618
-88.761920	39.001200	162.102200	5.998025
-87.560450	39.223820	151.737200	8.550597
-86.346370	39.434880	138.259900	10.965580
-85.120160	39.633990	123.007000	12.835930
-83.882300	39.820790	106.180900	15.602240
-82.633390	39.994930	87.310870	18.779890
-81.374080	40.156070	73.179570	18.628470
-80.105060	40.303890	75.060330	19.965870
-78.827100	40.438090	83.417930	14.205310
-77.540980	40.558400	76.188090	12.785320
-76.247540	40.664560	45.712000	15.772670
-74.947690	40.756360	34.247650	16.403710
-73.642300	40.833600	41.914320	21.338780
-72.332310	40.896110	81.929490	27.641500
-71.018690	40.943760	129.061200	20.468510
-69.702430	40.976440	156.327900	10.713740
-68.384490	40.994090	168.748500	4.687871
-67.065880	40.996670	173.767900	1.879121

-65.747600	40.984170	175.598800	.730020
-64.430650	40.956610	176.251500	.262972
-63.116020	40.914060	176.483700	.072250
-61.804690	40.856610	176.565100	.059133
-60.497620	40.784390	176.598200	.082829
-59.195770	40.697540	176.619300	.097380
-57.900040	40.596260	176.635300	.107391
-56.611330	40.480750	176.641000	.110421
-55.330490	40.351270	176.613500	.092837
-54.058330	40.208070	176.529400	.042517
-52.795620	40.051450	176.458400	.001942
-51.543080	39.881710	176.446200	.007969
-50.301390	39.699200	176.449600	.005547
-49.071130	39.504250	176.453700	.003228
-47.852850	39.297230	176.456200	.001675
-46.647030	39.078520	176.457600	.000829
-45.454070	38.848490	176.458100	.000381
-44.274280	38.607530	176.458400	.000166
-43.107920	38.356030	176.458500	.000112
-91.451740	39.437030	168.291200	4.164488
-90.263630	39.689750	163.628200	5.508794
-89.061680	39.931370	151.947000	8.085208
-87.846150	40.161480	132.394600	10.630820
-86.617400	40.379670	106.352800	11.524590
-85.375900	40.585540	80.447600	10.710800
-84.122160	40.778730	63.019060	10.633680
-82.856800	40.958850	57.283530	6.498416
-81.580460	41.125550	33.429860	10.854730
-80.293880	41.278500	37.619480	9.275429
-78.997860	41.417370	62.368620	7.191136
-77.693230	41.541890	71.916950	8.481771
-76.380870	41.651780	37.130030	6.174013
-75.061690	41.746810	19.183290	5.122261
-73.736660	41.826770	34.446670	9.161250
-72.406780	41.891490	65.423440	24.284200
-71.073040	41.940830	115.458100	22.501090
-69.736500	41.974670	150.430300	12.682060
-68.398190	41.992950	166.659900	5.855700
-67.059160	41.995620	173.202000	2.397804
-65.720480	41.982670	175.525700	.944307
-64.383220	41.954140	176.230700	.367618
-63.048400	41.910080	176.588900	.109589
-61.717090	41.850600	176.713200	.130036
-60.390290	41.775820	176.754400	.165594
-59.069010	41.685920	176.767900	.179821
-57.754210	41.581080	176.771300	.183315
-56.446830	41.461530	176.746900	.169378
-55.147770	41.327530	176.653400	.114496
-53.857880	41.179360	176.494900	.021108
-52.577970	41.017320	176.401800	.034135
-51.308780	40.841740	176.407400	.030321
-50.051000	40.652980	176.436300	.013972
-48.805250	40.451390	176.448900	.006224
-47.572090	40.237360	176.454500	.002863
-46.352010	40.011290	176.456900	.001273
-45.145370	39.773560	176.457900	.000558
-43.952510	39.524580	176.458300	.000236
-42.773630	39.264750	176.458500	.000130
-91.793200	40.348750	165.769300	5.036963
-90.591710	40.609810	158.997500	6.539060
-89.375760	40.859430	141.453300	8.980301
-88.145600	41.097210	109.808600	10.925440
-86.901570	41.322720	66.306390	9.267338
-85.644150	41.535550	31.930490	5.590989
-84.373860	41.735290	33.662890	7.913845
-83.091290	41.921560	25.028340	3.366094
-81.797160	42.093980	26.793740	9.586675

-80.492230	42.252200	35.482430	5.725109
-79.177290	42.395880	58.826610	8.213007
-77.853240	42.524730	58.841730	9.055516
-76.521000	42.638450	34.983320	9.311561
-75.181550	42.736820	21.297990	9.011145
-73.835900	42.819590	47.450110	7.059519
-72.485100	42.886590	81.259470	16.850620
-71.130210	42.937670	119.160200	19.918330
-69.772340	42.972710	149.473300	12.983340
-68.412590	42.991630	165.067700	6.844434
-67.052090	42.994400	172.451400	3.040789
-65.691960	42.980990	175.517200	1.157284
-64.333320	42.951450	176.363300	.421138
-62.977290	42.905830	176.741200	.164627
-61.624970	42.844260	176.895300	.212468
-60.277440	42.766850	176.881700	.231754
-58.935750	42.673790	176.835100	.216798
-57.600940	42.565280	176.770100	.182896
-56.273990	42.441570	176.687500	.132348
-54.955840	42.302930	176.536000	.044054
-53.647390	42.149640	176.350600	.068874
-52.349470	41.982030	176.305300	.093117
-51.062890	41.800450	176.373400	.052326
-49.788320	41.605260	176.424300	.021530
-48.526440	41.396860	176.444400	.009103
-47.277780	41.175640	176.452900	.003984
-46.042850	40.942000	176.456300	.001710
-44.822010	40.696380	176.457800	.000721
-43.615560	40.439180	176.458300	.000294
-42.423690	40.170830	176.458400	.000158
-92.150770	41.257820	164.791400	5.414898
-90.935390	41.527410	157.583300	6.745430
-89.704910	41.785250	138.710000	8.803231
-88.459540	42.030900	103.365100	11.274220
-87.199620	42.263930	57.110120	10.622870
-85.925610	42.483890	24.560710	9.039725
-84.638040	42.690380	17.630370	8.081386
-83.337520	42.882970	30.379990	10.048020
-82.024790	43.061280	34.995110	5.663329
-80.700610	43.224930	68.719220	11.899890
-79.365870	43.373580	75.080440	14.854420
-78.021450	43.506890	64.831120	12.712170
-76.668370	43.624570	19.756200	3.014969
-75.307630	43.726360	31.364150	7.052958
-73.940300	43.812040	52.139070	5.112930
-72.567500	43.881390	87.066090	15.805030
-71.190370	43.934270	121.182200	19.545880
-69.810050	43.970550	146.599900	14.598480
-68.427750	43.990140	163.631700	7.930277
-67.044650	43.993000	172.367300	3.309561
-65.661930	43.979120	175.921600	1.146020
-64.280810	43.948540	176.877900	.360057
-62.902460	43.901310	177.024800	.247182
-61.528050	43.837570	177.039000	.287523
-60.158730	43.757450	176.931600	.272190
-58.795620	43.661140	176.843600	.227816
-57.439790	43.548850	176.759100	.171396
-56.092310	43.420850	176.621600	.093314
-54.754150	43.277410	176.434600	.042884
-53.426270	43.118850	176.252100	.133822
-52.109530	42.945500	176.266400	.120173
-50.804760	42.757750	176.363000	.060154
-49.512690	42.555960	176.419200	.024959
-48.233980	42.340550	176.442300	.010661
-46.969190	42.111940	176.452000	.004644
-45.718810	41.870550	176.456100	.001976
-44.483210	41.616830	176.457700	.000815

-43.262650	41.351200	176.458300	.000328
-42.057300	41.074100	176.458400	.000179
-92.525320	42.164070	165.321700	5.502187
-91.295520	42.442420	158.484500	7.399858
-90.049940	42.708690	140.752300	11.001470
-88.788760	42.962440	109.206500	14.799680
-87.512310	43.203190	66.144680	17.077290
-86.221000	43.430500	34.453700	8.670505
-84.915410	43.643920	23.306930	7.807668
-83.596150	43.843030	18.745750	8.414145
-82.263960	44.027400	47.785960	12.781140
-80.919660	44.196660	78.887760	12.391740
-79.564150	44.350410	81.977180	14.302770
-78.198390	44.488330	60.154290	9.027493
-76.823410	44.610100	32.384730	10.505870
-75.440290	44.715440	14.279630	4.062289
-74.050180	44.804110	37.815780	5.213586
-72.654240	44.875890	66.563420	19.759400
-71.253690	44.930630	108.904700	23.222640
-69.849760	44.968180	142.491600	16.677920
-68.443720	44.988460	161.855100	8.818764
-67.036810	44.991420	171.691100	3.787910
-65.630330	44.977050	176.101300	1.308375
-64.225530	44.945390	177.234200	.499369
-62.823690	44.896510	177.401200	.415330
-61.426040	44.830540	177.260200	.400576
-60.033810	44.747610	177.124700	.355117
-58.648170	44.647940	176.982100	.281801
-57.270290	44.531750	176.825400	.192589
-55.901260	44.399320	176.646900	.098293
-54.542130	44.250930	176.422100	.071837
-53.193900	44.086940	176.222200	.161051
-51.857480	43.907680	176.267900	.124597
-50.533710	43.713560	176.369100	.058633
-49.223370	43.504980	176.421200	.024717
-47.927120	43.282370	176.442900	.010746
-46.645540	43.046150	176.452200	.004700
-45.379100	42.796800	176.456200	.001992
-44.128160	42.534760	176.457700	.000814
-42.892960	42.260480	176.458300	.000325
-41.673610	41.974410	176.458500	.000182
-92.917770	43.067330	166.983800	5.115067
-91.673000	43.354680	161.123500	7.140262
-90.411740	43.629630	145.723200	11.376730
-89.134110	43.891690	117.190100	17.519290
-87.840450	44.140390	77.837380	21.707000
-86.531140	44.375260	40.368530	21.557780
-85.206750	44.595830	25.982010	11.218290
-83.867900	44.801650	29.635200	7.409266
-82.515380	44.992280	35.310180	9.044386
-81.150000	45.167310	54.472610	11.230960
-79.772730	45.326340	55.337110	7.717178
-78.384560	45.469020	56.065600	7.153162
-76.986600	45.595010	37.228070	5.549021
-75.579970	45.704020	24.389390	7.449699
-74.165890	45.795780	34.071680	9.837017
-72.745610	45.870080	70.250960	18.321660
-71.320400	45.926740	111.748100	20.386660
-69.891600	45.965610	141.152500	15.723380
-68.460530	45.986610	158.991300	9.690773
-67.028560	45.989670	168.923200	4.915623
-65.597030	45.974800	174.279600	2.038171
-64.167300	45.942020	176.185100	.768503
-62.740720	45.891430	176.862700	.361056
-61.318600	45.823140	177.122300	.347692
-59.902260	45.737310	177.036000	.295037
-58.492950	45.634170	176.924900	.236687

-57.091900 45.513950 176.764900 .169800
-55.700260 45.376930 176.603800 .104464
-54.319140 45.223450 176.402700 .108591
-52.949590 45.053840 176.235300 .169845
-51.592580 44.868490 176.293900 .117692
-50.248970 44.667810 176.389300 .051162
-48.919560 44.452230 176.428900 .021646
-47.605040 44.222190 176.445800 .009599
-46.305980 43.978160 176.453300 .004224
-45.022840 43.720610 176.456500 .001810
-43.755960 43.450020 176.457800 .000734
-42.505550 43.166850 176.458400 .000301
-41.271660 42.871580 176.458500 .000159
-93.329110 43.967430 169.162300 4.305816
-92.068780 44.264030 164.727600 6.054888
-90.791210 44.547890 153.126000 9.842917
-89.496510 44.818510 130.797900 14.946240
-88.184940 45.075390 94.252300 21.919100
-86.856880 45.318050 50.531600 27.036760
-85.512890 45.545990 29.985500 20.789800
-84.153590 45.758730 34.043510 12.464430
-82.779790 45.955820 36.512530 8.574537
-81.392350 46.136820 28.997040 7.927855
-79.992260 46.301310 28.406370 7.104320
-78.580580 46.448910 43.521030 7.453395
-77.158460 46.579270 42.039080 7.210612
-75.727110 46.692070 47.656020 13.595270
-74.287820 46.787040 66.098830 18.376590
-72.841890 46.863950 101.170100 17.357790
-71.390720 46.922590 127.631500 14.261440
-69.935700 46.962830 143.455300 13.169050
-68.478260 46.984570 156.325600 10.393330
-67.019850 46.987740 165.662000 6.386722
-65.561920 46.972340 171.170600 3.453322
-64.105920 46.938410 173.941300 1.805042
-62.653260 46.886040 175.325000 .954087
-61.205390 46.815350 175.956500 .552581
-59.763680 46.726520 176.250400 .351583
-58.329470 46.619790 176.420800 .264524
-56.904060 46.495390 176.598900 .227230
-55.488670 46.353640 176.656500 .198073
-54.084500 46.194880 176.556000 .147811
-52.692630 46.019470 176.345900 .147532
-51.314070 45.827840 176.358600 .096236
-49.949730 45.620390 176.417800 .040871
-48.600430 45.397590 176.439800 .017487
-47.266860 45.159900 176.449800 .007798
-45.949600 44.907820 176.454700 .003457
-44.649100 44.641840 176.457000 .001481
-43.365670 44.362450 176.458000 .000608
-42.099450 44.070140 176.458400 .000248
-40.850440 43.765410 176.458500 .000145
-93.760410 44.864160 171.279700 3.325802
-92.483920 45.170280 168.211200 4.697949
-91.189420 45.463320 160.201200 7.857695
-89.876960 45.742750 143.773000 13.220010
-88.546760 46.008070 113.314300 22.884000
-87.199170 46.258760 75.613460 31.720870
-85.834720 46.494300 48.960490 32.574940
-84.454070 46.714200 51.291680 22.550220
-83.058010 46.917960 51.488820 18.370660
-81.647480 47.105130 38.972800 16.488790
-80.223460 47.275260 35.421410 20.005740
-78.787090 47.427960 45.758200 19.359070
-77.339580 47.562840 58.376480 24.255100
-75.882230 47.679570 76.890960 27.444100
-74.416370 47.777870 108.690200 21.658740

-72.943440	47.857470	133.723900	13.455960
-71.464880	47.918190	145.654600	9.875831
-69.982220	47.959850	152.836200	9.814789
-68.496960	47.982350	158.912400	8.644588
-67.010670	47.985630	164.918400	6.434678
-65.524890	47.969690	169.161000	4.322106
-64.041170	47.934570	171.354900	3.022946
-62.561040	47.880340	172.763300	2.213009
-61.086020	47.807170	173.630300	1.676960
-59.617590	47.715230	174.439900	1.213691
-58.157170	47.604770	175.398900	.705553
-56.706150	47.476050	176.270200	.306339
-55.265830	47.329400	176.716800	.230708
-53.837470	47.165180	176.722800	.186131
-52.422200	46.983780	176.494400	.118503
-51.021090	46.785640	176.433100	.070560
-49.635090	46.571200	176.445400	.030910
-48.265020	46.340950	176.450000	.013371
-46.911610	46.095370	176.453300	.005910
-45.575410	45.834990	176.455900	.002605
-44.256860	45.560320	176.457400	.001131
-42.956210	45.271870	176.458100	.000469
-41.673580	44.970160	176.458400	.000202
-40.408870	44.655690	176.458500	.000125
-94.212860	45.757310	173.007400	2.391428
-92.919560	46.073230	171.070100	3.329256
-91.607470	46.375730	166.220400	5.466570
-90.276550	46.664250	156.292000	9.600367
-88.926960	46.938270	137.427800	17.717380
-87.559010	47.197250	107.597700	31.500640
-86.173240	47.440650	83.342190	38.139620
-84.770290	47.667940	72.043300	34.681330
-83.350960	47.878600	67.831860	29.848950
-81.916200	48.072160	70.848050	24.708430
-80.467090	48.248140	69.492600	25.727630
-79.004780	48.406110	82.025950	30.721700
-77.530590	48.545680	97.707570	28.723510
-76.045850	48.666500	120.504300	22.665580
-74.552020	48.768250	140.495300	15.777170
-73.050600	48.850660	154.140900	9.832638
-71.543170	48.913520	160.616800	6.619557
-70.031330	48.956650	163.143600	5.948564
-68.516710	48.979950	166.130000	5.389811
-67.000980	48.983350	168.702100	4.341934
-65.485790	48.966840	170.415200	3.584269
-63.972820	48.930470	171.034200	3.215576
-62.463700	48.874330	171.379200	2.909356
-60.960050	48.798580	171.810900	2.619927
-59.463460	48.703410	172.602500	2.156425
-57.975440	48.589080	174.091500	1.349183
-56.497490	48.455880	175.719900	.517360
-55.030970	48.304140	176.538200	.173955
-53.577210	48.134270	176.662900	.138215
-52.137430	47.946660	176.493500	.074972
-50.712720	47.741790	176.418400	.052289
-49.304080	47.520120	176.436000	.024068
-47.912350	47.282170	176.448300	.010130
-46.538250	47.028450	176.454100	.004366
-45.182340	46.759510	176.456500	.001895
-43.845020	46.475880	176.457600	.000816
-42.526500	46.178100	176.458200	.000349
-41.226800	45.866700	176.458400	.000159
-39.945740	45.542200	176.458500	.000102
-94.687740	46.646660	174.251300	1.636749
-93.376940	46.972690	173.123100	2.204831
-92.046550	47.284930	170.533400	3.433376
-90.696440	47.582840	164.726500	6.157889

-89.326680	47.865840	153.799300	11.630200
-87.937550	48.133390	137.259400	21.050660
-86.529520	48.384910	115.393700	31.427120
-85.103260	48.619850	102.029000	34.442510
-83.659580	48.837660	101.102400	29.186120
-82.199460	49.037830	107.218100	24.575980
-80.724000	49.219880	118.345000	23.568600
-79.234450	49.383340	126.122800	22.418510
-77.732160	49.527770	136.877400	19.183780
-76.218590	49.652830	148.071400	14.765940
-74.695260	49.758160	157.225100	10.441540
-73.163800	49.843490	163.636200	6.979263
-71.625870	49.908580	167.319300	4.869885
-70.083210	49.953250	169.501100	3.831811
-68.537570	49.977380	171.347600	2.971460
-66.990740	49.980900	173.071600	2.270516
-65.444490	49.963800	173.549500	2.063729
-63.900620	49.926140	173.201000	2.140170
-62.360880	49.868010	172.991000	2.177233
-60.827010	49.789570	172.983800	2.091300
-59.300730	49.691040	173.359700	1.827177
-57.783640	49.572690	174.382100	1.231791
-56.277320	49.434830	175.656400	.524198
-54.783270	49.277820	176.461500	.148441
-53.302860	49.102080	176.623500	.106459
-51.837360	48.908040	176.506700	.047206
-50.387950	48.696200	176.428500	.034183
-48.955640	48.467040	176.434900	.020110
-47.541310	48.221130	176.449600	.007520
-46.145660	47.959000	176.454400	.003015
-44.769230	47.681210	176.456800	.001300
-43.412380	47.388330	176.457800	.000571
-42.075270	47.080920	176.458200	.000258
-40.757810	46.759540	176.458400	.000129
-39.459720	46.424710	176.458500	.000079
-95.186440	47.531940	175.095100	1.074383
-93.857410	47.868410	174.533900	1.350637
-92.508000	48.190720	173.330300	1.929711
-91.137910	48.498310	170.377000	3.422978
-89.747150	48.790600	164.529500	6.525530
-88.335950	49.067010	155.668700	11.474760
-86.904710	49.326930	145.287300	17.490720
-85.454100	49.569790	135.980500	20.772350
-83.984930	49.795020	136.720200	18.594330
-82.498220	50.002060	143.897400	15.434960
-80.995120	50.190400	149.853000	13.132150
-79.476920	50.359550	153.409700	12.732440
-77.945060	50.509060	157.918100	10.966470
-76.401090	50.638520	162.409100	8.625541
-74.846650	50.747580	165.933400	6.519286
-73.283450	50.835950	168.615000	4.787784
-71.713320	50.903350	170.380700	3.662061
-70.138080	50.949630	171.666900	2.978817
-68.559640	50.974620	173.522600	2.145013
-66.979900	50.978270	175.168300	1.325369
-65.400800	50.960560	175.439200	1.059798
-63.824260	50.921550	175.056000	1.089149
-62.252170	50.861340	174.938300	1.040702
-60.686400	50.780110	174.965000	1.003587
-59.128770	50.678080	175.019700	.916056
-57.581030	50.555550	175.392300	.655835
-56.044860	50.412850	176.122700	.274744
-54.521820	50.250360	176.496400	.091755
-53.013420	50.068520	176.600600	.089717
-51.520960	49.867810	176.546300	.052249
-50.045680	49.648740	176.487800	.017938
-48.588630	49.411830	176.455800	.008607

-47.150700	49.157670	176.455500	.003649
-45.732590	48.886830	176.456700	.001578
-44.334810	48.599900	176.457600	.000729
-42.957660	48.297460	176.458100	.000345
-41.601190	47.980120	176.458300	.000169
-40.265250	47.648430	176.458500	.000091
-38.949390	47.302930	176.458500	.000074
-95.710510	48.412890	175.697400	.650606
-94.362470	48.760130	175.515300	.723405
-92.993230	49.092850	175.100600	.916491
-91.602360	49.410460	173.798800	1.626812
-90.189750	49.712350	171.128600	3.054699
-88.755540	49.997940	167.385600	5.170013
-87.300090	50.266570	162.971700	7.569705
-85.824040	50.517650	159.807500	9.201035
-84.328210	50.750570	160.367900	8.700567
-82.813610	50.964740	162.438800	7.582258
-81.281460	51.159630	164.812000	6.825871
-79.733130	51.334700	166.543900	6.274692
-78.170130	51.489480	168.393800	5.410697
-76.594090	51.623530	169.687500	4.529712
-75.006800	51.736490	170.706600	3.719785
-73.410070	51.828010	171.727100	2.986835
-71.805870	51.897850	172.269000	2.630848
-70.196170	51.945790	172.808900	2.451186
-68.582990	51.971680	174.045700	1.943615
-66.968440	51.975460	175.800500	1.103290
-65.354560	51.957120	176.239700	.632304
-63.743440	51.916690	175.962600	.506754
-62.137130	51.854320	175.850700	.451595
-60.537630	51.770170	175.908800	.381403
-58.946900	51.664510	175.971100	.337805
-57.366820	51.537620	176.086700	.251650
-55.799180	51.389870	176.302400	.118185
-54.245650	51.221680	176.459100	.046418
-52.707820	51.033510	176.528300	.045490
-51.187080	50.825860	176.531400	.040495
-49.684720	50.599280	176.482900	.015938
-48.201800	50.354330	176.466900	.005298
-46.739240	50.091630	176.460200	.002148
-45.297720	49.811770	176.458400	.000836
-43.877700	49.515370	176.458200	.000368
-42.479410	49.203060	176.458300	.000193
-41.102810	48.875440	176.458500	.000127
-39.747590	48.533100	176.458500	.000064
-38.413150	48.176570	176.458600	.000058
-96.261670	49.289190	176.202700	.305760
-94.893750	49.647600	176.220300	.305838
-93.503820	49.991080	176.174300	.316577
-92.091320	50.319060	175.573300	.651849
-90.655950	50.630910	174.452500	1.280437
-89.197760	50.925990	172.903700	2.111665
-87.717060	51.203660	171.305300	3.004583
-86.214420	51.463270	170.231700	3.654100
-84.690660	51.704190	170.117900	3.758341
-83.146820	51.925780	170.660700	3.597593
-81.584160	52.127480	171.523600	3.237042
-80.004110	52.308710	172.448400	2.873931
-78.408280	52.468990	173.052700	2.453227
-76.798410	52.607840	173.274300	2.162874
-75.176390	52.724850	173.362800	1.967617
-73.544200	52.819690	173.510900	1.784628
-71.903920	52.892050	173.645200	1.748474
-70.257710	52.941730	173.590500	1.928158
-68.607750	52.968570	174.426700	1.710289
-66.956280	52.972490	175.960900	1.044319
-65.305560	52.953480	176.569000	.482699

-63.657820	52.911580	176.433200	.267780
-62.015270	52.846950	176.322500	.186956
-60.380080	52.759750	176.296600	.149081
-58.754360	52.650280	176.270200	.131743
-57.140120	52.518850	176.280200	.110628
-55.539290	52.365840	176.369500	.063518
-53.953660	52.191710	176.444500	.026184
-52.384890	51.996940	176.478800	.017200
-50.834480	51.782070	176.484900	.016197
-49.303730	51.547700	176.474400	.008833
-47.793770	51.294400	176.465100	.004274
-46.305490	51.022820	176.460600	.001655
-44.839570	50.733620	176.459000	.000621
-43.396390	50.427420	176.458500	.000223
-41.976090	50.104880	176.458500	.000100
-40.578500	49.766630	176.458500	.000074
-39.203150	49.413260	176.458500	.000064
-37.849210	49.045330	176.458600	.000050
-96.841810	50.160530	176.514600	.134390
-95.453030	50.530490	176.593300	.144209
-94.041510	50.885130	176.607900	.139689
-92.606450	51.223850	176.337900	.213329
-91.147380	51.546020	175.823400	.479987
-89.664200	51.850960	175.181900	.841125
-88.157130	52.138020	174.568200	1.193462
-86.626690	52.406500	174.156000	1.453607
-85.073680	52.655720	174.024700	1.593850
-83.499180	52.885050	174.141200	1.594165
-81.904440	53.093850	174.534500	1.439057
-80.290990	53.281520	174.962200	1.181050
-78.660530	53.447530	175.193400	.972117
-77.014920	53.591380	175.053400	.925924
-75.356160	53.712650	174.727200	1.016698
-73.686420	53.810940	174.492200	1.123520
-72.007930	53.885960	174.368400	1.230308
-70.322990	53.937460	174.242500	1.431854
-68.634010	53.965280	174.669400	1.446481
-66.943400	53.969350	175.884600	.958425
-65.253590	53.949630	176.548400	.435620
-63.567000	53.906200	176.568000	.174492
-61.886040	53.839190	176.484500	.092151
-60.213040	53.748820	176.407600	.066054
-58.550290	53.635360	176.353900	.072886
-56.899960	53.499180	176.351500	.068133
-55.264110	53.340690	176.402100	.039866
-53.644650	53.160350	176.442900	.017343
-52.043340	52.958710	176.461900	.007978
-50.461760	52.736320	176.467200	.006008
-48.901280	52.493820	176.465300	.004253
-47.363030	52.231850	176.462000	.002276
-45.847900	51.951060	176.459900	.001063
-44.356530	51.652150	176.458900	.000421
-42.889220	51.335790	176.458600	.000148
-41.445980	51.002650	176.458500	.000065
-40.026490	50.653380	176.458600	.000056
-38.630060	50.288590	176.458600	.000052
-37.255620	49.908830	176.458600	.000048
-97.453060	51.026530	176.561200	.077176
-96.042340	51.408490	176.649600	.112156
-94.608220	51.774700	176.671300	.120363
-93.149600	52.124570	176.577300	.097737
-91.665800	52.457450	176.397600	.149666
-90.156560	52.772640	176.152200	.279925
-88.621980	53.069450	175.899500	.425130
-87.062480	53.347160	175.717600	.541792
-85.478850	53.605050	175.641400	.613908
-83.872130	53.842430	175.649900	.636133

-82.243660	54.058630	175.813200	.543410
-80.595020	54.253030	176.008100	.416281
-78.928000	54.425040	176.120300	.316946
-77.244600	54.574130	175.912900	.335229
-75.546970	54.699840	175.450600	.537102
-73.837420	54.801750	175.097300	.757980
-72.118360	54.879540	174.844900	.916252
-70.392330	54.932960	174.821800	1.026770
-68.661900	54.961820	174.987100	1.078924
-66.929700	54.966030	175.887800	.739358
-65.198370	54.945580	176.382900	.340046
-63.470540	54.900540	176.528700	.115938
-61.748820	54.831050	176.486100	.053457
-60.035740	54.737340	176.428000	.043090
-58.333780	54.619720	176.374800	.059086
-56.645260	54.478570	176.379300	.052033
-54.972420	54.314330	176.417000	.028840
-53.317280	54.127510	176.443600	.012692
-51.681740	53.918690	176.456000	.005197
-50.067420	53.688470	176.460400	.002643
-48.475770	53.437500	176.460800	.001782
-46.907930	53.166490	176.460000	.001091
-45.364770	52.876130	176.459200	.000561
-43.846840	52.567130	176.458800	.000238
-42.354370	52.240220	176.458600	.000075
-40.887210	51.896080	176.458600	.000049
-39.444820	51.535370	176.458600	.000050
-38.026250	51.158730	176.458600	.000048
-36.630120	50.766700	176.458600	.000046
-98.097820	51.886770	176.534400	.045494
-96.663910	52.281200	176.562100	.059746
-95.206040	52.659450	176.593400	.075203
-93.722790	53.020900	176.606300	.085155
-92.213200	53.364910	176.582400	.087693
-90.676770	53.690770	176.522900	.094353
-89.113440	53.997730	176.446900	.116903
-87.523560	54.285050	176.373500	.146572
-85.907830	54.551970	176.316800	.173234
-84.267300	54.797760	176.292100	.185255
-82.603340	55.021710	176.326500	.156181
-80.917590	55.223140	176.396400	.121915
-79.211960	55.401440	176.476300	.147301
-77.488560	55.556020	176.290900	.141252
-75.749730	55.686380	175.841400	.312359
-73.997920	55.792100	175.440700	.564019
-72.235800	55.872810	175.094300	.772774
-70.466090	55.928230	175.067700	.809764
-68.691570	55.958180	175.391400	.734097
-66.915130	55.962550	175.912000	.502087
-65.139640	55.941330	176.364700	.202724
-63.367970	55.894590	176.495400	.065424
-61.602940	55.822490	176.476400	.029915
-59.847310	55.725280	176.422200	.036217
-58.103760	55.603290	176.373300	.057276
-56.374820	55.456930	176.388900	.045161
-54.662870	55.286670	176.424700	.023020
-52.970100	55.093080	176.444600	.010335
-51.298500	54.876750	176.453900	.004364
-49.649800	54.638340	176.457700	.001766
-48.025460	54.378550	176.458900	.000811
-46.426660	54.098120	176.459000	.000464
-44.854210	53.797780	176.458800	.000246
-43.308580	53.478290	176.458600	.000109
-41.789850	53.140400	176.458600	.000056
-40.297680	52.784830	176.458600	.000046
-38.831280	52.412250	176.458600	.000046
-37.389370	52.023280	176.458600	.000046

-35.970190	51.618480	176.458600	.000046
-98.778840	52.740810	176.504300	.027832
-97.320270	53.148240	176.512800	.031978
-95.837370	53.539010	176.526700	.039366
-94.328290	53.912530	176.540200	.048084
-92.791720	54.268120	176.559100	.060888
-91.226900	54.605080	176.582300	.078779
-89.633560	54.922620	176.597400	.093181
-88.011890	55.219970	176.601300	.098524
-86.362530	55.496330	176.596200	.094328
-84.686490	55.750910	176.583100	.082325
-82.985150	55.982970	176.569100	.072662
-81.260250	56.191770	176.576600	.100036
-79.513790	56.376650	176.624000	.178449
-77.748020	56.536990	176.488600	.164834
-75.965460	56.672260	176.036000	.207273
-74.168790	56.781970	175.604800	.468944
-72.360860	56.865740	175.177000	.713477
-70.544630	56.923280	175.185800	.728075
-68.723180	56.954370	175.518300	.583212
-66.899620	56.958910	176.019400	.341772
-65.077100	56.936880	176.342100	.137768
-63.258730	56.888360	176.453500	.052892
-61.447630	56.813510	176.465500	.020152
-59.646790	56.712620	176.413600	.033830
-57.859090	56.586040	176.370300	.056061
-56.087300	56.434210	176.394200	.040740
-54.333970	56.257640	176.428800	.019690
-52.601470	56.056940	176.445700	.008933
-50.891880	55.832740	176.453500	.003949
-49.207030	55.585770	176.456800	.001623
-47.548420	55.316760	176.458100	.000614
-45.917200	55.026500	176.458500	.000233
-44.314130	54.715760	176.458600	.000112
-42.739580	54.385350	176.458600	.000061
-41.193420	54.036030	176.458600	.000046
-39.675070	53.668560	176.458600	.000046
-38.183400	53.283620	176.458600	.000046
-36.716770	52.881820	176.458600	.000046
-35.272900	52.463700	176.458600	.000046
-99.499240	53.588130	176.482800	.016056
-98.014280	54.009130	176.485700	.017671
-96.504860	54.412960	176.492100	.021130
-94.968610	54.799050	176.500600	.025949
-93.403790	55.166730	176.512400	.033489
-91.809290	55.515250	176.529100	.045076
-90.184560	55.843850	176.558400	.063913
-88.529620	56.151670	176.602100	.091411
-86.844990	56.437890	176.642800	.117443
-85.131650	56.701690	176.669200	.134741
-83.390960	56.942230	176.685600	.148588
-81.624720	57.158780	176.703500	.174895
-79.835050	57.350580	176.737700	.231935
-78.024370	57.516980	176.621500	.215250
-76.195360	57.657400	176.153100	.147359
-74.350940	57.771310	175.683400	.404594
-72.494220	57.858320	175.300900	.657199
-70.628410	57.918090	175.201500	.710117
-68.756890	57.950390	175.568200	.538049
-66.883060	57.955100	176.033000	.287866
-65.010370	57.932220	176.302700	.129597
-63.142240	57.881810	176.426800	.051308
-61.282040	57.804080	176.446500	.027583
-59.433060	57.699310	176.409500	.031709
-57.598450	57.567890	176.373500	.052393
-55.781200	57.410300	176.401700	.035535
-53.984060	57.227100	176.433200	.016663

-52.209560	57.018940	176.447400	.007615
-50.459930	56.786500	176.453800	.003480
-48.737060	56.530560	176.456700	.001496
-47.042490	56.251900	176.458000	.000591
-45.377330	55.951360	176.458400	.000225
-43.742270	55.629770	176.458500	.000089
-42.137470	55.287960	176.458500	.000069
-40.562610	54.926740	176.458600	.000049
-39.016750	54.546860	176.458600	.000046
-37.498410	54.149020	176.458600	.000046
-36.005400	53.733830	176.458600	.000046
-34.534880	53.301770	176.458600	.000046
-100.262700	54.428120	176.470200	.008723
-98.749250	54.863350	176.471100	.009309
-97.211560	55.280850	176.474200	.011070
-95.646590	55.680060	176.478900	.013890
-94.052090	56.060360	176.485600	.018036
-92.426510	56.420970	176.495300	.024189
-90.768940	56.761100	176.510400	.034114
-89.079190	57.079890	176.533200	.049584
-87.357570	57.376450	176.567900	.072479
-85.605000	57.649890	176.617800	.104941
-83.822840	57.899360	176.671500	.142823
-82.012920	58.124030	176.727200	.189861
-80.177480	58.323120	176.794100	.256868
-78.319110	58.495910	176.722800	.252481
-76.440710	58.641750	176.279600	.102923
-74.545430	58.760110	175.834500	.329456
-72.636660	58.850530	175.449200	.582620
-70.717930	58.912650	175.250600	.706196
-68.792920	58.946230	175.507600	.563601
-66.865380	58.951130	175.992400	.297095
-64.939080	58.927340	176.278800	.126842
-63.017780	58.874950	176.402400	.057791
-61.105210	58.794160	176.436900	.027436
-59.204930	58.685300	176.409900	.031060
-57.320400	58.548780	176.379500	.047516
-55.454840	58.385130	176.409900	.030340
-53.611270	58.194950	176.437300	.013791
-51.792370	57.978940	176.449300	.006282
-50.000500	57.737840	176.454600	.002881
-48.237630	57.472490	176.457000	.001258
-46.505310	57.183730	176.458000	.000530
-44.804610	56.872430	176.458400	.000216
-43.136050	56.539500	176.458500	.000095
-41.499610	56.185780	176.458500	.000071
-39.894630	55.812120	176.458600	.000050
-38.319800	55.419290	176.458600	.000046
-36.773090	55.007980	176.458600	.000046
-35.251730	54.578750	176.458600	.000046
-33.752120	54.132060	176.458600	.000046
-101.073700	55.260130	176.463600	.004358
-99.529010	55.710320	176.463900	.004580
-97.960900	56.142140	176.465300	.005474
-96.365430	56.555100	176.467700	.007012
-94.739640	56.948590	176.471200	.009324
-93.081440	57.321860	176.476400	.012733
-91.389490	57.674070	176.484200	.017822
-89.663250	58.004340	176.495600	.025349
-87.902820	58.311730	176.512700	.036844
-86.109000	58.595320	176.538300	.054838
-84.283100	58.854170	176.579500	.084697
-82.427000	59.087400	176.649700	.138211
-80.543050	59.294160	176.742300	.217420
-78.634010	59.473690	176.739700	.235688
-76.703000	59.625280	176.409900	.089655
-74.753460	59.748340	176.032600	.249595

-72.789070 59.842370 175.681800 .490614
-70.813740 59.906970 175.318000 .689544
-68.831480 59.941910 175.420000 .613939
-66.846440 59.947010 175.932100 .329984
-64.862770 59.922250 176.256100 .137332
-62.884610 59.867760 176.391300 .056218
-60.916040 59.783740 176.436500 .026889
-58.961010 59.670550 176.418500 .023843
-57.023270 59.528640 176.398800 .035698
-55.106350 59.358580 176.419800 .023607
-53.213500 59.161040 176.442400 .010516
-51.347600 58.936760 176.451600 .004708
-49.511150 58.686560 176.455600 .002175
-47.706190 58.411320 176.457300 .000972
-45.934240 58.111950 176.458100 .000425
-44.196280 57.789400 176.458400 .000177
-42.492650 57.444580 176.458500 .000088
-40.823030 57.078400 176.458500 .000074
-39.186360 56.691730 176.458600 .000049
-37.580830 56.285340 176.458600 .000046
-36.003750 55.859910 176.458600 .000046
-34.451600 55.415950 176.458600 .000046
-32.919830 54.953830 176.458600 .000046
-101.937200 56.083360 176.460500 .001991
-100.358100 56.549350 176.460600 .002066
-98.756940 56.996240 176.461200 .002503
-97.128790 57.423640 176.462300 .003314
-95.469860 57.830970 176.464000 .004538
-93.777320 58.217490 176.466600 .006351
-92.049300 58.582370 176.470500 .009015
-90.284800 58.924680 176.476200 .012939
-88.483640 59.243460 176.484900 .018805
-86.646400 59.537720 176.498300 .028016
-84.774360 59.806450 176.520800 .044685
-82.869400 60.048700 176.562300 .079372
-80.933970 60.263580 176.636200 .142490
-78.971000 60.450230 176.659000 .166877
-76.983890 60.607900 176.450100 .072175
-74.976370 60.735940 176.176100 .197922
-72.952470 60.833800 175.881700 .406655
-70.916480 60.901050 175.437800 .644513
-68.872850 60.937420 175.371200 .650491
-66.826130 60.942730 175.869100 .369535
-64.780930 60.916960 176.243100 .151476
-62.741830 60.860230 176.397200 .060583
-60.713310 60.772790 176.453500 .027947
-58.699720 60.654990 176.446500 .010290
-56.705200 60.507380 176.429800 .016625
-54.733590 60.330550 176.439200 .012056
-52.788400 60.125220 176.449300 .006008
-50.872730 59.892220 176.454200 .002943
-48.989200 59.632420 176.456600 .001428
-47.139900 59.346780 176.457700 .000673
-45.326320 59.036270 176.458200 .000300
-43.549270 58.701890 176.458400 .000136
-41.808860 58.344600 176.458500 .000071
-40.104370 57.965370 176.458600 .000054
-38.434220 57.565060 176.458600 .000048
-36.795950 57.144450 176.458600 .000046
-35.186060 56.704150 176.458600 .000046
-33.600030 56.244660 176.458600 .000046
-32.032190 55.766200 176.458600 .000046
-102.859900 56.896880 176.459200 .000813
-101.242000 57.379640 176.459200 .000838
-99.604390 57.842470 176.459500 .001038
-97.940930 58.285070 176.459900 .001428
-96.246660 58.706940 176.460700 .002050

-94.517820	59.107400	176.461900	.002971
-92.751850	59.485580	176.463700	.004349
-90.947170	59.840560	176.466500	.006382
-89.103220	60.171320	176.470800	.009405
-87.220290	60.476800	176.477600	.014145
-85.299550	60.755960	176.488900	.022578
-83.342830	61.007760	176.509700	.039790
-81.352710	61.231220	176.543700	.068926
-79.332320	61.425420	176.528100	.061873
-77.285290	61.589540	176.375400	.074611
-75.215710	61.722870	176.193400	.205626
-73.128000	61.824790	175.990900	.352862
-71.026890	61.894870	175.572600	.586315
-68.917300	61.932760	175.369300	.663877
-66.804310	61.938290	175.834000	.401418
-64.692990	61.911450	176.248000	.164223
-62.588420	61.852340	176.414200	.068111
-60.495590	61.761240	176.469300	.036101
-58.419300	61.638580	176.471300	.017658
-56.364090	61.484900	176.455200	.004526
-54.334160	61.300890	176.452900	.003723
-52.333320	61.087320	176.454800	.002525
-50.364880	60.845090	176.456500	.001483
-48.431600	60.575160	176.457500	.000803
-46.535590	60.278550	176.458100	.000405
-44.678250	59.956310	176.458300	.000192
-42.860180	59.609490	176.458500	.000098
-41.081100	59.239120	176.458500	.000071
-39.339830	58.846180	176.458600	.000050
-37.634110	58.431540	176.458600	.000046
-35.960610	57.995940	176.458600	.000046
-34.314830	57.539980	176.458600	.000046
-32.690990	57.064010	176.458600	.000046
-31.082000	56.568130	176.458600	.000046
-103.849600	57.699570	176.458700	.000296
-102.187300	58.200280	176.458700	.000297
-100.509000	58.680030	176.458800	.000396
-98.806840	59.138710	176.459100	.000557
-97.074520	59.575940	176.459300	.000848
-95.307120	59.991050	176.459800	.001296
-93.501110	60.383240	176.460600	.001960
-91.654170	60.751550	176.461900	.002962
-89.765210	61.094930	176.463800	.004409
-87.834170	61.412270	176.466700	.006458
-85.861950	61.702450	176.470600	.009166
-83.850420	61.964360	176.473800	.011444
-81.802150	62.196920	176.466600	.008518
-79.720490	62.399150	176.418300	.038642
-77.609360	62.570130	176.286200	.142479
-75.473210	62.709080	176.162500	.233200
-73.316960	62.815350	176.030200	.333314
-71.145790	62.888430	175.692600	.513304
-68.965200	62.927950	175.507600	.600275
-66.780790	62.933720	175.873600	.385178
-64.598260	62.905720	176.264600	.159665
-62.423240	62.844070	176.420100	.065012
-60.261290	62.749080	176.464400	.031575
-58.117710	62.621230	176.466200	.013934
-55.997520	62.461110	176.460000	.004548
-53.905320	62.269450	176.457700	.001528
-51.845240	62.047150	176.457400	.000912
-49.820820	61.795150	176.457700	.000624
-47.834930	61.514500	176.458100	.000384
-45.889670	61.206310	176.458300	.000218
-43.986290	60.871700	176.458500	.000121
-42.125070	60.511780	176.458500	.000071
-40.305270	60.127630	176.458500	.000074

-38.525020 59.720250 176.458600 .000049
-36.781200 59.290500 176.458600 .000046
-35.069400 58.839090 176.458600 .000046
-33.383770 58.366500 176.458600 .000046
-31.716990 57.872960 176.458600 .000046
-30.060130 57.358350 176.458600 .000046
-104.916500 58.490070 176.458600 .000093
-103.202300 59.010120 176.458600 .000096
-101.477600 59.507980 176.458600 .000127
-99.732440 59.983750 176.458700 .000190
-97.958720 60.437220 176.458800 .000319
-96.150010 60.867820 176.459000 .000511
-94.301540 61.274790 176.459300 .000812
-92.410060 61.657170 176.459700 .001265
-90.473700 62.013880 176.460600 .001883
-88.491920 62.343760 176.461600 .002595
-86.465330 62.645610 176.462400 .003024
-84.395630 62.918240 176.461000 .002465
-82.285480 63.160470 176.450600 .010385
-80.138370 63.371240 176.414700 .042051
-77.958560 63.549540 176.335900 .107175
-75.750930 63.694510 176.254000 .169186
-73.520860 63.805420 176.151700 .237099
-71.274150 63.881700 175.972900 .348246
-69.016910 63.922960 175.807800 .418747
-66.755400 63.928990 176.070900 .268520
-64.495980 63.899750 176.316500 .116744
-62.244980 63.835380 176.418200 .046912
-60.008560 63.736250 176.450900 .019806
-57.792640 63.602840 176.459200 .008669
-55.602750 63.435840 176.459500 .003465
-53.443960 63.236060 176.458800 .001242
-51.320750 63.004450 176.458400 .000442
-49.236890 62.742080 176.458300 .000241
-47.195330 62.450080 176.458400 .000168
-45.198120 62.129630 176.458500 .000099
-43.246250 61.781950 176.458500 .000077
-41.339580 61.408210 176.458500 .000061
-39.476710 61.009530 176.458600 .000050
-37.654880 60.586900 176.458600 .000048
-35.869840 60.141170 176.458600 .000046
-34.115770 59.672970 176.458600 .000046
-32.385130 59.182660 176.458600 .000046
-30.668580 58.670220 176.458600 .000046
-28.954870 58.135290 176.458600 .000046
-106.073800 59.266680 176.458600 .000053
-104.297500 59.807790 176.458600 .000053
-102.518800 60.325180 176.458600 .000053
-100.725000 60.819250 176.458600 .000066
-98.905530 61.289990 176.458600 .000100
-97.052100 61.737010 176.458700 .000178
-95.158340 62.159620 176.458800 .000310
-93.219700 62.556880 176.459000 .000482
-91.233350 62.927700 176.459200 .000701
-89.198040 63.270870 176.459500 .000885
-87.113920 63.585110 176.459400 .000814
-84.982480 63.869130 176.457800 .001915
-82.806390 64.121680 176.451900 .007787
-80.589290 64.341560 176.436200 .022296
-78.335770 64.527670 176.406400 .048442
-76.051160 64.679080 176.366100 .079646
-73.741460 64.794950 176.311600 .117790
-71.413090 64.874680 176.233900 .167965
-69.072890 64.917820 176.166200 .202387
-66.727910 64.924110 176.281300 .130641
-64.385280 64.893550 176.380100 .063271
-62.052090 64.826280 176.429900 .028474

-59.735260 64.722690 176.450500 .012547
-57.441380 64.583340 176.457400 .005465
-55.176610 64.408980 176.458900 .002324
-52.946510 64.200520 176.458800 .000917
-50.755950 63.959000 176.458600 .000323
-48.608900 63.685590 176.458500 .000119
-46.508400 63.381530 176.458500 .000079
-44.456330 63.048090 176.458500 .000071
-42.453320 62.686580 176.458600 .000050
-40.498620 62.298210 176.458600 .000050
-38.589950 61.884150 176.458600 .000048
-36.723370 61.445380 176.458600 .000046
-34.893150 60.982680 176.458600 .000046
-33.091610 60.496560 176.458600 .000046
-31.309040 59.987160 176.458600 .000046
-29.533510 59.454220 176.458600 .000046
-27.750760 58.896990 176.458600 .000046
-107.339200 60.027230 176.458600 .000046
-105.487000 60.591610 176.458600 .000046
-103.643800 61.130270 176.458600 .000046
-101.793500 61.644070 176.458600 .000046
-99.922550 62.133320 176.458600 .000053
-98.020040 62.597820 176.458600 .000066
-96.077510 63.037030 176.458600 .000104
-94.088710 63.450070 176.458600 .000159
-92.049460 63.835860 176.458700 .000223
-89.957580 64.193130 176.458700 .000239
-87.812570 64.520550 176.458500 .000402
-85.615560 64.816710 176.457400 .001625
-83.369120 65.080260 176.454500 .004828
-81.077050 65.309890 176.447700 .011347
-78.744270 65.504390 176.435800 .021867
-76.376630 65.662700 176.418800 .035452
-73.980770 65.783920 176.396400 .051627
-71.563890 65.867360 176.370500 .069055
-69.133670 65.912500 176.355900 .077943
-66.698060 65.919100 176.386300 .055748
-64.265090 65.887100 176.420200 .032180
-61.842790 65.816700 176.442000 .016473
-59.438900 65.708320 176.452900 .007818
-57.060850 65.562580 176.457300 .003536
-54.715470 65.380330 176.458600 .001549
-52.408880 65.162570 176.458700 .000638
-50.146370 64.910470 176.458600 .000243
-47.932120 64.625290 176.458500 .000093
-45.769150 64.308400 176.458600 .000048
-43.659080 63.961160 176.458600 .000046
-41.602020 63.584970 176.458600 .000046
-39.596350 63.181100 176.458600 .000046
-37.638600 62.750720 176.458600 .000046
-35.723270 62.294770 176.458600 .000046
-33.842640 61.813940 176.458600 .000046
-31.986660 61.308540 176.458600 .000046
-30.142740 60.778430 176.458600 .000046
-28.295590 60.222970 176.458600 .000046
-26.427080 59.640870 176.458600 .000046
-108.736700 60.768890 176.458600 .000046
-106.789100 61.359350 176.458600 .000046
-104.866900 61.921510 176.458600 .000046
-102.949500 62.456830 176.458600 .000046
-101.019200 62.966030 176.458600 .000046
-99.061880 63.449270 176.458600 .000053
-97.066180 63.906190 176.458600 .000055
-95.023590 64.336020 176.458600 .000062
-92.928180 64.737730 176.458600 .000074
-90.776390 65.110020 176.458600 .000116
-88.566850 65.451470 176.458300 .000459

-86.300130	65.760610	176.457500	.001442
-83.978550	66.035940	176.455700	.003578
-81.606060	66.276030	176.451800	.007470
-79.187900	66.479550	176.445400	.013400
-76.730480	66.645290	176.436200	.021000
-74.241170	66.772270	176.424600	.029588
-71.728080	66.859700	176.413200	.037327
-69.199870	66.907030	176.409300	.039455
-66.665550	66.913940	176.420200	.031499
-64.134220	66.880400	176.435500	.020443
-61.614990	66.806620	176.447400	.011570
-59.116610	66.693070	176.454200	.005922
-56.647430	66.540450	176.457300	.002802
-54.215080	66.349720	176.458400	.001254
-51.826330	66.121990	176.458700	.000540
-49.486860	65.858530	176.458600	.000211
-47.201050	65.560780	176.458600	.000093
-44.971820	65.230190	176.458600	.000050
-42.800360	64.868270	176.458600	.000046
-40.685980	64.476460	176.458600	.000046
-38.625890	64.056100	176.458600	.000046
-36.614990	63.608330	176.458600	.000046
-34.645670	63.134020	176.458600	.000046
-32.707610	62.633670	176.458600	.000046
-30.787590	62.107330	176.458600	.000046
-28.869240	61.554470	176.458600	.000046
-26.932890	60.973880	176.458600	.000046
-24.955330	60.363580	176.458600	.000046

Q.04a

-89.453400	32.991660	185.869400	.018025
-88.345930	33.191160	185.866300	.021319
-87.228100	33.381660	185.853100	.034607
-86.100230	33.562890	185.829100	.058073
-84.962690	33.734530	185.792600	.091782
-83.815900	33.896310	185.744500	.133692
-82.660300	34.047950	185.691100	.178931
-81.496410	34.189190	185.643100	.221011
-80.324730	34.319790	185.607500	.255246
-79.145840	34.439500	185.580400	.282355
-77.960310	34.548100	185.546000	.310197
-76.768760	34.645400	185.495500	.343677
-75.571840	34.731200	185.452400	.368172
-74.370180	34.805370	185.460000	.356361
-73.164480	34.867740	185.535500	.297348

-71.955430	34.918200	185.645400	.211362
-70.743730	34.956650	185.746000	.130132
-69.530080	34.983020	185.815300	.070877
-68.315220	34.997260	185.854000	.034146
-67.099880	34.999340	185.872000	.014512
-65.884770	34.989250	185.879200	.005404
-64.670640	34.967020	185.881600	.001761
-63.458190	34.932690	185.882400	.000485
-62.248150	34.886320	185.882700	.000151
-61.041230	34.828000	185.882700	.000094
-59.838120	34.757850	185.882700	.000092
-58.639500	34.676010	185.882700	.000092
-57.446040	34.582610	185.882700	.000092
-56.258390	34.477850	185.882700	.000092
-55.077160	34.361910	185.882700	.000092
-53.902950	34.235010	185.882700	.000092
-52.736340	34.097380	185.882700	.000092
-51.577860	33.949260	185.882700	.000092
-50.428010	33.790910	185.882700	.000092
-49.287250	33.622600	185.882700	.000092
-48.156040	33.444620	185.882700	.000092
-47.034740	33.257250	185.882700	.000092
-45.923690	33.060790	185.882700	.000092
-44.823220	32.855560	185.882700	.000092
-89.701210	33.918400	185.847300	.037924
-88.583520	34.125020	185.836200	.046922
-87.455040	34.322360	185.798800	.076581
-86.316120	34.510110	185.731900	.128079
-85.167100	34.687960	185.631600	.201842
-84.008420	34.855620	185.499700	.294065
-82.840550	35.012790	185.352900	.394323
-81.663990	35.159200	185.219500	.488870
-80.479300	35.294580	185.122400	.565242
-79.287040	35.418700	185.056200	.623639
-78.087840	35.531310	184.975000	.687473
-76.882320	35.632210	184.841400	.777313
-75.671150	35.721200	184.713600	.856395
-74.455030	35.798110	184.730900	.838911
-73.234660	35.862810	184.949300	.697591
-72.010770	35.915150	185.258900	.483474
-70.784100	35.955040	185.530700	.285672
-69.555380	35.982390	185.712000	.148958
-68.325390	35.997170	185.810800	.069797
-67.094890	35.999320	185.856400	.029151
-65.864640	35.988860	185.874300	.010779
-64.635410	35.965800	185.880300	.003466
-63.407950	35.930180	185.882100	.000948
-62.183030	35.882080	185.882600	.000220
-60.961370	35.821590	185.882700	.000120
-59.743720	35.748840	185.882700	.000117
-58.530780	35.663950	185.882700	.000118
-57.323260	35.567100	185.882700	.000092
-56.121820	35.458470	185.882700	.000092
-54.927120	35.338260	185.882700	.000092
-53.739770	35.206700	185.882700	.000092
-52.560380	35.064020	185.882700	.000092
-51.389480	34.910500	185.882700	.000092
-50.227610	34.746390	185.882700	.000092
-49.075240	34.571990	185.882700	.000092
-47.932830	34.387580	185.882700	.000092
-46.800740	34.193490	185.882700	.000092
-45.679330	33.990000	185.882700	.000092
-44.568900	33.777440	185.882700	.000092
-89.960660	34.843320	185.764300	.100203
-88.832330	35.057210	185.721900	.127900
-87.692770	35.261540	185.593600	.208967
-86.542320	35.455950	185.369700	.347522

-85.381340	35.640140	185.037500	.545238
-84.210270	35.813810	184.602400	.792005
-83.029580	35.976620	184.114400	1.063048
-81.839790	36.128310	183.665300	1.322251
-80.641480	36.268600	183.332200	1.537548
-79.435230	36.397220	183.127500	1.693761
-78.221690	36.513940	182.882600	1.874560
-77.001530	36.618520	182.406800	2.193226
-75.775430	36.710780	181.887500	2.504742
-74.544130	36.790520	181.913900	2.487158
-73.308360	36.857590	182.714700	2.075528
-72.068890	36.911860	183.822700	1.423339
-70.826490	36.953220	184.748900	.815444
-69.581950	36.981590	185.342300	.407239
-68.336070	36.996900	185.657100	.183603
-67.089650	36.999130	185.800000	.075262
-65.843500	36.988290	185.856400	.027600
-64.598400	36.964370	185.875600	.008855
-63.355190	36.927440	185.881100	.002366
-62.114630	36.877570	185.882500	.000467
-60.877510	36.814860	185.882800	.000153
-59.644590	36.739430	185.882800	.000152
-58.416640	36.651430	185.882700	.000122
-57.194370	36.551040	185.882700	.000110
-55.978490	36.438440	185.882700	.000116
-54.769680	36.313860	185.882700	.000092
-53.568590	36.177530	185.882700	.000092
-52.375820	36.029710	185.882700	.000092
-51.191970	35.870660	185.882700	.000092
-50.017550	35.700660	185.882700	.000092
-48.853080	35.520030	185.882700	.000092
-47.698980	35.329070	185.882700	.000092
-46.555660	35.128100	185.882700	.000092
-45.423470	34.917440	185.882700	.000092
-44.302690	34.697410	185.882700	.000092
-90.232250	35.766320	185.535400	.240001
-89.092860	35.987640	185.396100	.315224
-87.941790	36.199090	185.008300	.519187
-86.779330	36.400320	184.340900	.864014
-85.605870	36.591000	183.358500	1.349316
-84.421870	36.770790	182.064400	1.951454
-83.227800	36.939400	180.600200	2.606196
-82.024180	37.096500	179.214900	3.261459
-80.811620	37.241800	178.162200	3.822404
-79.590740	37.375040	177.610400	4.177471
-78.362190	37.495960	176.938400	4.655545
-77.126670	37.604320	175.224500	5.678868
-75.884930	37.699920	172.986800	6.952091
-74.637700	37.782550	172.900000	6.817541
-73.385770	37.852060	175.684400	5.678544
-72.129940	37.908310	179.503800	3.924515
-70.871030	37.951180	182.511700	2.202599
-69.609860	37.980580	184.329500	1.059131
-68.347290	37.996460	185.250100	.455293
-67.084140	37.998780	185.655000	.179061
-65.821270	37.987530	185.811500	.064000
-64.559530	37.962740	185.864300	.020042
-63.299760	37.924460	185.879500	.005088
-62.042780	37.872770	185.882900	.000954
-60.789440	37.807780	185.883400	.000631
-59.540510	37.729610	185.883100	.000533
-58.296810	37.638420	185.882900	.000364
-57.059080	37.534390	185.882800	.000229
-55.828080	37.417740	185.882800	.000135
-54.604500	37.288680	185.882700	.000112
-53.389020	37.147460	185.882700	.000093
-52.182290	36.994370	185.882700	.000092

-50.984880	36.829660	185.882700	.000092
-49.797380	36.653650	185.882700	.000092
-48.620270	36.466650	185.882700	.000092
-47.454020	36.268990	185.882700	.000092
-46.299010	36.060990	185.882700	.000092
-45.155610	35.843010	185.882700	.000092
-44.024070	35.615370	185.882700	.000092
-90.516530	36.687310	185.010500	.509349
-89.365660	36.916220	184.619000	.689575
-88.202590	37.134950	183.588600	1.149453
-87.027650	37.343150	181.836400	1.908150
-85.841200	37.540460	179.275600	2.945676
-84.643710	37.726540	175.872600	4.182430
-83.435650	37.901050	171.941900	5.566891
-82.217600	38.063690	168.104800	7.003477
-80.990140	38.214140	164.937500	8.506202
-79.753940	38.352110	163.826300	9.083226
-78.509670	38.477340	162.269000	10.010530
-77.258070	38.589580	156.975800	12.903210
-75.999910	38.688600	148.004200	16.889440
-74.735980	38.774210	146.998800	16.414090
-73.467090	38.846230	155.438300	13.541810
-72.194090	38.904510	167.726200	9.505098
-70.917830	38.948930	176.805700	5.322644
-69.639210	38.979390	181.916800	2.461684
-68.359090	38.995840	184.337500	1.010374
-67.078360	38.998240	185.347200	.376556
-65.797930	38.986590	185.722200	.128504
-64.518690	38.960900	185.844700	.038558
-63.241520	38.921240	185.878800	.009001
-61.967320	38.867690	185.885800	.002362
-60.696920	38.800350	185.885900	.002290
-59.431210	38.719360	185.884900	.001808
-58.170970	38.624900	185.884000	.001213
-56.917050	38.517150	185.883400	.000738
-55.670200	38.396340	185.883000	.000411
-54.431160	38.262690	185.882800	.000203
-53.200640	38.116470	185.882700	.000118
-51.979310	37.957960	185.882700	.000119
-50.767770	37.787460	185.882700	.000092
-49.566610	37.605300	185.882700	.000092
-48.376340	37.411790	185.882700	.000092
-47.197410	37.207270	185.882700	.000092
-46.030250	36.992090	185.882700	.000092
-44.875180	36.766620	185.882700	.000092
-43.732470	36.531200	185.882700	.000092
-90.814130	37.606180	184.005400	.961388
-89.651300	37.842850	183.044400	1.331171
-88.475770	38.069030	180.593600	2.230252
-87.287830	38.284350	176.468000	3.650802
-86.087840	38.488450	170.597800	5.390972
-84.876290	38.680970	162.640700	7.422298
-83.653650	38.861550	153.351800	9.647054
-82.420500	39.029860	143.616100	12.597180
-81.177470	39.185590	134.572600	16.111560
-79.925230	39.328410	133.072700	17.071840
-78.664510	39.458070	132.635100	17.479920
-77.396060	39.574290	121.396500	21.813890
-76.120680	39.676830	100.764500	27.659940
-74.839220	39.765490	93.153380	26.486270
-73.552520	39.840080	107.430200	23.866950
-72.261480	39.900440	139.305900	19.731990
-70.967010	39.946460	164.050000	11.072570
-69.670030	39.978020	176.996400	4.957984
-68.371470	39.995060	182.644000	1.914819
-67.072270	39.997550	184.830800	.672012
-65.773400	39.985470	185.589600	.219557

-64.475770	39.958870	185.825800	.062318
-63.180340	39.917780	185.887100	.013973
-61.888020	39.862300	185.896900	.007723
-60.599740	39.792560	185.894500	.007067
-59.316390	39.708680	185.890600	.005248
-58.038840	39.610860	185.887500	.003496
-56.767930	39.499290	185.885400	.002126
-55.504470	39.374200	185.884000	.001177
-54.249260	39.235840	185.883300	.000589
-53.003000	39.084480	185.882900	.000268
-51.766410	38.920440	185.882700	.000117
-50.540120	38.744010	185.882700	.000116
-49.324710	38.555530	185.882700	.000092
-48.120720	38.355340	185.882700	.000092
-46.928620	38.143810	185.882700	.000092
-45.748810	37.921300	185.882700	.000092
-44.581620	37.688170	185.882700	.000092
-43.427300	37.444810	185.882700	.000092
-91.125630	38.522790	182.403900	1.589402
-89.950410	38.767390	180.318600	2.235086
-88.761920	39.001200	174.942700	3.712685
-87.560450	39.223820	165.973700	5.790757
-86.346370	39.434880	153.669400	8.029263
-85.120160	39.633990	137.768100	10.058650
-83.882300	39.820790	120.819700	11.698340
-82.633390	39.994930	102.207900	15.177790
-81.374080	40.156070	85.259600	19.727170
-80.105060	40.303890	84.740280	20.374830
-78.827100	40.438090	92.438000	18.275980
-77.540980	40.558400	84.245230	20.165550
-76.247540	40.664560	55.236890	24.538160
-74.947690	40.756360	38.541610	26.403150
-73.642300	40.833600	49.976300	29.217680
-72.332310	40.896110	92.497380	27.677270
-71.018690	40.943760	143.162300	18.063090
-69.702430	40.976440	169.541500	8.081321
-68.384490	40.994090	180.418100	2.953479
-67.065880	40.996670	184.287300	.964363
-65.747600	40.984170	185.468200	.319653
-64.430650	40.956610	185.838100	.089504
-63.116020	40.914060	185.925100	.026122
-61.804690	40.856610	185.930200	.022784
-60.497620	40.784390	185.918100	.018910
-59.195770	40.697540	185.906000	.013627
-57.900040	40.596260	185.896900	.008970
-56.611330	40.480750	185.890700	.005446
-55.330490	40.351270	185.886800	.002995
-54.058330	40.208070	185.884600	.001489
-52.795620	40.051450	185.883400	.000676
-51.543080	39.881710	185.882900	.000290
-50.301390	39.699200	185.882800	.000125
-49.071130	39.504250	185.882700	.000113
-47.852850	39.297230	185.882700	.000092
-46.647030	39.078520	185.882700	.000092
-45.454070	38.848490	185.882700	.000092
-44.274280	38.607530	185.882700	.000092
-43.107920	38.356030	185.882700	.000092
-91.451740	39.437030	180.365600	2.283628
-90.263630	39.689750	176.405600	3.251886
-89.061680	39.931370	166.035000	5.386086
-87.846150	40.161480	148.158300	8.233937
-86.617400	40.379670	122.287500	10.701770
-85.375900	40.585540	91.555590	10.542630
-84.122160	40.778730	73.297820	7.900553
-82.856800	40.958850	66.320580	6.901211
-81.580460	41.125550	39.146860	12.390410
-80.293880	41.278500	40.452160	10.483410

-78.997860	41.417370	66.523010	7.806933
-77.693230	41.541890	75.625590	9.637320
-76.380870	41.651780	41.151290	10.898920
-75.061690	41.746810	23.448570	13.048850
-73.736660	41.826770	34.628850	19.831260
-72.406780	41.891490	73.761220	27.853530
-71.073040	41.940830	127.310300	20.131660
-69.736500	41.974670	163.600300	9.646902
-68.398190	41.992950	178.733000	3.717462
-67.059160	41.995620	183.980500	1.226680
-65.720480	41.982670	185.463600	.425857
-64.383220	41.954140	185.926600	.144728
-63.048400	41.910080	186.028000	.060339
-61.717090	41.850600	186.012500	.055559
-60.390290	41.775820	185.975000	.044619
-59.069010	41.685920	185.943300	.031444
-57.754210	41.581080	185.919700	.020936
-56.446830	41.461530	185.903400	.012431
-55.147770	41.327530	185.893200	.006717
-53.857880	41.179360	185.887600	.003286
-52.577970	41.017320	185.884800	.001499
-51.308780	40.841740	185.883500	.000648
-50.051000	40.652980	185.882900	.000264
-48.805250	40.451390	185.882800	.000123
-47.572090	40.237360	185.882700	.000117
-46.352010	40.011290	185.882700	.000092
-45.145370	39.773560	185.882700	.000092
-43.952510	39.524580	185.882700	.000092
-42.773630	39.264750	185.882700	.000092
-91.793200	40.348750	178.614000	2.908000
-90.591710	40.609810	172.801600	4.155860
-89.375760	40.859430	157.029300	7.234769
-88.145600	41.097210	127.206100	11.637110
-86.901570	41.322720	81.956580	13.243440
-85.644150	41.535550	37.024800	12.365850
-84.373860	41.735290	35.776120	8.725824
-83.091290	41.921560	29.994390	4.201094
-81.797160	42.093980	30.943520	6.753821
-80.492230	42.252200	39.680580	7.206726
-79.177290	42.395880	62.261930	12.379060
-77.853240	42.524730	60.806280	11.707640
-76.521000	42.638450	34.229770	11.470280
-75.181550	42.736820	21.279110	8.374701
-73.835900	42.819590	48.766100	8.056973
-72.485100	42.886590	86.944690	19.011380
-71.130210	42.937670	131.606000	17.269480
-69.772340	42.972710	163.126900	9.404459
-68.412590	42.991630	177.725400	4.255426
-67.052090	42.994400	183.553600	1.647661
-65.691960	42.980990	185.522700	.597376
-64.333320	42.951450	186.141000	.216259
-62.977290	42.905830	186.267900	.126273
-61.624970	42.844260	186.197700	.121228
-60.277440	42.766850	186.101900	.094925
-58.935750	42.673790	186.027600	.070132
-57.600940	42.565280	185.971500	.045869
-56.273990	42.441570	185.931200	.026901
-54.955840	42.302930	185.906500	.013258
-53.647390	42.149640	185.893500	.006330
-52.349470	41.982030	185.887400	.002904
-51.062890	41.800450	185.884600	.001288
-49.788320	41.605260	185.883300	.000553
-48.526440	41.396860	185.882900	.000221
-47.277780	41.175640	185.882800	.000123
-46.042850	40.942000	185.882700	.000120
-44.822010	40.696380	185.882700	.000092
-43.615560	40.439180	185.882700	.000092

-42.423690 40.170830 185.882700 .000092
-92.150770 41.257820 177.968600 3.289698
-90.935390 41.527410 171.969900 4.797635
-89.704910 41.785250 155.425200 8.017080
-88.459540 42.030900 123.163000 12.336480
-87.199620 42.263930 72.649540 15.305880
-85.925610 42.483890 29.345100 11.084010
-84.638040 42.690380 17.103980 7.004652
-83.337520 42.882970 31.410050 9.600002
-82.024790 43.061280 40.513780 7.004534
-80.700610 43.224930 78.855880 11.922690
-79.365870 43.373580 88.038460 14.007490
-78.021450 43.506890 71.377460 12.819620
-76.668370 43.624570 24.779200 5.320048
-75.307630 43.726360 32.693490 5.382009
-73.940300 43.812040 58.643950 7.198913
-72.567500 43.881390 99.125930 15.581830
-71.190370 43.934270 136.116200 15.435720
-69.810050 43.970550 162.182900 10.274650
-68.427750 43.990140 177.988100 4.710809
-67.044650 43.993000 184.380500 1.625808
-65.661930 43.979120 186.261400 .525297
-64.280810 43.948540 186.824300 .266808
-62.902460 43.901310 186.850600 .303882
-61.528050 43.837570 186.612500 .273572
-60.158730 43.757450 186.374700 .211023
-58.795620 43.661140 186.211300 .152946
-57.439790 43.548850 186.073300 .098429
-56.092310 43.420850 185.986100 .054348
-54.754150 43.277410 185.931200 .024842
-53.426270 43.118850 185.904200 .010822
-52.109530 42.945500 185.892100 .005118
-50.804760 42.757750 185.886700 .002397
-49.512690 42.555960 185.884200 .001053
-48.233980 42.340550 185.883200 .000424
-46.969190 42.111940 185.882800 .000159
-45.718810 41.870550 185.882700 .000109
-44.483210 41.616830 185.882700 .000092
-43.262650 41.351200 185.882700 .000092
-42.057300 41.074100 185.882700 .000092
-92.525320 42.164070 178.145500 3.396423
-91.295520 42.442420 172.265300 5.126801
-90.049940 42.708690 156.017300 9.092574
-88.788760 42.962440 125.082000 14.761040
-87.512310 43.203190 80.173120 19.388150
-86.221000 43.430500 37.799470 12.875310
-84.915410 43.643920 23.587210 5.967147
-83.596150 43.843030 19.351900 9.177912
-82.263960 44.027400 53.853320 13.673100
-80.919660 44.196660 89.119500 14.446490
-79.564150 44.350410 99.246770 15.971930
-78.198390 44.488330 70.968720 7.084789
-76.823410 44.610100 32.534230 6.830170
-75.440290 44.715440 18.602840 6.917353
-74.050180 44.804110 43.614950 14.052880
-72.654240 44.875890 80.901690 21.715330
-71.253690 44.930630 127.937700 19.289860
-69.849760 44.968180 160.676400 11.468780
-68.443720 44.988460 178.592100 4.886334
-67.036810 44.991420 185.416800 1.703806
-65.630330 44.977050 187.135900 .589768
-64.225530 44.945390 187.640300 .537770
-62.823690 44.896510 187.563800 .592432
-61.426040 44.830540 187.195900 .507523
-60.033810 44.747610 186.772000 .368778
-58.648170 44.647940 186.395900 .237093
-57.270290 44.531750 186.161900 .138266

-55.901260	44.399320	186.028200	.070223
-54.542130	44.250930	185.961000	.034257
-53.193900	44.086940	185.919400	.017764
-51.857480	43.907680	185.899500	.008769
-50.533710	43.713560	185.890100	.004249
-49.223370	43.504980	185.885700	.001913
-47.927120	43.282370	185.883700	.000768
-46.645540	43.046150	185.882900	.000286
-45.379100	42.796800	185.882800	.000110
-44.128160	42.534760	185.882700	.000120
-42.892960	42.260480	185.882700	.000092
-41.673610	41.974410	185.882700	.000092
-92.917770	43.067330	178.926100	3.122588
-91.673000	43.354680	173.550800	4.679714
-90.411740	43.629630	158.628100	8.170453
-89.134110	43.891690	128.767600	13.675760
-87.840450	44.140390	84.061480	18.281230
-86.531140	44.375260	40.695270	16.932970
-85.206750	44.595830	22.747590	9.598501
-83.867900	44.801650	29.447720	8.458377
-82.515380	44.992280	36.843350	10.721990
-81.150000	45.167310	62.231760	11.295570
-79.772730	45.326340	67.005970	7.426298
-78.384560	45.469020	60.971460	5.773284
-76.986600	45.595010	42.072870	12.956130
-75.579970	45.704020	32.458400	16.858710
-74.165890	45.795780	45.572790	22.748680
-72.745610	45.870080	85.668920	23.004730
-71.320400	45.926740	128.000600	18.250000
-69.891600	45.965610	160.196700	10.663680
-68.460530	45.986610	177.108500	4.894255
-67.028560	45.989670	183.711100	2.059826
-65.597030	45.974800	185.888700	.784374
-64.167300	45.942020	186.882200	.372922
-62.740720	45.891430	187.522900	.572927
-61.318600	45.823140	187.362000	.559153
-59.902260	45.737310	186.729400	.336354
-58.492950	45.634170	186.294700	.167716
-57.091900	45.513950	186.056000	.090937
-55.700260	45.376930	186.008100	.066478
-54.319140	45.223450	185.969400	.052653
-52.949590	45.053840	185.936800	.029529
-51.592580	44.868490	185.910900	.015612
-50.248970	44.667810	185.895500	.007464
-48.919560	44.452230	185.887800	.003191
-47.605040	44.222190	185.884400	.001259
-46.305980	43.978160	185.883200	.000448
-45.022840	43.720610	185.882800	.000154
-43.755960	43.450020	185.882700	.000118
-42.505550	43.166850	185.882700	.000092
-41.271660	42.871580	185.882700	.000092
-93.329110	43.967430	180.195300	2.610702
-92.068780	44.264030	175.845700	3.847880
-90.791210	44.547890	163.843100	6.124701
-89.496510	44.818510	139.643300	9.575863
-88.184940	45.075390	99.475270	14.171320
-86.856880	45.318050	49.118290	19.564930
-85.512890	45.545990	27.862690	15.983590
-84.153590	45.758730	35.118400	12.564180
-82.779790	45.955820	39.949340	10.680250
-81.392350	46.136820	34.386940	14.400360
-79.992260	46.301310	39.510620	15.684360
-78.580580	46.448910	60.527950	17.911460
-77.158460	46.579270	61.259050	22.562740
-75.727110	46.692070	71.434440	23.570960
-74.287820	46.787040	85.038820	25.973410
-72.841890	46.863950	114.851600	21.193180

-71.390720 46.922590 141.840200 14.002270
-69.935700 46.962830 160.351000 8.795301
-68.478260 46.984570 174.215700 5.186851
-67.019850 46.987740 180.291400 3.022986
-65.561920 46.972340 183.315500 1.657994
-64.105920 46.938410 185.530500 .680640
-62.653260 46.886040 186.714600 .393241
-61.205390 46.815350 186.794000 .348246
-59.763680 46.726520 186.315600 .182148
-58.329470 46.619790 185.962900 .158284
-56.904060 46.495390 185.978700 .124624
-55.488670 46.353640 186.045200 .108713
-54.084500 46.194880 186.064000 .089400
-52.692630 46.019470 185.991400 .053896
-51.314070 45.827840 185.935600 .028316
-49.949730 45.620390 185.904900 .012908
-48.600430 45.397590 185.890300 .005127
-47.266860 45.159900 185.884900 .001808
-45.949600 44.907820 185.883200 .000621
-44.649100 44.641840 185.882800 .000188
-43.365670 44.362450 185.882700 .000126
-42.099450 44.070140 185.882700 .000092
-40.850440 43.765410 185.882700 .000092
-93.760410 44.864160 181.721700 2.043260
-92.483920 45.170280 178.629500 3.043297
-91.189420 45.463320 170.269000 5.304014
-89.876960 45.742750 152.401200 8.945345
-88.546760 46.008070 118.625500 17.084600
-87.199170 46.258760 77.220160 25.007870
-85.834720 46.494300 50.072630 29.867870
-84.454070 46.714200 58.802510 21.740530
-83.058010 46.917960 60.350140 21.730390
-81.647480 47.105130 54.049130 24.233100
-80.223460 47.275260 59.442440 28.574540
-78.787090 47.427960 75.659380 27.748030
-77.339580 47.562840 95.548190 27.204520
-75.882230 47.679570 111.369800 26.064910
-74.416370 47.777870 132.602000 20.766630
-72.943440 47.857470 149.391100 13.141910
-71.464880 47.918190 158.677900 8.595814
-69.982220 47.959850 167.732100 6.296120
-68.496960 47.982350 173.880700 4.697623
-67.010670 47.985630 178.386600 3.597864
-65.524890 47.969690 181.547700 2.410847
-64.041170 47.934570 183.757700 1.422309
-62.561040 47.880340 185.260200 .830017
-61.086020 47.807170 185.610000 .619905
-59.617590 47.715230 185.638500 .511841
-58.157170 47.604770 185.748900 .339526
-56.706150 47.476050 185.991200 .164083
-55.265830 47.329400 186.165100 .130339
-53.837470 47.165180 186.141400 .112047
-52.422200 46.983780 186.012000 .065101
-51.021090 46.785640 185.943200 .034585
-49.635090 46.571200 185.908500 .017312
-48.265020 46.340950 185.891000 .006662
-46.911610 46.095370 185.884300 .002307
-45.575410 45.834990 185.882800 .000739
-44.256860 45.560320 185.882500 .000243
-42.956210 45.271870 185.882500 .000090
-41.673580 44.970160 185.882600 .000054
-40.408870 44.655690 185.882700 .000090
-94.212860 45.757310 183.142000 1.441093
-92.919560 46.073230 181.254200 2.167304
-91.607470 46.375730 176.363300 3.694885
-90.276550 46.664250 166.229500 6.620533
-88.926960 46.938270 147.065100 13.049370

-87.559010	47.197250	118.071500	24.571750
-86.173240	47.440650	97.685490	30.757540
-84.770290	47.667940	93.508510	29.911020
-83.350960	47.878600	93.635700	29.498350
-81.916200	48.072160	97.237850	26.435180
-80.467090	48.248140	100.940300	29.267200
-79.004780	48.406110	117.162900	27.844150
-77.530590	48.545680	133.392300	21.730860
-76.045850	48.666500	149.292400	16.842830
-74.552020	48.768250	161.617100	11.433350
-73.050600	48.850660	169.517400	7.141119
-71.543170	48.913520	173.074000	4.792025
-70.031330	48.956650	175.237800	3.742684
-68.516710	48.979950	177.944400	3.350971
-67.000980	48.983350	179.833900	2.852769
-65.485790	48.966840	181.278000	2.416139
-63.972820	48.930470	182.655900	1.902517
-62.463700	48.874330	183.919400	1.401903
-60.960050	48.798580	184.559400	1.164889
-59.463460	48.703410	184.914100	.955235
-57.975440	48.589080	185.495900	.578488
-56.497490	48.455880	186.050800	.212560
-55.030970	48.304140	186.212300	.134920
-53.577210	48.134270	186.116900	.094472
-52.137430	47.946660	185.957100	.053486
-50.712720	47.741790	185.870700	.041810
-49.304080	47.520120	185.875200	.016567
-47.912350	47.282170	185.879700	.005794
-46.538250	47.028450	185.881200	.002214
-45.182340	46.759510	185.881800	.000872
-43.845020	46.475880	185.882200	.000336
-42.526500	46.178100	185.882500	.000140
-41.226800	45.866700	185.882500	.000058
-39.945740	45.542200	185.882600	.000058
-94.687740	46.646660	184.244600	.935324
-93.376940	46.972690	183.228600	1.348277
-92.046550	47.284930	180.866700	2.231764
-90.696440	47.582840	175.510600	4.108931
-89.326680	47.865840	165.738000	7.902836
-87.937550	48.133390	152.021800	15.005740
-86.529520	48.384910	137.321200	22.530400
-85.103260	48.619850	130.662700	25.825950
-83.659580	48.837660	130.107600	23.565380
-82.199460	49.037830	136.070400	21.085140
-80.724000	49.219880	145.215500	18.481040
-79.234450	49.383340	153.078400	15.613580
-77.732160	49.527770	161.614000	12.230090
-76.218590	49.652830	169.327300	8.639383
-74.695260	49.758160	174.729600	5.949027
-73.163800	49.843490	177.878300	4.044736
-71.625870	49.908580	179.451900	2.927472
-70.083210	49.953250	180.625300	2.362800
-68.537570	49.977380	181.937800	1.865414
-66.990740	49.980900	182.772900	1.660715
-65.444490	49.963800	182.828500	1.683194
-63.900620	49.926140	183.117700	1.595498
-62.360880	49.868010	183.856400	1.277626
-60.827010	49.789570	184.530700	1.040296
-59.300730	49.691040	185.045800	.832219
-57.783640	49.572690	185.623700	.505288
-56.277320	49.434830	186.042500	.215564
-54.783270	49.277820	186.219400	.145339
-53.302860	49.102080	186.130800	.098388
-51.837360	48.908040	185.931900	.038882
-50.387950	48.696200	185.837000	.048000
-48.955640	48.467040	185.855700	.022094
-47.541310	48.221130	185.872000	.007345

-46.145660	47.959000	185.878200	.002590
-44.769230	47.681210	185.880900	.001041
-43.412380	47.388330	185.881900	.000394
-42.075270	47.080920	185.882300	.000158
-40.757810	46.759540	185.882500	.000075
-39.459720	46.424710	185.882600	.000056
-95.186440	47.531940	185.001300	.561016
-93.857410	47.868410	184.589400	.730434
-92.508000	48.190720	183.661500	1.081035
-91.137910	48.498310	181.274600	2.048505
-89.747150	48.790600	176.635300	4.078544
-88.335950	49.067010	170.029400	7.199658
-86.904710	49.326930	163.611100	11.164310
-85.454100	49.569790	159.046900	13.277490
-83.984930	49.795020	160.113800	12.643360
-82.498220	50.002060	164.688100	10.368290
-80.995120	50.190400	169.085400	8.375005
-79.476920	50.359550	172.334600	7.207731
-77.945060	50.509060	175.972600	5.465628
-76.401090	50.638520	178.966000	3.932677
-74.846650	50.747580	180.783100	2.892554
-73.283450	50.835950	181.711600	2.269677
-71.713320	50.903350	182.179100	1.875268
-70.138080	50.949630	182.732800	1.592097
-68.559640	50.974620	184.092500	1.158613
-66.979900	50.978270	184.974600	.817473
-65.400800	50.960560	184.672700	.839369
-63.824260	50.921550	184.355200	.892930
-62.252170	50.861340	184.731800	.728105
-60.686400	50.780110	184.990100	.612448
-59.128770	50.678080	185.406000	.465218
-57.581030	50.555550	185.727100	.296330
-56.044860	50.412850	186.074700	.133544
-54.521820	50.250360	186.149800	.119951
-53.013420	50.068520	186.094700	.096060
-51.520960	49.867810	185.971600	.038672
-50.045680	49.648740	185.891800	.010129
-48.588630	49.411830	185.861400	.015922
-47.150700	49.157670	185.874100	.005476
-45.732590	48.886830	185.879000	.002005
-44.334810	48.599900	185.881000	.000873
-42.957660	48.297460	185.882000	.000360
-41.601190	47.980120	185.882300	.000154
-40.265250	47.648430	185.882500	.000071
-38.949390	47.302930	185.882600	.000055
-95.710510	48.412890	185.525900	.305561
-94.362470	48.760130	185.496800	.331304
-92.993230	49.092850	185.307600	.389270
-91.602360	49.410460	184.327500	.802996
-90.189750	49.712350	182.304400	1.683473
-88.755540	49.997940	179.769600	2.965869
-87.300090	50.266570	177.055600	4.274562
-85.824040	50.517650	175.785100	5.286233
-84.328210	50.750570	176.410600	5.046675
-82.813610	50.964740	177.813900	4.316786
-81.281460	51.159630	179.620200	3.527080
-79.733130	51.334700	181.002400	2.822259
-78.170130	51.489480	182.474100	2.084687
-76.594090	51.623530	183.309900	1.576057
-75.006800	51.736490	183.662700	1.337869
-73.410070	51.828010	183.769200	1.222750
-71.805870	51.897850	183.669000	1.195416
-70.196170	51.945790	183.774800	1.203097
-68.582990	51.971680	184.596100	.947618
-66.968440	51.975460	185.723800	.586627
-65.354560	51.957120	185.702800	.395928
-63.743440	51.916690	185.437600	.394197

-62.137130	51.854320	185.273900	.396009
-60.537630	51.770170	185.417400	.348181
-58.946900	51.664510	185.607600	.262723
-57.366820	51.537620	185.877800	.125639
-55.799180	51.389870	185.973900	.064165
-54.245650	51.221680	186.006500	.059919
-52.707820	51.033510	186.009600	.061045
-51.187080	50.825860	185.968900	.042399
-49.684720	50.599280	185.900500	.011718
-48.201800	50.354330	185.887000	.002875
-46.739240	50.091630	185.881000	.001613
-45.297720	49.811770	185.881100	.000962
-43.877700	49.515370	185.881700	.000498
-42.479410	49.203060	185.882100	.000242
-41.102810	48.875440	185.882400	.000118
-39.747590	48.533100	185.882500	.000055
-38.413150	48.176570	185.882600	.000061
-96.261670	49.289190	185.936500	.203137
-94.893750	49.647600	186.033800	.260059
-93.503820	49.991080	186.048500	.236580
-92.091320	50.319060	185.591200	.272499
-90.655950	50.630910	184.764200	.630412
-89.197760	50.925990	183.731500	1.104735
-87.717060	51.203660	182.863200	1.593841
-86.214420	51.463270	182.472300	1.895582
-84.690660	51.704190	182.575400	1.933669
-83.146820	51.925780	183.067900	1.757737
-81.584160	52.127480	183.818700	1.348783
-80.004110	52.308710	184.647500	.958789
-78.408280	52.468990	185.104700	.649453
-76.798410	52.607840	185.182300	.559103
-75.176390	52.724850	185.047400	.667068
-73.544200	52.819690	184.764200	.768570
-71.903920	52.892050	184.593900	.793794
-70.257710	52.941730	184.347500	.935289
-68.607750	52.968570	184.849900	.858090
-66.956280	52.972490	185.740900	.550721
-65.305560	52.953480	185.998900	.309722
-63.657820	52.911580	185.780900	.247797
-62.015270	52.846950	185.693500	.257192
-60.380080	52.759750	185.633500	.233671
-58.754360	52.650280	185.791400	.124622
-57.140120	52.518850	185.877600	.056064
-55.539290	52.365840	185.918700	.031568
-53.953660	52.191710	185.932200	.025459
-52.384890	51.996940	185.932100	.025155
-50.834480	51.782070	185.921700	.020514
-49.303730	51.547700	185.901700	.009484
-47.793770	51.294400	185.887700	.003530
-46.305490	51.022820	185.883300	.000831
-44.839570	50.733620	185.882200	.000292
-43.396390	50.427420	185.882200	.000207
-41.976090	50.104880	185.882400	.000123
-40.578500	49.766630	185.882500	.000080
-39.203150	49.413260	185.882600	.000054
-37.849210	49.045330	185.882700	.000090
-96.841810	50.160530	186.061700	.169059
-95.453030	50.530490	186.149900	.212306
-94.041510	50.885130	186.143400	.179792
-92.606450	51.223850	185.886200	.096883
-91.147380	51.546020	185.550700	.226375
-89.664200	51.850960	185.193800	.410232
-88.157130	52.138020	184.906700	.564744
-86.626690	52.406500	184.768000	.675087
-85.073680	52.655720	184.765400	.738688
-83.499180	52.885050	184.969800	.670095
-81.904440	53.093850	185.463600	.467783

-80.290990	53.281520	185.939100	.254245
-78.660530	53.447530	186.163400	.214312
-77.014920	53.591380	186.009200	.280975
-75.356160	53.712650	185.571900	.442751
-73.686420	53.810940	185.019500	.620667
-72.007930	53.885960	184.739100	.670748
-70.322990	53.937460	184.530000	.779330
-68.634010	53.965280	184.675700	.814806
-66.943400	53.969350	185.363800	.568098
-65.253590	53.949630	185.649600	.330429
-63.567000	53.906200	185.728000	.250256
-61.886040	53.839190	185.711900	.218849
-60.213040	53.748820	185.790500	.126102
-58.550290	53.635360	185.843900	.057339
-56.899960	53.499180	185.878300	.028676
-55.264110	53.340690	185.895200	.015011
-53.644650	53.160350	185.901600	.011552
-52.043340	52.958710	185.901600	.010051
-50.461760	52.736320	185.897400	.007965
-48.901280	52.493820	185.890600	.004686
-47.363030	52.231850	185.885700	.001803
-45.847900	51.951060	185.883300	.000558
-44.356530	51.652150	185.882600	.000116
-42.889220	51.335790	185.882500	.000081
-41.445980	51.002650	185.882500	.000072
-40.026490	50.653380	185.882600	.000074
-38.630060	50.288590	185.882700	.000087
-37.255620	49.908830	185.882700	.000094
-97.453060	51.026530	186.005100	.088212
-96.042340	51.408490	186.051800	.106706
-94.608220	51.774700	186.004300	.067673
-93.149600	52.124570	185.910300	.031387
-91.665800	52.457450	185.797100	.077714
-90.156560	52.772640	185.681300	.139215
-88.621980	53.069450	185.591100	.192987
-87.062480	53.347160	185.546900	.230077
-85.478850	53.605050	185.540000	.255120
-83.872130	53.842430	185.601800	.252846
-82.243660	54.058630	185.838000	.148701
-80.595020	54.253030	186.151100	.115357
-78.928000	54.425040	186.418600	.259861
-77.244600	54.574130	186.319600	.335977
-75.546970	54.699840	185.797900	.360255
-73.837420	54.801750	185.173300	.508310
-72.118360	54.879540	184.759400	.621216
-70.392330	54.932960	184.665300	.657741
-68.661900	54.961820	184.638100	.715581
-66.929700	54.966030	185.061500	.550131
-65.198370	54.945580	185.363800	.365602
-63.470540	54.900540	185.558400	.257155
-61.748820	54.831050	185.729700	.140483
-60.035740	54.737340	185.823800	.063094
-58.333780	54.619720	185.862800	.030746
-56.645260	54.478570	185.877500	.014371
-54.972420	54.314330	185.885900	.007091
-53.317280	54.127510	185.889200	.004787
-51.681740	53.918690	185.889500	.004099
-50.067420	53.688470	185.888000	.003156
-48.475770	53.437500	185.885800	.001909
-46.907930	53.166490	185.883900	.000911
-45.364770	52.876130	185.883000	.000320
-43.846840	52.567130	185.882700	.000117
-42.354370	52.240220	185.882700	.000087
-40.887210	51.896080	185.882600	.000062
-39.444820	51.535370	185.882700	.000092
-38.026250	51.158730	185.882700	.000094
-36.630120	50.766700	185.882700	.000092

-98.097820	51.886770	185.944000	.035680
-96.663910	52.281200	185.949300	.037538
-95.206040	52.659450	185.934600	.023632
-93.722790	53.020900	185.905100	.012089
-92.213200	53.364910	185.868900	.023137
-90.676770	53.690770	185.832600	.043431
-89.113440	53.997730	185.805600	.061241
-87.523560	54.285050	185.793700	.073225
-85.907830	54.551970	185.794800	.083225
-84.267300	54.797760	185.801800	.093892
-82.603340	55.021710	185.866100	.080755
-80.917590	55.223140	186.075600	.099556
-79.211960	55.401440	186.396100	.273579
-77.488560	55.556020	186.440200	.388810
-75.749730	55.686380	185.952800	.329973
-73.997920	55.792100	185.301400	.422882
-72.235800	55.872810	184.788700	.595647
-70.466090	55.928230	184.669300	.613232
-68.691570	55.958180	184.754400	.612810
-66.915130	55.962550	185.038300	.483234
-65.139640	55.941330	185.372900	.314219
-63.367970	55.894590	185.622200	.167599
-61.602940	55.822490	185.767700	.076639
-59.847310	55.725280	185.834700	.036326
-58.103760	55.603290	185.863900	.017285
-56.374820	55.456930	185.876700	.007860
-54.662870	55.286670	185.882200	.003358
-52.970100	55.093080	185.884400	.001813
-51.298500	54.876750	185.884800	.001479
-49.649800	54.638340	185.884400	.001182
-48.025460	54.378550	185.883700	.000765
-46.426660	54.098120	185.883100	.000387
-44.854210	53.797780	185.882800	.000155
-43.308580	53.478290	185.882700	.000117
-41.789850	53.140400	185.882700	.000097
-40.297680	52.784830	185.882700	.000097
-38.831280	52.412250	185.882700	.000097
-37.389370	52.023280	185.882700	.000092
-35.970190	51.618480	185.882700	.000092
-98.778840	52.740810	185.910600	.015965
-97.320270	53.148240	185.909700	.014427
-95.837370	53.539010	185.904600	.010979
-94.328290	53.912530	185.895500	.006130
-92.791720	54.268120	185.884100	.005999
-91.226900	54.605080	185.873200	.011929
-89.633560	54.922620	185.866000	.017681
-88.011890	55.219970	185.864500	.022131
-86.362530	55.496330	185.868000	.026673
-84.686490	55.750910	185.872100	.035541
-82.985150	55.982970	185.880700	.050560
-81.260250	56.191770	186.010000	.092687
-79.513790	56.376650	186.321400	.262513
-77.748020	56.536990	186.483400	.407747
-75.965460	56.672260	186.039800	.308446
-74.168790	56.781970	185.356700	.354893
-72.360860	56.865740	184.739300	.593846
-70.544630	56.923280	184.577300	.646533
-68.723180	56.954370	184.772900	.570913
-66.899620	56.958910	185.150000	.400641
-65.077100	56.936880	185.502600	.213701
-63.258730	56.888360	185.697100	.100158
-61.447630	56.813510	185.801100	.044972
-59.646790	56.712620	185.845600	.022575
-57.859090	56.586040	185.867400	.010591
-56.087300	56.434210	185.877000	.004560
-54.333970	56.257640	185.881100	.001770
-52.601470	56.056940	185.882700	.000676

-50.891880	55.832740	185.883100	.000462
-49.207030	55.585770	185.883100	.000383
-47.548420	55.316760	185.882900	.000262
-45.917200	55.026500	185.882800	.000136
-44.314130	54.715760	185.882700	.000119
-42.739580	54.385350	185.882700	.000096
-41.193420	54.036030	185.882700	.000097
-39.675070	53.668560	185.882700	.000097
-38.183400	53.283620	185.882700	.000092
-36.716770	52.881820	185.882700	.000092
-35.272900	52.463700	185.882700	.000092
-99.499240	53.588130	185.894300	.006998
-98.014280	54.009130	185.893100	.006188
-96.504860	54.412960	185.891200	.004827
-94.968610	54.799050	185.888300	.002977
-93.403790	55.166730	185.884900	.001616
-91.809290	55.515250	185.882000	.002834
-90.184560	55.843850	185.880700	.004762
-88.529620	56.151670	185.882000	.007060
-86.844990	56.437890	185.886300	.010706
-85.131650	56.701690	185.891600	.015795
-83.390960	56.942230	185.906400	.028978
-81.624720	57.158780	185.981600	.088690
-79.835050	57.350580	186.258000	.241811
-78.024370	57.516980	186.446300	.395606
-76.195360	57.657400	186.053500	.269296
-74.350940	57.771310	185.341400	.299211
-72.494220	57.858320	184.695400	.597641
-70.628410	57.918090	184.490000	.683053
-68.756890	57.950390	184.780100	.549757
-66.883060	57.955100	185.277500	.309735
-65.010370	57.932220	185.589500	.149408
-63.142240	57.881810	185.741900	.072189
-61.282040	57.804080	185.805400	.038989
-59.433060	57.699310	185.851800	.016790
-57.598450	57.567890	185.869700	.007348
-55.781200	57.410300	185.877600	.003175
-53.984060	57.227100	185.880900	.001206
-52.209560	57.018940	185.882100	.000399
-50.459930	56.786500	185.882600	.000135
-48.737060	56.530560	185.882700	.000135
-47.042490	56.251900	185.882700	.000096
-45.377330	55.951360	185.882700	.000122
-43.742270	55.629770	185.882700	.000099
-42.137470	55.287960	185.882700	.000097
-40.562610	54.926740	185.882700	.000097
-39.016750	54.546860	185.882700	.000094
-37.498410	54.149020	185.882700	.000092
-36.005400	53.733830	185.882700	.000092
-34.534880	53.301770	185.882700	.000092
-100.262700	54.428120	185.887000	.002890
-98.749250	54.863350	185.886300	.002480
-97.211560	55.280850	185.885600	.001970
-95.646590	55.680060	185.884700	.001327
-94.052090	56.060360	185.883800	.000731
-92.426510	56.420970	185.883200	.000885
-90.768940	56.761100	185.883700	.001899
-89.079190	57.079890	185.885800	.003685
-87.357570	57.376450	185.890100	.006406
-85.605000	57.649890	185.896100	.012006
-83.822840	57.899360	185.914500	.023644
-82.012920	58.124030	185.996500	.079201
-80.177480	58.323120	186.185500	.219505
-78.319110	58.495910	186.384300	.359279
-76.440710	58.641750	185.963300	.187551
-74.545430	58.760110	185.316400	.278054
-72.636660	58.850530	184.730600	.573605

-70.717930 58.912650 184.485400 .691016
-68.792920 58.946230 184.787800 .534786
-66.865380 58.951130 185.339500 .269898
-64.939080 58.927340 185.643900 .120149
-63.017780 58.874950 185.758500 .062913
-61.105210 58.794160 185.818200 .033503
-59.204930 58.685300 185.847900 .018075
-57.320400 58.548780 185.869500 .007543
-55.454840 58.385130 185.877800 .002776
-53.611270 58.194950 185.880900 .001055
-51.792370 57.978940 185.882000 .000377
-50.000500 57.737840 185.882400 .000122
-48.237630 57.472490 185.882500 .000045
-46.505310 57.183730 185.882500 .000037
-44.804610 56.872430 185.882600 .000046
-43.136050 56.539500 185.882600 .000060
-41.499610 56.185780 185.882600 .000056
-39.894630 55.812120 185.882700 .000090
-38.319800 55.419290 185.882700 .000095
-36.773090 55.007980 185.882700 .000092
-35.251730 54.578750 185.882700 .000092
-33.752120 54.132060 185.882700 .000092
-101.073700 55.260130 185.884000 .001099
-99.529010 55.710320 185.883700 .000918
-97.960900 56.142140 185.883500 .000728
-96.365430 56.555100 185.883200 .000527
-94.739640 56.948590 185.883100 .000404
-93.081440 57.321860 185.883300 .000634
-91.389490 57.674070 185.884300 .001446
-89.663250 58.004340 185.886400 .002827
-87.902820 58.311730 185.890300 .004786
-86.109000 58.595320 185.895200 .009769
-84.283100 58.854170 185.913100 .020831
-82.427000 59.087400 185.990400 .073884
-80.543050 59.294160 186.156200 .194765
-78.634010 59.473690 186.266400 .293588
-76.703000 59.625280 185.855300 .090689
-74.753460 59.748340 185.313200 .273396
-72.789070 59.842370 184.897800 .499786
-70.813740 59.906970 184.594300 .645115
-68.831480 59.941910 184.781500 .545449
-66.846440 59.947010 185.331400 .277922
-64.862770 59.922250 185.660500 .113190
-62.884610 59.867760 185.784300 .051450
-60.916040 59.783740 185.829300 .027336
-58.961010 59.670550 185.854300 .015596
-57.023270 59.528640 185.869100 .007621
-55.106350 59.358580 185.877900 .002819
-53.213500 59.161040 185.881000 .000928
-51.347600 58.936760 185.882000 .000330
-49.511150 58.686560 185.882400 .000127
-47.706190 58.411320 185.882500 .000062
-45.934240 58.111950 185.882500 .000035
-44.196280 57.789400 185.882500 .000034
-42.492650 57.444580 185.882600 .000046
-40.823030 57.078400 185.882600 .000058
-39.186360 56.691730 185.882600 .000055
-37.580830 56.285340 185.882700 .000087
-36.003750 55.859910 185.882700 .000092
-34.451600 55.415950 185.882700 .000092
-32.919830 54.953830 185.882700 .000092
-101.937200 56.083360 185.883000 .000385
-100.358100 56.549350 185.882900 .000298
-98.756940 56.996240 185.882900 .000249
-97.128790 57.423640 185.882800 .000197
-95.469860 57.830970 185.882900 .000238
-93.777320 58.217490 185.883300 .000523

-92.049300	58.582370	185.884200	.001232
-90.284800	58.924680	185.886100	.002298
-88.483640	59.243460	185.889200	.003883
-86.646400	59.537720	185.892000	.008487
-84.774360	59.806450	185.905500	.016144
-82.869400	60.048700	185.968800	.059165
-80.933970	60.263580	186.097900	.155543
-78.971000	60.450230	186.119800	.186179
-76.983890	60.607900	185.742000	.075285
-74.976370	60.735940	185.377600	.255287
-72.952470	60.833800	185.122300	.387029
-70.916480	60.901050	184.783800	.567675
-68.872850	60.937420	184.764900	.565850
-66.826130	60.942730	185.288200	.304720
-64.780930	60.916960	185.662400	.115369
-62.741830	60.860230	185.804300	.044188
-60.713310	60.772790	185.853600	.017550
-58.699720	60.654990	185.868500	.007837
-56.705200	60.507380	185.876000	.003966
-54.733590	60.330550	185.880000	.001589
-52.788400	60.125220	185.881700	.000562
-50.872730	59.892220	185.882200	.000211
-48.989200	59.632420	185.882400	.000099
-47.139900	59.346780	185.882500	.000054
-45.326320	59.036270	185.882500	.000039
-43.549270	58.701890	185.882500	.000039
-41.808860	58.344600	185.882600	.000046
-40.104370	57.965370	185.882600	.000057
-38.434220	57.565060	185.882600	.000050
-36.795950	57.144450	185.882700	.000085
-35.186060	56.704150	185.882700	.000092
-33.600030	56.244660	185.882700	.000092
-32.032190	55.766200	185.882700	.000092
-102.859900	56.896880	185.882800	.000133
-101.242000	57.379640	185.882800	.000133
-99.604390	57.842470	185.882700	.000126
-97.940930	58.285070	185.882700	.000116
-96.246660	58.706940	185.882800	.000164
-94.517820	59.107400	185.883200	.000428
-92.751850	59.485580	185.884000	.001009
-90.947170	59.840560	185.885500	.001838
-89.103220	60.171320	185.887600	.003231
-87.220290	60.476800	185.887900	.007788
-85.299550	60.755960	185.894700	.010237
-83.342830	61.007760	185.932900	.035684
-81.352710	61.231220	185.989600	.080697
-79.332320	61.425420	185.949500	.066286
-77.285290	61.589540	185.656900	.121817
-75.215710	61.722870	185.437600	.230800
-73.128000	61.824790	185.312100	.299603
-71.026890	61.894870	184.944400	.494828
-68.917300	61.932760	184.759900	.574371
-66.804310	61.938290	185.249900	.331072
-64.692990	61.911450	185.661800	.120292
-62.588420	61.852340	185.819500	.040814
-60.495590	61.761240	185.871900	.015123
-58.419300	61.638580	185.883100	.005902
-56.364090	61.484900	185.883100	.001717
-54.334160	61.300890	185.882400	.000439
-52.333320	61.087320	185.882400	.000180
-50.364880	60.845090	185.882500	.000092
-48.431600	60.575160	185.882500	.000057
-46.535590	60.278550	185.882500	.000043
-44.678250	59.956310	185.882600	.000057
-42.860180	59.609490	185.882600	.000062
-41.081100	59.239120	185.882700	.000085
-39.339830	58.846180	185.882700	.000095

-37.634110	58.431540	185.882700	.000102
-35.960610	57.995940	185.882700	.000092
-34.314830	57.539980	185.882700	.000092
-32.690990	57.064010	185.882700	.000092
-31.082000	56.568130	185.882700	.000092
-103.849600	57.699570	185.882700	.000113
-102.187300	58.200280	185.882700	.000120
-100.509000	58.680030	185.882700	.000091
-98.806840	59.138710	185.882700	.000115
-97.074520	59.575940	185.882800	.000127
-95.307120	59.991050	185.883000	.000339
-93.501110	60.383240	185.883700	.000785
-91.654170	60.751550	185.884800	.001413
-89.765210	61.094930	185.886000	.002651
-87.834170	61.412270	185.884700	.006551
-85.861950	61.702450	185.884600	.006402
-83.850420	61.964360	185.897400	.013376
-81.802150	62.196920	185.887100	.012575
-79.720490	62.399150	185.829200	.030776
-77.609360	62.570130	185.619200	.155619
-75.473210	62.709080	185.509600	.210244
-73.316960	62.815350	185.402300	.266550
-71.145790	62.888430	185.074200	.422727
-68.965200	62.927950	184.887800	.516897
-66.780790	62.933720	185.274200	.316400
-64.598260	62.905720	185.674300	.116129
-62.423240	62.844070	185.827100	.037078
-60.261290	62.749080	185.873400	.013136
-58.117710	62.621230	185.882600	.004636
-55.997520	62.461110	185.883100	.001450
-53.905320	62.269450	185.882900	.000420
-51.845240	62.047150	185.882700	.000135
-49.820820	61.795150	185.882700	.000109
-47.834930	61.514500	185.882700	.000085
-45.889670	61.206310	185.882700	.000090
-43.986290	60.871700	185.882700	.000097
-42.125070	60.511780	185.882700	.000094
-40.305270	60.127630	185.882700	.000094
-38.525020	59.720250	185.882700	.000094
-36.781200	59.290500	185.882700	.000092
-35.069400	58.839090	185.882700	.000092
-33.383770	58.366500	185.882700	.000092
-31.716990	57.872960	185.882700	.000092
-30.060130	57.358350	185.882700	.000092
-104.916500	58.490070	185.882700	.000092
-103.202300	59.010120	185.882700	.000092
-101.477600	59.507980	185.882700	.000092
-99.732440	59.983750	185.882700	.000120
-97.958720	60.437220	185.882800	.000107
-96.150010	60.867820	185.882900	.000243
-94.301540	61.274790	185.883400	.000567
-92.410060	61.657170	185.884100	.001016
-90.473700	62.013880	185.884700	.001909
-88.491920	62.343760	185.883100	.004896
-86.465330	62.645610	185.881200	.004789
-84.395630	62.918240	185.885400	.006779
-82.285480	63.160470	185.870400	.007101
-80.138370	63.371240	185.825100	.033445
-77.958560	63.549540	185.732600	.093341
-75.750930	63.694510	185.655000	.136400
-73.520860	63.805420	185.559100	.183235
-71.274150	63.881700	185.374300	.278385
-69.016910	63.922960	185.203900	.350185
-66.755400	63.928990	185.479500	.215130
-64.495980	63.899750	185.731500	.083012
-62.244980	63.835380	185.835800	.028671
-60.008560	63.736250	185.869800	.009666

-57.792640 63.602840 185.880100 .003135
-55.602750 63.435840 185.882500 .000985
-53.443960 63.236060 185.882800 .000335
-51.320750 63.004450 185.882700 .000120
-49.236890 62.742080 185.882700 .000111
-47.195330 62.450080 185.882700 .000097
-45.198120 62.129630 185.882700 .000094
-43.246250 61.781950 185.882700 .000094
-41.339580 61.408210 185.882700 .000094
-39.476710 61.009530 185.882700 .000094
-37.654880 60.586900 185.882700 .000092
-35.869840 60.141170 185.882700 .000092
-34.115770 59.672970 185.882700 .000092
-32.385130 59.182660 185.882700 .000092
-30.668580 58.670220 185.882700 .000092
-28.954870 58.135290 185.882700 .000092
-106.073800 59.266680 185.882700 .000092
-104.297500 59.807790 185.882700 .000092
-102.518800 60.325180 185.882700 .000092
-100.725000 60.819250 185.882700 .000091
-98.905530 61.289990 185.882700 .000105
-97.052100 61.737010 185.882800 .000167
-95.158340 62.159620 185.883200 .000396
-93.219700 62.556880 185.883600 .000673
-91.233350 62.927700 185.883900 .001136
-89.198040 63.270870 185.882900 .002886
-87.113920 63.585110 185.881100 .002911
-84.982480 63.869130 185.882400 .003093
-82.806390 64.121680 185.874200 .004485
-80.589290 64.341560 185.856000 .015969
-78.335770 64.527670 185.824500 .035764
-76.051160 64.679080 185.786900 .058070
-73.741460 64.794950 185.736300 .085388
-71.413090 64.874680 185.657700 .125218
-69.072890 64.917820 185.585400 .158887
-66.727910 64.924110 185.704700 .097360
-64.385280 64.893550 185.805300 .043071
-62.052090 64.826280 185.853600 .017553
-59.735260 64.722690 185.873300 .006491
-57.441380 64.583340 185.880300 .002122
-55.176610 64.408980 185.882400 .000656
-52.946510 64.200520 185.882700 .000209
-50.755950 63.959000 185.882800 .000101
-48.608900 63.685590 185.882700 .000120
-46.508400 63.381530 185.882700 .000094
-44.456330 63.048090 185.882700 .000092
-42.453320 62.686580 185.882700 .000092
-40.498620 62.298210 185.882700 .000092
-38.589950 61.884150 185.882700 .000092
-36.723370 61.445380 185.882700 .000092
-34.893150 60.982680 185.882700 .000092
-33.091610 60.496560 185.882700 .000092
-31.309040 59.987160 185.882700 .000092
-29.533510 59.454220 185.882700 .000092
-27.750760 58.896990 185.882700 .000092
-107.339200 60.027230 185.882700 .000092
-105.487000 60.591610 185.882700 .000092
-103.643800 61.130270 185.882700 .000092
-101.793500 61.644070 185.882700 .000091
-99.922550 62.133320 185.882700 .000116
-98.020040 62.597820 185.882800 .000119
-96.077510 63.037030 185.883000 .000263
-94.088710 63.450070 185.883300 .000465
-92.049460 63.835860 185.883500 .000639
-89.957580 64.193130 185.883100 .001137
-87.812570 64.520550 185.881800 .001454
-85.615560 64.816710 185.881900 .001378

-83.369120	65.080260	185.877800	.002473
-81.077050	65.309890	185.870900	.007212
-78.744270	65.504390	185.859900	.014423
-76.376630	65.662700	185.844900	.023237
-73.980770	65.783920	185.825100	.034166
-71.563890	65.867360	185.800400	.046648
-69.133670	65.912500	185.785100	.054908
-66.698060	65.919100	185.815100	.037475
-64.265090	65.887100	185.847400	.020576
-61.842790	65.816700	185.867400	.009584
-59.438900	65.708320	185.877100	.003844
-57.060850	65.562580	185.881100	.001353
-54.715470	65.380330	185.882400	.000422
-52.408880	65.162570	185.882700	.000140
-50.146370	64.910470	185.882700	.000100
-47.932120	64.625290	185.882700	.000093
-45.769150	64.308400	185.882700	.000094
-43.659080	63.961160	185.882700	.000092
-41.602020	63.584970	185.882700	.000092
-39.596350	63.181100	185.882700	.000092
-37.638600	62.750720	185.882700	.000092
-35.723270	62.294770	185.882700	.000092
-33.842640	61.813940	185.882700	.000092
-31.986660	61.308540	185.882700	.000092
-30.142740	60.778430	185.882700	.000092
-28.295590	60.222970	185.882700	.000092
-26.427080	59.640870	185.882700	.000092
-108.736700	60.768890	185.882700	.000092
-106.789100	61.359350	185.882700	.000092
-104.866900	61.921510	185.882700	.000092
-102.949500	62.456830	185.882700	.000091
-101.019200	62.966030	185.882700	.000118
-99.061880	63.449270	185.882800	.000111
-97.066180	63.906190	185.882900	.000202
-95.023590	64.336020	185.883100	.000350
-92.928180	64.737730	185.883200	.000451
-90.776390	65.110020	185.883100	.000563
-88.566850	65.451470	185.882300	.000533
-86.300130	65.760610	185.881600	.000590
-83.978550	66.035940	185.879400	.001814
-81.606060	66.276030	185.876000	.004409
-79.187900	66.479550	185.870700	.008045
-76.730480	66.645290	185.863100	.012624
-74.241170	66.772270	185.853400	.018089
-71.728080	66.859700	185.843300	.023454
-69.199870	66.907030	185.839100	.025072
-66.665550	66.913940	185.849200	.019772
-64.134220	66.880400	185.862800	.012210
-61.614990	66.806620	185.873000	.006247
-59.116610	66.693070	185.878800	.002758
-56.647430	66.540450	185.881500	.001051
-54.215080	66.349720	185.882400	.000347
-51.826330	66.121990	185.882700	.000130
-49.486860	65.858530	185.882700	.000095
-47.201050	65.560780	185.882700	.000092
-44.971820	65.230190	185.882700	.000092
-42.800360	64.868270	185.882700	.000092
-40.685980	64.476460	185.882700	.000092
-38.625890	64.056100	185.882700	.000092
-36.614990	63.608330	185.882700	.000092
-34.645670	63.134020	185.882700	.000092
-32.707610	62.633670	185.882700	.000092
-30.787590	62.107330	185.882700	.000092
-28.869240	61.554470	185.882700	.000092
-26.932890	60.973880	185.882700	.000092
-24.955330	60.363580	185.882700	.000092

Q.05a

-89.453400	32.991660	195.802500	.029161
-88.345930	33.191160	195.799300	.033140
-87.228100	33.381660	195.785200	.050500
-86.100230	33.562890	195.760500	.080593
-84.962690	33.734530	195.724700	.122387
-83.815900	33.896310	195.678500	.172928
-82.660300	34.047950	195.627200	.227068
-81.496410	34.189190	195.579800	.279190
-80.324730	34.319790	195.540500	.326774
-79.145840	34.439500	195.502200	.373769
-77.960310	34.548100	195.446600	.430668
-76.768760	34.645400	195.368600	.497047
-75.571840	34.731200	195.302200	.544427
-74.370180	34.805370	195.304200	.531020
-73.164480	34.867740	195.395100	.447290
-71.955430	34.918200	195.529300	.322611
-70.743730	34.956650	195.651500	.201806
-69.530080	34.983020	195.735400	.111257
-68.315220	34.997260	195.782500	.054632
-67.099880	34.999340	195.805100	.023955
-65.884770	34.989250	195.814600	.009400
-64.670640	34.967020	195.818200	.003302
-63.458190	34.932690	195.819500	.001027
-62.248150	34.886320	195.819900	.000276
-61.041230	34.828000	195.820000	.000057
-59.838120	34.757850	195.820000	.000013
-58.639500	34.676010	195.820000	.000013
-57.446040	34.582610	195.820000	.000021
-56.258390	34.477850	195.820000	.000000
-55.077160	34.361910	195.820000	.000000
-53.902950	34.235010	195.820000	.000000
-52.736340	34.097380	195.820000	.000000
-51.577860	33.949260	195.820000	.000000
-50.428010	33.790910	195.820000	.000000
-49.287250	33.622600	195.820000	.000000
-48.156040	33.444620	195.820000	.000000
-47.034740	33.257250	195.820000	.000000
-45.923690	33.060790	195.820000	.000000
-44.823220	32.855560	195.820000	.000000
-89.701210	33.918400	195.774400	.059134
-88.583520	34.125020	195.762700	.070158
-87.455040	34.322360	195.721800	.107643
-86.316120	34.510110	195.652600	.170620
-85.167100	34.687960	195.553600	.256725

-84.008420	34.855620	195.426400	.360135
-82.840550	35.012790	195.285500	.471152
-81.663990	35.159200	195.155500	.578792
-80.479300	35.294580	195.051800	.676594
-79.287040	35.418700	194.958300	.773548
-78.087840	35.531310	194.820300	.901781
-76.882320	35.632210	194.605100	1.071860
-75.671150	35.721200	194.405100	1.206695
-74.455030	35.798110	194.405000	1.179608
-73.234660	35.862810	194.669900	.987761
-72.010770	35.915150	195.051800	.700251
-70.784100	35.955040	195.385300	.425451
-69.555380	35.982390	195.606800	.227504
-68.325390	35.997170	195.727600	.108774
-67.094890	35.999320	195.784100	.046742
-65.864640	35.988860	195.807400	.018006
-64.635410	35.965800	195.816000	.006187
-63.407950	35.930180	195.818900	.001836
-62.183030	35.882080	195.819700	.000446
-60.961370	35.821590	195.819900	.000086
-59.743720	35.748840	195.820000	.000050
-58.530780	35.663950	195.820100	.000050
-57.323260	35.567100	195.820000	.000041
-56.121820	35.458470	195.820000	.000013
-54.927120	35.338260	195.820000	.000000
-53.739770	35.206700	195.820000	.000000
-52.560380	35.064020	195.820000	.000000
-51.389480	34.910500	195.820000	.000000
-50.227610	34.746390	195.820000	.000000
-49.075240	34.571990	195.820000	.000000
-47.932830	34.387580	195.820000	.000000
-46.800740	34.193490	195.820000	.000000
-45.679330	33.990000	195.820000	.000000
-44.568900	33.777440	195.820000	.000000
-89.960660	34.843320	195.668500	.149951
-88.832330	35.057210	195.621100	.183063
-87.692770	35.261540	195.478200	.279601
-86.542320	35.455950	195.241500	.436719
-85.381340	35.640140	194.907900	.646990
-84.210270	35.813810	194.483900	.897579
-83.029580	35.976620	194.013500	1.170600
-81.839790	36.128310	193.576800	1.441571
-80.641480	36.268600	193.231000	1.689346
-79.435230	36.397220	192.945200	1.931399
-78.221690	36.513940	192.508200	2.300372
-77.001530	36.618520	191.730000	2.876416
-75.775430	36.710780	190.958700	3.326348
-74.544130	36.790520	190.918900	3.264907
-73.308360	36.857590	191.901600	2.713912
-72.068890	36.911860	193.264200	1.922484
-70.826490	36.953220	194.410800	1.142899
-69.581950	36.981590	195.143000	.592932
-68.336070	36.996900	195.531700	.276298
-67.089650	36.999130	195.709900	.116561
-65.843500	36.988290	195.782200	.044389
-64.598400	36.964370	195.808500	.015001
-63.355190	36.927440	195.817000	.004313
-62.114630	36.877570	195.819500	.000914
-60.877510	36.814860	195.820100	.000161
-59.644590	36.739430	195.820200	.000211
-58.416640	36.651430	195.820200	.000174
-57.194370	36.551040	195.820200	.000133
-55.978490	36.438440	195.820100	.000067
-54.769680	36.313860	195.819900	.000063
-53.568590	36.177530	195.820000	.000025
-52.375820	36.029710	195.820000	.000000
-51.191970	35.870660	195.820000	.000000

-50.017550	35.700660	195.819900	.000015
-48.853080	35.520030	195.820000	.000000
-47.698980	35.329070	195.820000	.000000
-46.555660	35.128100	195.820000	.000000
-45.423470	34.917440	195.820000	.000043
-44.302690	34.697410	195.820000	.000000
-90.232250	35.766320	195.369900	.344134
-89.092860	35.987640	195.207100	.428848
-87.941790	36.199090	194.761200	.651607
-86.779330	36.400320	194.037000	1.000740
-85.605870	36.591000	193.036500	1.451847
-84.421870	36.770790	191.771800	1.985274
-83.227800	36.939400	190.355700	2.595730
-82.024180	37.096500	189.019800	3.228445
-80.811620	37.241800	187.961000	3.795748
-79.590740	37.375040	187.193100	4.311492
-78.362190	37.495960	185.884600	5.324326
-77.126670	37.604320	183.175600	7.111691
-75.884930	37.699920	180.122100	8.542902
-74.637700	37.782550	179.879500	8.300233
-73.385770	37.852060	183.272600	6.861691
-72.129940	37.908310	187.909900	4.906708
-70.871030	37.951180	191.603900	2.909798
-69.609860	37.980580	193.864600	1.455949
-68.347290	37.996460	195.010900	.652927
-67.084140	37.998780	195.519000	.267329
-65.821270	37.987530	195.719700	.099565
-64.559530	37.962740	195.791000	.032847
-63.299760	37.924460	195.813400	.008929
-62.042780	37.872770	195.819500	.001572
-60.789440	37.807780	195.820700	.000678
-59.540510	37.729610	195.820700	.000755
-58.296810	37.638420	195.820600	.000578
-57.059080	37.534390	195.820400	.000385
-55.828080	37.417740	195.820200	.000220
-54.604500	37.288680	195.820000	.000111
-53.389020	37.147460	195.820000	.000068
-52.182290	36.994370	195.819900	.000048
-50.984880	36.829660	195.820000	.000000
-49.797380	36.653650	195.819900	.000015
-48.620270	36.466650	195.819900	.000015
-47.454020	36.268990	195.819900	.000015
-46.299010	36.060990	195.820000	.000000
-45.155610	35.843010	195.820000	.000049
-44.024070	35.615370	195.820000	.000000
-90.516530	36.687310	194.663900	.693943
-89.365660	36.916220	194.186300	.879334
-88.202590	37.134950	192.966500	1.327367
-87.027650	37.343150	191.031100	1.984446
-85.841200	37.540460	188.435000	2.764594
-84.643710	37.726540	185.162300	3.697999
-83.435650	37.901050	181.400300	4.904890
-82.217600	38.063690	177.737300	6.239858
-80.990140	38.214140	174.788100	7.396170
-79.753940	38.352110	173.170700	8.135569
-78.509670	38.477340	169.623500	10.568760
-77.258070	38.589580	161.255700	15.287920
-75.999910	38.688600	150.594800	18.722990
-74.735980	38.774210	149.112300	18.111830
-73.467090	38.846230	159.251600	14.496240
-72.194090	38.904510	173.582400	11.078240
-70.917830	38.948930	184.506900	6.629553
-69.639210	38.979390	190.814100	3.233890
-68.359090	38.995840	193.842500	1.373179
-67.078360	38.998240	195.113900	.536003
-65.797930	38.986590	195.596000	.192925
-64.518690	38.960900	195.760600	.061599

-63.241520	38.921240	195.810100	.015395
-61.967320	38.867690	195.822000	.002483
-60.696920	38.800350	195.823400	.002602
-59.431210	38.719360	195.822800	.002410
-58.170970	38.624900	195.822000	.001791
-56.917050	38.517150	195.821200	.001170
-55.670200	38.396340	195.820600	.000627
-54.431160	38.262690	195.820200	.000301
-53.200640	38.116470	195.820000	.000143
-51.979310	37.957960	195.819900	.000057
-50.767770	37.787460	195.819900	.000047
-49.566610	37.605300	195.819900	.000015
-48.376340	37.411790	195.819900	.000015
-47.197410	37.207270	195.819900	.000015
-46.030250	36.992090	195.819900	.000015
-44.875180	36.766620	195.820000	.000053
-43.732470	36.531200	195.820000	.000000
-90.814130	37.606180	193.268300	1.237028
-89.651300	37.842850	192.061400	1.588820
-88.475770	38.069030	189.111800	2.372947
-87.287830	38.284350	184.556200	3.377017
-86.087840	38.488450	178.825700	4.309809
-84.876290	38.680970	171.716700	5.437061
-83.653650	38.861550	162.912400	7.399747
-82.420500	39.029860	153.757000	10.016920
-81.177470	39.185590	146.107000	12.070980
-79.925230	39.328410	143.952700	12.374700
-78.664510	39.458070	137.741500	16.049090
-77.396060	39.574290	119.343800	23.608970
-76.120680	39.676830	96.472610	26.879920
-74.839220	39.765490	90.254200	24.751180
-73.552520	39.840080	105.777000	21.461610
-72.261480	39.900440	139.963200	21.188430
-70.967010	39.946460	169.013800	13.158910
-69.670030	39.978020	184.711100	6.214346
-68.371470	39.995060	191.695900	2.494010
-67.072270	39.997550	194.438100	.908807
-65.773400	39.985470	195.412200	.317354
-64.475770	39.958870	195.729000	.096914
-63.180340	39.917780	195.816900	.021930
-61.888020	39.862300	195.833700	.006924
-60.599740	39.792560	195.832400	.008053
-59.316390	39.708680	195.828900	.006781
-58.038840	39.610860	195.826000	.005048
-56.767930	39.499290	195.823700	.003287
-55.504470	39.374200	195.821600	.001683
-54.249260	39.235840	195.820500	.000782
-53.003000	39.084480	195.820200	.000333
-51.766410	38.920440	195.820000	.000140
-50.540120	38.744010	195.820000	.000067
-49.324710	38.555530	195.819900	.000046
-48.120720	38.355340	195.819900	.000015
-46.928620	38.143810	195.819900	.000015
-45.748810	37.921300	195.819900	.000015
-44.581620	37.688170	195.820000	.000057
-43.427300	37.444810	195.820000	.000000
-91.125630	38.522790	190.997800	1.961718
-89.950410	38.767390	188.345400	2.564476
-88.761920	39.001200	181.895800	3.807998
-87.560450	39.223820	172.177700	4.994740
-86.346370	39.434880	161.138400	5.355610
-85.120160	39.633990	148.748100	5.692137
-83.882300	39.820790	133.596200	7.317558
-82.633390	39.994930	115.670700	10.581880
-81.374080	40.156070	97.575500	13.287930
-80.105060	40.303890	95.482170	12.347030
-78.827100	40.438090	96.785460	13.634610

-77.540980	40.558400	80.660710	16.555610
-76.247540	40.664560	49.009380	17.359360
-74.947690	40.756360	35.706990	15.093690
-73.642300	40.833600	46.407700	20.741710
-72.332310	40.896110	90.471440	25.854360
-71.018690	40.943760	144.764400	20.432980
-69.702430	40.976440	175.962100	9.589266
-68.384490	40.994090	189.054300	3.634016
-67.065880	40.996670	193.766500	1.256013
-65.747600	40.984170	195.250700	.441668
-64.430650	40.956610	195.738800	.132975
-63.116020	40.914060	195.859900	.029447
-61.804690	40.856610	195.869800	.021553
-60.497620	40.784390	195.856600	.021095
-59.195770	40.697540	195.844500	.017316
-57.900040	40.596260	195.836600	.013194
-56.611330	40.480750	195.829700	.008342
-55.330490	40.351270	195.823700	.004044
-54.058330	40.208070	195.821200	.001850
-52.795620	40.051450	195.820400	.000812
-51.543080	39.881710	195.820100	.000339
-50.301390	39.699200	195.820000	.000126
-49.071130	39.504250	195.819900	.000055
-47.852850	39.297230	195.819900	.000046
-46.647030	39.078520	195.819900	.000015
-45.454070	38.848490	195.819900	.000015
-44.274280	38.607530	195.819900	.000049
-43.107920	38.356030	195.820000	.000000
-91.451740	39.437030	188.086100	2.794467
-90.263630	39.689750	183.046100	3.781660
-89.061680	39.931370	170.616100	5.726174
-87.846150	40.161480	151.283100	6.978267
-86.617400	40.379670	127.247700	6.340165
-85.375900	40.585540	102.043200	5.405433
-84.122160	40.778730	87.414760	4.379388
-82.856800	40.958850	79.377770	5.396284
-81.580460	41.125550	50.633300	7.539472
-80.293880	41.278500	46.053630	5.657796
-78.997860	41.417370	68.662590	4.601447
-77.693230	41.541890	76.748560	5.333836
-76.380870	41.651780	40.759410	6.865026
-75.061690	41.746810	20.670190	5.272245
-73.736660	41.826770	35.521880	10.153820
-72.406780	41.891490	74.454950	23.862920
-71.073040	41.940830	130.970100	20.258170
-69.736500	41.974670	170.349700	10.427090
-68.398190	41.992950	187.460600	4.196463
-67.059160	41.995620	193.463000	1.539131
-65.720480	41.982670	195.304500	.572187
-64.383220	41.954140	195.861300	.179541
-63.048400	41.910080	195.983500	.056014
-61.717090	41.850600	195.955800	.055925
-60.390290	41.775820	195.911100	.049682
-59.069010	41.685920	195.880900	.040901
-57.754210	41.581080	195.861100	.031162
-56.446830	41.461530	195.836300	.016799
-55.147770	41.327530	195.826800	.008496
-53.857880	41.179360	195.822200	.003951
-52.577970	41.017320	195.820900	.001760
-51.308780	40.841740	195.820300	.000731
-50.051000	40.652980	195.820100	.000303
-48.805250	40.451390	195.820000	.000113
-47.572090	40.237360	195.819900	.000051
-46.352010	40.011290	195.819900	.000020
-45.145370	39.773560	195.819900	.000015
-43.952510	39.524580	195.819900	.000049
-42.773630	39.264750	195.820000	.000000

-91.793200	40.348750	185.529600	3.583886
-90.591710	40.609810	177.972900	5.009508
-89.375760	40.859430	158.469000	7.922453
-88.145600	41.097210	124.073700	10.710640
-86.901570	41.322720	78.347230	8.108902
-85.644150	41.535550	38.901790	3.972890
-84.373860	41.735290	43.261650	7.591066
-83.091290	41.921560	37.447500	3.437662
-81.797160	42.093980	36.940830	4.631444
-80.492230	42.252200	43.765300	6.870155
-79.177290	42.395880	65.684130	8.039695
-77.853240	42.524730	62.907230	6.755482
-76.521000	42.638450	37.672330	9.348027
-75.181550	42.736820	29.100230	7.085052
-73.835900	42.819590	52.577320	6.279877
-72.485100	42.886590	95.984060	15.005280
-71.130210	42.937670	140.087900	14.687130
-69.772340	42.972710	171.804100	8.906013
-68.412590	42.991630	186.677200	4.552042
-67.052090	42.994400	192.965500	2.056222
-65.691960	42.980990	195.379600	.759122
-64.333320	42.951450	196.163700	.215333
-62.977290	42.905830	196.262300	.142530
-61.624970	42.844260	196.131500	.133626
-60.277440	42.766850	196.017700	.109707
-58.935750	42.673790	195.942000	.085753
-57.600940	42.565280	195.877700	.056982
-56.273990	42.441570	195.848200	.034321
-54.955840	42.302930	195.828700	.017152
-53.647390	42.149640	195.824300	.007776
-52.349470	41.982030	195.822300	.003378
-51.062890	41.800450	195.821000	.001440
-49.788320	41.605260	195.820300	.000596
-48.526440	41.396860	195.820100	.000228
-47.277780	41.175640	195.820000	.000080
-46.042850	40.942000	195.819900	.000047
-44.822010	40.696380	195.819900	.000015
-43.615560	40.439180	195.819900	.000032
-42.423690	40.170830	195.820000	.000000
-92.150770	41.257820	184.584500	4.091657
-90.935390	41.527410	176.443600	5.729461
-89.704910	41.785250	155.301100	8.853417
-88.459540	42.030900	117.875600	10.940530
-87.199620	42.263930	64.882680	10.529480
-85.925610	42.483890	25.849040	5.146135
-84.638040	42.690380	19.124320	5.564531
-83.337520	42.882970	35.036810	5.963256
-82.024790	43.061280	45.842770	4.861713
-80.700610	43.224930	87.058910	8.609601
-79.365870	43.373580	93.896510	8.397597
-78.021450	43.506890	75.661830	6.349652
-76.668370	43.624570	26.491230	4.618338
-75.307630	43.726360	39.274970	4.940176
-73.940300	43.812040	68.746050	5.551093
-72.567500	43.881390	112.153200	10.973350
-71.190370	43.934270	148.750800	11.872000
-69.810050	43.970550	171.874500	9.301274
-68.427750	43.990140	186.437400	5.126933
-67.044650	43.993000	193.384400	2.087790
-65.661930	43.979120	196.098500	.642318
-64.280810	43.948540	196.972400	.367572
-62.902460	43.901310	196.821000	.390350
-61.528050	43.837570	196.433100	.299976
-60.158730	43.757450	196.159400	.223212
-58.795620	43.661140	195.994800	.167787
-57.439790	43.548850	195.908400	.120159
-56.092310	43.420850	195.856100	.072343

-54.754150 43.277410 195.843700 .032218
-53.426270 43.118850 195.833900 .013512
-52.109530 42.945500 195.826800 .005782
-50.804760 42.757750 195.823000 .002578
-49.512690 42.555960 195.821100 .001106
-48.233980 42.340550 195.820300 .000436
-46.969190 42.111940 195.820000 .000161
-45.718810 41.870550 195.819900 .000055
-44.483210 41.616830 195.819900 .000046
-43.262650 41.351200 195.819900 .000048
-42.057300 41.074100 195.820000 .000000
-92.525320 42.164070 185.058600 4.322939
-91.295520 42.442420 177.110700 6.324414
-90.049940 42.708690 156.212200 10.413490
-88.788760 42.962440 119.210500 14.161060
-87.512310 43.203190 69.656680 15.553720
-86.221000 43.430500 32.919080 9.639692
-84.915410 43.643920 24.079050 5.060366
-83.596150 43.843030 29.271010 8.872289
-82.263960 44.027400 67.244200 10.064100
-80.919660 44.196660 102.194200 10.242800
-79.564150 44.350410 110.609100 9.148108
-78.198390 44.488330 79.688010 5.288359
-76.823410 44.610100 39.214170 5.261677
-75.440290 44.715440 22.517700 3.752824
-74.050180 44.804110 52.320220 9.276512
-72.654240 44.875890 97.122220 15.353700
-71.253690 44.930630 141.868900 15.023410
-69.849760 44.968180 170.540300 10.553110
-68.443720 44.988460 185.859000 5.652718
-67.036810 44.991420 193.422000 2.302007
-65.630330 44.977050 196.558900 .704199
-64.225530 44.945390 197.416700 .632332
-62.823690 44.896510 197.157800 .594567
-61.426040 44.830540 196.617400 .441467
-60.033810 44.747610 196.232900 .331200
-58.648170 44.647940 196.039300 .257638
-57.270290 44.531750 195.939700 .182298
-55.901260 44.399320 195.903500 .093132
-54.542130 44.250930 195.877500 .043585
-53.193900 44.086940 195.854300 .020523
-51.857480 43.907680 195.836500 .009718
-50.533710 43.713560 195.827100 .004495
-49.223370 43.504980 195.822700 .001994
-47.927120 43.282370 195.820800 .000805
-46.645540 43.046150 195.820200 .000295
-45.379100 42.796800 195.820000 .000100
-44.128160 42.534760 195.819900 .000047
-42.892960 42.260480 195.819900 .000048
-41.673610 41.974410 195.820000 .000000
-92.917770 43.067330 186.497600 4.075287
-91.673000 43.354680 179.500500 5.944985
-90.411740 43.629630 160.765100 9.943556
-89.134110 43.891690 124.957300 15.314140
-87.840450 44.140390 76.012250 16.735780
-86.531140 44.375260 33.974720 12.931830
-85.206750 44.595830 28.441640 6.479904
-83.867900 44.801650 40.661790 10.663000
-82.515380 44.992280 44.769330 7.269707
-81.150000 45.167310 67.283530 9.477146
-79.772730 45.326340 71.517870 7.414389
-78.384560 45.469020 68.395010 4.225425
-76.986600 45.595010 51.066180 5.760724
-75.579970 45.704020 41.838500 6.483476
-74.165890 45.795780 58.169880 14.468390
-72.745610 45.870080 105.167700 16.834850
-71.320400 45.926740 148.090900 13.485470

-69.891600 45.965610 171.152500 10.070670
-68.460530 45.986610 183.756200 6.511417
-67.028560 45.989670 190.462000 3.491585
-65.597030 45.974800 194.040600 1.543487
-64.167300 45.942020 195.505700 .653239
-62.740720 45.891430 196.015300 .367334
-61.318600 45.823140 196.026300 .280378
-59.902260 45.737310 195.988600 .213115
-58.492950 45.634170 195.938100 .156412
-57.091900 45.513950 195.915500 .085509
-55.700260 45.376930 195.951200 .077145
-54.319140 45.223450 195.927000 .066415
-52.949590 45.053840 195.884300 .036325
-51.592580 44.868490 195.852800 .017026
-50.248970 44.667810 195.834500 .007779
-48.919560 44.452230 195.825500 .003356
-47.605040 44.222190 195.821800 .001360
-46.305980 43.978160 195.820500 .000492
-45.022840 43.720610 195.820000 .000168
-43.755960 43.450020 195.819900 .000055
-42.505550 43.166850 195.819900 .000032
-41.271660 42.871580 195.820000 .000000
-93.329110 43.967430 188.427600 3.485555
-92.068780 44.264030 182.906300 4.985504
-90.791210 44.547890 168.302000 7.858193
-89.496510 44.818510 139.765900 11.540240
-88.184940 45.075390 94.835010 15.484660
-86.856880 45.318050 42.490460 17.284190
-85.512890 45.545990 29.858690 10.277890
-84.153590 45.758730 43.200380 12.360990
-82.779790 45.955820 47.313350 10.070860
-81.392350 46.136820 30.518670 6.820807
-79.992260 46.301310 31.578660 5.211039
-78.580580 46.448910 64.689320 7.555165
-77.158460 46.579270 70.913990 10.383760
-75.727110 46.692070 84.644640 12.490610
-74.287820 46.787040 103.137500 16.685700
-72.841890 46.863950 139.658200 13.157570
-71.390720 46.922590 162.364700 9.836996
-69.935700 46.962830 173.542900 8.767883
-68.478260 46.984570 181.408500 7.307458
-67.019850 46.987740 187.033000 5.065428
-65.561920 46.972340 190.383900 3.218155
-64.105920 46.938410 192.811700 1.836972
-62.653260 46.886040 194.226200 1.019339
-61.205390 46.815350 195.041600 .564891
-59.763680 46.726520 195.438600 .328973
-58.329470 46.619790 195.699500 .219814
-56.904060 46.495390 195.928100 .181698
-55.488670 46.353640 196.021300 .177959
-54.084500 46.194880 196.044400 .128680
-52.692630 46.019470 195.958500 .067356
-51.314070 45.827840 195.884600 .030220
-49.949730 45.620390 195.846300 .012607
-48.600430 45.397590 195.829600 .005181
-47.266860 45.159900 195.823100 .002021
-45.949600 44.907820 195.820800 .000736
-44.649100 44.641840 195.820200 .000248
-43.365670 44.362450 195.820000 .000082
-42.099450 44.070140 195.819900 .000048
-40.850440 43.765410 195.820000 .000000
-93.760410 44.864160 190.446900 2.801573
-92.483920 45.170280 186.474800 4.022871
-91.189420 45.463320 175.908200 6.632107
-89.876960 45.742750 153.828600 10.686200
-88.546760 46.008070 112.301200 18.055640
-87.199170 46.258760 66.492580 22.543450

-85.834720	46.494300	42.867530	20.308890
-84.454070	46.714200	59.192240	11.795400
-83.058010	46.917960	56.225760	12.276140
-81.647480	47.105130	43.875420	13.763300
-80.223460	47.275260	47.346190	18.716850
-78.787090	47.427960	64.049320	22.023080
-77.339580	47.562840	96.377880	23.129500
-75.882230	47.679570	120.959200	20.029420
-74.416370	47.777870	148.093200	14.089720
-72.943440	47.857470	167.280000	8.739839
-71.464880	47.918190	175.565400	6.697657
-69.982220	47.959850	179.663500	6.687415
-68.496960	47.982350	182.875900	6.447023
-67.010670	47.985630	186.354600	5.263250
-65.524890	47.969690	189.363200	3.668438
-64.041170	47.934570	191.572800	2.403002
-62.561040	47.880340	193.149200	1.567337
-61.086020	47.807170	193.966200	1.126085
-59.617590	47.715230	194.804600	.748418
-58.157170	47.604770	195.521600	.431475
-56.706150	47.476050	196.018400	.285847
-55.265830	47.329400	196.297100	.258157
-53.837470	47.165180	196.273300	.207998
-52.422200	46.983780	196.076400	.115298
-51.021090	46.785640	195.928000	.047199
-49.635090	46.571200	195.859500	.017874
-48.265020	46.340950	195.833600	.006787
-46.911610	46.095370	195.824200	.002586
-45.575410	45.834990	195.821100	.000927
-44.256860	45.560320	195.820200	.000311
-42.956210	45.271870	195.820000	.000092
-41.673580	44.970160	195.819900	.000049
-40.408870	44.655690	195.820000	.000000
-94.212860	45.757310	192.247200	2.107625
-92.919560	46.073230	189.739000	2.999285
-91.607470	46.375730	183.318800	4.879559
-90.276550	46.664250	169.975200	8.486279
-88.926960	46.938270	144.266400	16.017790
-87.559010	47.197250	107.916000	26.062320
-86.173240	47.440650	85.566990	29.681160
-84.770290	47.667940	84.473290	25.942870
-83.350960	47.878600	83.795060	24.972340
-81.916200	48.072160	85.654130	23.354520
-80.467090	48.248140	86.776860	24.128650
-79.004780	48.406110	109.256100	26.583920
-77.530590	48.545680	133.044500	23.660970
-76.045850	48.666500	157.883000	14.903120
-74.552020	48.768250	173.832900	9.037613
-73.050600	48.850660	182.456400	5.499517
-71.543170	48.913520	185.749100	4.062467
-70.031330	48.956650	186.580600	4.049139
-68.516710	48.979950	187.862000	4.130021
-67.000980	48.983350	189.227300	3.681456
-65.485790	48.966840	190.598200	2.963382
-63.972820	48.930470	191.949200	2.228384
-62.463700	48.874330	192.901000	1.729680
-60.960050	48.798580	193.397600	1.472360
-59.463460	48.703410	194.013100	1.162788
-57.975440	48.589080	195.038300	.674485
-56.497490	48.455880	196.024200	.287792
-55.030970	48.304140	196.405800	.270229
-53.577210	48.134270	196.354400	.231757
-52.137430	47.946660	196.141700	.134875
-50.712720	47.741790	195.937300	.051863
-49.304080	47.520120	195.860800	.017995
-47.912350	47.282170	195.834200	.006992
-46.538250	47.028450	195.824400	.002672

-45.182340	46.759510	195.821200	.000968
-43.845020	46.475880	195.820200	.000325
-42.526500	46.178100	195.820000	.000092
-41.226800	45.866700	195.819900	.000050
-39.945740	45.542200	195.820000	.000000
-94.687740	46.646660	193.610300	1.490375
-93.376940	46.972690	192.235900	2.051913
-92.046550	47.284930	188.974400	3.193234
-90.696440	47.582840	181.584700	5.854421
-89.326680	47.865840	167.814200	11.579350
-87.937550	48.133390	148.119200	20.778940
-86.529520	48.384910	131.156700	26.541030
-85.103260	48.619850	123.709000	28.091930
-83.659580	48.837660	123.898000	24.889800
-82.199460	49.037830	131.643400	19.913220
-80.724000	49.219880	142.244700	18.811670
-79.234450	49.383340	153.063200	18.043130
-77.732160	49.527770	166.584500	14.060940
-76.218590	49.652830	178.662200	9.001407
-74.695260	49.758160	185.526800	5.580577
-73.163800	49.843490	189.077800	3.564620
-71.625870	49.908580	190.797100	2.515455
-70.083210	49.953250	191.660300	2.137830
-68.537570	49.977380	192.418000	1.947289
-66.990740	49.980900	193.113200	1.748707
-65.444490	49.963800	193.133300	1.671042
-63.900620	49.926140	193.477600	1.451326
-62.360880	49.868010	193.974700	1.207782
-60.827010	49.789570	194.177600	1.098808
-59.300730	49.691040	194.660200	.882358
-57.783640	49.572690	195.432600	.518718
-56.277320	49.434830	196.038800	.258595
-54.783270	49.277820	196.306600	.220268
-53.302860	49.102080	196.270200	.193175
-51.837360	48.908040	196.064300	.107464
-50.387950	48.696200	195.921600	.043408
-48.955640	48.467040	195.852500	.015064
-47.541310	48.221130	195.831400	.005754
-46.145660	47.959000	195.823600	.002333
-44.769230	47.681210	195.820900	.000860
-43.412380	47.388330	195.820200	.000291
-42.075270	47.080920	195.820000	.000087
-40.757810	46.759540	195.819900	.000050
-39.459720	46.424710	195.820000	.000000
-95.186440	47.531940	194.526300	.998877
-93.857410	47.868410	193.933900	1.272137
-92.508000	48.190720	192.564500	1.789646
-91.137910	48.498310	189.102100	3.257174
-89.747150	48.790600	182.381600	6.505474
-88.335950	49.067010	172.912000	11.665630
-86.904710	49.326930	163.987700	16.519210
-85.454100	49.569790	159.214700	17.178200
-83.984930	49.795020	161.459200	14.751210
-82.498220	50.002060	167.504400	12.038560
-80.995120	50.190400	173.298100	10.275320
-79.476920	50.359550	177.917100	9.511942
-77.945060	50.509060	184.026200	7.056260
-76.401090	50.638520	188.860800	4.520634
-74.846650	50.747580	191.268800	3.043954
-73.283450	50.835950	192.364000	2.238402
-71.713320	50.903350	192.885300	1.798451
-70.138080	50.949630	193.427700	1.474476
-68.559640	50.974620	194.612400	1.027845
-66.979900	50.978270	195.384600	.762202
-65.400800	50.960560	195.013500	.784498
-63.824260	50.921550	194.841300	.731090
-62.252170	50.861340	195.030800	.593455

-60.686400	50.780110	195.264300	.495133
-59.128770	50.678080	195.613800	.377687
-57.581030	50.555550	195.897800	.265948
-56.044860	50.412850	196.117100	.169361
-54.521820	50.250360	196.132000	.138076
-53.013420	50.068520	196.059000	.107651
-51.520960	49.867810	195.962600	.064383
-50.045680	49.648740	195.880400	.027580
-48.588630	49.411830	195.843400	.010854
-47.150700	49.157670	195.828300	.004584
-45.732590	48.886830	195.822500	.001827
-44.334810	48.599900	195.820600	.000668
-42.957660	48.297460	195.820100	.000216
-41.601190	47.980120	195.820000	.000070
-40.265250	47.648430	195.819900	.000050
-38.949390	47.302930	195.820000	.000000
-95.710510	48.412890	195.133300	.628092
-94.362470	48.760130	194.998500	.714398
-92.993230	49.092850	194.565200	.855910
-91.602360	49.410460	193.131000	1.503086
-90.189750	49.712350	190.306900	2.926330
-88.755540	49.997940	186.687000	4.968974
-87.300090	50.266570	183.093600	6.995278
-85.824040	50.517650	181.306300	7.896480
-84.328210	50.750570	182.374900	7.064734
-82.813610	50.964740	184.720300	5.894921
-81.281460	51.159630	187.332500	4.852535
-79.733130	51.334700	189.614900	3.950449
-78.170130	51.489480	192.173800	2.698809
-76.594090	51.623530	193.746600	1.792627
-75.006800	51.736490	194.236900	1.376138
-73.410070	51.828010	194.365400	1.183555
-71.805870	51.897850	194.266000	1.163636
-70.196170	51.945790	194.331400	1.145929
-68.582990	51.971680	195.020900	.887037
-66.968440	51.975460	196.218000	.511796
-65.354560	51.957120	196.213000	.389911
-63.743440	51.916690	195.888200	.291555
-62.137130	51.854320	195.839000	.218186
-60.537630	51.770170	195.879300	.183068
-58.946900	51.664510	196.057900	.182406
-57.366820	51.537620	196.084500	.147111
-55.799180	51.389870	196.025700	.100033
-54.245650	51.221680	195.975500	.069187
-52.707820	51.033510	195.929600	.048721
-51.187080	50.825860	195.887400	.031096
-49.684720	50.599280	195.852400	.015460
-48.201800	50.354330	195.833300	.007179
-46.739240	50.091630	195.824800	.003080
-45.297720	49.811770	195.821500	.001242
-43.877700	49.515370	195.820300	.000445
-42.479410	49.203060	195.820000	.000142
-41.102810	48.875440	195.819900	.000051
-39.747590	48.533100	195.819900	.000050
-38.413150	48.176570	195.820000	.000000
-96.261670	49.289190	195.584400	.349466
-94.893750	49.647600	195.577900	.369747
-93.503820	49.991080	195.542900	.351191
-92.091320	50.319060	194.924000	.614266
-90.655950	50.630910	193.880000	1.162866
-89.197760	50.925990	192.576500	1.881187
-87.717060	51.203660	191.413600	2.574495
-86.214420	51.463270	190.849100	2.973220
-84.690660	51.704190	191.087100	2.865261
-83.146820	51.925780	192.062900	2.440610
-81.584160	52.127480	193.400700	1.808573
-80.004110	52.308710	194.653200	1.182501

-78.408280	52.468990	195.514500	.728882
-76.798410	52.607840	195.822700	.540504
-75.176390	52.724850	195.634800	.513205
-73.544200	52.819690	195.305000	.598499
-71.903920	52.892050	195.203600	.649563
-70.257710	52.941730	194.975700	.830844
-68.607750	52.968570	195.414900	.755741
-66.956280	52.972490	196.362300	.484652
-65.305560	52.953480	196.756500	.368730
-63.657820	52.911580	196.616000	.328232
-62.015270	52.846950	196.366400	.259370
-60.380080	52.759750	196.222700	.201894
-58.754360	52.650280	196.125500	.155191
-57.140120	52.518850	196.021500	.101471
-55.539290	52.365840	195.941100	.057283
-53.953660	52.191710	195.898500	.035381
-52.384890	51.996940	195.870500	.023186
-50.834480	51.782070	195.849700	.014676
-49.303730	51.547700	195.834800	.008173
-47.793770	51.294400	195.826200	.004017
-46.305490	51.022820	195.822300	.001791
-44.839570	50.733620	195.820600	.000718
-43.396390	50.427420	195.820100	.000258
-41.976090	50.104880	195.820000	.000087
-40.578500	49.766630	195.819900	.000047
-39.203150	49.413260	195.819900	.000050
-37.849210	49.045330	195.820000	.000000
-96.841810	50.160530	195.781900	.173780
-95.453030	50.530490	195.839200	.173133
-94.041510	50.885130	195.811000	.145269
-92.606450	51.223850	195.575800	.238092
-91.147380	51.546020	195.199400	.441211
-89.664200	51.850960	194.759000	.692481
-88.157130	52.138020	194.398800	.920801
-86.626690	52.406500	194.244400	1.051709
-85.073680	52.655720	194.375600	1.023310
-83.499180	52.885050	194.910900	.824346
-81.904440	53.093850	195.715400	.480337
-80.290990	53.281520	196.290500	.261921
-78.660530	53.447530	196.507400	.282518
-77.014920	53.591380	196.411300	.281625
-75.356160	53.712650	195.924100	.269112
-73.686420	53.810940	195.262200	.498583
-72.007930	53.885960	195.064000	.597263
-70.322990	53.937460	195.018200	.692837
-68.634010	53.965280	195.151200	.765086
-66.943400	53.969350	195.978400	.507217
-65.253590	53.949630	196.457300	.290695
-63.567000	53.906200	196.498300	.261686
-61.886040	53.839190	196.333900	.222288
-60.213040	53.748820	196.140900	.154098
-58.550290	53.635360	196.039600	.111767
-56.899960	53.499180	195.945800	.064567
-55.264110	53.340690	195.888000	.033472
-53.644650	53.160350	195.859000	.019027
-52.043340	52.958710	195.842600	.011727
-50.461760	52.736320	195.832300	.007200
-48.901280	52.493820	195.826000	.004059
-47.363030	52.231850	195.822400	.002043
-45.847900	51.951060	195.820900	.000904
-44.356530	51.652150	195.820200	.000352
-42.889220	51.335790	195.820000	.000134
-41.445980	51.002650	195.819900	.000059
-40.026490	50.653380	195.819900	.000015
-38.630060	50.288590	195.819900	.000051
-37.255620	49.908830	195.820000	.000000
-97.453060	51.026530	195.833100	.071550

-96.042340	51.408490	195.865200	.068303
-94.608220	51.774700	195.841100	.057437
-93.149600	52.124570	195.757600	.091871
-91.665800	52.457450	195.636100	.163520
-90.156560	52.772640	195.501700	.247244
-88.621980	53.069450	195.399300	.320463
-87.062480	53.347160	195.370500	.362178
-85.478850	53.605050	195.441400	.347848
-83.872130	53.842430	195.681700	.261964
-82.243660	54.058630	196.030400	.142143
-80.595020	54.253030	196.242100	.159496
-78.928000	54.425040	196.378500	.240075
-77.244600	54.574130	196.341500	.252657
-75.546970	54.699840	195.911800	.195978
-73.837420	54.801750	195.192300	.460791
-72.118360	54.879540	194.802400	.655616
-70.392330	54.932960	194.970600	.616068
-68.661900	54.961820	195.112600	.637100
-66.929700	54.966030	195.664500	.458539
-65.198370	54.945580	196.034700	.245997
-63.470540	54.900540	196.135800	.159330
-61.748820	54.831050	196.040000	.105203
-60.035740	54.737340	195.984400	.074084
-58.333780	54.619720	195.941300	.061568
-56.645260	54.478570	195.885300	.034663
-54.972420	54.314330	195.854300	.018271
-53.317280	54.127510	195.838100	.010232
-51.681740	53.918690	195.829500	.005977
-50.067420	53.688470	195.824800	.003469
-48.475770	53.437500	195.822100	.001879
-46.907930	53.166490	195.820800	.000926
-45.364770	52.876130	195.820300	.000389
-43.846840	52.567130	195.820000	.000160
-42.354370	52.240220	195.819900	.000060
-40.887210	51.896080	195.819900	.000047
-39.444820	51.535370	195.819900	.000015
-38.026250	51.158730	195.820000	.000055
-36.630120	50.766700	195.820000	.000000
-98.097820	51.886770	195.835500	.024548
-96.663910	52.281200	195.841800	.022427
-95.206040	52.659450	195.834400	.020924
-93.722790	53.020900	195.809800	.032688
-92.213200	53.364910	195.773700	.056115
-90.676770	53.690770	195.735300	.082959
-89.113440	53.997730	195.709200	.105689
-87.523560	54.285050	195.708100	.117919
-85.907830	54.551970	195.737100	.115330
-84.267300	54.797760	195.796300	.096873
-82.603340	55.021710	195.875200	.077198
-80.917590	55.223140	195.956200	.096717
-79.211960	55.401440	196.107100	.175033
-77.488560	55.556020	196.191400	.222102
-75.749730	55.686380	195.937800	.170210
-73.997920	55.792100	195.267400	.394486
-72.235800	55.872810	194.762700	.656754
-70.466090	55.928230	194.941700	.586862
-68.691570	55.958180	195.323500	.435309
-66.915130	55.962550	195.611700	.318822
-65.139640	55.941330	195.882700	.162584
-63.367970	55.894590	195.927800	.093727
-61.602940	55.822490	195.899700	.057073
-59.847310	55.725280	195.890200	.033208
-58.103760	55.603290	195.868700	.025428
-56.374820	55.456930	195.848200	.015678
-54.662870	55.286670	195.835000	.009126
-52.970100	55.093080	195.827600	.005134
-51.298500	54.876750	195.823500	.002879

-49.649800	54.638340	195.821600	.001565
-48.025460	54.378550	195.820700	.000796
-46.426660	54.098120	195.820200	.000370
-44.854210	53.797780	195.820000	.000157
-43.308580	53.478290	195.819900	.000059
-41.789850	53.140400	195.819900	.000047
-40.297680	52.784830	195.819900	.000015
-38.831280	52.412250	195.819900	.000015
-37.389370	52.023280	195.820000	.000053
-35.970190	51.618480	195.820000	.000000
-98.778840	52.740810	195.829500	.008274
-97.320270	53.148240	195.830200	.007484
-95.837370	53.539010	195.827900	.007056
-94.328290	53.912530	195.820900	.010237
-92.791720	54.268120	195.810800	.017271
-91.226900	54.605080	195.800500	.025445
-89.633560	54.922620	195.794300	.032226
-88.011890	55.219970	195.795100	.035900
-86.362530	55.496330	195.802500	.036452
-84.686490	55.750910	195.809900	.039113
-82.985150	55.982970	195.802700	.055490
-81.260250	56.191770	195.820900	.086961
-79.513790	56.376650	195.934400	.150950
-77.748020	56.536990	196.087800	.202602
-75.965460	56.672260	195.964800	.162638
-74.168790	56.781970	195.384200	.329293
-72.360860	56.865740	194.792200	.646187
-70.544630	56.923280	194.923000	.595583
-68.723180	56.954370	195.347100	.375081
-66.899620	56.958910	195.647900	.206288
-65.077100	56.936880	195.796100	.094503
-63.258730	56.888360	195.838700	.048195
-61.447630	56.813510	195.849900	.030093
-59.646790	56.712620	195.847300	.016932
-57.859090	56.586040	195.838200	.010591
-56.087300	56.434210	195.830800	.006906
-54.333970	56.257640	195.825700	.004120
-52.601470	56.056940	195.822700	.002334
-50.891880	55.832740	195.821200	.001259
-49.207030	55.585770	195.820500	.000637
-47.548420	55.316760	195.820200	.000318
-45.917200	55.026500	195.820000	.000137
-44.314130	54.715760	195.819900	.000058
-42.739580	54.385350	195.819900	.000046
-41.193420	54.036030	195.819900	.000015
-39.675070	53.668560	195.819900	.000015
-38.183400	53.283620	195.819900	.000015
-36.716770	52.881820	195.820000	.000050
-35.272900	52.463700	195.820000	.000000
-99.499240	53.588130	195.824500	.003036
-98.014280	54.009130	195.824300	.002711
-96.504860	54.412960	195.823500	.002365
-94.968610	54.799050	195.821500	.002830
-93.403790	55.166730	195.818800	.004686
-91.809290	55.515250	195.815900	.007062
-90.184560	55.843850	195.814100	.009144
-88.529620	56.151670	195.813000	.010620
-86.844990	56.437890	195.810900	.012623
-85.131650	56.701690	195.804200	.019091
-83.390960	56.942230	195.787900	.037951
-81.624720	57.158780	195.774700	.079689
-79.835050	57.350580	195.855700	.144925
-78.024370	57.516980	195.999300	.189303
-76.195360	57.657400	195.945700	.150068
-74.350940	57.771310	195.521900	.279521
-72.494220	57.858320	194.913800	.597255
-70.628410	57.918090	194.838100	.632841

-68.756890	57.950390	195.287100	.373342
-66.883060	57.955100	195.603300	.169103
-65.010370	57.932220	195.733900	.078615
-63.142240	57.881810	195.798400	.031607
-61.282040	57.804080	195.818500	.013179
-59.433060	57.699310	195.826900	.006803
-57.598450	57.567890	195.825700	.004301
-55.781200	57.410300	195.823400	.002735
-53.984060	57.227100	195.821800	.001677
-52.209560	57.018940	195.820800	.000937
-50.459930	56.786500	195.820300	.000484
-48.737060	56.530560	195.820100	.000231
-47.042490	56.251900	195.820000	.000102
-45.377330	55.951360	195.819900	.000055
-43.742270	55.629770	195.819900	.000015
-42.137470	55.287960	195.819900	.000015
-40.562610	54.926740	195.819900	.000015
-39.016750	54.546860	195.819900	.000015
-37.498410	54.149020	195.819900	.000015
-36.005400	53.733830	195.820000	.000045
-34.534880	53.301770	195.820000	.000000
-100.262700	54.428120	195.821800	.001208
-98.749250	54.863350	195.821500	.001075
-97.211560	55.280850	195.821200	.000876
-95.646590	55.680060	195.820700	.000756
-94.052090	56.060360	195.819900	.001150
-92.426510	56.420970	195.818900	.001912
-90.768940	56.761100	195.817700	.002807
-89.079190	57.079890	195.815200	.004063
-87.357570	57.376450	195.809900	.006763
-85.605000	57.649890	195.799500	.013429
-83.822840	57.899360	195.785100	.029079
-82.012920	58.124030	195.783000	.066726
-80.177480	58.323120	195.830400	.137471
-78.319110	58.495910	195.937500	.181061
-76.440710	58.641750	195.898000	.136137
-74.545430	58.760110	195.626000	.232808
-72.636660	58.850530	195.029000	.534883
-70.717930	58.912650	194.761500	.660038
-68.792920	58.946230	195.131500	.431657
-66.865380	58.951130	195.532500	.188159
-64.939080	58.927340	195.704800	.078569
-63.017780	58.874950	195.772700	.034432
-61.105210	58.794160	195.805800	.011952
-59.204930	58.685300	195.817100	.003423
-57.320400	58.548780	195.820200	.001034
-55.454840	58.385130	195.820600	.000752
-53.611270	58.194950	195.820400	.000516
-51.792370	57.978940	195.820200	.000311
-50.000500	57.737840	195.820000	.000164
-48.237630	57.472490	195.819900	.000075
-46.505310	57.183730	195.819900	.000046
-44.804610	56.872430	195.819900	.000015
-43.136050	56.539500	195.819900	.000015
-41.499610	56.185780	195.819900	.000015
-39.894630	55.812120	195.819900	.000015
-38.319800	55.419290	195.819900	.000015
-36.773090	55.007980	195.819900	.000015
-35.251730	54.578750	195.820000	.000045
-33.752120	54.132060	195.820000	.000000
-101.073700	55.260130	195.820600	.000482
-99.529010	55.710320	195.820400	.000408
-97.960900	56.142140	195.820400	.000324
-96.365430	56.555100	195.820200	.000241
-94.739640	56.948590	195.819800	.000311
-93.081440	57.321860	195.819300	.000710
-91.389490	57.674070	195.818100	.001449

-89.663250	58.004340	195.815200	.002876
-87.902820	58.311730	195.808900	.005759
-86.109000	58.595320	195.797900	.011711
-84.283100	58.854170	195.784900	.025249
-82.427000	59.087400	195.791200	.057068
-80.543050	59.294160	195.851400	.121183
-78.634010	59.473690	195.898700	.170362
-76.703000	59.625280	195.841000	.122887
-74.753460	59.748340	195.599700	.211281
-72.789070	59.842370	195.113500	.474385
-70.813740	59.906970	194.693500	.677380
-68.831480	59.941910	194.964600	.514146
-66.846440	59.947010	195.434000	.237786
-64.862770	59.922250	195.666500	.095163
-62.884610	59.867760	195.760700	.038153
-60.916040	59.783740	195.798800	.014694
-58.961010	59.670550	195.813600	.004982
-57.023270	59.528640	195.818400	.001389
-55.106350	59.358580	195.819600	.000288
-53.213500	59.161040	195.819900	.000049
-51.347600	58.936760	195.819900	.000073
-49.511150	58.686560	195.819900	.000054
-47.706190	58.411320	195.819900	.000020
-45.934240	58.111950	195.819900	.000015
-44.196280	57.789400	195.819900	.000015
-42.492650	57.444580	195.819900	.000015
-40.823030	57.078400	195.819900	.000015
-39.186360	56.691730	195.819900	.000015
-37.580830	56.285340	195.819900	.000015
-36.003750	55.859910	195.819900	.000015
-34.451600	55.415950	195.820000	.000041
-32.919830	54.953830	195.820000	.000000
-101.937200	56.083360	195.820300	.000190
-100.358100	56.549350	195.820100	.000138
-98.756940	56.996240	195.820000	.000119
-97.128790	57.423640	195.819900	.000065
-95.469860	57.830970	195.819700	.000189
-93.777320	58.217490	195.819400	.000520
-92.049300	58.582370	195.818100	.001241
-90.284800	58.924680	195.815100	.002699
-88.483640	59.243460	195.808700	.005479
-86.646400	59.537720	195.797900	.010669
-84.774360	59.806450	195.785500	.021777
-82.869400	60.048700	195.793900	.046448
-80.933970	60.263580	195.859900	.097149
-78.971000	60.450230	195.888400	.137366
-76.983890	60.607900	195.750400	.133515
-74.976370	60.735940	195.557400	.222246
-72.952470	60.833800	195.133000	.440962
-70.916480	60.901050	194.710500	.658501
-68.872850	60.937420	194.826200	.582665
-66.826130	60.942730	195.313000	.302158
-64.780930	60.916960	195.621300	.119553
-62.741830	60.860230	195.746000	.045467
-60.713310	60.772790	195.793900	.017223
-58.699720	60.654990	195.811600	.006209
-56.705200	60.507380	195.817500	.002093
-54.733590	60.330550	195.819300	.000625
-52.788400	60.125220	195.819800	.000167
-50.872730	59.892220	195.819900	.000044
-48.989200	59.632420	195.819900	.000015
-47.139900	59.346780	195.819900	.000015
-45.326320	59.036270	195.819900	.000015
-43.549270	58.701890	195.819900	.000015
-41.808860	58.344600	195.819900	.000015
-40.104370	57.965370	195.819900	.000015
-38.434220	57.565060	195.819900	.000015

-36.795950	57.144450	195.819900	.000015
-35.186060	56.704150	195.819900	.000015
-33.600030	56.244660	195.820000	.000034
-32.032190	55.766200	195.820000	.000000
-102.859900	56.896880	195.820100	.000058
-101.242000	57.379640	195.820000	.000028
-99.604390	57.842470	195.819900	.000047
-97.940930	58.285070	195.819900	.000061
-96.246660	58.706940	195.819700	.000187
-94.517820	59.107400	195.819300	.000489
-92.751850	59.485580	195.818100	.001169
-90.947170	59.840560	195.815100	.002542
-89.103220	60.171320	195.808900	.005024
-87.220290	60.476800	195.798600	.009363
-85.299550	60.755960	195.786700	.017670
-83.342830	61.007760	195.793000	.030792
-81.352710	61.231220	195.843800	.052711
-79.332320	61.425420	195.852200	.068376
-77.285290	61.589540	195.702700	.111560
-75.215710	61.722870	195.491300	.224330
-73.128000	61.824790	195.203200	.375627
-71.026890	61.894870	194.773000	.602913
-68.917300	61.932760	194.722500	.623441
-66.804310	61.938290	195.202600	.358994
-64.692990	61.911450	195.584200	.139804
-62.588420	61.852340	195.734500	.051372
-60.495590	61.761240	195.790500	.018929
-58.419300	61.638580	195.810500	.006855
-56.364090	61.484900	195.817200	.002376
-54.334160	61.300890	195.819200	.000761
-52.333320	61.087320	195.819800	.000237
-50.364880	60.845090	195.819900	.000068
-48.431600	60.575160	195.819900	.000015
-46.535590	60.278550	195.819900	.000015
-44.678250	59.956310	195.819900	.000015
-42.860180	59.609490	195.819900	.000015
-41.081100	59.239120	195.819900	.000015
-39.339830	58.846180	195.819900	.000015
-37.634110	58.431540	195.819900	.000015
-35.960610	57.995940	195.819900	.000015
-34.314830	57.539980	195.819900	.000015
-32.690990	57.064010	195.820000	.000026
-31.082000	56.568130	195.820000	.000000
-103.849600	57.699570	195.820000	.000018
-102.187300	58.200280	195.820000	.000015
-100.509000	58.680030	195.819900	.000015
-98.806840	59.138710	195.819900	.000069
-97.074520	59.575940	195.819700	.000170
-95.307120	59.991050	195.819400	.000450
-93.501110	60.383240	195.818100	.001050
-91.654170	60.751550	195.815400	.002241
-89.765210	61.094930	195.809800	.004282
-87.834170	61.412270	195.800600	.007745
-85.861950	61.702450	195.789800	.013424
-83.850420	61.964360	195.793300	.014394
-81.802150	62.196920	195.818500	.014389
-79.720490	62.399150	195.794400	.046737
-77.609360	62.570130	195.667000	.126327
-75.473210	62.709080	195.503300	.199383
-73.316960	62.815350	195.280100	.307495
-71.145790	62.888430	194.912500	.491911
-68.965200	62.927950	194.786100	.563534
-66.780790	62.933720	195.181100	.357633
-64.598260	62.905720	195.578400	.141027
-62.423240	62.844070	195.734900	.050411
-60.261290	62.749080	195.790900	.018344
-58.117710	62.621230	195.810700	.006618

-55.997520	62.461110	195.817300	.002301
-53.905320	62.269450	195.819200	.000757
-51.845240	62.047150	195.819800	.000234
-49.820820	61.795150	195.819900	.000069
-47.834930	61.514500	195.819900	.000021
-45.889670	61.206310	195.819900	.000015
-43.986290	60.871700	195.819900	.000015
-42.125070	60.511780	195.819900	.000015
-40.305270	60.127630	195.819900	.000015
-38.525020	59.720250	195.819900	.000015
-36.781200	59.290500	195.819900	.000015
-35.069400	58.839090	195.819900	.000015
-33.383770	58.366500	195.819900	.000015
-31.716990	57.872960	195.820000	.000021
-30.060130	57.358350	195.820000	.000000
-104.916500	58.490070	195.820000	.000000
-103.202300	59.010120	195.820000	.000000
-101.477600	59.507980	195.819900	.000049
-99.732440	59.983750	195.819900	.000057
-97.958720	60.437220	195.819800	.000149
-96.150010	60.867820	195.819400	.000383
-94.301540	61.274790	195.818400	.000873
-92.410060	61.657170	195.816100	.001785
-90.473700	62.013880	195.811600	.003363
-88.491920	62.343760	195.804500	.005949
-86.465330	62.645610	195.798600	.009316
-84.395630	62.918240	195.808800	.003545
-82.285480	63.160470	195.824800	.016797
-80.138370	63.371240	195.805400	.042639
-77.958560	63.549540	195.721300	.087887
-75.750930	63.694510	195.618600	.132682
-73.520860	63.805420	195.474000	.194160
-71.274150	63.881700	195.242000	.309324
-69.016910	63.922960	195.093700	.382590
-66.755400	63.928990	195.375300	.244081
-64.495980	63.899750	195.636600	.103759
-62.244980	63.835380	195.753100	.039367
-60.008560	63.736250	195.796900	.014679
-57.792640	63.602840	195.812500	.005417
-55.602750	63.435840	195.817700	.001903
-53.443960	63.236060	195.819400	.000637
-51.320750	63.004450	195.819800	.000192
-49.236890	62.742080	195.819900	.000055
-47.195330	62.450080	195.819900	.000015
-45.198120	62.129630	195.820000	.000056
-43.246250	61.781950	195.819900	.000015
-41.339580	61.408210	195.819900	.000015
-39.476710	61.009530	195.819900	.000015
-37.654880	60.586900	195.820000	.000040
-35.869840	60.141170	195.819900	.000015
-34.115770	59.672970	195.819900	.000015
-32.385130	59.182660	195.819900	.000015
-30.668580	58.670220	195.820000	.000000
-28.954870	58.135290	195.820000	.000000
-106.073800	59.266680	195.820000	.000000
-104.297500	59.807790	195.820000	.000000
-102.518800	60.325180	195.820000	.000034
-100.725000	60.819250	195.819900	.000057
-98.905530	61.289990	195.819800	.000123
-97.052100	61.737010	195.819500	.000307
-95.158340	62.159620	195.818800	.000675
-93.219700	62.556880	195.817100	.001297
-91.233350	62.927700	195.814000	.002310
-89.198040	63.270870	195.809900	.003892
-87.113920	63.585110	195.810200	.003957
-84.982480	63.869130	195.820300	.002789
-82.806390	64.121680	195.833600	.014776

-80.589290 64.341560 195.820700 .021590
-78.335770 64.527670 195.784900 .036963
-76.051160 64.679080 195.733700 .058592
-73.741460 64.794950 195.662300 .089311
-71.413090 64.874680 195.562800 .138592
-69.072890 64.917820 195.485200 .178818
-66.727910 64.924110 195.612500 .115687
-64.385280 64.893550 195.723800 .055201
-62.052090 64.826280 195.780700 .023668
-59.735260 64.722690 195.805400 .009680
-57.441380 64.583340 195.815100 .003769
-55.176610 64.408980 195.818500 .001385
-52.946510 64.200520 195.819500 .000472
-50.755950 63.959000 195.819900 .000148
-48.608900 63.685590 195.819900 .000044
-46.508400 63.381530 195.819900 .000015
-44.456330 63.048090 195.820000 .000026
-42.453320 62.686580 195.820000 .000000
-40.498620 62.298210 195.819900 .000027
-38.589950 61.884150 195.819900 .000015
-36.723370 61.445380 195.820000 .000000
-34.893150 60.982680 195.820000 .000000
-33.091610 60.496560 195.819900 .000027
-31.309040 59.987160 195.819900 .000015
-29.533510 59.454220 195.820000 .000000
-27.750760 58.896990 195.820000 .000000
-107.339200 60.027230 195.820000 .000000
-105.487000 60.591610 195.820000 .000000
-103.643800 61.130270 195.820000 .000026
-101.793500 61.644070 195.819900 .000062
-99.922550 62.133320 195.819900 .000114
-98.020040 62.597820 195.819700 .000243
-96.077510 63.037030 195.819200 .000497
-94.088710 63.450070 195.818100 .000893
-92.049460 63.835860 195.816600 .001358
-89.957580 64.193130 195.815200 .001774
-87.812570 64.520550 195.817400 .001092
-85.615560 64.816710 195.826200 .003970
-83.369120 65.080260 195.831800 .008240
-81.077050 65.309890 195.824900 .010384
-78.744270 65.504390 195.809500 .015490
-76.376630 65.662700 195.786800 .024228
-73.980770 65.783920 195.757200 .037226
-71.563890 65.867360 195.723400 .053722
-69.133670 65.912500 195.703900 .065261
-66.698060 65.919100 195.737900 .047091
-64.265090 65.887100 195.775600 .026551
-61.842790 65.816700 195.799700 .013119
-59.438900 65.708320 195.811900 .005943
-57.060850 65.562580 195.817200 .002480
-54.715470 65.380330 195.819100 .000945
-52.408880 65.162570 195.819700 .000326
-50.146370 64.910470 195.819900 .000114
-47.932120 64.625290 195.820000 .000041
-45.769150 64.308400 195.820000 .000050
-43.659080 63.961160 195.820000 .000000
-41.602020 63.584970 195.820000 .000000
-39.596350 63.181100 195.820000 .000000
-37.638600 62.750720 195.820000 .000000
-35.723270 62.294770 195.820000 .000000
-33.842640 61.813940 195.820000 .000000
-31.986660 61.308540 195.820000 .000000
-30.142740 60.778430 195.820000 .000000
-28.295590 60.222970 195.820000 .000000
-26.427080 59.640870 195.820000 .000000
-108.736700 60.768890 195.820000 .000000
-106.789100 61.359350 195.820000 .000000

-104.866900	61.921510	195.820000	.000015
-102.949500	62.456830	195.820000	.000054
-101.019200	62.966030	195.819900	.000101
-99.061880	63.449270	195.819700	.000210
-97.066180	63.906190	195.819400	.000397
-95.023590	64.336020	195.818700	.000658
-92.928180	64.737730	195.817900	.000861
-90.776390	65.110020	195.818000	.000755
-88.566850	65.451470	195.820400	.000563
-86.300130	65.760610	195.826600	.003281
-83.978550	66.035940	195.828700	.005117
-81.606060	66.276030	195.825100	.006347
-79.187900	66.479550	195.816300	.008691
-76.730480	66.645290	195.803800	.013398
-74.241170	66.772270	195.788400	.020301
-71.728080	66.859700	195.773400	.027735
-69.199870	66.907030	195.767100	.031108
-66.665550	66.913940	195.778500	.025105
-64.134220	66.880400	195.794900	.016066
-61.614990	66.806620	195.807400	.008903
-59.116610	66.693070	195.814600	.004402
-56.647430	66.540450	195.818000	.001960
-54.215080	66.349720	195.819300	.000785
-51.826330	66.121990	195.819800	.000290
-49.486860	65.858530	195.819900	.000099
-47.201050	65.560780	195.820000	.000037
-44.971820	65.230190	195.820000	.000000
-42.800360	64.868270	195.820000	.000000
-40.685980	64.476460	195.820000	.000000
-38.625890	64.056100	195.820000	.000000
-36.614990	63.608330	195.820000	.000000
-34.645670	63.134020	195.820000	.000000
-32.707610	62.633670	195.820000	.000000
-30.787590	62.107330	195.820000	.000000
-28.869240	61.554470	195.820000	.000000
-26.932890	60.973880	195.820000	.000000
-24.955330	60.363580	195.820000	.000000

Q.06a

-89.453400	32.991660	206.277800	.023831
-88.345930	33.191160	206.276600	.026606
-87.228100	33.381660	206.271500	.039148
-86.100230	33.562890	206.263200	.060049
-84.962690	33.734530	206.251800	.087875
-83.815900	33.896310	206.237700	.120238

-82.660300	34.047950	206.222400	.153456
-81.496410	34.189190	206.208500	.184390
-80.324730	34.319790	206.196800	.214159
-79.145840	34.439500	206.180400	.250266
-77.960310	34.548100	206.146700	.304079
-76.768760	34.645400	206.089800	.376917
-75.571840	34.731200	206.031600	.443956
-74.370180	34.805370	206.011000	.462309
-73.164480	34.867740	206.049600	.412980
-71.955430	34.918200	206.122700	.313073
-70.743730	34.956650	206.193100	.201917
-69.530080	34.983020	206.240700	.112908
-68.315220	34.997260	206.265800	.055695
-67.099880	34.999340	206.277100	.024534
-65.884770	34.989250	206.281700	.009551
-64.670640	34.967020	206.283500	.003225
-63.458190	34.932690	206.284100	.000910
-62.248150	34.886320	206.284200	.000218
-61.041230	34.828000	206.284300	.000121
-59.838120	34.757850	206.284300	.000128
-58.639500	34.676010	206.284300	.000128
-57.446040	34.582610	206.284300	.000125
-56.258390	34.477850	206.284300	.000122
-55.077160	34.361910	206.284300	.000122
-53.902950	34.235010	206.284300	.000122
-52.736340	34.097380	206.284300	.000122
-51.577860	33.949260	206.284300	.000122
-50.428010	33.790910	206.284300	.000122
-49.287250	33.622600	206.284300	.000122
-48.156040	33.444620	206.284300	.000122
-47.034740	33.257250	206.284300	.000122
-45.923690	33.060790	206.284300	.000122
-44.823220	32.855560	206.284300	.000122
-89.701210	33.918400	206.267500	.048072
-88.583520	34.125020	206.262600	.056097
-87.455040	34.322360	206.246400	.083756
-86.316120	34.510110	206.220500	.128900
-85.167100	34.687960	206.185400	.188954
-84.008420	34.855620	206.141700	.259246
-82.840550	35.012790	206.093300	.331500
-81.663990	35.159200	206.050800	.396635
-80.479300	35.294580	206.019600	.454934
-79.287040	35.418700	205.982600	.521856
-78.087840	35.531310	205.896400	.631050
-76.882320	35.632210	205.735600	.797747
-75.671150	35.721200	205.555000	.964931
-74.455030	35.798110	205.489000	1.007245
-73.234660	35.862810	205.602500	.898240
-72.010770	35.915150	205.822500	.673532
-70.784100	35.955040	206.028700	.424877
-69.555380	35.982390	206.165700	.229402
-68.325390	35.997170	206.236600	.108903
-67.094890	35.999320	206.266900	.046180
-65.864640	35.988860	206.278500	.017532
-64.635410	35.965800	206.282600	.005780
-63.407950	35.930180	206.283900	.001537
-62.183030	35.882080	206.284200	.000281
-60.961370	35.821590	206.284300	.000103
-59.743720	35.748840	206.284300	.000121
-58.530780	35.663950	206.284300	.000106
-57.323260	35.567100	206.284300	.000128
-56.121820	35.458470	206.284300	.000128
-54.927120	35.338260	206.284300	.000122
-53.739770	35.206700	206.284300	.000122
-52.560380	35.064020	206.284300	.000122
-51.389480	34.910500	206.284300	.000122
-50.227610	34.746390	206.284300	.000122

-49.075240	34.571990	206.284300	.000122
-47.932830	34.387580	206.284300	.000122
-46.800740	34.193490	206.284300	.000122
-45.679330	33.990000	206.284300	.000122
-44.568900	33.777440	206.284300	.000122
-89.960660	34.843320	206.224700	.122841
-88.832330	35.057210	206.202900	.148198
-87.692770	35.261540	206.138100	.223162
-86.542320	35.455950	206.035600	.343351
-85.381340	35.640140	205.899100	.502535
-84.210270	35.813810	205.727300	.691279
-83.029580	35.976620	205.535400	.888218
-81.839790	36.128310	205.369100	1.062551
-80.641480	36.268600	205.261800	1.209283
-79.435230	36.397220	205.160400	1.361458
-78.221690	36.513940	204.898400	1.638919
-77.001530	36.618520	204.324500	2.138715
-75.775430	36.710780	203.633900	2.663951
-74.544130	36.790520	203.359600	2.782683
-73.308360	36.857590	203.821300	2.437967
-72.068890	36.911860	204.638000	1.829107
-70.826490	36.953220	205.388700	1.136432
-69.581950	36.981590	205.874200	.599524
-68.336070	36.996900	206.122700	.275230
-67.089650	36.999130	206.228100	.112663
-65.843500	36.988290	206.266700	.041424
-64.598400	36.964370	206.279500	.013316
-63.355190	36.927440	206.283400	.003347
-62.114630	36.877570	206.284300	.000409
-60.877510	36.814860	206.284500	.000329
-59.644590	36.739430	206.284400	.000320
-58.416640	36.651430	206.284400	.000188
-57.194370	36.551040	206.284400	.000100
-55.978490	36.438440	206.284400	.000093
-54.769680	36.313860	206.284300	.000122
-53.568590	36.177530	206.284300	.000122
-52.375820	36.029710	206.284300	.000122
-51.191970	35.870660	206.284300	.000122
-50.017550	35.700660	206.284300	.000122
-48.853080	35.520030	206.284300	.000122
-47.698980	35.329070	206.284300	.000122
-46.555660	35.128100	206.284300	.000122
-45.423470	34.917440	206.284300	.000122
-44.302690	34.697410	206.284300	.000122
-90.232250	35.766320	206.086700	.290926
-89.092860	35.987640	206.000400	.361274
-87.941790	36.199090	205.765600	.549688
-86.779330	36.400320	205.399000	.844308
-85.605870	36.591000	204.914600	1.230822
-84.421870	36.770790	204.298000	1.697173
-83.227800	36.939400	203.595500	2.197370
-82.024180	37.096500	202.993600	2.641996
-80.811620	37.241800	202.651500	2.980790
-79.590740	37.375040	202.438600	3.285995
-78.362190	37.495960	201.726200	3.964778
-77.126670	37.604320	199.805100	5.415638
-75.884930	37.699920	197.153800	7.117556
-74.637700	37.782550	196.058500	7.150834
-73.385770	37.852060	197.834100	6.303560
-72.129940	37.908310	200.902500	4.603097
-70.871030	37.951180	203.436100	2.842423
-69.609860	37.980580	205.014900	1.459946
-68.347290	37.996460	205.794900	.648242
-67.084140	37.998780	206.118800	.254121
-65.821270	37.987530	206.235600	.089243
-64.559530	37.962740	206.272700	.026998
-63.299760	37.924460	206.283000	.005942

-62.042780	37.872770	206.285000	.000855
-60.789440	37.807780	206.285100	.001353
-59.540510	37.729610	206.284800	.001037
-58.296810	37.638420	206.284700	.000619
-57.059080	37.534390	206.284500	.000326
-55.828080	37.417740	206.284500	.000130
-54.604500	37.288680	206.284300	.000100
-53.389020	37.147460	206.284300	.000120
-52.182290	36.994370	206.284300	.000119
-50.984880	36.829660	206.284300	.000120
-49.797380	36.653650	206.284300	.000120
-48.620270	36.466650	206.284300	.000120
-47.454020	36.268990	206.284300	.000120
-46.299010	36.060990	206.284300	.000122
-45.155610	35.843010	206.284300	.000122
-44.024070	35.615370	206.284300	.000122
-90.516530	36.687310	205.706900	.617993
-89.365660	36.916220	205.415000	.788148
-88.202590	37.134950	204.667100	1.206891
-87.027650	37.343150	203.511000	1.837718
-85.841200	37.540460	202.009700	2.645129
-84.643710	37.726540	200.054600	3.648401
-83.435650	37.901050	197.746600	4.793105
-82.217600	38.063690	195.789900	5.755745
-80.990140	38.214140	194.706800	6.583802
-79.753940	38.352110	194.540100	6.927008
-78.509670	38.477340	192.876000	8.390687
-77.258070	38.589580	187.292900	12.069880
-75.999910	38.688600	177.652100	16.584680
-74.735980	38.774210	173.132900	16.344020
-73.467090	38.846230	178.561000	14.195520
-72.194090	38.904510	189.924100	10.743530
-70.917830	38.948930	198.274200	6.217498
-69.639210	38.979390	202.868300	3.113337
-68.359090	38.995840	205.019800	1.323682
-67.078360	38.998240	205.875000	.491790
-65.797930	38.986590	206.172900	.161799
-64.518690	38.960900	206.264100	.044263
-63.241520	38.921240	206.286400	.007381
-61.967320	38.867690	206.289000	.004622
-60.696920	38.800350	206.287500	.004664
-59.431210	38.719360	206.286200	.003244
-58.170970	38.624900	206.285500	.001965
-56.917050	38.517150	206.285100	.001008
-55.670200	38.396340	206.284600	.000409
-54.431160	38.262690	206.284400	.000136
-53.200640	38.116470	206.284300	.000102
-51.979310	37.957960	206.284200	.000111
-50.767770	37.787460	206.284300	.000102
-49.566610	37.605300	206.284300	.000115
-48.376340	37.411790	206.284300	.000116
-47.197410	37.207270	206.284300	.000116
-46.030250	36.992090	206.284300	.000117
-44.875180	36.766620	206.284300	.000119
-43.732470	36.531200	206.284300	.000122
-90.814130	37.606180	204.837300	1.168318
-89.651300	37.842850	203.994100	1.519112
-88.475770	38.069030	201.910400	2.325122
-87.287830	38.284350	198.705500	3.494927
-86.087840	38.488450	194.687500	4.838161
-84.876290	38.680970	189.338500	6.509792
-83.653650	38.861550	182.776400	8.599990
-82.420500	39.029860	176.916900	10.712590
-81.177470	39.185590	173.622900	12.281340
-79.925230	39.328410	174.076100	12.866060
-78.664510	39.458070	172.742200	13.974930
-77.396060	39.574290	160.938000	19.325680

-76.120680	39.676830	136.806600	25.720650
-74.839220	39.765490	117.602100	26.203980
-73.552520	39.840080	127.590300	25.653850
-72.261480	39.900440	159.926600	21.346860
-70.967010	39.946460	186.065600	12.144500
-69.670030	39.978020	198.473500	5.505417
-68.371470	39.995060	203.581000	2.212512
-67.072270	39.997550	205.476200	.761361
-65.773400	39.985470	206.094700	.234852
-64.475770	39.958870	206.273700	.051900
-63.180340	39.917780	206.308000	.014137
-61.888020	39.862300	206.304600	.017607
-60.599740	39.792560	206.296200	.013883
-59.316390	39.708680	206.290700	.009153
-58.038840	39.610860	206.288000	.005568
-56.767930	39.499290	206.286300	.003088
-55.504470	39.374200	206.285000	.001104
-54.249260	39.235840	206.284400	.000339
-53.003000	39.084480	206.284200	.000120
-51.766410	38.920440	206.284200	.000086
-50.540120	38.744010	206.284200	.000094
-49.324710	38.555530	206.284300	.000087
-48.120720	38.355340	206.284300	.000101
-46.928620	38.143810	206.284300	.000120
-45.748810	37.921300	206.284300	.000111
-44.581620	37.688170	206.284300	.000115
-43.427300	37.444810	206.284300	.000120
-91.125630	38.522790	203.226400	1.942261
-89.950410	38.767390	201.165100	2.566821
-88.761920	39.001200	196.054900	3.973807
-87.560450	39.223820	188.070500	5.827888
-86.346370	39.434880	178.497700	7.599985
-85.120160	39.633990	166.601200	9.007165
-83.882300	39.820790	152.485800	10.965350
-82.633390	39.994930	138.147500	13.543520
-81.374080	40.156070	127.555700	18.443920
-80.105060	40.303890	128.564300	20.161080
-78.827100	40.438090	137.402000	16.214020
-77.540980	40.558400	128.970200	15.779680
-76.247540	40.664560	87.409030	20.011640
-74.947690	40.756360	55.168140	22.754170
-73.642300	40.833600	56.734130	31.591960
-72.332310	40.896110	107.283000	30.548300
-71.018690	40.943760	164.642000	18.115220
-69.702430	40.976440	191.903700	7.900040
-68.384490	40.994090	201.937200	2.841506
-67.065880	40.996670	205.196400	.900347
-65.747600	40.984170	206.106300	.262218
-64.430650	40.956610	206.373300	.053534
-63.116020	40.914060	206.390000	.057710
-61.804690	40.856610	206.354600	.051462
-60.497620	40.784390	206.323000	.036297
-59.195770	40.697540	206.304200	.023114
-57.900040	40.596260	206.295500	.015150
-56.611330	40.480750	206.289600	.007522
-55.330490	40.351270	206.285300	.002824
-54.058330	40.208070	206.284300	.000793
-52.795620	40.051450	206.284200	.000276
-51.543080	39.881710	206.284200	.000133
-50.301390	39.699200	206.284200	.000101
-49.071130	39.504250	206.284200	.000092
-47.852850	39.297230	206.284300	.000086
-46.647030	39.078520	206.284300	.000097
-45.454070	38.848490	206.284300	.000110
-44.274280	38.607530	206.284300	.000123
-43.107920	38.356030	206.284300	.000120
-91.451740	39.437030	200.942300	2.838642

-90.263630	39.689750	196.755600	3.840966
-89.061680	39.931370	186.114100	6.024290
-87.846150	40.161480	168.575400	8.861831
-86.617400	40.379670	144.336200	11.500550
-85.375900	40.585540	116.369400	12.956920
-84.122160	40.778730	102.657000	8.769627
-82.856800	40.958850	92.281880	8.350080
-81.580460	41.125550	66.207600	15.877870
-80.293880	41.278500	65.857410	19.490780
-78.997860	41.417370	92.260600	12.101520
-77.693230	41.541890	107.368700	7.269492
-76.380870	41.651780	65.222950	9.642857
-75.061690	41.746810	30.468340	10.844780
-73.736660	41.826770	39.914390	18.353650
-72.406780	41.891490	88.149990	26.291280
-71.073040	41.940830	149.658400	19.080700
-69.736500	41.974670	187.852500	8.171318
-68.398190	41.992950	201.346400	3.138765
-67.059160	41.995620	205.413800	1.048435
-65.720480	41.982670	206.438300	.303019
-64.383220	41.954140	206.717700	.142075
-63.048400	41.910080	206.623800	.167241
-61.717090	41.850600	206.489500	.129227
-60.390290	41.775820	206.394300	.084268
-59.069010	41.685920	206.341000	.054802
-57.754210	41.581080	206.315300	.032730
-56.446830	41.461530	206.291300	.014174
-55.147770	41.327530	206.284700	.004627
-53.857880	41.179360	206.283400	.001842
-52.577970	41.017320	206.283900	.000795
-51.308780	40.841740	206.284200	.000345
-50.051000	40.652980	206.284200	.000145
-48.805250	40.451390	206.284200	.000109
-47.572090	40.237360	206.284200	.000092
-46.352010	40.011290	206.284200	.000096
-45.145370	39.773560	206.284300	.000092
-43.952510	39.524580	206.284300	.000106
-42.773630	39.264750	206.284300	.000120
-91.793200	40.348750	198.778900	3.671280
-90.591710	40.609810	192.289100	4.973202
-89.375760	40.859430	174.883900	8.093601
-88.145600	41.097210	141.676900	12.814150
-86.901570	41.322720	93.046420	15.295420
-85.644150	41.535550	50.425710	13.488770
-84.373860	41.735290	49.697150	15.805240
-83.091290	41.921560	49.309060	11.528070
-81.797160	42.093980	44.884530	6.552138
-80.492230	42.252200	47.210790	9.383125
-79.177290	42.395880	71.956470	6.209569
-77.853240	42.524730	74.409210	10.002500
-76.521000	42.638450	39.529790	18.504870
-75.181550	42.736820	28.541150	14.933560
-73.835900	42.819590	63.401840	17.602420
-72.485100	42.886590	109.267200	18.070130
-71.130210	42.937670	159.774200	13.518770
-69.772340	42.972710	189.726300	7.067596
-68.412590	42.991630	202.017300	3.210627
-67.052090	42.994400	205.921800	1.349373
-65.691960	42.980990	207.042500	.376641
-64.333320	42.951450	207.457000	.371717
-62.977290	42.905830	207.169100	.428312
-61.624970	42.844260	206.800700	.287595
-60.277440	42.766850	206.561300	.182829
-58.935750	42.673790	206.422400	.104268
-57.600940	42.565280	206.324500	.049806
-56.273990	42.441570	206.294000	.020278
-54.955840	42.302930	206.280600	.008362

-53.647390	42.149640	206.282600	.003980
-52.349470	41.982030	206.284100	.002120
-51.062890	41.800450	206.284400	.001006
-49.788320	41.605260	206.284300	.000436
-48.526440	41.396860	206.284300	.000180
-47.277780	41.175640	206.284200	.000111
-46.042850	40.942000	206.284200	.000096
-44.822010	40.696380	206.284300	.000088
-43.615560	40.439180	206.284300	.000102
-42.423690	40.170830	206.284300	.000120
-92.150770	41.257820	197.801000	4.277126
-90.935390	41.527410	190.419100	5.763633
-89.704910	41.785250	170.672300	8.517200
-88.459540	42.030900	133.366400	12.092410
-87.199620	42.263930	76.697170	13.788830
-85.925610	42.483890	26.602360	11.680910
-84.638040	42.690380	23.157740	10.267500
-83.337520	42.882970	33.207460	12.423700
-82.024790	43.061280	50.700510	7.065719
-80.700610	43.224930	95.384280	7.711587
-79.365870	43.373580	100.789900	10.273730
-78.021450	43.506890	83.172710	10.619200
-76.668370	43.624570	34.017210	7.039742
-75.307630	43.726360	43.679030	10.365560
-73.940300	43.812040	86.047080	9.868703
-72.567500	43.881390	131.709200	13.345250
-71.190370	43.934270	169.316300	11.275050
-69.810050	43.970550	192.457000	7.435434
-68.427750	43.990140	205.372500	2.829499
-67.044650	43.993000	208.409100	.770921
-65.661930	43.979120	208.735500	.583190
-64.280810	43.948540	208.783500	.949291
-62.902460	43.901310	208.296400	.973258
-61.528050	43.837570	207.456500	.655219
-60.158730	43.757450	206.878600	.345242
-58.795620	43.661140	206.525900	.173809
-57.439790	43.548850	206.373500	.083671
-56.092310	43.420850	206.293600	.036861
-54.754150	43.277410	206.290900	.017295
-53.426270	43.118850	206.290000	.010954
-52.109530	42.945500	206.287600	.005695
-50.804760	42.757750	206.285800	.002694
-49.512690	42.555960	206.284900	.001150
-48.233980	42.340550	206.284400	.000459
-46.969190	42.111940	206.284300	.000166
-45.718810	41.870550	206.284300	.000114
-44.483210	41.616830	206.284300	.000112
-43.262650	41.351200	206.284300	.000118
-42.057300	41.074100	206.284300	.000122
-92.525320	42.164070	197.989000	4.630341
-91.295520	42.442420	190.603500	6.513132
-90.049940	42.708690	170.903000	10.008830
-88.788760	42.962440	134.751500	13.330880
-87.512310	43.203190	82.744450	17.252230
-86.221000	43.430500	38.049220	10.925590
-84.915410	43.643920	24.154330	6.177852
-83.596150	43.843030	24.606100	14.504010
-82.263960	44.027400	70.371930	13.906790
-80.919660	44.196660	118.278100	14.730660
-79.564150	44.350410	124.184100	13.235190
-78.198390	44.488330	90.245640	7.648930
-76.823410	44.610100	46.836570	8.160777
-75.440290	44.715440	32.896830	7.694616
-74.050180	44.804110	77.336070	11.494370
-72.654240	44.875890	124.894200	17.634260
-71.253690	44.930630	167.328600	15.079340
-69.849760	44.968180	195.910300	7.045154

-68.443720	44.988460	209.379700	2.459223
-67.036810	44.991420	211.662300	1.574946
-65.630330	44.977050	210.730300	1.457310
-64.225530	44.945390	210.314200	1.676810
-62.823690	44.896510	209.737000	1.654642
-61.426040	44.830540	208.953700	1.397786
-60.033810	44.747610	207.598600	.773460
-58.648170	44.647940	206.863700	.343918
-57.270290	44.531750	206.504500	.155042
-55.901260	44.399320	206.397900	.083177
-54.542130	44.250930	206.345100	.050601
-53.193900	44.086940	206.315600	.027784
-51.857480	43.907680	206.298200	.013730
-50.533710	43.713560	206.289800	.006163
-49.223370	43.504980	206.286200	.002556
-47.927120	43.282370	206.284900	.000982
-46.645540	43.046150	206.284400	.000348
-45.379100	42.796800	206.284300	.000136
-44.128160	42.534760	206.284300	.000124
-42.892960	42.260480	206.284300	.000121
-41.673610	41.974410	206.284300	.000122
-92.917770	43.067330	198.896700	4.505179
-91.673000	43.354680	192.074500	6.416520
-90.411740	43.629630	173.477800	10.154570
-89.134110	43.891690	137.686300	14.817310
-87.840450	44.140390	86.690370	16.652010
-86.531140	44.375260	38.825490	14.370880
-85.206750	44.595830	28.366390	12.767170
-83.867900	44.801650	34.950810	12.065660
-82.515380	44.992280	49.959730	12.327480
-81.150000	45.167310	76.310870	18.415500
-79.772730	45.326340	79.545350	15.025340
-78.384560	45.469020	76.464720	9.925850
-76.986600	45.595010	58.414870	11.210590
-75.579970	45.704020	55.223450	14.797400
-74.165890	45.795780	87.417140	18.961410
-72.745610	45.870080	138.301800	18.044240
-71.320400	45.926740	177.233100	10.778070
-69.891600	45.965610	200.050900	4.481716
-68.460530	45.986610	209.941500	2.179762
-67.028560	45.989670	210.893900	1.363164
-65.597030	45.974800	210.133300	1.170996
-64.167300	45.942020	210.311000	1.577242
-62.740720	45.891430	210.691200	2.002607
-61.318600	45.823140	210.543300	2.057482
-59.902260	45.737310	209.149500	1.478358
-58.492950	45.634170	207.602500	.699466
-57.091900	45.513950	206.912700	.334505
-55.700260	45.376930	206.603700	.186871
-54.319140	45.223450	206.460700	.112527
-52.949590	45.053840	206.371400	.060442
-51.592580	44.868490	206.320900	.027815
-50.248970	44.667810	206.297900	.011970
-48.919560	44.452230	206.288800	.004811
-47.605040	44.222190	206.285600	.001794
-46.305980	43.978160	206.284700	.000616
-45.022840	43.720610	206.284400	.000202
-43.755960	43.450020	206.284300	.000137
-42.505550	43.166850	206.284300	.000122
-41.271660	42.871580	206.284300	.000122
-93.329110	43.967430	200.288800	3.961893
-92.068780	44.264030	194.700800	5.621720
-90.791210	44.547890	179.387900	8.591293
-89.496510	44.818510	149.721600	12.110550
-88.184940	45.075390	103.503400	17.049330
-86.856880	45.318050	56.753190	19.735130
-85.512890	45.545990	36.902260	13.055180

-84.153590 45.758730 40.983490 13.242990
-82.779790 45.955820 42.357370 17.900650
-81.392350 46.136820 31.931590 10.423490
-79.992260 46.301310 34.403770 9.773892
-78.580580 46.448910 71.173250 9.668839
-77.158460 46.579270 84.726490 13.224960
-75.727110 46.692070 102.590100 17.173920
-74.287820 46.787040 133.030000 19.046260
-72.841890 46.863950 168.096900 12.422230
-71.390720 46.922590 190.075300 5.450119
-69.935700 46.962830 201.213700 2.856027
-68.478260 46.984570 205.979500 2.077713
-67.019850 46.987740 206.257500 1.828421
-65.561920 46.972340 207.154200 1.118048
-64.105920 46.938410 208.185400 .579050
-62.653260 46.886040 209.386400 1.229297
-61.205390 46.815350 210.364300 1.805095
-59.763680 46.726520 209.982000 1.756378
-58.329470 46.619790 208.407100 1.068377
-56.904060 46.495390 207.360800 .546444
-55.488670 46.353640 206.901500 .333274
-54.084500 46.194880 206.661300 .214443
-52.692630 46.019470 206.469400 .110705
-51.314070 45.827840 206.358800 .048796
-49.949730 45.620390 206.310700 .019659
-48.600430 45.397590 206.292600 .007686
-47.266860 45.159900 206.286700 .002799
-45.949600 44.907820 206.284900 .000941
-44.649100 44.641840 206.284400 .000311
-43.365670 44.362450 206.284300 .000123
-42.099450 44.070140 206.284300 .000124
-40.850440 43.765410 206.284300 .000122
-93.760410 44.864160 201.933700 3.279408
-92.483920 45.170280 197.949800 4.656883
-91.189420 45.463320 186.991000 7.522672
-89.876960 45.742750 163.680500 11.830770
-88.546760 46.008070 123.830000 19.144390
-87.199170 46.258760 80.086810 25.990370
-85.834720 46.494300 62.395670 26.396230
-84.454070 46.714200 63.279550 18.513910
-83.058010 46.917960 55.546010 16.668530
-81.647480 47.105130 45.189680 16.340050
-80.223460 47.275260 51.161550 18.194150
-78.787090 47.427960 80.212270 22.363290
-77.339580 47.562840 120.496500 20.533120
-75.882230 47.679570 146.505400 19.488340
-74.416370 47.777870 173.001200 13.202590
-72.943440 47.857470 190.733400 6.397259
-71.464880 47.918190 198.261900 2.888785
-69.982220 47.959850 201.546300 2.354581
-68.496960 47.982350 201.220500 3.358264
-67.010670 47.985630 201.670000 3.731322
-65.524890 47.969690 203.387900 2.785497
-64.041170 47.934570 204.847800 1.764339
-62.561040 47.880340 206.205600 .898874
-61.086020 47.807170 208.202400 .678809
-59.617590 47.715230 209.085300 1.132048
-58.157170 47.604770 208.910500 1.212245
-56.706150 47.476050 207.833200 .780972
-55.265830 47.329400 207.287500 .509601
-53.837470 47.165180 206.936300 .342152
-52.422200 46.983780 206.606800 .177820
-51.021090 46.785640 206.407500 .071654
-49.635090 46.571200 206.324600 .027646
-48.265020 46.340950 206.296400 .010055
-46.911610 46.095370 206.287500 .003627
-45.575410 45.834990 206.285100 .001208

-44.256860	45.560320	206.284400	.000385
-42.956210	45.271870	206.284300	.000129
-41.673580	44.970160	206.284300	.000111
-40.408870	44.655690	206.284300	.000122
-94.212860	45.757310	203.443400	2.523203
-92.919560	46.073230	201.011400	3.565489
-91.607470	46.375730	194.599900	5.617723
-90.276550	46.664250	181.012400	9.304259
-88.926960	46.938270	156.213600	16.159740
-87.559010	47.197250	124.413100	25.159680
-86.173240	47.440650	99.992720	29.782480
-84.770290	47.667940	86.504170	26.692910
-83.350960	47.878600	81.770680	21.155680
-81.916200	48.072160	82.755510	23.311540
-80.467090	48.248140	97.332280	25.628120
-79.004780	48.406110	128.386300	26.333440
-77.530590	48.545680	155.097600	22.583960
-76.045850	48.666500	178.852100	13.407380
-74.552020	48.768250	192.478500	7.624382
-73.050600	48.850660	199.653400	3.903264
-71.543170	48.913520	202.389000	2.183100
-70.031330	48.956650	202.729300	1.982363
-68.516710	48.979950	201.836800	2.889360
-67.000980	48.983350	201.490500	3.342596
-65.485790	48.966840	202.298600	3.054411
-63.972820	48.930470	203.428900	2.454433
-62.463700	48.874330	204.501800	1.833609
-60.960050	48.798580	205.785900	1.119525
-59.463460	48.703410	207.443100	.647234
-57.975440	48.589080	208.072800	.750999
-56.497490	48.455880	208.040100	.795024
-55.030970	48.304140	207.511100	.613349
-53.577210	48.134270	207.084400	.411029
-52.137430	47.946660	206.686500	.219482
-50.712720	47.741790	206.419000	.080116
-49.304080	47.520120	206.326000	.026967
-47.912350	47.282170	206.296700	.010600
-46.538250	47.028450	206.287600	.003748
-45.182340	46.759510	206.285100	.001263
-43.845020	46.475880	206.284400	.000397
-42.526500	46.178100	206.284300	.000131
-41.226800	45.866700	206.284300	.000098
-39.945740	45.542200	206.284300	.000122
-94.687740	46.646660	204.546600	1.829214
-93.376940	46.972690	203.244800	2.483334
-92.046550	47.284930	200.119800	3.793943
-90.696440	47.582840	192.834100	6.588482
-89.326680	47.865840	179.153700	11.989120
-87.937550	48.133390	159.701700	19.788120
-86.529520	48.384910	139.619200	24.716420
-85.103260	48.619850	126.426400	23.220510
-83.659580	48.837660	124.147100	20.720400
-82.199460	49.037830	139.445100	17.920560
-80.724000	49.219880	155.877600	17.655620
-79.234450	49.383340	168.836500	18.188670
-77.732160	49.527770	183.613100	13.626420
-76.218590	49.652830	194.636300	8.267285
-74.695260	49.758160	200.151000	5.007966
-73.163800	49.843490	202.843200	2.942818
-71.625870	49.908580	204.190100	1.707757
-70.083210	49.953250	204.790300	1.366836
-68.537570	49.977380	204.499100	1.525517
-66.990740	49.980900	203.772800	1.827448
-65.444490	49.963800	203.498300	2.041455
-63.900620	49.926140	204.048300	1.870929
-62.360880	49.868010	204.930100	1.506706
-60.827010	49.789570	205.642700	1.083128

-59.300730	49.691040	206.464200	.579933
-57.783640	49.572690	207.280900	.463087
-56.277320	49.434830	207.304000	.490112
-54.783270	49.277820	207.139700	.432638
-53.302860	49.102080	206.883900	.326190
-51.837360	48.908040	206.584000	.172733
-50.387950	48.696200	206.397200	.069669
-48.955640	48.467040	206.316500	.023467
-47.541310	48.221130	206.293900	.008449
-46.145660	47.959000	206.286900	.003270
-44.769230	47.681210	206.284900	.001091
-43.412380	47.388330	206.284400	.000347
-42.075270	47.080920	206.284300	.000135
-40.757810	46.759540	206.284200	.000110
-39.459720	46.424710	206.284300	.000119
-95.186440	47.531940	205.236700	1.254316
-93.857410	47.868410	204.666500	1.572111
-92.508000	48.190720	203.408500	2.210701
-91.137910	48.498310	199.998800	3.921445
-89.747150	48.790600	193.147900	7.469365
-88.335950	49.067010	183.089200	12.436080
-86.904710	49.326930	172.142600	16.436020
-85.454100	49.569790	164.945900	15.891270
-83.984930	49.795020	170.517900	11.229260
-82.498220	50.002060	179.401300	9.928257
-80.995120	50.190400	186.785700	8.887333
-79.476920	50.359550	191.975400	9.274776
-77.945060	50.509060	198.046600	6.776375
-76.401090	50.638520	202.332800	4.247377
-74.846650	50.747580	204.190000	2.730402
-73.283450	50.835950	204.988400	1.787065
-71.713320	50.903350	205.384400	1.178282
-70.138080	50.949630	205.909100	.835081
-68.559640	50.974620	206.569500	.752495
-66.979900	50.978270	206.130600	.655150
-65.400800	50.960560	205.405400	.834657
-63.824260	50.921550	205.381900	.878032
-62.252170	50.861340	205.598500	.786917
-60.686400	50.780110	205.916500	.606948
-59.128770	50.678080	206.313700	.343140
-57.581030	50.555550	206.682900	.218212
-56.044860	50.412850	206.792200	.237217
-54.521820	50.250360	206.709300	.231570
-53.013420	50.068520	206.580000	.176941
-51.520960	49.867810	206.449200	.103146
-50.045680	49.648740	206.348600	.044036
-48.588630	49.411830	206.306300	.016616
-47.150700	49.157670	206.291000	.006787
-45.732590	48.886830	206.286100	.002443
-44.334810	48.599900	206.284700	.000849
-42.957660	48.297460	206.284300	.000265
-41.601190	47.980120	206.284300	.000123
-40.265250	47.648430	206.284300	.000107
-38.949390	47.302930	206.284300	.000122
-95.710510	48.412890	205.660300	.813863
-94.362470	48.760130	205.498600	.908897
-92.993230	49.092850	205.121900	1.123321
-91.602360	49.410460	203.693700	1.973059
-90.189750	49.712350	200.756500	3.733357
-88.755540	49.997940	196.703600	6.163793
-87.300090	50.266570	192.464300	7.882345
-85.824040	50.517650	190.629300	7.811915
-84.328210	50.750570	193.289700	5.922526
-82.813610	50.964740	197.197200	4.692594
-81.281460	51.159630	199.823500	4.391037
-79.733130	51.334700	201.851000	3.879391
-78.170130	51.489480	204.084700	2.837783

-76.594090	51.623530	205.472600	1.795758
-75.006800	51.736490	205.908800	1.289886
-73.410070	51.828010	206.091000	.984620
-71.805870	51.897850	206.232700	.748979
-70.196170	51.945790	206.638400	.646479
-68.582990	51.971680	207.316000	.658010
-66.968440	51.975460	207.731300	.628392
-65.354560	51.957120	207.501200	.466171
-63.743440	51.916690	207.042500	.258984
-62.137130	51.854320	206.644600	.204643
-60.537630	51.770170	206.342000	.187218
-58.946900	51.664510	206.394300	.125323
-57.366820	51.537620	206.498300	.107253
-55.799180	51.389870	206.501700	.116585
-54.245650	51.221680	206.457800	.100845
-52.707820	51.033510	206.404100	.076653
-51.187080	50.825860	206.354100	.049025
-49.684720	50.599280	206.315300	.023606
-48.201800	50.354330	206.295700	.010613
-46.739240	50.091630	206.287900	.004213
-45.297720	49.811770	206.285200	.001612
-43.877700	49.515370	206.284500	.000567
-42.479410	49.203060	206.284300	.000179
-41.102810	48.875440	206.284300	.000114
-39.747590	48.533100	206.284300	.000118
-38.413150	48.176570	206.284300	.000122
-96.261670	49.289190	205.974400	.475682
-94.893750	49.647600	205.926200	.495869
-93.503820	49.991080	205.893600	.499569
-92.091320	50.319060	205.314700	.889940
-90.655950	50.630910	204.253900	1.643194
-89.197760	50.925990	202.835100	2.571303
-87.717060	51.203660	201.543200	3.344771
-86.214420	51.463270	201.281600	3.305018
-84.690660	51.704190	202.488800	2.540265
-83.146820	51.925780	204.070700	2.127236
-81.584160	52.127480	205.020100	1.667364
-80.004110	52.308710	205.730700	1.401740
-78.408280	52.468990	206.418200	.943386
-76.798410	52.607840	206.728600	.691629
-75.176390	52.724850	206.677900	.646015
-73.544200	52.819690	206.533200	.688894
-71.903920	52.892050	206.681800	.617384
-70.257710	52.941730	206.809200	.633234
-68.607750	52.968570	207.339300	.685676
-66.956280	52.972490	207.886600	.645869
-65.305560	52.953480	208.071600	.640818
-63.657820	52.911580	207.808800	.596495
-62.015270	52.846950	207.166200	.357299
-60.380080	52.759750	206.661700	.155726
-58.754360	52.650280	206.461700	.087770
-57.140120	52.518850	206.400200	.066799
-55.539290	52.365840	206.373600	.055025
-53.953660	52.191710	206.350300	.045188
-52.384890	51.996940	206.328500	.033193
-50.834480	51.782070	206.310100	.021287
-49.303730	51.547700	206.296500	.011578
-47.793770	51.294400	206.289000	.005407
-46.305490	51.022820	206.285800	.002325
-44.839570	50.733620	206.284700	.000897
-43.396390	50.427420	206.284400	.000316
-41.976090	50.104880	206.284300	.000139
-40.578500	49.766630	206.284300	.000113
-39.203150	49.413260	206.284300	.000111
-37.849210	49.045330	206.284300	.000122
-96.841810	50.160530	206.140700	.244191
-95.453030	50.530490	206.165900	.236117

-94.041510	50.885130	206.148300	.215778
-92.606450	51.223850	205.958000	.375603
-91.147380	51.546020	205.620600	.658463
-89.664200	51.850960	205.188500	1.005324
-88.157130	52.138020	204.853400	1.269465
-86.626690	52.406500	204.913100	1.265071
-85.073680	52.655720	205.535600	1.021246
-83.499180	52.885050	206.378900	.695863
-81.904440	53.093850	206.806500	.506967
-80.290990	53.281520	207.005200	.358059
-78.660530	53.447530	207.094600	.350482
-77.014920	53.591380	207.029800	.379714
-75.356160	53.712650	206.676300	.440936
-73.686420	53.810940	206.168100	.665308
-72.007930	53.885960	206.108500	.683115
-70.322990	53.937460	206.268200	.627427
-68.634010	53.965280	206.539700	.630195
-66.943400	53.969350	207.162700	.481403
-65.253590	53.949630	207.489800	.356570
-63.567000	53.906200	207.365800	.389520
-61.886040	53.839190	206.967700	.282618
-60.213040	53.748820	206.586200	.141311
-58.550290	53.635360	206.412000	.067465
-56.899960	53.499180	206.346700	.039687
-55.264110	53.340690	206.321200	.027975
-53.644650	53.160350	206.308000	.020396
-52.043340	52.958710	206.299300	.014298
-50.461760	52.736320	206.292800	.009133
-48.901280	52.493820	206.288400	.005105
-47.363030	52.231850	206.286000	.002524
-45.847900	51.951060	206.284900	.001107
-44.356530	51.652150	206.284400	.000440
-42.889220	51.335790	206.284300	.000176
-41.445980	51.002650	206.284300	.000133
-40.026490	50.653380	206.284300	.000122
-38.630060	50.288590	206.284300	.000122
-37.255620	49.908830	206.284300	.000122
-97.453060	51.026530	206.234200	.104861
-96.042340	51.408490	206.248800	.090982
-94.608220	51.774700	206.241000	.090708
-93.149600	52.124570	206.187500	.148024
-91.665800	52.457450	206.091100	.247898
-90.156560	52.772640	205.976800	.363266
-88.621980	53.069450	205.907000	.449573
-87.062480	53.347160	205.975700	.454038
-85.478850	53.605050	206.240300	.338256
-83.872130	53.842430	206.618300	.217573
-82.243660	54.058630	206.791100	.156490
-80.595020	54.253030	206.816300	.184905
-78.928000	54.425040	206.819300	.245502
-77.244600	54.574130	206.759000	.272154
-75.546970	54.699840	206.424400	.309365
-73.837420	54.801750	205.854200	.626657
-72.118360	54.879540	205.576400	.811458
-70.392330	54.932960	205.848600	.671763
-68.661900	54.961820	206.115900	.576565
-66.929700	54.966030	206.571400	.395941
-65.198370	54.945580	206.849800	.175414
-63.470540	54.900540	206.806200	.164553
-61.748820	54.831050	206.610600	.133514
-60.035740	54.737340	206.444700	.075795
-58.333780	54.619720	206.354800	.041003
-56.645260	54.478570	206.315300	.022848
-54.972420	54.314330	206.299300	.013995
-53.317280	54.127510	206.292400	.009176
-51.681740	53.918690	206.289000	.006033
-50.067420	53.688470	206.286900	.003743

-48.475770	53.437500	206.285500	.002104
-46.907930	53.166490	206.284900	.001045
-45.364770	52.876130	206.284500	.000469
-43.846840	52.567130	206.284400	.000185
-42.354370	52.240220	206.284300	.000142
-40.887210	51.896080	206.284300	.000125
-39.444820	51.535370	206.284300	.000122
-38.026250	51.158730	206.284300	.000122
-36.630120	50.766700	206.284300	.000122
-98.097820	51.886770	206.270700	.036431
-96.663910	52.281200	206.274700	.031619
-95.206040	52.659450	206.272600	.034025
-93.722790	53.020900	206.258800	.053585
-92.213200	53.364910	206.234800	.085947
-90.676770	53.690770	206.208800	.121914
-89.113440	53.997730	206.201300	.147119
-87.523560	54.285050	206.237700	.144672
-85.907830	54.551970	206.329300	.112109
-84.267300	54.797760	206.441700	.072586
-82.603340	55.021710	206.488900	.074875
-80.917590	55.223140	206.476700	.112263
-79.211960	55.401440	206.502000	.176156
-77.488560	55.556020	206.533500	.207726
-75.749730	55.686380	206.316000	.221995
-73.997920	55.792100	205.766100	.553282
-72.235800	55.872810	205.354900	.848615
-70.466090	55.928230	205.612200	.706938
-68.691570	55.958180	206.028800	.452042
-66.915130	55.962550	206.314900	.274555
-65.139640	55.941330	206.496400	.098687
-63.367970	55.894590	206.491200	.065664
-61.602940	55.822490	206.421000	.056910
-59.847310	55.725280	206.356000	.038486
-58.103760	55.603290	206.316700	.022010
-56.374820	55.456930	206.297800	.012050
-54.662870	55.286670	206.290000	.006804
-52.970100	55.093080	206.286900	.004048
-51.298500	54.876750	206.285600	.002459
-49.649800	54.638340	206.285000	.001446
-48.025460	54.378550	206.284700	.000793
-46.426660	54.098120	206.284400	.000397
-44.854210	53.797780	206.284300	.000193
-43.308580	53.478290	206.284300	.000142
-41.789850	53.140400	206.284300	.000128
-40.297680	52.784830	206.284300	.000122
-38.831280	52.412250	206.284300	.000122
-37.389370	52.023280	206.284300	.000122
-35.970190	51.618480	206.284300	.000122
-98.778840	52.740810	206.281100	.011626
-97.320270	53.148240	206.282200	.010086
-95.837370	53.539010	206.281600	.011444
-94.328290	53.912530	206.278300	.017615
-92.791720	54.268120	206.272900	.027343
-91.226900	54.605080	206.268200	.037617
-89.633560	54.922620	206.269700	.044100
-88.011890	55.219970	206.284000	.042939
-86.362530	55.496330	206.310900	.034553
-84.686490	55.750910	206.336200	.031539
-82.985150	55.982970	206.336400	.045567
-81.260250	56.191770	206.319800	.084804
-79.513790	56.376650	206.360600	.149209
-77.748020	56.536990	206.445700	.181788
-75.965460	56.672260	206.293800	.174567
-74.168790	56.781970	205.785900	.485261
-72.360860	56.865740	205.268900	.859192
-70.544630	56.923280	205.468000	.755108
-68.723180	56.954370	205.920200	.423797

-66.899620	56.958910	206.214200	.189361
-65.077100	56.936880	206.332100	.062984
-63.258730	56.888360	206.351100	.026569
-61.447630	56.813510	206.333400	.023743
-59.646790	56.712620	206.311400	.017284
-57.859090	56.586040	206.296700	.010424
-56.087300	56.434210	206.289300	.005740
-54.333970	56.257640	206.286300	.003090
-52.601470	56.056940	206.285100	.001691
-50.891880	55.832740	206.284700	.000947
-49.207030	55.585770	206.284500	.000519
-47.548420	55.316760	206.284400	.000273
-45.917200	55.026500	206.284300	.000147
-44.314130	54.715760	206.284300	.000137
-42.739580	54.385350	206.284300	.000122
-41.193420	54.036030	206.284300	.000122
-39.675070	53.668560	206.284300	.000122
-38.183400	53.283620	206.284300	.000122
-36.716770	52.881820	206.284300	.000122
-35.272900	52.463700	206.284300	.000122
-99.499240	53.588130	206.283700	.003469
-98.014280	54.009130	206.283900	.002980
-96.504860	54.412960	206.283800	.003447
-94.968610	54.799050	206.283000	.005197
-93.403790	55.166730	206.281900	.007901
-91.809290	55.515250	206.281100	.010631
-90.184560	55.843850	206.282000	.012248
-88.529620	56.151670	206.285500	.012177
-86.844990	56.437890	206.289500	.012210
-85.131650	56.701690	206.287300	.018141
-83.390960	56.942230	206.271000	.036346
-81.624720	57.158780	206.258000	.075100
-79.835050	57.350580	206.312400	.136111
-78.024370	57.516980	206.416300	.169298
-76.195360	57.657400	206.288900	.151210
-74.350940	57.771310	205.843200	.419976
-72.494220	57.858320	205.346300	.805767
-70.628410	57.918090	205.342200	.819286
-68.756890	57.950390	205.811600	.464796
-66.883060	57.955100	206.123700	.188050
-65.010370	57.932220	206.250300	.065061
-63.142240	57.881810	206.290100	.017861
-61.282040	57.804080	206.295800	.006900
-59.433060	57.699310	206.292000	.005741
-57.598450	57.567890	206.288100	.003968
-55.781200	57.410300	206.285800	.002320
-53.984060	57.227100	206.284900	.001240
-52.209560	57.018940	206.284500	.000646
-50.459930	56.786500	206.284400	.000330
-48.737060	56.530560	206.284300	.000182
-47.042490	56.251900	206.284300	.000118
-45.377330	55.951360	206.284300	.000126
-43.742270	55.629770	206.284300	.000121
-42.137470	55.287960	206.284300	.000120
-40.562610	54.926740	206.284300	.000120
-39.016750	54.546860	206.284300	.000120
-37.498410	54.149020	206.284300	.000121
-36.005400	53.733830	206.284300	.000122
-34.534880	53.301770	206.284300	.000122
-100.262700	54.428120	206.284200	.000981
-98.749250	54.863350	206.284200	.000822
-97.211560	55.280850	206.284200	.000935
-95.646590	55.680060	206.284000	.001393
-94.052090	56.060360	206.283800	.002099
-92.426510	56.420970	206.283500	.002891
-90.768940	56.761100	206.283300	.003532
-89.079190	57.079890	206.282500	.004385

-87.357570	57.376450	206.278500	.007398
-85.605000	57.649890	206.266900	.015983
-83.822840	57.899360	206.243500	.035569
-82.012920	58.124030	206.231500	.072108
-80.177480	58.323120	206.302800	.124146
-78.319110	58.495910	206.404000	.160216
-76.440710	58.641750	206.290400	.135762
-74.545430	58.760110	205.970100	.338190
-72.636660	58.850530	205.463300	.727548
-70.717930	58.912650	205.281100	.852330
-68.792920	58.946230	205.660800	.554464
-66.865380	58.951130	206.039300	.234033
-64.939080	58.927340	206.200900	.085687
-63.017780	58.874950	206.261000	.028649
-61.105210	58.794160	206.279900	.007985
-59.204930	58.685300	206.284400	.001527
-57.320400	58.548780	206.284800	.000664
-55.454840	58.385130	206.284600	.000608
-53.611270	58.194950	206.284400	.000393
-51.792370	57.978940	206.284300	.000212
-50.000500	57.737840	206.284300	.000132
-48.237630	57.472490	206.284300	.000114
-46.505310	57.183730	206.284300	.000111
-44.804610	56.872430	206.284300	.000111
-43.136050	56.539500	206.284300	.000117
-41.499610	56.185780	206.284300	.000119
-39.894630	55.812120	206.284300	.000120
-38.319800	55.419290	206.284300	.000120
-36.773090	55.007980	206.284300	.000120
-35.251730	54.578750	206.284300	.000121
-33.752120	54.132060	206.284300	.000122
-101.073700	55.260130	206.284300	.000278
-99.529010	55.710320	206.284300	.000224
-97.960900	56.142140	206.284300	.000270
-96.365430	56.555100	206.284200	.000387
-94.739640	56.948590	206.284100	.000613
-93.081440	57.321860	206.283900	.000996
-91.389490	57.674070	206.283100	.001714
-89.663250	58.004340	206.280700	.003453
-87.902820	58.311730	206.273900	.007681
-86.109000	58.595320	206.258400	.017168
-84.283100	58.854170	206.230800	.037331
-82.427000	59.087400	206.220200	.070055
-80.543050	59.294160	206.306200	.111125
-78.634010	59.473690	206.407600	.148057
-76.703000	59.625280	206.334800	.115358
-74.753460	59.748340	206.065700	.270660
-72.789070	59.842370	205.623600	.610372
-70.813740	59.906970	205.242500	.857892
-68.831480	59.941910	205.509200	.656870
-66.846440	59.947010	205.945200	.302548
-64.862770	59.922250	206.159000	.115153
-62.884610	59.867760	206.242600	.041438
-60.916040	59.783740	206.272000	.014113
-58.961010	59.670550	206.281100	.004399
-57.023270	59.528640	206.283600	.001177
-55.106350	59.358580	206.284100	.000257
-53.213500	59.161040	206.284200	.000082
-51.347600	58.936760	206.284200	.000079
-49.511150	58.686560	206.284200	.000092
-47.706190	58.411320	206.284200	.000094
-45.934240	58.111950	206.284300	.000094
-44.196280	57.789400	206.284300	.000101
-42.492650	57.444580	206.284300	.000107
-40.823030	57.078400	206.284300	.000109
-39.186360	56.691730	206.284300	.000111
-37.580830	56.285340	206.284300	.000117

-36.003750 55.859910 206.284300 .000119
-34.451600 55.415950 206.284300 .000120
-32.919830 54.953830 206.284300 .000122
-101.937200 56.083360 206.284300 .000106
-100.358100 56.549350 206.284300 .000128
-98.756940 56.996240 206.284300 .000147
-97.128790 57.423640 206.284300 .000172
-95.469860 57.830970 206.284200 .000309
-93.777320 58.217490 206.283900 .000675
-92.049300 58.582370 206.282900 .001560
-90.284800 58.924680 206.279900 .003662
-88.483640 59.243460 206.271900 .008260
-86.646400 59.537720 206.254700 .017993
-84.774360 59.806450 206.224800 .037933
-82.869400 60.048700 206.216400 .064675
-80.933970 60.263580 206.316200 .089724
-78.971000 60.450230 206.421200 .116489
-76.983890 60.607900 206.345800 .087512
-74.976370 60.735940 206.146100 .199521
-72.952470 60.833800 205.749300 .485505
-70.916480 60.901050 205.303800 .797062
-68.872850 60.937420 205.394200 .726355
-66.826130 60.942730 205.839300 .380694
-64.780930 60.916960 206.118200 .147620
-62.741830 60.860230 206.227700 .053025
-60.713310 60.772790 206.266700 .018667
-58.699720 60.654990 206.279400 .006349
-56.705200 60.507380 206.283100 .002056
-54.733590 60.330550 206.284000 .000615
-52.788400 60.125220 206.284200 .000190
-50.872730 59.892220 206.284200 .000085
-48.989200 59.632420 206.284200 .000083
-47.139900 59.346780 206.284200 .000089
-45.326320 59.036270 206.284200 .000090
-43.549270 58.701890 206.284300 .000090
-41.808860 58.344600 206.284300 .000096
-40.104370 57.965370 206.284300 .000106
-38.434220 57.565060 206.284300 .000122
-36.795950 57.144450 206.284300 .000111
-35.186060 56.704150 206.284300 .000117
-33.600030 56.244660 206.284300 .000119
-32.032190 55.766200 206.284300 .000122
-102.859900 56.896880 206.284300 .000120
-101.242000 57.379640 206.284300 .000120
-99.604390 57.842470 206.284300 .000120
-97.940930 58.285070 206.284300 .000138
-96.246660 58.706940 206.284200 .000260
-94.517820 59.107400 206.283900 .000631
-92.751850 59.485580 206.282800 .001575
-90.947170 59.840560 206.279500 .003731
-89.103220 60.171320 206.271100 .008283
-87.220290 60.476800 206.253400 .017821
-85.299550 60.755960 206.223000 .036423
-83.342830 61.007760 206.217900 .052984
-81.352710 61.231220 206.313300 .047485
-79.332320 61.425420 206.390100 .058285
-77.285290 61.589540 206.298400 .064083
-75.215710 61.722870 206.127900 .154822
-73.128000 61.824790 205.842500 .359231
-71.026890 61.894870 205.426500 .675081
-68.917300 61.932760 205.326900 .741228
-66.804310 61.938290 205.751800 .440116
-64.692990 61.911450 206.088000 .170551
-62.588420 61.852340 206.218000 .060314
-60.495590 61.761240 206.263600 .021039
-58.419300 61.638580 206.278400 .007216
-56.364090 61.484900 206.282700 .002395

-54.334160	61.300890	206.283900	.000762
-52.333320	61.087320	206.284100	.000248
-50.364880	60.845090	206.284200	.000109
-48.431600	60.575160	206.284200	.000086
-46.535590	60.278550	206.284200	.000096
-44.678250	59.956310	206.284200	.000096
-42.860180	59.609490	206.284200	.000100
-41.081100	59.239120	206.284300	.000099
-39.339830	58.846180	206.284300	.000107
-37.634110	58.431540	206.284300	.000109
-35.960610	57.995940	206.284300	.000111
-34.314830	57.539980	206.284300	.000117
-32.690990	57.064010	206.284300	.000119
-31.082000	56.568130	206.284300	.000122
-103.849600	57.699570	206.284300	.000121
-102.187300	58.200280	206.284300	.000119
-100.509000	58.680030	206.284300	.000115
-98.806840	59.138710	206.284300	.000127
-97.074520	59.575940	206.284200	.000240
-95.307120	59.991050	206.283900	.000597
-93.501110	60.383240	206.282700	.001489
-91.654170	60.751550	206.279400	.003506
-89.765210	61.094930	206.271300	.007767
-87.834170	61.412270	206.254200	.016563
-85.861950	61.702450	206.227300	.032251
-83.850420	61.964360	206.228600	.036323
-81.802150	62.196920	206.307500	.016054
-79.720490	62.399150	206.323000	.046360
-77.609360	62.570130	206.220700	.105994
-75.473210	62.709080	206.080900	.156687
-73.316960	62.815350	205.900500	.267147
-71.145790	62.888430	205.563200	.516284
-68.965200	62.927950	205.407400	.635322
-66.780790	62.933720	205.745700	.419626
-64.598260	62.905720	206.087000	.167421
-62.423240	62.844070	206.218700	.058122
-60.261290	62.749080	206.263900	.020233
-58.117710	62.621230	206.278500	.006932
-55.997520	62.461110	206.282700	.002323
-53.905320	62.269450	206.283900	.000744
-51.845240	62.047150	206.284200	.000245
-49.820820	61.795150	206.284200	.000102
-47.834930	61.514500	206.284200	.000087
-45.889670	61.206310	206.284200	.000094
-43.986290	60.871700	206.284300	.000094
-42.125070	60.511780	206.284300	.000104
-40.305270	60.127630	206.284300	.000122
-38.525020	59.720250	206.284300	.000111
-36.781200	59.290500	206.284300	.000117
-35.069400	58.839090	206.284300	.000119
-33.383770	58.366500	206.284300	.000120
-31.716990	57.872960	206.284300	.000120
-30.060130	57.358350	206.284300	.000122
-104.916500	58.490070	206.284300	.000122
-103.202300	59.010120	206.284300	.000121
-101.477600	59.507980	206.284300	.000116
-99.732440	59.983750	206.284300	.000117
-97.958720	60.437220	206.284200	.000220
-96.150010	60.867820	206.283900	.000529
-94.301540	61.274790	206.282900	.001284
-92.410060	61.657170	206.279900	.002977
-90.473700	62.013880	206.272900	.006548
-88.491920	62.343760	206.258700	.013937
-86.465330	62.645610	206.242100	.024142
-84.395630	62.918240	206.266300	.010377
-82.285480	63.160470	206.319400	.024575
-80.138370	63.371240	206.322200	.047315

-77.958560 63.549540 206.237800 .072269
-75.750930 63.694510 206.147800 .110736
-73.520860 63.805420 206.029100 .170746
-71.274150 63.881700 205.837300 .306562
-69.016910 63.922960 205.679800 .418541
-66.755400 63.928990 205.920400 .276750
-64.495980 63.899750 206.137800 .118533
-62.244980 63.835380 206.234300 .044206
-60.008560 63.736250 206.268500 .015758
-57.792640 63.602840 206.279700 .005558
-55.602750 63.435840 206.283100 .001895
-53.443960 63.236060 206.284000 .000632
-51.320750 63.004450 206.284200 .000222
-49.236890 62.742080 206.284200 .000097
-47.195330 62.450080 206.284300 .000083
-45.198120 62.129630 206.284300 .000110
-43.246250 61.781950 206.284300 .000122
-41.339580 61.408210 206.284300 .000111
-39.476710 61.009530 206.284300 .000117
-37.654880 60.586900 206.284300 .000119
-35.869840 60.141170 206.284300 .000120
-34.115770 59.672970 206.284300 .000120
-32.385130 59.182660 206.284300 .000120
-30.668580 58.670220 206.284300 .000122
-28.954870 58.135290 206.284300 .000122
-106.073800 59.266680 206.284300 .000122
-104.297500 59.807790 206.284300 .000122
-102.518800 60.325180 206.284300 .000120
-100.725000 60.819250 206.284300 .000117
-98.905530 61.289990 206.284200 .000191
-97.052100 61.737010 206.284000 .000423
-95.158340 62.159620 206.283200 .000997
-93.219700 62.556880 206.281000 .002205
-91.233350 62.927700 206.276100 .004756
-89.198040 63.270870 206.266600 .009639
-87.113920 63.585110 206.265400 .010418
-84.982480 63.869130 206.288300 .003935
-82.806390 64.121680 206.326700 .027530
-80.589290 64.341560 206.312400 .023226
-78.335770 64.527670 206.275700 .027830
-76.051160 64.679080 206.231200 .045790
-73.741460 64.794950 206.172600 .078266
-71.413090 64.874680 206.089800 .135594
-69.072890 64.917820 206.019800 .191757
-66.727910 64.924110 206.120700 .127720
-64.385280 64.893550 206.211500 .061285
-62.052090 64.826280 206.256700 .025438
-59.735260 64.722690 206.274900 .010036
-57.441380 64.583340 206.281500 .003762
-55.176610 64.408980 206.283600 .001347
-52.946510 64.200520 206.284100 .000461
-50.755950 63.959000 206.284200 .000178
-48.608900 63.685590 206.284300 .000098
-46.508400 63.381530 206.284300 .000096
-44.456330 63.048090 206.284300 .000120
-42.453320 62.686580 206.284300 .000119
-40.498620 62.298210 206.284300 .000117
-38.589950 61.884150 206.284300 .000119
-36.723370 61.445380 206.284300 .000122
-34.893150 60.982680 206.284300 .000120
-33.091610 60.496560 206.284300 .000120
-31.309040 59.987160 206.284300 .000120
-29.533510 59.454220 206.284300 .000122
-27.750760 58.896990 206.284300 .000122
-107.339200 60.027230 206.284300 .000122
-105.487000 60.591610 206.284300 .000122
-103.643800 61.130270 206.284300 .000120

-101.793500 61.644070 206.284300 .000118
-99.922550 62.133320 206.284200 .000149
-98.020040 62.597820 206.284100 .000324
-96.077510 63.037030 206.283500 .000710
-94.088710 63.450070 206.282300 .001444
-92.049460 63.835860 206.279700 .002660
-89.957580 64.193130 206.276500 .004277
-87.812570 64.520550 206.279100 .003141
-85.615560 64.816710 206.298900 .008393
-83.369120 65.080260 206.311000 .015283
-81.077050 65.309890 206.301700 .011710
-78.744270 65.504390 206.286100 .010354
-76.376630 65.662700 206.267400 .017060
-73.980770 65.783920 206.244200 .031086
-71.563890 65.867360 206.216300 .051088
-69.133670 65.912500 206.198600 .067367
-66.698060 65.919100 206.224200 .050044
-64.265090 65.887100 206.253500 .027809
-61.842790 65.816700 206.271300 .013361
-59.438900 65.708320 206.279600 .005845
-57.060850 65.562580 206.282900 .002373
-54.715470 65.380330 206.283900 .000913
-52.408880 65.162570 206.284200 .000342
-50.146370 64.910470 206.284300 .000146
-47.932120 64.625290 206.284300 .000118
-45.769150 64.308400 206.284300 .000114
-43.659080 63.961160 206.284300 .000120
-41.602020 63.584970 206.284300 .000122
-39.596350 63.181100 206.284300 .000122
-37.638600 62.750720 206.284300 .000120
-35.723270 62.294770 206.284300 .000122
-33.842640 61.813940 206.284300 .000122
-31.986660 61.308540 206.284300 .000122
-30.142740 60.778430 206.284300 .000122
-28.295590 60.222970 206.284300 .000122
-26.427080 59.640870 206.284300 .000122
-108.736700 60.768890 206.284300 .000122
-106.789100 61.359350 206.284300 .000122
-104.866900 61.921510 206.284300 .000120
-102.949500 62.456830 206.284300 .000117
-101.019200 62.966030 206.284200 .000148
-99.061880 63.449270 206.284100 .000281
-97.066180 63.906190 206.283800 .000569
-95.023590 64.336020 206.283000 .001029
-92.928180 64.737730 206.281800 .001568
-90.776390 65.110020 206.281300 .001699
-88.566850 65.451470 206.284700 .000960
-86.300130 65.760610 206.296900 .006980
-83.978550 66.035940 206.300200 .008905
-81.606060 66.276030 206.295800 .007312
-79.187900 66.479550 206.287500 .005500
-76.730480 66.645290 206.277600 .008522
-74.241170 66.772270 206.266300 .016105
-71.728080 66.859700 206.254700 .025182
-69.199870 66.907030 206.248800 .030490
-66.665550 66.913940 206.256900 .025127
-64.134220 66.880400 206.268500 .015957
-61.614990 66.806620 206.277000 .008650
-59.116610 66.693070 206.281400 .004164
-56.647430 66.540450 206.283400 .001837
-54.215080 66.349720 206.284000 .000738
-51.826330 66.121990 206.284200 .000286
-49.486860 65.858530 206.284300 .000150
-47.201050 65.560780 206.284300 .000119
-44.971820 65.230190 206.284300 .000120
-42.800360 64.868270 206.284300 .000122
-40.685980 64.476460 206.284300 .000122

-38.625890 64.056100 206.284300 .000122
-36.614990 63.608330 206.284300 .000122
-34.645670 63.134020 206.284300 .000122
-32.707610 62.633670 206.284300 .000122
-30.787590 62.107330 206.284300 .000122
-28.869240 61.554470 206.284300 .000122
-26.932890 60.973880 206.284300 .000122
-24.955330 60.363580 206.284300 .000122

Q.07a
-89.453400 32.991660 217.309100 .007127
-88.345930 33.191160 217.308500 .008095
-87.228100 33.381660 217.306000 .012765
-86.100230 33.562890 217.302000 .020887
-84.962690 33.734530 217.296200 .032615
-83.815900 33.896310 217.288100 .047858
-82.660300 34.047950 217.277700 .065259
-81.496410 34.189190 217.267700 .082533
-80.324730 34.319790 217.259100 .099651
-79.145840 34.439500 217.245500 .121644
-77.960310 34.548100 217.212800 .158031
-76.768760 34.645400 217.153900 .212188
-75.571840 34.731200 217.088300 .266985
-74.370180 34.805370 217.061000 .286655
-73.164480 34.867740 217.096800 .254105
-71.955430 34.918200 217.170500 .184298
-70.743730 34.956650 217.238000 .112685
-69.530080 34.983020 217.280300 .058846
-68.315220 34.997260 217.300100 .027156
-67.099880 34.999340 217.307600 .010972
-65.884770 34.989250 217.310200 .003860
-64.670640 34.967020 217.311000 .001135
-63.458190 34.932690 217.311200 .000258
-62.248150 34.886320 217.311200 .000036
-61.041230 34.828000 217.311200 .000031
-59.838120 34.757850 217.311200 .000031
-58.639500 34.676010 217.311200 .000031
-57.446040 34.582610 217.311200 .000031
-56.258390 34.477850 217.311200 .000031
-55.077160 34.361910 217.311200 .000031
-53.902950 34.235010 217.311200 .000031
-52.736340 34.097380 217.311200 .000031
-51.577860 33.949260 217.311200 .000031
-50.428010 33.790910 217.311200 .000031
-49.287250 33.622600 217.311200 .000031

-48.156040	33.444620	217.311200	.000031
-47.034740	33.257250	217.311200	.000031
-45.923690	33.060790	217.311200	.000031
-44.823220	32.855560	217.311200	.000031
-89.701210	33.918400	217.303400	.016839
-88.583520	34.125020	217.300300	.020349
-87.455040	34.322360	217.290300	.032859
-86.316120	34.510110	217.274500	.053979
-85.167100	34.687960	217.252800	.084062
-84.008420	34.855620	217.222500	.123099
-82.840550	35.012790	217.184600	.167147
-81.663990	35.159200	217.148400	.209798
-80.479300	35.294580	217.120800	.248900
-79.287040	35.418700	217.083300	.296386
-78.087840	35.531310	216.991900	.378282
-76.882320	35.632210	216.812900	.512752
-75.671150	35.721200	216.600900	.656691
-74.455030	35.798110	216.501300	.714565
-73.234660	35.862810	216.615500	.629801
-72.010770	35.915150	216.851800	.450083
-70.784100	35.955040	217.069900	.266577
-69.555380	35.982390	217.207100	.135812
-68.325390	35.997170	217.273700	.060083
-67.094890	35.999320	217.299600	.023733
-65.864640	35.988860	217.308200	.008196
-64.635410	35.965800	217.310600	.002376
-63.407950	35.930180	217.311300	.000517
-62.183030	35.882080	217.311300	.000110
-60.961370	35.821590	217.311200	.000048
-59.743720	35.748840	217.311200	.000079
-58.530780	35.663950	217.311200	.000066
-57.323260	35.567100	217.311200	.000056
-56.121820	35.458470	217.311200	.000036
-54.927120	35.338260	217.311200	.000036
-53.739770	35.206700	217.311200	.000031
-52.560380	35.064020	217.311200	.000031
-51.389480	34.910500	217.311200	.000031
-50.227610	34.746390	217.311200	.000031
-49.075240	34.571990	217.311200	.000031
-47.932830	34.387580	217.311200	.000031
-46.800740	34.193490	217.311200	.000031
-45.679330	33.990000	217.311200	.000031
-44.568900	33.777440	217.311200	.000031
-89.960660	34.843320	217.275200	.051468
-88.832330	35.057210	217.259500	.065143
-87.692770	35.261540	217.213300	.106762
-86.542320	35.455950	217.141700	.175011
-85.381340	35.640140	217.044100	.270741
-84.210270	35.813810	216.910800	.393103
-83.029580	35.976620	216.744200	.531386
-81.839790	36.128310	216.589700	.661031
-80.641480	36.268600	216.482600	.774627
-79.435230	36.397220	216.365700	.897199
-78.221690	36.513940	216.064100	1.126816
-77.001530	36.618520	215.408400	1.545708
-75.775430	36.710780	214.557800	2.048006
-74.544130	36.790520	214.137500	2.220236
-73.308360	36.857590	214.576200	1.966686
-72.068890	36.911860	215.532200	1.386700
-70.826490	36.953220	216.374000	.801993
-69.581950	36.981590	216.901200	.397172
-68.336070	36.996900	217.159600	.172722
-67.089650	36.999130	217.263800	.065988
-65.843500	36.988290	217.299000	.022396
-64.598400	36.964370	217.309300	.006333
-63.355190	36.927440	217.311500	.001325
-62.114630	36.877570	217.311600	.000384

-60.877510	36.814860	217.311300	.000237
-59.644590	36.739430	217.311100	.000152
-58.416640	36.651430	217.311100	.000145
-57.194370	36.551040	217.311100	.000107
-55.978490	36.438440	217.311200	.000103
-54.769680	36.313860	217.311200	.000064
-53.568590	36.177530	217.311200	.000059
-52.375820	36.029710	217.311200	.000036
-51.191970	35.870660	217.311200	.000031
-50.017550	35.700660	217.311200	.000031
-48.853080	35.520030	217.311200	.000031
-47.698980	35.329070	217.311200	.000031
-46.555660	35.128100	217.311200	.000031
-45.423470	34.917440	217.311200	.000031
-44.302690	34.697410	217.311200	.000031
-90.232250	35.766320	217.172500	.144203
-89.092860	35.987640	217.106000	.189811
-87.941790	36.199090	216.927000	.314618
-86.779330	36.400320	216.650000	.515162
-85.605870	36.591000	216.275800	.788979
-84.421870	36.770790	215.755900	1.140983
-83.227800	36.939400	215.101500	1.539638
-82.024180	37.096500	214.510500	1.901778
-80.811620	37.241800	214.151400	2.196940
-79.590740	37.375040	213.877900	2.455704
-78.362190	37.495960	213.016000	3.046639
-77.126670	37.604320	210.760700	4.341167
-75.884930	37.699920	207.428800	5.991061
-74.637700	37.782550	205.739400	6.432168
-73.385770	37.852060	207.491600	5.626765
-72.129940	37.908310	211.067700	3.990111
-70.871030	37.951180	214.139700	2.239968
-69.609860	37.980580	215.942100	1.065075
-68.347290	37.996460	216.804500	.446203
-67.084140	37.998780	217.151000	.165667
-65.821270	37.987530	217.270600	.053600
-64.559530	37.962740	217.306100	.014188
-63.299760	37.924460	217.313600	.002839
-62.042780	37.872770	217.313300	.001614
-60.789440	37.807780	217.311800	.001012
-59.540510	37.729610	217.310900	.000510
-58.296810	37.638420	217.310700	.000358
-57.059080	37.534390	217.310800	.000269
-55.828080	37.417740	217.311000	.000165
-54.604500	37.288680	217.311100	.000101
-53.389020	37.147460	217.311200	.000085
-52.182290	36.994370	217.311200	.000067
-50.984880	36.829660	217.311200	.000036
-49.797380	36.653650	217.311200	.000036
-48.620270	36.466650	217.311200	.000036
-47.454020	36.268990	217.311200	.000031
-46.299010	36.060990	217.311200	.000031
-45.155610	35.843010	217.311200	.000031
-44.024070	35.615370	217.311200	.000031
-90.516530	36.687310	216.873300	.354704
-89.365660	36.916220	216.643200	.483986
-88.202590	37.134950	216.057800	.813254
-87.027650	37.343150	215.152700	1.329198
-85.841200	37.540460	213.930800	2.013489
-84.643710	37.726540	212.177900	2.893883
-83.435650	37.901050	209.873000	3.938324
-82.217600	38.063690	207.854400	4.848437
-80.990140	38.214140	206.768200	5.529668
-79.753940	38.352110	206.427900	5.944345
-78.509670	38.477340	204.325100	7.213317
-77.258070	38.589580	197.600200	10.519880
-75.999910	38.688600	185.552000	15.272740

-74.735980	38.774210	179.199600	15.429680
-73.467090	38.846230	184.946800	13.909140
-72.194090	38.904510	197.742800	9.970533
-70.917830	38.948930	207.826700	5.549167
-69.639210	38.979390	213.439700	2.511240
-68.359090	38.995840	215.925100	.988057
-67.078360	38.998240	216.882900	.343886
-65.797930	38.986590	217.207200	.105340
-64.518690	38.960900	217.304300	.023706
-63.241520	38.921240	217.323200	.006217
-61.967320	38.867690	217.320300	.006140
-60.696920	38.800350	217.314500	.003843
-59.431210	38.719360	217.311000	.001924
-58.170970	38.624900	217.310100	.001091
-56.917050	38.517150	217.310400	.000657
-55.670200	38.396340	217.310700	.000350
-54.431160	38.262690	217.311000	.000169
-53.200640	38.116470	217.311200	.000110
-51.979310	37.957960	217.311200	.000072
-50.767770	37.787460	217.311200	.000067
-49.566610	37.605300	217.311200	.000056
-48.376340	37.411790	217.311200	.000036
-47.197410	37.207270	217.311200	.000036
-46.030250	36.992090	217.311200	.000036
-44.875180	36.766620	217.311200	.000031
-43.732470	36.531200	217.311200	.000031
-90.814130	37.606180	216.182200	.754631
-89.651300	37.842850	215.519000	1.068227
-88.475770	38.069030	213.875700	1.816506
-87.287830	38.284350	211.290200	2.943630
-86.087840	38.488450	207.928100	4.349231
-84.876290	38.680970	202.883700	6.247601
-83.653650	38.861550	195.779500	8.544269
-82.420500	39.029860	189.391300	10.597790
-81.177470	39.185590	186.203400	12.241770
-79.925230	39.328410	186.481700	12.634110
-78.664510	39.458070	184.310900	13.906590
-77.396060	39.574290	170.304400	19.459900
-76.120680	39.676830	140.909900	29.602280
-74.839220	39.765490	118.165200	26.572100
-73.552520	39.840080	127.286100	24.869210
-72.261480	39.900440	162.817000	20.787740
-70.967010	39.946460	192.774000	11.354780
-69.670030	39.978020	208.028300	4.945603
-68.371470	39.995060	214.260300	1.782050
-67.072270	39.997550	216.442600	.553762
-65.773400	39.985470	217.127400	.153513
-64.475770	39.958870	217.331500	.023873
-63.180340	39.917780	217.360400	.022473
-61.888020	39.862300	217.344400	.020368
-60.599740	39.792560	217.325400	.012826
-59.316390	39.708680	217.314200	.006998
-58.038840	39.610860	217.310600	.003928
-56.767930	39.499290	217.310500	.002162
-55.504470	39.374200	217.310900	.001017
-54.249260	39.235840	217.311100	.000424
-53.003000	39.084480	217.311200	.000159
-51.766410	38.920440	217.311200	.000075
-50.540120	38.744010	217.311200	.000068
-49.324710	38.555530	217.311200	.000072
-48.120720	38.355340	217.311200	.000082
-46.928620	38.143810	217.311200	.000067
-45.748810	37.921300	217.311200	.000056
-44.581620	37.688170	217.311200	.000031
-43.427300	37.444810	217.311200	.000031
-91.125630	38.522790	214.924400	1.379102
-89.950410	38.767390	213.308900	2.004519

-88.761920	39.001200	209.193500	3.374503
-87.560450	39.223820	202.477500	5.307835
-86.346370	39.434880	194.183800	7.705685
-85.120160	39.633990	182.543800	10.802250
-83.882300	39.820790	166.138400	14.272530
-82.633390	39.994930	149.404400	18.250240
-81.374080	40.156070	139.376400	23.016590
-80.105060	40.303890	141.221300	22.723590
-78.827100	40.438090	147.757900	19.849530
-77.540980	40.558400	140.186700	18.874390
-76.247540	40.664560	94.381600	24.738050
-74.947690	40.756360	61.406080	24.729990
-73.642300	40.833600	54.564960	28.607640
-72.332310	40.896110	101.530200	30.417200
-71.018690	40.943760	166.770600	18.284840
-69.702430	40.976440	199.779600	7.550771
-68.384490	40.994090	212.229900	2.492537
-67.065880	40.996670	216.098100	.678822
-65.747600	40.984170	217.128600	.166361
-64.430650	40.956610	217.496500	.055557
-63.116020	40.914060	217.481900	.078649
-61.804690	40.856610	217.416100	.059568
-60.497620	40.784390	217.361000	.038193
-59.195770	40.697540	217.329700	.023186
-57.900040	40.596260	217.317700	.014374
-56.611330	40.480750	217.315100	.008048
-55.330490	40.351270	217.313400	.003697
-54.058330	40.208070	217.312200	.001454
-52.795620	40.051450	217.311600	.000457
-51.543080	39.881710	217.311300	.000104
-50.301390	39.699200	217.311200	.000075
-49.071130	39.504250	217.311200	.000084
-47.852850	39.297230	217.311200	.000070
-46.647030	39.078520	217.311200	.000074
-45.454070	38.848490	217.311200	.000052
-44.274280	38.607530	217.311200	.000031
-43.107920	38.356030	217.311200	.000031
-91.451740	39.437030	213.183000	2.133430
-90.263630	39.689750	209.870200	3.086072
-89.061680	39.931370	201.061800	5.121767
-87.846150	40.161480	185.976500	8.426255
-86.617400	40.379670	164.435700	12.621720
-85.375900	40.585540	135.331200	18.877630
-84.122160	40.778730	116.024400	17.226390
-82.856800	40.958850	102.174600	18.175790
-81.580460	41.125550	79.708640	23.300490
-80.293880	41.278500	73.832040	28.145690
-78.997860	41.417370	98.804750	20.597250
-77.693230	41.541890	117.680200	15.521260
-76.380870	41.651780	81.633060	16.286400
-75.061690	41.746810	41.316170	18.245010
-73.736660	41.826770	42.753400	20.432950
-72.406780	41.891490	81.812230	29.563770
-71.073040	41.940830	148.816000	20.596850
-69.736500	41.974670	194.362000	8.668427
-68.398190	41.992950	211.058900	2.955813
-67.059160	41.995620	215.829500	.897479
-65.720480	41.982670	217.239900	.294536
-64.383220	41.954140	217.989100	.181773
-63.048400	41.910080	217.830400	.208975
-61.717090	41.850600	217.601000	.155863
-60.390290	41.775820	217.459700	.101262
-59.069010	41.685920	217.380200	.067524
-57.754210	41.581080	217.347000	.045519
-56.446830	41.461530	217.336200	.024670
-55.147770	41.327530	217.323500	.010969
-53.857880	41.179360	217.316300	.004002

-52.577970 41.017320 217.312700 .001130
-51.308780 40.841740 217.311500 .000324
-50.051000 40.652980 217.311200 .000180
-48.805250 40.451390 217.311200 .000090
-47.572090 40.237360 217.311200 .000059
-46.352010 40.011290 217.311200 .000045
-45.145370 39.773560 217.311200 .000031
-43.952510 39.524580 217.311200 .000031
-42.773630 39.264750 217.311200 .000031
-91.793200 40.348750 211.580500 2.771586
-90.591710 40.609810 206.486500 3.938670
-89.375760 40.859430 192.152500 6.790006
-88.145600 41.097210 162.928300 13.193220
-86.901570 41.322720 115.951600 23.241880
-85.644150 41.535550 72.717160 24.068150
-84.373860 41.735290 66.288060 11.630620
-83.091290 41.921560 57.686050 11.343630
-81.797160 42.093980 55.718540 8.575102
-80.492230 42.252200 58.686290 12.115120
-79.177290 42.395880 77.816770 7.252625
-77.853240 42.524730 81.573720 16.249220
-76.521000 42.638450 49.662260 15.544010
-75.181550 42.736820 41.454500 16.213790
-73.835900 42.819590 67.275630 12.700070
-72.485100 42.886590 113.666200 19.954170
-71.130210 42.937670 166.906500 14.612670
-69.772340 42.972710 199.059800 7.470848
-68.412590 42.991630 210.656100 3.654309
-67.052090 42.994400 214.720200 1.956703
-65.691960 42.980990 217.109500 .939613
-64.333320 42.951450 218.721600 .435746
-62.977290 42.905830 218.662900 .467315
-61.624970 42.844260 218.030300 .341081
-60.277440 42.766850 217.681100 .225857
-58.935750 42.673790 217.496400 .153989
-57.600940 42.565280 217.420600 .102501
-56.273990 42.441570 217.396900 .059744
-54.955840 42.302930 217.353300 .025816
-53.647390 42.149640 217.325000 .008245
-52.349470 41.982030 217.314400 .002219
-51.062890 41.800450 217.311300 .001258
-49.788320 41.605260 217.310900 .000732
-48.526440 41.396860 217.311100 .000308
-47.277780 41.175640 217.311200 .000113
-46.042850 40.942000 217.311200 .000056
-44.822010 40.696380 217.311200 .000041
-43.615560 40.439180 217.311200 .000031
-42.423690 40.170830 217.311200 .000031
-92.150770 41.257820 210.916600 3.180010
-90.935390 41.527410 205.190600 4.654397
-89.704910 41.785250 188.711400 7.668747
-88.459540 42.030900 154.325000 12.878880
-87.199620 42.263930 98.606000 20.132850
-85.925610 42.483890 45.207360 20.636740
-84.638040 42.690380 28.117580 13.504370
-83.337520 42.882970 39.652210 14.572290
-82.024790 43.061280 61.517800 13.223590
-80.700610 43.224930 115.022600 17.045990
-79.365870 43.373580 125.284600 18.012640
-78.021450 43.506890 108.028300 16.671710
-76.668370 43.624570 46.519200 8.709392
-75.307630 43.726360 52.013260 11.191490
-73.940300 43.812040 106.945000 19.430450
-72.567500 43.881390 147.432700 14.333680
-71.190370 43.934270 183.134600 11.429850
-69.810050 43.970550 201.469200 7.707364
-68.427750 43.990140 210.367400 4.351947

-67.044650	43.993000	214.483200	2.952787
-65.661930	43.979120	217.712200	.980284
-64.280810	43.948540	220.075000	.875282
-62.902460	43.901310	220.069700	.913374
-61.528050	43.837570	218.906300	.639475
-60.158730	43.757450	218.112800	.425776
-58.795620	43.661140	217.795700	.301665
-57.439790	43.548850	217.695100	.227363
-56.092310	43.420850	217.563300	.145963
-54.754150	43.277410	217.412900	.050032
-53.426270	43.118850	217.338500	.013414
-52.109530	42.945500	217.313800	.007265
-50.804760	42.757750	217.309200	.004729
-49.512690	42.555960	217.309600	.002420
-48.233980	42.340550	217.310600	.000963
-46.969190	42.111940	217.311000	.000303
-45.718810	41.870550	217.311200	.000102
-44.483210	41.616830	217.311200	.000056
-43.262650	41.351200	217.311200	.000041
-42.057300	41.074100	217.311200	.000031
-92.525320	42.164070	211.155600	3.244704
-91.295520	42.442420	205.500300	4.983549
-90.049940	42.708690	189.300800	8.667591
-88.788760	42.962440	156.509800	14.213880
-87.512310	43.203190	105.229200	20.546140
-86.221000	43.430500	51.709390	22.119410
-84.915410	43.643920	29.254930	10.112900
-83.596150	43.843030	21.388140	7.661170
-82.263960	44.027400	82.154100	20.624040
-80.919660	44.196660	143.540800	30.428430
-79.564150	44.350410	159.426100	25.368980
-78.198390	44.488330	119.702000	13.241550
-76.823410	44.610100	59.848840	14.363740
-75.440290	44.715440	49.607180	13.646540
-74.050180	44.804110	84.023120	20.794080
-72.654240	44.875890	139.313400	19.040060
-71.253690	44.930630	176.692000	15.242220
-69.849760	44.968180	199.326900	9.700069
-68.443720	44.988460	209.194600	5.750439
-67.036810	44.991420	214.885100	2.919254
-65.630330	44.977050	219.203600	.767432
-64.225530	44.945390	221.599500	1.330428
-62.823690	44.896510	221.486300	1.523507
-61.426040	44.830540	220.357700	1.008068
-60.033810	44.747610	218.999400	.732285
-58.648170	44.647940	218.500400	.544659
-57.270290	44.531750	218.239100	.421161
-55.901260	44.399320	217.919100	.262683
-54.542130	44.250930	217.478200	.072860
-53.193900	44.086940	217.327300	.041092
-51.857480	43.907680	217.301800	.025795
-50.533710	43.713560	217.302100	.013597
-49.223370	43.504980	217.306400	.006080
-47.927120	43.282370	217.309400	.002311
-46.645540	43.046150	217.310700	.000739
-45.379100	42.796800	217.311100	.000199
-44.128160	42.534760	217.311200	.000090
-42.892960	42.260480	217.311200	.000056
-41.673610	41.974410	217.311200	.000031
-92.917770	43.067330	211.996700	2.975307
-91.673000	43.354680	206.824200	4.755116
-90.411740	43.629630	191.679800	8.805145
-89.134110	43.891690	160.101400	15.604120
-87.840450	44.140390	110.711200	24.329710
-86.531140	44.375260	59.432690	25.821340
-85.206750	44.595830	36.246320	14.727870
-83.867900	44.801650	38.576360	13.432800

-82.515380 44.992280 58.168260 13.810840
-81.150000 45.167310 100.457100 17.118350
-79.772730 45.326340 111.353900 12.935710
-78.384560 45.469020 114.922700 11.118060
-76.986600 45.595010 94.334830 13.574890
-75.579970 45.704020 76.803570 22.208450
-74.165890 45.795780 103.131800 30.927750
-72.745610 45.870080 137.734300 24.361380
-71.320400 45.926740 176.534700 13.458760
-69.891600 45.965610 197.170500 9.381290
-68.460530 45.986610 207.898500 6.977311
-67.028560 45.989670 214.920800 2.868715
-65.597030 45.974800 219.850200 .826013
-64.167300 45.942020 221.851600 1.676309
-62.740720 45.891430 222.252500 1.822223
-61.318600 45.823140 221.683200 1.643109
-59.902260 45.737310 220.386900 1.036800
-58.492950 45.634170 219.280000 .744003
-57.091900 45.513950 218.683200 .520553
-55.700260 45.376930 218.017500 .284751
-54.319140 45.223450 217.405800 .176366
-52.949590 45.053840 217.208800 .156654
-51.592580 44.868490 217.258100 .071592
-50.248970 44.667810 217.288000 .030200
-48.919560 44.452230 217.301300 .011964
-47.605040 44.222190 217.307600 .004305
-46.305980 43.978160 217.310200 .001353
-45.022840 43.720610 217.311000 .000364
-43.755960 43.450020 217.311200 .000118
-42.505550 43.166850 217.311200 .000074
-41.271660 42.871580 217.311200 .000031
-93.329110 43.967430 213.163700 2.472155
-92.068780 44.264030 208.959700 4.066833
-90.791210 44.547890 196.272000 7.913265
-89.496510 44.818510 168.661700 14.192220
-88.184940 45.075390 120.964800 23.719020
-86.856880 45.318050 68.311260 29.254350
-85.512890 45.545990 46.253770 19.726300
-84.153590 45.758730 51.068910 10.164150
-82.779790 45.955820 59.851630 12.045370
-81.392350 46.136820 50.177920 12.452730
-79.992260 46.301310 62.672830 18.008530
-78.580580 46.448910 103.609600 18.096520
-77.158460 46.579270 118.559500 21.892200
-75.727110 46.692070 133.563100 23.493230
-74.287820 46.787040 138.383900 27.510340
-72.841890 46.863950 166.932500 16.271050
-71.390720 46.922590 185.256500 9.668513
-69.935700 46.962830 197.199200 7.775632
-68.478260 46.984570 207.314700 5.711451
-67.019850 46.987740 215.400100 2.373844
-65.561920 46.972340 219.500200 .918408
-64.105920 46.938410 221.228500 1.475116
-62.653260 46.886040 222.197700 1.786179
-61.205390 46.815350 222.744900 2.254977
-59.763680 46.726520 221.941200 1.783023
-58.329470 46.619790 220.276000 1.128271
-56.904060 46.495390 219.012900 .709042
-55.488670 46.353640 218.143500 .472015
-54.084500 46.194880 217.443100 .423526
-52.692630 46.019470 217.143900 .312589
-51.314070 45.827840 217.211600 .138348
-49.949730 45.620390 217.274100 .050009
-48.600430 45.397590 217.296900 .018139
-47.266860 45.159900 217.306000 .006243
-45.949600 44.907820 217.309600 .001945
-44.649100 44.641840 217.310800 .000529

-43.365670	44.362450	217.311100	.000141
-42.099450	44.070140	217.311200	.000070
-40.850440	43.765410	217.311200	.000031
-93.760410	44.864160	214.385300	1.869286
-92.483920	45.170280	211.268400	3.182694
-91.189420	45.463320	201.377900	6.692082
-89.876960	45.742750	177.031400	13.599630
-88.546760	46.008070	131.254700	26.430040
-87.199170	46.258760	82.347410	35.603020
-85.834720	46.494300	66.548150	30.039820
-84.454070	46.714200	79.992310	20.799930
-83.058010	46.917960	85.774260	20.325050
-81.647480	47.105130	78.453200	28.035970
-80.223460	47.275260	87.835330	34.276520
-78.787090	47.427960	116.051200	34.612000
-77.339580	47.562840	147.681700	28.366200
-75.882230	47.679570	166.481100	19.677370
-74.416370	47.777870	183.646800	14.257280
-72.943440	47.857470	194.457100	8.157421
-71.464880	47.918190	199.068100	5.091786
-69.982220	47.959850	203.718700	5.201693
-68.496960	47.982350	209.828200	3.772796
-67.010670	47.985630	215.924700	1.416047
-65.524890	47.969690	218.579700	.687915
-64.041170	47.934570	219.903700	.956427
-62.561040	47.880340	221.327200	1.530611
-61.086020	47.807170	222.585500	2.143822
-59.617590	47.715230	222.378800	2.261845
-58.157170	47.604770	221.221000	1.612051
-56.706150	47.476050	219.700700	1.151375
-55.265830	47.329400	218.761700	.882134
-53.837470	47.165180	217.909000	.723221
-52.422200	46.983780	217.344800	.407713
-51.021090	46.785640	217.235400	.176249
-49.635090	46.571200	217.275500	.060347
-48.265020	46.340950	217.296600	.020850
-46.911610	46.095370	217.305600	.006967
-45.575410	45.834990	217.309400	.002176
-44.256860	45.560320	217.310700	.000610
-42.956210	45.271870	217.311000	.000155
-41.673580	44.970160	217.311200	.000081
-40.408870	44.655690	217.311200	.000041
-94.212860	45.757310	215.510500	1.243175
-92.919560	46.073230	213.579100	2.118925
-91.607470	46.375730	207.626400	4.430416
-90.276550	46.664250	193.348800	9.138074
-88.926960	46.938270	165.614300	17.711830
-87.559010	47.197250	130.898200	28.200210
-86.173240	47.440650	113.913300	31.162900
-84.770290	47.667940	118.236200	30.695170
-83.350960	47.878600	122.583000	30.731840
-81.916200	48.072160	129.167200	29.039750
-80.467090	48.248140	137.276700	30.132050
-79.004780	48.406110	158.185600	27.910150
-77.530590	48.545680	177.929600	17.606900
-76.045850	48.666500	195.218300	11.031360
-74.552020	48.768250	203.979500	7.057419
-73.050600	48.850660	208.759800	4.220433
-71.543170	48.913520	209.998500	2.709351
-70.031330	48.956650	211.098500	2.357206
-68.516710	48.979950	214.011900	2.363475
-67.000980	48.983350	216.049500	1.163831
-65.485790	48.966840	216.711700	.961375
-63.972820	48.930470	218.102300	.740374
-62.463700	48.874330	219.983000	1.268546
-60.960050	48.798580	221.039800	1.495393
-59.463460	48.703410	221.383600	1.548109

-57.975440	48.589080	220.881300	1.600233
-56.497490	48.455880	220.007900	1.234151
-55.030970	48.304140	219.093600	1.037314
-53.577210	48.134270	218.247700	.757299
-52.137430	47.946660	217.502800	.333468
-50.712720	47.741790	217.267900	.137498
-49.304080	47.520120	217.283300	.048353
-47.912350	47.282170	217.298700	.017280
-46.538250	47.028450	217.306100	.006019
-45.182340	46.759510	217.309400	.001911
-43.845020	46.475880	217.310700	.000541
-42.526500	46.178100	217.311000	.000141
-41.226800	45.866700	217.311200	.000074
-39.945740	45.542200	217.311200	.000045
-94.687740	46.646660	216.395100	.699985
-93.376940	46.972690	215.417400	1.187494
-92.046550	47.284930	212.414900	2.558625
-90.696440	47.582840	204.790700	5.495852
-89.326680	47.865840	190.195900	11.777910
-87.937550	48.133390	171.854600	21.512640
-86.529520	48.384910	158.138600	29.990680
-85.103260	48.619850	156.192300	24.870810
-83.659580	48.837660	160.604200	22.873400
-82.199460	49.037830	171.181600	18.844630
-80.724000	49.219880	182.571900	16.403560
-79.234450	49.383340	191.354900	14.514920
-77.732160	49.527770	202.167500	9.565157
-76.218590	49.652830	209.040500	5.720788
-74.695260	49.758160	212.332100	3.807245
-73.163800	49.843490	214.171300	2.420080
-71.625870	49.908580	215.222000	1.621924
-70.083210	49.953250	216.331400	1.000793
-68.537570	49.977380	217.210500	.833816
-66.990740	49.980900	216.988100	.759288
-65.444490	49.963800	215.712100	1.545039
-63.900620	49.926140	216.413700	1.054213
-62.360880	49.868010	218.220600	.702373
-60.827010	49.789570	219.341600	.859623
-59.300730	49.691040	219.813700	.942840
-57.783640	49.572690	219.943300	1.034112
-56.277320	49.434830	219.365900	.917585
-54.783270	49.277820	218.752600	.718501
-53.302860	49.102080	218.104300	.500096
-51.837360	48.908040	217.480700	.237108
-50.387950	48.696200	217.293600	.079114
-48.955640	48.467040	217.289000	.029656
-47.541310	48.221130	217.301600	.011490
-46.145660	47.959000	217.307200	.004126
-44.769230	47.681210	217.309800	.001382
-43.412380	47.388330	217.310700	.000403
-42.075270	47.080920	217.311100	.000122
-40.757810	46.759540	217.311200	.000070
-39.459720	46.424710	217.311200	.000036
-95.186440	47.531940	217.013800	.280927
-93.857410	47.868410	216.699900	.473172
-92.508000	48.190720	215.375900	1.124070
-91.137910	48.498310	211.797700	2.874948
-89.747150	48.790600	204.949700	6.279154
-88.335950	49.067010	196.078800	11.174600
-86.904710	49.326930	189.003400	15.359870
-85.454100	49.569790	186.458700	16.588060
-83.984930	49.795020	191.628800	12.930470
-82.498220	50.002060	199.690000	8.863783
-80.995120	50.190400	205.396500	6.952615
-79.476920	50.359550	209.577100	5.623719
-77.945060	50.509060	213.701800	3.372949
-76.401090	50.638520	215.903400	1.979167

-74.846650	50.747580	216.581300	1.639195
-73.283450	50.835950	216.666700	1.300372
-71.713320	50.903350	216.900300	1.164018
-70.138080	50.949630	217.805700	.737008
-68.559640	50.974620	219.132000	.658517
-66.979900	50.978270	218.716200	.520084
-65.400800	50.960560	217.217600	.564381
-63.824260	50.921550	217.175200	.553358
-62.252170	50.861340	217.858900	.440891
-60.686400	50.780110	218.358900	.494921
-59.128770	50.678080	218.793500	.620474
-57.581030	50.555550	218.982000	.655426
-56.044860	50.412850	218.667600	.552157
-54.521820	50.250360	218.257000	.423250
-53.013420	50.068520	217.864800	.289389
-51.520960	49.867810	217.489000	.133183
-50.045680	49.648740	217.330500	.049503
-48.588630	49.411830	217.309200	.017640
-47.150700	49.157670	217.307600	.006663
-45.732590	48.886830	217.309100	.002473
-44.334810	48.599900	217.310300	.000839
-42.957660	48.297460	217.310900	.000255
-41.601190	47.980120	217.311200	.000105
-40.265250	47.648430	217.311200	.000067
-38.949390	47.302930	217.311200	.000036
-95.710510	48.412890	217.425200	.127645
-94.362470	48.760130	217.464400	.226150
-92.993230	49.092850	216.877600	.374776
-91.602360	49.410460	215.268200	1.234482
-90.189750	49.712350	212.509100	2.714032
-88.755540	49.997940	209.382100	4.523693
-87.300090	50.266570	206.782300	6.051621
-85.824040	50.517650	206.341900	6.366219
-84.328210	50.750570	209.075800	4.997843
-82.813610	50.964740	212.645100	3.215498
-81.281460	51.159630	214.704000	2.487988
-79.733130	51.334700	216.150900	1.577505
-78.170130	51.489480	217.429100	.847757
-76.594090	51.623530	217.868600	.634925
-75.006800	51.736490	217.648600	.700958
-73.410070	51.828010	217.453200	.849940
-71.805870	51.897850	217.531600	.618841
-70.196170	51.945790	218.285700	.739583
-68.582990	51.971680	219.470500	.841980
-66.968440	51.975460	220.050400	.868295
-65.354560	51.957120	219.457900	.702797
-63.743440	51.916690	218.848700	.786734
-62.137130	51.854320	218.429700	.571297
-60.537630	51.770170	218.145400	.422291
-58.946900	51.664510	218.201000	.400619
-57.366820	51.537620	218.231900	.365570
-55.799180	51.389870	218.007300	.287119
-54.245650	51.221680	217.786300	.206841
-52.707820	51.033510	217.607600	.135654
-51.187080	50.825860	217.418500	.068935
-49.684720	50.599280	217.341600	.027520
-48.201800	50.354330	217.316700	.010242
-46.739240	50.091630	217.310800	.003733
-45.297720	49.811770	217.310400	.001304
-43.877700	49.515370	217.310800	.000448
-42.479410	49.203060	217.311000	.000153
-41.102810	48.875440	217.311200	.000078
-39.747590	48.533100	217.311200	.000067
-38.413150	48.176570	217.311200	.000036
-96.261670	49.289190	217.665700	.260712
-94.893750	49.647600	217.707600	.381851
-93.503820	49.991080	217.429400	.200419

-92.091320	50.319060	216.640700	.461949
-90.655950	50.630910	215.689400	1.028582
-89.197760	50.925990	214.705500	1.628646
-87.717060	51.203660	214.088800	2.043684
-86.214420	51.463270	214.404700	2.062659
-84.690660	51.704190	215.811800	1.466941
-83.146820	51.925780	217.496600	.840025
-81.584160	52.127480	218.040700	.577177
-80.004110	52.308710	218.356400	.410685
-78.408280	52.468990	218.467100	.457931
-76.798410	52.607840	218.297000	.414267
-75.176390	52.724850	217.949800	.378593
-73.544200	52.819690	217.621700	.513859
-71.903920	52.892050	217.836100	.384432
-70.257710	52.941730	218.212100	.480551
-68.607750	52.968570	219.167500	.712602
-66.956280	52.972490	219.935200	.946630
-65.305560	52.953480	219.806200	.893161
-63.657820	52.911580	219.256500	.794989
-62.015270	52.846950	218.466100	.545101
-60.380080	52.759750	217.961200	.334093
-58.754360	52.650280	217.848900	.247917
-57.140120	52.518850	217.793000	.203311
-55.539290	52.365840	217.638500	.145970
-53.953660	52.191710	217.510600	.097517
-52.384890	51.996940	217.430500	.061082
-50.834480	51.782070	217.364300	.032031
-49.303730	51.547700	217.329300	.013924
-47.793770	51.294400	217.315500	.005365
-46.305490	51.022820	217.311600	.001858
-44.839570	50.733620	217.311000	.000633
-43.396390	50.427420	217.311000	.000204
-41.976090	50.104880	217.311200	.000096
-40.578500	49.766630	217.311200	.000059
-39.203150	49.413260	217.311200	.000049
-37.849210	49.045330	217.311200	.000031
-96.841810	50.160530	217.575000	.213717
-95.453030	50.530490	217.635100	.255710
-94.041510	50.885130	217.419200	.105429
-92.606450	51.223850	217.119000	.157321
-91.147380	51.546020	216.823700	.357604
-89.664200	51.850960	216.553500	.538846
-88.157130	52.138020	216.459500	.631983
-86.626690	52.406500	216.753300	.547544
-85.073680	52.655720	217.601500	.305790
-83.499180	52.885050	218.595800	.659058
-81.904440	53.093850	218.876400	.679416
-80.290990	53.281520	218.770000	.671759
-78.660530	53.447530	218.566300	.665948
-77.014920	53.591380	218.234100	.494253
-75.356160	53.712650	217.719000	.335260
-73.686420	53.810940	217.054800	.525342
-72.007930	53.885960	217.033600	.485812
-70.322990	53.937460	217.390900	.326196
-68.634010	53.965280	217.976100	.521546
-66.943400	53.969350	218.772700	.589439
-65.253590	53.949630	218.872200	.558206
-63.567000	53.906200	218.462600	.440335
-61.886040	53.839190	217.951500	.290697
-60.213040	53.748820	217.647400	.185999
-58.550290	53.635360	217.604600	.144314
-56.899960	53.499180	217.572400	.113325
-55.264110	53.340690	217.471100	.074482
-53.644650	53.160350	217.397000	.045579
-52.043340	52.958710	217.357700	.026977
-50.461760	52.736320	217.332800	.014082
-48.901280	52.493820	217.319000	.006342

-47.363030	52.231850	217.313300	.002442
-45.847900	51.951060	217.311600	.000848
-44.356530	51.652150	217.311200	.000265
-42.889220	51.335790	217.311200	.000100
-41.445980	51.002650	217.311200	.000056
-40.026490	50.653380	217.311200	.000049
-38.630060	50.288590	217.311200	.000036
-37.255620	49.908830	217.311200	.000031
-97.453060	51.026530	217.430300	.097988
-96.042340	51.408490	217.438200	.099052
-94.608220	51.774700	217.352300	.037067
-93.149600	52.124570	217.265000	.049462
-91.665800	52.457450	217.183200	.113010
-90.156560	52.772640	217.121500	.163849
-88.621980	53.069450	217.129900	.176878
-87.062480	53.347160	217.276700	.124479
-85.478850	53.605050	217.626300	.123573
-83.872130	53.842430	218.090000	.308355
-82.243660	54.058630	218.168800	.346241
-80.595020	54.253030	218.102100	.367164
-78.928000	54.425040	218.002100	.368162
-77.244600	54.574130	217.771700	.252420
-75.546970	54.699840	217.360300	.205604
-73.837420	54.801750	216.650200	.582469
-72.118360	54.879540	216.314600	.797625
-70.392330	54.932960	216.797000	.448555
-68.661900	54.961820	217.249800	.365154
-66.929700	54.966030	217.836800	.378164
-65.198370	54.945580	217.943200	.265366
-63.470540	54.900540	217.741400	.201157
-61.748820	54.831050	217.516000	.136390
-60.035740	54.737340	217.409700	.084899
-58.333780	54.619720	217.440800	.078995
-56.645260	54.478570	217.441400	.059102
-54.972420	54.314330	217.386300	.036743
-53.317280	54.127510	217.347900	.020754
-51.681740	53.918690	217.329300	.011518
-50.067420	53.688470	217.319200	.005858
-48.475770	53.437500	217.314100	.002596
-46.907930	53.166490	217.312000	.001000
-45.364770	52.876130	217.311400	.000349
-43.846840	52.567130	217.311200	.000105
-42.354370	52.240220	217.311200	.000031
-40.887210	51.896080	217.311200	.000031
-39.444820	51.535370	217.311200	.000031
-38.026250	51.158730	217.311200	.000031
-36.630120	50.766700	217.311200	.000031
-98.097820	51.886770	217.349100	.033871
-96.663910	52.281200	217.345300	.029431
-95.206040	52.659450	217.324700	.010818
-93.722790	53.020900	217.302900	.013832
-92.213200	53.364910	217.283200	.032075
-90.676770	53.690770	217.273200	.044192
-89.113440	53.997730	217.289300	.042253
-87.523560	54.285050	217.350300	.029815
-85.907830	54.551970	217.460900	.065921
-84.267300	54.797760	217.579900	.128028
-82.603340	55.021710	217.585300	.146077
-80.917590	55.223140	217.562900	.151478
-79.211960	55.401440	217.550000	.168709
-77.488560	55.556020	217.482900	.121780
-75.749730	55.686380	217.243200	.170295
-73.997920	55.792100	216.564000	.555340
-72.235800	55.872810	215.995400	1.050310
-70.466090	55.928230	216.422700	.640684
-68.691570	55.958180	217.012900	.382826
-66.915130	55.962550	217.379400	.213205

-65.139640	55.941330	217.501800	.115286
-63.367970	55.894590	217.437300	.091585
-61.602940	55.822490	217.343500	.064205
-59.847310	55.725280	217.304400	.040312
-58.103760	55.603290	217.342500	.035239
-56.374820	55.456930	217.356900	.024709
-54.662870	55.286670	217.339200	.015323
-52.970100	55.093080	217.325300	.008656
-51.298500	54.876750	217.317800	.004631
-49.649800	54.638340	217.314000	.002259
-48.025460	54.378550	217.312200	.000967
-46.426660	54.098120	217.311500	.000370
-44.854210	53.797780	217.311300	.000137
-43.308580	53.478290	217.311200	.000038
-41.789850	53.140400	217.311200	.000031
-40.297680	52.784830	217.311200	.000031
-38.831280	52.412250	217.311200	.000031
-37.389370	52.023280	217.311200	.000031
-35.970190	51.618480	217.311200	.000031
-98.778840	52.740810	217.322300	.010494
-97.320270	53.148240	217.320100	.008081
-95.837370	53.539010	217.315500	.003255
-94.328290	53.912530	217.310400	.003463
-92.791720	54.268120	217.306400	.008070
-91.226900	54.605080	217.306000	.010231
-89.633560	54.922620	217.314500	.008654
-88.011890	55.219970	217.336300	.013223
-86.362530	55.496330	217.368900	.031236
-84.686490	55.750910	217.393000	.050919
-82.985150	55.982970	217.375500	.061888
-81.260250	56.191770	217.348100	.065851
-79.513790	56.376650	217.353200	.068867
-77.748020	56.536990	217.369600	.071866
-75.965460	56.672260	217.238800	.150915
-74.168790	56.781970	216.615800	.509138
-72.360860	56.865740	215.868200	1.122414
-70.544630	56.923280	216.149400	.868312
-68.723180	56.954370	216.808300	.414940
-66.899620	56.958910	217.203000	.158601
-65.077100	56.936880	217.331900	.053138
-63.258730	56.888360	217.338700	.032354
-61.447630	56.813510	217.310500	.024873
-59.646790	56.712620	217.292100	.018580
-57.859090	56.586040	217.313200	.013601
-56.087300	56.434210	217.322300	.008620
-54.333970	56.257640	217.319600	.005370
-52.601470	56.056940	217.315900	.003187
-50.891880	55.832740	217.313400	.001695
-49.207030	55.585770	217.312100	.000803
-47.548420	55.316760	217.311500	.000332
-45.917200	55.026500	217.311300	.000132
-44.314130	54.715760	217.311200	.000038
-42.739580	54.385350	217.311200	.000031
-41.193420	54.036030	217.311200	.000031
-39.675070	53.668560	217.311200	.000031
-38.183400	53.283620	217.311200	.000031
-36.716770	52.881820	217.311200	.000031
-35.272900	52.463700	217.311200	.000031
-99.499240	53.588130	217.314200	.003101
-98.014280	54.009130	217.313500	.002228
-96.504860	54.412960	217.312400	.001009
-94.968610	54.799050	217.311300	.000810
-93.403790	55.166730	217.310600	.001767
-91.809290	55.515250	217.310900	.002012
-90.184560	55.843850	217.313800	.002387
-88.529620	56.151670	217.320000	.006418
-86.844990	56.437890	217.327400	.012876

-85.131650	56.701690	217.329000	.019557
-83.390960	56.942230	217.315000	.027226
-81.624720	57.158780	217.286100	.038948
-79.835050	57.350580	217.279800	.034409
-78.024370	57.516980	217.309000	.046361
-76.195360	57.657400	217.238000	.133716
-74.350940	57.771310	216.702600	.464131
-72.494220	57.858320	215.927600	1.066641
-70.628410	57.918090	215.921300	1.030067
-68.756890	57.950390	216.617600	.496788
-66.883060	57.955100	217.081500	.184690
-65.010370	57.932220	217.249000	.059657
-63.142240	57.881810	217.296700	.018512
-61.282040	57.804080	217.303400	.008538
-59.433060	57.699310	217.302500	.006443
-57.598450	57.567890	217.308900	.004510
-55.781200	57.410300	217.312800	.002619
-53.984060	57.227100	217.313200	.001645
-52.209560	57.018940	217.312600	.001039
-50.459930	56.786500	217.311800	.000559
-48.737060	56.530560	217.311500	.000263
-47.042490	56.251900	217.311300	.000114
-45.377330	55.951360	217.311200	.000031
-43.742270	55.629770	217.311200	.000031
-42.137470	55.287960	217.311200	.000031
-40.562610	54.926740	217.311200	.000031
-39.016750	54.546860	217.311200	.000031
-37.498410	54.149020	217.311200	.000031
-36.005400	53.733830	217.311200	.000031
-34.534880	53.301770	217.311200	.000031
-100.262700	54.428120	217.312000	.000861
-98.749250	54.863350	217.311700	.000588
-97.211560	55.280850	217.311500	.000284
-95.646590	55.680060	217.311300	.000187
-94.052090	56.060360	217.311100	.000334
-92.426510	56.420970	217.311200	.000367
-90.768940	56.761100	217.311700	.000857
-89.079190	57.079890	217.312600	.002352
-87.357570	57.376450	217.312500	.004776
-85.605000	57.649890	217.308600	.008760
-83.822840	57.899360	217.299600	.016346
-82.012920	58.124030	217.285600	.032446
-80.177480	58.323120	217.263100	.026780
-78.319110	58.495910	217.275600	.052402
-76.440710	58.641750	217.199400	.116547
-74.545430	58.760110	216.798900	.427068
-72.636660	58.850530	216.059300	.899589
-70.717930	58.912650	215.769200	1.157176
-68.792920	58.946230	216.404100	.605985
-66.865380	58.951130	216.973800	.240609
-64.939080	58.927340	217.195500	.086750
-63.017780	58.874950	217.273000	.030167
-61.105210	58.794160	217.297900	.010142
-59.204930	58.685300	217.305300	.003788
-57.320400	58.548780	217.309000	.001648
-55.454840	58.385130	217.310900	.000734
-53.611270	58.194950	217.311600	.000425
-51.792370	57.978940	217.311500	.000287
-50.000500	57.737840	217.311400	.000168
-48.237630	57.472490	217.311300	.000091
-46.505310	57.183730	217.311200	.000046
-44.804610	56.872430	217.311200	.000031
-43.136050	56.539500	217.311200	.000031
-41.499610	56.185780	217.311200	.000031
-39.894630	55.812120	217.311200	.000031
-38.319800	55.419290	217.311200	.000031
-36.773090	55.007980	217.311200	.000031

-35.251730 54.578750 217.311200 .000031
-33.752120 54.132060 217.311200 .000031
-101.073700 55.260130 217.311400 .000230
-99.529010 55.710320 217.311300 .000152
-97.960900 56.142140 217.311300 .000100
-96.365430 56.555100 217.311200 .000045
-94.739640 56.948590 217.311200 .000103
-93.081440 57.321860 217.311100 .000124
-91.389490 57.674070 217.311000 .000348
-89.663250 58.004340 217.310400 .001039
-87.902820 58.311730 217.308400 .002669
-86.109000 58.595320 217.303800 .006244
-84.283100 58.854170 217.297500 .016230
-82.427000 59.087400 217.292400 .037652
-80.543050 59.294160 217.276200 .019795
-78.634010 59.473690 217.242300 .044140
-76.703000 59.625280 217.169400 .132224
-74.753460 59.748340 216.848400 .345373
-72.789070 59.842370 216.219700 .733129
-70.813740 59.906970 215.712900 1.191454
-68.831480 59.941910 216.182900 .741367
-66.846440 59.947010 216.846600 .316355
-64.862770 59.922250 217.146000 .117752
-62.884610 59.867760 217.254700 .042510
-60.916040 59.783740 217.293000 .014782
-58.961010 59.670550 217.305400 .004824
-57.023270 59.528640 217.309400 .001459
-55.106350 59.358580 217.310800 .000371
-53.213500 59.161040 217.311200 .000109
-51.347600 58.936760 217.311200 .000071
-49.511150 58.686560 217.311200 .000050
-47.706190 58.411320 217.311200 .000049
-45.934240 58.111950 217.311200 .000041
-44.196280 57.789400 217.311200 .000041
-42.492650 57.444580 217.311200 .000036
-40.823030 57.078400 217.311200 .000031
-39.186360 56.691730 217.311200 .000031
-37.580830 56.285340 217.311200 .000031
-36.003750 55.859910 217.311200 .000031
-34.451600 55.415950 217.311200 .000031
-32.919830 54.953830 217.311200 .000031
-101.937200 56.083360 217.311300 .000089
-100.358100 56.549350 217.311200 .000039
-98.756940 56.996240 217.311200 .000031
-97.128790 57.423640 217.311200 .000052
-95.469860 57.830970 217.311200 .000085
-93.777320 58.217490 217.311100 .000145
-92.049300 58.582370 217.310800 .000389
-90.284800 58.924680 217.309800 .001053
-88.483640 59.243460 217.307600 .002579
-86.646400 59.537720 217.303400 .006112
-84.774360 59.806450 217.299300 .018433
-82.869400 60.048700 217.299000 .043803
-80.933970 60.263580 217.289200 .013142
-78.971000 60.450230 217.256100 .047546
-76.983890 60.607900 217.165100 .127184
-74.976370 60.735940 216.941100 .254986
-72.952470 60.833800 216.424700 .555963
-70.916480 60.901050 215.815900 1.045165
-68.872850 60.937420 216.006100 .882773
-66.826130 60.942730 216.695100 .404221
-64.780930 60.916960 217.093700 .150669
-62.741830 60.860230 217.237900 .053575
-60.713310 60.772790 217.288000 .018585
-58.699720 60.654990 217.304600 .006033
-56.705200 60.507380 217.309500 .001791
-54.733590 60.330550 217.310800 .000475

-52.788400	60.125220	217.311100	.000128
-50.872730	59.892220	217.311200	.000075
-48.989200	59.632420	217.311200	.000071
-47.139900	59.346780	217.311200	.000079
-45.326320	59.036270	217.311200	.000076
-43.549270	58.701890	217.311200	.000082
-41.808860	58.344600	217.311200	.000067
-40.104370	57.965370	217.311200	.000056
-38.434220	57.565060	217.311200	.000036
-36.795950	57.144450	217.311200	.000036
-35.186060	56.704150	217.311200	.000036
-33.600030	56.244660	217.311200	.000031
-32.032190	55.766200	217.311200	.000031
-102.859900	56.896880	217.311200	.000031
-101.242000	57.379640	217.311200	.000031
-99.604390	57.842470	217.311200	.000036
-97.940930	58.285070	217.311200	.000061
-96.246660	58.706940	217.311200	.000093
-94.517820	59.107400	217.311100	.000169
-92.751850	59.485580	217.310700	.000441
-90.947170	59.840560	217.309700	.001099
-89.103220	60.171320	217.307700	.002550
-87.220290	60.476800	217.304200	.006478
-85.299550	60.755960	217.302200	.020719
-83.342830	61.007760	217.305000	.044665
-81.352710	61.231220	217.298600	.005297
-79.332320	61.425420	217.276900	.055051
-77.285290	61.589540	217.214100	.100628
-75.215710	61.722870	217.046800	.175837
-73.128000	61.824790	216.674200	.387875
-71.026890	61.894870	216.052300	.805377
-68.917300	61.932760	215.907800	.978079
-66.804310	61.938290	216.563200	.473968
-64.692990	61.911450	217.052700	.175493
-62.588420	61.852340	217.226600	.060477
-60.495590	61.761240	217.284700	.020609
-58.419300	61.638580	217.303800	.006659
-56.364090	61.484900	217.309300	.001974
-54.334160	61.300890	217.310800	.000547
-52.333320	61.087320	217.311100	.000153
-50.364880	60.845090	217.311200	.000082
-48.431600	60.575160	217.311200	.000074
-46.535590	60.278550	217.311200	.000070
-44.678250	59.956310	217.311200	.000078
-42.860180	59.609490	217.311200	.000074
-41.081100	59.239120	217.311200	.000080
-39.339830	58.846180	217.311200	.000067
-37.634110	58.431540	217.311200	.000056
-35.960610	57.995940	217.311200	.000036
-34.314830	57.539980	217.311200	.000036
-32.690990	57.064010	217.311200	.000031
-31.082000	56.568130	217.311200	.000031
-103.849600	57.699570	217.311200	.000031
-102.187300	58.200280	217.311200	.000031
-100.509000	58.680030	217.311200	.000036
-98.806840	59.138710	217.311200	.000061
-97.074520	59.575940	217.311200	.000090
-95.307120	59.991050	217.311100	.000169
-93.501110	60.383240	217.310700	.000433
-91.654170	60.751550	217.309800	.001038
-89.765210	61.094930	217.308000	.002384
-87.834170	61.412270	217.305600	.007072
-85.861950	61.702450	217.305700	.022227
-83.850420	61.964360	217.310100	.036453
-81.802150	62.196920	217.303600	.017708
-79.720490	62.399150	217.293200	.056613
-77.609360	62.570130	217.257500	.061738

-75.473210 62.709080 217.159500 .106297
-73.316960 62.815350 216.905700 .244538
-71.145790 62.888430 216.377200 .547524
-68.965200 62.927950 216.070200 .914408
-66.780790 62.933720 216.563500 .461882
-64.598260 62.905720 217.050600 .172087
-62.423240 62.844070 217.228200 .058481
-60.261290 62.749080 217.285400 .019636
-58.117710 62.621230 217.303900 .006345
-55.997520 62.461110 217.309400 .001897
-53.905320 62.269450 217.310800 .000522
-51.845240 62.047150 217.311100 .000154
-49.820820 61.795150 217.311200 .000088
-47.834930 61.514500 217.311200 .000073
-45.889670 61.206310 217.311200 .000078
-43.986290 60.871700 217.311200 .000074
-42.125070 60.511780 217.311200 .000080
-40.305270 60.127630 217.311200 .000067
-38.525020 59.720250 217.311200 .000056
-36.781200 59.290500 217.311200 .000036
-35.069400 58.839090 217.311200 .000036
-33.383770 58.366500 217.311200 .000036
-31.716990 57.872960 217.311200 .000031
-30.060130 57.358350 217.311200 .000031
-104.916500 58.490070 217.311200 .000031
-103.202300 59.010120 217.311200 .000031
-101.477600 59.507980 217.311200 .000036
-99.732440 59.983750 217.311200 .000045
-97.958720 60.437220 217.311200 .000071
-96.150010 60.867820 217.311100 .000142
-94.301540 61.274790 217.310700 .000365
-92.410060 61.657170 217.310000 .000882
-90.473700 62.013880 217.308600 .002142
-88.491920 62.343760 217.307400 .007128
-86.465330 62.645610 217.309200 .019467
-84.395630 62.918240 217.310500 .016523
-82.285480 63.160470 217.307500 .037877
-80.138370 63.371240 217.303800 .035193
-77.958560 63.549540 217.286100 .033022
-75.750930 63.694510 217.230100 .057458
-73.520860 63.805420 217.099100 .134748
-71.274150 63.881700 216.804000 .304379
-69.016910 63.922960 216.521300 .520148
-66.755400 63.928990 216.834100 .304222
-64.495980 63.899750 217.126000 .121661
-62.244980 63.835380 217.248700 .043633
-60.008560 63.736250 217.291400 .015222
-57.792640 63.602840 217.305600 .005004
-55.602750 63.435840 217.309800 .001505
-53.443960 63.236060 217.310900 .000431
-51.320750 63.004450 217.311200 .000147
-49.236890 62.742080 217.311200 .000086
-47.195330 62.450080 217.311200 .000074
-45.198120 62.129630 217.311200 .000064
-43.246250 61.781950 217.311200 .000064
-41.339580 61.408210 217.311200 .000049
-39.476710 61.009530 217.311200 .000036
-37.654880 60.586900 217.311200 .000036
-35.869840 60.141170 217.311200 .000036
-34.115770 59.672970 217.311200 .000036
-32.385130 59.182660 217.311200 .000031
-30.668580 58.670220 217.311200 .000031
-28.954870 58.135290 217.311200 .000031
-106.073800 59.266680 217.311200 .000031
-104.297500 59.807790 217.311200 .000031
-102.518800 60.325180 217.311200 .000031
-100.725000 60.819250 217.311200 .000036

-98.905530	61.289990	217.311200	.000082
-97.052100	61.737010	217.311100	.000115
-95.158340	62.159620	217.310900	.000281
-93.219700	62.556880	217.310300	.000642
-91.233350	62.927700	217.309500	.001615
-89.198040	63.270870	217.309300	.005396
-87.113920	63.585110	217.310500	.012639
-84.982480	63.869130	217.311200	.007120
-82.806390	64.121680	217.310400	.024564
-80.589290	64.341560	217.311600	.017228
-78.335770	64.527670	217.300900	.015217
-76.051160	64.679080	217.273600	.028407
-73.741460	64.794950	217.218000	.061838
-71.413090	64.874680	217.113200	.126670
-69.072890	64.917820	216.986900	.196950
-66.727910	64.924110	217.108900	.130709
-64.385280	64.893550	217.221900	.060646
-62.052090	64.826280	217.277100	.024865
-59.735260	64.722690	217.299600	.009395
-57.441380	64.583340	217.307800	.003227
-55.176610	64.408980	217.310400	.001003
-52.946510	64.200520	217.311000	.000284
-50.755950	63.959000	217.311200	.000105
-48.608900	63.685590	217.311200	.000084
-46.508400	63.381530	217.311200	.000064
-44.456330	63.048090	217.311200	.000036
-42.453320	62.686580	217.311200	.000036
-40.498620	62.298210	217.311200	.000036
-38.589950	61.884150	217.311200	.000036
-36.723370	61.445380	217.311200	.000031
-34.893150	60.982680	217.311200	.000031
-33.091610	60.496560	217.311200	.000031
-31.309040	59.987160	217.311200	.000031
-29.533510	59.454220	217.311200	.000031
-27.750760	58.896990	217.311200	.000031
-107.339200	60.027230	217.311200	.000031
-105.487000	60.591610	217.311200	.000031
-103.643800	61.130270	217.311200	.000031
-101.793500	61.644070	217.311200	.000036
-99.922550	62.133320	217.311200	.000064
-98.020040	62.597820	217.311200	.000089
-96.077510	63.037030	217.311000	.000190
-94.088710	63.450070	217.310700	.000422
-92.049460	63.835860	217.310200	.000868
-89.957580	64.193130	217.310000	.002257
-87.812570	64.520550	217.311200	.003352
-85.615560	64.816710	217.311400	.008881
-83.369120	65.080260	217.313500	.010397
-81.077050	65.309890	217.313400	.006665
-78.744270	65.504390	217.308000	.006694
-76.376630	65.662700	217.295600	.013294
-73.980770	65.783920	217.273500	.026890
-71.563890	65.867360	217.241000	.047740
-69.133670	65.912500	217.210900	.066412
-66.698060	65.919100	217.239600	.049142
-64.265090	65.887100	217.273800	.026952
-61.842790	65.816700	217.295200	.012633
-59.438900	65.708320	217.305500	.005183
-57.060850	65.562580	217.309500	.001869
-54.715470	65.380330	217.310800	.000600
-52.408880	65.162570	217.311200	.000198
-50.146370	64.910470	217.311200	.000082
-47.932120	64.625290	217.311200	.000036
-45.769150	64.308400	217.311200	.000036
-43.659080	63.961160	217.311200	.000031
-41.602020	63.584970	217.311200	.000031
-39.596350	63.181100	217.311200	.000031

-37.638600 62.750720 217.311200 .000031
-35.723270 62.294770 217.311200 .000031
-33.842640 61.813940 217.311200 .000031
-31.986660 61.308540 217.311200 .000031
-30.142740 60.778430 217.311200 .000031
-28.295590 60.222970 217.311200 .000031
-26.427080 59.640870 217.311200 .000031
-108.736700 60.768890 217.311200 .000031
-106.789100 61.359350 217.311200 .000031
-104.866900 61.921510 217.311200 .000031
-102.949500 62.456830 217.311200 .000031
-101.019200 62.966030 217.311200 .000036
-99.061880 63.449270 217.311200 .000085
-97.066180 63.906190 217.311000 .000152
-95.023590 64.336020 217.310800 .000302
-92.928180 64.737730 217.310600 .000480
-90.776390 65.110020 217.310700 .000766
-88.566850 65.451470 217.311600 .000643
-86.300130 65.760610 217.312400 .003995
-83.978550 66.035940 217.313900 .004431
-81.606060 66.276030 217.313300 .003352
-79.187900 66.479550 217.310100 .003882
-76.730480 66.645290 217.303300 .007745
-74.241170 66.772270 217.292400 .014883
-71.728080 66.859700 217.279000 .023555
-69.199870 66.907030 217.270400 .029396
-66.665550 66.913940 217.278500 .024017
-64.134220 66.880400 217.291900 .015116
-61.614990 66.806620 217.302200 .007851
-59.116610 66.693070 217.307900 .003478
-56.647430 66.540450 217.310300 .001336
-54.215080 66.349720 217.311000 .000454
-51.826330 66.121990 217.311200 .000156
-49.486860 65.858530 217.311200 .000056
-47.201050 65.560780 217.311200 .000036
-44.971820 65.230190 217.311200 .000036
-42.800360 64.868270 217.311200 .000031
-40.685980 64.476460 217.311200 .000031
-38.625890 64.056100 217.311200 .000031
-36.614990 63.608330 217.311200 .000031
-34.645670 63.134020 217.311200 .000031
-32.707610 62.633670 217.311200 .000031
-30.787590 62.107330 217.311200 .000031
-28.869240 61.554470 217.311200 .000031
-26.932890 60.973880 217.311200 .000031
-24.955330 60.363580 217.311200 .000031

Q.08a

-89.453400	32.991660	228.908300	.055801
-88.345930	33.191160	228.906700	.060648
-87.228100	33.381660	228.899500	.082578
-86.100230	33.562890	228.887700	.119615
-84.962690	33.734530	228.871700	.170381
-83.815900	33.896310	228.851700	.233122
-82.660300	34.047950	228.828900	.303980
-81.496410	34.189190	228.805800	.381486
-80.324730	34.319790	228.779100	.478938
-79.145840	34.439500	228.733300	.629684
-77.960310	34.548100	228.648000	.857829
-76.768760	34.645400	228.525000	1.123308
-75.571840	34.731200	228.413200	1.315866
-74.370180	34.805370	228.382300	1.336427
-73.164480	34.867740	228.452100	1.189304
-71.955430	34.918200	228.578700	.935383
-70.743730	34.956650	228.707000	.652072
-69.530080	34.983020	228.804200	.401237
-68.315220	34.997260	228.864400	.218517
-67.099880	34.999340	228.896700	.105380
-65.884770	34.989250	228.912100	.045254
-64.670640	34.967020	228.918500	.017489
-63.458190	34.932690	228.921100	.006153
-62.248150	34.886320	228.922000	.001997
-61.041230	34.828000	228.922300	.000640
-59.838120	34.757850	228.922400	.000264
-58.639500	34.676010	228.922400	.000190
-57.446040	34.582610	228.922400	.000163
-56.258390	34.477850	228.922400	.000134
-55.077160	34.361910	228.922400	.000100
-53.902950	34.235010	228.922400	.000073
-52.736340	34.097380	228.922400	.000061
-51.577860	33.949260	228.922500	.000125
-50.428010	33.790910	228.922500	.000122
-49.287250	33.622600	228.922500	.000122
-48.156040	33.444620	228.922500	.000122
-47.034740	33.257250	228.922500	.000122
-45.923690	33.060790	228.922500	.000122
-44.823220	32.855560	228.922500	.000122
-89.701210	33.918400	228.893400	.096621
-88.583520	34.125020	228.887800	.109214
-87.455040	34.322360	228.869000	.152558
-86.316120	34.510110	228.839500	.223636
-85.167100	34.687960	228.799900	.321057
-84.008420	34.855620	228.749400	.444236
-82.840550	35.012790	228.691100	.584501
-81.663990	35.159200	228.637300	.726510
-80.479300	35.294580	228.584900	.891926
-79.287040	35.418700	228.494200	1.150318
-78.087840	35.531310	228.297400	1.577747
-76.882320	35.632210	227.980000	2.095753
-75.671150	35.721200	227.665900	2.471361
-74.455030	35.798110	227.584000	2.479619
-73.234660	35.862810	227.779700	2.157143
-72.010770	35.915150	228.112900	1.679023
-70.784100	35.955040	228.434200	1.151148
-69.555380	35.982390	228.667000	.694351
-68.325390	35.997170	228.804000	.368403
-67.094890	35.999320	228.872800	.172992
-65.864640	35.988860	228.903300	.072351
-64.635410	35.965800	228.915600	.027233
-63.407950	35.930180	228.920200	.009341
-62.183030	35.882080	228.921700	.002964
-60.961370	35.821590	228.922100	.000934

-59.743720	35.748840	228.922200	.000411
-58.530780	35.663950	228.922200	.000330
-57.323260	35.567100	228.922200	.000295
-56.121820	35.458470	228.922300	.000230
-54.927120	35.338260	228.922300	.000138
-53.739770	35.206700	228.922300	.000072
-52.560380	35.064020	228.922400	.000065
-51.389480	34.910500	228.922500	.000070
-50.227610	34.746390	228.922500	.000122
-49.075240	34.571990	228.922500	.000122
-47.932830	34.387580	228.922500	.000122
-46.800740	34.193490	228.922500	.000122
-45.679330	33.990000	228.922500	.000122
-44.568900	33.777440	228.922500	.000122
-89.960660	34.843320	228.840500	.215604
-88.832330	35.057210	228.818700	.252241
-87.692770	35.261540	228.755100	.358811
-86.542320	35.455950	228.657300	.527688
-85.381340	35.640140	228.528300	.758830
-84.210270	35.813810	228.358700	1.064375
-83.029580	35.976620	228.158900	1.420987
-81.839790	36.128310	227.985000	1.760299
-80.641480	36.268600	227.854900	2.102731
-79.435230	36.397220	227.638700	2.627163
-78.221690	36.513940	227.062000	3.610150
-77.001530	36.618520	225.973600	4.929514
-75.775430	36.710780	224.827100	5.877584
-74.544130	36.790520	224.576500	5.661387
-73.308360	36.857590	225.284000	4.889817
-72.068890	36.911860	226.428200	3.764629
-70.826490	36.953220	227.471100	2.560859
-69.581950	36.981590	228.193100	1.509069
-68.336070	36.996900	228.600500	.779476
-67.089650	36.999130	228.794900	.355618
-65.843500	36.988290	228.875700	.145778
-64.598400	36.964370	228.906300	.054357
-63.355190	36.927440	228.917100	.018561
-62.114630	36.877570	228.920700	.005846
-60.877510	36.814860	228.921800	.001832
-59.644590	36.739430	228.921900	.000895
-58.416640	36.651430	228.921900	.000859
-57.194370	36.551040	228.921800	.000833
-55.978490	36.438440	228.922000	.000605
-54.769680	36.313860	228.922100	.000307
-53.568590	36.177530	228.922300	.000111
-52.375820	36.029710	228.922300	.000072
-51.191970	35.870660	228.922400	.000064
-50.017550	35.700660	228.922500	.000075
-48.853080	35.520030	228.922500	.000122
-47.698980	35.329070	228.922500	.000122
-46.555660	35.128100	228.922500	.000122
-45.423470	34.917440	228.922500	.000122
-44.302690	34.697410	228.922500	.000122
-90.232250	35.766320	228.695300	.466369
-89.092860	35.987640	228.617600	.561640
-87.941790	36.199090	228.411100	.809117
-86.779330	36.400320	228.099500	1.185875
-85.605870	36.591000	227.694700	1.691016
-84.421870	36.770790	227.139200	2.413298
-83.227800	36.939400	226.438100	3.338948
-82.024180	37.096500	225.879200	4.122895
-80.811620	37.241800	225.591400	4.735348
-79.590740	37.375040	225.205400	5.581130
-78.362190	37.495960	223.492300	8.155519
-77.126670	37.604320	219.938900	11.152390
-75.884930	37.699920	215.772100	13.555270
-74.637700	37.782550	214.932200	12.837340

-73.385770	37.852060	217.555500	10.298670
-72.129940	37.908310	221.404800	8.195369
-70.871030	37.951180	224.747100	5.513604
-69.609860	37.980580	226.929100	3.153571
-68.347290	37.996460	228.089100	1.552931
-67.084140	37.998780	228.612200	.675567
-65.821270	37.987530	228.815400	.268388
-64.559530	37.962740	228.886900	.099879
-63.299760	37.924460	228.910900	.034572
-62.042780	37.872770	228.918700	.010950
-60.789440	37.807780	228.920900	.003433
-59.540510	37.729610	228.921000	.002027
-58.296810	37.638420	228.920600	.002291
-57.059080	37.534390	228.920400	.002407
-55.828080	37.417740	228.921100	.001622
-54.604500	37.288680	228.921800	.000631
-53.389020	37.147460	228.922200	.000133
-52.182290	36.994370	228.922400	.000137
-50.984880	36.829660	228.922400	.000111
-49.797380	36.653650	228.922500	.000089
-48.620270	36.466650	228.922500	.000075
-47.454020	36.268990	228.922500	.000122
-46.299010	36.060990	228.922500	.000122
-45.155610	35.843010	228.922500	.000122
-44.024070	35.615370	228.922500	.000122
-90.516530	36.687310	228.340500	.929554
-89.365660	36.916220	228.094500	1.150056
-88.202590	37.134950	227.475900	1.677526
-87.027650	37.343150	226.549300	2.422847
-85.841200	37.540460	225.398500	3.333597
-84.643710	37.726540	223.695400	4.896198
-83.435650	37.901050	221.343500	7.247075
-82.217600	38.063690	219.532500	8.948290
-80.990140	38.214140	219.041100	9.804927
-79.753940	38.352110	218.743900	11.064420
-78.509670	38.477340	214.291900	16.299530
-77.258070	38.589580	203.600200	24.257970
-75.999910	38.688600	188.995800	30.321090
-74.735980	38.774210	187.058300	25.907280
-73.467090	38.846230	195.435400	20.467940
-72.194090	38.904510	207.497800	16.044710
-70.917830	38.948930	217.717800	11.037930
-69.639210	38.979390	223.952200	5.975060
-68.359090	38.995840	227.010600	2.703574
-67.078360	38.998240	228.273600	1.063981
-65.797930	38.986590	228.711400	.410055
-64.518690	38.960900	228.853700	.157542
-63.241520	38.921240	228.900000	.057632
-61.967320	38.867690	228.915200	.018637
-60.696920	38.800350	228.919000	.005930
-59.431210	38.719360	228.918500	.004542
-58.170970	38.624900	228.916600	.006401
-56.917050	38.517150	228.916000	.007387
-55.670200	38.396340	228.918300	.004692
-54.431160	38.262690	228.921300	.001089
-53.200640	38.116470	228.922300	.000581
-51.979310	37.957960	228.922600	.000620
-50.767770	37.787460	228.922600	.000390
-49.566610	37.605300	228.922400	.000186
-48.376340	37.411790	228.922400	.000100
-47.197410	37.207270	228.922400	.000060
-46.030250	36.992090	228.922500	.000122
-44.875180	36.766620	228.922500	.000122
-43.732470	36.531200	228.922500	.000122
-90.814130	37.606180	227.589200	1.687003
-89.651300	37.842850	226.897600	2.149415
-88.475770	38.069030	225.186300	3.189225

-87.287830	38.284350	222.649400	4.495542
-86.087840	38.488450	219.759900	5.626068
-84.876290	38.680970	215.172800	8.377302
-83.653650	38.861550	207.718800	13.854340
-82.420500	39.029860	201.880500	18.431580
-81.177470	39.185590	201.148200	19.228600
-79.925230	39.328410	202.925800	19.159660
-78.664510	39.458070	195.442000	26.995320
-77.396060	39.574290	172.642000	38.906270
-76.120680	39.676830	140.709800	46.124500
-74.839220	39.765490	125.493800	40.902690
-73.552520	39.840080	142.625400	34.604570
-72.261480	39.900440	173.726500	29.475890
-70.967010	39.946460	201.839000	19.085610
-69.670030	39.978020	218.066300	9.773001
-68.371470	39.995060	225.268400	3.776720
-67.072270	39.997550	227.887800	1.184349
-65.773400	39.985470	228.601800	.466390
-64.475770	39.958870	228.806900	.225276
-63.180340	39.917780	228.883300	.094003
-61.888020	39.862300	228.910000	.030967
-60.599740	39.792560	228.915900	.010050
-59.316390	39.708680	228.912000	.010905
-58.038840	39.610860	228.904800	.018917
-56.767930	39.499290	228.900600	.026207
-55.504470	39.374200	228.911700	.011053
-54.249260	39.235840	228.921000	.002809
-53.003000	39.084480	228.923700	.003077
-51.766410	38.920440	228.923500	.002274
-50.540120	38.744010	228.922900	.001211
-49.324710	38.555530	228.922600	.000548
-48.120720	38.355340	228.922500	.000220
-46.928620	38.143810	228.922400	.000100
-45.748810	37.921300	228.922400	.000060
-44.581620	37.688170	228.922400	.000063
-43.427300	37.444810	228.922500	.000122
-91.125630	38.522790	226.254100	2.763271
-89.950410	38.767390	224.552200	3.649725
-88.761920	39.001200	220.296700	5.681683
-87.560450	39.223820	213.717600	7.911703
-86.346370	39.434880	207.012500	8.659871
-85.120160	39.633990	196.678300	12.526820
-83.882300	39.820790	179.351600	20.130050
-82.633390	39.994930	161.988700	30.605610
-81.374080	40.156070	160.070600	33.599410
-80.105060	40.303890	169.351500	29.252560
-78.827100	40.438090	170.017200	28.513970
-77.540980	40.558400	145.050300	33.005360
-76.247540	40.664560	96.572450	36.846340
-74.947690	40.756360	73.437160	29.092070
-73.642300	40.833600	71.319920	39.883870
-72.332310	40.896110	117.270900	42.894340
-71.018690	40.943760	176.672100	27.302970
-69.702430	40.976440	210.092500	12.291130
-68.384490	40.994090	223.724400	4.002295
-67.065880	40.996670	227.953800	1.000705
-65.747600	40.984170	228.562200	.423312
-64.430650	40.956610	228.725500	.419017
-63.116020	40.914060	228.846200	.195886
-61.804690	40.856610	228.904500	.056701
-60.497620	40.784390	228.912800	.021793
-59.195770	40.697540	228.897300	.028325
-57.900040	40.596260	228.868300	.065331
-56.611330	40.480750	228.855800	.073884
-55.330490	40.351270	228.901700	.021548
-54.058330	40.208070	228.929100	.014421
-52.795620	40.051450	228.929700	.011911

-51.543080	39.881710	228.926400	.006936
-50.301390	39.699200	228.924000	.003197
-49.071130	39.504250	228.922900	.001306
-47.852850	39.297230	228.922600	.000500
-46.647030	39.078520	228.922400	.000170
-45.454070	38.848490	228.922400	.000080
-44.274280	38.607530	228.922400	.000057
-43.107920	38.356030	228.922500	.000122
-91.451740	39.437030	224.386400	4.030478
-90.263630	39.689750	220.978300	5.489452
-89.061680	39.931370	212.078200	8.790022
-87.846150	40.161480	198.164000	11.436410
-86.617400	40.379670	179.280500	14.675520
-85.375900	40.585540	152.486200	22.034810
-84.122160	40.778730	126.132500	24.770730
-82.856800	40.958850	117.784900	23.780020
-81.580460	41.125550	103.255100	38.275250
-80.293880	41.278500	114.925000	37.674330
-78.997860	41.417370	139.071600	23.379800
-77.693230	41.541890	134.795200	19.780080
-76.380870	41.651780	87.508900	25.038820
-75.061690	41.746810	48.939740	24.166990
-73.736660	41.826770	60.937570	25.409010
-72.406780	41.891490	94.812210	39.293060
-71.073040	41.940830	162.261700	27.200760
-69.736500	41.974670	206.620100	12.032210
-68.398190	41.992950	223.686600	3.691749
-67.059160	41.995620	228.131700	1.074809
-65.720480	41.982670	228.518400	.920962
-64.383220	41.954140	228.464500	1.007471
-63.048400	41.910080	228.754900	.501376
-61.717090	41.850600	228.903800	.116773
-60.390290	41.775820	228.921000	.064207
-59.069010	41.685920	228.864500	.087013
-57.754210	41.581080	228.782000	.152767
-56.446830	41.461530	228.757900	.144916
-55.147770	41.327530	228.977000	.070362
-53.857880	41.179360	228.970800	.077772
-52.577970	41.017320	228.951200	.042198
-51.308780	40.841740	228.933600	.017414
-50.051000	40.652980	228.926400	.007029
-48.805250	40.451390	228.923600	.002656
-47.572090	40.237360	228.922800	.000934
-46.352010	40.011290	228.922500	.000318
-45.145370	39.773560	228.922400	.000102
-43.952510	39.524580	228.922400	.000081
-42.773630	39.264750	228.922500	.000122
-91.793200	40.348750	222.611400	5.184137
-90.591710	40.609810	217.573000	6.809872
-89.375760	40.859430	203.484500	11.043490
-88.145600	41.097210	174.974500	19.164960
-86.901570	41.322720	132.457800	25.799140
-85.644150	41.535550	89.108200	24.397030
-84.373860	41.735290	74.916730	15.750890
-83.091290	41.921560	61.120270	17.526870
-81.797160	42.093980	75.280170	16.121520
-80.492230	42.252200	101.317900	16.347990
-79.177290	42.395880	121.503800	13.023660
-77.853240	42.524730	106.531300	15.662100
-76.521000	42.638450	61.754640	19.660300
-75.181550	42.736820	48.193980	19.052040
-73.835900	42.819590	77.267650	18.015920
-72.485100	42.886590	131.842200	23.677880
-71.130210	42.937670	180.651000	19.052770
-69.772340	42.972710	213.265100	8.260552
-68.412590	42.991630	223.722300	4.612899
-67.052090	42.994400	227.060400	3.390757

-65.691960	42.980990	227.872000	2.310612
-64.333320	42.951450	228.459100	1.274660
-62.977290	42.905830	228.760600	.610207
-61.624970	42.844260	229.018900	.202449
-60.277440	42.766850	229.000700	.261111
-58.935750	42.673790	228.848000	.171021
-57.600940	42.565280	228.645700	.250904
-56.273990	42.441570	228.926500	.155375
-54.955840	42.302930	229.100100	.245875
-53.647390	42.149640	229.119200	.231429
-52.349470	41.982030	228.994000	.100720
-51.062890	41.800450	228.946100	.036014
-49.788320	41.605260	228.929700	.012636
-48.526440	41.396860	228.924600	.004521
-47.277780	41.175640	228.923000	.001562
-46.042850	40.942000	228.922600	.000502
-44.822010	40.696380	228.922500	.000181
-43.615560	40.439180	228.922500	.000122
-42.423690	40.170830	228.922500	.000121
-92.150770	41.257820	221.381900	6.523858
-90.935390	41.527410	214.908400	9.300066
-89.704910	41.785250	196.788200	15.959350
-88.459540	42.030900	161.254500	24.343490
-87.199620	42.263930	108.761300	28.493830
-85.925610	42.483890	58.354520	23.980520
-84.638040	42.690380	28.927530	8.280602
-83.337520	42.882970	41.047600	19.735950
-82.024790	43.061280	75.809890	12.621470
-80.700610	43.224930	149.718900	22.229530
-79.365870	43.373580	170.846700	30.314500
-78.021450	43.506890	139.932000	27.901990
-76.668370	43.624570	61.367080	13.916340
-75.307630	43.726360	62.450020	11.922870
-73.940300	43.812040	124.780800	13.534740
-72.567500	43.881390	159.523600	22.144150
-71.190370	43.934270	197.201500	12.814880
-69.810050	43.970550	212.683200	11.388130
-68.427750	43.990140	222.254700	7.836240
-67.044650	43.993000	226.523700	4.018962
-65.661930	43.979120	228.778700	1.175762
-64.280810	43.948540	230.015900	1.206488
-62.902460	43.901310	229.983300	1.338383
-61.528050	43.837570	229.610300	1.102489
-60.158730	43.757450	229.332200	.870443
-58.795620	43.661140	228.956700	.612685
-57.439790	43.548850	229.050800	.501798
-56.092310	43.420850	229.278600	.564252
-54.754150	43.277410	229.425700	.597908
-53.426270	43.118850	229.185100	.345355
-52.109530	42.945500	229.017700	.137353
-50.804760	42.757750	228.953900	.049524
-49.512690	42.555960	228.932400	.018234
-48.233980	42.340550	228.925400	.006590
-46.969190	42.111940	228.923200	.002285
-45.718810	41.870550	228.922700	.000749
-44.483210	41.616830	228.922500	.000244
-43.262650	41.351200	228.922500	.000126
-42.057300	41.074100	228.922500	.000121
-92.525320	42.164070	221.086000	7.192295
-91.295520	42.442420	214.013200	10.697070
-90.049940	42.708690	194.039200	19.033470
-88.788760	42.962440	156.788600	27.352290
-87.512310	43.203190	106.165100	31.949420
-86.221000	43.430500	61.756660	25.168250
-84.915410	43.643920	35.839960	14.717590
-83.596150	43.843030	35.352240	16.442940
-82.263960	44.027400	101.174200	27.228810

-80.919660	44.196660	173.961800	29.929740
-79.564150	44.350410	192.431000	33.784350
-78.198390	44.488330	145.316800	22.114130
-76.823410	44.610100	76.098110	9.741566
-75.440290	44.715440	66.937680	14.811880
-74.050180	44.804110	95.816670	24.794480
-72.654240	44.875890	147.485800	25.418460
-71.253690	44.930630	177.306300	28.845450
-69.849760	44.968180	204.414300	20.234540
-68.443720	44.988460	217.503200	13.786810
-67.036810	44.991420	224.979000	6.025599
-65.630330	44.977050	229.673000	1.288481
-64.225530	44.945390	231.711600	3.574552
-62.823690	44.896510	231.463300	3.434154
-61.426040	44.830540	230.633500	2.569511
-60.033810	44.747610	229.849800	1.737640
-58.648170	44.647940	229.506000	1.222223
-57.270290	44.531750	229.529800	1.039110
-55.901260	44.399320	229.836900	1.100573
-54.542130	44.250930	229.609200	.855483
-53.193900	44.086940	229.212800	.405803
-51.857480	43.907680	229.031400	.161428
-50.533710	43.713560	228.960700	.064072
-49.223370	43.504980	228.935100	.024825
-47.927120	43.282370	228.926300	.009134
-46.645540	43.046150	228.923500	.003167
-45.379100	42.796800	228.922700	.001041
-44.128160	42.534760	228.922600	.000322
-42.892960	42.260480	228.922500	.000138
-41.673610	41.974410	228.922500	.000122
-92.917770	43.067330	221.649600	6.790924
-91.673000	43.354680	214.959900	9.708168
-90.411740	43.629630	195.717600	16.309570
-89.134110	43.891690	157.354800	25.740510
-87.840450	44.140390	106.154600	29.525100
-86.531140	44.375260	62.831010	26.129820
-85.206750	44.595830	44.937200	16.131200
-83.867900	44.801650	58.553940	20.925720
-82.515380	44.992280	87.027000	18.719990
-81.150000	45.167310	124.890100	24.589770
-79.772730	45.326340	129.022300	19.102610
-78.384560	45.469020	127.316300	10.439370
-76.986600	45.595010	112.907600	17.232280
-75.579970	45.704020	94.711530	24.407860
-74.165890	45.795780	123.106600	27.127370
-72.745610	45.870080	133.877700	41.927930
-71.320400	45.926740	172.692400	31.922670
-69.891600	45.965610	198.057700	26.287220
-68.460530	45.986610	212.169500	20.547220
-67.028560	45.989670	220.970500	12.355730
-65.597030	45.974800	227.745600	2.637671
-64.167300	45.942020	230.650300	2.153153
-62.740720	45.891430	231.404700	3.233036
-61.318600	45.823140	231.179200	2.933361
-59.902260	45.737310	230.221700	1.851565
-58.492950	45.634170	229.751200	1.179203
-57.091900	45.513950	229.939000	1.202528
-55.700260	45.376930	229.983100	1.244070
-54.319140	45.223450	229.626500	.901726
-52.949590	45.053840	229.268700	.469901
-51.592580	44.868490	229.065300	.215357
-50.248970	44.667810	228.974500	.089977
-48.919560	44.452230	228.939500	.034820
-47.605040	44.222190	228.927500	.012604
-46.305980	43.978160	228.923800	.004303
-45.022840	43.720610	228.922800	.001387
-43.755960	43.450020	228.922600	.000426

-42.505550	43.166850	228.922500	.000161
-41.271660	42.871580	228.922500	.000122
-93.329110	43.967430	222.917500	5.740180
-92.068780	44.264030	217.475600	7.949501
-90.791210	44.547890	201.969300	12.425850
-89.496510	44.818510	169.800200	19.231710
-88.184940	45.075390	119.436000	29.103610
-86.856880	45.318050	69.211500	34.227000
-85.512890	45.545990	57.802440	22.725150
-84.153590	45.758730	80.079350	18.153450
-82.779790	45.955820	89.729970	19.284830
-81.392350	46.136820	75.080570	12.456980
-79.992260	46.301310	74.307980	23.610850
-78.580580	46.448910	106.944400	27.046490
-77.158460	46.579270	118.562800	30.620270
-75.727110	46.692070	147.527400	22.106650
-74.287820	46.787040	146.128100	33.978460
-72.841890	46.863950	170.027600	27.988500
-71.390720	46.922590	188.693500	20.978860
-69.935700	46.962830	202.993800	18.324820
-68.478260	46.984570	213.827300	18.417120
-67.019850	46.987740	222.520800	10.702260
-65.561920	46.972340	227.886200	3.330998
-64.105920	46.938410	230.808400	1.507190
-62.653260	46.886040	232.167000	3.443258
-61.205390	46.815350	232.295300	3.662479
-59.763680	46.726520	231.382600	2.721723
-58.329470	46.619790	230.764000	2.052976
-56.904060	46.495390	230.798800	2.077654
-55.488670	46.353640	230.719700	2.089723
-54.084500	46.194880	230.259800	1.644976
-52.692630	46.019470	229.569500	.866568
-51.314070	45.827840	229.164100	.360689
-49.949730	45.620390	229.002000	.134970
-48.600430	45.397590	228.946000	.048271
-47.266860	45.159900	228.928800	.016437
-45.949600	44.907820	228.924100	.005363
-44.649100	44.641840	228.922800	.001670
-43.365670	44.362450	228.922600	.000493
-42.099450	44.070140	228.922500	.000140
-40.850440	43.765410	228.922500	.000112
-93.760410	44.864160	224.538100	4.654358
-92.483920	45.170280	220.466300	6.595840
-91.189420	45.463320	208.055500	11.831950
-89.876960	45.742750	179.081100	21.804720
-88.546760	46.008070	132.660800	34.004440
-87.199170	46.258760	90.891840	41.256790
-85.834720	46.494300	83.019680	34.251170
-84.454070	46.714200	106.308400	21.972620
-83.058010	46.917960	112.032900	21.963640
-81.647480	47.105130	93.050250	34.399810
-80.223460	47.275260	86.481870	46.783970
-78.787090	47.427960	101.559300	52.524620
-77.339580	47.562840	135.535600	41.328000
-75.882230	47.679570	163.650100	31.731730
-74.416370	47.777870	192.565100	19.983950
-72.943440	47.857470	207.639000	13.433240
-71.464880	47.918190	214.093400	8.947275
-69.982220	47.959850	218.225900	6.448951
-68.496960	47.982350	223.030900	6.972808
-67.010670	47.985630	228.873800	3.568043
-65.524890	47.969690	231.935500	2.047652
-64.041170	47.934570	233.181000	3.676497
-62.561040	47.880340	234.181300	5.083703
-61.086020	47.807170	234.388900	5.478248
-59.617590	47.715230	233.446600	4.679505
-58.157170	47.604770	232.881500	4.139114

-56.706150	47.476050	232.742300	4.128422
-55.265830	47.329400	232.350500	3.884887
-53.837470	47.165180	231.382800	2.874609
-52.422200	46.983780	229.968000	1.376723
-51.021090	46.785640	229.263700	.506037
-49.635090	46.571200	229.016800	.172521
-48.265020	46.340950	228.947200	.055562
-46.911610	46.095370	228.928200	.017722
-45.575410	45.834990	228.923700	.005517
-44.256860	45.560320	228.922700	.001671
-42.956210	45.271870	228.922400	.000485
-41.673580	44.970160	228.922300	.000133
-40.408870	44.655690	228.922500	.000079
-94.212860	45.757310	226.250200	3.209304
-92.919560	46.073230	223.917300	4.534047
-91.607470	46.375730	217.005500	7.549319
-90.276550	46.664250	201.015000	14.193010
-88.926960	46.938270	171.248700	26.413180
-87.559010	47.197250	140.022200	38.174790
-86.173240	47.440650	127.652300	41.381420
-84.770290	47.667940	138.120800	33.203830
-83.350960	47.878600	135.104200	34.563400
-81.916200	48.072160	130.141800	38.295100
-80.467090	48.248140	130.022100	43.858750
-79.004780	48.406110	143.431200	44.232160
-77.530590	48.545680	166.904900	38.032600
-76.045850	48.666500	197.621500	21.300790
-74.552020	48.768250	215.087700	10.040400
-73.050600	48.850660	223.082100	5.000908
-71.543170	48.913520	224.860700	3.335177
-70.031330	48.956650	225.625100	2.533271
-68.516710	48.979950	229.170400	2.128596
-67.000980	48.983350	233.146000	3.507871
-65.485790	48.966840	234.888800	5.000992
-63.972820	48.930470	235.390600	5.433735
-62.463700	48.874330	235.844400	6.081774
-60.960050	48.798580	235.871000	6.393618
-59.463460	48.703410	235.294100	6.003037
-57.975440	48.589080	234.902600	5.844872
-56.497490	48.455880	234.459500	5.737340
-55.030970	48.304140	233.485400	4.975010
-53.577210	48.134270	231.757700	3.260789
-52.137430	47.946660	229.823700	1.253940
-50.712720	47.741790	229.048600	.334664
-49.304080	47.520120	228.943800	.117559
-47.912350	47.282170	228.927400	.040870
-46.538250	47.028450	228.923100	.013361
-45.182340	46.759510	228.922200	.004221
-43.845020	46.475880	228.922200	.001306
-42.526500	46.178100	228.922300	.000396
-41.226800	45.866700	228.922300	.000115
-39.945740	45.542200	228.922400	.000065
-94.687740	46.646660	227.708000	1.683860
-93.376940	46.972690	226.663100	2.361187
-92.046550	47.284930	223.409000	4.465683
-90.696440	47.582840	214.933500	9.475463
-89.326680	47.865840	198.551500	19.737830
-87.937550	48.133390	180.096300	31.221550
-86.529520	48.384910	166.793300	40.789550
-85.103260	48.619850	162.582900	41.047620
-83.659580	48.837660	164.469000	32.834460
-82.199460	49.037830	171.985600	25.479850
-80.724000	49.219880	179.884300	23.421040
-79.234450	49.383340	187.907800	25.037040
-77.732160	49.527770	204.426000	18.376580
-76.218590	49.652830	216.888700	10.918660
-74.695260	49.758160	221.972200	8.185150

-73.163800	49.843490	224.513400	6.289670
-71.625870	49.908580	225.887300	4.435958
-70.083210	49.953250	227.359900	3.099256
-68.537570	49.977380	230.031600	1.291835
-66.990740	49.980900	233.639300	3.999231
-65.444490	49.963800	234.568400	5.027944
-63.900620	49.926140	234.731700	4.854979
-62.360880	49.868010	234.338700	4.576238
-60.827010	49.789570	234.366000	4.643655
-59.300730	49.691040	234.726300	5.073017
-57.783640	49.572690	234.832700	5.429437
-56.277320	49.434830	234.234500	5.299226
-54.783270	49.277820	233.032100	4.387855
-53.302860	49.102080	231.260200	2.647734
-51.837360	48.908040	229.256800	.703420
-50.387950	48.696200	228.751700	.179144
-48.955640	48.467040	228.862900	.091774
-47.541310	48.221130	228.906100	.031241
-46.145660	47.959000	228.917700	.008251
-44.769230	47.681210	228.920900	.002760
-43.412380	47.388330	228.921900	.000930
-42.075270	47.080920	228.922200	.000308
-40.757810	46.759540	228.922200	.000124
-39.459720	46.424710	228.922400	.000070
-95.186440	47.531940	228.922300	.290246
-93.857410	47.868410	228.816300	.529835
-92.508000	48.190720	227.626300	1.390448
-91.137910	48.498310	223.654000	4.650210
-89.747150	48.790600	216.134900	10.680460
-88.335950	49.067010	206.417800	18.536500
-86.904710	49.326930	198.867800	23.815490
-85.454100	49.569790	195.737400	22.780970
-83.984930	49.795020	198.666900	17.135450
-82.498220	50.002060	204.224900	11.713420
-80.995120	50.190400	209.603900	10.426740
-79.476920	50.359550	214.153100	10.641150
-77.945060	50.509060	220.681100	8.032181
-76.401090	50.638520	224.671900	5.945482
-74.846650	50.747580	225.333500	6.478455
-73.283450	50.835950	225.684200	6.217582
-71.713320	50.903350	226.227700	5.189595
-70.138080	50.949630	227.004200	3.949136
-68.559640	50.974620	229.617000	1.099915
-66.979900	50.978270	232.754500	3.421798
-65.400800	50.960560	233.344200	4.411016
-63.824260	50.921550	232.735400	3.751166
-62.252170	50.861340	231.818600	2.822714
-60.686400	50.780110	231.648800	2.493360
-59.128770	50.678080	232.400700	3.096584
-57.581030	50.555550	232.717700	3.587296
-56.044860	50.412850	232.458000	3.579407
-54.521820	50.250360	231.794300	3.035648
-53.013420	50.068520	230.605000	1.855596
-51.520960	49.867810	229.320900	.533579
-50.045680	49.648740	228.904000	.092860
-48.588630	49.411830	228.896000	.050231
-47.150700	49.157670	228.911800	.015722
-45.732590	48.886830	228.918300	.005318
-44.334810	48.599900	228.920900	.001877
-42.957660	48.297460	228.921900	.000685
-41.601190	47.980120	228.922200	.000255
-40.265250	47.648430	228.922200	.000114
-38.949390	47.302930	228.922400	.000075
-95.710510	48.412890	230.068700	1.656092
-94.362470	48.760130	230.661800	2.329445
-92.993230	49.092850	230.633100	2.417385
-91.602360	49.410460	229.276900	1.287342

-90.189750	49.712350	226.335500	2.521179
-88.755540	49.997940	223.274400	5.109659
-87.300090	50.266570	220.203000	7.200386
-85.824040	50.517650	218.333300	8.139372
-84.328210	50.750570	219.012800	6.188096
-82.813610	50.964740	220.609700	5.097887
-81.281460	51.159630	222.146400	4.646448
-79.733130	51.334700	223.732700	4.957360
-78.170130	51.489480	226.462000	3.471675
-76.594090	51.623530	227.541100	3.084430
-75.006800	51.736490	226.866900	4.527380
-73.410070	51.828010	226.393100	5.281443
-71.805870	51.897850	226.418600	4.935475
-70.196170	51.945790	227.057800	4.069170
-68.582990	51.971680	228.777400	1.778059
-66.968440	51.975460	231.917400	2.441300
-65.354560	51.957120	232.377400	3.695263
-63.743440	51.916690	231.620600	3.038957
-62.137130	51.854320	230.576800	1.930433
-60.537630	51.770170	230.431500	1.543147
-58.946900	51.664510	230.697100	1.728189
-57.366820	51.537620	230.807800	1.901805
-55.799180	51.389870	230.552800	1.776540
-54.245650	51.221680	230.262400	1.534654
-52.707820	51.033510	229.875100	1.086716
-51.187080	50.825860	229.199100	.385460
-49.684720	50.599280	228.977600	.098851
-48.201800	50.354330	228.927100	.031585
-46.739240	50.091630	228.920300	.009694
-45.297720	49.811770	228.920600	.003156
-43.877700	49.515370	228.921500	.001127
-42.479410	49.203060	228.922000	.000448
-41.102810	48.875440	228.922200	.000195
-39.747590	48.533100	228.922300	.000084
-38.413150	48.176570	228.922400	.000075
-96.261670	49.289190	231.106600	3.012128
-94.893750	49.647600	232.020900	4.005883
-93.503820	49.991080	232.467300	4.383339
-92.091320	50.319060	231.660700	3.451965
-90.655950	50.630910	230.316100	1.971907
-89.197760	50.925990	228.706800	1.018108
-87.717060	51.203660	227.292200	1.663002
-86.214420	51.463270	226.318400	2.330041
-84.690660	51.704190	226.183500	2.278475
-83.146820	51.925780	226.484700	2.057648
-81.584160	52.127480	226.886000	2.073314
-80.004110	52.308710	227.644000	1.836445
-78.408280	52.468990	228.339100	1.360217
-76.798410	52.607840	228.201200	1.936652
-75.176390	52.724850	227.111500	3.862648
-73.544200	52.819690	226.195600	5.197068
-71.903920	52.892050	226.614500	4.726337
-70.257710	52.941730	227.063400	3.906535
-68.607750	52.968570	228.525400	2.145979
-66.956280	52.972490	230.967200	1.280389
-65.305560	52.953480	231.414600	2.326743
-63.657820	52.911580	230.752000	1.983123
-62.015270	52.846950	230.130400	1.370289
-60.380080	52.759750	229.802900	1.014040
-58.754360	52.650280	229.788600	.946490
-57.140120	52.518850	229.676700	.863192
-55.539290	52.365840	229.539800	.737729
-53.953660	52.191710	229.412300	.613989
-52.384890	51.996940	229.262300	.449786
-50.834480	51.782070	229.064400	.199840
-49.303730	51.547700	228.963200	.073664
-47.793770	51.294400	228.931500	.024602

-46.305490	51.022820	228.923300	.007596
-44.839570	50.733620	228.922000	.002239
-43.396390	50.427420	228.922100	.000694
-41.976090	50.104880	228.922200	.000263
-40.578500	49.766630	228.922300	.000118
-39.203150	49.413260	228.922300	.000078
-37.849210	49.045330	228.922400	.000073
-96.841810	50.160530	231.053100	2.952726
-95.453030	50.530490	231.699900	3.617329
-94.041510	50.885130	231.736100	3.671601
-92.606450	51.223850	231.091200	2.834752
-91.147380	51.546020	230.171200	1.665001
-89.664200	51.850960	229.289800	.602634
-88.157130	52.138020	228.613000	.469398
-86.626690	52.406500	228.247200	.862647
-85.073680	52.655720	228.184100	.886980
-83.499180	52.885050	228.306900	.799769
-81.904440	53.093850	228.550400	.674130
-80.290990	53.281520	228.743900	.527114
-78.660530	53.447530	228.753900	.631941
-77.014920	53.591380	228.439500	1.356275
-75.356160	53.712650	227.333000	3.277995
-73.686420	53.810940	225.935300	5.298121
-72.007930	53.885960	226.309600	4.888961
-70.322990	53.937460	227.309800	3.491749
-68.634010	53.965280	228.383500	2.115183
-66.943400	53.969350	230.178800	.673652
-65.253590	53.949630	230.460500	1.072240
-63.567000	53.906200	229.976300	.899418
-61.886040	53.839190	229.638700	.755535
-60.213040	53.748820	229.442700	.635628
-58.550290	53.635360	229.322200	.512840
-56.899960	53.499180	229.225500	.394720
-55.264110	53.340690	229.148000	.306132
-53.644650	53.160350	229.090100	.237224
-52.043340	52.958710	229.036800	.168378
-50.461760	52.736320	228.976700	.088649
-48.901280	52.493820	228.942400	.038648
-47.363030	52.231850	228.927900	.014545
-45.847900	51.951060	228.923400	.004901
-44.356530	51.652150	228.922400	.001483
-42.889220	51.335790	228.922300	.000426
-41.445980	51.002650	228.922300	.000141
-40.026490	50.653380	228.922300	.000080
-38.630060	50.288590	228.922400	.000069
-37.255620	49.908830	228.922500	.000112
-97.453060	51.026530	230.081600	1.763314
-96.042340	51.408490	230.378800	2.044494
-94.608220	51.774700	230.208600	1.841412
-93.149600	52.124570	229.813500	1.286445
-91.665800	52.457450	229.447600	.720217
-90.156560	52.772640	229.124500	.234228
-88.621980	53.069450	228.887300	.165162
-87.062480	53.347160	228.762900	.335724
-85.478850	53.605050	228.743700	.342147
-83.872130	53.842430	228.800300	.279119
-82.243660	54.058630	228.881700	.202703
-80.595020	54.253030	228.900700	.209813
-78.928000	54.425040	228.851100	.371079
-77.244600	54.574130	228.633700	.870619
-75.546970	54.699840	227.764200	2.456146
-73.837420	54.801750	226.077500	4.986190
-72.118360	54.879540	225.751200	5.438550
-70.392330	54.932960	227.115200	3.523268
-68.661900	54.961820	228.184000	1.955645
-66.929700	54.966030	229.353200	.673382
-65.198370	54.945580	229.496500	.329375

-63.470540	54.900540	229.294700	.194431
-61.748820	54.831050	229.218800	.257534
-60.035740	54.737340	229.203600	.349190
-58.333780	54.619720	229.132500	.299067
-56.645260	54.478570	229.055700	.199324
-54.972420	54.314330	229.008300	.134990
-53.317280	54.127510	228.980100	.095153
-51.681740	53.918690	228.959500	.064311
-50.067420	53.688470	228.941700	.036596
-48.475770	53.437500	228.930200	.017458
-46.907930	53.166490	228.924900	.007211
-45.364770	52.876130	228.923000	.002623
-43.846840	52.567130	228.922500	.000834
-42.354370	52.240220	228.922300	.000245
-40.887210	51.896080	228.922300	.000090
-39.444820	51.535370	228.922400	.000062
-38.026250	51.158730	228.922400	.000056
-36.630120	50.766700	228.922500	.000112
-98.097820	51.886770	229.390100	.769391
-96.663910	52.281200	229.427600	.814334
-95.206040	52.659450	229.366000	.697803
-93.722790	53.020900	229.238800	.485389
-92.213200	53.364910	229.116500	.280698
-90.676770	53.690770	229.012000	.103970
-89.113440	53.997730	228.935200	.046683
-87.523560	54.285050	228.894100	.111378
-85.907830	54.551970	228.886900	.122868
-84.267300	54.797760	228.902600	.099334
-82.603340	55.021710	228.924000	.080695
-80.917590	55.223140	228.923400	.107812
-79.211960	55.401440	228.899800	.207274
-77.488560	55.556020	228.800700	.515568
-75.749730	55.686380	228.204000	1.694956
-73.997920	55.792100	226.433000	4.427767
-72.235800	55.872810	225.278900	5.960111
-70.466090	55.928230	226.463500	4.197391
-68.691570	55.958180	227.915700	2.006931
-66.915130	55.962550	228.698900	.871796
-65.139640	55.941330	228.924500	.424266
-63.367970	55.894590	228.913400	.281026
-61.602940	55.822490	228.944200	.108241
-59.847310	55.725280	229.007100	.095877
-58.103760	55.603290	229.019700	.140935
-56.374820	55.456930	228.981600	.098587
-54.662870	55.286670	228.955800	.059982
-52.970100	55.093080	228.942300	.038578
-51.298500	54.876750	228.934400	.024876
-49.649800	54.638340	228.928800	.014401
-48.025460	54.378550	228.925200	.007228
-46.426660	54.098120	228.923400	.003166
-44.854210	53.797780	228.922600	.001215
-43.308580	53.478290	228.922400	.000405
-41.789850	53.140400	228.922300	.000117
-40.297680	52.784830	228.922400	.000055
-38.831280	52.412250	228.922400	.000054
-37.389370	52.023280	228.922400	.000054
-35.970190	51.618480	228.922500	.000125
-98.778840	52.740810	229.091100	.308021
-97.320270	53.148240	229.086900	.293483
-95.837370	53.539010	229.065400	.251167
-94.328290	53.912530	229.028300	.183174
-92.791720	54.268120	228.990600	.111962
-91.226900	54.605080	228.957800	.048667
-89.633560	54.922620	228.933800	.010768
-88.011890	55.219970	228.920700	.030670
-86.362530	55.496330	228.917900	.038223
-84.686490	55.750910	228.922300	.034149

-82.985150	55.982970	228.927800	.033094
-81.260250	56.191770	228.930400	.052144
-79.513790	56.376650	228.934200	.118411
-77.748020	56.536990	228.924600	.293377
-75.965460	56.672260	228.538800	1.132747
-74.168790	56.781970	226.811400	3.799527
-72.360860	56.865740	225.051600	6.182171
-70.544630	56.923280	225.854900	5.015329
-68.723180	56.954370	227.514500	2.457780
-66.899620	56.958910	228.447400	.986713
-65.077100	56.936880	228.749300	.451531
-63.258730	56.888360	228.816000	.278714
-61.447630	56.813510	228.852400	.170777
-59.646790	56.712620	228.920800	.040443
-57.859090	56.586040	228.938700	.027421
-56.087300	56.434210	228.942800	.034452
-54.333970	56.257640	228.934200	.023857
-52.601470	56.056940	228.929100	.015042
-50.891880	55.832740	228.926200	.009394
-49.207030	55.585770	228.924400	.005434
-47.548420	55.316760	228.923300	.002793
-45.917200	55.026500	228.922800	.001257
-44.314130	54.715760	228.922600	.000496
-42.739580	54.385350	228.922500	.000202
-41.193420	54.036030	228.922500	.000124
-39.675070	53.668560	228.922500	.000109
-38.183400	53.283620	228.922500	.000109
-36.716770	52.881820	228.922500	.000109
-35.272900	52.463700	228.922500	.000122
-99.499240	53.588130	228.980300	.119136
-98.014280	54.009130	228.974300	.106567
-96.504860	54.412960	228.967000	.090292
-94.968610	54.799050	228.956200	.067584
-93.403790	55.166730	228.944700	.043313
-91.809290	55.515250	228.934700	.021528
-90.184560	55.843850	228.927600	.005672
-88.529620	56.151670	228.923600	.006530
-86.844990	56.437890	228.922800	.009984
-85.131650	56.701690	228.924200	.010558
-83.390960	56.942230	228.927100	.013847
-81.624720	57.158780	228.932300	.030842
-79.835050	57.350580	228.948600	.083047
-78.024370	57.516980	228.975100	.198568
-76.195360	57.657400	228.703900	.806861
-74.350940	57.771310	227.192000	3.142608
-72.494220	57.858320	225.187400	6.008626
-70.628410	57.918090	225.320300	5.820306
-68.756890	57.950390	227.100500	3.145197
-66.883060	57.955100	228.256500	1.247732
-65.010370	57.932220	228.678500	.483648
-63.142240	57.881810	228.815800	.221084
-61.282040	57.804080	228.865200	.122747
-59.433060	57.699310	228.894300	.055278
-57.598450	57.567890	228.922100	.008087
-55.781200	57.410300	228.926800	.008804
-53.984060	57.227100	228.925900	.007721
-52.209560	57.018940	228.924500	.005293
-50.459930	56.786500	228.923600	.003354
-48.737060	56.530560	228.923000	.001930
-47.042490	56.251900	228.922700	.000991
-45.377330	55.951360	228.922600	.000458
-43.742270	55.629770	228.922500	.000214
-42.137470	55.287960	228.922500	.000126
-40.562610	54.926740	228.922500	.000121
-39.016750	54.546860	228.922500	.000122
-37.498410	54.149020	228.922500	.000122
-36.005400	53.733830	228.922500	.000122

-34.534880 53.301770 228.922500 .000122
-100.262700 54.428120 228.941100 .044104
-98.749250 54.863350 228.938200 .037890
-97.211560 55.280850 228.935700 .031621
-95.646590 55.680060 228.932500 .024020
-94.052090 56.060360 228.929100 .016025
-92.426510 56.420970 228.926200 .008867
-90.768940 56.761100 228.924200 .003475
-89.079190 57.079890 228.923100 .001255
-87.357570 57.376450 228.923000 .002426
-85.605000 57.649890 228.923600 .003990
-83.822840 57.899360 228.925300 .008836
-82.012920 58.124030 228.929700 .025639
-80.177480 58.323120 228.941800 .068948
-78.319110 58.495910 228.962400 .191446
-76.440710 58.641750 228.716000 .717925
-74.545430 58.760110 227.566600 2.607227
-72.636660 58.850530 225.528900 5.639971
-70.717930 58.912650 224.914400 6.525862
-68.792920 58.946230 226.551800 4.083389
-66.865380 58.951130 228.027600 1.646761
-64.939080 58.927340 228.609500 .607858
-63.017780 58.874950 228.804000 .235661
-61.105210 58.794160 228.875800 .098066
-59.204930 58.685300 228.903300 .041806
-57.320400 58.548780 228.917700 .010891
-55.454840 58.385130 228.922300 .000986
-53.611270 58.194950 228.923000 .001569
-51.792370 57.978940 228.922900 .001537
-50.000500 57.737840 228.922800 .001059
-48.237630 57.472490 228.922600 .000632
-46.505310 57.183730 228.922500 .000332
-44.804610 56.872430 228.922500 .000166
-43.136050 56.539500 228.922500 .000111
-41.499610 56.185780 228.922500 .000121
-39.894630 55.812120 228.922500 .000122
-38.319800 55.419290 228.922500 .000122
-36.773090 55.007980 228.922500 .000122
-35.251730 54.578750 228.922500 .000122
-33.752120 54.132060 228.922500 .000122
-101.073700 55.260130 228.928100 .015604
-99.529010 55.710320 228.927000 .013008
-97.960900 56.142140 228.926100 .010707
-96.365430 56.555100 228.925200 .008202
-94.739640 56.948590 228.924200 .005665
-93.081440 57.321860 228.923500 .003396
-91.389490 57.674070 228.923000 .001673
-89.663250 58.004340 228.922600 .000686
-87.902820 58.311730 228.922600 .000969
-86.109000 58.595320 228.922900 .002721
-84.283100 58.854170 228.923400 .008563
-82.427000 59.087400 228.923500 .027863
-80.543050 59.294160 228.920300 .092317
-78.634010 59.473690 228.889600 .263266
-76.703000 59.625280 228.705200 .784282
-74.753460 59.748340 227.813600 2.314845
-72.789070 59.842370 225.972700 5.170014
-70.813740 59.906970 224.717700 6.916104
-68.831480 59.941910 225.951700 5.055809
-66.846440 59.947010 227.706100 2.209103
-64.862770 59.922250 228.501400 .804332
-62.884610 59.867760 228.780000 .293202
-60.916040 59.783740 228.873500 .107949
-58.961010 59.670550 228.905900 .039394
-57.023270 59.528640 228.917500 .012819
-55.106350 59.358580 228.921200 .003443
-53.213500 59.161040 228.922100 .000536

-51.347600	58.936760	228.922500	.000190
-49.511150	58.686560	228.922500	.000253
-47.706190	58.411320	228.922400	.000165
-45.934240	58.111950	228.922400	.000088
-44.196280	57.789400	228.922400	.000053
-42.492650	57.444580	228.922400	.000054
-40.823030	57.078400	228.922400	.000051
-39.186360	56.691730	228.922400	.000061
-37.580830	56.285340	228.922400	.000061
-36.003750	55.859910	228.922500	.000099
-34.451600	55.415950	228.922500	.000112
-32.919830	54.953830	228.922500	.000122
-101.937200	56.083360	228.924000	.005280
-100.358100	56.549350	228.923600	.004291
-98.756940	56.996240	228.923400	.003482
-97.128790	57.423640	228.923200	.002678
-95.469860	57.830970	228.922900	.001896
-93.777320	58.217490	228.922600	.001205
-92.049300	58.582370	228.922500	.000670
-90.284800	58.924680	228.922400	.000314
-88.483640	59.243460	228.922400	.000666
-86.646400	59.537720	228.922400	.002466
-84.774360	59.806450	228.922200	.008503
-82.869400	60.048700	228.920700	.028171
-80.933970	60.263580	228.912500	.089716
-78.971000	60.450230	228.877600	.284863
-76.983890	60.607900	228.708300	.774772
-74.976370	60.735940	228.092000	1.953295
-72.952470	60.833800	226.526600	4.383240
-70.916480	60.901050	224.921400	6.682459
-68.872850	60.937420	225.482800	5.764360
-66.826130	60.942730	227.299600	2.863182
-64.780930	60.916960	228.361000	1.050330
-62.741830	60.860230	228.741200	.365766
-60.713310	60.772790	228.865100	.128140
-58.699720	60.654990	228.904700	.044097
-56.705200	60.507380	228.917100	.014529
-54.733590	60.330550	228.920900	.004475
-52.788400	60.125220	228.921900	.001241
-50.872730	59.892220	228.922200	.000276
-48.989200	59.632420	228.922200	.000094
-47.139900	59.346780	228.922300	.000069
-45.326320	59.036270	228.922300	.000052
-43.549270	58.701890	228.922300	.000059
-41.808860	58.344600	228.922300	.000062
-40.104370	57.965370	228.922300	.000070
-38.434220	57.565060	228.922400	.000043
-36.795950	57.144450	228.922400	.000050
-35.186060	56.704150	228.922400	.000056
-33.600030	56.244660	228.922400	.000054
-32.032190	55.766200	228.922500	.000122
-102.859900	56.896880	228.922900	.001716
-101.242000	57.379640	228.922800	.001355
-99.604390	57.842470	228.922600	.001073
-97.940930	58.285070	228.922600	.000826
-96.246660	58.706940	228.922400	.000592
-94.517820	59.107400	228.922400	.000386
-92.751850	59.485580	228.922300	.000210
-90.947170	59.840560	228.922300	.000134
-89.103220	60.171320	228.922300	.000572
-87.220290	60.476800	228.922100	.002122
-85.299550	60.755960	228.921700	.007132
-83.342830	61.007760	228.919800	.022831
-81.352710	61.231220	228.912800	.069891
-79.332320	61.425420	228.885700	.201621
-77.285290	61.589540	228.771600	.571556
-75.215710	61.722870	228.303500	1.443655

-73.128000 61.824790 227.138700 3.304986
-71.026890 61.894870 225.463700 5.838057
-68.917300 61.932760 225.218200 6.032986
-66.804310 61.938290 226.925500 3.429885
-64.692990 61.911450 228.233500 1.262819
-62.588420 61.852340 228.707500 .421069
-60.495590 61.761240 228.856900 .142705
-58.419300 61.638580 228.902900 .047787
-56.364090 61.484900 228.916800 .015527
-54.334160 61.300890 228.920800 .004855
-52.333320 61.087320 228.921800 .001440
-50.364880 60.845090 228.922100 .000416
-48.431600 60.575160 228.922200 .000137
-46.535590 60.278550 228.922200 .000096
-44.678250 59.956310 228.922200 .000092
-42.860180 59.609490 228.922300 .000053
-41.081100 59.239120 228.922300 .000061
-39.339830 58.846180 228.922300 .000066
-37.634110 58.431540 228.922300 .000061
-35.960610 57.995940 228.922400 .000052
-34.314830 57.539980 228.922400 .000056
-32.690990 57.064010 228.922400 .000054
-31.082000 56.568130 228.922500 .000122
-103.849600 57.699570 228.922600 .000529
-102.187300 58.200280 228.922600 .000407
-100.509000 58.680030 228.922400 .000317
-98.806840 59.138710 228.922400 .000232
-97.074520 59.575940 228.922300 .000174
-95.307120 59.991050 228.922300 .000123
-93.501110 60.383240 228.922200 .000095
-91.654170 60.751550 228.922300 .000101
-89.765210 61.094930 228.922200 .000450
-87.834170 61.412270 228.922100 .001572
-85.861950 61.702450 228.921600 .005105
-83.850420 61.964360 228.919600 .015713
-81.802150 62.196920 228.912200 .045949
-79.720490 62.399150 228.885800 .127449
-77.609360 62.570130 228.788500 .336642
-75.473210 62.709080 228.475200 .897381
-73.316960 62.815350 227.678600 2.208287
-71.145790 62.888430 226.225100 4.443982
-68.965200 62.927950 225.601100 5.277736
-66.780790 62.933720 226.897600 3.345772
-64.598260 62.905720 228.215600 1.276549
-62.423240 62.844070 228.705400 .416517
-60.261290 62.749080 228.857800 .138264
-58.117710 62.621230 228.903500 .045816
-55.997520 62.461110 228.917000 .014794
-53.905320 62.269450 228.920900 .004625
-51.845240 62.047150 228.921900 .001395
-49.820820 61.795150 228.922100 .000430
-47.834930 61.514500 228.922200 .000151
-45.889670 61.206310 228.922200 .000106
-43.986290 60.871700 228.922300 .000055
-42.125070 60.511780 228.922300 .000061
-40.305270 60.127630 228.922300 .000066
-38.525020 59.720250 228.922300 .000061
-36.781200 59.290500 228.922400 .000052
-35.069400 58.839090 228.922400 .000056
-33.383770 58.366500 228.922400 .000054
-31.716990 57.872960 228.922400 .000061
-30.060130 57.358350 228.922500 .000122
-104.916500 58.490070 228.922500 .000191
-103.202300 59.010120 228.922500 .000169
-101.477600 59.507980 228.922300 .000092
-99.732440 59.983750 228.922300 .000067
-97.958720 60.437220 228.922300 .000079

-96.150010 60.867820 228.922200 .000116
-94.301540 61.274790 228.922200 .000099
-92.410060 61.657170 228.922200 .000119
-90.473700 62.013880 228.922200 .000316
-88.491920 62.343760 228.922100 .001034
-86.465330 62.645610 228.921700 .003231
-84.395630 62.918240 228.920200 .009721
-82.285480 63.160470 228.914300 .027825
-80.138370 63.371240 228.894300 .075988
-77.958560 63.549540 228.829800 .198841
-75.750930 63.694510 228.644300 .498882
-73.520860 63.805420 228.199400 1.214337
-71.274150 63.881700 227.384500 2.547353
-69.016910 63.922960 226.728100 3.442235
-66.755400 63.928990 227.596000 2.216575
-64.495980 63.899750 228.412400 .902585
-62.244980 63.835380 228.758600 .316845
-60.008560 63.736250 228.872000 .108624
-57.792640 63.602840 228.907400 .036765
-55.602750 63.435840 228.918000 .012071
-53.443960 63.236060 228.921200 .003820
-51.320750 63.004450 228.922000 .001183
-49.236890 62.742080 228.922200 .000358
-47.195330 62.450080 228.922300 .000119
-45.198120 62.129630 228.922300 .000076
-43.246250 61.781950 228.922300 .000070
-41.339580 61.408210 228.922300 .000046
-39.476710 61.009530 228.922400 .000052
-37.654880 60.586900 228.922400 .000056
-35.869840 60.141170 228.922400 .000064
-34.115770 59.672970 228.922400 .000061
-32.385130 59.182660 228.922400 .000061
-30.668580 58.670220 228.922500 .000112
-28.954870 58.135290 228.922500 .000122
-106.073800 59.266680 228.922500 .000122
-104.297500 59.807790 228.922500 .000120
-102.518800 60.325180 228.922500 .000121
-100.725000 60.819250 228.922400 .000054
-98.905530 61.289990 228.922300 .000061
-97.052100 61.737010 228.922300 .000056
-95.158340 62.159620 228.922300 .000056
-93.219700 62.556880 228.922200 .000111
-91.233350 62.927700 228.922200 .000214
-89.198040 63.270870 228.922100 .000627
-87.113920 63.585110 228.921900 .001910
-84.982480 63.869130 228.920900 .005607
-82.806390 64.121680 228.917400 .015683
-80.589290 64.341560 228.906300 .041766
-78.335770 64.527670 228.873600 .105568
-76.051160 64.679080 228.789900 .250030
-73.741460 64.794950 228.611500 .541508
-71.413090 64.874680 228.296000 1.073194
-69.072890 64.917820 227.990200 1.539090
-66.727910 64.924110 228.348000 1.004265
-64.385280 64.893550 228.678500 .452924
-62.052090 64.826280 228.833400 .180221
-59.735260 64.722690 228.892300 .068278
-57.441380 64.583340 228.912900 .024638
-55.176610 64.408980 228.919500 .008458
-52.946510 64.200520 228.921500 .002779
-50.755950 63.959000 228.922100 .000880
-48.608900 63.685590 228.922300 .000275
-46.508400 63.381530 228.922300 .000108
-44.456330 63.048090 228.922300 .000050
-42.453320 62.686580 228.922400 .000052
-40.498620 62.298210 228.922400 .000056
-38.589950 61.884150 228.922400 .000064

-36.723370 61.445380 228.922400 .000061
-34.893150 60.982680 228.922400 .000061
-33.091610 60.496560 228.922500 .000112
-31.309040 59.987160 228.922500 .000112
-29.533510 59.454220 228.922500 .000112
-27.750760 58.896990 228.922500 .000122
-107.339200 60.027230 228.922500 .000121
-105.487000 60.591610 228.922500 .000121
-103.643800 61.130270 228.922500 .000122
-101.793500 61.644070 228.922500 .000122
-99.922550 62.133320 228.922500 .000073
-98.020040 62.597820 228.922500 .000073
-96.077510 63.037030 228.922400 .000064
-94.088710 63.450070 228.922400 .000070
-92.049460 63.835860 228.922300 .000145
-89.957580 64.193130 228.922200 .000391
-87.812570 64.520550 228.922100 .001147
-85.615560 64.816710 228.921500 .003265
-83.369120 65.080260 228.919700 .008794
-81.077050 65.309890 228.914200 .022343
-78.744270 65.504390 228.899700 .053159
-76.376630 65.662700 228.866500 .116248
-73.980770 65.783920 228.803500 .228608
-71.563890 65.867360 228.708900 .390399
-69.133670 65.912500 228.631900 .529044
-66.698060 65.919100 228.722000 .374592
-64.265090 65.887100 228.823000 .199457
-61.842790 65.816700 228.880600 .092634
-59.438900 65.708320 228.906700 .039126
-57.060850 65.562580 228.917000 .015289
-54.715470 65.380330 228.920700 .005611
-52.408880 65.162570 228.921800 .001945
-50.146370 64.910470 228.922200 .000638
-47.932120 64.625290 228.922300 .000206
-45.769150 64.308400 228.922400 .000080
-43.659080 63.961160 228.922400 .000063
-41.602020 63.584970 228.922400 .000064
-39.596350 63.181100 228.922400 .000061
-37.638600 62.750720 228.922400 .000061
-35.723270 62.294770 228.922500 .000112
-33.842640 61.813940 228.922500 .000112
-31.986660 61.308540 228.922500 .000112
-30.142740 60.778430 228.922500 .000112
-28.295590 60.222970 228.922500 .000125
-26.427080 59.640870 228.922500 .000122
-108.736700 60.768890 228.922500 .000122
-106.789100 61.359350 228.922500 .000122
-104.866900 61.921510 228.922500 .000122
-102.949500 62.456830 228.922500 .000122
-101.019200 62.966030 228.922500 .000112
-99.061880 63.449270 228.922500 .000112
-97.066180 63.906190 228.922500 .000073
-95.023590 64.336020 228.922500 .000085
-92.928180 64.737730 228.922400 .000117
-90.776390 65.110020 228.922300 .000318
-88.566850 65.451470 228.922200 .000916
-86.300130 65.760610 228.921700 .002485
-83.978550 66.035940 228.920500 .006368
-81.606060 66.276030 228.917200 .015303
-79.187900 66.479550 228.909100 .034039
-76.730480 66.645290 228.892400 .068848
-74.241170 66.772270 228.864400 .123256
-71.728080 66.859700 228.829200 .188342
-69.199870 66.907030 228.807000 .227548
-66.665550 66.913940 228.834000 .181240
-64.134220 66.880400 228.871500 .112088
-61.614990 66.806620 228.898100 .058803

-59.116610	66.693070	228.912300	.027348
-56.647430	66.540450	228.918700	.011592
-54.215080	66.349720	228.921100	.004545
-51.826330	66.121990	228.921900	.001668
-49.486860	65.858530	228.922300	.000583
-47.201050	65.560780	228.922400	.000203
-44.971820	65.230190	228.922400	.000101
-42.800360	64.868270	228.922400	.000068
-40.685980	64.476460	228.922400	.000061
-38.625890	64.056100	228.922500	.000112
-36.614990	63.608330	228.922500	.000112
-34.645670	63.134020	228.922500	.000112
-32.707610	62.633670	228.922500	.000112
-30.787590	62.107330	228.922500	.000125
-28.869240	61.554470	228.922500	.000125
-26.932890	60.973880	228.922500	.000125
-24.955330	60.363580	228.922500	.000122

Q.09a

-89.453400	32.991660	241.154400	.020382
-88.345930	33.191160	241.154000	.022224
-87.228100	33.381660	241.151900	.031626
-86.100230	33.562890	241.148600	.048457
-84.962690	33.734530	241.143600	.073888
-83.815900	33.896310	241.136200	.109176
-82.660300	34.047950	241.126200	.153916
-81.496410	34.189190	241.115000	.206930
-80.324730	34.319790	241.100900	.280914
-79.145840	34.439500	241.073800	.406096
-77.960310	34.548100	241.017800	.606394
-76.768760	34.645400	240.932800	.848863
-75.571840	34.731200	240.862400	1.007938
-74.370180	34.805370	240.859600	.992661
-73.164480	34.867740	240.926300	.822284
-71.955430	34.918200	241.009800	.593608
-70.743730	34.956650	241.076100	.372877
-69.530080	34.983020	241.116700	.206132
-68.315220	34.997260	241.139200	.099230
-67.099880	34.999340	241.150300	.042274
-65.884770	34.989250	241.155400	.015437
-64.670640	34.967020	241.157500	.004285
-63.458190	34.932690	241.158200	.000386
-62.248150	34.886320	241.158300	.000862
-61.041230	34.828000	241.158300	.000841
-59.838120	34.757850	241.158300	.000554

-58.639500	34.676010	241.158200	.000307
-57.446040	34.582610	241.158200	.000165
-56.258390	34.477850	241.158200	.000096
-55.077160	34.361910	241.158200	.000091
-53.902950	34.235010	241.158200	.000092
-52.736340	34.097380	241.158200	.000092
-51.577860	33.949260	241.158200	.000092
-50.428010	33.790910	241.158200	.000092
-49.287250	33.622600	241.158200	.000092
-48.156040	33.444620	241.158200	.000092
-47.034740	33.257250	241.158200	.000092
-45.923690	33.060790	241.158200	.000092
-44.823220	32.855560	241.158200	.000092
-89.701210	33.918400	241.150100	.039261
-88.583520	34.125020	241.148500	.044600
-87.455040	34.322360	241.143000	.064294
-86.316120	34.510110	241.134400	.098334
-85.167100	34.687960	241.122200	.150168
-84.008420	34.855620	241.103100	.224938
-82.840550	35.012790	241.076300	.318566
-81.663990	35.159200	241.047200	.424747
-80.479300	35.294580	241.017700	.557456
-79.287040	35.418700	240.957800	.778420
-78.087840	35.531310	240.811500	1.160545
-76.882320	35.632210	240.551600	1.671495
-75.671150	35.721200	240.313900	1.992455
-74.455030	35.798110	240.287700	1.926688
-73.234660	35.862810	240.501400	1.567248
-72.010770	35.915150	240.760700	1.114854
-70.784100	35.955040	240.953200	.700555
-69.555380	35.982390	241.064600	.381483
-68.325390	35.997170	241.118700	.181149
-67.094890	35.999320	241.143100	.073866
-65.864640	35.988860	241.153400	.025358
-64.635410	35.965800	241.157300	.005619
-63.407950	35.930180	241.158600	.001403
-62.183030	35.882080	241.158700	.002693
-60.961370	35.821590	241.158600	.002236
-59.743720	35.748840	241.158400	.001448
-58.530780	35.663950	241.158300	.000774
-57.323260	35.567100	241.158200	.000370
-56.121820	35.458470	241.158200	.000165
-54.927120	35.338260	241.158100	.000140
-53.739770	35.206700	241.158200	.000091
-52.560380	35.064020	241.158200	.000092
-51.389480	34.910500	241.158200	.000092
-50.227610	34.746390	241.158100	.000122
-49.075240	34.571990	241.158200	.000092
-47.932830	34.387580	241.158200	.000092
-46.800740	34.193490	241.158200	.000092
-45.679330	33.990000	241.158100	.000122
-44.568900	33.777440	241.158200	.000092
-89.960660	34.843320	241.134000	.095601
-88.832330	35.057210	241.126200	.111822
-87.692770	35.261540	241.104200	.160537
-86.542320	35.455950	241.072700	.242352
-85.381340	35.640140	241.031200	.369074
-84.210270	35.813810	240.964300	.558673
-83.029580	35.976620	240.861000	.808645
-81.839790	36.128310	240.755700	1.066613
-80.641480	36.268600	240.668000	1.368118
-79.435230	36.397220	240.513000	1.777817
-78.221690	36.513940	240.010500	2.709081
-77.001530	36.618520	238.992400	4.053046
-75.775430	36.710780	237.899300	4.795351
-74.544130	36.790520	237.774200	4.489259
-73.308360	36.857590	238.705900	3.576209

-72.068890	36.911860	239.705900	2.569784
-70.826490	36.953220	240.444800	1.613822
-69.581950	36.981590	240.853900	.883206
-68.336070	36.996900	241.042500	.412830
-67.089650	36.999130	241.118400	.163941
-65.843500	36.988290	241.147900	.050826
-64.598400	36.964370	241.158700	.006628
-63.355190	36.927440	241.161500	.008570
-62.114630	36.877570	241.161000	.010141
-60.877510	36.814860	241.159900	.007609
-59.644590	36.739430	241.159000	.004697
-58.416640	36.651430	241.158600	.002526
-57.194370	36.551040	241.158400	.001232
-55.978490	36.438440	241.158300	.000537
-54.769680	36.313860	241.158200	.000244
-53.568590	36.177530	241.158100	.000149
-52.375820	36.029710	241.158200	.000091
-51.191970	35.870660	241.158200	.000092
-50.017550	35.700660	241.158100	.000122
-48.853080	35.520030	241.158100	.000122
-47.698980	35.329070	241.158200	.000092
-46.555660	35.128100	241.158200	.000092
-45.423470	34.917440	241.158100	.000122
-44.302690	34.697410	241.158200	.000092
-90.232250	35.766320	241.080300	.216449
-89.092860	35.987640	241.046300	.257004
-87.941790	36.199090	240.958800	.365994
-86.779330	36.400320	240.840700	.542274
-85.605870	36.591000	240.698700	.799865
-84.421870	36.770790	240.445500	1.244040
-83.227800	36.939400	240.026500	1.879478
-82.024180	37.096500	239.618400	2.497640
-80.811620	37.241800	239.399700	3.004153
-79.590740	37.375040	239.074300	3.639161
-78.362190	37.495960	237.479900	5.889617
-77.126670	37.604320	233.545700	9.208258
-75.884930	37.699920	228.699800	10.951300
-74.637700	37.782550	228.387300	10.149780
-73.385770	37.852060	232.461500	7.237212
-72.129940	37.908310	236.223700	5.440977
-70.871030	37.951180	238.742100	3.538171
-69.609860	37.980580	240.182800	1.883037
-68.347290	37.996460	240.814400	.859723
-67.084140	37.998780	241.053500	.323647
-65.821270	37.987530	241.139000	.085577
-64.559530	37.962740	241.169800	.012613
-63.299760	37.924460	241.175700	.038081
-62.042780	37.872770	241.171500	.036309
-60.789440	37.807780	241.165600	.025191
-59.540510	37.729610	241.161600	.014779
-58.296810	37.638420	241.159700	.007863
-57.059080	37.534390	241.158800	.003864
-55.828080	37.417740	241.158500	.001780
-54.604500	37.288680	241.158300	.000780
-53.389020	37.147460	241.158200	.000339
-52.182290	36.994370	241.158100	.000164
-50.984880	36.829660	241.158200	.000092
-49.797380	36.653650	241.158100	.000121
-48.620270	36.466650	241.158100	.000122
-47.454020	36.268990	241.158100	.000122
-46.299010	36.060990	241.158200	.000092
-45.155610	35.843010	241.158100	.000122
-44.024070	35.615370	241.158200	.000092
-90.516530	36.687310	240.914800	.439383
-89.365660	36.916220	240.785000	.531284
-88.202590	37.134950	240.471400	.758790
-87.027650	37.343150	240.059400	1.074207

-85.841200	37.540460	239.595400	1.439870
-84.643710	37.726540	238.667100	2.314349
-83.435650	37.901050	237.011000	3.916993
-82.217600	38.063690	235.404400	5.400093
-80.990140	38.214140	234.888700	6.168137
-79.753940	38.352110	234.656300	6.778937
-78.509670	38.477340	230.022600	11.030240
-77.258070	38.589580	217.214700	17.692100
-75.999910	38.688600	197.813700	23.787860
-74.735980	38.774210	198.216700	21.160340
-73.467090	38.846230	213.242400	15.183810
-72.194090	38.904510	226.309800	9.961986
-70.917830	38.948930	234.033600	7.084923
-69.639210	38.979390	238.322600	3.696728
-68.359090	38.995840	240.240400	1.509732
-67.078360	38.998240	240.910300	.508498
-65.797930	38.986590	241.138900	.090032
-64.518690	38.960900	241.223800	.099846
-63.241520	38.921240	241.233700	.138065
-61.967320	38.867690	241.213000	.117696
-60.696920	38.800350	241.188400	.078552
-59.431210	38.719360	241.171900	.044519
-58.170970	38.624900	241.163700	.022861
-56.917050	38.517150	241.160400	.011261
-55.670200	38.396340	241.159100	.005334
-54.431160	38.262690	241.158600	.002429
-53.200640	38.116470	241.158300	.001046
-51.979310	37.957960	241.158200	.000437
-50.767770	37.787460	241.158100	.000181
-49.566610	37.605300	241.158100	.000123
-48.376340	37.411790	241.158100	.000121
-47.197410	37.207270	241.158100	.000122
-46.030250	36.992090	241.158100	.000122
-44.875180	36.766620	241.158100	.000122
-43.732470	36.531200	241.158200	.000092
-90.814130	37.606180	240.487300	.815386
-89.651300	37.842850	240.067000	1.016601
-88.475770	38.069030	239.074100	1.472332
-87.287830	38.284350	237.787300	1.906999
-86.087840	38.488450	236.428000	2.369656
-84.876290	38.680970	233.469500	3.968880
-83.653650	38.861550	227.659000	7.378605
-82.420500	39.029860	221.873700	10.896410
-81.177470	39.185590	220.173000	12.587810
-79.925230	39.328410	221.691300	12.309550
-78.664510	39.458070	213.490200	16.481040
-77.396060	39.574290	183.528600	26.163640
-76.120680	39.676830	142.925400	36.395470
-74.839220	39.765490	130.910900	33.878010
-73.552520	39.840080	162.719600	27.458640
-72.261480	39.900440	202.283200	20.188830
-70.967010	39.946460	223.482500	11.993660
-69.670030	39.978020	234.364600	6.396741
-68.371470	39.995060	239.112700	2.233405
-67.072270	39.997550	240.692200	.555557
-65.773400	39.985470	241.206900	.133138
-64.475770	39.958870	241.442500	.436004
-63.180340	39.917780	241.443500	.456612
-61.888020	39.862300	241.355100	.346598
-60.599740	39.792560	241.267500	.223733
-59.316390	39.708680	241.208800	.125365
-58.038840	39.610860	241.178900	.063750
-56.767930	39.499290	241.166200	.031032
-55.504470	39.374200	241.161400	.015000
-54.249260	39.235840	241.159500	.007058
-53.003000	39.084480	241.158700	.003186
-51.766410	38.920440	241.158400	.001341

-50.540120	38.744010	241.158200	.000534
-49.324710	38.555530	241.158100	.000201
-48.120720	38.355340	241.158100	.000140
-46.928620	38.143810	241.158100	.000122
-45.748810	37.921300	241.158100	.000122
-44.581620	37.688170	241.158100	.000122
-43.427300	37.444810	241.158200	.000094
-91.125630	38.522790	239.610500	1.402827
-89.950410	38.767390	238.469000	1.801401
-88.761920	39.001200	235.737900	2.549182
-87.560450	39.223820	231.837300	3.148952
-86.346370	39.434880	227.954900	4.520037
-85.120160	39.633990	220.630200	8.018281
-83.882300	39.820790	206.524000	13.084510
-82.633390	39.994930	188.508400	19.484350
-81.374080	40.156070	180.812500	25.574950
-80.105060	40.303890	189.786100	22.990380
-78.827100	40.438090	190.569600	18.979980
-77.540980	40.558400	158.733700	25.775090
-76.247540	40.664560	96.009280	34.171900
-74.947690	40.756360	66.854730	29.469630
-73.642300	40.833600	93.480010	32.446010
-72.332310	40.896110	158.067100	30.128630
-71.018690	40.943760	207.072200	18.518050
-69.702430	40.976440	228.928500	8.120348
-68.384490	40.994090	237.807700	2.672527
-67.065880	40.996670	240.478700	.574340
-65.747600	40.984170	241.465700	.437292
-64.430650	40.956610	242.161800	1.328627
-63.116020	40.914060	242.130200	1.421500
-61.804690	40.856610	241.804500	1.000520
-60.497620	40.784390	241.503500	.583695
-59.195770	40.697540	241.322900	.321719
-57.900040	40.596260	241.229000	.165346
-56.611330	40.480750	241.187800	.083061
-55.330490	40.351270	241.170100	.040583
-54.058330	40.208070	241.162800	.019465
-52.795620	40.051450	241.160000	.008980
-51.543080	39.881710	241.158800	.003907
-50.301390	39.699200	241.158400	.001560
-49.071130	39.504250	241.158200	.000574
-47.852850	39.297230	241.158200	.000220
-46.647030	39.078520	241.158100	.000122
-45.454070	38.848490	241.158100	.000122
-44.274280	38.607530	241.158100	.000122
-43.107920	38.356030	241.158200	.000094
-91.451740	39.437030	238.253700	2.187477
-90.263630	39.689750	235.739100	2.789016
-89.061680	39.931370	229.293400	4.047197
-87.846150	40.161480	219.434600	6.007590
-86.617400	40.379670	206.485100	8.088085
-85.375900	40.585540	188.188300	13.339490
-84.122160	40.778730	160.794400	20.587390
-82.856800	40.958850	139.156200	17.890650
-81.580460	41.125550	120.945900	33.313210
-80.293880	41.278500	135.726000	32.681170
-78.997860	41.417370	163.045800	20.259040
-77.693230	41.541890	155.008200	22.512570
-76.380870	41.651780	88.434920	30.724090
-75.061690	41.746810	42.899920	26.531760
-73.736660	41.826770	74.333710	25.452920
-72.406780	41.891490	139.622400	26.708770
-71.073040	41.940830	196.834800	17.585840
-69.736500	41.974670	226.071800	7.740182
-68.398190	41.992950	236.940500	2.988149
-67.059160	41.995620	239.710700	1.849237
-65.720480	41.982670	241.546200	.238213

-64.383220	41.954140	243.853000	2.751708
-63.048400	41.910080	243.988500	3.530036
-61.717090	41.850600	243.034900	2.647107
-60.390290	41.775820	242.155700	1.497261
-59.069010	41.685920	241.624200	.752591
-57.754210	41.581080	241.373500	.394774
-56.446830	41.461530	241.256400	.208403
-55.147770	41.327530	241.200800	.107714
-53.857880	41.179360	241.175100	.052306
-52.577970	41.017320	241.164300	.024085
-51.308780	40.841740	241.160300	.010504
-50.051000	40.652980	241.158900	.004263
-48.805250	40.451390	241.158400	.001587
-47.572090	40.237360	241.158200	.000534
-46.352010	40.011290	241.158100	.000181
-45.145370	39.773560	241.158100	.000120
-43.952510	39.524580	241.158100	.000122
-42.773630	39.264750	241.158200	.000094
-91.793200	40.348750	236.795300	3.210599
-90.591710	40.609810	232.516200	4.524760
-89.375760	40.859430	220.638500	8.399138
-88.145600	41.097210	198.589500	13.469980
-86.901570	41.322720	167.523400	16.639300
-85.644150	41.535550	129.816000	17.386480
-84.373860	41.735290	100.892400	10.174550
-83.091290	41.921560	73.248320	12.877730
-81.797160	42.093980	82.501400	12.664880
-80.492230	42.252200	119.815500	15.959710
-79.177290	42.395880	149.523700	10.952250
-77.853240	42.524730	134.213000	17.848000
-76.521000	42.638450	77.168200	24.159240
-75.181550	42.736820	62.078970	27.785030
-73.835900	42.819590	99.392200	17.738500
-72.485100	42.886590	167.059500	17.179830
-71.130210	42.937670	209.321600	10.849130
-69.772340	42.972710	229.381700	6.096350
-68.412590	42.991630	235.909600	4.415819
-67.052090	42.994400	237.937700	3.993634
-65.691960	42.980990	241.510300	.741222
-64.333320	42.951450	245.393200	4.397865
-62.977290	42.905830	246.993600	6.360744
-61.624970	42.844260	245.716500	5.348469
-60.277440	42.766850	243.608300	3.359223
-58.935750	42.673790	242.384300	1.782739
-57.600940	42.565280	241.746500	.915452
-56.273990	42.441570	241.454700	.501277
-54.955840	42.302930	241.295200	.269630
-53.647390	42.149640	241.215100	.135756
-52.349470	41.982030	241.179200	.062543
-51.062890	41.800450	241.165100	.026611
-49.788320	41.605260	241.160300	.010649
-48.526440	41.396860	241.158900	.003947
-47.277780	41.175640	241.158400	.001341
-46.042850	40.942000	241.158200	.000424
-44.822010	40.696380	241.158100	.000157
-43.615560	40.439180	241.158100	.000121
-42.423690	40.170830	241.158200	.000094
-92.150770	41.257820	235.730500	4.909568
-90.935390	41.527410	229.738900	8.221861
-89.704910	41.785250	213.591500	14.832540
-88.459540	42.030900	183.286700	21.230990
-87.199620	42.263930	138.974400	21.032610
-85.925610	42.483890	87.498310	15.007120
-84.638040	42.690380	46.493830	13.550400
-83.337520	42.882970	40.750720	19.561530
-82.024790	43.061280	88.763290	11.460350
-80.700610	43.224930	176.213100	20.748180

-79.365870 43.373580 204.915700 23.492660
-78.021450 43.506890 172.665000 14.351800
-76.668370 43.624570 100.693700 19.232130
-75.307630 43.726360 97.099520 17.389860
-73.940300 43.812040 159.494300 12.582590
-72.567500 43.881390 197.060800 12.435890
-71.190370 43.934270 217.868800 11.919880
-69.810050 43.970550 228.112900 10.781200
-68.427750 43.990140 236.357100 6.484161
-67.044650 43.993000 239.023300 3.433589
-65.661930 43.979120 242.650000 1.692451
-64.280810 43.948540 247.395800 6.487655
-62.902460 43.901310 249.101600 8.670095
-61.528050 43.837570 248.228300 7.818006
-60.158730 43.757450 246.362800 5.761338
-58.795620 43.661140 244.019800 3.744024
-57.439790 43.548850 242.761400 2.221205
-56.092310 43.420850 241.986400 1.228227
-54.754150 43.277410 241.549500 .645611
-53.426270 43.118850 241.327200 .327149
-52.109530 42.945500 241.222700 .152817
-50.804760 42.757750 241.179600 .064351
-49.512690 42.555960 241.164400 .024715
-48.233980 42.340550 241.159900 .008935
-46.969190 42.111940 241.158700 .003013
-45.718810 41.870550 241.158300 .000916
-44.483210 41.616830 241.158200 .000269
-43.262650 41.351200 241.158100 .000140
-42.057300 41.074100 241.158200 .000092
-92.525320 42.164070 235.680400 5.897475
-91.295520 42.442420 229.416200 10.243350
-90.049940 42.708690 211.762300 19.234040
-88.788760 42.962440 179.726300 24.929260
-87.512310 43.203190 132.417600 25.675290
-86.221000 43.430500 85.013190 23.036940
-84.915410 43.643920 41.117690 11.439180
-83.596150 43.843030 41.802300 19.736730
-82.263960 44.027400 120.028800 27.714850
-80.919660 44.196660 212.895400 33.531200
-79.564150 44.350410 236.312600 30.991760
-78.198390 44.488330 179.347900 14.830080
-76.823410 44.610100 101.545200 22.785810
-75.440290 44.715440 100.157300 20.890190
-74.050180 44.804110 133.922500 24.038680
-72.654240 44.875890 181.753600 21.274070
-71.253690 44.930630 205.778100 22.318960
-69.849760 44.968180 225.311500 16.360120
-68.443720 44.988460 236.506100 7.426125
-67.036810 44.991420 241.427700 1.861911
-65.630330 44.977050 244.729600 3.376096
-64.225530 44.945390 247.673000 7.146213
-62.823690 44.896510 249.320700 8.709859
-61.426040 44.830540 249.312800 8.469546
-60.033810 44.747610 247.843900 7.363540
-58.648170 44.647940 246.711700 6.008676
-57.270290 44.531750 244.750700 4.433921
-55.901260 44.399320 243.192700 2.823440
-54.542130 44.250930 242.170200 1.525794
-53.193900 44.086940 241.596300 .734751
-51.857480 43.907680 241.331700 .339050
-50.533710 43.713560 241.217300 .142429
-49.223370 43.504980 241.175400 .053602
-47.927120 43.282370 241.162600 .018425
-46.645540 43.046150 241.159200 .006018
-45.379100 42.796800 241.158400 .001843
-44.128160 42.534760 241.158200 .000516
-42.892960 42.260480 241.158100 .000164

-41.673610	41.974410	241.158200	.000091
-92.917770	43.067330	236.425400	5.481358
-91.673000	43.354680	230.958800	8.799198
-90.411740	43.629630	215.209400	15.181310
-89.134110	43.891690	183.031200	22.177310
-87.840450	44.140390	134.030800	25.354450
-86.531140	44.375260	79.306820	29.609810
-85.206750	44.595830	52.701370	22.273250
-83.867900	44.801650	75.535140	20.522850
-82.515380	44.992280	121.490200	24.870310
-81.150000	45.167310	178.306900	25.850880
-79.772730	45.326340	182.937800	17.756270
-78.384560	45.469020	170.922700	11.240210
-76.986600	45.595010	143.122900	21.476940
-75.579970	45.704020	131.306000	27.539200
-74.165890	45.795780	165.140300	21.153730
-72.745610	45.870080	186.431700	21.627840
-71.320400	45.926740	209.754500	16.804460
-69.891600	45.965610	226.563900	11.102910
-68.460530	45.986610	236.718000	5.585623
-67.028560	45.989670	240.636200	1.579228
-65.597030	45.974800	243.681900	2.513845
-64.167300	45.942020	246.088200	5.436835
-62.740720	45.891430	247.281800	6.908910
-61.318600	45.823140	247.480000	7.280750
-59.902260	45.737310	247.599800	7.304004
-58.492950	45.634170	247.224200	7.176552
-57.091900	45.513950	246.779400	6.514794
-55.700260	45.376930	245.290900	5.073711
-54.319140	45.223450	243.394200	3.264887
-52.949590	45.053840	242.216200	1.651221
-51.592580	44.868490	241.566900	.697318
-50.248970	44.667810	241.297000	.278540
-48.919560	44.452230	241.198800	.103004
-47.605040	44.222190	241.168400	.034503
-46.305980	43.978160	241.160400	.010711
-45.022840	43.720610	241.158600	.003176
-43.755960	43.450020	241.158200	.000889
-42.505550	43.166850	241.158200	.000246
-41.271660	42.871580	241.158200	.000100
-93.329110	43.967430	237.444200	4.495522
-92.068780	44.264030	233.274100	6.192856
-90.791210	44.547890	221.089600	9.236837
-89.496510	44.818510	192.949500	13.712780
-88.184940	45.075390	139.507700	23.561160
-86.856880	45.318050	72.745070	37.223220
-85.512890	45.545990	60.333340	29.625710
-84.153590	45.758730	98.460530	18.099950
-82.779790	45.955820	126.125500	18.028740
-81.392350	46.136820	121.515000	13.912090
-79.992260	46.301310	129.146800	19.313770
-78.580580	46.448910	155.565400	21.472430
-77.158460	46.579270	168.946000	22.918330
-75.727110	46.692070	189.696900	16.993140
-74.287820	46.787040	196.089200	16.521980
-72.841890	46.863950	214.038000	10.444060
-71.390720	46.922590	225.660100	5.302890
-69.935700	46.962830	232.315400	3.573970
-68.478260	46.984570	238.161500	2.675755
-67.019850	46.987740	242.112900	1.755140
-65.561920	46.972340	244.203000	3.720857
-64.105920	46.938410	246.080800	5.819129
-62.653260	46.886040	246.826500	6.847308
-61.205390	46.815350	246.473300	6.914361
-59.763680	46.726520	246.003200	6.836463
-58.329470	46.619790	246.732700	7.134498
-56.904060	46.495390	247.297700	7.408553

-55.488670 46.353640 247.043300 7.038322
-54.084500 46.194880 245.880600 5.552865
-52.692630 46.019470 243.471400 3.259185
-51.314070 45.827840 242.037100 1.396630
-49.949730 45.620390 241.432300 .490767
-48.600430 45.397590 241.235000 .167533
-47.266860 45.159900 241.177300 .055160
-45.949600 44.907820 241.162300 .016752
-44.649100 44.641840 241.159000 .004785
-43.365670 44.362450 241.158300 .001309
-42.099450 44.070140 241.158200 .000353
-40.850440 43.765410 241.158200 .000128
-93.760410 44.864160 238.374100 3.954759
-92.483920 45.170280 235.136000 5.425617
-91.189420 45.463320 224.906300 8.500648
-89.876960 45.742750 197.791500 14.903800
-88.546760 46.008070 144.679600 27.350360
-87.199170 46.258760 89.426280 40.480320
-85.834720 46.494300 77.165580 40.494080
-84.454070 46.714200 122.679800 24.433560
-83.058010 46.917960 136.181100 21.427050
-81.647480 47.105130 130.542200 29.925450
-80.223460 47.275260 137.481200 34.974300
-78.787090 47.427960 162.497700 32.411220
-77.339580 47.562840 192.526500 21.269040
-75.882230 47.679570 208.763400 14.070440
-74.416370 47.777870 227.057700 8.145495
-72.943440 47.857470 237.751800 6.665147
-71.464880 47.918190 240.001200 6.969404
-69.982220 47.959850 240.298100 3.935641
-68.496960 47.982350 241.518300 2.297621
-67.010670 47.985630 244.457500 3.782865
-65.524890 47.969690 247.010800 6.225530
-64.041170 47.934570 248.366600 7.939577
-62.561040 47.880340 249.141900 8.783468
-61.086020 47.807170 248.694200 8.732520
-59.617590 47.715230 247.488400 8.074310
-58.157170 47.604770 246.673600 7.613733
-56.706150 47.476050 246.990400 7.790804
-55.265830 47.329400 247.917300 8.133057
-53.837470 47.165180 247.332400 7.240183
-52.422200 46.983780 245.053000 4.718919
-51.021090 46.785640 242.635900 2.158638
-49.635090 46.571200 241.583600 .746535
-48.265020 46.340950 241.269300 .229100
-46.911610 46.095370 241.185000 .071256
-45.575410 45.834990 241.164100 .021796
-44.256860 45.560320 241.159300 .006226
-42.956210 45.271870 241.158300 .001683
-41.673580 44.970160 241.158200 .000445
-40.408870 44.655690 241.158200 .000177
-94.212860 45.757310 239.370600 3.014681
-92.919560 46.073230 237.497900 3.897828
-91.607470 46.375730 231.986300 5.400532
-90.276550 46.664250 217.564800 9.890716
-88.926960 46.938270 186.351200 20.280990
-87.559010 47.197250 148.546500 32.772640
-86.173240 47.440650 136.465000 35.816090
-84.770290 47.667940 155.771100 27.168400
-83.350960 47.878600 158.855700 29.564440
-81.916200 48.072160 162.989100 32.583850
-80.467090 48.248140 174.099500 32.886000
-79.004780 48.406110 192.836200 29.194710
-77.530590 48.545680 209.636000 21.888390
-76.045850 48.666500 228.082500 8.842746
-74.552020 48.768250 239.492800 3.965228
-73.050600 48.850660 245.362000 7.167755

-71.543170	48.913520	245.700900	7.052844
-70.031330	48.956650	243.038800	3.924043
-68.516710	48.979950	242.221100	1.885232
-67.000980	48.983350	243.849800	3.086070
-65.485790	48.966840	245.972400	5.403059
-63.972820	48.930470	248.388700	7.359327
-62.463700	48.874330	250.079100	8.960791
-60.960050	48.798580	251.131800	10.119160
-59.463460	48.703410	250.519200	10.127160
-57.975440	48.589080	248.948900	9.305326
-56.497490	48.455880	247.702500	8.626686
-55.030970	48.304140	247.690000	8.387930
-53.577210	48.134270	247.258200	7.354944
-52.137430	47.946660	244.971900	4.840403
-50.712720	47.741790	242.725400	2.260029
-49.304080	47.520120	241.618400	.801619
-47.912350	47.282170	241.272000	.249508
-46.538250	47.028450	241.187000	.076603
-45.182340	46.759510	241.164700	.023198
-43.845020	46.475880	241.159500	.006809
-42.526500	46.178100	241.158400	.001881
-41.226800	45.866700	241.158200	.000492
-39.945740	45.542200	241.158200	.000165
-94.687740	46.646660	240.189700	1.942986
-93.376940	46.972690	239.389400	2.213238
-92.046550	47.284930	237.169300	3.013050
-90.696440	47.582840	229.829700	6.854674
-89.326680	47.865840	214.396400	15.845520
-87.937550	48.133390	196.178900	25.299730
-86.529520	48.384910	183.720600	29.134860
-85.103260	48.619850	183.091400	26.599420
-83.659580	48.837660	187.968900	25.117600
-82.199460	49.037830	199.516200	21.339420
-80.724000	49.219880	212.074400	17.436810
-79.234450	49.383340	219.862700	16.289160
-77.732160	49.527770	229.789900	10.385160
-76.218590	49.652830	237.152200	4.247838
-74.695260	49.758160	241.323100	1.605704
-73.163800	49.843490	243.475000	3.173815
-71.625870	49.908580	243.893700	3.235867
-70.083210	49.953250	243.075400	1.668758
-68.537570	49.977380	242.141600	.981738
-66.990740	49.980900	242.981900	1.553617
-65.444490	49.963800	244.173100	2.897258
-63.900620	49.926140	245.349500	4.067957
-62.360880	49.868010	246.929300	5.756077
-60.827010	49.789570	249.154700	8.162708
-59.300730	49.691040	250.351800	9.862914
-57.783640	49.572690	249.914500	9.877706
-56.277320	49.434830	248.372200	8.796019
-54.783270	49.277820	246.958600	7.521262
-53.302860	49.102080	245.679300	5.862885
-51.837360	48.908040	243.673900	3.585232
-50.387950	48.696200	242.196400	1.639989
-48.955640	48.467040	241.522400	.621416
-47.541310	48.221130	241.275700	.223135
-46.145660	47.959000	241.188200	.074481
-44.769230	47.681210	241.165200	.022897
-43.412380	47.388330	241.159600	.006539
-42.075270	47.080920	241.158500	.001814
-40.757810	46.759540	241.158200	.000487
-39.459720	46.424710	241.158200	.000165
-95.186440	47.531940	240.844500	.744517
-93.857410	47.868410	240.803100	.592679
-92.508000	48.190720	240.149100	1.015386
-91.137910	48.498310	236.687100	4.033619
-89.747150	48.790600	229.168600	11.265720

-88.335950	49.067010	219.666700	18.903700
-86.904710	49.326930	213.114600	21.502900
-85.454100	49.569790	211.007200	18.902210
-83.984930	49.795020	216.540100	15.457900
-82.498220	50.002060	224.711200	10.873430
-80.995120	50.190400	231.382900	6.959923
-79.476920	50.359550	235.756700	5.186632
-77.945060	50.509060	239.146100	3.290196
-76.401090	50.638520	240.929400	1.501773
-74.846650	50.747580	241.161300	1.094198
-73.283450	50.835950	241.291800	1.211309
-71.713320	50.903350	241.955300	.924741
-70.138080	50.949630	242.245200	.737032
-68.559640	50.974620	242.552300	.646713
-66.979900	50.978270	242.916200	1.170846
-65.400800	50.960560	242.944300	1.491824
-63.824260	50.921550	242.647000	1.460801
-62.252170	50.861340	243.174200	2.335159
-60.686400	50.780110	245.426200	4.918246
-59.128770	50.678080	247.714100	7.536308
-57.581030	50.555550	248.089900	8.290129
-56.044860	50.412850	246.859500	7.221744
-54.521820	50.250360	245.195200	5.439733
-53.013420	50.068520	243.601500	3.623918
-51.520960	49.867810	242.418300	1.985781
-50.045680	49.648740	241.700000	.913743
-48.588630	49.411830	241.366200	.388677
-47.150700	49.157670	241.226100	.151968
-45.732590	48.886830	241.177400	.052690
-44.334810	48.599900	241.163000	.016507
-42.957660	48.297460	241.159300	.005015
-41.601190	47.980120	241.158400	.001444
-40.265250	47.648430	241.158200	.000401
-38.949390	47.302930	241.158200	.000145
-95.710510	48.412890	241.421200	.554625
-94.362470	48.760130	241.784000	1.043254
-92.993230	49.092850	241.636700	1.017106
-91.602360	49.410460	240.037200	1.592335
-90.189750	49.712350	236.760600	5.544691
-88.755540	49.997940	233.466000	9.392365
-87.300090	50.266570	230.752700	10.965300
-85.824040	50.517650	230.428800	10.491220
-84.328210	50.750570	232.994600	8.326655
-82.813610	50.964740	235.917400	5.440181
-81.281460	51.159630	238.771300	2.935842
-79.733130	51.334700	241.085300	1.382445
-78.170130	51.489480	242.247200	.661348
-76.594090	51.623530	241.778900	.527862
-75.006800	51.736490	240.570700	1.621472
-73.410070	51.828010	240.178000	2.286118
-71.805870	51.897850	240.798600	1.783869
-70.196170	51.945790	241.689600	1.279977
-68.582990	51.971680	242.272300	.832431
-66.968440	51.975460	242.909000	1.004938
-65.354560	51.957120	242.537600	1.059421
-63.743440	51.916690	241.806700	.636173
-62.137130	51.854320	241.814500	1.032412
-60.537630	51.770170	243.522400	3.049680
-58.946900	51.664510	245.231500	5.253428
-57.366820	51.537620	245.551300	5.782463
-55.799180	51.389870	244.430100	4.702364
-54.245650	51.221680	243.216800	3.142498
-52.707820	51.033510	242.271200	1.815398
-51.187080	50.825860	241.701900	.935618
-49.684720	50.599280	241.395400	.448293
-48.201800	50.354330	241.252300	.201206
-46.739240	50.091630	241.189900	.081236

-45.297720	49.811770	241.167600	.029177
-43.877700	49.515370	241.160700	.009921
-42.479410	49.203060	241.158700	.003170
-41.102810	48.875440	241.158300	.000942
-39.747590	48.533100	241.158200	.000277
-38.413150	48.176570	241.158200	.000108
-96.261670	49.289190	241.883000	1.213004
-94.893750	49.647600	242.115800	1.599097
-93.503820	49.991080	242.114800	1.394583
-92.091320	50.319060	241.144500	.725599
-90.655950	50.630910	239.961900	2.278552
-89.197760	50.925990	238.809500	3.776885
-87.717060	51.203660	238.047200	4.680063
-86.214420	51.463270	238.161000	4.687422
-84.690660	51.704190	239.055900	3.718458
-83.146820	51.925780	240.052300	2.500751
-81.584160	52.127480	241.189200	1.186101
-80.004110	52.308710	242.698400	.736527
-78.408280	52.468990	242.745800	1.094553
-76.798410	52.607840	241.629800	.927799
-75.176390	52.724850	240.189500	2.075398
-73.544200	52.819690	239.406300	2.950845
-71.903920	52.892050	240.083300	2.518882
-70.257710	52.941730	240.926300	1.891454
-68.607750	52.968570	241.630500	1.418874
-66.956280	52.972490	242.200700	.741833
-65.305560	52.953480	241.906100	.465333
-63.657820	52.911580	241.473200	.402437
-62.015270	52.846950	241.508800	.852110
-60.380080	52.759750	242.670600	2.311172
-58.754360	52.650280	243.623300	3.546965
-57.140120	52.518850	243.484000	3.525017
-55.539290	52.365840	242.783900	2.573710
-53.953660	52.191710	242.034000	1.532117
-52.384890	51.996940	241.599600	.816369
-50.834480	51.782070	241.370500	.417446
-49.303730	51.547700	241.251700	.204263
-47.793770	51.294400	241.194500	.092344
-46.305490	51.022820	241.170700	.037779
-44.839570	50.733620	241.162100	.014398
-43.396390	50.427420	241.159300	.005124
-41.976090	50.104880	241.158500	.001691
-40.578500	49.766630	241.158200	.000508
-39.203150	49.413260	241.158100	.000181
-37.849210	49.045330	241.158200	.000096
-96.841810	50.160530	241.756600	1.095959
-95.453030	50.530490	241.957100	1.266846
-94.041510	50.885130	241.752000	.985741
-92.606450	51.223850	241.317200	.419705
-91.147380	51.546020	240.886000	.876135
-89.664200	51.850960	240.521100	1.460873
-88.157130	52.138020	240.298600	1.826715
-86.626690	52.406500	240.334900	1.931963
-85.073680	52.655720	240.641900	1.601699
-83.499180	52.885050	241.005100	1.213071
-81.904440	53.093850	241.679800	.487520
-80.290990	53.281520	242.493800	1.083977
-78.660530	53.447530	242.584800	1.659369
-77.014920	53.591380	241.668800	1.444307
-75.356160	53.712650	240.232600	2.173791
-73.686420	53.810940	239.146800	3.182423
-72.007930	53.885960	239.566000	2.939980
-70.322990	53.937460	240.439100	2.273855
-68.634010	53.965280	240.938500	2.059262
-66.943400	53.969350	241.287100	1.414361
-65.253590	53.949630	241.363400	.543285
-63.567000	53.906200	241.345900	.396647

-61.886040	53.839190	241.763800	1.141243
-60.213040	53.748820	242.418300	2.024109
-58.550290	53.635360	242.654100	2.380542
-56.899960	53.499180	242.373800	1.982953
-55.264110	53.340690	241.842800	1.253414
-53.644650	53.160350	241.503400	.678860
-52.043340	52.958710	241.320700	.349426
-50.461760	52.736320	241.232800	.179218
-48.901280	52.493820	241.189900	.086394
-47.363030	52.231850	241.170500	.038721
-45.847900	51.951060	241.162500	.016245
-44.356530	51.652150	241.159600	.006403
-42.889220	51.335790	241.158600	.002329
-41.445980	51.002650	241.158300	.000776
-40.026490	50.653380	241.158200	.000254
-38.630060	50.288590	241.158100	.000140
-37.255620	49.908830	241.158200	.000091
-97.453060	51.026530	241.460800	.593291
-96.042340	51.408490	241.514500	.625958
-94.608220	51.774700	241.393100	.425619
-93.149600	52.124570	241.234000	.167816
-91.665800	52.457450	241.102500	.310479
-90.156560	52.772640	240.994400	.529007
-88.621980	53.069450	240.932500	.680832
-87.062480	53.347160	240.940800	.733835
-85.478850	53.605050	240.998900	.711284
-83.872130	53.842430	241.083900	.629871
-82.243660	54.058630	241.346000	.310206
-80.595020	54.253030	241.926900	1.062650
-78.928000	54.425040	242.369400	1.835857
-77.244600	54.574130	241.856200	1.770077
-75.546970	54.699840	240.558100	2.015841
-73.837420	54.801750	239.264400	3.091640
-72.118360	54.879540	239.195400	3.197382
-70.392330	54.932960	240.021400	2.572164
-68.661900	54.961820	240.281100	2.429023
-66.929700	54.966030	240.666500	1.859159
-65.198370	54.945580	241.077600	.599108
-63.470540	54.900540	241.655900	.728315
-61.748820	54.831050	242.138700	1.526938
-60.035740	54.737340	242.269300	1.798800
-58.333780	54.619720	242.086000	1.545914
-56.645260	54.478570	241.691800	1.012366
-54.972420	54.314330	241.431800	.555624
-53.317280	54.127510	241.285400	.284963
-51.681740	53.918690	241.215400	.147690
-50.067420	53.688470	241.182600	.073165
-48.475770	53.437500	241.168100	.034379
-46.907930	53.166490	241.162000	.015374
-45.364770	52.876130	241.159500	.006535
-43.846840	52.567130	241.158600	.002596
-42.354370	52.240220	241.158300	.000946
-40.887210	51.896080	241.158200	.000338
-39.444820	51.535370	241.158100	.000149
-38.026250	51.158730	241.158100	.000121
-36.630120	50.766700	241.158200	.000092
-98.097820	51.886770	241.265100	.222917
-96.663910	52.281200	241.266700	.218570
-95.206040	52.659450	241.229300	.129472
-93.722790	53.020900	241.185100	.049659
-92.213200	53.364910	241.147700	.101128
-90.676770	53.690770	241.118300	.176481
-89.113440	53.997730	241.101300	.230180
-87.523560	54.285050	241.099100	.257636
-85.907830	54.551970	241.100200	.279767
-84.267300	54.797760	241.089400	.302445
-82.603340	55.021710	241.132100	.326598

-80.917590	55.223140	241.529600	.887458
-79.211960	55.401440	242.087200	1.718322
-77.488560	55.556020	242.025000	1.930991
-75.749730	55.686380	240.981600	1.881087
-73.997920	55.792100	239.561200	2.873731
-72.235800	55.872810	238.909700	3.434675
-70.466090	55.928230	239.498600	3.001849
-68.691570	55.958180	239.836100	2.704557
-66.915130	55.962550	240.344700	1.783694
-65.139640	55.941330	241.279900	.196152
-63.367970	55.894590	242.046100	1.209835
-61.602940	55.822490	242.207500	1.640311
-59.847310	55.725280	242.017600	1.395556
-58.103760	55.603290	241.618600	.881049
-56.374820	55.456930	241.386400	.466442
-54.662870	55.286670	241.262300	.233881
-52.970100	55.093080	241.204400	.121504
-51.298500	54.876750	241.177300	.060482
-49.649800	54.638340	241.165800	.028752
-48.025460	54.378550	241.161200	.013202
-46.426660	54.098120	241.159300	.005876
-44.854210	53.797780	241.158600	.002461
-43.308580	53.478290	241.158300	.000970
-41.789850	53.140400	241.158200	.000363
-40.297680	52.784830	241.158100	.000164
-38.831280	52.412250	241.158100	.000121
-37.389370	52.023280	241.158100	.000121
-35.970190	51.618480	241.158200	.000092
-98.778840	52.740810	241.191300	.072729
-97.320270	53.148240	241.187700	.062927
-95.837370	53.539010	241.177500	.036300
-94.328290	53.912530	241.166000	.014013
-92.791720	54.268120	241.155900	.031368
-91.226900	54.605080	241.148200	.054623
-89.633560	54.922620	241.143300	.072275
-88.011890	55.219970	241.140500	.082599
-86.362530	55.496330	241.136300	.089723
-84.686490	55.750910	241.126300	.125361
-82.985150	55.982970	241.136500	.259053
-81.260250	56.191770	241.299900	.666951
-79.513790	56.376650	241.854300	1.488618
-77.748020	56.536990	242.066400	1.949834
-75.965460	56.672260	241.360900	1.874572
-74.168790	56.781970	239.890500	2.619708
-72.360860	56.865740	238.795900	3.599596
-70.544630	56.923280	239.003500	3.515959
-68.723180	56.954370	239.519900	2.848355
-66.899620	56.958910	240.506700	1.345940
-65.077100	56.936880	241.645500	.491377
-63.258730	56.888360	242.094300	1.383689
-61.447630	56.813510	242.024300	1.345341
-59.646790	56.712620	241.613800	.856387
-57.859090	56.586040	241.372600	.430947
-56.087300	56.434210	241.251300	.203797
-54.333970	56.257640	241.198400	.103482
-52.601470	56.056940	241.174500	.050932
-50.891880	55.832740	241.164400	.024031
-49.207030	55.585770	241.160500	.011014
-47.548420	55.316760	241.159100	.004970
-45.917200	55.026500	241.158500	.002151
-44.314130	54.715760	241.158300	.000876
-42.739580	54.385350	241.158200	.000350
-41.193420	54.036030	241.158100	.000164
-39.675070	53.668560	241.158100	.000121
-38.183400	53.283620	241.158100	.000121
-36.716770	52.881820	241.158100	.000122
-35.272900	52.463700	241.158200	.000092

-99.499240	53.588130	241.167500	.023024
-98.014280	54.009130	241.165600	.017809
-96.504860	54.412960	241.162900	.010345
-94.968610	54.799050	241.160000	.004193
-93.403790	55.166730	241.157400	.009341
-91.809290	55.515250	241.155500	.015884
-90.184560	55.843850	241.153900	.021053
-88.529620	56.151670	241.152300	.024229
-86.844990	56.437890	241.150500	.024180
-85.131650	56.701690	241.151400	.047550
-83.390960	56.942230	241.170200	.168014
-81.624720	57.158780	241.276200	.499971
-79.835050	57.350580	241.641700	1.195580
-78.024370	57.516980	242.039800	1.859375
-76.195360	57.657400	241.564200	1.881196
-74.350940	57.771310	240.251000	2.386892
-72.494220	57.858320	238.869100	3.614106
-70.628410	57.918090	238.632300	3.886482
-68.756890	57.950390	239.422700	2.842787
-66.883060	57.955100	240.841300	.925863
-65.010370	57.932220	241.733200	.748773
-63.142240	57.881810	241.923000	1.135592
-61.282040	57.804080	241.606800	.819009
-59.433060	57.699310	241.376000	.424805
-57.598450	57.567890	241.250800	.194194
-55.781200	57.410300	241.197000	.095234
-53.984060	57.227100	241.173300	.045538
-52.209560	57.018940	241.163700	.020933
-50.459930	56.786500	241.160100	.009373
-48.737060	56.530560	241.158900	.004187
-47.042490	56.251900	241.158500	.001804
-45.377330	55.951360	241.158300	.000747
-43.742270	55.629770	241.158200	.000303
-42.137470	55.287960	241.158100	.000157
-40.562610	54.926740	241.158100	.000121
-39.016750	54.546860	241.158100	.000121
-37.498410	54.149020	241.158100	.000122
-36.005400	53.733830	241.158100	.000122
-34.534880	53.301770	241.158200	.000092
-100.262700	54.428120	241.160500	.006810
-98.749250	54.863350	241.159800	.004895
-97.211560	55.280850	241.159100	.002777
-95.646590	55.680060	241.158400	.001244
-94.052090	56.060360	241.157800	.002631
-92.426510	56.420970	241.157400	.004303
-90.768940	56.761100	241.156900	.005495
-89.079190	57.079890	241.156400	.005780
-87.357570	57.376450	241.156300	.007374
-85.605000	57.649890	241.160800	.032772
-83.822840	57.899360	241.184500	.117635
-82.012920	58.124030	241.269000	.353499
-80.177480	58.323120	241.522200	.901263
-78.319110	58.495910	241.902800	1.609912
-76.440710	58.641750	241.737400	1.839377
-74.545430	58.760110	240.560300	2.131252
-72.636660	58.850530	239.073900	3.443132
-70.717930	58.912650	238.461300	3.987951
-68.792920	58.946230	239.493100	2.853240
-66.865380	58.951130	240.912700	.837696
-64.939080	58.927340	241.619500	.621822
-63.017780	58.874950	241.515900	.644116
-61.105210	58.794160	241.364000	.385810
-59.204930	58.685300	241.256600	.191210
-57.320400	58.548780	241.200400	.096159
-55.454840	58.385130	241.174400	.044937
-53.611270	58.194950	241.163800	.019851
-51.792370	57.978940	241.160100	.008571

-50.000500 57.737840 241.158800 .003724
-48.237630 57.472490 241.158400 .001571
-46.505310 57.183730 241.158200 .000648
-44.804610 56.872430 241.158200 .000267
-43.136050 56.539500 241.158100 .000149
-41.499610 56.185780 241.158100 .000121
-39.894630 55.812120 241.158100 .000122
-38.319800 55.419290 241.158100 .000122
-36.773090 55.007980 241.158100 .000122
-35.251730 54.578750 241.158100 .000122
-33.752120 54.132060 241.158200 .000092
-101.073700 55.260130 241.158600 .001829
-99.529010 55.710320 241.158400 .001264
-97.960900 56.142140 241.158300 .000692
-96.365430 56.555100 241.158200 .000367
-94.739640 56.948590 241.158100 .000705
-93.081440 57.321860 241.157900 .001065
-91.389490 57.674070 241.157900 .001158
-89.663250 58.004340 241.157800 .001200
-87.902820 58.311730 241.158300 .006025
-86.109000 58.595320 241.162500 .024995
-84.283100 58.854170 241.180800 .080851
-82.427000 59.087400 241.241300 .232738
-80.543050 59.294160 241.405800 .609786
-78.634010 59.473690 241.776700 1.260240
-76.703000 59.625280 241.756500 1.562088
-74.753460 59.748340 240.827300 1.757166
-72.789070 59.842370 239.291300 2.997448
-70.813740 59.906970 238.557700 3.890513
-68.831480 59.941910 239.309700 2.971190
-66.846440 59.947010 240.787000 1.147925
-64.862770 59.922250 241.214900 .265081
-62.884610 59.867760 241.267300 .225900
-60.916040 59.783740 241.233300 .141513
-58.961010 59.670550 241.201500 .088396
-57.023270 59.528640 241.178300 .046996
-55.106350 59.358580 241.165400 .021217
-53.213500 59.161040 241.160500 .008812
-51.347600 58.936760 241.158800 .003648
-49.511150 58.686560 241.158400 .001487
-47.706190 58.411320 241.158200 .000588
-45.934240 58.111950 241.158200 .000242
-44.196280 57.789400 241.158100 .000144
-42.492650 57.444580 241.158100 .000121
-40.823030 57.078400 241.158100 .000122
-39.186360 56.691730 241.158100 .000122
-37.580830 56.285340 241.158100 .000122
-36.003750 55.859910 241.158100 .000122
-34.451600 55.415950 241.158100 .000122
-32.919830 54.953830 241.158200 .000092
-101.937200 56.083360 241.158300 .000455
-100.358100 56.549350 241.158200 .000333
-98.756940 56.996240 241.158200 .000201
-97.128790 57.423640 241.158100 .000156
-95.469860 57.830970 241.158100 .000213
-93.777320 58.217490 241.158100 .000246
-92.049300 58.582370 241.158100 .000214
-90.284800 58.924680 241.158200 .001058
-88.483640 59.243460 241.158600 .004625
-86.646400 59.537720 241.161500 .016359
-84.774360 59.806450 241.172800 .050136
-82.869400 60.048700 241.208800 .136392
-80.933970 60.263580 241.307900 .355911
-78.971000 60.450230 241.526100 .752096
-76.983890 60.607900 241.576500 .960439
-74.976370 60.735940 240.670200 1.118051
-72.952470 60.833800 239.459700 2.436972

-70.916480 60.901050 238.494400 3.594454
-68.872850 60.937420 238.987400 3.291515
-66.826130 60.942730 240.215500 1.629910
-64.780930 60.916960 240.917500 .476034
-62.741830 60.860230 241.115500 .106823
-60.713310 60.772790 241.161500 .034984
-58.699720 60.654990 241.167600 .026356
-56.705200 60.507380 241.164100 .016589
-54.733590 60.330550 241.160600 .008216
-52.788400 60.125220 241.158900 .003578
-50.872730 59.892220 241.158400 .001472
-48.989200 59.632420 241.158200 .000574
-47.139900 59.346780 241.158200 .000230
-45.326320 59.036270 241.158100 .000127
-43.549270 58.701890 241.158100 .000124
-41.808860 58.344600 241.158100 .000122
-40.104370 57.965370 241.158100 .000125
-38.434220 57.565060 241.158100 .000125
-36.795950 57.144450 241.158100 .000122
-35.186060 56.704150 241.158100 .000122
-33.600030 56.244660 241.158100 .000122
-32.032190 55.766200 241.158200 .000092
-102.859900 56.896880 241.158200 .000126
-101.242000 57.379640 241.158100 .000144
-99.604390 57.842470 241.158100 .000120
-97.940930 58.285070 241.158100 .000124
-96.246660 58.706940 241.158100 .000135
-94.517820 59.107400 241.158100 .000136
-92.751850 59.485580 241.158100 .000185
-90.947170 59.840560 241.158200 .000745
-89.103220 60.171320 241.158500 .002702
-87.220290 60.476800 241.159900 .008789
-85.299550 60.755960 241.165000 .025230
-83.342830 61.007760 241.179600 .062185
-81.352710 61.231220 241.210300 .133481
-79.332320 61.425420 241.241200 .232596
-77.285290 61.589540 241.073900 .247231
-75.215710 61.722870 240.357600 .991123
-73.128000 61.824790 239.284100 2.205243
-71.026890 61.894870 238.308700 3.403630
-68.917300 61.932760 238.377200 3.506830
-66.804310 61.938290 239.720900 2.139227
-64.692990 61.911450 240.693800 .795611
-62.588420 61.852340 241.028000 .215523
-60.495590 61.761240 241.119900 .059308
-58.419300 61.638580 241.146600 .016327
-56.364090 61.484900 241.155400 .003507
-54.334160 61.300890 241.157700 .001286
-52.333320 61.087320 241.158100 .000876
-50.364880 60.845090 241.158100 .000444
-48.431600 60.575160 241.158100 .000199
-46.535590 60.278550 241.158100 .000129
-44.678250 59.956310 241.158100 .000125
-42.860180 59.609490 241.158100 .000128
-41.081100 59.239120 241.158100 .000128
-39.339830 58.846180 241.158100 .000128
-37.634110 58.431540 241.158100 .000128
-35.960610 57.995940 241.158100 .000128
-34.314830 57.539980 241.158100 .000122
-32.690990 57.064010 241.158100 .000122
-31.082000 56.568130 241.158200 .000092
-103.849600 57.699570 241.158200 .000091
-102.187300 58.200280 241.158100 .000120
-100.509000 58.680030 241.158100 .000122
-98.806840 59.138710 241.158100 .000122
-97.074520 59.575940 241.158100 .000122
-95.307120 59.991050 241.158100 .000121

-93.501110	60.383240	241.158100	.000144
-91.654170	60.751550	241.158200	.000405
-89.765210	61.094930	241.158300	.001289
-87.834170	61.412270	241.158700	.003765
-85.861950	61.702450	241.160400	.009882
-83.850420	61.964360	241.164200	.021029
-81.802150	62.196920	241.166700	.032126
-79.720490	62.399150	241.141500	.041708
-77.609360	62.570130	240.984600	.291016
-75.473210	62.709080	240.376100	1.066336
-73.316960	62.815350	239.505200	2.060348
-71.145790	62.888430	238.500000	3.017062
-68.965200	62.927950	238.218800	3.306192
-66.780790	62.933720	239.490800	2.156476
-64.598260	62.905720	240.598400	.872460
-62.423240	62.844070	240.993900	.261500
-60.261290	62.749080	241.109600	.078611
-58.117710	62.621230	241.143800	.024872
-55.997520	62.461110	241.154300	.006894
-53.905320	62.269450	241.157200	.001486
-51.845240	62.047150	241.157800	.000256
-49.820820	61.795150	241.158000	.000116
-47.834930	61.514500	241.158100	.000133
-45.889670	61.206310	241.158100	.000125
-43.986290	60.871700	241.158100	.000128
-42.125070	60.511780	241.158100	.000128
-40.305270	60.127630	241.158100	.000128
-38.525020	59.720250	241.158100	.000128
-36.781200	59.290500	241.158100	.000128
-35.069400	58.839090	241.158100	.000128
-33.383770	58.366500	241.158100	.000122
-31.716990	57.872960	241.158100	.000122
-30.060130	57.358350	241.158200	.000092
-104.916500	58.490070	241.158200	.000092
-103.202300	59.010120	241.158200	.000092
-101.477600	59.507980	241.158100	.000122
-99.732440	59.983750	241.158100	.000122
-97.958720	60.437220	241.158100	.000122
-96.150010	60.867820	241.158200	.000094
-94.301540	61.274790	241.158100	.000123
-92.410060	61.657170	241.158100	.000197
-90.473700	62.013880	241.158200	.000512
-88.491920	62.343760	241.158300	.001286
-86.465330	62.645610	241.158600	.002763
-84.395630	62.918240	241.158800	.004143
-82.285480	63.160470	241.155300	.007889
-80.138370	63.371240	241.133200	.048118
-77.958560	63.549540	241.043100	.207996
-75.750930	63.694510	240.782900	.629828
-73.520860	63.805420	240.225400	1.311632
-71.274150	63.881700	239.462700	2.058980
-69.016910	63.922960	239.129000	2.282963
-66.755400	63.928990	239.958100	1.545615
-64.495980	63.899750	240.724000	.634723
-62.244980	63.835380	241.029300	.202820
-60.008560	63.736250	241.121800	.062754
-57.792640	63.602840	241.148100	.019275
-55.602750	63.435840	241.155500	.005545
-53.443960	63.236060	241.157400	.001493
-51.320750	63.004450	241.157900	.000406
-49.236890	62.742080	241.158100	.000146
-47.195330	62.450080	241.158100	.000136
-45.198120	62.129630	241.158100	.000110
-43.246250	61.781950	241.158100	.000128
-41.339580	61.408210	241.158100	.000128
-39.476710	61.009530	241.158100	.000128
-37.654880	60.586900	241.158200	.000079

-35.869840	60.141170	241.158100	.000128
-34.115770	59.672970	241.158100	.000125
-32.385130	59.182660	241.158100	.000122
-30.668580	58.670220	241.158200	.000067
-28.954870	58.135290	241.158200	.000092
-106.073800	59.266680	241.158200	.000092
-104.297500	59.807790	241.158200	.000092
-102.518800	60.325180	241.158200	.000092
-100.725000	60.819250	241.158100	.000122
-98.905530	61.289990	241.158100	.000122
-97.052100	61.737010	241.158200	.000092
-95.158340	62.159620	241.158200	.000094
-93.219700	62.556880	241.158100	.000127
-91.233350	62.927700	241.158100	.000201
-89.198040	63.270870	241.158200	.000314
-87.113920	63.585110	241.158200	.000456
-84.982480	63.869130	241.157800	.001473
-82.806390	64.121680	241.155100	.009242
-80.589290	64.341560	241.143300	.036611
-78.335770	64.527670	241.105200	.106620
-76.051160	64.679080	241.004400	.275033
-73.741460	64.794950	240.802400	.569627
-71.413090	64.874680	240.502200	.933433
-69.072890	64.917820	240.255100	1.115323
-66.727910	64.924110	240.639500	.738252
-64.385280	64.893550	240.954300	.312470
-62.052090	64.826280	241.091800	.109000
-59.735260	64.722690	241.138600	.036696
-57.441380	64.583340	241.152800	.011794
-55.176610	64.408980	241.156700	.003612
-52.946510	64.200520	241.157700	.001077
-50.755950	63.959000	241.158000	.000329
-48.608900	63.685590	241.158100	.000163
-46.508400	63.381530	241.158100	.000127
-44.456330	63.048090	241.158200	.000095
-42.453320	62.686580	241.158200	.000079
-40.498620	62.298210	241.158100	.000128
-38.589950	61.884150	241.158100	.000125
-36.723370	61.445380	241.158200	.000067
-34.893150	60.982680	241.158200	.000067
-33.091610	60.496560	241.158100	.000122
-31.309040	59.987160	241.158100	.000122
-29.533510	59.454220	241.158200	.000094
-27.750760	58.896990	241.158200	.000092
-107.339200	60.027230	241.158200	.000092
-105.487000	60.591610	241.158200	.000092
-103.643800	61.130270	241.158200	.000092
-101.793500	61.644070	241.158200	.000092
-99.922550	62.133320	241.158100	.000122
-98.020040	62.597820	241.158200	.000092
-96.077510	63.037030	241.158200	.000092
-94.088710	63.450070	241.158200	.000094
-92.049460	63.835860	241.158100	.000121
-89.957580	64.193130	241.158200	.000086
-87.812570	64.520550	241.158100	.000346
-85.615560	64.816710	241.157800	.001593
-83.369120	65.080260	241.156500	.006238
-81.077050	65.309890	241.151800	.020204
-78.744270	65.504390	241.137200	.053248
-76.376630	65.662700	241.102700	.114669
-73.980770	65.783920	241.038600	.212363
-71.563890	65.867360	240.949600	.332877
-69.133670	65.912500	240.890600	.398674
-66.698060	65.919100	240.986400	.267451
-64.265090	65.887100	241.082200	.125028
-61.842790	65.816700	241.130600	.051814
-59.438900	65.708320	241.149600	.019391

-57.060850	65.562580	241.155700	.006650
-54.715470	65.380330	241.157400	.002165
-52.408880	65.162570	241.158000	.000704
-50.146370	64.910470	241.158100	.000242
-47.932120	64.625290	241.158200	.000126
-45.769150	64.308400	241.158100	.000132
-43.659080	63.961160	241.158200	.000073
-41.602020	63.584970	241.158200	.000067
-39.596350	63.181100	241.158200	.000067
-37.638600	62.750720	241.158100	.000122
-35.723270	62.294770	241.158200	.000067
-33.842640	61.813940	241.158200	.000067
-31.986660	61.308540	241.158200	.000094
-30.142740	60.778430	241.158100	.000122
-28.295590	60.222970	241.158200	.000094
-26.427080	59.640870	241.158200	.000092
-108.736700	60.768890	241.158200	.000092
-106.789100	61.359350	241.158200	.000092
-104.866900	61.921510	241.158200	.000092
-102.949500	62.456830	241.158200	.000092
-101.019200	62.966030	241.158200	.000092
-99.061880	63.449270	241.158200	.000092
-97.066180	63.906190	241.158200	.000092
-95.023590	64.336020	241.158200	.000092
-92.928180	64.737730	241.158200	.000073
-90.776390	65.110020	241.158200	.000140
-88.566850	65.451470	241.158100	.000459
-86.300130	65.760610	241.157900	.001543
-83.978550	66.035940	241.157200	.004649
-81.606060	66.276030	241.154900	.012827
-79.187900	66.479550	241.148300	.031080
-76.730480	66.645290	241.133300	.063015
-74.241170	66.772270	241.108000	.105505
-71.728080	66.859700	241.078000	.145988
-69.199870	66.907030	241.061200	.165402
-66.665550	66.913940	241.090400	.117808
-64.134220	66.880400	241.123700	.066205
-61.614990	66.806620	241.144400	.031154
-59.116610	66.693070	241.153500	.012649
-56.647430	66.540450	241.156800	.004650
-54.215080	66.349720	241.157800	.001648
-51.826330	66.121990	241.158100	.000574
-49.486860	65.858530	241.158100	.000204
-47.201050	65.560780	241.158200	.000106
-44.971820	65.230190	241.158200	.000079
-42.800360	64.868270	241.158200	.000073
-40.685980	64.476460	241.158200	.000067
-38.625890	64.056100	241.158200	.000067
-36.614990	63.608330	241.158200	.000067
-34.645670	63.134020	241.158200	.000094
-32.707610	62.633670	241.158200	.000094
-30.787590	62.107330	241.158200	.000094
-28.869240	61.554470	241.158200	.000092
-26.932890	60.973880	241.158200	.000092
-24.955330	60.363580	241.158200	.000092

Q.10a

-89.453400	32.991660	254.044800	.000281
-88.345930	33.191160	254.044700	.000374
-87.228100	33.381660	254.044500	.000717
-86.100230	33.562890	254.044300	.001070
-84.962690	33.734530	254.044200	.001671
-83.815900	33.896310	254.043000	.003181
-82.660300	34.047950	254.039800	.006234
-81.496410	34.189190	254.036300	.009921
-80.324730	34.319790	254.034300	.014163
-79.145840	34.439500	254.028600	.024773
-77.960310	34.548100	254.002500	.052105
-76.768760	34.645400	253.941700	.104931
-75.571840	34.731200	253.872100	.160229
-74.370180	34.805370	253.858400	.172496
-73.164480	34.867740	253.916900	.132440
-71.955430	34.918200	253.985200	.076389
-70.743730	34.956650	254.024800	.032302
-69.530080	34.983020	254.039500	.010036
-68.315220	34.997260	254.043500	.002308
-67.099880	34.999340	254.044600	.000539
-65.884770	34.989250	254.044800	.000181
-64.670640	34.967020	254.044800	.000129
-63.458190	34.932690	254.044900	.000096
-62.248150	34.886320	254.044900	.000107
-61.041230	34.828000	254.044900	.000107
-59.838120	34.757850	254.044900	.000107
-58.639500	34.676010	254.044900	.000107
-57.446040	34.582610	254.044900	.000107
-56.258390	34.477850	254.044900	.000107
-55.077160	34.361910	254.044900	.000107
-53.902950	34.235010	254.044900	.000107
-52.736340	34.097380	254.044900	.000107
-51.577860	33.949260	254.044900	.000107
-50.428010	33.790910	254.044900	.000107
-49.287250	33.622600	254.044900	.000107
-48.156040	33.444620	254.044900	.000107
-47.034740	33.257250	254.044900	.000107
-45.923690	33.060790	254.044900	.000107
-44.823220	32.855560	254.044900	.000107
-89.701210	33.918400	254.044000	.001610
-88.583520	34.125020	254.043400	.002241
-87.455040	34.322360	254.041700	.004094
-86.316120	34.510110	254.040400	.006376
-85.167100	34.687960	254.039100	.009551
-84.008420	34.855620	254.032700	.016050
-82.840550	35.012790	254.017100	.027083
-81.663990	35.159200	253.998900	.040718
-80.479300	35.294580	253.986500	.056751
-79.287040	35.418700	253.959200	.090672
-78.087840	35.531310	253.849100	.181752
-76.882320	35.632210	253.588400	.359157
-75.671150	35.721200	253.272700	.551420
-74.455030	35.798110	253.207100	.576463
-73.234660	35.862810	253.452700	.439601
-72.010770	35.915150	253.744800	.263265

-70.784100	35.955040	253.932800	.124005
-69.555380	35.982390	254.012400	.044160
-68.325390	35.997170	254.036900	.011911
-67.094890	35.999320	254.043000	.002601
-65.864640	35.988860	254.044200	.000637
-64.635410	35.965800	254.044600	.000236
-63.407950	35.930180	254.044800	.000134
-62.183030	35.882080	254.044900	.000107
-60.961370	35.821590	254.044900	.000107
-59.743720	35.748840	254.044900	.000107
-58.530780	35.663950	254.044900	.000107
-57.323260	35.567100	254.044900	.000107
-56.121820	35.458470	254.044900	.000107
-54.927120	35.338260	254.044900	.000107
-53.739770	35.206700	254.044900	.000107
-52.560380	35.064020	254.044900	.000107
-51.389480	34.910500	254.044900	.000107
-50.227610	34.746390	254.044900	.000107
-49.075240	34.571990	254.044900	.000107
-47.932830	34.387580	254.044900	.000107
-46.800740	34.193490	254.044900	.000107
-45.679330	33.990000	254.044900	.000107
-44.568900	33.777440	254.044900	.000107
-89.960660	34.843320	254.037900	.009646
-88.832330	35.057210	254.033200	.013100
-87.692770	35.261540	254.021000	.022869
-86.542320	35.455950	254.008800	.035543
-85.381340	35.640140	253.996000	.053254
-84.210270	35.813810	253.959100	.082916
-83.029580	35.976620	253.878600	.129253
-81.839790	36.128310	253.783000	.182469
-80.641480	36.268600	253.712000	.241287
-79.435230	36.397220	253.579200	.363571
-78.221690	36.513940	253.080200	.697324
-77.001530	36.618520	251.829200	1.408828
-75.775430	36.710780	250.240400	2.138559
-74.544130	36.790520	249.993400	2.141022
-73.308360	36.857590	251.211200	1.590591
-72.068890	36.911860	252.527000	.949416
-70.826490	36.953220	253.418600	.478987
-69.581950	36.981590	253.845800	.192338
-68.336070	36.996900	253.994100	.059077
-67.089650	36.999130	254.033600	.014038
-65.843500	36.988290	254.041900	.003045
-64.598400	36.964370	254.043700	.000908
-63.355190	36.927440	254.044400	.000281
-62.114630	36.877570	254.044900	.000109
-60.877510	36.814860	254.044900	.000093
-59.644590	36.739430	254.044900	.000131
-58.416640	36.651430	254.044900	.000093
-57.194370	36.551040	254.044900	.000107
-55.978490	36.438440	254.044900	.000107
-54.769680	36.313860	254.044900	.000107
-53.568590	36.177530	254.044900	.000107
-52.375820	36.029710	254.044900	.000107
-51.191970	35.870660	254.044900	.000107
-50.017550	35.700660	254.044900	.000107
-48.853080	35.520030	254.044900	.000107
-47.698980	35.329070	254.044900	.000107
-46.555660	35.128100	254.044900	.000107
-45.423470	34.917440	254.044900	.000107
-44.302690	34.697410	254.044900	.000107
-90.232250	35.766320	254.002900	.041936
-89.092860	35.987640	253.974500	.057136
-87.941790	36.199090	253.905700	.096010
-86.779330	36.400320	253.829000	.149048
-85.605870	36.591000	253.751100	.219219

-84.421870	36.770790	253.575900	.334481
-83.227800	36.939400	253.224400	.499167
-82.024180	37.096500	252.820200	.680371
-80.811620	37.241800	252.539600	.840577
-79.590740	37.375040	252.101400	1.150960
-78.362190	37.495960	250.244100	2.229900
-77.126670	37.604320	245.054800	4.632896
-75.884930	37.699920	238.108500	7.093915
-74.637700	37.782550	237.779600	6.817667
-73.385770	37.852060	243.193300	4.781289
-72.129940	37.908310	248.230400	2.792000
-70.871030	37.951180	251.473800	1.404782
-69.609860	37.980580	253.155600	.606787
-68.347290	37.996460	253.805200	.205765
-67.084140	37.998780	253.992500	.053140
-65.821270	37.987530	254.032800	.011451
-64.559530	37.962740	254.040700	.002696
-63.299760	37.924460	254.043900	.000732
-62.042780	37.872770	254.045200	.000555
-60.789440	37.807780	254.045200	.000460
-59.540510	37.729610	254.044900	.000242
-58.296810	37.638420	254.044900	.000146
-57.059080	37.534390	254.044900	.000130
-55.828080	37.417740	254.044900	.000107
-54.604500	37.288680	254.044900	.000107
-53.389020	37.147460	254.044900	.000116
-52.182290	36.994370	254.044900	.000107
-50.984880	36.829660	254.044900	.000107
-49.797380	36.653650	254.044900	.000107
-48.620270	36.466650	254.044900	.000107
-47.454020	36.268990	254.044900	.000107
-46.299010	36.060990	254.044900	.000107
-45.155610	35.843010	254.044900	.000107
-44.024070	35.615370	254.044900	.000107
-90.516530	36.687310	253.854900	.137691
-89.365660	36.916220	253.725100	.189346
-88.202590	37.134950	253.427500	.314141
-87.027650	37.343150	253.086300	.478529
-85.841200	37.540460	252.756900	.685206
-84.643710	37.726540	252.071200	1.028835
-83.435650	37.901050	250.733600	1.590057
-82.217600	38.063690	249.286600	2.131510
-80.990140	38.214140	248.508400	2.461435
-79.753940	38.352110	247.653300	3.001661
-78.509670	38.477340	241.734800	5.768621
-77.258070	38.589580	224.519800	11.772870
-75.999910	38.688600	197.429700	17.548380
-74.735980	38.774210	200.250400	17.071680
-73.467090	38.846230	219.987900	12.341270
-72.194090	38.904510	236.744300	6.832382
-70.917830	38.948930	246.218800	3.343091
-69.639210	38.979390	251.170900	1.425258
-68.359090	38.995840	253.239500	.503066
-67.078360	38.998240	253.873800	.133404
-65.797930	38.986590	254.008900	.028376
-64.518690	38.960900	254.033500	.006590
-63.241520	38.921240	254.045700	.003375
-61.967320	38.867690	254.049200	.003791
-60.696920	38.800350	254.047800	.002665
-59.431210	38.719360	254.045800	.001309
-58.170970	38.624900	254.044900	.000500
-56.917050	38.517150	254.044900	.000206
-55.670200	38.396340	254.044900	.000124
-54.431160	38.262690	254.044900	.000092
-53.200640	38.116470	254.044900	.000090
-51.979310	37.957960	254.044900	.000098
-50.767770	37.787460	254.044900	.000093

-49.566610	37.605300	254.044900	.000083
-48.376340	37.411790	254.044900	.000107
-47.197410	37.207270	254.044900	.000107
-46.030250	36.992090	254.044900	.000107
-44.875180	36.766620	254.044900	.000107
-43.732470	36.531200	254.044900	.000107
-90.814130	37.606180	253.401100	.352195
-89.651300	37.842850	252.948200	.497706
-88.475770	38.069030	251.965200	.827164
-87.287830	38.284350	250.864200	1.267484
-86.087840	38.488450	249.995100	1.737575
-84.876290	38.680970	247.844000	2.533690
-83.653650	38.861550	243.192600	3.752070
-82.420500	39.029860	238.262600	4.980327
-81.177470	39.185590	236.644400	5.690114
-79.925230	39.328410	236.189500	6.499882
-78.664510	39.458070	225.904000	10.576600
-77.396060	39.574290	184.575800	18.005720
-76.120680	39.676830	135.755300	22.440920
-74.839220	39.765490	121.238300	21.795810
-73.552520	39.840080	160.520800	19.878260
-72.261480	39.900440	210.983500	14.448930
-70.967010	39.946460	235.734900	6.188689
-69.670030	39.978020	247.359100	2.540763
-68.371470	39.995060	252.225600	.807474
-67.072270	39.997550	253.724800	.185706
-65.773400	39.985470	253.985600	.044882
-64.475770	39.958870	254.026600	.015333
-63.180340	39.917780	254.067900	.019557
-61.888020	39.862300	254.072800	.018828
-60.599740	39.792560	254.061400	.011954
-59.316390	39.708680	254.051000	.005917
-58.038840	39.610860	254.046300	.002444
-56.767930	39.499290	254.045300	.001003
-55.504470	39.374200	254.045000	.000431
-54.249260	39.235840	254.044900	.000195
-53.003000	39.084480	254.044900	.000099
-51.766410	38.920440	254.044900	.000107
-50.540120	38.744010	254.044900	.000109
-49.324710	38.555530	254.044900	.000096
-48.120720	38.355340	254.044900	.000087
-46.928620	38.143810	254.044900	.000107
-45.748810	37.921300	254.044900	.000107
-44.581620	37.688170	254.044900	.000107
-43.427300	37.444810	254.044900	.000107
-91.125630	38.522790	252.399400	.728657
-89.950410	38.767390	251.195500	1.071346
-88.761920	39.001200	248.577500	1.562397
-87.560450	39.223820	245.402100	1.893595
-86.346370	39.434880	243.953900	3.436209
-85.120160	39.633990	239.897900	5.794161
-83.882300	39.820790	228.938700	7.503198
-82.633390	39.994930	212.160700	8.355989
-81.374080	40.156070	207.828900	9.853305
-80.105060	40.303890	214.500400	9.396016
-78.827100	40.438090	212.236600	8.656298
-77.540980	40.558400	173.338100	13.129400
-76.247540	40.664560	86.159610	16.249090
-74.947690	40.756360	66.216750	14.130170
-73.642300	40.833600	85.679640	15.285520
-72.332310	40.896110	159.367900	18.682510
-71.018690	40.943760	219.941600	10.269260
-69.702430	40.976440	243.096300	3.366871
-68.384490	40.994090	251.701400	.978981
-67.065880	40.996670	253.995500	.268989
-65.747600	40.984170	253.987500	.098049
-64.430650	40.956610	254.030100	.061544

-63.116020 40.914060 254.199600 .088209
-61.804690 40.856610 254.180900 .073466
-60.497620 40.784390 254.118900 .043887
-59.195770 40.697540 254.074200 .021306
-57.900040 40.596260 254.054600 .009518
-56.611330 40.480750 254.049000 .004416
-55.330490 40.351270 254.046800 .002095
-54.058330 40.208070 254.045600 .000850
-52.795620 40.051450 254.045000 .000294
-51.543080 39.881710 254.044900 .000103
-50.301390 39.699200 254.044900 .000103
-49.071130 39.504250 254.044900 .000119
-47.852850 39.297230 254.044900 .000101
-46.647030 39.078520 254.044900 .000087
-45.454070 38.848490 254.044900 .000107
-44.274280 38.607530 254.044900 .000107
-43.107920 38.356030 254.044900 .000107
-91.451740 39.437030 250.849200 1.245544
-90.263630 39.689750 248.249900 1.623748
-89.061680 39.931370 242.022800 1.806747
-87.846150 40.161480 234.402200 4.161626
-86.617400 40.379670 226.290800 5.194335
-85.375900 40.585540 215.973700 5.832303
-84.122160 40.778730 192.970200 9.968979
-82.856800 40.958850 175.697200 11.506370
-81.580460 41.125550 156.137300 13.343300
-80.293880 41.278500 174.005300 13.971820
-78.997860 41.417370 204.796300 8.370169
-77.693230 41.541890 182.840600 14.737380
-76.380870 41.651780 93.492510 16.300510
-75.061690 41.746810 39.967370 11.787630
-73.736660 41.826770 76.946940 21.355890
-72.406780 41.891490 145.642300 16.183600
-71.073040 41.940830 210.358300 11.193060
-69.736500 41.974670 241.612600 4.078034
-68.398190 41.992950 252.144300 1.363437
-67.059160 41.995620 254.191200 .348820
-65.720480 41.982670 254.231800 .197985
-64.383220 41.954140 254.063300 .357924
-63.048400 41.910080 254.797400 .268943
-61.717090 41.850600 254.582900 .251820
-60.390290 41.775820 254.313300 .134209
-59.069010 41.685920 254.152400 .063266
-57.754210 41.581080 254.085800 .029710
-56.446830 41.461530 254.065200 .015992
-55.147770 41.327530 254.054500 .007539
-53.857880 41.179360 254.048500 .003007
-52.577970 41.017320 254.045800 .000957
-51.308780 40.841740 254.045000 .000256
-50.051000 40.652980 254.044900 .000102
-48.805250 40.451390 254.044900 .000126
-47.572090 40.237360 254.044900 .000112
-46.352010 40.011290 254.044900 .000101
-45.145370 39.773560 254.044900 .000087
-43.952510 39.524580 254.044900 .000107
-42.773630 39.264750 254.044900 .000107
-91.793200 40.348750 249.238300 1.791095
-90.591710 40.609810 244.694600 1.880312
-89.375760 40.859430 232.649500 3.939798
-88.145600 41.097210 210.212500 13.560410
-86.901570 41.322720 182.736000 20.166420
-85.644150 41.535550 153.515100 15.793320
-84.373860 41.735290 127.611900 12.154120
-83.091290 41.921560 98.623530 10.223920
-81.797160 42.093980 111.590600 11.318360
-80.492230 42.252200 155.831500 17.916920
-79.177290 42.395880 198.298400 13.155710

-77.853240 42.524730 173.331800 10.520450
-76.521000 42.638450 89.314640 13.206250
-75.181550 42.736820 66.275500 23.282890
-73.835900 42.819590 113.894300 9.449662
-72.485100 42.886590 189.284200 10.255910
-71.130210 42.937670 234.348600 5.785266
-69.772340 42.972710 250.464100 3.802838
-68.412590 42.991630 252.620700 1.213198
-67.052090 42.994400 253.945700 .812840
-65.691960 42.980990 254.437300 .858444
-64.333320 42.951450 255.732400 .714172
-62.977290 42.905830 256.220000 .822942
-61.624970 42.844260 255.830500 .640345
-60.277440 42.766850 254.840500 .354796
-58.935750 42.673790 254.353100 .166773
-57.600940 42.565280 254.171200 .086209
-56.273990 42.441570 254.119600 .049205
-54.955840 42.302930 254.077800 .022570
-53.647390 42.149640 254.056300 .007895
-52.349470 41.982030 254.047800 .002328
-51.062890 41.800450 254.045300 .000531
-49.788320 41.605260 254.044900 .000147
-48.526440 41.396860 254.044800 .000143
-47.277780 41.175640 254.044800 .000130
-46.042850 40.942000 254.044900 .000112
-44.822010 40.696380 254.044900 .000101
-43.615560 40.439180 254.044900 .000107
-42.423690 40.170830 254.044900 .000107
-92.150770 41.257820 248.294900 2.208979
-90.935390 41.527410 242.076000 3.200700
-89.704910 41.785250 224.276000 3.848578
-88.459540 42.030900 190.127500 11.589950
-87.199620 42.263930 144.863800 19.791730
-85.925610 42.483890 96.279500 16.535490
-84.638040 42.690380 56.076760 16.137480
-83.337520 42.882970 48.876720 13.951560
-82.024790 43.061280 102.314300 16.319570
-80.700610 43.224930 215.711200 20.543270
-79.365870 43.373580 263.858900 18.734090
-78.021450 43.506890 233.072300 17.819910
-76.668370 43.624570 123.024600 13.327820
-75.307630 43.726360 102.910300 15.797250
-73.940300 43.812040 170.975900 12.345330
-72.567500 43.881390 227.417700 8.367297
-71.190370 43.934270 249.505200 7.821807
-69.810050 43.970550 253.201600 3.568594
-68.427750 43.990140 256.199600 1.706182
-67.044650 43.993000 257.688500 1.072451
-65.661930 43.979120 259.776600 1.436168
-64.280810 43.948540 260.917200 1.906705
-62.902460 43.901310 260.507300 1.908731
-61.528050 43.837570 258.597000 1.345228
-60.158730 43.757450 256.080000 .769868
-58.795620 43.661140 254.962300 .368143
-57.439790 43.548850 254.528900 .230096
-56.092310 43.420850 254.297100 .133672
-54.754150 43.277410 254.135900 .052459
-53.426270 43.118850 254.069700 .015831
-52.109530 42.945500 254.049900 .003607
-50.804760 42.757750 254.045100 .000707
-49.512690 42.555960 254.044400 .000296
-48.233980 42.340550 254.044600 .000174
-46.969190 42.111940 254.044800 .000145
-45.718810 41.870550 254.044800 .000130
-44.483210 41.616830 254.044900 .000112
-43.262650 41.351200 254.044900 .000107
-42.057300 41.074100 254.044900 .000107

-92.525320	42.164070	248.647400	2.269238
-91.295520	42.442420	242.481100	3.895574
-90.049940	42.708690	224.494300	6.058249
-88.788760	42.962440	189.752100	7.885470
-87.512310	43.203190	140.020100	12.563950
-86.221000	43.430500	89.778600	14.673710
-84.915410	43.643920	44.496140	7.550836
-83.596150	43.843030	61.846860	18.742520
-82.263960	44.027400	154.955300	25.346980
-80.919660	44.196660	266.899000	31.322980
-79.564150	44.350410	309.617400	27.790470
-78.198390	44.488330	246.321400	13.511530
-76.823410	44.610100	142.363100	8.800352
-75.440290	44.715440	112.923100	12.375730
-74.050180	44.804110	148.604900	12.712180
-72.654240	44.875890	202.956000	13.001300
-71.253690	44.930630	234.811600	7.318137
-69.849760	44.968180	253.729300	4.891008
-68.443720	44.988460	260.748700	2.498570
-67.036810	44.991420	264.948000	2.554085
-65.630330	44.977050	268.307500	3.532810
-64.225530	44.945390	269.801300	3.995325
-62.823690	44.896510	268.011300	3.592615
-61.426040	44.830540	262.673400	2.409654
-60.033810	44.747610	258.482000	1.294990
-58.648170	44.647940	256.000200	.718153
-57.270290	44.531750	255.084900	.450762
-55.901260	44.399320	254.586900	.253598
-54.542130	44.250930	254.204500	.077377
-53.193900	44.086940	254.077100	.015589
-51.857480	43.907680	254.046200	.003613
-50.533710	43.713560	254.042100	.001852
-49.223370	43.504980	254.042800	.001050
-47.927120	43.282370	254.043900	.000537
-46.645540	43.046150	254.044500	.000219
-45.379100	42.796800	254.044800	.000139
-44.128160	42.534760	254.044900	.000141
-42.892960	42.260480	254.044900	.000087
-41.673610	41.974410	254.044900	.000107
-92.917770	43.067330	249.683700	2.052081
-91.673000	43.354680	244.058900	3.812972
-90.411740	43.629630	226.720000	7.384299
-89.134110	43.891690	189.668000	12.629000
-87.840450	44.140390	134.249100	17.757760
-86.531140	44.375260	75.415050	20.861190
-85.206750	44.595830	63.361050	11.687630
-83.867900	44.801650	111.889800	21.898710
-82.515380	44.992280	170.403900	35.108740
-81.150000	45.167310	236.689500	34.837840
-79.772730	45.326340	230.487200	23.230710
-78.384560	45.469020	210.862400	14.272520
-76.986600	45.595010	178.375700	7.339489
-75.579970	45.704020	155.151800	8.931753
-74.165890	45.795780	191.717600	13.487810
-72.745610	45.870080	217.425100	11.201170
-71.320400	45.926740	238.582000	6.196560
-69.891600	45.965610	253.093500	2.534320
-68.460530	45.986610	262.251400	2.233406
-67.028560	45.989670	268.217300	3.772717
-65.597030	45.974800	274.832100	5.166987
-64.167300	45.942020	276.585800	5.731480
-62.740720	45.891430	272.765900	4.884158
-61.318600	45.823140	266.509400	3.325510
-59.902260	45.737310	259.736200	1.925994
-58.492950	45.634170	256.498500	.961096
-57.091900	45.513950	255.154100	.437023
-55.700260	45.376930	254.294500	.212116

-54.319140 45.223450 254.090500 .072252
-52.949590 45.053840 254.003000 .053960
-51.592580 44.868490 254.025700 .015387
-50.248970 44.667810 254.033100 .006219
-48.919560 44.452230 254.039000 .002956
-47.605040 44.222190 254.042400 .001253
-46.305980 43.978160 254.044000 .000494
-45.022840 43.720610 254.044600 .000176
-43.755960 43.450020 254.044800 .000153
-42.505550 43.166850 254.044900 .000090
-41.271660 42.871580 254.044900 .000107
-93.329110 43.967430 250.897900 1.629288
-92.068780 44.264030 246.441800 3.154878
-90.791210 44.547890 232.283600 6.788765
-89.496510 44.818510 198.988000 11.342100
-88.184940 45.075390 134.715900 17.578210
-86.856880 45.318050 61.084010 23.214040
-85.512890 45.545990 57.993440 13.756790
-84.153590 45.758730 126.243300 27.412030
-82.779790 45.955820 159.607300 28.322270
-81.392350 46.136820 144.479700 18.239080
-79.992260 46.301310 141.298500 17.888510
-78.580580 46.448910 164.873300 13.032570
-77.158460 46.579270 187.271500 8.883141
-75.727110 46.692070 219.393200 11.750290
-74.287820 46.787040 229.105700 9.462362
-72.841890 46.863950 244.446000 6.233615
-71.390720 46.922590 253.919000 2.803084
-69.935700 46.962830 257.262100 2.009673
-68.478260 46.984570 263.298100 3.036317
-67.019850 46.987740 272.603000 4.779872
-65.561920 46.972340 277.633300 6.158804
-64.105920 46.938410 280.834800 6.633562
-62.653260 46.886040 276.181900 5.721888
-61.205390 46.815350 267.939900 4.152944
-59.763680 46.726520 261.134400 2.677878
-58.329470 46.619790 257.262100 1.394164
-56.904060 46.495390 255.184600 .723629
-55.488670 46.353640 254.345100 .384581
-54.084500 46.194880 253.997800 .274048
-52.692630 46.019470 254.019100 .072912
-51.314070 45.827840 254.001300 .025866
-49.949730 45.620390 254.017200 .014204
-48.600430 45.397590 254.032500 .006163
-47.266860 45.159900 254.040000 .002397
-45.949600 44.907820 254.043200 .000849
-44.649100 44.641840 254.044300 .000286
-43.365670 44.362450 254.044700 .000124
-42.099450 44.070140 254.044900 .000114
-40.850440 43.765410 254.044900 .000107
-93.760410 44.864160 252.000700 1.150723
-92.483920 45.170280 248.820900 2.376350
-91.189420 45.463320 237.922400 5.825785
-89.876960 45.742750 208.165500 11.312970
-88.546760 46.008070 150.943600 20.594390
-87.199170 46.258760 88.082510 27.294370
-85.834720 46.494300 80.372260 23.059760
-84.454070 46.714200 133.302000 13.380890
-83.058010 46.917960 143.731200 10.090090
-81.647480 47.105130 131.910600 18.946290
-80.223460 47.275260 132.022600 27.257630
-78.787090 47.427960 163.996600 22.101710
-77.339580 47.562840 204.997000 16.140960
-75.882230 47.679570 224.164000 9.558046
-74.416370 47.777870 245.693500 6.302168
-72.943440 47.857470 259.331600 4.115986
-71.464880 47.918190 261.428300 2.378438

-69.982220	47.959850	263.220700	2.523406
-68.496960	47.982350	267.615300	3.598942
-67.010670	47.985630	273.371400	5.110546
-65.524890	47.969690	279.031900	6.140629
-64.041170	47.934570	280.346200	6.518275
-62.561040	47.880340	277.618100	5.859960
-61.086020	47.807170	271.666300	4.629951
-59.617590	47.715230	265.967800	3.223992
-58.157170	47.604770	260.365900	2.108893
-56.706150	47.476050	256.423700	1.233459
-55.265830	47.329400	254.819600	.652876
-53.837470	47.165180	254.418800	.269847
-52.422200	46.983780	254.160700	.112324
-51.021090	46.785640	254.000100	.048292
-49.635090	46.571200	253.981600	.029777
-48.265020	46.340950	254.022300	.011156
-46.911610	46.095370	254.037700	.003487
-45.575410	45.834990	254.042600	.001093
-44.256860	45.560320	254.044100	.000341
-42.956210	45.271870	254.044700	.000129
-41.673580	44.970160	254.044900	.000145
-40.408870	44.655690	254.044900	.000087
-94.212860	45.757310	252.957900	.673775
-92.919560	46.073230	251.213700	1.377020
-91.607470	46.375730	245.524800	3.304614
-90.276550	46.664250	230.871700	7.138564
-88.926960	46.938270	199.459000	13.684980
-87.559010	47.197250	160.464800	22.940760
-86.173240	47.440650	146.089800	25.000880
-84.770290	47.667940	162.616500	18.519500
-83.350960	47.878600	158.038700	23.901210
-81.916200	48.072160	158.357600	26.714000
-80.467090	48.248140	173.302200	25.400410
-79.004780	48.406110	198.514400	21.086890
-77.530590	48.545680	217.734500	13.581020
-76.045850	48.666500	236.738600	8.012673
-74.552020	48.768250	248.398600	3.745099
-73.050600	48.850660	256.374400	1.279338
-71.543170	48.913520	260.755100	1.604176
-70.031330	48.956650	262.326300	2.065075
-68.516710	48.979950	265.739300	2.936982
-67.000980	48.983350	270.143500	4.081363
-65.485790	48.966840	273.651900	4.903524
-63.972820	48.930470	276.302200	5.362844
-62.463700	48.874330	275.114100	5.252720
-60.960050	48.798580	273.205100	4.796515
-59.463460	48.703410	269.914400	4.097681
-57.975440	48.589080	264.982800	2.895361
-56.497490	48.455880	258.726200	1.525525
-55.030970	48.304140	255.610500	.771032
-53.577210	48.134270	254.761400	.319974
-52.137430	47.946660	254.358400	.140527
-50.712720	47.741790	254.115100	.049650
-49.304080	47.520120	254.037200	.016424
-47.912350	47.282170	254.020900	.014110
-46.538250	47.028450	254.039500	.002644
-45.182340	46.759510	254.043400	.000653
-43.845020	46.475880	254.044300	.000215
-42.526500	46.178100	254.044700	.000124
-41.226800	45.866700	254.044900	.000144
-39.945740	45.542200	254.044900	.000110
-94.687740	46.646660	253.655500	.289983
-93.376940	46.972690	252.962800	.575640
-92.046550	47.284930	250.685900	1.490815
-90.696440	47.582840	243.212900	3.698613
-89.326680	47.865840	228.066900	7.484109
-87.937550	48.133390	211.141400	13.192920

-86.529520	48.384910	197.985600	16.954070
-85.103260	48.619850	192.315700	18.733920
-83.659580	48.837660	190.751200	19.822230
-82.199460	49.037830	202.316500	18.412050
-80.724000	49.219880	219.214400	14.839600
-79.234450	49.383340	228.737800	10.698800
-77.732160	49.527770	238.485300	7.194764
-76.218590	49.652830	245.223600	4.366550
-74.695260	49.758160	250.193800	2.347655
-73.163800	49.843490	253.809600	1.130315
-71.625870	49.908580	256.913400	.992118
-70.083210	49.953250	259.310000	1.268008
-68.537570	49.977380	261.462400	1.998270
-66.990740	49.980900	265.368400	2.684807
-65.444490	49.963800	266.838000	3.176989
-63.900620	49.926140	268.021400	3.506763
-62.360880	49.868010	267.851000	3.523450
-60.827010	49.789570	266.957300	3.416036
-59.300730	49.691040	266.362400	3.173971
-57.783640	49.572690	262.940000	2.402013
-56.277320	49.434830	258.647800	1.329269
-54.783270	49.277820	255.837000	.647265
-53.302860	49.102080	254.863000	.305885
-51.837360	48.908040	254.420400	.163159
-50.387950	48.696200	254.238300	.077360
-48.955640	48.467040	254.125200	.033657
-47.541310	48.221130	254.083000	.015931
-46.145660	47.959000	254.052200	.004219
-44.769230	47.681210	254.046200	.000824
-43.412380	47.388330	254.045000	.000171
-42.075270	47.080920	254.044900	.000093
-40.757810	46.759540	254.044900	.000093
-39.459720	46.424710	254.044900	.000107
-95.186440	47.531940	254.161400	.112824
-93.857410	47.868410	254.247800	.274800
-92.508000	48.190720	253.477900	.513958
-91.137910	48.498310	250.125100	1.507905
-89.747150	48.790600	243.346400	3.817273
-88.335950	49.067010	234.900200	6.310901
-86.904710	49.326930	228.431200	8.689836
-85.454100	49.569790	223.850700	9.850804
-83.984930	49.795020	226.285300	9.868979
-82.498220	50.002060	234.274100	8.148767
-80.995120	50.190400	242.258000	5.689690
-79.476920	50.359550	246.508200	4.131877
-77.945060	50.509060	249.303500	2.747717
-76.401090	50.638520	251.354900	1.811823
-74.846650	50.747580	252.749100	1.126486
-73.283450	50.835950	254.072100	.694408
-71.713320	50.903350	256.067500	.762372
-70.138080	50.949630	258.203300	1.056515
-68.559640	50.974620	260.310200	1.427504
-66.979900	50.978270	261.569500	2.010941
-65.400800	50.960560	261.367600	2.021793
-63.824260	50.921550	261.241800	1.925366
-62.252170	50.861340	260.464700	1.971977
-60.686400	50.780110	259.735400	1.884594
-59.128770	50.678080	259.304400	1.737131
-57.581030	50.555550	258.580800	1.278998
-56.044860	50.412850	256.305700	.840491
-54.521820	50.250360	255.065800	.395149
-53.013420	50.068520	254.498600	.202819
-51.520960	49.867810	254.294300	.100361
-50.045680	49.648740	254.167200	.056892
-48.588630	49.411830	254.113100	.028334
-47.150700	49.157670	254.067300	.012248
-45.732590	48.886830	254.051300	.003515

-44.334810	48.599900	254.046500	.000974
-42.957660	48.297460	254.045100	.000242
-41.601190	47.980120	254.044900	.000085
-40.265250	47.648430	254.044900	.000107
-38.949390	47.302930	254.044900	.000107
-95.710510	48.412890	254.599600	.377394
-94.362470	48.760130	255.092900	.676835
-92.993230	49.092850	254.743300	.597100
-91.602360	49.410460	253.243200	.501735
-90.189750	49.712350	250.462000	1.398236
-88.755540	49.997940	248.129900	2.529994
-87.300090	50.266570	245.855900	3.249855
-85.824040	50.517650	244.033200	3.844941
-84.328210	50.750570	245.536900	3.743351
-82.813610	50.964740	247.970000	2.793519
-81.281460	51.159630	251.231000	1.933652
-79.733130	51.334700	253.307300	1.488710
-78.170130	51.489480	254.303000	1.022519
-76.594090	51.623530	254.344500	.657485
-75.006800	51.736490	254.223800	.549206
-73.410070	51.828010	254.738400	.457320
-71.805870	51.897850	256.189900	.651123
-70.196170	51.945790	258.038100	1.018805
-68.582990	51.971680	260.090400	1.400197
-66.968440	51.975460	261.087200	1.719969
-65.354560	51.957120	258.579400	1.526266
-63.743440	51.916690	256.927100	1.192289
-62.137130	51.854320	256.430100	.900959
-60.537630	51.770170	256.057800	.826754
-58.946900	51.664510	255.857900	.728308
-57.366820	51.537620	255.477400	.609489
-55.799180	51.389870	254.871400	.359132
-54.245650	51.221680	254.450600	.188404
-52.707820	51.033510	254.251800	.090262
-51.187080	50.825860	254.132100	.050959
-49.684720	50.599280	254.090100	.024344
-48.201800	50.354330	254.068400	.013076
-46.739240	50.091630	254.054600	.005356
-45.297720	49.811770	254.048200	.001951
-43.877700	49.515370	254.045700	.000609
-42.479410	49.203060	254.045000	.000171
-41.102810	48.875440	254.044900	.000084
-39.747590	48.533100	254.044900	.000107
-38.413150	48.176570	254.044900	.000107
-96.261670	49.289190	254.979600	.588761
-94.893750	49.647600	255.197100	.735046
-93.503820	49.991080	255.088700	.664033
-92.091320	50.319060	254.066300	.246902
-90.655950	50.630910	253.187800	.478441
-89.197760	50.925990	252.436900	.771444
-87.717060	51.203660	251.795300	1.034635
-86.214420	51.463270	251.654800	1.260062
-84.690660	51.704190	252.172000	1.153269
-83.146820	51.925780	252.930900	.839141
-81.584160	52.127480	254.378000	.703449
-80.004110	52.308710	255.954800	1.054049
-78.408280	52.468990	256.032900	.910041
-76.798410	52.607840	255.159300	.480273
-75.176390	52.724850	254.638100	.372194
-73.544200	52.819690	254.933200	.453980
-71.903920	52.892050	256.048000	.607080
-70.257710	52.941730	257.811800	.927205
-68.607750	52.968570	260.127100	1.428738
-66.956280	52.972490	261.178400	1.789134
-65.305560	52.953480	258.040100	1.231993
-63.657820	52.911580	255.605000	.734733
-62.015270	52.846950	254.826200	.432002

-60.380080	52.759750	254.672800	.303792
-58.754360	52.650280	254.578700	.261862
-57.140120	52.518850	254.471600	.202010
-55.539290	52.365840	254.304800	.130008
-53.953660	52.191710	254.176600	.071966
-52.384890	51.996940	254.108000	.037576
-50.834480	51.782070	254.074100	.017426
-49.303730	51.547700	254.059400	.008812
-47.793770	51.294400	254.052500	.004533
-46.305490	51.022820	254.048200	.002112
-44.839570	50.733620	254.046000	.000826
-43.396390	50.427420	254.045100	.000288
-41.976090	50.104880	254.044900	.000087
-40.578500	49.766630	254.044900	.000090
-39.203150	49.413260	254.044900	.000080
-37.849210	49.045330	254.044900	.000107
-96.841810	50.160530	254.680200	.416927
-95.453030	50.530490	254.909700	.535694
-94.041510	50.885130	254.577400	.356994
-92.606450	51.223850	254.170500	.147688
-91.147380	51.546020	253.886300	.134642
-89.664200	51.850960	253.663900	.217781
-88.157130	52.138020	253.508200	.292743
-86.626690	52.406500	253.516700	.306479
-85.073680	52.655720	253.809200	.276083
-83.499180	52.885050	254.241600	.253783
-81.904440	53.093850	255.040900	.415804
-80.290990	53.281520	255.964500	.865712
-78.660530	53.447530	256.131100	.993862
-77.014920	53.591380	255.380600	.618876
-75.356160	53.712650	254.688800	.373121
-73.686420	53.810940	254.643800	.432838
-72.007930	53.885960	255.685200	.545165
-70.322990	53.937460	257.046000	.730333
-68.634010	53.965280	259.290600	1.243529
-66.943400	53.969350	260.265300	1.602205
-65.253590	53.949630	257.674100	.999100
-63.567000	53.906200	255.218700	.529152
-61.886040	53.839190	254.414000	.229891
-60.213040	53.748820	254.231800	.120056
-58.550290	53.635360	254.182400	.085249
-56.899960	53.499180	254.150400	.064046
-55.264110	53.340690	254.112100	.041394
-53.644650	53.160350	254.078700	.023448
-52.043340	52.958710	254.060800	.011724
-50.461760	52.736320	254.052300	.005525
-48.901280	52.493820	254.048800	.002719
-47.363030	52.231850	254.046800	.001436
-45.847900	51.951060	254.045700	.000678
-44.356530	51.652150	254.045100	.000282
-42.889220	51.335790	254.044900	.000107
-41.445980	51.002650	254.044900	.000087
-40.026490	50.653380	254.044900	.000083
-38.630060	50.288590	254.044900	.000107
-37.255620	49.908830	254.044900	.000107
-97.453060	51.026530	254.314100	.182802
-96.042340	51.408490	254.370800	.213522
-94.608220	51.774700	254.215600	.123669
-93.149600	52.124570	254.099100	.054886
-91.665800	52.457450	254.026600	.036517
-90.156560	52.772640	253.974800	.054259
-88.621980	53.069450	253.947500	.071345
-87.062480	53.347160	253.972700	.069842
-85.478850	53.605050	254.084700	.058107
-83.872130	53.842430	254.341200	.124923
-82.243660	54.058630	254.670000	.302275
-80.595020	54.253030	255.359600	.694445

-78.928000	54.425040	255.849900	.989716
-77.244600	54.574130	255.470800	.767788
-75.546970	54.699840	254.658000	.369352
-73.837420	54.801750	254.364000	.360470
-72.118360	54.879540	254.862000	.422138
-70.392330	54.932960	255.658800	.438219
-68.661900	54.961820	256.957500	.653670
-66.929700	54.966030	257.956200	.970040
-65.198370	54.945580	256.298200	.625101
-63.470540	54.900540	254.816900	.333157
-61.748820	54.831050	254.289700	.122588
-60.035740	54.737340	254.121400	.052345
-58.333780	54.619720	254.076500	.027881
-56.645260	54.478570	254.066300	.017407
-54.972420	54.314330	254.057700	.011148
-53.317280	54.127510	254.051700	.006076
-51.681740	53.918690	254.048000	.002984
-50.067420	53.688470	254.046300	.001360
-48.475770	53.437500	254.045500	.000700
-46.907930	53.166490	254.045200	.000370
-45.364770	52.876130	254.045000	.000179
-43.846840	52.567130	254.044900	.000086
-42.354370	52.240220	254.044900	.000087
-40.887210	51.896080	254.044900	.000083
-39.444820	51.535370	254.044900	.000083
-38.026250	51.158730	254.044900	.000107
-36.630120	50.766700	254.044900	.000107
-98.097820	51.886770	254.121600	.055867
-96.663910	52.281200	254.120900	.056013
-95.206040	52.659450	254.090000	.035376
-93.722790	53.020900	254.064200	.018848
-92.213200	53.364910	254.047500	.010639
-90.676770	53.690770	254.037100	.011010
-89.113440	53.997730	254.035100	.013084
-87.523560	54.285050	254.049600	.011879
-85.907830	54.551970	254.094700	.026781
-84.267300	54.797760	254.193400	.079812
-82.603340	55.021710	254.389800	.202365
-80.917590	55.223140	254.879800	.495252
-79.211960	55.401440	255.601800	.913506
-77.488560	55.556020	255.579700	.897652
-75.749730	55.686380	254.819400	.469983
-73.997920	55.792100	254.263200	.302387
-72.235800	55.872810	254.255400	.385844
-70.466090	55.928230	254.373600	.255006
-68.691570	55.958180	254.765900	.344927
-66.915130	55.962550	255.513500	.371456
-65.139640	55.941330	254.925200	.334936
-63.367970	55.894590	254.467200	.154533
-61.602940	55.822490	254.189300	.069842
-59.847310	55.725280	254.086300	.025810
-58.103760	55.603290	254.056000	.009582
-56.374820	55.456930	254.048400	.004455
-54.662870	55.286670	254.046900	.002298
-52.970100	55.093080	254.045600	.001248
-51.298500	54.876750	254.045300	.000553
-49.649800	54.638340	254.045000	.000266
-48.025460	54.378550	254.044900	.000142
-46.426660	54.098120	254.044900	.000089
-44.854210	53.797780	254.044900	.000107
-43.308580	53.478290	254.044900	.000090
-41.789850	53.140400	254.044900	.000083
-40.297680	52.784830	254.044900	.000083
-38.831280	52.412250	254.044900	.000080
-37.389370	52.023280	254.044900	.000107
-35.970190	51.618480	254.044900	.000107
-98.778840	52.740810	254.064900	.015388

-97.320270	53.148240	254.062200	.013650
-95.837370	53.539010	254.056000	.009596
-94.328290	53.912530	254.050500	.005824
-92.791720	54.268120	254.046700	.003207
-91.226900	54.605080	254.044900	.001947
-89.633560	54.922620	254.045600	.001645
-88.011890	55.219970	254.051600	.004298
-86.362530	55.496330	254.070000	.015607
-84.686490	55.750910	254.118000	.046244
-82.985150	55.982970	254.239900	.128022
-81.260250	56.191770	254.560900	.339548
-79.513790	56.376650	255.316600	.770498
-77.748020	56.536990	255.687500	.984935
-75.965460	56.672260	255.126800	.639618
-74.168790	56.781970	254.359300	.295140
-72.360860	56.865740	253.942000	.396928
-70.544630	56.923280	253.598100	.358445
-68.723180	56.954370	253.823500	.281022
-66.899620	56.958910	254.233400	.190326
-65.077100	56.936880	254.368700	.111121
-63.258730	56.888360	254.272600	.081170
-61.447630	56.813510	254.150900	.046205
-59.646790	56.712620	254.079000	.019156
-57.859090	56.586040	254.055400	.006506
-56.087300	56.434210	254.047800	.002188
-54.333970	56.257640	254.045400	.000784
-52.601470	56.056940	254.045000	.000240
-50.891880	55.832740	254.044900	.000119
-49.207030	55.585770	254.044900	.000120
-47.548420	55.316760	254.044900	.000122
-45.917200	55.026500	254.044900	.000101
-44.314130	54.715760	254.044900	.000087
-42.739580	54.385350	254.044900	.000083
-41.193420	54.036030	254.044900	.000083
-39.675070	53.668560	254.044900	.000080
-38.183400	53.283620	254.044900	.000107
-36.716770	52.881820	254.044900	.000107
-35.272900	52.463700	254.044900	.000107
-99.499240	53.588130	254.049600	.004090
-98.014280	54.009130	254.048500	.003305
-96.504860	54.412960	254.047200	.002411
-94.968610	54.799050	254.046000	.001511
-93.403790	55.166730	254.045300	.000831
-91.809290	55.515250	254.045000	.000449
-90.184560	55.843850	254.045300	.000630
-88.529620	56.151670	254.047400	.002385
-86.844990	56.437890	254.055200	.008407
-85.131650	56.701690	254.081300	.027236
-83.390960	56.942230	254.160800	.082343
-81.624720	57.158780	254.383200	.234738
-79.835050	57.350580	255.009600	.597306
-78.024370	57.516980	255.653700	.970550
-76.195360	57.657400	255.399600	.796836
-74.350940	57.771310	254.546000	.346478
-72.494220	57.858320	253.721400	.379673
-70.628410	57.918090	253.263900	.476247
-68.756890	57.950390	253.456500	.350239
-66.883060	57.955100	253.965600	.118856
-65.010370	57.932220	254.155000	.053288
-63.142240	57.881810	254.182900	.059180
-61.282040	57.804080	254.163400	.054247
-59.433060	57.699310	254.090800	.025812
-57.598450	57.567890	254.061400	.009731
-55.781200	57.410300	254.050300	.003423
-53.984060	57.227100	254.046300	.001095
-52.209560	57.018940	254.045100	.000293
-50.459930	56.786500	254.044900	.000116

-48.737060	56.530560	254.044800	.000116
-47.042490	56.251900	254.044800	.000122
-45.377330	55.951360	254.044900	.000107
-43.742270	55.629770	254.044900	.000083
-42.137470	55.287960	254.044900	.000083
-40.562610	54.926740	254.044900	.000080
-39.016750	54.546860	254.044900	.000107
-37.498410	54.149020	254.044900	.000107
-36.005400	53.733830	254.044900	.000107
-34.534880	53.301770	254.044900	.000107
-100.262700	54.428120	254.045800	.000971
-98.749250	54.863350	254.045500	.000733
-97.211560	55.280850	254.045200	.000503
-95.646590	55.680060	254.045000	.000317
-94.052090	56.060360	254.044900	.000165
-92.426510	56.420970	254.044900	.000133
-90.768940	56.761100	254.044900	.000228
-89.079190	57.079890	254.045500	.001036
-87.357570	57.376450	254.049000	.004334
-85.605000	57.649890	254.063700	.015952
-83.822840	57.899360	254.114600	.052256
-82.012920	58.124030	254.265300	.155525
-80.177480	58.323120	254.690200	.414262
-78.319110	58.495910	255.421100	.834288
-76.440710	58.641750	255.538400	.888660
-74.545430	58.760110	254.700600	.441977
-72.636660	58.850530	253.669900	.371775
-70.717930	58.912650	253.050700	.560302
-68.792920	58.946230	253.404400	.409390
-66.865380	58.951130	253.873400	.144415
-64.939080	58.927340	254.053400	.037048
-63.017780	58.874950	254.146300	.050429
-61.105210	58.794160	254.151100	.051458
-59.204930	58.685300	254.126700	.039795
-57.320400	58.548780	254.071300	.015710
-55.454840	58.385130	254.053700	.005274
-53.611270	58.194950	254.047400	.001643
-51.792370	57.978940	254.045400	.000458
-50.000500	57.737840	254.044900	.000111
-48.237630	57.472490	254.044900	.000107
-46.505310	57.183730	254.044900	.000103
-44.804610	56.872430	254.044900	.000104
-43.136050	56.539500	254.044900	.000080
-41.499610	56.185780	254.044900	.000080
-39.894630	55.812120	254.044900	.000107
-38.319800	55.419290	254.044900	.000107
-36.773090	55.007980	254.044900	.000107
-35.251730	54.578750	254.044900	.000107
-33.752120	54.132060	254.044900	.000107
-101.073700	55.260130	254.045000	.000206
-99.529010	55.710320	254.044900	.000145
-97.960900	56.142140	254.044900	.000119
-96.365430	56.555100	254.044900	.000116
-94.739640	56.948590	254.044900	.000086
-93.081440	57.321860	254.044900	.000117
-91.389490	57.674070	254.044900	.000119
-89.663250	58.004340	254.045000	.000432
-87.902820	58.311730	254.046500	.002162
-86.109000	58.595320	254.054300	.008895
-84.283100	58.854170	254.084200	.030890
-82.427000	59.087400	254.175000	.093083
-80.543050	59.294160	254.413100	.258217
-78.634010	59.473690	255.072000	.625956
-76.703000	59.625280	255.393100	.818855
-74.753460	59.748340	254.733600	.484676
-72.789070	59.842370	253.628500	.332191
-70.813740	59.906970	253.017000	.578806

-68.831480 59.941910 253.310200 .479788
-66.846440 59.947010 253.797600 .185160
-64.862770 59.922250 253.999100 .046734
-62.884610 59.867760 254.077000 .021869
-60.916040 59.783740 254.128000 .043312
-58.961010 59.670550 254.111600 .032884
-57.023270 59.528640 254.083700 .018982
-55.106350 59.358580 254.056500 .006311
-53.213500 59.161040 254.047900 .001887
-51.347600 58.936760 254.045500 .000502
-49.511150 58.686560 254.044900 .000120
-47.706190 58.411320 254.044900 .000093
-45.934240 58.111950 254.044900 .000090
-44.196280 57.789400 254.044900 .000087
-42.492650 57.444580 254.044900 .000080
-40.823030 57.078400 254.044900 .000107
-39.186360 56.691730 254.044900 .000107
-37.580830 56.285340 254.044900 .000107
-36.003750 55.859910 254.044900 .000107
-34.451600 55.415950 254.044900 .000107
-32.919830 54.953830 254.044900 .000107
-101.937200 56.083360 254.044900 .000101
-100.358100 56.549350 254.044900 .000107
-98.756940 56.996240 254.044900 .000087
-97.128790 57.423640 254.044900 .000087
-95.469860 57.830970 254.044900 .000090
-93.777320 58.217490 254.044900 .000104
-92.049300 58.582370 254.044900 .000100
-90.284800 58.924680 254.044900 .000175
-88.483640 59.243460 254.045500 .000995
-86.646400 59.537720 254.049200 .004537
-84.774360 59.806450 254.064700 .016477
-82.869400 60.048700 254.114300 .050983
-80.933970 60.263580 254.246800 .140770
-78.971000 60.450230 254.603000 .349679
-76.983890 60.607900 254.949400 .566957
-74.976370 60.735940 254.218900 .159762
-72.952470 60.833800 253.393900 .324899
-70.916480 60.901050 252.960800 .596216
-68.872850 60.937420 253.119700 .554646
-66.826130 60.942730 253.652000 .251438
-64.780930 60.916960 253.926100 .074108
-62.741830 60.860230 253.991900 .025971
-60.713310 60.772790 254.029800 .008013
-58.699720 60.654990 254.059600 .008789
-56.705200 60.507380 254.058000 .006554
-54.733590 60.330550 254.050300 .002849
-52.788400 60.125220 254.046400 .000986
-50.872730 59.892220 254.045100 .000285
-48.989200 59.632420 254.044900 .000086
-47.139900 59.346780 254.044900 .000087
-45.326320 59.036270 254.044900 .000087
-43.549270 58.701890 254.044900 .000083
-41.808860 58.344600 254.044900 .000107
-40.104370 57.965370 254.044900 .000107
-38.434220 57.565060 254.044900 .000107
-36.795950 57.144450 254.044900 .000107
-35.186060 56.704150 254.044900 .000107
-33.600030 56.244660 254.044900 .000107
-32.032190 55.766200 254.044900 .000107
-102.859900 56.896880 254.044900 .000107
-101.242000 57.379640 254.044900 .000107
-99.604390 57.842470 254.044900 .000110
-97.940930 58.285070 254.044900 .000083
-96.246660 58.706940 254.044900 .000083
-94.517820 59.107400 254.044900 .000093
-92.751850 59.485580 254.044900 .000093

-90.947170	59.840560	254.044900	.000097
-89.103220	60.171320	254.045000	.000386
-87.220290	60.476800	254.046300	.001913
-85.299550	60.755960	254.052700	.007203
-83.342830	61.007760	254.072800	.021459
-81.352710	61.231220	254.118700	.052511
-79.332320	61.425420	254.189700	.097053
-77.285290	61.589540	254.111700	.059438
-75.215710	61.722870	253.547100	.286375
-73.128000	61.824790	252.805000	.662352
-71.026890	61.894870	252.385000	.812737
-68.917300	61.932760	252.730300	.706594
-66.804310	61.938290	253.406200	.347815
-64.692990	61.911450	253.795600	.116488
-62.588420	61.852340	253.906000	.060686
-60.495590	61.761240	253.952100	.044535
-58.419300	61.638580	253.997600	.023635
-56.364090	61.484900	254.030300	.007047
-54.334160	61.300890	254.042400	.001311
-52.333320	61.087320	254.044400	.000184
-50.364880	60.845090	254.044800	.000145
-48.431600	60.575160	254.044800	.000140
-46.535590	60.278550	254.044800	.000118
-44.678250	59.956310	254.044900	.000101
-42.860180	59.609490	254.044900	.000107
-41.081100	59.239120	254.044900	.000107
-39.339830	58.846180	254.044900	.000107
-37.634110	58.431540	254.044900	.000107
-35.960610	57.995940	254.044900	.000107
-34.314830	57.539980	254.044900	.000107
-32.690990	57.064010	254.044900	.000107
-31.082000	56.568130	254.044900	.000107
-103.849600	57.699570	254.044900	.000107
-102.187300	58.200280	254.044900	.000107
-100.509000	58.680030	254.044900	.000107
-98.806840	59.138710	254.044900	.000107
-97.074520	59.575940	254.044900	.000087
-95.307120	59.991050	254.044900	.000101
-93.501110	60.383240	254.044900	.000112
-91.654170	60.751550	254.044900	.000137
-89.765210	61.094930	254.044900	.000150
-87.834170	61.412270	254.045200	.000630
-85.861950	61.702450	254.047000	.002404
-83.850420	61.964360	254.052700	.006649
-81.802150	62.196920	254.061600	.012375
-79.720490	62.399150	254.054500	.007904
-77.609360	62.570130	253.952400	.079991
-75.473210	62.709080	253.347300	.416653
-73.316960	62.815350	252.675100	.786636
-71.145790	62.888430	252.241800	.936212
-68.965200	62.927950	252.239900	.876396
-66.780790	62.933720	253.138300	.442648
-64.598260	62.905720	253.711500	.152410
-62.423240	62.844070	253.883800	.068316
-60.261290	62.749080	253.957700	.039705
-58.117710	62.621230	254.009000	.017795
-55.997520	62.461110	254.033600	.006353
-53.905320	62.269450	254.041700	.001795
-51.845240	62.047150	254.044000	.000473
-49.820820	61.795150	254.044700	.000145
-47.834930	61.514500	254.044800	.000144
-45.889670	61.206310	254.044900	.000130
-43.986290	60.871700	254.044900	.000098
-42.125070	60.511780	254.044900	.000107
-40.305270	60.127630	254.044900	.000107
-38.525020	59.720250	254.044900	.000107
-36.781200	59.290500	254.044900	.000107

-35.069400 58.839090 254.044900 .000107
-33.383770 58.366500 254.044900 .000107
-31.716990 57.872960 254.044900 .000107
-30.060130 57.358350 254.044900 .000107
-104.916500 58.490070 254.044900 .000107
-103.202300 59.010120 254.044900 .000107
-101.477600 59.507980 254.044900 .000107
-99.732440 59.983750 254.044900 .000107
-97.958720 60.437220 254.044900 .000107
-96.150010 60.867820 254.044900 .000107
-94.301540 61.274790 254.044900 .000107
-92.410060 61.657170 254.044900 .000112
-90.473700 62.013880 254.044900 .000128
-88.491920 62.343760 254.044900 .000160
-86.465330 62.645610 254.045200 .000551
-84.395630 62.918240 254.045900 .001227
-82.285480 63.160470 254.045200 .000519
-80.138370 63.371240 254.030000 .010982
-77.958560 63.549540 253.963200 .056090
-75.750930 63.694510 253.768600 .198350
-73.520860 63.805420 253.294100 .449022
-71.274150 63.881700 252.742100 .703826
-69.016910 63.922960 252.768100 .641302
-66.755400 63.928990 253.293000 .362345
-64.495980 63.899750 253.769800 .130997
-62.244980 63.835380 253.944600 .048765
-60.008560 63.736250 254.005600 .021494
-57.792640 63.602840 254.028700 .009100
-55.602750 63.435840 254.038900 .003455
-53.443960 63.236060 254.043000 .001158
-51.320750 63.004450 254.044200 .000359
-49.236890 62.742080 254.044700 .000142
-47.195330 62.450080 254.044900 .000136
-45.198120 62.129630 254.044900 .000107
-43.246250 61.781950 254.044900 .000107
-41.339580 61.408210 254.044900 .000107
-39.476710 61.009530 254.044900 .000107
-37.654880 60.586900 254.044900 .000107
-35.869840 60.141170 254.044900 .000107
-34.115770 59.672970 254.044900 .000107
-32.385130 59.182660 254.044900 .000107
-30.668580 58.670220 254.044900 .000107
-28.954870 58.135290 254.044900 .000107
-106.073800 59.266680 254.044900 .000107
-104.297500 59.807790 254.044900 .000107
-102.518800 60.325180 254.044900 .000107
-100.725000 60.819250 254.044900 .000107
-98.905530 61.289990 254.044900 .000107
-97.052100 61.737010 254.044900 .000107
-95.158340 62.159620 254.044900 .000107
-93.219700 62.556880 254.044900 .000107
-91.233350 62.927700 254.044900 .000107
-89.198040 63.270870 254.044900 .000106
-87.113920 63.585110 254.044900 .000086
-84.982480 63.869130 254.044800 .000125
-82.806390 64.121680 254.043300 .001462
-80.589290 64.341560 254.034900 .008065
-78.335770 64.527670 254.004900 .029357
-76.051160 64.679080 253.930300 .079334
-73.741460 64.794950 253.791000 .170439
-71.413090 64.874680 253.603400 .271547
-69.072890 64.917820 253.438500 .312465
-66.727910 64.924110 253.743300 .168868
-64.385280 64.893550 253.927500 .067397
-62.052090 64.826280 254.000800 .025436
-59.735260 64.722690 254.028000 .009821
-57.441380 64.583340 254.038200 .004008

-55.176610	64.408980	254.042400	.001564
-52.946510	64.200520	254.044100	.000547
-50.755950	63.959000	254.044600	.000180
-48.608900	63.685590	254.044800	.000140
-46.508400	63.381530	254.044900	.000096
-44.456330	63.048090	254.044900	.000107
-42.453320	62.686580	254.044900	.000107
-40.498620	62.298210	254.044900	.000107
-38.589950	61.884150	254.044900	.000107
-36.723370	61.445380	254.044900	.000107
-34.893150	60.982680	254.044900	.000107
-33.091610	60.496560	254.044900	.000107
-31.309040	59.987160	254.044900	.000107
-29.533510	59.454220	254.044900	.000107
-27.750760	58.896990	254.044900	.000107
-107.339200	60.027230	254.044900	.000107
-105.487000	60.591610	254.044900	.000107
-103.643800	61.130270	254.044900	.000107
-101.793500	61.644070	254.044900	.000107
-99.922550	62.133320	254.044900	.000107
-98.020040	62.597820	254.044900	.000107
-96.077510	63.037030	254.044900	.000107
-94.088710	63.450070	254.044900	.000107
-92.049460	63.835860	254.044900	.000107
-89.957580	64.193130	254.044900	.000107
-87.812570	64.520550	254.044900	.000107
-85.615560	64.816710	254.044800	.000142
-83.369120	65.080260	254.044200	.000833
-81.077050	65.309890	254.041200	.003744
-78.744270	65.504390	254.030400	.012010
-76.376630	65.662700	254.005000	.029975
-73.980770	65.783920	253.962100	.058415
-71.563890	65.867360	253.912900	.087758
-69.133670	65.912500	253.887500	.099744
-66.698060	65.919100	253.949800	.059418
-64.265090	65.887100	254.002300	.027491
-61.842790	65.816700	254.028300	.010967
-59.438900	65.708320	254.038800	.004164
-57.060850	65.562580	254.042700	.001569
-54.715470	65.380330	254.044000	.000600
-52.408880	65.162570	254.044600	.000205
-50.146370	64.910470	254.044900	.000124
-47.932120	64.625290	254.044900	.000087
-45.769150	64.308400	254.044900	.000107
-43.659080	63.961160	254.044900	.000107
-41.602020	63.584970	254.044900	.000107
-39.596350	63.181100	254.044900	.000107
-37.638600	62.750720	254.044900	.000107
-35.723270	62.294770	254.044900	.000107
-33.842640	61.813940	254.044900	.000107
-31.986660	61.308540	254.044900	.000107
-30.142740	60.778430	254.044900	.000107
-28.295590	60.222970	254.044900	.000107
-26.427080	59.640870	254.044900	.000107
-108.736700	60.768890	254.044900	.000107
-106.789100	61.359350	254.044900	.000107
-104.866900	61.921510	254.044900	.000107
-102.949500	62.456830	254.044900	.000107
-101.019200	62.966030	254.044900	.000107
-99.061880	63.449270	254.044900	.000107
-97.066180	63.906190	254.044900	.000107
-95.023590	64.336020	254.044900	.000107
-92.928180	64.737730	254.044900	.000107
-90.776390	65.110020	254.044900	.000107
-88.566850	65.451470	254.044900	.000107
-86.300130	65.760610	254.044900	.000114
-83.978550	66.035940	254.044700	.000443

-81.606060	66.276030	254.043500	.001872
-79.187900	66.479550	254.039300	.005798
-76.730480	66.645290	254.029200	.013657
-74.241170	66.772270	254.012700	.025148
-71.728080	66.859700	253.995800	.036010
-69.199870	66.907030	253.990200	.037978
-66.665550	66.913940	254.008000	.026141
-64.134220	66.880400	254.026800	.013394
-61.614990	66.806620	254.037700	.005745
-59.116610	66.693070	254.042400	.002153
-56.647430	66.540450	254.044000	.000768
-54.215080	66.349720	254.044600	.000264
-51.826330	66.121990	254.044800	.000131
-49.486860	65.858530	254.044900	.000090
-47.201050	65.560780	254.044900	.000107
-44.971820	65.230190	254.044900	.000107
-42.800360	64.868270	254.044900	.000107
-40.685980	64.476460	254.044900	.000107
-38.625890	64.056100	254.044900	.000107
-36.614990	63.608330	254.044900	.000107
-34.645670	63.134020	254.044900	.000107
-32.707610	62.633670	254.044900	.000107
-30.787590	62.107330	254.044900	.000107
-28.869240	61.554470	254.044900	.000107
-26.932890	60.973880	254.044900	.000107
-24.955330	60.363580	254.044900	.000107

Q.11a

-89.453400	32.991660	267.619100	.000094
-88.345930	33.191160	267.619100	.000099
-87.228100	33.381660	267.619100	.000092
-86.100230	33.562890	267.619100	.000164
-84.962690	33.734530	267.619200	.000460
-83.815900	33.896310	267.619300	.000718
-82.660300	34.047950	267.619300	.000699
-81.496410	34.189190	267.619100	.000432
-80.324730	34.319790	267.619000	.000596
-79.145840	34.439500	267.618800	.002812
-77.960310	34.548100	267.615800	.011880
-76.768760	34.645400	267.605400	.030335
-75.571840	34.731200	267.591500	.053483
-74.370180	34.805370	267.589500	.061233
-73.164480	34.867740	267.602400	.044266
-71.955430	34.918200	267.613000	.022094
-70.743730	34.956650	267.617500	.006480

-69.530080	34.983020	267.618700	.001286
-68.315220	34.997260	267.619000	.000135
-67.099880	34.999340	267.619100	.000113
-65.884770	34.989250	267.619100	.000098
-64.670640	34.967020	267.619100	.000092
-63.458190	34.932690	267.619100	.000092
-62.248150	34.886320	267.619100	.000092
-61.041230	34.828000	267.619100	.000092
-59.838120	34.757850	267.619100	.000092
-58.639500	34.676010	267.619100	.000092
-57.446040	34.582610	267.619100	.000092
-56.258390	34.477850	267.619100	.000092
-55.077160	34.361910	267.619100	.000092
-53.902950	34.235010	267.619100	.000092
-52.736340	34.097380	267.619100	.000092
-51.577860	33.949260	267.619100	.000092
-50.428010	33.790910	267.619100	.000092
-49.287250	33.622600	267.619100	.000092
-48.156040	33.444620	267.619100	.000092
-47.034740	33.257250	267.619100	.000092
-45.923690	33.060790	267.619100	.000092
-44.823220	32.855560	267.619100	.000092
-89.701210	33.918400	267.619000	.000191
-88.583520	34.125020	267.619000	.000218
-87.455040	34.322360	267.619000	.000205
-86.316120	34.510110	267.619300	.000636
-85.167100	34.687960	267.620500	.002322
-84.008420	34.855620	267.621300	.004159
-82.840550	35.012790	267.621300	.004285
-81.663990	35.159200	267.619800	.002807
-80.479300	35.294580	267.619000	.003401
-79.287040	35.418700	267.617100	.014567
-78.087840	35.531310	267.595800	.051465
-76.882320	35.632210	267.527900	.132684
-75.671150	35.721200	267.434200	.241051
-74.455030	35.798110	267.408500	.276982
-73.234660	35.862810	267.489600	.213732
-72.010770	35.915150	267.569200	.111473
-70.784100	35.955040	267.604500	.042456
-69.555380	35.982390	267.615800	.009610
-68.325390	35.997170	267.618500	.001401
-67.094890	35.999320	267.619000	.000327
-65.864640	35.988860	267.619100	.000239
-64.635410	35.965800	267.619000	.000110
-63.407950	35.930180	267.619100	.000091
-62.183030	35.882080	267.619100	.000092
-60.961370	35.821590	267.619100	.000092
-59.743720	35.748840	267.619100	.000092
-58.530780	35.663950	267.619100	.000092
-57.323260	35.567100	267.619100	.000092
-56.121820	35.458470	267.619100	.000092
-54.927120	35.338260	267.619100	.000092
-53.739770	35.206700	267.619100	.000092
-52.560380	35.064020	267.619100	.000092
-51.389480	34.910500	267.619100	.000092
-50.227610	34.746390	267.619100	.000092
-49.075240	34.571990	267.619100	.000092
-47.932830	34.387580	267.619100	.000092
-46.800740	34.193490	267.619100	.000092
-45.679330	33.990000	267.619100	.000092
-44.568900	33.777440	267.619100	.000092
-89.960660	34.843320	267.618700	.001292
-88.832330	35.057210	267.618500	.001671
-87.692770	35.261540	267.618100	.001948
-86.542320	35.455950	267.620800	.002497
-85.381340	35.640140	267.630300	.011599
-84.210270	35.813810	267.639200	.022334

-83.029580	35.976620	267.640700	.026200
-81.839790	36.128310	267.628600	.019049
-80.641480	36.268600	267.620000	.019663
-79.435230	36.397220	267.601100	.071786
-78.221690	36.513940	267.455100	.241690
-77.001530	36.618520	267.002600	.627544
-75.775430	36.710780	266.336200	1.132628
-74.544130	36.790520	266.133800	1.346120
-73.308360	36.857590	266.617600	1.050449
-72.068890	36.911860	267.192300	.597991
-70.826490	36.953220	267.487300	.242475
-69.581950	36.981590	267.586300	.072775
-68.336070	36.996900	267.613300	.012479
-67.089650	36.999130	267.618600	.001141
-65.843500	36.988290	267.619000	.001024
-64.598400	36.964370	267.619000	.000460
-63.355190	36.927440	267.619100	.000162
-62.114630	36.877570	267.619100	.000144
-60.877510	36.814860	267.619100	.000092
-59.644590	36.739430	267.619100	.000112
-58.416640	36.651430	267.619100	.000124
-57.194370	36.551040	267.619100	.000126
-55.978490	36.438440	267.619100	.000090
-54.769680	36.313860	267.619100	.000097
-53.568590	36.177530	267.619100	.000092
-52.375820	36.029710	267.619100	.000092
-51.191970	35.870660	267.619100	.000092
-50.017550	35.700660	267.619100	.000092
-48.853080	35.520030	267.619100	.000092
-47.698980	35.329070	267.619100	.000092
-46.555660	35.128100	267.619100	.000092
-45.423470	34.917440	267.619100	.000092
-44.302690	34.697410	267.619100	.000092
-90.232250	35.766320	267.616000	.007832
-89.092860	35.987640	267.612600	.010769
-87.941790	36.199090	267.607000	.015238
-86.779330	36.400320	267.621500	.010336
-85.605870	36.591000	267.680600	.036863
-84.421870	36.770790	267.744700	.086467
-83.227800	36.939400	267.763900	.117731
-82.024180	37.096500	267.690900	.100112
-80.811620	37.241800	267.627700	.092972
-79.590740	37.375040	267.522600	.261696
-78.362190	37.495960	266.787600	.926216
-77.126670	37.604320	264.349400	2.346200
-75.884930	37.699920	260.619200	4.578158
-74.637700	37.782550	259.211200	4.991005
-73.385770	37.852060	261.938500	3.946504
-72.129940	37.908310	264.923200	2.427560
-70.871030	37.951180	266.715600	1.064012
-69.609860	37.980580	267.388500	.342511
-68.347290	37.996460	267.576200	.077250
-67.084140	37.998780	267.616500	.008168
-65.821270	37.987530	267.619200	.003431
-64.559530	37.962740	267.618300	.001794
-63.299760	37.924460	267.619200	.000787
-62.042780	37.872770	267.619400	.000685
-60.789440	37.807780	267.619300	.000581
-59.540510	37.729610	267.619300	.000537
-58.296810	37.638420	267.619500	.000655
-57.059080	37.534390	267.619400	.000542
-55.828080	37.417740	267.619200	.000261
-54.604500	37.288680	267.619100	.000097
-53.389020	37.147460	267.619100	.000104
-52.182290	36.994370	267.619100	.000092
-50.984880	36.829660	267.619100	.000092
-49.797380	36.653650	267.619100	.000092

-48.620270	36.466650	267.619100	.000092
-47.454020	36.268990	267.619100	.000092
-46.299010	36.060990	267.619100	.000092
-45.155610	35.843010	267.619100	.000092
-44.024070	35.615370	267.619100	.000092
-90.516530	36.687310	267.595700	.037370
-89.365660	36.916220	267.568800	.055238
-88.202590	37.134950	267.521400	.084583
-87.027650	37.343150	267.568400	.083552
-85.841200	37.540460	267.858800	.120559
-84.643710	37.726540	268.185500	.271944
-83.435650	37.901050	268.362700	.451942
-82.217600	38.063690	268.027900	.462222
-80.990140	38.214140	267.674300	.378432
-79.753940	38.352110	267.362900	.722593
-78.509670	38.477340	264.392200	2.730564
-77.258070	38.589580	254.236300	7.661020
-75.999910	38.688600	236.035300	14.646570
-74.735980	38.774210	230.401900	14.002610
-73.467090	38.846230	242.459500	10.486710
-72.194090	38.904510	255.428600	7.119155
-70.917830	38.948930	263.154100	3.449100
-69.639210	38.979390	266.435300	1.168715
-68.359090	38.995840	267.410800	.274977
-67.078360	38.998240	267.615100	.037150
-65.797930	38.986590	267.621900	.012390
-64.518690	38.960900	267.615100	.006185
-63.241520	38.921240	267.621300	.003629
-61.967320	38.867690	267.622900	.004046
-60.696920	38.800350	267.621800	.003514
-59.431210	38.719360	267.621900	.003225
-58.170970	38.624900	267.622400	.003384
-56.917050	38.517150	267.622100	.002700
-55.670200	38.396340	267.620200	.001218
-54.431160	38.262690	267.619200	.000305
-53.200640	38.116470	267.619100	.000098
-51.979310	37.957960	267.619100	.000109
-50.767770	37.787460	267.619100	.000092
-49.566610	37.605300	267.619100	.000092
-48.376340	37.411790	267.619100	.000092
-47.197410	37.207270	267.619100	.000092
-46.030250	36.992090	267.619100	.000092
-44.875180	36.766620	267.619100	.000092
-43.732470	36.531200	267.619100	.000092
-90.814130	37.606180	267.490800	.137795
-89.651300	37.842850	267.346600	.207504
-88.475770	38.069030	267.092000	.313816
-87.287830	38.284350	267.205900	.457573
-86.087840	38.488450	268.347700	.743885
-84.876290	38.680970	269.675800	1.148450
-83.653650	38.861550	270.735600	1.965544
-82.420500	39.029860	269.238200	2.045789
-81.177470	39.185590	267.840200	1.853311
-79.925230	39.328410	267.676600	1.643770
-78.664510	39.458070	258.746600	5.649833
-77.396060	39.574290	226.480500	17.693320
-76.120680	39.676830	159.280600	32.911570
-74.839220	39.765490	142.635500	23.409040
-73.552520	39.840080	181.467800	20.826830
-72.261480	39.900440	226.099500	14.954150
-70.967010	39.946460	251.976300	7.749387
-69.670030	39.978020	263.419200	2.745040
-68.371470	39.995060	266.980800	.583720
-67.072270	39.997550	267.686300	.107861
-65.773400	39.985470	267.641200	.048227
-64.475770	39.958870	267.601000	.019618
-63.180340	39.917780	267.638900	.016304

-61.888020	39.862300	267.644100	.019469
-60.599740	39.792560	267.637500	.015978
-59.316390	39.708680	267.635400	.014047
-58.038840	39.610860	267.636500	.013643
-56.767930	39.499290	267.633400	.010777
-55.504470	39.374200	267.623900	.003828
-54.249260	39.235840	267.619800	.000746
-53.003000	39.084480	267.619000	.000087
-51.766410	38.920440	267.619000	.000115
-50.540120	38.744010	267.619100	.000111
-49.324710	38.555530	267.619100	.000094
-48.120720	38.355340	267.619100	.000092
-46.928620	38.143810	267.619100	.000092
-45.748810	37.921300	267.619100	.000092
-44.581620	37.688170	267.619100	.000092
-43.427300	37.444810	267.619100	.000092
-91.125630	38.522790	267.135000	.373044
-89.950410	38.767390	266.598400	.527936
-88.761920	39.001200	265.746600	.969780
-87.560450	39.223820	265.938600	1.989336
-86.346370	39.434880	269.889700	3.835533
-85.120160	39.633990	273.921400	5.827603
-83.882300	39.820790	279.617600	8.300433
-82.633390	39.994930	275.021400	8.221432
-81.374080	40.156070	269.528400	7.285645
-80.105060	40.303890	271.079000	5.872957
-78.827100	40.438090	259.593100	5.802555
-77.540980	40.558400	205.294500	18.506670
-76.247540	40.664560	94.824440	31.728390
-74.947690	40.756360	6.904687	20.034380
-73.642300	40.833600	45.605930	43.201720
-72.332310	40.896110	158.928600	27.350480
-71.018690	40.943760	228.878900	12.192500
-69.702430	40.976440	258.047100	4.241735
-68.384490	40.994090	266.937000	.825309
-67.065880	40.996670	268.316200	.548289
-65.747600	40.984170	267.715100	.161343
-64.430650	40.956610	267.535600	.058974
-63.116020	40.914060	267.745700	.082789
-61.804690	40.856610	267.749800	.072028
-60.497620	40.784390	267.706000	.058988
-59.195770	40.697540	267.689200	.049196
-57.900040	40.596260	267.687000	.046449
-56.611330	40.480750	267.672900	.036646
-55.330490	40.351270	267.631100	.008172
-54.058330	40.208070	267.619300	.000499
-52.795620	40.051450	267.618300	.000760
-51.543080	39.881710	267.618700	.000331
-50.301390	39.699200	267.619000	.000123
-49.071130	39.504250	267.619100	.000104
-47.852850	39.297230	267.619100	.000092
-46.647030	39.078520	267.619100	.000092
-45.454070	38.848490	267.619100	.000092
-44.274280	38.607530	267.619100	.000092
-43.107920	38.356030	267.619100	.000092
-91.451740	39.437030	266.352700	.724417
-90.263630	39.689750	264.942400	1.051824
-89.061680	39.931370	262.968400	2.362248
-87.846150	40.161480	261.786000	6.004309
-86.617400	40.379670	268.566600	10.844080
-85.375900	40.585540	277.100300	15.281070
-84.122160	40.778730	286.797100	21.878670
-82.856800	40.958850	268.358900	20.657670
-81.580460	41.125550	256.610700	16.391550
-80.293880	41.278500	274.594400	17.152150
-78.997860	41.417370	293.187000	18.073790
-77.693230	41.541890	259.812100	22.090170

-76.380870 41.651780 143.924300 26.413030
-75.061690 41.746810 140.453300 79.192040
-73.736660 41.826770 30.487800 41.865000
-72.406780 41.891490 94.217730 46.813260
-71.073040 41.940830 208.652300 15.344580
-69.736500 41.974670 256.338400 4.837564
-68.398190 41.992950 269.398200 1.484354
-67.059160 41.995620 270.169700 1.170485
-65.720480 41.982670 267.325600 .274163
-64.383220 41.954140 267.247500 .277379
-63.048400 41.910080 268.305500 .340604
-61.717090 41.850600 268.159900 .253304
-60.390290 41.775820 267.943200 .171841
-59.069010 41.685920 267.856400 .147723
-57.754210 41.581080 267.836300 .143119
-56.446830 41.461530 267.791300 .121492
-55.147770 41.327530 267.623700 .006036
-53.857880 41.179360 267.609100 .008375
-52.577970 41.017320 267.614500 .003888
-51.308780 40.841740 267.617800 .001266
-50.051000 40.652980 267.618800 .000298
-48.805250 40.451390 267.619000 .000115
-47.572090 40.237360 267.619100 .000102
-46.352010 40.011290 267.619100 .000092
-45.145370 39.773560 267.619100 .000092
-43.952510 39.524580 267.619100 .000092
-42.773630 39.264750 267.619100 .000092
-91.793200 40.348750 265.268200 1.149286
-90.591710 40.609810 262.024500 1.676396
-89.375760 40.859430 254.855600 3.328907
-88.145600 41.097210 240.401500 6.952527
-86.901570 41.322720 214.153000 8.467371
-85.644150 41.535550 195.414400 9.334646
-84.373860 41.735290 181.284000 18.514920
-83.091290 41.921560 164.549400 21.800470
-81.797160 42.093980 160.765900 16.923690
-80.492230 42.252200 220.758100 17.502230
-79.177290 42.395880 292.372400 19.121080
-77.853240 42.524730 295.248200 32.318280
-76.521000 42.638450 91.238350 40.516330
-75.181550 42.736820 7.756972 24.427970
-73.835900 42.819590 109.227800 37.862080
-72.485100 42.886590 198.326500 25.372290
-71.130210 42.937670 251.815400 11.598850
-69.772340 42.972710 263.865000 3.160742
-68.412590 42.991630 271.003500 1.274731
-67.052090 42.994400 269.226300 1.062837
-65.691960 42.980990 265.815200 1.302291
-64.333320 42.951450 267.040500 .842692
-62.977290 42.905830 270.754100 1.352532
-61.624970 42.844260 269.432700 .735250
-60.277440 42.766850 268.584800 .462620
-58.935750 42.673790 268.285500 .410603
-57.600940 42.565280 268.073600 .307606
-56.273990 42.441570 267.657900 .047214
-54.955840 42.302930 267.465100 .126193
-53.647390 42.149640 267.569800 .038591
-52.349470 41.982030 267.605500 .011155
-51.062890 41.800450 267.616000 .002809
-49.788320 41.605260 267.618400 .000601
-48.526440 41.396860 267.619000 .000143
-47.277780 41.175640 267.619100 .000120
-46.042850 40.942000 267.619100 .000092
-44.822010 40.696380 267.619100 .000092
-43.615560 40.439180 267.619100 .000092
-42.423690 40.170830 267.619100 .000092
-92.150770 41.257820 264.703800 1.611760

-90.935390 41.527410 259.619500 2.776728
-89.704910 41.785250 244.022200 5.472552
-88.459540 42.030900 207.848600 11.867940
-87.199620 42.263930 150.026300 25.503590
-85.925610 42.483890 101.658500 21.309910
-84.638040 42.690380 64.025850 23.107030
-83.337520 42.882970 5.173763 13.152550
-82.024790 43.061280 88.791530 22.232980
-80.700610 43.224930 285.752200 29.806800
-79.365870 43.373580 339.857500 28.344260
-78.021450 43.506890 330.088900 29.257480
-76.668370 43.624570 235.040200 24.703910
-75.307630 43.726360 182.710200 39.858170
-73.940300 43.812040 202.698000 24.490040
-72.567500 43.881390 258.833100 15.512410
-71.190370 43.934270 262.201100 7.980680
-69.810050 43.970550 276.395100 2.928439
-68.427750 43.990140 285.040100 4.662388
-67.044650 43.993000 274.343600 2.720456
-65.661930 43.979120 270.777700 1.776746
-64.280810 43.948540 272.573700 1.960258
-62.902460 43.901310 274.448800 2.608997
-61.528050 43.837570 271.657400 1.587081
-60.158730 43.757450 269.221100 .766257
-58.795620 43.661140 268.219100 .414423
-57.439790 43.548850 267.796500 .271208
-56.092310 43.420850 267.355900 .355441
-54.754150 43.277410 267.469400 .153970
-53.426270 43.118850 267.567300 .048900
-52.109530 42.945500 267.604700 .014321
-50.804760 42.757750 267.615900 .003505
-49.512690 42.555960 267.618500 .000724
-48.233980 42.340550 267.619000 .000139
-46.969190 42.111940 267.619100 .000109
-45.718810 41.870550 267.619100 .000092
-44.483210 41.616830 267.619100 .000092
-43.262650 41.351200 267.619100 .000092
-42.057300 41.074100 267.619100 .000092
-92.525320 42.164070 265.076300 1.764573
-91.295520 42.442420 260.120900 3.524103
-90.049940 42.708690 243.850100 7.794984
-88.788760 42.962440 208.760900 14.362080
-87.512310 43.203190 156.872900 19.903700
-86.221000 43.430500 114.826500 15.544150
-84.915410 43.643920 103.448400 40.203300
-83.596150 43.843030 8.125046 22.818660
-82.263960 44.027400 110.173400 41.415000
-80.919660 44.196660 390.655700 58.953740
-79.564150 44.350410 415.306100 46.711010
-78.198390 44.488330 368.270200 30.968400
-76.823410 44.610100 258.685800 11.930640
-75.440290 44.715440 160.229900 32.769130
-74.050180 44.804110 199.543800 21.825800
-72.654240 44.875890 238.844100 15.139990
-71.253690 44.930630 272.189700 4.549896
-69.849760 44.968180 290.222200 6.437048
-68.443720 44.988460 313.868000 13.853960
-67.036810 44.991420 289.556300 6.466722
-65.630330 44.977050 282.598600 5.310542
-64.225530 44.945390 279.914900 4.515572
-62.823690 44.896510 278.363700 3.967154
-61.426040 44.830540 275.129800 2.898297
-60.033810 44.747610 270.511700 1.444784
-58.648170 44.647940 269.159900 .898339
-57.270290 44.531750 268.597500 .681319
-55.901260 44.399320 267.621300 .538155
-54.542130 44.250930 267.563900 .173643

-53.193900	44.086940	267.589700	.051670
-51.857480	43.907680	267.611100	.014360
-50.533710	43.713560	267.617500	.003632
-49.223370	43.504980	267.618800	.000712
-47.927120	43.282370	267.619000	.000140
-46.645540	43.046150	267.619000	.000078
-45.379100	42.796800	267.619100	.000092
-44.128160	42.534760	267.619100	.000092
-42.892960	42.260480	267.619100	.000092
-41.673610	41.974410	267.619100	.000092
-92.917770	43.067330	265.580500	1.582771
-91.673000	43.354680	261.152700	3.459324
-90.411740	43.629630	245.587000	8.160558
-89.134110	43.891690	210.601100	14.937060
-87.840450	44.140390	157.056900	22.653370
-86.531140	44.375260	101.468200	22.779550
-85.206750	44.595830	76.651310	17.589390
-83.867900	44.801650	140.042100	29.608340
-82.515380	44.992280	214.109600	36.084080
-81.150000	45.167310	273.404700	29.354260
-79.772730	45.326340	274.691100	15.740180
-78.384560	45.469020	277.554300	11.228580
-76.986600	45.595010	265.528100	8.739584
-75.579970	45.704020	258.309200	10.869760
-74.165890	45.795780	256.930800	8.069625
-72.745610	45.870080	280.655900	4.676880
-71.320400	45.926740	290.549100	6.002349
-69.891600	45.965610	311.045500	11.141070
-68.460530	45.986610	317.108300	14.095710
-67.028560	45.989670	283.497300	6.566684
-65.597030	45.974800	278.898700	4.322871
-64.167300	45.942020	280.193900	4.524732
-62.740720	45.891430	280.563100	4.557771
-61.318600	45.823140	277.459300	3.458728
-59.902260	45.737310	272.416100	1.783812
-58.492950	45.634170	270.060200	1.039636
-57.091900	45.513950	268.261100	.729012
-55.700260	45.376930	267.576000	.408390
-54.319140	45.223450	267.453800	.228431
-52.949590	45.053840	267.609600	.072238
-51.592580	44.868490	267.629700	.023622
-50.248970	44.667810	267.623600	.008150
-48.919560	44.452230	267.620000	.002225
-47.605040	44.222190	267.619200	.000465
-46.305980	43.978160	267.619100	.000117
-45.022840	43.720610	267.619100	.000092
-43.755960	43.450020	267.619100	.000092
-42.505550	43.166850	267.619100	.000092
-41.271660	42.871580	267.619100	.000092
-93.329110	43.967430	266.033000	1.261477
-92.068780	44.264030	262.448400	2.744734
-90.791210	44.547890	250.277800	5.802987
-89.496510	44.818510	222.637700	8.647316
-88.184940	45.075390	169.265300	13.630840
-86.856880	45.318050	92.957660	28.575700
-85.512890	45.545990	47.535820	41.306480
-84.153590	45.758730	144.878400	30.395620
-82.779790	45.955820	170.520000	24.001130
-81.392350	46.136820	181.701700	12.465490
-79.992260	46.301310	178.333600	25.364690
-78.580580	46.448910	233.059400	8.335824
-77.158460	46.579270	273.478400	9.431053
-75.727110	46.692070	274.999900	7.646625
-74.287820	46.787040	292.843000	7.996742
-72.841890	46.863950	303.868500	9.769079
-71.390720	46.922590	308.244800	10.740850
-69.935700	46.962830	321.179900	15.289340

-68.478260	46.984570	301.209000	9.652125
-67.019850	46.987740	285.140000	5.700456
-65.561920	46.972340	283.917200	5.440938
-64.105920	46.938410	285.615800	6.034570
-62.653260	46.886040	287.657600	6.686672
-61.205390	46.815350	282.280600	4.901614
-59.763680	46.726520	275.000500	2.713689
-58.329470	46.619790	271.098400	1.539953
-56.904060	46.495390	268.498200	1.020359
-55.488670	46.353640	267.499300	.858205
-54.084500	46.194880	267.973400	.341582
-52.692630	46.019470	267.888700	.157388
-51.314070	45.827840	267.723700	.060933
-49.949730	45.620390	267.648000	.020665
-48.600430	45.397590	267.625000	.006116
-47.266860	45.159900	267.619800	.001389
-45.949600	44.907820	267.619100	.000230
-44.649100	44.641840	267.619100	.000091
-43.365670	44.362450	267.619100	.000092
-42.099450	44.070140	267.619100	.000092
-40.850440	43.765410	267.619100	.000092
-93.760410	44.864160	266.514300	.963103
-92.483920	45.170280	263.880300	2.247854
-91.189420	45.463320	254.978600	5.352046
-89.876960	45.742750	229.575800	11.262530
-88.546760	46.008070	167.438100	27.223860
-87.199170	46.258760	96.472920	52.058750
-85.834720	46.494300	97.602810	51.514740
-84.454070	46.714200	184.451100	19.675550
-83.058010	46.917960	210.461800	11.502610
-81.647480	47.105130	212.460000	9.491650
-80.223460	47.275260	216.645200	13.495580
-78.787090	47.427960	233.253800	11.890060
-77.339580	47.562840	256.185900	6.197120
-75.882230	47.679570	279.550300	4.468376
-74.416370	47.777870	292.031400	7.384709
-72.943440	47.857470	297.072100	8.450544
-71.464880	47.918190	299.388300	8.761249
-69.982220	47.959850	292.965100	7.346002
-68.496960	47.982350	282.745700	4.832046
-67.010670	47.985630	282.690300	4.971195
-65.524890	47.969690	285.468900	5.705825
-64.041170	47.934570	290.793700	7.165049
-62.561040	47.880340	288.346300	6.476084
-61.086020	47.807170	284.951900	5.627957
-59.617590	47.715230	279.758500	4.176335
-58.157170	47.604770	274.864700	2.797859
-56.706150	47.476050	270.643900	1.738110
-55.265830	47.329400	269.926900	1.210889
-53.837470	47.165180	269.721100	.928113
-52.422200	46.983780	268.584800	.407175
-51.021090	46.785640	267.902800	.125004
-49.635090	46.571200	267.688400	.037513
-48.265020	46.340950	267.632800	.010570
-46.911610	46.095370	267.621100	.002460
-45.575410	45.834990	267.619300	.000445
-44.256860	45.560320	267.619100	.000094
-42.956210	45.271870	267.619100	.000092
-41.673580	44.970160	267.619100	.000092
-40.408870	44.655690	267.619100	.000092
-94.212860	45.757310	266.943500	.661067
-92.919560	46.073230	265.403900	1.546415
-91.607470	46.375730	261.286400	3.193779
-90.276550	46.664250	249.674400	6.741709
-88.926960	46.938270	221.982400	14.450910
-87.559010	47.197250	191.026400	28.579250
-86.173240	47.440650	196.576100	24.773620

-84.770290	47.667940	215.778700	15.245520
-83.350960	47.878600	225.437000	12.928960
-81.916200	48.072160	235.007600	10.314400
-80.467090	48.248140	241.937600	9.745644
-79.004780	48.406110	247.401200	8.878404
-77.530590	48.545680	266.390400	2.941629
-76.045850	48.666500	276.181000	3.054829
-74.552020	48.768250	281.723900	5.068534
-73.050600	48.850660	282.805500	5.208643
-71.543170	48.913520	282.376400	4.757036
-70.031330	48.956650	276.460100	3.301517
-68.516710	48.979950	262.004300	5.091704
-67.000980	48.983350	267.904000	2.476439
-65.485790	48.966840	276.733300	2.990686
-63.972820	48.930470	283.301400	4.811255
-62.463700	48.874330	285.689900	5.497744
-60.960050	48.798580	287.211600	6.023350
-59.463460	48.703410	286.319100	6.023089
-57.975440	48.589080	279.512600	4.113787
-56.497490	48.455880	272.162400	1.998076
-55.030970	48.304140	270.525000	1.238053
-53.577210	48.134270	269.977500	.981672
-52.137430	47.946660	268.720600	.448750
-50.712720	47.741790	267.934000	.138222
-49.304080	47.520120	267.694500	.040877
-47.912350	47.282170	267.633900	.011164
-46.538250	47.028450	267.621200	.002612
-45.182340	46.759510	267.619300	.000462
-43.845020	46.475880	267.619100	.000099
-42.526500	46.178100	267.619100	.000092
-41.226800	45.866700	267.619100	.000092
-39.945740	45.542200	267.619100	.000092
-94.687740	46.646660	267.196000	.447652
-93.376940	46.972690	266.328200	1.012297
-92.046550	47.284930	265.359500	1.674598
-90.696440	47.582840	261.234400	3.745229
-89.326680	47.865840	247.522200	8.680196
-87.937550	48.133390	231.549600	16.617050
-86.529520	48.384910	225.096400	19.615430
-85.103260	48.619850	227.410100	17.192380
-83.659580	48.837660	241.749400	9.984788
-82.199460	49.037830	251.689500	6.827760
-80.724000	49.219880	262.142100	3.621288
-79.234450	49.383340	266.531100	2.281329
-77.732160	49.527770	271.281900	1.947333
-76.218590	49.652830	273.082000	2.252247
-74.695260	49.758160	275.371800	3.363214
-73.163800	49.843490	276.652400	3.661124
-71.625870	49.908580	276.626200	3.497125
-70.083210	49.953250	274.629400	2.840067
-68.537570	49.977380	269.600600	1.768628
-66.990740	49.980900	270.639000	1.382361
-65.444490	49.963800	274.855000	2.357122
-63.900620	49.926140	277.874200	3.282148
-62.360880	49.868010	279.808500	3.798855
-60.827010	49.789570	281.101300	4.225590
-59.300730	49.691040	282.004500	4.544305
-57.783640	49.572690	279.325300	3.874388
-56.277320	49.434830	271.769400	1.585790
-54.783270	49.277820	269.277500	.733052
-53.302860	49.102080	268.548000	.422449
-51.837360	48.908040	268.058600	.201426
-50.387950	48.696200	267.766100	.076132
-48.955640	48.467040	267.657000	.025564
-47.541310	48.221130	267.626500	.007413
-46.145660	47.959000	267.620100	.001664
-44.769230	47.681210	267.619200	.000301

-43.412380	47.388330	267.619100	.000092
-42.075270	47.080920	267.619100	.000092
-40.757810	46.759540	267.619100	.000092
-39.459720	46.424710	267.619100	.000092
-95.186440	47.531940	267.364100	.348052
-93.857410	47.868410	266.921400	.764518
-92.508000	48.190720	267.897200	.955800
-91.137910	48.498310	266.044600	1.554806
-89.747150	48.790600	261.125200	3.437048
-88.335950	49.067010	258.236700	5.014471
-86.904710	49.326930	259.238000	5.089028
-85.454100	49.569790	254.167100	5.842739
-83.984930	49.795020	255.361300	5.081379
-82.498220	50.002060	261.507400	2.753244
-80.995120	50.190400	268.232300	1.492084
-79.476920	50.359550	271.459900	2.113751
-77.945060	50.509060	271.374200	1.546809
-76.401090	50.638520	272.137500	1.657299
-74.846650	50.747580	273.913800	2.636235
-73.283450	50.835950	275.276900	3.251464
-71.713320	50.903350	274.542200	2.704205
-70.138080	50.949630	274.157300	2.613863
-68.559640	50.974620	271.568900	1.888095
-66.979900	50.978270	272.660400	2.062951
-65.400800	50.960560	272.304100	1.801901
-63.824260	50.921550	271.748700	1.700194
-62.252170	50.861340	272.084500	1.747719
-60.686400	50.780110	272.829600	1.945784
-59.128770	50.678080	273.674000	2.109547
-57.581030	50.555550	271.896200	1.611888
-56.044860	50.412850	269.420200	.785495
-54.521820	50.250360	268.298600	.357545
-53.013420	50.068520	267.922100	.178806
-51.520960	49.867810	267.753500	.081757
-50.045680	49.648740	267.665300	.033416
-48.588630	49.411830	267.631000	.011757
-47.150700	49.157670	267.621200	.003235
-45.732590	48.886830	267.619300	.000681
-44.334810	48.599900	267.619100	.000116
-42.957660	48.297460	267.619100	.000092
-41.601190	47.980120	267.619100	.000092
-40.265250	47.648430	267.619100	.000092
-38.949390	47.302930	267.619100	.000092
-95.710510	48.412890	267.578100	.340628
-94.362470	48.760130	267.614400	.715445
-92.993230	49.092850	268.945300	.945182
-91.602360	49.410460	267.902900	.465213
-90.189750	49.712350	266.995600	.747921
-88.755540	49.997940	267.336300	1.006131
-87.300090	50.266570	267.712300	1.274717
-85.824040	50.517650	266.567600	1.277287
-84.328210	50.750570	266.346500	1.335381
-82.813610	50.964740	265.942200	1.038853
-81.281460	51.159630	268.326600	.882628
-79.733130	51.334700	271.466600	2.147378
-78.170130	51.489480	270.766800	1.393075
-76.594090	51.623530	270.806200	1.363005
-75.006800	51.736490	272.948300	2.372421
-73.410070	51.828010	274.681300	3.199650
-71.805870	51.897850	272.990100	2.199152
-70.196170	51.945790	272.914900	1.970556
-68.582990	51.971680	271.934000	1.799221
-66.968440	51.975460	271.944400	1.726357
-65.354560	51.957120	270.783900	1.237862
-63.743440	51.916690	270.067900	1.033759
-62.137130	51.854320	269.133000	.761491
-60.537630	51.770170	269.547800	.866530

-58.946900	51.664510	269.434400	.791351
-57.366820	51.537620	268.874600	.585558
-55.799180	51.389870	268.184000	.310141
-54.245650	51.221680	267.822400	.153320
-52.707820	51.033510	267.696800	.067381
-51.187080	50.825860	267.650100	.028888
-49.684720	50.599280	267.629400	.011468
-48.201800	50.354330	267.621500	.003781
-46.739240	50.091630	267.619400	.000971
-45.297720	49.811770	267.619100	.000184
-43.877700	49.515370	267.619100	.000091
-42.479410	49.203060	267.619100	.000092
-41.102810	48.875440	267.619100	.000092
-39.747590	48.533100	267.619100	.000092
-38.413150	48.176570	267.619100	.000092
-96.261670	49.289190	267.894100	.405714
-94.893750	49.647600	268.699500	.929486
-93.503820	49.991080	268.927800	.646713
-92.091320	50.319060	268.664100	.566956
-90.655950	50.630910	268.272900	.398970
-89.197760	50.925990	268.154700	.300949
-87.717060	51.203660	268.120300	.281168
-86.214420	51.463270	267.895100	.277550
-84.690660	51.704190	267.989400	.348291
-83.146820	51.925780	268.522000	.503105
-81.584160	52.127480	269.336100	.917703
-80.004110	52.308710	270.815900	1.886271
-78.408280	52.468990	270.246900	1.487908
-76.798410	52.607840	269.731300	1.078930
-75.176390	52.724850	271.423900	1.875354
-73.544200	52.819690	273.436200	2.905073
-71.903920	52.892050	271.085300	1.576742
-70.257710	52.941730	270.663600	1.209911
-68.607750	52.968570	270.980000	1.470722
-66.956280	52.972490	270.322300	1.376410
-65.305560	52.953480	268.838000	.729770
-63.657820	52.911580	267.730100	.418382
-62.015270	52.846950	267.214500	.338392
-60.380080	52.759750	267.855100	.235448
-58.754360	52.650280	268.003000	.243510
-57.140120	52.518850	267.907600	.179344
-55.539290	52.365840	267.750500	.107927
-53.953660	52.191710	267.663500	.049924
-52.384890	51.996940	267.633400	.020124
-50.834480	51.782070	267.624300	.007801
-49.303730	51.547700	267.620600	.002824
-47.793770	51.294400	267.619400	.000873
-46.305490	51.022820	267.619100	.000198
-44.839570	50.733620	267.619100	.000092
-43.396390	50.427420	267.619100	.000092
-41.976090	50.104880	267.619100	.000092
-40.578500	49.766630	267.619100	.000092
-39.203150	49.413260	267.619100	.000092
-37.849210	49.045330	267.619100	.000092
-96.841810	50.160530	268.037100	.308526
-95.453030	50.530490	268.536200	.524565
-94.041510	50.885130	268.352800	.387060
-92.606450	51.223850	268.072300	.262938
-91.147380	51.546020	267.925800	.176907
-89.664200	51.850960	267.876300	.121997
-88.157130	52.138020	267.867500	.097911
-86.626690	52.406500	267.863600	.096085
-85.073680	52.655720	268.032900	.177344
-83.499180	52.885050	268.535900	.431488
-81.904440	53.093850	268.683100	.593422
-80.290990	53.281520	269.470500	1.272289
-78.660530	53.447530	270.039400	1.754149

-77.014920	53.591380	269.349400	1.052365
-75.356160	53.712650	270.541000	1.551378
-73.686420	53.810940	272.986700	2.836340
-72.007930	53.885960	270.329100	1.289647
-70.322990	53.937460	269.308000	.687126
-68.634010	53.965280	269.495100	.881862
-66.943400	53.969350	269.197900	1.227258
-65.253590	53.949630	267.435300	.585715
-63.567000	53.906200	266.929800	.349637
-61.886040	53.839190	267.261900	.169997
-60.213040	53.748820	267.555800	.049752
-58.550290	53.635360	267.662700	.055048
-56.899960	53.499180	267.666200	.046588
-55.264110	53.340690	267.641400	.027684
-53.644650	53.160350	267.625700	.012131
-52.043340	52.958710	267.620900	.004445
-50.461760	52.736320	267.619700	.001495
-48.901280	52.493820	267.619200	.000520
-47.363030	52.231850	267.619100	.000164
-45.847900	51.951060	267.619100	.000091
-44.356530	51.652150	267.619100	.000092
-42.889220	51.335790	267.619100	.000092
-41.445980	51.002650	267.619100	.000092
-40.026490	50.653380	267.619100	.000092
-38.630060	50.288590	267.619100	.000092
-37.255620	49.908830	267.619100	.000092
-97.453060	51.026530	267.803700	.117689
-96.042340	51.408490	267.896100	.160404
-94.608220	51.774700	267.847800	.132620
-93.149600	52.124570	267.766400	.093764
-91.665800	52.457450	267.716100	.066614
-90.156560	52.772640	267.697200	.050809
-88.621980	53.069450	267.696200	.046095
-87.062480	53.347160	267.714900	.051069
-85.478850	53.605050	267.798100	.088800
-83.872130	53.842430	267.992400	.191804
-82.243660	54.058630	268.189000	.345864
-80.595020	54.253030	268.686400	.829714
-78.928000	54.425040	269.967300	1.876499
-77.244600	54.574130	269.387700	1.219358
-75.546970	54.699840	270.217700	1.454424
-73.837420	54.801750	272.728800	2.834242
-72.118360	54.879540	270.179000	1.238454
-70.392330	54.932960	268.380900	.288882
-68.661900	54.961820	267.377600	.670500
-66.929700	54.966030	266.661800	1.279257
-65.198370	54.945580	267.058600	.486801
-63.470540	54.900540	267.264100	.185554
-61.748820	54.831050	267.445800	.066068
-60.035740	54.737340	267.565000	.023052
-58.333780	54.619720	267.613500	.008715
-56.645260	54.478570	267.623600	.008107
-54.972420	54.314330	267.621500	.005172
-53.317280	54.127510	267.619800	.002197
-51.681740	53.918690	267.619200	.000671
-50.067420	53.688470	267.619100	.000232
-48.475770	53.437500	267.619100	.000100
-46.907930	53.166490	267.619100	.000092
-45.364770	52.876130	267.619100	.000092
-43.846840	52.567130	267.619100	.000092
-42.354370	52.240220	267.619100	.000092
-40.887210	51.896080	267.619100	.000092
-39.444820	51.535370	267.619100	.000092
-38.026250	51.158730	267.619100	.000092
-36.630120	50.766700	267.619100	.000092
-98.097820	51.886770	267.677700	.039402
-96.663910	52.281200	267.689400	.044972

-95.206040	52.659450	267.677600	.039161
-93.722790	53.020900	267.656100	.029098
-92.213200	53.364910	267.642000	.021277
-90.676770	53.690770	267.636000	.016982
-89.113440	53.997730	267.636000	.016482
-87.523560	54.285050	267.645100	.021438
-85.907830	54.551970	267.677700	.038340
-84.267300	54.797760	267.753100	.087361
-82.603340	55.021710	267.910200	.209614
-80.917590	55.223140	268.356600	.576235
-79.211960	55.401440	269.442800	1.475976
-77.488560	55.556020	269.817700	1.597561
-75.749730	55.686380	270.135800	1.460776
-73.997920	55.792100	272.229700	2.619057
-72.235800	55.872810	270.235900	1.241439
-70.466090	55.928230	267.577000	.382869
-68.691570	55.958180	265.196900	1.866565
-66.915130	55.962550	266.000000	1.219786
-65.139640	55.941330	267.049100	.414110
-63.367970	55.894590	267.410800	.129428
-61.602940	55.822490	267.542800	.043781
-59.847310	55.725280	267.596300	.015964
-58.103760	55.603290	267.614700	.003842
-56.374820	55.456930	267.619000	.000752
-54.662870	55.286670	267.619200	.000684
-52.970100	55.093080	267.619100	.000260
-51.298500	54.876750	267.619100	.000109
-49.649800	54.638340	267.619100	.000091
-48.025460	54.378550	267.619100	.000092
-46.426660	54.098120	267.619100	.000092
-44.854210	53.797780	267.619100	.000092
-43.308580	53.478290	267.619100	.000092
-41.789850	53.140400	267.619100	.000092
-40.297680	52.784830	267.619100	.000092
-38.831280	52.412250	267.619100	.000092
-37.389370	52.023280	267.619100	.000092
-35.970190	51.618480	267.619100	.000092
-98.778840	52.740810	267.633400	.011878
-97.320270	53.148240	267.633800	.011661
-95.837370	53.539010	267.630900	.010495
-94.328290	53.912530	267.626300	.007951
-92.791720	54.268120	267.623100	.005568
-91.226900	54.605080	267.621600	.004277
-89.633560	54.922620	267.621700	.004362
-88.011890	55.219970	267.624500	.006804
-86.362530	55.496330	267.635400	.015795
-84.686490	55.750910	267.667600	.042348
-82.985150	55.982970	267.764500	.124235
-81.260250	56.191770	268.073600	.370382
-79.513790	56.376650	268.910500	1.026246
-77.748020	56.536990	270.233800	1.959344
-75.965460	56.672260	270.149700	1.527799
-74.168790	56.781970	271.661300	2.342366
-72.360860	56.865740	270.236700	1.213398
-70.544630	56.923280	266.809200	.911241
-68.723180	56.954370	264.899000	1.993096
-66.899620	56.958910	266.436300	.843292
-65.077100	56.936880	267.224500	.279596
-63.258730	56.888360	267.501200	.087849
-61.447630	56.813510	267.586500	.028734
-59.646790	56.712620	267.611500	.008494
-57.859090	56.586040	267.617800	.002028
-56.087300	56.434210	267.619000	.000278
-54.333970	56.257640	267.619000	.000078
-52.601470	56.056940	267.619100	.000091
-50.891880	55.832740	267.619100	.000092
-49.207030	55.585770	267.619100	.000092

-47.548420 55.316760 267.619100 .000092
-45.917200 55.026500 267.619100 .000092
-44.314130 54.715760 267.619100 .000092
-42.739580 54.385350 267.619100 .000092
-41.193420 54.036030 267.619100 .000092
-39.675070 53.668560 267.619100 .000092
-38.183400 53.283620 267.619100 .000092
-36.716770 52.881820 267.619100 .000092
-35.272900 52.463700 267.619100 .000092
-99.499240 53.588130 267.621700 .003152
-98.014280 54.009130 267.621400 .002779
-96.504860 54.412960 267.620800 .002413
-94.968610 54.799050 267.620100 .001766
-93.403790 55.166730 267.619500 .001145
-91.809290 55.515250 267.619300 .000791
-90.184560 55.843850 267.619300 .000866
-88.529620 56.151670 267.620000 .001882
-86.844990 56.437890 267.623400 .006166
-85.131650 56.701690 267.637800 .022074
-83.390960 56.942230 267.695100 .074833
-81.624720 57.158780 267.900500 .241951
-79.835050 57.350580 268.535800 .737223
-78.024370 57.516980 270.115100 1.866348
-76.195360 57.657400 270.277000 1.679541
-74.350940 57.771310 271.118100 2.055410
-72.494220 57.858320 269.580300 .839411
-70.628410 57.918090 265.987600 1.657669
-68.756890 57.950390 266.334000 1.050451
-66.883060 57.955100 267.039000 .418618
-65.010370 57.932220 267.425000 .144816
-63.142240 57.881810 267.565000 .049269
-61.282040 57.804080 267.606800 .014462
-59.433060 57.699310 267.616900 .003636
-57.598450 57.567890 267.618700 .000725
-55.781200 57.410300 267.619000 .000147
-53.984060 57.227100 267.619100 .000099
-52.209560 57.018940 267.619100 .000092
-50.459930 56.786500 267.619100 .000092
-48.737060 56.530560 267.619100 .000092
-47.042490 56.251900 267.619100 .000092
-45.377330 55.951360 267.619100 .000092
-43.742270 55.629770 267.619100 .000092
-42.137470 55.287960 267.619100 .000092
-40.562610 54.926740 267.619100 .000092
-39.016750 54.546860 267.619100 .000092
-37.498410 54.149020 267.619100 .000092
-36.005400 53.733830 267.619100 .000092
-34.534880 53.301770 267.619100 .000092
-100.262700 54.428120 267.619400 .000692
-98.749250 54.863350 267.619300 .000538
-97.211560 55.280850 267.619200 .000441
-95.646590 55.680060 267.619200 .000312
-94.052090 56.060360 267.619100 .000193
-92.426510 56.420970 267.619100 .000154
-90.768940 56.761100 267.619100 .000188
-89.079190 57.079890 267.619200 .000509
-87.357570 57.376450 267.620200 .002515
-85.605000 57.649890 267.626800 .011493
-83.822840 57.899360 267.658600 .044855
-82.012920 58.124030 267.785500 .153392
-80.177480 58.323120 268.209000 .483040
-78.319110 58.495910 269.378100 1.335667
-76.440710 58.641750 270.396500 1.872466
-74.545430 58.760110 270.190400 1.525684
-72.636660 58.850530 268.328700 .316681
-70.717930 58.912650 267.125600 .920051
-68.792920 58.946230 267.249100 .447044

-66.865380 58.951130 267.458400 .169094
-64.939080 58.927340 267.562600 .058710
-63.017780 58.874950 267.603500 .018179
-61.105210 58.794160 267.616000 .004876
-59.204930 58.685300 267.618500 .001054
-57.320400 58.548780 267.619000 .000232
-55.454840 58.385130 267.619000 .000131
-53.611270 58.194950 267.619100 .000099
-51.792370 57.978940 267.619100 .000092
-50.000500 57.737840 267.619100 .000092
-48.237630 57.472490 267.619100 .000092
-46.505310 57.183730 267.619100 .000092
-44.804610 56.872430 267.619100 .000092
-43.136050 56.539500 267.619100 .000092
-41.499610 56.185780 267.619100 .000092
-39.894630 55.812120 267.619100 .000092
-38.319800 55.419290 267.619100 .000092
-36.773090 55.007980 267.619100 .000092
-35.251730 54.578750 267.619100 .000092
-33.752120 54.132060 267.619100 .000092
-101.073700 55.260130 267.619100 .000147
-99.529010 55.710320 267.619100 .000098
-97.960900 56.142140 267.619100 .000109
-96.365430 56.555100 267.619100 .000103
-94.739640 56.948590 267.619100 .000107
-93.081440 57.321860 267.619000 .000098
-91.389490 57.674070 267.619000 .000123
-89.663250 58.004340 267.619100 .000191
-87.902820 58.311730 267.619400 .001032
-86.109000 58.595320 267.622000 .005671
-84.283100 58.854170 267.637300 .024598
-82.427000 59.087400 267.703200 .086018
-80.543050 59.294160 267.928000 .270262
-78.634010 59.473690 268.553200 .766815
-76.703000 59.625280 269.888800 1.755352
-74.753460 59.748340 269.032800 .893114
-72.789070 59.842370 267.727100 .419755
-70.813740 59.906970 268.299200 .362669
-68.831480 59.941910 267.912000 .182531
-66.846440 59.947010 267.727300 .061467
-64.862770 59.922250 267.649800 .018850
-62.884610 59.867760 267.625600 .004283
-60.916040 59.783740 267.619900 .000932
-58.961010 59.670550 267.619100 .000221
-57.023270 59.528640 267.619000 .000142
-55.106350 59.358580 267.619000 .000136
-53.213500 59.161040 267.619000 .000083
-51.347600 58.936760 267.619100 .000092
-49.511150 58.686560 267.619100 .000092
-47.706190 58.411320 267.619100 .000092
-45.934240 58.111950 267.619100 .000092
-44.196280 57.789400 267.619100 .000092
-42.492650 57.444580 267.619100 .000092
-40.823030 57.078400 267.619100 .000092
-39.186360 56.691730 267.619100 .000092
-37.580830 56.285340 267.619100 .000092
-36.003750 55.859910 267.619100 .000092
-34.451600 55.415950 267.619100 .000092
-32.919830 54.953830 267.619100 .000092
-101.937200 56.083360 267.619100 .000091
-100.358100 56.549350 267.619100 .000092
-98.756940 56.996240 267.619100 .000099
-97.128790 57.423640 267.619100 .000102
-95.469860 57.830970 267.619100 .000107
-93.777320 58.217490 267.619100 .000124
-92.049300 58.582370 267.619000 .000124
-90.284800 58.924680 267.619000 .000136

-88.483640	59.243460	267.619100	.000409
-86.646400	59.537720	267.620100	.002480
-84.774360	59.806450	267.626400	.011787
-82.869400	60.048700	267.657000	.044033
-80.933970	60.263580	267.767800	.139255
-78.971000	60.450230	268.094400	.413776
-76.983890	60.607900	268.872400	1.045643
-74.976370	60.735940	268.169800	.478582
-72.952470	60.833800	267.634800	.300883
-70.916480	60.901050	268.373000	.304964
-68.872850	60.937420	268.254500	.222322
-66.826130	60.942730	267.881200	.086741
-64.780930	60.916960	267.701500	.035175
-62.741830	60.860230	267.638800	.011967
-60.713310	60.772790	267.622400	.003363
-58.699720	60.654990	267.619400	.000728
-56.705200	60.507380	267.619000	.000155
-54.733590	60.330550	267.619000	.000139
-52.788400	60.125220	267.619000	.000109
-50.872730	59.892220	267.619100	.000097
-48.989200	59.632420	267.619100	.000092
-47.139900	59.346780	267.619100	.000092
-45.326320	59.036270	267.619100	.000092
-43.549270	58.701890	267.619100	.000092
-41.808860	58.344600	267.619100	.000092
-40.104370	57.965370	267.619100	.000092
-38.434220	57.565060	267.619100	.000092
-36.795950	57.144450	267.619100	.000092
-35.186060	56.704150	267.619100	.000092
-33.600030	56.244660	267.619100	.000092
-32.032190	55.766200	267.619100	.000092
-102.859900	56.896880	267.619100	.000092
-101.242000	57.379640	267.619100	.000092
-99.604390	57.842470	267.619100	.000097
-97.940930	58.285070	267.619100	.000099
-96.246660	58.706940	267.619100	.000104
-94.517820	59.107400	267.619000	.000100
-92.751850	59.485580	267.619000	.000124
-90.947170	59.840560	267.619000	.000139
-89.103220	60.171320	267.619100	.000193
-87.220290	60.476800	267.619300	.000881
-85.299550	60.755960	267.621200	.004405
-83.342830	61.007760	267.631000	.016634
-81.352710	61.231220	267.663900	.047997
-79.332320	61.425420	267.729900	.102823
-77.285290	61.589540	267.715000	.101515
-75.215710	61.722870	266.707500	.814570
-73.128000	61.824790	266.376800	1.187078
-71.026890	61.894870	267.511300	.672708
-68.917300	61.932760	268.216300	.343412
-66.804310	61.938290	267.897700	.138950
-64.692990	61.911450	267.713400	.046051
-62.588420	61.852340	267.643100	.014034
-60.495590	61.761240	267.623400	.003699
-58.419300	61.638580	267.619500	.000786
-56.364090	61.484900	267.619100	.000177
-54.334160	61.300890	267.619000	.000139
-52.333320	61.087320	267.619000	.000098
-50.364880	60.845090	267.619100	.000094
-48.431600	60.575160	267.619100	.000092
-46.535590	60.278550	267.619100	.000092
-44.678250	59.956310	267.619100	.000092
-42.860180	59.609490	267.619100	.000092
-41.081100	59.239120	267.619100	.000092
-39.339830	58.846180	267.619100	.000092
-37.634110	58.431540	267.619100	.000092
-35.960610	57.995940	267.619100	.000092

-34.314830 57.539980 267.619100 .000092
-32.690990 57.064010 267.619100 .000092
-31.082000 56.568130 267.619100 .000092
-103.849600 57.699570 267.619100 .000092
-102.187300 58.200280 267.619100 .000092
-100.509000 58.680030 267.619100 .000094
-98.806840 59.138710 267.619100 .000097
-97.074520 59.575940 267.619100 .000102
-95.307120 59.991050 267.619000 .000100
-93.501110 60.383240 267.619000 .000123
-91.654170 60.751550 267.619000 .000138
-89.765210 61.094930 267.619000 .000147
-87.834170 61.412270 267.619100 .000249
-85.861950 61.702450 267.619400 .001194
-83.850420 61.964360 267.621400 .004284
-81.802150 62.196920 267.626600 .009874
-79.720490 62.399150 267.624400 .010739
-77.609360 62.570130 267.543700 .071859
-75.473210 62.709080 267.154800 .453663
-73.316960 62.815350 266.211900 1.292592
-71.145790 62.888430 265.904200 1.759653
-68.965200 62.927950 267.491100 .802725
-66.780790 62.933720 267.711800 .261097
-64.598260 62.905720 267.671400 .068730
-62.423240 62.844070 267.635400 .015477
-60.261290 62.749080 267.622300 .003084
-58.117710 62.621230 267.619400 .000543
-55.997520 62.461110 267.619000 .000130
-53.905320 62.269450 267.619000 .000133
-51.845240 62.047150 267.619000 .000080
-49.820820 61.795150 267.619100 .000092
-47.834930 61.514500 267.619100 .000092
-45.889670 61.206310 267.619100 .000092
-43.986290 60.871700 267.619100 .000092
-42.125070 60.511780 267.619100 .000092
-40.305270 60.127630 267.619100 .000092
-38.525020 59.720250 267.619100 .000092
-36.781200 59.290500 267.619100 .000092
-35.069400 58.839090 267.619100 .000092
-33.383770 58.366500 267.619100 .000092
-31.716990 57.872960 267.619100 .000092
-30.060130 57.358350 267.619100 .000092
-104.916500 58.490070 267.619100 .000092
-103.202300 59.010120 267.619100 .000092
-101.477600 59.507980 267.619100 .000092
-99.732440 59.983750 267.619100 .000094
-97.958720 60.437220 267.619100 .000097
-96.150010 60.867820 267.619100 .000094
-94.301540 61.274790 267.619000 .000100
-92.410060 61.657170 267.619000 .000123
-90.473700 62.013880 267.619000 .000138
-88.491920 62.343760 267.619000 .000116
-86.465330 62.645610 267.619100 .000227
-84.395630 62.918240 267.619300 .000606
-82.285480 63.160470 267.619100 .000825
-80.138370 63.371240 267.611600 .009749
-77.958560 63.549540 267.567800 .048208
-75.750930 63.694510 267.422700 .183229
-73.520860 63.805420 267.121700 .489192
-71.274150 63.881700 266.856500 .842536
-69.016910 63.922960 266.933700 .926069
-66.755400 63.928990 267.513600 .301005
-64.495980 63.899750 267.618600 .075267
-62.244980 63.835380 267.622800 .015303
-60.008560 63.736250 267.620000 .002559
-57.792640 63.602840 267.619100 .000361
-55.602750 63.435840 267.619000 .000141

-53.443960	63.236060	267.619100	.000102
-51.320750	63.004450	267.619100	.000097
-49.236890	62.742080	267.619100	.000092
-47.195330	62.450080	267.619100	.000092
-45.198120	62.129630	267.619100	.000092
-43.246250	61.781950	267.619100	.000092
-41.339580	61.408210	267.619100	.000092
-39.476710	61.009530	267.619100	.000092
-37.654880	60.586900	267.619100	.000092
-35.869840	60.141170	267.619100	.000092
-34.115770	59.672970	267.619100	.000092
-32.385130	59.182660	267.619100	.000092
-30.668580	58.670220	267.619100	.000092
-28.954870	58.135290	267.619100	.000092
-106.073800	59.266680	267.619100	.000092
-104.297500	59.807790	267.619100	.000092
-102.518800	60.325180	267.619100	.000092
-100.725000	60.819250	267.619100	.000092
-98.905530	61.289990	267.619100	.000094
-97.052100	61.737010	267.619100	.000092
-95.158340	62.159620	267.619100	.000092
-93.219700	62.556880	267.619000	.000100
-91.233350	62.927700	267.619000	.000123
-89.198040	63.270870	267.619100	.000092
-87.113920	63.585110	267.619100	.000091
-84.982480	63.869130	267.619000	.000155
-82.806390	64.121680	267.618700	.000924
-80.589290	64.341560	267.615800	.005776
-78.335770	64.527670	267.600600	.023284
-76.051160	64.679080	267.555400	.064899
-73.741460	64.794950	267.474000	.148083
-71.413090	64.874680	267.408200	.252792
-69.072890	64.917820	267.433800	.286349
-66.727910	64.924110	267.573700	.121809
-64.385280	64.893550	267.613500	.034821
-62.052090	64.826280	267.619000	.008056
-59.735260	64.722690	267.619000	.001420
-57.441380	64.583340	267.619000	.000190
-55.176610	64.408980	267.619000	.000144
-52.946510	64.200520	267.619100	.000092
-50.755950	63.959000	267.619100	.000092
-48.608900	63.685590	267.619100	.000092
-46.508400	63.381530	267.619100	.000092
-44.456330	63.048090	267.619100	.000092
-42.453320	62.686580	267.619100	.000092
-40.498620	62.298210	267.619100	.000092
-38.589950	61.884150	267.619100	.000092
-36.723370	61.445380	267.619100	.000092
-34.893150	60.982680	267.619100	.000092
-33.091610	60.496560	267.619100	.000092
-31.309040	59.987160	267.619100	.000092
-29.533510	59.454220	267.619100	.000092
-27.750760	58.896990	267.619100	.000092
-107.339200	60.027230	267.619100	.000092
-105.487000	60.591610	267.619100	.000092
-103.643800	61.130270	267.619100	.000092
-101.793500	61.644070	267.619100	.000092
-99.922550	62.133320	267.619100	.000092
-98.020040	62.597820	267.619100	.000092
-96.077510	63.037030	267.619100	.000092
-94.088710	63.450070	267.619100	.000092
-92.049460	63.835860	267.619000	.000100
-89.957580	64.193130	267.619100	.000092
-87.812570	64.520550	267.619100	.000092
-85.615560	64.816710	267.619100	.000100
-83.369120	65.080260	267.619000	.000374
-81.077050	65.309890	267.618300	.002039

-78.744270	65.504390	267.614700	.007708
-76.376630	65.662700	267.603000	.021114
-73.980770	65.783920	267.582800	.042457
-71.563890	65.867360	267.568200	.064670
-69.133670	65.912500	267.576600	.073262
-66.698060	65.919100	267.605400	.034986
-64.265090	65.887100	267.616300	.011884
-61.842790	65.816700	267.618600	.002882
-59.438900	65.708320	267.619000	.000505
-57.060850	65.562580	267.619000	.000116
-54.715470	65.380330	267.619000	.000130
-52.408880	65.162570	267.619100	.000092
-50.146370	64.910470	267.619100	.000092
-47.932120	64.625290	267.619100	.000092
-45.769150	64.308400	267.619100	.000092
-43.659080	63.961160	267.619100	.000092
-41.602020	63.584970	267.619100	.000092
-39.596350	63.181100	267.619100	.000092
-37.638600	62.750720	267.619100	.000092
-35.723270	62.294770	267.619100	.000092
-33.842640	61.813940	267.619100	.000092
-31.986660	61.308540	267.619100	.000092
-30.142740	60.778430	267.619100	.000092
-28.295590	60.222970	267.619100	.000092
-26.427080	59.640870	267.619100	.000092
-108.736700	60.768890	267.619100	.000092
-106.789100	61.359350	267.619100	.000092
-104.866900	61.921510	267.619100	.000092
-102.949500	62.456830	267.619100	.000092
-101.019200	62.966030	267.619100	.000092
-99.061880	63.449270	267.619100	.000092
-97.066180	63.906190	267.619100	.000092
-95.023590	64.336020	267.619100	.000092
-92.928180	64.737730	267.619100	.000092
-90.776390	65.110020	267.619100	.000092
-88.566850	65.451470	267.619100	.000092
-86.300130	65.760610	267.619100	.000094
-83.978550	66.035940	267.619000	.000128
-81.606060	66.276030	267.618900	.000727
-79.187900	66.479550	267.618000	.002863
-76.730480	66.645290	267.614800	.007635
-74.241170	66.772270	267.609000	.014893
-71.728080	66.859700	267.604800	.021340
-69.199870	66.907030	267.607300	.021443
-66.665550	66.913940	267.614300	.012162
-64.134220	66.880400	267.617700	.004512
-61.614990	66.806620	267.618800	.001162
-59.116610	66.693070	267.619000	.000279
-56.647430	66.540450	267.619100	.000101
-54.215080	66.349720	267.619100	.000094
-51.826330	66.121990	267.619100	.000092
-49.486860	65.858530	267.619100	.000092
-47.201050	65.560780	267.619100	.000092
-44.971820	65.230190	267.619100	.000092
-42.800360	64.868270	267.619100	.000092
-40.685980	64.476460	267.619100	.000092
-38.625890	64.056100	267.619100	.000092
-36.614990	63.608330	267.619100	.000092
-34.645670	63.134020	267.619100	.000092
-32.707610	62.633670	267.619100	.000092
-30.787590	62.107330	267.619100	.000092
-28.869240	61.554470	267.619100	.000092
-26.932890	60.973880	267.619100	.000092
-24.955330	60.363580	267.619100	.000092

Q.12a

-89.453400	32.991660	281.926700	.004822
-88.345930	33.191160	281.929700	.012294
-87.228100	33.381660	281.938200	.032609
-86.100230	33.562890	281.954500	.067576
-84.962690	33.734530	281.975700	.109714
-83.815900	33.896310	281.993900	.143763
-82.660300	34.047950	282.003100	.158231
-81.496410	34.189190	281.999800	.143412
-80.324730	34.319790	281.978600	.091336
-79.145840	34.439500	281.923000	.027121
-77.960310	34.548100	281.789900	.235879
-76.768760	34.645400	281.554000	.553781
-75.571840	34.731200	281.308800	.840025
-74.370180	34.805370	281.230400	.932389
-73.164480	34.867740	281.334400	.829331
-71.955430	34.918200	281.511000	.653770
-70.743730	34.956650	281.689000	.435812
-69.530080	34.983020	281.814300	.239355
-68.315220	34.997260	281.882500	.104760
-67.099880	34.999340	281.912100	.035778
-65.884770	34.989250	281.922000	.009294
-64.670640	34.967020	281.924600	.001997
-63.458190	34.932690	281.925200	.000488
-62.248150	34.886320	281.925200	.000210
-61.041230	34.828000	281.925400	.000163
-59.838120	34.757850	281.925400	.000101
-58.639500	34.676010	281.925400	.000131
-57.446040	34.582610	281.925500	.000185
-56.258390	34.477850	281.925500	.000185
-55.077160	34.361910	281.925500	.000183
-53.902950	34.235010	281.925500	.000183
-52.736340	34.097380	281.925500	.000183
-51.577860	33.949260	281.925500	.000183
-50.428010	33.790910	281.925500	.000183
-49.287250	33.622600	281.925500	.000183
-48.156040	33.444620	281.925500	.000183
-47.034740	33.257250	281.925500	.000183
-45.923690	33.060790	281.925500	.000183
-44.823220	32.855560	281.925500	.000183
-89.701210	33.918400	281.927600	.009698
-88.583520	34.125020	281.936800	.030099
-87.455040	34.322360	281.964500	.087804
-86.316120	34.510110	282.020400	.192438
-85.167100	34.687960	282.095200	.319607
-84.008420	34.855620	282.156600	.416832
-82.840550	35.012790	282.186700	.453032

-81.663990	35.159200	282.177800	.419505
-80.479300	35.294580	282.126100	.310795
-79.287040	35.418700	281.997300	.107958
-78.087840	35.531310	281.625900	.419335
-76.882320	35.632210	280.973600	1.093567
-75.671150	35.721200	280.200600	1.775296
-74.455030	35.798110	280.005400	1.875428
-73.234660	35.862810	280.317700	1.624384
-72.010770	35.915150	280.825300	1.238090
-70.784100	35.955040	281.297800	.845088
-69.555380	35.982390	281.641500	.460329
-68.325390	35.997170	281.824100	.195137
-67.094890	35.999320	281.898000	.060589
-65.864640	35.988860	281.920000	.013821
-64.635410	35.965800	281.924300	.003029
-63.407950	35.930180	281.924900	.000969
-62.183030	35.882080	281.925000	.000451
-60.961370	35.821590	281.925200	.000317
-59.743720	35.748840	281.925200	.000198
-58.530780	35.663950	281.925400	.000143
-57.323260	35.567100	281.925400	.000140
-56.121820	35.458470	281.925400	.000131
-54.927120	35.338260	281.925400	.000129
-53.739770	35.206700	281.925500	.000185
-52.560380	35.064020	281.925500	.000183
-51.389480	34.910500	281.925500	.000183
-50.227610	34.746390	281.925500	.000183
-49.075240	34.571990	281.925500	.000183
-47.932830	34.387580	281.925500	.000183
-46.800740	34.193490	281.925500	.000183
-45.679330	33.990000	281.925500	.000183
-44.568900	33.777440	281.925500	.000183
-89.960660	34.843320	281.929700	.029010
-88.832330	35.057210	281.965500	.100085
-87.692770	35.261540	282.079500	.315017
-86.542320	35.455950	282.320000	.712531
-85.381340	35.640140	282.645300	1.193746
-84.210270	35.813810	282.901400	1.539040
-83.029580	35.976620	283.015900	1.644068
-81.839790	36.128310	282.985600	1.524517
-80.641480	36.268600	282.818300	1.243068
-79.435230	36.397220	282.420900	.655149
-78.221690	36.513940	281.253300	.789495
-77.001530	36.618520	278.855100	2.881568
-75.775430	36.710780	275.896900	4.624259
-74.544130	36.790520	275.691100	4.415380
-73.308360	36.857590	276.752000	3.693956
-72.068890	36.911860	278.276600	2.960236
-70.826490	36.953220	279.893500	1.966250
-69.581950	36.981590	281.019200	1.078586
-68.336070	36.996900	281.632600	.428601
-67.089650	36.999130	281.866400	.139421
-65.843500	36.988290	281.922400	.054895
-64.598400	36.964370	281.926200	.019983
-63.355190	36.927440	281.924600	.004455
-62.114630	36.877570	281.924400	.001328
-60.877510	36.814860	281.924700	.000972
-59.644590	36.739430	281.925000	.000644
-58.416640	36.651430	281.925100	.000392
-57.194370	36.551040	281.925300	.000203
-55.978490	36.438440	281.925400	.000172
-54.769680	36.313860	281.925400	.000141
-53.568590	36.177530	281.925400	.000133
-52.375820	36.029710	281.925400	.000129
-51.191970	35.870660	281.925500	.000183
-50.017550	35.700660	281.925400	.000097
-48.853080	35.520030	281.925500	.000183

-47.698980	35.329070	281.925500	.000183
-46.555660	35.128100	281.925500	.000183
-45.423470	34.917440	281.925500	.000183
-44.302690	34.697410	281.925500	.000183
-90.232250	35.766320	281.923300	.076339
-89.092860	35.987640	282.048200	.307425
-87.941790	36.199090	282.469300	1.024517
-86.779330	36.400320	283.411700	2.396238
-85.605870	36.591000	284.746100	4.082229
-84.421870	36.770790	285.694200	5.170037
-83.227800	36.939400	286.129100	5.483493
-82.024180	37.096500	286.043200	5.147032
-80.811620	37.241800	285.427800	4.276214
-79.590740	37.375040	284.580000	3.059326
-78.362190	37.495960	280.873400	1.805041
-77.126670	37.604320	272.090500	7.235090
-75.884930	37.699920	262.501600	11.671510
-74.637700	37.782550	262.529300	10.111850
-73.385770	37.852060	268.456700	7.114573
-72.129940	37.908310	271.475600	6.237197
-70.871030	37.951180	275.874100	4.502573
-69.609860	37.980580	279.437400	2.329202
-68.347290	37.996460	281.243500	1.001285
-67.084140	37.998780	281.889500	.507053
-65.821270	37.987530	281.975300	.276960
-64.559530	37.962740	281.944100	.099733
-63.299760	37.924460	281.924200	.018165
-62.042780	37.872770	281.921800	.004741
-60.789440	37.807780	281.923500	.003280
-59.540510	37.729610	281.924600	.002263
-58.296810	37.638420	281.925000	.001497
-57.059080	37.534390	281.925300	.000945
-55.828080	37.417740	281.925400	.000476
-54.604500	37.288680	281.925400	.000240
-53.389020	37.147460	281.925400	.000153
-52.182290	36.994370	281.925400	.000133
-50.984880	36.829660	281.925400	.000129
-49.797380	36.653650	281.925400	.000099
-48.620270	36.466650	281.925400	.000097
-47.454020	36.268990	281.925500	.000183
-46.299010	36.060990	281.925500	.000183
-45.155610	35.843010	281.925500	.000183
-44.024070	35.615370	281.925500	.000183
-90.516530	36.687310	281.848700	.145117
-89.365660	36.916220	282.207900	.743639
-88.202590	37.134950	283.536300	2.776083
-87.027650	37.343150	286.919500	6.829528
-85.841200	37.540460	292.085100	12.363860
-84.643710	37.726540	295.450700	15.576410
-83.435650	37.901050	296.726800	16.259050
-82.217600	38.063690	296.075700	14.942370
-80.990140	38.214140	294.052200	12.735580
-79.753940	38.352110	293.040500	11.116830
-78.509670	38.477340	282.136700	5.846486
-77.258070	38.589580	257.260900	15.182070
-75.999910	38.688600	221.440600	28.110560
-74.735980	38.774210	233.998200	20.374340
-73.467090	38.846230	248.016200	15.467180
-72.194090	38.904510	258.190900	11.737160
-70.917830	38.948930	266.617000	8.985174
-69.639210	38.979390	276.195400	5.041079
-68.359090	38.995840	281.099100	2.868342
-67.078360	38.998240	282.420500	2.007031
-65.797930	38.986590	282.262000	.983597
-64.518690	38.960900	282.011600	.317645
-63.241520	38.921240	281.919000	.036435
-61.967320	38.867690	281.910300	.025056

-60.696920	38.800350	281.918800	.017484
-59.431210	38.719360	281.923700	.009090
-58.170970	38.624900	281.925400	.006438
-56.917050	38.517150	281.925900	.004047
-55.670200	38.396340	281.925900	.002229
-54.431160	38.262690	281.925600	.000898
-53.200640	38.116470	281.925400	.000311
-51.979310	37.957960	281.925400	.000152
-50.767770	37.787460	281.925400	.000129
-49.566610	37.605300	281.925400	.000104
-48.376340	37.411790	281.925400	.000097
-47.197410	37.207270	281.925400	.000097
-46.030250	36.992090	281.925500	.000183
-44.875180	36.766620	281.925500	.000183
-43.732470	36.531200	281.925500	.000183
-90.814130	37.606180	281.472500	.342846
-89.651300	37.842850	282.230200	1.282870
-88.475770	38.069030	285.482200	5.076266
-87.287830	38.284350	296.264800	15.806710
-86.087840	38.488450	314.416300	31.958250
-84.876290	38.680970	325.392000	41.247500
-83.653650	38.861550	327.689000	40.998150
-82.420500	39.029860	323.753300	36.150940
-81.177470	39.185590	315.785600	30.079330
-79.925230	39.328410	319.368800	30.758810
-78.664510	39.458070	304.447400	25.389040
-77.396060	39.574290	244.371000	19.975550
-76.120680	39.676830	146.993000	69.987740
-74.839220	39.765490	167.131000	46.516140
-73.552520	39.840080	206.510800	30.982480
-72.261480	39.900440	223.464000	24.815690
-70.967010	39.946460	250.359200	16.821330
-69.670030	39.978020	271.589000	9.759226
-68.371470	39.995060	283.145500	8.507762
-67.072270	39.997550	285.000900	6.155538
-65.773400	39.985470	283.260400	2.682420
-64.475770	39.958870	282.102300	.573940
-63.180340	39.917780	281.863800	.109644
-61.888020	39.862300	281.870300	.124575
-60.599740	39.792560	281.905900	.074908
-59.316390	39.708680	281.924100	.042289
-58.038840	39.610860	281.929000	.027815
-56.767930	39.499290	281.930400	.018086
-55.504470	39.374200	281.928600	.009175
-54.249260	39.235840	281.926700	.003835
-53.003000	39.084480	281.925800	.001145
-51.766410	38.920440	281.925500	.000295
-50.540120	38.744010	281.925400	.000103
-49.324710	38.555530	281.925300	.000040
-48.120720	38.355340	281.925400	.000102
-46.928620	38.143810	281.925400	.000097
-45.748810	37.921300	281.925400	.000097
-44.581620	37.688170	281.925500	.000185
-43.427300	37.444810	281.925500	.000183
-91.125630	38.522790	280.189500	1.502749
-89.950410	38.767390	280.388700	1.499559
-88.761920	39.001200	285.514600	4.977847
-87.560450	39.223820	311.545900	27.435410
-86.346370	39.434880	360.696400	60.644490
-85.120160	39.633990	390.229200	80.412090
-83.882300	39.820790	395.337600	81.336680
-82.633390	39.994930	379.920900	66.535320
-81.374080	40.156070	352.207000	50.627700
-80.105060	40.303890	374.776600	61.720100
-78.827100	40.438090	390.325900	77.357140
-77.540980	40.558400	330.086900	55.417440
-76.247540	40.664560	192.020600	74.412950

-74.947690	40.756360	71.270020	65.098880
-73.642300	40.833600	140.096500	121.983600
-72.332310	40.896110	144.708200	39.223510
-71.018690	40.943760	220.033400	28.345050
-69.702430	40.976440	270.564300	18.578830
-68.384490	40.994090	290.396500	18.364260
-67.065880	40.996670	291.059500	12.853850
-65.747600	40.984170	285.125700	5.044085
-64.430650	40.956610	281.769600	.457487
-63.116020	40.914060	281.476300	.657426
-61.804690	40.856610	281.765800	.398923
-60.497620	40.784390	281.900700	.228905
-59.195770	40.697540	281.945100	.156724
-57.900040	40.596260	281.951000	.115114
-56.611330	40.480750	281.954500	.076290
-55.330490	40.351270	281.942300	.037837
-54.058330	40.208070	281.931700	.013472
-52.795620	40.051450	281.927000	.003902
-51.543080	39.881710	281.925600	.000688
-50.301390	39.699200	281.925400	.000121
-49.071130	39.504250	281.925300	.000076
-47.852850	39.297230	281.925300	.000066
-46.647030	39.078520	281.925400	.000106
-45.454070	38.848490	281.925400	.000099
-44.274280	38.607530	281.925400	.000099
-43.107920	38.356030	281.925500	.000183
-91.451740	39.437030	276.519400	5.918370
-90.263630	39.689750	272.877000	8.787893
-89.061680	39.931370	271.886900	10.901440
-87.846150	40.161480	303.936000	24.422790
-86.617400	40.379670	389.170600	76.824300
-85.375900	40.585540	446.618900	102.237700
-84.122160	40.778730	455.977400	102.765600
-82.856800	40.958850	417.335700	80.952320
-81.580460	41.125550	361.793300	42.559520
-80.293880	41.278500	423.233000	74.574570
-78.997860	41.417370	504.327500	119.367900
-77.693230	41.541890	460.125600	112.901900
-76.380870	41.651780	228.302900	55.733420
-75.061690	41.746810	116.712000	68.379820
-73.736660	41.826770	57.511040	45.468630
-72.406780	41.891490	86.277280	63.569760
-71.073040	41.940830	205.893800	34.608350
-69.736500	41.974670	279.761300	27.974080
-68.398190	41.992950	299.081800	24.346350
-67.059160	41.995620	293.447900	13.610770
-65.720480	41.982670	285.155900	4.579808
-64.383220	41.954140	280.056500	2.119476
-63.048400	41.910080	280.392000	2.375968
-61.717090	41.850600	281.629700	.971301
-60.390290	41.775820	282.041300	.618510
-59.069010	41.685920	282.091400	.533222
-57.754210	41.581080	282.071900	.464464
-56.446830	41.461530	282.066600	.287759
-55.147770	41.327530	281.999500	.139502
-53.857880	41.179360	281.949000	.042888
-52.577970	41.017320	281.930300	.008990
-51.308780	40.841740	281.925900	.001459
-50.051000	40.652980	281.925200	.000656
-48.805250	40.451390	281.925200	.000347
-47.572090	40.237360	281.925300	.000144
-46.352010	40.011290	281.925300	.000066
-45.145370	39.773560	281.925400	.000106
-43.952510	39.524580	281.925400	.000075
-42.773630	39.264750	281.925500	.000183
-91.793200	40.348750	271.227700	10.968500
-90.591710	40.609810	258.969000	20.128850

-89.375760	40.859430	234.433100	35.758260
-88.145600	41.097210	231.753400	38.651450
-86.901570	41.322720	259.025700	45.340260
-85.644150	41.535550	302.930500	50.226250
-84.373860	41.735290	360.680800	75.966610
-83.091290	41.921560	377.403000	122.468100
-81.797160	42.093980	285.168900	47.639300
-80.492230	42.252200	408.987300	64.084910
-79.177290	42.395880	541.479900	127.039500
-77.853240	42.524730	516.201800	124.379200
-76.521000	42.638450	234.719900	113.018800
-75.181550	42.736820	117.671100	95.237440
-73.835900	42.819590	184.314700	38.052090
-72.485100	42.886590	310.655800	76.110050
-71.130210	42.937670	371.050400	88.402310
-69.772340	42.972710	335.995800	53.028560
-68.412590	42.991630	302.982900	26.565330
-67.052090	42.994400	284.522800	13.118200
-65.691960	42.980990	289.648700	8.313167
-64.333320	42.951450	281.298800	5.950605
-62.977290	42.905830	281.135500	3.245861
-61.624970	42.844260	282.742000	2.220420
-60.277440	42.766850	282.990100	2.041319
-58.935750	42.673790	282.830000	1.889936
-57.600940	42.565280	282.718400	1.487811
-56.273990	42.441570	282.580700	1.060275
-54.955840	42.302930	282.195200	.403845
-53.647390	42.149640	281.995200	.106834
-52.349470	41.982030	281.932400	.012211
-51.062890	41.800450	281.923500	.007365
-49.788320	41.605260	281.924100	.003581
-48.526440	41.396860	281.924800	.001502
-47.277780	41.175640	281.925200	.000481
-46.042850	40.942000	281.925300	.000152
-44.822010	40.696380	281.925300	.000074
-43.615560	40.439180	281.925400	.000075
-42.423690	40.170830	281.925400	.000125
-92.150770	41.257820	267.624200	15.951750
-90.935390	41.527410	251.150200	27.260590
-89.704910	41.785250	209.983400	48.720380
-88.459540	42.030900	165.485000	45.064990
-87.199620	42.263930	122.505600	47.448140
-85.925610	42.483890	117.910900	37.027880
-84.638040	42.690380	148.642500	76.000060
-83.337520	42.882970	72.104370	57.268100
-82.024790	43.061280	165.089400	80.187530
-80.700610	43.224930	510.327300	104.699900
-79.365870	43.373580	685.211400	179.101300
-78.021450	43.506890	677.403500	176.728300
-76.668370	43.624570	522.519800	113.190700
-75.307630	43.726360	421.940700	110.691100
-73.940300	43.812040	393.548200	71.044170
-72.567500	43.881390	426.583600	92.281270
-71.190370	43.934270	410.363800	90.496780
-69.810050	43.970550	370.928800	71.929930
-68.427750	43.990140	322.676000	40.496940
-67.044650	43.993000	298.733500	23.240650
-65.661930	43.979120	316.656800	30.109660
-64.280810	43.948540	302.518900	19.952670
-62.902460	43.901310	293.470500	12.179470
-61.528050	43.837570	289.899800	8.387173
-60.158730	43.757450	287.067400	6.356078
-58.795620	43.661140	285.783400	5.317560
-57.439790	43.548850	285.224000	4.640824
-56.092310	43.420850	284.433000	2.881744
-54.754150	43.277410	282.706300	.945651
-53.426270	43.118850	282.018400	.117569

-52.109530	42.945500	281.907700	.084533
-50.804760	42.757750	281.906000	.051038
-49.512690	42.555960	281.918300	.017443
-48.233980	42.340550	281.923400	.005188
-46.969190	42.111940	281.924800	.001528
-45.718810	41.870550	281.925200	.000397
-44.483210	41.616830	281.925300	.000109
-43.262650	41.351200	281.925400	.000082
-42.057300	41.074100	281.925400	.000097
-92.525320	42.164070	266.468700	18.416210
-91.295520	42.442420	247.275800	33.754710
-90.049940	42.708690	199.110100	63.425870
-88.788760	42.962440	152.784000	60.624000
-87.512310	43.203190	138.261800	48.423560
-86.221000	43.430500	102.955900	41.593570
-84.915410	43.643920	93.852370	40.965610
-83.596150	43.843030	106.205400	52.639400
-82.263960	44.027400	285.563000	68.020260
-80.919660	44.196660	697.539400	199.958900
-79.564150	44.350410	818.463000	221.962000
-78.198390	44.488330	764.906900	201.028900
-76.823410	44.610100	599.328900	127.923100
-75.440290	44.715440	421.982100	61.177030
-74.050180	44.804110	330.587800	70.935360
-72.654240	44.875890	344.106100	60.840130
-71.253690	44.930630	377.222400	67.555530
-69.849760	44.968180	375.320900	68.130440
-68.443720	44.988460	337.851200	49.306900
-67.036810	44.991420	324.622100	40.069810
-65.630330	44.977050	348.535400	51.978160
-64.225530	44.945390	339.220300	45.458600
-62.823690	44.896510	320.593600	31.980510
-61.426040	44.830540	307.132000	21.571270
-60.033810	44.747610	296.949300	14.413330
-58.648170	44.647940	292.935200	11.104480
-57.270290	44.531750	291.619800	8.771765
-55.901260	44.399320	288.044600	5.371184
-54.542130	44.250930	282.892100	.957808
-53.193900	44.086940	281.566100	.768398
-51.857480	43.907680	281.685000	.505459
-50.533710	43.713560	281.843200	.194753
-49.223370	43.504980	281.900200	.062231
-47.927120	43.282370	281.919400	.015897
-46.645540	43.046150	281.924100	.003940
-45.379100	42.796800	281.925000	.000961
-44.128160	42.534760	281.925200	.000223
-42.892960	42.260480	281.925300	.000069
-41.673610	41.974410	281.925400	.000097
-92.917770	43.067330	269.127400	16.338150
-91.673000	43.354680	255.130800	27.490140
-90.411740	43.629630	221.784300	47.781430
-89.134110	43.891690	187.471600	43.224930
-87.840450	44.140390	153.149700	44.213810
-86.531140	44.375260	95.881810	57.528150
-85.206750	44.595830	122.798100	45.891610
-83.867900	44.801650	265.082600	79.022430
-82.515380	44.992280	416.278100	102.636900
-81.150000	45.167310	597.167200	157.680600
-79.772730	45.326340	656.073700	177.767400
-78.384560	45.469020	648.282300	162.736900
-76.986600	45.595010	577.109300	121.190800
-75.579970	45.704020	464.020800	63.044970
-74.165890	45.795780	394.673800	48.369250
-72.745610	45.870080	410.279700	59.823600
-71.320400	45.926740	412.301000	78.224240
-69.891600	45.965610	397.223000	77.987140
-68.460530	45.986610	356.568600	58.752430

-67.028560	45.989670	350.429300	55.287490
-65.597030	45.974800	366.619300	64.456470
-64.167300	45.942020	364.263600	62.661180
-62.740720	45.891430	346.068700	51.069960
-61.318600	45.823140	325.111200	35.880790
-59.902260	45.737310	308.233300	23.437590
-58.492950	45.634170	299.791800	15.950490
-57.091900	45.513950	294.149500	10.677170
-55.700260	45.376930	287.916100	4.984757
-54.319140	45.223450	279.608400	3.409211
-52.949590	45.053840	279.404000	3.429547
-51.592580	44.868490	280.940300	1.607397
-50.248970	44.667810	281.671000	.535044
-48.919560	44.452230	281.867600	.149638
-47.605040	44.222190	281.912800	.037344
-46.305980	43.978160	281.923100	.008481
-45.022840	43.720610	281.924900	.001919
-43.755960	43.450020	281.925200	.000416
-42.505550	43.166850	281.925300	.000091
-41.271660	42.871580	281.925400	.000101
-93.329110	43.967430	272.481800	12.348160
-92.068780	44.264030	265.427100	16.341270
-90.791210	44.547890	252.490000	19.969400
-89.496510	44.818510	242.172900	29.125280
-88.184940	45.075390	201.310300	41.614090
-86.856880	45.318050	133.632900	72.602810
-85.512890	45.545990	92.243000	63.098920
-84.153590	45.758730	245.903300	44.306940
-82.779790	45.955820	373.418900	75.934720
-81.392350	46.136820	457.204400	99.832180
-79.992260	46.301310	475.687600	100.482300
-78.580580	46.448910	499.431400	94.340550
-77.158460	46.579270	514.304600	98.437220
-75.727110	46.692070	481.437700	90.135750
-74.287820	46.787040	462.952700	89.642700
-72.841890	46.863950	471.421800	97.738290
-71.390720	46.922590	460.311700	98.368270
-69.935700	46.962830	408.316300	79.190190
-68.478260	46.984570	373.054700	62.474190
-67.019850	46.987740	379.121600	66.843150
-65.561920	46.972340	388.039200	76.439270
-64.105920	46.938410	389.259200	76.197790
-62.653260	46.886040	363.309100	63.534390
-61.205390	46.815350	333.721900	45.267050
-59.763680	46.726520	311.788300	28.812990
-58.329470	46.619790	299.273300	17.759120
-56.904060	46.495390	291.459500	10.315770
-55.488670	46.353640	284.532400	4.883784
-54.084500	46.194880	276.536600	7.365925
-52.692630	46.019470	277.155000	6.199771
-51.314070	45.827840	280.141500	2.892722
-49.949730	45.620390	281.501900	.940526
-48.600430	45.397590	281.847300	.251278
-47.266860	45.159900	281.910400	.061313
-45.949600	44.907820	281.922700	.013862
-44.649100	44.641840	281.924900	.002980
-43.365670	44.362450	281.925200	.000621
-42.099450	44.070140	281.925200	.000128
-40.850440	43.765410	281.925400	.000111
-93.760410	44.864160	274.038700	10.481340
-92.483920	45.170280	268.782000	12.935780
-91.189420	45.463320	261.062000	12.567310
-89.876960	45.742750	244.917100	14.791500
-88.546760	46.008070	193.776600	28.057610
-87.199170	46.258760	114.312400	65.033390
-85.834720	46.494300	127.596200	65.066670
-84.454070	46.714200	308.601500	62.793750

-83.058010	46.917960	411.937700	77.130600
-81.647480	47.105130	440.471500	75.881780
-80.223460	47.275260	421.194400	57.946850
-78.787090	47.427960	394.754800	50.578690
-77.339580	47.562840	401.429800	62.621730
-75.882230	47.679570	425.845600	81.303530
-74.416370	47.777870	438.502300	94.314580
-72.943440	47.857470	453.307400	102.166200
-71.464880	47.918190	443.195800	90.279950
-69.982220	47.959850	378.300600	58.042960
-68.496960	47.982350	343.485600	38.559200
-67.010670	47.985630	379.624500	58.609410
-65.524890	47.969690	404.798300	75.976720
-64.041170	47.934570	413.316800	82.721920
-62.561040	47.880340	382.331900	70.878270
-61.086020	47.807170	347.887700	51.635760
-59.617590	47.715230	323.478700	34.575440
-58.157170	47.604770	306.175300	21.777250
-56.706150	47.476050	293.759300	12.673420
-55.265830	47.329400	286.236300	7.610376
-53.837470	47.165180	279.953900	7.512503
-52.422200	46.983780	278.467900	6.528467
-51.021090	46.785640	280.496300	3.141120
-49.635090	46.571200	281.606900	1.057303
-48.265020	46.340950	281.874300	.295128
-46.911610	46.095370	281.918300	.072154
-45.575410	45.834990	281.923800	.015794
-44.256860	45.560320	281.925000	.003326
-42.956210	45.271870	281.925200	.000664
-41.673580	44.970160	281.925200	.000132
-40.408870	44.655690	281.925300	.000053
-94.212860	45.757310	275.252000	9.448550
-92.919560	46.073230	271.816100	11.152140
-91.607470	46.375730	270.324500	9.155461
-90.276550	46.664250	264.901200	10.591530
-88.926960	46.938270	237.878600	19.528950
-87.559010	47.197250	204.696800	36.017330
-86.173240	47.440650	225.917400	28.609340
-84.770290	47.667940	322.142400	32.948940
-83.350960	47.878600	392.212500	64.880380
-81.916200	48.072160	417.509800	70.256070
-80.467090	48.248140	391.173600	47.971710
-79.004780	48.406110	331.609600	52.486740
-77.530590	48.545680	343.565200	38.779160
-76.045850	48.666500	370.938900	59.939610
-74.552020	48.768250	392.136000	76.585300
-73.050600	48.850660	400.905900	82.435360
-71.543170	48.913520	386.813200	69.535480
-70.031330	48.956650	334.976100	36.348230
-68.516710	48.979950	287.138400	43.015800
-67.000980	48.983350	331.800200	35.997810
-65.485790	48.966840	375.680100	62.233160
-63.972820	48.930470	403.164400	74.767620
-62.463700	48.874330	389.482800	67.643170
-60.960050	48.798580	365.121500	52.235880
-59.463460	48.703410	344.583200	38.937830
-57.975440	48.589080	322.214400	27.442550
-56.497490	48.455880	301.956700	17.799190
-55.030970	48.304140	292.013500	11.736930
-53.577210	48.134270	286.541900	8.933117
-52.137430	47.946660	282.290700	4.884250
-50.712720	47.741790	281.609800	2.117720
-49.304080	47.520120	281.774200	.707850
-47.912350	47.282170	281.880500	.194648
-46.538250	47.028450	281.912600	.048839
-45.182340	46.759510	281.922300	.011489
-43.845020	46.475880	281.924600	.002310

-42.526500	46.178100	281.925000	.000501
-41.226800	45.866700	281.925200	.000127
-39.945740	45.542200	281.925300	.000050
-94.687740	46.646660	276.774600	8.108077
-93.376940	46.972690	275.500700	9.461952
-92.046550	47.284930	279.916600	8.915761
-90.696440	47.582840	278.761700	13.122700
-89.326680	47.865840	259.627300	20.241210
-87.937550	48.133390	248.527000	29.139290
-86.529520	48.384910	275.290600	13.937410
-85.103260	48.619850	324.355800	30.619810
-83.659580	48.837660	375.594800	57.624660
-82.199460	49.037830	403.098300	66.227710
-80.724000	49.219880	371.230900	50.881130
-79.234450	49.383340	331.250300	33.192590
-77.732160	49.527770	334.406400	39.078530
-76.218590	49.652830	344.207600	50.447370
-74.695260	49.758160	355.519900	60.207200
-73.163800	49.843490	366.963300	67.278010
-71.625870	49.908580	359.132400	60.928620
-70.083210	49.953250	331.553600	38.713190
-68.537570	49.977380	303.920500	23.068810
-66.990740	49.980900	323.863600	33.956850
-65.444490	49.963800	342.820800	49.156670
-63.900620	49.926140	359.533900	55.704530
-62.360880	49.868010	356.172500	52.496490
-60.827010	49.789570	340.491300	42.502810
-59.300730	49.691040	332.391100	33.818150
-57.783640	49.572690	321.256900	26.153410
-56.277320	49.434830	304.171100	18.951710
-54.783270	49.277820	293.483300	12.444110
-53.302860	49.102080	286.941300	6.976754
-51.837360	48.908040	283.279900	3.325204
-50.387950	48.696200	282.097600	1.153740
-48.955640	48.467040	281.899500	.341925
-47.541310	48.221130	281.889200	.091775
-46.145660	47.959000	281.909600	.026642
-44.769230	47.681210	281.920500	.006934
-43.412380	47.388330	281.924100	.001938
-42.075270	47.080920	281.924900	.000478
-40.757810	46.759540	281.925100	.000153
-39.459720	46.424710	281.925300	.000063
-95.186440	47.531940	279.238500	6.317147
-93.857410	47.868410	280.422500	8.283497
-92.508000	48.190720	288.640000	10.649710
-91.137910	48.498310	288.344600	11.185640
-89.747150	48.790600	282.361500	8.814238
-88.335950	49.067010	295.115000	10.647410
-86.904710	49.326930	329.637500	38.383170
-85.454100	49.569790	348.144300	48.887020
-83.984930	49.795020	362.801200	55.758240
-82.498220	50.002060	357.848000	53.175510
-80.995120	50.190400	334.741100	41.726280
-79.476920	50.359550	321.691900	34.493080
-77.945060	50.509060	324.853200	38.210120
-76.401090	50.638520	331.537500	45.265190
-74.846650	50.747580	338.804100	50.849880
-73.283450	50.835950	348.964800	57.767190
-71.713320	50.903350	354.844600	60.036170
-70.138080	50.949630	340.627100	48.826590
-68.559640	50.974620	317.237600	35.766890
-66.979900	50.978270	324.331800	37.923150
-65.400800	50.960560	322.876800	38.375400
-63.824260	50.921550	319.768900	35.112170
-62.252170	50.861340	315.596800	30.007620
-60.686400	50.780110	308.091500	24.297510
-59.128770	50.678080	304.958300	20.364560

-57.581030	50.555550	303.267900	18.161230
-56.044860	50.412850	294.986600	12.971480
-54.521820	50.250360	288.501800	7.918160
-53.013420	50.068520	284.678600	4.115838
-51.520960	49.867810	282.755600	1.641803
-50.045680	49.648740	282.105400	.556118
-48.588630	49.411830	281.946800	.168803
-47.150700	49.157670	281.914300	.041835
-45.732590	48.886830	281.917500	.011472
-44.334810	48.599900	281.922600	.004193
-42.957660	48.297460	281.924500	.001247
-41.601190	47.980120	281.925000	.000371
-40.265250	47.648430	281.925100	.000140
-38.949390	47.302930	281.925300	.000054
-95.710510	48.412890	282.969500	5.546541
-94.362470	48.760130	286.440900	9.237575
-92.993230	49.092850	294.004400	12.149400
-91.602360	49.410460	293.009200	11.259910
-90.189750	49.712350	293.495700	10.614180
-88.755540	49.997940	306.089000	21.900270
-87.300090	50.266570	324.149100	37.332730
-85.824040	50.517650	332.754200	45.277790
-84.328210	50.750570	336.794600	46.893140
-82.813610	50.964740	325.744900	40.087310
-81.281460	51.159630	313.514000	32.301510
-79.733130	51.334700	314.690800	31.103240
-78.170130	51.489480	319.725600	37.066830
-76.594090	51.623530	323.812100	40.910940
-75.006800	51.736490	328.091800	44.038030
-73.410070	51.828010	333.134900	48.607290
-71.805870	51.897850	339.589900	52.973770
-70.196170	51.945790	343.372400	52.778800
-68.582990	51.971680	329.794900	45.335340
-66.968440	51.975460	327.515400	42.034850
-65.354560	51.957120	314.647100	32.416190
-63.743440	51.916690	302.727900	22.413510
-62.137130	51.854320	296.015100	15.448960
-60.537630	51.770170	291.737200	11.285420
-58.946900	51.664510	291.610300	10.429230
-57.366820	51.537620	290.486500	8.974156
-55.799180	51.389870	287.265400	6.476225
-54.245650	51.221680	284.551300	3.673085
-52.707820	51.033510	283.002900	1.746312
-51.187080	50.825860	282.283400	.712244
-49.684720	50.599280	282.023900	.253633
-48.201800	50.354330	281.943900	.073176
-46.739240	50.091630	281.924100	.014866
-45.297720	49.811770	281.923000	.003927
-43.877700	49.515370	281.924300	.001565
-42.479410	49.203060	281.924900	.000619
-41.102810	48.875440	281.925100	.000227
-39.747590	48.533100	281.925200	.000090
-38.413150	48.176570	281.925400	.000093
-96.261670	49.289190	287.012900	6.970073
-94.893750	49.647600	291.924900	10.402270
-93.503820	49.991080	295.034400	11.744550
-92.091320	50.319060	293.521600	10.626090
-90.655950	50.630910	292.625600	10.839950
-89.197760	50.925990	296.201000	15.397050
-87.717060	51.203660	302.457300	22.681930
-86.214420	51.463270	308.298000	28.466330
-84.690660	51.704190	315.971500	33.372170
-83.146820	51.925780	316.310300	32.447480
-81.584160	52.127480	312.861900	29.379120
-80.004110	52.308710	315.799700	32.280610
-78.408280	52.468990	315.908300	33.579030
-76.798410	52.607840	316.555900	35.428880

-75.176390	52.724850	319.866600	37.628310
-73.544200	52.819690	323.806400	40.334550
-71.903920	52.892050	326.705900	45.135670
-70.257710	52.941730	338.077600	50.926610
-68.607750	52.968570	342.145100	51.476210
-66.956280	52.972490	333.287900	45.612900
-65.305560	52.953480	311.982700	30.442130
-63.657820	52.911580	295.756600	16.321120
-62.015270	52.846950	288.557500	8.571840
-60.380080	52.759750	285.726300	5.256397
-58.754360	52.650280	285.322400	4.284362
-57.140120	52.518850	284.773300	3.736844
-55.539290	52.365840	283.651500	2.450199
-53.953660	52.191710	282.782100	1.357644
-52.384890	51.996940	282.288300	.653502
-50.834480	51.782070	282.055500	.271492
-49.303730	51.547700	281.963500	.093916
-47.793770	51.294400	281.933600	.025908
-46.305490	51.022820	281.925800	.004993
-44.839570	50.733620	281.924800	.001010
-43.396390	50.427420	281.924900	.000522
-41.976090	50.104880	281.925100	.000248
-40.578500	49.766630	281.925100	.000134
-39.203150	49.413260	281.925300	.000063
-37.849210	49.045330	281.925400	.000078
-96.841810	50.160530	287.349700	5.851539
-95.453030	50.530490	290.592500	7.844524
-94.041510	50.885130	290.229700	7.867646
-92.606450	51.223850	288.179000	6.753267
-91.147380	51.546020	287.082600	6.288529
-89.664200	51.850960	287.619800	7.455637
-88.157130	52.138020	289.589200	10.170300
-86.626690	52.406500	293.369100	14.383900
-85.073680	52.655720	300.461200	20.147350
-83.499180	52.885050	306.589400	23.842400
-81.904440	53.093850	303.978600	22.939310
-80.290990	53.281520	301.431700	21.497240
-78.660530	53.447530	300.046800	21.224500
-77.014920	53.591380	303.926600	24.499340
-75.356160	53.712650	312.407300	30.409880
-73.686420	53.810940	320.079800	34.419470
-72.007930	53.885960	320.685000	37.584370
-70.322990	53.937460	328.181800	43.824600
-68.634010	53.965280	341.542400	50.281770
-66.943400	53.969350	336.817300	45.294030
-65.253590	53.949630	310.557600	28.053100
-63.567000	53.906200	292.856500	13.094820
-61.886040	53.839190	285.805300	5.457815
-60.213040	53.748820	283.551700	2.520180
-58.550290	53.635360	283.040300	1.701125
-56.899960	53.499180	282.737700	1.240535
-55.264110	53.340690	282.414200	.795801
-53.644650	53.160350	282.176600	.449304
-52.043340	52.958710	282.034300	.214414
-50.461760	52.736320	281.965000	.086245
-48.901280	52.493820	281.937100	.029082
-47.363030	52.231850	281.928000	.008172
-45.847900	51.951060	281.925600	.001904
-44.356530	51.652150	281.925100	.000390
-42.889220	51.335790	281.925100	.000173
-41.445980	51.002650	281.925100	.000130
-40.026490	50.653380	281.925200	.000084
-38.630060	50.288590	281.925300	.000054
-37.255620	49.908830	281.925400	.000075
-97.453060	51.026530	284.730400	3.244833
-96.042340	51.408490	285.651600	3.909537
-94.608220	51.774700	285.194500	3.643369

-93.149600	52.124570	284.263000	2.972935
-91.665800	52.457450	283.739200	2.600145
-90.156560	52.772640	283.793700	2.885995
-88.621980	53.069450	284.415500	3.873988
-87.062480	53.347160	286.043500	6.006740
-85.478850	53.605050	289.339900	9.433183
-83.872130	53.842430	292.628300	12.200740
-82.243660	54.058630	291.977900	12.230900
-80.595020	54.253030	290.014600	10.751880
-78.928000	54.425040	288.672700	9.743192
-77.244600	54.574130	292.919300	13.745950
-75.546970	54.699840	301.613200	21.050570
-73.837420	54.801750	309.121800	25.679260
-72.118360	54.879540	309.204000	26.756440
-70.392330	54.932960	311.572300	30.424620
-68.661900	54.961820	320.406700	35.508030
-66.929700	54.966030	323.206400	35.223650
-65.198370	54.945580	302.876300	20.946570
-63.470540	54.900540	289.508300	9.379555
-61.748820	54.831050	284.295200	3.499223
-60.035740	54.737340	282.707000	1.310021
-58.333780	54.619720	282.293400	.638491
-56.645260	54.478570	282.144700	.385580
-54.972420	54.314330	282.055000	.241112
-53.317280	54.127510	281.991800	.131296
-51.681740	53.918690	281.954000	.060626
-50.067420	53.688470	281.935700	.023956
-48.475770	53.437500	281.928500	.008377
-46.907930	53.166490	281.926000	.002636
-45.364770	52.876130	281.925300	.000692
-43.846840	52.567130	281.925200	.000157
-42.354370	52.240220	281.925200	.000095
-40.887210	51.896080	281.925200	.000084
-39.444820	51.535370	281.925300	.000060
-38.026250	51.158730	281.925300	.000045
-36.630120	50.766700	281.925400	.000099
-98.097820	51.886770	282.921100	1.318260
-96.663910	52.281200	283.073400	1.410606
-95.206040	52.659450	282.906800	1.280214
-93.722790	53.020900	282.620900	1.022389
-92.213200	53.364910	282.463700	.883291
-90.676770	53.690770	282.477600	.966107
-89.113440	53.997730	282.688800	1.338880
-87.523560	54.285050	283.218600	2.125545
-85.907830	54.551970	284.333300	3.565070
-84.267300	54.797760	285.710300	5.162933
-82.603340	55.021710	286.049600	5.820643
-80.917590	55.223140	284.893500	4.465866
-79.211960	55.401440	283.917000	3.692998
-77.488560	55.556020	287.596700	7.497207
-75.749730	55.686380	294.938500	14.382020
-73.997920	55.792100	301.387200	19.066110
-72.235800	55.872810	302.073900	19.005170
-70.466090	55.928230	298.485700	17.939900
-68.691570	55.958180	299.237300	19.062030
-66.915130	55.962550	299.468400	17.784410
-65.139640	55.941330	291.421300	11.230410
-63.367970	55.894590	285.482200	4.928687
-61.602940	55.822490	283.070000	1.847432
-59.847310	55.725280	282.283900	.645897
-58.103760	55.603290	282.053700	.245881
-56.374820	55.456930	281.985900	.121673
-54.662870	55.286670	281.957600	.067238
-52.970100	55.093080	281.941100	.034486
-51.298500	54.876750	281.932000	.015490
-49.649800	54.638340	281.927800	.006367
-48.025460	54.378550	281.926000	.002360

-46.426660	54.098120	281.925400	.000757
-44.854210	53.797780	281.925200	.000207
-43.308580	53.478290	281.925200	.000089
-41.789850	53.140400	281.925200	.000066
-40.297680	52.784830	281.925300	.000060
-38.831280	52.412250	281.925300	.000054
-37.389370	52.023280	281.925400	.000080
-35.970190	51.618480	281.925500	.000185
-98.778840	52.740810	282.217800	.435489
-97.320270	53.148240	282.225800	.426997
-95.837370	53.539010	282.179800	.380858
-94.328290	53.912530	282.111900	.309850
-92.791720	54.268120	282.072200	.267095
-91.226900	54.605080	282.075800	.285133
-89.633560	54.922620	282.146600	.410988
-88.011890	55.219970	282.331400	.712721
-86.362530	55.496330	282.719400	1.289370
-84.686490	55.750910	283.425400	2.316026
-82.985150	55.982970	283.984900	3.114481
-81.260250	56.191770	283.109200	1.897680
-79.513790	56.376650	282.376700	1.416855
-77.748020	56.536990	285.518200	4.712915
-75.965460	56.672260	291.611800	10.817290
-74.168790	56.781970	296.939800	15.234530
-72.360860	56.865740	299.650500	15.897010
-70.544630	56.923280	293.982000	12.628960
-68.723180	56.954370	289.922100	9.517637
-66.899620	56.958910	288.182800	7.673531
-65.077100	56.936880	285.252100	4.554569
-63.258730	56.888360	283.264200	2.098080
-61.447630	56.813510	282.392600	.818873
-59.646790	56.712620	282.069900	.274779
-57.859090	56.586040	281.970200	.093106
-56.087300	56.434210	281.942300	.038006
-54.333970	56.257640	281.933000	.018127
-52.601470	56.056940	281.928800	.008504
-50.891880	55.832740	281.926800	.003852
-49.207030	55.585770	281.925800	.001609
-47.548420	55.316760	281.925400	.000593
-45.917200	55.026500	281.925300	.000176
-44.314130	54.715760	281.925200	.000089
-42.739580	54.385350	281.925200	.000064
-41.193420	54.036030	281.925300	.000060
-39.675070	53.668560	281.925300	.000057
-38.183400	53.283620	281.925300	.000045
-36.716770	52.881820	281.925400	.000078
-35.272900	52.463700	281.925500	.000183
-99.499240	53.588130	282.005100	.127003
-98.014280	54.009130	281.997700	.113892
-96.504860	54.412960	281.986800	.101827
-94.968610	54.799050	281.971800	.084451
-93.403790	55.166730	281.962100	.072858
-91.809290	55.515250	281.965000	.078889
-90.184560	55.843850	281.992800	.125869
-88.529620	56.151670	282.074100	.256259
-86.844990	56.437890	282.286300	.592903
-85.131650	56.701690	282.795900	1.398709
-83.390960	56.942230	283.334900	2.184870
-81.624720	57.158780	282.406100	.775144
-79.835050	57.350580	281.897200	.884514
-78.024370	57.516980	284.572800	3.391751
-76.195360	57.657400	289.548900	8.401624
-74.350940	57.771310	293.675300	12.162670
-72.494220	57.858320	297.971500	14.525630
-70.628410	57.918090	294.238200	11.918650
-68.756890	57.950390	288.127200	7.073285
-66.883060	57.955100	284.895000	3.835299

-65.010370 57.932220 283.235400 1.916673
-63.142240 57.881810 282.443700 .845961
-61.282040 57.804080 282.102800 .315994
-59.433060 57.699310 281.978500 .104141
-57.598450 57.567890 281.940600 .033910
-55.781200 57.410300 281.930200 .012352
-53.984060 57.227100 281.927200 .004898
-52.209560 57.018940 281.926100 .002109
-50.459930 56.786500 281.925700 .000901
-48.737060 56.530560 281.925400 .000348
-47.042490 56.251900 281.925300 .000127
-45.377330 55.951360 281.925200 .000070
-43.742270 55.629770 281.925200 .000087
-42.137470 55.287960 281.925200 .000061
-40.562610 54.926740 281.925300 .000058
-39.016750 54.546860 281.925300 .000049
-37.498410 54.149020 281.925400 .000080
-36.005400 53.733830 281.925400 .000075
-34.534880 53.301770 281.925500 .000183
-100.262700 54.428120 281.945000 .033101
-98.749250 54.863350 281.941800 .028239
-97.211560 55.280850 281.939000 .024877
-95.646590 55.680060 281.935900 .020925
-94.052090 56.060360 281.934300 .019113
-92.426510 56.420970 281.937600 .024877
-90.768940 56.761100 281.954200 .051060
-89.079190 57.079890 282.005300 .129504
-87.357570 57.376450 282.166100 .386283
-85.605000 57.649890 282.611100 1.118547
-83.822840 57.899360 283.041100 1.720897
-82.012920 58.124030 281.985400 .188685
-80.177480 58.323120 281.598700 .891943
-78.319110 58.495910 283.524400 1.948042
-76.440710 58.641750 286.967900 5.464427
-74.545430 58.760110 289.774500 8.398049
-72.636660 58.850530 295.456800 12.739090
-70.717930 58.912650 295.836100 12.767110
-68.792920 58.946230 289.119500 7.837604
-66.865380 58.951130 284.630000 3.449376
-64.939080 58.927340 282.837700 1.301394
-63.017780 58.874950 282.218800 .451165
-61.105210 58.794160 282.012300 .144241
-59.204930 58.685300 281.949000 .044699
-57.320400 58.548780 281.931500 .013971
-55.454840 58.385130 281.926900 .004373
-53.611270 58.194950 281.925900 .001423
-51.792370 57.978940 281.925500 .000492
-50.000500 57.737840 281.925400 .000181
-48.237630 57.472490 281.925400 .000105
-46.505310 57.183730 281.925300 .000043
-44.804610 56.872430 281.925200 .000058
-43.136050 56.539500 281.925200 .000060
-41.499610 56.185780 281.925300 .000058
-39.894630 55.812120 281.925300 .000049
-38.319800 55.419290 281.925400 .000080
-36.773090 55.007980 281.925400 .000075
-35.251730 54.578750 281.925400 .000099
-33.752120 54.132060 281.925500 .000183
-101.073700 55.260130 281.929800 .008143
-99.529010 55.710320 281.928800 .006635
-97.960900 56.142140 281.928100 .005758
-96.365430 56.555100 281.927600 .005091
-94.739640 56.948590 281.928000 .005766
-93.081440 57.321860 281.931300 .011261
-91.389490 57.674070 281.944700 .032286
-89.663250 58.004340 281.986800 .095521
-87.902820 58.311730 282.129500 .325099

-86.109000 58.595320 282.534400 .995314
-84.283100 58.854170 282.849400 1.417207
-82.427000 59.087400 281.621700 .512011
-80.543050 59.294160 281.220200 1.209358
-78.634010 59.473690 281.976200 .501832
-76.703000 59.625280 282.819500 1.864049
-74.753460 59.748340 284.707000 4.884212
-72.789070 59.842370 291.396500 9.935201
-70.813740 59.906970 296.677700 13.186840
-68.831480 59.941910 290.902200 9.377866
-66.846440 59.947010 285.380300 4.281637
-64.862770 59.922250 282.989400 1.514769
-62.884610 59.867760 282.224800 .461336
-60.916040 59.783740 282.003700 .127073
-58.961010 59.670550 281.944200 .033543
-57.023270 59.528640 281.929500 .008756
-55.106350 59.358580 281.926100 .002207
-53.213500 59.161040 281.925500 .000533
-51.347600 58.936760 281.925400 .000149
-49.511150 58.686560 281.925300 .000055
-47.706190 58.411320 281.925300 .000043
-45.934240 58.111950 281.925300 .000041
-44.196280 57.789400 281.925300 .000047
-42.492650 57.444580 281.925300 .000056
-40.823030 57.078400 281.925300 .000049
-39.186360 56.691730 281.925400 .000080
-37.580830 56.285340 281.925400 .000075
-36.003750 55.859910 281.925400 .000073
-34.451600 55.415950 281.925500 .000185
-32.919830 54.953830 281.925500 .000183
-101.937200 56.083360 281.926300 .001937
-100.358100 56.549350 281.926000 .001506
-98.756940 56.996240 281.925900 .001325
-97.128790 57.423640 281.925900 .001415
-95.469860 57.830970 281.926500 .002564
-93.777320 58.217490 281.929700 .007610
-92.049300 58.582370 281.941500 .026160
-90.284800 58.924680 281.979100 .082030
-88.483640 59.243460 282.112100 .298568
-86.646400 59.537720 282.487100 .921451
-84.774360 59.806450 282.704000 1.172563
-82.869400 60.048700 281.402200 .836577
-80.933970 60.263580 281.160500 1.280517
-78.971000 60.450230 281.333500 1.099976
-76.983890 60.607900 280.970400 2.212617
-74.976370 60.735940 280.899300 5.283988
-72.952470 60.833800 286.370200 7.747787
-70.916480 60.901050 294.506600 11.890120
-68.872850 60.937420 292.326200 10.481220
-66.826130 60.942730 286.492900 5.412755
-64.780930 60.916960 283.356600 1.996735
-62.741830 60.860230 282.313100 .603889
-60.713310 60.772790 282.022500 .160169
-58.699720 60.654990 281.947600 .038762
-56.705200 60.507380 281.929900 .009134
-54.733590 60.330550 281.926100 .002049
-52.788400 60.125220 281.925400 .000416
-50.872730 59.892220 281.925400 .000109
-48.989200 59.632420 281.925400 .000078
-47.139900 59.346780 281.925300 .000039
-45.326320 59.036270 281.925300 .000056
-43.549270 58.701890 281.925300 .000041
-41.808860 58.344600 281.925300 .000046
-40.104370 57.965370 281.925300 .000056
-38.434220 57.565060 281.925400 .000075
-36.795950 57.144450 281.925400 .000073
-35.186060 56.704150 281.925400 .000099

-33.600030 56.244660 281.925500 .000183
-32.032190 55.766200 281.925500 .000183
-102.859900 56.896880 281.925500 .000436
-101.242000 57.379640 281.925500 .000345
-99.604390 57.842470 281.925500 .000347
-97.940930 58.285070 281.925600 .000581
-96.246660 58.706940 281.926200 .001686
-94.517820 59.107400 281.929000 .006092
-92.751850 59.485580 281.939200 .022114
-90.947170 59.840560 281.971800 .070295
-89.103220 60.171320 282.090500 .266812
-87.220290 60.476800 282.415600 .797023
-85.299550 60.755960 282.566200 .971602
-83.342830 61.007760 281.321200 .980749
-81.352710 61.231220 281.308000 1.045361
-79.332320 61.425420 281.480600 .838562
-77.285290 61.589540 281.116700 1.905066
-75.215710 61.722870 280.764600 3.845136
-73.128000 61.824790 282.846500 6.936421
-71.026890 61.894870 289.931000 9.757886
-68.917300 61.932760 292.753100 10.663440
-66.804310 61.938290 287.371500 6.282787
-64.692990 61.911450 283.711500 2.436787
-62.588420 61.852340 282.400800 .743715
-60.495590 61.761240 282.041400 .195925
-58.419300 61.638580 281.951600 .046608
-56.364090 61.484900 281.930700 .010453
-54.334160 61.300890 281.926200 .002284
-52.333320 61.087320 281.925400 .000458
-50.364880 60.845090 281.925400 .000119
-48.431600 60.575160 281.925400 .000099
-46.535590 60.278550 281.925400 .000078
-44.678250 59.956310 281.925400 .000078
-42.860180 59.609490 281.925300 .000056
-41.081100 59.239120 281.925300 .000039
-39.339830 58.846180 281.925400 .000078
-37.634110 58.431540 281.925400 .000073
-35.960610 57.995940 281.925400 .000099
-34.314830 57.539980 281.925500 .000185
-32.690990 57.064010 281.925500 .000183
-31.082000 56.568130 281.925500 .000183
-103.849600 57.699570 281.925500 .000207
-102.187300 58.200280 281.925500 .000208
-100.509000 58.680030 281.925500 .000193
-98.806840 59.138710 281.925500 .000354
-97.074520 59.575940 281.926000 .001270
-95.307120 59.991050 281.928200 .004796
-93.501110 60.383240 281.936400 .017572
-91.654170 60.751550 281.962100 .055627
-89.765210 61.094930 282.053600 .204890
-87.834170 61.412270 282.310100 .635925
-85.861950 61.702450 282.405000 .713078
-83.850420 61.964360 281.407000 .816333
-81.802150 62.196920 281.471500 .795140
-79.720490 62.399150 281.676800 .490092
-77.609360 62.570130 281.596000 .928190
-75.473210 62.709080 281.361600 2.414772
-73.316960 62.815350 282.172600 4.378928
-71.145790 62.888430 285.811600 8.005518
-68.965200 62.927950 290.968800 9.352059
-66.780790 62.933720 287.002500 5.965436
-64.598260 62.905720 283.672000 2.407525
-62.423240 62.844070 282.395800 .752876
-60.261290 62.749080 282.039200 .198338
-58.117710 62.621230 281.950900 .046967
-55.997520 62.461110 281.930500 .010514
-53.905320 62.269450 281.926200 .002251

-51.845240 62.047150 281.925400 .000457
-49.820820 61.795150 281.925400 .000106
-47.834930 61.514500 281.925400 .000127
-45.889670 61.206310 281.925400 .000073
-43.986290 60.871700 281.925400 .000073
-42.125070 60.511780 281.925400 .000078
-40.305270 60.127630 281.925400 .000078
-38.525020 59.720250 281.925400 .000073
-36.781200 59.290500 281.925400 .000073
-35.069400 58.839090 281.925500 .000185
-33.383770 58.366500 281.925500 .000183
-31.716990 57.872960 281.925500 .000183
-30.060130 57.358350 281.925500 .000183
-104.916500 58.490070 281.925500 .000180
-103.202300 59.010120 281.925500 .000181
-101.477600 59.507980 281.925500 .000181
-99.732440 59.983750 281.925500 .000260
-97.958720 60.437220 281.925800 .000902
-96.150010 60.867820 281.927300 .003451
-94.301540 61.274790 281.933200 .012524
-92.410060 61.657170 281.951300 .039443
-90.473700 62.013880 282.014200 .141689
-88.491920 62.343760 282.187100 .430269
-86.465330 62.645610 282.250600 .491510
-84.395630 62.918240 281.555200 .584363
-82.285480 63.160470 281.627800 .508528
-80.138370 63.371240 281.787600 .280768
-77.958560 63.549540 281.816400 .421372
-75.750930 63.694510 281.821100 1.021436
-73.520860 63.805420 282.158800 2.443405
-71.274150 63.881700 283.955400 4.371356
-69.016910 63.922960 286.754700 6.004764
-66.755400 63.928990 284.990000 3.944932
-64.495980 63.899750 283.054000 1.697832
-62.244980 63.835380 282.249500 .548165
-60.008560 63.736250 282.007400 .149917
-57.792640 63.602840 281.944200 .036161
-55.602750 63.435840 281.929200 .008170
-53.443960 63.236060 281.926100 .001812
-51.320750 63.004450 281.925500 .000361
-49.236890 62.742080 281.925400 .000102
-47.195330 62.450080 281.925400 .000127
-45.198120 62.129630 281.925400 .000099
-43.246250 61.781950 281.925400 .000073
-41.339580 61.408210 281.925400 .000073
-39.476710 61.009530 281.925400 .000075
-37.654880 60.586900 281.925400 .000073
-35.869840 60.141170 281.925500 .000185
-34.115770 59.672970 281.925500 .000183
-32.385130 59.182660 281.925500 .000183
-30.668580 58.670220 281.925500 .000183
-28.954870 58.135290 281.925500 .000183
-106.073800 59.266680 281.925500 .000183
-104.297500 59.807790 281.925500 .000183
-102.518800 60.325180 281.925500 .000176
-100.725000 60.819250 281.925500 .000215
-98.905530 61.289990 281.925600 .000604
-97.052100 61.737010 281.926600 .002203
-95.158340 62.159620 281.930200 .007773
-93.219700 62.556880 281.941100 .024164
-91.233350 62.927700 281.976900 .082203
-89.198040 63.270870 282.069500 .232702
-87.113920 63.585110 282.105100 .278749
-84.982480 63.869130 281.709900 .342277
-82.806390 64.121680 281.760100 .274332
-80.589290 64.341560 281.855000 .137149
-78.335770 64.527670 281.887200 .180125

-76.051160 64.679080 281.926900 .407731
-73.741460 64.794950 282.096300 .959428
-71.413090 64.874680 282.691900 1.940074
-69.072890 64.917820 283.682500 2.634684
-66.727910 64.924110 283.100500 1.846746
-64.385280 64.893550 282.403700 .823651
-62.052090 64.826280 282.077300 .283863
-59.735260 64.722690 281.966900 .081192
-57.441380 64.583340 281.935400 .020458
-55.176610 64.408980 281.927500 .004960
-52.946510 64.200520 281.925800 .001122
-50.755950 63.959000 281.925500 .000274
-48.608900 63.685590 281.925500 .000182
-46.508400 63.381530 281.925500 .000185
-44.456330 63.048090 281.925500 .000183
-42.453320 62.686580 281.925400 .000127
-40.498620 62.298210 281.925400 .000073
-38.589950 61.884150 281.925400 .000073
-36.723370 61.445380 281.925500 .000183
-34.893150 60.982680 281.925500 .000183
-33.091610 60.496560 281.925500 .000183
-31.309040 59.987160 281.925500 .000183
-29.533510 59.454220 281.925500 .000183
-27.750760 58.896990 281.925500 .000183
-107.339200 60.027230 281.925500 .000183
-105.487000 60.591610 281.925500 .000183
-103.643800 61.130270 281.925500 .000178
-101.793500 61.644070 281.925500 .000206
-99.922550 62.133320 281.925500 .000392
-98.020040 62.597820 281.926100 .001298
-96.077510 63.037030 281.928000 .004267
-94.088710 63.450070 281.933300 .012641
-92.049460 63.835860 281.946300 .031196
-89.957580 64.193130 281.981500 .096858
-87.812570 64.520550 281.988900 .094894
-85.615560 64.816710 281.838000 .134088
-83.369120 65.080260 281.856800 .118723
-81.077050 65.309890 281.897300 .049607
-78.744270 65.504390 281.911800 .066147
-76.376630 65.662700 281.932000 .152047
-73.980770 65.783920 281.998400 .337331
-71.563890 65.867360 282.167900 .661994
-69.133670 65.912500 282.410500 .911296
-66.698060 65.919100 282.276000 .657153
-64.265090 65.887100 282.085500 .314527
-61.842790 65.816700 281.982300 .116253
-59.438900 65.708320 281.942400 .035281
-57.060850 65.562580 281.929800 .009858
-54.715470 65.380330 281.926400 .002584
-52.408880 65.162570 281.925600 .000638
-50.146370 64.910470 281.925500 .000201
-47.932120 64.625290 281.925500 .000184
-45.769150 64.308400 281.925500 .000183
-43.659080 63.961160 281.925500 .000183
-41.602020 63.584970 281.925500 .000183
-39.596350 63.181100 281.925500 .000183
-37.638600 62.750720 281.925500 .000183
-35.723270 62.294770 281.925500 .000183
-33.842640 61.813940 281.925500 .000183
-31.986660 61.308540 281.925500 .000183
-30.142740 60.778430 281.925500 .000183
-28.295590 60.222970 281.925500 .000183
-26.427080 59.640870 281.925500 .000183
-108.736700 60.768890 281.925500 .000183
-106.789100 61.359350 281.925500 .000183
-104.866900 61.921510 281.925500 .000178
-102.949500 62.456830 281.925500 .000201

-101.019200 62.966030 281.925500 .000330
-99.061880 63.449270 281.925900 .000960
-97.066180 63.906190 281.927000 .002820
-95.023590 64.336020 281.930100 .007704
-92.928180 64.737730 281.935900 .016372
-90.776390 65.110020 281.943800 .027248
-88.566850 65.451470 281.946800 .041271
-86.300130 65.760610 281.890700 .062966
-83.978550 66.035940 281.899700 .039420
-81.606060 66.276030 281.911400 .024479
-79.187900 66.479550 281.919500 .030303
-76.730480 66.645290 281.930000 .066565
-74.241170 66.772270 281.958300 .147325
-71.728080 66.859700 282.016100 .262017
-69.199870 66.907030 282.080400 .349023
-66.665550 66.913940 282.048300 .263745
-64.134220 66.880400 281.989800 .139895
-61.614990 66.806620 281.951200 .055723
-59.116610 66.693070 281.934100 .019158
-56.647430 66.540450 281.927900 .006088
-54.215080 66.349720 281.926000 .001792
-51.826330 66.121990 281.925500 .000480
-49.486860 65.858530 281.925500 .000186
-47.201050 65.560780 281.925500 .000180
-44.971820 65.230190 281.925500 .000183
-42.800360 64.868270 281.925500 .000183
-40.685980 64.476460 281.925500 .000183
-38.625890 64.056100 281.925500 .000183
-36.614990 63.608330 281.925500 .000183
-34.645670 63.134020 281.925500 .000183
-32.707610 62.633670 281.925500 .000183
-30.787590 62.107330 281.925500 .000183
-28.869240 61.554470 281.925500 .000183
-26.932890 60.973880 281.925500 .000183
-24.955330 60.363580 281.925500 .000183

Q.13a
-89.453400 32.991660 297.217200 .812710
-88.345930 33.191160 297.255000 .904465
-87.228100 33.381660 297.395600 1.256811
-86.100230 33.562890 297.599900 1.758261
-84.962690 33.734530 297.815800 2.280138
-83.815900 33.896310 297.992100 2.696547
-82.660300 34.047950 298.095900 2.933928
-81.496410 34.189190 298.119000 2.958788

-80.324730	34.319790	298.061300	2.750346
-79.145840	34.439500	297.911400	2.252448
-77.960310	34.548100	297.582600	1.485706
-76.768760	34.645400	297.047500	1.105958
-75.571840	34.731200	296.388400	1.428095
-74.370180	34.805370	296.150700	1.179316
-73.164480	34.867740	296.328900	.852513
-71.955430	34.918200	296.679500	.487195
-70.743730	34.956650	296.983600	.470471
-69.530080	34.983020	297.131500	.625541
-68.315220	34.997260	297.159500	.602392
-67.099880	34.999340	297.128700	.479474
-65.884770	34.989250	297.078800	.321155
-64.670640	34.967020	297.036600	.183324
-63.458190	34.932690	297.010800	.093636
-62.248150	34.886320	296.998400	.045135
-61.041230	34.828000	296.993400	.023034
-59.838120	34.757850	296.991800	.014205
-58.639500	34.676010	296.991500	.011044
-57.446040	34.582610	296.991100	.008788
-56.258390	34.477850	296.990300	.006141
-55.077160	34.361910	296.989500	.003383
-53.902950	34.235010	296.989000	.001588
-52.736340	34.097380	296.988700	.000614
-51.577860	33.949260	296.988600	.000209
-50.428010	33.790910	296.988600	.000158
-49.287250	33.622600	296.988500	.000183
-48.156040	33.444620	296.988500	.000183
-47.034740	33.257250	296.988500	.000183
-45.923690	33.060790	296.988500	.000183
-44.823220	32.855560	296.988500	.000183
-89.701210	33.918400	297.478400	1.483362
-88.583520	34.125020	297.609600	1.760823
-87.455040	34.322360	298.000200	2.573437
-86.316120	34.510110	298.557100	3.702300
-85.167100	34.687960	299.135000	4.839263
-84.008420	34.855620	299.597500	5.732256
-82.840550	35.012790	299.863400	6.226236
-81.663990	35.159200	299.915900	6.308231
-80.479300	35.294580	299.833200	6.029567
-79.287040	35.418700	299.600500	5.335262
-78.087840	35.531310	298.974000	4.085748
-76.882320	35.632210	298.041500	4.040236
-75.671150	35.721200	296.267300	3.399365
-74.455030	35.798110	295.338700	2.733659
-73.234660	35.862810	295.869100	1.645461
-72.010770	35.915150	296.782900	1.558359
-70.784100	35.955040	297.411700	1.851566
-69.555380	35.982390	297.622600	1.864791
-68.325390	35.997170	297.569600	1.607597
-67.094890	35.999320	297.411400	1.179203
-65.864640	35.988860	297.242000	.745691
-64.635410	35.965800	297.114500	.404935
-63.407950	35.930180	297.043400	.195204
-62.183030	35.882080	297.011800	.093119
-60.961370	35.821590	297.000500	.049137
-59.743720	35.748840	296.997500	.033609
-58.530780	35.663950	296.997300	.027990
-57.323260	35.567100	296.996500	.023365
-56.121820	35.458470	296.993700	.015117
-54.927120	35.338260	296.991000	.007845
-53.739770	35.206700	296.989500	.003079
-52.560380	35.064020	296.988800	.001035
-51.389480	34.910500	296.988600	.000248
-50.227610	34.746390	296.988500	.000183
-49.075240	34.571990	296.988500	.000176
-47.932830	34.387580	296.988500	.000181

-46.800740	34.193490	296.988500	.000181
-45.679330	33.990000	296.988500	.000181
-44.568900	33.777440	296.988500	.000183
-89.960660	34.843320	298.418600	3.609339
-88.832330	35.057210	298.941300	4.546767
-87.692770	35.261540	300.347500	6.982805
-86.542320	35.455950	302.324100	10.243530
-85.381340	35.640140	304.345700	13.482780
-84.210270	35.813810	305.923200	15.919070
-83.029580	35.976620	306.787300	17.207720
-81.839790	36.128310	306.941900	17.424340
-80.641480	36.268600	306.710800	16.951760
-79.435230	36.397220	306.126600	15.532880
-78.221690	36.513940	305.160500	14.186730
-77.001530	36.618520	301.611600	13.593150
-75.775430	36.710780	296.564200	13.137070
-74.544130	36.790520	293.582900	8.172956
-73.308360	36.857590	294.737000	6.918462
-72.068890	36.911860	297.687400	7.087070
-70.826490	36.953220	299.490100	7.198600
-69.581950	36.981590	299.793400	6.589912
-68.336070	36.996900	299.311800	5.255430
-67.089650	36.999130	298.593200	3.720659
-65.843500	36.988290	297.897800	2.248899
-64.598400	36.964370	297.420900	1.170232
-63.355190	36.927440	297.169200	.548884
-62.114630	36.877570	297.064300	.255273
-60.877510	36.814860	297.029700	.144441
-59.644590	36.739430	297.023300	.108109
-58.416640	36.651430	297.024100	.098303
-57.194370	36.551040	297.020200	.080819
-55.978490	36.438440	297.008400	.051743
-54.769680	36.313860	296.997000	.023192
-53.568590	36.177530	296.991200	.008248
-52.375820	36.029710	296.989100	.002002
-51.191970	35.870660	296.988600	.000300
-50.017550	35.700660	296.988500	.000218
-48.853080	35.520030	296.988500	.000200
-47.698980	35.329070	296.988500	.000179
-46.555660	35.128100	296.988500	.000181
-45.423470	34.917440	296.988500	.000181
-44.302690	34.697410	296.988500	.000183
-90.232250	35.766320	301.030500	8.608936
-89.092860	35.987640	302.926500	11.487800
-87.941790	36.199090	307.714400	18.322570
-86.779330	36.400320	314.470400	27.438090
-85.605870	36.591000	321.334500	36.268060
-84.421870	36.770790	326.487000	42.682270
-83.227800	36.939400	329.348500	45.980290
-82.024180	37.096500	329.450300	46.163220
-80.811620	37.241800	328.403200	44.894240
-79.590740	37.375040	328.151800	43.272530
-78.362190	37.495960	319.950900	32.633280
-77.126670	37.604320	307.845700	37.561140
-75.884930	37.699920	290.372800	39.786550
-74.637700	37.782550	287.909300	34.366600
-73.385770	37.852060	296.811800	23.168710
-72.129940	37.908310	304.651300	25.098900
-70.871030	37.951180	307.885500	23.512120
-69.609860	37.980580	307.476500	19.903870
-68.347290	37.996460	305.388200	15.614350
-67.084140	37.998780	302.705800	10.877280
-65.821270	37.987530	300.068600	6.440921
-64.559530	37.962740	298.339400	3.183111
-63.299760	37.924460	297.542700	1.458610
-62.042780	37.872770	297.226400	.699934
-60.789440	37.807780	297.129400	.419043

-59.540510	37.729610	297.117500	.349175
-58.296810	37.638420	297.123400	.326253
-57.059080	37.534390	297.110000	.277010
-55.828080	37.417740	297.059900	.160964
-54.604500	37.288680	297.015200	.066857
-53.389020	37.147460	296.995000	.018074
-52.182290	36.994370	296.989300	.003031
-50.984880	36.829660	296.988300	.000697
-49.797380	36.653650	296.988300	.000807
-48.620270	36.466650	296.988400	.000484
-47.454020	36.268990	296.988500	.000261
-46.299010	36.060990	296.988500	.000179
-45.155610	35.843010	296.988500	.000180
-44.024070	35.615370	296.988500	.000181
-90.516530	36.687310	307.278300	18.543980
-89.365660	36.916220	313.348100	26.186290
-88.202590	37.134950	328.834000	44.047930
-87.027650	37.343150	351.655300	67.962530
-85.841200	37.540460	373.398700	90.080050
-84.643710	37.726540	389.669000	105.795200
-83.435650	37.901050	398.014800	112.614100
-82.217600	38.063690	396.703200	111.693600
-80.990140	38.214140	393.229000	109.036600
-79.753940	38.352110	388.190500	99.692150
-78.509670	38.477340	363.801800	79.465640
-77.258070	38.589580	309.723500	56.004140
-75.999910	38.688600	240.750000	63.175380
-74.735980	38.774210	278.197700	130.384800
-73.467090	38.846230	301.945800	88.830220
-72.194090	38.904510	342.079900	79.959650
-70.917830	38.948930	343.417000	71.549810
-69.639210	38.979390	333.960400	54.914860
-68.359090	38.995840	325.278900	41.870760
-67.078360	38.998240	316.155900	29.809150
-65.797930	38.986590	306.634400	17.022730
-64.518690	38.960900	300.864300	7.762071
-63.241520	38.921240	298.461800	3.463618
-61.967320	38.867690	297.676900	1.767537
-60.696920	38.800350	297.445200	1.182388
-59.431210	38.719360	297.429200	1.039699
-58.170970	38.624900	297.469700	1.022446
-56.917050	38.517150	297.466400	.885864
-55.670200	38.396340	297.213000	.455847
-54.431160	38.262690	297.057500	.149363
-53.200640	38.116470	296.997900	.028388
-51.979310	37.957960	296.986200	.004974
-50.767770	37.787460	296.986400	.005511
-49.566610	37.605300	296.987600	.003110
-48.376340	37.411790	296.988200	.001411
-47.197410	37.207270	296.988400	.000563
-46.030250	36.992090	296.988500	.000235
-44.875180	36.766620	296.988500	.000175
-43.732470	36.531200	296.988500	.000181
-90.814130	37.606180	319.538500	34.290390
-89.651300	37.842850	337.985800	53.138430
-88.475770	38.069030	386.953700	96.917810
-87.287830	38.284350	457.968400	153.651500
-86.087840	38.488450	523.480100	206.503700
-84.876290	38.680970	568.999200	239.834400
-83.653650	38.861550	590.460300	253.362300
-82.420500	39.029860	581.965600	245.648800
-81.177470	39.185590	567.515100	235.445700
-79.925230	39.328410	561.866000	225.854900
-78.664510	39.458070	515.540400	186.302200
-77.396060	39.574290	381.368200	121.422900
-76.120680	39.676830	222.427200	116.592500
-74.839220	39.765490	194.392000	139.297900

-73.552520	39.840080	283.198900	187.094100
-72.261480	39.900440	402.570200	198.890200
-70.967010	39.946460	448.138000	179.712900
-69.670030	39.978020	412.820100	139.188500
-68.371470	39.995060	380.081700	101.712800
-67.072270	39.997550	350.017200	68.988620
-65.773400	39.985470	321.727000	38.413490
-64.475770	39.958870	306.396900	17.011820
-63.180340	39.917780	300.581900	7.229528
-61.888020	39.862300	298.830300	4.168243
-60.599740	39.792560	298.390200	3.131023
-59.316390	39.708680	298.460400	2.950675
-58.038840	39.610860	298.762100	3.044021
-56.767930	39.499290	298.717800	2.672343
-55.504470	39.374200	297.637800	1.081648
-54.249260	39.235840	297.094600	.238473
-53.003000	39.084480	296.973600	.030320
-51.766410	38.920440	296.967200	.044720
-50.540120	38.744010	296.978400	.024468
-49.324710	38.555530	296.985000	.010042
-48.120720	38.355340	296.987500	.003739
-46.928620	38.143810	296.988200	.001322
-45.748810	37.921300	296.988500	.000455
-44.581620	37.688170	296.988500	.000202
-43.427300	37.444810	296.988500	.000182
-91.125630	38.522790	339.383700	53.652450
-89.950410	38.767390	387.608300	92.477110
-88.761920	39.001200	507.580400	175.039600
-87.560450	39.223820	676.005700	284.079400
-86.346370	39.434880	833.150100	386.310200
-85.120160	39.633990	941.406100	456.953900
-83.882300	39.820790	992.294900	485.300500
-82.633390	39.994930	966.450300	460.647900
-81.374080	40.156070	936.312000	440.802500
-80.105060	40.303890	951.426900	447.707800
-78.827100	40.438090	937.531400	427.946400
-77.540980	40.558400	749.241300	299.738900
-76.247540	40.664560	388.082100	145.862900
-74.947690	40.756360	123.210200	108.564200
-73.642300	40.833600	199.303900	124.200000
-72.332310	40.896110	376.230300	236.343200
-71.018690	40.943760	564.678000	309.611400
-69.702430	40.976440	538.922800	250.876100
-68.384490	40.994090	459.838100	176.171500
-67.065880	40.996670	386.196000	101.892100
-65.747600	40.984170	332.458300	52.627830
-64.430650	40.956610	311.844000	26.428730
-63.116020	40.914060	304.335000	13.538410
-61.804690	40.856610	301.941600	9.395428
-60.497620	40.784390	301.322600	8.053484
-59.195770	40.697540	301.857700	7.931983
-57.900040	40.596260	302.569700	8.118588
-56.611330	40.480750	301.648700	6.649742
-55.330490	40.351270	298.209900	1.855076
-54.058330	40.208070	296.874700	.231786
-52.795620	40.051450	296.808900	.303447
-51.543080	39.881710	296.899300	.177184
-50.301390	39.699200	296.955500	.074584
-49.071130	39.504250	296.978300	.026525
-47.852850	39.297230	296.985700	.008764
-46.647030	39.078520	296.987800	.002769
-45.454070	38.848490	296.988400	.000856
-44.274280	38.607530	296.988500	.000267
-43.107920	38.356030	296.988500	.000186
-91.451740	39.437030	356.371300	62.787210
-90.263630	39.689750	428.564300	107.901400
-89.061680	39.931370	590.488700	188.070500

-87.846150	40.161480	825.080600	307.322000
-86.617400	40.379670	1062.055000	440.535600
-85.375900	40.585540	1258.698000	586.445100
-84.122160	40.778730	1356.535000	646.113300
-82.856800	40.958850	1320.302000	614.548800
-81.580460	41.125550	1309.351000	573.996000
-80.293880	41.278500	1412.186000	653.564000
-78.997860	41.417370	1481.038000	698.632900
-77.693230	41.541890	1310.387000	586.343200
-76.380870	41.651780	775.627500	280.678600
-75.061690	41.746810	203.422600	103.740500
-73.736660	41.826770	71.574520	65.827580
-72.406780	41.891490	355.602600	273.688000
-71.073040	41.940830	598.218400	360.380800
-69.736500	41.974670	590.919100	299.952100
-68.398190	41.992950	460.205200	175.467600
-67.059160	41.995620	348.360700	70.677910
-65.720480	41.982670	306.916100	28.319880
-64.383220	41.954140	310.423100	25.606490
-63.048400	41.910080	313.574300	24.726400
-61.717090	41.850600	311.119500	22.003330
-60.390290	41.775820	309.619500	19.437810
-59.069010	41.685920	309.839800	18.317590
-57.754210	41.581080	308.986100	16.235520
-56.446830	41.461530	303.928800	9.146217
-55.147770	41.327530	297.234300	2.232831
-53.857880	41.179360	295.500900	2.267358
-52.577970	41.017320	296.238500	1.186660
-51.308780	40.841740	296.737800	.456638
-50.051000	40.652980	296.909900	.166462
-48.805250	40.451390	296.966400	.053991
-47.572090	40.237360	296.982700	.016732
-46.352010	40.011290	296.987100	.005073
-45.145370	39.773560	296.988200	.001461
-43.952510	39.524580	296.988500	.000429
-42.773630	39.264750	296.988500	.000202
-91.793200	40.348750	335.393200	42.453450
-90.591710	40.609810	365.809700	54.923250
-89.375760	40.859430	440.587100	89.294800
-88.145600	41.097210	578.364700	143.772400
-86.901570	41.322720	746.055500	236.154400
-85.644150	41.535550	920.398500	366.361900
-84.373860	41.735290	1039.527000	478.778400
-83.091290	41.921560	1036.559000	435.038500
-81.797160	42.093980	1173.364000	457.732200
-80.492230	42.252200	1511.243000	645.246200
-79.177290	42.395880	1708.998000	792.583700
-77.853240	42.524730	1559.055000	701.239700
-76.521000	42.638450	1009.796000	396.151500
-75.181550	42.736820	365.817100	133.908000
-73.835900	42.819590	231.253200	108.029900
-72.485100	42.886590	496.824600	323.444600
-71.130210	42.937670	725.879400	407.954000
-69.772340	42.972710	618.952800	323.940700
-68.412590	42.991630	413.353700	153.630700
-67.052090	42.994400	303.685700	51.277090
-65.691960	42.980990	306.575300	39.437120
-64.333320	42.951450	350.686500	58.226910
-62.977290	42.905830	355.168300	65.349560
-61.624970	42.844260	340.116400	53.450120
-60.277440	42.766850	327.957900	41.595420
-58.935750	42.673790	321.681900	33.311210
-57.600940	42.565280	314.841100	25.649430
-56.273990	42.441570	304.379500	13.891020
-54.955840	42.302930	293.946400	9.490276
-53.647390	42.149640	292.978100	6.344027
-52.349470	41.982030	295.294600	2.423535

-51.062890	41.800450	296.491100	.863140
-49.788320	41.605260	296.854100	.278820
-48.526440	41.396860	296.953000	.084985
-47.277780	41.175640	296.979600	.025104
-46.042850	40.942000	296.986400	.007590
-44.822010	40.696380	296.988000	.002183
-43.615560	40.439180	296.988400	.000595
-42.423690	40.170830	296.988500	.000235
-92.150770	41.257820	304.216200	24.140000
-90.935390	41.527410	290.067000	32.819540
-89.704910	41.785250	267.331100	64.553490
-88.459540	42.030900	238.996600	68.651180
-87.199620	42.263930	275.736700	101.937900
-85.925610	42.483890	303.123600	127.364000
-84.638040	42.690380	340.809700	152.878000
-83.337520	42.882970	353.052500	201.015700
-82.024790	43.061280	757.531300	277.247300
-80.700610	43.224930	1390.387000	603.157000
-79.365870	43.373580	1738.288000	828.135600
-78.021450	43.506890	1650.724000	781.139200
-76.668370	43.624570	1212.985000	471.280800
-75.307630	43.726360	725.744100	165.448000
-73.940300	43.812040	630.002300	195.619600
-72.567500	43.881390	792.369500	346.741100
-71.190370	43.934270	867.759800	449.634100
-69.810050	43.970550	709.226900	340.166900
-68.427750	43.990140	521.958900	218.125900
-67.044650	43.993000	449.400600	146.843200
-65.661930	43.979120	483.890900	158.726500
-64.280810	43.948540	505.515500	182.479900
-62.902460	43.901310	463.979600	155.953700
-61.528050	43.837570	404.698100	111.374900
-60.158730	43.757450	362.607800	79.053130
-58.795620	43.661140	342.853500	64.877140
-57.439790	43.548850	332.474600	48.331410
-56.092310	43.420850	313.260700	32.062890
-54.754150	43.277410	295.039900	12.269550
-53.426270	43.118850	292.867100	5.915441
-52.109530	42.945500	295.107100	3.104669
-50.804760	42.757750	296.327700	1.343750
-49.512690	42.555960	296.790200	.449615
-48.233980	42.340550	296.938900	.124063
-46.969190	42.111940	296.977400	.033284
-45.718810	41.870550	296.986000	.009365
-44.483210	41.616830	296.987900	.002711
-43.262650	41.351200	296.988400	.000702
-42.057300	41.074100	296.988500	.000255
-92.525320	42.164070	293.682100	13.308640
-91.295520	42.442420	283.268300	35.069010
-90.049940	42.708690	259.188100	44.670930
-88.788760	42.962440	277.855400	102.571900
-87.512310	43.203190	273.131200	108.036500
-86.221000	43.430500	256.375100	140.437900
-84.915410	43.643920	218.080400	146.946200
-83.596150	43.843030	314.207200	131.904500
-82.263960	44.027400	775.310700	280.941300
-80.919660	44.196660	1438.797000	623.463300
-79.564150	44.350410	1754.419000	851.369600
-78.198390	44.488330	1646.178000	783.879800
-76.823410	44.610100	1273.487000	502.330500
-75.440290	44.715440	883.914400	221.589500
-74.050180	44.804110	742.971900	151.887300
-72.654240	44.875890	798.000500	294.025200
-71.253690	44.930630	821.651600	344.660500
-69.849760	44.968180	750.408000	334.493100
-68.443720	44.988460	670.193200	287.435500
-67.036810	44.991420	663.936400	282.817000

-65.630330	44.977050	687.756500	302.946400
-64.225530	44.945390	665.944500	297.578700
-62.823690	44.896510	581.934800	245.485500
-61.426040	44.830540	487.737100	176.426600
-60.033810	44.747610	421.194600	130.506400
-58.648170	44.647940	390.297000	105.737200
-57.270290	44.531750	367.367900	82.110730
-55.901260	44.399320	332.851900	43.062440
-54.542130	44.250930	295.621200	10.873180
-53.193900	44.086940	288.876600	12.324220
-51.857480	43.907680	293.647200	6.572660
-50.533710	43.713560	295.876900	2.821620
-49.223370	43.504980	296.662100	.862706
-47.927120	43.282370	296.915000	.214131
-46.645540	43.046150	296.975200	.048635
-45.379100	42.796800	296.986200	.011420
-44.128160	42.534760	296.988000	.002798
-42.892960	42.260480	296.988400	.000700
-41.673610	41.974410	296.988500	.000228
-92.917770	43.067330	296.946200	6.757174
-91.673000	43.354680	302.644100	17.275980
-90.411740	43.629630	325.802800	51.770820
-89.134110	43.891690	366.243200	79.273510
-87.840450	44.140390	400.476400	96.011920
-86.531140	44.375260	327.336300	82.696210
-85.206750	44.595830	357.398700	106.334300
-83.867900	44.801650	524.154500	118.609500
-82.515380	44.992280	899.917200	312.393400
-81.150000	45.167310	1330.830000	594.318100
-79.772730	45.326340	1510.081000	723.671400
-78.384560	45.469020	1459.092000	688.447100
-76.986600	45.595010	1239.639000	517.813600
-75.579970	45.704020	969.210100	296.191200
-74.165890	45.795780	821.532300	231.743000
-72.745610	45.870080	787.022600	251.427700
-71.320400	45.926740	783.884700	293.720900
-69.891600	45.965610	760.441500	298.231800
-68.460530	45.986610	741.485400	309.101200
-67.028560	45.989670	750.191600	331.494700
-65.597030	45.974800	737.626500	342.038600
-64.167300	45.942020	689.558700	322.853900
-62.740720	45.891430	616.183300	279.405200
-61.318600	45.823140	538.508200	227.124800
-59.902260	45.737310	478.065300	181.636700
-58.492950	45.634170	437.192700	147.537700
-57.091900	45.513950	395.089000	112.572400
-55.700260	45.376930	334.726500	53.223630
-54.319140	45.223450	281.011800	38.857540
-52.949590	45.053840	274.035600	37.123500
-51.592580	44.868490	287.366200	16.990890
-50.248970	44.667810	294.848000	5.291158
-48.919560	44.452230	296.546800	1.461828
-47.605040	44.222190	296.907900	.338778
-46.305980	43.978160	296.980200	.076553
-45.022840	43.720610	296.988800	.017729
-43.755960	43.450020	296.988900	.003818
-42.505550	43.166850	296.988600	.000703
-41.271660	42.871580	296.988600	.000222
-93.329110	43.967430	295.462100	7.849133
-92.068780	44.264030	304.350600	15.460600
-90.791210	44.547890	331.728000	42.024850
-89.496510	44.818510	368.971100	67.780780
-88.184940	45.075390	351.495500	80.650350
-86.856880	45.318050	253.784100	74.341620
-85.512890	45.545990	366.285900	49.031940
-84.153590	45.758730	670.300200	168.379000
-82.779790	45.955820	974.502400	359.685000

-81.392350	46.136820	1174.962000	509.385400
-79.992260	46.301310	1249.916000	579.542800
-78.580580	46.448910	1242.292000	575.638000
-77.158460	46.579270	1137.937000	482.390200
-75.727110	46.692070	988.125500	382.538000
-74.287820	46.787040	893.340300	339.971100
-72.841890	46.863950	851.636200	333.487700
-71.390720	46.922590	816.050500	323.790100
-69.935700	46.962830	763.388200	289.751700
-68.478260	46.984570	749.149600	290.348300
-67.019850	46.987740	776.535900	327.075300
-65.561920	46.972340	754.503200	336.872700
-64.105920	46.938410	707.221600	325.059100
-62.653260	46.886040	648.476000	296.654000
-61.205390	46.815350	584.176100	260.164200
-59.763680	46.726520	526.885700	223.730900
-58.329470	46.619790	473.767300	188.861900
-56.904060	46.495390	412.728000	140.960100
-55.488670	46.353640	348.436600	89.744540
-54.084500	46.194880	295.553300	64.787060
-52.692630	46.019470	279.416800	46.783250
-51.314070	45.827840	288.999200	22.367380
-49.949730	45.620390	295.607900	6.814789
-48.600430	45.397590	297.029400	2.061325
-47.266860	45.159900	297.037400	.503412
-45.949600	44.907820	297.010800	.129908
-44.649100	44.641840	296.996700	.036213
-43.365670	44.362450	296.990600	.008315
-42.099450	44.070140	296.989000	.001732
-40.850440	43.765410	296.988600	.000460
-93.760410	44.864160	294.589800	8.807892
-92.483920	45.170280	302.763400	19.006260
-91.189420	45.463320	327.523400	49.743850
-89.876960	45.742750	346.576000	67.377010
-88.546760	46.008070	317.916100	77.649800
-87.199170	46.258760	287.351100	65.287340
-85.834720	46.494300	460.238600	92.299240
-84.454070	46.714200	805.061100	271.478800
-83.058010	46.917960	1050.536000	438.511900
-81.647480	47.105130	1137.582000	515.850600
-80.223460	47.275260	1110.458000	504.879800
-78.787090	47.427960	1003.546000	414.871000
-77.339580	47.562840	854.281000	322.647200
-75.882230	47.679570	765.151800	287.559300
-74.416370	47.777870	772.056300	312.148700
-72.943440	47.857470	801.470900	345.212400
-71.464880	47.918190	777.429400	314.123600
-69.982220	47.959850	690.204500	256.304400
-68.496960	47.982350	658.596100	204.623500
-67.010670	47.985630	726.490300	250.522000
-65.524890	47.969690	767.666600	296.670700
-64.041170	47.934570	769.365300	320.964500
-62.561040	47.880340	732.204800	314.760000
-61.086020	47.807170	674.697000	291.060100
-59.617590	47.715230	613.608300	262.068400
-58.157170	47.604770	536.340500	221.256700
-56.706150	47.476050	450.934100	174.289700
-55.265830	47.329400	392.962900	133.363300
-53.837470	47.165180	345.480000	91.966840
-52.422200	46.983780	316.560700	57.706880
-51.021090	46.785640	303.270000	24.080730
-49.635090	46.571200	300.060700	11.632040
-48.265020	46.340950	298.292100	3.686925
-46.911610	46.095370	297.363500	1.048308
-45.575410	45.834990	297.066500	.234647
-44.256860	45.560320	297.005500	.053924
-42.956210	45.271870	296.992400	.012879

-41.673580	44.970160	296.989300	.002591
-40.408870	44.655690	296.988700	.000702
-94.212860	45.757310	297.301300	9.570322
-92.919560	46.073230	308.718700	27.968420
-91.607470	46.375730	340.309200	59.668120
-90.276550	46.664250	378.634800	89.962920
-88.926960	46.938270	385.780200	91.234410
-87.559010	47.197250	406.698600	92.683940
-86.173240	47.440650	573.132100	178.456000
-84.770290	47.667940	821.854900	338.002600
-83.350960	47.878600	1006.599000	465.599200
-81.916200	48.072160	1065.092000	491.631400
-80.467090	48.248140	969.817700	404.383700
-79.004780	48.406110	753.079000	262.379800
-77.530590	48.545680	564.710700	151.326300
-76.045850	48.666500	523.490200	149.729700
-74.552020	48.768250	586.241500	203.905000
-73.050600	48.850660	646.247300	251.909000
-71.543170	48.913520	630.285300	251.477100
-70.031330	48.956650	525.922500	158.129000
-68.516710	48.979950	460.351500	102.947500
-67.000980	48.983350	519.431000	112.221300
-65.485790	48.966840	614.390300	171.518100
-63.972820	48.930470	686.130600	223.167800
-62.463700	48.874330	720.727800	269.172600
-60.960050	48.798580	723.879500	281.737300
-59.463460	48.703410	695.423800	274.937100
-57.975440	48.589080	604.114900	236.844600
-56.497490	48.455880	476.470100	170.150300
-55.030970	48.304140	395.856700	121.746700
-53.577210	48.134270	353.433500	84.730090
-52.137430	47.946660	321.775800	46.531570
-50.712720	47.741790	304.572200	20.179370
-49.304080	47.520120	299.005000	6.477150
-47.912350	47.282170	297.821700	3.189963
-46.538250	47.028450	297.247100	.839524
-45.182340	46.759510	297.057700	.212922
-43.845020	46.475880	297.002400	.046886
-42.526500	46.178100	296.991100	.009181
-41.226800	45.866700	296.989000	.001987
-39.945740	45.542200	296.988700	.000499
-94.687740	46.646660	306.320700	21.182940
-93.376940	46.972690	324.137600	44.408550
-92.046550	47.284930	360.705400	78.509340
-90.696440	47.582840	397.407200	104.197200
-89.326680	47.865840	424.167900	107.205100
-87.937550	48.133390	477.258200	134.399200
-86.529520	48.384910	603.866000	207.017200
-85.103260	48.619850	772.038200	324.971400
-83.659580	48.837660	915.802400	409.745700
-82.199460	49.037830	940.439300	403.565700
-80.724000	49.219880	778.948600	303.929400
-79.234450	49.383340	542.654500	148.447900
-77.732160	49.527770	421.142900	95.321430
-76.218590	49.652830	441.844500	114.979400
-74.695260	49.758160	496.597600	147.333300
-73.163800	49.843490	528.387100	178.663900
-71.625870	49.908580	532.609400	186.405200
-70.083210	49.953250	467.660000	147.731600
-68.537570	49.977380	372.503200	67.696590
-66.990740	49.980900	370.137400	60.341050
-65.444490	49.963800	421.356400	79.252320
-63.900620	49.926140	474.648100	98.760730
-62.360880	49.868010	532.051900	137.312000
-60.827010	49.789570	602.874300	205.400800
-59.300730	49.691040	637.294200	238.527200
-57.783640	49.572690	578.285600	204.365200

-56.277320	49.434830	455.756700	139.942600
-54.783270	49.277820	369.948800	82.378910
-53.302860	49.102080	332.337500	51.430920
-51.837360	48.908040	311.053300	26.536640
-50.387950	48.696200	300.260300	9.030607
-48.955640	48.467040	297.540700	3.475271
-47.541310	48.221130	296.934300	.992535
-46.145660	47.959000	296.902500	.202803
-44.769230	47.681210	296.956800	.054858
-43.412380	47.388330	296.983100	.010729
-42.075270	47.080920	296.987900	.003299
-40.757810	46.759540	296.988300	.000610
-39.459720	46.424710	296.988500	.000197
-95.186440	47.531940	325.257500	45.112700
-93.857410	47.868410	350.111500	72.104110
-92.508000	48.190720	385.831200	105.404400
-91.137910	48.498310	410.492600	121.464100
-89.747150	48.790600	435.830900	128.368100
-88.335950	49.067010	498.502200	157.182100
-86.904710	49.326930	603.663500	227.412000
-85.454100	49.569790	712.672200	300.982700
-83.984930	49.795020	787.432500	337.702300
-82.498220	50.002060	736.361100	302.314700
-80.995120	50.190400	554.001500	182.225800
-79.476920	50.359550	400.881500	109.565100
-77.945060	50.509060	387.573700	103.940300
-76.401090	50.638520	439.279400	129.581300
-74.846650	50.747580	461.490600	138.555900
-73.283450	50.835950	468.013100	145.031400
-71.713320	50.903350	487.021500	161.557000
-70.138080	50.949630	471.970800	148.679000
-68.559640	50.974620	390.841200	79.927520
-66.979900	50.978270	349.189100	53.539580
-65.400800	50.960560	352.157100	56.419920
-63.824260	50.921550	356.039200	51.694270
-62.252170	50.861340	384.186300	63.358940
-60.686400	50.780110	453.759300	118.216700
-59.128770	50.678080	502.268800	161.386700
-57.581030	50.555550	476.008100	148.705600
-56.044860	50.412850	394.245000	92.153330
-54.521820	50.250360	337.168600	50.355720
-53.013420	50.068520	313.903800	26.789130
-51.520960	49.867810	303.467000	12.945420
-50.045680	49.648740	298.614700	4.668253
-48.588630	49.411830	297.102700	1.425322
-47.150700	49.157670	296.815700	.374505
-45.732590	48.886830	296.884800	.163306
-44.334810	48.599900	296.960900	.044464
-42.957660	48.297460	296.982600	.012636
-41.601190	47.980120	296.987100	.003361
-40.265250	47.648430	296.988200	.000844
-38.949390	47.302930	296.988500	.000303
-95.710510	48.412890	356.604300	78.053100
-94.362470	48.760130	388.838300	107.957600
-92.993230	49.092850	422.030400	136.782200
-91.602360	49.410460	434.780500	144.981800
-90.189750	49.712350	445.276900	144.415100
-88.755540	49.997940	483.269100	164.911800
-87.300090	50.266570	541.219100	208.638000
-85.824040	50.517650	588.520400	241.883400
-84.328210	50.750570	603.021000	248.779400
-82.813610	50.964740	534.748400	192.636800
-81.281460	51.159630	417.162800	131.626100
-79.733130	51.334700	360.251400	101.513600
-78.170130	51.489480	395.142800	122.332100
-76.594090	51.623530	436.869000	145.059800
-75.006800	51.736490	443.838300	135.368500

-73.410070	51.828010	447.371500	135.769700
-71.805870	51.897850	463.217600	152.785500
-70.196170	51.945790	463.218600	139.807700
-68.582990	51.971680	405.185000	86.571340
-66.968440	51.975460	349.448200	59.631320
-65.354560	51.957120	345.405000	56.271340
-63.743440	51.916690	337.775100	39.977470
-62.137130	51.854320	346.463000	40.210420
-60.537630	51.770170	384.461800	74.261430
-58.946900	51.664510	406.830600	103.211300
-57.366820	51.537620	386.252700	87.171330
-55.799180	51.389870	343.494900	54.255930
-54.245650	51.221680	316.094200	27.914360
-52.707820	51.033510	304.858500	14.035910
-51.187080	50.825860	299.940200	6.401247
-49.684720	50.599280	297.857900	2.399436
-48.201800	50.354330	297.122600	.698850
-46.739240	50.091630	296.943600	.165253
-45.297720	49.811770	296.959200	.054983
-43.877700	49.515370	296.978300	.022878
-42.479410	49.203060	296.985000	.007817
-41.102810	48.875440	296.987600	.002330
-39.747590	48.533100	296.988300	.000680
-38.413150	48.176570	296.988500	.000271
-96.261670	49.289190	386.613600	103.365500
-94.893750	49.647600	420.688100	132.774900
-93.503820	49.991080	442.362000	148.562600
-92.091320	50.319060	436.652600	142.334500
-90.655950	50.630910	423.974500	129.195500
-89.197760	50.925990	422.516400	126.703400
-87.717060	51.203660	432.260300	135.685500
-86.214420	51.463270	443.916700	148.954800
-84.690660	51.704190	450.367900	149.695700
-83.146820	51.925780	422.850400	133.376100
-81.584160	52.127480	378.118200	102.131900
-80.004110	52.308710	366.375200	101.502100
-78.408280	52.468990	390.028300	115.200600
-76.798410	52.607840	419.632300	131.852700
-75.176390	52.724850	442.901900	135.400900
-73.544200	52.819690	455.283800	139.953800
-71.903920	52.892050	458.904500	151.490300
-70.257710	52.941730	464.210200	151.656900
-68.607750	52.968570	426.413400	101.063000
-66.956280	52.972490	368.445300	66.808270
-65.305560	52.953480	354.641000	60.316300
-63.657820	52.911580	350.117200	53.162960
-62.015270	52.846950	349.086000	44.607090
-60.380080	52.759750	357.472300	57.571530
-58.754360	52.650280	356.088600	62.913220
-57.140120	52.518850	339.930700	50.036240
-55.539290	52.365840	319.531900	30.263410
-53.953660	52.191710	306.706400	15.795810
-52.384890	51.996940	300.723200	7.404984
-50.834480	51.782070	298.323700	3.157479
-49.303730	51.547700	297.410700	1.204751
-47.793770	51.294400	297.080800	.377435
-46.305490	51.022820	296.995400	.094627
-44.839570	50.733620	296.983900	.026840
-43.396390	50.427420	296.985200	.008522
-41.976090	50.104880	296.987200	.003146
-40.578500	49.766630	296.988100	.001066
-39.203150	49.413260	296.988400	.000373
-37.849210	49.045330	296.988500	.000230
-96.841810	50.160530	380.727400	96.186830
-95.453030	50.530490	402.186600	112.207700
-94.041510	50.885130	405.708300	115.077800
-92.606450	51.223850	388.136500	99.151140

-91.147380	51.546020	368.284700	80.760440
-89.664200	51.850960	354.920900	69.533660
-88.157130	52.138020	350.732900	67.108990
-86.626690	52.406500	353.652600	70.085220
-85.073680	52.655720	361.307900	78.595830
-83.499180	52.885050	356.370300	73.197490
-81.904440	53.093850	339.610900	70.138930
-80.290990	53.281520	329.602100	60.330820
-78.660530	53.447530	340.761100	69.824040
-77.014920	53.591380	379.346700	97.003090
-75.356160	53.712650	429.382400	125.310300
-73.686420	53.810940	460.459000	140.525600
-72.007930	53.885960	459.604700	147.895600
-70.322990	53.937460	462.578700	153.917300
-68.634010	53.965280	446.737500	124.879900
-66.943400	53.969350	401.935700	84.371090
-65.253590	53.949630	374.374200	67.890750
-63.567000	53.906200	360.766400	60.543370
-61.886040	53.839190	346.281500	45.252450
-60.213040	53.748820	338.350000	44.066170
-58.550290	53.635360	330.761000	40.336320
-56.899960	53.499180	321.274600	31.140590
-55.264110	53.340690	310.101800	19.006730
-53.644650	53.160350	302.298900	9.331929
-52.043340	52.958710	298.870900	4.060418
-50.461760	52.736320	297.621700	1.636123
-48.901280	52.493820	297.185000	.605900
-47.363030	52.231850	297.040000	.198672
-45.847900	51.951060	296.998200	.057313
-44.356530	51.652150	296.988900	.013412
-42.889220	51.335790	296.987900	.003187
-41.445980	51.002650	296.988200	.000921
-40.026490	50.653380	296.988400	.000375
-38.630060	50.288590	296.988500	.000227
-37.255620	49.908830	296.988500	.000189
-97.453060	51.026530	344.762300	59.511020
-96.042340	51.408490	352.705600	65.666520
-94.608220	51.774700	348.040800	60.688110
-93.149600	52.124570	335.195800	48.525560
-91.665800	52.457450	324.402300	37.956620
-90.156560	52.772640	317.442800	30.972120
-88.621980	53.069450	314.760300	28.253870
-87.062480	53.347160	315.180200	28.096050
-85.478850	53.605050	316.956400	28.559210
-83.872130	53.842430	312.236200	30.850130
-82.243660	54.058630	296.079800	27.068180
-80.595020	54.253030	282.642800	28.105420
-78.928000	54.425040	290.694500	34.055070
-77.244600	54.574130	334.977600	58.236670
-75.546970	54.699840	395.477000	100.112700
-73.837420	54.801750	439.508900	127.423100
-72.118360	54.879540	444.311100	134.375500
-70.392330	54.932960	438.959600	136.091000
-68.661900	54.961820	435.658900	125.364200
-66.929700	54.966030	408.525100	87.230790
-65.198370	54.945580	381.287900	71.044530
-63.470540	54.900540	355.218000	52.323180
-61.748820	54.831050	334.522600	38.165410
-60.035740	54.737340	323.549100	31.819770
-58.333780	54.619720	320.138500	29.623100
-56.645260	54.478570	314.293800	23.428030
-54.972420	54.314330	305.695100	13.313250
-53.317280	54.127510	300.237400	6.052983
-51.681740	53.918690	298.061600	2.471827
-50.067420	53.688470	297.326900	.932398
-48.475770	53.437500	297.089300	.329194
-46.907930	53.166490	297.015700	.107402

-45.364770	52.876130	296.994700	.030955
-43.846840	52.567130	296.989600	.007792
-42.354370	52.240220	296.988600	.001462
-40.887210	51.896080	296.988500	.000279
-39.444820	51.535370	296.988500	.000195
-38.026250	51.158730	296.988500	.000172
-36.630120	50.766700	296.988500	.000177
-98.097820	51.886770	315.691900	28.000250
-96.663910	52.281200	317.106300	28.206190
-95.206040	52.659450	314.188000	25.122020
-93.722790	53.020900	309.386300	19.921430
-92.213200	53.364910	305.794400	15.425510
-90.676770	53.690770	303.553300	12.324990
-89.113440	53.997730	302.365400	10.391320
-87.523560	54.285050	301.334900	8.603490
-85.907830	54.551970	298.626600	6.912057
-84.267300	54.797760	289.382000	14.353240
-82.603340	55.021710	271.343100	31.086650
-80.917590	55.223140	256.845500	39.354890
-79.211960	55.401440	264.168600	35.660440
-77.488560	55.556020	307.149200	33.623230
-75.749730	55.686380	365.683000	76.086370
-73.997920	55.792100	415.328400	111.064200
-72.235800	55.872810	430.747000	121.634900
-70.466090	55.928230	419.997400	117.988300
-68.691570	55.958180	410.016300	102.346900
-66.915130	55.962550	389.262700	72.628560
-65.139640	55.941330	362.763300	54.259420
-63.367970	55.894590	337.033200	37.822780
-61.602940	55.822490	320.219500	27.294620
-59.847310	55.725280	315.352400	24.293680
-58.103760	55.603290	316.699300	26.010780
-56.374820	55.456930	311.250600	19.732740
-54.662870	55.286670	303.480300	10.301170
-52.970100	55.093080	299.226700	4.400314
-51.298500	54.876750	297.688700	1.686608
-49.649800	54.638340	297.197800	.597313
-48.025460	54.378550	297.046800	.196605
-46.426660	54.098120	297.003600	.060624
-44.854210	53.797780	296.992200	.017362
-43.308580	53.478290	296.989300	.004280
-41.789850	53.140400	296.988600	.000854
-40.297680	52.784830	296.988500	.000173
-38.831280	52.412250	296.988500	.000160
-37.389370	52.023280	296.988500	.000172
-35.970190	51.618480	296.988500	.000178
-98.778840	52.740810	303.136700	11.377760
-97.320270	53.148240	303.047800	10.764670
-95.837370	53.539010	302.058900	9.349618
-94.328290	53.912530	300.762000	7.485537
-92.791720	54.268120	299.706500	5.723539
-91.226900	54.605080	298.908400	4.236971
-89.633560	54.922620	298.179700	2.772213
-88.011890	55.219970	296.712200	1.709952
-86.362530	55.496330	292.136800	8.020416
-84.686490	55.750910	280.051800	23.059280
-82.985150	55.982970	260.162800	44.512700
-81.260250	56.191770	245.064600	52.423940
-79.513790	56.376650	253.242500	44.013550
-77.748020	56.536990	294.285700	22.088130
-75.965460	56.672260	346.852300	59.019540
-74.168790	56.781970	398.544200	98.705870
-72.360860	56.865740	429.325000	116.767900
-70.544630	56.923280	422.813700	111.341600
-68.723180	56.954370	400.102700	86.666340
-66.899620	56.958910	369.025200	56.069690
-65.077100	56.936880	339.931500	36.126370

-63.258730 56.888360 320.290900 25.432900
-61.447630 56.813510 310.700200 18.765190
-59.646790 56.712620 312.031900 21.134220
-57.859090 56.586040 315.251700 24.129060
-56.087300 56.434210 309.536100 17.512090
-54.333970 56.257640 302.251300 8.571849
-52.601470 56.056940 298.699700 3.472386
-50.891880 55.832740 297.504600 1.270243
-49.207030 55.585770 297.134700 .422967
-47.548420 55.316760 297.026900 .130058
-45.917200 55.026500 296.998000 .038171
-44.314130 54.715760 296.990800 .010534
-42.739580 54.385350 296.989000 .002611
-41.193420 54.036030 296.988600 .000543
-39.675070 53.668560 296.988500 .000175
-38.183400 53.283620 296.988500 .000162
-36.716770 52.881820 296.988500 .000175
-35.272900 52.463700 296.988500 .000181
-99.499240 53.588130 298.958800 4.286028
-98.014280 54.009130 298.721100 3.771572
-96.504860 54.412960 298.444500 3.224815
-94.968610 54.799050 298.088200 2.554274
-93.403790 55.166730 297.746900 1.850224
-91.809290 55.515250 297.408000 1.077495
-90.184560 55.843850 296.818100 .511675
-88.529620 56.151670 295.124800 3.731690
-86.844990 56.437890 289.704500 12.027000
-85.131650 56.701690 276.042400 29.130830
-83.390960 56.942230 253.912100 53.863940
-81.624720 57.158780 238.537800 60.612020
-79.835050 57.350580 250.290800 46.655630
-78.024370 57.516980 288.985500 17.743210
-76.195360 57.657400 334.277500 45.911540
-74.350940 57.771310 385.926500 88.364230
-72.494220 57.858320 431.841000 115.122000
-70.628410 57.918090 436.389700 112.529400
-68.756890 57.950390 402.779300 84.502540
-66.883060 57.955100 355.273300 46.314310
-65.010370 57.932220 324.452700 27.006300
-63.142240 57.881810 309.873600 16.309230
-61.282040 57.804080 306.529100 14.590700
-59.433060 57.699310 310.674300 19.182760
-57.598450 57.567890 314.063000 22.553710
-55.781200 57.410300 308.053000 15.659690
-53.984060 57.227100 301.325400 7.261467
-52.209560 57.018940 298.347300 2.849879
-50.459930 56.786500 297.389500 1.006794
-48.737060 56.530560 297.098700 .321073
-47.042490 56.251900 297.016200 .094524
-45.377330 55.951360 296.995200 .026710
-43.742270 55.629770 296.990100 .007238
-42.137470 55.287960 296.988900 .001785
-40.562610 54.926740 296.988600 .000389
-39.016750 54.546860 296.988600 .000149
-37.498410 54.149020 296.988500 .000171
-36.005400 53.733830 296.988500 .000175
-34.534880 53.301770 296.988500 .000181
-100.262700 54.428120 297.571100 1.459039
-98.749250 54.863350 297.467900 1.220478
-97.211560 55.280850 297.379600 1.015308
-95.646590 55.680060 297.276400 .775091
-94.052090 56.060360 297.158800 .476465
-92.426510 56.420970 296.960400 .100735
-90.768940 56.761100 296.388600 1.420690
-89.079190 57.079890 294.520800 5.063040
-87.357570 57.376450 288.600400 14.006360
-85.605000 57.649890 273.434400 32.527080

-83.822840	57.899360	249.029600	58.588240
-82.012920	58.124030	234.059700	65.210140
-80.177480	58.323120	250.329300	47.007400
-78.319110	58.495910	287.394300	15.029410
-76.440710	58.641750	326.421300	35.159220
-74.545430	58.760110	376.582700	78.816370
-72.636660	58.850530	430.990400	110.298600
-70.717930	58.912650	447.840800	116.969900
-68.792920	58.946230	407.246300	85.936010
-66.865380	58.951130	349.110700	45.158660
-64.939080	58.927340	316.443800	21.684020
-63.017780	58.874950	305.338100	11.930690
-61.105210	58.794160	304.482200	10.976500
-59.204930	58.685300	309.773900	17.155800
-57.320400	58.548780	312.852400	20.756240
-55.454840	58.385130	306.802600	13.794210
-53.611270	58.194950	300.668300	6.202852
-51.792370	57.978940	298.095400	2.337155
-50.000500	57.737840	297.309300	.805259
-48.237630	57.472490	297.074400	.250372
-46.505310	57.183730	297.009500	.071567
-44.804610	56.872430	296.993400	.019848
-43.136050	56.539500	296.989700	.005276
-41.499610	56.185780	296.988800	.001288
-39.894630	55.812120	296.988600	.000308
-38.319800	55.419290	296.988600	.000156
-36.773090	55.007980	296.988500	.000176
-35.251730	54.578750	296.988500	.000175
-33.752120	54.132060	296.988500	.000183
-101.073700	55.260130	297.146900	.458355
-99.529010	55.710320	297.112300	.370261
-97.960900	56.142140	297.086000	.295678
-96.365430	56.555100	297.053400	.201533
-94.739640	56.948590	296.996900	.032255
-93.081440	57.321860	296.830300	.431719
-91.389490	57.674070	296.235400	1.825896
-89.663250	58.004340	294.237000	5.679713
-87.902820	58.311730	287.945000	14.958740
-86.109000	58.595320	271.460100	34.390110
-84.283100	58.854170	244.967100	60.932510
-82.427000	59.087400	231.107800	66.935240
-80.543050	59.294160	252.957100	44.417130
-78.634010	59.473690	289.685600	10.979380
-76.703000	59.625280	326.838200	33.957390
-74.753460	59.748340	368.607500	66.096720
-72.789070	59.842370	422.012800	104.831100
-70.813740	59.906970	446.556700	114.788800
-68.831480	59.941910	412.135200	91.574840
-66.846440	59.947010	349.307900	46.296660
-64.862770	59.922250	313.711800	19.354450
-62.884610	59.867760	301.221500	6.928175
-60.916040	59.783740	300.007100	3.899941
-58.961010	59.670550	305.688800	10.656300
-57.023270	59.528640	309.282100	15.668230
-55.106350	59.358580	304.788200	10.839970
-53.213500	59.161040	299.896300	4.742595
-51.347600	58.936760	297.851100	1.782012
-49.511150	58.686560	297.228600	.599553
-47.706190	58.411320	297.051900	.183730
-45.934240	58.111950	297.003900	.052282
-44.196280	57.789400	296.992100	.014330
-42.492650	57.444580	296.989400	.003798
-40.823030	57.078400	296.988700	.000929
-39.186360	56.691730	296.988600	.000230
-37.580830	56.285340	296.988600	.000153
-36.003750	55.859910	296.988500	.000175
-34.451600	55.415950	296.988500	.000178

-32.919830	54.953830	296.988500	.000183
-101.937200	56.083360	297.029400	.137842
-100.358100	56.549350	297.019100	.106816
-98.756940	56.996240	297.010700	.077862
-97.128790	57.423640	296.996300	.026576
-95.469860	57.830970	296.953800	.110697
-93.777320	58.217490	296.790200	.552923
-92.049300	58.582370	296.176800	1.961167
-90.284800	58.924680	294.111900	5.854174
-88.483640	59.243460	287.650600	15.082010
-86.646400	59.537720	270.615400	34.701500
-84.774360	59.806450	243.388400	60.389510
-82.869400	60.048700	232.089000	64.667220
-80.933970	60.263580	260.647100	37.417530
-78.971000	60.450230	302.119600	16.179020
-76.983890	60.607900	332.845300	32.573400
-74.976370	60.735940	362.359600	63.860070
-72.952470	60.833800	397.902000	87.939740
-70.916480	60.901050	431.528700	109.617500
-68.872850	60.937420	415.040300	93.228170
-66.826130	60.942730	353.980400	48.961400
-64.780930	60.916960	310.857400	17.323200
-62.741830	60.860230	292.806500	12.087970
-60.713310	60.772790	289.523800	13.253320
-58.699720	60.654990	295.970200	3.451200
-56.705200	60.507380	301.604900	5.467558
-54.733590	60.330550	300.760600	5.224079
-52.788400	60.125220	298.508600	2.589436
-50.872730	59.892220	297.465500	1.026801
-48.989200	59.632420	297.130500	.363145
-47.139900	59.346780	297.026900	.114322
-45.326320	59.036270	296.998100	.033038
-43.549270	58.701890	296.990800	.009268
-41.808860	58.344600	296.989100	.002457
-40.104370	57.965370	296.988700	.000601
-38.434220	57.565060	296.988600	.000200
-36.795950	57.144450	296.988600	.000149
-35.186060	56.704150	296.988500	.000176
-33.600030	56.244660	296.988500	.000180
-32.032190	55.766200	296.988500	.000183
-102.859900	56.896880	296.998700	.039383
-101.242000	57.379640	296.995700	.028420
-99.604390	57.842470	296.992000	.013494
-97.940930	58.285070	296.982000	.024310
-96.246660	58.706940	296.942800	.148109
-94.517820	59.107400	296.780900	.572313
-92.751850	59.485580	296.171800	1.927343
-90.947170	59.840560	294.144700	5.623227
-89.103220	60.171320	287.779400	14.337250
-87.220290	60.476800	271.040600	32.747460
-85.299550	60.755960	245.487200	55.864210
-83.342830	61.007760	238.966800	55.984380
-81.352710	61.231220	276.980500	28.551210
-79.332320	61.425420	317.199600	20.745030
-77.285290	61.589540	341.000200	46.994270
-75.215710	61.722870	347.548700	48.057130
-73.128000	61.824790	368.227500	71.404270
-71.026890	61.894870	405.908900	93.904980
-68.917300	61.932760	412.366300	89.938550
-66.804310	61.938290	357.156800	50.235130
-64.692990	61.911450	306.092100	19.767530
-62.588420	61.852340	283.981800	25.257830
-60.495590	61.761240	280.823000	25.695550
-58.419300	61.638580	287.573200	15.436850
-56.364090	61.484900	295.130800	3.491092
-54.334160	61.300890	297.361200	.489694
-52.333320	61.087320	297.357200	.705770

-50.364880	60.845090	297.156100	.395873
-48.431600	60.575160	297.047800	.162533
-46.535590	60.278550	297.005800	.055072
-44.678250	59.956310	296.993100	.017035
-42.860180	59.609490	296.989700	.004997
-41.081100	59.239120	296.988800	.001361
-39.339830	58.846180	296.988600	.000350
-37.634110	58.431540	296.988600	.000166
-35.960610	57.995940	296.988500	.000178
-34.314830	57.539980	296.988500	.000180
-32.690990	57.064010	296.988500	.000181
-31.082000	56.568130	296.988500	.000183
-103.849600	57.699570	296.991000	.010272
-102.187300	58.200280	296.989900	.005687
-100.509000	58.680030	296.987500	.004120
-98.806840	59.138710	296.979000	.035886
-97.074520	59.575940	296.942300	.147406
-95.307120	59.991050	296.789500	.531895
-93.501110	60.383240	296.221600	1.745110
-91.654170	60.751550	294.336500	4.982689
-89.765210	61.094930	288.340700	12.646660
-87.834170	61.412270	273.154800	28.097630
-85.861950	61.702450	251.565400	46.247170
-83.850420	61.964360	255.541400	42.619930
-81.802150	62.196920	295.627700	20.822440
-79.720490	62.399150	330.222400	41.853950
-77.609360	62.570130	335.683100	36.512820
-75.473210	62.709080	331.393400	38.639370
-73.316960	62.815350	339.540900	48.292370
-71.145790	62.888430	375.912300	72.358800
-68.965200	62.927950	394.300200	76.734480
-66.780790	62.933720	350.907900	45.606760
-64.598260	62.905720	304.607800	19.602930
-62.423240	62.844070	285.010900	23.496780
-60.261290	62.749080	283.621900	22.140940
-58.117710	62.621230	289.613700	11.862050
-55.997520	62.461110	294.681300	4.265865
-53.905320	62.269450	296.598100	.886568
-51.845240	62.047150	296.987800	.030607
-49.820820	61.795150	297.021000	.084333
-47.834930	61.514500	297.004900	.049769
-45.889670	61.206310	296.994200	.020027
-43.986290	60.871700	296.990300	.006997
-42.125070	60.511780	296.989100	.002241
-40.305270	60.127630	296.988700	.000646
-38.525020	59.720250	296.988600	.000219
-36.781200	59.290500	296.988600	.000151
-35.069400	58.839090	296.988500	.000180
-33.383770	58.366500	296.988500	.000180
-31.716990	57.872960	296.988500	.000181
-30.060130	57.358350	296.988500	.000183
-104.916500	58.490070	296.989000	.002120
-103.202300	59.010120	296.988500	.000276
-101.477600	59.507980	296.986800	.007648
-99.732440	59.983750	296.979400	.034162
-97.958720	60.437220	296.947500	.127764
-96.150010	60.867820	296.816800	.445178
-94.301540	61.274790	296.336700	1.425309
-92.410060	61.657170	294.756300	3.965950
-90.473700	62.013880	289.635900	10.090880
-88.491920	62.343760	276.874200	22.320120
-86.465330	62.645610	264.426200	31.583900
-84.395630	62.918240	276.154900	23.416320
-82.285480	63.160470	312.950000	31.114510
-80.138370	63.371240	329.507500	34.682640
-77.958560	63.549540	324.541200	30.770340
-75.750930	63.694510	315.144000	22.163980

-73.520860	63.805420	319.049000	28.921740
-71.274150	63.881700	342.058500	45.906140
-69.016910	63.922960	358.786800	52.568680
-66.755400	63.928990	332.824600	33.697840
-64.495980	63.899750	303.335600	13.780080
-62.244980	63.835380	292.201000	11.429830
-60.008560	63.736250	291.745200	9.522738
-57.792640	63.602840	294.079400	5.656814
-55.602750	63.435840	295.965400	2.243062
-53.443960	63.236060	296.715500	.669022
-51.320750	63.004450	296.935600	.143429
-49.236890	62.742080	296.984900	.010475
-47.195330	62.450080	296.990600	.007749
-45.198120	62.129630	296.989900	.005227
-43.246250	61.781950	296.989100	.002307
-41.339580	61.408210	296.988700	.000812
-39.476710	61.009530	296.988600	.000272
-37.654880	60.586900	296.988600	.000170
-35.869840	60.141170	296.988500	.000181
-34.115770	59.672970	296.988500	.000181
-32.385130	59.182660	296.988500	.000181
-30.668580	58.670220	296.988500	.000183
-28.954870	58.135290	296.988500	.000183
-106.073800	59.266680	296.988600	.000163
-104.297500	59.807790	296.988300	.001395
-102.518800	60.325180	296.986900	.007132
-100.725000	60.819250	296.981100	.028000
-98.905530	61.289990	296.956600	.099316
-97.052100	61.737010	296.859100	.332024
-95.158340	62.159620	296.510700	1.023761
-93.219700	62.556880	295.370600	2.748328
-91.233350	62.927700	291.733900	7.074476
-89.198040	63.270870	283.999400	13.965740
-87.113920	63.585110	279.263100	16.973840
-84.982480	63.869130	294.514100	11.879370
-82.806390	64.121680	317.133200	24.631570
-80.589290	64.341560	320.265800	26.052500
-78.335770	64.527670	311.205600	16.356540
-76.051160	64.679080	305.267000	12.004850
-73.741460	64.794950	306.562200	15.011570
-71.413090	64.874680	315.611400	22.676250
-69.072890	64.917820	324.215300	27.202640
-66.727910	64.924110	312.990900	18.018320
-64.385280	64.893550	300.946900	7.304356
-62.052090	64.826280	296.351600	3.733407
-59.735260	64.722690	295.731300	3.100215
-57.441380	64.583340	296.173500	1.946884
-55.176610	64.408980	296.624200	.912009
-52.946510	64.200520	296.864900	.333584
-50.755950	63.959000	296.957200	.091508
-48.608900	63.685590	296.982700	.018783
-46.508400	63.381530	296.987900	.002043
-44.456330	63.048090	296.988700	.000589
-42.453320	62.686580	296.988600	.000529
-40.498620	62.298210	296.988600	.000260
-38.589950	61.884150	296.988600	.000169
-36.723370	61.445380	296.988600	.000155
-34.893150	60.982680	296.988500	.000183
-33.091610	60.496560	296.988500	.000181
-31.309040	59.987160	296.988500	.000181
-29.533510	59.454220	296.988500	.000183
-27.750760	58.896990	296.988500	.000183
-107.339200	60.027230	296.988500	.000382
-105.487000	60.591610	296.988300	.001477
-103.643800	61.130270	296.987200	.006092
-101.793500	61.644070	296.982900	.022252
-99.922550	62.133320	296.965700	.074199

-98.020040	62.597820	296.901900	.231810
-96.077510	63.037030	296.687600	.669985
-94.088710	63.450070	296.060600	1.690611
-92.049460	63.835860	294.336900	3.759367
-89.957580	64.193130	291.013300	6.984072
-87.812570	64.520550	291.072200	6.351402
-85.615560	64.816710	302.357800	8.164413
-83.369120	65.080260	311.476500	14.951490
-81.077050	65.309890	308.862500	13.999150
-78.744270	65.504390	302.904200	7.752473
-76.376630	65.662700	300.695500	6.563446
-73.980770	65.783920	300.892100	7.308127
-71.563890	65.867360	303.157700	9.663478
-69.133670	65.912500	305.896200	11.396910
-66.698060	65.919100	302.412600	7.910253
-64.265090	65.887100	298.909200	3.690861
-61.842790	65.816700	297.276600	1.295147
-59.438900	65.708320	296.791100	.805572
-57.060850	65.562580	296.777200	.593543
-54.715470	65.380330	296.876000	.316586
-52.408880	65.162570	296.946500	.125984
-50.146370	64.910470	296.976200	.041388
-47.932120	64.625290	296.985500	.011647
-45.769150	64.308400	296.987900	.002910
-43.659080	63.961160	296.988500	.000557
-41.602020	63.584970	296.988500	.000175
-39.596350	63.181100	296.988600	.000155
-37.638600	62.750720	296.988500	.000183
-35.723270	62.294770	296.988500	.000183
-33.842640	61.813940	296.988500	.000183
-31.986660	61.308540	296.988500	.000183
-30.142740	60.778430	296.988500	.000183
-28.295590	60.222970	296.988500	.000183
-26.427080	59.640870	296.988500	.000183
-108.736700	60.768890	296.988500	.000555
-106.789100	61.359350	296.988200	.001674
-104.866900	61.921510	296.987200	.006259
-102.949500	62.456830	296.983200	.021339
-101.019200	62.966030	296.968500	.066411
-99.061880	63.449270	296.919000	.191911
-97.066180	63.906190	296.770800	.507450
-95.023590	64.336020	296.402100	1.166249
-92.928180	64.737730	295.640600	2.137413
-90.776390	65.110020	294.439900	3.590249
-88.566850	65.451470	296.032700	1.656109
-86.300130	65.760610	302.214800	4.575400
-83.978550	66.035940	305.855100	10.690240
-81.606060	66.276030	302.597800	6.883264
-79.187900	66.479550	300.284500	5.368982
-76.730480	66.645290	299.145600	4.336560
-74.241170	66.772270	299.023500	4.393033
-71.728080	66.859700	299.663500	5.212827
-69.199870	66.907030	300.285600	5.613718
-66.665550	66.913940	299.287000	4.251384
-64.134220	66.880400	298.085900	2.299125
-61.614990	66.806620	297.306700	.882047
-59.116610	66.693070	296.996200	.313202
-56.647430	66.540450	296.935300	.200802
-54.215080	66.349720	296.952300	.117438
-51.826330	66.121990	296.972500	.055064
-49.486860	65.858530	296.982700	.022364
-47.201050	65.560780	296.986600	.008471
-44.971820	65.230190	296.988000	.002810
-42.800360	64.868270	296.988400	.000779
-40.685980	64.476460	296.988500	.000228
-38.625890	64.056100	296.988500	.000181
-36.614990	63.608330	296.988500	.000181

-34.645670	63.134020	296.988500	.000183
-32.707610	62.633670	296.988500	.000183
-30.787590	62.107330	296.988500	.000183
-28.869240	61.554470	296.988500	.000183
-26.932890	60.973880	296.988500	.000183
-24.955330	60.363580	296.988500	.000183

Q.14a

-89.453400	32.991660	313.391800	1.324609
-88.345930	33.191160	313.542100	1.651884
-87.228100	33.381660	313.980200	2.578954
-86.100230	33.562890	314.583500	3.806782
-84.962690	33.734530	315.104900	4.862069
-83.815900	33.896310	315.312100	5.356372
-82.660300	34.047950	315.213200	5.416391
-81.496410	34.189190	315.200200	5.826016
-80.324730	34.319790	315.756900	7.394931
-79.145840	34.439500	316.931200	10.277750
-77.960310	34.548100	318.026900	13.247140
-76.768760	34.645400	318.417300	15.187960
-75.571840	34.731200	318.125900	15.367950
-74.370180	34.805370	317.948600	14.008770
-73.164480	34.867740	317.201800	11.468410
-71.955430	34.918200	316.332200	8.710141
-70.743730	34.956650	315.461000	6.446598
-69.530080	34.983020	314.874900	4.877115
-68.315220	34.997260	314.423100	3.744842
-67.099880	34.999340	314.011800	2.778614
-65.884770	34.989250	313.624500	1.889423
-64.670640	34.967020	313.294600	1.124129
-63.458190	34.932690	313.070700	.571769
-62.248150	34.886320	312.948500	.249852
-61.041230	34.828000	312.893000	.094091
-59.838120	34.757850	312.871900	.030920
-58.639500	34.676010	312.864800	.008941
-57.446040	34.582610	312.862800	.002404
-56.258390	34.477850	312.862200	.000675
-55.077160	34.361910	312.862100	.000246
-53.902950	34.235010	312.862100	.000177
-52.736340	34.097380	312.862100	.000149
-51.577860	33.949260	312.862100	.000153
-50.428010	33.790910	312.862100	.000153
-49.287250	33.622600	312.862100	.000153
-48.156040	33.444620	312.862100	.000153
-47.034740	33.257250	312.862100	.000153

-45.923690	33.060790	312.862100	.000153
-44.823220	32.855560	312.862100	.000153
-89.701210	33.918400	313.870400	2.300035
-88.583520	34.125020	314.284300	3.120034
-87.455040	34.322360	315.381600	5.175815
-86.316120	34.510110	316.948800	7.974390
-85.167100	34.687960	318.342900	10.420560
-84.008420	34.855620	318.822100	11.449710
-82.840550	35.012790	318.358400	11.205450
-81.663990	35.159200	317.841500	11.197070
-80.479300	35.294580	318.853400	13.658990
-79.287040	35.418700	321.548800	18.481350
-78.087840	35.531310	324.062000	23.422480
-76.882320	35.632210	324.684800	26.069110
-75.671150	35.721200	324.503000	26.716490
-74.455030	35.798110	323.609600	24.409960
-73.234660	35.862810	323.015200	21.381610
-72.010770	35.915150	320.773300	16.462660
-70.784100	35.955040	319.037600	12.782150
-69.555380	35.982390	317.644700	9.954201
-68.325390	35.997170	316.646900	7.864551
-67.094890	35.999320	315.688100	5.961264
-65.864640	35.988860	314.718200	4.077874
-64.635410	35.965800	313.891900	2.403070
-63.407950	35.930180	313.342700	1.208548
-62.183030	35.882080	313.053700	.516209
-60.961370	35.821590	312.929800	.194039
-59.743720	35.748840	312.883500	.064873
-58.530780	35.663950	312.868400	.020040
-57.323260	35.567100	312.863900	.006076
-56.121820	35.458470	312.862700	.002034
-54.927120	35.338260	312.862300	.000788
-53.739770	35.206700	312.862100	.000334
-52.560380	35.064020	312.862100	.000188
-51.389480	34.910500	312.862100	.000147
-50.227610	34.746390	312.862100	.000153
-49.075240	34.571990	312.862100	.000153
-47.932830	34.387580	312.862100	.000153
-46.800740	34.193490	312.862100	.000153
-45.679330	33.990000	312.862100	.000153
-44.568900	33.777440	312.862100	.000153
-89.960660	34.843320	315.354900	5.100672
-88.832330	35.057210	316.680000	7.410683
-87.692770	35.261540	320.153000	13.069100
-86.542320	35.455950	325.340800	21.026540
-85.381340	35.640140	330.106200	28.145920
-84.210270	35.813810	331.609300	30.859970
-83.029580	35.976620	329.351100	28.799190
-81.839790	36.128310	326.196200	26.626810
-80.641480	36.268600	328.213600	30.784770
-79.435230	36.397220	336.290200	42.060280
-78.221690	36.513940	346.432500	51.671730
-77.001530	36.618520	339.917000	56.823080
-75.775430	36.710780	340.327100	56.815090
-74.544130	36.790520	348.284100	59.876900
-73.308360	36.857590	341.703100	49.255600
-72.068890	36.911860	336.605600	41.841830
-70.826490	36.953220	330.866900	31.520900
-69.581950	36.981590	327.428100	25.670410
-68.336070	36.996900	324.550900	20.779850
-67.089650	36.999130	321.669900	16.009140
-65.843500	36.988290	318.657300	11.089590
-64.598400	36.964370	315.981300	6.468674
-63.355190	36.927440	314.274300	3.193269
-62.114630	36.877570	313.416700	1.372761
-60.877510	36.814860	313.055000	.515545
-59.644590	36.739430	312.925600	.181126

-58.416640	36.651430	312.882500	.061649
-57.194370	36.551040	312.869200	.022223
-55.978490	36.438440	312.864900	.009133
-54.769680	36.313860	312.863300	.004185
-53.568590	36.177530	312.862600	.001894
-52.375820	36.029710	312.862300	.000776
-51.191970	35.870660	312.862100	.000301
-50.017550	35.700660	312.862100	.000177
-48.853080	35.520030	312.862100	.000153
-47.698980	35.329070	312.862100	.000153
-46.555660	35.128100	312.862100	.000153
-45.423470	34.917440	312.862100	.000153
-44.302690	34.697410	312.862100	.000153
-90.232250	35.766320	318.513600	10.422620
-89.092860	35.987640	322.433700	16.310410
-87.941790	36.199090	332.669600	30.708400
-86.779330	36.400320	349.498200	52.556240
-85.605870	36.591000	365.755100	72.679290
-84.421870	36.770790	370.006900	78.755740
-83.227800	36.939400	360.819300	70.075580
-82.024180	37.096500	344.998200	57.922520
-80.811620	37.241800	348.535400	65.019170
-79.590740	37.375040	383.565200	95.207710
-78.362190	37.495960	385.215100	105.337400
-77.126670	37.604320	366.764300	109.389900
-75.884930	37.699920	363.434100	110.477300
-74.637700	37.782550	390.886900	113.277600
-73.385770	37.852060	411.533400	123.502800
-72.129940	37.908310	379.878900	95.128900
-70.871030	37.951180	364.245900	77.902460
-69.609860	37.980580	354.538100	62.038670
-68.347290	37.996460	347.738300	52.297080
-67.084140	37.998780	339.822200	41.786830
-65.821270	37.987530	330.436500	29.204920
-64.559530	37.962740	321.676400	16.189840
-63.299760	37.924460	316.664300	7.793643
-62.042780	37.872770	314.310900	3.278354
-60.789440	37.807780	313.374400	1.272797
-59.540510	37.729610	313.035400	.465820
-58.296810	37.638420	312.925100	.179914
-57.059080	37.534390	312.888200	.077775
-55.828080	37.417740	312.874700	.038572
-54.604500	37.288680	312.868500	.020242
-53.389020	37.147460	312.865200	.010103
-52.182290	36.994370	312.863400	.004468
-50.984880	36.829660	312.862500	.001669
-49.797380	36.653650	312.862200	.000519
-48.620270	36.466650	312.862100	.000188
-47.454020	36.268990	312.862100	.000151
-46.299010	36.060990	312.862100	.000153
-45.155610	35.843010	312.862100	.000153
-44.024070	35.615370	312.862100	.000153
-90.516530	36.687310	323.936900	18.255320
-89.365660	36.916220	333.565500	30.760260
-88.202590	37.134950	360.746400	63.371820
-87.027650	37.343150	415.028600	120.005400
-85.841200	37.540460	470.735200	173.646400
-84.643710	37.726540	483.151900	188.698700
-83.435650	37.901050	448.126200	159.733900
-82.217600	38.063690	377.656000	118.182000
-80.990140	38.214140	389.583000	128.062800
-79.753940	38.352110	466.263100	181.437200
-78.509670	38.477340	469.335900	197.253200
-77.258070	38.589580	364.029000	154.263600
-75.999910	38.688600	324.080100	127.038200
-74.735980	38.774210	517.780300	249.019700
-73.467090	38.846230	509.130400	193.320900

-72.194090	38.904510	511.704500	207.350100
-70.917830	38.948930	451.049400	163.112400
-69.639210	38.979390	429.452200	141.386900
-68.359090	38.995840	414.271600	122.671100
-67.078360	38.998240	391.163600	98.112740
-65.797930	38.986590	364.297000	71.193480
-64.518690	38.960900	336.548900	38.422060
-63.241520	38.921240	321.783800	16.561460
-61.967320	38.867690	316.166100	6.921371
-60.696920	38.800350	314.049000	2.729768
-59.431210	38.719360	313.306300	1.112707
-58.170970	38.624900	313.046300	.494647
-56.917050	38.517150	312.954100	.258199
-55.670200	38.396340	312.913800	.149131
-54.431160	38.262690	312.891200	.086505
-53.200640	38.116470	312.877300	.046341
-51.979310	37.957960	312.869000	.021802
-50.767770	37.787460	312.864800	.008756
-49.566610	37.605300	312.862900	.002942
-48.376340	37.411790	312.862300	.000821
-47.197410	37.207270	312.862100	.000235
-46.030250	36.992090	312.862100	.000150
-44.875180	36.766620	312.862100	.000153
-43.732470	36.531200	312.862100	.000153
-90.814130	37.606180	330.009200	25.692850
-89.651300	37.842850	347.914100	46.105170
-88.475770	38.069030	413.440000	110.351800
-87.287830	38.284350	583.626800	240.777400
-86.087840	38.488450	767.929500	371.222600
-84.876290	38.680970	816.428300	415.903700
-83.653650	38.861550	701.894600	352.677400
-82.420500	39.029860	475.300500	253.266300
-81.177470	39.185590	383.513200	205.118600
-79.925230	39.328410	586.549400	302.670300
-78.664510	39.458070	625.074600	313.699800
-77.396060	39.574290	457.072400	247.523900
-76.120680	39.676830	315.446400	292.817900
-74.839220	39.765490	459.003400	249.419700
-73.552520	39.840080	779.173000	509.337900
-72.261480	39.900440	661.615700	283.925700
-70.967010	39.946460	666.839800	302.814100
-69.670030	39.978020	624.761200	275.855500
-68.371470	39.995060	584.860400	243.436500
-67.072270	39.997550	511.969800	194.748300
-65.773400	39.985470	431.547000	135.738300
-64.475770	39.958870	367.768000	75.165120
-63.180340	39.917780	331.778700	31.920750
-61.888020	39.862300	319.276700	12.443480
-60.599740	39.792560	315.370400	5.364412
-59.316390	39.708680	313.929600	2.462919
-58.038840	39.610860	313.398600	1.324236
-56.767930	39.499290	313.176500	.812630
-55.504470	39.374200	313.058800	.525791
-54.249260	39.235840	312.980100	.326986
-53.003000	39.084480	312.925800	.184239
-51.766410	38.920440	312.892400	.091060
-50.540120	38.744010	312.874500	.038702
-49.324710	38.555530	312.866500	.013986
-48.120720	38.355340	312.863400	.004245
-46.928620	38.143810	312.862400	.001074
-45.748810	37.921300	312.862100	.000258
-44.581620	37.688170	312.862100	.000149
-43.427300	37.444810	312.862100	.000153
-91.125630	38.522790	329.256100	27.602170
-89.950410	38.767390	351.019300	51.806950
-88.761920	39.001200	484.512800	151.148600
-87.560450	39.223820	899.033800	369.382600

-86.346370	39.434880	1406.703000	643.432600
-85.120160	39.633990	1586.001000	761.278800
-83.882300	39.820790	1384.636000	686.030200
-82.633390	39.994930	828.216500	483.225700
-81.374080	40.156070	385.327300	362.217300
-80.105060	40.303890	687.332300	408.808200
-78.827100	40.438090	1096.590000	524.461500
-77.540980	40.558400	1014.254000	444.290600
-76.247540	40.664560	632.367400	456.766600
-74.947690	40.756360	492.176600	640.550800
-73.642300	40.833600	768.361000	754.598400
-72.332310	40.896110	788.119600	465.235900
-71.018690	40.943760	945.950700	419.367400
-69.702430	40.976440	955.375200	418.600800
-68.384490	40.994090	843.210100	356.659800
-67.065880	40.996670	635.262600	238.756900
-65.747600	40.984170	468.975800	164.757100
-64.430650	40.956610	384.560000	96.263940
-63.116020	40.914060	341.522800	44.799070
-61.804690	40.856610	324.136100	20.130200
-60.497620	40.784390	317.961900	9.778084
-59.195770	40.697540	315.474400	5.436514
-57.900040	40.596260	314.408100	3.438619
-56.611330	40.480750	313.902700	2.418511
-55.330490	40.351270	313.562700	1.695555
-54.058330	40.208070	313.296800	1.100976
-52.795620	40.051450	313.102500	.641338
-51.543080	39.881710	312.978200	.327912
-50.301390	39.699200	312.911200	.145493
-49.071130	39.504250	312.880100	.055430
-47.852850	39.297230	312.867700	.017987
-46.647030	39.078520	312.863600	.004908
-45.454070	38.848490	312.862400	.001126
-44.274280	38.607530	312.862100	.000252
-43.107920	38.356030	312.862100	.000176
-91.451740	39.437030	318.284100	28.208230
-90.263630	39.689750	329.493700	84.069350
-89.061680	39.931370	481.163700	157.835400
-87.846150	40.161480	1067.736000	384.406100
-86.617400	40.379670	1962.291000	775.436900
-85.375900	40.585540	2421.540000	1006.111000
-84.122160	40.778730	2288.292000	964.165500
-82.856800	40.958850	1493.171000	634.890700
-81.580460	41.125550	713.904400	435.912800
-80.293880	41.278500	1017.173000	527.097200
-78.997860	41.417370	1717.820000	725.079200
-77.693230	41.541890	1933.972000	762.465900
-76.380870	41.651780	1433.351000	590.277700
-75.061690	41.746810	738.649700	361.460600
-73.736660	41.826770	457.702000	416.792400
-72.406780	41.891490	624.447100	426.945400
-71.073040	41.940830	1079.529000	528.652300
-69.736500	41.974670	1117.866000	502.536100
-68.398190	41.992950	836.172100	337.124500
-67.059160	41.995620	523.113500	203.161800
-65.720480	41.982670	326.660900	120.361500
-64.383220	41.954140	330.757700	77.152000
-63.048400	41.910080	338.686300	50.060870
-61.717090	41.850600	331.934600	30.406150
-60.390290	41.775820	324.174700	18.571350
-59.069010	41.685920	319.380200	11.844660
-57.754210	41.581080	317.302100	8.717251
-56.446830	41.461530	316.159400	6.760399
-55.147770	41.327530	315.202100	5.019192
-53.857880	41.179360	314.341300	3.355533
-52.577970	41.017320	313.681300	1.979719
-51.308780	40.841740	313.260800	1.031079

-50.051000 40.652980 313.031600 .469577
-48.805250 40.451390 312.925600 .185951
-47.572090 40.237360 312.882900 .063486
-46.352010 40.011290 312.867900 .018557
-45.145370 39.773560 312.863500 .004584
-43.952510 39.524580 312.862400 .000966
-42.773630 39.264750 312.862100 .000282
-91.793200 40.348750 329.172100 41.618170
-90.591710 40.609810 305.935000 63.243860
-89.375760 40.859430 314.077600 204.507200
-88.145600 41.097210 687.583100 266.907300
-86.901570 41.322720 1397.334000 517.567600
-85.644150 41.535550 1905.707000 778.649500
-84.373860 41.735290 1969.960000 791.909700
-83.091290 41.921560 1431.876000 488.525000
-81.797160 42.093980 1001.418000 319.942400
-80.492230 42.252200 1578.656000 548.101700
-79.177290 42.395880 2345.402000 931.076700
-77.853240 42.524730 2556.695000 1033.516000
-76.521000 42.638450 2212.635000 845.212600
-75.181550 42.736820 1531.495000 554.715300
-73.835900 42.819590 1120.649000 525.434100
-72.485100 42.886590 1125.629000 640.903900
-71.130210 42.937670 1380.507000 702.263200
-69.772340 42.972710 1068.571000 575.656000
-68.412590 42.991630 604.843600 373.751100
-67.052090 42.994400 282.828900 201.041800
-65.691960 42.980990 233.263100 144.600000
-64.333320 42.951450 342.373700 93.914350
-62.977290 42.905830 392.052800 86.247700
-61.624970 42.844260 366.771200 60.774010
-60.277440 42.766850 340.951600 37.611600
-58.935750 42.673790 328.805800 25.351160
-57.600940 42.565280 325.203800 20.979630
-56.273990 42.441570 322.855500 17.792700
-54.955840 42.302930 320.194900 13.709100
-53.647390 42.149640 317.528100 9.305552
-52.349470 41.982030 315.427200 5.520232
-51.062890 41.800450 314.091700 2.868167
-49.788320 41.605260 313.383800 1.319086
-48.526440 41.396860 313.057000 .532994
-47.277780 41.175640 312.926800 .188376
-46.042850 40.942000 312.881000 .057747
-44.822010 40.696380 312.866900 .015235
-43.615560 40.439180 312.863100 .003459
-42.423690 40.170830 312.862300 .000988
-92.150770 41.257820 365.803300 50.265030
-90.935390 41.527410 401.719300 84.172420
-89.704910 41.785250 384.257400 142.544900
-88.459540 42.030900 342.970900 254.360500
-87.199620 42.263930 540.807700 253.582900
-85.925610 42.483890 749.954000 384.170300
-84.638040 42.690380 803.336400 419.729100
-83.337520 42.882970 697.762900 333.236400
-82.024790 43.061280 1165.794000 320.398800
-80.700610 43.224930 2362.406000 811.338000
-79.365870 43.373580 3180.136000 1260.760000
-78.021450 43.506890 3249.127000 1318.429000
-76.668370 43.624570 2887.970000 1091.200000
-75.307630 43.726360 2398.340000 835.981000
-73.940300 43.812040 2019.493000 737.085100
-72.567500 43.881390 1788.839000 778.674700
-71.190370 43.934270 1669.813000 772.967500
-69.810050 43.970550 1331.365000 622.011800
-68.427750 43.990140 851.167100 423.060600
-67.044650 43.993000 617.528400 298.497400
-65.661930 43.979120 550.109300 230.664400

-64.280810	43.948540	617.339000	205.047300
-62.902460	43.901310	566.332800	164.034900
-61.528050	43.837570	451.168900	105.595800
-60.158730	43.757450	377.389600	67.710450
-58.795620	43.661140	347.456900	48.749180
-57.439790	43.548850	347.113200	48.440860
-56.092310	43.420850	342.146400	44.223640
-54.754150	43.277410	334.403900	34.800610
-53.426270	43.118850	326.503300	23.610950
-52.109530	42.945500	320.222700	13.888690
-50.804760	42.757750	316.315600	7.169335
-49.512690	42.555960	314.289000	3.258106
-48.233980	42.340550	313.390900	1.321691
-46.969190	42.111940	313.036400	.473980
-45.718810	41.870550	312.913600	.149734
-44.483210	41.616830	312.875600	.041273
-43.262650	41.351200	312.865200	.009983
-42.057300	41.074100	312.863000	.002971
-92.525320	42.164070	390.957300	77.170410
-91.295520	42.442420	451.649700	86.201820
-90.049940	42.708690	544.131400	144.528700
-88.788760	42.962440	582.376400	254.614600
-87.512310	43.203190	714.390000	269.176100
-86.221000	43.430500	848.316100	325.536300
-84.915410	43.643920	974.853600	486.873000
-83.596150	43.843030	1130.919000	443.562300
-82.263960	44.027400	2049.681000	694.816700
-80.919660	44.196660	3432.712000	1221.215000
-79.564150	44.350410	4003.093000	1542.543000
-78.198390	44.488330	3605.549000	1444.728000
-76.823410	44.610100	2764.287000	1100.767000
-75.440290	44.715440	1999.409000	748.946500
-74.050180	44.804110	1576.282000	548.788700
-72.654240	44.875890	1342.835000	543.172100
-71.253690	44.930630	1452.744000	595.513200
-69.849760	44.968180	1413.903000	580.029200
-68.443720	44.988460	1175.378000	497.549000
-67.036810	44.991420	998.338500	401.743600
-65.630330	44.977050	904.003700	329.420500
-64.225530	44.945390	876.721200	277.262400
-62.823690	44.896510	729.763500	196.382900
-61.426040	44.830540	529.456300	116.303700
-60.033810	44.747610	415.640800	84.479900
-58.648170	44.647940	379.453100	84.414380
-57.270290	44.531750	406.309500	102.184800
-55.901260	44.399320	400.427900	104.201000
-54.542130	44.250930	374.095900	82.655620
-53.193900	44.086940	349.851100	55.095510
-51.857480	43.907680	332.139500	31.648790
-50.533710	43.713560	321.592300	15.959270
-49.223370	43.504980	316.360500	7.148768
-47.927120	43.282370	314.114600	2.845658
-46.645540	43.046150	313.270200	1.021263
-45.379100	42.796800	312.981400	.325586
-44.128160	42.534760	312.893600	.092091
-42.892960	42.260480	312.869600	.023171
-41.673610	41.974410	312.864300	.007106
-92.917770	43.067330	404.181500	98.616890
-91.673000	43.354680	484.933600	129.593800
-90.411740	43.629630	696.552900	182.639200
-89.134110	43.891690	1035.731000	273.625000
-87.840450	44.140390	1428.815000	404.678100
-86.531140	44.375260	1710.061000	583.613200
-85.206750	44.595830	1904.335000	692.758100
-83.867900	44.801650	2156.998000	810.733000
-82.515380	44.992280	2855.318000	1107.650000
-81.150000	45.167310	3718.870000	1496.002000

-79.772730	45.326340	3908.936000	1608.010000
-78.384560	45.469020	3379.990000	1382.824000
-76.986600	45.595010	2461.853000	944.117900
-75.579970	45.704020	1624.166000	515.230600
-74.165890	45.795780	1134.355000	305.090900
-72.745610	45.870080	942.640400	324.647600
-71.320400	45.926740	1132.517000	365.531900
-69.891600	45.965610	1288.047000	474.526100
-68.460530	45.986610	1185.894000	509.740500
-67.028560	45.989670	1069.001000	469.663400
-65.597030	45.974800	905.147600	388.493800
-64.167300	45.942020	817.525600	274.606100
-62.740720	45.891430	657.541600	167.830800
-61.318600	45.823140	480.123400	108.995000
-59.902260	45.737310	414.013400	106.727100
-58.492950	45.634170	441.102900	140.195900
-57.091900	45.513950	535.264400	189.281600
-55.700260	45.376930	551.789900	211.087800
-54.319140	45.223450	488.071800	176.213700
-52.949590	45.053840	410.533500	117.242800
-51.592580	44.868490	359.058800	64.872090
-50.248970	44.667810	332.587200	31.713050
-48.919560	44.452230	320.365900	13.686530
-47.605040	44.222190	315.452300	5.335564
-46.305980	43.978160	313.673700	1.870060
-45.022840	43.720610	313.096600	.596510
-43.755960	43.450020	312.923300	.169427
-42.505550	43.166850	312.876800	.043554
-41.271660	42.871580	312.866400	.013588
-93.329110	43.967430	421.276200	121.907900
-92.068780	44.264030	508.928400	162.847400
-90.791210	44.547890	754.005900	265.014500
-89.496510	44.818510	1183.839000	408.615100
-88.184940	45.075390	1651.207000	582.825000
-86.856880	45.318050	1932.167000	730.490100
-85.512890	45.545990	2231.401000	893.958000
-84.153590	45.758730	2642.606000	1048.974000
-82.779790	45.955820	3134.551000	1291.197000
-81.392350	46.136820	3468.039000	1481.210000
-79.992260	46.301310	3382.595000	1453.782000
-78.580580	46.448910	2875.424000	1187.570000
-77.158460	46.579270	2223.011000	799.633000
-75.727110	46.692070	1669.098000	484.191900
-74.287820	46.787040	1319.774000	332.937800
-72.841890	46.863950	1101.151000	300.656200
-71.390720	46.922590	1131.900000	344.494000
-69.935700	46.962830	1165.453000	384.712300
-68.478260	46.984570	1241.443000	467.766100
-67.019850	46.987740	1291.162000	501.828800
-65.561920	46.972340	1128.561000	472.197900
-64.105920	46.938410	931.994700	346.515200
-62.653260	46.886040	705.692400	224.329100
-61.205390	46.815350	528.959400	169.620900
-59.763680	46.726520	504.292500	183.946300
-58.329470	46.619790	587.548600	237.662400
-56.904060	46.495390	753.755900	306.307200
-55.488670	46.353640	806.413300	339.129900
-54.084500	46.194880	718.616000	295.717900
-52.692630	46.019470	549.892400	206.287600
-51.314070	45.827840	415.074100	114.761500
-49.949730	45.620390	351.906600	54.103740
-48.600430	45.397590	326.746300	22.787280
-47.266860	45.159900	317.339900	8.430407
-45.949600	44.907820	314.217000	2.892825
-44.649100	44.641840	313.239000	.900141
-43.365670	44.362450	312.959700	.256501
-42.099450	44.070140	312.885200	.065926

-40.850440 43.765410 312.869000 .020669
-93.760410 44.864160 431.559400 135.005100
-92.483920 45.170280 510.338000 180.247200
-91.189420 45.463320 711.490800 289.475600
-89.876960 45.742750 1065.956000 440.645400
-88.546760 46.008070 1445.835000 585.622900
-87.199170 46.258760 1704.797000 717.112200
-85.834720 46.494300 2052.289000 886.720200
-84.454070 46.714200 2566.814000 1085.454000
-83.058010 46.917960 2986.958000 1270.227000
-81.647480 47.105130 3100.432000 1345.012000
-80.223460 47.275260 2775.751000 1188.721000
-78.787090 47.427960 2114.018000 874.042000
-77.339580 47.562840 1538.550000 571.785300
-75.882230 47.679570 1337.669000 437.740000
-74.416370 47.777870 1373.740000 426.247700
-72.943440 47.857470 1349.040000 418.009900
-71.464880 47.918190 1237.109000 371.997700
-69.982220 47.959850 1116.651000 360.393200
-68.496960 47.982350 1209.299000 377.343100
-67.010670 47.985630 1476.523000 495.929700
-65.524890 47.969690 1485.268000 521.925400
-64.041170 47.934570 1338.209000 451.943500
-62.561040 47.880340 1102.839000 345.253400
-61.086020 47.807170 930.455600 290.083600
-59.617590 47.715230 878.336300 313.123700
-58.157170 47.604770 907.334000 359.189400
-56.706150 47.476050 979.675500 413.302500
-55.265830 47.329400 1021.517000 429.741000
-53.837470 47.165180 929.070100 373.476300
-52.422200 46.983780 698.464800 266.852900
-51.021090 46.785640 483.910500 152.794800
-49.635090 46.571200 373.847400 73.140960
-48.265020 46.340950 333.202100 30.098340
-46.911610 46.095370 319.108300 11.016330
-45.575410 45.834990 314.625900 3.567485
-44.256860 45.560320 313.343900 1.102934
-42.956210 45.271870 312.983200 .307583
-41.673580 44.970160 312.890800 .079493
-40.408870 44.655690 312.870400 .024650
-94.212860 45.757310 443.204800 146.069800
-92.919560 46.073230 516.366600 187.820700
-91.607470 46.375730 681.682700 291.455400
-90.276550 46.664250 958.695000 416.383800
-88.926960 46.938270 1263.956000 528.654700
-87.559010 47.197250 1506.234000 649.787700
-86.173240 47.440650 1813.668000 822.460300
-84.770290 47.667940 2235.077000 1020.913000
-83.350960 47.878600 2605.537000 1171.971000
-81.916200 48.072160 2676.694000 1163.249000
-80.467090 48.248140 2267.159000 952.393100
-79.004780 48.406110 1474.853000 634.714700
-77.530590 48.545680 935.011500 399.044900
-76.045850 48.666500 886.948500 317.298100
-74.552020 48.768250 1119.942000 366.724200
-73.050600 48.850660 1246.465000 423.030900
-71.543170 48.913520 1187.055000 396.564300
-70.031330 48.956650 995.680800 320.519100
-68.516710 48.979950 975.706100 334.237900
-67.000980 48.983350 1185.411000 398.892600
-65.485790 48.966840 1405.150000 498.277000
-63.972820 48.930470 1412.078000 472.415600
-62.463700 48.874330 1344.448000 414.922500
-60.960050 48.798580 1323.230000 403.275900
-59.463460 48.703410 1334.437000 426.462700
-57.975440 48.589080 1240.255000 442.863600
-56.497490 48.455880 1089.691000 450.392500

-55.030970	48.304140	979.619400	420.763500
-53.577210	48.134270	871.441100	354.612200
-52.137430	47.946660	665.624900	253.446600
-50.712720	47.741790	468.597500	145.678300
-49.304080	47.520120	369.018900	69.373900
-47.912350	47.282170	331.603400	28.965570
-46.538250	47.028450	318.724200	10.314420
-45.182340	46.759510	314.539600	3.403494
-43.845020	46.475880	313.305700	1.016879
-42.526500	46.178100	312.975200	.288870
-41.226800	45.866700	312.888300	.073219
-39.945740	45.542200	312.869700	.022696
-94.687740	46.646660	462.849600	162.706100
-93.376940	46.972690	535.159100	203.161400
-92.046550	47.284930	657.349200	290.832200
-90.696440	47.582840	839.365200	389.839800
-89.326680	47.865840	1070.402000	477.209500
-87.937550	48.133390	1334.950000	585.688700
-86.529520	48.384910	1649.387000	735.099200
-85.103260	48.619850	1994.505000	897.814900
-83.659580	48.837660	2285.659000	1006.695000
-82.199460	49.037830	2330.221000	981.229100
-80.724000	49.219880	1922.367000	821.035200
-79.234450	49.383340	1301.568000	576.207100
-77.732160	49.527770	923.697800	364.080400
-76.218590	49.652830	880.639600	293.804100
-74.695260	49.758160	979.300800	326.736400
-73.163800	49.843490	1012.210000	367.761100
-71.625870	49.908580	1016.621000	354.576800
-70.083210	49.953250	908.035200	293.897900
-68.537570	49.977380	733.292100	262.409300
-66.990740	49.980900	781.811400	285.859800
-65.444490	49.963800	987.714400	350.973800
-63.900620	49.926140	995.542500	355.196900
-62.360880	49.868010	937.272500	336.789000
-60.827010	49.789570	1097.217000	399.297100
-59.300730	49.691040	1224.825000	443.996000
-57.783640	49.572690	1150.726000	425.104900
-56.277320	49.434830	900.455100	380.359200
-54.783270	49.277820	723.825400	321.102000
-53.302860	49.102080	622.972200	254.938200
-51.837360	48.908040	503.155200	176.083800
-50.387950	48.696200	398.172000	99.242150
-48.955640	48.467040	344.835300	47.456580
-47.541310	48.221130	324.104600	19.478580
-46.145660	47.959000	316.337000	7.084948
-44.769230	47.681210	313.865600	2.283522
-43.412380	47.388330	313.153700	.725363
-42.075270	47.080920	312.937200	.204890
-40.757810	46.759540	312.879800	.052355
-39.459720	46.424710	312.867200	.015848
-95.186440	47.531940	502.443500	191.967500
-93.857410	47.868410	578.731800	227.817500
-92.508000	48.190720	655.548500	294.408700
-91.137910	48.498310	734.239500	355.564800
-89.747150	48.790600	884.564200	414.671300
-88.335950	49.067010	1149.031000	501.893100
-86.904710	49.326930	1473.193000	620.646900
-85.454100	49.569790	1737.814000	750.036500
-83.984930	49.795020	1904.939000	826.257300
-82.498220	50.002060	1815.069000	818.977500
-80.995120	50.190400	1498.730000	717.496900
-79.476920	50.359550	1166.450000	552.348800
-77.945060	50.509060	1031.597000	407.194000
-76.401090	50.638520	982.851600	342.909000
-74.846650	50.747580	944.418000	324.511200
-73.283450	50.835950	898.811600	336.269200

-71.713320	50.903350	922.766400	336.012500
-70.138080	50.949630	907.739700	292.333700
-68.559640	50.974620	696.354300	221.200600
-66.979900	50.978270	585.100500	223.001800
-65.400800	50.960560	720.856200	249.028600
-63.824260	50.921550	601.538900	233.432100
-62.252170	50.861340	487.493700	232.824500
-60.686400	50.780110	656.668200	291.474600
-59.128770	50.678080	845.191200	357.663500
-57.581030	50.555550	796.350400	331.031800
-56.044860	50.412850	621.336500	263.186000
-54.521820	50.250360	497.136000	197.624100
-53.013420	50.068520	439.343600	143.787800
-51.520960	49.867810	390.034100	95.346840
-50.045680	49.648740	349.399300	53.174120
-48.588630	49.411830	328.117600	25.604810
-47.150700	49.157670	318.342000	10.850060
-45.732590	48.886830	314.609700	3.921288
-44.334810	48.599900	313.418700	1.369406
-42.957660	48.297460	313.021300	.431636
-41.601190	47.980120	312.903300	.121019
-40.265250	47.648430	312.871300	.029475
-38.949390	47.302930	312.864700	.008656
-95.710510	48.412890	560.737100	223.827000
-94.362470	48.760130	639.538700	250.686700
-92.993230	49.092850	685.203400	285.068300
-91.602360	49.410460	692.449800	300.542300
-90.189750	49.712350	751.532500	327.090300
-88.755540	49.997940	917.669100	390.009500
-87.300090	50.266570	1131.549000	487.863800
-85.824040	50.517650	1307.006000	590.065400
-84.328210	50.750570	1398.378000	646.911900
-82.813610	50.964740	1305.735000	647.588300
-81.281460	51.159630	1095.693000	572.523200
-79.733130	51.334700	956.921100	461.053600
-78.170130	51.489480	918.344200	376.412800
-76.594090	51.623530	907.867200	326.303600
-75.006800	51.736490	883.484900	304.950500
-73.410070	51.828010	862.972700	307.751400
-71.805870	51.897850	906.441200	331.112500
-70.196170	51.945790	951.910200	312.651300
-68.582990	51.971680	816.219800	241.282900
-66.968440	51.975460	676.853800	227.978000
-65.354560	51.957120	740.239700	228.826700
-63.743440	51.916690	603.863800	185.507400
-62.137130	51.854320	418.370200	159.652600
-60.537630	51.770170	499.156200	200.834700
-58.946900	51.664510	564.069400	243.159600
-57.366820	51.537620	522.564900	207.153500
-55.799180	51.389870	435.659300	149.310900
-54.245650	51.221680	386.652800	101.004600
-52.707820	51.033510	360.980900	69.428750
-51.187080	50.825860	341.941400	44.503170
-49.684720	50.599280	327.726600	24.922810
-48.201800	50.354330	319.434400	12.428960
-46.739240	50.091630	315.322600	5.267638
-45.297720	49.811770	313.713600	2.038189
-43.877700	49.515370	313.127900	.708016
-42.479410	49.203060	312.937500	.219505
-41.102810	48.875440	312.880400	.058352
-39.747590	48.533100	312.866000	.013567
-38.413150	48.176570	312.863000	.003777
-96.261670	49.289190	599.710400	226.235500
-94.893750	49.647600	673.933000	242.032500
-93.503820	49.991080	683.517600	244.397900
-92.091320	50.319060	644.725800	231.494900
-90.655950	50.630910	627.320100	232.881900

-89.197760	50.925990	667.370900	272.903800
-87.717060	51.203660	757.348100	341.917200
-86.214420	51.463270	875.462900	419.889400
-84.690660	51.704190	1019.764000	464.883500
-83.146820	51.925780	1030.072000	464.122200
-81.584160	52.127480	932.957800	407.885300
-80.004110	52.308710	825.917400	337.878000
-78.408280	52.468990	771.034200	290.334400
-76.798410	52.607840	792.127100	264.697500
-75.176390	52.724850	835.570100	251.634000
-73.544200	52.819690	851.089000	250.520100
-71.903920	52.892050	869.593600	281.770400
-70.257710	52.941730	931.829500	295.855100
-68.607750	52.968570	904.204500	261.852400
-66.956280	52.972490	782.925800	237.727800
-65.305560	52.953480	773.354600	237.478200
-63.657820	52.911580	637.321700	192.134700
-62.015270	52.846950	479.852300	133.014000
-60.380080	52.759750	446.202400	142.977600
-58.754360	52.650280	427.649100	136.955500
-57.140120	52.518850	383.857500	104.659600
-55.539290	52.365840	355.807000	69.552190
-53.953660	52.191710	340.705500	46.024710
-52.384890	51.996940	330.558700	30.115650
-50.834480	51.782070	323.416600	18.866140
-49.303730	51.547700	318.434800	10.682320
-47.793770	51.294400	315.396700	5.335291
-46.305490	51.022820	313.867500	2.349802
-44.839570	50.733620	313.212900	.909749
-43.396390	50.427420	312.972200	.313306
-41.976090	50.104880	312.891700	.092593
-40.578500	49.766630	312.869000	.023590
-39.203150	49.413260	312.863300	.005114
-37.849210	49.045330	312.862300	.001380
-96.841810	50.160530	552.381900	187.171300
-95.453030	50.530490	592.280900	190.206100
-94.041510	50.885130	573.538600	181.482500
-92.606450	51.223850	518.813700	160.001200
-91.147380	51.546020	483.252900	151.838200
-89.664200	51.850960	477.924000	167.925300
-88.157130	52.138020	508.967600	209.045700
-86.626690	52.406500	598.143100	261.890000
-85.073680	52.655720	742.913800	305.607200
-83.499180	52.885050	830.024400	311.615900
-81.904440	53.093850	790.063400	284.062100
-80.290990	53.281520	691.160200	241.018600
-78.660530	53.447530	623.906200	219.719300
-77.014920	53.591380	662.971600	213.394500
-75.356160	53.712650	739.722400	215.888600
-73.686420	53.810940	772.706800	207.740600
-72.007930	53.885960	750.211200	224.595100
-70.322990	53.937460	781.794900	250.696600
-68.634010	53.965280	828.693500	247.996700
-66.943400	53.969350	776.906200	234.346900
-65.253590	53.949630	682.455400	221.448200
-63.567000	53.906200	549.593100	174.490600
-61.886040	53.839190	436.535700	116.480000
-60.213040	53.748820	387.554300	87.056030
-58.550290	53.635360	350.517300	65.114750
-56.899960	53.499180	330.540900	43.945190
-55.264110	53.340690	324.731300	28.328950
-53.644650	53.160350	321.514200	17.856900
-52.043340	52.958710	318.606800	11.477610
-50.461760	52.736320	316.323800	7.097362
-48.901280	52.493820	314.720900	4.032347
-47.363030	52.231850	313.733400	2.041785
-45.847900	51.951060	313.214800	.900796

-44.356530	51.652150	312.987600	.349228
-42.889220	51.335790	312.900300	.115944
-41.445980	51.002650	312.872000	.032972
-40.026490	50.653380	312.864200	.007868
-38.630060	50.288590	312.862300	.001596
-37.255620	49.908830	312.862000	.000511
-97.453060	51.026530	445.142100	124.343000
-96.042340	51.408490	457.378600	121.392400
-94.608220	51.774700	435.238700	110.242400
-93.149600	52.124570	403.255500	94.091030
-91.665800	52.457450	384.295500	85.136180
-90.156560	52.772640	380.491600	89.709010
-88.621980	53.069450	393.411000	108.829100
-87.062480	53.347160	433.073300	142.736600
-85.478850	53.605050	512.244200	176.602700
-83.872130	53.842430	570.459400	188.726200
-82.243660	54.058630	547.542400	177.876200
-80.595020	54.253030	477.560500	162.334600
-78.928000	54.425040	442.264500	165.009800
-77.244600	54.574130	510.519100	175.275600
-75.546970	54.699840	625.996700	200.637700
-73.837420	54.801750	710.515400	205.522400
-72.118360	54.879540	703.829500	206.650400
-70.392330	54.932960	691.626800	226.747400
-68.661900	54.961820	755.281100	233.653300
-66.929700	54.966030	740.938200	221.059200
-65.198370	54.945580	603.796000	192.651800
-63.470540	54.900540	459.466200	138.546800
-61.748820	54.831050	380.710500	80.981680
-60.035740	54.737340	341.385800	47.111520
-58.333780	54.619720	317.665600	28.230360
-56.645260	54.478570	311.098400	17.872490
-54.972420	54.314330	312.800900	9.649992
-53.317280	54.127510	314.233600	5.287511
-51.681740	53.918690	314.185200	3.314576
-50.067420	53.688470	313.776700	2.151148
-48.475770	53.437500	313.384900	1.267661
-46.907930	53.166490	313.116900	.655201
-45.364770	52.876130	312.968600	.293231
-43.846840	52.567130	312.899700	.111638
-42.354370	52.240220	312.873300	.036020
-40.887210	51.896080	312.864800	.009659
-39.444820	51.535370	312.862400	.002145
-38.026250	51.158730	312.862000	.000479
-36.630120	50.766700	312.862000	.000232
-98.097820	51.886770	367.599200	66.902910
-96.663910	52.281200	368.320700	63.245060
-95.206040	52.659450	358.119400	55.263430
-93.722790	53.020900	346.915200	46.438710
-92.213200	53.364910	340.471300	41.158280
-90.676770	53.690770	339.426700	42.178950
-89.113440	53.997730	344.919500	50.618050
-87.523560	54.285050	358.227800	65.671350
-85.907830	54.551970	381.288200	83.870550
-84.267300	54.797760	399.154700	90.871480
-82.603340	55.021710	373.603700	88.037610
-80.917590	55.223140	325.177800	95.232110
-79.211960	55.401440	310.214300	112.704700
-77.488560	55.556020	389.559700	128.160900
-75.749730	55.686380	526.620100	178.632100
-73.997920	55.792100	675.948700	212.227600
-72.235800	55.872810	732.241100	220.202500
-70.466090	55.928230	711.559700	231.868600
-68.691570	55.958180	742.697200	241.239400
-66.915130	55.962550	725.863900	227.439300
-65.139640	55.941330	556.867100	178.916700
-63.367970	55.894590	412.422500	109.015400

-61.602940	55.822490	349.667300	53.845870
-59.847310	55.725280	320.644600	24.328170
-58.103760	55.603290	304.273100	15.126220
-56.374820	55.456930	303.884900	11.551990
-54.662870	55.286670	308.923300	6.319524
-52.970100	55.093080	311.769000	2.438337
-51.298500	54.876750	312.728900	.703990
-49.649800	54.638340	312.941100	.346706
-48.025460	54.378550	312.950700	.252109
-46.426660	54.098120	312.916000	.151856
-44.854210	53.797780	312.886600	.072736
-43.308580	53.478290	312.871000	.028243
-41.789850	53.140400	312.864600	.008875
-40.297680	52.784830	312.862500	.002252
-38.831280	52.412250	312.862000	.000520
-37.389370	52.023280	312.861900	.000173
-35.970190	51.618480	312.862100	.000195
-98.778840	52.740810	333.608700	31.052870
-97.320270	53.148240	331.972200	28.164290
-95.837370	53.539010	328.769000	24.234160
-94.328290	53.912530	325.312800	20.193790
-92.791720	54.268120	323.026300	17.644440
-91.226900	54.605080	322.577800	17.790250
-89.633560	54.922620	324.349100	20.840930
-88.011890	55.219970	328.165300	26.132680
-86.362530	55.496330	331.718300	31.130150
-84.686490	55.750910	327.753800	33.572330
-82.985150	55.982970	299.295200	42.701830
-81.260250	56.191770	254.460900	70.794150
-79.513790	56.376650	246.914100	83.107240
-77.748020	56.536990	316.496200	88.619510
-75.965460	56.672260	440.301500	147.030800
-74.168790	56.781970	630.413800	201.241500
-72.360860	56.865740	760.786200	230.236800
-70.544630	56.923280	763.536500	248.150600
-68.723180	56.954370	764.715100	258.400900
-66.899620	56.958910	713.701400	238.148600
-65.077100	56.936880	529.610400	170.145200
-63.258730	56.888360	389.156700	89.161910
-61.447630	56.813510	331.915400	35.695690
-59.646790	56.712620	309.380600	13.420870
-57.859090	56.586040	298.360800	12.909770
-56.087300	56.434210	301.614800	11.416880
-54.333970	56.257640	307.932200	6.989120
-52.601470	56.056940	311.138500	3.259841
-50.891880	55.832740	312.314500	1.235547
-49.207030	55.585770	312.708000	.404399
-47.548420	55.316760	312.828900	.106953
-45.917200	55.026500	312.859100	.022296
-44.314130	54.715760	312.863500	.008597
-42.739580	54.385350	312.862900	.004179
-41.193420	54.036030	312.862200	.001452
-39.675070	53.668560	312.862000	.000419
-38.183400	53.283620	312.861900	.000162
-36.716770	52.881820	312.862000	.000155
-35.272900	52.463700	312.862100	.000170
-99.499240	53.588130	320.424400	13.035680
-98.014280	54.009130	319.393100	11.396940
-96.504860	54.412960	318.268900	9.618796
-94.968610	54.799050	317.109400	7.910958
-93.403790	55.166730	316.283800	6.773631
-91.809290	55.515250	315.998700	6.543385
-90.184560	55.843850	316.201700	7.184574
-88.529620	56.151670	316.196900	8.173121
-86.844990	56.437890	313.677800	10.102000
-85.131650	56.701690	302.222900	18.953540
-83.390960	56.942230	270.518700	40.784580

-81.624720	57.158780	226.386000	73.577290
-79.835050	57.350580	225.660900	74.891750
-78.024370	57.516980	279.567800	71.309510
-76.195360	57.657400	376.429400	122.321000
-74.350940	57.771310	578.325300	179.539200
-72.494220	57.858320	765.431800	230.475700
-70.628410	57.918090	812.247600	260.191300
-68.756890	57.950390	793.104400	271.240800
-66.883060	57.955100	703.319000	241.701900
-65.010370	57.932220	512.963700	161.191500
-63.142240	57.881810	374.836900	75.105740
-61.282040	57.804080	316.957200	26.656780
-59.433060	57.699310	299.200700	16.574760
-57.598450	57.567890	294.487400	16.028580
-55.781200	57.410300	301.229600	12.144120
-53.984060	57.227100	307.918200	7.509814
-52.209560	57.018940	311.076300	3.587377
-50.459930	56.786500	312.262200	1.382864
-48.737060	56.530560	312.667600	.507626
-47.042490	56.251900	312.805100	.162792
-45.377330	55.951360	312.847100	.045017
-43.742270	55.629770	312.858600	.010602
-42.137470	55.287960	312.861300	.002195
-40.562610	54.926740	312.861800	.000495
-39.016750	54.546860	312.861900	.000178
-37.498410	54.149020	312.861900	.000116
-36.005400	53.733830	312.862000	.000137
-34.534880	53.301770	312.862100	.000163
-100.262700	54.428120	315.431500	5.024883
-98.749250	54.863350	314.980500	4.228741
-97.211560	55.280850	314.573100	3.476765
-95.646590	55.680060	314.181900	2.785666
-94.052090	56.060360	313.871300	2.250438
-92.426510	56.420970	313.626000	1.906815
-90.768940	56.761100	313.183100	1.892050
-89.079190	57.079890	311.684200	3.779518
-87.357570	57.376450	306.771400	10.259200
-85.605000	57.649890	292.371600	23.761140
-83.822840	57.899360	257.813600	45.467490
-82.012920	58.124030	214.905400	77.834010
-80.177480	58.323120	225.104200	70.143250
-78.319110	58.495910	277.693800	55.207700
-76.440710	58.641750	365.608500	92.616380
-74.545430	58.760110	550.623400	157.397500
-72.636660	58.850530	756.384500	229.745400
-70.717930	58.912650	850.173000	269.537800
-68.792920	58.946230	823.887800	279.599700
-66.865380	58.951130	694.644800	241.768900
-64.939080	58.927340	500.514500	156.008000
-63.017780	58.874950	366.694900	66.003260
-61.105210	58.794160	307.548200	28.819120
-59.204930	58.685300	289.044300	29.349650
-57.320400	58.548780	290.125400	21.080140
-55.454840	58.385130	300.685400	13.566160
-53.611270	58.194950	308.103500	7.492547
-51.792370	57.978940	311.215100	3.290896
-50.000500	57.737840	312.312100	1.271478
-48.237630	57.472490	312.683900	.464363
-46.505310	57.183730	312.808700	.151567
-44.804610	56.872430	312.847500	.043699
-43.136050	56.539500	312.858400	.011101
-41.499610	56.185780	312.861100	.002502
-39.894630	55.812120	312.861800	.000534
-38.319800	55.419290	312.861900	.000152
-36.773090	55.007980	312.862000	.000130
-35.251730	54.578750	312.862000	.000133
-33.752120	54.132060	312.862100	.000163

-101.073700 55.260130 313.669700 1.771477
-99.529010 55.710320 313.499700 1.433056
-97.960900 56.142140 313.359600 1.136229
-96.365430 56.555100 313.224700 .852441
-94.739640 56.948590 313.078200 .568461
-93.081440 57.321860 312.820500 .547745
-91.389490 57.674070 312.112100 1.860453
-89.663250 58.004340 309.988600 5.377560
-87.902820 58.311730 304.015700 13.103480
-86.109000 58.595320 287.815900 27.112080
-84.283100 58.854170 250.221900 47.665400
-82.427000 59.087400 209.471200 78.739240
-80.543050 59.294160 237.882000 61.138430
-78.634010 59.473690 311.392300 30.092310
-76.703000 59.625280 403.397500 69.357930
-74.753460 59.748340 550.472900 151.749500
-72.789070 59.842370 734.844300 231.880000
-70.813740 59.906970 868.025100 275.531200
-68.831480 59.941910 858.323400 284.747200
-66.846440 59.947010 695.493300 242.380700
-64.862770 59.922250 495.312100 156.587300
-62.884610 59.867760 371.459500 68.894240
-60.916040 59.783740 312.681700 24.779190
-58.961010 59.670550 291.157400 27.060170
-57.023270 59.528640 290.927300 22.043750
-55.106350 59.358580 301.244700 13.171080
-53.213500 59.161040 308.711900 6.224886
-51.347600 58.936760 311.485500 2.707989
-49.511150 58.686560 312.422200 1.003034
-47.706190 58.411320 312.721600 .363529
-45.934240 58.111950 312.820300 .117619
-44.196280 57.789400 312.850600 .033924
-42.492650 57.444580 312.859200 .008638
-40.823030 57.078400 312.861300 .001956
-39.186360 56.691730 312.861800 .000433
-37.580830 56.285340 312.861900 .000146
-36.003750 55.859910 312.862000 .000118
-34.451600 55.415950 312.862000 .000145
-32.919830 54.953830 312.862100 .000163
-101.937200 56.083360 313.096700 .568869
-100.358100 56.549350 313.038900 .439846
-98.756940 56.996240 312.991200 .325592
-97.128790 57.423640 312.936600 .199132
-95.469860 57.830970 312.835400 .189117
-93.777320 58.217490 312.559300 .807211
-92.049300 58.582370 311.727400 2.447461
-90.284800 58.924680 309.317900 6.281974
-88.483640 59.243460 302.803000 14.356090
-86.646400 59.537720 285.420600 28.418870
-84.774360 59.806450 245.519600 48.257050
-82.869400 60.048700 209.704400 75.908920
-80.933970 60.263580 258.927800 47.055630
-78.971000 60.450230 347.607600 31.572590
-76.983890 60.607900 430.543300 76.523550
-74.976370 60.735940 528.765100 146.854300
-72.952470 60.833800 677.912400 225.871300
-70.916480 60.901050 849.469400 273.887800
-68.872850 60.937420 887.600600 285.987600
-66.826130 60.942730 705.470700 242.678700
-64.780930 60.916960 498.988900 161.115800
-62.741830 60.860230 386.642300 80.745900
-60.713310 60.772790 335.706500 27.362050
-58.699720 60.654990 310.959000 9.208740
-56.705200 60.507380 303.495400 10.153940
-54.733590 60.330550 307.106600 6.898740
-52.788400 60.125220 310.512200 3.892891
-50.872730 59.892220 312.068800 1.586064

-48.989200 59.632420 312.588000 .639984
-47.139900 59.346780 312.772300 .235033
-45.326320 59.036270 312.834800 .077004
-43.549270 58.701890 312.854500 .022250
-41.808860 58.344600 312.860100 .005676
-40.104370 57.965370 312.861600 .001285
-38.434220 57.565060 312.861900 .000300
-36.795950 57.144450 312.862000 .000143
-35.186060 56.704150 312.862000 .000110
-33.600030 56.244660 312.862100 .000163
-32.032190 55.766200 312.862100 .000159
-102.859900 56.896880 312.924700 .165152
-101.242000 57.379640 312.906100 .119027
-99.604390 57.842470 312.888300 .073066
-97.940930 58.285070 312.856500 .055388
-96.246660 58.706940 312.766400 .282435
-94.517820 59.107400 312.478800 .940025
-92.751850 59.485580 311.594200 2.604207
-90.947170 59.840560 309.048900 6.457644
-89.103220 60.171320 302.216300 14.419480
-87.220290 60.476800 283.607200 28.175730
-85.299550 60.755960 243.489300 48.108910
-83.342830 61.007760 219.083900 68.678760
-81.352710 61.231220 290.170100 32.186810
-79.332320 61.425420 372.471800 46.514690
-77.285290 61.589540 423.279200 73.748530
-75.215710 61.722870 475.252600 129.731600
-73.128000 61.824790 594.494100 202.684200
-71.026890 61.894870 797.599800 259.072000
-68.917300 61.932760 897.047700 277.574100
-66.804310 61.938290 721.669000 236.058100
-64.692990 61.911450 507.703200 160.702100
-62.588420 61.852340 400.474700 89.461180
-60.495590 61.761240 355.065800 42.702570
-58.419300 61.638580 329.710700 16.468420
-56.364090 61.484900 316.490200 4.060374
-54.334160 61.300890 312.616700 1.221058
-52.333320 61.087320 312.379400 1.004166
-50.364880 60.845090 312.586800 .619384
-48.431600 60.575160 312.743700 .297390
-46.535590 60.278550 312.818800 .118821
-44.678250 59.956310 312.848100 .040582
-42.860180 59.609490 312.858000 .012034
-41.081100 59.239120 312.861000 .003101
-39.339830 58.846180 312.861800 .000729
-37.634110 58.431540 312.861900 .000199
-35.960610 57.995940 312.862000 .000122
-34.314830 57.539980 312.862000 .000153
-32.690990 57.064010 312.862100 .000163
-31.082000 56.568130 312.862100 .000153
-103.849600 57.699570 312.877000 .042356
-102.187300 58.200280 312.871100 .026073
-100.509000 58.680030 312.862100 .013427
-98.806840 59.138710 312.837200 .080309
-97.074520 59.575940 312.752300 .300271
-95.307120 59.991050 312.470700 .913449
-93.501110 60.383240 311.595400 2.467921
-91.654170 60.751550 309.066700 6.048323
-89.765210 61.094930 301.924600 13.384400
-87.834170 61.412270 281.931700 26.617780
-85.861950 61.702450 245.657000 47.378520
-83.850420 61.964360 244.520500 54.289940
-81.802150 62.196920 323.391600 34.843360
-79.720490 62.399150 380.895100 52.659150
-77.609360 62.570130 394.448700 61.272370
-75.473210 62.709080 415.250100 100.111600
-73.316960 62.815350 508.507400 163.670600

-71.145790	62.888430	703.759000	220.774200
-68.965200	62.927950	839.052100	244.995300
-66.780790	62.933720	695.076100	210.028800
-64.598260	62.905720	496.497400	142.594000
-62.423240	62.844070	393.089400	81.290390
-60.261290	62.749080	350.984100	42.568180
-58.117710	62.621230	329.420800	19.649520
-55.997520	62.461110	318.079600	7.154889
-53.905320	62.269450	314.038100	2.005772
-51.845240	62.047150	313.012100	.334116
-49.820820	61.795150	312.837100	.115766
-47.834930	61.514500	312.832400	.088788
-45.889670	61.206310	312.847400	.043719
-43.986290	60.871700	312.856600	.016605
-42.125070	60.511780	312.860300	.005170
-40.305270	60.127630	312.861500	.001379
-38.525020	59.720250	312.861900	.000347
-36.781200	59.290500	312.862000	.000141
-35.069400	58.839090	312.862000	.000127
-33.383770	58.366500	312.862100	.000166
-31.716990	57.872960	312.862100	.000163
-30.060130	57.358350	312.862100	.000153
-104.916500	58.490070	312.865100	.008965
-103.202300	59.010120	312.862700	.003473
-101.477600	59.507980	312.856800	.018669
-99.732440	59.983750	312.835600	.080044
-97.958720	60.437220	312.760900	.267551
-96.150010	60.867820	312.511700	.787712
-94.301540	61.274790	311.742900	2.095862
-92.410060	61.657170	309.547200	5.055835
-90.473700	62.013880	303.065600	10.989910
-88.491920	62.343760	284.934900	22.779490
-86.465330	62.645610	262.460500	39.187130
-84.395630	62.918240	282.130500	27.571390
-82.285480	63.160470	347.321200	39.611580
-80.138370	63.371240	371.551900	47.677350
-77.958560	63.549540	361.992600	44.011830
-75.750930	63.694510	368.989500	67.830570
-73.520860	63.805420	426.048600	112.611200
-71.274150	63.881700	556.525800	161.582200
-69.016910	63.922960	666.043000	182.748700
-66.755400	63.928990	573.346200	158.853800
-64.495980	63.899750	437.613400	107.407000
-62.244980	63.835380	365.945600	60.072720
-60.008560	63.736250	335.833300	30.487600
-57.792640	63.602840	322.270500	14.310860
-55.602750	63.435840	316.130200	5.790100
-53.443960	63.236060	313.821400	1.934264
-51.320750	63.004450	313.104200	.553460
-49.236890	62.742080	312.906900	.120321
-47.195330	62.450080	312.864300	.020546
-45.198120	62.129630	312.859400	.010042
-43.246250	61.781950	312.860500	.004810
-41.339580	61.408210	312.861400	.001690
-39.476710	61.009530	312.861800	.000501
-37.654880	60.586900	312.861900	.000155
-35.869840	60.141170	312.862000	.000130
-34.115770	59.672970	312.862100	.000166
-32.385130	59.182660	312.862100	.000163
-30.668580	58.670220	312.862100	.000163
-28.954870	58.135290	312.862100	.000153
-106.073800	59.266680	312.862400	.001148
-104.297500	59.807790	312.861100	.003585
-102.518800	60.325180	312.856600	.018251
-100.725000	60.819250	312.839800	.067285
-98.905530	61.289990	312.780400	.214235
-97.052100	61.737010	312.588500	.609638

-95.158340	62.159620	312.012800	1.573146
-93.219700	62.556880	310.391700	3.670711
-91.233350	62.927700	305.544100	7.784983
-89.198040	63.270870	294.335300	14.939490
-87.113920	63.585110	286.881400	20.830130
-84.982480	63.869130	311.976700	15.353980
-82.806390	64.121680	349.692600	36.369970
-80.589290	64.341560	348.879800	29.500790
-78.335770	64.527670	336.465200	26.746230
-76.051160	64.679080	341.431900	39.990860
-73.741460	64.794950	365.782500	65.961120
-71.413090	64.874680	421.016600	97.948030
-69.072890	64.917820	476.185000	111.893300
-66.727910	64.924110	432.838500	99.041890
-64.385280	64.893550	372.689500	66.061720
-62.052090	64.826280	339.721100	36.873160
-59.735260	64.722690	324.508000	18.729670
-57.441380	64.583340	317.616200	8.692142
-55.176610	64.408980	314.624400	3.623798
-52.946510	64.200520	313.449100	1.346213
-50.755950	63.959000	313.032100	.430129
-48.608900	63.685590	312.903400	.113884
-46.508400	63.381530	312.869800	.023383
-44.456330	63.048090	312.862800	.003365
-42.453320	62.686580	312.861800	.000755
-40.498620	62.298210	312.861800	.000371
-38.589950	61.884150	312.861900	.000153
-36.723370	61.445380	312.862000	.000147
-34.893150	60.982680	312.862000	.000145
-33.091610	60.496560	312.862100	.000166
-31.309040	59.987160	312.862100	.000163
-29.533510	59.454220	312.862100	.000153
-27.750760	58.896990	312.862100	.000153
-107.339200	60.027230	312.861800	.000940
-105.487000	60.591610	312.861000	.003955
-103.643800	61.130270	312.857400	.016018
-101.793500	61.644070	312.844200	.055133
-99.922550	62.133320	312.800500	.166268
-98.020040	62.597820	312.667200	.448966
-96.077510	63.037030	312.296800	1.092052
-94.088710	63.450070	311.366300	2.381535
-92.049460	63.835860	309.124700	4.477448
-89.957580	64.193130	304.392800	7.353034
-87.812570	64.520550	304.954600	7.068472
-85.615560	64.816710	322.747900	11.848130
-83.369120	65.080260	336.413700	19.569550
-81.077050	65.309890	328.719600	15.223790
-78.744270	65.504390	324.309100	15.783160
-76.376630	65.662700	326.956700	22.450520
-73.980770	65.783920	336.278100	35.039790
-71.563890	65.867360	353.179700	51.202960
-69.133670	65.912500	371.089000	59.701480
-66.698060	65.919100	357.101500	52.483020
-64.265090	65.887100	338.139300	35.738140
-61.842790	65.816700	325.557900	20.694580
-59.438900	65.708320	318.625500	10.724020
-57.060850	65.562580	315.272800	5.058617
-54.715470	65.380330	313.784000	2.152993
-52.408880	65.162570	313.180100	.814555
-50.146370	64.910470	312.959600	.268962
-47.932120	64.625290	312.888200	.076233
-45.769150	64.308400	312.868000	.018217
-43.659080	63.961160	312.863200	.003524
-41.602020	63.584970	312.862200	.000518
-39.596350	63.181100	312.862100	.000159
-37.638600	62.750720	312.862100	.000166
-35.723270	62.294770	312.862100	.000153

-33.842640	61.813940	312.862100	.000153
-31.986660	61.308540	312.862100	.000153
-30.142740	60.778430	312.862100	.000153
-28.295590	60.222970	312.862100	.000153
-26.427080	59.640870	312.862100	.000153
-108.736700	60.768890	312.861700	.001454
-106.789100	61.359350	312.860800	.004511
-104.866900	61.921510	312.857200	.016542
-102.949500	62.456830	312.844900	.053159
-101.019200	62.966030	312.806800	.151028
-99.061880	63.449270	312.701000	.381022
-97.066180	63.906190	312.434000	.859805
-95.023590	64.336020	311.859600	1.718772
-92.928180	64.737730	310.872000	2.843723
-90.776390	65.110020	309.454500	3.580061
-88.566850	65.451470	311.242600	2.832905
-86.300130	65.760610	322.025900	8.494092
-83.978550	66.035940	324.777300	10.757460
-81.606060	66.276030	321.202800	10.289900
-79.187900	66.479550	320.098100	11.265600
-76.730480	66.645290	321.494500	15.330320
-74.241170	66.772270	326.117600	22.612030
-71.728080	66.859700	332.887700	31.047600
-69.199870	66.907030	338.049600	35.739950
-66.665550	66.913940	334.014600	31.777220
-64.134220	66.880400	326.647100	22.774300
-61.614990	66.806620	320.407700	13.928690
-59.116610	66.693070	316.485200	7.499630
-56.647430	66.540450	314.432300	3.616447
-54.215080	66.349720	313.480900	1.566844
-51.826330	66.121990	313.082900	.604516
-49.486860	65.858530	312.932900	.205742
-47.201050	65.560780	312.882300	.061349
-44.971820	65.230190	312.867100	.015918
-42.800360	64.868270	312.863200	.003562
-40.685980	64.476460	312.862300	.000695
-38.625890	64.056100	312.862100	.000187
-36.614990	63.608330	312.862100	.000151
-34.645670	63.134020	312.862100	.000153
-32.707610	62.633670	312.862100	.000153
-30.787590	62.107330	312.862100	.000153
-28.869240	61.554470	312.862100	.000153
-26.932890	60.973880	312.862100	.000153
-24.955330	60.363580	312.862100	.000153

Q.15a

-89.453400	32.991660	330.370200	1.245879
-88.345930	33.191160	330.755900	1.781573
-87.228100	33.381660	331.813700	3.194101
-86.100230	33.562890	333.447900	5.297545
-84.962690	33.734530	335.334600	7.679787
-83.815900	33.896310	337.058300	9.846634
-82.660300	34.047950	338.362100	11.468680
-81.496410	34.189190	339.124300	12.361970
-80.324730	34.319790	339.177900	12.381300
-79.145840	34.439500	338.295400	11.345590
-77.960310	34.548100	336.443600	9.478332
-76.768760	34.645400	334.014500	7.388643
-75.571840	34.731200	332.496000	6.511608
-74.370180	34.805370	332.381400	6.428389
-73.164480	34.867740	332.252800	5.727758
-71.955430	34.918200	332.143300	4.972913
-70.743730	34.956650	332.165600	4.329323
-69.530080	34.983020	332.130200	3.873277
-68.315220	34.997260	331.901800	3.366658
-67.099880	34.999340	331.435500	2.684511
-65.884770	34.989250	330.839100	1.872865
-64.670640	34.967020	330.291300	1.107123
-63.458190	34.932690	329.917000	.551461
-62.248150	34.886320	329.715500	.233799
-61.041230	34.828000	329.627500	.085882
-59.838120	34.757850	329.595000	.027471
-58.639500	34.676010	329.584900	.007594
-57.446040	34.582610	329.582300	.002183
-56.258390	34.477850	329.581900	.000886
-55.077160	34.361910	329.581900	.000393
-53.902950	34.235010	329.582000	.000144
-52.736340	34.097380	329.582000	.000066
-51.577860	33.949260	329.582100	.000032
-50.428010	33.790910	329.582100	.000031
-49.287250	33.622600	329.582100	.000031
-48.156040	33.444620	329.582100	.000031
-47.034740	33.257250	329.582100	.000031
-45.923690	33.060790	329.582100	.000031
-44.823220	32.855560	329.582100	.000031
-89.701210	33.918400	331.030700	2.085116
-88.583520	34.125020	331.995100	3.283987
-87.455040	34.322360	334.510800	6.268229
-86.316120	34.510110	338.481400	10.745990
-85.167100	34.687960	343.053300	15.793340
-84.008420	34.855620	347.087800	20.260050
-82.840550	35.012790	350.027300	23.473570
-81.663990	35.159200	351.786400	25.231660
-80.479300	35.294580	352.001300	25.247700
-79.287040	35.418700	350.183600	23.317970
-78.087840	35.531310	345.910600	18.932060
-76.882320	35.632210	340.413000	13.926320
-75.671150	35.721200	337.161100	13.092330
-74.455030	35.798110	338.054300	14.189610
-73.234660	35.862810	338.383700	14.042610
-72.010770	35.915150	337.636400	11.663580
-70.784100	35.955040	337.065000	10.245240
-69.555380	35.982390	336.581900	9.073359
-68.325390	35.997170	335.741400	7.826531
-67.094890	35.999320	334.413900	6.207559
-65.864640	35.988860	332.789400	4.283721
-64.635410	35.965800	331.322100	2.465166
-63.407950	35.930180	330.370900	1.192436
-62.183030	35.882080	329.884600	.485194
-60.961370	35.821590	329.679800	.168130
-59.743720	35.748840	329.607300	.049557
-58.530780	35.663950	329.586000	.013320

-57.323260	35.567100	329.581400	.005478
-56.121820	35.458470	329.581200	.002994
-54.927120	35.338260	329.581600	.001332
-53.739770	35.206700	329.581900	.000499
-52.560380	35.064020	329.582000	.000145
-51.389480	34.910500	329.582000	.000064
-50.227610	34.746390	329.582000	.000000
-49.075240	34.571990	329.582100	.000031
-47.932830	34.387580	329.582100	.000031
-46.800740	34.193490	329.582100	.000031
-45.679330	33.990000	329.582000	.000000
-44.568900	33.777440	329.582100	.000031
-89.960660	34.843320	332.995600	4.444390
-88.832330	35.057210	335.879600	7.631131
-87.692770	35.261540	343.386300	15.406760
-86.542320	35.455950	355.746200	27.332740
-85.381340	35.640140	369.850400	40.689600
-84.210270	35.813810	381.803300	52.091750
-83.029580	35.976620	390.181800	59.905230
-81.839790	36.128310	395.161200	64.033600
-80.641480	36.268600	396.071400	64.284710
-79.435230	36.397220	390.895900	58.982440
-78.221690	36.513940	378.677500	47.618350
-77.001530	36.618520	362.766400	34.800850
-75.775430	36.710780	353.419500	31.665190
-74.544130	36.790520	365.000700	44.595290
-73.308360	36.857590	358.530300	38.536740
-72.068890	36.911860	357.779600	35.118950
-70.826490	36.953220	355.200000	28.896260
-69.581950	36.981590	352.846000	25.723970
-68.336070	36.996900	349.897500	22.373440
-67.089650	36.999130	345.323900	17.765100
-65.843500	36.988290	339.960800	12.338880
-64.598400	36.964370	335.054300	6.975684
-63.355190	36.927440	331.930800	3.238959
-62.114630	36.877570	330.451300	1.292824
-60.877510	36.814860	329.850900	.430014
-59.644590	36.739430	329.642900	.118044
-58.416640	36.651430	329.584500	.037418
-57.194370	36.551040	329.575100	.023285
-55.978490	36.438440	329.577200	.013064
-54.769680	36.313860	329.579900	.005712
-53.568590	36.177530	329.581200	.002044
-52.375820	36.029710	329.581900	.000622
-51.191970	35.870660	329.582000	.000165
-50.017550	35.700660	329.582000	.000040
-48.853080	35.520030	329.582000	.000000
-47.698980	35.329070	329.582100	.000031
-46.555660	35.128100	329.582100	.000031
-45.423470	34.917440	329.582000	.000000
-44.302690	34.697410	329.582100	.000031
-90.232250	35.766320	336.681800	8.578945
-89.092860	35.987640	344.492000	16.169220
-87.941790	36.199090	365.802200	34.914660
-86.779330	36.400320	402.538000	65.138210
-85.605870	36.591000	445.034200	98.951000
-84.421870	36.770790	479.018200	126.310600
-83.227800	36.939400	501.244000	144.106000
-82.024180	37.096500	514.856300	153.327700
-80.811620	37.241800	517.354000	153.832900
-79.590740	37.375040	504.242800	143.383500
-78.362190	37.495960	474.144700	110.730100
-77.126670	37.604320	397.295700	79.961910
-75.884930	37.699920	392.124300	84.984480
-74.637700	37.782550	452.054400	119.006700
-73.385770	37.852060	430.787100	106.448600
-72.129940	37.908310	409.593200	83.998700

-70.871030	37.951180	407.114600	76.824490
-69.609860	37.980580	400.397100	66.316350
-68.347290	37.996460	391.209200	58.355180
-67.084140	37.998780	377.652800	46.840470
-65.821270	37.987530	360.603900	32.494930
-64.559530	37.962740	345.448200	18.280820
-63.299760	37.924460	336.097500	8.176254
-62.042780	37.872770	331.794000	3.044545
-60.789440	37.807780	330.212000	.981076
-59.540510	37.729610	329.684800	.273860
-58.296810	37.638420	329.549800	.142520
-57.059080	37.534390	329.542200	.099073
-55.828080	37.417740	329.559700	.052281
-54.604500	37.288680	329.572900	.021930
-53.389020	37.147460	329.579100	.007486
-52.182290	36.994370	329.581200	.002187
-50.984880	36.829660	329.581900	.000558
-49.797380	36.653650	329.582000	.000114
-48.620270	36.466650	329.582000	.000018
-47.454020	36.268990	329.582000	.000000
-46.299010	36.060990	329.582100	.000031
-45.155610	35.843010	329.582000	.000000
-44.024070	35.615370	329.582100	.000031
-90.516530	36.687310	341.390300	13.932840
-89.365660	36.916220	359.906300	28.729230
-88.202590	37.134950	412.595600	68.973300
-87.027650	37.343150	517.124000	139.216600
-85.841200	37.540460	639.926100	219.550300
-84.643710	37.726540	729.846100	279.661000
-83.435650	37.901050	780.006300	314.937300
-82.217600	38.063690	817.272700	332.476900
-80.990140	38.214140	828.214800	335.360400
-79.753940	38.352110	805.459200	312.782900
-78.509670	38.477340	674.591200	244.521400
-77.258070	38.589580	463.712500	160.657300
-75.999910	38.688600	374.536000	115.603300
-74.735980	38.774210	647.015900	387.585400
-73.467090	38.846230	608.137100	253.418300
-72.194090	38.904510	570.429700	185.014000
-70.917830	38.948930	536.033600	163.951500
-69.639210	38.979390	526.051800	154.449300
-68.359090	38.995840	505.060300	136.064800
-67.078360	38.998240	471.209400	115.994700
-65.797930	38.986590	419.631100	79.693740
-64.518690	38.960900	369.279700	40.680060
-63.241520	38.921240	344.923900	17.713560
-61.967320	38.867690	334.466000	6.274787
-60.696920	38.800350	330.682900	1.844555
-59.431210	38.719360	329.564800	.750630
-58.170970	38.624900	329.340100	.580398
-56.917050	38.517150	329.405100	.370504
-55.670200	38.396340	329.496100	.186362
-54.431160	38.262690	329.549500	.073575
-53.200640	38.116470	329.572300	.024047
-51.979310	37.957960	329.579700	.006484
-50.767770	37.787460	329.581600	.001516
-49.566610	37.605300	329.581900	.000343
-48.376340	37.411790	329.582000	.000089
-47.197410	37.207270	329.582000	.000013
-46.030250	36.992090	329.582000	.000000
-44.875180	36.766620	329.582000	.000000
-43.732470	36.531200	329.582100	.000031
-90.814130	37.606180	343.190900	17.472050
-89.651300	37.842850	374.845500	40.328200
-88.475770	38.069030	484.279200	113.301500
-87.287830	38.284350	788.899700	273.291600
-86.087840	38.488450	1140.633000	449.695700

-84.876290 38.680970 1356.715000 564.586200
-83.653650 38.861550 1457.080000 619.534500
-82.420500 39.029860 1548.313000 643.019200
-81.177470 39.185590 1589.895000 655.007900
-79.925230 39.328410 1525.863000 631.282400
-78.664510 39.458070 1272.380000 493.862600
-77.396060 39.574290 761.641700 279.429700
-76.120680 39.676830 330.247900 384.079800
-74.839220 39.765490 1397.894000 1838.657000
-73.552520 39.840080 795.340200 585.209800
-72.261480 39.900440 948.273400 469.320600
-70.967010 39.946460 875.269300 324.562000
-69.670030 39.978020 839.052600 311.915800
-68.371470 39.995060 818.394800 312.230100
-67.072270 39.997550 701.923900 254.652400
-65.773400 39.985470 573.124900 194.714100
-64.475770 39.958870 425.370500 84.655090
-63.180340 39.917780 358.330500 30.434750
-61.888020 39.862300 337.596400 10.149650
-60.599740 39.792560 330.562000 3.417902
-59.316390 39.708680 328.548100 2.495242
-58.038840 39.610860 328.460400 2.027844
-56.767930 39.499290 328.901500 1.235726
-55.504470 39.374200 329.298900 .558627
-54.249260 39.235840 329.486900 .213071
-53.003000 39.084480 329.556600 .064290
-51.766410 38.920440 329.577300 .016184
-50.540120 38.744010 329.581700 .003632
-49.324710 38.555530 329.582200 .001020
-48.120720 38.355340 329.582100 .000331
-46.928620 38.143810 329.582000 .000095
-45.748810 37.921300 329.582000 .000040
-44.581620 37.688170 329.582000 .000000
-43.427300 37.444810 329.582100 .000031
-91.125630 38.522790 330.278500 19.682770
-89.950410 38.767390 354.962700 41.119080
-88.761920 39.001200 532.402400 152.978500
-87.560450 39.223820 1320.992000 429.892200
-86.346370 39.434880 2257.579000 864.468500
-85.120160 39.633990 2762.992000 1064.792000
-83.882300 39.820790 2967.894000 1128.074000
-82.633390 39.994930 3085.846000 1156.666000
-81.374080 40.156070 3164.665000 1180.754000
-80.105060 40.303890 3149.187000 1178.161000
-78.827100 40.438090 2896.380000 1027.924000
-77.540980 40.558400 2101.087000 655.275900
-76.247540 40.664560 687.905800 532.716300
-74.947690 40.756360 265.312600 390.030600
-73.642300 40.833600 942.625600 917.955600
-72.332310 40.896110 1250.761000 775.773500
-71.018690 40.943760 1595.405000 763.595200
-69.702430 40.976440 1459.232000 592.712000
-68.384490 40.994090 1336.510000 572.331700
-67.065880 40.996670 927.196100 377.598600
-65.747600 40.984170 681.232400 295.269800
-64.430650 40.956610 486.330700 142.521600
-63.116020 40.914060 374.876100 45.428710
-61.804690 40.856610 337.811500 14.372630
-60.497620 40.784390 326.962700 8.047969
-59.195770 40.697540 324.434800 8.009756
-57.900040 40.596260 325.378300 6.126413
-56.611330 40.480750 327.488200 3.326018
-55.330490 40.351270 328.754700 1.484109
-54.058330 40.208070 329.362700 .486223
-52.795620 40.051450 329.541500 .139513
-51.543080 39.881710 329.581700 .034641
-50.301390 39.699200 329.586000 .011961

-49.071130 39.504250 329.584100 .004969
-47.852850 39.297230 329.582700 .001762
-46.647030 39.078520 329.582200 .000503
-45.454070 38.848490 329.582000 .000120
-44.274280 38.607530 329.582000 .000041
-43.107920 38.356030 329.582100 .000031
-91.451740 39.437030 301.183700 25.527820
-90.263630 39.689750 255.384200 52.332590
-89.061680 39.931370 343.714800 275.809100
-87.846150 40.161480 1546.474000 558.710400
-86.617400 40.379670 3163.320000 1083.789000
-85.375900 40.585540 4193.928000 1537.187000
-84.122160 40.778730 4621.987000 1697.276000
-82.856800 40.958850 4815.030000 1748.994000
-81.580460 41.125550 4984.453000 1805.770000
-80.293880 41.278500 5248.560000 1909.274000
-78.997860 41.417370 5376.562000 1929.139000
-77.693230 41.541890 4905.867000 1716.641000
-76.380870 41.651780 3097.796000 1135.139000
-75.061690 41.746810 626.062300 461.693500
-73.736660 41.826770 323.718000 491.125600
-72.406780 41.891490 1221.029000 772.143500
-71.073040 41.940830 2024.684000 991.535700
-69.736500 41.974670 1852.478000 814.339200
-68.398190 41.992950 1273.920000 531.177900
-67.059160 41.995620 707.847000 381.067500
-65.720480 41.982670 355.611900 175.735000
-64.383220 41.954140 393.838500 246.695700
-63.048400 41.910080 351.781800 64.236270
-61.717090 41.850600 332.052600 27.054790
-60.390290 41.775820 313.136700 23.176030
-59.069010 41.685920 314.451200 19.682790
-57.754210 41.581080 317.069000 14.733900
-56.446830 41.461530 323.964300 8.004077
-55.147770 41.327530 327.938700 2.860699
-53.857880 41.179360 329.222800 .921868
-52.577970 41.017320 329.604100 .250984
-51.308780 40.841740 329.634300 .108689
-50.051000 40.652980 329.610800 .053285
-48.805250 40.451390 329.593300 .021911
-47.572090 40.237360 329.585600 .007552
-46.352010 40.011290 329.582900 .002183
-45.145370 39.773560 329.582200 .000525
-43.952510 39.524580 329.582000 .000115
-42.773630 39.264750 329.582100 .000037
-91.793200 40.348750 326.250400 34.062930
-90.591710 40.609810 288.674500 61.533300
-89.375760 40.859430 148.292200 206.059100
-88.145600 41.097210 562.588700 552.507700
-86.901570 41.322720 1877.611000 550.177800
-85.644150 41.535550 3017.492000 777.005600
-84.373860 41.735290 3893.610000 1144.514000
-83.091290 41.921560 4503.761000 1477.190000
-81.797160 42.093980 5116.469000 1767.204000
-80.492230 42.252200 5841.571000 2045.666000
-79.177290 42.395880 6347.799000 2210.641000
-77.853240 42.524730 6217.274000 2180.754000
-76.521000 42.638450 4794.763000 1563.168000
-75.181550 42.736820 2473.510000 643.916100
-73.835900 42.819590 1383.354000 457.936000
-72.485100 42.886590 1267.027000 561.596500
-71.130210 42.937670 1783.849000 763.613700
-69.772340 42.972710 1616.291000 659.101000
-68.412590 42.991630 883.478000 452.270700
-67.052090 42.994400 233.900200 244.443100
-65.691960 42.980990 223.862000 305.795700
-64.333320 42.951450 448.503700 335.390400

-62.977290 42.905830 395.495700 192.326900
-61.624970 42.844260 324.443400 55.364200
-60.277440 42.766850 286.349400 60.356330
-58.935750 42.673790 289.500800 52.149720
-57.600940 42.565280 301.012400 36.159390
-56.273990 42.441570 319.903400 13.437930
-54.955840 42.302930 327.426400 4.750663
-53.647390 42.149640 329.881400 1.366475
-52.349470 41.982030 330.039600 .744704
-51.062890 41.800450 329.872900 .445823
-49.788320 41.605260 329.710200 .204606
-48.526440 41.396860 329.627900 .079217
-47.277780 41.175640 329.595900 .026353
-46.042850 40.942000 329.585600 .007649
-44.822010 40.696380 329.582900 .001918
-43.615560 40.439180 329.582200 .000420
-42.423690 40.170830 329.582100 .000117
-92.150770 41.257820 451.481500 112.199000
-90.935390 41.527410 554.174400 183.772200
-89.704910 41.785250 620.319000 340.961000
-88.459540 42.030900 474.289300 448.028900
-87.199620 42.263930 587.281600 465.574700
-85.925610 42.483890 1228.704000 441.470100
-84.638040 42.690380 2450.105000 623.801100
-83.337520 42.882970 3505.208000 1077.133000
-82.024790 43.061280 4568.192000 1570.344000
-80.700610 43.224930 5678.811000 1996.287000
-79.365870 43.373580 6203.158000 2177.812000
-78.021450 43.506890 6120.449000 2181.527000
-76.668370 43.624570 5425.785000 1976.585000
-75.307630 43.726360 4190.646000 1429.427000
-73.940300 43.812040 3130.558000 1160.719000
-72.567500 43.881390 1803.908000 532.237100
-71.190370 43.934270 1651.374000 544.103900
-69.810050 43.970550 1678.885000 649.135400
-68.427750 43.990140 1358.180000 648.291100
-67.044650 43.993000 737.552400 410.467100
-65.661930 43.979120 667.351200 350.396200
-64.280810 43.948540 695.850200 387.552100
-62.902460 43.901310 502.052500 188.571200
-61.528050 43.837570 329.258500 120.420400
-60.158730 43.757450 274.443600 195.679900
-58.795620 43.661140 270.911900 145.410600
-57.439790 43.548850 306.141300 52.884640
-56.092310 43.420850 322.319500 29.643770
-54.754150 43.277410 333.126500 7.054274
-53.426270 43.118850 333.026700 4.216662
-52.109530 42.945500 331.847800 2.858884
-50.804760 42.757750 330.657900 1.466783
-49.512690 42.555960 330.017800 .643655
-48.233980 42.340550 329.732600 .238534
-46.969190 42.111940 329.627100 .077545
-45.718810 41.870550 329.593800 .022320
-44.483210 41.616830 329.584700 .005697
-43.262650 41.351200 329.582500 .001296
-42.057300 41.074100 329.582200 .000350
-92.525320 42.164070 515.942100 141.450700
-91.295520 42.442420 729.529500 264.575100
-90.049940 42.708690 1110.841000 435.852300
-88.788760 42.962440 1233.695000 467.307700
-87.512310 43.203190 971.039200 339.175400
-86.221000 43.430500 1244.782000 405.689900
-84.915410 43.643920 2374.517000 631.115200
-83.596150 43.843030 3686.767000 1217.081000
-82.263960 44.027400 4947.218000 1726.841000
-80.919660 44.196660 6054.367000 2139.044000
-79.564150 44.350410 6320.705000 2247.231000

-78.198390	44.488330	5655.671000	2037.960000
-76.823410	44.610100	4582.604000	1617.419000
-75.440290	44.715440	3593.726000	1192.903000
-74.050180	44.804110	2465.239000	709.316500
-72.654240	44.875890	1114.514000	370.907500
-71.253690	44.930630	1116.941000	351.296400
-69.849760	44.968180	1739.056000	628.452200
-68.443720	44.988460	1405.919000	522.278300
-67.036810	44.991420	1066.420000	531.788300
-65.630330	44.977050	955.562100	367.474200
-64.225530	44.945390	1073.236000	374.910900
-62.823690	44.896510	850.864400	220.403700
-61.426040	44.830540	537.261100	197.711600
-60.033810	44.747610	377.501400	213.092400
-58.648170	44.647940	382.860200	180.381000
-57.270290	44.531750	381.009900	78.680820
-55.901260	44.399320	373.404000	44.369380
-54.542130	44.250930	351.953600	19.598520
-53.193900	44.086940	343.344600	13.951290
-51.857480	43.907680	336.755100	8.079276
-50.533710	43.713560	332.782100	3.990501
-49.223370	43.504980	330.801700	1.668366
-47.927120	43.282370	329.992800	.608316
-46.645540	43.046150	329.703700	.193886
-45.379100	42.796800	329.613700	.055211
-44.128160	42.534760	329.589300	.014099
-42.892960	42.260480	329.583600	.003260
-41.673610	41.974410	329.582400	.000917
-92.917770	43.067330	553.193900	169.729800
-91.673000	43.354680	747.494700	249.514400
-90.411740	43.629630	1303.815000	489.429000
-89.134110	43.891690	1973.164000	738.041300
-87.840450	44.140390	2196.726000	670.837400
-86.531140	44.375260	2403.816000	683.086800
-85.206750	44.595830	3277.473000	1052.219000
-83.867900	44.801650	4372.722000	1543.978000
-82.515380	44.992280	5384.869000	1957.577000
-81.150000	45.167310	6205.998000	2231.642000
-79.772730	45.326340	6216.090000	2237.961000
-78.384560	45.469020	5460.286000	2007.278000
-76.986600	45.595010	4229.078000	1459.417000
-75.579970	45.704020	2782.593000	741.451200
-74.165890	45.795780	1530.177000	303.865300
-72.745610	45.870080	590.340400	444.405300
-71.320400	45.926740	982.869200	337.835200
-69.891600	45.965610	1466.866000	467.913400
-68.460530	45.986610	1136.613000	365.964400
-67.028560	45.989670	841.722900	380.388700
-65.597030	45.974800	1191.758000	419.288800
-64.167300	45.942020	1344.442000	440.160200
-62.740720	45.891430	1251.938000	358.449900
-61.318600	45.823140	887.270300	183.052500
-59.902260	45.737310	595.785600	146.248600
-58.492950	45.634170	473.925000	115.306600
-57.091900	45.513950	442.497400	86.855420
-55.700260	45.376930	401.871200	55.175080
-54.319140	45.223450	386.430500	48.129600
-52.949590	45.053840	367.130900	34.085420
-51.592580	44.868490	348.277900	19.005610
-50.248970	44.667810	337.526900	9.006245
-48.919560	44.452230	332.489100	3.658132
-47.605040	44.222190	330.521900	1.300100
-46.305980	43.978160	329.855400	.409749
-45.022840	43.720610	329.652900	.114877
-43.755960	43.450020	329.598400	.029132
-42.505550	43.166850	329.585400	.006743
-41.271660	42.871580	329.582900	.001921

-93.329110	43.967430	563.893300	177.922100
-92.068780	44.264030	749.595400	252.959400
-90.791210	44.547890	1272.538000	456.892000
-89.496510	44.818510	2104.898000	807.357700
-88.184940	45.075390	2794.557000	1055.642000
-86.856880	45.318050	3195.187000	1137.194000
-85.512890	45.545990	3926.110000	1438.281000
-84.153590	45.758730	4802.226000	1797.895000
-82.779790	45.955820	5418.998000	2027.826000
-81.392350	46.136820	5703.167000	2105.174000
-79.992260	46.301310	5593.409000	2057.160000
-78.580580	46.448910	4991.936000	1849.180000
-77.158460	46.579270	3993.478000	1413.391000
-75.727110	46.692070	2806.862000	829.384300
-74.287820	46.787040	1664.240000	379.015800
-72.841890	46.863950	993.368000	466.746600
-71.390720	46.922590	1289.817000	487.395800
-69.935700	46.962830	1443.499000	460.832700
-68.478260	46.984570	1165.911000	308.404200
-67.019850	46.987740	1181.953000	282.706100
-65.561920	46.972340	1309.171000	342.244600
-64.105920	46.938410	1491.377000	461.396400
-62.653260	46.886040	1396.702000	395.002100
-61.205390	46.815350	986.904700	167.015000
-59.763680	46.726520	688.868200	166.506600
-58.329470	46.619790	579.871800	117.434100
-56.904060	46.495390	504.996100	100.371700
-55.488670	46.353640	485.903900	110.311600
-54.084500	46.194880	479.281500	108.036800
-52.692630	46.019470	431.603300	79.156850
-51.314070	45.827840	374.858600	39.755910
-49.949730	45.620390	346.377700	17.101460
-48.600430	45.397590	335.375600	6.695815
-47.266860	45.159900	331.374200	2.312807
-45.949600	44.907820	330.084100	.711537
-44.649100	44.641840	329.709900	.197172
-43.365670	44.362450	329.611300	.049359
-42.099450	44.070140	329.588100	.011396
-40.850440	43.765410	329.583600	.003225
-93.760410	44.864160	552.583100	167.768800
-92.483920	45.170280	688.580600	221.710000
-91.189420	45.463320	1010.086000	354.263700
-89.876960	45.742750	1594.216000	566.969400
-88.546760	46.008070	2202.668000	787.441000
-87.199170	46.258760	2675.532000	941.597600
-85.834720	46.494300	3183.778000	1112.814000
-84.454070	46.714200	4042.486000	1498.510000
-83.058010	46.917960	4644.195000	1733.224000
-81.647480	47.105130	4864.694000	1804.663000
-80.223460	47.275260	4600.892000	1689.516000
-78.787090	47.427960	3661.916000	1243.947000
-77.339580	47.562840	2833.021000	921.039700
-75.882230	47.679570	2367.929000	773.258200
-74.416370	47.777870	2126.246000	708.530200
-72.943440	47.857470	1880.545000	688.035800
-71.464880	47.918190	1700.295000	580.891400
-69.982220	47.959850	1521.701000	456.607900
-68.496960	47.982350	1268.523000	259.772500
-67.010670	47.985630	1421.252000	262.803700
-65.524890	47.969690	1615.057000	419.950300
-64.041170	47.934570	1618.689000	432.532900
-62.561040	47.880340	1434.578000	331.932000
-61.086020	47.807170	1140.905000	173.701000
-59.617590	47.715230	914.191000	151.777700
-58.157170	47.604770	777.524200	125.983400
-56.706150	47.476050	685.230400	163.381900
-55.265830	47.329400	692.006400	209.310900

-53.837470	47.165180	674.203900	219.968900
-52.422200	46.983780	546.216700	149.778300
-51.021090	46.785640	420.490200	70.569490
-49.635090	46.571200	359.038200	27.011260
-48.265020	46.340950	338.667700	9.781364
-46.911610	46.095370	332.255000	3.279093
-45.575410	45.834990	330.309100	.991638
-44.256860	45.560320	329.763100	.269754
-42.956210	45.271870	329.623000	.066876
-41.673580	44.970160	329.590500	.015296
-40.408870	44.655690	329.584100	.004306
-94.212860	45.757310	546.969100	156.828800
-92.919560	46.073230	647.086900	191.520400
-91.607470	46.375730	819.989200	269.639200
-90.276550	46.664250	1129.442000	391.952600
-88.926960	46.938270	1333.376000	400.600900
-87.559010	47.197250	1456.246000	399.633200
-86.173240	47.440650	1911.987000	567.146400
-84.770290	47.667940	2764.901000	906.801100
-83.350960	47.878600	3685.354000	1327.443000
-81.916200	48.072160	4141.593000	1533.569000
-80.467090	48.248140	3581.411000	1247.666000
-79.004780	48.406110	2332.254000	742.150100
-77.530590	48.545680	1586.867000	593.681600
-76.045850	48.666500	1564.563000	513.839100
-74.552020	48.768250	1809.404000	578.345900
-73.050600	48.850660	1843.166000	605.738300
-71.543170	48.913520	1739.066000	556.063900
-70.031330	48.956650	1558.829000	486.521600
-68.516710	48.979950	1405.159000	388.361600
-67.000980	48.983350	1394.900000	376.333600
-65.485790	48.966840	1434.620000	349.702100
-63.972820	48.930470	1600.368000	457.787800
-62.463700	48.874330	1545.797000	401.129000
-60.960050	48.798580	1481.313000	319.042100
-59.463460	48.703410	1435.200000	289.892800
-57.975440	48.589080	1287.559000	297.517500
-56.497490	48.455880	1051.165000	304.577500
-55.030970	48.304140	942.344800	340.990700
-53.577210	48.134270	840.873400	316.212900
-52.137430	47.946660	625.237500	194.149700
-50.712720	47.741790	437.878100	80.293970
-49.304080	47.520120	361.827300	29.557650
-47.912350	47.282170	339.557200	10.777860
-46.538250	47.028450	332.498900	3.569977
-45.182340	46.759510	330.361600	1.063597
-43.845020	46.475880	329.775000	.288384
-42.526500	46.178100	329.625500	.070815
-41.226800	45.866700	329.590900	.016120
-39.945740	45.542200	329.584300	.004514
-94.687740	46.646660	558.610400	156.197300
-93.376940	46.972690	646.676100	184.861400
-92.046550	47.284930	739.354300	239.563300
-90.696440	47.582840	710.534500	211.325800
-89.326680	47.865840	577.616300	190.342700
-87.937550	48.133390	673.070900	275.732400
-86.529520	48.384910	1284.617000	332.566200
-85.103260	48.619850	2267.893000	698.114000
-83.659580	48.837660	3208.483000	1149.284000
-82.199460	49.037830	3460.803000	1283.526000
-80.724000	49.219880	2886.944000	1021.507000
-79.234450	49.383340	1741.110000	593.523400
-77.732160	49.527770	1390.568000	475.591100
-76.218590	49.652830	1528.598000	491.268700
-74.695260	49.758160	1502.958000	435.043700
-73.163800	49.843490	1381.470000	367.071900
-71.625870	49.908580	1408.562000	378.435900

-70.083210	49.953250	1475.315000	458.174200
-68.537570	49.977380	1270.533000	406.224300
-66.990740	49.980900	1108.435000	329.326200
-65.444490	49.963800	1152.064000	365.393500
-63.900620	49.926140	1188.913000	358.474500
-62.360880	49.868010	1244.810000	383.978700
-60.827010	49.789570	1471.396000	467.992800
-59.300730	49.691040	1617.642000	524.891400
-57.783640	49.572690	1431.042000	445.631200
-56.277320	49.434830	1117.381000	382.154800
-54.783270	49.277820	877.054900	320.753700
-53.302860	49.102080	711.442900	248.673600
-51.837360	48.908040	526.171000	142.510400
-50.387950	48.696200	400.598700	60.591260
-48.955640	48.467040	352.172200	23.629570
-47.541310	48.221130	337.075200	8.926087
-46.145660	47.959000	331.876100	2.991109
-44.769230	47.681210	330.206900	.895202
-43.412380	47.388330	329.737300	.241028
-42.075270	47.080920	329.616900	.059222
-40.757810	46.759540	329.589100	.013384
-39.459720	46.424710	329.583800	.003735
-95.186440	47.531940	594.772900	169.067700
-93.857410	47.868410	697.836200	205.560100
-92.508000	48.190720	739.236000	239.918000
-91.137910	48.498310	647.869900	182.872300
-89.747150	48.790600	464.580200	171.506200
-88.335950	49.067010	710.239900	200.050000
-86.904710	49.326930	1370.384000	392.574800
-85.454100	49.569790	2101.106000	738.361400
-83.984930	49.795020	2579.469000	967.821600
-82.498220	50.002060	2616.741000	1009.452000
-80.995120	50.190400	2184.686000	830.829400
-79.476920	50.359550	1627.897000	571.801800
-77.945060	50.509060	1602.201000	575.479600
-76.401090	50.638520	1639.768000	574.159300
-74.846650	50.747580	1465.812000	445.104400
-73.283450	50.835950	1279.465000	341.333600
-71.713320	50.903350	1310.706000	357.428100
-70.138080	50.949630	1412.425000	413.058300
-68.559640	50.974620	1295.181000	412.967700
-66.979900	50.978270	1049.984000	350.764600
-65.400800	50.960560	938.704000	283.166000
-63.824260	50.921550	878.039400	293.584300
-62.252170	50.861340	676.570100	279.672300
-60.686400	50.780110	1102.994000	400.575400
-59.128770	50.678080	1275.571000	463.774700
-57.581030	50.555550	1125.640000	389.937700
-56.044860	50.412850	797.507400	263.678600
-54.521820	50.250360	608.773200	189.205900
-53.013420	50.068520	510.696300	138.242800
-51.520960	49.867810	424.257200	79.830930
-50.045680	49.648740	370.289200	39.760930
-48.588630	49.411830	345.555600	17.700430
-47.150700	49.157670	334.738600	6.288071
-45.732590	48.886830	331.079600	2.063538
-44.334810	48.599900	329.992800	.617690
-42.957660	48.297460	329.684000	.166013
-41.601190	47.980120	329.604500	.040240
-40.265250	47.648430	329.586500	.009064
-38.949390	47.302930	329.583200	.002492
-95.710510	48.412890	651.760300	189.309200
-94.362470	48.760130	772.304500	238.147500
-92.993230	49.092850	782.013100	251.502800
-91.602360	49.410460	701.658900	213.311900
-90.189750	49.712350	624.334200	190.272700
-88.755540	49.997940	789.315600	238.266100

-87.300090	50.266570	1192.339000	419.740900
-85.824040	50.517650	1554.443000	584.788600
-84.328210	50.750570	1779.164000	701.859300
-82.813610	50.964740	1697.655000	692.372800
-81.281460	51.159630	1518.314000	606.363400
-79.733130	51.334700	1356.438000	511.704400
-78.170130	51.489480	1389.713000	520.473600
-76.594090	51.623530	1377.029000	489.118200
-75.006800	51.736490	1252.664000	401.819500
-73.410070	51.828010	1138.314000	334.619000
-71.805870	51.897850	1149.716000	320.935200
-70.196170	51.945790	1209.933000	330.670000
-68.582990	51.971680	1145.090000	357.089200
-66.968440	51.975460	921.051400	332.584400
-65.354560	51.957120	996.108200	363.916300
-63.743440	51.916690	917.061300	271.646400
-62.137130	51.854320	789.040700	211.514200
-60.537630	51.770170	873.646100	279.504700
-58.946900	51.664510	907.159900	331.277400
-57.366820	51.537620	698.959500	222.805700
-55.799180	51.389870	538.245400	149.435500
-54.245650	51.221680	454.007700	101.433600
-52.707820	51.033510	404.318400	66.596230
-51.187080	50.825860	370.705200	40.263740
-49.684720	50.599280	349.707000	21.842580
-48.201800	50.354330	337.384400	9.260479
-46.739240	50.091630	332.165200	3.462314
-45.297720	49.811770	330.382400	1.176015
-43.877700	49.515370	329.803700	.351295
-42.479410	49.203060	329.636300	.093484
-41.102810	48.875440	329.593900	.022829
-39.747590	48.533100	329.584400	.005032
-38.413150	48.176570	329.582600	.001360
-96.261670	49.289190	702.940900	206.418600
-94.893750	49.647600	793.088100	250.447600
-93.503820	49.991080	754.891500	247.878000
-92.091320	50.319060	630.232300	247.698900
-90.655950	50.630910	575.857700	230.134200
-89.197760	50.925990	632.760700	198.407700
-87.717060	51.203660	795.344200	262.558100
-86.214420	51.463270	1040.814000	387.910600
-84.690660	51.704190	1300.223000	516.571700
-83.146820	51.925780	1329.877000	531.202000
-81.584160	52.127480	1189.214000	454.473200
-80.004110	52.308710	1053.890000	390.494700
-78.408280	52.468990	991.229400	380.796800
-76.798410	52.607840	1046.709000	388.226500
-75.176390	52.724850	1084.715000	362.463000
-73.544200	52.819690	1021.413000	301.971600
-71.903920	52.892050	938.517000	256.323300
-70.257710	52.941730	956.152600	223.566800
-68.607750	52.968570	805.090300	213.768300
-66.956280	52.972490	434.424800	238.051000
-65.305560	52.953480	560.451700	195.360900
-63.657820	52.911580	817.783300	257.742700
-62.015270	52.846950	710.900900	175.058200
-60.380080	52.759750	724.571200	220.296300
-58.754360	52.650280	603.960600	171.393500
-57.140120	52.518850	488.093800	118.617900
-55.539290	52.365840	423.678000	80.246040
-53.953660	52.191710	383.551200	50.922210
-52.384890	51.996940	359.648700	31.367200
-50.834480	51.782070	345.710200	18.144580
-49.303730	51.547700	337.350700	9.425896
-47.793770	51.294400	332.704700	4.168205
-46.305490	51.022820	330.703900	1.631687
-44.839570	50.733620	329.935700	.553324

-43.396390	50.427420	329.679400	.164929
-41.976090	50.104880	329.605900	.044681
-40.578500	49.766630	329.587200	.010757
-39.203150	49.413260	329.583000	.002325
-37.849210	49.045330	329.582300	.000603
-96.841810	50.160530	622.076800	170.241400
-95.453030	50.530490	669.553800	195.516600
-94.041510	50.885130	605.730300	182.638500
-92.606450	51.223850	517.243200	155.642800
-91.147380	51.546020	475.426300	126.130200
-89.664200	51.850960	482.030800	113.737100
-88.157130	52.138020	551.479400	158.195600
-86.626690	52.406500	693.575700	237.176200
-85.073680	52.655720	950.403700	352.830300
-83.499180	52.885050	1032.202000	389.099800
-81.904440	53.093850	885.059500	322.079400
-80.290990	53.281520	684.767800	257.281900
-78.660530	53.447530	605.308700	266.731500
-77.014920	53.591380	793.205600	304.740100
-75.356160	53.712650	977.754300	334.331100
-73.686420	53.810940	1007.092000	305.324500
-72.007930	53.885960	921.456100	256.078800
-70.322990	53.937460	860.822600	209.989300
-68.634010	53.965280	718.442100	220.411400
-66.943400	53.969350	346.620800	274.595800
-65.253590	53.949630	428.206800	149.096300
-63.567000	53.906200	631.943000	159.866200
-61.886040	53.839190	637.580600	164.315900
-60.213040	53.748820	549.143200	131.311200
-58.550290	53.635360	452.461300	91.833060
-56.899960	53.499180	400.947100	62.749250
-55.264110	53.340690	370.042700	39.375010
-53.644650	53.160350	351.404300	23.711370
-52.043340	52.958710	341.193800	13.769210
-50.461760	52.736320	335.497500	7.519546
-48.901280	52.493820	332.348800	3.764352
-47.363030	52.231850	330.728800	1.677889
-45.847900	51.951060	329.997100	.651129
-44.356530	51.652150	329.712400	.220588
-42.889220	51.335790	329.618200	.066993
-41.445980	51.002650	329.590900	.017934
-40.026490	50.653380	329.583900	.004252
-38.630060	50.288590	329.582300	.000877
-37.255620	49.908830	329.582100	.000220
-97.453060	51.026530	486.661700	101.314400
-96.042340	51.408490	498.647500	108.633500
-94.608220	51.774700	454.903600	89.389990
-93.149600	52.124570	422.795500	73.109790
-91.665800	52.457450	404.561400	63.343110
-90.156560	52.772640	405.203500	65.806790
-88.621980	53.069450	431.748600	87.648030
-87.062480	53.347160	485.562300	124.186300
-85.478850	53.605050	588.616600	175.400600
-83.872130	53.842430	668.968300	214.061500
-82.243660	54.058630	548.147700	179.773300
-80.595020	54.253030	404.752400	155.969000
-78.928000	54.425040	383.729800	179.917000
-77.244600	54.574130	603.006000	216.556900
-75.546970	54.699840	846.311700	293.636000
-73.837420	54.801750	959.828700	303.295200
-72.118360	54.879540	920.441700	270.339400
-70.392330	54.932960	846.769200	227.274700
-68.661900	54.961820	805.615300	243.929800
-66.929700	54.966030	658.114900	219.277600
-65.198370	54.945580	636.567600	180.852000
-63.470540	54.900540	628.748800	164.374600
-61.748820	54.831050	540.186900	126.110400

-60.035740	54.737340	440.573800	81.561440
-58.333780	54.619720	388.972700	52.585690
-56.645260	54.478570	361.925100	32.102630
-54.972420	54.314330	346.349200	18.606640
-53.317280	54.127510	338.095900	10.412910
-51.681740	53.918690	333.825500	5.593046
-50.067420	53.688470	331.620600	2.869199
-48.475770	53.437500	330.499300	1.375144
-46.907930	53.166490	329.954300	.595056
-45.364770	52.876130	329.714800	.227665
-43.846840	52.567130	329.623800	.077621
-42.354370	52.240220	329.593500	.023234
-40.887210	51.896080	329.584800	.006130
-39.444820	51.535370	329.582600	.001395
-38.026250	51.158730	329.582100	.000272
-36.630120	50.766700	329.582100	.000067
-98.097820	51.886770	397.549500	53.750100
-96.663910	52.281200	395.746900	50.701110
-95.206040	52.659450	382.493700	43.829690
-93.722790	53.020900	370.997700	37.006720
-92.213200	53.364910	363.207100	32.142460
-90.676770	53.690770	362.568700	33.102330
-89.113440	53.997730	371.797300	42.113860
-87.523560	54.285050	390.909300	59.124840
-85.907830	54.551970	416.091800	79.113690
-84.267300	54.797760	432.127100	97.420140
-82.603340	55.021710	367.745800	109.632300
-80.917590	55.223140	272.638800	110.429800
-79.211960	55.401440	296.366100	137.104500
-77.488560	55.556020	476.939600	144.976500
-75.749730	55.686380	709.024000	231.086000
-73.997920	55.792100	909.631500	299.561400
-72.235800	55.872810	928.442800	278.394700
-70.466090	55.928230	845.896700	228.534100
-68.691570	55.958180	878.227200	242.186400
-66.915130	55.962550	878.205900	270.494200
-65.139640	55.941330	752.443100	209.155000
-63.367970	55.894590	599.766900	143.823300
-61.602940	55.822490	463.837600	93.246270
-59.847310	55.725280	395.441400	56.762220
-58.103760	55.603290	362.763000	32.629870
-56.374820	55.456930	345.456500	17.568190
-54.662870	55.286670	336.954200	9.107219
-52.970100	55.093080	332.957200	4.547654
-51.298500	54.876750	331.100600	2.209739
-49.649800	54.638340	330.249400	1.040608
-48.025460	54.378550	329.862400	.465758
-46.426660	54.098120	329.690500	.192169
-44.854210	53.797780	329.619800	.071877
-43.308580	53.478290	329.593700	.023856
-41.789850	53.140400	329.585100	.006991
-40.297680	52.784830	329.582700	.001780
-38.831280	52.412250	329.582200	.000382
-37.389370	52.023280	329.582000	.000075
-35.970190	51.618480	329.582100	.000031
-98.778840	52.740810	357.603800	26.006210
-97.320270	53.148240	354.794900	23.461730
-95.837370	53.539010	350.562800	20.206940
-94.328290	53.912530	346.047100	16.720290
-92.791720	54.268120	342.803300	14.351120
-91.226900	54.605080	342.043600	14.368240
-89.633560	54.922620	344.463000	17.828390
-88.011890	55.219970	349.948000	25.555460
-86.362530	55.496330	354.743600	37.499280
-84.686490	55.750910	348.966400	55.798220
-82.985150	55.982970	296.500600	101.645400
-81.260250	56.191770	209.560400	105.964300

-79.513790 56.376650 278.700400 149.322900
-77.748020 56.536990 398.340400 99.918850
-75.965460 56.672260 576.506000 161.445200
-74.168790 56.781970 845.477700 270.805100
-72.360860 56.865740 954.488300 291.833600
-70.544630 56.923280 908.112400 242.232300
-68.723180 56.954370 941.648400 258.435400
-66.899620 56.958910 930.369100 286.331700
-65.077100 56.936880 733.773100 200.996900
-63.258730 56.888360 551.005400 127.786000
-61.447630 56.813510 442.044100 78.607880
-59.646790 56.712620 382.301400 46.785240
-57.859090 56.586040 353.600200 24.814880
-56.087300 56.434210 339.925400 11.996350
-54.333970 56.257640 333.783500 5.400040
-52.601470 56.056940 331.221500 2.325529
-50.891880 55.832740 330.205800 .971738
-49.207030 55.585770 329.815900 .398257
-47.548420 55.316760 329.667800 .158114
-45.917200 55.026500 329.612200 .059652
-44.314130 54.715760 329.591800 .020809
-42.739580 54.385350 329.584900 .006587
-41.193420 54.036030 329.582700 .001828
-39.675070 53.668560 329.582200 .000444
-38.183400 53.283620 329.582000 .000084
-36.716770 52.881820 329.582000 .000013
-35.272900 52.463700 329.582100 .000031
-99.499240 53.588130 340.218800 11.229520
-98.014280 54.009130 338.677600 9.825473
-96.504860 54.412960 337.069900 8.270766
-94.968610 54.799050 335.396300 6.729331
-93.403790 55.166730 334.112100 5.560849
-91.809290 55.515250 333.504200 5.215551
-90.184560 55.843850 333.540600 6.657390
-88.529620 56.151670 333.626400 11.750120
-86.844990 56.437890 331.693300 21.873580
-85.131650 56.701690 321.659800 45.805540
-83.390960 56.942230 266.711500 104.498800
-81.624720 57.158780 183.849200 98.970540
-79.835050 57.350580 276.051600 156.922700
-78.024370 57.516980 346.555100 82.588210
-76.195360 57.657400 456.210700 126.740700
-74.350940 57.771310 741.905800 213.934600
-72.494220 57.858320 968.136500 288.289700
-70.628410 57.918090 1010.446000 277.079700
-68.756890 57.950390 998.850100 282.771500
-66.883060 57.955100 901.590400 280.816200
-65.010370 57.932220 668.843500 189.365200
-63.142240 57.881810 523.307900 118.485900
-61.282040 57.804080 454.182200 89.055530
-59.433060 57.699310 385.791500 45.274960
-57.598450 57.567890 353.687700 23.997340
-55.781200 57.410300 339.484300 11.067980
-53.984060 57.227100 333.316400 4.550837
-52.209560 57.018940 330.858600 1.711302
-50.459930 56.786500 329.990400 .608120
-48.737060 56.530560 329.706100 .205602
-47.042490 56.251900 329.618400 .067328
-45.377330 55.951360 329.592300 .021259
-43.742270 55.629770 329.584800 .006401
-42.137470 55.287960 329.582700 .001782
-40.562610 54.926740 329.582200 .000440
-39.016750 54.546860 329.582000 .000098
-37.498410 54.149020 329.582000 .000000
-36.005400 53.733830 329.582000 .000000
-34.534880 53.301770 329.582100 .000031
-100.262700 54.428120 333.237000 4.386475

-98.749250	54.863350	332.599100	3.702109
-97.211560	55.280850	331.996700	3.030090
-95.646590	55.680060	331.402300	2.365551
-94.052090	56.060360	330.854200	1.742324
-92.426510	56.420970	330.296800	1.455339
-90.768940	56.761100	329.434200	3.047953
-89.079190	57.079890	327.568700	8.014436
-87.357570	57.376450	322.682800	17.687090
-85.605000	57.649890	309.810000	42.272210
-83.822840	57.899360	256.353000	101.199600
-82.012920	58.124030	170.835300	94.492110
-80.177480	58.323120	278.920200	144.477000
-78.319110	58.495910	328.755500	70.202700
-76.440710	58.641750	392.346800	119.178900
-74.545430	58.760110	662.715600	166.245500
-72.636660	58.850530	981.851500	282.643400
-70.717930	58.912650	1088.419000	312.110300
-68.792920	58.946230	1039.549000	304.321600
-66.865380	58.951130	846.840900	265.560200
-64.939080	58.927340	606.852700	170.875500
-63.017780	58.874950	498.380600	114.164100
-61.105210	58.794160	450.628700	83.829080
-59.204930	58.685300	407.834000	68.512570
-57.320400	58.548780	359.345200	25.597180
-55.454840	58.385130	340.929000	11.536840
-53.611270	58.194950	333.682900	4.663021
-51.792370	57.978940	330.938100	1.669534
-50.000500	57.737840	329.985500	.542897
-48.237630	57.472490	329.692600	.164728
-46.505310	57.183730	329.610200	.046436
-44.804610	56.872430	329.588600	.012247
-43.136050	56.539500	329.583400	.003006
-41.499610	56.185780	329.582300	.000682
-39.894630	55.812120	329.582000	.000127
-38.319800	55.419290	329.582000	.000013
-36.773090	55.007980	329.582000	.000000
-35.251730	54.578750	329.582000	.000000
-33.752120	54.132060	329.582100	.000031
-101.073700	55.260130	330.738700	1.549223
-99.529010	55.710320	330.493000	1.251727
-97.960900	56.142140	330.282000	.978419
-96.365430	56.555100	330.055700	.673638
-94.739640	56.948590	329.748100	.326786
-93.081440	57.321860	329.175200	.893665
-91.389490	57.674070	327.900200	2.958349
-89.663250	58.004340	325.096000	7.602232
-87.902820	58.311730	318.651600	16.673450
-86.109000	58.595320	303.516400	40.356100
-84.283100	58.854170	256.641600	102.093600
-82.427000	59.087400	169.863300	108.670000
-80.543050	59.294160	295.707100	133.476000
-78.634010	59.473690	351.822500	71.901230
-76.703000	59.625280	432.207200	87.917260
-74.753460	59.748340	694.222800	169.723000
-72.789070	59.842370	968.516400	286.941800
-70.813740	59.906970	1138.333000	343.912700
-68.831480	59.941910	1063.632000	321.465600
-66.846440	59.947010	816.462500	250.885300
-64.862770	59.922250	563.944500	148.749500
-62.884610	59.867760	442.005400	79.442920
-60.916040	59.783740	418.987500	82.258930
-58.961010	59.670550	394.110600	57.196150
-57.023270	59.528640	366.400300	36.471550
-55.106350	59.358580	341.889500	10.689960
-53.213500	59.161040	333.491600	4.054532
-51.347600	58.936760	330.836200	1.464790
-49.511150	58.686560	329.955900	.473052

-47.706190 58.411320 329.683300 .140265
-45.934240 58.111950 329.607100 .038469
-44.196280 57.789400 329.587600 .009688
-42.492650 57.444580 329.583100 .002212
-40.823030 57.078400 329.582200 .000437
-39.186360 56.691730 329.582000 .000071
-37.580830 56.285340 329.582000 .000000
-36.003750 55.859910 329.582000 .000000
-34.451600 55.415950 329.582000 .000000
-32.919830 54.953830 329.582100 .000031
-101.937200 56.083360 329.915300 .492923
-100.358100 56.549350 329.830000 .377367
-98.756940 56.996240 329.753800 .262470
-97.128790 57.423640 329.642700 .100420
-95.469860 57.830970 329.398700 .326239
-93.777320 58.217490 328.775400 1.204594
-92.049300 58.582370 327.215900 3.202216
-90.284800 58.924680 323.650100 7.516994
-88.483640 59.243460 315.795700 16.211050
-86.646400 59.537720 299.919000 39.832380
-84.774360 59.806450 256.542000 92.493010
-82.869400 60.048700 192.249100 121.876000
-80.933970 60.263580 310.796000 126.344600
-78.971000 60.450230 372.486200 96.345950
-76.983890 60.607900 527.240700 126.203300
-74.976370 60.735940 686.906300 177.876400
-72.952470 60.833800 926.836400 289.397600
-70.916480 60.901050 1093.492000 347.924800
-68.872850 60.937420 1094.347000 339.396200
-66.826130 60.942730 805.651300 236.117900
-64.780930 60.916960 516.700800 123.897100
-62.741830 60.860230 356.071500 51.298020
-60.713310 60.772790 313.603600 37.546630
-58.699720 60.654990 329.399500 32.001200
-56.705200 60.507380 335.630000 12.619290
-54.733590 60.330550 333.487400 4.470996
-52.788400 60.125220 331.143200 1.933783
-50.872730 59.892220 330.196300 .771947
-48.989200 59.632420 329.789600 .265309
-47.139900 59.346780 329.642900 .082865
-45.326320 59.036270 329.598000 .024019
-43.549270 58.701890 329.585800 .006257
-41.808860 58.344600 329.582800 .001451
-40.104370 57.965370 329.582200 .000284
-38.434220 57.565060 329.582000 .000048
-36.795950 57.144450 329.582000 .000000
-35.186060 56.704150 329.582000 .000000
-33.600030 56.244660 329.582000 .000000
-32.032190 55.766200 329.582100 .000031
-102.859900 56.896880 329.668500 .140866
-101.242000 57.379640 329.640400 .097463
-99.604390 57.842470 329.606700 .040379
-97.940930 58.285070 329.527100 .103124
-96.246660 58.706940 329.290400 .454230
-94.517820 59.107400 328.604600 1.323351
-92.751850 59.485580 326.761100 3.284909
-90.947170 59.840560 322.329800 7.398225
-89.103220 60.171320 312.837400 16.195000
-87.220290 60.476800 293.912900 37.440620
-85.299550 60.755960 248.582700 86.119080
-83.342830 61.007760 220.980300 141.787300
-81.352710 61.231220 327.926800 97.853000
-79.332320 61.425420 457.432200 134.884400
-77.285290 61.589540 550.757400 142.317500
-75.215710 61.722870 671.468000 196.031700
-73.128000 61.824790 770.493400 244.854100
-71.026890 61.894870 1007.823000 328.289800

-68.917300	61.932760	1118.521000	360.787800
-66.804310	61.938290	801.842000	233.410200
-64.692990	61.911450	454.146600	106.580900
-62.588420	61.852340	266.508800	82.224350
-60.495590	61.761240	215.046900	101.050500
-58.419300	61.638580	262.031200	53.945080
-56.364090	61.484900	305.925100	20.981850
-54.334160	61.300890	324.161400	5.778084
-52.333320	61.087320	328.606100	1.492114
-50.364880	60.845090	329.467800	.339294
-48.431600	60.575160	329.590900	.062809
-46.535590	60.278550	329.593200	.018190
-44.678250	59.956310	329.586400	.006750
-42.860180	59.609490	329.583300	.002215
-41.081100	59.239120	329.582300	.000585
-39.339830	58.846180	329.582100	.000121
-37.634110	58.431540	329.582000	.000013
-35.960610	57.995940	329.582000	.000000
-34.314830	57.539980	329.582000	.000000
-32.690990	57.064010	329.582000	.000000
-31.082000	56.568130	329.582100	.000031
-103.849600	57.699570	329.601700	.035054
-102.187300	58.200280	329.591600	.017137
-100.509000	58.680030	329.570300	.023756
-98.806840	59.138710	329.499400	.139522
-97.074520	59.575940	329.263300	.466109
-95.307120	59.991050	328.536700	1.309291
-93.501110	60.383240	326.477300	3.244439
-91.654170	60.751550	321.364600	7.278984
-89.765210	61.094930	309.933600	15.762070
-87.834170	61.412270	280.116300	31.473830
-85.861950	61.702450	238.723100	79.988670
-83.850420	61.964360	262.168900	186.277000
-81.802150	62.196920	405.821400	128.857300
-79.720490	62.399150	503.934300	149.974100
-77.609360	62.570130	569.930200	165.150800
-75.473210	62.709080	552.145600	144.901900
-73.316960	62.815350	623.976100	187.222800
-71.145790	62.888430	878.004200	279.669900
-68.965200	62.927950	1044.494000	345.061600
-66.780790	62.933720	767.684300	227.424800
-64.598260	62.905720	431.043800	97.683070
-62.423240	62.844070	273.379600	72.889210
-60.261290	62.749080	251.653900	63.926460
-58.117710	62.621230	285.573200	37.598410
-55.997520	62.461110	312.481600	16.509770
-53.905320	62.269450	324.250600	5.949229
-51.845240	62.047150	328.093300	1.857919
-49.820820	61.795150	329.220900	.492276
-47.834930	61.514500	329.505900	.113684
-45.889670	61.206310	329.567700	.022806
-43.986290	60.871700	329.579900	.003699
-42.125070	60.511780	329.581800	.000459
-40.305270	60.127630	329.582000	.000049
-38.525020	59.720250	329.582000	.000000
-36.781200	59.290500	329.582000	.000000
-35.069400	58.839090	329.582000	.000000
-33.383770	58.366500	329.582000	.000000
-31.716990	57.872960	329.582000	.000000
-30.060130	57.358350	329.582100	.000031
-104.916500	58.490070	329.585400	.006418
-103.202300	59.010120	329.580600	.003426
-101.477600	59.507980	329.563800	.033718
-99.732440	59.983750	329.500500	.132317
-97.958720	60.437220	329.282300	.420409
-96.150010	60.867820	328.593900	1.176987
-94.301540	61.274790	326.594200	2.920399

-92.410060	61.657170	321.523300	6.780256
-90.473700	62.013880	309.685400	14.352080
-88.491920	62.343760	276.886800	28.259750
-86.465330	62.645610	230.256600	76.235070
-84.395630	62.918240	306.927200	109.699600
-82.285480	63.160470	453.979800	145.687200
-80.138370	63.371240	528.085000	164.095900
-77.958560	63.549540	496.607500	118.819900
-75.750930	63.694510	455.127200	95.244260
-73.520860	63.805420	498.263800	121.107500
-71.274150	63.881700	661.210400	186.774000
-69.016910	63.922960	812.947800	248.617500
-66.755400	63.928990	629.244400	166.576900
-64.495980	63.899750	412.456800	72.892380
-62.244980	63.835380	320.541300	33.807160
-60.008560	63.736250	307.033200	25.560620
-57.792640	63.602840	312.191400	18.118220
-55.602750	63.435840	321.360500	9.215967
-53.443960	63.236060	326.425900	3.806806
-51.320750	63.004450	328.569100	1.319189
-49.236890	62.742080	329.297500	.402979
-47.195330	62.450080	329.509100	.110861
-45.198120	62.129630	329.565500	.027078
-43.246250	61.781950	329.578700	.006001
-41.339580	61.408210	329.581400	.001171
-39.476710	61.009530	329.581900	.000211
-37.654880	60.586900	329.582000	.000066
-35.869840	60.141170	329.582000	.000000
-34.115770	59.672970	329.582000	.000000
-32.385130	59.182660	329.582000	.000000
-30.668580	58.670220	329.582100	.000031
-28.954870	58.135290	329.582100	.000031
-106.073800	59.266680	329.581900	.000553
-104.297500	59.807790	329.578900	.006656
-102.518800	60.325180	329.565300	.030666
-100.725000	60.819250	329.513100	.110517
-98.905530	61.289990	329.333000	.343944
-97.052100	61.737010	328.765600	.961076
-95.158340	62.159620	327.141700	2.394669
-93.219700	62.556880	322.933400	5.419473
-91.233350	62.927700	312.939400	11.371660
-89.198040	63.270870	282.826000	25.194690
-87.113920	63.585110	258.108400	57.200450
-84.982480	63.869130	348.661100	75.451740
-82.806390	64.121680	461.901200	130.255800
-80.589290	64.341560	475.527500	114.325200
-78.335770	64.527670	412.807900	64.158470
-76.051160	64.679080	393.373200	55.861510
-73.741460	64.794950	412.261900	68.639700
-71.413090	64.874680	470.027200	95.437740
-69.072890	64.917820	544.824300	122.524500
-66.727910	64.924110	461.941800	85.171200
-64.385280	64.893550	378.712000	41.319100
-62.052090	64.826280	337.898400	15.335220
-59.735260	64.722690	324.681200	9.490343
-57.441380	64.583340	324.023000	7.114175
-55.176610	64.408980	326.356100	4.075769
-52.946510	64.200520	328.199400	1.840796
-50.755950	63.959000	329.088700	.702909
-48.608900	63.685590	329.426500	.235710
-46.508400	63.381530	329.539200	.069685
-44.456330	63.048090	329.571600	.018626
-42.453320	62.686580	329.579800	.004327
-40.498620	62.298210	329.581600	.000897
-38.589950	61.884150	329.581900	.000177
-36.723370	61.445380	329.582000	.000063
-34.893150	60.982680	329.582100	.000036

-33.091610	60.496560	329.582000	.000000
-31.309040	59.987160	329.582000	.000000
-29.533510	59.454220	329.582100	.000031
-27.750760	58.896990	329.582100	.000031
-107.339200	60.027230	329.581300	.001813
-105.487000	60.591610	329.578900	.006656
-103.643800	61.130270	329.568000	.026394
-101.793500	61.644070	329.526500	.090696
-99.922550	62.133320	329.388200	.274038
-98.020040	62.597820	328.974600	.742201
-96.077510	63.037030	327.837400	1.804216
-94.088710	63.450070	325.153700	3.832882
-92.049460	63.835860	319.174300	7.674660
-89.957580	64.193130	307.262000	13.673210
-87.812570	64.520550	310.001200	20.170740
-85.615560	64.816710	369.462000	47.248870
-83.369120	65.080260	428.755500	85.757910
-81.077050	65.309890	392.611500	50.201630
-78.744270	65.504390	368.049400	34.472390
-76.376630	65.662700	361.525000	31.710330
-73.980770	65.783920	367.788300	36.487150
-71.563890	65.867360	384.289900	46.148700
-69.133670	65.912500	401.861700	52.431380
-66.698060	65.919100	379.699500	40.232080
-64.265090	65.887100	353.783600	22.394630
-61.842790	65.816700	336.798000	9.165425
-59.438900	65.708320	329.757000	3.680969
-57.060850	65.562580	328.231700	2.456779
-54.715470	65.380330	328.562300	1.531509
-52.408880	65.162570	329.072300	.763089
-50.146370	64.910470	329.376300	.318767
-47.932120	64.625290	329.512700	.114377
-45.769150	64.308400	329.561900	.036283
-43.659080	63.961160	329.577100	.009861
-41.602020	63.584970	329.581000	.002374
-39.596350	63.181100	329.581800	.000531
-37.638600	62.750720	329.582000	.000103
-35.723270	62.294770	329.582000	.000061
-33.842640	61.813940	329.582100	.000031
-31.986660	61.308540	329.582100	.000031
-30.142740	60.778430	329.582000	.000000
-28.295590	60.222970	329.582100	.000031
-26.427080	59.640870	329.582100	.000031
-108.736700	60.768890	329.581000	.002514
-106.789100	61.359350	329.578500	.007470
-104.866900	61.921510	329.567400	.027175
-102.949500	62.456830	329.528000	.087871
-101.019200	62.966030	329.405300	.251832
-99.061880	63.449270	329.064500	.650651
-97.066180	63.906190	328.216700	1.487304
-95.023590	64.336020	326.444200	2.925372
-92.928180	64.737730	323.776600	4.861738
-90.776390	65.110020	320.347600	8.438037
-88.566850	65.451470	325.451800	14.607470
-86.300130	65.760610	370.122800	38.068970
-83.978550	66.035940	374.583100	39.112570
-81.606060	66.276030	362.185600	28.657930
-79.187900	66.479550	353.813000	23.910020
-76.730480	66.645290	349.257800	21.404480
-74.241170	66.772270	351.194900	23.228050
-71.728080	66.859700	357.892600	27.797900
-69.199870	66.907030	361.792800	29.427890
-66.665550	66.913940	354.893400	24.019530
-64.134220	66.880400	343.653000	14.690640
-61.614990	66.806620	335.074100	6.909554
-59.116610	66.693070	330.859800	2.612516
-56.647430	66.540450	329.452900	1.099940

-54.215080	66.349720	329.246500	.664460
-51.826330	66.121990	329.360400	.372139
-49.486860	65.858530	329.480300	.173377
-47.201050	65.560780	329.544500	.068677
-44.971820	65.230190	329.570700	.022623
-42.800360	64.868270	329.579100	.006507
-40.685980	64.476460	329.581400	.001660
-38.625890	64.056100	329.581900	.000384
-36.614990	63.608330	329.582000	.000094
-34.645670	63.134020	329.582100	.000033
-32.707610	62.633670	329.582100	.000031
-30.787590	62.107330	329.582100	.000031
-28.869240	61.554470	329.582100	.000031
-26.932890	60.973880	329.582100	.000031
-24.955330	60.363580	329.582100	.000031

-89.453400	32.991660	330.370200	1.245879
-88.345930	33.191160	330.755900	1.781573
-87.228100	33.381660	331.813700	3.194101
-86.100230	33.562890	333.447900	5.297545
-84.962690	33.734530	335.334600	7.679787
-83.815900	33.896310	337.058300	9.846634
-82.660300	34.047950	338.362100	11.468680
-81.496410	34.189190	339.124300	12.361970
-80.324730	34.319790	339.177900	12.381300
-79.145840	34.439500	338.295400	11.345590
-77.960310	34.548100	336.443600	9.478332
-76.768760	34.645400	334.014500	7.388643
-75.571840	34.731200	332.496000	6.511608
-74.370180	34.805370	332.381400	6.428389
-73.164480	34.867740	332.252800	5.727758
-71.955430	34.918200	332.143300	4.972913
-70.743730	34.956650	332.165600	4.329323
-69.530080	34.983020	332.130200	3.873277
-68.315220	34.997260	331.901800	3.366658
-67.099880	34.999340	331.435500	2.684511
-65.884770	34.989250	330.839100	1.872865
-64.670640	34.967020	330.291300	1.107123
-63.458190	34.932690	329.917000	.551461
-62.248150	34.886320	329.715500	.233799
-61.041230	34.828000	329.627500	.085882
-59.838120	34.757850	329.595000	.027471
-58.639500	34.676010	329.584900	.007594
-57.446040	34.582610	329.582300	.002183
-56.258390	34.477850	329.581900	.000886

-55.077160	34.361910	329.581900	.000393
-53.902950	34.235010	329.582000	.000144
-52.736340	34.097380	329.582000	.000066
-51.577860	33.949260	329.582100	.000032
-50.428010	33.790910	329.582100	.000031
-49.287250	33.622600	329.582100	.000031
-48.156040	33.444620	329.582100	.000031
-47.034740	33.257250	329.582100	.000031
-45.923690	33.060790	329.582100	.000031
-44.823220	32.855560	329.582100	.000031
-89.701210	33.918400	331.030700	2.085116
-88.583520	34.125020	331.995100	3.283987
-87.455040	34.322360	334.510800	6.268229
-86.316120	34.510110	338.481400	10.745990
-85.167100	34.687960	343.053300	15.793340
-84.008420	34.855620	347.087800	20.260050
-82.840550	35.012790	350.027300	23.473570
-81.663990	35.159200	351.786400	25.231660
-80.479300	35.294580	352.001300	25.247700
-79.287040	35.418700	350.183600	23.317970
-78.087840	35.531310	345.910600	18.932060
-76.882320	35.632210	340.413000	13.926320
-75.671150	35.721200	337.161100	13.092330
-74.455030	35.798110	338.054300	14.189610
-73.234660	35.862810	338.383700	14.042610
-72.010770	35.915150	337.636400	11.663580
-70.784100	35.955040	337.065000	10.245240
-69.555380	35.982390	336.581900	9.073359
-68.325390	35.997170	335.741400	7.826531
-67.094890	35.999320	334.413900	6.207559
-65.864640	35.988860	332.789400	4.283721
-64.635410	35.965800	331.322100	2.465166
-63.407950	35.930180	330.370900	1.192436
-62.183030	35.882080	329.884600	.485194
-60.961370	35.821590	329.679800	.168130
-59.743720	35.748840	329.607300	.049557
-58.530780	35.663950	329.586000	.013320
-57.323260	35.567100	329.581400	.005478
-56.121820	35.458470	329.581200	.002994
-54.927120	35.338260	329.581600	.001332
-53.739770	35.206700	329.581900	.000499
-52.560380	35.064020	329.582000	.000145
-51.389480	34.910500	329.582000	.000064
-50.227610	34.746390	329.582000	.000000
-49.075240	34.571990	329.582100	.000031
-47.932830	34.387580	329.582100	.000031
-46.800740	34.193490	329.582100	.000031
-45.679330	33.990000	329.582000	.000000
-44.568900	33.777440	329.582100	.000031
-89.960660	34.843320	332.995600	4.444390
-88.832330	35.057210	335.879600	7.631131
-87.692770	35.261540	343.386300	15.406760
-86.542320	35.455950	355.746200	27.332740
-85.381340	35.640140	369.850400	40.689600
-84.210270	35.813810	381.803300	52.091750
-83.029580	35.976620	390.181800	59.905230
-81.839790	36.128310	395.161200	64.033600
-80.641480	36.268600	396.071400	64.284710
-79.435230	36.397220	390.895900	58.982440
-78.221690	36.513940	378.677500	47.618350
-77.001530	36.618520	362.766400	34.800850
-75.775430	36.710780	353.419500	31.665190
-74.544130	36.790520	365.000700	44.595290
-73.308360	36.857590	358.530300	38.536740
-72.068890	36.911860	357.779600	35.118950
-70.826490	36.953220	355.200000	28.896260
-69.581950	36.981590	352.846000	25.723970

-68.336070	36.996900	349.897500	22.373440
-67.089650	36.999130	345.323900	17.765100
-65.843500	36.988290	339.960800	12.338880
-64.598400	36.964370	335.054300	6.975684
-63.355190	36.927440	331.930800	3.238959
-62.114630	36.877570	330.451300	1.292824
-60.877510	36.814860	329.850900	.430014
-59.644590	36.739430	329.642900	.118044
-58.416640	36.651430	329.584500	.037418
-57.194370	36.551040	329.575100	.023285
-55.978490	36.438440	329.577200	.013064
-54.769680	36.313860	329.579900	.005712
-53.568590	36.177530	329.581200	.002044
-52.375820	36.029710	329.581900	.000622
-51.191970	35.870660	329.582000	.000165
-50.017550	35.700660	329.582000	.000040
-48.853080	35.520030	329.582000	.000000
-47.698980	35.329070	329.582100	.000031
-46.555660	35.128100	329.582100	.000031
-45.423470	34.917440	329.582000	.000000
-44.302690	34.697410	329.582100	.000031
-90.232250	35.766320	336.681800	8.578945
-89.092860	35.987640	344.492000	16.169220
-87.941790	36.199090	365.802200	34.914660
-86.779330	36.400320	402.538000	65.138210
-85.605870	36.591000	445.034200	98.951000
-84.421870	36.770790	479.018200	126.310600
-83.227800	36.939400	501.244000	144.106000
-82.024180	37.096500	514.856300	153.327700
-80.811620	37.241800	517.354000	153.832900
-79.590740	37.375040	504.242800	143.383500
-78.362190	37.495960	474.144700	110.730100
-77.126670	37.604320	397.295700	79.961910
-75.884930	37.699920	392.124300	84.984480
-74.637700	37.782550	452.054400	119.006700
-73.385770	37.852060	430.787100	106.448600
-72.129940	37.908310	409.593200	83.998700
-70.871030	37.951180	407.114600	76.824490
-69.609860	37.980580	400.397100	66.316350
-68.347290	37.996460	391.209200	58.355180
-67.084140	37.998780	377.652800	46.840470
-65.821270	37.987530	360.603900	32.494930
-64.559530	37.962740	345.448200	18.280820
-63.299760	37.924460	336.097500	8.176254
-62.042780	37.872770	331.794000	3.044545
-60.789440	37.807780	330.212000	.981076
-59.540510	37.729610	329.684800	.273860
-58.296810	37.638420	329.549800	.142520
-57.059080	37.534390	329.542200	.099073
-55.828080	37.417740	329.559700	.052281
-54.604500	37.288680	329.572900	.021930
-53.389020	37.147460	329.579100	.007486
-52.182290	36.994370	329.581200	.002187
-50.984880	36.829660	329.581900	.000558
-49.797380	36.653650	329.582000	.000114
-48.620270	36.466650	329.582000	.000018
-47.454020	36.268990	329.582000	.000000
-46.299010	36.060990	329.582100	.000031
-45.155610	35.843010	329.582000	.000000
-44.024070	35.615370	329.582100	.000031
-90.516530	36.687310	341.390300	13.932840
-89.365660	36.916220	359.906300	28.729230
-88.202590	37.134950	412.595600	68.973300
-87.027650	37.343150	517.124000	139.216600
-85.841200	37.540460	639.926100	219.550300
-84.643710	37.726540	729.846100	279.661000
-83.435650	37.901050	780.006300	314.937300

-82.217600	38.063690	817.272700	332.476900
-80.990140	38.214140	828.214800	335.360400
-79.753940	38.352110	805.459200	312.782900
-78.509670	38.477340	674.591200	244.521400
-77.258070	38.589580	463.712500	160.657300
-75.999910	38.688600	374.536000	115.603300
-74.735980	38.774210	647.015900	387.585400
-73.467090	38.846230	608.137100	253.418300
-72.194090	38.904510	570.429700	185.014000
-70.917830	38.948930	536.033600	163.951500
-69.639210	38.979390	526.051800	154.449300
-68.359090	38.995840	505.060300	136.064800
-67.078360	38.998240	471.209400	115.994700
-65.797930	38.986590	419.631100	79.693740
-64.518690	38.960900	369.279700	40.680060
-63.241520	38.921240	344.923900	17.713560
-61.967320	38.867690	334.466000	6.274787
-60.696920	38.800350	330.682900	1.844555
-59.431210	38.719360	329.564800	.750630
-58.170970	38.624900	329.340100	.580398
-56.917050	38.517150	329.405100	.370504
-55.670200	38.396340	329.496100	.186362
-54.431160	38.262690	329.549500	.073575
-53.200640	38.116470	329.572300	.024047
-51.979310	37.957960	329.579700	.006484
-50.767770	37.787460	329.581600	.001516
-49.566610	37.605300	329.581900	.000343
-48.376340	37.411790	329.582000	.000089
-47.197410	37.207270	329.582000	.000013
-46.030250	36.992090	329.582000	.000000
-44.875180	36.766620	329.582000	.000000
-43.732470	36.531200	329.582100	.000031
-90.814130	37.606180	343.190900	17.472050
-89.651300	37.842850	374.845500	40.328200
-88.475770	38.069030	484.279200	113.301500
-87.287830	38.284350	788.899700	273.291600
-86.087840	38.488450	1140.633000	449.695700
-84.876290	38.680970	1356.715000	564.586200
-83.653650	38.861550	1457.080000	619.534500
-82.420500	39.029860	1548.313000	643.019200
-81.177470	39.185590	1589.895000	655.007900
-79.925230	39.328410	1525.863000	631.282400
-78.664510	39.458070	1272.380000	493.862600
-77.396060	39.574290	761.641700	279.429700
-76.120680	39.676830	330.247900	384.079800
-74.839220	39.765490	1397.894000	1838.657000
-73.552520	39.840080	795.340200	585.209800
-72.261480	39.900440	948.273400	469.320600
-70.967010	39.946460	875.269300	324.562000
-69.670030	39.978020	839.052600	311.915800
-68.371470	39.995060	818.394800	312.230100
-67.072270	39.997550	701.923900	254.652400
-65.773400	39.985470	573.124900	194.714100
-64.475770	39.958870	425.370500	84.655090
-63.180340	39.917780	358.330500	30.434750
-61.888020	39.862300	337.596400	10.149650
-60.599740	39.792560	330.562000	3.417902
-59.316390	39.708680	328.548100	2.495242
-58.038840	39.610860	328.460400	2.027844
-56.767930	39.499290	328.901500	1.235726
-55.504470	39.374200	329.298900	.558627
-54.249260	39.235840	329.486900	.213071
-53.003000	39.084480	329.556600	.064290
-51.766410	38.920440	329.577300	.016184
-50.540120	38.744010	329.581700	.003632
-49.324710	38.555530	329.582200	.001020
-48.120720	38.355340	329.582100	.000331

-46.928620	38.143810	329.582000	.000095
-45.748810	37.921300	329.582000	.000040
-44.581620	37.688170	329.582000	.000000
-43.427300	37.444810	329.582100	.000031
-91.125630	38.522790	330.278500	19.682770
-89.950410	38.767390	354.962700	41.119080
-88.761920	39.001200	532.402400	152.978500
-87.560450	39.223820	1320.992000	429.892200
-86.346370	39.434880	2257.579000	864.468500
-85.120160	39.633990	2762.992000	1064.792000
-83.882300	39.820790	2967.894000	1128.074000
-82.633390	39.994930	3085.846000	1156.666000
-81.374080	40.156070	3164.665000	1180.754000
-80.105060	40.303890	3149.187000	1178.161000
-78.827100	40.438090	2896.380000	1027.924000
-77.540980	40.558400	2101.087000	655.275900
-76.247540	40.664560	687.905800	532.716300
-74.947690	40.756360	265.312600	390.030600
-73.642300	40.833600	942.625600	917.955600
-72.332310	40.896110	1250.761000	775.773500
-71.018690	40.943760	1595.405000	763.595200
-69.702430	40.976440	1459.232000	592.712000
-68.384490	40.994090	1336.510000	572.331700
-67.065880	40.996670	927.196100	377.598600
-65.747600	40.984170	681.232400	295.269800
-64.430650	40.956610	486.330700	142.521600
-63.116020	40.914060	374.876100	45.428710
-61.804690	40.856610	337.811500	14.372630
-60.497620	40.784390	326.962700	8.047969
-59.195770	40.697540	324.434800	8.009756
-57.900040	40.596260	325.378300	6.126413
-56.611330	40.480750	327.488200	3.326018
-55.330490	40.351270	328.754700	1.484109
-54.058330	40.208070	329.362700	.486223
-52.795620	40.051450	329.541500	.139513
-51.543080	39.881710	329.581700	.034641
-50.301390	39.699200	329.586000	.011961
-49.071130	39.504250	329.584100	.004969
-47.852850	39.297230	329.582700	.001762
-46.647030	39.078520	329.582200	.000503
-45.454070	38.848490	329.582000	.000120
-44.274280	38.607530	329.582000	.000041
-43.107920	38.356030	329.582100	.000031
-91.451740	39.437030	301.183700	25.527820
-90.263630	39.689750	255.384200	52.332590
-89.061680	39.931370	343.714800	275.809100
-87.846150	40.161480	1546.474000	558.710400
-86.617400	40.379670	3163.320000	1083.789000
-85.375900	40.585540	4193.928000	1537.187000
-84.122160	40.778730	4621.987000	1697.276000
-82.856800	40.958850	4815.030000	1748.994000
-81.580460	41.125550	4984.453000	1805.770000
-80.293880	41.278500	5248.560000	1909.274000
-78.997860	41.417370	5376.562000	1929.139000
-77.693230	41.541890	4905.867000	1716.641000
-76.380870	41.651780	3097.796000	1135.139000
-75.061690	41.746810	626.062300	461.693500
-73.736660	41.826770	323.718000	491.125600
-72.406780	41.891490	1221.029000	772.143500
-71.073040	41.940830	2024.684000	991.535700
-69.736500	41.974670	1852.478000	814.339200
-68.398190	41.992950	1273.920000	531.177900
-67.059160	41.995620	707.847000	381.067500
-65.720480	41.982670	355.611900	175.735000
-64.383220	41.954140	393.838500	246.695700
-63.048400	41.910080	351.781800	64.236270
-61.717090	41.850600	332.052600	27.054790

-60.390290	41.775820	313.136700	23.176030
-59.069010	41.685920	314.451200	19.682790
-57.754210	41.581080	317.069000	14.733900
-56.446830	41.461530	323.964300	8.004077
-55.147770	41.327530	327.938700	2.860699
-53.857880	41.179360	329.222800	.921868
-52.577970	41.017320	329.604100	.250984
-51.308780	40.841740	329.634300	.108689
-50.051000	40.652980	329.610800	.053285
-48.805250	40.451390	329.593300	.021911
-47.572090	40.237360	329.585600	.007552
-46.352010	40.011290	329.582900	.002183
-45.145370	39.773560	329.582200	.000525
-43.952510	39.524580	329.582000	.000115
-42.773630	39.264750	329.582100	.000037
-91.793200	40.348750	326.250400	34.062930
-90.591710	40.609810	288.674500	61.533300
-89.375760	40.859430	148.292200	206.059100
-88.145600	41.097210	562.588700	552.507700
-86.901570	41.322720	1877.611000	550.177800
-85.644150	41.535550	3017.492000	777.005600
-84.373860	41.735290	3893.610000	1144.514000
-83.091290	41.921560	4503.761000	1477.190000
-81.797160	42.093980	5116.469000	1767.204000
-80.492230	42.252200	5841.571000	2045.666000
-79.177290	42.395880	6347.799000	2210.641000
-77.853240	42.524730	6217.274000	2180.754000
-76.521000	42.638450	4794.763000	1563.168000
-75.181550	42.736820	2473.510000	643.916100
-73.835900	42.819590	1383.354000	457.936000
-72.485100	42.886590	1267.027000	561.596500
-71.130210	42.937670	1783.849000	763.613700
-69.772340	42.972710	1616.291000	659.101000
-68.412590	42.991630	883.478000	452.270700
-67.052090	42.994400	233.900200	244.443100
-65.691960	42.980990	223.862000	305.795700
-64.333320	42.951450	448.503700	335.390400
-62.977290	42.905830	395.495700	192.326900
-61.624970	42.844260	324.443400	55.364200
-60.277440	42.766850	286.349400	60.356330
-58.935750	42.673790	289.500800	52.149720
-57.600940	42.565280	301.012400	36.159390
-56.273990	42.441570	319.903400	13.437930
-54.955840	42.302930	327.426400	4.750663
-53.647390	42.149640	329.881400	1.366475
-52.349470	41.982030	330.039600	.744704
-51.062890	41.800450	329.872900	.445823
-49.788320	41.605260	329.710200	.204606
-48.526440	41.396860	329.627900	.079217
-47.277780	41.175640	329.595900	.026353
-46.042850	40.942000	329.585600	.007649
-44.822010	40.696380	329.582900	.001918
-43.615560	40.439180	329.582200	.000420
-42.423690	40.170830	329.582100	.000117
-92.150770	41.257820	451.481500	112.199000
-90.935390	41.527410	554.174400	183.772200
-89.704910	41.785250	620.319000	340.961000
-88.459540	42.030900	474.289300	448.028900
-87.199620	42.263930	587.281600	465.574700
-85.925610	42.483890	1228.704000	441.470100
-84.638040	42.690380	2450.105000	623.801100
-83.337520	42.882970	3505.208000	1077.133000
-82.024790	43.061280	4568.192000	1570.344000
-80.700610	43.224930	5678.811000	1996.287000
-79.365870	43.373580	6203.158000	2177.812000
-78.021450	43.506890	6120.449000	2181.527000
-76.668370	43.624570	5425.785000	1976.585000

-75.307630	43.726360	4190.646000	1429.427000
-73.940300	43.812040	3130.558000	1160.719000
-72.567500	43.881390	1803.908000	532.237100
-71.190370	43.934270	1651.374000	544.103900
-69.810050	43.970550	1678.885000	649.135400
-68.427750	43.990140	1358.180000	648.291100
-67.044650	43.993000	737.552400	410.467100
-65.661930	43.979120	667.351200	350.396200
-64.280810	43.948540	695.850200	387.552100
-62.902460	43.901310	502.052500	188.571200
-61.528050	43.837570	329.258500	120.420400
-60.158730	43.757450	274.443600	195.679900
-58.795620	43.661140	270.911900	145.410600
-57.439790	43.548850	306.141300	52.884640
-56.092310	43.420850	322.319500	29.643770
-54.754150	43.277410	333.126500	7.054274
-53.426270	43.118850	333.026700	4.216662
-52.109530	42.945500	331.847800	2.858884
-50.804760	42.757750	330.657900	1.466783
-49.512690	42.555960	330.017800	.643655
-48.233980	42.340550	329.732600	.238534
-46.969190	42.111940	329.627100	.077545
-45.718810	41.870550	329.593800	.022320
-44.483210	41.616830	329.584700	.005697
-43.262650	41.351200	329.582500	.001296
-42.057300	41.074100	329.582200	.000350
-92.525320	42.164070	515.942100	141.450700
-91.295520	42.442420	729.529500	264.575100
-90.049940	42.708690	1110.841000	435.852300
-88.788760	42.962440	1233.695000	467.307700
-87.512310	43.203190	971.039200	339.175400
-86.221000	43.430500	1244.782000	405.689900
-84.915410	43.643920	2374.517000	631.115200
-83.596150	43.843030	3686.767000	1217.081000
-82.263960	44.027400	4947.218000	1726.841000
-80.919660	44.196660	6054.367000	2139.044000
-79.564150	44.350410	6320.705000	2247.231000
-78.198390	44.488330	5655.671000	2037.960000
-76.823410	44.610100	4582.604000	1617.419000
-75.440290	44.715440	3593.726000	1192.903000
-74.050180	44.804110	2465.239000	709.316500
-72.654240	44.875890	1114.514000	370.907500
-71.253690	44.930630	1116.941000	351.296400
-69.849760	44.968180	1739.056000	628.452200
-68.443720	44.988460	1405.919000	522.278300
-67.036810	44.991420	1066.420000	531.788300
-65.630330	44.977050	955.562100	367.474200
-64.225530	44.945390	1073.236000	374.910900
-62.823690	44.896510	850.864400	220.403700
-61.426040	44.830540	537.261100	197.711600
-60.033810	44.747610	377.501400	213.092400
-58.648170	44.647940	382.860200	180.381000
-57.270290	44.531750	381.009900	78.680820
-55.901260	44.399320	373.404000	44.369380
-54.542130	44.250930	351.953600	19.598520
-53.193900	44.086940	343.344600	13.951290
-51.857480	43.907680	336.755100	8.079276
-50.533710	43.713560	332.782100	3.990501
-49.223370	43.504980	330.801700	1.668366
-47.927120	43.282370	329.992800	.608316
-46.645540	43.046150	329.703700	.193886
-45.379100	42.796800	329.613700	.055211
-44.128160	42.534760	329.589300	.014099
-42.892960	42.260480	329.583600	.003260
-41.673610	41.974410	329.582400	.000917
-92.917770	43.067330	553.193900	169.729800
-91.673000	43.354680	747.494700	249.514400

-90.411740	43.629630	1303.815000	489.429000
-89.134110	43.891690	1973.164000	738.041300
-87.840450	44.140390	2196.726000	670.837400
-86.531140	44.375260	2403.816000	683.086800
-85.206750	44.595830	3277.473000	1052.219000
-83.867900	44.801650	4372.722000	1543.978000
-82.515380	44.992280	5384.869000	1957.577000
-81.150000	45.167310	6205.998000	2231.642000
-79.772730	45.326340	6216.090000	2237.961000
-78.384560	45.469020	5460.286000	2007.278000
-76.986600	45.595010	4229.078000	1459.417000
-75.579970	45.704020	2782.593000	741.451200
-74.165890	45.795780	1530.177000	303.865300
-72.745610	45.870080	590.340400	444.405300
-71.320400	45.926740	982.869200	337.835200
-69.891600	45.965610	1466.866000	467.913400
-68.460530	45.986610	1136.613000	365.964400
-67.028560	45.989670	841.722900	380.388700
-65.597030	45.974800	1191.758000	419.288800
-64.167300	45.942020	1344.442000	440.160200
-62.740720	45.891430	1251.938000	358.449900
-61.318600	45.823140	887.270300	183.052500
-59.902260	45.737310	595.785600	146.248600
-58.492950	45.634170	473.925000	115.306600
-57.091900	45.513950	442.497400	86.855420
-55.700260	45.376930	401.871200	55.175080
-54.319140	45.223450	386.430500	48.129600
-52.949590	45.053840	367.130900	34.085420
-51.592580	44.868490	348.277900	19.005610
-50.248970	44.667810	337.526900	9.006245
-48.919560	44.452230	332.489100	3.658132
-47.605040	44.222190	330.521900	1.300100
-46.305980	43.978160	329.855400	.409749
-45.022840	43.720610	329.652900	.114877
-43.755960	43.450020	329.598400	.029132
-42.505550	43.166850	329.585400	.006743
-41.271660	42.871580	329.582900	.001921
-93.329110	43.967430	563.893300	177.922100
-92.068780	44.264030	749.595400	252.959400
-90.791210	44.547890	1272.538000	456.892000
-89.496510	44.818510	2104.898000	807.357700
-88.184940	45.075390	2794.557000	1055.642000
-86.856880	45.318050	3195.187000	1137.194000
-85.512890	45.545990	3926.110000	1438.281000
-84.153590	45.758730	4802.226000	1797.895000
-82.779790	45.955820	5418.998000	2027.826000
-81.392350	46.136820	5703.167000	2105.174000
-79.992260	46.301310	5593.409000	2057.160000
-78.580580	46.448910	4991.936000	1849.180000
-77.158460	46.579270	3993.478000	1413.391000
-75.727110	46.692070	2806.862000	829.384300
-74.287820	46.787040	1664.240000	379.015800
-72.841890	46.863950	993.368000	466.746600
-71.390720	46.922590	1289.817000	487.395800
-69.935700	46.962830	1443.499000	460.832700
-68.478260	46.984570	1165.911000	308.404200
-67.019850	46.987740	1181.953000	282.706100
-65.561920	46.972340	1309.171000	342.244600
-64.105920	46.938410	1491.377000	461.396400
-62.653260	46.886040	1396.702000	395.002100
-61.205390	46.815350	986.904700	167.015000
-59.763680	46.726520	688.868200	166.506600
-58.329470	46.619790	579.871800	117.434100
-56.904060	46.495390	504.996100	100.371700
-55.488670	46.353640	485.903900	110.311600
-54.084500	46.194880	479.281500	108.036800
-52.692630	46.019470	431.603300	79.156850

-51.314070	45.827840	374.858600	39.755910
-49.949730	45.620390	346.377700	17.101460
-48.600430	45.397590	335.375600	6.695815
-47.266860	45.159900	331.374200	2.312807
-45.949600	44.907820	330.084100	.711537
-44.649100	44.641840	329.709900	.197172
-43.365670	44.362450	329.611300	.049359
-42.099450	44.070140	329.588100	.011396
-40.850440	43.765410	329.583600	.003225
-93.760410	44.864160	552.583100	167.768800
-92.483920	45.170280	688.580600	221.710000
-91.189420	45.463320	1010.086000	354.263700
-89.876960	45.742750	1594.216000	566.969400
-88.546760	46.008070	2202.668000	787.441000
-87.199170	46.258760	2675.532000	941.597600
-85.834720	46.494300	3183.778000	1112.814000
-84.454070	46.714200	4042.486000	1498.510000
-83.058010	46.917960	4644.195000	1733.224000
-81.647480	47.105130	4864.694000	1804.663000
-80.223460	47.275260	4600.892000	1689.516000
-78.787090	47.427960	3661.916000	1243.947000
-77.339580	47.562840	2833.021000	921.039700
-75.882230	47.679570	2367.929000	773.258200
-74.416370	47.777870	2126.246000	708.530200
-72.943440	47.857470	1880.545000	688.035800
-71.464880	47.918190	1700.295000	580.891400
-69.982220	47.959850	1521.701000	456.607900
-68.496960	47.982350	1268.523000	259.772500
-67.010670	47.985630	1421.252000	262.803700
-65.524890	47.969690	1615.057000	419.950300
-64.041170	47.934570	1618.689000	432.532900
-62.561040	47.880340	1434.578000	331.932000
-61.086020	47.807170	1140.905000	173.701000
-59.617590	47.715230	914.191000	151.777700
-58.157170	47.604770	777.524200	125.983400
-56.706150	47.476050	685.230400	163.381900
-55.265830	47.329400	692.006400	209.310900
-53.837470	47.165180	674.203900	219.968900
-52.422200	46.983780	546.216700	149.778300
-51.021090	46.785640	420.490200	70.569490
-49.635090	46.571200	359.038200	27.011260
-48.265020	46.340950	338.667700	9.781364
-46.911610	46.095370	332.255000	3.279093
-45.575410	45.834990	330.309100	.991638
-44.256860	45.560320	329.763100	.269754
-42.956210	45.271870	329.623000	.066876
-41.673580	44.970160	329.590500	.015296
-40.408870	44.655690	329.584100	.004306
-94.212860	45.757310	546.969100	156.828800
-92.919560	46.073230	647.086900	191.520400
-91.607470	46.375730	819.989200	269.639200
-90.276550	46.664250	1129.442000	391.952600
-88.926960	46.938270	1333.376000	400.600900
-87.559010	47.197250	1456.246000	399.633200
-86.173240	47.440650	1911.987000	567.146400
-84.770290	47.667940	2764.901000	906.801100
-83.350960	47.878600	3685.354000	1327.443000
-81.916200	48.072160	4141.593000	1533.569000
-80.467090	48.248140	3581.411000	1247.666000
-79.004780	48.406110	2332.254000	742.150100
-77.530590	48.545680	1586.867000	593.681600
-76.045850	48.666500	1564.563000	513.839100
-74.552020	48.768250	1809.404000	578.345900
-73.050600	48.850660	1843.166000	605.738300
-71.543170	48.913520	1739.066000	556.063900
-70.031330	48.956650	1558.829000	486.521600
-68.516710	48.979950	1405.159000	388.361600

-67.000980	48.983350	1394.900000	376.333600
-65.485790	48.966840	1434.620000	349.702100
-63.972820	48.930470	1600.368000	457.787800
-62.463700	48.874330	1545.797000	401.129000
-60.960050	48.798580	1481.313000	319.042100
-59.463460	48.703410	1435.200000	289.892800
-57.975440	48.589080	1287.559000	297.517500
-56.497490	48.455880	1051.165000	304.577500
-55.030970	48.304140	942.344800	340.990700
-53.577210	48.134270	840.873400	316.212900
-52.137430	47.946660	625.237500	194.149700
-50.712720	47.741790	437.878100	80.293970
-49.304080	47.520120	361.827300	29.557650
-47.912350	47.282170	339.557200	10.777860
-46.538250	47.028450	332.498900	3.569977
-45.182340	46.759510	330.361600	1.063597
-43.845020	46.475880	329.775000	.288384
-42.526500	46.178100	329.625500	.070815
-41.226800	45.866700	329.590900	.016120
-39.945740	45.542200	329.584300	.004514
-94.687740	46.646660	558.610400	156.197300
-93.376940	46.972690	646.676100	184.861400
-92.046550	47.284930	739.354300	239.563300
-90.696440	47.582840	710.534500	211.325800
-89.326680	47.865840	577.616300	190.342700
-87.937550	48.133390	673.070900	275.732400
-86.529520	48.384910	1284.617000	332.566200
-85.103260	48.619850	2267.893000	698.114000
-83.659580	48.837660	3208.483000	1149.284000
-82.199460	49.037830	3460.803000	1283.526000
-80.724000	49.219880	2886.944000	1021.507000
-79.234450	49.383340	1741.110000	593.523400
-77.732160	49.527770	1390.568000	475.591100
-76.218590	49.652830	1528.598000	491.268700
-74.695260	49.758160	1502.958000	435.043700
-73.163800	49.843490	1381.470000	367.071900
-71.625870	49.908580	1408.562000	378.435900
-70.083210	49.953250	1475.315000	458.174200
-68.537570	49.977380	1270.533000	406.224300
-66.990740	49.980900	1108.435000	329.326200
-65.444490	49.963800	1152.064000	365.393500
-63.900620	49.926140	1188.913000	358.474500
-62.360880	49.868010	1244.810000	383.978700
-60.827010	49.789570	1471.396000	467.992800
-59.300730	49.691040	1617.642000	524.891400
-57.783640	49.572690	1431.042000	445.631200
-56.277320	49.434830	1117.381000	382.154800
-54.783270	49.277820	877.054900	320.753700
-53.302860	49.102080	711.442900	248.673600
-51.837360	48.908040	526.171000	142.510400
-50.387950	48.696200	400.598700	60.591260
-48.955640	48.467040	352.172200	23.629570
-47.541310	48.221130	337.075200	8.926087
-46.145660	47.959000	331.876100	2.991109
-44.769230	47.681210	330.206900	.895202
-43.412380	47.388330	329.737300	.241028
-42.075270	47.080920	329.616900	.059222
-40.757810	46.759540	329.589100	.013384
-39.459720	46.424710	329.583800	.003735
-95.186440	47.531940	594.772900	169.067700
-93.857410	47.868410	697.836200	205.560100
-92.508000	48.190720	739.236000	239.918000
-91.137910	48.498310	647.869900	182.872300
-89.747150	48.790600	464.580200	171.506200
-88.335950	49.067010	710.239900	200.050000
-86.904710	49.326930	1370.384000	392.574800
-85.454100	49.569790	2101.106000	738.361400

-83.984930	49.795020	2579.469000	967.821600
-82.498220	50.002060	2616.741000	1009.452000
-80.995120	50.190400	2184.686000	830.829400
-79.476920	50.359550	1627.897000	571.801800
-77.945060	50.509060	1602.201000	575.479600
-76.401090	50.638520	1639.768000	574.159300
-74.846650	50.747580	1465.812000	445.104400
-73.283450	50.835950	1279.465000	341.333600
-71.713320	50.903350	1310.706000	357.428100
-70.138080	50.949630	1412.425000	413.058300
-68.559640	50.974620	1295.181000	412.967700
-66.979900	50.978270	1049.984000	350.764600
-65.400800	50.960560	938.704000	283.166000
-63.824260	50.921550	878.039400	293.584300
-62.252170	50.861340	676.570100	279.672300
-60.686400	50.780110	1102.994000	400.575400
-59.128770	50.678080	1275.571000	463.774700
-57.581030	50.555550	1125.640000	389.937700
-56.044860	50.412850	797.507400	263.678600
-54.521820	50.250360	608.773200	189.205900
-53.013420	50.068520	510.696300	138.242800
-51.520960	49.867810	424.257200	79.830930
-50.045680	49.648740	370.289200	39.760930
-48.588630	49.411830	345.555600	17.700430
-47.150700	49.157670	334.738600	6.288071
-45.732590	48.886830	331.079600	2.063538
-44.334810	48.599900	329.992800	.617690
-42.957660	48.297460	329.684000	.166013
-41.601190	47.980120	329.604500	.040240
-40.265250	47.648430	329.586500	.009064
-38.949390	47.302930	329.583200	.002492
-95.710510	48.412890	651.760300	189.309200
-94.362470	48.760130	772.304500	238.147500
-92.993230	49.092850	782.013100	251.502800
-91.602360	49.410460	701.658900	213.311900
-90.189750	49.712350	624.334200	190.272700
-88.755540	49.997940	789.315600	238.266100
-87.300090	50.266570	1192.339000	419.740900
-85.824040	50.517650	1554.443000	584.788600
-84.328210	50.750570	1779.164000	701.859300
-82.813610	50.964740	1697.655000	692.372800
-81.281460	51.159630	1518.314000	606.363400
-79.733130	51.334700	1356.438000	511.704400
-78.170130	51.489480	1389.713000	520.473600
-76.594090	51.623530	1377.029000	489.118200
-75.006800	51.736490	1252.664000	401.819500
-73.410070	51.828010	1138.314000	334.619000
-71.805870	51.897850	1149.716000	320.935200
-70.196170	51.945790	1209.933000	330.670000
-68.582990	51.971680	1145.090000	357.089200
-66.968440	51.975460	921.051400	332.584400
-65.354560	51.957120	996.108200	363.916300
-63.743440	51.916690	917.061300	271.646400
-62.137130	51.854320	789.040700	211.514200
-60.537630	51.770170	873.646100	279.504700
-58.946900	51.664510	907.159900	331.277400
-57.366820	51.537620	698.959500	222.805700
-55.799180	51.389870	538.245400	149.435500
-54.245650	51.221680	454.007700	101.433600
-52.707820	51.033510	404.318400	66.596230
-51.187080	50.825860	370.705200	40.263740
-49.684720	50.599280	349.707000	21.842580
-48.201800	50.354330	337.384400	9.260479
-46.739240	50.091630	332.165200	3.462314
-45.297720	49.811770	330.382400	1.176015
-43.877700	49.515370	329.803700	.351295
-42.479410	49.203060	329.636300	.093484

-41.102810	48.875440	329.593900	.022829
-39.747590	48.533100	329.584400	.005032
-38.413150	48.176570	329.582600	.001360
-96.261670	49.289190	702.940900	206.418600
-94.893750	49.647600	793.088100	250.447600
-93.503820	49.991080	754.891500	247.878000
-92.091320	50.319060	630.232300	247.698900
-90.655950	50.630910	575.857700	230.134200
-89.197760	50.925990	632.760700	198.407700
-87.717060	51.203660	795.344200	262.558100
-86.214420	51.463270	1040.814000	387.910600
-84.690660	51.704190	1300.223000	516.571700
-83.146820	51.925780	1329.877000	531.202000
-81.584160	52.127480	1189.214000	454.473200
-80.004110	52.308710	1053.890000	390.494700
-78.408280	52.468990	991.229400	380.796800
-76.798410	52.607840	1046.709000	388.226500
-75.176390	52.724850	1084.715000	362.463000
-73.544200	52.819690	1021.413000	301.971600
-71.903920	52.892050	938.517000	256.323300
-70.257710	52.941730	956.152600	223.566800
-68.607750	52.968570	805.090300	213.768300
-66.956280	52.972490	434.424800	238.051000
-65.305560	52.953480	560.451700	195.360900
-63.657820	52.911580	817.783300	257.742700
-62.015270	52.846950	710.900900	175.058200
-60.380080	52.759750	724.571200	220.296300
-58.754360	52.650280	603.960600	171.393500
-57.140120	52.518850	488.093800	118.617900
-55.539290	52.365840	423.678000	80.246040
-53.953660	52.191710	383.551200	50.922210
-52.384890	51.996940	359.648700	31.367200
-50.834480	51.782070	345.710200	18.144580
-49.303730	51.547700	337.350700	9.425896
-47.793770	51.294400	332.704700	4.168205
-46.305490	51.022820	330.703900	1.631687
-44.839570	50.733620	329.935700	.553324
-43.396390	50.427420	329.679400	.164929
-41.976090	50.104880	329.605900	.044681
-40.578500	49.766630	329.587200	.010757
-39.203150	49.413260	329.583000	.002325
-37.849210	49.045330	329.582300	.000603
-96.841810	50.160530	622.076800	170.241400
-95.453030	50.530490	669.553800	195.516600
-94.041510	50.885130	605.730300	182.638500
-92.606450	51.223850	517.243200	155.642800
-91.147380	51.546020	475.426300	126.130200
-89.664200	51.850960	482.030800	113.737100
-88.157130	52.138020	551.479400	158.195600
-86.626690	52.406500	693.575700	237.176200
-85.073680	52.655720	950.403700	352.830300
-83.499180	52.885050	1032.202000	389.099800
-81.904440	53.093850	885.059500	322.079400
-80.290990	53.281520	684.767800	257.281900
-78.660530	53.447530	605.308700	266.731500
-77.014920	53.591380	793.205600	304.740100
-75.356160	53.712650	977.754300	334.331100
-73.686420	53.810940	1007.092000	305.324500
-72.007930	53.885960	921.456100	256.078800
-70.322990	53.937460	860.822600	209.989300
-68.634010	53.965280	718.442100	220.411400
-66.943400	53.969350	346.620800	274.595800
-65.253590	53.949630	428.206800	149.096300
-63.567000	53.906200	631.943000	159.866200
-61.886040	53.839190	637.580600	164.315900
-60.213040	53.748820	549.143200	131.311200
-58.550290	53.635360	452.461300	91.833060

-56.899960	53.499180	400.947100	62.749250
-55.264110	53.340690	370.042700	39.375010
-53.644650	53.160350	351.404300	23.711370
-52.043340	52.958710	341.193800	13.769210
-50.461760	52.736320	335.497500	7.519546
-48.901280	52.493820	332.348800	3.764352
-47.363030	52.231850	330.728800	1.677889
-45.847900	51.951060	329.997100	.651129
-44.356530	51.652150	329.712400	.220588
-42.889220	51.335790	329.618200	.066993
-41.445980	51.002650	329.590900	.017934
-40.026490	50.653380	329.583900	.004252
-38.630060	50.288590	329.582300	.000877
-37.255620	49.908830	329.582100	.000220
-97.453060	51.026530	486.661700	101.314400
-96.042340	51.408490	498.647500	108.633500
-94.608220	51.774700	454.903600	89.389990
-93.149600	52.124570	422.795500	73.109790
-91.665800	52.457450	404.561400	63.343110
-90.156560	52.772640	405.203500	65.806790
-88.621980	53.069450	431.748600	87.648030
-87.062480	53.347160	485.562300	124.186300
-85.478850	53.605050	588.616600	175.400600
-83.872130	53.842430	668.968300	214.061500
-82.243660	54.058630	548.147700	179.773300
-80.595020	54.253030	404.752400	155.969000
-78.928000	54.425040	383.729800	179.917000
-77.244600	54.574130	603.006000	216.556900
-75.546970	54.699840	846.311700	293.636000
-73.837420	54.801750	959.828700	303.295200
-72.118360	54.879540	920.441700	270.339400
-70.392330	54.932960	846.769200	227.274700
-68.661900	54.961820	805.615300	243.929800
-66.929700	54.966030	658.114900	219.277600
-65.198370	54.945580	636.567600	180.852000
-63.470540	54.900540	628.748800	164.374600
-61.748820	54.831050	540.186900	126.110400
-60.035740	54.737340	440.573800	81.561440
-58.333780	54.619720	388.972700	52.585690
-56.645260	54.478570	361.925100	32.102630
-54.972420	54.314330	346.349200	18.606640
-53.317280	54.127510	338.095900	10.412910
-51.681740	53.918690	333.825500	5.593046
-50.067420	53.688470	331.620600	2.869199
-48.475770	53.437500	330.499300	1.375144
-46.907930	53.166490	329.954300	.595056
-45.364770	52.876130	329.714800	.227665
-43.846840	52.567130	329.623800	.077621
-42.354370	52.240220	329.593500	.023234
-40.887210	51.896080	329.584800	.006130
-39.444820	51.535370	329.582600	.001395
-38.026250	51.158730	329.582100	.000272
-36.630120	50.766700	329.582100	.000067
-98.097820	51.886770	397.549500	53.750100
-96.663910	52.281200	395.746900	50.701110
-95.206040	52.659450	382.493700	43.829690
-93.722790	53.020900	370.997700	37.006720
-92.213200	53.364910	363.207100	32.142460
-90.676770	53.690770	362.568700	33.102330
-89.113440	53.997730	371.797300	42.113860
-87.523560	54.285050	390.909300	59.124840
-85.907830	54.551970	416.091800	79.113690
-84.267300	54.797760	432.127100	97.420140
-82.603340	55.021710	367.745800	109.632300
-80.917590	55.223140	272.638800	110.429800
-79.211960	55.401440	296.366100	137.104500
-77.488560	55.556020	476.939600	144.976500

-75.749730	55.686380	709.024000	231.086000
-73.997920	55.792100	909.631500	299.561400
-72.235800	55.872810	928.442800	278.394700
-70.466090	55.928230	845.896700	228.534100
-68.691570	55.958180	878.227200	242.186400
-66.915130	55.962550	878.205900	270.494200
-65.139640	55.941330	752.443100	209.155000
-63.367970	55.894590	599.766900	143.823300
-61.602940	55.822490	463.837600	93.246270
-59.847310	55.725280	395.441400	56.762220
-58.103760	55.603290	362.763000	32.629870
-56.374820	55.456930	345.456500	17.568190
-54.662870	55.286670	336.954200	9.107219
-52.970100	55.093080	332.957200	4.547654
-51.298500	54.876750	331.100600	2.209739
-49.649800	54.638340	330.249400	1.040608
-48.025460	54.378550	329.862400	.465758
-46.426660	54.098120	329.690500	.192169
-44.854210	53.797780	329.619800	.071877
-43.308580	53.478290	329.593700	.023856
-41.789850	53.140400	329.585100	.006991
-40.297680	52.784830	329.582700	.001780
-38.831280	52.412250	329.582200	.000382
-37.389370	52.023280	329.582000	.000075
-35.970190	51.618480	329.582100	.000031
-98.778840	52.740810	357.603800	26.006210
-97.320270	53.148240	354.794900	23.461730
-95.837370	53.539010	350.562800	20.206940
-94.328290	53.912530	346.047100	16.720290
-92.791720	54.268120	342.803300	14.351120
-91.226900	54.605080	342.043600	14.368240
-89.633560	54.922620	344.463000	17.828390
-88.011890	55.219970	349.948000	25.555460
-86.362530	55.496330	354.743600	37.499280
-84.686490	55.750910	348.966400	55.798220
-82.985150	55.982970	296.500600	101.645400
-81.260250	56.191770	209.560400	105.964300
-79.513790	56.376650	278.700400	149.322900
-77.748020	56.536990	398.340400	99.918850
-75.965460	56.672260	576.506000	161.445200
-74.168790	56.781970	845.477700	270.805100
-72.360860	56.865740	954.488300	291.833600
-70.544630	56.923280	908.112400	242.232300
-68.723180	56.954370	941.648400	258.435400
-66.899620	56.958910	930.369100	286.331700
-65.077100	56.936880	733.773100	200.996900
-63.258730	56.888360	551.005400	127.786000
-61.447630	56.813510	442.044100	78.607880
-59.646790	56.712620	382.301400	46.785240
-57.859090	56.586040	353.600200	24.814880
-56.087300	56.434210	339.925400	11.996350
-54.333970	56.257640	333.783500	5.400040
-52.601470	56.056940	331.221500	2.325529
-50.891880	55.832740	330.205800	.971738
-49.207030	55.585770	329.815900	.398257
-47.548420	55.316760	329.667800	.158114
-45.917200	55.026500	329.612200	.059652
-44.314130	54.715760	329.591800	.020809
-42.739580	54.385350	329.584900	.006587
-41.193420	54.036030	329.582700	.001828
-39.675070	53.668560	329.582200	.000444
-38.183400	53.283620	329.582000	.000084
-36.716770	52.881820	329.582000	.000013
-35.272900	52.463700	329.582100	.000031
-99.499240	53.588130	340.218800	11.229520
-98.014280	54.009130	338.677600	9.825473
-96.504860	54.412960	337.069900	8.270766

-94.968610	54.799050	335.396300	6.729331
-93.403790	55.166730	334.112100	5.560849
-91.809290	55.515250	333.504200	5.215551
-90.184560	55.843850	333.540600	6.657390
-88.529620	56.151670	333.626400	11.750120
-86.844990	56.437890	331.693300	21.873580
-85.131650	56.701690	321.659800	45.805540
-83.390960	56.942230	266.711500	104.498800
-81.624720	57.158780	183.849200	98.970540
-79.835050	57.350580	276.051600	156.922700
-78.024370	57.516980	346.555100	82.588210
-76.195360	57.657400	456.210700	126.740700
-74.350940	57.771310	741.905800	213.934600
-72.494220	57.858320	968.136500	288.289700
-70.628410	57.918090	1010.446000	277.079700
-68.756890	57.950390	998.850100	282.771500
-66.883060	57.955100	901.590400	280.816200
-65.010370	57.932220	668.843500	189.365200
-63.142240	57.881810	523.307900	118.485900
-61.282040	57.804080	454.182200	89.055530
-59.433060	57.699310	385.791500	45.274960
-57.598450	57.567890	353.687700	23.997340
-55.781200	57.410300	339.484300	11.067980
-53.984060	57.227100	333.316400	4.550837
-52.209560	57.018940	330.858600	1.711302
-50.459930	56.786500	329.990400	.608120
-48.737060	56.530560	329.706100	.205602
-47.042490	56.251900	329.618400	.067328
-45.377330	55.951360	329.592300	.021259
-43.742270	55.629770	329.584800	.006401
-42.137470	55.287960	329.582700	.001782
-40.562610	54.926740	329.582200	.000440
-39.016750	54.546860	329.582000	.000098
-37.498410	54.149020	329.582000	.000000
-36.005400	53.733830	329.582000	.000000
-34.534880	53.301770	329.582100	.000031
-100.262700	54.428120	333.237000	4.386475
-98.749250	54.863350	332.599100	3.702109
-97.211560	55.280850	331.996700	3.030090
-95.646590	55.680060	331.402300	2.365551
-94.052090	56.060360	330.854200	1.742324
-92.426510	56.420970	330.296800	1.455339
-90.768940	56.761100	329.434200	3.047953
-89.079190	57.079890	327.568700	8.014436
-87.357570	57.376450	322.682800	17.687090
-85.605000	57.649890	309.810000	42.272210
-83.822840	57.899360	256.353000	101.199600
-82.012920	58.124030	170.835300	94.492110
-80.177480	58.323120	278.920200	144.477000
-78.319110	58.495910	328.755500	70.202700
-76.440710	58.641750	392.346800	119.178900
-74.545430	58.760110	662.715600	166.245500
-72.636660	58.850530	981.851500	282.643400
-70.717930	58.912650	1088.419000	312.110300
-68.792920	58.946230	1039.549000	304.321600
-66.865380	58.951130	846.840900	265.560200
-64.939080	58.927340	606.852700	170.875500
-63.017780	58.874950	498.380600	114.164100
-61.105210	58.794160	450.628700	83.829080
-59.204930	58.685300	407.834000	68.512570
-57.320400	58.548780	359.345200	25.597180
-55.454840	58.385130	340.929000	11.536840
-53.611270	58.194950	333.682900	4.663021
-51.792370	57.978940	330.938100	1.669534
-50.000500	57.737840	329.985500	.542897
-48.237630	57.472490	329.692600	.164728
-46.505310	57.183730	329.610200	.046436

-44.804610 56.872430 329.588600 .012247
-43.136050 56.539500 329.583400 .003006
-41.499610 56.185780 329.582300 .000682
-39.894630 55.812120 329.582000 .000127
-38.319800 55.419290 329.582000 .000013
-36.773090 55.007980 329.582000 .000000
-35.251730 54.578750 329.582000 .000000
-33.752120 54.132060 329.582100 .000031
-101.073700 55.260130 330.738700 1.549223
-99.529010 55.710320 330.493000 1.251727
-97.960900 56.142140 330.282000 .978419
-96.365430 56.555100 330.055700 .673638
-94.739640 56.948590 329.748100 .326786
-93.081440 57.321860 329.175200 .893665
-91.389490 57.674070 327.900200 2.958349
-89.663250 58.004340 325.096000 7.602232
-87.902820 58.311730 318.651600 16.673450
-86.109000 58.595320 303.516400 40.356100
-84.283100 58.854170 256.641600 102.093600
-82.427000 59.087400 169.863300 108.670000
-80.543050 59.294160 295.707100 133.476000
-78.634010 59.473690 351.822500 71.901230
-76.703000 59.625280 432.207200 87.917260
-74.753460 59.748340 694.222800 169.723000
-72.789070 59.842370 968.516400 286.941800
-70.813740 59.906970 1138.333000 343.912700
-68.831480 59.941910 1063.632000 321.465600
-66.846440 59.947010 816.462500 250.885300
-64.862770 59.922250 563.944500 148.749500
-62.884610 59.867760 442.005400 79.442920
-60.916040 59.783740 418.987500 82.258930
-58.961010 59.670550 394.110600 57.196150
-57.023270 59.528640 366.400300 36.471550
-55.106350 59.358580 341.889500 10.689960
-53.213500 59.161040 333.491600 4.054532
-51.347600 58.936760 330.836200 1.464790
-49.511150 58.686560 329.955900 .473052
-47.706190 58.411320 329.683300 .140265
-45.934240 58.111950 329.607100 .038469
-44.196280 57.789400 329.587600 .009688
-42.492650 57.444580 329.583100 .002212
-40.823030 57.078400 329.582200 .000437
-39.186360 56.691730 329.582000 .000071
-37.580830 56.285340 329.582000 .000000
-36.003750 55.859910 329.582000 .000000
-34.451600 55.415950 329.582000 .000000
-32.919830 54.953830 329.582100 .000031
-101.937200 56.083360 329.915300 .492923
-100.358100 56.549350 329.830000 .377367
-98.756940 56.996240 329.753800 .262470
-97.128790 57.423640 329.642700 .100420
-95.469860 57.830970 329.398700 .326239
-93.777320 58.217490 328.775400 1.204594
-92.049300 58.582370 327.215900 3.202216
-90.284800 58.924680 323.650100 7.516994
-88.483640 59.243460 315.795700 16.211050
-86.646400 59.537720 299.919000 39.832380
-84.774360 59.806450 256.542000 92.493010
-82.869400 60.048700 192.249100 121.876000
-80.933970 60.263580 310.796000 126.344600
-78.971000 60.450230 372.486200 96.345950
-76.983890 60.607900 527.240700 126.203300
-74.976370 60.735940 686.906300 177.876400
-72.952470 60.833800 926.836400 289.397600
-70.916480 60.901050 1093.492000 347.924800
-68.872850 60.937420 1094.347000 339.396200
-66.826130 60.942730 805.651300 236.117900

-64.780930 60.916960 516.700800 123.897100
-62.741830 60.860230 356.071500 51.298020
-60.713310 60.772790 313.603600 37.546630
-58.699720 60.654990 329.399500 32.001200
-56.705200 60.507380 335.630000 12.619290
-54.733590 60.330550 333.487400 4.470996
-52.788400 60.125220 331.143200 1.933783
-50.872730 59.892220 330.196300 .771947
-48.989200 59.632420 329.789600 .265309
-47.139900 59.346780 329.642900 .082865
-45.326320 59.036270 329.598000 .024019
-43.549270 58.701890 329.585800 .006257
-41.808860 58.344600 329.582800 .001451
-40.104370 57.965370 329.582200 .000284
-38.434220 57.565060 329.582000 .000048
-36.795950 57.144450 329.582000 .000000
-35.186060 56.704150 329.582000 .000000
-33.600030 56.244660 329.582000 .000000
-32.032190 55.766200 329.582100 .000031
-102.859900 56.896880 329.668500 .140866
-101.242000 57.379640 329.640400 .097463
-99.604390 57.842470 329.606700 .040379
-97.940930 58.285070 329.527100 .103124
-96.246660 58.706940 329.290400 .454230
-94.517820 59.107400 328.604600 1.323351
-92.751850 59.485580 326.761100 3.284909
-90.947170 59.840560 322.329800 7.398225
-89.103220 60.171320 312.837400 16.195000
-87.220290 60.476800 293.912900 37.440620
-85.299550 60.755960 248.582700 86.119080
-83.342830 61.007760 220.980300 141.787300
-81.352710 61.231220 327.926800 97.853000
-79.332320 61.425420 457.432200 134.884400
-77.285290 61.589540 550.757400 142.317500
-75.215710 61.722870 671.468000 196.031700
-73.128000 61.824790 770.493400 244.854100
-71.026890 61.894870 1007.823000 328.289800
-68.917300 61.932760 1118.521000 360.787800
-66.804310 61.938290 801.842000 233.410200
-64.692990 61.911450 454.146600 106.580900
-62.588420 61.852340 266.508800 82.224350
-60.495590 61.761240 215.046900 101.050500
-58.419300 61.638580 262.031200 53.945080
-56.364090 61.484900 305.925100 20.981850
-54.334160 61.300890 324.161400 5.778084
-52.333320 61.087320 328.606100 1.492114
-50.364880 60.845090 329.467800 .339294
-48.431600 60.575160 329.590900 .062809
-46.535590 60.278550 329.593200 .018190
-44.678250 59.956310 329.586400 .006750
-42.860180 59.609490 329.583300 .002215
-41.081100 59.239120 329.582300 .000585
-39.339830 58.846180 329.582100 .000121
-37.634110 58.431540 329.582000 .000013
-35.960610 57.995940 329.582000 .000000
-34.314830 57.539980 329.582000 .000000
-32.690990 57.064010 329.582000 .000000
-31.082000 56.568130 329.582100 .000031
-103.849600 57.699570 329.601700 .035054
-102.187300 58.200280 329.591600 .017137
-100.509000 58.680030 329.570300 .023756
-98.806840 59.138710 329.499400 .139522
-97.074520 59.575940 329.263300 .466109
-95.307120 59.991050 328.536700 1.309291
-93.501110 60.383240 326.477300 3.244439
-91.654170 60.751550 321.364600 7.278984
-89.765210 61.094930 309.933600 15.762070

-87.834170 61.412270 280.116300 31.473830
-85.861950 61.702450 238.723100 79.988670
-83.850420 61.964360 262.168900 186.277000
-81.802150 62.196920 405.821400 128.857300
-79.720490 62.399150 503.934300 149.974100
-77.609360 62.570130 569.930200 165.150800
-75.473210 62.709080 552.145600 144.901900
-73.316960 62.815350 623.976100 187.222800
-71.145790 62.888430 878.004200 279.669900
-68.965200 62.927950 1044.494000 345.061600
-66.780790 62.933720 767.684300 227.424800
-64.598260 62.905720 431.043800 97.683070
-62.423240 62.844070 273.379600 72.889210
-60.261290 62.749080 251.653900 63.926460
-58.117710 62.621230 285.573200 37.598410
-55.997520 62.461110 312.481600 16.509770
-53.905320 62.269450 324.250600 5.949229
-51.845240 62.047150 328.093300 1.857919
-49.820820 61.795150 329.220900 .492276
-47.834930 61.514500 329.505900 .113684
-45.889670 61.206310 329.567700 .022806
-43.986290 60.871700 329.579900 .003699
-42.125070 60.511780 329.581800 .000459
-40.305270 60.127630 329.582000 .000049
-38.525020 59.720250 329.582000 .000000
-36.781200 59.290500 329.582000 .000000
-35.069400 58.839090 329.582000 .000000
-33.383770 58.366500 329.582000 .000000
-31.716990 57.872960 329.582000 .000000
-30.060130 57.358350 329.582100 .000031
-104.916500 58.490070 329.585400 .006418
-103.202300 59.010120 329.580600 .003426
-101.477600 59.507980 329.563800 .033718
-99.732440 59.983750 329.500500 .132317
-97.958720 60.437220 329.282300 .420409
-96.150010 60.867820 328.593900 1.176987
-94.301540 61.274790 326.594200 2.920399
-92.410060 61.657170 321.523300 6.780256
-90.473700 62.013880 309.685400 14.352080
-88.491920 62.343760 276.886800 28.259750
-86.465330 62.645610 230.256600 76.235070
-84.395630 62.918240 306.927200 109.699600
-82.285480 63.160470 453.979800 145.687200
-80.138370 63.371240 528.085000 164.095900
-77.958560 63.549540 496.607500 118.819900
-75.750930 63.694510 455.127200 95.244260
-73.520860 63.805420 498.263800 121.107500
-71.274150 63.881700 661.210400 186.774000
-69.016910 63.922960 812.947800 248.617500
-66.755400 63.928990 629.244400 166.576900
-64.495980 63.899750 412.456800 72.892380
-62.244980 63.835380 320.541300 33.807160
-60.008560 63.736250 307.033200 25.560620
-57.792640 63.602840 312.191400 18.118220
-55.602750 63.435840 321.360500 9.215967
-53.443960 63.236060 326.425900 3.806806
-51.320750 63.004450 328.569100 1.319189
-49.236890 62.742080 329.297500 .402979
-47.195330 62.450080 329.509100 .110861
-45.198120 62.129630 329.565500 .027078
-43.246250 61.781950 329.578700 .006001
-41.339580 61.408210 329.581400 .001171
-39.476710 61.009530 329.581900 .000211
-37.654880 60.586900 329.582000 .000066
-35.869840 60.141170 329.582000 .000000
-34.115770 59.672970 329.582000 .000000
-32.385130 59.182660 329.582000 .000000

-30.668580 58.670220 329.582100 .000031
-28.954870 58.135290 329.582100 .000031
-106.073800 59.266680 329.581900 .000553
-104.297500 59.807790 329.578900 .006656
-102.518800 60.325180 329.565300 .030666
-100.725000 60.819250 329.513100 .110517
-98.905530 61.289990 329.333000 .343944
-97.052100 61.737010 328.765600 .961076
-95.158340 62.159620 327.141700 2.394669
-93.219700 62.556880 322.933400 5.419473
-91.233350 62.927700 312.939400 11.371660
-89.198040 63.270870 282.826000 25.194690
-87.113920 63.585110 258.108400 57.200450
-84.982480 63.869130 348.661100 75.451740
-82.806390 64.121680 461.901200 130.255800
-80.589290 64.341560 475.527500 114.325200
-78.335770 64.527670 412.807900 64.158470
-76.051160 64.679080 393.373200 55.861510
-73.741460 64.794950 412.261900 68.639700
-71.413090 64.874680 470.027200 95.437740
-69.072890 64.917820 544.824300 122.524500
-66.727910 64.924110 461.941800 85.171200
-64.385280 64.893550 378.712000 41.319100
-62.052090 64.826280 337.898400 15.335220
-59.735260 64.722690 324.681200 9.490343
-57.441380 64.583340 324.023000 7.114175
-55.176610 64.408980 326.356100 4.075769
-52.946510 64.200520 328.199400 1.840796
-50.755950 63.959000 329.088700 .702909
-48.608900 63.685590 329.426500 .235710
-46.508400 63.381530 329.539200 .069685
-44.456330 63.048090 329.571600 .018626
-42.453320 62.686580 329.579800 .004327
-40.498620 62.298210 329.581600 .000897
-38.589950 61.884150 329.581900 .000177
-36.723370 61.445380 329.582000 .000063
-34.893150 60.982680 329.582100 .000036
-33.091610 60.496560 329.582000 .000000
-31.309040 59.987160 329.582000 .000000
-29.533510 59.454220 329.582100 .000031
-27.750760 58.896990 329.582100 .000031
-107.339200 60.027230 329.581300 .001813
-105.487000 60.591610 329.578900 .006656
-103.643800 61.130270 329.568000 .026394
-101.793500 61.644070 329.526500 .090696
-99.922550 62.133320 329.388200 .274038
-98.020040 62.597820 328.974600 .742201
-96.077510 63.037030 327.837400 1.804216
-94.088710 63.450070 325.153700 3.832882
-92.049460 63.835860 319.174300 7.674660
-89.957580 64.193130 307.262000 13.673210
-87.812570 64.520550 310.001200 20.170740
-85.615560 64.816710 369.462000 47.248870
-83.369120 65.080260 428.755500 85.757910
-81.077050 65.309890 392.611500 50.201630
-78.744270 65.504390 368.049400 34.472390
-76.376630 65.662700 361.525000 31.710330
-73.980770 65.783920 367.788300 36.487150
-71.563890 65.867360 384.289900 46.148700
-69.133670 65.912500 401.861700 52.431380
-66.698060 65.919100 379.699500 40.232080
-64.265090 65.887100 353.783600 22.394630
-61.842790 65.816700 336.798000 9.165425
-59.438900 65.708320 329.757000 3.680969
-57.060850 65.562580 328.231700 2.456779
-54.715470 65.380330 328.562300 1.531509
-52.408880 65.162570 329.072300 .763089

-50.146370	64.910470	329.376300	.318767
-47.932120	64.625290	329.512700	.114377
-45.769150	64.308400	329.561900	.036283
-43.659080	63.961160	329.577100	.009861
-41.602020	63.584970	329.581000	.002374
-39.596350	63.181100	329.581800	.000531
-37.638600	62.750720	329.582000	.000103
-35.723270	62.294770	329.582000	.000061
-33.842640	61.813940	329.582100	.000031
-31.986660	61.308540	329.582100	.000031
-30.142740	60.778430	329.582000	.000000
-28.295590	60.222970	329.582100	.000031
-26.427080	59.640870	329.582100	.000031
-108.736700	60.768890	329.581000	.002514
-106.789100	61.359350	329.578500	.007470
-104.866900	61.921510	329.567400	.027175
-102.949500	62.456830	329.528000	.087871
-101.019200	62.966030	329.405300	.251832
-99.061880	63.449270	329.064500	.650651
-97.066180	63.906190	328.216700	1.487304
-95.023590	64.336020	326.444200	2.925372
-92.928180	64.737730	323.776600	4.861738
-90.776390	65.110020	320.347600	8.438037
-88.566850	65.451470	325.451800	14.607470
-86.300130	65.760610	370.122800	38.068970
-83.978550	66.035940	374.583100	39.112570
-81.606060	66.276030	362.185600	28.657930
-79.187900	66.479550	353.813000	23.910020
-76.730480	66.645290	349.257800	21.404480
-74.241170	66.772270	351.194900	23.228050
-71.728080	66.859700	357.892600	27.797900
-69.199870	66.907030	361.792800	29.427890
-66.665550	66.913940	354.893400	24.019530
-64.134220	66.880400	343.653000	14.690640
-61.614990	66.806620	335.074100	6.909554
-59.116610	66.693070	330.859800	2.612516
-56.647430	66.540450	329.452900	1.099940
-54.215080	66.349720	329.246500	.664460
-51.826330	66.121990	329.360400	.372139
-49.486860	65.858530	329.480300	.173377
-47.201050	65.560780	329.544500	.068677
-44.971820	65.230190	329.570700	.022623
-42.800360	64.868270	329.579100	.006507
-40.685980	64.476460	329.581400	.001660
-38.625890	64.056100	329.581900	.000384
-36.614990	63.608330	329.582000	.000094
-34.645670	63.134020	329.582100	.000033
-32.707610	62.633670	329.582100	.000031
-30.787590	62.107330	329.582100	.000031
-28.869240	61.554470	329.582100	.000031
-26.932890	60.973880	329.582100	.000031
-24.955330	60.363580	329.582100	.000031

Q.16a

-89.453400	32.991660	347.833500	1.096995
-88.345930	33.191160	348.064800	1.446266
-87.228100	33.381660	348.755900	2.417611
-86.100230	33.562890	349.892500	3.944462
-84.962690	33.734530	351.311400	5.793193
-83.815900	33.896310	352.706900	7.599692
-82.660300	34.047950	353.824000	9.056848
-81.496410	34.189190	354.551400	9.997955
-80.324730	34.319790	354.836100	10.329790
-79.145840	34.439500	354.603900	9.961102
-77.960310	34.548100	353.751400	8.855009
-76.768760	34.645400	352.243000	7.269646
-75.571840	34.731200	350.607100	5.829886
-74.370180	34.805370	349.729700	4.905482
-73.164480	34.867740	349.383100	4.486191
-71.955430	34.918200	349.290300	4.096415
-70.743730	34.956650	349.333700	3.818181
-69.530080	34.983020	349.279000	3.444764
-68.315220	34.997260	349.055400	2.960056
-67.099880	34.999340	348.647400	2.312011
-65.884770	34.989250	348.155300	1.597464
-64.670640	34.967020	347.729100	.957484
-63.458190	34.932690	347.447000	.495342
-62.248150	34.886320	347.298100	.221112
-61.041230	34.828000	347.231900	.084342
-59.838120	34.757850	347.206600	.026814
-58.639500	34.676010	347.198400	.006576
-57.446040	34.582610	347.196300	.000979
-56.258390	34.477850	347.196000	.000388
-55.077160	34.361910	347.196000	.000307
-53.902950	34.235010	347.196100	.000163
-52.736340	34.097380	347.196200	.000100
-51.577860	33.949260	347.196200	.000070
-50.428010	33.790910	347.196200	.000061
-49.287250	33.622600	347.196200	.000061
-48.156040	33.444620	347.196200	.000061
-47.034740	33.257250	347.196200	.000061
-45.923690	33.060790	347.196200	.000061
-44.823220	32.855560	347.196200	.000061
-89.701210	33.918400	348.335000	1.673778
-88.583520	34.125020	348.899100	2.392355
-87.455040	34.322360	350.473100	4.246020
-86.316120	34.510110	353.140000	7.204924
-85.167100	34.687960	356.527700	10.815980
-84.008420	34.855620	359.809400	14.315700
-82.840550	35.012790	362.357200	17.085260
-81.663990	35.159200	363.983800	18.846430
-80.479300	35.294580	364.629900	19.474270
-79.287040	35.418700	364.159600	18.757410
-78.087840	35.531310	362.227600	16.564530
-76.882320	35.632210	358.716600	13.479910
-75.671150	35.721200	354.597500	10.532100
-74.455030	35.798110	353.003300	9.400545
-73.234660	35.862810	352.649500	8.417235
-72.010770	35.915150	352.419500	8.036550
-70.784100	35.955040	352.417400	7.439256
-69.555380	35.982390	352.316400	6.836586

-68.325390	35.997170	351.733900	5.871665
-67.094890	35.999320	350.689000	4.592007
-65.864640	35.988860	349.437700	3.143804
-64.635410	35.965800	348.385300	1.854302
-63.407950	35.930180	347.727700	.941374
-62.183030	35.882080	347.400400	.414706
-60.961370	35.821590	347.264300	.157207
-59.743720	35.748840	347.214700	.049054
-58.530780	35.663950	347.199200	.010972
-57.323260	35.567100	347.195600	.001742
-56.121820	35.458470	347.195300	.001602
-54.927120	35.338260	347.195700	.000975
-53.739770	35.206700	347.196000	.000443
-52.560380	35.064020	347.196100	.000178
-51.389480	34.910500	347.196200	.000104
-50.227610	34.746390	347.196200	.000087
-49.075240	34.571990	347.196200	.000064
-47.932830	34.387580	347.196200	.000061
-46.800740	34.193490	347.196200	.000061
-45.679330	33.990000	347.196200	.000061
-44.568900	33.777440	347.196200	.000061
-89.960660	34.843320	349.759200	3.045655
-88.832330	35.057210	351.387300	4.738316
-87.692770	35.261540	355.843000	8.983156
-86.542320	35.455950	363.758500	15.939850
-85.381340	35.640140	374.094000	24.580310
-84.210270	35.813810	383.882700	32.857510
-83.029580	35.976620	391.220500	39.287940
-81.839790	36.128310	395.804900	43.328130
-80.641480	36.268600	397.668100	44.774490
-79.435230	36.397220	396.373200	43.101490
-78.221690	36.513940	390.988900	37.936300
-77.001530	36.618520	380.514000	30.201810
-75.775430	36.710780	366.737600	23.380800
-74.544130	36.790520	364.240300	21.097680
-73.308360	36.857590	363.373600	20.675420
-72.068890	36.911860	363.669600	18.634810
-70.826490	36.953220	363.322200	17.810790
-69.581950	36.981590	362.888100	16.333120
-68.336070	36.996900	361.170300	14.192560
-67.089650	36.999130	357.817700	11.055970
-65.843500	36.988290	353.813900	7.553251
-64.598400	36.964370	350.531000	4.369421
-63.355190	36.927440	348.601400	2.184612
-62.114630	36.877570	347.706100	.957539
-60.877510	36.814860	347.356400	.368248
-59.644590	36.739430	347.235600	.117292
-58.416640	36.651430	347.199200	.027231
-57.194370	36.551040	347.191900	.008625
-55.978490	36.438440	347.192600	.006739
-54.769680	36.313860	347.194300	.003700
-53.568590	36.177530	347.195300	.001549
-52.375820	36.029710	347.195800	.000558
-51.191970	35.870660	347.196000	.000160
-50.017550	35.700660	347.196000	.000085
-48.853080	35.520030	347.196000	.000048
-47.698980	35.329070	347.196000	.000031
-46.555660	35.128100	347.196000	.000032
-45.423470	34.917440	347.196000	.000032
-44.302690	34.697410	347.196200	.000061
-90.232250	35.766320	352.279100	4.735488
-89.092860	35.987640	356.337800	8.102781
-87.941790	36.199090	367.854500	16.777110
-86.779330	36.400320	390.238200	31.932690
-85.605870	36.591000	420.584800	51.396930
-84.421870	36.770790	448.622600	69.623090
-83.227800	36.939400	468.530400	83.311550

-82.024180	37.096500	480.679300	91.990190
-80.811620	37.241800	485.678500	95.359330
-79.590740	37.375040	482.633400	92.108870
-78.362190	37.495960	467.979900	80.682590
-77.126670	37.604320	436.411800	61.995770
-75.884930	37.699920	390.596900	43.017270
-74.637700	37.782550	406.146400	51.203830
-73.385770	37.852060	392.168400	44.511500
-72.129940	37.908310	392.302600	42.366100
-70.871030	37.951180	393.780400	38.214200
-69.609860	37.980580	392.487500	36.011210
-68.347290	37.996460	387.529400	31.412750
-67.084140	37.998780	377.713800	25.000150
-65.821270	37.987530	365.467400	16.836750
-64.559530	37.962740	355.722100	9.332911
-63.299760	37.924460	350.489200	4.496838
-62.042780	37.872770	348.281800	1.943675
-60.789440	37.807780	347.498400	.753034
-59.540510	37.729610	347.250400	.247900
-58.296810	37.638420	347.182200	.069454
-57.059080	37.534390	347.174700	.036791
-55.828080	37.417740	347.182400	.024294
-54.604500	37.288680	347.189700	.012361
-53.389020	37.147460	347.193600	.005067
-52.182290	36.994370	347.195200	.001750
-50.984880	36.829660	347.195800	.000530
-49.797380	36.653650	347.196000	.000163
-48.620270	36.466650	347.196000	.000093
-47.454020	36.268990	347.196000	.000064
-46.299010	36.060990	347.196000	.000031
-45.155610	35.843010	347.196000	.000031
-44.024070	35.615370	347.196200	.000061
-90.516530	36.687310	354.866800	5.559711
-89.365660	36.916220	362.852600	10.384580
-88.202590	37.134950	388.508900	24.905290
-87.027650	37.343150	446.140500	54.046960
-85.841200	37.540460	531.186500	96.083720
-84.643710	37.726540	605.149500	131.624800
-83.435650	37.901050	653.086500	156.688300
-82.217600	38.063690	682.437200	172.484200
-80.990140	38.214140	695.403400	179.905200
-79.753940	38.352110	690.234600	175.305000
-78.509670	38.477340	654.812300	154.018200
-77.258070	38.589580	560.276500	111.066100
-75.999910	38.688600	389.443500	75.913370
-74.735980	38.774210	434.315000	96.814710
-73.467090	38.846230	519.183300	101.849900
-72.194090	38.904510	462.288200	81.581310
-70.917830	38.948930	465.075800	75.920140
-69.639210	38.979390	467.056900	70.496380
-68.359090	38.995840	454.955300	64.543980
-67.078360	38.998240	429.386400	51.251890
-65.797930	38.986590	393.774700	35.197800
-64.518690	38.960900	365.868800	17.972980
-63.241520	38.921240	353.351400	7.796968
-61.967320	38.867690	348.917800	3.286392
-60.696920	38.800350	347.532300	1.298300
-59.431210	38.719360	347.156900	.470236
-58.170970	38.624900	347.084600	.201296
-56.917050	38.517150	347.110100	.127519
-55.670200	38.396340	347.149500	.074846
-54.431160	38.262690	347.176000	.035279
-53.200640	38.116470	347.188900	.013819
-51.979310	37.957960	347.193800	.004653
-50.767770	37.787460	347.195300	.001366
-49.566610	37.605300	347.195900	.000381
-48.376340	37.411790	347.196000	.000134

-47.197410 37.207270 347.196000 .000088
-46.030250 36.992090 347.196000 .000070
-44.875180 36.766620 347.196000 .000031
-43.732470 36.531200 347.196200 .000061
-90.814130 37.606180 353.315600 6.849231
-89.651300 37.842850 363.586500 9.131323
-88.475770 38.069030 405.926100 25.178720
-87.287830 38.284350 540.303900 75.765770
-86.087840 38.488450 777.702800 161.406900
-84.876290 38.680970 957.201800 226.100900
-83.653650 38.861550 1053.348000 264.949600
-82.420500 39.029860 1115.910000 288.190700
-81.177470 39.185590 1148.382000 300.756200
-79.925230 39.328410 1143.369000 300.407500
-78.664510 39.458070 1081.307000 271.635600
-77.396060 39.574290 843.683000 189.883900
-76.120680 39.676830 326.263400 155.380900
-74.839220 39.765490 776.606200 481.561200
-73.552520 39.840080 506.357000 218.044600
-72.261480 39.900440 677.578700 143.242200
-70.967010 39.946460 621.037500 130.292600
-69.670030 39.978020 639.199600 126.861400
-68.371470 39.995060 622.665500 117.629000
-67.072270 39.997550 557.775000 99.737530
-65.773400 39.985470 461.153400 68.143320
-64.475770 39.958870 381.850200 32.106030
-63.180340 39.917780 354.542700 12.091280
-61.888020 39.862300 348.273000 4.843297
-60.599740 39.792560 346.925700 2.152383
-59.316390 39.708680 346.643700 1.023642
-58.038840 39.610860 346.709000 .618466
-56.767930 39.499290 346.904000 .377855
-55.504470 39.374200 347.060600 .193295
-54.249260 39.235840 347.142900 .084269
-53.003000 39.084480 347.178300 .030719
-51.766410 38.920440 347.190900 .009714
-50.540120 38.744010 347.194600 .002704
-49.324710 38.555530 347.195700 .000693
-48.120720 38.355340 347.195900 .000183
-46.928620 38.143810 347.196000 .000109
-45.748810 37.921300 347.196000 .000092
-44.581620 37.688170 347.196000 .000043
-43.427300 37.444810 347.196200 .000061
-91.125630 38.522790 341.817800 16.354090
-89.950410 38.767390 336.610100 21.758010
-88.761920 39.001200 351.534800 26.154400
-87.560450 39.223820 612.608600 88.898640
-86.346370 39.434880 1228.964000 239.140800
-85.120160 39.633990 1651.201000 345.424700
-83.882300 39.820790 1857.189000 397.287800
-82.633390 39.994930 1952.873000 427.643500
-81.374080 40.156070 2011.034000 450.285600
-80.105060 40.303890 2036.399000 462.692000
-78.827100 40.438090 2013.690000 437.997800
-77.540980 40.558400 1635.590000 343.703700
-76.247540 40.664560 557.127400 275.039100
-74.947690 40.756360 49.677550 188.661200
-73.642300 40.833600 739.695900 383.195900
-72.332310 40.896110 675.166600 236.427700
-71.018690 40.943760 948.846200 196.947100
-69.702430 40.976440 949.375500 196.482300
-68.384490 40.994090 910.052700 189.211000
-67.065880 40.996670 655.943700 146.389000
-65.747600 40.984170 484.120500 89.987480
-64.430650 40.956610 385.828200 42.799900
-63.116020 40.914060 347.019900 18.618810
-61.804690 40.856610 343.238100 8.393056

-60.497620	40.784390	344.420500	4.316481
-59.195770	40.697540	344.976500	2.711373
-57.900040	40.596260	345.558400	1.833119
-56.611330	40.480750	346.349300	.932544
-55.330490	40.351270	346.858300	.423477
-54.058330	40.208070	347.084300	.156502
-52.795620	40.051450	347.162900	.051365
-51.543080	39.881710	347.187600	.014204
-50.301390	39.699200	347.194200	.003446
-49.071130	39.504250	347.195600	.000737
-47.852850	39.297230	347.195900	.000180
-46.647030	39.078520	347.196000	.000088
-45.454070	38.848490	347.196000	.000104
-44.274280	38.607530	347.196000	.000052
-43.107920	38.356030	347.196200	.000061
-91.451740	39.437030	324.491700	25.709560
-90.263630	39.689750	259.824800	38.464450
-89.061680	39.931370	116.416700	110.438800
-87.846150	40.161480	548.325700	168.629100
-86.617400	40.379670	1424.456000	233.938000
-85.375900	40.585540	2244.955000	409.271900
-84.122160	40.778730	2725.019000	519.414200
-82.856800	40.958850	2959.422000	574.132400
-81.580460	41.125550	3102.872000	611.813800
-80.293880	41.278500	3227.791000	652.511500
-78.997860	41.417370	3282.656000	668.146200
-77.693230	41.541890	3002.918000	595.447600
-76.380870	41.651780	1815.432000	361.338100
-75.061690	41.746810	202.911800	188.957600
-73.736660	41.826770	84.991080	198.330500
-72.406780	41.891490	736.546500	167.910500
-71.073040	41.940830	1159.443000	256.167400
-69.736500	41.974670	1099.461000	237.382900
-68.398190	41.992950	797.352300	193.840500
-67.059160	41.995620	407.616800	227.589200
-65.720480	41.982670	128.795300	119.853800
-64.383220	41.954140	246.718100	72.625660
-63.048400	41.910080	311.806900	29.465390
-61.717090	41.850600	332.423300	16.134280
-60.390290	41.775820	338.704400	9.964085
-59.069010	41.685920	340.253100	7.667867
-57.754210	41.581080	341.995500	5.080155
-56.446830	41.461530	345.206800	2.258347
-55.147770	41.327530	346.541000	.683568
-53.857880	41.179360	347.029500	.210351
-52.577970	41.017320	347.163800	.048270
-51.308780	40.841740	347.191500	.010869
-50.051000	40.652980	347.196000	.003436
-48.805250	40.451390	347.196300	.001309
-47.572090	40.237360	347.196100	.000386
-46.352010	40.011290	347.196000	.000130
-45.145370	39.773560	347.196000	.000108
-43.952510	39.524580	347.196000	.000031
-42.773630	39.264750	347.196200	.000061
-91.793200	40.348750	349.731700	18.171510
-90.591710	40.609810	306.612500	42.346710
-89.375760	40.859430	161.670000	145.338600
-88.145600	41.097210	246.111400	139.034100
-86.901570	41.322720	764.218500	166.380300
-85.644150	41.535550	1498.627000	176.434200
-84.373860	41.735290	2344.366000	377.956800
-83.091290	41.921560	2956.814000	555.070800
-81.797160	42.093980	3403.347000	670.927200
-80.492230	42.252200	3760.545000	754.587300
-79.177290	42.395880	3946.483000	793.570600
-77.853240	42.524730	3721.404000	734.273800
-76.521000	42.638450	2767.011000	487.611700

-75.181550	42.736820	1313.258000	205.513800
-73.835900	42.819590	656.702600	202.589300
-72.485100	42.886590	879.133200	219.124800
-71.130210	42.937670	1218.320000	281.653000
-69.772340	42.972710	910.485500	212.471200
-68.412590	42.991630	469.686900	194.269300
-67.052090	42.994400	80.792280	134.927300
-65.691960	42.980990	35.032360	116.583300
-64.333320	42.951450	263.719900	184.277900
-62.977290	42.905830	300.341800	79.750170
-61.624970	42.844260	324.025600	28.452470
-60.277440	42.766850	327.362400	23.935400
-58.935750	42.673790	325.582700	23.143900
-57.600940	42.565280	330.960700	15.003100
-56.273990	42.441570	342.994400	4.325150
-54.955840	42.302930	346.598200	1.048903
-53.647390	42.149640	347.172200	.232450
-52.349470	41.982030	347.243600	.118978
-51.062890	41.800450	347.223600	.058445
-49.788320	41.605260	347.206100	.021966
-48.526440	41.396860	347.198900	.006994
-47.277780	41.175640	347.196700	.001910
-46.042850	40.942000	347.196200	.000427
-44.822010	40.696380	347.196000	.000104
-43.615560	40.439180	347.196000	.000031
-42.423690	40.170830	347.196200	.000061
-92.150770	41.257820	417.882300	36.501510
-90.935390	41.527410	496.343200	60.779300
-89.704910	41.785250	574.809000	101.583500
-88.459540	42.030900	563.013200	101.645100
-87.199620	42.263930	386.027200	239.786500
-85.925610	42.483890	794.467200	203.498200
-84.638040	42.690380	1768.236000	255.011400
-83.337520	42.882970	2621.258000	497.815500
-82.024790	43.061280	3306.087000	678.951600
-80.700610	43.224930	3843.275000	791.424600
-79.365870	43.373580	3957.399000	815.968400
-78.021450	43.506890	3685.835000	742.525800
-76.668370	43.624570	3068.472000	580.161400
-75.307630	43.726360	2240.793000	374.680000
-73.940300	43.812040	1608.241000	312.620000
-72.567500	43.881390	1098.332000	226.853500
-71.190370	43.934270	1142.560000	245.235600
-69.810050	43.970550	1105.222000	243.797700
-68.427750	43.990140	837.309800	198.192800
-67.044650	43.993000	361.403800	113.974700
-65.661930	43.979120	386.632300	116.777200
-64.280810	43.948540	425.988600	149.107600
-62.902460	43.901310	324.281900	71.953170
-61.528050	43.837570	304.161100	58.562330
-60.158730	43.757450	293.870000	61.050640
-58.795620	43.661140	297.422500	60.640390
-57.439790	43.548850	331.323700	21.388670
-56.092310	43.420850	342.438700	6.972760
-54.754150	43.277410	348.264800	1.467780
-53.426270	43.118850	348.035200	.991049
-52.109530	42.945500	347.534500	.502751
-50.804760	42.757750	347.312500	.194259
-49.512690	42.555960	347.231300	.066535
-48.233980	42.340550	347.205400	.019670
-46.969190	42.111940	347.198200	.005104
-45.718810	41.870550	347.196500	.001154
-44.483210	41.616830	347.196100	.000226
-43.262650	41.351200	347.196100	.000090
-42.057300	41.074100	347.196200	.000061
-92.525320	42.164070	453.057300	58.713330
-91.295520	42.442420	550.440300	89.034430

-90.049940	42.708690	798.812000	149.849500
-88.788760	42.962440	978.071900	172.967500
-87.512310	43.203190	812.387000	144.322300
-86.221000	43.430500	704.457500	193.902100
-84.915410	43.643920	1402.712000	226.477200
-83.596150	43.843030	2408.480000	477.274000
-82.263960	44.027400	3238.246000	674.856800
-80.919660	44.196660	3858.046000	806.108300
-79.564150	44.350410	3909.103000	804.516400
-78.198390	44.488330	3340.799000	671.080800
-76.823410	44.610100	2514.532000	452.869700
-75.440290	44.715440	1817.928000	259.062900
-74.050180	44.804110	1146.960000	187.619700
-72.654240	44.875890	458.927000	221.301100
-71.253690	44.930630	683.218400	142.514100
-69.849760	44.968180	1201.071000	238.105600
-68.443720	44.988460	898.663900	159.487200
-67.036810	44.991420	395.129200	192.024700
-65.630330	44.977050	570.066300	121.993600
-64.225530	44.945390	608.262200	90.369150
-62.823690	44.896510	417.811800	75.752160
-61.426040	44.830540	287.082700	132.607500
-60.033810	44.747610	283.469100	117.128100
-58.648170	44.647940	325.398200	60.665580
-57.270290	44.531750	367.627900	21.078140
-55.901260	44.399320	372.181500	17.830780
-54.542130	44.250930	355.533600	7.217821
-53.193900	44.086940	349.870800	2.958981
-51.857480	43.907680	348.082800	1.144650
-50.533710	43.713560	347.468700	.422543
-49.223370	43.504980	347.272400	.136689
-47.927120	43.282370	347.215500	.039441
-46.645540	43.046150	347.200600	.010124
-45.379100	42.796800	347.196900	.002282
-44.128160	42.534760	347.196200	.000443
-42.892960	42.260480	347.196100	.000099
-41.673610	41.974410	347.196200	.000061
-92.917770	43.067330	456.129600	64.144600
-91.673000	43.354680	543.044500	90.899120
-90.411740	43.629630	783.575400	154.598400
-89.134110	43.891690	1034.113000	225.231800
-87.840450	44.140390	833.917100	159.525600
-86.531140	44.375260	282.334900	249.924800
-85.206750	44.595830	1116.721000	208.587900
-83.867900	44.801650	2251.443000	432.086500
-82.515380	44.992280	3078.566000	653.487700
-81.150000	45.167310	3572.410000	748.993200
-79.772730	45.326340	3520.302000	731.365400
-78.384560	45.469020	2971.982000	581.545500
-76.986600	45.595010	2120.176000	330.586500
-75.579970	45.704020	1166.444000	105.945300
-74.165890	45.795780	356.592800	254.258800
-72.745610	45.870080	62.441490	80.971310
-71.320400	45.926740	399.776500	192.277500
-69.891600	45.965610	905.204800	135.024200
-68.460530	45.986610	638.519000	161.213800
-67.028560	45.989670	163.258800	153.938600
-65.597030	45.974800	602.766000	111.694900
-64.167300	45.942020	710.994100	98.299740
-62.740720	45.891430	574.033400	71.062780
-61.318600	45.823140	501.119300	70.595660
-59.902260	45.737310	473.811600	43.649430
-58.492950	45.634170	450.799700	41.369610
-57.091900	45.513950	419.224500	40.936670
-55.700260	45.376930	375.824100	21.171360
-54.319140	45.223450	358.482100	10.154820
-52.949590	45.053840	351.440600	4.520494

-51.592580	44.868490	348.632700	1.882442
-50.248970	44.667810	347.639600	.687209
-48.919560	44.452230	347.321500	.223220
-47.605040	44.222190	347.228100	.064339
-46.305980	43.978160	347.203400	.016518
-45.022840	43.720610	347.197500	.003783
-43.755960	43.450020	347.196300	.000754
-42.505550	43.166850	347.196100	.000128
-41.271660	42.871580	347.196200	.000061
-93.329110	43.967430	437.517600	57.330480
-92.068780	44.264030	500.833800	73.681160
-90.791210	44.547890	673.947300	114.187900
-89.496510	44.818510	861.006600	145.279800
-88.184940	45.075390	699.929700	92.401760
-86.856880	45.318050	569.012900	214.751700
-85.512890	45.545990	1446.557000	194.008800
-84.153590	45.758730	2466.884000	482.826500
-82.779790	45.955820	2981.235000	620.102400
-81.392350	46.136820	3113.000000	659.097200
-79.992260	46.301310	2951.138000	602.554300
-78.580580	46.448910	2483.603000	469.718800
-77.158460	46.579270	1835.309000	297.807300
-75.727110	46.692070	1257.640000	120.617500
-74.287820	46.787040	674.256800	98.955740
-72.841890	46.863950	261.299400	259.423800
-71.390720	46.922590	575.015400	149.048400
-69.935700	46.962830	1164.279000	219.936100
-68.478260	46.984570	1014.987000	154.352700
-67.019850	46.987740	813.705000	123.904500
-65.561920	46.972340	830.661600	117.178000
-64.105920	46.938410	824.483200	116.376500
-62.653260	46.886040	762.776400	98.935710
-61.205390	46.815350	675.429400	83.426960
-59.763680	46.726520	590.344500	73.863200
-58.329470	46.619790	501.331300	57.760520
-56.904060	46.495390	428.719400	39.158010
-55.488670	46.353640	386.046100	24.154430
-54.084500	46.194880	363.631300	13.089560
-52.692630	46.019470	353.380200	6.300585
-51.314070	45.827840	349.312100	2.628582
-49.949730	45.620390	347.856300	.971716
-48.600430	45.397590	347.383200	.318333
-47.266860	45.159900	347.244100	.092736
-45.949600	44.907820	347.207300	.024111
-44.649100	44.641840	347.198400	.005578
-43.365670	44.362450	347.196400	.001140
-42.099450	44.070140	347.196100	.000197
-40.850440	43.765410	347.196200	.000063
-93.760410	44.864160	412.539900	44.151250
-92.483920	45.170280	444.662000	48.139320
-91.189420	45.463320	508.396800	56.447500
-89.876960	45.742750	560.390900	57.213410
-88.546760	46.008070	556.069100	107.464300
-87.199170	46.258760	806.940500	91.667460
-85.834720	46.494300	1516.315000	235.659000
-84.454070	46.714200	2243.869000	440.449800
-83.058010	46.917960	2576.521000	528.502000
-81.647480	47.105130	2608.473000	533.117400
-80.223460	47.275260	2325.288000	452.826100
-78.787090	47.427960	1690.718000	319.729700
-77.339580	47.562840	1269.153000	236.920100
-75.882230	47.679570	1149.030000	205.078500
-74.416370	47.777870	1219.770000	196.258700
-72.943440	47.857470	1134.288000	234.842500
-71.464880	47.918190	1162.842000	215.032000
-69.982220	47.959850	1278.447000	207.267500
-68.496960	47.982350	1202.148000	193.615400

-67.010670	47.985630	1041.996000	141.405900
-65.524890	47.969690	809.747900	90.661610
-64.041170	47.934570	708.328400	88.300510
-62.561040	47.880340	724.908500	70.637590
-61.086020	47.807170	720.205300	69.811070
-59.617590	47.715230	658.372100	70.033400
-58.157170	47.604770	564.146700	54.712270
-56.706150	47.476050	467.002900	43.319190
-55.265830	47.329400	404.296100	28.220460
-53.837470	47.165180	370.960200	16.124160
-52.422200	46.983780	356.001100	7.849837
-51.021090	46.785640	350.168400	3.325709
-49.635090	46.571200	348.113600	1.238418
-48.265020	46.340950	347.454400	.409571
-46.911610	46.095370	347.262400	.120643
-45.575410	45.834990	347.211500	.031679
-44.256860	45.560320	347.199300	.007428
-42.956210	45.271870	347.196700	.001533
-41.673580	44.970160	347.196100	.000274
-40.408870	44.655690	347.196200	.000094
-94.212860	45.757310	398.033400	35.084000
-92.919560	46.073230	418.865500	36.354640
-91.607470	46.375730	469.502000	44.571660
-90.276550	46.664250	552.146100	58.646580
-88.926960	46.938270	581.428600	65.973530
-87.559010	47.197250	683.273600	97.711150
-86.173240	47.440650	1035.703000	171.345600
-84.770290	47.667940	1533.345000	271.804200
-83.350960	47.878600	2012.162000	387.669900
-81.916200	48.072160	2183.474000	418.636100
-80.467090	48.248140	1835.157000	346.102900
-79.004780	48.406110	1204.087000	251.418700
-77.530590	48.545680	875.703500	240.696900
-76.045850	48.666500	972.645600	199.222100
-74.552020	48.768250	1114.278000	196.914400
-73.050600	48.850660	1154.346000	195.660400
-71.543170	48.913520	1155.564000	203.416500
-70.031330	48.956650	1245.717000	227.876000
-68.516710	48.979950	1260.631000	218.502700
-67.000980	48.983350	1151.558000	188.494900
-65.485790	48.966840	870.019500	119.498900
-63.972820	48.930470	751.519800	86.831440
-62.463700	48.874330	666.768200	123.967100
-60.960050	48.798580	698.990300	105.770100
-59.463460	48.703410	726.572000	64.073970
-57.975440	48.589080	683.196000	67.842610
-56.497490	48.455880	526.763000	47.956700
-55.030970	48.304140	425.176000	32.543050
-53.577210	48.134270	378.215800	18.094370
-52.137430	47.946660	358.401100	8.871256
-50.712720	47.741790	350.888200	3.772125
-49.304080	47.520120	348.316300	1.410973
-47.912350	47.282170	347.508800	.468226
-46.538250	47.028450	347.275900	.138379
-45.182340	46.759510	347.214600	.036532
-43.845020	46.475880	347.199900	.008617
-42.526500	46.178100	347.196700	.001793
-41.226800	45.866700	347.196200	.000333
-39.945740	45.542200	347.196200	.000100
-94.687740	46.646660	397.537900	32.693200
-93.376940	46.972690	413.631000	33.636910
-92.046550	47.284930	450.324000	42.335320
-90.696440	47.582840	453.132800	44.800460
-89.326680	47.865840	386.406300	117.971700
-87.937550	48.133390	451.730600	162.306900
-86.529520	48.384910	766.582000	141.538800
-85.103260	48.619850	1251.999000	195.887400

-83.659580	48.837660	1730.887000	296.983400
-82.199460	49.037830	1885.222000	356.830100
-80.724000	49.219880	1652.827000	316.770800
-79.234450	49.383340	1207.420000	245.194200
-77.732160	49.527770	1093.108000	213.718400
-76.218590	49.652830	1121.673000	202.463600
-74.695260	49.758160	1076.501000	182.763600
-73.163800	49.843490	976.983600	165.368300
-71.625870	49.908580	969.187700	171.967600
-70.083210	49.953250	1070.878000	194.059400
-68.537570	49.977380	1051.097000	195.114600
-66.990740	49.980900	939.034100	163.260000
-65.444490	49.963800	794.819600	147.410700
-63.900620	49.926140	637.138300	123.860700
-62.360880	49.868010	591.556800	96.612220
-60.827010	49.789570	649.845600	77.118210
-59.300730	49.691040	789.076400	102.883500
-57.783640	49.572690	725.228800	91.064980
-56.277320	49.434830	561.197800	59.081410
-54.783270	49.277820	432.868600	35.086900
-53.302860	49.102080	379.598000	18.806640
-51.837360	48.908040	358.667400	8.928169
-50.387950	48.696200	350.937100	3.763418
-48.955640	48.467040	348.322000	1.399566
-47.541310	48.221130	347.508100	.462462
-46.145660	47.959000	347.275400	.136258
-44.769230	47.681210	347.214400	.035859
-43.412380	47.388330	347.199800	.008425
-42.075270	47.080920	347.196700	.001752
-40.757810	46.759540	347.196200	.000323
-39.459720	46.424710	347.196200	.000094
-95.186440	47.531940	415.015000	38.357100
-93.857410	47.868410	439.038600	41.978260
-92.508000	48.190720	472.890600	53.816480
-91.137910	48.498310	470.267300	58.008930
-89.747150	48.790600	463.368000	97.287350
-88.335950	49.067010	663.195100	83.450510
-86.904710	49.326930	1010.175000	155.380800
-85.454100	49.569790	1282.788000	215.094300
-83.984930	49.795020	1473.426000	274.705600
-82.498220	50.002060	1523.119000	302.443400
-80.995120	50.190400	1383.795000	289.608000
-79.476920	50.359550	1239.210000	251.974600
-77.945060	50.509060	1200.092000	231.148200
-76.401090	50.638520	1154.750000	210.573200
-74.846650	50.747580	1081.820000	179.277100
-73.283450	50.835950	966.000900	154.290300
-71.713320	50.903350	919.039000	150.171000
-70.138080	50.949630	956.218100	165.421400
-68.559640	50.974620	895.318700	161.316000
-66.979900	50.978270	744.596900	130.201400
-65.400800	50.960560	583.421600	115.843800
-63.824260	50.921550	394.279800	115.191700
-62.252170	50.861340	103.521700	125.029400
-60.686400	50.780110	480.339400	83.841450
-59.128770	50.678080	671.879500	101.766800
-57.581030	50.555550	645.739500	90.007660
-56.044860	50.412850	491.546600	56.152440
-54.521820	50.250360	409.725500	31.890060
-53.013420	50.068520	372.159800	16.428670
-51.520960	49.867810	356.190500	7.762462
-50.045680	49.648740	350.157800	3.226274
-48.588630	49.411830	348.093900	1.185435
-47.150700	49.157670	347.445900	.387609
-45.732590	48.886830	347.259400	.112924
-44.334810	48.599900	347.210700	.029366
-42.957660	48.297460	347.199100	.006813

-41.601190	47.980120	347.196600	.001394
-40.265250	47.648430	347.196100	.000245
-38.949390	47.302930	347.196200	.000066
-95.710510	48.412890	452.027800	50.971820
-94.362470	48.760130	496.812700	59.650480
-92.993230	49.092850	544.531900	75.510850
-91.602360	49.410460	575.340500	81.480970
-90.189750	49.712350	608.118000	81.713550
-88.755540	49.997940	755.958200	114.118400
-87.300090	50.266570	939.105000	163.863400
-85.824040	50.517650	1020.999000	189.326800
-84.328210	50.750570	1086.292000	217.440800
-82.813610	50.964740	1078.729000	237.751300
-81.281460	51.159630	1091.657000	236.244200
-79.733130	51.334700	1054.044000	226.742900
-78.170130	51.489480	972.365800	201.918200
-76.594090	51.623530	905.625100	171.657300
-75.006800	51.736490	879.480800	147.359900
-73.410070	51.828010	870.281200	137.565000
-71.805870	51.897850	823.809300	133.343500
-70.196170	51.945790	844.383700	144.533000
-68.582990	51.971680	740.960400	132.948800
-66.968440	51.975460	602.051800	104.360100
-65.354560	51.957120	661.967700	110.128300
-63.743440	51.916690	556.948000	92.156240
-62.137130	51.854320	410.063100	71.261240
-60.537630	51.770170	469.411800	71.530400
-58.946900	51.664510	571.103500	98.494960
-57.366820	51.537620	492.975900	60.635790
-55.799180	51.389870	428.420800	42.713730
-54.245650	51.221680	385.447700	24.321510
-52.707820	51.033510	362.643100	12.570060
-51.187080	50.825860	352.853600	5.795179
-49.684720	50.599280	349.094000	2.359269
-48.201800	50.354330	347.776900	.853207
-46.739240	50.091630	347.357400	.274260
-45.297720	49.811770	347.236800	.078265
-43.877700	49.515370	347.205300	.019851
-42.479410	49.203060	347.197800	.004463
-41.102810	48.875440	347.196400	.000891
-39.747590	48.533100	347.196100	.000144
-38.413150	48.176570	347.196200	.000073
-96.261670	49.289190	503.577500	64.680730
-94.893750	49.647600	575.721700	80.769700
-93.503820	49.991080	626.548100	97.172230
-92.091320	50.319060	632.262500	98.734130
-90.655950	50.630910	625.568800	96.041200
-89.197760	50.925990	638.924400	104.291200
-87.717060	51.203660	675.479100	123.763900
-86.214420	51.463270	724.121900	143.689100
-84.690660	51.704190	800.551500	163.324200
-83.146820	51.925780	885.678900	174.519600
-81.584160	52.127480	914.900200	188.048400
-80.004110	52.308710	869.598200	186.398600
-78.408280	52.468990	693.271700	179.759900
-76.798410	52.607840	617.673000	153.447700
-75.176390	52.724850	664.941500	120.619800
-73.544200	52.819690	715.815100	110.139800
-71.903920	52.892050	680.029000	105.924700
-70.257710	52.941730	657.437500	108.205200
-68.607750	52.968570	452.634200	89.590510
-66.956280	52.972490	286.474500	85.496090
-65.305560	52.953480	502.761700	90.384000
-63.657820	52.911580	697.625200	128.536500
-62.015270	52.846950	605.334000	92.151890
-60.380080	52.759750	564.279200	94.854060
-58.754360	52.650280	474.425400	53.183830

-57.140120	52.518850	420.155700	38.655700
-55.539290	52.365840	391.574700	29.588370
-53.953660	52.191710	368.421600	17.493610
-52.384890	51.996940	355.551500	8.515426
-50.834480	51.782070	350.252300	3.741373
-49.303730	51.547700	348.218300	1.485389
-47.793770	51.294400	347.505700	.524572
-46.305490	51.022820	347.281200	.163598
-44.839570	50.733620	347.217000	.044905
-43.396390	50.427420	347.200600	.010843
-41.976090	50.104880	347.196800	.002307
-40.578500	49.766630	347.196100	.000421
-39.203150	49.413260	347.196000	.000067
-37.849210	49.045330	347.196200	.000083
-96.841810	50.160530	505.507800	63.899020
-95.453030	50.530490	556.228300	77.624690
-94.041510	50.885130	563.607800	80.040870
-92.606450	51.223850	536.034800	74.606320
-91.147380	51.546020	504.410200	70.057140
-89.664200	51.850960	489.348400	71.855160
-88.157130	52.138020	501.108000	82.157420
-86.626690	52.406500	539.881800	97.051960
-85.073680	52.655720	630.989900	112.797100
-83.499180	52.885050	718.359600	132.522700
-81.904440	53.093850	773.504300	146.953500
-80.290990	53.281520	720.076700	159.321800
-78.660530	53.447530	592.617400	159.906400
-77.014920	53.591380	519.637600	135.476300
-75.356160	53.712650	566.490200	106.640600
-73.686420	53.810940	623.931000	93.314350
-72.007930	53.885960	595.180400	89.054940
-70.322990	53.937460	544.033600	81.850110
-68.634010	53.965280	263.997300	134.621200
-66.943400	53.969350	120.299200	123.154400
-65.253590	53.949630	475.482200	89.822810
-63.567000	53.906200	644.023900	114.468300
-61.886040	53.839190	594.765400	89.152400
-60.213040	53.748820	479.293100	54.085860
-58.550290	53.635360	410.668000	33.046620
-56.899960	53.499180	379.680700	20.225100
-55.264110	53.340690	367.704300	16.434730
-53.644650	53.160350	358.759000	11.768100
-52.043340	52.958710	351.553700	5.288426
-50.461760	52.736320	348.642300	2.088676
-48.901280	52.493820	347.648000	.776301
-47.363030	52.231850	347.325000	.259967
-45.847900	51.951060	347.228800	.075253
-44.356530	51.652150	347.203200	.018652
-42.889220	51.335790	347.197300	.003866
-41.445980	51.002650	347.196200	.000630
-40.026490	50.653380	347.196000	.000118
-38.630060	50.288590	347.196000	.000082
-37.255620	49.908830	347.196200	.000109
-97.453060	51.026530	435.887300	44.907280
-96.042340	51.408490	459.019400	48.926690
-94.608220	51.774700	448.833000	48.434480
-93.149600	52.124570	428.490100	44.727150
-91.665800	52.457450	415.015900	41.401120
-90.156560	52.772640	409.596900	41.861450
-88.621980	53.069450	415.666700	47.365030
-87.062480	53.347160	435.604100	57.423500
-85.478850	53.605050	477.505200	71.451690
-83.872130	53.842430	541.788600	87.632550
-82.243660	54.058630	574.571700	110.755500
-80.595020	54.253030	615.391200	132.998900
-78.928000	54.425040	609.483700	143.750600
-77.244600	54.574130	564.070600	121.907000

-75.546970	54.699840	587.011200	101.866100
-73.837420	54.801750	649.869800	96.615810
-72.118360	54.879540	640.423200	94.608960
-70.392330	54.932960	568.592300	81.957850
-68.661900	54.961820	314.282200	108.817900
-66.929700	54.966030	225.987600	125.178800
-65.198370	54.945580	466.937600	71.475900
-63.470540	54.900540	525.042300	72.574760
-61.748820	54.831050	464.130700	49.639180
-60.035740	54.737340	406.150800	30.602380
-58.333780	54.619720	373.234100	15.259340
-56.645260	54.478570	355.671400	9.582137
-54.972420	54.314330	351.128700	5.036966
-53.317280	54.127510	351.822300	6.059529
-51.681740	53.918690	349.191900	2.608189
-50.067420	53.688470	347.600100	.822006
-48.475770	53.437500	347.266200	.199508
-46.907930	53.166490	347.196400	.045981
-45.364770	52.876130	347.189000	.013056
-43.846840	52.567130	347.191900	.006796
-42.354370	52.240220	347.194400	.003066
-40.887210	51.896080	347.195400	.001112
-39.444820	51.535370	347.195900	.000362
-38.026250	51.158730	347.196000	.000140
-36.630120	50.766700	347.196200	.000124
-98.097820	51.886770	387.271100	25.890910
-96.663910	52.281200	389.832100	26.309040
-95.206040	52.659450	386.622900	25.301910
-93.722790	53.020900	379.990600	23.070850
-92.213200	53.364910	374.515100	21.427890
-90.676770	53.690770	372.149500	21.644220
-89.113440	53.997730	374.625800	24.404220
-87.523560	54.285050	383.164900	29.782430
-85.907830	54.551970	398.309200	38.514960
-84.267300	54.797760	418.517300	53.580620
-82.603340	55.021710	438.872800	79.848870
-80.917590	55.223140	514.212800	109.289600
-79.211960	55.401440	595.221300	132.150000
-77.488560	55.556020	630.818600	121.986300
-75.749730	55.686380	617.126200	106.856300
-73.997920	55.792100	664.897900	103.612000
-72.235800	55.872810	684.378400	103.086300
-70.466090	55.928230	619.597500	94.213420
-68.691570	55.958180	513.619100	64.382580
-66.915130	55.962550	477.312900	66.192620
-65.139640	55.941330	498.383200	67.935730
-63.367970	55.894590	432.219100	51.609300
-61.602940	55.822490	375.327300	34.826560
-59.847310	55.725280	354.383000	16.986500
-58.103760	55.603290	345.188900	9.407542
-56.374820	55.456930	340.782000	8.438745
-54.662870	55.286670	341.191700	7.631924
-52.970100	55.093080	345.012100	3.168665
-51.298500	54.876750	345.895600	1.898755
-49.649800	54.638340	346.477700	1.061855
-48.025460	54.378550	346.837600	.560141
-46.426660	54.098120	347.038700	.265437
-44.854210	53.797780	347.134900	.112944
-43.308580	53.478290	347.174700	.042588
-41.789850	53.140400	347.189400	.014159
-40.297680	52.784830	347.194200	.004189
-38.831280	52.412250	347.195500	.001128
-37.389370	52.023280	347.195900	.000314
-35.970190	51.618480	347.196100	.000153
-98.778840	52.740810	363.708900	13.196650
-97.320270	53.148240	363.026300	12.759440
-95.837370	53.539010	361.653400	11.951890

-94.328290	53.912530	359.264500	10.849310
-92.791720	54.268120	357.115100	9.945726
-91.226900	54.605080	356.084000	9.742385
-89.633560	54.922620	356.571700	10.684820
-88.011890	55.219970	358.393600	13.151750
-86.362530	55.496330	360.242300	18.637640
-84.686490	55.750910	359.745700	32.271970
-82.985150	55.982970	356.779400	59.260540
-81.260250	56.191770	385.664200	88.291860
-79.513790	56.376650	532.630800	116.393100
-77.748020	56.536990	640.938000	121.711500
-75.965460	56.672260	662.593400	112.703000
-74.168790	56.781970	655.367700	106.797100
-72.360860	56.865740	673.224800	105.290200
-70.544630	56.923280	679.761000	106.443600
-68.723180	56.954370	654.834000	92.587390
-66.899620	56.958910	618.572600	93.151160
-65.077100	56.936880	496.991600	62.887650
-63.258730	56.888360	387.197200	41.544500
-61.447630	56.813510	327.329300	32.690540
-59.646790	56.712620	304.958900	30.716500
-57.859090	56.586040	308.614000	27.801570
-56.087300	56.434210	320.002500	22.998970
-54.333970	56.257640	330.576900	16.279880
-52.601470	56.056940	337.846800	10.079930
-50.891880	55.832740	342.268600	5.898674
-49.207030	55.585770	344.769000	3.220241
-47.548420	55.316760	346.093200	1.618834
-45.917200	55.026500	346.736000	.740091
-44.314130	54.715760	347.022200	.305131
-42.739580	54.385350	347.137000	.113150
-41.193420	54.036030	347.177800	.037555
-39.675070	53.668560	347.190900	.011089
-38.183400	53.283620	347.194700	.002956
-36.716770	52.881820	347.195700	.000744
-35.272900	52.463700	347.196000	.000268
-99.499240	53.588130	353.294800	6.142501
-98.014280	54.009130	352.699600	5.648223
-96.504860	54.412960	352.067300	5.149312
-94.968610	54.799050	351.209300	4.567060
-93.403790	55.166730	350.411100	3.995380
-91.809290	55.515250	349.818400	3.608340
-90.184560	55.843850	349.149800	3.688249
-88.529620	56.151670	347.148800	5.474288
-86.844990	56.437890	340.795300	11.686310
-85.131650	56.701690	325.708400	26.263940
-83.390960	56.942230	297.460800	52.435940
-81.624720	57.158780	284.003000	71.477330
-79.835050	57.350580	419.966400	92.406300
-78.024370	57.516980	611.190400	113.679300
-76.195360	57.657400	669.580100	113.605500
-74.350940	57.771310	637.387100	109.365100
-72.494220	57.858320	679.467100	103.113300
-70.628410	57.918090	740.763100	112.390500
-68.756890	57.950390	752.667100	121.154000
-66.883060	57.955100	640.998400	103.680600
-65.010370	57.932220	486.838300	64.387470
-63.142240	57.881810	388.321900	30.356610
-61.282040	57.804080	322.735200	25.577840
-59.433060	57.699310	281.168700	42.662890
-57.598450	57.567890	265.777000	52.349310
-55.781200	57.410300	278.013900	46.566510
-53.984060	57.227100	308.701500	31.484680
-52.209560	57.018940	325.749800	19.827110
-50.459930	56.786500	335.679300	11.753430
-48.737060	56.530560	341.408600	6.497703
-47.042490	56.251900	344.570900	3.311042

-45.377330 55.951360 346.119400 1.537485
-43.742270 55.629770 346.790700 .642444
-42.137470 55.287960 347.057600 .241139
-40.562610 54.926740 347.153400 .081352
-39.016750 54.546860 347.184100 .024618
-37.498410 54.149020 347.193000 .006662
-36.005400 53.733830 347.195300 .001671
-34.534880 53.301770 347.195900 .000563
-100.262700 54.428120 349.257000 2.554030
-98.749250 54.863350 348.961700 2.249017
-97.211560 55.280850 348.705600 1.977425
-95.646590 55.680060 348.402500 1.670881
-94.052090 56.060360 348.056100 1.315506
-92.426510 56.420970 347.517300 1.055916
-90.768940 56.761100 346.105300 2.180362
-89.079190 57.079890 341.737500 6.203228
-87.357570 57.376450 329.738400 14.691950
-85.605000 57.649890 302.718500 30.851290
-83.822840 57.899360 248.545100 58.635590
-82.012920 58.124030 196.104400 68.458550
-80.177480 58.323120 332.238100 68.683800
-78.319110 58.495910 529.757700 92.831060
-76.440710 58.641750 640.903400 110.049400
-74.545430 58.760110 649.532900 107.758600
-72.636660 58.850530 720.125500 106.587900
-70.717930 58.912650 815.402500 121.517400
-68.792920 58.946230 798.914400 134.387500
-66.865380 58.951130 637.155200 111.614800
-64.939080 58.927340 482.622200 67.986850
-63.017780 58.874950 394.325100 31.048250
-61.105210 58.794160 343.339100 12.476420
-59.204930 58.685300 299.043600 31.910100
-57.320400 58.548780 260.909100 55.780440
-55.454840 58.385130 246.835200 68.113010
-53.611270 58.194950 270.372100 50.907390
-51.792370 57.978940 301.498800 33.633440
-50.000500 57.737840 323.075100 20.278080
-48.237630 57.472490 335.346700 11.213270
-46.505310 57.183730 341.730400 5.709534
-44.804610 56.872430 344.973700 2.663009
-43.136050 56.539500 346.384000 1.129061
-41.499610 56.185780 346.921600 .429610
-39.894630 55.812120 347.111200 .146928
-38.319800 55.419290 347.172400 .045231
-36.773090 55.007980 347.190000 .012548
-35.251730 54.578750 347.194600 .003187
-33.752120 54.132060 347.195700 .001047
-101.073700 55.260130 347.839800 .949235
-99.529010 55.710320 347.719500 .800391
-97.960900 56.142140 347.622700 .669125
-96.365430 56.555100 347.501400 .509738
-94.739640 56.948590 347.281900 .359359
-93.081440 57.321860 346.685400 .989967
-91.389490 57.674070 344.763500 3.199737
-89.663250 58.004340 338.789600 8.463512
-87.902820 58.311730 322.743300 18.858600
-86.109000 58.595320 286.693600 38.026550
-84.283100 58.854170 213.391000 66.348000
-82.427000 59.087400 119.410100 83.420270
-80.543050 59.294160 283.512800 53.420560
-78.634010 59.473690 458.314500 71.188510
-76.703000 59.625280 605.514400 96.844880
-74.753460 59.748340 683.893200 105.378600
-72.789070 59.842370 775.242900 114.204700
-70.813740 59.906970 852.890400 129.065400
-68.831480 59.941910 821.257200 143.292400
-66.846440 59.947010 641.342800 114.526000

-64.862770 59.922250 484.657100 71.604780
-62.884610 59.867760 400.655400 36.272170
-60.916040 59.783740 358.714400 11.362240
-58.961010 59.670550 331.092700 13.468850
-57.023270 59.528640 302.045500 32.319920
-55.106350 59.358580 269.616900 50.474720
-53.213500 59.161040 258.411400 58.510480
-51.347600 58.936760 271.170200 47.445180
-49.511150 58.686560 298.572400 30.743080
-47.706190 58.411320 324.395500 17.241870
-45.934240 58.111950 337.633200 8.469531
-44.196280 57.789400 343.325300 3.860525
-42.492650 57.444580 345.808200 1.622182
-40.823030 57.078400 346.745000 .623722
-39.186360 56.691730 347.059000 .215854
-37.580830 56.285340 347.157500 .067375
-36.003750 55.859910 347.186100 .018987
-34.451600 55.415950 347.193700 .004928
-32.919830 54.953830 347.195400 .001606
-101.937200 56.083360 347.381100 .317622
-100.358100 56.549350 347.338000 .253953
-98.756940 56.996240 347.300200 .193100
-97.128790 57.423640 347.232700 .126188
-95.469860 57.830970 347.037500 .361854
-93.777320 58.217490 346.369500 1.310175
-92.049300 58.582370 344.123300 3.822450
-90.284800 58.924680 337.172100 9.705610
-88.483640 59.243460 318.722400 21.028730
-86.646400 59.537720 277.737400 42.231140
-84.774360 59.806450 197.059900 69.443390
-82.869400 60.048700 95.080280 84.556880
-80.933970 60.263580 277.124300 46.519710
-78.971000 60.450230 421.086700 47.329210
-76.983890 60.607900 562.604300 68.236890
-74.976370 60.735940 664.457600 95.270200
-72.952470 60.833800 755.763600 113.410700
-70.916480 60.901050 849.796300 136.179900
-68.872850 60.937420 844.057700 145.215100
-66.826130 60.942730 652.674200 115.026700
-64.780930 60.916960 490.619000 74.279730
-62.741830 60.860230 409.741300 41.646390
-60.713310 60.772790 372.477700 19.391560
-58.699720 60.654990 353.401300 4.202338
-56.705200 60.507380 338.831800 9.141281
-54.733590 60.330550 321.985300 20.716050
-52.788400 60.125220 299.463600 31.026860
-50.872730 59.892220 284.901900 37.574170
-48.989200 59.632420 287.725200 35.068070
-47.139900 59.346780 307.633500 22.263840
-45.326320 59.036270 332.131100 10.576730
-43.549270 58.701890 342.007300 4.485590
-41.808860 58.344600 345.368300 1.835070
-40.104370 57.965370 346.610400 .699944
-38.434220 57.565060 347.023300 .245756
-36.795950 57.144450 347.148200 .077913
-35.186060 56.704150 347.183600 .022361
-33.600030 56.244660 347.193100 .005911
-32.032190 55.766200 347.195200 .001948
-102.859900 56.896880 347.244700 .095511
-101.242000 57.379640 347.230000 .070266
-99.604390 57.842470 347.211300 .044070
-97.940930 58.285070 347.157800 .104676
-96.246660 58.706940 346.962400 .433641
-94.517820 59.107400 346.254900 1.397029
-92.751850 59.485580 343.869900 3.927117
-90.947170 59.840560 336.611500 9.713864
-89.103220 60.171320 317.625200 20.613560

-87.220290	60.476800	274.746900	40.551340
-85.299550	60.755960	200.132900	64.162570
-83.342830	61.007760	133.083400	79.172420
-81.352710	61.231220	294.329600	48.648270
-79.332320	61.425420	411.128200	34.374880
-77.285290	61.589540	503.134400	45.576480
-75.215710	61.722870	563.415100	65.714720
-73.128000	61.824790	648.369800	101.820100
-71.026890	61.894870	791.254600	127.054400
-68.917300	61.932760	871.785900	146.103100
-66.804310	61.938290	671.485400	112.718900
-64.692990	61.911450	502.131500	74.539380
-62.588420	61.852340	424.333500	45.100120
-60.495590	61.761240	391.020000	27.500760
-58.419300	61.638580	376.893300	17.329700
-56.364090	61.484900	368.488400	11.530200
-54.334160	61.300890	360.918900	8.537867
-52.333320	61.087320	352.479400	8.709384
-50.364880	60.845090	338.205700	10.954550
-48.431600	60.575160	322.760600	14.911560
-46.535590	60.278550	320.278900	16.163550
-44.678250	59.956310	331.356400	8.715890
-42.860180	59.609490	342.149700	3.607544
-41.081100	59.239120	345.649000	1.435181
-39.339830	58.846180	346.695700	.557145
-37.634110	58.431540	347.045000	.199473
-35.960610	57.995940	347.153700	.065238
-34.314830	57.539980	347.185000	.019250
-32.690990	57.064010	347.193400	.005240
-31.082000	56.568130	347.195300	.001778
-103.849600	57.699570	347.207600	.025403
-102.187300	58.200280	347.201900	.015763
-100.509000	58.680030	347.189000	.024401
-98.806840	59.138710	347.140100	.117703
-97.074520	59.575940	346.950000	.423822
-95.307120	59.991050	346.259200	1.306906
-93.501110	60.383240	343.973900	3.561116
-91.654170	60.751550	337.155000	8.555197
-89.765210	61.094930	319.762400	17.314720
-87.834170	61.412270	279.009700	31.243360
-85.861950	61.702450	229.145500	53.807760
-83.850420	61.964360	208.891200	68.318330
-81.802150	62.196920	341.437900	45.441880
-79.720490	62.399150	429.338800	38.410720
-77.609360	62.570130	465.136400	32.498570
-75.473210	62.709080	471.960100	53.746740
-73.316960	62.815350	532.845900	80.618860
-71.145790	62.888430	699.873400	107.729800
-68.965200	62.927950	824.169100	133.149200
-66.780790	62.933720	675.164400	102.753900
-64.598260	62.905720	510.729100	68.419990
-62.423240	62.844070	440.576600	45.481400
-60.261290	62.749080	413.037100	33.995680
-58.117710	62.621230	402.401700	28.832870
-55.997520	62.461110	397.097700	25.868960
-53.905320	62.269450	392.466100	23.405410
-51.845240	62.047150	390.858000	22.579430
-49.820820	61.795150	388.520000	21.758590
-47.834930	61.514500	377.957900	16.906840
-45.889670	61.206310	358.986700	7.192004
-43.986290	60.871700	350.095600	3.256136
-42.125070	60.511780	346.749100	1.038800
-40.305270	60.127630	346.756000	.515627
-38.525020	59.720250	347.012600	.243021
-36.781200	59.290500	347.129900	.098312
-35.069400	58.839090	347.174800	.034791
-33.383770	58.366500	347.189800	.010981

-31.716990 57.872960 347.194400 .003158
-30.060130 57.358350 347.195600 .001148
-104.916500 58.490070 347.198200 .005644
-103.202300 59.010120 347.195400 .004873
-101.477600 59.507980 347.184900 .026082
-99.732440 59.983750 347.141700 .106965
-97.958720 60.437220 346.973400 .365159
-96.150010 60.867820 346.374100 1.090991
-94.301540 61.274790 344.429200 2.882849
-92.410060 61.657170 338.886900 6.601809
-90.473700 62.013880 325.183300 12.887060
-88.491920 62.343760 295.122300 21.895310
-86.465330 62.645610 250.036600 37.072640
-84.395630 62.918240 275.455400 44.052610
-82.285480 63.160470 383.563900 41.451190
-80.138370 63.371240 423.192800 34.451850
-77.958560 63.549540 426.547500 27.736380
-75.750930 63.694510 422.694600 38.509560
-73.520860 63.805420 454.122900 56.119580
-71.274150 63.881700 559.166300 77.342600
-69.016910 63.922960 672.217800 95.727710
-66.755400 63.928990 580.068400 77.512350
-64.495980 63.899750 478.271500 54.260420
-62.244980 63.835380 430.105200 39.616880
-60.008560 63.736250 407.925200 31.779210
-57.792640 63.602840 397.618900 27.258660
-55.602750 63.435840 391.213200 24.077050
-53.443960 63.236060 384.106300 20.576310
-51.320750 63.004450 379.350300 17.921420
-49.236890 62.742080 376.874200 16.081000
-47.195330 62.450080 371.033100 12.717340
-45.198120 62.129630 360.895100 7.302503
-43.246250 61.781950 351.420500 2.813173
-41.339580 61.408210 348.194900 .758025
-39.476710 61.009530 347.427400 .192207
-37.654880 60.586900 347.227700 .033638
-35.869840 60.141170 347.193100 .013210
-34.115770 59.672970 347.192400 .007168
-32.385130 59.182660 347.194500 .003054
-30.668580 58.670220 347.195500 .001074
-28.954870 58.135290 347.195900 .000480
-106.073800 59.266680 347.196200 .001265
-104.297500 59.807790 347.194200 .004830
-102.518800 60.325180 347.186000 .022654
-100.725000 60.819250 347.151100 .086633
-98.905530 61.289990 347.018900 .285436
-97.052100 61.737010 346.562000 .826629
-95.158340 62.159620 345.142900 2.098707
-93.219700 62.556880 341.282800 4.581155
-91.233350 62.927700 332.013900 8.593893
-89.198040 63.270870 313.212300 13.575470
-87.113920 63.585110 288.386700 24.362350
-84.982480 63.869130 333.129200 18.397790
-82.806390 64.121680 392.804100 30.166230
-80.589290 64.341560 404.516600 20.319420
-78.335770 64.527670 389.729100 19.222630
-76.051160 64.679080 387.865600 25.631510
-73.741460 64.794950 403.510600 34.993450
-71.413090 64.874680 442.408400 47.489130
-69.072890 64.917820 494.092100 55.054780
-66.727910 64.924110 457.758700 49.008420
-64.385280 64.893550 417.204700 37.148910
-62.052090 64.826280 393.856000 27.898730
-59.735260 64.722690 380.711200 21.909940
-57.441380 64.583340 374.046400 18.028440
-55.176610 64.408980 370.207300 15.310730
-52.946510 64.200520 365.832200 13.003380

-50.755950	63.959000	362.403000	11.080630
-48.608900	63.685590	360.500400	9.306324
-46.508400	63.381530	357.433900	7.130303
-44.456330	63.048090	353.575300	4.506798
-42.453320	62.686580	349.920700	2.193840
-40.498620	62.298210	348.211100	.919525
-38.589950	61.884150	347.510200	.335087
-36.723370	61.445380	347.279300	.108862
-34.893150	60.982680	347.215800	.029718
-33.091610	60.496560	347.200100	.006803
-31.309040	59.987160	347.196700	.001165
-29.533510	59.454220	347.196200	.000134
-27.750760	58.896990	347.196100	.000105
-107.339200	60.027230	347.195700	.001196
-105.487000	60.591610	347.194300	.004530
-103.643800	61.130270	347.187600	.019039
-101.793500	61.644070	347.160700	.069151
-99.922550	62.133320	347.064100	.218433
-98.020040	62.597820	346.750500	.604698
-96.077510	63.037030	345.841500	1.460388
-94.088710	63.450070	343.572900	3.004964
-92.049460	63.835860	338.922900	5.110935
-89.957580	64.193130	331.312400	7.232718
-87.812570	64.520550	328.730600	8.325354
-85.615560	64.816710	359.825800	12.093960
-83.369120	65.080260	381.736200	14.415650
-81.077050	65.309890	376.357100	12.203030
-78.744270	65.504390	369.636300	12.994560
-76.376630	65.662700	367.924200	16.416650
-73.980770	65.783920	374.145300	21.354510
-71.563890	65.867360	388.022000	27.038220
-69.133670	65.912500	401.293000	31.279010
-66.698060	65.919100	393.330800	28.879480
-64.265090	65.887100	379.575500	23.513010
-61.842790	65.816700	369.181800	18.149680
-59.438900	65.708320	362.912200	13.943810
-57.060850	65.562580	359.476400	11.101920
-54.715470	65.380330	357.390900	9.195615
-52.408880	65.162570	355.550100	7.715965
-50.146370	64.910470	354.077000	6.474242
-47.932120	64.625290	352.996800	5.327376
-45.769150	64.308400	351.639600	4.058264
-43.659080	63.961160	350.070900	2.694621
-41.602020	63.584970	348.664600	1.511248
-39.596350	63.181100	347.812800	.727035
-37.638600	62.750720	347.417200	.305898
-35.723270	62.294770	347.267000	.111488
-33.842640	61.813940	347.216300	.035575
-31.986660	61.308540	347.201100	.009892
-30.142740	60.778430	347.197200	.002370
-28.295590	60.222970	347.196400	.000483
-26.427080	59.640870	347.196200	.000113
-108.736700	60.768890	347.195600	.001593
-106.789100	61.359350	347.194100	.005076
-104.866900	61.921510	347.187300	.019516
-102.949500	62.456830	347.162100	.066397
-101.019200	62.966030	347.077700	.197425
-99.061880	63.449270	346.827900	.514713
-97.066180	63.906190	346.175400	1.165795
-95.023590	64.336020	344.740300	2.226461
-92.928180	64.737730	342.417400	3.467926
-90.776390	65.110020	340.146200	4.248354
-88.566850	65.451470	342.721000	3.904514
-86.300130	65.760610	359.873600	6.465246
-83.978550	66.035940	366.944900	8.693324
-81.606060	66.276030	365.124900	8.911400
-79.187900	66.479550	361.897900	10.134920

-76.730480	66.645290	360.225700	12.266890
-74.241170	66.772270	362.891300	15.331720
-71.728080	66.859700	369.130400	18.665900
-69.199870	66.907030	373.904000	20.883130
-66.665550	66.913940	371.541900	19.967210
-64.134220	66.880400	365.455500	16.939390
-61.614990	66.806620	360.036300	13.278910
-59.116610	66.693070	356.446200	10.177080
-56.647430	66.540450	354.295500	7.988193
-54.215080	66.349720	352.930500	6.500563
-51.826330	66.121990	351.880200	5.402575
-49.486860	65.858530	351.039900	4.502242
-47.201050	65.560780	350.345700	3.668353
-44.971820	65.230190	349.610700	2.799216
-42.800360	64.868270	348.803600	1.917235
-40.685980	64.476460	348.085900	1.142918
-38.625890	64.056100	347.610200	.591622
-36.614990	63.608330	347.363700	.265567
-34.645670	63.134020	347.255600	.104236
-32.707610	62.633670	347.214600	.035909
-30.787590	62.107330	347.201300	.010843
-28.869240	61.554470	347.197400	.002846
-26.932890	60.973880	347.196400	.000655
-24.955330	60.363580	347.196200	.000177

Q.17a

-89.453400	32.991660	366.473400	1.047745
-88.345930	33.191160	366.741200	1.377598
-87.228100	33.381660	367.513400	2.258243
-86.100230	33.562890	368.796700	3.663107
-84.962690	33.734530	370.439500	5.407437
-83.815900	33.896310	372.103900	7.158027
-82.660300	34.047950	373.452700	8.588845
-81.496410	34.189190	374.282400	9.480229
-80.324730	34.319790	374.478800	9.669163
-79.145840	34.439500	373.877200	8.991892
-77.960310	34.548100	372.245000	7.381350
-76.768760	34.645400	369.646200	5.166901
-75.571840	34.731200	367.037000	3.195274
-74.370180	34.805370	365.733200	2.152763
-73.164480	34.867740	365.700300	1.814422
-71.955430	34.918200	366.452300	1.882027
-70.743730	34.956650	367.192100	2.126491
-69.530080	34.983020	367.587000	2.274890
-68.315220	34.997260	367.547900	2.151155

-67.099880	34.999340	367.204600	1.775443
-65.884770	34.989250	366.737600	1.267197
-64.670640	34.967020	366.323800	.789235
-63.458190	34.932690	366.043200	.436507
-62.248150	34.886320	365.885700	.219190
-61.041230	34.828000	365.808500	.102444
-59.838120	34.757850	365.773700	.045241
-58.639500	34.676010	365.758700	.018927
-57.446040	34.582610	365.752500	.007435
-56.258390	34.477850	365.750200	.002709
-55.077160	34.361910	365.749300	.000926
-53.902950	34.235010	365.749000	.000302
-52.736340	34.097380	365.748900	.000088
-51.577860	33.949260	365.748900	.000029
-50.428010	33.790910	365.748900	.000013
-49.287250	33.622600	365.748900	.000000
-48.156040	33.444620	365.748900	.000000
-47.034740	33.257250	365.748900	.000000
-45.923690	33.060790	365.748900	.000000
-44.823220	32.855560	365.748900	.000000
-89.701210	33.918400	366.917100	1.395325
-88.583520	34.125020	367.502800	1.996669
-87.455040	34.322360	369.104900	3.515074
-86.316120	34.510110	371.855300	6.012280
-85.167100	34.687960	375.463900	9.193531
-84.008420	34.855620	379.092600	12.360290
-82.840550	35.012790	381.969600	14.904130
-81.663990	35.159200	383.724600	16.485650
-80.479300	35.294580	384.232200	16.909400
-79.287040	35.418700	383.232900	15.888850
-78.087840	35.531310	380.013000	13.192070
-76.882320	35.632210	374.354700	9.268100
-75.671150	35.721200	368.598600	5.837091
-74.455030	35.798110	366.004300	4.100585
-73.234660	35.862810	366.582600	3.855311
-72.010770	35.915150	368.012200	4.062829
-70.784100	35.955040	369.561100	4.484396
-69.555380	35.982390	370.202600	4.609874
-68.325390	35.997170	369.954300	4.246770
-67.094890	35.999320	369.052200	3.419654
-65.864640	35.988860	367.916800	2.383061
-64.635410	35.965800	366.964100	1.450262
-63.407950	35.930180	366.353600	.789813
-62.183030	35.882080	366.027900	.398579
-60.961370	35.821590	365.872300	.191159
-59.743720	35.748840	365.801900	.088662
-58.530780	35.663950	365.771100	.039540
-57.323260	35.567100	365.757800	.016759
-56.121820	35.458470	365.752200	.006682
-54.927120	35.338260	365.750100	.002529
-53.739770	35.206700	365.749300	.000899
-52.560380	35.064020	365.749000	.000303
-51.389480	34.910500	365.748900	.000093
-50.227610	34.746390	365.748900	.000064
-49.075240	34.571990	365.748900	.000000
-47.932830	34.387580	365.748900	.000000
-46.800740	34.193490	365.748900	.000000
-45.679330	33.990000	365.748900	.000061
-44.568900	33.777440	365.748900	.000000
-89.960660	34.843320	368.047500	2.037086
-88.832330	35.057210	369.566800	3.297234
-87.692770	35.261540	373.647500	6.451414
-86.542320	35.455950	381.048700	11.988820
-85.381340	35.640140	391.107500	19.265950
-84.210270	35.813810	401.084400	26.476240
-83.029580	35.976620	408.744400	32.084110
-81.839790	36.128310	413.327900	35.533380

-80.641480	36.268600	414.896500	36.632170
-79.435230	36.397220	412.942700	34.876240
-78.221690	36.513940	405.361700	29.324260
-77.001530	36.618520	389.667900	20.745830
-75.775430	36.710780	373.311600	12.783030
-74.544130	36.790520	368.881400	10.565090
-73.308360	36.857590	370.195200	9.769433
-72.068890	36.911860	375.081300	11.149410
-70.826490	36.953220	377.872500	11.597030
-69.581950	36.981590	379.201400	11.553330
-68.336070	36.996900	378.061800	10.387300
-67.089650	36.999130	375.248900	8.214581
-65.843500	36.988290	371.779700	5.596053
-64.598400	36.964370	369.010100	3.334460
-63.355190	36.927440	367.348200	1.814621
-62.114630	36.877570	366.497800	.936199
-60.877510	36.814860	366.095300	.473793
-59.644590	36.739430	365.906900	.235004
-58.416640	36.651430	365.819000	.113104
-57.194370	36.551040	365.778900	.051774
-55.978490	36.438440	365.761200	.022404
-54.769680	36.313860	365.753500	.009107
-53.568590	36.177530	365.750500	.003492
-52.375820	36.029710	365.749400	.001261
-51.191970	35.870660	365.749000	.000419
-50.017550	35.700660	365.748900	.000126
-48.853080	35.520030	365.748900	.000064
-47.698980	35.329070	365.748900	.000058
-46.555660	35.128100	365.748900	.000058
-45.423470	34.917440	365.748900	.000061
-44.302690	34.697410	365.748900	.000022
-90.232250	35.766320	369.581200	2.372375
-89.092860	35.987640	372.916800	4.503526
-87.941790	36.199090	382.319700	10.624690
-86.779330	36.400320	401.227200	22.486830
-85.605870	36.591000	428.672900	39.166110
-84.421870	36.770790	455.185000	55.309400
-83.227800	36.939400	474.452500	67.429180
-82.024180	37.096500	485.457600	74.601720
-80.811620	37.241800	489.544900	77.367470
-79.590740	37.375040	487.027200	74.709750
-78.362190	37.495960	470.828600	63.119910
-77.126670	37.604320	428.617600	45.591420
-75.884930	37.699920	380.802100	27.828340
-74.637700	37.782550	375.302800	20.783210
-73.385770	37.852060	389.019100	27.118490
-72.129940	37.908310	395.677300	26.622880
-70.871030	37.951180	403.313600	28.487090
-69.609860	37.980580	403.935200	27.268140
-68.347290	37.996460	400.239400	24.199890
-67.084140	37.998780	391.907200	18.842060
-65.821270	37.987530	381.658400	12.436970
-64.559530	37.962740	373.964400	7.228756
-63.299760	37.924460	369.702900	3.935871
-62.042780	37.872770	367.668000	2.120510
-60.789440	37.807780	366.694900	1.143085
-59.540510	37.729610	366.210200	.611258
-58.296810	37.638420	365.965300	.314444
-57.059080	37.534390	365.846300	.153961
-55.828080	37.417740	365.790800	.070748
-54.604500	37.288680	365.765900	.030480
-53.389020	37.147460	365.755200	.012138
-52.182290	36.994370	365.750900	.004498
-50.984880	36.829660	365.749500	.001513
-49.797380	36.653650	365.748900	.000474
-48.620270	36.466650	365.748800	.000163
-47.454020	36.268990	365.748900	.000070

-46.299010	36.060990	365.748900	.000066
-45.155610	35.843010	365.748900	.000078
-44.024070	35.615370	365.748900	.000026
-90.516530	36.687310	369.788400	3.575005
-89.365660	36.916220	375.203600	4.767604
-88.202590	37.134950	393.028400	14.155350
-87.027650	37.343150	437.982800	37.799890
-85.841200	37.540460	510.780300	74.291110
-84.643710	37.726540	577.671400	108.647500
-83.435650	37.901050	621.903400	132.596300
-82.217600	38.063690	644.988800	145.954400
-80.990140	38.214140	653.342700	152.173600
-79.753940	38.352110	654.181500	148.353300
-78.509670	38.477340	622.118100	131.356700
-77.258070	38.589580	527.201900	93.069750
-75.999910	38.688600	381.886600	64.876430
-74.735980	38.774210	382.972000	55.333430
-73.467090	38.846230	433.918400	59.921320
-72.194090	38.904510	464.664200	64.297160
-70.917830	38.948930	467.005900	61.395180
-69.639210	38.979390	468.325100	59.494340
-68.359090	38.995840	455.947000	51.883100
-67.078360	38.998240	433.785000	39.710630
-65.797930	38.986590	404.502100	24.994660
-64.518690	38.960900	384.207300	13.938560
-63.241520	38.921240	374.622100	7.724553
-61.967320	38.867690	370.311900	4.442240
-60.696920	38.800350	368.209400	2.611759
-59.431210	38.719360	367.032700	1.500514
-58.170970	38.624900	366.381700	.823050
-56.917050	38.517150	366.046400	.424672
-55.670200	38.396340	365.882300	.205902
-54.431160	38.262690	365.804800	.091904
-53.200640	38.116470	365.770400	.037755
-51.979310	37.957960	365.756300	.014035
-50.767770	37.787460	365.751100	.004765
-49.566610	37.605300	365.749400	.001465
-48.376340	37.411790	365.748900	.000410
-47.197410	37.207270	365.748800	.000149
-46.030250	36.992090	365.748900	.000078
-44.875180	36.766620	365.748900	.000085
-43.732470	36.531200	365.748900	.000032
-90.814130	37.606180	364.223100	10.107980
-89.651300	37.842850	367.541700	9.917029
-88.475770	38.069030	390.063900	14.285260
-87.287830	38.284350	484.726800	51.755110
-86.087840	38.488450	679.803800	123.839000
-84.876290	38.680970	844.145900	187.742300
-83.653650	38.861550	939.158500	231.926900
-82.420500	39.029860	980.798900	250.833800
-81.177470	39.185590	992.423600	267.123100
-79.925230	39.328410	1001.151000	270.819100
-78.664510	39.458070	971.951700	248.356800
-77.396060	39.574290	806.712600	175.288500
-76.120680	39.676830	493.694900	128.029100
-74.839220	39.765490	329.029300	80.270540
-73.552520	39.840080	532.294700	144.529500
-72.261480	39.900440	611.495000	122.527100
-70.967010	39.946460	628.222600	120.194900
-69.670030	39.978020	614.674100	110.147000
-68.371470	39.995060	586.174400	96.279670
-67.072270	39.997550	529.186600	71.497970
-65.773400	39.985470	447.429200	41.862570
-64.475770	39.958870	402.324900	23.277880
-63.180340	39.917780	383.603400	13.700310
-61.888020	39.862300	376.183900	8.799273
-60.599740	39.792560	371.933300	5.680487

-59.316390 39.708680 369.174300 3.493141
-58.038840 39.610860 367.491000 2.014033
-56.767930 39.499290 366.600100 1.090759
-55.504470 39.374200 366.142300 .546638
-54.249260 39.235840 365.917100 .251506
-53.003000 39.084480 365.814100 .103685
-51.766410 38.920440 365.771500 .038752
-50.540120 38.744010 365.755900 .012932
-49.324710 38.555530 365.750700 .003919
-48.120720 38.355340 365.749300 .001077
-46.928620 38.143810 365.748800 .000264
-45.748810 37.921300 365.748800 .000090
-44.581620 37.688170 365.748700 .000061
-43.427300 37.444810 365.748900 .000039
-91.125630 38.522790 347.945700 21.407890
-89.950410 38.767390 331.417900 25.535140
-88.761920 39.001200 318.322400 29.755030
-87.560450 39.223820 482.365800 39.968810
-86.346370 39.434880 941.303500 137.425400
-85.120160 39.633990 1332.360000 223.343000
-83.882300 39.820790 1555.940000 277.310500
-82.633390 39.994930 1633.850000 319.254300
-81.374080 40.156070 1657.670000 350.557500
-80.105060 40.303890 1696.351000 381.869500
-78.827100 40.438090 1725.704000 350.545300
-77.540980 40.558400 1509.133000 264.171200
-76.247540 40.664560 879.830700 141.774900
-74.947690 40.756360 335.933700 230.762600
-73.642300 40.833600 475.967000 152.135000
-72.332310 40.896110 837.931900 154.993900
-71.018690 40.943760 919.086300 166.083800
-69.702430 40.976440 877.712000 160.816100
-68.384490 40.994090 803.164200 134.986600
-67.065880 40.996670 658.295700 94.387500
-65.747600 40.984170 482.766100 53.342260
-64.430650 40.956610 418.361300 30.320050
-63.116020 40.914060 400.039200 22.788650
-61.804690 40.856610 389.533000 17.021290
-60.497620 40.784390 381.121700 11.939820
-59.195770 40.697540 374.408500 7.698053
-57.900040 40.596260 370.265000 4.601921
-56.611330 40.480750 368.016800 2.591388
-55.330490 40.351270 366.823400 1.334176
-54.058330 40.208070 366.206800 .613698
-52.795620 40.051450 365.924000 .253545
-51.543080 39.881710 365.808500 .092401
-50.301390 39.699200 365.767100 .030492
-49.071130 39.504250 365.753800 .008920
-47.852850 39.297230 365.749900 .002371
-46.647030 39.078520 365.749000 .000562
-45.454070 38.848490 365.748800 .000155
-44.274280 38.607530 365.748700 .000061
-43.107920 38.356030 365.748900 .000039
-91.451740 39.437030 328.618100 28.734450
-90.263630 39.689750 266.340300 48.756280
-89.061680 39.931370 169.669600 116.345900
-87.846150 40.161480 422.973400 155.525000
-86.617400 40.379670 996.066500 159.186500
-85.375900 40.585540 1640.767000 211.138000
-84.122160 40.778730 2137.144000 288.495400
-82.856800 40.958850 2387.474000 333.343600
-81.580460 41.125550 2501.216000 385.980700
-80.293880 41.278500 2641.922000 429.844300
-78.997860 41.417370 2737.684000 446.469700
-77.693230 41.541890 2530.058000 370.270000
-76.380870 41.651780 1626.979000 227.238300
-75.061690 41.746810 550.076700 206.602600

-73.736660 41.826770 402.569600 247.773700
-72.406780 41.891490 852.861000 131.185800
-71.073040 41.940830 1164.334000 171.080500
-69.736500 41.974670 1080.226000 195.690200
-68.398190 41.992950 896.658100 154.444900
-67.059160 41.995620 600.455000 87.285100
-65.720480 41.982670 393.625700 39.056380
-64.383220 41.954140 411.130800 41.736730
-63.048400 41.910080 434.364200 38.358800
-61.717090 41.850600 422.692400 32.917420
-60.390290 41.775820 402.716100 23.906730
-59.069010 41.685920 386.459300 15.898620
-57.754210 41.581080 376.452000 9.817286
-56.446830 41.461530 371.496500 5.671335
-55.147770 41.327530 368.456300 2.948551
-53.857880 41.179360 366.874300 1.339799
-52.577970 41.017320 366.159400 .533310
-51.308780 40.841740 365.883300 .191142
-50.051000 40.652980 365.788100 .060207
-48.805250 40.451390 365.759100 .017306
-47.572090 40.237360 365.751100 .004368
-46.352010 40.011290 365.749200 .000993
-45.145370 39.773560 365.748800 .000208
-43.952510 39.524580 365.748700 .000064
-42.773630 39.264750 365.748900 .000058
-91.793200 40.348750 350.994000 22.957880
-90.591710 40.609810 315.412000 52.132800
-89.375760 40.859430 274.421400 141.424000
-88.145600 41.097210 264.305800 150.045600
-86.901570 41.322720 572.275300 126.361400
-85.644150 41.535550 1109.700000 110.393400
-84.373860 41.735290 1844.388000 215.975900
-83.091290 41.921560 2403.418000 362.594600
-81.797160 42.093980 2790.604000 464.808900
-80.492230 42.252200 3149.764000 534.486100
-79.177290 42.395880 3385.048000 541.333300
-77.853240 42.524730 3206.230000 491.584000
-76.521000 42.638450 2428.368000 302.179600
-75.181550 42.736820 1275.821000 145.312200
-73.835900 42.819590 806.927100 160.398300
-72.485100 42.886590 845.889700 113.216300
-71.130210 42.937670 1016.713000 171.982600
-69.772340 42.972710 1035.017000 197.894800
-68.412590 42.991630 778.010800 181.339300
-67.052090 42.994400 356.725600 163.711000
-65.691960 42.980990 238.561700 127.389200
-64.333320 42.951450 438.034300 58.680690
-62.977290 42.905830 539.172700 67.227870
-61.624970 42.844260 498.917600 62.521020
-60.277440 42.766850 445.369900 45.923090
-58.935750 42.673790 410.215800 30.341520
-57.600940 42.565280 389.815600 19.958640
-56.273990 42.441570 379.470600 11.541070
-54.955840 42.302930 372.180500 5.807593
-53.647390 42.149640 368.184800 2.511136
-52.349470 41.982030 366.571900 .957310
-51.062890 41.800450 365.997200 .324485
-49.788320 41.605260 365.817100 .101552
-48.526440 41.396860 365.765500 .028086
-47.277780 41.175640 365.752400 .007256
-46.042850 40.942000 365.749400 .001704
-44.822010 40.696380 365.748900 .000398
-43.615560 40.439180 365.748700 .000111
-42.423690 40.170830 365.748900 .000078
-92.150770 41.257820 406.387500 26.504300
-90.935390 41.527410 449.006300 51.584060
-89.704910 41.785250 417.447400 113.333200

-88.459540	42.030900	341.672200	149.649100
-87.199620	42.263930	284.249100	138.328000
-85.925610	42.483890	776.246600	146.398400
-84.638040	42.690380	1645.678000	214.713500
-83.337520	42.882970	2349.692000	393.473200
-82.024790	43.061280	2867.593000	534.137300
-80.700610	43.224930	3349.030000	585.146100
-79.365870	43.373580	3552.384000	611.004200
-78.021450	43.506890	3336.981000	528.244200
-76.668370	43.624570	2600.444000	376.023200
-75.307630	43.726360	1692.734000	212.864300
-73.940300	43.812040	972.190000	173.880300
-72.567500	43.881390	651.950800	163.874500
-71.190370	43.934270	689.611600	168.571300
-69.810050	43.970550	1042.900000	224.241900
-68.427750	43.990140	1152.583000	187.350600
-67.044650	43.993000	748.237100	142.547300
-65.661930	43.979120	579.172700	105.482900
-64.280810	43.948540	712.219900	108.079800
-62.902460	43.901310	740.577800	107.014900
-61.528050	43.837570	630.861900	102.537600
-60.158730	43.757450	516.406000	81.081190
-58.795620	43.661140	451.858000	56.147890
-57.439790	43.548850	419.963300	35.382420
-56.092310	43.420850	396.969400	21.208900
-54.754150	43.277410	378.922700	9.691508
-53.426270	43.118850	370.215700	3.815605
-52.109530	42.945500	367.035000	1.369173
-50.804760	42.757750	366.100300	.465270
-49.512690	42.555960	365.836100	.148001
-48.233980	42.340550	365.768600	.045445
-46.969190	42.111940	365.752700	.012943
-45.718810	41.870550	365.749300	.003558
-44.483210	41.616830	365.748700	.000946
-43.262650	41.351200	365.748700	.000253
-42.057300	41.074100	365.748800	.000120
-92.525320	42.164070	408.804700	33.481750
-91.295520	42.442420	466.417100	57.959370
-90.049940	42.708690	563.541700	97.256140
-88.788760	42.962440	537.089900	110.566700
-87.512310	43.203190	541.975900	105.517300
-86.221000	43.430500	934.620500	143.080100
-84.915410	43.643920	1743.880000	254.715000
-83.596150	43.843030	2464.364000	398.301100
-82.263960	44.027400	2986.703000	512.363200
-80.919660	44.196660	3392.757000	580.802700
-79.564150	44.350410	3460.875000	565.599800
-78.198390	44.488330	2989.801000	472.107200
-76.823410	44.610100	2025.298000	262.693600
-75.440290	44.715440	996.291100	131.296300
-74.050180	44.804110	496.328800	118.163800
-72.654240	44.875890	191.218900	113.606400
-71.253690	44.930630	474.617700	213.984500
-69.849760	44.968180	1314.917000	215.987100
-68.443720	44.988460	1696.315000	278.379800
-67.036810	44.991420	1236.847000	197.821900
-65.630330	44.977050	918.768400	158.596800
-64.225530	44.945390	904.143700	139.459200
-62.823690	44.896510	910.709800	139.215800
-61.426040	44.830540	801.357800	119.353000
-60.033810	44.747610	633.187300	114.842600
-58.648170	44.647940	527.964400	80.799230
-57.270290	44.531750	465.955200	51.896190
-55.901260	44.399320	417.794700	26.299710
-54.542130	44.250930	384.008600	11.077960
-53.193900	44.086940	370.427100	4.247839
-51.857480	43.907680	366.980600	1.709237

-50.533710 43.713560 366.035900 .678577
-49.223370 43.504980 365.818000 .248294
-47.927120 43.282370 365.763400 .084005
-46.645540 43.046150 365.751600 .026207
-45.379100 42.796800 365.749100 .007595
-44.128160 42.534760 365.748700 .002048
-42.892960 42.260480 365.748700 .000529
-41.673610 41.974410 365.748800 .000190
-92.917770 43.067330 390.906400 24.672000
-91.673000 43.354680 425.732100 39.263490
-90.411740 43.629630 487.532300 62.280720
-89.134110 43.891690 411.412100 102.889400
-87.840450 44.140390 186.386400 117.068100
-86.531140 44.375260 339.882600 202.478200
-85.206750 44.595830 1300.762000 224.030900
-83.867900 44.801650 2138.601000 399.649600
-82.515380 44.992280 2658.120000 509.078900
-81.150000 45.167310 2966.879000 526.230200
-79.772730 45.326340 2910.023000 498.395900
-78.384560 45.469020 2402.923000 372.688500
-76.986600 45.595010 1527.888000 186.155400
-75.579970 45.704020 724.854600 178.548900
-74.165890 45.795780 303.371900 128.104800
-72.745610 45.870080 251.247600 123.026500
-71.320400 45.926740 620.609000 172.735300
-69.891600 45.965610 1550.994000 233.607800
-68.460530 45.986610 1785.712000 285.574500
-67.028560 45.989670 1219.745000 219.764900
-65.597030 45.974800 804.296600 228.465000
-64.167300 45.942020 795.555400 164.994600
-62.740720 45.891430 914.077500 137.423400
-61.318600 45.823140 903.238600 137.744000
-59.902260 45.737310 763.050700 118.468100
-58.492950 45.634170 601.870200 94.728440
-57.091900 45.513950 487.777800 53.114280
-55.700260 45.376930 403.706500 26.790200
-54.319140 45.223450 370.840100 15.308550
-52.949590 45.053840 364.696900 8.091179
-51.592580 44.868490 365.363800 3.356360
-50.248970 44.667810 365.790100 1.238623
-48.919560 44.452230 365.781500 .449887
-47.605040 44.222190 365.762600 .151518
-46.305980 43.978160 365.752800 .047154
-45.022840 43.720610 365.749700 .013510
-43.755960 43.450020 365.748900 .003611
-42.505550 43.166850 365.748800 .000911
-41.271660 42.871580 365.748800 .000302
-93.329110 43.967430 368.428200 16.687430
-92.068780 44.264030 375.091600 22.060480
-90.791210 44.547890 370.952100 50.282550
-89.496510 44.818510 317.279500 108.571100
-88.184940 45.075390 127.208600 74.702930
-86.856880 45.318050 324.702600 130.854800
-85.512890 45.545990 1185.821000 205.254700
-84.153590 45.758730 1984.277000 333.729900
-82.779790 45.955820 2346.838000 416.731200
-81.392350 46.136820 2421.446000 463.471900
-79.992260 46.301310 2303.088000 413.979000
-78.580580 46.448910 1955.548000 296.525000
-77.158460 46.579270 1462.108000 177.673000
-75.727110 46.692070 1078.033000 119.895000
-74.287820 46.787040 840.863800 127.486800
-72.841890 46.863950 646.715800 114.434100
-71.390720 46.922590 966.168000 119.799100
-69.935700 46.962830 1598.520000 265.484600
-68.478260 46.984570 1676.288000 236.290800
-67.019850 46.987740 1104.902000 232.059700

-65.561920	46.972340	798.269300	188.554200
-64.105920	46.938410	782.176100	152.548400
-62.653260	46.886040	869.972000	148.107100
-61.205390	46.815350	905.854200	153.525200
-59.763680	46.726520	823.495500	130.120300
-58.329470	46.619790	642.266200	110.790000
-56.904060	46.495390	476.277100	69.324070
-55.488670	46.353640	382.816700	47.087900
-54.084500	46.194880	359.219900	30.161560
-52.692630	46.019470	364.401800	12.792420
-51.314070	45.827840	366.515200	4.780491
-49.949730	45.620390	366.186600	1.831083
-48.600430	45.397590	365.919500	.668143
-47.266860	45.159900	365.803100	.226795
-45.949600	44.907820	365.764900	.070885
-44.649100	44.641840	365.752900	.020285
-43.365670	44.362450	365.749700	.005360
-42.099450	44.070140	365.748900	.001324
-40.850440	43.765410	365.748900	.000422
-93.760410	44.864160	358.105300	18.657950
-92.483920	45.170280	354.568500	26.321230
-91.189420	45.463320	369.215800	30.849320
-89.876960	45.742750	362.347500	52.714120
-88.546760	46.008070	394.036300	76.580470
-87.199170	46.258760	662.862700	85.534350
-85.834720	46.494300	1250.236000	175.063000
-84.454070	46.714200	1766.861000	273.355600
-83.058010	46.917960	2000.267000	347.640100
-81.647480	47.105130	2056.387000	359.004000
-80.223460	47.275260	1912.794000	308.516200
-78.787090	47.427960	1539.776000	250.611400
-77.339580	47.562840	1213.215000	174.140400
-75.882230	47.679570	1087.713000	124.998100
-74.416370	47.777870	1042.808000	119.746700
-72.943440	47.857470	960.691400	101.353100
-71.464880	47.918190	1123.371000	150.152000
-69.982220	47.959850	1437.448000	189.300900
-68.496960	47.982350	1324.556000	206.584500
-67.010670	47.985630	1047.825000	156.950500
-65.524890	47.969690	947.462100	110.881500
-64.041170	47.934570	925.852800	106.117400
-62.561040	47.880340	923.852600	137.020500
-61.086020	47.807170	960.844500	135.814600
-59.617590	47.715230	915.415000	140.581400
-58.157170	47.604770	756.685900	104.215100
-56.706150	47.476050	531.129900	86.455510
-55.265830	47.329400	410.730700	55.043720
-53.837470	47.165180	383.318100	29.375330
-52.422200	46.983780	376.200900	13.510460
-51.021090	46.785640	370.162800	5.840761
-49.635090	46.571200	367.244300	2.345541
-48.265020	46.340950	366.203400	.866038
-46.911610	46.095370	365.878800	.295421
-45.575410	45.834990	365.784100	.092237
-44.256860	45.560320	365.757800	.026467
-42.956210	45.271870	365.750900	.006955
-41.673580	44.970160	365.749200	.001704
-40.408870	44.655690	365.748900	.000513
-94.212860	45.757310	364.769000	21.234100
-92.919560	46.073230	368.412100	27.923470
-91.607470	46.375730	411.238700	33.473830
-90.276550	46.664250	494.596400	47.175740
-88.926960	46.938270	594.731400	55.508620
-87.559010	47.197250	778.430000	85.372730
-86.173240	47.440650	1095.342000	161.927800
-84.770290	47.667940	1390.563000	244.289600
-83.350960	47.878600	1650.419000	287.286300

-81.916200	48.072160	1798.877000	281.810700
-80.467090	48.248140	1657.093000	262.146700
-79.004780	48.406110	1272.543000	216.357200
-77.530590	48.545680	920.207200	202.776200
-76.045850	48.666500	776.581500	156.309300
-74.552020	48.768250	750.028100	120.921800
-73.050600	48.850660	787.384200	102.172100
-71.543170	48.913520	917.442600	118.951400
-70.031330	48.956650	1074.135000	172.431600
-68.516710	48.979950	1028.145000	174.845300
-67.000980	48.983350	939.883000	143.363300
-65.485790	48.966840	951.470100	118.909500
-63.972820	48.930470	971.797200	105.758000
-62.463700	48.874330	946.624600	103.144900
-60.960050	48.798580	979.039700	116.848700
-59.463460	48.703410	1007.418000	111.191300
-57.975440	48.589080	866.532200	103.966200
-56.497490	48.455880	620.552400	78.138540
-55.030970	48.304140	461.306700	53.330200
-53.577210	48.134270	406.263100	28.318280
-52.137430	47.946660	383.284200	14.131750
-50.712720	47.741790	372.318000	6.369452
-49.304080	47.520120	367.862100	2.577774
-47.912350	47.282170	366.374200	.951800
-46.538250	47.028450	365.927500	.325196
-45.182340	46.759510	365.796800	.101837
-43.845020	46.475880	365.760700	.029139
-42.526500	46.178100	365.751400	.007632
-41.226800	45.866700	365.749400	.001860
-39.945740	45.542200	365.749000	.000550
-94.687740	46.646660	386.089300	26.976260
-93.376940	46.972690	398.580700	34.388120
-92.046550	47.284930	455.419800	46.234300
-90.696440	47.582840	523.449200	64.197420
-89.326680	47.865840	598.679300	79.501590
-87.937550	48.133390	749.397500	112.743700
-86.529520	48.384910	971.877300	151.248800
-85.103260	48.619850	1201.812000	193.823400
-83.659580	48.837660	1458.338000	213.845600
-82.199460	49.037830	1620.455000	247.587100
-80.724000	49.219880	1541.541000	234.352400
-79.234450	49.383340	1237.000000	211.329200
-77.732160	49.527770	976.914100	162.096300
-76.218590	49.652830	799.856000	123.590000
-74.695260	49.758160	668.725200	131.732500
-73.163800	49.843490	637.692300	132.736400
-71.625870	49.908580	743.307400	121.084300
-70.083210	49.953250	883.635500	140.075800
-68.537570	49.977380	891.315300	161.581000
-66.990740	49.980900	868.252700	154.485800
-65.444490	49.963800	887.467000	137.526700
-63.900620	49.926140	842.339400	120.771600
-62.360880	49.868010	738.666400	108.296600
-60.827010	49.789570	790.047200	100.092000
-59.300730	49.691040	869.472100	102.629000
-57.783640	49.572690	804.329800	90.613690
-56.277320	49.434830	606.398900	67.956550
-54.783270	49.277820	465.405000	47.061210
-53.302860	49.102080	406.171100	25.069440
-51.837360	48.908040	380.240600	12.673150
-50.387950	48.696200	370.539200	5.634133
-48.955640	48.467040	367.459600	2.248903
-47.541310	48.221130	366.311300	.858017
-46.145660	47.959000	365.913400	.296569
-44.769230	47.681210	365.794600	.093605
-43.412380	47.388330	365.760200	.026841
-42.075270	47.080920	365.751300	.007007

-40.757810	46.759540	365.749300	.001687
-39.459720	46.424710	365.748900	.000490
-95.186440	47.531940	426.240700	42.547390
-93.857410	47.868410	454.437700	51.518900
-92.508000	48.190720	521.390700	70.607830
-91.137910	48.498310	586.762200	89.423710
-89.747150	48.790600	656.655900	104.755200
-88.335950	49.067010	812.226600	116.487600
-86.904710	49.326930	995.854100	148.909000
-85.454100	49.569790	1128.249000	163.145700
-83.984930	49.795020	1239.698000	205.854800
-82.498220	50.002060	1337.996000	228.039700
-80.995120	50.190400	1304.592000	246.356800
-79.476920	50.359550	1172.937000	220.392100
-77.945060	50.509060	1052.292000	177.816300
-76.401090	50.638520	924.090400	146.442100
-74.846650	50.747580	822.480700	119.809000
-73.283450	50.835950	769.170700	118.426100
-71.713320	50.903350	795.421500	128.002400
-70.138080	50.949630	852.633500	143.380300
-68.559640	50.974620	865.260800	150.862700
-66.979900	50.978270	847.696700	156.584900
-65.400800	50.960560	826.440700	154.223200
-63.824260	50.921550	703.577800	121.264900
-62.252170	50.861340	495.191300	140.211700
-60.686400	50.780110	492.317300	108.574600
-59.128770	50.678080	606.005100	79.623890
-57.581030	50.555550	611.553200	65.558290
-56.044860	50.412850	497.538500	59.423340
-54.521820	50.250360	428.638900	35.139670
-53.013420	50.068520	395.720000	19.150970
-51.520960	49.867810	378.449700	9.471910
-50.045680	49.648740	370.347700	4.300940
-48.588630	49.411830	367.142600	1.819517
-47.150700	49.157670	366.179500	.676846
-45.732590	48.886830	365.882500	.235895
-44.334810	48.599900	365.785600	.074464
-42.957660	48.297460	365.758100	.021403
-41.601190	47.980120	365.750900	.005575
-40.265250	47.648430	365.749200	.001327
-38.949390	47.302930	365.748900	.000371
-95.710510	48.412890	489.532300	64.966460
-94.362470	48.760130	545.739500	74.142180
-92.993230	49.092850	628.254000	90.494870
-91.602360	49.410460	688.537600	102.601700
-90.189750	49.712350	732.990500	110.892800
-88.755540	49.997940	811.841200	127.147000
-87.300090	50.266570	899.619100	141.182300
-85.824040	50.517650	916.790700	169.515400
-84.328210	50.750570	948.396900	198.301100
-82.813610	50.964740	1009.340000	230.717700
-81.281460	51.159630	1050.939000	229.257000
-79.733130	51.334700	1018.495000	219.682300
-78.170130	51.489480	946.639000	191.943100
-76.594090	51.623530	877.531300	163.386400
-75.006800	51.736490	846.419000	144.749500
-73.410070	51.828010	850.993400	135.088200
-71.805870	51.897850	849.583700	137.517800
-70.196170	51.945790	863.471000	136.598800
-68.582990	51.971680	862.253000	138.746400
-66.968440	51.975460	862.469900	146.013100
-65.354560	51.957120	882.861400	142.533800
-63.743440	51.916690	797.837300	122.072900
-62.137130	51.854320	594.891000	91.448890
-60.537630	51.770170	494.100400	77.000940
-58.946900	51.664510	490.612700	64.746670
-57.366820	51.537620	474.444600	51.505790

-55.799180	51.389870	413.616200	41.490680
-54.245650	51.221680	387.725000	24.528500
-52.707820	51.033510	379.610500	12.764400
-51.187080	50.825860	372.876200	6.545035
-49.684720	50.599280	368.673500	3.080310
-48.201800	50.354330	366.828000	1.278941
-46.739240	50.091630	366.092600	.491509
-45.297720	49.811770	365.846500	.168671
-43.877700	49.515370	365.775800	.053131
-42.479410	49.203060	365.755600	.015221
-41.102810	48.875440	365.750300	.003973
-39.747590	48.533100	365.749100	.000952
-38.413150	48.176570	365.749000	.000271
-96.261670	49.289190	563.390500	77.380930
-94.893750	49.647600	641.523700	85.517190
-93.503820	49.991080	703.292700	99.353360
-92.091320	50.319060	710.435200	101.201100
-90.655950	50.630910	692.752100	100.907200
-89.197760	50.925990	680.539000	108.929500
-87.717060	51.203660	683.600700	124.447000
-86.214420	51.463270	690.300000	142.925400
-84.690660	51.704190	740.672500	165.924600
-83.146820	51.925780	849.282500	171.326900
-81.584160	52.127480	928.508500	187.328500
-80.004110	52.308710	932.363600	178.983800
-78.408280	52.468990	852.038000	175.614400
-76.798410	52.607840	790.073600	160.131700
-75.176390	52.724850	799.104400	138.445500
-73.544200	52.819690	825.476300	129.665200
-71.903920	52.892050	805.583300	125.311200
-70.257710	52.941730	783.065200	111.989000
-68.607750	52.968570	771.632800	104.923200
-66.956280	52.972490	788.558600	119.116100
-65.305560	52.953480	837.768700	143.644300
-63.657820	52.911580	830.528200	136.933500
-62.015270	52.846950	710.111300	104.893000
-60.380080	52.759750	560.981600	73.437620
-58.754360	52.650280	492.750700	52.469720
-57.140120	52.518850	439.964000	37.278630
-55.539290	52.365840	379.921000	30.891470
-53.953660	52.191710	362.538800	20.157670
-52.384890	51.996940	366.209900	9.495469
-50.834480	51.782070	367.212600	4.274566
-49.303730	51.547700	366.734500	1.910833
-47.793770	51.294400	366.178800	.804729
-46.305490	51.022820	365.894500	.304344
-44.839570	50.733620	365.795800	.105424
-43.396390	50.427420	365.762500	.033338
-41.976090	50.104880	365.752500	.009630
-40.578500	49.766630	365.749600	.002536
-39.203150	49.413260	365.748900	.000613
-37.849210	49.045330	365.748900	.000173
-96.841810	50.160530	564.943100	71.313170
-95.453030	50.530490	616.082200	81.481450
-94.041510	50.885130	631.384400	82.098030
-92.606450	51.223850	596.917100	81.052450
-91.147380	51.546020	555.157200	80.674680
-89.664200	51.850960	529.275500	81.842960
-88.157130	52.138020	525.108000	87.143060
-86.626690	52.406500	548.417400	97.703500
-85.073680	52.655720	623.830700	106.943400
-83.499180	52.885050	737.730700	127.585300
-81.904440	53.093850	833.438600	140.350700
-80.290990	53.281520	844.319300	154.960400
-78.660530	53.447530	789.798000	153.505000
-77.014920	53.591380	719.720100	145.001800
-75.356160	53.712650	709.209500	129.422000

-73.686420	53.810940	715.359400	115.503300
-72.007930	53.885960	668.758900	104.021000
-70.322990	53.937460	598.410700	85.878520
-68.634010	53.965280	569.678600	72.867560
-66.943400	53.969350	629.878200	82.531130
-65.253590	53.949630	711.761400	113.243700
-63.567000	53.906200	711.400600	120.764200
-61.886040	53.839190	635.275800	95.747280
-60.213040	53.748820	528.429500	69.107260
-58.550290	53.635360	475.348900	49.229100
-56.899960	53.499180	432.813300	31.191250
-55.264110	53.340690	379.561600	19.357570
-53.644650	53.160350	356.059300	16.207870
-52.043340	52.958710	359.495700	8.478554
-50.461760	52.736320	364.096300	3.433949
-48.901280	52.493820	365.427100	1.346368
-47.363030	52.231850	365.709600	.515589
-45.847900	51.951060	365.753100	.188551
-44.356530	51.652150	365.753900	.064039
-42.889220	51.335790	365.751200	.020118
-41.445980	51.002650	365.749600	.005818
-40.026490	50.653380	365.748900	.001535
-38.630060	50.288590	365.748800	.000379
-37.255620	49.908830	365.748900	.000127
-97.453060	51.026530	487.842200	57.322630
-96.042340	51.408490	510.307200	55.141390
-94.608220	51.774700	499.587800	59.213470
-93.149600	52.124570	474.041400	56.603460
-91.665800	52.457450	453.444100	51.002260
-90.156560	52.772640	441.677700	48.430880
-88.621980	53.069450	441.695400	50.603280
-87.062480	53.347160	457.733800	58.537400
-85.478850	53.605050	502.629400	73.481520
-83.872130	53.842430	577.786400	87.430730
-82.243660	54.058630	640.110400	111.104200
-80.595020	54.253030	695.389500	130.788800
-78.928000	54.425040	712.541000	141.138200
-77.244600	54.574130	670.479600	138.606600
-75.546970	54.699840	648.275900	125.510500
-73.837420	54.801750	654.792400	106.007700
-72.118360	54.879540	606.657700	84.501900
-70.392330	54.932960	488.550500	66.105880
-68.661900	54.961820	414.921800	77.177600
-66.929700	54.966030	489.285000	53.435300
-65.198370	54.945580	577.065700	71.078640
-63.470540	54.900540	571.362400	79.930270
-61.748820	54.831050	515.090100	69.537040
-60.035740	54.737340	465.197300	53.558090
-58.333780	54.619720	447.176800	40.173020
-56.645260	54.478570	425.781300	28.406430
-54.972420	54.314330	387.602000	13.594950
-53.317280	54.127510	362.714700	9.521100
-51.681740	53.918690	359.654300	6.288533
-50.067420	53.688470	363.436100	2.731264
-48.475770	53.437500	365.103800	1.010851
-46.907930	53.166490	365.558800	.376872
-45.364770	52.876130	365.696600	.133172
-43.846840	52.567130	365.735200	.044507
-42.354370	52.240220	365.745500	.013760
-40.887210	51.896080	365.748000	.003950
-39.444820	51.535370	365.748500	.001057
-38.026250	51.158730	365.748700	.000271
-36.630120	50.766700	365.748800	.000122
-98.097820	51.886770	423.665600	34.721990
-96.663910	52.281200	427.043900	34.737130
-95.206040	52.659450	421.323400	33.299480
-93.722790	53.020900	411.828200	30.176610

-92.213200	53.364910	403.516300	27.026520
-90.676770	53.690770	398.772500	25.522130
-89.113440	53.997730	399.322400	26.681090
-87.523560	54.285050	407.281100	31.422070
-85.907830	54.551970	424.908600	40.809170
-84.267300	54.797760	452.404000	56.079780
-82.603340	55.021710	485.575600	79.258080
-80.917590	55.223140	562.332200	98.970690
-79.211960	55.401440	641.795700	127.151100
-77.488560	55.556020	663.786800	129.632900
-75.749730	55.686380	636.294100	125.368200
-73.997920	55.792100	633.062200	103.099500
-72.235800	55.872810	591.946400	75.167960
-70.466090	55.928230	452.145900	65.546610
-68.691570	55.958180	368.746200	91.136760
-66.915130	55.962550	426.654400	54.899470
-65.139640	55.941330	491.539800	48.270950
-63.367970	55.894590	483.553600	52.109520
-61.602940	55.822490	447.592200	46.839380
-59.847310	55.725280	427.518200	37.826790
-58.103760	55.603290	423.350000	31.896000
-56.374820	55.456930	410.764500	23.908440
-54.662870	55.286670	388.729700	12.640240
-52.970100	55.093080	369.180000	5.589112
-51.298500	54.876750	364.780900	2.842866
-49.649800	54.638340	365.082800	1.302603
-48.025460	54.378550	365.411800	.572492
-46.426660	54.098120	365.621900	.228915
-44.854210	53.797780	365.704300	.086326
-43.308580	53.478290	365.734800	.029740
-41.789850	53.140400	365.744800	.009527
-40.297680	52.784830	365.747700	.002794
-38.831280	52.412250	365.748400	.000756
-37.389370	52.023280	365.748700	.000204
-35.970190	51.618480	365.748900	.000109
-98.778840	52.740810	390.997700	18.223690
-97.320270	53.148240	389.969700	17.430810
-95.837370	53.539010	387.738300	16.291950
-94.328290	53.912530	384.159300	14.608370
-92.791720	54.268120	380.903700	12.993650
-91.226900	54.605080	378.958500	12.110360
-89.633560	54.922620	378.970200	12.504190
-88.011890	55.219970	381.220100	14.666840
-86.362530	55.496330	384.897600	19.776780
-84.686490	55.750910	386.914300	30.968790
-82.985150	55.982970	393.402600	51.138560
-81.260250	56.191770	448.585500	74.481670
-79.513790	56.376650	570.004400	99.775730
-77.748020	56.536990	654.020100	127.318700
-75.965460	56.672260	655.002900	122.349300
-74.168790	56.781970	619.999600	104.482500
-72.360860	56.865740	582.574300	77.471340
-70.544630	56.923280	496.617900	59.626830
-68.723180	56.954370	428.240700	58.285980
-66.899620	56.958910	450.783400	46.567640
-65.077100	56.936880	461.827500	43.081380
-63.258730	56.888360	442.394200	41.259850
-61.447630	56.813510	420.527700	34.971390
-59.646790	56.712620	411.039200	29.599980
-57.859090	56.586040	410.804600	26.848940
-56.087300	56.434210	397.345900	19.664990
-54.333970	56.257640	382.367700	10.921960
-52.601470	56.056940	370.934700	4.661181
-50.891880	55.832740	366.949600	1.779248
-49.207030	55.585770	365.860900	.687445
-47.548420	55.316760	365.701800	.273592
-45.917200	55.026500	365.705100	.114149

-44.314130	54.715760	365.727700	.045230
-42.739580	54.385350	365.740800	.016721
-41.193420	54.036030	365.746200	.005588
-39.675070	53.668560	365.747900	.001725
-38.183400	53.283620	365.748500	.000493
-36.716770	52.881820	365.748700	.000150
-35.272900	52.463700	365.748900	.000106
-99.499240	53.588130	375.908300	8.699308
-98.014280	54.009130	375.011400	8.030271
-96.504860	54.412960	373.948800	7.312868
-94.968610	54.799050	372.605500	6.456632
-93.403790	55.166730	371.323100	5.620315
-91.809290	55.515250	370.372300	5.033159
-90.184560	55.843850	369.731800	4.853070
-88.529620	56.151670	368.578500	5.730195
-86.844990	56.437890	364.052800	10.211790
-85.131650	56.701690	350.400800	22.378650
-83.390960	56.942230	334.006900	41.738560
-81.624720	57.158780	362.934300	54.761670
-79.835050	57.350580	479.354800	75.578450
-78.024370	57.516980	620.825900	109.937200
-76.195360	57.657400	657.636500	122.738900
-74.350940	57.771310	619.503300	109.903500
-72.494220	57.858320	606.796900	84.615230
-70.628410	57.918090	568.097800	62.912310
-68.756890	57.950390	513.347200	49.569500
-66.883060	57.955100	474.223900	46.706830
-65.010370	57.932220	442.866600	41.199280
-63.142240	57.881810	424.927900	35.285380
-61.282040	57.804080	412.799700	30.539410
-59.433060	57.699310	406.743600	26.774190
-57.598450	57.567890	404.908700	24.359670
-55.781200	57.410300	390.126700	16.678870
-53.984060	57.227100	377.078400	9.023070
-52.209560	57.018940	370.029400	4.053884
-50.459930	56.786500	367.100600	1.594544
-48.737060	56.530560	366.109700	.558910
-47.042490	56.251900	365.818600	.182542
-45.377330	55.951360	365.752800	.060718
-43.742270	55.629770	365.744500	.021482
-42.137470	55.287960	365.746100	.007780
-40.562610	54.926740	365.747700	.002719
-39.016750	54.546860	365.748300	.000891
-37.498410	54.149020	365.748600	.000264
-36.005400	53.733830	365.748700	.000104
-34.534880	53.301770	365.748900	.000076
-100.262700	54.428120	369.564900	3.805200
-98.749250	54.863350	369.066400	3.392572
-97.211560	55.280850	368.609500	3.008730
-95.646590	55.680060	368.093200	2.580724
-94.052090	56.060360	367.545300	2.128515
-92.426510	56.420970	366.910300	1.663317
-90.768940	56.761100	365.732400	1.726697
-89.079190	57.079890	362.427900	4.535478
-87.357570	57.376450	352.507000	12.033030
-85.605000	57.649890	326.799500	27.389680
-83.822840	57.899360	289.383600	48.289600
-82.012920	58.124030	298.092100	50.409530
-80.177480	58.323120	399.910100	51.279380
-78.319110	58.495910	554.673800	85.896720
-76.440710	58.641750	643.094400	115.183700
-74.545430	58.760110	647.109900	113.516000
-72.636660	58.850530	658.532600	99.255520
-70.717930	58.912650	642.819200	79.370150
-68.792920	58.946230	566.659900	61.612080
-66.865380	58.951130	480.481200	51.387620
-64.939080	58.927340	431.792400	37.259470

-63.017780 58.874950 413.153200 29.015190
-61.105210 58.794160 408.252300 26.824390
-59.204930 58.685300 407.149000 25.222870
-57.320400 58.548780 402.857600 22.616030
-55.454840 58.385130 386.442100 14.466300
-53.611270 58.194950 374.473800 7.379824
-51.792370 57.978940 369.071700 3.307655
-50.000500 57.737840 366.903100 1.336697
-48.237630 57.472490 366.106600 .482604
-46.505310 57.183730 365.844500 .157612
-44.804610 56.872430 365.769700 .046688
-43.136050 56.539500 365.752100 .013269
-41.499610 56.185780 365.748700 .003837
-39.894630 55.812120 365.748500 .001184
-38.319800 55.419290 365.748600 .000388
-36.773090 55.007980 365.748700 .000137
-35.251730 54.578750 365.748700 .000080
-33.752120 54.132060 365.748900 .000062
-101.073700 55.260130 367.075700 1.525980
-99.529010 55.710320 366.853200 1.317255
-97.960900 56.142140 366.667700 1.125700
-96.365430 56.555100 366.460000 .912197
-94.739640 56.948590 366.172900 .634191
-93.081440 57.321860 365.598500 .595499
-91.389490 57.674070 364.017900 2.259602
-89.663250 58.004340 359.258400 6.784538
-87.902820 58.311730 345.254800 16.952250
-86.109000 58.595320 308.872800 37.027690
-84.283100 58.854170 251.906300 62.454930
-82.427000 59.087400 245.576000 60.451820
-80.543050 59.294160 341.784200 36.828760
-78.634010 59.473690 491.394100 62.189430
-76.703000 59.625280 620.810900 101.050200
-74.753460 59.748340 675.825700 116.171900
-72.789070 59.842370 710.305800 113.713500
-70.813740 59.906970 693.579600 96.744320
-68.831480 59.941910 597.585800 73.110780
-66.846440 59.947010 491.397800 52.881080
-64.862770 59.922250 424.559300 32.092470
-62.884610 59.867760 395.772400 18.088610
-60.916040 59.783740 391.882900 14.915600
-58.961010 59.670550 397.334000 17.979370
-57.023270 59.528640 396.353200 18.042330
-55.106350 59.358580 382.732100 11.191490
-53.213500 59.161040 372.442700 5.561615
-51.347600 58.936760 368.205300 2.453463
-49.511150 58.686560 366.612500 1.001025
-47.706190 58.411320 366.029400 .370972
-45.934240 58.111950 365.831200 .124249
-44.196280 57.789400 365.770300 .037899
-42.492650 57.444580 365.753600 .010488
-40.823030 57.078400 365.749600 .002672
-39.186360 56.691730 365.748900 .000640
-37.580830 56.285340 365.748700 .000174
-36.003750 55.859910 365.748700 .000085
-34.451600 55.415950 365.748700 .000064
-32.919830 54.953830 365.748900 .000042
-101.937200 56.083360 366.175000 .563198
-100.358100 56.549350 366.088300 .466561
-98.756940 56.996240 366.014800 .376540
-97.128790 57.423640 365.916800 .259175
-95.469860 57.830970 365.710600 .186142
-93.777320 58.217490 365.121000 .877549
-92.049300 58.582370 363.280500 2.995280
-90.284800 58.924680 357.616300 8.354078
-88.483640 59.243460 340.750300 20.634290
-86.646400 59.537720 296.219000 45.196320

-84.774360	59.806450	224.663500	75.049000
-82.869400	60.048700	210.996900	73.153850
-80.933970	60.263580	310.806500	35.787940
-78.971000	60.450230	448.150100	40.608340
-76.983890	60.607900	574.121800	78.882420
-74.976370	60.735940	641.820600	103.436200
-72.952470	60.833800	694.903900	117.086700
-70.916480	60.901050	708.186000	106.718100
-68.872850	60.937420	631.397000	80.697700
-66.826130	60.942730	503.770200	52.319450
-64.780930	60.916960	412.535000	25.862450
-62.741830	60.860230	364.204400	16.058220
-60.713310	60.772790	352.656200	14.881830
-58.699720	60.654990	366.196400	4.702302
-56.705200	60.507380	376.799700	6.050903
-54.733590	60.330550	373.609300	5.181663
-52.788400	60.125220	369.262800	2.959396
-50.872730	59.892220	367.149000	1.426825
-48.989200	59.632420	366.268500	.615814
-47.139900	59.346780	365.926200	.237748
-45.326320	59.036270	365.803800	.082877
-43.549270	58.701890	365.764200	.026201
-41.808860	58.344600	365.752600	.007510
-40.104370	57.965370	365.749600	.001928
-38.434220	57.565060	365.748900	.000439
-36.795950	57.144450	365.748700	.000097
-35.186060	56.704150	365.748700	.000067
-33.600030	56.244660	365.748700	.000061
-32.032190	55.766200	365.748900	.000026
-102.859900	56.896880	365.875400	.189899
-101.242000	57.379640	365.844100	.148926
-99.604390	57.842470	365.812300	.104669
-97.940930	58.285070	365.750700	.054642
-96.246660	58.706940	365.568400	.269766
-94.517820	59.107400	364.970000	1.035173
-92.751850	59.485580	363.047700	3.199921
-90.947170	59.840560	357.107000	8.669483
-89.103220	60.171320	339.467900	21.181090
-87.220290	60.476800	292.899100	46.103850
-85.299550	60.755960	217.659400	76.214130
-83.342830	61.007760	205.480200	76.630350
-81.352710	61.231220	311.551500	36.891620
-79.332320	61.425420	430.052400	26.998600
-77.285290	61.589540	502.151400	55.854290
-75.215710	61.722870	542.383400	78.814160
-73.128000	61.824790	597.562300	98.805440
-71.026890	61.894870	665.834600	107.216400
-68.917300	61.932760	657.051900	85.793980
-66.804310	61.938290	512.412500	50.653630
-64.692990	61.911450	390.736900	28.815980
-62.588420	61.852340	330.166400	37.639510
-60.495590	61.761240	317.662300	39.053570
-58.419300	61.638580	337.756800	21.473300
-56.364090	61.484900	358.797700	6.274237
-54.334160	61.300890	365.792400	.873598
-52.333320	61.087320	366.550900	.723546
-50.364880	60.845090	366.246600	.538615
-48.431600	60.575160	365.974200	.279753
-46.535590	60.278550	365.834900	.119784
-44.678250	59.956310	365.777500	.044687
-42.860180	59.609490	365.757300	.014866
-41.081100	59.239120	365.751000	.004434
-39.339830	58.846180	365.749300	.001170
-37.634110	58.431540	365.748900	.000275
-35.960610	57.995940	365.748700	.000070
-34.314830	57.539980	365.748700	.000061
-32.690990	57.064010	365.748700	.000061

-31.082000 56.568130 365.748900 .000022
-103.849600 57.699570 365.783300 .057839
-102.187300 58.200280 365.771800 .040722
-100.509000 58.680030 365.754700 .019291
-98.806840 59.138710 365.706300 .068517
-97.074520 59.575940 365.539200 .297444
-95.307120 59.991050 364.973800 .997022
-93.501110 60.383240 363.162900 2.943779
-91.654170 60.751550 357.632700 7.724935
-89.765210 61.094930 341.526900 18.358190
-87.834170 61.412270 300.075300 38.469490
-85.861950 61.702450 235.498000 63.184840
-83.850420 61.964360 237.388700 62.557000
-81.802150 62.196920 344.589900 31.003610
-79.720490 62.399150 431.492000 29.735980
-77.609360 62.570130 463.794900 39.860990
-75.473210 62.709080 458.832300 57.848300
-73.316960 62.815350 494.666000 77.510680
-71.145790 62.888430 588.182200 86.557190
-68.965200 62.927950 617.106300 84.528190
-66.780790 62.933720 501.694000 46.298480
-64.598260 62.905720 382.756300 31.225370
-62.423240 62.844070 332.103300 34.962910
-60.261290 62.749080 328.237500 30.682690
-58.117710 62.621230 345.117300 17.287110
-55.997520 62.461110 358.976400 6.554857
-53.905320 62.269450 364.173700 1.856960
-51.845240 62.047150 365.565000 .335563
-49.820820 61.795150 365.816300 .083219
-47.834930 61.514500 365.810900 .080579
-45.889670 61.206310 365.778900 .044196
-43.986290 60.871700 365.760100 .018762
-42.125070 60.511780 365.752400 .006755
-40.305270 60.127630 365.749700 .002123
-38.525020 59.720250 365.748900 .000576
-36.781200 59.290500 365.748800 .000136
-35.069400 58.839090 365.748700 .000067
-33.383770 58.366500 365.748700 .000061
-31.716990 57.872960 365.748700 .000061
-30.060130 57.358350 365.748900 .000000
-104.916500 58.490070 365.757100 .015258
-103.202300 59.010120 365.752300 .007968
-101.477600 59.507980 365.740600 .014497
-99.732440 59.983750 365.700500 .073741
-97.958720 60.437220 365.556600 .265595
-96.150010 60.867820 365.073800 .839068
-94.301540 61.274790 363.556200 2.376101
-92.410060 61.657170 359.027800 6.018443
-90.473700 62.013880 346.364200 13.644460
-88.491920 62.343760 315.364300 26.700520
-86.465330 62.645610 275.385300 41.558940
-84.395630 62.918240 297.222200 36.212130
-82.285480 63.160470 382.206600 24.391590
-80.138370 63.371240 429.464100 27.515360
-77.958560 63.549540 429.405300 28.790190
-75.750930 63.694510 419.923300 34.090720
-73.520860 63.805420 432.294600 47.750120
-71.274150 63.881700 490.197100 60.168440
-69.016910 63.922960 531.597500 59.478480
-66.755400 63.928990 457.973600 37.850670
-64.495980 63.899750 384.237200 21.283650
-62.244980 63.835380 355.480800 17.525150
-60.008560 63.736250 352.632400 14.581160
-57.792640 63.602840 357.823600 8.549015
-55.602750 63.435840 362.341300 3.932301
-53.443960 63.236060 364.613300 1.453492
-51.320750 63.004450 365.451000 .431244

-49.236890	62.742080	365.696100	.091926
-47.195330	62.450080	365.749600	.011820
-45.198120	62.129630	365.754600	.009321
-43.246250	61.781950	365.751900	.005544
-41.339580	61.408210	365.750000	.002353
-39.476710	61.009530	365.749100	.000795
-37.654880	60.586900	365.748900	.000240
-35.869840	60.141170	365.748700	.000070
-34.115770	59.672970	365.748700	.000064
-32.385130	59.182660	365.748700	.000061
-30.668580	58.670220	365.748900	.000058
-28.954870	58.135290	365.748900	.000000
-106.073800	59.266680	365.750200	.002973
-104.297500	59.807790	365.747700	.002657
-102.518800	60.325180	365.739100	.016341
-100.725000	60.819250	365.707300	.062962
-98.905530	61.289990	365.595100	.209974
-97.052100	61.737010	365.229500	.631808
-95.158340	62.159620	364.121200	1.709512
-93.219700	62.556880	360.990800	4.109190
-91.233350	62.927700	352.604200	8.717339
-89.198040	63.270870	334.232000	15.340140
-87.113920	63.585110	319.873400	21.214850
-84.982480	63.869130	348.673200	15.208480
-82.806390	64.121680	399.935200	20.271800
-80.589290	64.341560	411.387200	19.205990
-78.335770	64.527670	399.873600	18.612030
-76.051160	64.679080	394.372800	19.811670
-73.741460	64.794950	399.639100	24.696370
-71.413090	64.874680	420.797000	32.586510
-69.072890	64.917820	441.179200	32.271870
-66.727910	64.924110	410.413600	23.347010
-64.385280	64.893550	380.744000	11.807180
-62.052090	64.826280	366.721800	7.113892
-59.735260	64.722690	363.016200	5.438285
-57.441380	64.583340	363.255400	3.593982
-55.176610	64.408980	364.400000	1.887641
-52.946510	64.200520	365.184600	.815319
-50.755950	63.959000	365.555900	.292411
-48.608900	63.685590	365.695300	.087126
-46.508400	63.381530	365.737600	.020500
-44.456330	63.048090	365.747500	.003249
-42.453320	62.686580	365.749100	.000696
-40.498620	62.298210	365.749000	.000470
-38.589950	61.884150	365.748800	.000222
-36.723370	61.445380	365.748900	.000075
-34.893150	60.982680	365.748900	.000061
-33.091610	60.496560	365.748800	.000048
-31.309040	59.987160	365.748800	.000043
-29.533510	59.454220	365.748900	.000013
-27.750760	58.896990	365.748900	.000000
-107.339200	60.027230	365.748600	.000822
-105.487000	60.591610	365.746900	.003713
-103.643800	61.130270	365.740100	.014856
-101.793500	61.644070	365.715600	.051196
-99.922550	62.133320	365.633100	.160076
-98.020040	62.597820	365.380600	.455298
-96.077510	63.037030	364.680400	1.155469
-94.088710	63.450070	362.895400	2.574570
-92.049460	63.835860	358.875000	4.937917
-89.957580	64.193130	351.735700	7.493051
-87.812570	64.520550	350.589500	7.799380
-85.615560	64.816710	372.966200	7.620311
-83.369120	65.080260	394.064200	12.792230
-81.077050	65.309890	390.227100	12.314600
-78.744270	65.504390	383.210600	11.269470
-76.376630	65.662700	380.007600	11.369690

-73.980770 65.783920 381.992300 13.173670
-71.563890 65.867360 388.552900 15.957990
-69.133670 65.912500 393.731600 16.732110
-66.698060 65.919100 384.846500 12.356630
-64.265090 65.887100 374.617400 6.933390
-61.842790 65.816700 368.274000 3.478597
-59.438900 65.708320 365.719600 2.108050
-57.060850 65.562580 365.152000 1.407900
-54.715470 65.380330 365.286300 .813641
-52.408880 65.162570 365.511200 .391127
-50.146370 64.910470 365.652800 .160005
-47.932120 64.625290 365.716700 .056434
-45.769150 64.308400 365.739700 .017288
-43.659080 63.961160 365.746700 .004537
-41.602020 63.584970 365.748500 .000977
-39.596350 63.181100 365.748800 .000167
-37.638600 62.750720 365.748900 .000061
-35.723270 62.294770 365.748900 .000000
-33.842640 61.813940 365.748900 .000000
-31.986660 61.308540 365.748900 .000000
-30.142740 60.778430 365.748900 .000061
-28.295590 60.222970 365.748900 .000000
-26.427080 59.640870 365.748900 .000000
-108.736700 60.768890 365.748200 .001529
-106.789100 61.359350 365.746500 .004448
-104.866900 61.921510 365.739800 .015355
-102.949500 62.456830 365.716900 .049213
-101.019200 62.966030 365.644800 .144331
-99.061880 63.449270 365.442900 .382983
-97.066180 63.906190 364.943700 .903291
-95.023590 64.336020 363.857000 1.834705
-92.928180 64.737730 361.935400 3.121682
-90.776390 65.110020 359.833600 4.017166
-88.566850 65.451470 362.137000 2.912669
-86.300130 65.760610 376.677800 5.629788
-83.978550 66.035940 383.000800 8.461151
-81.606060 66.276030 380.448600 8.728499
-79.187900 66.479550 376.768400 8.177906
-76.730480 66.645290 374.755300 8.005210
-74.241170 66.772270 375.382700 8.720231
-71.728080 66.859700 377.882400 9.907632
-69.199870 66.907030 379.217900 10.013460
-66.665550 66.913940 376.055700 7.928714
-64.134220 66.880400 371.455500 4.936235
-61.614990 66.806620 368.069700 2.550083
-59.116610 66.693070 366.312000 1.283657
-56.647430 66.540450 365.678900 .738046
-54.215080 66.349720 365.579600 .431831
-51.826330 66.121990 365.634900 .225717
-49.486860 65.858530 365.694400 .102789
-47.201050 65.560780 365.727500 .041233
-44.971820 65.230190 365.741600 .014687
-42.800360 64.868270 365.746700 .004677
-40.685980 64.476460 365.748300 .001313
-38.625890 64.056100 365.748800 .000340
-36.614990 63.608330 365.748900 .000088
-34.645670 63.134020 365.748900 .000029
-32.707610 62.633670 365.748900 .000000
-30.787590 62.107330 365.748900 .000000
-28.869240 61.554470 365.748900 .000000
-26.932890 60.973880 365.748900 .000000
-24.955330 60.363580 365.748900 .000000

Q.18a

-89.453400	32.991660	386.762700	1.125989
-88.345930	33.191160	387.055000	1.294769
-87.228100	33.381660	388.038200	1.846684
-86.100230	33.562890	389.580800	2.692436
-84.962690	33.734530	391.459400	3.696131
-83.815900	33.896310	393.370700	4.691795
-82.660300	34.047950	395.022300	5.519042
-81.496410	34.189190	396.133100	6.015152
-80.324730	34.319790	396.345200	5.983725
-79.145840	34.439500	395.214700	5.233822
-77.960310	34.548100	392.432700	3.742175
-76.768760	34.645400	388.375600	1.960686
-75.571840	34.731200	384.731500	1.041254
-74.370180	34.805370	383.263300	1.105551
-73.164480	34.867740	383.920100	.786712
-71.955430	34.918200	385.401700	.540370
-70.743730	34.956650	386.620500	.870641
-69.530080	34.983020	387.145100	1.125144
-68.315220	34.997260	387.080000	1.118348
-67.099880	34.999340	386.675200	.908844
-65.884770	34.989250	386.178800	.618965
-64.670640	34.967020	385.768500	.356405
-63.458190	34.932690	385.509200	.175052
-62.248150	34.886320	385.377200	.073238
-61.041230	34.828000	385.322000	.025804
-59.838120	34.757850	385.302700	.007303
-58.639500	34.676010	385.297300	.001400
-57.446040	34.582610	385.296300	.000275
-56.258390	34.477850	385.296200	.000311
-55.077160	34.361910	385.296300	.000201
-53.902950	34.235010	385.296400	.000108
-52.736340	34.097380	385.296400	.000049
-51.577860	33.949260	385.296600	.000194
-50.428010	33.790910	385.296600	.000190
-49.287250	33.622600	385.296600	.000216
-48.156040	33.444620	385.296600	.000244
-47.034740	33.257250	385.296600	.000244
-45.923690	33.060790	385.296600	.000244
-44.823220	32.855560	385.296600	.000244
-89.701210	33.918400	388.040600	1.793052
-88.583520	34.125020	388.762900	2.143290
-87.455040	34.322360	390.852200	3.117418
-86.316120	34.510110	394.104900	4.592043
-85.167100	34.687960	398.047400	6.329672
-84.008420	34.855620	401.987300	8.025963
-82.840550	35.012790	405.346900	9.417610
-81.663990	35.159200	407.662900	10.267350
-80.479300	35.294580	408.269800	10.265000

-79.287040	35.418700	406.171300	9.031833
-78.087840	35.531310	400.458600	6.434697
-76.882320	35.632210	391.542800	3.301846
-75.671150	35.721200	383.480300	1.795093
-74.455030	35.798110	380.877500	1.687006
-73.234660	35.862810	383.129900	1.170955
-72.010770	35.915150	386.564700	1.355375
-70.784100	35.955040	388.989600	2.057668
-69.555380	35.982390	389.862400	2.414665
-68.325390	35.997170	389.487800	2.268184
-67.094890	35.999320	388.446300	1.788008
-65.864640	35.988860	387.243300	1.180189
-64.635410	35.965800	386.290600	.654064
-63.407950	35.930180	385.719800	.306537
-62.183030	35.882080	385.446700	.121756
-60.961370	35.821590	385.339400	.039811
-59.743720	35.748840	385.305100	.009842
-58.530780	35.663950	385.296400	.001433
-57.323260	35.567100	385.295300	.001182
-56.121820	35.458470	385.295600	.000916
-54.927120	35.338260	385.296000	.000519
-53.739770	35.206700	385.296200	.000241
-52.560380	35.064020	385.296300	.000094
-51.389480	34.910500	385.296400	.000060
-50.227610	34.746390	385.296400	.000070
-49.075240	34.571990	385.296600	.000194
-47.932830	34.387580	385.296600	.000188
-46.800740	34.193490	385.296600	.000244
-45.679330	33.990000	385.296600	.000244
-44.568900	33.777440	385.296600	.000244
-89.960660	34.843320	391.803300	3.491329
-88.832330	35.057210	393.872700	4.313150
-87.692770	35.261540	399.356400	6.370813
-86.542320	35.455950	407.966500	9.516952
-85.381340	35.640140	418.394400	13.221260
-84.210270	35.813810	428.547300	16.740670
-83.029580	35.976620	437.005400	19.551020
-81.839790	36.128310	442.980900	21.309430
-80.641480	36.268600	444.959500	21.420540
-79.435230	36.397220	440.205500	18.955290
-78.221690	36.513940	425.938800	13.408060
-77.001530	36.618520	401.029800	7.015985
-75.775430	36.710780	378.947900	4.123429
-74.544130	36.790520	374.842700	4.303986
-73.308360	36.857590	382.398800	4.050839
-72.068890	36.911860	392.320200	5.056030
-70.826490	36.953220	397.947900	6.086866
-69.581950	36.981590	399.315700	6.336783
-68.336070	36.996900	397.718300	5.715042
-67.089650	36.999130	394.434000	4.381883
-65.843500	36.988290	390.754100	2.799358
-64.598400	36.964370	387.941500	1.483225
-63.355190	36.927440	386.355500	.661315
-62.114630	36.877570	385.646800	.249651
-60.877510	36.814860	385.387100	.077258
-59.644590	36.739430	385.309400	.016882
-58.416640	36.651430	385.292600	.004322
-57.194370	36.551040	385.291800	.004438
-55.978490	36.438440	385.293500	.003013
-54.769680	36.313860	385.295000	.001604
-53.568590	36.177530	385.295800	.000721
-52.375820	36.029710	385.296200	.000280
-51.191970	35.870660	385.296300	.000116
-50.017550	35.700660	385.296300	.000043
-48.853080	35.520030	385.296400	.000052
-47.698980	35.329070	385.296400	.000077
-46.555660	35.128100	385.296400	.000085

-45.423470	34.917440	385.296300	.000031
-44.302690	34.697410	385.296600	.000244
-90.232250	35.766320	399.436400	6.189419
-89.092860	35.987640	404.716200	7.866677
-87.941790	36.199090	418.244700	11.952150
-86.779330	36.400320	440.205900	18.527890
-85.605870	36.591000	467.108500	26.363520
-84.421870	36.770790	492.308700	33.504860
-83.227800	36.939400	512.544600	38.985880
-82.024180	37.096500	527.274600	42.526150
-80.811620	37.241800	533.486100	43.079190
-79.590740	37.375040	524.403500	38.422090
-78.362190	37.495960	489.991600	27.339550
-77.126670	37.604320	421.729300	16.227900
-75.884930	37.699920	362.030000	10.393030
-74.637700	37.782550	362.646300	16.237670
-73.385770	37.852060	391.626300	17.075070
-72.129940	37.908310	413.568300	16.078750
-70.871030	37.951180	425.760000	16.709610
-69.609860	37.980580	426.355500	15.831780
-68.347290	37.996460	420.447100	13.691190
-67.084140	37.998780	410.669000	10.262030
-65.821270	37.987530	399.758800	6.278318
-64.559530	37.962740	391.749900	3.071101
-63.299760	37.924460	387.645800	1.262834
-62.042780	37.872770	385.992900	.444737
-60.789440	37.807780	385.447200	.130952
-59.540510	37.729610	385.302300	.030462
-58.296810	37.638420	385.277200	.015945
-57.059080	37.534390	385.280700	.013556
-55.828080	37.417740	385.287700	.008587
-54.604500	37.288680	385.292500	.004405
-53.389020	37.147460	385.294900	.001937
-52.182290	36.994370	385.296000	.000766
-50.984880	36.829660	385.296300	.000300
-49.797380	36.653650	385.296300	.000113
-48.620270	36.466650	385.296300	.000045
-47.454020	36.268990	385.296300	.000054
-46.299010	36.060990	385.296400	.000047
-45.155610	35.843010	385.296300	.000031
-44.024070	35.615370	385.296600	.000244
-90.516530	36.687310	411.767200	9.180202
-89.365660	36.916220	423.387500	12.028760
-88.202590	37.134950	453.453400	19.364640
-87.027650	37.343150	506.396300	32.479050
-85.841200	37.540460	573.850100	48.432530
-84.643710	37.726540	632.560100	62.421110
-83.435650	37.901050	676.212300	72.499630
-82.217600	38.063690	710.059900	78.813080
-80.990140	38.214140	730.261500	80.178880
-79.753940	38.352110	714.836300	73.461990
-78.509670	38.477340	635.512600	58.509590
-77.258070	38.589580	475.051600	37.091380
-75.999910	38.688600	293.800800	22.819500
-74.735980	38.774210	330.763400	56.269740
-73.467090	38.846230	441.124900	55.309680
-72.194090	38.904510	491.031500	44.236480
-70.917830	38.948930	501.792400	38.859700
-69.639210	38.979390	496.576000	35.483150
-68.359090	38.995840	478.255400	29.594990
-67.078360	38.998240	451.769000	21.674090
-65.797930	38.986590	420.312000	12.512690
-64.518690	38.960900	398.717300	5.366959
-63.241520	38.921240	389.358100	1.887445
-61.967320	38.867690	386.272400	.627002
-60.696920	38.800350	385.425400	.205397
-59.431210	38.719360	385.247300	.077042

-58.170970	38.624900	385.235500	.048796
-56.917050	38.517150	385.255300	.036127
-55.670200	38.396340	385.275700	.022286
-54.431160	38.262690	385.288700	.011517
-53.200640	38.116470	385.294600	.005406
-51.979310	37.957960	385.296500	.002543
-50.767770	37.787460	385.296800	.001215
-49.566610	37.605300	385.296500	.000530
-48.376340	37.411790	385.296300	.000188
-47.197410	37.207270	385.296300	.000061
-46.030250	36.992090	385.296300	.000061
-44.875180	36.766620	385.296300	.000031
-43.732470	36.531200	385.296600	.000188
-90.814130	37.606180	426.327000	10.995860
-89.651300	37.842850	447.118400	14.846130
-88.475770	38.069030	504.738500	26.408880
-87.287830	38.284350	628.161900	49.750760
-86.087840	38.488450	794.113200	79.734690
-84.876290	38.680970	925.711800	105.558200
-83.653650	38.861550	1010.606000	122.325200
-82.420500	39.029860	1082.585000	131.513200
-81.177470	39.185590	1135.027000	135.203600
-79.925230	39.328410	1117.328000	132.257800
-78.664510	39.458070	989.188700	113.797300
-77.396060	39.574290	658.099100	79.179020
-76.120680	39.676830	118.451300	143.511600
-74.839220	39.765490	259.248200	157.472400
-73.552520	39.840080	536.278900	131.210200
-72.261480	39.900440	692.192900	96.116740
-70.967010	39.946460	694.224300	76.289520
-69.670030	39.978020	657.789600	65.021760
-68.371470	39.995060	612.575900	53.948390
-67.072270	39.997550	545.528400	38.251120
-65.773400	39.985470	457.889800	19.936580
-64.475770	39.958870	406.611600	6.927994
-63.180340	39.917780	389.054800	2.328091
-61.888020	39.862300	385.507300	1.158502
-60.599740	39.792560	385.094400	.497195
-59.316390	39.708680	385.112000	.221786
-58.038840	39.610860	385.164900	.140798
-56.767930	39.499290	385.218200	.098476
-55.504470	39.374200	385.265400	.060678
-54.249260	39.235840	385.293000	.034336
-53.003000	39.084480	385.302600	.019430
-51.766410	38.920440	385.302700	.010847
-50.540120	38.744010	385.300100	.005540
-49.324710	38.555530	385.298000	.002493
-48.120720	38.355340	385.296900	.000962
-46.928620	38.143810	385.296500	.000311
-45.748810	37.921300	385.296300	.000082
-44.581620	37.688170	385.296300	.000036
-43.427300	37.444810	385.296600	.000190
-91.125630	38.522790	434.424500	12.001230
-89.950410	38.767390	458.538300	18.786330
-88.761920	39.001200	541.292900	36.060290
-87.560450	39.223820	799.740800	64.988840
-86.346370	39.434880	1177.884000	119.644100
-85.120160	39.633990	1464.396000	161.872500
-83.882300	39.820790	1623.580000	185.495500
-82.633390	39.994930	1745.902000	194.849000
-81.374080	40.156070	1849.859000	203.745400
-80.105060	40.303890	1876.454000	213.186700
-78.827100	40.438090	1784.085000	201.928100
-77.540980	40.558400	1403.609000	142.510400
-76.247540	40.664560	462.568800	238.786800
-74.947690	40.756360	136.289300	199.328800
-73.642300	40.833600	649.119000	313.399500

-72.332310	40.896110	957.429700	130.472500
-71.018690	40.943760	1036.538000	121.312500
-69.702430	40.976440	946.548100	97.927070
-68.384490	40.994090	843.918800	78.732390
-67.065880	40.996670	686.335200	54.247400
-65.747600	40.984170	487.780300	23.782890
-64.430650	40.956610	389.379500	7.987309
-63.116020	40.914060	378.725800	6.497895
-61.804690	40.856610	381.964300	3.436033
-60.497620	40.784390	384.312900	1.330393
-59.195770	40.697540	385.047900	.608880
-57.900040	40.596260	385.171300	.434457
-56.611330	40.480750	385.229800	.304395
-55.330490	40.351270	385.313600	.190356
-54.058330	40.208070	385.355900	.117911
-52.795620	40.051450	385.353500	.073067
-51.543080	39.881710	385.334100	.042387
-50.301390	39.699200	385.315900	.021770
-49.071130	39.504250	385.304700	.009764
-47.852850	39.297230	385.299400	.003812
-46.647030	39.078520	385.297200	.001296
-45.454070	38.848490	385.296600	.000394
-44.274280	38.607530	385.296300	.000088
-43.107920	38.356030	385.296500	.000135
-91.451740	39.437030	424.797900	17.418690
-90.263630	39.689750	421.122900	36.102570
-89.061680	39.931370	457.672300	63.171910
-87.846150	40.161480	819.736600	111.681600
-86.617400	40.379670	1468.112000	207.581300
-85.375900	40.585540	2023.572000	258.762000
-84.122160	40.778730	2335.399000	261.836300
-82.856800	40.958850	2492.422000	266.811300
-81.580460	41.125550	2621.707000	279.990700
-80.293880	41.278500	2791.796000	306.758900
-78.997860	41.417370	2922.073000	333.516700
-77.693230	41.541890	2683.587000	318.979600
-76.380870	41.651780	1826.189000	227.627800
-75.061690	41.746810	786.578500	135.852000
-73.736660	41.826770	386.685500	291.880500
-72.406780	41.891490	938.826200	126.183100
-71.073040	41.940830	1275.814000	160.421600
-69.736500	41.974670	1168.314000	131.370300
-68.398190	41.992950	971.839700	108.238100
-67.059160	41.995620	671.073200	88.088600
-65.720480	41.982670	346.352700	49.200770
-64.383220	41.954140	286.967900	34.092590
-63.048400	41.910080	341.254700	17.612040
-61.717090	41.850600	376.124300	6.941986
-60.390290	41.775820	384.861800	2.604730
-59.069010	41.685920	385.988900	1.781275
-57.754210	41.581080	385.700000	1.379171
-56.446830	41.461530	385.541400	.942294
-55.147770	41.327530	385.650100	.598320
-53.857880	41.179360	385.664600	.388857
-52.577970	41.017320	385.569700	.247424
-51.308780	40.841740	385.455900	.141887
-50.051000	40.652980	385.373900	.072077
-48.805250	40.451390	385.328700	.032179
-47.572090	40.237360	385.307900	.012630
-46.352010	40.011290	385.300000	.004368
-45.145370	39.773560	385.297300	.001320
-43.952510	39.524580	385.296600	.000359
-42.773630	39.264750	385.296700	.000284
-91.793200	40.348750	410.366200	17.253270
-90.591710	40.609810	368.226300	35.001750
-89.375760	40.859430	209.396800	143.067100
-88.145600	41.097210	275.731100	181.803700

-86.901570	41.322720	817.694200	94.469760
-85.644150	41.535550	1580.175000	146.454900
-84.373860	41.735290	2162.090000	219.487000
-83.091290	41.921560	2445.692000	273.760700
-81.797160	42.093980	2713.997000	321.372300
-80.492230	42.252200	3149.543000	355.563000
-79.177290	42.395880	3465.180000	394.860800
-77.853240	42.524730	3395.036000	395.435200
-76.521000	42.638450	2629.009000	287.299900
-75.181550	42.736820	1602.335000	168.000500
-73.835900	42.819590	763.990000	230.201900
-72.485100	42.886590	778.025100	139.921200
-71.130210	42.937670	1184.403000	236.021500
-69.772340	42.972710	1061.394000	155.350100
-68.412590	42.991630	819.461300	117.966000
-67.052090	42.994400	364.365800	161.842500
-65.691960	42.980990	100.939200	137.682000
-64.333320	42.951450	161.012700	95.419080
-62.977290	42.905830	317.600700	26.774600
-61.624970	42.844260	381.851700	9.364305
-60.277440	42.766850	392.230500	5.819741
-58.935750	42.673790	390.809100	5.220187
-57.600940	42.565280	387.820400	4.193285
-56.273990	42.441570	386.704100	2.753981
-54.955840	42.302930	387.064900	1.689484
-53.647390	42.149640	386.815700	1.151333
-52.349470	41.982030	386.311600	.733347
-51.062890	41.800450	385.851400	.414693
-49.788320	41.605260	385.554400	.206263
-48.526440	41.396860	385.400800	.090999
-47.277780	41.175640	385.333600	.035670
-46.042850	40.942000	385.308000	.012430
-44.822010	40.696380	385.299600	.003843
-43.615560	40.439180	385.297100	.001077
-42.423690	40.170830	385.296800	.000415
-92.150770	41.257820	452.507400	19.402580
-90.935390	41.527410	495.865500	40.459080
-89.704910	41.785250	548.823000	184.326400
-88.459540	42.030900	362.415400	224.083900
-87.199620	42.263930	251.379500	237.998400
-85.925610	42.483890	1149.250000	184.516200
-84.638040	42.690380	2071.855000	214.539800
-83.337520	42.882970	2554.462000	283.109500
-82.024790	43.061280	2958.035000	339.381000
-80.700610	43.224930	3438.759000	398.086100
-79.365870	43.373580	3682.624000	436.428900
-78.021450	43.506890	3496.263000	407.642500
-76.668370	43.624570	2862.819000	317.786800
-75.307630	43.726360	1901.717000	191.716200
-73.940300	43.812040	1145.053000	377.959600
-72.567500	43.881390	305.976400	246.525100
-71.190370	43.934270	656.048800	202.519900
-69.810050	43.970550	1217.180000	162.940800
-68.427750	43.990140	1271.953000	137.207700
-67.044650	43.993000	838.139900	116.164600
-65.661930	43.979120	580.088500	67.785820
-64.280810	43.948540	474.820500	50.192290
-62.902460	43.901310	453.866700	28.900200
-61.528050	43.837570	428.613000	20.760850
-60.158730	43.757450	414.103300	16.855410
-58.795620	43.661140	403.726200	13.973200
-57.439790	43.548850	394.531700	11.065220
-56.092310	43.420850	390.736400	7.551099
-54.754150	43.277410	391.597900	4.524802
-53.426270	43.118850	390.566000	3.064202
-52.109530	42.945500	388.524600	1.936984
-50.804760	42.757750	386.959200	1.066689

-49.512690 42.555960 386.037400 .518452
-48.233980 42.340550 385.587500 .224364
-46.969190 42.111940 385.398300 .087034
-45.718810 41.870550 385.328200 .030305
-44.483210 41.616830 385.305100 .009475
-43.262650 41.351200 385.298600 .002713
-42.057300 41.074100 385.297100 .000968
-92.525320 42.164070 441.954500 22.110490
-91.295520 42.442420 491.728900 43.202660
-90.049940 42.708690 576.838000 94.394520
-88.788760 42.962440 443.599100 153.078300
-87.512310 43.203190 370.071800 163.114900
-86.221000 43.430500 1177.495000 235.146000
-84.915410 43.643920 2109.724000 299.739500
-83.596150 43.843030 2655.700000 302.418200
-82.263960 44.027400 3105.514000 366.607200
-80.919660 44.196660 3666.096000 434.973500
-79.564150 44.350410 3782.827000 442.051500
-78.198390 44.488330 3346.706000 372.650600
-76.823410 44.610100 2423.617000 240.740300
-75.440290 44.715440 1356.748000 142.927800
-74.050180 44.804110 440.096800 180.360200
-72.654240 44.875890 186.690000 106.616500
-71.253690 44.930630 586.757400 166.088800
-69.849760 44.968180 1503.607000 188.910000
-68.443720 44.988460 1912.505000 279.543300
-67.036810 44.991420 1440.052000 160.024600
-65.630330 44.977050 1024.360000 115.914600
-64.225530 44.945390 776.138100 85.479400
-62.823690 44.896510 608.261100 57.610950
-61.426040 44.830540 512.468000 40.872310
-60.033810 44.747610 463.933600 33.142520
-58.648170 44.647940 437.754600 27.581220
-57.270290 44.531750 417.822400 21.521260
-55.901260 44.399320 408.741800 16.300460
-54.542130 44.250930 407.112200 11.263740
-53.193900 44.086940 401.055900 7.622165
-51.857480 43.907680 394.383100 4.616703
-50.533710 43.713560 389.681500 2.443647
-49.223370 43.504980 387.154000 1.147820
-47.927120 43.282370 385.999300 .484075
-46.645540 43.046150 385.536100 .184474
-45.379100 42.796800 385.370300 .063628
-44.128160 42.534760 385.316900 .019875
-42.892960 42.260480 385.301500 .005715
-41.673610 41.974410 385.298100 .002074
-92.917770 43.067330 431.724100 19.859420
-91.673000 43.354680 487.920200 36.479980
-90.411740 43.629630 597.292800 67.948780
-89.134110 43.891690 697.050200 329.328000
-87.840450 44.140390 228.600300 226.631400
-86.531140 44.375260 165.947800 219.205400
-85.206750 44.595830 1215.501000 219.480100
-83.867900 44.801650 2159.079000 239.928600
-82.515380 44.992280 2719.224000 317.077300
-81.150000 45.167310 3128.862000 367.747300
-79.772730 45.326340 3246.988000 370.275600
-78.384560 45.469020 2914.048000 309.885700
-76.986600 45.595010 2083.630000 192.654800
-75.579970 45.704020 1101.861000 142.976800
-74.165890 45.795780 289.619600 237.364200
-72.745610 45.870080 76.889500 56.494640
-71.320400 45.926740 398.237500 212.777500
-69.891600 45.965610 1554.897000 206.076700
-68.460530 45.986610 2000.648000 274.448200
-67.028560 45.989670 1463.463000 192.723400
-65.597030 45.974800 1092.929000 131.524000

-64.167300	45.942020	825.567200	98.644230
-62.740720	45.891430	693.807400	68.157910
-61.318600	45.823140	595.817100	52.191200
-59.902260	45.737310	542.282700	46.458010
-58.492950	45.634170	508.612700	43.799610
-57.091900	45.513950	491.912400	36.356340
-55.700260	45.376930	474.859100	30.615180
-54.319140	45.223450	452.958000	24.669210
-52.949590	45.053840	428.627900	17.118760
-51.592580	44.868490	407.896800	9.916513
-50.248970	44.667810	395.479600	4.979943
-48.919560	44.452230	389.357400	2.232785
-47.605040	44.222190	386.760700	.909009
-46.305980	43.978160	385.778400	.337480
-45.022840	43.720610	385.441700	.114569
-43.755960	43.450020	385.336300	.035482
-42.505550	43.166850	385.306400	.010205
-41.271660	42.871580	385.299700	.003681
-93.329110	43.967430	404.076700	14.328370
-92.068780	44.264030	408.968100	21.466280
-90.791210	44.547890	406.137200	38.052510
-89.496510	44.818510	277.481100	102.358200
-88.184940	45.075390	143.163600	133.860700
-86.856880	45.318050	77.005540	104.698300
-85.512890	45.545990	1028.860000	104.311500
-84.153590	45.758730	1899.403000	198.713100
-82.779790	45.955820	2337.710000	263.077200
-81.392350	46.136820	2545.586000	297.154200
-79.992260	46.301310	2577.967000	293.409100
-78.580580	46.448910	2393.722000	253.231300
-77.158460	46.579270	2003.745000	182.972700
-75.727110	46.692070	1516.081000	178.182200
-74.287820	46.787040	823.863900	116.889500
-72.841890	46.863950	159.910600	204.782400
-71.390720	46.922590	453.909800	175.963100
-69.935700	46.962830	1654.509000	377.628900
-68.478260	46.984570	1712.470000	202.255800
-67.019850	46.987740	1358.711000	156.633300
-65.561920	46.972340	1147.570000	116.978800
-64.105920	46.938410	898.297700	104.818100
-62.653260	46.886040	756.299100	74.767970
-61.205390	46.815350	680.644200	64.426410
-59.763680	46.726520	662.318900	68.049180
-58.329470	46.619790	639.224900	68.198560
-56.904060	46.495390	608.058200	64.799500
-55.488670	46.353640	578.694900	59.662560
-54.084500	46.194880	543.605100	49.670250
-52.692630	46.019470	486.813300	33.081120
-51.314070	45.827840	434.948700	18.168610
-49.949730	45.620390	405.854900	8.727365
-48.600430	45.397590	392.882100	3.740116
-47.266860	45.159900	387.871300	1.458939
-45.949600	44.907820	386.108200	.525327
-44.649100	44.641840	385.533500	.174333
-43.365670	44.362450	385.360400	.053313
-42.099450	44.070140	385.312500	.015214
-40.850440	43.765410	385.301400	.005439
-93.760410	44.864160	394.075300	11.856680
-92.483920	45.170280	392.136300	15.115540
-91.189420	45.463320	386.394100	25.936020
-89.876960	45.742750	361.230700	60.680550
-88.546760	46.008070	358.343800	182.901700
-87.199170	46.258760	173.635700	167.064300
-85.834720	46.494300	804.364900	75.898500
-84.454070	46.714200	1537.038000	150.804600
-83.058010	46.917960	1991.403000	203.687700
-81.647480	47.105130	2170.251000	232.375600

-80.223460	47.275260	2144.371000	233.351100
-78.787090	47.427960	1967.049000	194.969000
-77.339580	47.562840	1676.897000	149.084600
-75.882230	47.679570	1406.125000	119.431600
-74.416370	47.777870	1196.617000	113.176200
-72.943440	47.857470	1041.593000	255.721700
-71.464880	47.918190	892.298900	84.233480
-69.982220	47.959850	1228.740000	117.918100
-68.496960	47.982350	1156.050000	126.300100
-67.010670	47.985630	1095.593000	103.865500
-65.524890	47.969690	1136.130000	98.909260
-64.041170	47.934570	1046.512000	92.595160
-62.561040	47.880340	873.339600	75.888270
-61.086020	47.807170	760.969500	66.841650
-59.617590	47.715230	732.700000	70.086470
-58.157170	47.604770	720.997700	77.262590
-56.706150	47.476050	711.963400	84.339300
-55.265830	47.329400	713.605700	86.733920
-53.837470	47.165180	684.691900	77.618550
-52.422200	46.983780	578.373100	51.048240
-51.021090	46.785640	472.184200	26.656120
-49.635090	46.571200	418.505900	12.182550
-48.265020	46.340950	396.756000	5.061260
-46.911610	46.095370	388.933900	1.921975
-45.575410	45.834990	386.392200	.673611
-44.256860	45.560320	385.607600	.219601
-42.956210	45.271870	385.378700	.066154
-41.673580	44.970160	385.316800	.018699
-40.408870	44.655690	385.302700	.006612
-94.212860	45.757310	402.814900	13.859420
-92.919560	46.073230	402.192500	18.063220
-91.607470	46.375730	403.246500	32.593270
-90.276550	46.664250	396.500000	72.406210
-88.926960	46.938270	388.504400	97.316900
-87.559010	47.197250	284.321100	82.459180
-86.173240	47.440650	454.681900	74.757380
-84.770290	47.667940	860.010000	81.163680
-83.350960	47.878600	1473.606000	132.193100
-81.916200	48.072160	1869.246000	189.765300
-80.467090	48.248140	1915.175000	194.343600
-79.004780	48.406110	1603.829000	160.605400
-77.530590	48.545680	1262.076000	124.266700
-76.045850	48.666500	1019.987000	96.115940
-74.552020	48.768250	895.680500	71.287280
-73.050600	48.850660	828.292500	81.602740
-71.543170	48.913520	814.377900	77.844310
-70.031330	48.956650	880.527600	90.426850
-68.516710	48.979950	736.239700	145.666900
-67.000980	48.983350	842.588400	96.682740
-65.485790	48.966840	1010.347000	87.932400
-63.972820	48.930470	1078.280000	105.014500
-62.463700	48.874330	924.149200	89.677240
-60.960050	48.798580	819.697000	68.077730
-59.463460	48.703410	797.104400	64.886150
-57.975440	48.589080	780.257100	72.309270
-56.497490	48.455880	760.807000	85.866020
-55.030970	48.304140	763.478600	87.610870
-53.577210	48.134270	739.943900	82.525270
-52.137430	47.946660	599.954200	53.063310
-50.712720	47.741790	475.615700	28.566140
-49.304080	47.520120	418.805100	12.961380
-47.912350	47.282170	396.786900	5.212298
-46.538250	47.028450	389.002200	1.952696
-45.182340	46.759510	386.403200	.685081
-43.845020	46.475880	385.608100	.222165
-42.526500	46.178100	385.378400	.066698
-41.226800	45.866700	385.316600	.018701

-39.945740	45.542200	385.302500	.006542
-94.687740	46.646660	431.143500	22.261090
-93.376940	46.972690	448.144500	27.549100
-92.046550	47.284930	488.002500	46.252430
-90.696440	47.582840	519.950900	67.470860
-89.326680	47.865840	497.803500	66.011370
-87.937550	48.133390	425.370700	66.411310
-86.529520	48.384910	215.069300	164.059900
-85.103260	48.619850	433.411400	239.546200
-83.659580	48.837660	1275.153000	100.396000
-82.199460	49.037830	1763.086000	190.745200
-80.724000	49.219880	1729.301000	181.501200
-79.234450	49.383340	1397.645000	141.299400
-77.732160	49.527770	1098.740000	105.166300
-76.218590	49.652830	895.434900	80.756440
-74.695260	49.758160	764.749200	68.928360
-73.163800	49.843490	704.079500	70.462410
-71.625870	49.908580	722.287300	69.833720
-70.083210	49.953250	776.275500	78.104760
-68.537570	49.977380	704.390600	97.481220
-66.990740	49.980900	796.328400	76.524730
-65.444490	49.963800	890.176300	85.847020
-63.900620	49.926140	898.355000	86.072510
-62.360880	49.868010	805.819500	92.664670
-60.827010	49.789570	792.943700	75.033560
-59.300730	49.691040	839.544700	78.522860
-57.783640	49.572690	845.981400	81.100850
-56.277320	49.434830	806.946200	83.775780
-54.783270	49.277820	748.090300	79.327780
-53.302860	49.102080	642.206800	65.710780
-51.837360	48.908040	527.208200	41.640950
-50.387950	48.696200	445.079900	22.163460
-48.955640	48.467040	407.892100	10.228190
-47.541310	48.221130	393.208600	4.216572
-46.145660	47.959000	387.880900	1.601558
-44.769230	47.681210	386.107900	.559297
-43.412380	47.388330	385.533300	.182297
-42.075270	47.080920	385.359200	.054644
-40.757810	46.759540	385.311800	.015245
-39.459720	46.424710	385.301100	.005294
-95.186440	47.531940	478.538100	35.569140
-93.857410	47.868410	516.395300	43.166440
-92.508000	48.190720	584.912300	61.226330
-91.137910	48.498310	619.466400	67.533230
-89.747150	48.790600	623.995100	63.290960
-88.335950	49.067010	648.240100	64.769870
-86.904710	49.326930	678.276200	71.087890
-85.454100	49.569790	864.156000	85.288860
-83.984930	49.795020	1286.613000	138.690300
-82.498220	50.002060	1485.617000	163.296800
-80.995120	50.190400	1426.517000	158.986800
-79.476920	50.359550	1222.565000	131.384200
-77.945060	50.509060	1064.023000	107.290300
-76.401090	50.638520	970.386500	96.339860
-74.846650	50.747580	878.150100	75.399710
-73.283450	50.835950	840.633500	69.917470
-71.713320	50.903350	816.967200	66.430790
-70.138080	50.949630	818.530000	71.959400
-68.559640	50.974620	766.436800	80.407680
-66.979900	50.978270	881.422100	67.911090
-65.400800	50.960560	864.923600	71.035320
-63.824260	50.921550	585.010300	135.375900
-62.252170	50.861340	364.101900	186.622800
-60.686400	50.780110	548.309500	81.638400
-59.128770	50.678080	757.929700	75.309650
-57.581030	50.555550	804.730500	83.786590
-56.044860	50.412850	730.997700	75.916560

-54.521820	50.250360	622.721700	61.925090
-53.013420	50.068520	528.697800	43.399400
-51.520960	49.867810	459.155000	26.829110
-50.045680	49.648740	418.602400	14.288930
-48.588630	49.411830	398.699300	6.679515
-47.150700	49.157670	390.113600	2.827775
-45.732590	48.886830	386.936600	1.088862
-44.334810	48.599900	385.818200	.387489
-42.957660	48.297460	385.447700	.126569
-41.601190	47.980120	385.336300	.037777
-40.265250	47.648430	385.306000	.010386
-38.949390	47.302930	385.299300	.003552
-95.710510	48.412890	544.889800	49.266150
-94.362470	48.760130	610.814800	59.222860
-92.993230	49.092850	688.429900	73.369960
-91.602360	49.410460	722.123900	73.962750
-90.189750	49.712350	740.674900	69.434090
-88.755540	49.997940	761.348800	70.470920
-87.300090	50.266570	799.908400	77.337400
-85.824040	50.517650	872.262900	84.915370
-84.328210	50.750570	1013.466000	112.592600
-82.813610	50.964740	1105.993000	138.641900
-81.281460	51.159630	1124.334000	133.343300
-79.733130	51.334700	1023.786000	119.082400
-78.170130	51.489480	914.745400	104.539100
-76.594090	51.623530	866.011900	89.485440
-75.006800	51.736490	854.485200	82.612260
-73.410070	51.828010	875.077000	80.808220
-71.805870	51.897850	862.933600	77.995640
-70.196170	51.945790	832.565200	75.330060
-68.582990	51.971680	804.509000	77.715630
-66.968440	51.975460	941.205600	85.644390
-65.354560	51.957120	973.389000	95.596570
-63.743440	51.916690	801.502200	81.599850
-62.137130	51.854320	572.152300	69.345030
-60.537630	51.770170	582.059400	71.998660
-58.946900	51.664510	695.283300	71.444440
-57.366820	51.537620	679.520300	69.561120
-55.799180	51.389870	592.186200	56.696410
-54.245650	51.221680	511.502700	40.110440
-52.707820	51.033510	455.323900	26.482060
-51.187080	50.825860	421.712200	15.578610
-49.684720	50.599280	402.266400	8.175293
-48.201800	50.354330	392.331000	3.878236
-46.739240	50.091630	387.942700	1.669144
-45.297720	49.811770	386.211600	.653940
-43.877700	49.515370	385.585200	.233304
-42.479410	49.203060	385.379500	.075893
-41.102810	48.875440	385.317800	.022293
-39.747590	48.533100	385.301400	.005988
-38.413150	48.176570	385.297700	.001985
-96.261670	49.289190	612.793000	57.362750
-94.893750	49.647600	705.717000	70.734470
-93.503820	49.991080	761.578400	77.593080
-92.091320	50.319060	744.744600	70.852200
-90.655950	50.630910	699.367400	61.583980
-89.197760	50.925990	662.719900	58.319250
-87.717060	51.203660	660.697000	60.678490
-86.214420	51.463270	700.642000	70.541740
-84.690660	51.704190	806.101400	88.507900
-83.146820	51.925780	919.785100	105.715200
-81.584160	52.127480	987.612400	116.321600
-80.004110	52.308710	910.056400	105.800000
-78.408280	52.468990	799.181900	97.061080
-76.798410	52.607840	767.511100	83.467220
-75.176390	52.724850	802.991300	78.146190
-73.544200	52.819690	847.759500	78.417450

-71.903920	52.892050	829.549100	73.466170
-70.257710	52.941730	763.234300	65.681990
-68.607750	52.968570	703.359900	80.103510
-66.956280	52.972490	851.099900	73.294370
-65.305560	52.953480	927.369500	93.590820
-63.657820	52.911580	882.035200	91.695280
-62.015270	52.846950	771.194000	83.194950
-60.380080	52.759750	672.070300	66.740210
-58.754360	52.650280	626.497400	62.162420
-57.140120	52.518850	557.050700	50.799110
-55.539290	52.365840	491.390600	36.056080
-53.953660	52.191710	446.100000	24.016560
-52.384890	51.996940	418.607700	14.713090
-50.834480	51.782070	402.298800	8.351521
-49.303730	51.547700	393.217000	4.346644
-47.793770	51.294400	388.641700	2.066653
-46.305490	51.022820	386.575500	.890530
-44.839570	50.733620	385.739300	.347588
-43.396390	50.427420	385.435600	.123406
-41.976090	50.104880	385.335600	.039465
-40.578500	49.766630	385.306100	.011316
-39.203150	49.413260	385.298400	.002919
-37.849210	49.045330	385.296900	.000934
-96.841810	50.160530	602.015100	50.935050
-95.453030	50.530490	670.576800	62.140480
-94.041510	50.885130	656.120800	57.030380
-92.606450	51.223850	607.757900	51.579630
-91.147380	51.546020	562.885400	44.478970
-89.664200	51.850960	533.465900	40.772990
-88.157130	52.138020	530.441400	41.879170
-86.626690	52.406500	564.268700	48.648080
-85.073680	52.655720	651.740700	60.270190
-83.499180	52.885050	774.747400	86.949590
-81.904440	53.093850	818.079300	89.874750
-80.290990	53.281520	812.284100	97.117580
-78.660530	53.447530	742.016500	90.952230
-77.014920	53.591380	698.627200	80.122160
-75.356160	53.712650	714.185500	73.105830
-73.686420	53.810940	733.861800	67.441880
-72.007930	53.885960	683.928600	56.708260
-70.322990	53.937460	561.350500	65.378080
-68.634010	53.965280	499.092100	115.460800
-66.943400	53.969350	695.782700	72.484090
-65.253590	53.949630	823.860200	78.673870
-63.567000	53.906200	809.100500	85.966480
-61.886040	53.839190	725.250600	74.512640
-60.213040	53.748820	617.040000	60.583140
-58.550290	53.635360	534.058700	46.471510
-56.899960	53.499180	474.610900	32.396260
-55.264110	53.340690	436.509900	21.332820
-53.644650	53.160350	413.767600	13.145340
-52.043340	52.958710	400.362400	7.682949
-50.461760	52.736320	392.778100	4.220126
-48.901280	52.493820	388.736700	2.153790
-47.363030	52.231850	386.742400	1.005866
-45.847900	51.951060	385.846800	.427167
-44.356530	51.652150	385.485900	.165003
-42.889220	51.335790	385.354900	.057414
-41.445980	51.002650	385.312400	.017876
-40.026490	50.653380	385.300100	.004954
-38.630060	50.288590	385.297100	.001213
-37.255620	49.908830	385.296500	.000360
-97.453060	51.026530	513.374200	35.914860
-96.042340	51.408490	532.066000	36.802050
-94.608220	51.774700	517.998700	35.368720
-93.149600	52.124570	490.113300	31.223790
-91.665800	52.457450	467.553200	26.976850

-90.156560	52.772640	454.227800	24.326190
-88.621980	53.069450	453.105500	24.492530
-87.062480	53.347160	466.782700	28.376060
-85.478850	53.605050	497.223100	36.646920
-83.872130	53.842430	519.531900	52.916160
-82.243660	54.058630	510.328100	75.248470
-80.595020	54.253030	578.467100	75.431840
-78.928000	54.425040	641.441200	90.482640
-77.244600	54.574130	640.392600	84.540630
-75.546970	54.699840	654.977300	72.693660
-73.837420	54.801750	687.952900	62.819820
-72.118360	54.879540	648.105700	50.970320
-70.392330	54.932960	483.155900	71.766420
-68.661900	54.961820	415.759200	123.409700
-66.929700	54.966030	624.958200	58.689010
-65.198370	54.945580	732.532500	66.995300
-63.470540	54.900540	706.813600	69.546440
-61.748820	54.831050	614.353000	60.108150
-60.035740	54.737340	525.477800	44.289290
-58.333780	54.619720	465.321900	30.028800
-56.645260	54.478570	429.588900	19.248520
-54.972420	54.314330	409.475100	11.684380
-53.317280	54.127510	398.089600	6.809341
-51.681740	53.918690	391.776900	3.795029
-50.067420	53.688470	388.403700	2.003321
-48.475770	53.437500	386.686300	.985769
-46.907930	53.166490	385.867300	.447178
-45.364770	52.876130	385.510000	.185794
-43.846840	52.567130	385.368300	.069830
-42.354370	52.240220	385.318000	.023550
-40.887210	51.896080	385.302000	.007052
-39.444820	51.535370	385.297500	.001859
-38.026250	51.158730	385.296500	.000407
-36.630120	50.766700	385.296300	.000121
-98.097820	51.886770	444.575300	21.046220
-96.663910	52.281200	447.078700	20.370860
-95.206040	52.659450	439.834800	19.062180
-93.722790	53.020900	429.360100	16.653200
-92.213200	53.364910	420.342900	14.166520
-90.676770	53.690770	414.682500	12.542030
-89.113440	53.997730	413.382400	12.241770
-87.523560	54.285050	415.334000	13.873670
-85.907830	54.551970	411.252100	21.721580
-84.267300	54.797760	375.849000	49.658910
-82.603340	55.021710	295.427800	96.908960
-80.917590	55.223140	305.047600	105.709500
-79.211960	55.401440	503.912000	97.636150
-77.488560	55.556020	609.086100	89.211440
-75.749730	55.686380	637.216100	79.559560
-73.997920	55.792100	672.567000	62.144450
-72.235800	55.872810	647.278900	51.388950
-70.466090	55.928230	485.743900	77.035180
-68.691570	55.958180	450.440500	110.997100
-66.915130	55.962550	645.191200	57.783130
-65.139640	55.941330	688.053600	61.682980
-63.367970	55.894590	610.806400	57.344070
-61.602940	55.822490	524.180500	43.086770
-59.847310	55.725280	462.609100	29.220590
-58.103760	55.603290	426.717200	18.276670
-56.374820	55.456930	407.009800	10.790820
-54.662870	55.286670	396.425300	6.133794
-52.970100	55.093080	390.840400	3.365831
-51.298500	54.876750	387.959000	1.774748
-49.649800	54.638340	386.514100	.888916
-48.025460	54.378550	385.818500	.418098
-46.426660	54.098120	385.503500	.182690
-44.854210	53.797780	385.371200	.072985

-43.308580	53.478290	385.320600	.026382
-41.789850	53.140400	385.303200	.008511
-40.297680	52.784830	385.297900	.002413
-38.831280	52.412250	385.296600	.000585
-37.389370	52.023280	385.296300	.000116
-35.970190	51.618480	385.296300	.000056
-98.778840	52.740810	410.475200	10.698600
-97.320270	53.148240	409.181700	10.044770
-95.837370	53.539010	406.507500	9.115258
-94.328290	53.912530	402.666000	7.858055
-92.791720	54.268120	399.106900	6.630216
-91.226900	54.605080	396.617800	5.721150
-89.633560	54.922620	395.121100	5.279833
-88.011890	55.219970	392.567700	6.489627
-86.362530	55.496330	382.171500	13.954280
-84.686490	55.750910	346.559500	33.502990
-82.985150	55.982970	252.809400	75.151710
-81.260250	56.191770	131.526700	133.360300
-79.513790	56.376650	278.892300	97.983090
-77.748020	56.536990	558.867200	101.520500
-75.965460	56.672260	639.475300	85.844220
-74.168790	56.781970	666.353000	63.422590
-72.360860	56.865740	656.778500	49.727850
-70.544630	56.923280	552.499400	65.154800
-68.723180	56.954370	591.921300	55.756770
-66.899620	56.958910	690.043000	67.155280
-65.077100	56.936880	626.700600	57.471650
-63.258730	56.888360	539.209200	44.363680
-61.447630	56.813510	472.006700	31.194430
-59.646790	56.712620	430.520100	19.194050
-57.859090	56.586040	407.929800	11.080000
-56.087300	56.434210	396.238000	6.056536
-54.333970	56.257640	390.459300	3.183786
-52.601470	56.056940	387.676500	1.620854
-50.891880	55.832740	386.360200	.795323
-49.207030	55.585770	385.751800	.373452
-47.548420	55.316760	385.480700	.165950
-45.917200	55.026500	385.365500	.068577
-44.314130	54.715760	385.320000	.025999
-42.739580	54.385350	385.303500	.008902
-41.193420	54.036030	385.298200	.002708
-39.675070	53.668560	385.296700	.000711
-38.183400	53.283620	385.296300	.000138
-36.716770	52.881820	385.296200	.000070
-35.272900	52.463700	385.296300	.000053
-99.499240	53.588130	395.211200	4.918768
-98.014280	54.009130	394.203400	4.468263
-96.504860	54.412960	393.064000	3.975051
-94.968610	54.799050	391.642400	3.384769
-93.403790	55.166730	390.271600	2.794936
-91.809290	55.515250	389.104400	2.253332
-90.184560	55.843850	387.709600	1.924031
-88.529620	56.151670	384.160500	3.742367
-86.844990	56.437890	373.266800	10.631580
-85.131650	56.701690	346.249900	23.231730
-83.390960	56.942230	278.471200	47.220080
-81.624720	57.158780	152.353200	81.701160
-79.835050	57.350580	128.197500	131.314900
-78.024370	57.516980	406.876300	70.657470
-76.195360	57.657400	619.671300	88.342030
-74.350940	57.771310	665.593000	68.254890
-72.494220	57.858320	690.312700	54.360090
-70.628410	57.918090	670.514800	48.078660
-68.756890	57.950390	686.920400	56.243410
-66.883060	57.955100	668.492200	63.896060
-65.010370	57.932220	568.305200	50.215740
-63.142240	57.881810	497.129200	36.809990

-61.282040 57.804080 449.881100 24.738240
-59.433060 57.699310 417.726200 14.584010
-57.598450 57.567890 399.840400 7.588484
-55.781200 57.410300 391.661600 3.743609
-53.984060 57.227100 387.975500 1.768738
-52.209560 57.018940 386.397500 .808898
-50.459930 56.786500 385.738400 .359876
-48.737060 56.530560 385.468100 .154522
-47.042490 56.251900 385.359600 .063139
-45.377330 55.951360 385.318200 .024188
-43.742270 55.629770 385.303100 .008514
-42.137470 55.287960 385.298200 .002719
-40.562610 54.926740 385.296600 .000760
-39.016750 54.546860 385.296300 .000161
-37.498410 54.149020 385.296200 .000034
-36.005400 53.733830 385.296200 .000062
-34.534880 53.301770 385.296300 .000043
-100.262700 54.428120 388.925000 2.085445
-98.749250 54.863350 388.420000 1.838292
-97.211560 55.280850 387.953900 1.594318
-95.646590 55.680060 387.434900 1.327518
-94.052090 56.060360 386.898100 1.034037
-92.426510 56.420970 386.272300 .696115
-90.768940 56.761100 385.057400 .938252
-89.079190 57.079890 381.624700 3.284752
-87.357570 57.376450 372.061300 8.891421
-85.605000 57.649890 350.766200 17.849230
-83.822840 57.899360 307.196400 34.115970
-82.012920 58.124030 220.981200 54.008140
-80.177480 58.323120 193.241600 67.113010
-78.319110 58.495910 276.622700 103.214900
-76.440710 58.641750 542.518900 69.519260
-74.545430 58.760110 677.134500 78.114430
-72.636660 58.850530 764.996300 66.419620
-70.717930 58.912650 751.995200 61.752740
-68.792920 58.946230 704.515900 63.938440
-66.865380 58.951130 631.070800 61.746080
-64.939080 58.927340 526.993700 44.811760
-63.017780 58.874950 468.898700 29.804490
-61.105210 58.794160 442.902200 23.548960
-59.204930 58.685300 415.341600 13.835820
-57.320400 58.548780 398.289200 6.687885
-55.454840 58.385130 390.159300 2.881542
-53.611270 58.194950 387.086800 1.187796
-51.792370 57.978940 385.933000 .472821
-50.000500 57.737840 385.516900 .183360
-48.237630 57.472490 385.370700 .069170
-46.505310 57.183730 385.320300 .025090
-44.804610 56.872430 385.303400 .008610
-43.136050 56.539500 385.298200 .002737
-41.499610 56.185780 385.296700 .000781
-39.894630 55.812120 385.296200 .000183
-38.319800 55.419290 385.296200 .000058
-36.773090 55.007980 385.296200 .000052
-35.251730 54.578750 385.296200 .000043
-33.752120 54.132060 385.296300 .000052
-101.073700 55.260130 386.532200 .816432
-99.529010 55.710320 386.317500 .695176
-97.960900 56.142140 386.138500 .584809
-96.365430 56.555100 385.949100 .463661
-94.739640 56.948590 385.717200 .305783
-93.081440 57.321860 385.310700 .218309
-91.389490 57.674070 384.236900 1.034002
-89.663250 58.004340 381.079800 3.262271
-87.902820 58.311730 372.763500 8.094939
-86.109000 58.595320 355.029900 16.885850
-84.283100 58.854170 321.164700 35.010140

-82.427000	59.087400	266.137200	59.659630
-80.543050	59.294160	267.089100	36.925810
-78.634010	59.473690	313.133000	53.729180
-76.703000	59.625280	503.749900	58.948410
-74.753460	59.748340	716.563600	76.742430
-72.789070	59.842370	806.360500	83.171660
-70.813740	59.906970	776.419300	72.882840
-68.831480	59.941910	711.910200	67.494820
-66.846440	59.947010	613.421300	59.871220
-64.862770	59.922250	505.116600	39.191220
-62.884610	59.867760	446.670100	23.083850
-60.916040	59.783740	426.750600	16.908820
-58.961010	59.670550	413.586400	13.270500
-57.023270	59.528640	398.140700	6.373544
-55.106350	59.358580	389.858500	2.507394
-53.213500	59.161040	386.724600	.906783
-51.347600	58.936760	385.741600	.320935
-49.511150	58.686560	385.432700	.110346
-47.706190	58.411320	385.336700	.036716
-45.934240	58.111950	385.307600	.011736
-44.196280	57.789400	385.299200	.003565
-42.492650	57.444580	385.296800	.000985
-40.823030	57.078400	385.296300	.000235
-39.186360	56.691730	385.296100	.000056
-37.580830	56.285340	385.296200	.000060
-36.003750	55.859910	385.296200	.000053
-34.451600	55.415950	385.296200	.000041
-32.919830	54.953830	385.296300	.000042
-101.937200	56.083360	385.686000	.293335
-100.358100	56.549350	385.604600	.240491
-98.756940	56.996240	385.539600	.192978
-97.128790	57.423640	385.465700	.134792
-95.469860	57.830970	385.339700	.061123
-93.777320	58.217490	385.015800	.306532
-92.049300	58.582370	384.030200	1.126075
-90.284800	58.924680	381.131700	3.170072
-88.483640	59.243460	373.778200	7.624119
-86.646400	59.537720	358.953700	16.746840
-84.774360	59.806450	331.470300	38.186910
-82.869400	60.048700	294.987300	65.793940
-80.933970	60.263580	331.472400	26.920420
-78.971000	60.450230	390.401200	28.929880
-76.983890	60.607900	532.750400	44.477480
-74.976370	60.735940	702.900800	71.940930
-72.952470	60.833800	788.177400	88.880680
-70.916480	60.901050	786.540300	80.159900
-68.872850	60.937420	736.096000	70.706210
-66.826130	60.942730	611.326400	56.622610
-64.780930	60.916960	490.620100	34.773260
-62.741830	60.860230	423.922200	18.385540
-60.713310	60.772790	399.422800	8.543898
-58.699720	60.654990	395.025900	4.560544
-56.705200	60.507380	391.058300	2.740806
-54.733590	60.330550	387.578500	1.242346
-52.788400	60.125220	386.066400	.489450
-50.872730	59.892220	385.540300	.177741
-48.989200	59.632420	385.370500	.060393
-47.139900	59.346780	385.317700	.019446
-45.326320	59.036270	385.302000	.005927
-43.549270	58.701890	385.297600	.001672
-41.808860	58.344600	385.296400	.000422
-40.104370	57.965370	385.296200	.000089
-38.434220	57.565060	385.296100	.000049
-36.795950	57.144450	385.296100	.000052
-35.186060	56.704150	385.296200	.000057
-33.600030	56.244660	385.296200	.000034
-32.032190	55.766200	385.296300	.000045

-102.859900 56.896880 385.409400 .096390
-101.242000 57.379640 385.381300 .075280
-99.604390 57.842470 385.357500 .054565
-97.940930 58.285070 385.321500 .025332
-96.246660 58.706940 385.229700 .082860
-94.517820 59.107400 384.938600 .353670
-92.751850 59.485580 384.018400 1.108552
-90.947170 59.840560 381.384500 2.944643
-89.103220 60.171320 375.025800 6.918351
-87.220290 60.476800 363.124300 15.658880
-85.299550 60.755960 344.133500 35.342580
-83.342830 61.007760 328.517700 56.264720
-81.352710 61.231220 385.143600 26.886590
-79.332320 61.425420 457.723400 25.755670
-77.285290 61.589540 549.813200 43.474670
-75.215710 61.722870 624.073700 56.815800
-73.128000 61.824790 707.545100 74.990390
-71.026890 61.894870 782.978700 86.338220
-68.917300 61.932760 770.437400 77.294580
-66.804310 61.938290 616.939500 53.305730
-64.692990 61.911450 475.924300 32.521030
-62.588420 61.852340 400.504300 20.403470
-60.495590 61.761240 373.854000 15.936740
-58.419300 61.638580 377.109200 7.688848
-56.364090 61.484900 383.260600 2.291343
-54.334160 61.300890 385.146100 .499956
-52.333320 61.087320 385.385500 .135110
-50.364880 60.845090 385.349700 .052755
-48.431600 60.575160 385.317400 .020309
-46.535590 60.278550 385.303300 .007145
-44.678250 59.956310 385.298200 .002280
-42.860180 59.609490 385.296700 .000654
-41.081100 59.239120 385.296200 .000148
-39.339830 58.846180 385.296100 .000047
-37.634110 58.431540 385.296100 .000046
-35.960610 57.995940 385.296200 .000058
-34.314830 57.539980 385.296200 .000053
-32.690990 57.064010 385.296200 .000061
-31.082000 56.568130 385.296300 .000029
-103.849600 57.699570 385.326100 .028721
-102.187300 58.200280 385.317000 .020614
-100.509000 58.680030 385.306800 .010964
-98.806840 59.138710 385.282800 .019677
-97.074520 59.575940 385.204200 .099005
-95.307120 59.991050 384.938800 .341229
-93.501110 60.383240 384.111300 .997446
-91.654170 60.751550 381.848100 2.531839
-89.765210 61.094930 376.728200 5.837366
-87.834170 61.412270 368.451300 13.278510
-85.861950 61.702450 359.597200 30.415430
-83.850420 61.964360 375.341000 40.198440
-81.802150 62.196920 439.094500 27.105990
-79.720490 62.399150 495.671600 35.964100
-77.609360 62.570130 506.930100 32.012600
-75.473210 62.709080 517.373000 33.200840
-73.316960 62.815350 580.707000 51.351280
-71.145790 62.888430 698.796900 73.743970
-68.965200 62.927950 762.543300 91.197170
-66.780790 62.933720 601.778400 48.850740
-64.598260 62.905720 461.072300 29.237730
-62.423240 62.844070 397.800600 17.784790
-60.261290 62.749080 380.501400 11.184450
-58.117710 62.621230 380.699700 5.242993
-55.997520 62.461110 383.605100 1.787082
-53.905320 62.269450 384.839700 .514583
-51.845240 62.047150 385.183400 .134225
-49.820820 61.795150 385.272300 .030726

-47.834930 61.514500 385.292000 .006486
-45.889670 61.206310 385.295600 .001360
-43.986290 60.871700 385.296200 .000333
-42.125070 60.511780 385.296200 .000089
-40.305270 60.127630 385.296100 .000051
-38.525020 59.720250 385.296100 .000051
-36.781200 59.290500 385.296200 .000056
-35.069400 58.839090 385.296200 .000052
-33.383770 58.366500 385.296200 .000048
-31.716990 57.872960 385.296300 .000039
-30.060130 57.358350 385.296300 .000043
-104.916500 58.490070 385.303400 .007568
-103.202300 59.010120 385.300200 .004386
-101.477600 59.507980 385.294500 .003929
-99.732440 59.983750 385.275500 .024316
-97.958720 60.437220 385.208300 .091770
-96.150010 60.867820 384.983200 .292152
-94.301540 61.274790 384.312900 .810197
-92.410060 61.657170 382.625700 1.962243
-90.473700 62.013880 379.297800 4.456212
-88.491920 62.343760 375.246000 11.384540
-86.465330 62.645610 379.348500 25.380630
-84.395630 62.918240 411.512100 29.826080
-82.285480 63.160470 458.966300 34.709920
-80.138370 63.371240 429.050400 30.603660
-77.958560 63.549540 375.450900 43.218730
-75.750930 63.694510 357.940500 55.187210
-73.520860 63.805420 411.677200 50.343190
-71.274150 63.881700 522.718000 52.543780
-69.016910 63.922960 614.819800 57.314880
-66.755400 63.928990 531.113100 35.771990
-64.495980 63.899750 443.119500 20.282010
-62.244980 63.835380 401.615500 10.444480
-60.008560 63.736250 387.972500 5.025712
-57.792640 63.602840 384.932500 2.287697
-55.602750 63.435840 384.811800 .927089
-53.443960 63.236060 385.055000 .324948
-51.320750 63.004450 385.205000 .101856
-49.236890 62.742080 385.265700 .030141
-47.195330 62.450080 385.286800 .008620
-45.198120 62.129630 385.293600 .002359
-43.246250 61.781950 385.295500 .000646
-41.339580 61.408210 385.296100 .000213
-39.476710 61.009530 385.296200 .000097
-37.654880 60.586900 385.296200 .000072
-35.869840 60.141170 385.296200 .000063
-34.115770 59.672970 385.296200 .000062
-32.385130 59.182660 385.296300 .000039
-30.668580 58.670220 385.296300 .000054
-28.954870 58.135290 385.296400 .000087
-106.073800 59.266680 385.297700 .001607
-104.297500 59.807790 385.296400 .000738
-102.518800 60.325180 385.292300 .005253
-100.725000 60.819250 385.277200 .021867
-98.905530 61.289990 385.223800 .075035
-97.052100 61.737010 385.052900 .225826
-95.158340 62.159620 384.576400 .594593
-93.219700 62.556880 383.484800 1.364226
-91.233350 62.927700 381.768000 3.202072
-89.198040 63.270870 381.842800 8.773472
-87.113920 63.585110 390.108400 16.261250
-84.982480 63.869130 415.046400 26.572220
-82.806390 64.121680 383.114500 32.153560
-80.589290 64.341560 290.504100 80.209800
-78.335770 64.527670 216.834400 119.744900
-76.051160 64.679080 236.328300 105.337500
-73.741460 64.794950 319.517800 61.105420

-71.413090 64.874680 412.255200 33.384590
-69.072890 64.917820 473.615900 26.786090
-66.727910 64.924110 451.587500 19.805900
-64.385280 64.893550 417.013700 11.982810
-62.052090 64.826280 397.327800 5.948223
-59.735260 64.722690 388.951100 2.641622
-57.441380 64.583340 386.053700 1.120562
-55.176610 64.408980 385.336900 .439901
-52.946510 64.200520 385.236800 .160973
-50.755950 63.959000 385.255400 .056546
-48.608900 63.685590 385.277800 .019512
-46.508400 63.381530 385.289500 .006536
-44.456330 63.048090 385.294100 .002101
-42.453320 62.686580 385.295700 .000662
-40.498620 62.298210 385.296100 .000229
-38.589950 61.884150 385.296200 .000091
-36.723370 61.445380 385.296300 .000078
-34.893150 60.982680 385.296300 .000066
-33.091610 60.496560 385.296200 .000071
-31.309040 59.987160 385.296300 .000031
-29.533510 59.454220 385.296500 .000143
-27.750760 58.896990 385.296600 .000244
-107.339200 60.027230 385.296600 .000288
-105.487000 60.591610 385.295600 .001128
-103.643800 61.130270 385.292400 .005088
-101.793500 61.644070 385.280200 .018493
-99.922550 62.133320 385.239100 .059361
-98.020040 62.597820 385.114300 .169521
-96.077510 63.037030 384.785600 .421362
-94.088710 63.450070 384.081500 .897804
-92.049460 63.835860 383.085800 1.733690
-89.957580 64.193130 381.634000 3.464911
-87.812570 64.520550 379.670500 4.872659
-85.615560 64.816710 361.721800 22.643130
-83.369120 65.080260 319.509700 50.906890
-81.077050 65.309890 280.197600 71.471830
-78.744270 65.504390 278.395300 71.881470
-76.376630 65.662700 304.771100 57.488140
-73.980770 65.783920 348.019900 32.914430
-71.563890 65.867360 388.947100 15.832640
-69.133670 65.912500 414.550800 11.615460
-66.698060 65.919100 411.067700 9.890431
-64.265090 65.887100 400.306800 6.563563
-61.842790 65.816700 392.132500 3.556324
-59.438900 65.708320 387.845200 1.660061
-57.060850 65.562580 386.065200 .681997
-54.715470 65.380330 385.466500 .250448
-52.408880 65.162570 385.310000 .085030
-50.146370 64.910470 385.285000 .028778
-47.932120 64.625290 385.288000 .010390
-45.769150 64.308400 385.292600 .003887
-43.659080 63.961160 385.295000 .001395
-41.602020 63.584970 385.296000 .000474
-39.596350 63.181100 385.296300 .000179
-37.638600 62.750720 385.296200 .000079
-35.723270 62.294770 385.296300 .000049
-33.842640 61.813940 385.296400 .000050
-31.986660 61.308540 385.296400 .000046
-30.142740 60.778430 385.296300 .000031
-28.295590 60.222970 385.296600 .000244
-26.427080 59.640870 385.296600 .000244
-108.736700 60.768890 385.296100 .000433
-106.789100 61.359350 385.295300 .001468
-104.866900 61.921510 385.292000 .005466
-102.949500 62.456830 385.280000 .018323
-101.019200 62.966030 385.241900 .055350
-99.061880 63.449270 385.133600 .148540

-97.066180	63.906190	384.864100	.346354
-95.023590	64.336020	384.299300	.689645
-92.928180	64.737730	383.241100	1.178730
-90.776390	65.110020	381.707600	2.115507
-88.566850	65.451470	375.914500	6.380887
-86.300130	65.760610	361.728100	18.051120
-83.978550	66.035940	337.946200	33.404990
-81.606060	66.276030	325.609500	42.085560
-79.187900	66.479550	332.690000	39.540650
-76.730480	66.645290	348.224800	29.990690
-74.241170	66.772270	367.686100	17.656550
-71.728080	66.859700	385.876700	8.815073
-69.199870	66.907030	397.079500	6.509837
-66.665550	66.913940	397.794300	5.893563
-64.134220	66.880400	393.751300	4.289949
-61.614990	66.806620	389.720700	2.544329
-59.116610	66.693070	387.205800	1.281095
-56.647430	66.540450	385.980400	.554774
-54.215080	66.349720	385.493400	.208902
-51.826330	66.121990	385.336600	.069097
-49.486860	65.858530	385.298100	.020974
-47.201050	65.560780	385.292900	.006830
-44.971820	65.230190	385.294100	.002660
-42.800360	64.868270	385.295500	.001038
-40.685980	64.476460	385.296100	.000372
-38.625890	64.056100	385.296300	.000133
-36.614990	63.608330	385.296300	.000064
-34.645670	63.134020	385.296400	.000046
-32.707610	62.633670	385.296400	.000045
-30.787590	62.107330	385.296500	.000143
-28.869240	61.554470	385.296500	.000136
-26.932890	60.973880	385.296600	.000244
-24.955330	60.363580	385.296600	.000244

Q.19a

-89.453400	32.991660	407.877200	2.061248
-88.345930	33.191160	408.167900	2.294090
-87.228100	33.381660	409.221600	3.133546
-86.100230	33.562890	410.840400	4.404743
-84.962690	33.734530	412.750300	5.877912
-83.815900	33.896310	414.621200	7.298935
-82.660300	34.047950	416.150800	8.427073
-81.496410	34.189190	417.066600	9.040781
-80.324730	34.319790	417.004400	8.879262
-79.145840	34.439500	415.484300	7.701719

-77.960310	34.548100	412.248200	5.522254
-76.768760	34.645400	407.855700	3.065559
-75.571840	34.731200	404.145900	1.949196
-74.370180	34.805370	402.927600	1.954158
-73.164480	34.867740	404.033600	1.459243
-71.955430	34.918200	405.940100	1.168998
-70.743730	34.956650	407.379300	1.556961
-69.530080	34.983020	407.973900	1.834603
-68.315220	34.997260	407.882000	1.731725
-67.099880	34.999340	407.420300	1.366304
-65.884770	34.989250	406.868400	.913194
-64.670640	34.967020	406.414400	.519547
-63.458190	34.932690	406.126000	.251741
-62.248150	34.886320	405.979300	.105193
-61.041230	34.828000	405.918700	.039263
-59.838120	34.757850	405.898800	.015258
-58.639500	34.676010	405.893900	.008231
-57.446040	34.582610	405.892300	.006039
-56.258390	34.477850	405.890700	.004294
-55.077160	34.361910	405.888700	.002418
-53.902950	34.235010	405.887300	.001029
-52.736340	34.097380	405.886600	.000277
-51.577860	33.949260	405.886500	.000203
-50.428010	33.790910	405.886400	.000161
-49.287250	33.622600	405.886400	.000191
-48.156040	33.444620	405.886400	.000185
-47.034740	33.257250	405.886500	.000201
-45.923690	33.060790	405.886400	.000187
-44.823220	32.855560	405.886500	.000244
-89.701210	33.918400	409.529700	3.345965
-88.583520	34.125020	410.265600	3.855125
-87.455040	34.322360	412.470900	5.352738
-86.316120	34.510110	415.796500	7.559072
-85.167100	34.687960	419.688600	10.081910
-84.008420	34.855620	423.442100	12.471940
-82.840550	35.012790	426.465500	14.343890
-81.663990	35.159200	428.325200	15.360560
-80.479300	35.294580	428.401600	15.139120
-79.287040	35.418700	425.664500	13.205320
-78.087840	35.531310	419.300700	9.486710
-76.882320	35.632210	410.036500	5.083216
-75.671150	35.721200	402.079100	3.181532
-74.455030	35.798110	399.967800	3.024834
-73.234660	35.862810	403.022900	2.319859
-72.010770	35.915150	407.126000	2.487590
-70.784100	35.955040	409.906300	3.425448
-69.555380	35.982390	410.785400	3.753296
-68.325390	35.997170	410.314500	3.395993
-67.094890	35.999320	409.166400	2.589082
-65.864640	35.988860	407.899700	1.667852
-64.635410	35.965800	406.908100	.901619
-63.407950	35.930180	406.317100	.410733
-62.183030	35.882080	406.038800	.158636
-60.961370	35.821590	405.935700	.055854
-59.743720	35.748840	405.907100	.023066
-58.530780	35.663950	405.902500	.015491
-57.323260	35.567100	405.900700	.012956
-56.121820	35.458470	405.896400	.008974
-54.927120	35.338260	405.891400	.004662
-53.739770	35.206700	405.888000	.001520
-52.560380	35.064020	405.886600	.000212
-51.389480	34.910500	405.886200	.000340
-50.227610	34.746390	405.886100	.000307
-49.075240	34.571990	405.886300	.000231
-47.932830	34.387580	405.886400	.000197
-46.800740	34.193490	405.886400	.000185
-45.679330	33.990000	405.886400	.000161

-44.568900	33.777440	405.886500	.000248
-89.960660	34.843320	414.457900	6.787843
-88.832330	35.057210	416.607700	8.035673
-87.692770	35.261540	422.340600	11.228890
-86.542320	35.455950	430.897200	15.840320
-85.381340	35.640140	440.871900	21.060120
-84.210270	35.813810	450.301200	25.905540
-83.029580	35.976620	457.697700	29.580290
-81.839790	36.128310	462.326300	31.581140
-80.641480	36.268600	463.078500	31.248380
-79.435230	36.397220	457.143000	27.543570
-78.221690	36.513940	441.850000	19.992410
-77.001530	36.618520	416.961800	10.555260
-75.775430	36.710780	395.872000	6.487974
-74.544130	36.790520	392.207600	6.477095
-73.308360	36.857590	401.654800	5.077939
-72.068890	36.911860	412.565600	7.399323
-70.826490	36.953220	418.686600	9.006656
-69.581950	36.981590	420.033700	9.312559
-68.336070	36.996900	418.133800	8.105273
-67.089650	36.999130	414.704800	6.022276
-65.843500	36.988290	411.068500	3.729568
-64.598400	36.964370	408.342700	1.903307
-63.355190	36.927440	406.827300	.798603
-62.114630	36.877570	406.182200	.283060
-60.877510	36.814860	405.974800	.095826
-59.644590	36.739430	405.933600	.047859
-58.416640	36.651430	405.934400	.041328
-57.194370	36.551040	405.931500	.036353
-55.978490	36.438440	405.916700	.024555
-54.769680	36.313860	405.899400	.010744
-53.568590	36.177530	405.889000	.002458
-52.375820	36.029710	405.885300	.001050
-51.191970	35.870660	405.884900	.001404
-50.017550	35.700660	405.885300	.000992
-48.853080	35.520030	405.885800	.000513
-47.698980	35.329070	405.886300	.000298
-46.555660	35.128100	405.886400	.000175
-45.423470	34.917440	405.886400	.000146
-44.302690	34.697410	405.886500	.000201
-90.232250	35.766320	424.928100	12.954350
-89.092860	35.987640	430.599600	15.651730
-87.941790	36.199090	444.720400	21.999340
-86.779330	36.400320	465.857600	31.147770
-85.605870	36.591000	490.651700	41.573920
-84.421870	36.770790	513.458400	50.951080
-83.227800	36.939400	530.500100	57.722500
-82.024180	37.096500	541.439000	61.431840
-80.811620	37.241800	545.138200	61.448800
-79.590740	37.375040	534.044000	55.204130
-78.362190	37.495960	498.244400	40.073520
-77.126670	37.604320	433.244400	21.319400
-75.884930	37.699920	374.903800	14.116010
-74.637700	37.782550	375.901200	16.083440
-73.385770	37.852060	408.235900	20.861940
-72.129940	37.908310	432.291500	19.994670
-70.871030	37.951180	444.130200	22.403080
-69.609860	37.980580	444.329000	21.379170
-68.347290	37.996460	438.084100	18.154820
-67.084140	37.998780	428.519900	13.203750
-65.821270	37.987530	418.427600	7.779361
-64.559530	37.962740	411.208400	3.577116
-63.299760	37.924460	407.606700	1.311538
-62.042780	37.872770	406.300800	.400576
-60.789440	37.807780	405.995000	.147498
-59.540510	37.729610	405.993700	.104609
-58.296810	37.638420	406.030800	.112450

-57.059080	37.534390	406.026900	.103441
-55.828080	37.417740	405.974600	.064296
-54.604500	37.288680	405.916700	.023491
-53.389020	37.147460	405.886900	.001849
-52.182290	36.994370	405.879500	.005983
-50.984880	36.829660	405.880700	.005341
-49.797380	36.653650	405.883200	.003073
-48.620270	36.466650	405.884800	.001490
-47.454020	36.268990	405.885600	.000629
-46.299010	36.060990	405.886100	.000263
-45.155610	35.843010	405.886200	.000121
-44.024070	35.615370	405.886400	.000182
-90.516530	36.687310	443.706300	21.812740
-89.365660	36.916220	456.996500	26.847840
-88.202590	37.134950	488.933900	38.223550
-87.027650	37.343150	537.868000	55.177770
-85.841200	37.540460	597.144800	75.429000
-84.643710	37.726540	648.934900	92.491990
-83.435650	37.901050	683.633100	103.169600
-82.217600	38.063690	705.783900	109.086300
-80.990140	38.214140	722.482100	111.996800
-79.753940	38.352110	704.332200	102.509900
-78.509670	38.477340	630.434000	76.791330
-77.258070	38.589580	472.000600	43.595510
-75.999910	38.688600	296.875600	25.237550
-74.735980	38.774210	332.526900	57.397320
-73.467090	38.846230	457.781500	66.437620
-72.194090	38.904510	504.092200	60.553290
-70.917830	38.948930	512.653100	50.962370
-69.639210	38.979390	503.962600	45.600360
-68.359090	38.995840	485.569000	37.670970
-67.078360	38.998240	461.353700	27.464170
-65.797930	38.986590	433.969100	14.934290
-64.518690	38.960900	415.461900	5.619281
-63.241520	38.921240	407.896300	1.484050
-61.967320	38.867690	405.938100	.523593
-60.696920	38.800350	405.865800	.288760
-59.431210	38.719360	406.141600	.241913
-58.170970	38.624900	406.321200	.309707
-56.917050	38.517150	406.321400	.291660
-55.670200	38.396340	406.136200	.172461
-54.431160	38.262690	405.943100	.041585
-53.200640	38.116470	405.866200	.016532
-51.979310	37.957960	405.857000	.023740
-50.767770	37.787460	405.867400	.016255
-49.566610	37.605300	405.876900	.008607
-48.376340	37.411790	405.882400	.003758
-47.197410	37.207270	405.884800	.001494
-46.030250	36.992090	405.885700	.000539
-44.875180	36.766620	405.886000	.000201
-43.732470	36.531200	405.886300	.000131
-90.814130	37.606180	471.848200	31.790760
-89.651300	37.842850	499.534000	40.012700
-88.475770	38.069030	566.536600	58.962820
-87.287830	38.284350	674.832000	90.036730
-86.087840	38.488450	814.860000	131.270000
-84.876290	38.680970	928.904100	162.647800
-83.653650	38.861550	989.824300	176.466400
-82.420500	39.029860	1030.543000	185.168600
-81.177470	39.185590	1075.633000	191.803800
-79.925230	39.328410	1062.434000	185.585600
-78.664510	39.458070	944.538800	154.665000
-77.396060	39.574290	627.963300	97.096280
-76.120680	39.676830	102.825900	119.274800
-74.839220	39.765490	208.410800	84.316430
-73.552520	39.840080	572.611100	142.538100
-72.261480	39.900440	696.867800	137.251700

-70.967010	39.946460	687.886400	114.800100
-69.670030	39.978020	640.824500	91.112620
-68.371470	39.995060	594.105800	75.382060
-67.072270	39.997550	533.751800	53.621840
-65.773400	39.985470	461.318900	26.184720
-64.475770	39.958870	418.514600	7.184642
-63.180340	39.917780	405.068700	2.170104
-61.888020	39.862300	404.014000	1.707554
-60.599740	39.792560	405.440200	.758910
-59.316390	39.708680	406.621800	.626180
-58.038840	39.610860	407.224900	.875473
-56.767930	39.499290	407.281000	.915958
-55.504470	39.374200	406.620100	.514182
-54.249260	39.235840	405.931000	.072598
-53.003000	39.084480	405.773500	.077268
-51.766410	38.920440	405.789200	.070585
-50.540120	38.744010	405.832900	.042415
-49.324710	38.555530	405.862800	.020084
-48.120720	38.355340	405.877300	.008304
-46.928620	38.143810	405.883200	.003033
-45.748810	37.921300	405.885200	.001037
-44.581620	37.688170	405.885800	.000352
-43.427300	37.444810	405.886300	.000194
-91.125630	38.522790	504.964100	39.637190
-89.950410	38.767390	556.433400	51.788210
-88.761920	39.001200	685.243400	81.145260
-87.560450	39.223820	904.215700	134.807200
-86.346370	39.434880	1206.103000	217.462200
-85.120160	39.633990	1449.937000	280.618800
-83.882300	39.820790	1563.931000	303.674600
-82.633390	39.994930	1617.200000	309.756100
-81.374080	40.156070	1695.454000	311.938100
-80.105060	40.303890	1724.301000	326.180600
-78.827100	40.438090	1642.016000	306.101300
-77.540980	40.558400	1249.209000	220.167600
-76.247540	40.664560	402.482400	226.377400
-74.947690	40.756360	117.078800	179.321200
-73.642300	40.833600	635.519000	150.260400
-72.332310	40.896110	879.777600	134.060300
-71.018690	40.943760	976.097800	190.366800
-69.702430	40.976440	880.898900	161.653100
-68.384490	40.994090	776.328900	125.182600
-67.065880	40.996670	641.004600	82.160340
-65.747600	40.984170	473.097700	31.238390
-64.430650	40.956610	400.729800	9.617704
-63.116020	40.914060	392.823800	7.865355
-61.804690	40.856610	399.744000	4.223458
-60.497620	40.784390	405.278700	1.636589
-59.195770	40.697540	408.455800	1.827474
-57.900040	40.596260	410.110900	2.720082
-56.611330	40.480750	410.887300	3.489150
-55.330490	40.351270	406.956100	.759229
-54.058330	40.208070	405.684200	.430880
-52.795620	40.051450	405.426600	.281526
-51.543080	39.881710	405.623300	.174656
-50.301390	39.699200	405.763700	.088912
-49.071130	39.504250	405.837500	.038789
-47.852850	39.297230	405.869300	.014742
-46.647030	39.078520	405.880700	.005110
-45.454070	38.848490	405.884500	.001636
-44.274280	38.607530	405.885600	.000529
-43.107920	38.356030	405.886100	.000215
-91.451740	39.437030	529.798000	41.842180
-90.263630	39.689750	608.093900	56.665610
-89.061680	39.931370	802.374700	89.014800
-87.846150	40.161480	1117.975000	156.621000
-86.617400	40.379670	1582.666000	265.972800

-85.375900	40.585540	2023.571000	372.908300
-84.122160	40.778730	2259.477000	421.302400
-82.856800	40.958850	2334.624000	421.861900
-81.580460	41.125550	2410.123000	438.589100
-80.293880	41.278500	2542.277000	482.802000
-78.997860	41.417370	2618.306000	516.171400
-77.693230	41.541890	2381.720000	457.435900
-76.380870	41.651780	1572.463000	241.568000
-75.061690	41.746810	673.119900	90.633970
-73.736660	41.826770	328.569900	215.246000
-72.406780	41.891490	645.293800	112.306300
-71.073040	41.940830	1072.886000	178.442500
-69.736500	41.974670	1031.553000	195.519500
-68.398190	41.992950	870.368100	125.246500
-67.059160	41.995620	610.622700	62.222370
-65.720480	41.982670	358.410800	32.302410
-64.383220	41.954140	311.936100	35.904120
-63.048400	41.910080	363.803300	18.617510
-61.717090	41.850600	395.864200	7.891022
-60.390290	41.775820	409.340300	4.652491
-59.069010	41.685920	414.942400	5.936202
-57.754210	41.581080	419.770600	9.107055
-56.446830	41.461530	418.315900	8.398664
-55.147770	41.327530	404.580800	2.341796
-53.857880	41.179360	402.749700	2.339608
-52.577970	41.017320	404.525800	.904820
-51.308780	40.841740	405.301100	.363541
-50.051000	40.652980	405.670100	.145882
-48.805250	40.451390	405.811400	.055946
-47.572090	40.237360	405.862800	.019571
-46.352010	40.011290	405.879400	.006300
-45.145370	39.773560	405.884300	.001930
-43.952510	39.524580	405.885600	.000585
-42.773630	39.264750	405.886100	.000232
-91.793200	40.348750	524.599500	36.510500
-90.591710	40.609810	599.091700	51.106500
-89.375760	40.859430	748.612100	77.474880
-88.145600	41.097210	893.521200	98.142950
-86.901570	41.322720	1229.428000	172.338800
-85.644150	41.535550	1819.840000	301.576800
-84.373860	41.735290	2275.140000	410.526100
-83.091290	41.921560	2494.222000	461.389500
-81.797160	42.093980	2704.267000	513.545900
-80.492230	42.252200	3033.637000	590.953700
-79.177290	42.395880	3245.117000	646.696700
-77.853240	42.524730	3105.584000	615.345300
-76.521000	42.638450	2359.901000	410.953200
-75.181550	42.736820	1328.765000	147.620400
-73.835900	42.819590	647.353800	121.952400
-72.485100	42.886590	520.045000	169.859100
-71.130210	42.937670	979.378200	167.100800
-69.772340	42.972710	947.402300	210.006900
-68.412590	42.991630	763.644300	123.277700
-67.052090	42.994400	390.981400	107.191100
-65.691960	42.980990	103.735900	110.626800
-64.333320	42.951450	160.454800	76.072880
-62.977290	42.905830	344.442800	39.878780
-61.624970	42.844260	413.105300	20.467250
-60.277440	42.766850	426.785300	15.445790
-58.935750	42.673790	435.297200	18.158400
-57.600940	42.565280	439.019800	20.821400
-56.273990	42.441570	422.592100	8.119124
-54.955840	42.302930	394.794700	7.915344
-53.647390	42.149640	396.021800	7.238366
-52.349470	41.982030	402.469900	2.336945
-51.062890	41.800450	405.040100	.578816
-49.788320	41.605260	405.657700	.173688

-48.526440	41.396860	405.828500	.058935
-47.277780	41.175640	405.874100	.021391
-46.042850	40.942000	405.884400	.007810
-44.822010	40.696380	405.886200	.002788
-43.615560	40.439180	405.886300	.000954
-42.423690	40.170830	405.886300	.000428
-92.150770	41.257820	488.637800	30.559700
-90.935390	41.527410	517.860200	47.048620
-89.704910	41.785250	503.572000	53.287430
-88.459540	42.030900	324.975400	129.430700
-87.199620	42.263930	350.189100	209.311200
-85.925610	42.483890	1233.093000	211.904600
-84.638040	42.690380	2043.063000	372.477200
-83.337520	42.882970	2518.575000	476.270200
-82.024790	43.061280	2929.350000	570.380100
-80.700610	43.224930	3408.337000	687.463600
-79.365870	43.373580	3605.323000	761.376700
-78.021450	43.506890	3407.474000	703.648900
-76.668370	43.624570	2779.028000	522.997300
-75.307630	43.726360	1862.924000	286.949900
-73.940300	43.812040	1132.769000	139.914500
-72.567500	43.881390	477.981100	158.491900
-71.190370	43.934270	746.011900	182.879700
-69.810050	43.970550	1089.260000	224.463000
-68.427750	43.990140	1163.597000	207.353100
-67.044650	43.993000	728.395700	121.794200
-65.661930	43.979120	374.317100	70.481930
-64.280810	43.948540	212.355200	93.937810
-62.902460	43.901310	394.987100	44.605760
-61.528050	43.837570	472.696600	50.062420
-60.158730	43.757450	477.223100	43.170770
-58.795620	43.661140	476.212600	39.177590
-57.439790	43.548850	458.720600	26.712770
-56.092310	43.420850	410.065900	8.417518
-54.754150	43.277410	387.014400	15.924260
-53.426270	43.118850	396.028700	8.053232
-52.109530	42.945500	403.718200	2.055716
-50.804760	42.757750	405.734600	.679064
-49.512690	42.555960	406.008100	.322284
-48.233980	42.340550	405.980900	.154730
-46.969190	42.111940	405.932600	.067086
-45.718810	41.870550	405.904700	.026180
-44.483210	41.616830	405.892700	.009219
-43.262650	41.351200	405.888200	.003038
-42.057300	41.074100	405.887100	.001301
-92.525320	42.164070	451.322000	23.903450
-91.295520	42.442420	462.168000	42.074830
-90.049940	42.708690	453.218800	92.054760
-88.788760	42.962440	246.058300	99.138170
-87.512310	43.203190	203.832700	124.599700
-86.221000	43.430500	1022.866000	151.860000
-84.915410	43.643920	1915.248000	324.445400
-83.596150	43.843030	2514.257000	501.459800
-82.263960	44.027400	3043.424000	638.750900
-80.919660	44.196660	3621.660000	738.320600
-79.564150	44.350410	3773.031000	778.867200
-78.198390	44.488330	3413.972000	698.994000
-76.823410	44.610100	2607.604000	478.404800
-75.440290	44.715440	1628.057000	225.620700
-74.050180	44.804110	796.233200	152.536300
-72.654240	44.875890	114.077200	164.504300
-71.253690	44.930630	571.718100	135.781300
-69.849760	44.968180	1372.806000	304.355700
-68.443720	44.988460	1717.090000	334.343800
-67.036810	44.991420	1161.147000	212.138900
-65.630330	44.977050	621.773100	100.803500
-64.225530	44.945390	397.649700	78.785860

```
-62.823690 44.896510 499.698800 56.592840
-61.426040 44.830540 568.262100 75.882400
-60.033810 44.747610 558.488300 73.401700
-58.648170 44.647940 528.323200 56.075340
-57.270290 44.531750 466.710300 25.486070
-55.901260 44.399320 395.442500 23.721030
-54.542130 44.250930 391.304000 19.692960
-53.193900 44.086940 405.058100 6.411178
-51.857480 43.907680 408.902100 3.343462
-50.533710 43.713560 408.053600 1.943167
-49.223370 43.504980 407.000700 .999485
-47.927120 43.282370 406.369100 .455134
-46.645540 43.046150 406.071000 .185322
-45.379100 42.796800 405.949600 .068103
-44.128160 42.534760 405.905900 .022795
-42.892960 42.260480 405.892000 .007188
-41.673610 41.974410 405.888500 .002916
-92.917770 43.067330 440.073300 27.274840
-91.673000 43.354680 481.528000 54.551380
-90.411740 43.629630 529.876000 76.487380
-89.134110 43.891690 488.434200 155.645000
-87.840450 44.140390 52.403330 92.346210
-86.531140 44.375260 394.047700 146.214600
-85.206750 44.595830 1303.444000 245.981400
-83.867900 44.801650 2167.552000 422.132900
-82.515380 44.992280 2762.227000 571.080200
-81.150000 45.167310 3193.953000 672.057600
-79.772730 45.326340 3288.540000 679.620800
-78.384560 45.469020 2942.520000 561.594600
-76.986600 45.595010 2127.560000 316.347500
-75.579970 45.704020 1085.329000 124.859100
-74.165890 45.795780 179.948900 204.420000
-72.745610 45.870080 78.378040 70.532280
-71.320400 45.926740 428.687300 232.770500
-69.891600 45.965610 1470.687000 326.879200
-68.460530 45.986610 1822.268000 367.387000
-67.028560 45.989670 1225.244000 242.859100
-65.597030 45.974800 811.722300 140.415300
-64.167300 45.942020 685.041600 84.195380
-62.740720 45.891430 685.092700 104.327000
-61.318600 45.823140 661.610300 105.284000
-59.902260 45.737310 615.587600 88.493260
-58.492950 45.634170 548.894000 67.188490
-57.091900 45.513950 469.542100 41.648810
-55.700260 45.376930 423.108700 29.071830
-54.319140 45.223450 428.453000 18.032550
-52.949590 45.053840 428.738300 13.842880
-51.592580 44.868490 419.952900 8.798481
-50.248970 44.667810 412.860800 4.715328
-48.919560 44.452230 408.884200 2.217984
-47.605040 44.222190 407.053600 .947515
-46.305980 43.978160 406.300400 .368098
-45.022840 43.720610 406.020800 .130221
-43.755960 43.450020 405.926400 .042205
-42.505550 43.166850 405.897400 .012914
-41.271660 42.871580 405.890400 .005031
-93.329110 43.967430 424.739100 29.020820
-92.068780 44.264030 429.971400 42.473980
-90.791210 44.547890 412.768600 73.391460
-89.496510 44.818510 180.442100 128.492000
-88.184940 45.075390 209.416900 156.792300
-86.856880 45.318050 296.705500 177.963500
-85.512890 45.545990 1143.458000 156.603900
-84.153590 45.758730 2001.059000 361.825800
-82.779790 45.955820 2447.411000 485.428400
-81.392350 46.136820 2599.482000 517.571500
-79.992260 46.301310 2542.716000 494.559300
```

-78.580580	46.448910	2272.433000	404.761900
-77.158460	46.579270	1773.045000	240.747800
-75.727110	46.692070	1132.068000	66.183660
-74.287820	46.787040	623.160500	117.176000
-72.841890	46.863950	450.213600	147.805000
-71.390720	46.922590	860.009500	173.439400
-69.935700	46.962830	1627.888000	310.114100
-68.478260	46.984570	1622.530000	311.807800
-67.019850	46.987740	1170.572000	201.625400
-65.561920	46.972340	910.598600	123.212600
-64.105920	46.938410	860.776900	123.613300
-62.653260	46.886040	768.983600	117.071000
-61.205390	46.815350	686.782400	94.219770
-59.763680	46.726520	636.811900	85.222170
-58.329470	46.619790	588.656900	77.362120
-56.904060	46.495390	537.959700	64.986020
-55.488670	46.353640	521.188400	55.949720
-54.084500	46.194880	512.963300	52.622320
-52.692630	46.019470	477.099000	36.510080
-51.314070	45.827840	441.861000	19.792440
-49.949730	45.620390	421.532300	9.561817
-48.600430	45.397590	411.790100	3.971094
-47.266860	45.159900	407.946600	1.533445
-45.949600	44.907820	406.571700	.566004
-44.649100	44.641840	406.098900	.193418
-43.365670	44.362450	405.947600	.061023
-42.099450	44.070140	405.903000	.018223
-40.850440	43.765410	405.892100	.006908
-93.760410	44.864160	430.287500	33.394310
-92.483920	45.170280	439.325700	48.840590
-91.189420	45.463320	387.598500	57.706350
-89.876960	45.742750	248.721500	77.277150
-88.546760	46.008070	85.045040	74.625840
-87.199170	46.258760	201.292400	140.876500
-85.834720	46.494300	938.099800	105.474400
-84.454070	46.714200	1632.720000	242.130400
-83.058010	46.917960	2004.308000	358.196100
-81.647480	47.105130	2078.876000	389.028700
-80.223460	47.275260	1952.449000	340.587300
-78.787090	47.427960	1647.481000	237.212800
-77.339580	47.562840	1322.223000	164.445900
-75.882230	47.679570	1168.753000	121.562100
-74.416370	47.777870	1100.119000	108.283200
-72.943440	47.857470	1097.056000	152.223400
-71.464880	47.918190	1090.068000	189.139200
-69.982220	47.959850	1270.943000	215.075700
-68.496960	47.982350	1147.912000	185.869800
-67.010670	47.985630	951.382300	115.740500
-65.524890	47.969690	867.588700	89.270160
-64.041170	47.934570	870.131400	94.892110
-62.561040	47.880340	755.261200	75.957840
-61.086020	47.807170	634.674100	50.161570
-59.617590	47.715230	595.650100	52.900600
-58.157170	47.604770	595.728900	66.576000
-56.706150	47.476050	598.624600	79.398790
-55.265830	47.329400	621.676000	89.484050
-53.837470	47.165180	609.288800	86.373850
-52.422200	46.983780	536.537900	60.047530
-51.021090	46.785640	466.139800	30.316260
-49.635090	46.571200	430.262100	13.945380
-48.265020	46.340950	414.662800	5.676168
-46.911610	46.095370	408.674200	1.986879
-45.575410	45.834990	406.738000	.678848
-44.256860	45.560320	406.139300	.224600
-42.956210	45.271870	405.956900	.069103
-41.673580	44.970160	405.905000	.020174
-40.408870	44.655690	405.892500	.007451

-94.212860	45.757310	451.011900	40.222480
-92.919560	46.073230	458.124100	52.596930
-91.607470	46.375730	398.584800	58.250140
-90.276550	46.664250	164.665500	80.789370
-88.926960	46.938270	101.529100	96.823720
-87.559010	47.197250	415.624500	110.335700
-86.173240	47.440650	580.409400	93.046390
-84.770290	47.667940	946.353500	91.401290
-83.350960	47.878600	1463.479000	206.658600
-81.916200	48.072160	1733.709000	297.842300
-80.467090	48.248140	1631.688000	255.063500
-79.004780	48.406110	1241.105000	170.969300
-77.530590	48.545680	964.763500	135.412700
-76.045850	48.666500	932.634800	92.968470
-74.552020	48.768250	985.073300	88.929230
-73.050600	48.850660	992.408400	114.024500
-71.543170	48.913520	949.137900	102.031400
-70.031330	48.956650	932.217400	105.774800
-68.516710	48.979950	814.754200	92.004410
-67.000980	48.983350	797.779700	74.750020
-65.485790	48.966840	771.625800	67.925460
-63.972820	48.930470	792.554900	64.507210
-62.463700	48.874330	694.581000	43.709120
-60.960050	48.798580	584.993200	51.685600
-59.463460	48.703410	566.703600	50.476340
-57.975440	48.589080	587.097200	45.009650
-56.497490	48.455880	599.321000	72.790290
-55.030970	48.304140	617.147400	89.447370
-53.577210	48.134270	602.894300	82.915610
-52.137430	47.946660	525.130700	54.683400
-50.712720	47.741790	455.027700	26.059810
-49.304080	47.520120	423.573900	10.556960
-47.912350	47.282170	412.243600	4.297597
-46.538250	47.028450	408.122900	1.697599
-45.182340	46.759510	406.611500	.615821
-43.845020	46.475880	406.100900	.202285
-42.526500	46.178100	405.945300	.061423
-41.226800	45.866700	405.901500	.017599
-39.945740	45.542200	405.891300	.006404
-94.687740	46.646660	488.882100	49.793350
-93.376940	46.972690	514.339200	62.581440
-92.046550	47.284930	498.256200	63.841370
-90.696440	47.582840	389.847200	47.517300
-89.326680	47.865840	317.140000	33.367010
-87.937550	48.133390	403.800500	57.625800
-86.529520	48.384910	376.656200	151.245400
-85.103260	48.619850	645.951800	161.073000
-83.659580	48.837660	1284.354000	157.890000
-82.199460	49.037830	1612.291000	279.989900
-80.724000	49.219880	1454.836000	244.546600
-79.234450	49.383340	1074.635000	156.699300
-77.732160	49.527770	858.003100	111.311900
-76.218590	49.652830	805.876200	89.117090
-74.695260	49.758160	808.009700	81.808320
-73.163800	49.843490	813.094600	80.911390
-71.625870	49.908580	788.235600	75.867340
-70.083210	49.953250	732.001200	75.069490
-68.537570	49.977380	663.759600	78.357870
-66.990740	49.980900	730.275500	66.527930
-65.444490	49.963800	712.724900	88.050990
-63.900620	49.926140	693.611500	68.557910
-62.360880	49.868010	623.318300	49.503850
-60.827010	49.789570	622.916100	48.081970
-59.300730	49.691040	660.488000	61.086040
-57.783640	49.572690	663.001000	73.107840
-56.277320	49.434830	627.194100	79.140600
-54.783270	49.277820	589.334900	78.229120

-53.302860	49.102080	538.424300	63.342670
-51.837360	48.908040	477.916200	36.999770
-50.387950	48.696200	434.247000	16.886250
-48.955640	48.467040	416.320600	7.513806
-47.541310	48.221130	409.423100	3.178210
-46.145660	47.959000	407.012700	1.232335
-44.769230	47.681210	406.283500	.446167
-43.412380	47.388330	406.016200	.150159
-42.075270	47.080920	405.921900	.045098
-40.757810	46.759540	405.895100	.012749
-39.459720	46.424710	405.889000	.004622
-95.186440	47.531940	539.388000	64.432710
-93.857410	47.868410	577.015400	73.717580
-92.508000	48.190720	589.045100	78.905910
-91.137910	48.498310	545.808000	60.897400
-89.747150	48.790600	526.020100	44.888400
-88.335950	49.067010	620.385700	63.060750
-86.904710	49.326930	729.278000	70.105510
-85.454100	49.569790	949.130200	105.623600
-83.984930	49.795020	1312.554000	224.688200
-82.498220	50.002060	1406.262000	267.818100
-80.995120	50.190400	1228.241000	223.717100
-79.476920	50.359550	970.861300	148.025900
-77.945060	50.509060	866.499800	108.933900
-76.401090	50.638520	842.936200	96.858890
-74.846650	50.747580	846.531700	94.638850
-73.283450	50.835950	863.035300	103.262800
-71.713320	50.903350	852.778300	104.901400
-70.138080	50.949630	770.882600	74.981980
-68.559640	50.974620	666.275000	64.309900
-66.979900	50.978270	747.857500	75.421930
-65.400800	50.960560	730.098600	87.085450
-63.824260	50.921550	591.436200	78.341730
-62.252170	50.861340	426.051200	104.025000
-60.686400	50.780110	519.741900	63.126690
-59.128770	50.678080	656.210000	84.603480
-57.581030	50.555550	653.477900	89.916070
-56.044860	50.412850	585.234700	76.854940
-54.521820	50.250360	522.929500	56.105730
-53.013420	50.068520	476.097800	36.806180
-51.520960	49.867810	442.045900	20.721660
-50.045680	49.648740	421.905200	10.397840
-48.588630	49.411830	412.242400	4.846354
-47.150700	49.157670	407.979600	2.049866
-45.732590	48.886830	406.592900	.820245
-44.334810	48.599900	406.128100	.301305
-42.957660	48.297460	405.956000	.097446
-41.601190	47.980120	405.903900	.029025
-40.265250	47.648430	405.890100	.008256
-38.949390	47.302930	405.887200	.003050
-95.710510	48.412890	599.707000	80.439670
-94.362470	48.760130	656.982800	91.324460
-92.993230	49.092850	686.187000	98.738590
-91.602360	49.410460	687.347100	94.362450
-90.189750	49.712350	708.236000	85.113670
-88.755540	49.997940	773.816700	99.432650
-87.300090	50.266570	848.261500	123.023400
-85.824040	50.517650	937.517300	156.873400
-84.328210	50.750570	1059.463000	197.769200
-82.813610	50.964740	1086.411000	206.672600
-81.281460	51.159630	994.043000	171.210100
-79.733130	51.334700	853.270700	127.237400
-78.170130	51.489480	781.033100	104.557400
-76.594090	51.623530	786.731300	105.250300
-75.006800	51.736490	827.315800	116.214800
-73.410070	51.828010	867.511200	130.240000
-71.805870	51.897850	871.965900	137.039300

-70.196170	51.945790	827.347800	110.837700
-68.582990	51.971680	710.204800	79.798650
-66.968440	51.975460	759.387800	86.099870
-65.354560	51.957120	808.752500	117.499000
-63.743440	51.916690	761.170200	101.011500
-62.137130	51.854320	605.923100	73.083500
-60.537630	51.770170	580.924800	81.785320
-58.946900	51.664510	612.053300	83.595860
-57.366820	51.537620	572.022000	74.443710
-55.799180	51.389870	509.313800	49.683590
-54.245650	51.221680	465.246200	31.008420
-52.707820	51.033510	438.046800	18.568140
-51.187080	50.825860	422.540600	10.650360
-49.684720	50.599280	413.829600	5.705167
-48.201800	50.354330	409.277600	2.789974
-46.739240	50.091630	407.136600	1.216283
-45.297720	49.811770	406.313000	.484564
-43.877700	49.515370	406.014800	.171216
-42.479410	49.203060	405.920100	.055193
-41.102810	48.875440	405.893700	.016623
-39.747590	48.533100	405.887400	.004821
-38.413150	48.176570	405.886300	.001887
-96.261670	49.289190	657.759500	91.820480
-94.893750	49.647600	739.654300	104.767200
-93.503820	49.991080	774.654300	109.036100
-92.091320	50.319060	768.131700	103.668200
-90.655950	50.630910	741.392000	99.662090
-89.197760	50.925990	718.707100	100.429500
-87.717060	51.203660	719.228000	105.187100
-86.214420	51.463270	748.486900	117.205000
-84.690660	51.704190	821.907000	139.631000
-83.146820	51.925780	878.463900	151.547100
-81.584160	52.127480	878.549600	139.837300
-80.004110	52.308710	784.615600	114.556200
-78.408280	52.468990	698.537600	99.531460
-76.798410	52.607840	705.511700	97.898990
-75.176390	52.724850	768.411300	107.771100
-73.544200	52.819690	810.548200	112.923500
-71.903920	52.892050	791.959200	109.277800
-70.257710	52.941730	745.231800	87.917400
-68.607750	52.968570	643.350200	52.104540
-66.956280	52.972490	664.829600	62.539880
-65.305560	52.953480	761.999500	106.017900
-63.657820	52.911580	792.106700	126.502800
-62.015270	52.846950	709.920800	100.318100
-60.380080	52.759750	616.860200	81.119950
-58.754360	52.650280	569.099800	73.384580
-57.140120	52.518850	517.337800	56.331440
-55.539290	52.365840	470.202600	35.255080
-53.953660	52.191710	439.199900	19.526460
-52.384890	51.996940	422.928300	10.883390
-50.834480	51.782070	414.337000	5.993414
-49.303730	51.547700	409.911800	3.185322
-47.793770	51.294400	407.625600	1.541974
-46.305490	51.022820	406.538800	.658791
-44.839570	50.733620	406.101900	.252318
-43.396390	50.427420	405.948400	.087581
-41.976090	50.104880	405.901300	.028122
-40.578500	49.766630	405.888900	.008501
-39.203150	49.413260	405.886400	.002536
-37.849210	49.045330	405.886000	.001059
-96.841810	50.160530	646.967800	83.272420
-95.453030	50.530490	712.097700	90.819640
-94.041510	50.885130	713.446100	97.921990
-92.606450	51.223850	677.389800	93.422030
-91.147380	51.546020	635.416200	83.389030
-89.664200	51.850960	600.380200	74.996830

-88.157130	52.138020	588.281600	73.144330
-86.626690	52.406500	608.952300	80.855230
-85.073680	52.655720	671.015700	96.795070
-83.499180	52.885050	757.163300	112.094400
-81.904440	53.093850	788.855700	119.873000
-80.290990	53.281520	749.879800	108.655200
-78.660530	53.447530	670.862700	95.563030
-77.014920	53.591380	653.306600	90.689470
-75.356160	53.712650	683.827100	94.155620
-73.686420	53.810940	680.876300	84.760380
-72.007930	53.885960	597.360800	65.518670
-70.322990	53.937460	474.648800	93.683940
-68.634010	53.965280	383.860800	129.641400
-66.943400	53.969350	471.381000	70.167020
-65.253590	53.949630	631.750200	55.057310
-63.567000	53.906200	666.493400	85.378490
-61.886040	53.839190	597.102200	67.465640
-60.213040	53.748820	522.567900	43.879430
-58.550290	53.635360	494.719800	34.546320
-56.899960	53.499180	484.593300	39.489170
-55.264110	53.340690	461.545400	34.676740
-53.644650	53.160350	433.901900	18.755750
-52.043340	52.958710	417.476700	8.024083
-50.461760	52.736320	410.948600	3.797258
-48.901280	52.493820	408.071600	1.819749
-47.363030	52.231850	406.769500	.819271
-45.847900	51.951060	406.208000	.335614
-44.356530	51.652150	405.990100	.124508
-42.889220	51.335790	405.915200	.042253
-41.445980	51.002650	405.892900	.013282
-40.026490	50.653380	405.887300	.003960
-38.630060	50.288590	405.886100	.001200
-37.255620	49.908830	405.886000	.000541
-97.453060	51.026530	555.443100	58.920720
-96.042340	51.408490	581.549100	64.351310
-94.608220	51.774700	583.516200	67.102250
-93.149600	52.124570	570.226200	66.072700
-91.665800	52.457450	552.950100	64.025920
-90.156560	52.772640	535.632300	61.071900
-88.621980	53.069450	526.624800	58.632560
-87.062480	53.347160	531.369800	59.202820
-85.478850	53.605050	558.800000	66.366540
-83.872130	53.842430	601.030500	76.350100
-82.243660	54.058630	625.128900	85.576280
-80.595020	54.253030	627.751300	85.518290
-78.928000	54.425040	604.588400	87.183290
-77.244600	54.574130	611.431200	87.314350
-75.546970	54.699840	648.147400	88.896350
-73.837420	54.801750	656.919000	79.497510
-72.118360	54.879540	571.687000	55.254810
-70.392330	54.932960	398.066100	93.927720
-68.661900	54.961820	292.096400	154.389400
-66.929700	54.966030	415.753700	65.175450
-65.198370	54.945580	536.093400	45.993310
-63.470540	54.900540	519.219500	56.305950
-61.748820	54.831050	446.362400	58.719730
-60.035740	54.737340	392.954000	65.774960
-58.333780	54.619720	386.818200	51.297100
-56.645260	54.478570	416.186900	19.159040
-54.972420	54.314330	436.262500	15.355590
-53.317280	54.127510	427.936900	15.408110
-51.681740	53.918690	415.349300	7.075382
-50.067420	53.688470	409.105900	2.472597
-48.475770	53.437500	407.109300	1.024424
-46.907930	53.166490	406.344500	.428522
-45.364770	52.876130	406.046700	.168649
-43.846840	52.567130	405.936800	.060896

-42.354370	52.240220	405.900100	.020037
-40.887210	51.896080	405.889400	.006074
-39.444820	51.535370	405.886700	.001707
-38.026250	51.158730	405.886100	.000509
-36.630120	50.766700	405.886300	.000271
-98.097820	51.886770	479.633100	33.746170
-96.663910	52.281200	486.237000	35.596350
-95.206040	52.659450	488.554100	37.453110
-93.722790	53.020900	490.207900	41.255080
-92.213200	53.364910	494.053000	46.940640
-90.676770	53.690770	499.692000	52.559650
-89.113440	53.997730	503.609300	55.718560
-87.523560	54.285050	505.070600	55.643490
-85.907830	54.551970	509.172300	54.433600
-84.267300	54.797760	517.337600	53.764960
-82.603340	55.021710	518.979100	54.930860
-80.917590	55.223140	522.029300	61.172890
-79.211960	55.401440	548.529300	73.517090
-77.488560	55.556020	594.278300	82.116630
-75.749730	55.686380	642.002800	94.474170
-73.997920	55.792100	663.441000	89.892720
-72.235800	55.872810	608.481500	59.268570
-70.466090	55.928230	466.598900	48.779510
-68.691570	55.958180	417.351300	58.460910
-66.915130	55.962550	505.182900	36.444700
-65.139640	55.941330	532.327900	44.854700
-63.367970	55.894590	473.552500	41.229540
-61.602940	55.822490	402.678800	51.154020
-59.847310	55.725280	352.786100	73.239990
-58.103760	55.603290	331.116500	84.872400
-56.374820	55.456930	345.488800	63.716270
-54.662870	55.286670	376.716600	30.580740
-52.970100	55.093080	401.786700	7.763449
-51.298500	54.876750	407.657400	1.151415
-49.649800	54.638340	407.226500	1.023728
-48.025460	54.378550	406.423700	.455748
-46.426660	54.098120	406.097300	.199333
-44.854210	53.797780	405.961400	.079874
-43.308580	53.478290	405.910300	.028788
-41.789850	53.140400	405.892900	.009362
-40.297680	52.784830	405.887700	.002721
-38.831280	52.412250	405.886500	.000726
-37.389370	52.023280	405.886400	.000250
-35.970190	51.618480	405.886400	.000213
-98.778840	52.740810	439.946900	18.218330
-97.320270	53.148240	440.139100	18.228780
-95.837370	53.539010	441.243700	19.022340
-94.328290	53.912530	443.217500	21.184680
-92.791720	54.268120	447.875400	25.590670
-91.226900	54.605080	456.320500	32.676510
-89.633560	54.922620	467.557500	41.023080
-88.011890	55.219970	481.183600	48.805220
-86.362530	55.496330	492.490300	53.687100
-84.686490	55.750910	494.810600	53.433580
-82.985150	55.982970	482.723200	49.171350
-81.260250	56.191770	465.364700	52.249230
-79.513790	56.376650	496.707600	64.632880
-77.748020	56.536990	589.038700	78.962900
-75.965460	56.672260	647.128800	94.718600
-74.168790	56.781970	668.540500	97.009730
-72.360860	56.865740	637.121300	75.232560
-70.544630	56.923280	546.540800	55.534760
-68.723180	56.954370	532.595200	52.045320
-66.899620	56.958910	570.219300	61.407210
-65.077100	56.936880	531.255700	52.607300
-63.258730	56.888360	470.037200	32.504760
-61.447630	56.813510	420.098000	21.165110

-59.646790 56.712620 387.029600 30.295410
-57.859090 56.586040 370.423800 38.812940
-56.087300 56.434210 370.295100 36.201990
-54.333970 56.257640 383.802000 22.205720
-52.601470 56.056940 400.388800 5.975307
-50.891880 55.832740 405.669100 .938752
-49.207030 55.585770 406.055500 .179383
-47.548420 55.316760 406.027800 .122832
-45.917200 55.026500 405.956800 .068135
-44.314130 54.715760 405.914900 .031074
-42.739580 54.385350 405.896100 .012062
-41.193420 54.036030 405.889100 .004011
-39.675070 53.668560 405.887000 .001160
-38.183400 53.283620 405.886500 .000331
-36.716770 52.881820 405.886400 .000217
-35.272900 52.463700 405.886400 .000193
-99.499240 53.588130 420.620900 9.172546
-98.014280 54.009130 420.074200 8.897493
-96.504860 54.412960 420.323600 9.124585
-94.968610 54.799050 421.169200 9.984780
-93.403790 55.166730 423.259600 11.920410
-91.809290 55.515250 427.351300 15.359910
-90.184560 55.843850 434.190200 20.630980
-88.529620 56.151670 444.885200 28.324540
-86.844990 56.437890 457.407700 37.408430
-85.131650 56.701690 468.136600 45.933620
-83.390960 56.942230 464.116800 52.014940
-81.624720 57.158780 434.845900 58.032190
-79.835050 57.350580 466.608300 62.396740
-78.024370 57.516980 569.113600 74.668430
-76.195360 57.657400 652.943700 94.120420
-74.350940 57.771310 676.742400 101.064400
-72.494220 57.858320 667.996800 90.224430
-70.628410 57.918090 620.719500 75.954260
-68.756890 57.950390 601.796800 75.883960
-66.883060 57.955100 581.049300 71.464840
-65.010370 57.932220 516.907400 51.707400
-63.142240 57.881810 465.911900 31.017290
-61.282040 57.804080 433.138200 17.480010
-59.433060 57.699310 410.607600 8.994574
-57.598450 57.567890 398.754300 10.099660
-55.781200 57.410300 395.794500 10.472580
-53.984060 57.227100 398.442700 7.169584
-52.209560 57.018940 403.515300 2.429232
-50.459930 56.786500 405.228900 .688794
-48.737060 56.530560 405.752700 .174476
-47.042490 56.251900 405.877500 .031018
-45.377330 55.951360 405.897100 .011265
-43.742270 55.629770 405.893700 .008335
-42.137470 55.287960 405.889300 .003891
-40.562610 54.926740 405.887300 .001448
-39.016750 54.546860 405.886600 .000439
-37.498410 54.149020 405.886400 .000213
-36.005400 53.733830 405.886400 .000212
-34.534880 53.301770 405.886400 .000174
-100.262700 54.428120 411.789600 4.221871
-98.749250 54.863350 411.381000 3.994725
-97.211560 55.280850 411.422300 4.053202
-95.646590 55.680060 411.812000 4.446434
-94.052090 56.060360 412.714800 5.305952
-92.426510 56.420970 414.390900 6.789421
-90.768940 56.761100 417.140700 9.101617
-89.079190 57.079890 421.307300 12.672730
-87.357570 57.376450 426.351200 17.801100
-85.605000 57.649890 429.771600 25.268730
-83.822840 57.899360 416.410300 37.136590
-82.012920 58.124030 378.868000 55.473740

-80.177480 58.323120 426.063000 52.407230
-78.319110 58.495910 540.516500 63.589310
-76.440710 58.641750 646.689900 91.733540
-74.545430 58.760110 698.221400 107.158800
-72.636660 58.850530 709.891700 99.750680
-70.717930 58.912650 678.260000 92.049560
-68.792920 58.946230 632.758400 88.085700
-66.865380 58.951130 575.811100 71.235530
-64.939080 58.927340 502.859000 45.640160
-63.017780 58.874950 459.117500 28.574950
-61.105210 58.794160 437.345900 18.953820
-59.204930 58.685300 419.811300 10.339420
-57.320400 58.548780 408.791800 3.872197
-55.454840 58.385130 404.443800 2.565480
-53.611270 58.194950 404.011400 2.036001
-51.792370 57.978940 404.894400 1.025150
-50.000500 57.737840 405.492900 .422739
-48.237630 57.472490 405.757400 .148765
-46.505310 57.183730 405.854000 .041790
-44.804610 56.872430 405.881300 .008052
-43.136050 56.539500 405.886400 .001005
-41.499610 56.185780 405.886700 .000751
-39.894630 55.812120 405.886400 .000358
-38.319800 55.419290 405.886300 .000143
-36.773090 55.007980 405.886200 .000077
-35.251730 54.578750 405.886200 .000061
-33.752120 54.132060 405.886400 .000176
-101.073700 55.260130 408.065000 1.768080
-99.529010 55.710320 407.854200 1.629509
-97.960900 56.142140 407.855100 1.644244
-96.365430 56.555100 408.027200 1.825670
-94.739640 56.948590 408.414200 2.221672
-93.081440 57.321860 409.079200 2.888697
-91.389490 57.674070 410.007600 3.926025
-89.663250 58.004340 410.911100 5.678875
-87.902820 58.311730 410.784600 9.121328
-86.109000 58.595320 405.043100 18.013320
-84.283100 58.854170 373.758800 40.375610
-82.427000 59.087400 314.082600 71.659920
-80.543050 59.294160 375.962600 40.873080
-78.634010 59.473690 496.173400 42.653560
-76.703000 59.625280 625.733900 84.502690
-74.753460 59.748340 717.155500 104.933800
-72.789070 59.842370 734.683700 106.415800
-70.813740 59.906970 711.745200 102.059800
-68.831480 59.941910 658.639800 94.028350
-66.846440 59.947010 577.687700 68.415270
-64.862770 59.922250 496.103100 41.402200
-62.884610 59.867760 450.816800 24.053510
-60.916040 59.783740 432.520000 15.971510
-58.961010 59.670550 421.405200 10.345460
-57.023270 59.528640 412.088300 4.766860
-55.106350 59.358580 407.220300 1.471041
-53.213500 59.161040 405.722500 .626692
-51.347600 58.936760 405.605500 .387693
-49.511150 58.686560 405.727200 .196372
-47.706190 58.411320 405.821800 .080611
-45.934240 58.111950 405.865700 .026798
-44.196280 57.789400 405.881000 .007319
-42.492650 57.444580 405.885100 .001518
-40.823030 57.078400 405.886000 .000232
-39.186360 56.691730 405.886200 .000093
-37.580830 56.285340 405.886100 .000077
-36.003750 55.859910 405.886100 .000075
-34.451600 55.415950 405.886200 .000053
-32.919830 54.953830 405.886300 .000054
-101.937200 56.083360 406.627000 .674439

-100.358100	56.549350	406.536300	.605710
-98.756940	56.996240	406.532200	.606595
-97.128790	57.423640	406.599500	.682825
-95.469860	57.830970	406.741600	.851399
-93.777320	58.217490	406.937300	1.150115
-92.049300	58.582370	407.037300	1.758036
-90.284800	58.924680	406.528200	3.379644
-88.483640	59.243460	404.047200	7.576151
-86.646400	59.537720	393.439400	19.349680
-84.774360	59.806450	350.665500	50.187630
-82.869400	60.048700	277.839500	95.231310
-80.933970	60.263580	360.184300	42.278760
-78.971000	60.450230	482.292800	32.440110
-76.983890	60.607900	604.203200	73.442400
-74.976370	60.735940	686.973800	96.215210
-72.952470	60.833800	707.341600	104.168600
-70.916480	60.901050	718.779500	103.941800
-68.872850	60.937420	688.331700	98.642810
-66.826130	60.942730	585.691200	68.117110
-64.780930	60.916960	491.344200	37.565070
-62.741830	60.860230	439.977300	18.435000
-60.713310	60.772790	419.677600	8.927016
-58.699720	60.654990	413.299800	4.801100
-56.705200	60.507380	409.475800	2.570644
-54.733590	60.330550	406.991800	.921845
-52.788400	60.125220	406.056000	.271463
-50.872730	59.892220	405.846900	.132812
-48.989200	59.632420	405.840300	.074020
-47.139900	59.346780	405.862700	.033734
-45.326320	59.036270	405.877300	.012729
-43.549270	58.701890	405.883400	.004010
-41.808860	58.344600	405.885300	.001105
-40.104370	57.965370	405.885900	.000261
-38.434220	57.565060	405.886000	.000095
-36.795950	57.144450	405.886100	.000113
-35.186060	56.704150	405.886100	.000085
-33.600030	56.244660	405.886200	.000052
-32.032190	55.766200	405.886200	.000039
-102.859900	56.896880	406.118700	.235855
-101.242000	57.379640	406.084100	.205621
-99.604390	57.842470	406.080700	.203372
-97.940930	58.285070	406.100400	.230283
-96.246660	58.706940	406.130600	.300376
-94.517820	59.107400	406.114500	.500205
-92.751850	59.485580	405.845300	1.163392
-90.947170	59.840560	404.714000	3.039144
-89.103220	60.171320	401.082200	7.691675
-87.220290	60.476800	387.906300	20.848170
-85.299550	60.755960	345.613100	49.430610
-83.342830	61.007760	288.836600	89.187870
-81.352710	61.231220	387.960800	29.494610
-79.332320	61.425420	496.921800	34.578300
-77.285290	61.589540	575.672700	60.117050
-75.215710	61.722870	603.248500	73.512050
-73.128000	61.824790	624.504900	81.637250
-71.026890	61.894870	688.779900	95.378010
-68.917300	61.932760	705.988900	98.120770
-66.804310	61.938290	592.610000	68.433270
-64.692990	61.911450	483.859700	33.164060
-62.588420	61.852340	428.356200	15.908890
-60.495590	61.761240	407.458600	9.537508
-58.419300	61.638580	404.574800	4.754861
-56.364090	61.484900	405.641400	1.519801
-54.334160	61.300890	405.986500	.390360
-52.333320	61.087320	405.925200	.120552
-50.364880	60.845090	405.877400	.048570
-48.431600	60.575160	405.872300	.025919

-46.535590	60.278550	405.878000	.012711
-44.678250	59.956310	405.882700	.005203
-42.860180	59.609490	405.884900	.001855
-41.081100	59.239120	405.885600	.000594
-39.339830	58.846180	405.885900	.000187
-37.634110	58.431540	405.886000	.000087
-35.960610	57.995940	405.886000	.000091
-34.314830	57.539980	405.886100	.000081
-32.690990	57.064010	405.886100	.000067
-31.082000	56.568130	405.886200	.000034
-103.849600	57.699570	405.953600	.075695
-102.187300	58.200280	405.941500	.063689
-100.509000	58.680030	405.938800	.061285
-98.806840	59.138710	405.939300	.071791
-97.074520	59.575940	405.924100	.130735
-95.307120	59.991050	405.824700	.364818
-93.501110	60.383240	405.422100	1.062157
-91.654170	60.751550	404.068800	2.876912
-89.765210	61.094930	399.775200	7.485704
-87.834170	61.412270	386.080900	20.715460
-85.861950	61.702450	359.080500	32.558490
-83.850420	61.964360	341.175600	45.490910
-81.802150	62.196920	438.040600	22.608190
-79.720490	62.399150	507.060100	34.894220
-77.609360	62.570130	509.354900	37.011120
-75.473210	62.709080	489.510900	35.894870
-73.316960	62.815350	505.362600	48.473950
-71.145790	62.888430	596.742700	69.156210
-68.965200	62.927950	672.625000	82.976260
-66.780790	62.933720	574.927100	59.674420
-64.598260	62.905720	473.199100	28.794480
-62.423240	62.844070	424.382400	13.359350
-60.261290	62.749080	408.398300	7.379292
-58.117710	62.621230	405.551000	3.277036
-55.997520	62.461110	405.767300	1.065127
-53.905320	62.269450	405.885600	.320330
-51.845240	62.047150	405.876400	.095706
-49.820820	61.795150	405.873800	.029218
-47.834930	61.514500	405.878200	.011532
-45.889670	61.206310	405.882300	.005243
-43.986290	60.871700	405.884600	.002211
-42.125070	60.511780	405.885400	.000835
-40.305270	60.127630	405.885900	.000289
-38.525020	59.720250	405.886000	.000131
-36.781200	59.290500	405.886100	.000104
-35.069400	58.839090	405.886100	.000100
-33.383770	58.366500	405.886100	.000088
-31.716990	57.872960	405.886100	.000070
-30.060130	57.358350	405.886300	.000099
-104.916500	58.490070	405.904100	.022189
-103.202300	59.010120	405.900100	.017692
-101.477600	59.507980	405.897900	.016860
-99.732440	59.983750	405.891800	.031570
-97.958720	60.437220	405.863200	.101761
-96.150010	60.867820	405.748400	.321878
-94.301540	61.274790	405.353700	.923021
-92.410060	61.657170	404.129500	2.396235
-90.473700	62.013880	400.450000	5.980441
-88.491920	62.343760	390.329300	14.627340
-86.465330	62.645610	377.596200	23.505060
-84.395630	62.918240	397.480300	21.460240
-82.285480	63.160470	464.864000	21.859930
-80.138370	63.371240	460.579600	18.615730
-77.958560	63.549540	413.250800	31.661460
-75.750930	63.694510	379.556300	50.723070
-73.520860	63.805420	393.691900	50.475470
-71.274150	63.881700	471.076100	45.166520

-69.016910 63.922960 552.786700 52.252170
-66.755400 63.928990 511.409600 40.057910
-64.495980 63.899750 451.972300 20.031430
-62.244980 63.835380 422.077800 9.330008
-60.008560 63.736250 410.890700 4.163640
-57.792640 63.602840 407.253900 1.742359
-55.602750 63.435840 406.215100 .666397
-53.443960 63.236060 405.936400 .230711
-51.320750 63.004450 405.878800 .073490
-49.236890 62.742080 405.875500 .022876
-47.195330 62.450080 405.880400 .007780
-45.198120 62.129630 405.883800 .002961
-43.246250 61.781950 405.885200 .001133
-41.339580 61.408210 405.885900 .000425
-39.476710 61.009530 405.886000 .000188
-37.654880 60.586900 405.886300 .000163
-35.869840 60.141170 405.886200 .000113
-34.115770 59.672970 405.886100 .000096
-32.385130 59.182660 405.886300 .000123
-30.668580 58.670220 405.886400 .000163
-28.954870 58.135290 405.886400 .000187
-106.073800 59.266680 405.890600 .005822
-104.297500 59.807790 405.889100 .004330
-102.518800 60.325180 405.887200 .007099
-100.725000 60.819250 405.880200 .025300
-98.905530 61.289990 405.852100 .085503
-97.052100 61.737010 405.752500 .260005
-95.158340 62.159620 405.438600 .707432
-93.219700 62.556880 404.532800 1.741220
-91.233350 62.927700 401.932700 4.114985
-89.198040 63.270870 397.325000 7.874405
-87.113920 63.585110 395.799000 13.676540
-84.982480 63.869130 419.254900 10.439330
-82.806390 64.121680 426.680200 14.457160
-80.589290 64.341560 376.254800 45.236240
-78.335770 64.527670 323.074400 83.483080
-76.051160 64.679080 314.874600 88.189510
-73.741460 64.794950 348.740400 60.730200
-71.413090 64.874680 407.016500 30.502140
-69.072890 64.917820 459.174700 24.997130
-66.727910 64.924110 451.572300 19.925710
-64.385280 64.893550 429.901500 11.725750
-62.052090 64.826280 416.441500 6.166963
-59.735260 64.722690 409.974500 2.927568
-57.441380 64.583340 407.227400 1.231266
-55.176610 64.408980 406.249300 .460284
-52.946510 64.200520 405.957700 .154734
-50.755950 63.959000 405.890000 .048159
-48.608900 63.685590 405.881200 .014893
-46.508400 63.381530 405.882900 .004988
-44.456330 63.048090 405.884900 .001828
-42.453320 62.686580 405.885700 .000675
-40.498620 62.298210 405.886100 .000268
-38.589950 61.884150 405.886200 .000152
-36.723370 61.445380 405.886300 .000136
-34.893150 60.982680 405.886400 .000138
-33.091610 60.496560 405.886300 .000145
-31.309040 59.987160 405.886300 .000117
-29.533510 59.454220 405.886400 .000183
-27.750760 58.896990 405.886400 .000171
-107.339200 60.027230 405.887200 .001349
-105.487000 60.591610 405.886600 .001652
-103.643800 61.130270 405.884700 .006197
-101.793500 61.644070 405.878000 .021861
-99.922550 62.133320 405.853900 .069336
-98.020040 62.597820 405.775900 .197422
-96.077510 63.037030 405.551000 .503762

-94.088710 63.450070 404.966100 1.135225
-92.049460 63.835860 403.587700 2.336434
-89.957580 64.193130 400.633200 4.187037
-87.812570 64.520550 398.336900 4.894032
-85.615560 64.816710 400.867300 10.063020
-83.369120 65.080260 383.668500 25.860500
-81.077050 65.309890 355.152600 46.097720
-78.744270 65.504390 341.358700 57.517650
-76.376630 65.662700 350.661000 49.839720
-73.980770 65.783920 373.850100 31.724890
-71.563890 65.867360 400.908300 15.140190
-69.133670 65.912500 422.173100 10.649030
-66.698060 65.919100 422.937800 9.040772
-64.265090 65.887100 416.989000 6.422751
-61.842790 65.816700 411.640900 3.799870
-59.438900 65.708320 408.392000 1.925482
-57.060850 65.562580 406.806100 .839576
-54.715470 65.380330 406.167700 .318335
-52.408880 65.162570 405.954600 .106049
-50.146370 64.910470 405.896900 .031742
-47.932120 64.625290 405.885600 .009058
-45.769150 64.308400 405.884900 .002858
-43.659080 63.961160 405.885600 .001066
-41.602020 63.584970 405.886000 .000409
-39.596350 63.181100 405.886200 .000184
-37.638600 62.750720 405.886300 .000152
-35.723270 62.294770 405.886400 .000115
-33.842640 61.813940 405.886400 .000165
-31.986660 61.308540 405.886400 .000163
-30.142740 60.778430 405.886400 .000165
-28.295590 60.222970 405.886400 .000173
-26.427080 59.640870 405.886500 .000196
-108.736700 60.768890 405.886500 .000702
-106.789100 61.359350 405.885900 .001880
-104.866900 61.921510 405.883800 .006710
-102.949500 62.456830 405.876700 .021874
-101.019200 62.966030 405.852700 .064753
-99.061880 63.449270 405.780900 .173045
-97.066180 63.906190 405.590100 .408777
-95.023590 64.336020 405.137400 .852924
-92.928180 64.737730 404.161500 1.546558
-90.776390 65.110020 402.299300 2.611191
-88.566850 65.451470 399.543400 4.727709
-86.300130 65.760610 395.096500 11.572400
-83.978550 66.035940 381.748800 23.137520
-81.606060 66.276030 370.354900 30.162070
-79.187900 66.479550 370.981800 29.997290
-76.730480 66.645290 378.576200 23.909440
-74.241170 66.772270 389.837600 15.803760
-71.728080 66.859700 402.342200 8.827139
-69.199870 66.907030 411.621300 6.046783
-66.665550 66.913940 413.825400 5.451162
-64.134220 66.880400 412.037700 4.211916
-61.614990 66.806620 409.514100 2.694180
-59.116610 66.693070 407.640400 1.453651
-56.647430 66.540450 406.599200 .670847
-54.215080 66.349720 406.131500 .268024
-51.826330 66.121990 405.956600 .093671
-49.486860 65.858530 405.902000 .028792
-47.201050 65.560780 405.888300 .007919
-44.971820 65.230190 405.885900 .002167
-42.800360 64.868270 405.885800 .000743
-40.685980 64.476460 405.886100 .000315
-38.625890 64.056100 405.886300 .000175
-36.614990 63.608330 405.886300 .000113
-34.645670 63.134020 405.886400 .000165
-32.707610 62.633670 405.886400 .000163

-30.787590	62.107330	405.886400	.000183
-28.869240	61.554470	405.886400	.000163
-26.932890	60.973880	405.886500	.000198
-24.955330	60.363580	405.886500	.000253

Q.20a

-89.453400	32.991660	429.701300	1.570431
-88.345930	33.191160	430.050400	1.797418
-87.228100	33.381660	431.256900	2.574723
-86.100230	33.562890	433.127700	3.772078
-84.962690	33.734530	435.371000	5.197033
-83.815900	33.896310	437.611400	6.614039
-82.660300	34.047950	439.505200	7.803463
-81.496410	34.189190	440.772000	8.571581
-80.324730	34.319790	441.039500	8.667441
-79.145840	34.439500	439.723800	7.774246
-77.960310	34.548100	436.400600	5.745800
-76.768760	34.645400	431.608400	3.140426
-75.571840	34.731200	427.401100	1.660873
-74.370180	34.805370	426.003500	1.614779
-73.164480	34.867740	427.239400	1.196666
-71.955430	34.918200	429.208700	1.411674
-70.743730	34.956650	430.507500	1.993830
-69.530080	34.983020	430.800400	2.155172
-68.315220	34.997260	430.363900	1.887309
-67.099880	34.999340	429.605100	1.399467
-65.884770	34.989250	428.836500	.889963
-64.670640	34.967020	428.246900	.487584
-63.458190	34.932690	427.884500	.231996
-62.248150	34.886320	427.702000	.097748
-61.041230	34.828000	427.624200	.037865
-59.838120	34.757850	427.595000	.014168
-58.639500	34.676010	427.584600	.005314
-57.446040	34.582610	427.580800	.002014
-56.258390	34.477850	427.579500	.000766
-55.077160	34.361910	427.578800	.000305
-53.902950	34.235010	427.578600	.000161
-52.736340	34.097380	427.578400	.000204
-51.577860	33.949260	427.578300	.000305
-50.428010	33.790910	427.578300	.000305
-49.287250	33.622600	427.578300	.000305
-48.156040	33.444620	427.578300	.000305
-47.034740	33.257250	427.578300	.000305
-45.923690	33.060790	427.578300	.000305
-44.823220	32.855560	427.578300	.000305

-89.701210	33.918400	431.376700	2.624791
-88.583520	34.125020	432.197100	3.121654
-87.455040	34.322360	434.601200	4.561929
-86.316120	34.510110	438.277000	6.739261
-85.167100	34.687960	442.663100	9.303846
-84.008420	34.855620	446.972000	11.807130
-82.840550	35.012790	450.537700	13.870270
-81.663990	35.159200	452.940900	15.207020
-80.479300	35.294580	453.609700	15.446040
-79.287040	35.418700	451.346400	13.940860
-78.087840	35.531310	444.967000	10.242690
-76.882320	35.632210	435.079100	5.191183
-75.671150	35.721200	426.126700	2.550320
-74.455030	35.798110	423.805500	2.729423
-73.234660	35.862810	427.278400	1.817833
-72.010770	35.915150	431.609900	2.805287
-70.784100	35.955040	434.099300	4.053850
-69.555380	35.982390	434.448800	4.274288
-68.325390	35.997170	433.403200	3.685903
-67.094890	35.999320	431.758400	2.697621
-65.864640	35.988860	430.117300	1.681893
-64.635410	35.965800	428.882200	.893403
-63.407950	35.930180	428.152900	.411166
-62.183030	35.882080	427.803300	.169577
-60.961370	35.821590	427.662000	.066531
-59.743720	35.748840	427.609700	.026229
-58.530780	35.663950	427.590600	.010456
-57.323260	35.567100	427.583300	.004062
-56.121820	35.458470	427.580200	.001531
-54.927120	35.338260	427.579100	.000554
-53.739770	35.206700	427.578700	.000204
-52.560380	35.064020	427.578600	.000115
-51.389480	34.910500	427.578400	.000244
-50.227610	34.746390	427.578300	.000305
-49.075240	34.571990	427.578300	.000305
-47.932830	34.387580	427.578300	.000305
-46.800740	34.193490	427.578300	.000305
-45.679330	33.990000	427.578300	.000305
-44.568900	33.777440	427.578300	.000305
-89.960660	34.843320	436.217400	5.539074
-88.832330	35.057210	438.454300	6.790293
-87.692770	35.261540	444.362200	10.026770
-86.542320	35.455950	453.360200	14.851330
-85.381340	35.640140	464.108000	20.487530
-84.210270	35.813810	474.467500	25.863490
-83.029580	35.976620	482.767000	30.148750
-81.839790	36.128310	488.352400	32.937080
-80.641480	36.268600	490.403400	33.575680
-79.435230	36.397220	485.921800	30.588150
-78.221690	36.513940	471.189200	22.480070
-77.001530	36.618520	445.682100	10.693090
-75.775430	36.710780	421.268700	4.969143
-74.544130	36.790520	417.197000	5.688171
-73.308360	36.857590	428.669600	3.665446
-72.068890	36.911860	440.194900	7.377413
-70.826490	36.953220	445.701300	10.153920
-69.581950	36.981590	445.809900	10.439860
-68.336070	36.996900	442.760300	8.880872
-67.089650	36.999130	438.369300	6.450338
-65.843500	36.988290	433.985800	3.947988
-64.598400	36.964370	430.751600	2.040293
-63.355190	36.927440	428.927100	.916577
-62.114630	36.877570	428.100500	.383008
-60.877510	36.814860	427.778700	.159884
-59.644590	36.739430	427.659200	.068645
-58.416640	36.651430	427.611200	.028803
-57.194370	36.551040	427.591100	.011427

-55.978490	36.438440	427.583000	.004265
-54.769680	36.313860	427.579900	.001547
-53.568590	36.177530	427.578900	.000539
-52.375820	36.029710	427.578600	.000200
-51.191970	35.870660	427.578400	.000193
-50.017550	35.700660	427.578300	.000263
-48.853080	35.520030	427.578300	.000276
-47.698980	35.329070	427.578300	.000289
-46.555660	35.128100	427.578300	.000295
-45.423470	34.917440	427.578300	.000301
-44.302690	34.697410	427.578300	.000305
-90.232250	35.766320	445.955900	10.968030
-89.092860	35.987640	451.483900	13.772230
-87.941790	36.199090	465.253700	20.510310
-86.779330	36.400320	486.485200	30.551180
-85.605870	36.591000	512.196800	42.247540
-84.421870	36.770790	536.433200	53.102040
-83.227800	36.939400	554.922800	61.431170
-82.024180	37.096500	567.234700	66.842940
-80.811620	37.241800	573.670400	68.506300
-79.590740	37.375040	566.929500	63.396430
-78.362190	37.495960	536.040500	47.167640
-77.126670	37.604320	471.833000	21.595290
-75.884930	37.699920	402.153400	11.154960
-74.637700	37.782550	398.031200	12.529730
-73.385770	37.852060	437.146500	9.211164
-72.129940	37.908310	465.222100	18.656000
-70.871030	37.951180	475.910000	24.115710
-69.609860	37.980580	473.803700	23.927010
-68.347290	37.996460	465.318500	20.148180
-67.084140	37.998780	454.045100	14.484300
-65.821270	37.987530	442.716100	8.630408
-64.559530	37.962740	434.628500	4.284991
-63.299760	37.924460	430.422500	1.900113
-62.042780	37.872770	428.682400	.835069
-60.789440	37.807780	428.037200	.388210
-59.540510	37.729610	427.781600	.180095
-58.296810	37.638420	427.664300	.078618
-57.059080	37.534390	427.610400	.031355
-55.828080	37.417740	427.589400	.012052
-54.604500	37.288680	427.582000	.004590
-53.389020	37.147460	427.579700	.001737
-52.182290	36.994370	427.578800	.000619
-50.984880	36.829660	427.578500	.000224
-49.797380	36.653650	427.578400	.000189
-48.620270	36.466650	427.578300	.000217
-47.454020	36.268990	427.578300	.000223
-46.299010	36.060990	427.578300	.000247
-45.155610	35.843010	427.578300	.000252
-44.024070	35.615370	427.578300	.000305
-90.516530	36.687310	462.228900	19.065000
-89.365660	36.916220	474.333100	24.399990
-88.202590	37.134950	503.723900	36.645270
-87.027650	37.343150	550.556500	55.136780
-85.841200	37.540460	609.579900	76.667370
-84.643710	37.726540	663.446800	96.098260
-83.435650	37.901050	701.092000	110.216800
-82.217600	38.063690	724.619200	119.670800
-80.990140	38.214140	744.413700	123.306800
-79.753940	38.352110	740.998300	116.963300
-78.509670	38.477340	686.145400	88.708530
-77.258070	38.589580	536.883900	40.034440
-75.999910	38.688600	331.960600	25.375020
-74.735980	38.774210	337.871700	28.133320
-73.467090	38.846230	473.717500	23.465220
-72.194090	38.904510	533.891100	41.612200
-70.917830	38.948930	547.204900	49.837850

-69.639210	38.979390	536.147500	48.663540
-68.359090	38.995840	514.692300	40.613630
-67.078360	38.998240	487.872600	28.826790
-65.797930	38.986590	459.669200	16.559010
-64.518690	38.960900	440.928500	7.773670
-63.241520	38.921240	432.526400	3.538416
-61.967320	38.867690	429.619700	1.768708
-60.696920	38.800350	428.585500	.916526
-59.431210	38.719360	428.080300	.454422
-58.170970	38.624900	427.791700	.202021
-56.917050	38.517150	427.654400	.083939
-55.670200	38.396340	427.604000	.034267
-54.431160	38.262690	427.588200	.014347
-53.200640	38.116470	427.582500	.006055
-51.979310	37.957960	427.580200	.002521
-50.767770	37.787460	427.579000	.000949
-49.566610	37.605300	427.578600	.000345
-48.376340	37.411790	427.578400	.000163
-47.197410	37.207270	427.578300	.000192
-46.030250	36.992090	427.578300	.000212
-44.875180	36.766620	427.578300	.000242
-43.732470	36.531200	427.578300	.000287
-90.814130	37.606180	484.357300	28.163920
-89.651300	37.842850	507.632200	36.394130
-88.475770	38.069030	564.547700	54.496340
-87.287830	38.284350	661.254800	82.390470
-86.087840	38.488450	794.803000	115.593300
-84.876290	38.680970	910.704800	144.382900
-83.653650	38.861550	978.591800	165.379300
-82.420500	39.029860	1012.935000	180.247700
-81.177470	39.185590	1067.664000	185.674500
-79.925230	39.328410	1093.472000	184.343600
-78.664510	39.458070	1033.817000	145.335400
-77.396060	39.574290	705.720100	53.431700
-76.120680	39.676830	54.327530	108.247800
-74.839220	39.765490	288.664500	141.363100
-73.552520	39.840080	613.582600	65.654740
-72.261480	39.900440	704.017000	71.507280
-70.967010	39.946460	702.833400	81.714580
-69.670030	39.978020	663.429100	81.117520
-68.371470	39.995060	615.736700	68.403110
-67.072270	39.997550	557.299400	48.006520
-65.773400	39.985470	487.011800	25.995870
-64.475770	39.958870	446.898600	12.219100
-63.180340	39.917780	433.877000	6.544097
-61.888020	39.862300	430.993700	3.754145
-60.599740	39.792560	429.858400	2.090311
-59.316390	39.708680	428.819900	1.065122
-58.038840	39.610860	428.086400	.489917
-56.767930	39.499290	427.750700	.217879
-55.504470	39.374200	427.647900	.100517
-54.249260	39.235840	427.613600	.046533
-53.003000	39.084480	427.597400	.022084
-51.766410	38.920440	427.587600	.010163
-50.540120	38.744010	427.582300	.004297
-49.324710	38.555530	427.579700	.001578
-48.120720	38.355340	427.578700	.000512
-46.928620	38.143810	427.578400	.000219
-45.748810	37.921300	427.578300	.000205
-44.581620	37.688170	427.578300	.000204
-43.427300	37.444810	427.578300	.000256
-91.125630	38.522790	505.973400	33.671290
-89.950410	38.767390	544.174300	42.127090
-88.761920	39.001200	641.574300	59.233590
-87.560450	39.223820	827.908300	92.962710
-86.346370	39.434880	1136.348000	143.791600
-85.120160	39.633990	1378.359000	179.098600

-83.882300	39.820790	1480.965000	196.242600
-82.633390	39.994930	1462.270000	188.429800
-81.374080	40.156070	1634.436000	243.903900
-80.105060	40.303890	1739.955000	250.571500
-78.827100	40.438090	1815.484000	253.091000
-77.540980	40.558400	1619.791000	319.816300
-76.247540	40.664560	164.930300	256.267500
-74.947690	40.756360	45.933070	115.539800
-73.642300	40.833600	1505.463000	757.865900
-72.332310	40.896110	1119.450000	236.315500
-71.018690	40.943760	1018.036000	137.726900
-69.702430	40.976440	905.062300	122.390600
-68.384490	40.994090	815.717300	106.282000
-67.065880	40.996670	704.299100	84.237820
-65.747600	40.984170	521.012700	41.093020
-64.430650	40.956610	436.944400	22.011770
-63.116020	40.914060	430.668000	13.953560
-61.804690	40.856610	434.018300	7.875371
-60.497620	40.784390	433.268700	4.471203
-59.195770	40.697540	430.586700	2.326461
-57.900040	40.596260	428.649900	1.120693
-56.611330	40.480750	427.960900	.554231
-55.330490	40.351270	427.810200	.281144
-54.058330	40.208070	427.728400	.153002
-52.795620	40.051450	427.664400	.079889
-51.543080	39.881710	427.621100	.038831
-50.301390	39.699200	427.596100	.016909
-49.071130	39.504250	427.584500	.006476
-47.852850	39.297230	427.580100	.002152
-46.647030	39.078520	427.578700	.000643
-45.454070	38.848490	427.578400	.000240
-44.274280	38.607530	427.578300	.000200
-43.107920	38.356030	427.578300	.000236
-91.451740	39.437030	512.473600	29.054580
-90.263630	39.689750	560.832300	30.868340
-89.061680	39.931370	695.366100	46.332890
-87.846150	40.161480	936.489400	94.494190
-86.617400	40.379670	1620.010000	229.456500
-85.375900	40.585540	2127.702000	350.481200
-84.122160	40.778730	2331.532000	351.799800
-82.856800	40.958850	2320.463000	315.677000
-81.580460	41.125550	2360.635000	300.333900
-80.293880	41.278500	2586.008000	359.112300
-78.997860	41.417370	2785.008000	395.670300
-77.693230	41.541890	2664.706000	420.713600
-76.380870	41.651780	1896.896000	387.392500
-75.061690	41.746810	890.853800	151.049700
-73.736660	41.826770	699.157200	177.358500
-72.406780	41.891490	606.747900	226.424100
-71.073040	41.940830	1250.189000	244.622700
-69.736500	41.974670	1133.795000	188.653100
-68.398190	41.992950	1049.827000	191.170900
-67.059160	41.995620	885.851600	203.216800
-65.720480	41.982670	385.693900	93.048860
-64.383220	41.954140	358.972700	62.067690
-63.048400	41.910080	419.033200	30.712110
-61.717090	41.850600	446.166800	15.220750
-60.390290	41.775820	442.641600	8.677657
-59.069010	41.685920	434.369800	4.783054
-57.754210	41.581080	429.339400	2.538072
-56.446830	41.461530	428.352600	1.326660
-55.147770	41.327530	428.402000	.780586
-53.857880	41.179360	428.175800	.481118
-52.577970	41.017320	427.914600	.266565
-51.308780	40.841740	427.741200	.131525
-50.051000	40.652980	427.646400	.057787
-48.805250	40.451390	427.602400	.022328

-47.572090 40.237360 427.585400 .007592
-46.352010 40.011290 427.579900 .002291
-45.145370 39.773560 427.578700 .000652
-43.952510 39.524580 427.578300 .000273
-42.773630 39.264750 427.578400 .000180
-91.793200 40.348750 485.391600 16.874200
-90.591710 40.609810 514.431000 22.832750
-89.375760 40.859430 642.492700 62.900600
-88.145600 41.097210 1107.562000 186.866200
-86.901570 41.322720 1545.088000 204.395100
-85.644150 41.535550 2014.275000 230.636500
-84.373860 41.735290 2479.111000 319.194200
-83.091290 41.921560 2636.576000 318.627900
-81.797160 42.093980 2803.116000 348.758500
-80.492230 42.252200 3104.815000 394.134800
-79.177290 42.395880 3421.296000 493.447600
-77.853240 42.524730 3277.073000 486.210700
-76.521000 42.638450 2304.577000 192.639300
-75.181550 42.736820 1300.051000 129.248700
-73.835900 42.819590 465.947500 302.567300
-72.485100 42.886590 519.015700 181.031400
-71.130210 42.937670 1260.380000 355.282500
-69.772340 42.972710 950.854100 118.846300
-68.412590 42.991630 913.806400 130.354100
-67.052090 42.994400 486.884400 208.201400
-65.691960 42.980990 175.349300 154.997000
-64.333320 42.951450 157.399600 147.870200
-62.977290 42.905830 433.788400 52.483660
-61.624970 42.844260 498.439200 29.786780
-60.277440 42.766850 469.951400 25.364950
-58.935750 42.673790 438.237100 9.147752
-57.600940 42.565280 425.222400 8.424522
-56.273990 42.441570 428.107100 3.720256
-54.955840 42.302930 431.550900 3.118369
-53.647390 42.149640 429.604600 1.425187
-52.349470 41.982030 428.669400 .779703
-51.062890 41.800450 428.096000 .384505
-49.788320 41.605260 427.792300 .168896
-48.526440 41.396860 427.654300 .065740
-47.277780 41.175640 427.601000 .022572
-46.042850 40.942000 427.583800 .006927
-44.822010 40.696380 427.579300 .001963
-43.615560 40.439180 427.578400 .000571
-42.423690 40.170830 427.578300 .000303
-92.150770 41.257820 425.180600 23.719680
-90.935390 41.527410 358.752800 58.278340
-89.704910 41.785250 159.087900 175.706300
-88.459540 42.030900 402.982100 250.706500
-87.199620 42.263930 596.801500 217.227000
-85.925610 42.483890 1463.515000 132.006500
-84.638040 42.690380 2323.589000 299.881300
-83.337520 42.882970 2838.774000 400.090100
-82.024790 43.061280 3225.592000 457.705400
-80.700610 43.224930 3642.816000 504.742600
-79.365870 43.373580 3737.275000 477.430100
-78.021450 43.506890 3567.219000 465.841200
-76.668370 43.624570 3036.105000 489.285900
-75.307630 43.726360 1843.615000 158.956700
-73.940300 43.812040 1530.696000 673.770200
-72.567500 43.881390 260.699100 234.669500
-71.190370 43.934270 743.857700 111.668300
-69.810050 43.970550 1308.870000 239.393100
-68.427750 43.990140 1330.570000 162.199200
-67.044650 43.993000 852.689500 149.109000
-65.661930 43.979120 722.127800 147.850100
-64.280810 43.948540 438.927300 121.371500
-62.902460 43.901310 681.678000 112.744700

-61.528050	43.837570	629.975500	88.399670
-60.158730	43.757450	427.500200	45.296390
-58.795620	43.661140	440.986900	24.134180
-57.439790	43.548850	433.383200	11.940170
-56.092310	43.420850	447.196800	16.488480
-54.754150	43.277410	436.909600	5.973508
-53.426270	43.118850	433.131800	3.593480
-52.109530	42.945500	430.519800	1.969799
-50.804760	42.757750	428.953900	.967585
-49.512690	42.555960	428.142800	.423897
-48.233980	42.340550	427.778900	.165190
-46.969190	42.111940	427.638800	.057347
-45.718810	41.870550	427.593300	.017831
-44.483210	41.616830	427.581100	.005103
-43.262650	41.351200	427.578600	.001444
-42.057300	41.074100	427.578300	.000623
-92.525320	42.164070	408.866800	24.061500
-91.295520	42.442420	343.775800	53.315990
-90.049940	42.708690	153.241800	130.526000
-88.788760	42.962440	81.323090	142.585400
-87.512310	43.203190	661.476200	217.918000
-86.221000	43.430500	1232.611000	214.214100
-84.915410	43.643920	2154.214000	379.460500
-83.596150	43.843030	2727.138000	377.488300
-82.263960	44.027400	3283.292000	442.559200
-80.919660	44.196660	4091.580000	702.892500
-79.564150	44.350410	4172.865000	664.780200
-78.198390	44.488330	3746.153000	552.931500
-76.823410	44.610100	2808.721000	337.068600
-75.440290	44.715440	1694.934000	297.777800
-74.050180	44.804110	98.999120	182.014300
-72.654240	44.875890	131.316200	76.094560
-71.253690	44.930630	377.105900	179.322700
-69.849760	44.968180	1435.855000	180.249500
-68.443720	44.988460	2166.630000	473.711600
-67.036810	44.991420	1490.466000	220.195900
-65.630330	44.977050	1024.206000	131.217300
-64.225530	44.945390	811.749400	93.788930
-62.823690	44.896510	884.882800	171.352500
-61.426040	44.830540	655.384600	60.002410
-60.033810	44.747610	433.413100	77.550960
-58.648170	44.647940	426.477700	42.083450
-57.270290	44.531750	471.230000	34.999290
-55.901260	44.399320	457.123900	17.700120
-54.542130	44.250930	449.405000	12.728650
-53.193900	44.086940	440.725900	7.829966
-51.857480	43.907680	434.458100	4.344476
-50.533710	43.713560	430.715000	2.118036
-49.223370	43.504980	428.837100	.919562
-47.927120	43.282370	428.023000	.357167
-46.645540	43.046150	427.713800	.124263
-45.379100	42.796800	427.612700	.039117
-44.128160	42.534760	427.585100	.011312
-42.892960	42.260480	427.579000	.003183
-41.673610	41.974410	427.578300	.001280
-92.917770	43.067330	436.499800	12.325620
-91.673000	43.354680	438.587900	17.925050
-90.411740	43.629630	512.968400	87.538280
-89.134110	43.891690	685.564200	299.015100
-87.840450	44.140390	42.341360	93.135920
-86.531140	44.375260	100.157200	141.307500
-85.206750	44.595830	1172.689000	157.036400
-83.867900	44.801650	2443.399000	370.973000
-82.515380	44.992280	3067.771000	456.204300
-81.150000	45.167310	3461.279000	490.708300
-79.772730	45.326340	3565.713000	528.345600
-78.384560	45.469020	3253.978000	482.725100

-76.986600	45.595010	2371.167000	293.160600
-75.579970	45.704020	925.383600	252.007300
-74.165890	45.795780	285.406600	183.815600
-72.745610	45.870080	120.829900	76.532630
-71.320400	45.926740	138.007000	202.653400
-69.891600	45.965610	1572.911000	182.262300
-68.460530	45.986610	2375.342000	550.538100
-67.028560	45.989670	1379.114000	128.313500
-65.597030	45.974800	1068.569000	123.648100
-64.167300	45.942020	933.818300	103.644600
-62.740720	45.891430	846.329700	84.360240
-61.318600	45.823140	791.044900	114.595700
-59.902260	45.737310	592.954700	55.054330
-58.492950	45.634170	578.801500	70.465320
-57.091900	45.513950	533.370600	46.264390
-55.700260	45.376930	500.577100	34.080730
-54.319140	45.223450	475.704200	23.831340
-52.949590	45.053840	455.980000	15.409080
-51.592580	44.868490	441.739500	8.482622
-50.248970	44.667810	433.720700	4.055941
-48.919560	44.452230	429.958100	1.727064
-47.605040	44.222190	428.404500	.664527
-46.305980	43.978160	427.832200	.231149
-45.022840	43.720610	427.644700	.072787
-43.755960	43.450020	427.592300	.021145
-42.505550	43.166850	427.580200	.005916
-41.271660	42.871580	427.578400	.002250
-93.329110	43.967430	458.641100	18.890240
-92.068780	44.264030	484.066800	30.721660
-90.791210	44.547890	554.334900	78.677280
-89.496510	44.818510	597.340100	191.409300
-88.184940	45.075390	918.245900	584.004600
-86.856880	45.318050	100.017400	120.540400
-85.512890	45.545990	1285.254000	191.677000
-84.153590	45.758730	2338.789000	402.411600
-82.779790	45.955820	2687.495000	405.374700
-81.392350	46.136820	2717.276000	383.975500
-79.992260	46.301310	2515.061000	305.919200
-78.580580	46.448910	2275.995000	278.071300
-77.158460	46.579270	1798.944000	192.033700
-75.727110	46.692070	1269.258000	111.002400
-74.287820	46.787040	866.423300	182.344500
-72.841890	46.863950	178.434600	183.467400
-71.390720	46.922590	611.870500	77.681120
-69.935700	46.962830	2186.517000	697.065200
-68.478260	46.984570	1838.667000	257.894800
-67.019850	46.987740	1247.802000	147.331500
-65.561920	46.972340	1005.303000	123.629100
-64.105920	46.938410	982.574400	142.259300
-62.653260	46.886040	816.146100	84.574810
-61.205390	46.815350	832.274000	124.481500
-59.763680	46.726520	782.565900	99.856930
-58.329470	46.619790	671.839800	70.959320
-56.904060	46.495390	600.796900	61.765280
-55.488670	46.353640	558.586000	51.782120
-54.084500	46.194880	523.998100	39.985790
-52.692630	46.019470	483.466000	26.468340
-51.314070	45.827840	452.739700	14.567600
-49.949730	45.620390	437.578500	6.773649
-48.600430	45.397590	431.326500	2.795517
-47.266860	45.159900	428.867800	1.058838
-45.949600	44.907820	427.979100	.367123
-44.649100	44.641840	427.688200	.115679
-43.365670	44.362450	427.603400	.033212
-42.099450	44.070140	427.582500	.009084
-40.850440	43.765410	427.578800	.003277
-93.760410	44.864160	474.023900	27.117130

-92.483920	45.170280	502.269700	40.624860
-91.189420	45.463320	540.145600	73.749210
-89.876960	45.742750	404.319900	65.248920
-88.546760	46.008070	89.091660	105.104400
-87.199170	46.258760	32.158630	74.967850
-85.834720	46.494300	1100.931000	216.365400
-84.454070	46.714200	1837.929000	322.835800
-83.058010	46.917960	2052.906000	269.689900
-81.647480	47.105130	2017.804000	250.451500
-80.223460	47.275260	1777.235000	212.353600
-78.787090	47.427960	1310.199000	147.976100
-77.339580	47.562840	867.204600	252.336800
-75.882230	47.679570	1024.741000	92.331300
-74.416370	47.777870	1230.705000	150.612300
-72.943440	47.857470	1329.225000	340.898900
-71.464880	47.918190	905.491400	135.205700
-69.982220	47.959850	1356.450000	152.857500
-68.496960	47.982350	1173.728000	160.262300
-67.010670	47.985630	859.119600	99.858910
-65.524890	47.969690	717.573500	75.011430
-64.041170	47.934570	841.061200	131.827500
-62.561040	47.880340	630.772900	78.972290
-61.086020	47.807170	684.072900	121.797200
-59.617590	47.715230	905.492900	212.707200
-58.157170	47.604770	777.030900	95.533060
-56.706150	47.476050	713.559300	103.404700
-55.265830	47.329400	672.769300	97.363460
-53.837470	47.165180	631.444200	82.122760
-52.422200	46.983780	531.210700	43.582240
-51.021090	46.785640	462.863600	19.804130
-49.635090	46.571200	439.927200	9.759978
-48.265020	46.340950	432.257100	3.952383
-46.911610	46.095370	429.293500	1.437006
-45.575410	45.834990	428.139300	.494244
-44.256860	45.560320	427.741100	.156006
-42.956210	45.271870	427.619500	.044723
-41.673580	44.970160	427.587100	.011824
-40.408870	44.655690	427.580300	.003989
-94.212860	45.757310	492.585100	35.721320
-92.919560	46.073230	526.277600	49.122640
-91.607470	46.375730	555.783800	73.665410
-90.276550	46.664250	433.349200	44.899220
-88.926960	46.938270	526.241300	168.263700
-87.559010	47.197250	683.093100	340.116500
-86.173240	47.440650	458.734800	118.474800
-84.770290	47.667940	745.609000	191.538200
-83.350960	47.878600	1413.386000	137.131000
-81.916200	48.072160	1625.112000	190.896800
-80.467090	48.248140	1354.855000	151.928600
-79.004780	48.406110	749.742500	227.276700
-77.530590	48.545680	636.651600	199.929200
-76.045850	48.666500	771.973300	116.030200
-74.552020	48.768250	975.565600	68.541100
-73.050600	48.850660	928.882600	79.368700
-71.543170	48.913520	960.947900	89.770940
-70.031330	48.956650	851.520000	86.814990
-68.516710	48.979950	610.991500	145.396700
-67.000980	48.983350	745.814200	163.197000
-65.485790	48.966840	565.001300	65.710150
-63.972820	48.930470	902.290100	302.789100
-62.463700	48.874330	171.063400	195.430400
-60.960050	48.798580	87.946040	138.926900
-59.463460	48.703410	513.582200	119.308100
-57.975440	48.589080	818.583300	184.588400
-56.497490	48.455880	694.684100	77.209000
-55.030970	48.304140	679.426900	82.955830
-53.577210	48.134270	691.131800	118.692900

-52.137430	47.946660	568.830600	58.165940
-50.712720	47.741790	469.868300	21.267120
-49.304080	47.520120	435.271200	11.734210
-47.912350	47.282170	432.768000	4.601412
-46.538250	47.028450	429.711700	1.710976
-45.182340	46.759510	428.336200	.583656
-43.845020	46.475880	427.811200	.186353
-42.526500	46.178100	427.641400	.053931
-41.226800	45.866700	427.593800	.014383
-39.945740	45.542200	427.582800	.004701
-94.687740	46.646660	520.111800	46.540270
-93.376940	46.972690	566.909200	64.631290
-92.046550	47.284930	567.042400	63.411930
-90.696440	47.582840	496.947000	53.891700
-89.326680	47.865840	328.423900	93.132670
-87.937550	48.133390	267.523800	122.034100
-86.529520	48.384910	197.369700	181.849500
-85.103260	48.619850	334.571300	280.637700
-83.659580	48.837660	1260.069000	150.147100
-82.199460	49.037830	1526.174000	220.256400
-80.724000	49.219880	1248.057000	131.821000
-79.234450	49.383340	894.399500	98.942920
-77.732160	49.527770	812.634300	81.035220
-76.218590	49.652830	782.841200	66.643300
-74.695260	49.758160	882.553800	65.164380
-73.163800	49.843490	885.216500	73.055400
-71.625870	49.908580	817.781500	74.087180
-70.083210	49.953250	741.953100	96.563770
-68.537570	49.977380	512.366000	161.599400
-66.990740	49.980900	444.327900	131.128800
-65.444490	49.963800	380.313500	149.462700
-63.900620	49.926140	783.834800	162.258200
-62.360880	49.868010	768.516700	291.646800
-60.827010	49.789570	570.109300	182.232500
-59.300730	49.691040	560.968800	87.655850
-57.783640	49.572690	735.390400	137.327300
-56.277320	49.434830	680.233300	79.462300
-54.783270	49.277820	680.899400	110.940200
-53.302860	49.102080	606.472600	66.952980
-51.837360	48.908040	539.766700	47.949570
-50.387950	48.696200	489.506200	31.502330
-48.955640	48.467040	460.322800	19.879180
-47.541310	48.221130	437.329400	5.059908
-46.145660	47.959000	430.806300	1.922314
-44.769230	47.681210	428.592500	.668206
-43.412380	47.388330	427.881300	.213275
-42.075270	47.080920	427.662100	.062723
-40.757810	46.759540	427.599800	.017068
-39.459720	46.424710	427.585200	.005810
-95.186440	47.531940	556.287200	58.929680
-93.857410	47.868410	614.892300	79.558780
-92.508000	48.190720	603.402700	70.791090
-91.137910	48.498310	546.875600	46.763890
-89.747150	48.790600	505.500400	41.732860
-88.335950	49.067010	550.310500	45.777780
-86.904710	49.326930	744.432600	104.459800
-85.454100	49.569790	993.022300	165.553100
-83.984930	49.795020	1320.518000	217.679100
-82.498220	50.002060	1335.936000	182.187100
-80.995120	50.190400	1171.406000	155.736300
-79.476920	50.359550	977.109400	117.294800
-77.945060	50.509060	924.149000	113.167000
-76.401090	50.638520	887.002900	87.157120
-74.846650	50.747580	984.622100	110.579200
-73.283450	50.835950	960.904500	100.360700
-71.713320	50.903350	964.243600	119.458900
-70.138080	50.949630	914.537200	110.533100

-68.559640	50.974620	657.277100	94.085880
-66.979900	50.978270	721.989100	88.324170
-65.400800	50.960560	693.661600	103.679400
-63.824260	50.921550	549.213300	68.321550
-62.252170	50.861340	138.065400	163.435000
-60.686400	50.780110	327.558700	95.455990
-59.128770	50.678080	670.993000	119.687400
-57.581030	50.555550	655.649500	70.697020
-56.044860	50.412850	653.890700	107.860500
-54.521820	50.250360	572.764400	58.645070
-53.013420	50.068520	523.764500	44.065350
-51.520960	49.867810	487.260600	28.848960
-50.045680	49.648740	461.074300	17.110570
-48.588630	49.411830	444.826000	9.152884
-47.150700	49.157670	434.682900	4.145596
-45.732590	48.886830	430.171600	1.647693
-44.334810	48.599900	428.458600	.593587
-42.957660	48.297460	427.853500	.196296
-41.601190	47.980120	427.657400	.059550
-40.265250	47.648430	427.599700	.017051
-38.949390	47.302930	427.585700	.006076
-95.710510	48.412890	600.570300	68.923230
-94.362470	48.760130	673.510600	88.781560
-92.993230	49.092850	657.482600	69.152850
-91.602360	49.410460	650.314300	54.789910
-90.189750	49.712350	706.209700	68.748060
-88.755540	49.997940	781.004800	96.110550
-87.300090	50.266570	841.312700	104.327100
-85.824040	50.517650	904.754500	111.111200
-84.328210	50.750570	1006.925000	134.458700
-82.813610	50.964740	1049.880000	154.197800
-81.281460	51.159630	986.029600	128.579900
-79.733130	51.334700	911.761400	120.722000
-78.170130	51.489480	836.571800	101.070700
-76.594090	51.623530	851.311700	94.490290
-75.006800	51.736490	964.180800	117.897800
-73.410070	51.828010	919.131900	98.796880
-71.805870	51.897850	914.156200	101.221000
-70.196170	51.945790	935.838600	117.491500
-68.582990	51.971680	764.205000	66.375130
-66.968440	51.975460	679.414600	60.789940
-65.354560	51.957120	775.863800	70.943770
-63.743440	51.916690	839.467000	125.631200
-62.137130	51.854320	649.969400	96.185870
-60.537630	51.770170	494.809100	63.188560
-58.946900	51.664510	645.879200	89.356700
-57.366820	51.537620	651.664200	104.313600
-55.799180	51.389870	554.786900	56.338500
-54.245650	51.221680	509.851500	43.563680
-52.707820	51.033510	479.505900	28.675980
-51.187080	50.825860	458.797000	17.916200
-49.684720	50.599280	445.170000	10.364910
-48.201800	50.354330	436.633000	5.484982
-46.739240	50.091630	431.609500	2.582575
-45.297720	49.811770	429.182300	1.080756
-43.877700	49.515370	428.154900	.408729
-42.479410	49.203060	427.766600	.140612
-41.102810	48.875440	427.634900	.044523
-39.747590	48.533100	427.594400	.013217
-38.413150	48.176570	427.584200	.004974
-96.261670	49.289190	647.670800	71.834570
-94.893750	49.647600	748.888100	92.241730
-93.503820	49.991080	773.790800	89.729650
-92.091320	50.319060	788.533400	101.683500
-90.655950	50.630910	747.967400	84.756210
-89.197760	50.925990	704.537000	72.785020
-87.717060	51.203660	701.530300	80.000810

-86.214420	51.463270	733.183300	96.110630
-84.690660	51.704190	816.459000	123.914400
-83.146820	51.925780	864.670400	111.696800
-81.584160	52.127480	936.345200	135.656400
-80.004110	52.308710	850.381700	104.128600
-78.408280	52.468990	748.547600	100.205600
-76.798410	52.607840	794.713700	102.175600
-75.176390	52.724850	956.459700	138.656100
-73.544200	52.819690	932.164600	128.221900
-71.903920	52.892050	841.897600	98.474500
-70.257710	52.941730	860.804100	111.192300
-68.607750	52.968570	820.931700	125.985800
-66.956280	52.972490	544.560200	91.913570
-65.305560	52.953480	743.192000	63.942410
-63.657820	52.911580	893.737200	125.124800
-62.015270	52.846950	883.078100	152.859200
-60.380080	52.759750	724.356100	109.553500
-58.754360	52.650280	664.568100	96.494070
-57.140120	52.518850	600.549600	68.791280
-55.539290	52.365840	514.309400	43.820990
-53.953660	52.191710	477.740300	30.226150
-52.384890	51.996940	456.554400	18.148190
-50.834480	51.782070	443.944600	10.362200
-49.303730	51.547700	436.373200	5.675066
-47.793770	51.294400	432.013100	2.933466
-46.305490	51.022820	429.603000	1.387474
-44.839570	50.733620	428.411600	.595959
-43.396390	50.427420	427.887900	.232162
-41.976090	50.104880	427.683300	.082631
-40.578500	49.766630	427.611000	.026899
-39.203150	49.413260	427.587900	.008267
-37.849210	49.045330	427.582000	.003247
-96.841810	50.160530	645.712100	65.154500
-95.453030	50.530490	751.852100	104.869100
-94.041510	50.885130	721.449800	78.418860
-92.606450	51.223850	668.945100	63.606920
-91.147380	51.546020	632.894700	63.220070
-89.664200	51.850960	604.925400	64.635540
-88.157130	52.138020	597.996300	69.235900
-86.626690	52.406500	616.223100	76.818880
-85.073680	52.655720	670.371300	81.969270
-83.499180	52.885050	781.959500	109.536700
-81.904440	53.093850	848.666100	117.440300
-80.290990	53.281520	858.837900	131.126800
-78.660530	53.447530	762.226000	107.262100
-77.014920	53.591380	743.418300	96.638140
-75.356160	53.712650	864.698100	119.269600
-73.686420	53.810940	776.839900	90.596580
-72.007930	53.885960	639.964300	75.048350
-70.322990	53.937460	511.849000	104.925200
-68.634010	53.965280	473.714200	129.799700
-66.943400	53.969350	290.594500	187.828000
-65.253590	53.949630	723.133900	110.861800
-63.567000	53.906200	793.514800	103.129100
-61.886040	53.839190	742.658900	105.395500
-60.213040	53.748820	643.923600	84.554300
-58.550290	53.635360	587.088700	61.192100
-56.899960	53.499180	566.451000	60.441540
-55.264110	53.340690	512.153400	40.454220
-53.644650	53.160350	462.057300	20.474920
-52.043340	52.958710	445.303500	11.869960
-50.461760	52.736320	436.488900	6.040370
-48.901280	52.493820	431.972600	3.038320
-47.363030	52.231850	429.664400	1.477732
-45.847900	51.951060	428.508100	.680068
-44.356530	51.652150	427.960000	.290275
-42.889220	51.335790	427.722200	.114238

-41.445980	51.002650	427.628000	.041224
-40.026490	50.653380	427.594200	.013709
-38.630060	50.288590	427.583000	.004292
-37.255620	49.908830	427.580300	.001727
-97.453060	51.026530	558.582600	48.251000
-96.042340	51.408490	583.564400	47.711580
-94.608220	51.774700	597.715800	56.628320
-93.149600	52.124570	604.696200	69.776440
-91.665800	52.457450	592.210100	70.198170
-90.156560	52.772640	566.155100	60.708540
-88.621980	53.069450	550.452800	56.070040
-87.062480	53.347160	551.364400	58.422460
-85.478850	53.605050	564.155500	62.719410
-83.872130	53.842430	574.174000	66.000850
-82.243660	54.058630	580.697800	81.278270
-80.595020	54.253030	665.498000	88.095210
-78.928000	54.425040	681.974900	92.917860
-77.244600	54.574130	691.415700	83.677760
-75.546970	54.699840	821.167700	114.719000
-73.837420	54.801750	759.827000	95.290490
-72.118360	54.879540	650.393700	73.219310
-70.392330	54.932960	520.376000	56.671700
-68.661900	54.961820	366.962900	145.491500
-66.929700	54.966030	337.223500	154.209500
-65.198370	54.945580	586.377000	52.496250
-63.470540	54.900540	645.231500	99.651710
-61.748820	54.831050	542.502000	68.077560
-60.035740	54.737340	468.045700	50.698260
-58.333780	54.619720	448.150100	36.154030
-56.645260	54.478570	486.829200	30.165010
-54.972420	54.314330	521.336400	63.213660
-53.317280	54.127510	454.913800	14.229900
-51.681740	53.918690	439.042000	7.419470
-50.067420	53.688470	432.551200	3.513983
-48.475770	53.437500	429.782800	1.600295
-46.907930	53.166490	428.538000	.722043
-45.364770	52.876130	427.980600	.313568
-43.846840	52.567130	427.738200	.129527
-42.354370	52.240220	427.637800	.050120
-40.887210	51.896080	427.598800	.018089
-39.444820	51.535370	427.585000	.006030
-38.026250	51.158730	427.580400	.001883
-36.630120	50.766700	427.579300	.000761
-98.097820	51.886770	497.243800	32.201550
-96.663910	52.281200	503.022300	33.006550
-95.206040	52.659450	508.711200	35.308750
-93.722790	53.020900	514.209800	38.248490
-92.213200	53.364910	526.603900	45.858240
-90.676770	53.690770	548.617400	61.861480
-89.113440	53.997730	558.646000	69.835530
-87.523560	54.285050	544.808100	59.304700
-85.907830	54.551970	527.038800	49.428040
-84.267300	54.797760	514.603700	54.406630
-82.603340	55.021710	472.520500	78.154270
-80.917590	55.223140	479.817700	88.292590
-79.211960	55.401440	629.486200	100.138700
-77.488560	55.556020	690.179300	80.269600
-75.749730	55.686380	791.587000	102.996200
-73.997920	55.792100	742.524700	91.513870
-72.235800	55.872810	680.496900	80.698720
-70.466090	55.928230	584.899300	57.255510
-68.691570	55.958180	498.390000	54.417940
-66.915130	55.962550	605.918300	64.367460
-65.139640	55.941330	664.678500	113.981700
-63.367970	55.894590	556.112600	59.202110
-61.602940	55.822490	470.356200	48.237270
-59.847310	55.725280	405.760000	43.494750

-58.103760	55.603290	369.127800	52.864620
-56.374820	55.456930	351.148700	65.706570
-54.662870	55.286670	375.018500	50.117100
-52.970100	55.093080	436.248200	10.147570
-51.298500	54.876750	431.563700	2.628562
-49.649800	54.638340	429.699100	1.476615
-48.025460	54.378550	428.531200	.725392
-46.426660	54.098120	427.980400	.322139
-44.854210	53.797780	427.739700	.135031
-43.308580	53.478290	427.639800	.053449
-41.789850	53.140400	427.600600	.020091
-40.297680	52.784830	427.585800	.007075
-38.831280	52.412250	427.580800	.002300
-37.389370	52.023280	427.579100	.000698
-35.970190	51.618480	427.578800	.000274
-98.778840	52.740810	461.702700	18.080020
-97.320270	53.148240	462.464200	18.319920
-95.837370	53.539010	464.897400	19.759640
-94.328290	53.912530	468.376400	22.018320
-92.791720	54.268120	474.427200	25.178650
-91.226900	54.605080	484.020600	29.514710
-89.633560	54.922620	497.840200	36.075490
-88.011890	55.219970	524.891000	52.742580
-86.362530	55.496330	546.198100	68.646870
-84.686490	55.750910	530.218100	60.434050
-82.985150	55.982970	490.107200	56.807020
-81.260250	56.191770	397.613400	112.846400
-79.513790	56.376650	489.643200	82.046390
-77.748020	56.536990	717.690600	105.187500
-75.965460	56.672260	794.830700	98.268230
-74.168790	56.781970	721.727100	85.330300
-72.360860	56.865740	702.968000	91.568840
-70.544630	56.923280	619.022600	58.958490
-68.723180	56.954370	649.216400	69.082600
-66.899620	56.958910	716.258500	125.654400
-65.077100	56.936880	591.666800	54.755450
-63.258730	56.888360	508.706900	40.593370
-61.447630	56.813510	462.202100	28.892340
-59.646790	56.712620	432.181100	19.831000
-57.859090	56.586040	421.551600	16.194450
-56.087300	56.434210	409.232100	14.389560
-54.333970	56.257640	413.591900	9.557652
-52.601470	56.056940	425.305600	2.827807
-50.891880	55.832740	427.993300	.691106
-49.207030	55.585770	428.091000	.441395
-47.548420	55.316760	427.875900	.249696
-45.917200	55.026500	427.716200	.120402
-44.314130	54.715760	427.634200	.051225
-42.739580	54.385350	427.599400	.020141
-41.193420	54.036030	427.585800	.007343
-39.675070	53.668560	427.580900	.002490
-38.183400	53.283620	427.579100	.000766
-36.716770	52.881820	427.578700	.000214
-35.272900	52.463700	427.578500	.000176
-99.499240	53.588130	442.991900	8.924485
-98.014280	54.009130	442.786600	8.868522
-96.504860	54.412960	443.759500	9.564333
-94.968610	54.799050	445.576400	10.906030
-93.403790	55.166730	448.673100	13.018210
-91.809290	55.515250	453.556100	16.066750
-90.184560	55.843850	460.750800	20.099530
-88.529620	56.151670	471.384100	25.162200
-86.844990	56.437890	483.123000	30.837690
-85.131650	56.701690	502.957800	44.561610
-83.390960	56.942230	520.386500	66.455660
-81.624720	57.158780	489.453900	76.774030
-79.835050	57.350580	411.899700	129.604700

-78.024370 57.516980 666.327200 81.337970
-76.195360 57.657400 816.556700 114.953600
-74.350940 57.771310 720.376600 81.705210
-72.494220 57.858320 700.669600 78.615770
-70.628410 57.918090 727.446400 90.595730
-68.756890 57.950390 722.443200 103.177700
-66.883060 57.955100 651.412300 70.999060
-65.010370 57.932220 551.119400 47.930130
-63.142240 57.881810 486.643200 30.824240
-61.282040 57.804080 452.858500 17.523500
-59.433060 57.699310 437.756000 10.043120
-57.598450 57.567890 434.597400 8.190274
-55.781200 57.410300 424.456200 5.727357
-53.984060 57.227100 423.316900 4.021635
-52.209560 57.018940 426.025800 1.648335
-50.459930 56.786500 427.240800 .523043
-48.737060 56.530560 427.585800 .153486
-47.042490 56.251900 427.631700 .067516
-45.377330 55.951360 427.611300 .035333
-43.742270 55.629770 427.593500 .016501
-42.137470 55.287960 427.584300 .006648
-40.562610 54.926740 427.580500 .002399
-39.016750 54.546860 427.579100 .000786
-37.498410 54.149020 427.578600 .000256
-36.005400 53.733830 427.578400 .000190
-34.534880 53.301770 427.578300 .000305
-100.262700 54.428120 433.979100 3.971696
-98.749250 54.863350 433.718200 3.863054
-97.211560 55.280850 434.086900 4.163356
-95.646590 55.680060 434.964700 4.849380
-94.052090 56.060360 436.478100 5.987435
-92.426510 56.420970 438.827500 7.673082
-90.768940 56.761100 442.188300 9.972974
-89.079190 57.079890 446.613100 12.886130
-87.357570 57.376450 451.128500 16.134910
-85.605000 57.649890 454.778700 19.924480
-83.822840 57.899360 452.473900 25.479460
-82.012920 58.124030 429.138300 39.982320
-80.177480 58.323120 491.500400 60.228140
-78.319110 58.495910 589.753100 73.434600
-76.440710 58.641750 799.421400 119.183000
-74.545430 58.760110 740.907100 79.445500
-72.636660 58.850530 746.072000 89.973240
-70.717930 58.912650 788.044600 114.576600
-68.792920 58.946230 685.160200 72.354970
-66.865380 58.951130 618.313800 66.569010
-64.939080 58.927340 528.131200 41.141360
-63.017780 58.874950 474.579900 24.757770
-61.105210 58.794160 444.013200 11.781320
-59.204930 58.685300 439.323400 6.855271
-57.320400 58.548780 437.963800 6.150937
-55.454840 58.385130 429.691900 2.610337
-53.611270 58.194950 427.176900 1.582858
-51.792370 57.978940 427.128900 .795641
-50.000500 57.737840 427.392000 .321876
-48.237630 57.472490 427.533900 .106369
-46.505310 57.183730 427.575100 .032154
-44.804610 56.872430 427.582000 .011450
-43.136050 56.539500 427.581100 .004727
-41.499610 56.185780 427.579700 .001918
-39.894630 55.812120 427.578900 .000718
-38.319800 55.419290 427.578500 .000269
-36.773090 55.007980 427.578400 .000145
-35.251730 54.578750 427.578300 .000213
-33.752120 54.132060 427.578300 .000305
-101.073700 55.260130 430.013300 1.605554
-99.529010 55.710320 429.853400 1.525942

-97.960900	56.142140	429.988400	1.647969
-96.365430	56.555100	430.374600	1.963367
-94.739640	56.948590	431.047500	2.497327
-93.081440	57.321860	432.057200	3.279872
-91.389490	57.674070	433.357000	4.308269
-89.663250	58.004340	434.557800	5.493535
-87.902820	58.311730	434.375300	6.743110
-86.109000	58.595320	429.854500	9.690223
-84.283100	58.854170	414.286900	17.285950
-82.427000	59.087400	351.881200	55.056460
-80.543050	59.294160	456.000400	29.685580
-78.634010	59.473690	527.806300	44.984870
-76.703000	59.625280	645.783300	67.778040
-74.753460	59.748340	761.131700	121.948100
-72.789070	59.842370	748.018600	96.187020
-70.813740	59.906970	747.477900	85.239330
-68.831480	59.941910	677.085400	71.127380
-66.846440	59.947010	601.051600	59.492030
-64.862770	59.922250	514.942600	36.045040
-62.884610	59.867760	467.584500	20.872930
-60.916040	59.783740	442.112300	9.500305
-58.961010	59.670550	436.133700	4.524973
-57.023270	59.528640	437.704800	5.765235
-55.106350	59.358580	430.761500	1.817177
-53.213500	59.161040	428.365100	.864856
-51.347600	58.936760	427.700700	.398472
-49.511150	58.686560	427.580500	.171025
-47.706190	58.411320	427.572400	.064440
-45.934240	58.111950	427.576700	.021545
-44.196280	57.789400	427.578600	.006621
-42.492650	57.444580	427.578900	.001944
-40.823030	57.078400	427.578700	.000623
-39.186360	56.691730	427.578400	.000257
-37.580830	56.285340	427.578400	.000165
-36.003750	55.859910	427.578300	.000194
-34.451600	55.415950	427.578300	.000196
-32.919830	54.953830	427.578300	.000305
-101.937200	56.083360	428.422000	.588443
-100.358100	56.549350	428.345900	.545964
-98.756940	56.996240	428.393600	.591560
-97.128790	57.423640	428.547200	.721464
-95.469860	57.830970	428.803900	.937289
-93.777320	58.217490	429.128800	1.229533
-92.049300	58.582370	429.333900	1.566312
-90.284800	58.924680	428.781000	2.049667
-88.483640	59.243460	425.716100	4.051844
-86.646400	59.537720	415.704400	11.335880
-84.774360	59.806450	388.865800	26.448370
-82.869400	60.048700	285.932800	97.430820
-80.933970	60.263580	425.882100	24.204390
-78.971000	60.450230	483.951300	27.966570
-76.983890	60.607900	494.457600	64.732130
-74.976370	60.735940	518.711700	63.991670
-72.952470	60.833800	570.930100	90.434780
-70.916480	60.901050	689.726200	81.751340
-68.872850	60.937420	683.368900	69.935800
-66.826130	60.942730	588.386800	50.901760
-64.780930	60.916960	505.272700	32.011300
-62.741830	60.860230	461.509600	18.206550
-60.713310	60.772790	440.258700	8.748218
-58.699720	60.654990	432.459800	3.336115
-56.705200	60.507380	432.593600	2.527121
-54.733590	60.330550	429.660300	1.102869
-52.788400	60.125220	428.317000	.536002
-50.872730	59.892220	427.809100	.237231
-48.989200	59.632420	427.644300	.098468
-47.139900	59.346780	427.596500	.037676

-45.326320 59.036270 427.583400 .013113
-43.549270 58.701890 427.579900 .004060
-41.808860 58.344600 427.578900 .001153
-40.104370 57.965370 427.578600 .000309
-38.434220 57.565060 427.578300 .000214
-36.795950 57.144450 427.578300 .000194
-35.186060 56.704150 427.578300 .000193
-33.600030 56.244660 427.578300 .000193
-32.032190 55.766200 427.578300 .000305
-102.859900 56.896880 427.843600 .195328
-101.242000 57.379640 427.812600 .176171
-99.604390 57.842470 427.827300 .190790
-97.940930 58.285070 427.876700 .235056
-96.246660 58.706940 427.940500 .301619
-94.517820 59.107400 427.939900 .386381
-92.751850 59.485580 427.602500 .641581
-90.947170 59.840560 426.118800 1.789936
-89.103220 60.171320 421.351300 5.435071
-87.220290 60.476800 407.846100 15.176030
-85.299550 60.755960 373.410200 35.231190
-83.342830 61.007760 259.356400 114.430700
-81.352710 61.231220 413.109100 22.667540
-79.332320 61.425420 466.300200 21.121240
-77.285290 61.589540 492.977300 56.859540
-75.215710 61.722870 194.090700 163.298500
-73.128000 61.824790 105.331800 137.045100
-71.026890 61.894870 594.554100 180.472200
-68.917300 61.932760 694.536300 94.692860
-66.804310 61.938290 575.927800 44.266290
-64.692990 61.911450 493.064500 27.256930
-62.588420 61.852340 453.393600 15.502430
-60.495590 61.761240 435.661900 7.851773
-58.419300 61.638580 430.220200 3.442950
-56.364090 61.484900 429.408500 1.291236
-54.334160 61.300890 428.527400 .577479
-52.333320 61.087320 427.988700 .279414
-50.364880 60.845090 427.736900 .129350
-48.431600 60.575160 427.634700 .055359
-46.535590 60.278550 427.597200 .021714
-44.678250 59.956310 427.584400 .007687
-42.860180 59.609490 427.580200 .002447
-41.081100 59.239120 427.578900 .000691
-39.339830 58.846180 427.578600 .000181
-37.634110 58.431540 427.578300 .000197
-35.960610 57.995940 427.578300 .000185
-34.314830 57.539980 427.578300 .000190
-32.690990 57.064010 427.578300 .000186
-31.082000 56.568130 427.578300 .000305
-103.849600 57.699570 427.653900 .058662
-102.187300 58.200280 427.642700 .050980
-100.509000 58.680030 427.645100 .053904
-98.806840 59.138710 427.651500 .063867
-97.074520 59.575940 427.633800 .085656
-95.307120 59.991050 427.494300 .211433
-93.501110 60.383240 426.925700 .705354
-91.654170 60.751550 425.042500 2.180911
-89.765210 61.094930 419.477400 6.229892
-87.834170 61.412270 403.953000 16.523770
-85.861950 61.702450 358.259300 43.691640
-83.850420 61.964360 294.025900 87.069300
-81.802150 62.196920 430.056200 17.530890
-79.720490 62.399150 511.017200 39.412120
-77.609360 62.570130 457.710600 31.242020
-75.473210 62.709080 343.889100 70.488040
-73.316960 62.815350 81.608790 118.570200
-71.145790 62.888430 101.353400 137.277700
-68.965200 62.927950 646.965300 174.489200

-66.780790 62.933720 537.127500 38.125780
-64.598260 62.905720 472.595900 20.456460
-62.423240 62.844070 445.411300 11.716960
-60.261290 62.749080 434.117900 5.740725
-58.117710 62.621230 429.788800 2.421695
-55.997520 62.461110 428.505600 .890911
-53.905320 62.269450 427.995800 .325371
-51.845240 62.047150 427.762900 .131523
-49.820820 61.795150 427.656200 .058995
-47.834930 61.514500 427.609300 .026385
-45.889670 61.206310 427.589900 .010879
-43.986290 60.871700 427.582200 .004010
-42.125070 60.511780 427.579600 .001303
-40.305270 60.127630 427.578700 .000370
-38.525020 59.720250 427.578400 .000166
-36.781200 59.290500 427.578300 .000185
-35.069400 58.839090 427.578300 .000190
-33.383770 58.366500 427.578300 .000186
-31.716990 57.872960 427.578300 .000190
-30.060130 57.358350 427.578300 .000305
-104.916500 58.490070 427.597900 .015912
-103.202300 59.010120 427.594000 .012997
-101.477600 59.507980 427.592200 .012605
-99.732440 59.983750 427.584100 .018677
-97.958720 60.437220 427.540000 .064530
-96.150010 60.867820 427.365100 .230377
-94.301540 61.274790 426.772700 .726407
-92.410060 61.657170 424.971300 2.094231
-90.473700 62.013880 419.873900 5.622345
-88.491920 62.343760 406.198500 13.921660
-86.465330 62.645610 368.684700 34.320470
-84.395630 62.918240 355.651900 50.295120
-82.285480 63.160470 491.565300 34.570220
-80.138370 63.371240 483.475300 22.867000
-77.958560 63.549540 421.904200 12.514310
-75.750930 63.694510 351.519300 30.271290
-73.520860 63.805420 279.573300 56.225730
-71.274150 63.881700 246.320400 59.184290
-69.016910 63.922960 405.141000 39.217340
-66.755400 63.928990 460.667700 25.426050
-64.495980 63.899750 446.713300 12.678610
-62.244980 63.835380 436.984600 7.089172
-60.008560 63.736250 431.501100 3.405129
-57.792640 63.602840 428.998100 1.456177
-55.602750 63.435840 428.079200 .559169
-53.443960 63.236060 427.761200 .195979
-51.320750 63.004450 427.650200 .065841
-49.236890 62.742080 427.608200 .024234
-47.195330 62.450080 427.590900 .010367
-45.198120 62.129630 427.583400 .004458
-43.246250 61.781950 427.580200 .001700
-41.339580 61.408210 427.579100 .000570
-39.476710 61.009530 427.578600 .000167
-37.654880 60.586900 427.578300 .000208
-35.869840 60.141170 427.578300 .000194
-34.115770 59.672970 427.578300 .000196
-32.385130 59.182660 427.578300 .000196
-30.668580 58.670220 427.578300 .000208
-28.954870 58.135290 427.578300 .000305
-106.073800 59.266680 427.582900 .003837
-104.297500 59.807790 427.581500 .002781
-102.518800 60.325180 427.578900 .004047
-100.725000 60.819250 427.567800 .016797
-98.905530 61.289990 427.523300 .062963
-97.052100 61.737010 427.368500 .204438
-95.158340 62.159620 426.885300 .599454
-93.219700 62.556880 425.498900 1.623784

-91.233350	62.927700	421.760000	4.102968
-89.198040	63.270870	412.305100	9.247457
-87.113920	63.585110	385.458200	26.398610
-84.982480	63.869130	430.427300	11.214300
-82.806390	64.121680	480.830600	25.656820
-80.589290	64.341560	443.088700	14.432480
-78.335770	64.527670	408.883500	15.312200
-76.051160	64.679080	375.185500	20.773460
-73.741460	64.794950	346.272100	32.323190
-71.413090	64.874680	348.929400	32.353860
-69.072890	64.917820	396.313600	21.268950
-66.727910	64.924110	426.412700	11.776070
-64.385280	64.893550	431.966600	6.803981
-62.052090	64.826280	430.953300	3.800308
-59.735260	64.722690	429.301400	1.886785
-57.441380	64.583340	428.273700	.829423
-55.176610	64.408980	427.817000	.325549
-52.946510	64.200520	427.653000	.115428
-50.755950	63.959000	427.602000	.036929
-48.608900	63.685590	427.587100	.011090
-46.508400	63.381530	427.582100	.003643
-44.456330	63.048090	427.580000	.001454
-42.453320	62.686580	427.579100	.000570
-40.498620	62.298210	427.578700	.000185
-38.589950	61.884150	427.578400	.000206
-36.723370	61.445380	427.578300	.000234
-34.893150	60.982680	427.578300	.000234
-33.091610	60.496560	427.578300	.000240
-31.309040	59.987160	427.578300	.000242
-29.533510	59.454220	427.578300	.000244
-27.750760	58.896990	427.578300	.000305
-107.339200	60.027230	427.579400	.000784
-105.487000	60.591610	427.578600	.000887
-103.643800	61.130270	427.576000	.004006
-101.793500	61.644070	427.565700	.015767
-99.922550	62.133320	427.528700	.053158
-98.020040	62.597820	427.411600	.158810
-96.077510	63.037030	427.078300	.428210
-94.088710	63.450070	426.228500	1.052056
-92.049460	63.835860	424.299000	2.313781
-89.957580	64.193130	420.597200	4.276211
-87.812570	64.520550	415.076600	7.172490
-85.615560	64.816710	447.473000	13.721500
-83.369120	65.080260	440.198000	12.553680
-81.077050	65.309890	427.987300	11.253440
-78.744270	65.504390	414.485700	7.670264
-76.376630	65.662700	400.652600	11.995530
-73.980770	65.783920	390.729900	18.136250
-71.563890	65.867360	392.796600	18.826770
-69.133670	65.912500	408.521700	13.323430
-66.698060	65.919100	421.676500	7.586825
-64.265090	65.887100	426.959900	4.142919
-61.842790	65.816700	428.193800	2.191489
-59.438900	65.708320	428.113600	1.071865
-57.060850	65.562580	427.840900	.473364
-54.715470	65.380330	427.672700	.189084
-52.408880	65.162570	427.604100	.068154
-50.146370	64.910470	427.583900	.022359
-47.932120	64.625290	427.579700	.006553
-45.769150	64.308400	427.578900	.001741
-43.659080	63.961160	427.578700	.000475
-41.602020	63.584970	427.578600	.000178
-39.596350	63.181100	427.578400	.000206
-37.638600	62.750720	427.578300	.000240
-35.723270	62.294770	427.578300	.000240
-33.842640	61.813940	427.578300	.000242
-31.986660	61.308540	427.578300	.000244

-30.142740 60.778430 427.578300 .000247
-28.295590 60.222970 427.578300 .000252
-26.427080 59.640870 427.578300 .000305
-108.736700 60.768890 427.578600 .000357
-106.789100 61.359350 427.577900 .001136
-104.866900 61.921510 427.575100 .004566
-102.949500 62.456830 427.564500 .016099
-101.019200 62.966030 427.529800 .050063
-99.061880 63.449270 427.430100 .138158
-97.066180 63.906190 427.175200 .339666
-95.023590 64.336020 426.614200 .741466
-92.928180 64.737730 425.615500 1.369906
-90.776390 65.110020 424.429300 1.876868
-88.566850 65.451470 425.114600 1.088220
-86.300130 65.760610 433.153900 4.683184
-83.978550 66.035940 431.953500 7.506008
-81.606060 66.276030 425.369200 6.564218
-79.187900 66.479550 417.589200 6.310334
-76.730480 66.645290 410.208900 9.500165
-74.241170 66.772270 405.844500 12.610770
-71.728080 66.859700 407.419100 12.709030
-69.199870 66.907030 414.633500 9.567670
-66.665550 66.913940 421.738000 5.891465
-64.134220 66.880400 425.725600 3.233915
-61.614990 66.806620 427.279900 1.652040
-59.116610 66.693070 427.663400 .791383
-56.647430 66.540450 427.668400 .351224
-54.215080 66.349720 427.616900 .142133
-51.826330 66.121990 427.588300 .052761
-49.486860 65.858530 427.579200 .017953
-47.201050 65.560780 427.578000 .005635
-44.971820 65.230190 427.578200 .001605
-42.800360 64.868270 427.578400 .000417
-40.685980 64.476460 427.578300 .000256
-38.625890 64.056100 427.578300 .000245
-36.614990 63.608330 427.578300 .000242
-34.645670 63.134020 427.578300 .000244
-32.707610 62.633670 427.578300 .000247
-30.787590 62.107330 427.578300 .000252
-28.869240 61.554470 427.578300 .000268
-26.932890 60.973880 427.578300 .000285
-24.955330 60.363580 427.578300 .000305

Q.21a
-89.453400 32.991660 450.930100 .896613

-88.345930	33.191160	451.024000	1.020424
-87.228100	33.381660	451.384800	1.492863
-86.100230	33.562890	451.978400	2.242207
-84.962690	33.734530	452.755300	3.174952
-83.815900	33.896310	453.623400	4.171574
-82.660300	34.047950	454.456500	5.098908
-81.496410	34.189190	455.114400	5.808288
-80.324730	34.319790	455.439200	6.100822
-79.145840	34.439500	455.215000	5.711116
-77.960310	34.548100	454.197800	4.471305
-76.768760	34.645400	452.355000	2.614046
-75.571840	34.731200	450.444600	1.294232
-74.370180	34.805370	449.660400	1.214058
-73.164480	34.867740	450.098800	.918277
-71.955430	34.918200	450.846500	.724050
-70.743730	34.956650	451.298000	1.038865
-69.530080	34.983020	451.374500	1.190055
-68.315220	34.997260	451.225000	1.074752
-67.099880	34.999340	450.991900	.808305
-65.884770	34.989250	450.763800	.515510
-64.670640	34.967020	450.597300	.281913
-63.458190	34.932690	450.501600	.134376
-62.248150	34.886320	450.456800	.057213
-61.041230	34.828000	450.438400	.022068
-59.838120	34.757850	450.431300	.007337
-58.639500	34.676010	450.428400	.001722
-57.446040	34.582610	450.427500	.001284
-56.258390	34.477850	450.427400	.001192
-55.077160	34.361910	450.427600	.000684
-53.902950	34.235010	450.427700	.000289
-52.736340	34.097380	450.427800	.000142
-51.577860	33.949260	450.427800	.000125
-50.428010	33.790910	450.427800	.000122
-49.287250	33.622600	450.427800	.000122
-48.156040	33.444620	450.427800	.000122
-47.034740	33.257250	450.427800	.000122
-45.923690	33.060790	450.427800	.000122
-44.823220	32.855560	450.427800	.000122
-89.701210	33.918400	451.520100	1.658566
-88.583520	34.125020	451.799400	1.958698
-87.455040	34.322360	452.686800	2.887807
-86.316120	34.510110	454.117800	4.316269
-85.167100	34.687960	455.978000	6.063242
-84.008420	34.855620	458.041000	7.899638
-82.840550	35.012790	460.012600	9.594515
-81.663990	35.159200	461.593600	10.914480
-80.479300	35.294580	462.459700	11.517880
-79.287040	35.418700	462.118200	10.871390
-78.087840	35.531310	459.834600	8.485694
-76.882320	35.632210	455.262500	4.784438
-75.671150	35.721200	450.190600	2.371452
-74.455030	35.798110	448.374200	2.460736
-73.234660	35.862810	449.889900	1.615791
-72.010770	35.915150	451.855600	1.551478
-70.784100	35.955040	452.867000	2.303752
-69.555380	35.982390	452.920400	2.568238
-68.325390	35.997170	452.471700	2.282378
-67.094890	35.999320	451.850000	1.699881
-65.864640	35.988860	451.249800	1.064970
-64.635410	35.965800	450.824000	.568260
-63.407950	35.930180	450.593700	.265813
-62.183030	35.882080	450.491400	.112483
-60.961370	35.821590	450.451100	.043434
-59.743720	35.748840	450.435000	.013511
-58.530780	35.663950	450.428200	.003029
-57.323260	35.567100	450.425800	.004776
-56.121820	35.458470	450.426100	.003871

-54.927120	35.338260	450.427000	.001982
-53.739770	35.206700	450.427500	.000739
-52.560380	35.064020	450.427700	.000221
-51.389480	34.910500	450.427800	.000123
-50.227610	34.746390	450.427800	.000121
-49.075240	34.571990	450.427800	.000122
-47.932830	34.387580	450.427800	.000122
-46.800740	34.193490	450.427800	.000122
-45.679330	33.990000	450.427800	.000122
-44.568900	33.777440	450.427800	.000122
-89.960660	34.843320	453.532200	3.877870
-88.832330	35.057210	454.500500	4.696043
-87.692770	35.261540	457.242500	6.901673
-86.542320	35.455950	461.580900	10.193220
-85.381340	35.640140	467.166200	14.140870
-84.210270	35.813810	473.283800	18.195510
-83.029580	35.976620	479.073100	21.874860
-81.839790	36.128310	483.765400	24.800560
-80.641480	36.268600	486.595500	26.282780
-79.435230	36.397220	486.110400	25.014050
-78.221690	36.513940	479.932300	19.641370
-77.001530	36.618520	465.697900	10.793080
-75.775430	36.710780	448.517600	6.039020
-74.544130	36.790520	443.490000	6.419514
-73.308360	36.857590	449.764500	3.770031
-72.068890	36.911860	456.148700	4.549443
-70.826490	36.953220	458.838400	6.351435
-69.581950	36.981590	458.629900	6.818924
-68.336070	36.996900	457.046600	5.999279
-67.089650	36.999130	454.990500	4.450226
-65.843500	36.988290	452.987500	2.735698
-64.598400	36.964370	451.608900	1.429603
-63.355190	36.927440	450.903400	.658775
-62.114630	36.877570	450.610500	.282747
-60.877510	36.814860	450.496100	.111043
-59.644590	36.739430	450.447400	.032348
-58.416640	36.651430	450.423800	.013613
-57.194370	36.551040	450.416200	.020419
-55.978490	36.438440	450.419500	.014874
-54.769680	36.313860	450.424400	.006840
-53.568590	36.177530	450.426800	.002218
-52.375820	36.029710	450.427600	.000524
-51.191970	35.870660	450.427800	.000123
-50.017550	35.700660	450.427800	.000128
-48.853080	35.520030	450.427800	.000122
-47.698980	35.329070	450.427800	.000120
-46.555660	35.128100	450.427800	.000122
-45.423470	34.917440	450.427800	.000122
-44.302690	34.697410	450.427800	.000122
-90.232250	35.766320	458.643400	8.355802
-89.092860	35.987640	461.667300	10.328850
-87.941790	36.199090	469.549800	15.135310
-86.779330	36.400320	481.798800	22.152810
-85.605870	36.591000	497.462900	30.397230
-84.421870	36.770790	514.338700	38.616430
-83.227800	36.939400	530.069300	45.945560
-82.024180	37.096500	542.933000	51.860370
-80.811620	37.241800	551.581200	55.408470
-79.590740	37.375040	552.041400	53.605530
-78.362190	37.495960	537.562300	42.186740
-77.126670	37.604320	494.913800	23.677380
-75.884930	37.699920	437.562400	15.548930
-74.637700	37.782550	424.447300	16.436260
-73.385770	37.852060	451.769800	10.458780
-72.129940	37.908310	470.870500	12.756110
-70.871030	37.951180	477.372200	16.775850
-69.609860	37.980580	475.553400	16.986060

-68.347290	37.996460	470.499500	14.774650
-67.084140	37.998780	464.182500	10.867160
-65.821270	37.987530	457.823800	6.544746
-64.559530	37.962740	453.627100	3.243375
-63.299760	37.924460	451.663000	1.483672
-62.042780	37.872770	450.909200	.648287
-60.789440	37.807780	450.615700	.263537
-59.540510	37.729610	450.472400	.072793
-58.296810	37.638420	450.396200	.058842
-57.059080	37.534390	450.373000	.075419
-55.828080	37.417740	450.394100	.049457
-54.604500	37.288680	450.416000	.019824
-53.389020	37.147460	450.425600	.005098
-52.182290	36.994370	450.427600	.000979
-50.984880	36.829660	450.427900	.000455
-49.797380	36.653650	450.427800	.000229
-48.620270	36.466650	450.427800	.000132
-47.454020	36.268990	450.427800	.000115
-46.299010	36.060990	450.427800	.000115
-45.155610	35.843010	450.427800	.000121
-44.024070	35.615370	450.427800	.000122
-90.516530	36.687310	469.765900	15.797410
-89.365660	36.916220	478.066800	19.889910
-88.202590	37.134950	498.464000	29.198760
-87.027650	37.343150	529.594400	42.416150
-85.841200	37.540460	569.551500	57.970990
-84.643710	37.726540	611.645800	72.875920
-83.435650	37.901050	649.731900	85.428530
-82.217600	38.063690	681.167600	95.932080
-80.990140	38.214140	705.200100	104.541000
-79.753940	38.352110	712.558200	102.966400
-78.509670	38.477340	685.148400	85.747310
-77.258070	38.589580	575.253000	46.805370
-75.999910	38.688600	366.158200	43.514260
-74.735980	38.774210	348.870000	39.544400
-73.467090	38.846230	464.309200	24.945200
-72.194090	38.904510	517.282400	35.633830
-70.917830	38.948930	528.411300	39.181960
-69.639210	38.979390	520.148500	38.342680
-68.359090	38.995840	505.882800	32.745810
-67.078360	38.998240	488.699400	24.273720
-65.797930	38.986590	469.292900	13.608080
-64.518690	38.960900	457.877200	6.439857
-63.241520	38.921240	453.149200	2.804546
-61.967320	38.867690	451.572800	1.338901
-60.696920	38.800350	450.905700	.565947
-59.431210	38.719360	450.508900	.177596
-58.170970	38.624900	450.266900	.213269
-56.917050	38.517150	450.199700	.247385
-55.670200	38.396340	450.312300	.134938
-54.431160	38.262690	450.397600	.044220
-53.200640	38.116470	450.425600	.008707
-51.979310	37.957960	450.429800	.004047
-50.767770	37.787460	450.428900	.002533
-49.566610	37.605300	450.428200	.000984
-48.376340	37.411790	450.427900	.000281
-47.197410	37.207270	450.427800	.000115
-46.030250	36.992090	450.427800	.000112
-44.875180	36.766620	450.427800	.000120
-43.732470	36.531200	450.427800	.000122
-90.814130	37.606180	489.976000	25.765790
-89.651300	37.842850	510.001600	33.101650
-88.475770	38.069030	557.488900	49.241650
-87.287830	38.284350	628.536500	70.835310
-86.087840	38.488450	723.173300	96.241420
-84.876290	38.680970	819.610500	118.604700
-83.653650	38.861550	902.863000	135.915100

-82.420500	39.029860	969.055000	154.878500
-81.177470	39.185590	1033.360000	167.601500
-79.925230	39.328410	1061.370000	180.077400
-78.664510	39.458070	1041.921000	157.664000
-77.396060	39.574290	814.906400	92.785370
-76.120680	39.676830	177.309500	169.412400
-74.839220	39.765490	123.512800	78.940250
-73.552520	39.840080	543.289600	46.276590
-72.261480	39.900440	652.068200	67.804130
-70.967010	39.946460	659.145300	76.532390
-69.670030	39.978020	626.427900	72.739970
-68.371470	39.995060	592.231500	63.327740
-67.072270	39.997550	549.886100	47.262130
-65.773400	39.985470	492.537800	22.785380
-64.475770	39.958870	463.828500	10.061630
-63.180340	39.917780	455.402500	4.718995
-61.888020	39.862300	452.986900	2.478690
-60.599740	39.792560	451.637200	1.247381
-59.316390	39.708680	450.507600	.471037
-58.038840	39.610860	449.743300	.719596
-56.767930	39.499290	449.532200	.808847
-55.504470	39.374200	450.101900	.269621
-54.249260	39.235840	450.394000	.069337
-53.003000	39.084480	450.446300	.029200
-51.766410	38.920440	450.441600	.022801
-50.540120	38.744010	450.433400	.010284
-49.324710	38.555530	450.429400	.003624
-48.120720	38.355340	450.428100	.001002
-46.928620	38.143810	450.427800	.000236
-45.748810	37.921300	450.427800	.000114
-44.581620	37.688170	450.427800	.000116
-43.427300	37.444810	450.427800	.000122
-91.125630	38.522790	518.824700	35.366130
-89.950410	38.767390	560.894200	47.132160
-88.761920	39.001200	664.080400	66.702240
-87.560450	39.223820	807.473500	98.153380
-86.346370	39.434880	1012.087000	124.423100
-85.120160	39.633990	1211.028000	149.729600
-83.882300	39.820790	1371.623000	174.293600
-82.633390	39.994930	1502.604000	191.342600
-81.374080	40.156070	1633.830000	228.955800
-80.105060	40.303890	1729.524000	256.589600
-78.827100	40.438090	1802.779000	246.148400
-77.540980	40.558400	1608.310000	190.818300
-76.247540	40.664560	671.752900	221.020200
-74.947690	40.756360	83.754370	165.535100
-73.642300	40.833600	804.152100	176.657800
-72.332310	40.896110	909.675000	95.754080
-71.018690	40.943760	922.269900	103.770400
-69.702430	40.976440	842.008200	107.622700
-68.384490	40.994090	778.707000	94.499570
-67.065880	40.996670	691.972100	62.531770
-65.747600	40.984170	514.006000	37.312260
-64.430650	40.956610	464.199800	11.243480
-63.116020	40.914060	457.330700	7.148321
-61.804690	40.856610	456.816000	5.172282
-60.497620	40.784390	453.554300	2.715861
-59.195770	40.697540	450.238200	1.523074
-57.900040	40.596260	447.781400	2.087533
-56.611330	40.480750	447.074100	1.810936
-55.330490	40.351270	450.000900	.736321
-54.058330	40.208070	450.598200	.287286
-52.795620	40.051450	450.564300	.166039
-51.543080	39.881710	450.485200	.080652
-50.301390	39.699200	450.446800	.032605
-49.071130	39.504250	450.433100	.010716
-47.852850	39.297230	450.429000	.002989

-46.647030	39.078520	450.428000	.000681
-45.454070	38.848490	450.427800	.000124
-44.274280	38.607530	450.427800	.000115
-43.107920	38.356030	450.427800	.000122
-91.451740	39.437030	545.621700	39.077680
-90.263630	39.689750	620.602200	49.199880
-89.061680	39.931370	812.044600	68.818130
-87.846150	40.161480	1042.724000	97.582780
-86.617400	40.379670	1343.160000	127.232700
-85.375900	40.585540	1630.161000	164.179200
-84.122160	40.778730	1913.385000	196.557600
-82.856800	40.958850	2126.479000	234.921900
-81.580460	41.125550	2337.997000	276.611100
-80.293880	41.278500	2552.159000	333.757100
-78.997860	41.417370	2720.733000	358.701700
-77.693230	41.541890	2590.042000	315.629600
-76.380870	41.651780	1906.948000	211.913900
-75.061690	41.746810	1110.946000	109.827600
-73.736660	41.826770	828.292800	156.881500
-72.406780	41.891490	806.637000	125.369900
-71.073040	41.940830	1082.851000	110.058300
-69.736500	41.974670	1051.726000	124.573100
-68.398190	41.992950	991.431300	110.107000
-67.059160	41.995620	780.816800	104.352100
-65.720480	41.982670	443.320400	41.191330
-64.383220	41.954140	426.587600	29.336890
-63.048400	41.910080	460.651300	14.210620
-61.717090	41.850600	469.884400	11.253020
-60.390290	41.775820	458.955800	5.756588
-59.069010	41.685920	448.685500	4.685528
-57.754210	41.581080	443.575900	5.220348
-56.446830	41.461530	444.159100	5.410527
-55.147770	41.327530	451.938000	2.022846
-53.857880	41.179360	451.814400	.893400
-52.577970	41.017320	450.946200	.542678
-51.308780	40.841740	450.598400	.223888
-50.051000	40.652980	450.478900	.081271
-48.805250	40.451390	450.441800	.026557
-47.572090	40.237360	450.431200	.007645
-46.352010	40.011290	450.428500	.001929
-45.145370	39.773560	450.427900	.000401
-43.952510	39.524580	450.427800	.000138
-42.773630	39.264750	450.427800	.000122
-91.793200	40.348750	543.452100	30.427820
-90.591710	40.609810	622.723000	26.361410
-89.375760	40.859430	816.575600	56.524990
-88.145600	41.097210	1032.053000	86.962740
-86.901570	41.322720	1207.154000	75.825400
-85.644150	41.535550	1439.009000	114.561700
-84.373860	41.735290	1863.495000	184.529500
-83.091290	41.921560	2193.012000	255.224200
-81.797160	42.093980	2548.814000	333.525800
-80.492230	42.252200	2969.646000	398.135400
-79.177290	42.395880	3247.389000	436.329100
-77.853240	42.524730	3105.542000	408.379900
-76.521000	42.638450	2493.935000	285.670600
-75.181550	42.736820	1716.814000	163.787700
-73.835900	42.819590	1137.434000	93.734930
-72.485100	42.886590	796.847000	126.577500
-71.130210	42.937670	1008.201000	116.244700
-69.772340	42.972710	996.675800	121.180100
-68.412590	42.991630	860.997100	108.101900
-67.052090	42.994400	333.945600	155.656100
-65.691960	42.980990	192.692900	132.633600
-64.333320	42.951450	384.999400	68.366680
-62.977290	42.905830	501.111200	29.856700
-61.624970	42.844260	513.590200	22.616470

-60.277440	42.766850	465.886400	15.894070
-58.935750	42.673790	449.285000	11.805010
-57.600940	42.565280	451.033100	9.172051
-56.273990	42.441570	462.401700	7.579761
-54.955840	42.302930	459.501900	4.410683
-53.647390	42.149640	453.842800	2.638522
-52.349470	41.982030	451.538200	1.176103
-51.062890	41.800450	450.785600	.462645
-49.788320	41.605260	450.538900	.173237
-48.526440	41.396860	450.460200	.058920
-47.277780	41.175640	450.436400	.018197
-46.042850	40.942000	450.430000	.005047
-44.822010	40.696380	450.428300	.001229
-43.615560	40.439180	450.427900	.000260
-42.423690	40.170830	450.427800	.000095
-92.150770	41.257820	499.282000	14.150850
-90.935390	41.527410	480.308500	38.843010
-89.704910	41.785250	380.392800	168.314300
-88.459540	42.030900	441.259400	201.370700
-87.199620	42.263930	746.630900	128.363600
-85.925610	42.483890	1228.653000	92.008170
-84.638040	42.690380	1938.045000	191.473300
-83.337520	42.882970	2454.061000	293.968900
-82.024790	43.061280	2898.322000	387.953600
-80.700610	43.224930	3353.905000	453.249400
-79.365870	43.373580	3495.046000	481.897800
-78.021450	43.506890	3288.545000	432.395000
-76.668370	43.624570	2679.402000	327.742600
-75.307630	43.726360	1807.975000	171.087700
-73.940300	43.812040	1017.115000	119.279500
-72.567500	43.881390	398.398700	150.200800
-71.190370	43.934270	610.104400	110.927400
-69.810050	43.970550	1058.513000	154.719200
-68.427750	43.990140	1218.617000	127.618700
-67.044650	43.993000	690.161300	104.977700
-65.661930	43.979120	635.985800	75.659450
-64.280810	43.948540	630.289100	52.942260
-62.902460	43.901310	647.295700	55.411180
-61.528050	43.837570	590.634400	41.542290
-60.158730	43.757450	456.617700	33.182840
-58.795620	43.661140	462.230800	23.886160
-57.439790	43.548850	473.857000	15.633840
-56.092310	43.420850	474.659900	13.325780
-54.754150	43.277410	462.495600	8.462222
-53.426270	43.118850	455.208400	4.400465
-52.109530	42.945500	452.269700	1.991497
-50.804760	42.757750	451.109900	.866701
-49.512690	42.555960	450.666400	.349810
-48.233980	42.340550	450.504600	.128007
-46.969190	42.111940	450.450400	.042354
-45.718810	41.870550	450.433800	.012616
-44.483210	41.616830	450.429200	.003349
-43.262650	41.351200	450.428000	.000788
-42.057300	41.074100	450.427900	.000244
-92.525320	42.164070	475.882700	12.328020
-91.295520	42.442420	458.410600	32.901310
-90.049940	42.708690	396.558200	102.175400
-88.788760	42.962440	509.064000	119.366900
-87.512310	43.203190	725.567800	114.985300
-86.221000	43.430500	965.465100	90.375050
-84.915410	43.643920	1654.657000	198.185700
-83.596150	43.843030	2366.812000	302.423600
-82.263960	44.027400	2989.102000	408.404900
-80.919660	44.196660	3584.964000	489.409700
-79.564150	44.350410	3679.204000	484.675800
-78.198390	44.488330	3279.938000	409.585000
-76.823410	44.610100	2415.841000	237.502700

-75.440290	44.715440	1282.125000	103.633800
-74.050180	44.804110	339.173800	161.160800
-72.654240	44.875890	123.016600	162.445100
-71.253690	44.930630	569.085700	102.957100
-69.849760	44.968180	1440.764000	172.652000
-68.443720	44.988460	1967.680000	257.763000
-67.036810	44.991420	1314.751000	141.631100
-65.630330	44.977050	988.075400	94.306740
-64.225530	44.945390	786.739100	70.588210
-62.823690	44.896510	716.299700	55.493900
-61.426040	44.830540	598.181000	40.545610
-60.033810	44.747610	442.160900	63.485840
-58.648170	44.647940	468.283300	37.846240
-57.270290	44.531750	501.219500	29.809430
-55.901260	44.399320	493.619000	24.560300
-54.542130	44.250930	469.031900	13.534130
-53.193900	44.086940	458.484300	6.522906
-51.857480	43.907680	454.025400	3.591005
-50.533710	43.713560	451.930800	1.697473
-49.223370	43.504980	450.990400	.718506
-47.927120	43.282370	450.618100	.274940
-46.645540	43.046150	450.485700	.094286
-45.379100	42.796800	450.443700	.029020
-44.128160	42.534760	450.431600	.008007
-42.892960	42.260480	450.428600	.001995
-41.673610	41.974410	450.428000	.000626
-92.917770	43.067330	471.125700	13.059810
-91.673000	43.354680	480.134300	16.620440
-90.411740	43.629630	513.342500	42.663480
-89.134110	43.891690	515.422400	116.345400
-87.840450	44.140390	189.769200	187.704000
-86.531140	44.375260	70.995430	138.571400
-85.206750	44.595830	1030.586000	168.308300
-83.867900	44.801650	2140.415000	277.247500
-82.515380	44.992280	2766.813000	384.624800
-81.150000	45.167310	3143.271000	420.852500
-79.772730	45.326340	3139.715000	407.626200
-78.384560	45.469020	2747.946000	309.271200
-76.986600	45.595010	1845.967000	136.579300
-75.579970	45.704020	766.874900	198.106100
-74.165890	45.795780	326.556700	150.779000
-72.745610	45.870080	129.286400	85.145780
-71.320400	45.926740	302.599800	204.836200
-69.891600	45.965610	1644.246000	202.101000
-68.460530	45.986610	2079.581000	269.443500
-67.028560	45.989670	1298.231000	162.496500
-65.597030	45.974800	945.108000	107.015000
-64.167300	45.942020	747.028700	62.513530
-62.740720	45.891430	696.884700	45.781630
-61.318600	45.823140	602.153000	41.670850
-59.902260	45.737310	518.098400	44.077320
-58.492950	45.634170	549.572500	44.412390
-57.091900	45.513950	548.912600	42.294620
-55.700260	45.376930	512.760300	33.587060
-54.319140	45.223450	473.585300	19.323190
-52.949590	45.053840	463.657300	13.164910
-51.592580	44.868490	458.032500	6.649757
-50.248970	44.667810	453.887400	3.317651
-48.919560	44.452230	451.750300	1.429406
-47.605040	44.222190	450.870700	.547536
-46.305980	43.978160	450.561100	.188763
-45.022840	43.720610	450.464100	.058516
-43.755960	43.450020	450.436700	.016328
-42.505550	43.166850	450.429800	.004161
-41.271660	42.871580	450.428300	.001322
-93.329110	43.967430	468.660100	18.532620
-92.068780	44.264030	480.212000	27.457650

-90.791210	44.547890	490.804200	60.737780
-89.496510	44.818510	386.730500	149.015000
-88.184940	45.075390	341.088600	192.736600
-86.856880	45.318050	167.747300	123.410400
-85.512890	45.545990	1058.912000	104.951700
-84.153590	45.758730	2015.039000	231.181100
-82.779790	45.955820	2393.269000	296.247200
-81.392350	46.136820	2380.019000	315.393400
-79.992260	46.301310	2151.062000	242.819900
-78.580580	46.448910	1782.575000	140.730600
-77.158460	46.579270	1366.987000	75.431400
-75.727110	46.692070	1168.182000	89.572830
-74.287820	46.787040	1093.760000	189.872600
-72.841890	46.863950	267.482800	217.175900
-71.390720	46.922590	502.225700	181.335700
-69.935700	46.962830	1816.203000	292.485600
-68.478260	46.984570	1747.158000	206.420400
-67.019850	46.987740	1021.357000	128.491200
-65.561920	46.972340	778.702900	73.907620
-64.105920	46.938410	623.398600	44.549640
-62.653260	46.886040	649.820300	51.480340
-61.205390	46.815350	654.869800	42.028200
-59.763680	46.726520	632.354300	40.640460
-58.329470	46.619790	611.509700	49.412280
-56.904060	46.495390	572.849400	52.196930
-55.488670	46.353640	533.861200	44.762950
-54.084500	46.194880	512.062300	35.952670
-52.692630	46.019470	488.814000	23.622890
-51.314070	45.827840	469.022900	12.857110
-49.949730	45.620390	458.099600	6.007617
-48.600430	45.397590	453.186200	2.531292
-47.266860	45.159900	451.313200	.951454
-45.949600	44.907820	450.685200	.322838
-44.649100	44.641840	450.496200	.099306
-43.365670	44.362450	450.444400	.027696
-42.099450	44.070140	450.431500	.007092
-40.850440	43.765410	450.428800	.002263
-93.760410	44.864160	475.684000	24.870650
-92.483920	45.170280	489.276200	33.141650
-91.189420	45.463320	492.791400	49.885910
-89.876960	45.742750	337.476700	67.108260
-88.546760	46.008070	170.547000	129.133900
-87.199170	46.258760	112.058600	167.274900
-85.834720	46.494300	869.984000	96.735320
-84.454070	46.714200	1534.242000	153.806700
-83.058010	46.917960	1715.717000	190.158700
-81.647480	47.105130	1592.849000	172.973100
-80.223460	47.275260	1311.500000	114.020800
-78.787090	47.427960	853.914800	137.437700
-77.339580	47.562840	622.265900	193.232100
-75.882230	47.679570	955.860200	55.192250
-74.416370	47.777870	1388.375000	152.169000
-72.943440	47.857470	1280.886000	192.919500
-71.464880	47.918190	1100.273000	85.726150
-69.982220	47.959850	1427.111000	144.750200
-68.496960	47.982350	1083.141000	121.279600
-67.010670	47.985630	458.729100	112.072000
-65.524890	47.969690	286.759600	124.829200
-64.041170	47.934570	487.754500	45.779800
-62.561040	47.880340	529.336900	44.376500
-61.086020	47.807170	629.117700	59.136490
-59.617590	47.715230	699.156800	55.744050
-58.157170	47.604770	699.557000	64.566730
-56.706150	47.476050	662.904800	73.496850
-55.265830	47.329400	650.873200	70.223140
-53.837470	47.165180	633.730200	58.947430
-52.422200	46.983780	553.133200	38.206690

-51.021090 46.785640 490.327800 20.513220
-49.635090 46.571200 464.273400 9.284057
-48.265020 46.340950 454.978100 3.710573
-46.911610 46.095370 451.814800 1.356405
-45.575410 45.834990 450.817700 .451429
-44.256860 45.560320 450.529100 .137207
-42.956210 45.271870 450.452000 .038040
-41.673580 44.970160 450.433200 .009743
-40.408870 44.655690 450.429200 .003087
-94.212860 45.757310 491.141400 32.619240
-92.919560 46.073230 516.022700 41.312900
-91.607470 46.375730 544.220000 55.286520
-90.276550 46.664250 515.044200 44.460750
-88.926960 46.938270 583.003300 85.793560
-87.559010 47.197250 555.469500 122.087900
-86.173240 47.440650 339.434100 176.701000
-84.770290 47.667940 515.150100 187.358700
-83.350960 47.878600 1058.567000 94.082230
-81.916200 48.072160 1291.160000 134.612000
-80.467090 48.248140 1024.029000 61.734680
-79.004780 48.406110 508.977400 180.460600
-77.530590 48.545680 451.691500 177.802100
-76.045850 48.666500 699.203600 113.329300
-74.552020 48.768250 1093.904000 80.157390
-73.050600 48.850660 1136.548000 104.959900
-71.543170 48.913520 1088.114000 108.875400
-70.031330 48.956650 1129.025000 110.990800
-68.516710 48.979950 838.196400 69.716740
-67.000980 48.983350 372.538100 83.150190
-65.485790 48.966840 236.877700 93.166670
-63.972820 48.930470 570.015000 86.869510
-62.463700 48.874330 323.521600 130.968900
-60.960050 48.798580 315.444000 113.959000
-59.463460 48.703410 545.704200 43.373180
-57.975440 48.589080 680.352100 55.270320
-56.497490 48.455880 706.049400 72.660540
-55.030970 48.304140 722.359600 78.478100
-53.577210 48.134270 700.937600 75.940890
-52.137430 47.946660 588.004200 46.249760
-50.712720 47.741790 501.026200 23.705420
-49.304080 47.520120 466.925000 10.815300
-47.912350 47.282170 455.672000 4.179567
-46.538250 47.028450 451.995000 1.506396
-45.182340 46.759510 450.863600 .497460
-43.845020 46.475880 450.540100 .150294
-42.526500 46.178100 450.454600 .041547
-41.226800 45.866700 450.433700 .010618
-39.945740 45.542200 450.429300 .003369
-94.687740 46.646660 515.380100 42.725900
-93.376940 46.972690 547.400900 50.903480
-92.046550 47.284930 569.719200 57.401500
-90.696440 47.582840 553.189100 58.746640
-89.326680 47.865840 588.209500 58.254580
-87.937550 48.133390 552.841600 47.480520
-86.529520 48.384910 215.187100 150.086900
-85.103260 48.619850 308.393800 232.352000
-83.659580 48.837660 1117.031000 126.280700
-82.199460 49.037830 1354.382000 144.865400
-80.724000 49.219880 1182.831000 117.601400
-79.234450 49.383340 831.442300 71.505520
-77.732160 49.527770 752.887600 55.740900
-76.218590 49.652830 797.406900 69.026130
-74.695260 49.758160 1068.762000 81.650550
-73.163800 49.843490 1039.265000 113.040200
-71.625870 49.908580 987.349400 108.640200
-70.083210 49.953250 978.940600 90.495640
-68.537570 49.977380 784.832800 66.857250

-66.990740	49.980900	387.510300	88.777550
-65.444490	49.963800	187.279300	139.543000
-63.900620	49.926140	634.401700	103.860400
-62.360880	49.868010	603.763200	98.387750
-60.827010	49.789570	572.137300	54.046160
-59.300730	49.691040	602.578400	57.626790
-57.783640	49.572690	641.382000	64.148190
-56.277320	49.434830	645.527300	70.910800
-54.783270	49.277820	643.498700	75.850620
-53.302860	49.102080	597.724400	57.401830
-51.837360	48.908040	527.029600	38.087060
-50.387950	48.696200	482.185800	19.878290
-48.955640	48.467040	462.267700	8.723714
-47.541310	48.221130	454.443400	3.523084
-46.145660	47.959000	451.672700	1.287286
-44.769230	47.681210	450.781400	.428266
-43.412380	47.388330	450.520300	.130178
-42.075270	47.080920	450.450100	.036067
-40.757810	46.759540	450.432800	.009221
-39.459720	46.424710	450.429100	.002924
-95.186440	47.531940	548.733000	54.837330
-93.857410	47.868410	589.311000	61.158880
-92.508000	48.190720	599.422100	65.596060
-91.137910	48.498310	595.020100	64.761630
-89.747150	48.790600	634.067300	70.011360
-88.335950	49.067010	687.816200	62.788010
-86.904710	49.326930	722.864300	48.799510
-85.454100	49.569790	946.430300	118.908500
-83.984930	49.795020	1193.827000	137.899700
-82.498220	50.002060	1258.330000	163.203600
-80.995120	50.190400	1146.164000	136.087800
-79.476920	50.359550	963.168900	101.125700
-77.945060	50.509060	944.092000	88.755100
-76.401090	50.638520	993.818400	80.410810
-74.846650	50.747580	1168.078000	112.974100
-73.283450	50.835950	1083.852000	116.283100
-71.713320	50.903350	1043.014000	118.318600
-70.138080	50.949630	1020.657000	98.627660
-68.559640	50.974620	818.183800	63.552190
-66.979900	50.978270	654.328500	53.003170
-65.400800	50.960560	547.687700	73.348330
-63.824260	50.921550	499.596200	88.174770
-62.252170	50.861340	225.913800	160.484200
-60.686400	50.780110	444.961500	72.614590
-59.128770	50.678080	617.278400	64.809030
-57.581030	50.555550	624.660300	66.513630
-56.044860	50.412850	600.131200	76.489970
-54.521820	50.250360	559.526200	56.284950
-53.013420	50.068520	516.335600	41.324060
-51.520960	49.867810	484.644700	24.455190
-50.045680	49.648740	466.565200	12.816070
-48.588630	49.411830	457.104100	5.813239
-47.150700	49.157670	452.830800	2.404371
-45.732590	48.886830	451.201000	.893270
-44.334810	48.599900	450.653500	.301027
-42.957660	48.297460	450.488000	.092142
-41.601190	47.980120	450.442400	.025577
-40.265250	47.648430	450.431100	.006537
-38.949390	47.302930	450.428700	.002081
-95.710510	48.412890	595.786000	67.146000
-94.362470	48.760130	653.978300	73.176800
-92.993230	49.092850	679.072100	71.739530
-91.602360	49.410460	711.053600	70.145970
-90.189750	49.712350	774.564900	76.364860
-88.755540	49.997940	827.567000	84.422300
-87.300090	50.266570	857.421100	90.087110
-85.824040	50.517650	921.793800	108.559100

-84.328210	50.750570	974.963100	142.802500
-82.813610	50.964740	1005.410000	151.728100
-81.281460	51.159630	981.116900	129.069400
-79.733130	51.334700	894.897900	110.854500
-78.170130	51.489480	877.917800	96.670920
-76.594090	51.623530	964.177400	101.014100
-75.006800	51.736490	1134.881000	125.642200
-73.410070	51.828010	1021.189000	121.591200
-71.805870	51.897850	986.866900	113.969300
-70.196170	51.945790	1004.748000	97.813240
-68.582990	51.971680	828.699500	62.087190
-66.968440	51.975460	691.920200	48.405990
-65.354560	51.957120	736.221900	60.689090
-63.743440	51.916690	734.763100	83.203830
-62.137130	51.854320	563.323500	77.863360
-60.537630	51.770170	523.996500	81.518630
-58.946900	51.664510	607.498800	65.759190
-57.366820	51.537620	593.605400	70.456660
-55.799180	51.389870	538.000400	52.652710
-54.245650	51.221680	508.009200	37.740620
-52.707820	51.033510	489.053000	24.188860
-51.187080	50.825860	472.326600	14.541540
-49.684720	50.599280	460.180900	7.422902
-48.201800	50.354330	454.151400	3.509462
-46.739240	50.091630	451.738000	1.441200
-45.297720	49.811770	450.849800	.536865
-43.877700	49.515370	450.552300	.181674
-42.479410	49.203060	450.461300	.055547
-41.102810	48.875440	450.435900	.015380
-39.747590	48.533100	450.429600	.003889
-38.413150	48.176570	450.428300	.001231
-96.261670	49.289190	653.657700	72.968210
-94.893750	49.647600	756.559300	74.408150
-93.503820	49.991080	811.114200	79.758980
-92.091320	50.319060	825.824300	84.245970
-90.655950	50.630910	792.983500	79.412450
-89.197760	50.925990	741.276300	79.079630
-87.717060	51.203660	714.811500	86.174280
-86.214420	51.463270	727.576900	100.496900
-84.690660	51.704190	777.166000	118.221500
-83.146820	51.925780	842.226600	111.277200
-81.584160	52.127480	904.628900	114.016900
-80.004110	52.308710	858.815300	96.036330
-78.408280	52.468990	783.863000	100.219800
-76.798410	52.607840	867.703700	101.293900
-75.176390	52.724850	1059.159000	118.676600
-73.544200	52.819690	944.808700	112.398900
-71.903920	52.892050	914.629200	101.103100
-70.257710	52.941730	949.792400	85.559060
-68.607750	52.968570	824.115300	55.090460
-66.956280	52.972490	642.202900	46.989470
-65.305560	52.953480	739.852400	61.817790
-63.657820	52.911580	824.058400	89.048750
-62.015270	52.846950	774.181900	87.819050
-60.380080	52.759750	672.715000	76.312790
-58.754360	52.650280	616.902500	67.390140
-57.140120	52.518850	553.685700	50.282600
-55.539290	52.365840	491.187100	35.258340
-53.953660	52.191710	473.840700	21.240540
-52.384890	51.996940	466.522300	12.383730
-50.834480	51.782070	459.814500	7.553826
-49.303730	51.547700	454.710500	4.021251
-47.793770	51.294400	452.140200	1.837123
-46.305490	51.022820	451.045300	.758714
-44.839570	50.733620	450.630300	.283981
-43.396390	50.427420	450.488100	.095655
-41.976090	50.104880	450.444000	.029164

-40.578500	49.766630	450.431600	.008017
-39.203150	49.413260	450.428600	.001990
-37.849210	49.045330	450.428000	.000621
-96.841810	50.160530	662.185200	62.986230
-95.453030	50.530490	751.457000	77.088620
-94.041510	50.885130	749.312700	73.812790
-92.606450	51.223850	703.289600	73.156070
-91.147380	51.546020	653.535500	73.176330
-89.664200	51.850960	609.853600	70.916420
-88.157130	52.138020	588.281200	68.658090
-86.626690	52.406500	591.789600	71.404480
-85.073680	52.655720	629.930200	69.840470
-83.499180	52.885050	687.793200	81.556550
-81.904440	53.093850	729.252800	76.470810
-80.290990	53.281520	768.519800	82.587620
-78.660530	53.447530	724.937200	86.871710
-77.014920	53.591380	811.490800	93.119060
-75.356160	53.712650	972.611800	111.003500
-73.686420	53.810940	801.795200	99.742940
-72.007930	53.885960	763.494900	83.648410
-70.322990	53.937460	784.917100	58.428250
-68.634010	53.965280	707.848800	49.840630
-66.943400	53.969350	576.511300	71.334130
-65.253590	53.949630	729.631100	65.664330
-63.567000	53.906200	786.773200	90.973170
-61.886040	53.839190	747.370100	91.966160
-60.213040	53.748820	642.835600	76.315850
-58.550290	53.635360	565.403300	54.841640
-56.899960	53.499180	514.719300	35.919840
-55.264110	53.340690	471.671800	19.740660
-53.644650	53.160350	454.078000	11.790420
-52.043340	52.958710	453.845200	6.087816
-50.461760	52.736320	453.130900	3.328048
-48.901280	52.493820	451.873700	1.770322
-47.363030	52.231850	451.061800	.828124
-45.847900	51.951060	450.670500	.348086
-44.356530	51.652150	450.510200	.130304
-42.889220	51.335790	450.452700	.044054
-41.445980	51.002650	450.434500	.013385
-40.026490	50.653380	450.429300	.003640
-38.630060	50.288590	450.428100	.000878
-37.255620	49.908830	450.427900	.000281
-97.453060	51.026530	568.629500	53.159150
-96.042340	51.408490	603.812900	50.417690
-94.608220	51.774700	605.210700	58.860830
-93.149600	52.124570	595.764200	62.587420
-91.665800	52.457450	578.792100	59.771440
-90.156560	52.772640	554.993400	54.461810
-88.621980	53.069450	536.111100	49.432060
-87.062480	53.347160	522.642600	44.993560
-85.478850	53.605050	500.514400	45.041670
-83.872130	53.842430	437.656400	72.703680
-82.243660	54.058630	351.901400	122.707300
-80.595020	54.253030	505.163100	74.235280
-78.928000	54.425040	635.307900	69.542400
-77.244600	54.574130	779.151300	82.204260
-75.546970	54.699840	933.058800	107.159600
-73.837420	54.801750	750.973300	93.266960
-72.118360	54.879540	697.129200	68.586690
-70.392330	54.932960	627.797400	39.515640
-68.661900	54.961820	523.139400	74.828670
-66.929700	54.966030	529.470400	82.357570
-65.198370	54.945580	687.077400	54.423530
-63.470540	54.900540	724.008000	87.181240
-61.748820	54.831050	661.222700	81.689700
-60.035740	54.737340	569.786900	60.598420
-58.333780	54.619720	512.255100	38.610060

-56.645260	54.478570	489.299400	23.653700
-54.972420	54.314330	469.805500	13.665670
-53.317280	54.127510	448.292000	6.360588
-51.681740	53.918690	449.202900	3.355160
-50.067420	53.688470	450.652200	1.347125
-48.475770	53.437500	450.726600	.684215
-46.907930	53.166490	450.596900	.325756
-45.364770	52.876130	450.500900	.136598
-43.846840	52.567130	450.454200	.051787
-42.354370	52.240220	450.436200	.017553
-40.887210	51.896080	450.430100	.005309
-39.444820	51.535370	450.428300	.001405
-38.026250	51.158730	450.427900	.000332
-36.630120	50.766700	450.427800	.000115
-98.097820	51.886770	505.654900	31.390250
-96.663910	52.281200	511.308200	33.793000
-95.206040	52.659450	515.132600	35.369360
-93.722790	53.020900	517.588600	36.844470
-92.213200	53.364910	522.579300	39.594580
-90.676770	53.690770	530.647800	43.902370
-89.113440	53.997730	530.985200	45.065360
-87.523560	54.285050	514.759800	38.195560
-85.907830	54.551970	477.893700	31.121410
-84.267300	54.797760	384.066300	59.415120
-82.603340	55.021710	181.559000	157.434600
-80.917590	55.223140	181.836500	170.686500
-79.211960	55.401440	476.010100	89.352670
-77.488560	55.556020	761.267200	74.704970
-75.749730	55.686380	904.789400	102.685200
-73.997920	55.792100	742.601000	88.664220
-72.235800	55.872810	684.409600	57.374500
-70.466090	55.928230	521.333600	61.352160
-68.691570	55.958180	377.204600	139.281000
-66.915130	55.962550	544.239900	72.440140
-65.139640	55.941330	662.343000	63.921040
-63.367970	55.894590	645.673900	70.937640
-61.602940	55.822490	588.569800	63.624110
-59.847310	55.725280	521.782300	42.070570
-58.103760	55.603290	479.520900	24.830030
-56.374820	55.456930	467.783800	14.306110
-54.662870	55.286670	462.211400	7.790467
-52.970100	55.093080	452.072400	3.436177
-51.298500	54.876750	450.533100	1.174379
-49.649800	54.638340	450.435400	.564246
-48.025460	54.378550	450.459500	.268915
-46.426660	54.098120	450.454700	.116936
-44.854210	53.797780	450.442400	.048523
-43.308580	53.478290	450.434000	.018032
-41.789850	53.140400	450.429800	.006042
-40.297680	52.784830	450.428400	.001794
-38.831280	52.412250	450.427900	.000467
-37.389370	52.023280	450.427800	.000121
-35.970190	51.618480	450.427800	.000126
-98.778840	52.740810	473.583300	16.265850
-97.320270	53.148240	474.089300	16.474190
-95.837370	53.539010	475.246000	17.577810
-94.328290	53.912530	476.723300	19.392590
-92.791720	54.268120	480.474000	21.880420
-91.226900	54.605080	487.501100	25.213240
-89.633560	54.922620	496.318400	29.081630
-88.011890	55.219970	506.699300	33.963750
-86.362530	55.496330	503.597900	35.756960
-84.686490	55.750910	456.841400	37.029200
-82.985150	55.982970	336.168600	67.739070
-81.260250	56.191770	133.801600	150.387500
-79.513790	56.376650	252.873100	172.555800
-77.748020	56.536990	730.481600	80.524040

-75.965460 56.672260 877.870500 96.560510
-74.168790 56.781970 732.212900 82.920790
-72.360860 56.865740 659.468700 64.095540
-70.544630 56.923280 471.834700 112.752200
-68.723180 56.954370 463.855400 101.919100
-66.899620 56.958910 653.914500 59.143910
-65.077100 56.936880 621.757100 58.245120
-63.258730 56.888360 573.431600 58.937420
-61.447630 56.813510 534.121500 45.966110
-59.646790 56.712620 494.871300 27.751930
-57.859090 56.586040 462.745600 15.913980
-56.087300 56.434210 456.324900 8.790488
-54.333970 56.257640 454.645100 4.260737
-52.601470 56.056940 451.723100 1.638222
-50.891880 55.832740 450.669900 .599177
-49.207030 55.585770 450.447800 .242247
-47.548420 55.316760 450.422400 .099472
-45.917200 55.026500 450.426200 .042603
-44.314130 54.715760 450.428300 .016335
-42.739580 54.385350 450.428300 .005851
-41.193420 54.036030 450.428100 .001905
-39.675070 53.668560 450.427900 .000550
-38.183400 53.283620 450.427800 .000139
-36.716770 52.881820 450.427800 .000125
-35.272900 52.463700 450.427800 .000122
-99.499240 53.588130 459.245400 7.571713
-98.014280 54.009130 458.940400 7.398702
-96.504860 54.412960 459.293700 7.792557
-94.968610 54.799050 460.038900 8.601980
-93.403790 55.166730 461.643500 10.053010
-91.809290 55.515250 464.502700 12.384150
-90.184560 55.843850 468.843600 15.550160
-88.529620 56.151670 475.275800 19.176650
-86.844990 56.437890 479.771700 23.740850
-85.131650 56.701690 480.005200 29.594720
-83.390960 56.942230 458.557600 44.217310
-81.624720 57.158780 341.572900 67.788560
-79.835050 57.350580 258.287400 158.379100
-78.024370 57.516980 689.419700 77.833980
-76.195360 57.657400 847.719100 94.175150
-74.350940 57.771310 713.125900 80.435840
-72.494220 57.858320 642.183900 71.022320
-70.628410 57.918090 553.842500 75.490200
-68.756890 57.950390 589.932800 54.130630
-66.883060 57.955100 672.914600 57.780950
-65.010370 57.932220 601.490800 58.308390
-63.142240 57.881810 545.607100 50.267040
-61.282040 57.804080 497.828700 31.051380
-59.433060 57.699310 476.572000 21.147730
-57.598450 57.567890 455.028900 11.526550
-55.781200 57.410300 452.029400 5.770581
-53.984060 57.227100 451.636300 2.388950
-52.209560 57.018940 450.916500 .927128
-50.459930 56.786500 450.553200 .328411
-48.737060 56.530560 450.442900 .105968
-47.042490 56.251900 450.424000 .039342
-45.377330 55.951360 450.424700 .014826
-43.742270 55.629770 450.426600 .005669
-42.137470 55.287960 450.427500 .002010
-40.562610 54.926740 450.427700 .000652
-39.016750 54.546860 450.427800 .000203
-37.498410 54.149020 450.427800 .000120
-36.005400 53.733830 450.427800 .000119
-34.534880 53.301770 450.427800 .000122
-100.262700 54.428120 453.481600 3.170729
-98.749250 54.863350 453.252100 3.010657
-97.211560 55.280850 453.349500 3.128250

-95.646590	55.680060	453.676200	3.487879
-94.052090	56.060360	454.356000	4.152215
-92.426510	56.420970	455.526700	5.204164
-90.768940	56.761100	457.232000	6.694140
-89.079190	57.079890	459.299900	8.782149
-87.357570	57.376450	460.589700	11.672080
-85.605000	57.649890	460.811500	16.950320
-83.822840	57.899360	453.485500	23.611760
-82.012920	58.124030	416.384100	34.200810
-80.177480	58.323120	430.068700	48.991420
-78.319110	58.495910	686.876100	77.647610
-76.440710	58.641750	773.369800	88.544220
-74.545430	58.760110	691.083900	82.164770
-72.636660	58.850530	665.346200	73.641120
-70.717930	58.912650	648.248800	61.282310
-68.792920	58.946230	650.833300	52.689050
-66.865380	58.951130	657.782200	61.911380
-64.939080	58.927340	589.112600	54.008620
-63.017780	58.874950	530.271200	39.603650
-61.105210	58.794160	499.313800	32.083840
-59.204930	58.685300	470.869800	14.660940
-57.320400	58.548780	451.680800	10.406020
-55.454840	58.385130	450.557200	4.574055
-53.611270	58.194950	450.692400	1.613242
-51.792370	57.978940	450.571000	.569451
-50.000500	57.737840	450.470600	.191184
-48.237630	57.472490	450.434100	.060126
-46.505310	57.183730	450.426400	.017664
-44.804610	56.872430	450.426300	.005679
-43.136050	56.539500	450.427000	.002057
-41.499610	56.185780	450.427600	.000768
-39.894630	55.812120	450.427700	.000280
-38.319800	55.419290	450.427800	.000131
-36.773090	55.007980	450.427800	.000113
-35.251730	54.578750	450.427800	.000120
-33.752120	54.132060	450.427800	.000122
-101.073700	55.260130	451.397900	1.209565
-99.529010	55.710320	451.292100	1.111901
-97.960900	56.142140	451.317500	1.143387
-96.365430	56.555100	451.443900	1.288172
-94.739640	56.948590	451.707100	1.567023
-93.081440	57.321860	452.149700	2.007378
-91.389490	57.674070	452.760500	2.607065
-89.663250	58.004340	453.336200	3.339000
-87.902820	58.311730	453.240100	4.524230
-86.109000	58.595320	451.450900	7.311882
-84.283100	58.854170	443.154400	14.063310
-82.427000	59.087400	417.700300	26.700680
-80.543050	59.294160	484.649800	34.332240
-78.634010	59.473690	663.171800	69.625620
-76.703000	59.625280	602.715500	68.193120
-74.753460	59.748340	609.562800	72.176680
-72.789070	59.842370	692.747100	72.248530
-70.813740	59.906970	674.600400	61.365720
-68.831480	59.941910	687.853100	64.241840
-66.846440	59.947010	650.405600	58.478040
-64.862770	59.922250	575.076100	49.712330
-62.884610	59.867760	512.432900	32.774500
-60.916040	59.783740	489.303200	22.362190
-58.961010	59.670550	471.368800	14.704370
-57.023270	59.528640	453.467800	8.280112
-55.106350	59.358580	450.717400	3.734091
-53.213500	59.161040	450.543100	1.198598
-51.347600	58.936760	450.473400	.388504
-49.511150	58.686560	450.440900	.124966
-47.706190	58.411320	450.429500	.037777
-45.934240	58.111950	450.427100	.010573

-44.196280	57.789400	450.427100	.002793
-42.492650	57.444580	450.427500	.000854
-40.823030	57.078400	450.427600	.000328
-39.186360	56.691730	450.427800	.000144
-37.580830	56.285340	450.427800	.000114
-36.003750	55.859910	450.427800	.000116
-34.451600	55.415950	450.427800	.000122
-32.919830	54.953830	450.427800	.000122
-101.937200	56.083360	450.712000	.419033
-100.358100	56.549350	450.672800	.373541
-98.756940	56.996240	450.679200	.381312
-97.128790	57.423640	450.723500	.436062
-95.469860	57.830970	450.814600	.541193
-93.777320	58.217490	450.960100	.694822
-92.049300	58.582370	451.123200	.868107
-90.284800	58.924680	451.116900	1.069068
-88.483640	59.243460	450.415600	2.069277
-86.646400	59.537720	447.775400	5.866507
-84.774360	59.806450	437.336700	14.687440
-82.869400	60.048700	409.134100	27.000410
-80.933970	60.263580	485.108000	27.809520
-78.971000	60.450230	601.647600	62.933210
-76.983890	60.607900	351.490200	123.886600
-74.976370	60.735940	401.212600	82.495380
-72.952470	60.833800	550.495600	70.033000
-70.916480	60.901050	641.490000	62.076310
-68.872850	60.937420	712.653100	68.058380
-66.826130	60.942730	647.111100	58.279580
-64.780930	60.916960	552.256500	40.857650
-62.741830	60.860230	490.199700	23.930620
-60.713310	60.772790	466.430400	12.439390
-58.699720	60.654990	459.652800	6.032461
-56.705200	60.507380	452.190600	3.484567
-54.733590	60.330550	450.701400	1.705722
-52.788400	60.125220	450.474500	.624440
-50.872730	59.892220	450.436500	.213409
-48.989200	59.632420	450.428000	.071019
-47.139900	59.346780	450.427100	.022154
-45.326320	59.036270	450.427200	.006226
-43.549270	58.701890	450.427500	.001627
-41.808860	58.344600	450.427600	.000423
-40.104370	57.965370	450.427700	.000175
-38.434220	57.565060	450.427800	.000109
-36.795950	57.144450	450.427800	.000112
-35.186060	56.704150	450.427800	.000117
-33.600030	56.244660	450.427800	.000122
-32.032190	55.766200	450.427800	.000122
-102.859900	56.896880	450.504800	.131829
-101.242000	57.379640	450.491800	.113761
-99.604390	57.842470	450.493700	.115703
-97.940930	58.285070	450.507900	.134613
-96.246660	58.706940	450.535200	.167589
-94.517820	59.107400	450.567800	.202027
-92.751850	59.485580	450.551000	.239650
-90.947170	59.840560	450.270200	.660140
-89.103220	60.171320	449.125000	2.461668
-87.220290	60.476800	445.447500	7.589876
-85.299550	60.755960	431.795700	18.376680
-83.342830	61.007760	399.243300	30.431280
-81.352710	61.231220	458.898900	18.344910
-79.332320	61.425420	507.110500	44.226280
-77.285290	61.589540	308.586200	76.194900
-75.215710	61.722870	148.168200	125.274200
-73.128000	61.824790	165.958200	159.117800
-71.026890	61.894870	502.546400	82.202060
-68.917300	61.932760	707.968100	70.846870
-66.804310	61.938290	637.565100	56.567010

-64.692990 61.911450 526.284000 33.910870
-62.588420 61.852340 466.352700 18.743860
-60.495590 61.761240 445.216000 13.366430
-58.419300 61.638580 446.134400 6.311665
-56.364090 61.484900 448.877200 1.550013
-54.334160 61.300890 450.012200 .465183
-52.333320 61.087320 450.311900 .200201
-50.364880 60.845090 450.393000 .084597
-48.431600 60.575160 450.417400 .031664
-46.535590 60.278550 450.424800 .010555
-44.678250 59.956310 450.426900 .003179
-42.860180 59.609490 450.427500 .000862
-41.081100 59.239120 450.427700 .000246
-39.339830 58.846180 450.427800 .000118
-37.634110 58.431540 450.427800 .000111
-35.960610 57.995940 450.427800 .000114
-34.314830 57.539980 450.427800 .000120
-32.690990 57.064010 450.427800 .000122
-31.082000 56.568130 450.427800 .000122
-103.849600 57.699570 450.447000 .037603
-102.187300 58.200280 450.443300 .031349
-100.509000 58.680030 450.443800 .031705
-98.806840 59.138710 450.447500 .036751
-97.074520 59.575940 450.452400 .042426
-95.307120 59.991050 450.443800 .056412
-93.501110 60.383240 450.353800 .221313
-91.654170 60.751550 449.933700 .885041
-89.765210 61.094930 448.447800 2.969878
-87.834170 61.412270 443.425600 8.537135
-85.861950 61.702450 428.493600 19.196790
-83.850420 61.964360 405.978500 25.611120
-81.802150 62.196920 457.625200 16.137100
-79.720490 62.399150 460.099400 35.768130
-77.609360 62.570130 310.322700 81.235550
-75.473210 62.709080 158.869400 124.544600
-73.316960 62.815350 81.157660 121.483600
-71.145790 62.888430 181.986800 154.868900
-68.965200 62.927950 606.171300 83.795280
-66.780790 62.933720 593.865500 46.535440
-64.598260 62.905720 497.748700 27.328900
-62.423240 62.844070 457.946500 14.741020
-60.261290 62.749080 448.029700 8.393144
-58.117710 62.621230 448.000500 3.979793
-55.997520 62.461110 449.338500 1.372276
-53.905320 62.269450 450.023000 .412804
-51.845240 62.047150 450.289400 .137454
-49.820820 61.795150 450.383900 .047887
-47.834930 61.514500 450.414700 .016720
-45.889670 61.206310 450.424300 .005592
-43.986290 60.871700 450.426900 .001699
-42.125070 60.511780 450.427600 .000492
-40.305270 60.127630 450.427700 .000177
-38.525020 59.720250 450.427800 .000115
-36.781200 59.290500 450.427800 .000111
-35.069400 58.839090 450.427800 .000116
-33.383770 58.366500 450.427800 .000122
-31.716990 57.872960 450.427800 .000122
-30.060130 57.358350 450.427800 .000122
-104.916500 58.490070 450.432200 .009702
-103.202300 59.010120 450.431200 .007764
-101.477600 59.507980 450.431200 .007660
-99.732440 59.983750 450.431700 .008312
-97.958720 60.437220 450.429700 .013802
-96.150010 60.867820 450.408900 .062319
-94.301540 61.274790 450.302600 .258574
-92.410060 61.657170 449.875200 .901308
-90.473700 62.013880 448.417400 2.845075

-88.491920	62.343760	443.667900	7.561369
-86.465330	62.645610	432.025800	15.470260
-84.395630	62.918240	436.005200	17.356410
-82.285480	63.160470	483.878800	19.249420
-80.138370	63.371240	458.684400	23.433320
-77.958560	63.549540	355.929200	52.795590
-75.750930	63.694510	258.662700	86.834090
-73.520860	63.805420	184.992600	117.363100
-71.274150	63.881700	229.258900	94.087980
-69.016910	63.922960	459.667800	44.837230
-66.755400	63.928990	496.682200	32.863530
-64.495980	63.899750	472.563100	16.414630
-62.244980	63.835380	456.335200	7.564351
-60.008560	63.736250	450.962900	3.588903
-57.792640	63.602840	449.804700	1.728596
-55.602750	63.435840	449.947400	.722045
-53.443960	63.236060	450.194700	.281941
-51.320750	63.004450	450.335900	.104302
-49.236890	62.742080	450.396500	.037066
-47.195330	62.450080	450.418100	.012743
-45.198120	62.129630	450.425100	.004096
-43.246250	61.781950	450.427100	.001225
-41.339580	61.408210	450.427600	.000364
-39.476710	61.009530	450.427700	.000148
-37.654880	60.586900	450.427800	.000112
-35.869840	60.141170	450.427800	.000114
-34.115770	59.672970	450.427800	.000120
-32.385130	59.182660	450.427800	.000122
-30.668580	58.670220	450.427800	.000122
-28.954870	58.135290	450.427800	.000122
-106.073800	59.266680	450.428700	.002260
-104.297500	59.807790	450.428500	.001695
-102.518800	60.325180	450.428400	.001592
-100.725000	60.819250	450.428000	.003074
-98.905530	61.289990	450.424600	.014303
-97.052100	61.737010	450.404400	.060744
-95.158340	62.159620	450.314900	.220936
-93.219700	62.556880	449.974900	.728377
-91.233350	62.927700	448.887600	2.139032
-89.198040	63.270870	445.228500	5.266130
-87.113920	63.585110	440.907200	10.317690
-84.982480	63.869130	457.838200	7.764305
-82.806390	64.121680	471.159900	11.707850
-80.589290	64.341560	429.270000	22.156520
-78.335770	64.527670	377.661900	44.622280
-76.051160	64.679080	336.373500	63.961380
-73.741460	64.794950	318.244200	70.331770
-71.413090	64.874680	349.967500	47.121960
-69.072890	64.917820	426.535700	22.171630
-66.727910	64.924110	458.464000	12.793840
-64.385280	64.893550	457.320300	7.243212
-62.052090	64.826280	453.253900	3.620130
-59.735260	64.722690	451.048200	1.707578
-57.441380	64.583340	450.330000	.770227
-55.176610	64.408980	450.254800	.341446
-52.946510	64.200520	450.324500	.145975
-50.755950	63.959000	450.382500	.060197
-48.608900	63.685590	450.411200	.023538
-46.508400	63.381530	450.422300	.008484
-44.456330	63.048090	450.426300	.002756
-42.453320	62.686580	450.427400	.000833
-40.498620	62.298210	450.427700	.000254
-38.589950	61.884150	450.427800	.000125
-36.723370	61.445380	450.427800	.000116
-34.893150	60.982680	450.427800	.000117
-33.091610	60.496560	450.427800	.000121
-31.309040	59.987160	450.427800	.000122

-29.533510 59.454220 450.427800 .000122
-27.750760 58.896990 450.427800 .000122
-107.339200 60.027230 450.428000 .000486
-105.487000 60.591610 450.427900 .000348
-103.643800 61.130270 450.427900 .000644
-101.793500 61.644070 450.427300 .003018
-99.922550 62.133320 450.424000 .012911
-98.020040 62.597820 450.408400 .047687
-96.077510 63.037030 450.344600 .159459
-94.088710 63.450070 450.129200 .466029
-92.049460 63.835860 449.502600 1.218266
-89.957580 64.193130 447.936600 2.541138
-87.812570 64.520550 445.524400 2.988142
-85.615560 64.816710 452.838100 5.899397
-83.369120 65.080260 442.883700 13.345100
-81.077050 65.309890 424.446200 20.741670
-78.744270 65.504390 410.931700 27.566130
-76.376630 65.662700 401.885500 33.336910
-73.980770 65.783920 400.628900 33.406960
-71.563890 65.867360 414.219300 24.962500
-69.133670 65.912500 437.184400 11.902860
-66.698060 65.919100 449.311600 5.823045
-64.265090 65.887100 451.821100 3.252739
-61.842790 65.816700 451.371400 1.771551
-59.438900 65.708320 450.728700 .849243
-57.060850 65.562580 450.436500 .368757
-54.715470 65.380330 450.376000 .152896
-52.408880 65.162570 450.388900 .067614
-50.146370 64.910470 450.408500 .030653
-47.932120 64.625290 450.420100 .013000
-45.769150 64.308400 450.425200 .004913
-43.659080 63.961160 450.427100 .001641
-41.602020 63.584970 450.427600 .000506
-39.596350 63.181100 450.427700 .000176
-37.638600 62.750720 450.427800 .000113
-35.723270 62.294770 450.427800 .000121
-33.842640 61.813940 450.427800 .000122
-31.986660 61.308540 450.427800 .000122
-30.142740 60.778430 450.427800 .000122
-28.295590 60.222970 450.427800 .000122
-26.427080 59.640870 450.427800 .000122
-108.736700 60.768890 450.427800 .000157
-106.789100 61.359350 450.427800 .000188
-104.866900 61.921510 450.427700 .000783
-102.949500 62.456830 450.426900 .003369
-101.019200 62.966030 450.423300 .012829
-99.061880 63.449270 450.408200 .043194
-97.066180 63.906190 450.353500 .128498
-95.023590 64.336020 450.184600 .343009
-92.928180 64.737730 449.769400 .732487
-90.776390 65.110020 448.914500 1.259046
-88.566850 65.451470 447.615500 2.134961
-86.300130 65.760610 447.788900 5.368054
-83.978550 66.035940 440.392900 10.221220
-81.606060 66.276030 433.589900 13.976510
-79.187900 66.479550 429.256700 17.094500
-76.730480 66.645290 426.596000 19.338940
-74.241170 66.772270 427.342900 19.056140
-71.728080 66.859700 433.364500 14.392100
-69.199870 66.907030 442.240500 8.228446
-66.665550 66.913940 448.167100 4.086092
-64.134220 66.880400 450.370400 2.071795
-61.614990 66.806620 450.705900 1.096153
-59.116610 66.693070 450.571200 .550435
-56.647430 66.540450 450.453600 .236864
-54.215080 66.349720 450.412500 .093865
-51.826330 66.121990 450.410800 .039679

-49.486860 65.858530 450.417900 .018844
-47.201050 65.560780 450.423400 .008531
-44.971820 65.230190 450.426300 .003420
-42.800360 64.868270 450.427300 .001203
-40.685980 64.476460 450.427600 .000399
-38.625890 64.056100 450.427800 .000147
-36.614990 63.608330 450.427800 .000116
-34.645670 63.134020 450.427800 .000122
-32.707610 62.633670 450.427800 .000122
-30.787590 62.107330 450.427800 .000122
-28.869240 61.554470 450.427800 .000122
-26.932890 60.973880 450.427800 .000122
-24.955330 60.363580 450.427800 .000122

Q.22a
-89.453400 32.991660 475.004900 1.032534
-88.345930 33.191160 475.099500 1.165563
-87.228100 33.381660 475.467900 1.667291
-86.100230 33.562890 476.091400 2.483856
-84.962690 33.734530 476.947500 3.538433
-83.815900 33.896310 477.961600 4.706820
-82.660300 34.047950 478.982900 5.804843
-81.496410 34.189190 479.774200 6.584846
-80.324730 34.319790 480.025200 6.745243
-79.145840 34.439500 479.437900 6.006557
-77.960310 34.548100 477.833300 4.331603
-76.768760 34.645400 475.409300 2.233799
-75.571840 34.731200 473.220100 1.107005
-74.370180 34.805370 472.670900 1.162629
-73.164480 34.867740 473.567900 .718699
-71.955430 34.918200 474.633500 .601618
-70.743730 34.956650 475.239700 1.018133
-69.530080 34.983020 475.366500 1.197066
-68.315220 34.997260 475.232800 1.096384
-67.099880 34.999340 475.014200 .839668
-65.884770 34.989250 474.805200 .550213
-64.670640 34.967020 474.653800 .310863
-63.458190 34.932690 474.566700 .151846
-62.248150 34.886320 474.525100 .063217
-61.041230 34.828000 474.507900 .021938
-59.838120 34.757850 474.501700 .010762
-58.639500 34.676010 474.500700 .010299
-57.446040 34.582610 474.502100 .008587
-56.258390 34.477850 474.504100 .007247
-55.077160 34.361910 474.504500 .006500

-53.902950	34.235010	474.503600	.005044
-52.736340	34.097380	474.502300	.003207
-51.577860	33.949260	474.501400	.001707
-50.428010	33.790910	474.500900	.000761
-49.287250	33.622600	474.500600	.000351
-48.156040	33.444620	474.500500	.000202
-47.034740	33.257250	474.500500	.000157
-45.923690	33.060790	474.500500	.000153
-44.823220	32.855560	474.500500	.000153
-89.701210	33.918400	475.606500	1.785223
-88.583520	34.125020	475.891300	2.089870
-87.455040	34.322360	476.806800	3.032705
-86.316120	34.510110	478.315200	4.525043
-85.167100	34.687960	480.373700	6.438004
-84.008420	34.855620	482.809100	8.554157
-82.840550	35.012790	485.287900	10.562280
-81.663990	35.159200	487.280700	12.055640
-80.479300	35.294580	488.047500	12.491270
-79.287040	35.418700	486.848100	11.299770
-78.087840	35.531310	483.119100	8.279045
-76.882320	35.632210	477.094800	4.318992
-75.671150	35.721200	471.288500	2.194300
-74.455030	35.798110	470.239100	1.976602
-73.234660	35.862810	472.877400	1.331394
-72.010770	35.915150	475.539300	1.703566
-70.784100	35.955040	476.767100	2.439849
-69.555380	35.982390	476.873800	2.616592
-68.325390	35.997170	476.429500	2.290030
-67.094890	35.999320	475.826600	1.707547
-65.864640	35.988860	475.262000	1.092235
-64.635410	35.965800	474.867500	.601615
-63.407950	35.930180	474.650500	.285142
-62.183030	35.882080	474.552500	.113794
-60.961370	35.821590	474.513300	.039706
-59.743720	35.748840	474.499200	.029769
-58.530780	35.663950	474.497400	.029864
-57.323260	35.567100	474.502600	.023520
-56.121820	35.458470	474.509300	.017802
-54.927120	35.338260	474.510900	.014448
-53.739770	35.206700	474.508100	.010479
-52.560380	35.064020	474.504600	.006222
-51.389480	34.910500	474.502400	.003117
-50.227610	34.746390	474.501200	.001323
-49.075240	34.571990	474.500700	.000500
-47.932830	34.387580	474.500600	.000210
-46.800740	34.193490	474.500500	.000161
-45.679330	33.990000	474.500500	.000094
-44.568900	33.777440	474.500500	.000153
-89.960660	34.843320	477.677600	3.921549
-88.832330	35.057210	478.682500	4.726218
-87.692770	35.261540	481.522400	6.885247
-86.542320	35.455950	486.084000	10.201890
-85.381340	35.640140	492.248900	14.411410
-84.210270	35.813810	499.501400	19.034900
-83.029580	35.976620	506.910300	23.444560
-81.839790	36.128310	513.073400	26.861320
-80.641480	36.268600	515.800700	28.141380
-79.435230	36.397220	512.856100	25.871320
-78.221690	36.513940	502.238500	19.318410
-77.001530	36.618520	483.818100	10.316130
-75.775430	36.710780	464.808600	5.243126
-74.544130	36.790520	462.339400	4.922578
-73.308360	36.857590	472.043100	4.049322
-72.068890	36.911860	479.865300	5.864032
-70.826490	36.953220	482.860700	6.996335
-69.581950	36.981590	482.522200	6.938649
-68.336070	36.996900	480.871600	5.903020

-67.089650	36.999130	478.834800	4.332374
-65.843500	36.988290	476.935300	2.723193
-64.598400	36.964370	475.626200	1.470581
-63.355190	36.927440	474.942400	.682364
-62.114630	36.877570	474.644300	.265575
-60.877510	36.814860	474.526200	.109722
-59.644590	36.739430	474.482400	.111466
-58.416640	36.651430	474.478400	.108874
-57.194370	36.551040	474.501100	.082303
-55.978490	36.438440	474.528300	.057081
-54.769680	36.313860	474.535200	.042243
-53.568590	36.177530	474.524000	.027588
-52.375820	36.029710	474.512300	.015225
-51.191970	35.870660	474.505300	.007069
-50.017550	35.700660	474.502300	.002871
-48.853080	35.520030	474.501000	.001029
-47.698980	35.329070	474.500600	.000350
-46.555660	35.128100	474.500500	.000190
-45.423470	34.917440	474.500500	.000092
-44.302690	34.697410	474.500500	.000153
-90.232250	35.766320	482.972800	8.081226
-89.092860	35.987640	486.133000	9.973702
-87.941790	36.199090	494.290500	14.532090
-86.779330	36.400320	506.997700	21.316910
-85.605870	36.591000	524.086100	29.863510
-84.421870	36.770790	544.044400	39.200250
-83.227800	36.939400	564.477400	48.139290
-82.024180	37.096500	582.213700	55.384560
-80.811620	37.241800	591.171900	58.781480
-79.590740	37.375040	584.981400	55.083150
-78.362190	37.495960	557.641600	41.931090
-77.126670	37.604320	504.957300	22.346570
-75.884930	37.699920	442.654200	12.546220
-74.637700	37.782550	447.867700	12.686850
-73.385770	37.852060	476.020200	13.257860
-72.129940	37.908310	496.275800	16.489600
-70.871030	37.951180	502.158900	18.106370
-69.609860	37.980580	499.588600	16.992630
-68.347290	37.996460	494.029900	14.154210
-67.084140	37.998780	487.727200	10.296710
-65.821270	37.987530	481.687500	6.354431
-64.559530	37.962740	477.686400	3.351121
-63.299760	37.924460	475.684800	1.521799
-62.042780	37.872770	474.861700	.595494
-60.789440	37.807780	474.526600	.342530
-59.540510	37.729610	474.395100	.392419
-58.296810	37.638420	474.388900	.367910
-57.059080	37.534390	474.478100	.265806
-55.828080	37.417740	474.588300	.174605
-54.604500	37.288680	474.610200	.117880
-53.389020	37.147460	474.570100	.069572
-52.182290	36.994370	474.532000	.034406
-50.984880	36.829660	474.512300	.014886
-49.797380	36.653650	474.504200	.005586
-48.620270	36.466650	474.501600	.001884
-47.454020	36.268990	474.500700	.000561
-46.299010	36.060990	474.500500	.000220
-45.155610	35.843010	474.500500	.000097
-44.024070	35.615370	474.500500	.000153
-90.516530	36.687310	494.430500	14.663440
-89.365660	36.916220	503.173500	18.497010
-88.202590	37.134950	524.162400	26.939450
-87.027650	37.343150	555.628200	38.852520
-85.841200	37.540460	598.373800	53.955520
-84.643710	37.726540	647.634900	70.479000
-83.435650	37.901050	697.780100	86.268200
-82.217600	38.063690	745.159400	99.856460

-80.990140	38.214140	771.970900	108.091900
-79.753940	38.352110	762.212000	104.114600
-78.509670	38.477340	702.592500	80.728440
-77.258070	38.589580	563.995400	45.406440
-75.999910	38.688600	363.298900	20.610260
-74.735980	38.774210	391.592000	22.558690
-73.467090	38.846230	531.804900	50.681400
-72.194090	38.904510	551.309200	39.465610
-70.917830	38.948930	555.637800	39.646880
-69.639210	38.979390	544.454300	36.559010
-68.359090	38.995840	528.813800	30.230950
-67.078360	38.998240	511.272700	21.833470
-65.797930	38.986590	493.455000	13.191520
-64.518690	38.960900	482.319600	6.761250
-63.241520	38.921240	477.269600	3.023328
-61.967320	38.867690	475.257600	1.291109
-60.696920	38.800350	474.394700	1.094393
-59.431210	38.719360	474.009000	1.252759
-58.170970	38.624900	474.012500	1.127092
-56.917050	38.517150	474.345800	.773831
-55.670200	38.396340	474.793200	.479997
-54.431160	38.262690	474.849000	.310293
-53.200640	38.116470	474.676500	.153510
-51.979310	37.957960	474.572100	.067150
-50.767770	37.787460	474.524100	.025975
-49.566610	37.605300	474.507400	.009183
-48.376340	37.411790	474.502200	.002840
-47.197410	37.207270	474.500900	.000792
-46.030250	36.992090	474.500500	.000211
-44.875180	36.766620	474.500500	.000097
-43.732470	36.531200	474.500500	.000151
-90.814130	37.606180	514.704000	22.701590
-89.651300	37.842850	535.849700	29.403560
-88.475770	38.069030	586.017900	43.029130
-87.287830	38.284350	653.897900	59.434240
-86.087840	38.488450	751.704500	81.255670
-84.876290	38.680970	862.243600	106.263900
-83.653650	38.861550	971.142100	129.863900
-82.420500	39.029860	1090.290000	148.567700
-81.177470	39.185590	1165.204000	165.983700
-79.925230	39.328410	1159.210000	169.785400
-78.664510	39.458070	1067.526000	140.802800
-77.396060	39.574290	749.302700	75.481830
-76.120680	39.676830	64.703160	115.418900
-74.839220	39.765490	283.932600	156.543800
-73.552520	39.840080	660.631100	75.273140
-72.261480	39.900440	728.695300	89.311260
-70.967010	39.946460	687.414600	69.845230
-69.670030	39.978020	649.309400	65.964410
-68.371470	39.995060	611.838600	55.255900
-67.072270	39.997550	569.877700	39.917960
-65.773400	39.985470	517.901900	23.231490
-64.475770	39.958870	490.759100	11.876510
-63.180340	39.917780	479.869700	5.382499
-61.888020	39.862300	475.805800	2.959162
-60.599740	39.792560	473.665100	3.301422
-59.316390	39.708680	472.550700	3.754907
-58.038840	39.610860	472.573100	3.238930
-56.767930	39.499290	473.773300	2.078095
-55.504470	39.374200	475.609100	1.261626
-54.249260	39.235840	475.616500	.756240
-53.003000	39.084480	474.906600	.295671
-51.766410	38.920440	474.619600	.101945
-50.540120	38.744010	474.535600	.035880
-49.324710	38.555530	474.509200	.011909
-48.120720	38.355340	474.502500	.003597
-46.928620	38.143810	474.500900	.000903

-45.748810 37.921300 474.500500 .000198
-44.581620 37.688170 474.500500 .000086
-43.427300 37.444810 474.500500 .000174
-91.125630 38.522790 541.127000 28.616750
-89.950410 38.767390 588.184300 38.760400
-88.761920 39.001200 702.813000 54.321830
-87.560450 39.223820 824.866500 69.020760
-86.346370 39.434880 1023.049000 91.963880
-85.120160 39.633990 1249.010000 123.526300
-83.882300 39.820790 1457.277000 153.352900
-82.633390 39.994930 1703.224000 183.042100
-81.374080 40.156070 1885.009000 216.070000
-80.105060 40.303890 1924.894000 232.880800
-78.827100 40.438090 1876.495000 212.028900
-77.540980 40.558400 1485.794000 174.209500
-76.247540 40.664560 324.736800 222.757200
-74.947690 40.756360 72.611100 161.025200
-73.642300 40.833600 963.108600 493.774900
-72.332310 40.896110 945.494500 122.175300
-71.018690 40.943760 944.782600 102.204600
-69.702430 40.976440 853.168800 94.374040
-68.384490 40.994090 785.703900 82.699810
-67.065880 40.996670 695.986000 59.162880
-65.747600 40.984170 551.756800 35.565160
-64.430650 40.956610 499.993900 17.815290
-63.116020 40.914060 483.088900 9.361102
-61.804690 40.856610 476.341800 7.417118
-60.497620 40.784390 470.973900 9.635973
-59.195770 40.697540 467.383900 10.715520
-57.900040 40.596260 467.446200 8.839151
-56.611330 40.480750 471.694100 5.000045
-55.330490 40.351270 477.019600 3.215393
-54.058330 40.208070 476.649400 1.769735
-52.795620 40.051450 475.128300 .360517
-51.543080 39.881710 474.628400 .117060
-50.301390 39.699200 474.525500 .047405
-49.071130 39.504250 474.506000 .017557
-47.852850 39.297230 474.501400 .005692
-46.647030 39.078520 474.500500 .001517
-45.454070 38.848490 474.500400 .000358
-44.274280 38.607530 474.500400 .000144
-43.107920 38.356030 474.500500 .000159
-91.451740 39.437030 558.696800 26.118340
-90.263630 39.689750 642.359200 34.955060
-89.061680 39.931370 852.102900 90.543900
-87.846150 40.161480 997.184700 76.429230
-86.617400 40.379670 1271.331000 99.960190
-85.375900 40.585540 1658.024000 162.832800
-84.122160 40.778730 2009.400000 176.677600
-82.856800 40.958850 2329.743000 223.361500
-81.580460 41.125550 2638.249000 281.060400
-80.293880 41.278500 2849.363000 310.651700
-78.997860 41.417370 2937.273000 323.336000
-77.693230 41.541890 2694.733000 297.263200
-76.380870 41.651780 1800.178000 217.371700
-75.061690 41.746810 849.917600 126.738200
-73.736660 41.826770 542.528000 166.765800
-72.406780 41.891490 705.961800 87.426190
-71.073040 41.940830 1102.763000 124.054300
-69.736500 41.974670 1069.318000 123.380800
-68.398190 41.992950 989.291800 115.143900
-67.059160 41.995620 804.220500 108.478700
-65.720480 41.982670 525.624900 49.262780
-64.383220 41.954140 496.325800 29.101080
-63.048400 41.910080 487.232500 18.944080
-61.717090 41.850600 476.284800 18.742530
-60.390290 41.775820 457.950900 24.266410

-59.069010	41.685920	449.082100	24.469710
-57.754210	41.581080	452.251200	18.078500
-56.446830	41.461530	467.320100	8.709467
-55.147770	41.327530	473.941600	1.889715
-53.857880	41.179360	473.019000	2.254054
-52.577970	41.017320	473.914200	.760726
-51.308780	40.841740	474.421200	.273696
-50.051000	40.652980	474.502300	.108567
-48.805250	40.451390	474.502900	.041713
-47.572090	40.237360	474.501400	.013667
-46.352010	40.011290	474.500600	.003885
-45.145370	39.773560	474.500500	.000914
-43.952510	39.524580	474.500400	.000239
-42.773630	39.264750	474.500500	.000143
-91.793200	40.348750	529.704300	13.814790
-90.591710	40.609810	571.964400	22.408220
-89.375760	40.859430	664.028700	66.880540
-88.145600	41.097210	693.682400	98.456530
-86.901570	41.322720	912.480500	91.232670
-85.644150	41.535550	1418.289000	96.586490
-84.373860	41.735290	2056.440000	182.025700
-83.091290	41.921560	2424.689000	229.580000
-81.797160	42.093980	2811.403000	289.108900
-80.492230	42.252200	3280.225000	350.402100
-79.177290	42.395880	3542.254000	383.979900
-77.853240	42.524730	3372.475000	361.605000
-76.521000	42.638450	2665.674000	246.725700
-75.181550	42.736820	1679.735000	126.717100
-73.835900	42.819590	818.235100	142.039500
-72.485100	42.886590	543.710100	161.213500
-71.130210	42.937670	1139.284000	252.079500
-69.772340	42.972710	1107.398000	131.576400
-68.412590	42.991630	972.442100	130.609200
-67.052090	42.994400	631.664700	125.411000
-65.691960	42.980990	406.742800	127.354200
-64.333320	42.951450	509.420300	52.196970
-62.977290	42.905830	509.692600	35.001830
-61.624970	42.844260	468.943400	37.373490
-60.277440	42.766850	409.454500	38.080960
-58.935750	42.673790	405.814000	33.949480
-57.600940	42.565280	434.898200	19.121290
-56.273990	42.441570	476.046900	9.415533
-54.955840	42.302930	480.281100	5.232577
-53.647390	42.149640	476.515800	2.745700
-52.349470	41.982030	475.285100	1.305783
-51.062890	41.800450	474.822000	.603665
-49.788320	41.605260	474.611900	.260393
-48.526440	41.396860	474.535400	.099855
-47.277780	41.175640	474.510300	.033958
-46.042850	40.942000	474.503100	.010159
-44.822010	40.696380	474.501000	.002630
-43.615560	40.439180	474.500500	.000583
-42.423690	40.170830	474.500500	.000194
-92.150770	41.257820	467.823800	19.174960
-90.935390	41.527410	394.416000	39.549770
-89.704910	41.785250	154.505500	119.749600
-88.459540	42.030900	134.162300	150.617500
-87.199620	42.263930	484.316800	152.296500
-85.925610	42.483890	1153.150000	117.463600
-84.638040	42.690380	1988.883000	197.357300
-83.337520	42.882970	2538.344000	268.994100
-82.024790	43.061280	3050.489000	346.239600
-80.700610	43.224930	3618.796000	395.228600
-79.365870	43.373580	3831.185000	409.726400
-78.021450	43.506890	3608.974000	374.318600
-76.668370	43.624570	2928.531000	299.573300
-75.307630	43.726360	2009.512000	207.728800

-73.940300	43.812040	1102.343000	181.849100
-72.567500	43.881390	326.094300	180.158400
-71.190370	43.934270	765.076800	104.323700
-69.810050	43.970550	1259.400000	157.630700
-68.427750	43.990140	1359.950000	143.986600
-67.044650	43.993000	950.026400	126.239200
-65.661930	43.979120	737.522700	91.557790
-64.280810	43.948540	693.250200	61.079340
-62.902460	43.901310	557.277400	45.853230
-61.528050	43.837570	450.129600	59.271010
-60.158730	43.757450	352.411000	52.833240
-58.795620	43.661140	405.245400	30.382050
-57.439790	43.548850	476.052400	20.394840
-56.092310	43.420850	504.553100	17.527230
-54.754150	43.277410	491.343600	8.633759
-53.426270	43.118850	481.401600	4.953299
-52.109530	42.945500	477.247700	2.655070
-50.804760	42.757750	475.541900	1.297145
-49.512690	42.555960	474.863200	.567184
-48.233980	42.340550	474.615600	.221547
-46.969190	42.111940	474.533800	.077509
-45.718810	41.870550	474.509200	.024172
-44.483210	41.616830	474.502500	.006642
-43.262650	41.351200	474.500900	.001605
-42.057300	41.074100	474.500600	.000492
-92.525320	42.164070	460.165700	19.372810
-91.295520	42.442420	401.874800	40.679270
-90.049940	42.708690	256.906000	87.644430
-88.788760	42.962440	342.811700	75.615650
-87.512310	43.203190	665.685900	182.855700
-86.221000	43.430500	1007.445000	106.462900
-84.915410	43.643920	1737.513000	181.183200
-83.596150	43.843030	2488.816000	256.671900
-82.263960	44.027400	3120.402000	336.226500
-80.919660	44.196660	3800.800000	448.761700
-79.564150	44.350410	3943.508000	445.334800
-78.198390	44.488330	3493.445000	369.977700
-76.823410	44.610100	2455.124000	176.836600
-75.440290	44.715440	1239.163000	151.702300
-74.050180	44.804110	513.856500	126.769000
-72.654240	44.875890	138.308800	143.208900
-71.253690	44.930630	628.295700	94.087460
-69.849760	44.968180	1574.840000	171.756000
-68.443720	44.988460	2059.245000	293.298300
-67.036810	44.991420	1525.226000	164.102800
-65.630330	44.977050	1088.339000	103.963200
-64.225530	44.945390	795.661600	69.131140
-62.823690	44.896510	417.734500	79.541840
-61.426040	44.830540	211.189000	114.009800
-60.033810	44.747610	244.608300	110.814000
-58.648170	44.647940	400.642300	60.044530
-57.270290	44.531750	520.589900	38.927330
-55.901260	44.399320	528.081400	23.705130
-54.542130	44.250930	502.021600	14.371040
-53.193900	44.086940	487.578600	8.751932
-51.857480	43.907680	480.367600	5.014375
-50.533710	43.713560	476.863400	2.515804
-49.223370	43.504980	475.353900	1.116594
-47.927120	43.282370	474.778000	.442072
-46.645540	43.046150	474.581800	.156808
-45.379100	42.796800	474.522000	.049940
-44.128160	42.534760	474.505600	.014224
-42.892960	42.260480	474.501600	.003629
-41.673610	41.974410	474.500800	.001137
-92.917770	43.067330	486.043300	11.292920
-91.673000	43.354680	493.955700	19.487450
-90.411740	43.629630	529.443000	38.824970

-89.134110	43.891690	581.285500	166.790500
-87.840450	44.140390	278.107200	149.714100
-86.531140	44.375260	82.613000	172.846000
-85.206750	44.595830	1043.382000	138.865000
-83.867900	44.801650	2255.478000	241.936600
-82.515380	44.992280	2903.427000	320.532100
-81.150000	45.167310	3246.593000	347.337100
-79.772730	45.326340	3195.909000	338.149400
-78.384560	45.469020	2772.923000	273.054100
-76.986600	45.595010	1922.041000	106.075500
-75.579970	45.704020	967.367200	168.747800
-74.165890	45.795780	421.061100	160.066300
-72.745610	45.870080	125.631700	87.543130
-71.320400	45.926740	292.148300	193.519000
-69.891600	45.965610	1659.450000	176.611900
-68.460530	45.986610	2198.028000	327.889500
-67.028560	45.989670	1562.098000	147.712700
-65.597030	45.974800	1141.660000	112.360900
-64.167300	45.942020	918.719400	87.460660
-62.740720	45.891430	589.803000	37.469050
-61.318600	45.823140	271.908800	124.352500
-59.902260	45.737310	385.741700	92.412640
-58.492950	45.634170	569.834700	62.875050
-57.091900	45.513950	596.166700	36.546020
-55.700260	45.376930	556.181500	31.700890
-54.319140	45.223450	516.934000	22.818530
-52.949590	45.053840	498.585900	15.655840
-51.592580	44.868490	486.560000	8.857786
-50.248970	44.667810	479.539200	4.476543
-48.919560	44.452230	476.322400	1.990340
-47.605040	44.222190	475.087000	.788191
-46.305980	43.978160	474.671200	.280336
-45.022840	43.720610	474.545500	.089841
-43.755960	43.450020	474.511300	.025943
-42.505550	43.166850	474.502800	.006814
-41.271660	42.871580	474.501200	.002194
-93.329110	43.967430	505.063600	20.720450
-92.068780	44.264030	531.306800	32.395880
-90.791210	44.547890	583.545200	57.980990
-89.496510	44.818510	547.358900	135.350300
-88.184940	45.075390	489.705900	295.472400
-86.856880	45.318050	269.681600	157.973500
-85.512890	45.545990	999.628600	66.440310
-84.153590	45.758730	2025.871000	232.441000
-82.779790	45.955820	2348.756000	235.365600
-81.392350	46.136820	2267.789000	235.797400
-79.992260	46.301310	1976.485000	178.400300
-78.580580	46.448910	1650.925000	104.946600
-77.158460	46.579270	1392.924000	143.005200
-75.727110	46.692070	1367.645000	184.607700
-74.287820	46.787040	1309.917000	312.833700
-72.841890	46.863950	572.917400	183.562800
-71.390720	46.922590	668.890300	166.502000
-69.935700	46.962830	1848.815000	376.459500
-68.478260	46.984570	1867.147000	186.275300
-67.019850	46.987740	1312.024000	112.541000
-65.561920	46.972340	936.758900	72.423450
-64.105920	46.938410	858.428900	72.792470
-62.653260	46.886040	806.058800	96.772200
-61.205390	46.815350	620.799000	62.027670
-59.763680	46.726520	562.374900	44.239200
-58.329470	46.619790	655.000100	51.256810
-56.904060	46.495390	646.627900	47.633570
-55.488670	46.353640	600.480700	47.545070
-54.084500	46.194880	570.448700	37.508950
-52.692630	46.019470	530.097700	25.670780
-51.314070	45.827840	499.626500	14.608860

-49.949730	45.620390	484.400800	7.223442
-48.600430	45.397590	477.952600	3.146266
-47.266860	45.159900	475.577300	1.229675
-45.949600	44.907820	474.805100	.434221
-44.649100	44.641840	474.579600	.138901
-43.365670	44.362450	474.519300	.040251
-42.099450	44.070140	474.504700	.010686
-40.850440	43.765410	474.501600	.003490
-93.760410	44.864160	519.801800	31.536150
-92.483920	45.170280	546.037600	41.767780
-91.189420	45.463320	551.807800	66.597110
-89.876960	45.742750	339.571800	64.418840
-88.546760	46.008070	53.470010	110.496900
-87.199170	46.258760	124.157700	164.043300
-85.834720	46.494300	924.314800	153.391500
-84.454070	46.714200	1406.575000	175.531700
-83.058010	46.917960	1531.632000	136.744500
-81.647480	47.105130	1432.271000	147.782500
-80.223460	47.275260	1204.653000	88.119890
-78.787090	47.427960	655.590200	77.769940
-77.339580	47.562840	221.015300	195.079000
-75.882230	47.679570	755.479400	86.348150
-74.416370	47.777870	1324.106000	169.653900
-72.943440	47.857470	1290.921000	215.799800
-71.464880	47.918190	1212.241000	106.741200
-69.982220	47.959850	1473.249000	127.307400
-68.496960	47.982350	1198.063000	103.075300
-67.010670	47.985630	800.364400	83.616120
-65.524890	47.969690	382.366600	177.130200
-64.041170	47.934570	462.521500	64.819180
-62.561040	47.880340	671.116100	64.548690
-61.086020	47.807170	677.503700	93.576290
-59.617590	47.715230	516.002000	67.955380
-58.157170	47.604770	603.493600	45.119360
-56.706150	47.476050	699.190400	60.541660
-55.265830	47.329400	724.027200	67.019200
-53.837470	47.165180	695.222200	55.420570
-52.422200	46.983780	594.477600	36.638800
-51.021090	46.785640	520.197500	20.890300
-49.635090	46.571200	490.382000	9.884833
-48.265020	46.340950	479.761200	4.226705
-46.911610	46.095370	476.092900	1.620796
-45.575410	45.834990	474.941300	.566536
-44.256860	45.560320	474.612500	.180394
-42.956210	45.271870	474.526900	.052275
-41.673580	44.970160	474.506300	.013950
-40.408870	44.655690	474.502100	.004575
-94.212860	45.757310	542.104700	42.533270
-92.919560	46.073230	579.736000	52.427690
-91.607470	46.375730	612.324000	67.858800
-90.276550	46.664250	525.855000	49.359460
-88.926960	46.938270	563.034200	99.123250
-87.559010	47.197250	716.630600	251.888100
-86.173240	47.440650	595.221300	194.519900
-84.770290	47.667940	299.930300	182.940500
-83.350960	47.878600	832.136700	175.872900
-81.916200	48.072160	1367.786000	165.634000
-80.467090	48.248140	1183.453000	155.558000
-79.004780	48.406110	299.253900	160.982100
-77.530590	48.545680	83.993030	138.106900
-76.045850	48.666500	529.588800	88.264470
-74.552020	48.768250	1159.137000	116.124600
-73.050600	48.850660	1297.459000	120.281200
-71.543170	48.913520	1264.690000	118.530300
-70.031330	48.956650	1207.642000	104.536500
-68.516710	48.979950	923.177600	91.794150
-67.000980	48.983350	763.366900	60.185920

-65.485790	48.966840	442.888500	72.499920
-63.972820	48.930470	362.608000	61.858430
-62.463700	48.874330	491.553000	59.317750
-60.960050	48.798580	543.187500	58.300400
-59.463460	48.703410	507.263900	62.380330
-57.975440	48.589080	481.393600	63.887940
-56.497490	48.455880	627.792400	48.574460
-55.030970	48.304140	727.959800	62.322510
-53.577210	48.134270	744.779700	77.203830
-52.137430	47.946660	632.700300	43.958230
-50.712720	47.741790	532.689600	22.820810
-49.304080	47.520120	492.863600	11.070060
-47.912350	47.282170	480.344500	4.555573
-46.538250	47.028450	476.271700	1.747633
-45.182340	46.759510	474.989300	.608060
-43.845020	46.475880	474.624700	.193513
-42.526500	46.178100	474.529600	.056156
-41.226800	45.866700	474.506900	.015036
-39.945740	45.542200	474.502200	.004949
-94.687740	46.646660	575.470200	54.383080
-93.376940	46.972690	624.341700	64.383900
-92.046550	47.284930	655.937900	69.342900
-90.696440	47.582840	645.635100	65.058900
-89.326680	47.865840	705.747100	62.906900
-87.937550	48.133390	702.003000	124.646800
-86.529520	48.384910	120.439500	143.963000
-85.103260	48.619850	115.768500	181.821800
-83.659580	48.837660	1207.130000	284.301600
-82.199460	49.037830	1587.507000	197.977700
-80.724000	49.219880	1412.713000	177.310800
-79.234450	49.383340	893.992400	94.390650
-77.732160	49.527770	824.341900	107.824800
-76.218590	49.652830	893.230300	58.830960
-74.695260	49.758160	1181.731000	95.936590
-73.163800	49.843490	1161.876000	101.557600
-71.625870	49.908580	1103.946000	97.720280
-70.083210	49.953250	1077.324000	85.928030
-68.537570	49.977380	913.642000	72.629440
-66.990740	49.980900	762.290700	48.633430
-65.444490	49.963800	417.266100	118.736300
-63.900620	49.926140	396.758400	78.605730
-62.360880	49.868010	589.689200	160.295800
-60.827010	49.789570	682.660500	100.244000
-59.300730	49.691040	641.296100	48.821090
-57.783640	49.572690	592.672700	52.140910
-56.277320	49.434830	641.028000	51.560230
-54.783270	49.277820	672.883200	70.245000
-53.302860	49.102080	636.691200	52.346120
-51.837360	48.908040	565.710200	35.395710
-50.387950	48.696200	515.040600	19.873530
-48.955640	48.467040	489.459100	9.285957
-47.541310	48.221130	479.418100	3.998178
-46.145660	47.959000	475.991500	1.526003
-44.769230	47.681210	474.917500	.536296
-43.412380	47.388330	474.607300	.171479
-42.075270	47.080920	474.525900	.049960
-40.757810	46.759540	474.506100	.013415
-39.459720	46.424710	474.502000	.004439
-95.186440	47.531940	618.725600	66.161710
-93.857410	47.868410	677.607200	74.338480
-92.508000	48.190720	698.841500	74.488000
-91.137910	48.498310	701.419200	67.561410
-89.747150	48.790600	747.523600	66.538890
-88.335950	49.067010	743.577000	55.365070
-86.904710	49.326930	619.859900	48.768650
-85.454100	49.569790	915.500100	199.747200
-83.984930	49.795020	1248.041000	128.352200

-82.498220 50.002060 1361.790000 146.780600
-80.995120 50.190400 1231.164000 125.037000
-79.476920 50.359550 1034.044000 102.572200
-77.945060 50.509060 1046.847000 93.041240
-76.401090 50.638520 1085.854000 78.452160
-74.846650 50.747580 1222.054000 109.581800
-73.283450 50.835950 1121.260000 101.890300
-71.713320 50.903350 1077.286000 97.719990
-70.138080 50.949630 1077.014000 85.252180
-68.559640 50.974620 938.008900 55.475670
-66.979900 50.978270 894.271000 63.252050
-65.400800 50.960560 662.902800 92.842290
-63.824260 50.921550 252.236200 131.492500
-62.252170 50.861340 136.027300 127.162300
-60.686400 50.780110 488.163700 45.616610
-59.128770 50.678080 683.418000 75.181650
-57.581030 50.555550 684.440600 59.344750
-56.044860 50.412850 663.699500 73.318440
-54.521820 50.250360 605.763300 52.456080
-53.013420 50.068520 552.520300 38.350640
-51.520960 49.867810 515.123800 24.294690
-50.045680 49.648740 495.269500 13.764820
-48.588630 49.411830 483.118900 6.674177
-47.150700 49.157670 477.566400 2.859823
-45.732590 48.886830 475.474800 1.121092
-44.334810 48.599900 474.780900 .397244
-42.957660 48.297460 474.574300 .128020
-41.601190 47.980120 474.518300 .037466
-40.265250 47.648430 474.504600 .010084
-38.949390 47.302930 474.501600 .003368
-95.710510 48.412890 672.729900 73.603800
-94.362470 48.760130 748.726000 78.467380
-92.993230 49.092850 774.048300 70.847780
-91.602360 49.410460 793.840400 65.280000
-90.189750 49.712350 826.776700 66.305280
-88.755540 49.997940 818.564100 57.161280
-87.300090 50.266570 795.472200 54.761740
-85.824040 50.517650 892.406300 76.265760
-84.328210 50.750570 982.423900 106.582700
-82.813610 50.964740 1041.276000 120.165000
-81.281460 51.159630 1032.723000 107.230800
-79.733130 51.334700 988.889200 99.705970
-78.170130 51.489480 981.079500 87.341670
-76.594090 51.623530 1024.241000 87.654460
-75.006800 51.736490 1156.562000 115.890800
-73.410070 51.828010 1036.377000 98.983600
-71.805870 51.897850 1010.266000 92.096750
-70.196170 51.945790 1044.885000 84.114770
-68.582990 51.971680 919.195100 50.552520
-66.968440 51.975460 892.266200 44.216030
-65.354560 51.957120 855.145900 53.185180
-63.743440 51.916690 719.020800 111.420700
-62.137130 51.854320 484.456600 44.011890
-60.537630 51.770170 551.969800 51.693810
-58.946900 51.664510 680.755900 64.302990
-57.366820 51.537620 683.425200 69.984380
-55.799180 51.389870 625.833000 55.173150
-54.245650 51.221680 565.646900 39.776750
-52.707820 51.033510 528.554400 26.868370
-51.187080 50.825860 504.086100 16.802540
-49.684720 50.599280 487.817700 8.764273
-48.201800 50.354330 479.590300 4.270582
-46.739240 50.091630 476.271400 1.856321
-45.297720 49.811770 475.068800 .726575
-43.877700 49.515370 474.666600 .258446
-42.479410 49.203060 474.544700 .083440
-41.102810 48.875440 474.511300 .024440

-39.747590	48.533100	474.503000	.006575
-38.413150	48.176570	474.501200	.002181
-96.261670	49.289190	728.045700	69.509360
-94.893750	49.647600	834.454700	72.830590
-93.503820	49.991080	865.048500	70.558080
-92.091320	50.319060	847.848600	65.293750
-90.655950	50.630910	801.281200	56.069540
-89.197760	50.925990	744.185500	53.592980
-87.717060	51.203660	711.293000	57.361770
-86.214420	51.463270	730.829000	72.312500
-84.690660	51.704190	778.687500	88.839290
-83.146820	51.925780	853.264700	85.286270
-81.584160	52.127480	945.495800	101.137300
-80.004110	52.308710	904.754600	78.416500
-78.408280	52.468990	839.406400	84.988690
-76.798410	52.607840	919.327500	93.858760
-75.176390	52.724850	1085.033000	114.360600
-73.544200	52.819690	953.692100	90.799900
-71.903920	52.892050	930.248700	77.901530
-70.257710	52.941730	982.424700	79.941800
-68.607750	52.968570	876.538500	42.072310
-66.956280	52.972490	816.453700	32.971870
-65.305560	52.953480	834.707200	48.367390
-63.657820	52.911580	813.749500	60.753900
-62.015270	52.846950	722.947400	59.702640
-60.380080	52.759750	685.833700	63.163130
-58.754360	52.650280	672.211200	62.679010
-57.140120	52.518850	639.402400	53.002060
-55.539290	52.365840	590.443500	44.025620
-53.953660	52.191710	537.839600	30.407410
-52.384890	51.996940	507.241200	18.904960
-50.834480	51.782070	490.838500	10.799150
-49.303730	51.547700	481.489400	5.628326
-47.793770	51.294400	477.174100	2.629557
-46.305490	51.022820	475.447100	1.117274
-44.839570	50.733620	474.805300	.433215
-43.396390	50.427420	474.590000	.152860
-41.976090	50.104880	474.524500	.049098
-40.578500	49.766630	474.506500	.014284
-39.203150	49.413260	474.501800	.003810
-37.849210	49.045330	474.500900	.001265
-96.841810	50.160530	717.023400	56.577900
-95.453030	50.530490	801.596900	67.434350
-94.041510	50.885130	785.215000	54.647710
-92.606450	51.223850	734.417200	53.434370
-91.147380	51.546020	682.051300	53.262830
-89.664200	51.850960	635.983800	52.104460
-88.157130	52.138020	610.596000	51.992450
-86.626690	52.406500	613.571500	55.531150
-85.073680	52.655720	648.148200	57.188450
-83.499180	52.885050	711.770100	63.287930
-81.904440	53.093850	773.096900	66.455060
-80.290990	53.281520	800.917600	74.595650
-78.660530	53.447530	760.522700	77.032400
-77.014920	53.591380	862.583300	88.751200
-75.356160	53.712650	1013.703000	109.455800
-73.686420	53.810940	836.938000	86.649080
-72.007930	53.885960	803.426500	65.087330
-70.322990	53.937460	832.497400	52.962960
-68.634010	53.965280	741.034400	46.990570
-66.943400	53.969350	678.255400	52.744040
-65.253590	53.949630	743.235600	37.200900
-63.567000	53.906200	756.985200	58.949720
-61.886040	53.839190	737.193400	68.427040
-60.213040	53.748820	657.310400	56.811780
-58.550290	53.635360	592.489700	40.893570
-56.899960	53.499180	571.471300	33.924450

-55.264110	53.340690	561.111100	33.218430
-53.644650	53.160350	523.000000	23.652650
-52.043340	52.958710	495.484800	13.587060
-50.461760	52.736320	483.468900	7.079607
-48.901280	52.493820	478.148200	3.450966
-47.363030	52.231850	475.882200	1.549758
-45.847900	51.951060	474.980200	.640942
-44.356530	51.652150	474.653300	.243080
-42.889220	51.335790	474.545100	.084464
-41.445980	51.002650	474.512700	.026723
-40.026490	50.653380	474.503600	.007657
-38.630060	50.288590	474.501200	.002019
-37.255620	49.908830	474.500700	.000674
-97.453060	51.026530	613.727700	46.312770
-96.042340	51.408490	644.343800	42.883140
-94.608220	51.774700	645.996800	46.849530
-93.149600	52.124570	637.437200	50.487560
-91.665800	52.457450	618.335400	49.840800
-90.156560	52.772640	591.241800	45.778570
-88.621980	53.069450	569.554300	41.976770
-87.062480	53.347160	556.644300	39.374260
-85.478850	53.605050	545.621200	39.640510
-83.872130	53.842430	514.984900	58.469260
-82.243660	54.058630	471.875500	101.012800
-80.595020	54.253030	572.819300	69.402870
-78.928000	54.425040	672.597900	63.520080
-77.244600	54.574130	837.608200	83.291310
-75.546970	54.699840	982.192300	108.176700
-73.837420	54.801750	801.183500	79.325420
-72.118360	54.879540	751.996300	56.495270
-70.392330	54.932960	693.604700	45.606250
-68.661900	54.961820	513.714300	70.510090
-66.929700	54.966030	502.165000	91.681820
-65.198370	54.945580	651.599700	47.928200
-63.470540	54.900540	693.385100	72.240590
-61.748820	54.831050	627.843000	54.283070
-60.035740	54.737340	528.674300	37.663680
-58.333780	54.619720	480.636600	24.970150
-56.645260	54.478570	489.898300	14.139900
-54.972420	54.314330	516.750200	17.072470
-53.317280	54.127510	510.346200	15.376360
-51.681740	53.918690	489.571900	8.741313
-50.067420	53.688470	479.880900	4.321112
-48.475770	53.437500	476.480700	2.001457
-46.907930	53.166490	475.207200	.870537
-45.364770	52.876130	474.736800	.349710
-43.846840	52.567130	474.574000	.129575
-42.354370	52.240220	474.521900	.044007
-40.887210	51.896080	474.506300	.013650
-39.444820	51.535370	474.501800	.003833
-38.026250	51.158730	474.500800	.000999
-36.630120	50.766700	474.500600	.000353
-98.097820	51.886770	539.860800	30.328120
-96.663910	52.281200	545.682300	30.370810
-95.206040	52.659450	548.914000	31.016290
-93.722790	53.020900	552.546800	32.728180
-92.213200	53.364910	559.251500	36.373420
-90.676770	53.690770	569.264800	42.580280
-89.113440	53.997730	570.642900	45.192460
-87.523560	54.285050	556.217800	39.477490
-85.907830	54.551970	528.376800	32.125480
-84.267300	54.797760	468.360100	49.679100
-82.603340	55.021710	341.011900	129.811500
-80.917590	55.223140	297.395400	169.128800
-79.211960	55.401440	528.053800	90.999360
-77.488560	55.556020	816.065700	75.796770
-75.749730	55.686380	950.333800	103.192100

-73.997920	55.792100	786.186600	72.585400
-72.235800	55.872810	724.208400	54.301500
-70.466090	55.928230	570.590200	64.622290
-68.691570	55.958180	302.203500	151.946900
-66.915130	55.962550	484.079200	86.707500
-65.139640	55.941330	665.050800	76.501650
-63.367970	55.894590	652.997800	53.989890
-61.602940	55.822490	564.358000	41.496460
-59.847310	55.725280	476.436900	34.469370
-58.103760	55.603290	428.277200	36.932450
-56.374820	55.456930	415.377400	34.144600
-54.662870	55.286670	438.702600	20.591120
-52.970100	55.093080	474.613000	4.596253
-51.298500	54.876750	478.746000	2.514726
-49.649800	54.638340	476.619100	1.835587
-48.025460	54.378550	475.356800	.946079
-46.426660	54.098120	474.817300	.426971
-44.854210	53.797780	474.606600	.173129
-43.308580	53.478290	474.533500	.063718
-41.789850	53.140400	474.510100	.021344
-40.297680	52.784830	474.503100	.006499
-38.831280	52.412250	474.501100	.001794
-37.389370	52.023280	474.500700	.000449
-35.970190	51.618480	474.500500	.000224
-98.778840	52.740810	502.285800	16.866240
-97.320270	53.148240	502.415000	16.546910
-95.837370	53.539010	503.588700	16.948880
-94.328290	53.912530	505.498900	18.097870
-92.791720	54.268120	510.352800	20.458420
-91.226900	54.605080	519.437500	24.717290
-89.633560	54.922620	531.203900	30.433400
-88.011890	55.219970	546.291100	38.174120
-86.362530	55.496330	550.251200	44.067320
-84.686490	55.750910	517.199500	44.368790
-82.985150	55.982970	421.530400	81.106070
-81.260250	56.191770	259.870000	173.611200
-79.513790	56.376650	354.108300	161.795000
-77.748020	56.536990	787.262300	83.248970
-75.965460	56.672260	918.582900	96.300370
-74.168790	56.781970	760.512000	67.261450
-72.360860	56.865740	670.955900	60.948650
-70.544630	56.923280	492.575000	81.494580
-68.723180	56.954370	378.504500	124.908400
-66.899620	56.958910	619.190200	92.680560
-65.077100	56.936880	648.929700	50.972000
-63.258730	56.888360	610.386200	43.912600
-61.447630	56.813510	548.572900	30.865440
-59.646790	56.712620	493.158200	18.115490
-57.859090	56.586040	460.440300	17.551070
-56.087300	56.434210	447.751400	18.384870
-54.333970	56.257640	454.221500	12.579670
-52.601470	56.056940	471.033600	3.507307
-50.891880	55.832740	474.765100	.573194
-49.207030	55.585770	474.952300	.484209
-47.548420	55.316760	474.768400	.335998
-45.917200	55.026500	474.613400	.171851
-44.314130	54.715760	474.541400	.073728
-42.739580	54.385350	474.513700	.027785
-41.193420	54.036030	474.504400	.009353
-39.675070	53.668560	474.501500	.002837
-38.183400	53.283620	474.500700	.000769
-36.716770	52.881820	474.500600	.000205
-35.272900	52.463700	474.500500	.000164
-99.499240	53.588130	485.105700	8.227747
-98.014280	54.009130	484.640700	7.905692
-96.504860	54.412960	484.967500	8.048960
-94.968610	54.799050	485.855900	8.684000

-93.403790	55.166730	487.888300	10.057100
-91.809290	55.515250	491.618900	12.415180
-90.184560	55.843850	497.575800	15.880270
-88.529620	56.151670	507.176000	20.929910
-86.844990	56.437890	516.655100	27.412680
-85.131650	56.701690	523.394800	37.788010
-83.390960	56.942230	508.917800	57.593070
-81.624720	57.158780	423.716600	92.709210
-79.835050	57.350580	403.618600	135.450200
-78.024370	57.516980	769.178900	81.740460
-76.195360	57.657400	880.140300	94.137580
-74.350940	57.771310	735.906300	68.765430
-72.494220	57.858320	632.879500	68.872120
-70.628410	57.918090	553.179100	76.299290
-68.756890	57.950390	552.324200	75.919530
-66.883060	57.955100	664.572800	57.330370
-65.010370	57.932220	631.704200	46.009770
-63.142240	57.881810	578.000200	38.457800
-61.282040	57.804080	522.435600	24.896640
-59.433060	57.699310	494.751300	13.464890
-57.598450	57.567890	477.009900	8.087801
-55.781200	57.410300	468.809200	6.561955
-53.984060	57.227100	468.720000	4.841781
-52.209560	57.018940	472.584300	1.901707
-50.459930	56.786500	474.070400	.509806
-48.737060	56.530560	474.504000	.096299
-47.042490	56.251900	474.552300	.082052
-45.377330	55.951360	474.531600	.053874
-43.742270	55.629770	474.513200	.026024
-42.137470	55.287960	474.505000	.010400
-40.562610	54.926740	474.501800	.003600
-39.016750	54.546860	474.500800	.001099
-37.498410	54.149020	474.500600	.000299
-36.005400	53.733830	474.500500	.000120
-34.534880	53.301770	474.500500	.000153
-100.262700	54.428120	478.158900	3.608886
-98.749250	54.863350	477.849200	3.381787
-97.211560	55.280850	477.935600	3.418211
-95.646590	55.680060	478.323500	3.739376
-94.052090	56.060360	479.180500	4.442928
-92.426510	56.420970	480.699900	5.630624
-90.768940	56.761100	483.057200	7.358458
-89.079190	57.079890	486.361800	9.728992
-87.357570	57.376450	489.641300	12.946480
-85.605000	57.649890	492.430100	19.114790
-83.822840	57.899360	486.842100	32.319630
-82.012920	58.124030	458.345400	50.792480
-80.177480	58.323120	519.934300	57.497010
-78.319110	58.495910	776.158800	86.756000
-76.440710	58.641750	817.416600	82.333020
-74.545430	58.760110	714.506200	82.733380
-72.636660	58.850530	652.317700	81.414970
-70.717930	58.912650	654.561100	65.565780
-68.792920	58.946230	647.611200	47.897830
-66.865380	58.951130	673.686200	54.417820
-64.939080	58.927340	626.613300	47.167960
-63.017780	58.874950	559.893700	34.028770
-61.105210	58.794160	517.210700	21.733850
-59.204930	58.685300	493.407000	11.206770
-57.320400	58.548780	480.644500	6.464553
-55.454840	58.385130	474.711300	3.080435
-53.611270	58.194950	473.341000	1.744592
-51.792370	57.978940	473.810800	.865178
-50.000500	57.737840	474.271400	.319085
-48.237630	57.472490	474.455400	.079827
-46.505310	57.183730	474.502100	.015625
-44.804610	56.872430	474.506100	.012279

-43.136050	56.539500	474.503700	.007363
-41.499610	56.185780	474.501700	.003257
-39.894630	55.812120	474.500900	.001177
-38.319800	55.419290	474.500600	.000364
-36.773090	55.007980	474.500500	.000142
-35.251730	54.578750	474.500500	.000094
-33.752120	54.132060	474.500500	.000153
-101.073700	55.260130	475.651100	1.437019
-99.529010	55.710320	475.515200	1.311502
-97.960900	56.142140	475.531900	1.317496
-96.365430	56.555100	475.676600	1.460753
-94.739640	56.948590	475.998200	1.775906
-93.081440	57.321860	476.563300	2.298318
-91.389490	57.674070	477.391800	3.027670
-89.663250	58.004340	478.287200	3.950527
-87.902820	58.311730	478.372300	5.567422
-86.109000	58.595320	475.635800	11.242080
-84.283100	58.854170	461.439500	26.625200
-82.427000	59.087400	430.431500	48.682570
-80.543050	59.294160	542.095300	33.420940
-78.634010	59.473690	753.003700	73.239780
-76.703000	59.625280	688.682500	68.607220
-74.753460	59.748340	669.398200	84.493660
-72.789070	59.842370	708.822600	84.759930
-70.813740	59.906970	687.956000	49.549750
-68.831480	59.941910	700.563400	54.293250
-66.846440	59.947010	686.106600	54.253240
-64.862770	59.922250	616.881200	45.747740
-62.884610	59.867760	543.066700	29.617080
-60.916040	59.783740	508.587600	17.462590
-58.961010	59.670550	492.854900	10.441780
-57.023270	59.528640	482.103000	5.802500
-55.106350	59.358580	476.459200	2.420299
-53.213500	59.161040	474.696200	.874466
-51.347600	58.936760	474.416400	.361543
-49.511150	58.686560	474.443100	.144183
-47.706190	58.411320	474.482700	.046848
-45.934240	58.111950	474.497300	.011280
-44.196280	57.789400	474.500900	.002719
-42.492650	57.444580	474.501100	.001657
-40.823030	57.078400	474.500800	.000840
-39.186360	56.691730	474.500600	.000328
-37.580830	56.285340	474.500500	.000142
-36.003750	55.859910	474.500500	.000094
-34.451600	55.415950	474.500500	.000092
-32.919830	54.953830	474.500500	.000153
-101.937200	56.083360	474.833600	.522100
-100.358100	56.549350	474.783100	.463700
-98.756940	56.996240	474.785800	.462940
-97.128790	57.423640	474.832900	.519252
-95.469860	57.830970	474.935000	.638662
-93.777320	58.217490	475.095900	.814923
-92.049300	58.582370	475.247000	1.028905
-90.284800	58.924680	475.047900	1.590914
-88.483640	59.243460	473.398000	4.465085
-86.646400	59.537720	467.180800	14.180560
-84.774360	59.806450	443.942000	37.617650
-82.869400	60.048700	396.390900	71.436970
-80.933970	60.263580	525.950900	28.365750
-78.971000	60.450230	680.894000	67.791900
-76.983890	60.607900	498.882000	93.383720
-74.976370	60.735940	548.380100	80.518850
-72.952470	60.833800	606.402600	59.235930
-70.916480	60.901050	675.509400	48.787900
-68.872850	60.937420	739.687300	54.691200
-66.826130	60.942730	693.625100	53.300620
-64.780930	60.916960	595.275300	39.962080

-62.741830 60.860230 523.392800 23.891140
-60.713310 60.772790 492.537000 12.413160
-58.699720 60.654990 483.165200 6.072236
-56.705200 60.507380 478.557000 3.229928
-54.733590 60.330550 475.808000 1.416846
-52.788400 60.125220 474.820800 .513950
-50.872730 59.892220 474.555700 .179347
-48.989200 59.632420 474.503500 .063717
-47.139900 59.346780 474.498400 .021427
-45.326320 59.036270 474.499800 .006167
-43.549270 58.701890 474.500400 .001423
-41.808860 58.344600 474.500600 .000409
-40.104370 57.965370 474.500500 .000201
-38.434220 57.565060 474.500500 .000123
-36.795950 57.144450 474.500500 .000092
-35.186060 56.704150 474.500500 .000092
-33.600030 56.244660 474.500500 .000092
-32.032190 55.766200 474.500500 .000153
-102.859900 56.896880 474.589600 .173681
-101.242000 57.379640 474.573200 .149755
-99.604390 57.842470 474.573100 .147673
-97.940930 58.285070 474.585200 .164353
-96.246660 58.706940 474.606000 .193517
-94.517820 59.107400 474.609200 .228073
-92.751850 59.485580 474.467800 .467423
-90.947170 59.840560 473.704100 1.740192
-89.103220 60.171320 470.885800 6.036613
-87.220290 60.476800 461.655400 18.743060
-85.299550 60.755960 429.802900 48.635770
-83.342830 61.007760 367.959100 93.983840
-81.352710 61.231220 490.449500 31.339240
-79.332320 61.425420 582.160500 45.396820
-77.285290 61.589540 400.163900 74.881950
-75.215710 61.722870 303.170200 129.474400
-73.128000 61.824790 301.243900 151.421100
-71.026890 61.894870 554.867400 76.232600
-68.917300 61.932760 748.526700 62.234530
-66.804310 61.938290 689.520300 51.391190
-64.692990 61.911450 570.606000 33.109680
-62.588420 61.852340 502.976400 19.032960
-60.495590 61.761240 476.775700 10.439060
-58.419300 61.638580 473.242900 4.579753
-56.364090 61.484900 474.499400 1.451158
-54.334160 61.300890 474.698600 .552565
-52.333320 61.087320 474.590700 .219935
-50.364880 60.845090 474.526800 .081233
-48.431600 60.575160 474.506000 .028092
-46.535590 60.278550 474.501300 .009212
-44.678250 59.956310 474.500700 .002705
-42.860180 59.609490 474.500400 .000714
-41.081100 59.239120 474.500500 .000160
-39.339830 58.846180 474.500500 .000094
-37.634110 58.431540 474.500500 .000091
-35.960610 57.995940 474.500500 .000092
-34.314830 57.539980 474.500500 .000092
-32.690990 57.064010 474.500500 .000092
-31.082000 56.568130 474.500500 .000153
-103.849600 57.699570 474.522700 .052839
-102.187300 58.200280 474.517900 .043864
-100.509000 58.680030 474.517100 .041616
-98.806840 59.138710 474.517600 .042183
-97.074520 59.575940 474.509800 .044369
-95.307120 59.991050 474.453900 .141850
-93.501110 60.383240 474.188400 .598936
-91.654170 60.751550 473.160000 2.136944
-89.765210 61.094930 469.605200 7.026378
-87.834170 61.412270 457.792100 20.900840

-85.861950	61.702450	422.351000	50.815190
-83.850420	61.964360	378.715000	84.209820
-81.802150	62.196920	489.701200	26.007140
-79.720490	62.399150	517.123800	45.162320
-77.609360	62.570130	329.066400	76.958840
-75.473210	62.709080	162.659000	137.235100
-73.316960	62.815350	136.001100	139.613400
-71.145790	62.888430	305.237400	119.422600
-68.965200	62.927950	664.946400	97.899800
-66.780790	62.933720	646.598600	44.079880
-64.598260	62.905720	541.938100	26.720570
-62.423240	62.844070	492.860100	14.068780
-60.261290	62.749080	477.469600	6.604898
-58.117710	62.621230	474.273700	2.657781
-55.997520	62.461110	474.306200	.890127
-53.905320	62.269450	474.435900	.286332
-51.845240	62.047150	474.484100	.088961
-49.820820	61.795150	474.497300	.028358
-47.834930	61.514500	474.499900	.009665
-45.889670	61.206310	474.500300	.003284
-43.986290	60.871700	474.500200	.001050
-42.125070	60.511780	474.500300	.000327
-40.305270	60.127630	474.500500	.000101
-38.525020	59.720250	474.500500	.000089
-36.781200	59.290500	474.500500	.000092
-35.069400	58.839090	474.500500	.000092
-33.383770	58.366500	474.500500	.000092
-31.716990	57.872960	474.500500	.000092
-30.060130	57.358350	474.500500	.000153
-104.916500	58.490070	474.505800	.014583
-103.202300	59.010120	474.504300	.011350
-101.477600	59.507980	474.503400	.009245
-99.732440	59.983750	474.500400	.007971
-97.958720	60.437220	474.484800	.038722
-96.150010	60.867820	474.414000	.171233
-94.301540	61.274790	474.132500	.623348
-92.410060	61.657170	473.128200	2.036470
-90.473700	62.013880	469.802500	6.182940
-88.491920	62.343760	458.776100	17.244600
-86.465330	62.645610	432.383100	38.895240
-84.395630	62.918240	435.266600	48.901610
-82.285480	63.160470	532.727600	40.941270
-80.138370	63.371240	500.988300	29.114050
-77.958560	63.549540	369.136300	54.112600
-75.750930	63.694510	246.413800	103.170900
-73.520860	63.805420	174.410700	131.556600
-71.274150	63.881700	268.408100	93.339420
-69.016910	63.922960	510.278900	44.037930
-66.755400	63.928990	543.702300	27.396460
-64.495980	63.899750	507.812300	16.057160
-62.244980	63.835380	486.386300	8.275189
-60.008560	63.736250	477.808300	3.654220
-57.792640	63.602840	475.031700	1.462939
-55.602750	63.435840	474.468800	.526185
-53.443960	63.236060	474.438400	.166273
-51.320750	63.004450	474.472200	.044970
-49.236890	62.742080	474.491100	.010927
-47.195330	62.450080	474.497900	.003100
-45.198120	62.129630	474.499800	.001113
-43.246250	61.781950	474.500300	.000400
-41.339580	61.408210	474.500300	.000224
-39.476710	61.009530	474.500400	.000167
-37.654880	60.586900	474.500500	.000151
-35.869840	60.141170	474.500500	.000092
-34.115770	59.672970	474.500500	.000092
-32.385130	59.182660	474.500500	.000092
-30.668580	58.670220	474.500500	.000104

-28.954870 58.135290 474.500500 .000153
-106.073800 59.266680 474.501600 .003581
-104.297500 59.807790 474.501200 .002350
-102.518800 60.325180 474.500400 .001336
-100.725000 60.819250 474.497000 .009093
-98.905530 61.289990 474.481800 .041477
-97.052100 61.737010 474.420100 .154369
-95.158340 62.159620 474.189500 .514475
-93.219700 62.556880 473.419500 1.572300
-91.233350 62.927700 470.919400 4.414244
-89.198040 63.270870 463.158800 11.306070
-87.113920 63.585110 450.764900 25.498610
-84.982480 63.869130 483.813900 16.163830
-82.806390 64.121680 511.782300 26.723420
-80.589290 64.341560 455.736500 22.422000
-78.335770 64.527670 385.831100 43.762220
-76.051160 64.679080 331.491300 73.403590
-73.741460 64.794950 312.229600 84.223250
-71.413090 64.874680 362.629900 57.907590
-69.072890 64.917820 459.322300 23.137230
-66.727910 64.924110 489.334200 11.749880
-64.385280 64.893550 486.238900 7.192408
-62.052090 64.826280 479.961500 4.018466
-59.735260 64.722690 476.353000 1.915146
-57.441380 64.583340 474.911900 .779132
-55.176610 64.408980 474.531400 .274737
-52.946510 64.200520 474.476300 .084496
-50.755950 63.959000 474.484600 .023426
-48.608900 63.685590 474.494200 .006876
-46.508400 63.381530 474.498400 .002500
-44.456330 63.048090 474.499900 .000926
-42.453320 62.686580 474.500300 .000351
-40.498620 62.298210 474.500400 .000155
-38.589950 61.884150 474.500500 .000145
-36.723370 61.445380 474.500500 .000157
-34.893150 60.982680 474.500500 .000102
-33.091610 60.496560 474.500500 .000092
-31.309040 59.987160 474.500500 .000092
-29.533510 59.454220 474.500500 .000118
-27.750760 58.896990 474.500500 .000153
-107.339200 60.027230 474.500700 .000729
-105.487000 60.591610 474.500500 .000275
-103.643800 61.130270 474.499800 .002069
-101.793500 61.644070 474.496800 .009597
-99.922550 62.133320 474.484500 .036034
-98.020040 62.597820 474.438100 .120601
-96.077510 63.037030 474.280700 .366398
-94.088710 63.450070 473.802000 1.005853
-92.049460 63.835860 472.523700 2.472408
-89.957580 64.193130 469.246700 5.141247
-87.812570 64.520550 465.228800 7.398378
-85.615560 64.816710 481.838900 12.515900
-83.369120 65.080260 470.316400 17.344650
-81.077050 65.309890 444.689800 18.480960
-78.744270 65.504390 425.285300 27.636160
-76.376630 65.662700 412.022500 40.486480
-73.980770 65.783920 411.426900 42.771390
-71.563890 65.867360 432.042600 29.789180
-69.133670 65.912500 461.225100 13.908000
-66.698060 65.919100 475.292800 5.747766
-64.265090 65.887100 477.551800 2.860595
-61.842790 65.816700 476.434000 1.701758
-59.438900 65.708320 475.276800 .886466
-57.060850 65.562580 474.717500 .381341
-54.715470 65.380330 474.533500 .136241
-52.408880 65.162570 474.495100 .040720
-50.146370 64.910470 474.494000 .011342

-47.932120	64.625290	474.497500	.004200
-45.769150	64.308400	474.499500	.001906
-43.659080	63.961160	474.500200	.000785
-41.602020	63.584970	474.500400	.000304
-39.596350	63.181100	474.500500	.000182
-37.638600	62.750720	474.500500	.000147
-35.723270	62.294770	474.500500	.000159
-33.842640	61.813940	474.500500	.000135
-31.986660	61.308540	474.500500	.000136
-30.142740	60.778430	474.500500	.000092
-28.295590	60.222970	474.500500	.000149
-26.427080	59.640870	474.500500	.000153
-108.736700	60.768890	474.500500	.000191
-106.789100	61.359350	474.500400	.000585
-104.866900	61.921510	474.499600	.002724
-102.949500	62.456830	474.496500	.010381
-101.019200	62.966030	474.484500	.035182
-99.061880	63.449270	474.444000	.107572
-97.066180	63.906190	474.317200	.295817
-95.023590	64.336020	473.976100	.722185
-92.928180	64.737730	473.208900	1.491341
-90.776390	65.110020	471.796400	2.426545
-88.566850	65.451470	470.252200	2.500858
-86.300130	65.760610	473.254800	6.736858
-83.978550	66.035940	464.002600	10.072930
-81.606060	66.276030	453.911400	12.673350
-79.187900	66.479550	447.469700	17.826130
-76.730480	66.645290	443.616800	23.234260
-74.241170	66.772270	444.871000	24.331180
-71.728080	66.859700	453.753600	18.148030
-69.199870	66.907030	465.334500	10.102030
-66.665550	66.913940	472.604200	4.390900
-64.134220	66.880400	475.035200	1.742900
-61.614990	66.806620	475.192700	.864802
-59.116610	66.693070	474.866000	.477418
-56.647430	66.540450	474.628200	.226852
-54.215080	66.349720	474.529400	.087205
-51.826330	66.121990	474.501800	.026835
-49.486860	65.858530	474.498000	.007131
-47.201050	65.560780	474.499000	.002869
-44.971820	65.230190	474.499900	.001507
-42.800360	64.868270	474.500200	.000675
-40.685980	64.476460	474.500400	.000309
-38.625890	64.056100	474.500500	.000170
-36.614990	63.608330	474.500500	.000163
-34.645670	63.134020	474.500500	.000138
-32.707610	62.633670	474.500500	.000146
-30.787590	62.107330	474.500500	.000149
-28.869240	61.554470	474.500500	.000149
-26.932890	60.973880	474.500500	.000150
-24.955330	60.363580	474.500500	.000151

Q.23a

```
-89.453400 32.991660 500.661300 1.614886
-88.345930 33.191160 500.791600 1.822651
-87.228100 33.381660 501.290000 2.602843
-86.100230 33.562890 502.127500 3.873677
-84.962690 33.734530 503.259400 5.516497
-83.815900 33.896310 504.564700 7.331441
-82.660300 34.047950 505.831100 9.028542
-81.496410 34.189190 506.771800 10.255680
-80.324730 34.319790 507.085000 10.644560
-79.145840 34.439500 506.576000 9.903739
-77.960310 34.548100 505.191000 7.990326
-76.768760 34.645400 503.089400 5.339332
-75.571840 34.731200 500.977100 2.989488
-74.370180 34.805370 499.999700 1.828461
-73.164480 34.867740 500.319300 1.603258
-71.955430 34.918200 500.996100 1.987978
-70.743730 34.956650 501.382200 2.381461
-69.530080 34.983020 501.357700 2.382918
-68.315220 34.997260 501.081800 2.023999
-67.099880 34.999340 500.719800 1.488979
-65.884770 34.989250 500.382900 .956973
-64.670640 34.967020 500.135300 .539412
-63.458190 34.932690 499.987100 .269350
-62.248150 34.886320 499.912500 .121483
-61.041230 34.828000 499.880500 .052974
-59.838120 34.757850 499.868700 .027475
-58.639500 34.676010 499.865600 .020862
-57.446040 34.582610 499.865600 .019546
-56.258390 34.477850 499.865800 .018180
-55.077160 34.361910 499.864700 .015031
-53.902950 34.235010 499.862300 .010475
-52.736340 34.097380 499.860000 .006176
-51.577860 33.949260 499.858200 .003121
-50.428010 33.790910 499.857500 .001365
-49.287250 33.622600 499.857100 .000529
-48.156040 33.444620 499.857000 .000204
-47.034740 33.257250 499.857000 .000145
-45.923690 33.060790 499.857000 .000149
-44.823220 32.855560 499.857000 .000153
-89.701210 33.918400 501.424100 2.771508
-88.583520 34.125020 501.770400 3.234712
-87.455040 34.322360 502.873500 4.671875
-86.316120 34.510110 504.696300 6.954251
-85.167100 34.687960 507.180900 9.897731
-84.008420 34.855620 510.071700 13.151670
-82.840550 35.012790 512.916500 16.213580
-81.663990 35.159200 515.074400 18.473510
-80.479300 35.294580 515.847800 19.261140
-79.287040 35.418700 514.785000 17.981280
-78.087840 35.531310 511.689700 14.407220
-76.882320 35.632210 506.754800 9.320387
-75.671150 35.721200 501.462400 4.874269
-74.455030 35.798110 499.366000 2.995371
-73.234660 35.862810 500.708200 2.833146
-72.010770 35.915150 502.658000 3.879786
-70.784100 35.955040 503.537600 4.693744
-69.555380 35.982390 503.381800 4.669041
-68.325390 35.997170 502.679200 3.939153
-67.094890 35.999320 501.816900 2.878423
```

-65.864640	35.988860	501.020000	1.822850
-64.635410	35.965800	500.451600	1.010336
-63.407950	35.930180	500.125800	.496492
-62.183030	35.882080	499.970200	.223151
-60.961370	35.821590	499.906300	.099824
-59.743720	35.748840	499.883700	.056220
-58.530780	35.663950	499.878100	.045972
-57.323260	35.567100	499.879100	.043918
-56.121820	35.458470	499.880200	.040694
-54.927120	35.338260	499.876800	.032739
-53.739770	35.206700	499.870100	.021775
-52.560380	35.064020	499.863700	.012024
-51.389480	34.910500	499.859900	.005698
-50.227610	34.746390	499.857900	.002344
-49.075240	34.571990	499.857300	.000854
-47.932830	34.387580	499.857000	.000275
-46.800740	34.193490	499.857000	.000144
-45.679330	33.990000	499.857000	.000151
-44.568900	33.777440	499.857000	.000153
-89.960660	34.843320	503.855400	6.009506
-88.832330	35.057210	504.962600	7.210461
-87.692770	35.261540	508.077000	10.427350
-86.542320	35.455950	513.160600	15.428080
-85.381340	35.640140	520.132100	21.843100
-84.210270	35.813810	528.303000	28.942500
-83.029580	35.976620	536.411000	35.647750
-81.839790	36.128310	542.696000	40.710510
-80.641480	36.268600	545.130600	42.672740
-79.435230	36.397220	542.390400	40.018740
-78.221690	36.513940	533.665600	31.910520
-77.001530	36.618520	519.279400	20.175180
-75.775430	36.710780	502.276200	9.814735
-74.544130	36.790520	496.362300	6.597457
-73.308360	36.857590	502.189300	6.418358
-72.068890	36.911860	508.715100	9.541905
-70.826490	36.953220	511.258400	11.598540
-69.581950	36.981590	510.443400	11.362390
-68.336070	36.996900	508.211300	9.544495
-67.089650	36.999130	505.586100	6.937047
-65.843500	36.988290	503.173000	4.348791
-64.598400	36.964370	501.492900	2.372824
-63.355190	36.927440	500.577500	1.159711
-62.114630	36.877570	500.161400	.527780
-60.877510	36.814860	499.997300	.249433
-59.644590	36.739430	499.940200	.154733
-58.416640	36.651430	499.926300	.133171
-57.194370	36.551040	499.930800	.128095
-55.978490	36.438440	499.934700	.116437
-54.769680	36.313860	499.922000	.090783
-53.568590	36.177530	499.896900	.057088
-52.375820	36.029710	499.876400	.029689
-51.191970	35.870660	499.864700	.013090
-50.017550	35.700660	499.859600	.005103
-48.853080	35.520030	499.857700	.001739
-47.698980	35.329070	499.857100	.000536
-46.555660	35.128100	499.857000	.000190
-45.423470	34.917440	499.857000	.000151
-44.302690	34.697410	499.857000	.000153
-90.232250	35.766320	509.607900	12.262640
-89.092860	35.987640	512.854700	15.017790
-87.941790	36.199090	521.282000	21.732820
-86.779330	36.400320	534.763000	31.885200
-85.605870	36.591000	553.537300	45.027370
-84.421870	36.770790	575.536800	59.558590
-83.227800	36.939400	597.544700	73.418260
-82.024180	37.096500	615.177300	84.243290
-80.811620	37.241800	622.625200	88.987980

-79.590740	37.375040	616.074800	84.013200
-78.362190	37.495960	593.593000	67.305200
-77.126670	37.604320	553.549900	40.867940
-75.884930	37.699920	495.955400	19.638250
-74.637700	37.782550	485.866500	16.113000
-73.385770	37.852060	507.654400	15.085180
-72.129940	37.908310	527.082300	22.574390
-70.871030	37.951180	533.321000	27.014680
-69.609860	37.980580	530.170800	26.240060
-68.347290	37.996460	523.428300	21.836180
-67.084140	37.998780	515.857400	15.786790
-65.821270	37.987530	508.765900	9.687263
-64.559530	37.962740	504.061600	5.186494
-63.299760	37.924460	501.655700	2.517112
-62.042780	37.872770	500.629900	1.172401
-60.789440	37.807780	500.241300	.598773
-59.540510	37.729610	500.104600	.413734
-58.296810	37.638420	500.073100	.373768
-57.059080	37.534390	500.088000	.355753
-55.828080	37.417740	500.105600	.322714
-54.604500	37.288680	500.058300	.240936
-53.389020	37.147460	499.970100	.139217
-52.182290	36.994370	499.907200	.067126
-50.984880	36.829660	499.875700	.027732
-49.797380	36.653650	499.862900	.009976
-48.620270	36.466650	499.858600	.003250
-47.454020	36.268990	499.857300	.000940
-46.299010	36.060990	499.857000	.000243
-45.155610	35.843010	499.857000	.000149
-44.024070	35.615370	499.857000	.000153
-90.516530	36.687310	521.400400	22.144180
-89.365660	36.916220	529.985400	27.662470
-88.202590	37.134950	550.820400	40.085670
-87.027650	37.343150	583.139500	58.079850
-85.841200	37.540460	630.028600	82.358760
-84.643710	37.726540	684.177900	109.121000
-83.435650	37.901050	738.738000	134.763100
-82.217600	38.063690	786.262900	156.185300
-80.990140	38.214140	807.344800	167.216200
-79.753940	38.352110	792.946400	160.469300
-78.509670	38.477340	740.712700	129.648300
-77.258070	38.589580	633.747000	74.963460
-75.999910	38.688600	448.689400	43.336530
-74.735980	38.774210	426.532000	32.478710
-73.467090	38.846230	541.269500	50.736950
-72.194090	38.904510	583.826800	52.207920
-70.917830	38.948930	591.771200	57.633310
-69.639210	38.979390	579.414200	54.560720
-68.359090	38.995840	561.437000	45.374180
-67.078360	38.998240	541.657200	32.773510
-65.797930	38.986590	521.509800	19.200350
-64.518690	38.960900	509.335200	9.918597
-63.241520	38.921240	503.806900	4.821488
-61.967320	38.867690	501.637300	2.357610
-60.696920	38.800350	500.830500	1.338915
-59.431210	38.719360	500.533900	1.039652
-58.170970	38.624900	500.463300	.963245
-56.917050	38.517150	500.525400	.929886
-55.670200	38.396340	500.663600	.894808
-54.431160	38.262690	500.504300	.666836
-53.200640	38.116470	500.137000	.300201
-51.979310	37.957960	499.966100	.127506
-50.767770	37.787460	499.893700	.048411
-49.566610	37.605300	499.867900	.016338
-48.376340	37.411790	499.859600	.004841
-47.197410	37.207270	499.857500	.001297
-46.030250	36.992090	499.857000	.000283

-44.875180	36.766620	499.857000	.000150
-43.732470	36.531200	499.857000	.000153
-90.814130	37.606180	541.702600	34.494980
-89.651300	37.842850	562.063000	44.414350
-88.475770	38.069030	611.563400	65.879420
-87.287830	38.284350	677.224400	90.908700
-86.087840	38.488450	788.214700	130.901300
-84.876290	38.680970	912.954700	174.841400
-83.653650	38.861550	1037.453000	214.511600
-82.420500	39.029860	1156.555000	248.362800
-81.177470	39.185590	1212.577000	273.649900
-79.925230	39.328410	1188.820000	274.652300
-78.664510	39.458070	1096.484000	229.805500
-77.396060	39.574290	830.780300	127.174000
-76.120680	39.676830	219.262500	125.791000
-74.839220	39.765490	244.783800	75.149170
-73.552520	39.840080	624.266800	79.236950
-72.261480	39.900440	755.507500	121.769900
-70.967010	39.946460	737.396500	109.658300
-69.670030	39.978020	693.705800	101.170000
-68.371470	39.995060	650.425500	84.439830
-67.072270	39.997550	604.371200	61.740650
-65.773400	39.985470	545.804600	32.469810
-64.475770	39.958870	518.109600	16.257060
-63.180340	39.917780	507.269700	8.035309
-61.888020	39.862300	503.643100	4.382897
-60.599740	39.792560	502.355700	3.052437
-59.316390	39.708680	501.794000	2.664013
-58.038840	39.610860	501.711500	2.627460
-56.767930	39.499290	501.950200	2.672432
-55.504470	39.374200	502.329300	2.424664
-54.249260	39.235840	501.685400	1.640018
-53.003000	39.084480	500.558800	.684832
-51.766410	38.920440	500.036200	.191112
-50.540120	38.744010	499.909200	.064518
-49.324710	38.555530	499.870700	.020802
-48.120720	38.355340	499.860200	.006096
-46.928620	38.143810	499.857500	.001507
-45.748810	37.921300	499.857000	.000316
-44.581620	37.688170	499.856900	.000148
-43.427300	37.444810	499.856900	.000120
-91.125630	38.522790	568.638000	45.267680
-89.950410	38.767390	616.388600	63.395790
-88.761920	39.001200	720.471900	91.518210
-87.560450	39.223820	837.055600	116.092000
-86.346370	39.434880	1070.853000	156.346600
-85.120160	39.633990	1338.320000	217.747400
-83.882300	39.820790	1594.156000	267.473900
-82.633390	39.994930	1862.567000	305.090600
-81.374080	40.156070	1998.301000	353.682900
-80.105060	40.303890	1970.325000	388.936100
-78.827100	40.438090	1887.412000	350.272400
-77.540980	40.558400	1580.329000	216.544300
-76.247540	40.664560	532.482600	162.685000
-74.947690	40.756360	23.306140	89.207840
-73.642300	40.833600	966.366000	207.809800
-72.332310	40.896110	1085.329000	138.612100
-71.018690	40.943760	1037.287000	161.543500
-69.702430	40.976440	914.167100	150.452500
-68.384490	40.994090	835.033800	130.999200
-67.065880	40.996670	730.120600	90.484090
-65.747600	40.984170	578.224900	43.689510
-64.430650	40.956610	526.296300	21.661780
-63.116020	40.914060	512.434400	12.456780
-61.804690	40.856610	508.331100	8.489382
-60.497620	40.784390	506.672300	7.538036
-59.195770	40.697540	505.486000	7.357337

-57.900040	40.596260	504.790700	7.101551
-56.611330	40.480750	505.631100	7.200195
-55.330490	40.351270	505.973300	4.585867
-54.058330	40.208070	503.899200	2.665643
-52.795620	40.051450	500.685400	.772201
-51.543080	39.881710	500.079300	.258791
-50.301390	39.699200	499.896900	.078064
-49.071130	39.504250	499.867300	.028809
-47.852850	39.297230	499.859300	.009883
-46.647030	39.078520	499.857300	.002916
-45.454070	38.848490	499.856900	.000734
-44.274280	38.607530	499.856900	.000218
-43.107920	38.356030	499.856900	.000143
-91.451740	39.437030	591.374300	47.806300
-90.263630	39.689750	674.929200	69.097720
-89.061680	39.931370	900.462500	105.244000
-87.846150	40.161480	971.309900	94.578740
-86.617400	40.379670	1340.389000	113.061900
-85.375900	40.585540	1823.899000	187.088200
-84.122160	40.778730	2234.873000	255.963800
-82.856800	40.958850	2597.454000	326.739200
-81.580460	41.125550	2880.964000	396.868500
-80.293880	41.278500	3018.144000	453.984100
-78.997860	41.417370	3026.239000	458.052400
-77.693230	41.541890	2758.028000	368.928900
-76.380870	41.651780	1892.304000	192.448600
-75.061690	41.746810	878.046000	188.396600
-73.736660	41.826770	1042.402000	115.773200
-72.406780	41.891490	1090.287000	101.696200
-71.073040	41.940830	1188.394000	137.583800
-69.736500	41.974670	1122.975000	172.451500
-68.398190	41.992950	1019.384000	143.017100
-67.059160	41.995620	832.253100	112.680900
-65.720480	41.982670	495.182600	36.643280
-64.383220	41.954140	521.692700	27.568440
-63.048400	41.910080	523.736600	21.880970
-61.717090	41.850600	518.529900	15.628540
-60.390290	41.775820	514.228900	13.998970
-59.069010	41.685920	509.165700	12.902050
-57.754210	41.581080	507.772600	11.466560
-56.446830	41.461530	508.397400	9.432015
-55.147770	41.327530	502.670000	2.635238
-53.857880	41.179360	496.616500	2.675775
-52.577970	41.017320	498.935900	1.057645
-51.308780	40.841740	499.671000	.402100
-50.051000	40.652980	499.866900	.162786
-48.805250	40.451390	499.869400	.069774
-47.572090	40.237360	499.862300	.025443
-46.352010	40.011290	499.858600	.008087
-45.145370	39.773560	499.857100	.002208
-43.952510	39.524580	499.856900	.000513
-42.773630	39.264750	499.856900	.000182
-91.793200	40.348750	572.956800	30.404470
-90.591710	40.609810	637.830800	38.638880
-89.375760	40.859430	820.487500	79.026850
-88.145600	41.097210	803.235100	119.385200
-86.901570	41.322720	825.675500	158.639000
-85.644150	41.535550	1394.831000	77.467900
-84.373860	41.735290	2143.896000	209.419900
-83.091290	41.921560	2572.701000	360.112100
-81.797160	42.093980	2997.585000	471.463300
-80.492230	42.252200	3453.757000	548.650200
-79.177290	42.395880	3715.275000	567.526100
-77.853240	42.524730	3541.442000	506.670800
-76.521000	42.638450	2774.372000	341.056000
-75.181550	42.736820	1961.336000	167.555500
-73.835900	42.819590	1264.068000	97.115880

-72.485100 42.886590 563.462400 215.460500
-71.130210 42.937670 1168.732000 205.230300
-69.772340 42.972710 1025.971000 190.606000
-68.412590 42.991630 922.792400 157.830000
-67.052090 42.994400 415.805100 121.266500
-65.691960 42.980990 208.441200 124.794400
-64.333320 42.951450 550.086100 55.622790
-62.977290 42.905830 551.423500 34.337400
-61.624970 42.844260 526.427200 22.730810
-60.277440 42.766850 491.615800 17.116560
-58.935750 42.673790 470.109300 17.877370
-57.600940 42.565280 460.718400 22.207830
-56.273990 42.441570 495.559800 9.230193
-54.955840 42.302930 498.766200 5.951674
-53.647390 42.149640 499.893800 3.179540
-52.349470 41.982030 500.290000 1.582274
-51.062890 41.800450 500.278500 .894577
-49.788320 41.605260 500.058500 .441549
-48.526440 41.396860 499.936800 .186906
-47.277780 41.175640 499.884500 .069899
-46.042850 40.942000 499.865300 .023037
-44.822010 40.696380 499.859000 .006642
-43.615560 40.439180 499.857400 .001691
-42.423690 40.170830 499.857100 .000537
-92.150770 41.257820 513.422600 13.549430
-90.935390 41.527410 491.440200 32.501940
-89.704910 41.785250 382.034300 107.303000
-88.459540 42.030900 334.001600 205.845900
-87.199620 42.263930 181.140000 185.254200
-85.925610 42.483890 862.422100 120.528200
-84.638040 42.690380 1970.677000 217.310900
-83.337520 42.882970 2648.958000 384.101100
-82.024790 43.061280 3265.601000 522.160200
-80.700610 43.224930 3932.539000 609.974000
-79.365870 43.373580 4177.190000 647.401700
-78.021450 43.506890 3942.429000 577.571800
-76.668370 43.624570 3214.003000 416.878300
-75.307630 43.726360 2322.184000 231.455300
-73.940300 43.812040 1417.399000 182.096700
-72.567500 43.881390 77.860390 120.000000
-71.190370 43.934270 540.115300 179.131500
-69.810050 43.970550 1279.645000 196.456300
-68.427750 43.990140 1303.012000 183.677200
-67.044650 43.993000 725.696700 121.998800
-65.661930 43.979120 606.618600 59.388400
-64.280810 43.948540 674.398800 71.417280
-62.902460 43.901310 556.098500 41.897030
-61.528050 43.837570 461.657800 38.948240
-60.158730 43.757450 426.596200 44.257330
-58.795620 43.661140 451.535900 31.609110
-57.439790 43.548850 509.228700 14.784730
-56.092310 43.420850 524.245300 15.472130
-54.754150 43.277410 513.954300 10.339920
-53.426270 43.118850 507.209500 7.018628
-52.109530 42.945500 503.574600 4.311151
-50.804760 42.757750 501.544100 2.314580
-49.512690 42.555960 500.543400 1.090879
-48.233980 42.340550 500.109600 .457998
-46.969190 42.111940 499.941100 .171042
-45.718810 41.870550 499.882100 .056920
-44.483210 41.616830 499.863600 .016885
-43.262650 41.351200 499.858600 .004501
-42.057300 41.074100 499.857400 .001495
-92.525320 42.164070 484.477600 17.741570
-91.295520 42.442420 428.143300 42.457200
-90.049940 42.708690 236.130000 89.896400
-88.788760 42.962440 84.037320 119.156400

-87.512310 43.203190 292.647200 207.316400
-86.221000 43.430500 724.847500 76.095690
-84.915410 43.643920 1670.823000 209.623100
-83.596150 43.843030 2515.337000 392.146200
-82.263960 44.027400 3215.346000 553.481100
-80.919660 44.196660 4122.688000 618.983600
-79.564150 44.350410 4336.544000 623.780100
-78.198390 44.488330 3921.313000 536.605600
-76.823410 44.610100 2844.631000 340.247500
-75.440290 44.715440 1731.424000 160.447900
-74.050180 44.804110 971.849400 163.988900
-72.654240 44.875890 179.925300 174.555300
-71.253690 44.930630 789.709900 180.277600
-69.849760 44.968180 1690.740000 229.611100
-68.443720 44.988460 2162.751000 310.736900
-67.036810 44.991420 1391.751000 160.574000
-65.630330 44.977050 921.684400 91.927090
-64.225530 44.945390 739.482200 74.427520
-62.823690 44.896510 450.229500 84.870280
-61.426040 44.830540 459.658800 68.075220
-60.033810 44.747610 451.361100 66.973880
-58.648170 44.647940 506.994400 50.712220
-57.270290 44.531750 568.315100 39.420360
-55.901260 44.399320 557.710800 34.303020
-54.542130 44.250930 533.856800 24.236500
-53.193900 44.086940 518.353500 15.836790
-51.857480 43.907680 509.190200 9.422503
-50.533710 43.713560 504.021400 4.932572
-49.223370 43.504980 501.515100 2.293971
-47.927120 43.282370 500.453800 .953454
-46.645540 43.046150 500.052300 .355346
-45.379100 42.796800 499.915000 .118754
-44.128160 42.534760 499.872500 .035639
-42.892960 42.260480 499.860700 .009746
-41.673610 41.974410 499.858100 .003320
-92.917770 43.067330 501.709400 12.491120
-91.673000 43.354680 496.707400 19.430540
-90.411740 43.629630 498.415700 42.549220
-89.134110 43.891690 527.951500 110.529100
-87.840450 44.140390 345.891400 139.109800
-86.531140 44.375260 10.749690 55.569990
-85.206750 44.595830 763.199500 216.235500
-83.867900 44.801650 2267.122000 319.787100
-82.515380 44.992280 2929.421000 460.243400
-81.150000 45.167310 3295.524000 503.351600
-79.772730 45.326340 3374.934000 483.113800
-78.384560 45.469020 3073.254000 384.579500
-76.986600 45.595010 2240.023000 205.202200
-75.579970 45.704020 1490.449000 93.460360
-74.165890 45.795780 357.095100 268.676300
-72.745610 45.870080 94.110130 97.076240
-71.320400 45.926740 371.630400 196.525100
-69.891600 45.965610 1726.307000 218.729400
-68.460530 45.986610 2291.078000 342.354700
-67.028560 45.989670 1344.526000 201.274900
-65.597030 45.974800 927.021500 105.530200
-64.167300 45.942020 862.368000 107.440600
-62.740720 45.891430 603.632800 61.079590
-61.318600 45.823140 159.984300 144.855500
-59.902260 45.737310 443.524000 45.153590
-58.492950 45.634170 605.178500 53.239200
-57.091900 45.513950 620.063800 64.424250
-55.700260 45.376930 586.118200 56.717710
-54.319140 45.223450 561.991900 43.424320
-52.949590 45.053840 539.338800 29.359600
-51.592580 44.868490 520.046900 17.466280
-50.248970 44.667810 508.758500 9.092424

-48.919560	44.452230	503.296000	4.169275
-47.605040	44.222190	501.053800	1.709772
-46.305980	43.978160	500.238300	.632112
-45.022840	43.720610	499.968500	.210955
-43.755960	43.450020	499.886800	.063498
-42.505550	43.166850	499.864200	.017522
-41.271660	42.871580	499.859200	.005992
-93.329110	43.967430	524.103700	26.022190
-92.068780	44.264030	555.933100	40.950190
-90.791210	44.547890	652.841700	77.993530
-89.496510	44.818510	841.178300	156.910700
-88.184940	45.075390	1050.744000	455.275800
-86.856880	45.318050	263.042300	169.251100
-85.512890	45.545990	702.167700	77.628360
-84.153590	45.758730	2011.320000	246.009300
-82.779790	45.955820	2321.122000	306.509200
-81.392350	46.136820	2214.124000	306.958200
-79.992260	46.301310	1912.842000	246.100800
-78.580580	46.448910	1611.740000	156.000900
-77.158460	46.579270	1294.716000	84.995160
-75.727110	46.692070	1458.401000	131.575100
-74.287820	46.787040	1583.305000	301.648600
-72.841890	46.863950	466.993600	306.120700
-71.390720	46.922590	502.826500	224.666500
-69.935700	46.962830	1993.652000	385.393900
-68.478260	46.984570	1748.596000	212.340900
-67.019850	46.987740	1126.065000	105.444500
-65.561920	46.972340	550.402800	113.417500
-64.105920	46.938410	701.840600	83.226490
-62.653260	46.886040	671.068300	79.987530
-61.205390	46.815350	422.015200	103.693600
-59.763680	46.726520	560.545200	55.727420
-58.329470	46.619790	716.380100	69.619840
-56.904060	46.495390	713.227400	92.814100
-55.488670	46.353640	668.253700	91.349400
-54.084500	46.194880	649.286500	73.348660
-52.692630	46.019470	590.086500	51.594680
-51.314070	45.827840	541.107200	29.463950
-49.949730	45.620390	516.566600	14.658490
-48.600430	45.397590	505.984800	6.539179
-47.266860	45.159900	501.896900	2.627841
-45.949600	44.907820	500.483200	.957518
-44.649100	44.641840	500.035300	.317041
-43.365670	44.362450	499.903900	.095178
-42.099450	44.070140	499.868300	.026291
-40.850440	43.765410	499.860400	.008964
-93.760410	44.864160	539.314300	40.041550
-92.483920	45.170280	565.044900	54.168930
-91.189420	45.463320	617.085400	78.132550
-89.876960	45.742750	521.556000	77.967730
-88.546760	46.008070	116.406500	120.101500
-87.199170	46.258760	15.855470	63.884790
-85.834720	46.494300	796.750700	109.575800
-84.454070	46.714200	1465.961000	180.589100
-83.058010	46.917960	1531.516000	154.245500
-81.647480	47.105130	1245.419000	174.775100
-80.223460	47.275260	1097.008000	81.577120
-78.787090	47.427960	689.774700	104.830900
-77.339580	47.562840	221.783200	207.090900
-75.882230	47.679570	957.607700	105.346400
-74.416370	47.777870	1541.878000	181.895600
-72.943440	47.857470	1578.077000	198.935600
-71.464880	47.918190	1405.762000	134.562500
-69.982220	47.959850	1532.158000	164.343300
-68.496960	47.982350	1249.829000	161.031500
-67.010670	47.985630	837.040500	53.105020
-65.524890	47.969690	253.576000	205.097100

-64.041170	47.934570	384.027900	95.961160
-62.561040	47.880340	658.038100	51.012170
-61.086020	47.807170	797.047100	82.398060
-59.617590	47.715230	716.883400	76.965230
-58.157170	47.604770	871.370200	106.760800
-56.706150	47.476050	881.059400	127.899200
-55.265830	47.329400	874.538200	124.333700
-53.837470	47.165180	828.876900	106.140700
-52.422200	46.983780	681.803200	76.154850
-51.021090	46.785640	571.246500	42.094710
-49.635090	46.571200	525.194800	19.812010
-48.265020	46.340950	508.575600	8.542713
-46.911610	46.095370	502.648300	3.367764
-45.575410	45.834990	500.685900	1.210132
-44.256860	45.560320	500.087200	.396674
-42.956210	45.271870	499.916600	.118422
-41.673580	44.970160	499.871300	.032572
-40.408870	44.655690	499.861200	.011032
-94.212860	45.757310	558.656700	54.603840
-92.919560	46.073230	589.307400	67.724440
-91.607470	46.375730	637.028000	84.875840
-90.276550	46.664250	574.363600	65.015600
-88.926960	46.938270	595.727900	64.214840
-87.559010	47.197250	784.352400	184.409400
-86.173240	47.440650	699.809500	185.736000
-84.770290	47.667940	210.649200	188.584800
-83.350960	47.878600	538.846700	235.159700
-81.916200	48.072160	1271.820000	129.357600
-80.467090	48.248140	1109.132000	106.549900
-79.004780	48.406110	118.172500	188.571600
-77.530590	48.545680	198.841000	136.886200
-76.045850	48.666500	677.053300	77.422910
-74.552020	48.768250	1265.671000	121.793300
-73.050600	48.850660	1343.513000	167.066100
-71.543170	48.913520	1324.479000	161.321900
-70.031330	48.956650	1312.283000	168.471900
-68.516710	48.979950	1083.065000	135.723400
-67.000980	48.983350	998.737200	91.736780
-65.485790	48.966840	751.638200	119.512800
-63.972820	48.930470	362.970700	130.043600
-62.463700	48.874330	731.193700	97.684980
-60.960050	48.798580	903.811700	83.889540
-59.463460	48.703410	944.884600	96.915070
-57.975440	48.589080	854.815400	102.062600
-56.497490	48.455880	893.666700	127.624000
-55.030970	48.304140	926.505300	130.585800
-53.577210	48.134270	912.983500	122.607100
-52.137430	47.946660	722.046000	84.431140
-50.712720	47.741790	577.286600	46.877190
-49.304080	47.520120	526.604600	21.177550
-47.912350	47.282170	508.662000	8.878386
-46.538250	47.028450	502.692000	3.487316
-45.182340	46.759510	500.694900	1.248915
-43.845020	46.475880	500.087900	.407779
-42.526500	46.178100	499.916100	.121053
-41.226800	45.866700	499.871100	.033104
-39.945740	45.542200	499.861100	.011105
-94.687740	46.646660	587.922200	71.448910
-93.376940	46.972690	630.741000	84.554360
-92.046550	47.284930	667.251400	94.442010
-90.696440	47.582840	665.523100	86.668800
-89.326680	47.865840	704.796000	84.381960
-87.937550	48.133390	755.858500	88.420820
-86.529520	48.384910	223.717400	152.436500
-85.103260	48.619850	97.652650	170.141400
-83.659580	48.837660	1255.552000	217.306200
-82.199460	49.037830	1544.110000	185.303900

-80.724000	49.219880	1367.580000	163.430000
-79.234450	49.383340	736.323900	81.551770
-77.732160	49.527770	744.856500	64.652270
-76.218590	49.652830	819.597700	76.968250
-74.695260	49.758160	1225.177000	112.066200
-73.163800	49.843490	1217.125000	159.287300
-71.625870	49.908580	1137.447000	169.046800
-70.083210	49.953250	1151.199000	155.176600
-68.537570	49.977380	1058.219000	134.736800
-66.990740	49.980900	997.232100	98.942600
-65.444490	49.963800	669.149700	76.267500
-63.900620	49.926140	314.320600	104.683000
-62.360880	49.868010	568.133000	54.446210
-60.827010	49.789570	930.293200	106.033800
-59.300730	49.691040	982.769200	110.532900
-57.783640	49.572690	935.702900	112.799500
-56.277320	49.434830	832.302500	120.796200
-54.783270	49.277820	808.892800	121.814000
-53.302860	49.102080	733.878100	99.902010
-51.837360	48.908040	626.009900	69.127390
-50.387950	48.696200	553.368200	36.638540
-48.955640	48.467040	518.790800	17.229880
-47.541310	48.221130	506.576000	7.482895
-46.145660	47.959000	502.019700	2.950930
-44.769230	47.681210	500.505300	1.059620
-43.412380	47.388330	500.037100	.345833
-42.075270	47.080920	499.903300	.102321
-40.757810	46.759540	499.868000	.027703
-39.459720	46.424710	499.860000	.009207
-95.186440	47.531940	628.811000	89.549390
-93.857410	47.868410	682.684000	100.778200
-92.508000	48.190720	714.488600	105.692200
-91.137910	48.498310	722.708700	103.252200
-89.747150	48.790600	784.979200	109.075600
-88.335950	49.067010	869.366100	100.961500
-86.904710	49.326930	885.903700	86.401500
-85.454100	49.569790	1076.818000	153.262600
-83.984930	49.795020	1310.728000	171.493100
-82.498220	50.002060	1374.234000	199.416400
-80.995120	50.190400	1185.537000	156.362700
-79.476920	50.359550	905.215200	116.754300
-77.945060	50.509060	1010.592000	93.058890
-76.401090	50.638520	1121.700000	104.405800
-74.846650	50.747580	1328.122000	147.608500
-73.283450	50.835950	1229.330000	162.062500
-71.713320	50.903350	1143.733000	151.303300
-70.138080	50.949630	1157.759000	137.656300
-68.559640	50.974620	1070.186000	129.471600
-66.979900	50.978270	1114.639000	113.215300
-65.400800	50.960560	908.564800	90.410360
-63.824260	50.921550	322.341900	144.100800
-62.252170	50.861340	217.601400	148.449000
-60.686400	50.780110	644.397900	72.075070
-59.128770	50.678080	872.439500	105.779600
-57.581030	50.555550	845.954200	114.644300
-56.044860	50.412850	774.540700	117.970900
-54.521820	50.250360	671.974900	94.448840
-53.013420	50.068520	604.943300	70.792260
-51.520960	49.867810	560.186500	45.282020
-50.045680	49.648740	524.725200	25.314090
-48.588630	49.411830	510.608800	12.082460
-47.150700	49.157670	503.864700	5.313658
-45.732590	48.886830	501.235100	2.135696
-44.334810	48.599900	500.278700	.770590
-42.957660	48.297460	499.975600	.251193
-41.601190	47.980120	499.887400	.073657
-40.265250	47.648430	499.864100	.019662

-38.949390	47.302930	499.858800	.006430
-95.710510	48.412890	682.848800	104.308500
-94.362470	48.760130	759.184500	112.958600
-92.993230	49.092850	793.062700	110.084300
-91.602360	49.410460	834.495200	103.831000
-90.189750	49.712350	897.891400	99.751830
-88.755540	49.997940	916.691000	98.542910
-87.300090	50.266570	913.677800	100.856100
-85.824040	50.517650	960.371300	131.889800
-84.328210	50.750570	1036.203000	173.245500
-82.813610	50.964740	1050.347000	182.874600
-81.281460	51.159630	1020.803000	163.937100
-79.733130	51.334700	1002.250000	133.103800
-78.170130	51.489480	1016.259000	127.756200
-76.594090	51.623530	1071.109000	151.354700
-75.006800	51.736490	1274.293000	166.008600
-73.410070	51.828010	1182.459000	166.351200
-71.805870	51.897850	1121.596000	151.786600
-70.196170	51.945790	1153.482000	125.896900
-68.582990	51.971680	1072.670000	111.607200
-66.968440	51.975460	1115.143000	107.411400
-65.354560	51.957120	1051.116000	109.693200
-63.743440	51.916690	900.305200	106.938000
-62.137130	51.854320	637.442700	49.487530
-60.537630	51.770170	683.211700	74.993580
-58.946900	51.664510	781.737700	102.836700
-57.366820	51.537620	767.037500	113.723700
-55.799180	51.389870	687.121000	96.258110
-54.245650	51.221680	616.850200	71.084520
-52.707820	51.033510	574.313200	48.592430
-51.187080	50.825860	547.478800	31.676920
-49.684720	50.599280	520.677100	17.382340
-48.201800	50.354330	507.542200	8.293387
-46.739240	50.091630	502.561300	3.601686
-45.297720	49.811770	500.734700	1.417291
-43.877700	49.515370	500.123800	.507206
-42.479410	49.203060	499.931200	.163490
-41.102810	48.875440	499.875700	.047295
-39.747590	48.533100	499.861100	.012360
-38.413150	48.176570	499.858000	.003954
-96.261670	49.289190	743.382000	106.487100
-94.893750	49.647600	861.483200	100.760100
-93.503820	49.991080	918.006100	97.499030
-92.091320	50.319060	899.552500	92.028950
-90.655950	50.630910	841.010900	89.166100
-89.197760	50.925990	789.207000	96.040600
-87.717060	51.203660	765.317600	104.752100
-86.214420	51.463270	778.492700	119.609900
-84.690660	51.704190	831.953100	130.673400
-83.146820	51.925780	857.587500	130.422200
-81.584160	52.127480	1003.712000	133.683500
-80.004110	52.308710	965.411200	126.413800
-78.408280	52.468990	910.291400	139.910100
-76.798410	52.607840	1000.978000	156.590400
-75.176390	52.724850	1199.642000	163.057700
-73.544200	52.819690	1098.064000	155.146500
-71.903920	52.892050	1056.719000	135.130100
-70.257710	52.941730	1100.240000	108.485600
-68.607750	52.968570	1009.309000	73.266010
-66.956280	52.972490	1021.037000	70.969670
-65.305560	52.953480	995.325000	109.063800
-63.657820	52.911580	890.320500	108.189800
-62.015270	52.846950	782.329500	83.405010
-60.380080	52.759750	747.687800	91.211780
-58.754360	52.650280	725.754000	96.913280
-57.140120	52.518850	694.367500	88.836340
-55.539290	52.365840	640.158800	77.834630

-53.953660 52.191710 581.481800 55.181560
-52.384890 51.996940 546.000000 35.555950
-50.834480 51.782070 525.334800 21.597810
-49.303730 51.547700 511.441400 11.361450
-47.793770 51.294400 504.363700 5.296982
-46.305490 51.022820 501.467400 2.259503
-44.839570 50.733620 500.392400 .882729
-43.396390 50.427420 500.019600 .311687
-41.976090 50.104880 499.902200 .099140
-40.578500 49.766630 499.868200 .028241
-39.203150 49.413260 499.859500 .007243
-37.849210 49.045330 499.857600 .002256
-96.841810 50.160530 739.182400 89.339660
-95.453030 50.530490 850.830100 93.112790
-94.041510 50.885130 815.689800 88.258790
-92.606450 51.223850 761.037000 93.624970
-91.147380 51.546020 709.930600 91.167400
-89.664200 51.850960 667.921300 87.616020
-88.157130 52.138020 646.651000 85.177960
-86.626690 52.406500 650.536500 86.147150
-85.073680 52.655720 682.038500 83.107670
-83.499180 52.885050 759.424900 80.130920
-81.904440 53.093850 822.224900 85.115910
-80.290990 53.281520 868.045800 112.692500
-78.660530 53.447530 809.736100 132.653800
-77.014920 53.591380 916.056600 150.314600
-75.356160 53.712650 1115.172000 159.045800
-73.686420 53.810940 983.968800 154.444000
-72.007930 53.885960 906.813700 131.174900
-70.322990 53.937460 943.820800 85.635540
-68.634010 53.965280 867.591800 50.539440
-66.943400 53.969350 826.813000 56.027970
-65.253590 53.949630 840.820600 62.507310
-63.567000 53.906200 840.236700 92.295430
-61.886040 53.839190 810.985500 93.050540
-60.213040 53.748820 711.064500 80.516960
-58.550290 53.635360 638.735700 67.750910
-56.899960 53.499180 611.104500 61.534060
-55.264110 53.340690 598.573200 58.647430
-53.644650 53.160350 556.637100 42.300720
-52.043340 52.958710 527.766800 24.576740
-50.461760 52.736320 513.177200 13.373500
-48.901280 52.493820 505.780500 6.780358
-47.363030 52.231850 502.267800 3.158329
-45.847900 51.951060 500.750700 1.342390
-44.356530 51.652150 500.157500 .517607
-42.889220 51.335790 499.949000 .180669
-41.445980 51.002650 499.882600 .056869
-40.026490 50.653380 499.863300 .016041
-38.630060 50.288590 499.858400 .004046
-37.255620 49.908830 499.857300 .001248
-97.453060 51.026530 639.100400 71.571620
-96.042340 51.408490 667.156300 71.433360
-94.608220 51.774700 673.614700 77.437930
-93.149600 52.124570 668.578900 80.966870
-91.665800 52.457450 649.788800 79.368190
-90.156560 52.772640 621.463400 73.916760
-88.621980 53.069450 599.742000 67.680350
-87.062480 53.347160 587.385300 60.401110
-85.478850 53.605050 576.944800 51.070140
-83.872130 53.842430 542.193700 56.258680
-82.243660 54.058630 492.740600 93.314000
-80.595020 54.253030 625.270400 80.518800
-78.928000 54.425040 703.788900 110.470900
-77.244600 54.574130 867.546700 136.684500
-75.546970 54.699840 1051.882000 149.660400
-73.837420 54.801750 927.999900 144.792800

-72.118360	54.879540	848.255900	114.019900
-70.392330	54.932960	796.625100	77.460520
-68.661900	54.961820	603.270400	124.311500
-66.929700	54.966030	529.173300	186.966200
-65.198370	54.945580	647.738300	76.596610
-63.470540	54.900540	718.021000	82.900150
-61.748820	54.831050	681.127300	74.665340
-60.035740	54.737340	593.173200	51.517390
-58.333780	54.619720	537.130100	30.283670
-56.645260	54.478570	530.356600	19.345070
-54.972420	54.314330	551.506300	28.701390
-53.317280	54.127510	540.690600	27.516550
-51.681740	53.918690	517.800700	15.971280
-50.067420	53.688470	507.285200	7.979373
-48.475770	53.437500	502.940700	3.869623
-46.907930	53.166490	501.096800	1.771933
-45.364770	52.876130	500.315200	.742851
-43.846840	52.567130	500.011800	.283264
-42.354370	52.240220	499.904700	.098091
-40.887210	51.896080	499.870400	.030673
-39.444820	51.535370	499.860200	.008579
-38.026250	51.158730	499.857700	.002140
-36.630120	50.766700	499.857100	.000658
-98.097820	51.886770	565.979100	44.723860
-96.663910	52.281200	572.747000	46.047140
-95.206040	52.659450	575.466900	48.301180
-93.722790	53.020900	578.959500	51.767430
-92.213200	53.364910	587.237200	56.529660
-90.676770	53.690770	601.416300	63.027780
-89.113440	53.997730	604.948200	64.997010
-87.523560	54.285050	587.197400	56.079410
-85.907830	54.551970	554.271300	38.120370
-84.267300	54.797760	495.194600	47.012650
-82.603340	55.021710	365.087800	145.145200
-80.917590	55.223140	324.995200	170.787200
-79.211960	55.401440	568.286900	100.653400
-77.488560	55.556020	841.120000	117.695300
-75.749730	55.686380	1004.775000	138.377300
-73.997920	55.792100	896.648700	135.251800
-72.235800	55.872810	795.548000	95.337270
-70.466090	55.928230	687.508100	70.199330
-68.691570	55.958180	401.059800	191.001500
-66.915130	55.962550	547.214500	125.309400
-65.139640	55.941330	648.130600	71.106640
-63.367970	55.894590	631.304600	60.407460
-61.602940	55.822490	606.644300	55.501400
-59.847310	55.725280	523.645900	37.880510
-58.103760	55.603290	482.252000	32.647390
-56.374820	55.456930	456.520700	35.383810
-54.662870	55.286670	466.231600	24.691350
-52.970100	55.093080	505.520500	6.090563
-51.298500	54.876750	505.550400	5.717477
-49.649800	54.638340	502.932000	3.639765
-48.025460	54.378550	501.228600	1.887317
-46.426660	54.098120	500.423200	.882829
-44.854210	53.797780	500.068700	.371538
-43.308580	53.478290	499.929300	.141743
-41.789850	53.140400	499.879500	.048921
-40.297680	52.784830	499.863300	.015196
-38.831280	52.412250	499.858500	.004213
-37.389370	52.023280	499.857200	.001035
-35.970190	51.618480	499.857000	.000331
-98.778840	52.740810	528.974500	24.646020
-97.320270	53.148240	529.207600	24.609480
-95.837370	53.539010	530.494900	25.742550
-94.328290	53.912530	532.753000	27.962720
-92.791720	54.268120	537.761500	31.680740

-91.226900	54.605080	546.543200	37.204150
-89.633560	54.922620	558.225800	43.499630
-88.011890	55.219970	576.639800	50.434550
-86.362530	55.496330	584.517800	51.914050
-84.686490	55.750910	548.123700	38.956700
-82.985150	55.982970	456.826800	70.456500
-81.260250	56.191770	283.992900	174.695700
-79.513790	56.376650	344.645300	160.109300
-77.748020	56.536990	831.572600	117.383200
-75.965460	56.672260	965.042500	127.549700
-74.168790	56.781970	865.801700	126.344700
-72.360860	56.865740	748.038900	87.617610
-70.544630	56.923280	576.032200	90.604220
-68.723180	56.954370	439.964300	168.843400
-66.899620	56.958910	740.270600	94.522700
-65.077100	56.936880	673.081000	73.578230
-63.258730	56.888360	565.033500	59.436240
-61.447630	56.813510	539.455800	36.085250
-59.646790	56.712620	533.048000	25.345110
-57.859090	56.586040	508.434700	15.239420
-56.087300	56.434210	488.046100	15.983030
-54.333970	56.257640	486.988100	12.364490
-52.601470	56.056940	498.259200	2.708187
-50.891880	55.832740	501.117200	1.661992
-49.207030	55.585770	500.767900	1.280233
-47.548420	55.316760	500.350900	.759675
-45.917200	55.026500	500.077200	.378187
-44.314130	54.715760	499.943100	.164231
-42.739580	54.385350	499.887100	.063369
-41.193420	54.036030	499.866300	.021895
-39.675070	53.668560	499.859500	.006762
-38.183400	53.283620	499.857500	.001847
-36.716770	52.881820	499.857000	.000437
-35.272900	52.463700	499.857000	.000172
-99.499240	53.588130	511.582700	12.168170
-98.014280	54.009130	511.135900	11.858600
-96.504860	54.412960	511.532100	12.296590
-94.968610	54.799050	512.516800	13.425230
-93.403790	55.166730	514.678500	15.424500
-91.809290	55.515250	518.600900	18.519850
-90.184560	55.843850	524.725100	22.685790
-88.529620	56.151670	534.011500	27.946690
-86.844990	56.437890	543.351100	32.931050
-85.131650	56.701690	555.006700	38.537680
-83.390960	56.942230	548.874100	48.777430
-81.624720	57.158780	451.391500	76.393390
-79.835050	57.350580	373.540600	140.271200
-78.024370	57.516980	797.069700	110.786100
-76.195360	57.657400	928.449400	123.425600
-74.350940	57.771310	840.162400	118.519700
-72.494220	57.858320	707.155900	94.776450
-70.628410	57.918090	572.394400	106.221600
-68.756890	57.950390	628.234100	86.877140
-66.883060	57.955100	747.594200	83.596540
-65.010370	57.932220	680.993200	77.866940
-63.142240	57.881810	598.904500	56.635300
-61.282040	57.804080	509.325800	28.281950
-59.433060	57.699310	517.542100	15.635620
-57.598450	57.567890	514.452200	10.536460
-55.781200	57.410300	501.605500	5.203433
-53.984060	57.227100	497.366800	3.820071
-52.209560	57.018940	499.311400	1.313644
-50.459930	56.786500	499.957900	.509154
-48.737060	56.530560	500.072500	.379308
-47.042490	56.251900	500.005100	.260901
-45.377330	55.951360	499.931200	.140690
-43.742270	55.629770	499.887500	.063453

-42.137470 55.287960 499.867900 .025039
-40.562610 54.926740 499.860400 .008686
-39.016750 54.546860 499.857900 .002659
-37.498410 54.149020 499.857100 .000703
-36.005400 53.733830 499.857000 .000186
-34.534880 53.301770 499.857000 .000148
-100.262700 54.428120 504.172800 5.415831
-98.749250 54.863350 503.863400 5.143356
-97.211560 55.280850 503.991200 5.311999
-95.646590 55.680060 504.454300 5.876017
-94.052090 56.060360 505.406100 6.894296
-92.426510 56.420970 507.012100 8.396081
-90.768940 56.761100 509.440900 10.306790
-89.079190 57.079890 512.916100 12.500390
-87.357570 57.376450 516.992600 14.844030
-85.605000 57.649890 522.042700 18.867300
-83.822840 57.899360 520.289600 27.681340
-82.012920 58.124030 474.908100 40.236270
-80.177480 58.323120 513.033400 56.609910
-78.319110 58.495910 798.685100 110.680000
-76.440710 58.641750 853.164900 108.104800
-74.545430 58.760110 820.412400 113.531500
-72.636660 58.850530 669.013300 115.807800
-70.717930 58.912650 694.218400 78.229520
-68.792920 58.946230 704.197000 83.399650
-66.865380 58.951130 749.722300 93.471830
-64.939080 58.927340 675.841500 77.974510
-63.017780 58.874950 583.455000 50.142590
-61.105210 58.794160 529.103000 27.112330
-59.204930 58.685300 515.341200 13.922930
-57.320400 58.548780 514.610000 10.015420
-55.454840 58.385130 504.993100 4.380407
-53.611270 58.194950 500.753500 1.633445
-51.792370 57.978940 499.972000 .700504
-50.000500 57.737840 499.919700 .279905
-48.237630 57.472490 499.920200 .141692
-46.505310 57.183730 499.901400 .087285
-44.804610 56.872430 499.879700 .047370
-43.136050 56.539500 499.866700 .022056
-41.499610 56.185780 499.860500 .008805
-39.894630 55.812120 499.858000 .003060
-38.319800 55.419290 499.857200 .000908
-36.773090 55.007980 499.857000 .000226
-35.251730 54.578750 499.857000 .000150
-33.752120 54.132060 499.857000 .000153
-101.073700 55.260130 501.328100 2.181465
-99.529010 55.710320 501.182500 2.021701
-97.960900 56.142140 501.215500 2.076570
-96.365430 56.555100 501.402600 2.332737
-94.739640 56.948590 501.787700 2.799430
-93.081440 57.321860 502.432100 3.460437
-91.389490 57.674070 503.360600 4.212250
-89.663250 58.004340 504.447800 4.926445
-87.902820 58.311730 505.264600 6.292262
-86.109000 58.595320 504.911500 12.310220
-84.283100 58.854170 496.505400 28.152010
-82.427000 59.087400 448.885000 48.459880
-80.543050 59.294160 528.008000 40.143410
-78.634010 59.473690 775.484700 93.389900
-76.703000 59.625280 723.301600 72.245320
-74.753460 59.748340 704.531400 110.308000
-72.789070 59.842370 716.066300 82.449570
-70.813740 59.906970 691.911700 70.717480
-68.831480 59.941910 763.870600 89.383290
-66.846440 59.947010 754.815800 94.981120
-64.862770 59.922250 660.947000 74.291370
-62.884610 59.867760 570.720500 45.962020

-60.916040 59.783740 525.870500 23.795870
-58.961010 59.670550 514.369600 13.056760
-57.023270 59.528640 513.013400 8.733376
-55.106350 59.358580 505.023600 3.989761
-53.213500 59.161040 501.388200 1.474926
-51.347600 58.936760 500.269900 .532620
-49.511150 58.686560 499.979400 .204733
-47.706190 58.411320 499.901400 .083275
-45.934240 58.111950 499.875200 .037100
-44.196280 57.789400 499.864800 .017314
-42.492650 57.444580 499.860000 .007542
-40.823030 57.078400 499.858000 .002918
-39.186360 56.691730 499.857200 .000970
-37.580830 56.285340 499.857000 .000284
-36.003750 55.859910 499.857000 .000148
-34.451600 55.415950 499.857000 .000153
-32.919830 54.953830 499.857000 .000153
-101.937200 56.083360 500.325000 .799523
-100.358100 56.549350 500.265700 .720917
-98.756940 56.996240 500.273300 .737089
-97.128790 57.423640 500.338200 .837789
-95.469860 57.830970 500.470200 1.013541
-93.777320 58.217490 500.675800 1.230074
-92.049300 58.582370 500.913100 1.468142
-90.284800 58.924680 500.964000 2.375838
-88.483640 59.243460 500.290600 6.358918
-86.646400 59.537720 496.718800 17.615930
-84.774360 59.806450 479.957000 44.893330
-82.869400 60.048700 412.058600 91.253230
-80.933970 60.263580 517.785900 40.159600
-78.971000 60.450230 720.137800 80.747710
-76.983890 60.607900 475.490600 120.165000
-74.976370 60.735940 574.299600 89.968500
-72.952470 60.833800 539.900300 76.462270
-70.916480 60.901050 658.479700 69.342890
-68.872850 60.937420 785.700300 88.946510
-66.826130 60.942730 753.826400 91.717150
-64.780930 60.916960 633.897900 67.796100
-62.741830 60.860230 554.859000 38.755080
-60.713310 60.772790 518.886700 19.188510
-58.699720 60.654990 507.574400 9.350959
-56.705200 60.507380 506.576300 5.383532
-54.733590 60.330550 502.855900 2.598535
-52.788400 60.125220 500.912900 1.058646
-50.872730 59.892220 500.207600 .410263
-48.989200 59.632420 499.968400 .155182
-47.139900 59.346780 499.891300 .057421
-45.326320 59.036270 499.867700 .021461
-43.549270 58.701890 499.860500 .008113
-41.808860 58.344600 499.858000 .002967
-40.104370 57.965370 499.857200 .000997
-38.434220 57.565060 499.857000 .000301
-36.795950 57.144450 499.857000 .000150
-35.186060 56.704150 499.857000 .000153
-33.600030 56.244660 499.857000 .000153
-32.032190 55.766200 499.857000 .000153
-102.859900 56.896880 499.995300 .266453
-101.242000 57.379640 499.974000 .233110
-99.604390 57.842470 499.974900 .235998
-97.940930 58.285070 499.993600 .267038
-96.246660 58.706940 500.025900 .312960
-94.517820 59.107400 500.050900 .387129
-92.751850 59.485580 499.977900 .861158
-90.947170 59.840560 499.490100 2.777374
-89.103220 60.171320 497.813000 8.167260
-87.220290 60.476800 491.491100 22.600960
-85.299550 60.755960 467.258100 59.256310

-83.342830	61.007760	380.425000	130.860400
-81.352710	61.231220	498.621900	47.364520
-79.332320	61.425420	606.587100	51.887410
-77.285290	61.589540	429.328100	119.023800
-75.215710	61.722870	346.597800	129.820100
-73.128000	61.824790	252.918700	175.148300
-71.026890	61.894870	497.087900	119.741300
-68.917300	61.932760	769.960900	89.839710
-66.804310	61.938290	741.359100	86.024880
-64.692990	61.911450	608.166300	58.584460
-62.588420	61.852340	538.303900	30.776490
-60.495590	61.761240	509.303900	14.153660
-58.419300	61.638580	502.106900	6.246061
-56.364090	61.484900	501.845700	2.897735
-54.334160	61.300890	500.977900	1.365069
-52.333320	61.087320	500.348700	.601111
-50.364880	60.845090	500.048100	.250051
-48.431600	60.575160	499.922600	.097422
-46.535590	60.278550	499.877800	.036039
-44.678250	59.956310	499.863200	.012792
-42.860180	59.609490	499.858700	.004343
-41.081100	59.239120	499.857400	.001366
-39.339830	58.846180	499.857000	.000399
-37.634110	58.431540	499.857000	.000158
-35.960610	57.995940	499.857000	.000153
-34.314830	57.539980	499.857000	.000153
-32.690990	57.064010	499.857000	.000153
-31.082000	56.568130	499.857000	.000153
-103.849600	57.699570	499.894700	.080692
-102.187300	58.200280	499.887600	.067957
-100.509000	58.680030	499.886900	.066778
-98.806840	59.138710	499.889100	.072021
-97.074520	59.575940	499.884900	.096923
-95.307120	59.991050	499.841000	.287062
-93.501110	60.383240	499.640500	1.002100
-91.654170	60.751550	498.910200	3.115930
-89.765210	61.094930	496.528300	8.903848
-87.834170	61.412270	487.826100	24.994350
-85.861950	61.702450	458.711700	65.744930
-83.850420	61.964360	394.483500	120.677800
-81.802150	62.196920	507.983800	40.137970
-79.720490	62.399150	557.594700	47.411860
-77.609360	62.570130	386.318700	103.607800
-75.473210	62.709080	243.853500	136.547600
-73.316960	62.815350	155.479500	149.651800
-71.145790	62.888430	243.302200	182.103800
-68.965200	62.927950	702.286600	104.873000
-66.780790	62.933720	686.800300	74.740090
-64.598260	62.905720	578.258500	46.663300
-62.423240	62.844070	526.759200	23.196780
-60.261290	62.749080	508.010200	10.563590
-58.117710	62.621230	502.051200	4.492285
-55.997520	62.461110	500.775800	1.882147
-53.905320	62.269450	500.288100	.778391
-51.845240	62.047150	500.052200	.317591
-49.820820	61.795150	499.937000	.128378
-47.834930	61.514500	499.886400	.050448
-45.889670	61.206310	499.866700	.018941
-43.986290	60.871700	499.859800	.006692
-42.125070	60.511780	499.857700	.002175
-40.305270	60.127630	499.857100	.000638
-38.525020	59.720250	499.857000	.000182
-36.781200	59.290500	499.857000	.000156
-35.069400	58.839090	499.857000	.000153
-33.383770	58.366500	499.857000	.000153
-31.716990	57.872960	499.857000	.000153
-30.060130	57.358350	499.857000	.000153

-104.916500 58.490070 499.866400 .022081
-103.202300 59.010120 499.864100 .017597
-101.477600 59.507980 499.862900 .016146
-99.732440 59.983750 499.859800 .023639
-97.958720 60.437220 499.844400 .083628
-96.150010 60.867820 499.781200 .302744
-94.301540 61.274790 499.558500 .970172
-92.410060 61.657170 498.828400 2.830835
-90.473700 62.013880 496.408500 7.772830
-88.491920 62.343760 488.176700 20.690370
-86.465330 62.645610 467.991600 46.873620
-84.395630 62.918240 454.986500 62.610680
-82.285480 63.160470 566.698400 38.505660
-80.138370 63.371240 527.250800 29.935960
-77.958560 63.549540 415.270700 67.109680
-75.750930 63.694510 305.938100 115.625800
-73.520860 63.805420 226.268800 135.062100
-71.274150 63.881700 281.256900 102.911200
-69.016910 63.922960 524.075000 71.577220
-66.755400 63.928990 577.455600 50.201530
-64.495980 63.899750 539.045900 29.396960
-62.244980 63.835380 515.749200 14.919120
-60.008560 63.736250 505.549100 6.989929
-57.792640 63.602840 501.642400 2.996562
-55.602750 63.435840 500.409800 1.193057
-53.443960 63.236060 500.045500 .447286
-51.320750 63.004450 499.929700 .162919
-49.236890 62.742080 499.885700 .059772
-47.195330 62.450080 499.867700 .022335
-45.198120 62.129630 499.860700 .008284
-43.246250 61.781950 499.858000 .002905
-41.339580 61.408210 499.857100 .000921
-39.476710 61.009530 499.857000 .000260
-37.654880 60.586900 499.857000 .000150
-35.869840 60.141170 499.857000 .000156
-34.115770 59.672970 499.857000 .000153
-32.385130 59.182660 499.857000 .000153
-30.668580 58.670220 499.857000 .000153
-28.954870 58.135290 499.857000 .000153
-106.073800 59.266680 499.859000 .005383
-104.297500 59.807790 499.858200 .003934
-102.518800 60.325180 499.857000 .005403
-100.725000 60.819250 499.852900 .020834
-98.905530 61.289990 499.836500 .078131
-97.052100 61.737010 499.778700 .258789
-95.158340 62.159620 499.590600 .776532
-93.219700 62.556880 499.011500 2.139253
-91.233350 62.927700 497.054800 5.580325
-89.198040 63.270870 491.984800 13.472130
-87.113920 63.585110 480.073400 27.390130
-84.982480 63.869130 514.922500 22.600160
-82.806390 64.121680 532.151600 22.336290
-80.589290 64.341560 467.068800 27.777060
-78.335770 64.527670 392.178600 68.097570
-76.051160 64.679080 342.483600 108.406300
-73.741460 64.794950 333.938900 105.811100
-71.413090 64.874680 392.481300 60.622210
-69.072890 64.917820 484.193100 27.365770
-66.727910 64.924110 516.720900 20.314110
-64.385280 64.893550 513.633000 13.287690
-62.052090 64.826280 506.974800 7.777393
-59.735260 64.722690 502.830600 3.955192
-57.441380 64.583340 500.871500 1.735499
-55.176610 64.408980 500.164900 .681529
-52.946510 64.200520 499.948400 .244883
-50.755950 63.959000 499.885600 .082076
-48.608900 63.685590 499.866500 .026542

-46.508400	63.381530	499.860200	.008769
-44.456330	63.048090	499.858000	.003010
-42.453320	62.686580	499.857200	.001026
-40.498620	62.298210	499.857000	.000306
-38.589950	61.884150	499.857000	.000155
-36.723370	61.445380	499.857000	.000156
-34.893150	60.982680	499.857000	.000153
-33.091610	60.496560	499.857000	.000153
-31.309040	59.987160	499.857000	.000153
-29.533510	59.454220	499.857000	.000153
-27.750760	58.896990	499.857000	.000153
-107.339200	60.027230	499.857300	.001160
-105.487000	60.591610	499.856900	.001239
-103.643800	61.130270	499.855800	.004884
-101.793500	61.644070	499.851800	.018924
-99.922550	62.133320	499.837600	.064564
-98.020040	62.597820	499.791000	.198174
-96.077510	63.037030	499.652400	.549871
-94.088710	63.450070	499.272900	1.374039
-92.049460	63.835860	498.269800	3.121711
-89.957580	64.193130	495.544100	6.305501
-87.812570	64.520550	492.381400	8.621195
-85.615560	64.816710	501.401200	10.665480
-83.369120	65.080260	482.663500	19.724220
-81.077050	65.309890	459.401600	26.491390
-78.744270	65.504390	444.093900	40.048110
-76.376630	65.662700	433.383300	52.474910
-73.980770	65.783920	435.027700	51.575470
-71.563890	65.867360	455.990500	34.782450
-69.133670	65.912500	486.119900	15.905390
-66.698060	65.919100	500.919100	7.964332
-64.265090	65.887100	503.552700	5.536323
-61.842790	65.816700	502.498500	3.663797
-59.438900	65.708320	501.137000	2.004478
-57.060850	65.562580	500.349500	.929802
-54.715470	65.380330	500.018700	.376468
-52.408880	65.162570	499.904300	.134572
-50.146370	64.910470	499.869700	.042814
-47.932120	64.625290	499.860300	.012391
-45.769150	64.308400	499.857700	.003395
-43.659080	63.961160	499.857300	.000978
-41.602020	63.584970	499.857000	.000291
-39.596350	63.181100	499.857000	.000154
-37.638600	62.750720	499.857000	.000156
-35.723270	62.294770	499.857000	.000153
-33.842640	61.813940	499.857000	.000153
-31.986660	61.308540	499.857000	.000153
-30.142740	60.778430	499.857000	.000153
-28.295590	60.222970	499.857000	.000153
-26.427080	59.640870	499.857000	.000153
-108.736700	60.768890	499.856900	.000500
-106.789100	61.359350	499.856600	.001390
-104.866900	61.921510	499.855500	.005458
-102.949500	62.456830	499.851100	.019293
-101.019200	62.966030	499.837100	.060843
-99.061880	63.449270	499.794300	.172397
-97.066180	63.906190	499.674900	.439867
-95.023590	64.336020	499.379100	.981457
-92.928180	64.737730	498.705000	1.879014
-90.776390	65.110020	497.337500	3.040034
-88.566850	65.451470	495.319600	3.412929
-86.300130	65.760610	495.092500	6.793096
-83.978550	66.035940	485.450200	13.477730
-81.606060	66.276030	474.943800	19.199900
-79.187900	66.479550	469.220700	25.385420
-76.730480	66.645290	466.358400	30.402140
-74.241170	66.772270	468.325200	30.307820

-71.728080 66.859700 477.728000 22.523050
-69.199870 66.907030 489.869600 12.540940
-66.665550 66.913940 497.782500 6.012237
-64.134220 66.880400 500.646500 3.329032
-61.614990 66.806620 500.910300 2.133454
-59.116610 66.693070 500.497000 1.240432
-56.647430 66.540450 500.146200 .619535
-54.215080 66.349720 499.964000 .265592
-51.826330 66.121990 499.890300 .098195
-49.486860 65.858530 499.865800 .031590
-47.201050 65.560780 499.858900 .008880
-44.971820 65.230190 499.857200 .002190
-42.800360 64.868270 499.857000 .000504
-40.685980 64.476460 499.857000 .000173
-38.625890 64.056100 499.857000 .000150
-36.614990 63.608330 499.857000 .000153
-34.645670 63.134020 499.857000 .000153
-32.707610 62.633670 499.857000 .000153
-30.787590 62.107330 499.857000 .000153
-28.869240 61.554470 499.857000 .000153
-26.932890 60.973880 499.857000 .000153
-24.955330 60.363580 499.857000 .000153

Q.24a
-89.453400 32.991660 527.011700 .884018
-88.345930 33.191160 527.083700 .995323
-87.228100 33.381660 527.384900 1.460088
-86.100230 33.562890 527.904800 2.237650
-84.962690 33.734530 528.637400 3.275593
-83.815900 33.896310 529.529200 4.469758
-82.660300 34.047950 530.451000 5.644051
-81.496410 34.189190 531.188000 6.555579
-80.324730 34.319790 531.496200 6.919767
-79.145840 34.439500 531.206300 6.477867
-77.960310 34.548100 530.231400 5.146282
-76.768760 34.645400 528.628200 3.238469
-75.571840 34.731200 526.895600 1.650982
-74.370180 34.805370 525.984100 1.141062
-73.164480 34.867740 526.152300 .856022
-71.955430 34.918200 526.747800 .719469
-70.743730 34.956650 527.187100 .967288
-69.530080 34.983020 527.307300 1.118073
-68.315220 34.997260 527.217300 1.027960
-67.099880 34.999340 527.039600 .785091
-65.884770 34.989250 526.856500 .509993

-64.670640	34.967020	526.721100	.288952
-63.458190	34.932690	526.644300	.150436
-62.248150	34.886320	526.610300	.080902
-61.041230	34.828000	526.598100	.052455
-59.838120	34.757850	526.594100	.041845
-58.639500	34.676010	526.591300	.035554
-57.446040	34.582610	526.588000	.028972
-56.258390	34.477850	526.584200	.021710
-55.077160	34.361910	526.580100	.014521
-53.902950	34.235010	526.576400	.008442
-52.736340	34.097380	526.573500	.004235
-51.577860	33.949260	526.572000	.001843
-50.428010	33.790910	526.571200	.000722
-49.287250	33.622600	526.571000	.000282
-48.156040	33.444620	526.570900	.000216
-47.034740	33.257250	526.570900	.000186
-45.923690	33.060790	526.570900	.000183
-44.823220	32.855560	526.570900	.000183
-89.701210	33.918400	527.537500	1.673899
-88.583520	34.125020	527.756200	1.947602
-87.455040	34.322360	528.490800	2.857485
-86.316120	34.510110	529.730800	4.331975
-85.167100	34.687960	531.479800	6.280380
-84.008420	34.855620	533.630300	8.517124
-82.840550	35.012790	535.881600	10.733090
-81.663990	35.159200	537.729700	12.501240
-80.479300	35.294580	538.564800	13.279340
-79.287040	35.418700	537.971000	12.531180
-78.087840	35.531310	535.700600	9.984033
-76.882320	35.632210	531.705100	6.162698
-75.671150	35.721200	527.054700	2.921115
-74.455030	35.798110	524.915400	1.853249
-73.234660	35.862810	525.604400	1.448526
-72.010770	35.915150	527.321700	1.621917
-70.784100	35.955040	528.391000	2.277711
-69.555380	35.982390	528.587100	2.515302
-68.325390	35.997170	528.269000	2.242474
-67.094890	35.999320	527.769300	1.683112
-65.864640	35.988860	527.279700	1.076781
-64.635410	35.965800	526.930200	.602148
-63.407950	35.930180	526.744400	.315258
-62.183030	35.882080	526.668300	.177065
-60.961370	35.821590	526.643900	.123083
-59.743720	35.748840	526.635500	.102142
-58.530780	35.663950	526.628000	.087035
-57.323260	35.567100	526.618200	.069663
-56.121820	35.458470	526.607300	.051184
-54.927120	35.338260	526.595100	.033134
-53.739770	35.206700	526.584200	.018424
-52.560380	35.064020	526.576900	.008775
-51.389480	34.910500	526.573400	.003574
-50.227610	34.746390	526.571700	.001301
-49.075240	34.571990	526.571100	.000435
-47.932830	34.387580	526.570900	.000244
-46.800740	34.193490	526.570900	.000193
-45.679330	33.990000	526.570800	.000183
-44.568900	33.777440	526.570900	.000183
-89.960660	34.843320	529.359200	4.006413
-88.832330	35.057210	530.133200	4.756771
-87.692770	35.261540	532.417500	6.891846
-86.542320	35.455950	536.160300	10.233840
-85.381340	35.640140	541.447300	14.615290
-84.210270	35.813810	547.945700	19.625200
-83.029580	35.976620	554.805100	24.625320
-81.839790	36.128310	560.607800	28.746440
-80.641480	36.268600	563.403300	30.739470
-79.435230	36.397220	561.931800	29.240480

-78.221690	36.513940	555.344200	23.415910
-77.001530	36.618520	542.721900	14.088740
-75.775430	36.710780	526.929900	6.239876
-74.544130	36.790520	520.954200	4.293635
-73.308360	36.857590	524.290800	3.618348
-72.068890	36.911860	529.948100	5.036993
-70.826490	36.953220	533.137300	6.596225
-69.581950	36.981590	533.419000	6.891427
-68.336070	36.996900	532.212300	6.021187
-67.089650	36.999130	530.496800	4.482624
-65.843500	36.988290	528.827100	2.839660
-64.598400	36.964370	527.683400	1.584410
-63.355190	36.927440	527.106600	.846794
-62.114630	36.877570	526.891300	.504369
-60.877510	36.814860	526.828700	.373321
-59.644590	36.739430	526.804700	.318085
-58.416640	36.651430	526.775400	.269225
-57.194370	36.551040	526.736900	.211632
-55.978490	36.438440	526.697800	.152249
-54.769680	36.313860	526.652600	.095816
-53.568590	36.177530	526.612900	.050363
-52.375820	36.029710	526.588700	.022589
-51.191970	35.870660	526.577300	.008779
-50.017550	35.700660	526.572900	.002952
-48.853080	35.520030	526.571400	.000906
-47.698980	35.329070	526.571000	.000281
-46.555660	35.128100	526.570900	.000211
-45.423470	34.917440	526.570800	.000183
-44.302690	34.697410	526.570900	.000183
-90.232250	35.766320	534.141500	8.819725
-89.092860	35.987640	536.639700	10.616230
-87.941790	36.199090	543.269300	15.133940
-86.779330	36.400320	553.777000	21.975580
-85.605870	36.591000	568.705600	30.957650
-84.421870	36.770790	586.997400	41.263730
-83.227800	36.939400	606.420800	51.680950
-82.024180	37.096500	623.726500	60.662900
-80.811620	37.241800	632.548100	65.547710
-79.590740	37.375040	629.248100	63.114450
-78.362190	37.495960	611.544900	50.760380
-77.126670	37.604320	573.730500	29.549790
-75.884930	37.699920	521.890400	13.769990
-74.637700	37.782550	506.903600	9.884612
-73.385770	37.852060	522.912000	10.818060
-72.129940	37.908310	541.009200	14.749070
-70.871030	37.951180	548.814500	17.498970
-69.609860	37.980580	548.263700	17.476740
-68.347290	37.996460	544.139800	15.022490
-67.084140	37.998780	538.727700	11.121680
-65.821270	37.987530	533.296900	6.959407
-64.559530	37.962740	529.756200	3.849170
-63.299760	37.924460	528.119200	2.117595
-62.042780	37.872770	527.567600	1.350947
-60.789440	37.807780	527.431400	1.069628
-59.540510	37.729610	527.367100	.933673
-58.296810	37.638420	527.258300	.784702
-57.059080	37.534390	527.117400	.603266
-55.828080	37.417740	526.994400	.429321
-54.604500	37.288680	526.834500	.258979
-53.389020	37.147460	526.693000	.126769
-52.182290	36.994370	526.617700	.051973
-50.984880	36.829660	526.586400	.018594
-49.797380	36.653650	526.575400	.005873
-48.620270	36.466650	526.571900	.001620
-47.454020	36.268990	526.571100	.000414
-46.299010	36.060990	526.570900	.000215
-45.155610	35.843010	526.570800	.000183

-44.024070	35.615370	526.570900	.000180
-90.516530	36.687310	545.055100	17.066260
-89.365660	36.916220	552.224800	20.749830
-88.202590	37.134950	569.719400	29.026060
-87.027650	37.343150	595.758400	40.852080
-85.841200	37.540460	633.925400	56.692120
-84.643710	37.726540	680.706200	75.635700
-83.435650	37.901050	730.602400	94.931340
-82.217600	38.063690	778.152500	112.384200
-80.990140	38.214140	803.705700	123.581600
-79.753940	38.352110	797.871600	121.668700
-78.509670	38.477340	757.219100	99.250050
-77.258070	38.589580	656.250800	56.621310
-75.999910	38.688600	498.238100	33.595720
-74.735980	38.774210	457.042300	25.086810
-73.467090	38.846230	525.544600	28.750710
-72.194090	38.904510	581.194400	36.273300
-70.917830	38.948930	596.947800	40.861870
-69.639210	38.979390	589.947600	39.345110
-68.359090	38.995840	577.038600	33.513400
-67.078360	38.998240	561.538500	24.750570
-65.797930	38.986590	544.630200	15.109030
-64.518690	38.960900	534.643100	8.302952
-63.241520	38.921240	530.575100	4.753373
-61.967320	38.867690	529.455700	3.327714
-60.696920	38.800350	529.266400	2.837722
-59.431210	38.719360	529.127500	2.549760
-58.170970	38.624900	528.747600	2.125339
-56.917050	38.517150	528.255200	1.593086
-55.670200	38.396340	527.868800	1.043737
-54.431160	38.262690	527.349100	.605875
-53.200640	38.116470	526.890300	.277590
-51.979310	37.957960	526.672800	.098734
-50.767770	37.787460	526.600000	.030605
-49.566610	37.605300	526.578300	.008341
-48.376340	37.411790	526.572600	.002006
-47.197410	37.207270	526.571100	.000387
-46.030250	36.992090	526.570800	.000195
-44.875180	36.766620	526.570800	.000178
-43.732470	36.531200	526.570900	.000229
-90.814130	37.606180	566.234600	28.741370
-89.651300	37.842850	584.975300	35.310510
-88.475770	38.069030	626.718000	47.826020
-87.287830	38.284350	682.998200	64.348280
-86.087840	38.488450	768.746500	86.742740
-84.876290	38.680970	877.055100	116.903900
-83.653650	38.861550	993.013200	149.513000
-82.420500	39.029860	1109.165000	180.887300
-81.177470	39.185590	1176.115000	204.526600
-79.925230	39.328410	1173.865000	208.276900
-78.664510	39.458070	1103.971000	177.532400
-77.396060	39.574290	891.329300	103.356100
-76.120680	39.676830	512.699600	85.069950
-74.839220	39.765490	333.471900	41.287180
-73.552520	39.840080	539.876900	62.327620
-72.261480	39.900440	686.997600	73.770270
-70.967010	39.946460	718.293300	78.401870
-69.670030	39.978020	691.642800	75.653970
-68.371470	39.995060	658.241600	65.143600
-67.072270	39.997550	617.574500	47.689560
-65.773400	39.985470	569.057900	28.525810
-64.475770	39.958870	543.846900	15.385430
-63.180340	39.917780	535.997000	9.637441
-61.888020	39.862300	534.572800	7.573701
-60.599740	39.792560	534.644400	6.853927
-59.316390	39.708680	534.320300	6.150363
-58.038840	39.610860	533.013500	5.013091

-56.767930	39.499290	531.250200	3.505004
-55.504470	39.374200	529.926700	2.274687
-54.249260	39.235840	528.415200	1.160782
-53.003000	39.084480	527.215300	.423031
-51.766410	38.920440	526.738200	.135120
-50.540120	38.744010	526.604600	.030457
-49.324710	38.555530	526.576700	.005585
-48.120720	38.355340	526.571500	.000810
-46.928620	38.143810	526.570700	.000397
-45.748810	37.921300	526.570700	.000219
-44.581620	37.688170	526.570800	.000161
-43.427300	37.444810	526.570900	.000205
-91.125630	38.522790	600.035500	41.638330
-89.950410	38.767390	643.653000	51.452040
-88.761920	39.001200	734.564300	68.760060
-87.560450	39.223820	830.480700	79.541180
-86.346370	39.434880	981.400400	101.803800
-85.120160	39.633990	1198.489000	148.391100
-83.882300	39.820790	1433.456000	199.993000
-82.633390	39.994930	1664.463000	255.299200
-81.374080	40.156070	1814.755000	300.032300
-80.105060	40.303890	1850.443000	319.503500
-78.827100	40.438090	1785.786000	297.593400
-77.540980	40.558400	1488.835000	211.969900
-76.247540	40.664560	831.247100	98.048420
-74.947690	40.756360	349.911000	212.491000
-73.642300	40.833600	588.875900	108.016000
-72.332310	40.896110	813.733600	126.165600
-71.018690	40.943760	899.719400	118.305000
-69.702430	40.976440	864.150400	118.534800
-68.384490	40.994090	805.060900	105.331800
-67.065880	40.996670	716.422900	78.777680
-65.747600	40.984170	601.290500	42.012750
-64.430650	40.956610	555.415900	24.450800
-63.116020	40.914060	546.877300	17.102930
-61.804690	40.856610	547.292500	15.101150
-60.497620	40.784390	547.329800	13.835700
-59.195770	40.697540	545.385100	12.270940
-57.900040	40.596260	541.330700	9.713687
-56.611330	40.480750	536.407500	6.267536
-55.330490	40.351270	531.697800	3.186620
-54.058330	40.208070	528.635200	1.425607
-52.795620	40.051450	527.114600	.364721
-51.543080	39.881710	526.627700	.067732
-50.301390	39.699200	526.560500	.024531
-49.071130	39.504250	526.561000	.014278
-47.852850	39.297230	526.567000	.006060
-46.647030	39.078520	526.569500	.002191
-45.454070	38.848490	526.570500	.000702
-44.274280	38.607530	526.570700	.000250
-43.107920	38.356030	526.570800	.000255
-91.451740	39.437030	638.298100	50.028800
-90.263630	39.689750	721.850700	64.269870
-89.061680	39.931370	879.557900	85.442040
-87.846150	40.161480	995.508500	85.441860
-86.617400	40.379670	1124.458000	89.329250
-85.375900	40.585540	1440.166000	151.826700
-84.122160	40.778730	1853.076000	234.641300
-82.856800	40.958850	2219.905000	322.424400
-81.580460	41.125550	2494.868000	396.124700
-80.293880	41.278500	2646.266000	440.782500
-78.997860	41.417370	2642.504000	439.098600
-77.693230	41.541890	2354.541000	368.263300
-76.380870	41.651780	1631.576000	192.852000
-75.061690	41.746810	909.265000	106.839200
-73.736660	41.826770	746.298800	92.196360
-72.406780	41.891490	720.236600	61.757940

-71.073040	41.940830	939.804500	112.249100
-69.736500	41.974670	978.568700	145.545800
-68.398190	41.992950	927.039900	145.179700
-67.059160	41.995620	775.211900	110.723400
-65.720480	41.982670	603.232800	53.699130
-64.383220	41.954140	558.451800	31.859430
-63.048400	41.910080	565.055300	26.213800
-61.717090	41.850600	566.163800	23.588050
-60.390290	41.775820	557.936300	20.981960
-59.069010	41.685920	547.299700	19.240870
-57.754210	41.581080	539.539400	14.606980
-56.446830	41.461530	533.853700	6.111061
-55.147770	41.327530	527.312000	1.524490
-53.857880	41.179360	524.509200	1.799249
-52.577970	41.017320	525.174900	.885074
-51.308780	40.841740	526.069900	.341258
-50.051000	40.652980	526.421600	.135418
-48.805250	40.451390	526.530200	.045610
-47.572090	40.237360	526.559800	.014612
-46.352010	40.011290	526.567900	.004429
-45.145370	39.773560	526.570100	.001300
-43.952510	39.524580	526.570600	.000398
-42.773630	39.264750	526.570800	.000252
-91.793200	40.348750	648.224700	45.790540
-90.591710	40.609810	749.338800	61.197810
-89.375760	40.859430	929.519000	104.030800
-88.145600	41.097210	979.889900	112.049800
-86.901570	41.322720	926.214600	119.351300
-85.644150	41.535550	1237.420000	92.447340
-84.373860	41.735290	1853.825000	189.260600
-83.091290	41.921560	2405.776000	340.057200
-81.797160	42.093980	2854.302000	453.231700
-80.492230	42.252200	3198.237000	530.880200
-79.177290	42.395880	3304.149000	552.029200
-77.853240	42.524730	3028.564000	479.835100
-76.521000	42.638450	2315.132000	300.553400
-75.181550	42.736820	1467.791000	115.758100
-73.835900	42.819590	867.172400	70.717770
-72.485100	42.886590	496.669800	152.688300
-71.130210	42.937670	826.108900	140.689900
-69.772340	42.972710	995.837300	161.787600
-68.412590	42.991630	952.086800	155.001800
-67.052090	42.994400	748.223100	113.614100
-65.691960	42.980990	591.241400	81.322390
-64.333320	42.951450	578.895200	41.862030
-62.977290	42.905830	581.287100	35.505770
-61.624970	42.844260	558.626500	28.065770
-60.277440	42.766850	526.087800	18.801820
-58.935750	42.673790	505.567300	20.315360
-57.600940	42.565280	506.586200	22.350430
-56.273990	42.441570	515.249400	7.285536
-54.955840	42.302930	518.572300	6.250870
-53.647390	42.149640	522.043800	3.454689
-52.349470	41.982030	524.805100	1.450357
-51.062890	41.800450	526.014100	.542778
-49.788320	41.605260	526.414000	.181873
-48.526440	41.396860	526.528300	.059126
-47.277780	41.175640	526.559800	.018617
-46.042850	40.942000	526.568100	.005488
-44.822010	40.696380	526.570100	.001517
-43.615560	40.439180	526.570600	.000425
-42.423690	40.170830	526.570800	.000260
-92.150770	41.257820	598.707300	25.172570
-90.935390	41.527410	638.238000	27.850170
-89.704910	41.785250	702.389500	35.356170
-88.459540	42.030900	682.607600	53.647380
-87.199620	42.263930	621.764200	160.109600

-85.925610	42.483890	995.535500	98.908810
-84.638040	42.690380	1765.768000	193.372500
-83.337520	42.882970	2476.151000	375.326700
-82.024790	43.061280	3086.324000	515.840100
-80.700610	43.224930	3594.414000	615.121800
-79.365870	43.373580	3737.138000	630.657700
-78.021450	43.506890	3408.205000	544.016000
-76.668370	43.624570	2615.513000	352.127600
-75.307630	43.726360	1687.853000	140.701700
-73.940300	43.812040	934.029900	104.330000
-72.567500	43.881390	348.746400	149.045700
-71.190370	43.934270	739.392900	125.886000
-69.810050	43.970550	1168.891000	187.446300
-68.427750	43.990140	1261.174000	203.033000
-67.044650	43.993000	984.399200	145.073800
-65.661930	43.979120	783.769300	89.074080
-64.280810	43.948540	688.707800	57.984290
-62.902460	43.901310	583.413800	23.581930
-61.528050	43.837570	513.307700	28.270200
-60.158730	43.757450	490.314000	32.621520
-58.795620	43.661140	506.687100	20.261360
-57.439790	43.548850	528.833800	15.426190
-56.092310	43.420850	531.522600	11.500590
-54.754150	43.277410	523.587800	8.342788
-53.426270	43.118850	523.931300	4.122743
-52.109530	42.945500	525.669100	1.704932
-50.804760	42.757750	526.377700	.676198
-49.512690	42.555960	526.544600	.273424
-48.233980	42.340550	526.572800	.105018
-46.969190	42.111940	526.574000	.036206
-45.718810	41.870550	526.572100	.011081
-44.483210	41.616830	526.571200	.002958
-43.262650	41.351200	526.570800	.000698
-42.057300	41.074100	526.570900	.000238
-92.525320	42.164070	542.522200	7.726737
-91.295520	42.442420	525.458900	14.976100
-90.049940	42.708690	474.503300	43.533310
-88.788760	42.962440	392.571400	85.361290
-87.512310	43.203190	424.341700	70.411950
-86.221000	43.430500	775.593300	73.325610
-84.915410	43.643920	1598.561000	229.149800
-83.596150	43.843030	2430.045000	399.894700
-82.263960	44.027400	3109.114000	535.840800
-80.919660	44.196660	3665.863000	646.858700
-79.564150	44.350410	3796.928000	651.485700
-78.198390	44.488330	3379.289000	531.705300
-76.823410	44.610100	2475.451000	284.584500
-75.440290	44.715440	1515.986000	144.113600
-74.050180	44.804110	803.513700	154.031400
-72.654240	44.875890	319.650300	157.175400
-71.253690	44.930630	770.150000	147.756600
-69.849760	44.968180	1508.545000	252.463100
-68.443720	44.988460	1739.276000	323.969900
-67.036810	44.991420	1324.482000	189.575300
-65.630330	44.977050	992.163300	118.482700
-64.225530	44.945390	799.084900	67.892430
-62.823690	44.896510	625.437000	49.129250
-61.426040	44.830540	545.540400	29.031220
-60.033810	44.747610	548.285200	20.296960
-58.648170	44.647940	569.439400	25.184310
-57.270290	44.531750	572.482600	25.224780
-55.901260	44.399320	550.326500	18.280370
-54.542130	44.250930	531.172400	10.886620
-53.193900	44.086940	527.755600	6.117831
-51.857480	43.907680	527.795800	3.276896
-50.533710	43.713560	527.377600	1.654937
-49.223370	43.504980	526.936100	.749690

-47.927120 43.282370 526.705900 .298886
-46.645540 43.046150 526.614400 .104735
-45.379100 42.796800 526.583300 .032415
-44.128160 42.534760 526.573900 .008855
-42.892960 42.260480 526.571500 .002135
-41.673610 41.974410 526.571000 .000623
-92.917770 43.067330 513.376200 7.472275
-91.673000 43.354680 479.355900 16.360780
-90.411740 43.629630 377.479100 35.428330
-89.134110 43.891690 237.405800 82.400800
-87.840450 44.140390 239.905500 81.575000
-86.531140 44.375260 325.284900 179.620400
-85.206750 44.595830 1160.134000 188.093100
-83.867900 44.801650 2165.203000 357.022700
-82.515380 44.992280 2788.532000 476.443700
-81.150000 45.167310 3113.160000 520.468300
-79.772730 45.326340 3119.611000 491.509100
-78.384560 45.469020 2793.433000 396.673300
-76.986600 45.595010 2133.529000 215.258900
-75.579970 45.704020 1389.370000 76.632630
-74.165890 45.795780 763.919700 206.271200
-72.745610 45.870080 282.144700 172.118900
-71.320400 45.926740 666.147500 150.001800
-69.891600 45.965610 1539.209000 228.873300
-68.460530 45.986610 1796.009000 325.212100
-67.028560 45.989670 1305.948000 172.200800
-65.597030 45.974800 942.820000 90.710360
-64.167300 45.942020 834.150000 74.206620
-62.740720 45.891430 725.757600 51.550850
-61.318600 45.823140 650.429200 31.025090
-59.902260 45.737310 634.206200 32.760280
-58.492950 45.634170 629.313100 38.600060
-57.091900 45.513950 603.556700 35.923150
-55.700260 45.376930 571.242700 26.843200
-54.319140 45.223450 548.908800 19.304650
-52.949590 45.053840 539.559800 12.888230
-51.592580 44.868490 533.813400 7.759994
-50.248970 44.667810 529.862800 3.942084
-48.919560 44.452230 527.841200 1.746918
-47.605040 44.222190 526.994600 .677762
-46.305980 43.978160 526.697500 .233676
-45.022840 43.720610 526.605100 .071739
-43.755960 43.450020 526.579200 .019648
-42.505550 43.166850 526.572600 .004829
-41.271660 42.871580 526.571400 .001450
-93.329110 43.967430 515.199700 5.864004
-92.068780 44.264030 495.251200 14.477210
-90.791210 44.547890 430.387100 54.327690
-89.496510 44.818510 314.370600 116.503500
-88.184940 45.075390 287.470500 125.307900
-86.856880 45.318050 296.025600 119.471700
-85.512890 45.545990 976.623700 139.821100
-84.153590 45.758730 1853.311000 294.553900
-82.779790 45.955820 2243.406000 345.462000
-81.392350 46.136820 2231.749000 313.573700
-79.992260 46.301310 2030.356000 228.927200
-78.580580 46.448910 1752.955000 123.179500
-77.158460 46.579270 1504.581000 74.569760
-75.727110 46.692070 1405.497000 92.822620
-74.287820 46.787040 1236.246000 182.891000
-72.841890 46.863950 874.752500 87.013690
-71.390720 46.922590 934.437300 151.013600
-69.935700 46.962830 1534.568000 285.572700
-68.478260 46.984570 1535.440000 207.665300
-67.019850 46.987740 1025.767000 84.344100
-65.561920 46.972340 734.441200 55.483790
-64.105920 46.938410 743.020300 64.109370

-62.653260	46.886040	758.909300	62.248530
-61.205390	46.815350	712.286000	40.452840
-59.763680	46.726520	690.667500	38.646530
-58.329470	46.619790	684.113300	48.084500
-56.904060	46.495390	657.361700	51.138970
-55.488670	46.353640	628.983800	46.866290
-54.084500	46.194880	605.412000	38.424260
-52.692630	46.019470	574.792700	27.567930
-51.314070	45.827840	548.574600	15.716460
-49.949730	45.620390	534.946100	7.641298
-48.600430	45.397590	529.404100	3.209036
-47.266860	45.159900	527.449800	1.202570
-45.949600	44.907820	526.819600	.403776
-44.649100	44.641840	526.635400	.122077
-43.365670	44.362450	526.586300	.033160
-42.099450	44.070140	526.574200	.008171
-40.850440	43.765410	526.571700	.002429
-93.760410	44.864160	532.847400	14.417180
-92.483920	45.170280	531.242400	20.802670
-91.189420	45.463320	499.229200	39.373990
-89.876960	45.742750	375.114700	62.925610
-88.546760	46.008070	263.218800	122.560800
-87.199170	46.258760	310.785300	119.098700
-85.834720	46.494300	845.134800	74.751620
-84.454070	46.714200	1411.587000	170.623500
-83.058010	46.917960	1614.350000	187.294600
-81.647480	47.105130	1487.060000	141.731800
-80.223460	47.275260	1188.901000	77.154600
-78.787090	47.427960	886.617200	178.019700
-77.339580	47.562840	844.439100	206.626800
-75.882230	47.679570	1134.835000	56.478560
-74.416370	47.777870	1431.034000	152.087300
-72.943440	47.857470	1386.382000	172.655600
-71.464880	47.918190	1331.301000	143.818300
-69.982220	47.959850	1438.650000	169.572000
-68.496960	47.982350	1155.756000	107.510200
-67.010670	47.985630	719.539100	105.167000
-65.524890	47.969690	501.632500	145.919600
-64.041170	47.934570	564.469100	105.747100
-62.561040	47.880340	672.470100	42.407640
-61.086020	47.807170	698.430200	38.065830
-59.617590	47.715230	720.634200	43.032390
-58.157170	47.604770	749.471200	64.709000
-56.706150	47.476050	754.886200	78.825840
-55.265830	47.329400	742.678100	79.048080
-53.837470	47.165180	710.851600	70.868680
-52.422200	46.983780	632.044700	47.081920
-51.021090	46.785640	570.320700	25.317600
-49.635090	46.571200	541.345700	11.390880
-48.265020	46.340950	531.098600	4.601504
-46.911610	46.095370	527.865200	1.664529
-45.575410	45.834990	526.919100	.545553
-44.256860	45.560320	526.658100	.161570
-42.956210	45.271870	526.591200	.043257
-41.673580	44.970160	526.575100	.010535
-40.408870	44.655690	526.572000	.003064
-94.212860	45.757310	559.783500	30.030960
-92.919560	46.073230	572.639500	37.150960
-91.607470	46.375730	573.559200	46.018650
-90.276550	46.664250	520.480300	41.191850
-88.926960	46.938270	481.345800	44.294950
-87.559010	47.197250	550.561600	79.287820
-86.173240	47.440650	721.581100	59.195360
-84.770290	47.667940	915.587200	84.415210
-83.350960	47.878600	1139.994000	96.736600
-81.916200	48.072160	1202.679000	88.287730
-80.467090	48.248140	1051.506000	44.322860

-79.004780	48.406110	798.539900	128.142200
-77.530590	48.545680	810.892600	106.905700
-76.045850	48.666500	1064.320000	52.912510
-74.552020	48.768250	1307.250000	130.366800
-73.050600	48.850660	1334.122000	150.637000
-71.543170	48.913520	1304.131000	147.309900
-70.031330	48.956650	1273.515000	147.833500
-68.516710	48.979950	1054.377000	100.674400
-67.000980	48.983350	778.212300	42.659690
-65.485790	48.966840	630.899200	55.508310
-63.972820	48.930470	648.223400	33.923330
-62.463700	48.874330	708.293900	52.404210
-60.960050	48.798580	715.383400	85.228850
-59.463460	48.703410	736.539900	65.735150
-57.975440	48.589080	785.295800	77.769180
-56.497490	48.455880	820.054900	97.287920
-55.030970	48.304140	827.464500	107.477400
-53.577210	48.134270	774.347600	96.112820
-52.137430	47.946660	654.902500	58.584110
-50.712720	47.741790	574.451100	29.535130
-49.304080	47.520120	541.870800	12.778380
-47.912350	47.282170	531.116900	4.953213
-46.538250	47.028450	527.836600	1.764717
-45.182340	46.759510	526.898300	.569248
-43.845020	46.475880	526.650600	.166103
-42.526500	46.178100	526.588900	.043608
-41.226800	45.866700	526.574600	.010382
-39.945740	45.542200	526.571800	.002951
-94.687740	46.646660	595.725800	49.379190
-93.376940	46.972690	620.935900	57.528380
-92.046550	47.284930	646.896300	66.139550
-90.696440	47.582840	647.025600	62.332170
-89.326680	47.865840	661.052700	50.546370
-87.937550	48.133390	693.915800	35.961620
-86.529520	48.384910	678.374100	84.888050
-85.103260	48.619850	769.324200	126.563200
-83.659580	48.837660	1083.817000	95.737520
-82.199460	49.037830	1285.024000	157.334300
-80.724000	49.219880	1243.814000	159.664200
-79.234450	49.383340	1039.223000	78.922090
-77.732160	49.527770	1002.971000	69.233990
-76.218590	49.652830	1085.137000	96.204920
-74.695260	49.758160	1206.158000	134.661100
-73.163800	49.843490	1198.837000	145.645100
-71.625870	49.908580	1172.817000	144.232900
-70.083210	49.953250	1149.739000	136.608500
-68.537570	49.977380	1011.328000	102.210600
-66.990740	49.980900	809.129000	55.168670
-65.444490	49.963800	679.189800	79.717970
-63.900620	49.926140	663.185400	42.227870
-62.360880	49.868010	730.411700	86.966450
-60.827010	49.789570	806.250700	98.561250
-59.300730	49.691040	843.269800	94.776680
-57.783640	49.572690	846.203100	100.766600
-56.277320	49.434830	839.006500	110.554700
-54.783270	49.277820	804.970900	105.293400
-53.302860	49.102080	723.752400	82.255530
-51.837360	48.908040	608.550400	52.162090
-50.387950	48.696200	550.036900	24.967760
-48.955640	48.467040	533.432900	10.756790
-47.541310	48.221130	528.831200	4.118983
-46.145660	47.959000	527.276400	1.456279
-44.769230	47.681210	526.760600	.468256
-43.412380	47.388330	526.616500	.134868
-42.075270	47.080920	526.580800	.034791
-40.757810	46.759540	526.572800	.008046
-39.459720	46.424710	526.571300	.002215

-95.186440	47.531940	645.682700	71.049590
-93.857410	47.868410	685.645100	80.399140
-92.508000	48.190720	730.756700	89.052510
-91.137910	48.498310	769.086000	92.233300
-89.747150	48.790600	823.105700	93.585290
-88.335950	49.067010	878.494000	92.055510
-86.904710	49.326930	879.339500	74.184200
-85.454100	49.569790	927.533700	71.619090
-83.984930	49.795020	1107.810000	136.314800
-82.498220	50.002060	1196.728000	167.536100
-80.995120	50.190400	1170.506000	160.854200
-79.476920	50.359550	1045.147000	124.638300
-77.945060	50.509060	1039.666000	114.792000
-76.401090	50.638520	1110.641000	124.423300
-74.846650	50.747580	1208.231000	157.441100
-73.283450	50.835950	1177.100000	155.057000
-71.713320	50.903350	1140.142000	146.603900
-70.138080	50.949630	1108.692000	130.094900
-68.559640	50.974620	996.173800	97.319370
-66.979900	50.978270	855.927700	71.799670
-65.400800	50.960560	749.639500	64.985550
-63.824260	50.921550	632.139700	67.679820
-62.252170	50.861340	583.547400	101.480600
-60.686400	50.780110	705.845500	67.392850
-59.128770	50.678080	820.279400	101.354800
-57.581030	50.555550	829.010800	107.163900
-56.044860	50.412850	782.249100	100.005200
-54.521820	50.250360	719.489500	83.674660
-53.013420	50.068520	648.858800	58.529400
-51.520960	49.867810	577.197200	34.825560
-50.045680	49.648740	540.741600	17.781990
-48.588630	49.411830	530.269700	7.514972
-47.150700	49.157670	527.736000	2.945823
-45.732590	48.886830	526.915800	1.036125
-44.334810	48.599900	526.660200	.329771
-42.957660	48.297460	526.591200	.093467
-41.601190	47.980120	526.574800	.023461
-40.265250	47.648430	526.571400	.005256
-38.949390	47.302930	526.570900	.001416
-95.710510	48.412890	717.105100	91.277950
-94.362470	48.760130	782.681300	100.932700
-92.993230	49.092850	850.285500	111.600300
-91.602360	49.410460	898.344700	117.451000
-90.189750	49.712350	931.863100	123.208300
-88.755540	49.997940	937.545100	116.858300
-87.300090	50.266570	910.656800	103.321700
-85.824040	50.517650	895.020300	100.595300
-84.328210	50.750570	918.848000	116.563000
-82.813610	50.964740	943.617700	127.393100
-81.281460	51.159630	976.592000	131.097100
-79.733130	51.334700	947.678300	117.975600
-78.170130	51.489480	964.190200	116.275300
-76.594090	51.623530	1056.064000	132.550300
-75.006800	51.736490	1164.307000	161.096200
-73.410070	51.828010	1138.817000	155.180800
-71.805870	51.897850	1110.228000	142.633500
-70.196170	51.945790	1092.128000	123.154700
-68.582990	51.971680	990.456700	90.418880
-66.968440	51.975460	872.192700	71.050270
-65.354560	51.957120	860.094000	85.417560
-63.743440	51.916690	789.362100	84.184820
-62.137130	51.854320	693.982900	62.271940
-60.537630	51.770170	726.993900	77.674290
-58.946900	51.664510	776.410700	94.374950
-57.366820	51.537620	755.303000	91.534360
-55.799180	51.389870	698.235800	77.758660
-54.245650	51.221680	638.799600	56.869060

-52.707820	51.033510	595.459600	38.188900
-51.187080	50.825860	560.863800	22.463830
-49.684720	50.599280	539.836200	11.374400
-48.201800	50.354330	530.899000	5.094196
-46.739240	50.091630	527.795400	1.982458
-45.297720	49.811770	526.884500	.697424
-43.877700	49.515370	526.643100	.217023
-42.479410	49.203060	526.586000	.059823
-41.102810	48.875440	526.573500	.014629
-39.747590	48.533100	526.571200	.003137
-38.413150	48.176570	526.570800	.000833
-96.261670	49.289190	789.854000	101.017900
-94.893750	49.647600	882.148100	116.212300
-93.503820	49.991080	940.054100	129.333000
-92.091320	50.319060	934.310200	125.217700
-90.655950	50.630910	890.632900	112.962800
-89.197760	50.925990	833.422100	100.752300
-87.717060	51.203660	780.712900	89.010130
-86.214420	51.463270	741.711700	81.165420
-84.690660	51.704190	715.741100	75.635310
-83.146820	51.925780	727.327000	83.334610
-81.584160	52.127480	838.211900	98.405330
-80.004110	52.308710	884.901300	106.927400
-78.408280	52.468990	881.055800	106.870900
-76.798410	52.607840	969.430900	126.357300
-75.176390	52.724850	1091.691000	154.977900
-73.544200	52.819690	1079.108000	147.261600
-71.903920	52.892050	1059.127000	132.065000
-70.257710	52.941730	1052.813000	110.981500
-68.607750	52.968570	952.177700	72.894470
-66.956280	52.972490	819.685500	62.235570
-65.305560	52.953480	833.604100	71.441220
-63.657820	52.911580	847.851800	94.017300
-62.015270	52.846950	795.362100	90.391000
-60.380080	52.759750	757.249400	87.329280
-58.754360	52.650280	729.154600	82.145490
-57.140120	52.518850	687.401600	71.959640
-55.539290	52.365840	640.072400	56.995100
-53.953660	52.191710	597.890500	41.159250
-52.384890	51.996940	567.723500	26.236530
-50.834480	51.782070	547.930500	15.192940
-49.303730	51.547700	535.690900	7.741351
-47.793770	51.294400	529.766200	3.427492
-46.305490	51.022820	527.536600	1.343406
-44.839570	50.733620	526.831800	.463117
-43.396390	50.427420	526.635900	.141941
-41.976090	50.104880	526.585200	.038699
-40.578500	49.766630	526.573500	.009240
-39.203150	49.413260	526.571100	.001901
-37.849210	49.045330	526.570900	.000480
-96.841810	50.160530	781.071500	93.609150
-95.453030	50.530490	849.215900	110.125400
-94.041510	50.885130	856.321200	105.370900
-92.606450	51.223850	811.010800	97.981910
-91.147380	51.546020	752.440500	87.976930
-89.664200	51.850960	698.584600	76.422130
-88.157130	52.138020	655.872300	62.910290
-86.626690	52.406500	618.734800	47.722970
-85.073680	52.655720	577.023800	39.670410
-83.499180	52.885050	555.454300	53.779540
-81.904440	53.093850	646.646800	75.315390
-80.290990	53.281520	755.748700	93.726800
-78.660530	53.447530	791.004200	89.200810
-77.014920	53.591380	890.083400	111.258300
-75.356160	53.712650	1018.578000	143.124100
-73.686420	53.810940	1003.424000	134.586000
-72.007930	53.885960	970.756200	113.959400

-70.322990	53.937460	959.440100	87.749200
-68.634010	53.965280	862.818500	53.400580
-66.943400	53.969350	737.558900	56.561050
-65.253590	53.949630	754.817000	50.959470
-63.567000	53.906200	792.443400	81.398690
-61.886040	53.839190	771.746100	87.775040
-60.213040	53.748820	709.518700	73.208720
-58.550290	53.635360	658.724100	58.865340
-56.899960	53.499180	627.744300	49.018570
-55.264110	53.340690	605.828300	42.184410
-53.644650	53.160350	579.182000	31.571570
-52.043340	52.958710	555.339400	20.116790
-50.461760	52.736320	540.072100	11.025770
-48.901280	52.493820	532.159400	5.407547
-47.363030	52.231850	528.608600	2.361032
-45.847900	51.951060	527.234400	.916369
-44.356530	51.652150	526.766700	.317703
-42.889220	51.335790	526.623300	.098579
-41.445980	51.002650	526.583400	.027209
-40.026490	50.653380	526.573400	.006589
-38.630060	50.288590	526.571300	.001366
-37.255620	49.908830	526.570900	.000390
-97.453060	51.026530	681.022600	67.244620
-96.042340	51.408490	708.669100	71.696310
-94.608220	51.774700	704.581300	72.011830
-93.149600	52.124570	681.933900	70.697320
-91.665800	52.457450	654.057100	65.422620
-90.156560	52.772640	625.682300	56.503180
-88.621980	53.069450	597.986600	44.333120
-87.062480	53.347160	562.310900	28.174360
-85.478850	53.605050	496.642000	28.583460
-83.872130	53.842430	394.908700	74.435570
-82.243660	54.058630	357.520700	110.906100
-80.595020	54.253030	467.896900	74.948720
-78.928000	54.425040	634.284100	68.777210
-77.244600	54.574130	813.520800	90.766850
-75.546970	54.699840	961.089500	129.267800
-73.837420	54.801750	947.137000	123.801000
-72.118360	54.879540	888.653200	94.766440
-70.392330	54.932960	832.982400	62.398000
-68.661900	54.961820	723.708700	63.194990
-66.929700	54.966030	660.417100	72.980780
-65.198370	54.945580	684.171100	46.725610
-63.470540	54.900540	702.168500	66.878090
-61.748820	54.831050	679.975400	66.830520
-60.035740	54.737340	616.283400	49.060040
-58.333780	54.619720	568.036700	31.167840
-56.645260	54.478570	560.149400	21.146680
-54.972420	54.314330	568.058400	24.218090
-53.317280	54.127510	564.754900	22.106900
-51.681740	53.918690	549.735500	15.699320
-50.067420	53.688470	537.172900	8.747160
-48.475770	53.437500	530.720000	4.181574
-46.907930	53.166490	528.078400	1.799496
-45.364770	52.876130	527.079000	.702770
-43.846840	52.567130	526.727200	.248507
-42.354370	52.240220	526.614600	.079215
-40.887210	51.896080	526.582100	.022700
-39.444820	51.535370	526.573400	.005820
-38.026250	51.158730	526.571400	.001324
-36.630120	50.766700	526.571000	.000375
-98.097820	51.886770	597.991700	41.015340
-96.663910	52.281200	603.313000	41.113000
-95.206040	52.659450	602.262900	42.299930
-93.722790	53.020900	597.787400	42.618840
-92.213200	53.364910	594.295700	42.891130
-90.676770	53.690770	591.376100	43.340140

-89.113440	53.997730	582.962600	40.350510
-87.523560	54.285050	559.914700	30.124010
-85.907830	54.551970	506.478100	25.648230
-84.267300	54.797760	392.328600	60.772590
-82.603340	55.021710	256.693800	107.736800
-80.917590	55.223140	208.950900	136.588600
-79.211960	55.401440	407.754300	86.913480
-77.488560	55.556020	721.626700	70.845920
-75.749730	55.686380	911.028000	115.518200
-73.997920	55.792100	906.296300	112.939700
-72.235800	55.872810	830.113500	78.593310
-70.466090	55.928230	720.869200	60.288230
-68.691570	55.958180	619.437000	87.534960
-66.915130	55.962550	650.194000	62.565700
-65.139640	55.941330	670.869500	57.683910
-63.367970	55.894590	641.850400	58.900960
-61.602940	55.822490	600.853300	44.883620
-59.847310	55.725280	551.784200	31.147550
-58.103760	55.603290	509.664400	25.027670
-56.374820	55.456930	501.878700	24.624460
-54.662870	55.286670	519.150200	17.184900
-52.970100	55.093080	539.417700	10.310420
-51.298500	54.876750	543.081300	11.746850
-49.649800	54.638340	536.016400	7.564089
-48.025460	54.378550	530.486400	3.791042
-46.426660	54.098120	528.011100	1.663766
-44.854210	53.797780	527.068400	.665068
-43.308580	53.478290	526.728900	.241963
-41.789850	53.140400	526.616800	.080009
-40.297680	52.784830	526.583300	.024021
-38.831280	52.412250	526.573900	.006532
-37.389370	52.023280	526.571500	.001628
-35.970190	51.618480	526.571100	.000496
-98.778840	52.740810	555.256800	21.087330
-97.320270	53.148240	555.200400	20.688080
-95.837370	53.539010	554.738900	20.868940
-94.328290	53.912530	554.416100	21.428050
-92.791720	54.268120	555.763100	22.941440
-91.226900	54.605080	559.137500	25.668840
-89.633560	54.922620	562.247700	28.620520
-88.011890	55.219970	561.248400	31.213470
-86.362530	55.496330	543.817400	33.173830
-84.686490	55.750910	489.257000	37.637900
-82.985150	55.982970	357.981200	80.773800
-81.260250	56.191770	188.159900	133.771600
-79.513790	56.376650	268.332500	133.073500
-77.748020	56.536990	653.527600	65.107920
-75.965460	56.672260	863.822000	105.318900
-74.168790	56.781970	866.719300	100.652000
-72.360860	56.865740	784.082800	69.194590
-70.544630	56.923280	667.831400	68.174420
-68.723180	56.954370	618.509300	78.479530
-66.899620	56.958910	695.687300	66.210130
-65.077100	56.936880	681.835700	58.251790
-63.258730	56.888360	612.131700	47.542540
-61.447630	56.813510	557.864900	36.246030
-59.646790	56.712620	528.410500	15.918660
-57.859090	56.586040	508.674400	15.663980
-56.087300	56.434210	503.604400	16.001490
-54.333970	56.257640	514.873800	9.350825
-52.601470	56.056940	532.896400	6.247129
-50.891880	55.832740	540.827000	10.150660
-49.207030	55.585770	536.885100	7.859150
-47.548420	55.316760	531.204000	4.113326
-45.917200	55.026500	528.317100	1.872297
-44.314130	54.715760	527.183300	.761807
-42.739580	54.385350	526.770700	.284413

-41.193420 54.036030 526.630600 .096850
-39.675070 53.668560 526.587300 .030101
-38.183400 53.283620 526.575000 .008525
-36.716770 52.881820 526.571800 .002225
-35.272900 52.463700 526.571200 .000718
-99.499240 53.588130 537.152200 9.601950
-98.014280 54.009130 536.463300 9.101027
-96.504860 54.412960 536.231300 9.043043
-94.968610 54.799050 536.299600 9.383839
-93.403790 55.166730 537.185100 10.383020
-91.809290 55.515250 539.213100 12.259980
-90.184560 55.843850 542.200900 15.062680
-88.529620 56.151670 545.742000 19.214240
-86.844990 56.437890 546.799300 24.887460
-85.131650 56.701690 538.896700 32.758370
-83.390960 56.942230 496.991200 54.689850
-81.624720 57.158780 360.695100 90.004040
-79.835050 57.350580 348.209800 112.746400
-78.024370 57.516980 652.357900 81.817670
-76.195360 57.657400 823.497600 99.120810
-74.350940 57.771310 829.324200 91.623840
-72.494220 57.858320 756.339900 67.667580
-70.628410 57.918090 673.114900 64.821840
-68.756890 57.950390 671.522300 63.325040
-66.883060 57.955100 731.299400 70.465210
-65.010370 57.932220 680.254500 60.344100
-63.142240 57.881810 603.906500 43.007700
-61.282040 57.804080 546.468300 21.139040
-59.433060 57.699310 521.393100 11.738920
-57.598450 57.567890 508.161300 12.508650
-55.781200 57.410300 508.324600 12.286210
-53.984060 57.227100 517.526700 6.295915
-52.209560 57.018940 529.906500 3.118296
-50.459930 56.786500 539.318700 8.776163
-48.737060 56.530560 538.941800 8.749864
-47.042490 56.251900 532.966800 5.065213
-45.377330 55.951360 529.012200 2.317187
-43.742270 55.629770 527.407000 .944038
-42.137470 55.287960 526.841500 .354669
-40.562610 54.926740 526.652300 .122587
-39.016750 54.546860 526.593400 .038840
-37.498410 54.149020 526.576700 .011261
-36.005400 53.733830 526.572300 .003026
-34.534880 53.301770 526.571400 .001009
-100.262700 54.428120 530.133000 3.940219
-98.749250 54.863350 529.743000 3.609391
-97.211560 55.280850 529.628200 3.520193
-95.646590 55.680060 529.697900 3.661218
-94.052090 56.060360 530.081800 4.129375
-92.426510 56.420970 530.926200 5.058308
-90.768940 56.761100 532.313000 6.600160
-89.079190 57.079890 534.368400 9.145653
-87.357570 57.376450 537.195800 13.607640
-85.605000 57.649890 541.177700 22.098280
-83.822840 57.899360 533.507400 34.380440
-82.012920 58.124030 486.813900 45.760770
-80.177480 58.323120 491.267500 57.897880
-78.319110 58.495910 686.112700 90.846230
-76.440710 58.641750 768.919300 92.651430
-74.545430 58.760110 780.007100 84.222650
-72.636660 58.850530 751.957800 66.319830
-70.717930 58.912650 711.393800 61.324070
-68.792920 58.946230 725.746900 64.529940
-66.865380 58.951130 735.608900 73.497120
-64.939080 58.927340 668.886000 62.073850
-63.017780 58.874950 595.038300 39.221470
-61.105210 58.794160 547.266500 17.721240

-59.204930 58.685300 522.512900 6.470312
-57.320400 58.548780 509.997900 11.546330
-55.454840 58.385130 508.292600 12.653320
-53.611270 58.194950 513.984700 9.047046
-51.792370 57.978940 523.955800 2.034007
-50.000500 57.737840 534.921600 5.567698
-48.237630 57.472490 539.645600 8.870572
-46.505310 57.183730 534.514600 5.815073
-44.804610 56.872430 529.677200 2.685205
-43.136050 56.539500 527.618300 1.099167
-41.499610 56.185780 526.908000 .414763
-39.894630 55.812120 526.673400 .145446
-38.319800 55.419290 526.599700 .046863
-36.773090 55.007980 526.578400 .013840
-35.251730 54.578750 526.572700 .003809
-33.752120 54.132060 526.571500 .001286
-101.073700 55.260130 527.668900 1.461823
-99.529010 55.710320 527.506800 1.290570
-97.960900 56.142140 527.464100 1.238461
-96.365430 56.555100 527.503500 1.296008
-94.739640 56.948590 527.666700 1.501013
-93.081440 57.321860 528.016800 1.915183
-91.389490 57.674070 528.655400 2.653937
-89.663250 58.004340 529.826500 4.082162
-87.902820 58.311730 532.224400 7.296328
-86.109000 58.595320 536.753500 14.327700
-84.283100 58.854170 536.077900 24.278310
-82.427000 59.087400 519.216400 32.421940
-80.543050 59.294160 565.640700 46.843700
-78.634010 59.473690 670.145600 78.045100
-76.703000 59.625280 646.532300 68.683430
-74.753460 59.748340 682.938400 71.275850
-72.789070 59.842370 719.549100 71.818730
-70.813740 59.906970 737.659900 63.854550
-68.831480 59.941910 755.248000 72.334990
-66.846440 59.947010 732.980500 75.650900
-64.862770 59.922250 656.220900 59.493140
-62.884610 59.867760 586.563700 35.883450
-60.916040 59.783740 546.828700 15.975560
-58.961010 59.670550 527.033000 4.163625
-57.023270 59.528640 516.538700 7.183228
-55.106350 59.358580 512.026500 10.741970
-53.213500 59.161040 512.003800 10.372780
-51.347600 58.936760 516.851900 6.913285
-49.511150 58.686560 526.916600 .401388
-47.706190 58.411320 535.973900 6.452390
-45.934240 58.111950 533.977700 5.196924
-44.196280 57.789400 529.768600 2.615619
-42.492650 57.444580 527.686800 1.119727
-40.823030 57.078400 526.937300 .431200
-39.186360 56.691730 526.684800 .154218
-37.580830 56.285340 526.603600 .050732
-36.003750 55.859910 526.579600 .015271
-34.451600 55.415950 526.573000 .004271
-32.919830 54.953830 526.571500 .001490
-101.937200 56.083360 526.883200 .488869
-100.358100 56.549350 526.827500 .415910
-98.756940 56.996240 526.813000 .393840
-97.128790 57.423640 526.831400 .420471
-95.469860 57.830970 526.899900 .512491
-93.777320 58.217490 527.062000 .710266
-92.049300 58.582370 527.423600 1.139985
-90.284800 58.924680 528.279100 2.245511
-88.483640 59.243460 530.394300 5.144382
-86.646400 59.537720 534.243200 11.514170
-84.774360 59.806450 532.433700 20.289080
-82.869400 60.048700 518.702900 27.037830

-80.933970 60.263580 569.799300 32.644070
-78.971000 60.450230 606.589700 55.885590
-76.983890 60.607900 500.685300 96.840670
-74.976370 60.735940 507.732100 89.381230
-72.952470 60.833800 604.299100 75.308060
-70.916480 60.901050 690.987800 67.182170
-68.872850 60.937420 758.918200 75.537020
-66.826130 60.942730 728.061100 73.636690
-64.780930 60.916960 642.459000 54.369000
-62.741830 60.860230 577.689600 31.795660
-60.713310 60.772790 545.414700 14.956090
-58.699720 60.654990 530.503100 4.416771
-56.705200 60.507380 523.118400 2.854070
-54.733590 60.330550 519.343400 5.669196
-52.788400 60.125220 516.308200 7.810749
-50.872730 59.892220 515.663100 7.701782
-48.989200 59.632420 520.441500 4.551851
-47.139900 59.346780 529.225300 2.033743
-45.326320 59.036270 531.856300 3.681996
-43.549270 58.701890 529.556400 2.240178
-41.808860 58.344600 527.701000 1.024741
-40.104370 57.965370 526.939000 .402446
-38.434220 57.565060 526.684600 .145726
-36.795950 57.144450 526.603800 .048554
-35.186060 56.704150 526.579700 .014873
-33.600030 56.244660 526.573100 .004239
-32.032190 55.766200 526.571600 .001494
-102.859900 56.896880 526.653500 .147548
-101.242000 57.379640 526.636200 .120834
-99.604390 57.842470 526.632500 .114021
-97.940930 58.285070 526.641300 .127729
-96.246660 58.706940 526.673500 .173819
-94.517820 59.107400 526.762200 .296332
-92.751850 59.485580 527.001200 .658026
-90.947170 59.840560 527.654100 1.717172
-89.103220 60.171320 529.327700 4.480569
-87.220290 60.476800 531.832100 10.432640
-85.299550 60.755960 526.475600 19.371970
-83.342830 61.007760 507.572800 29.099210
-81.352710 61.231220 542.311900 21.889010
-79.332320 61.425420 552.372800 36.564270
-77.285290 61.589540 409.170300 104.382100
-75.215710 61.722870 345.244000 139.556800
-73.128000 61.824790 396.090700 142.277600
-71.026890 61.894870 559.127000 89.939960
-68.917300 61.932760 719.608000 74.643660
-66.804310 61.938290 712.348600 68.600190
-64.692990 61.911450 627.966200 47.801810
-62.588420 61.852340 569.656600 27.175790
-60.495590 61.761240 543.403000 13.316330
-58.419300 61.638580 531.929900 5.011476
-56.364090 61.484900 526.546800 .809695
-54.334160 61.300890 523.963900 2.410237
-52.333320 61.087320 521.792500 3.926696
-50.364880 60.845090 519.661300 5.104475
-48.431600 60.575160 520.008700 4.668575
-46.535590 60.278550 524.938800 1.834413
-44.678250 59.956310 529.077400 2.039958
-42.860180 59.609490 528.839800 1.694799
-41.081100 59.239120 527.510600 .769278
-39.339830 58.846180 526.881700 .317727
-37.634110 58.431540 526.665300 .116032
-35.960610 57.995940 526.598400 .039485
-34.314830 57.539980 526.578400 .012285
-32.690990 57.064010 526.572800 .003565
-31.082000 56.568130 526.571500 .001284
-103.849600 57.699570 526.590900 .040202

-102.187300 58.200280 526.586200 .031820
-100.509000 58.680030 526.585800 .030713
-98.806840 59.138710 526.590900 .038907
-97.074520 59.575940 526.608600 .069041
-95.307120 59.991050 526.664600 .173041
-93.501110 60.383240 526.828700 .510171
-91.654170 60.751550 527.279400 1.486969
-89.765210 61.094930 528.342300 4.009234
-87.834170 61.412270 529.383100 9.637211
-85.861950 61.702450 523.484600 18.732220
-83.850420 61.964360 507.660100 29.160940
-81.802150 62.196920 532.651100 19.874120
-79.720490 62.399150 516.573500 36.831940
-77.609360 62.570130 392.212300 104.611100
-75.473210 62.709080 282.692600 160.473000
-73.316960 62.815350 271.580700 169.184300
-71.145790 62.888430 404.788200 141.071000
-68.965200 62.927950 627.931600 82.258910
-66.780790 62.933720 664.787400 58.247240
-64.598260 62.905720 603.632800 37.315070
-62.423240 62.844070 558.761000 20.749530
-60.261290 62.749080 538.824800 10.038970
-58.117710 62.621230 530.676600 4.010919
-55.997520 62.461110 527.271200 .924874
-53.905320 62.269450 525.800800 .794946
-51.845240 62.047150 524.760600 1.739714
-49.820820 61.795150 523.640400 2.438236
-47.834930 61.514500 523.152800 2.499982
-45.889670 61.206310 524.470900 1.593441
-43.986290 60.871700 527.106200 .555541
-42.125070 60.511780 527.350700 .578621
-40.305270 60.127630 526.972500 .382625
-38.525020 59.720250 526.725600 .173088
-36.781200 59.290500 526.625200 .070270
-35.069400 58.839090 526.588100 .025274
-33.383770 58.366500 526.575900 .008220
-31.716990 57.872960 526.572100 .002470
-30.060130 57.358350 526.571400 .000921
-104.916500 58.490070 526.575400 .009899
-103.202300 59.010120 526.574200 .007693
-101.477600 59.507980 526.574500 .008291
-99.732440 59.983750 526.577700 .014326
-97.958720 60.437220 526.588500 .039034
-96.150010 60.867820 526.624500 .128263
-94.301540 61.274790 526.731900 .415969
-92.410060 61.657170 527.009900 1.239156
-90.473700 62.013880 527.554900 3.389277
-88.491920 62.343760 528.046200 8.314423
-86.465330 62.645610 525.856500 16.084050
-84.395630 62.918240 523.135500 20.132030
-82.285480 63.160470 538.917600 13.643150
-80.138370 63.371240 512.582200 29.316310
-77.958560 63.549540 426.276200 76.729300
-75.750930 63.694510 340.879800 124.998900
-73.520860 63.805420 308.268200 137.159700
-71.274150 63.881700 380.184600 103.701400
-69.016910 63.922960 537.530300 48.978180
-66.755400 63.928990 586.356200 34.612630
-64.495980 63.899750 565.364200 23.059740
-62.244980 63.835380 543.927600 12.658550
-60.008560 63.736250 533.382400 6.148837
-57.792640 63.602840 528.925700 2.563168
-55.602750 63.435840 527.127000 .748091
-53.443960 63.236060 526.385700 .212543
-51.320750 63.004450 525.953900 .668799
-49.236890 62.742080 525.560500 .990705
-47.195330 62.450080 525.323200 1.077728

-45.198120	62.129630	525.594400	.736584
-43.246250	61.781950	526.446400	.169188
-41.339580	61.408210	526.716300	.156740
-39.476710	61.009530	526.686300	.128444
-37.654880	60.586900	526.630600	.074119
-35.869840	60.141170	526.595000	.033634
-34.115770	59.672970	526.579200	.013126
-32.385130	59.182660	526.573400	.004447
-30.668580	58.670220	526.571500	.001377
-28.954870	58.135290	526.571100	.000561
-106.073800	59.266680	526.571800	.002221
-104.297500	59.807790	526.571600	.001815
-102.518800	60.325180	526.572000	.002713
-100.725000	60.819250	526.573700	.007484
-98.905530	61.289990	526.580000	.026304
-97.052100	61.737010	526.600900	.093019
-95.158340	62.159620	526.662600	.302987
-93.219700	62.556880	526.801100	.897596
-91.233350	62.927700	527.051000	2.426226
-89.198040	63.270870	527.292400	5.918559
-87.113920	63.585110	528.081500	10.266530
-84.982480	63.869130	528.293800	7.217760
-82.806390	64.121680	527.341700	14.700140
-80.589290	64.341560	494.213300	35.185830
-78.335770	64.527670	443.777300	68.683140
-76.051160	64.679080	403.753000	98.992990
-73.741460	64.794950	394.235300	99.321440
-71.413090	64.874680	434.067500	66.383630
-69.072890	64.917820	509.335700	26.514480
-66.727910	64.924110	541.192400	14.117270
-64.385280	64.893550	539.976700	9.823045
-62.052090	64.826280	533.664900	6.065354
-59.735260	64.722690	529.624900	3.130099
-57.441380	64.583340	527.692700	1.372176
-55.176610	64.408980	526.880000	.452961
-52.946510	64.200520	526.547200	.056534
-50.755950	63.959000	526.373000	.237877
-48.608900	63.685590	526.246300	.368036
-46.508400	63.381530	526.180800	.402997
-44.456330	63.048090	526.239000	.306373
-42.453320	62.686580	526.466000	.098677
-40.498620	62.298210	526.577000	.015796
-38.589950	61.884150	526.599200	.035823
-36.723370	61.445380	526.589800	.025926
-34.893150	60.982680	526.579700	.013470
-33.091610	60.496560	526.574200	.005601
-31.309040	59.987160	526.571900	.002005
-29.533510	59.454220	526.571200	.000666
-27.750760	58.896990	526.571000	.000302
-107.339200	60.027230	526.571100	.000526
-105.487000	60.591610	526.571000	.000573
-103.643800	61.130270	526.571400	.001338
-101.793500	61.644070	526.572400	.004776
-99.922550	62.133320	526.575300	.017486
-98.020040	62.597820	526.584500	.060189
-96.077510	63.037030	526.607300	.186858
-94.088710	63.450070	526.643400	.507041
-92.049460	63.835860	526.586300	1.208686
-89.957580	64.193130	526.114800	2.421952
-87.812570	64.520550	524.624700	2.983203
-85.615560	64.816710	521.403600	8.368006
-83.369120	65.080260	511.356700	19.189170
-81.077050	65.309890	491.972200	32.625480
-78.744270	65.504390	474.045600	48.207340
-76.376630	65.662700	463.859900	59.209240
-73.980770	65.783920	466.047300	56.577840
-71.563890	65.867360	485.603100	36.718270

-69.133670 65.912500 513.142200 16.098890
-66.698060 65.919100 527.608600 5.978272
-64.265090 65.887100 530.077300 3.629617
-61.842790 65.816700 528.985800 2.479167
-59.438900 65.708320 527.764400 1.419660
-57.060850 65.562580 527.046100 .666373
-54.715470 65.380330 526.718600 .241265
-52.408880 65.162570 526.579900 .036331
-50.146370 64.910470 526.511400 .079796
-47.932120 64.625290 526.468300 .133159
-45.769150 64.308400 526.449600 .147186
-43.659080 63.961160 526.468400 .115807
-41.602020 63.584970 526.524200 .053424
-39.596350 63.181100 526.563400 .009130
-37.638600 62.750720 526.575700 .006711
-35.723270 62.294770 526.575900 .007639
-33.842640 61.813940 526.573600 .004551
-31.986660 61.308540 526.572000 .002087
-30.142740 60.778430 526.571200 .000806
-28.295590 60.222970 526.571000 .000308
-26.427080 59.640870 526.570900 .000253
-108.736700 60.768890 526.570900 .000302
-106.789100 61.359350 526.571000 .000389
-104.866900 61.921510 526.571200 .001137
-102.949500 62.456830 526.571700 .004089
-101.019200 62.966030 526.573000 .014312
-99.061880 63.449270 526.575000 .045577
-97.066180 63.906190 526.574200 .126648
-95.023590 64.336020 526.538600 .299311
-92.928180 64.737730 526.325800 .592739
-90.776390 65.110020 525.492700 1.202656
-88.566850 65.451470 523.247100 3.222187
-86.300130 65.760610 518.562000 9.566271
-83.978550 66.035940 509.517000 17.891610
-81.606060 66.276030 500.609400 25.609940
-79.187900 66.479550 495.476100 31.932580
-76.730480 66.645290 493.921000 35.559680
-74.241170 66.772270 497.084700 32.754370
-71.728080 66.859700 506.279100 22.724910
-69.199870 66.907030 517.791900 11.584510
-66.665550 66.913940 524.943100 4.540019
-64.134220 66.880400 527.307600 1.867276
-61.614990 66.806620 527.485700 1.216928
-59.116610 66.693070 527.121800 .770402
-56.647430 66.540450 526.819500 .393783
-54.215080 66.349720 526.658300 .156234
-51.826330 66.121990 526.585000 .033924
-49.486860 65.858530 526.549600 .031969
-47.201050 65.560780 526.529600 .060867
-44.971820 65.230190 526.521700 .068727
-42.800360 64.868270 526.528800 .056504
-40.685980 64.476460 526.547900 .031069
-38.625890 64.056100 526.563600 .010461
-36.614990 63.608330 526.570600 .000486
-34.645670 63.134020 526.572100 .002119
-32.707610 62.633670 526.571800 .001744
-30.787590 62.107330 526.571400 .000925
-28.869240 61.554470 526.571100 .000386
-26.932890 60.973880 526.570900 .000261
-24.955330 60.363580 526.570900 .000210

Q.25a

-89.453400	32.991660	554.794400	.358679
-88.345930	33.191160	554.814400	.411352
-87.228100	33.381660	554.896800	.627504
-86.100230	33.562890	555.046000	.990700
-84.962690	33.734530	555.274700	1.484270
-83.815900	33.896310	555.585100	2.066849
-82.660300	34.047950	555.937100	2.650613
-81.496410	34.189190	556.224900	3.103659
-80.324730	34.319790	556.323000	3.281591
-79.145840	34.439500	556.206400	3.061762
-77.960310	34.548100	555.879200	2.391231
-76.768760	34.645400	555.267900	1.377412
-75.571840	34.731200	554.456800	.439799
-74.370180	34.805370	553.944400	.636048
-73.164480	34.867740	553.946200	.838363
-71.955430	34.918200	554.229400	.659039
-70.743730	34.956650	554.522800	.325352
-69.530080	34.983020	554.717200	.030049
-68.315220	34.997260	554.803400	.177703
-67.099880	34.999340	554.810200	.224160
-65.884770	34.989250	554.778500	.181221
-64.670640	34.967020	554.747100	.114289
-63.458190	34.932690	554.728100	.061159
-62.248150	34.886320	554.720200	.030786
-61.041230	34.828000	554.717200	.016742
-59.838120	34.757850	554.716300	.011335
-58.639500	34.676010	554.715800	.009307
-57.446040	34.582610	554.715600	.008266
-56.258390	34.477850	554.715200	.007014
-55.077160	34.361910	554.714500	.005268
-53.902950	34.235010	554.713700	.003323
-52.736340	34.097380	554.713100	.001731
-51.577860	33.949260	554.712900	.000785
-50.428010	33.790910	554.712800	.000340
-49.287250	33.622600	554.712700	.000189
-48.156040	33.444620	554.712700	.000182
-47.034740	33.257250	554.712700	.000183
-45.923690	33.060790	554.712700	.000183
-44.823220	32.855560	554.712700	.000183
-89.701210	33.918400	554.938100	.727823
-88.583520	34.125020	555.018700	.869950
-87.455040	34.322360	555.292200	1.337690
-86.316120	34.510110	555.775300	2.102255
-85.167100	34.687960	556.514000	3.133875
-84.008420	34.855620	557.522800	4.351068
-82.840550	35.012790	558.682300	5.582557
-81.663990	35.159200	559.643200	6.573247
-80.479300	35.294580	560.003700	7.014287
-79.287040	35.418700	559.682100	6.640422
-78.087840	35.531310	558.685700	5.307574

-76.882320	35.632210	556.770900	3.143831
-75.671150	35.721200	554.000700	1.155384
-74.455030	35.798110	552.278200	1.216433
-73.234660	35.862810	552.287300	1.505154
-72.010770	35.915150	553.229500	1.186049
-70.784100	35.955040	554.195900	.477743
-69.555380	35.982390	554.824000	.249230
-68.325390	35.997170	555.083100	.566284
-67.094890	35.999320	555.071700	.598624
-65.864640	35.988860	554.939900	.451948
-64.635410	35.965800	554.823400	.272853
-63.407950	35.930180	554.761400	.144099
-62.183030	35.882080	554.736700	.073177
-60.961370	35.821590	554.728800	.041732
-59.743720	35.748840	554.726300	.029419
-58.530780	35.663950	554.724800	.024459
-57.323260	35.567100	554.723300	.021211
-56.121820	35.458470	554.721600	.017748
-54.927120	35.338260	554.718700	.012743
-53.739770	35.206700	554.715800	.007604
-52.560380	35.064020	554.713900	.003806
-51.389480	34.910500	554.713100	.001617
-50.227610	34.746390	554.712800	.000592
-49.075240	34.571990	554.712700	.000227
-47.932830	34.387580	554.712700	.000182
-46.800740	34.193490	554.712700	.000183
-45.679330	33.990000	554.712700	.000183
-44.568900	33.777440	554.712700	.000183
-89.960660	34.843320	555.560300	1.896752
-88.832330	35.057210	555.940600	2.328045
-87.692770	35.261540	557.081400	3.564155
-86.542320	35.455950	559.030700	5.513488
-85.381340	35.640140	561.952800	8.106325
-84.210270	35.813810	565.901700	11.141390
-83.029580	35.976620	570.410200	14.231810
-81.839790	36.128310	574.224600	16.819140
-80.641480	36.268600	575.790800	18.105150
-79.435230	36.397220	574.772000	17.374790
-78.221690	36.513940	571.098200	14.131100
-77.001530	36.618520	564.069600	8.753449
-75.775430	36.710780	552.649400	3.380251
-74.544130	36.790520	546.191200	2.740150
-73.308360	36.857590	545.761000	3.095452
-72.068890	36.911860	549.375300	2.300443
-70.826490	36.953220	553.031000	.940660
-69.581950	36.981590	555.482500	1.317649
-68.336070	36.996900	556.450600	1.978888
-67.089650	36.999130	556.307200	1.900655
-65.843500	36.988290	555.680300	1.356393
-64.598400	36.964370	555.164300	.802385
-63.355190	36.927440	554.909100	.423744
-62.114630	36.877570	554.814900	.224658
-60.877510	36.814860	554.786900	.134107
-59.644590	36.739430	554.777500	.097890
-58.416640	36.651430	554.769300	.080537
-57.194370	36.551040	554.760600	.069145
-55.978490	36.438440	554.751600	.055546
-54.769680	36.313860	554.738200	.038690
-53.568590	36.177530	554.725300	.021814
-52.375820	36.029710	554.717500	.010152
-51.191970	35.870660	554.714100	.004126
-50.017550	35.700660	554.713000	.001459
-48.853080	35.520030	554.712800	.000450
-47.698980	35.329070	554.712700	.000194
-46.555660	35.128100	554.712700	.000182
-45.423470	34.917440	554.712600	.000209
-44.302690	34.697410	554.712700	.000186

-90.232250	35.766320	557.684600	4.531831
-89.092860	35.987640	559.229900	5.674648
-87.941790	36.199090	563.432600	8.562699
-86.779330	36.400320	570.305700	12.966970
-85.605870	36.591000	580.370500	18.714690
-84.421870	36.770790	593.679400	25.400610
-83.227800	36.939400	608.882400	32.394580
-82.024180	37.096500	622.314800	38.545490
-80.811620	37.241800	628.148300	41.900040
-79.590740	37.375040	625.286900	40.685140
-78.362190	37.495960	613.851600	33.857560
-77.126670	37.604320	590.505600	20.826680
-75.884930	37.699920	545.745400	10.600170
-74.637700	37.782550	530.920400	6.420844
-73.385770	37.852060	527.172500	5.837872
-72.129940	37.908310	538.532100	4.031262
-70.871030	37.951180	550.706500	2.996218
-69.609860	37.980580	558.909200	4.883788
-68.347290	37.996460	561.940200	6.196462
-67.084140	37.998780	561.037200	5.504610
-65.821270	37.987530	558.362000	3.720721
-64.559530	37.962740	556.362100	2.104043
-63.299760	37.924460	555.432700	1.133602
-62.042780	37.872770	555.114300	.625307
-60.789440	37.807780	555.020600	.402689
-59.540510	37.729610	554.983600	.305106
-58.296810	37.638420	554.945800	.253106
-57.059080	37.534390	554.902500	.208974
-55.828080	37.417740	554.866800	.167014
-54.604500	37.288680	554.807300	.108990
-53.389020	37.147460	554.755400	.056410
-52.182290	36.994370	554.728100	.024565
-50.984880	36.829660	554.717000	.008968
-49.797380	36.653650	554.713600	.003008
-48.620270	36.466650	554.712800	.000893
-47.454020	36.268990	554.712500	.000278
-46.299010	36.060990	554.712700	.000301
-45.155610	35.843010	554.712600	.000225
-44.024070	35.615370	554.712700	.000196
-90.516530	36.687310	563.771600	9.437045
-89.365660	36.916220	568.983300	11.951090
-88.202590	37.134950	582.112100	17.801540
-87.027650	37.343150	602.449000	26.070360
-85.841200	37.540460	631.912000	36.857200
-84.643710	37.726540	669.856300	49.626780
-83.435650	37.901050	713.959000	63.380920
-82.217600	38.063690	757.817600	76.359310
-80.990140	38.214140	775.884200	84.241310
-79.753940	38.352110	768.127200	83.543790
-78.509670	38.477340	738.930200	71.013230
-77.258070	38.589580	665.875000	43.692040
-75.999910	38.688600	518.848000	31.127450
-74.735980	38.774210	487.291700	28.049840
-73.467090	38.846230	493.375500	22.331490
-72.194090	38.904510	517.215900	10.998050
-70.917830	38.948930	549.931900	10.243270
-69.639210	38.979390	575.325900	15.329170
-68.359090	38.995840	582.717200	17.160730
-67.078360	38.998240	577.956000	14.564170
-65.797930	38.986590	566.784300	9.013373
-64.518690	38.960900	559.825000	4.925799
-63.241520	38.921240	557.015900	2.647927
-61.967320	38.867690	556.112200	1.597944
-60.696920	38.800350	555.846000	1.113704
-59.431210	38.719360	555.720900	.902777
-58.170970	38.624900	555.561600	.748855
-56.917050	38.517150	555.387600	.609539

-55.670200	38.396340	555.312900	.486183
-54.431160	38.262690	555.048300	.299346
-53.200640	38.116470	554.833100	.127810
-51.979310	37.957960	554.750500	.047844
-50.767770	37.787460	554.722800	.016294
-49.566610	37.605300	554.714700	.004617
-48.376340	37.411790	554.712800	.001280
-47.197410	37.207270	554.712500	.000325
-46.030250	36.992090	554.712500	.000182
-44.875180	36.766620	554.712600	.000220
-43.732470	36.531200	554.712700	.000205
-90.814130	37.606180	577.599200	16.841090
-89.651300	37.842850	592.347300	21.804670
-88.475770	38.069030	628.753300	31.973320
-87.287830	38.284350	679.744600	44.609810
-86.087840	38.488450	754.109900	60.509730
-84.876290	38.680970	849.835200	82.201830
-83.653650	38.861550	963.339800	105.543500
-82.420500	39.029860	1090.596000	125.924000
-81.177470	39.185590	1141.006000	143.643200
-79.925230	39.328410	1124.706000	148.693600
-78.664510	39.458070	1070.861000	130.178700
-77.396060	39.574290	900.094800	84.730700
-76.120680	39.676830	492.573200	104.905200
-74.839220	39.765490	341.607600	57.131070
-73.552520	39.840080	441.203900	71.634190
-72.261480	39.900440	506.605400	39.678810
-70.967010	39.946460	569.486800	30.773860
-69.670030	39.978020	639.956200	35.726650
-68.371470	39.995060	656.691500	40.264020
-67.072270	39.997550	634.883100	31.923690
-65.773400	39.985470	589.744500	18.622340
-64.475770	39.958870	568.320400	9.882273
-63.180340	39.917780	561.112800	5.786579
-61.888020	39.862300	559.206700	3.815888
-60.599740	39.792560	558.765100	3.027743
-59.316390	39.708680	558.543600	2.649117
-58.038840	39.610860	557.920100	2.242233
-56.767930	39.499290	557.156500	1.826396
-55.504470	39.374200	556.689200	1.149710
-54.249260	39.235840	555.757200	.683269
-53.003000	39.084480	555.022300	.269110
-51.766410	38.920440	554.777400	.071291
-50.540120	38.744010	554.726300	.019132
-49.324710	38.555530	554.714900	.005126
-48.120720	38.355340	554.712800	.000976
-46.928620	38.143810	554.712500	.000225
-45.748810	37.921300	554.712500	.000187
-44.581620	37.688170	554.712500	.000214
-43.427300	37.444810	554.712700	.000223
-91.125630	38.522790	601.485500	25.872180
-89.950410	38.767390	639.275900	34.561550
-88.761920	39.001200	730.395900	48.743600
-87.560450	39.223820	836.707600	62.592990
-86.346370	39.434880	982.849400	70.652700
-85.120160	39.633990	1193.436000	95.904500
-83.882300	39.820790	1445.327000	131.079700
-82.633390	39.994930	1734.359000	149.336300
-81.374080	40.156070	1856.170000	183.417500
-80.105060	40.303890	1847.784000	212.058400
-78.827100	40.438090	1808.327000	194.883200
-77.540980	40.558400	1605.649000	126.227500
-76.247540	40.664560	876.875500	96.231290
-74.947690	40.756360	135.808700	201.605900
-73.642300	40.833600	354.596900	106.418100
-72.332310	40.896110	526.561600	80.026810
-71.018690	40.943760	659.176000	69.446960

-69.702430	40.976440	805.927800	58.315490
-68.384490	40.994090	837.899400	61.731590
-67.065880	40.996670	759.744400	45.319490
-65.747600	40.984170	628.279100	26.104480
-64.430650	40.956610	582.872000	16.158930
-63.116020	40.914060	572.964700	12.063800
-61.804690	40.856610	569.119700	8.848049
-60.497620	40.784390	568.555000	7.382318
-59.195770	40.697540	567.920400	6.764990
-57.900040	40.596260	565.513800	5.926739
-56.611330	40.480750	561.998700	4.200268
-55.330490	40.351270	558.864700	2.430898
-54.058330	40.208070	556.406100	.971527
-52.795620	40.051450	555.083800	.252716
-51.543080	39.881710	554.767800	.065465
-50.301390	39.699200	554.713100	.011219
-49.071130	39.504250	554.710900	.002304
-47.852850	39.297230	554.711700	.000730
-46.647030	39.078520	554.712300	.000339
-45.454070	38.848490	554.712400	.000223
-44.274280	38.607530	554.712400	.000143
-43.107920	38.356030	554.712700	.000233
-91.451740	39.437030	630.959100	33.430230
-90.263630	39.689750	708.340600	45.340680
-89.061680	39.931370	890.788900	60.364740
-87.846150	40.161480	1019.679000	67.032320
-86.617400	40.379670	1127.868000	92.340680
-85.375900	40.585540	1470.430000	129.475400
-84.122160	40.778730	1926.530000	123.807200
-82.856800	40.958850	2356.094000	175.313600
-81.580460	41.125550	2607.730000	214.240900
-80.293880	41.278500	2730.546000	251.761900
-78.997860	41.417370	2751.168000	261.891400
-77.693230	41.541890	2542.867000	212.887500
-76.380870	41.651780	1829.015000	127.659200
-75.061690	41.746810	878.434100	96.132580
-73.736660	41.826770	467.908600	147.978600
-72.406780	41.891490	575.453400	83.730340
-71.073040	41.940830	821.108300	87.084720
-69.736500	41.974670	968.412400	87.942570
-68.398190	41.992950	969.102900	83.790310
-67.059160	41.995620	766.308700	79.400490
-65.720480	41.982670	567.416200	25.494520
-64.383220	41.954140	593.282000	23.785990
-63.048400	41.910080	606.556800	22.144570
-61.717090	41.850600	594.299600	16.721770
-60.390290	41.775820	584.019300	13.110550
-59.069010	41.685920	576.313000	13.093190
-57.754210	41.581080	570.564400	11.062360
-56.446830	41.461530	564.307600	6.040834
-55.147770	41.327530	554.430600	1.887551
-53.857880	41.179360	552.597200	1.154694
-52.577970	41.017320	553.882600	.442844
-51.308780	40.841740	554.506200	.139452
-50.051000	40.652980	554.672100	.030690
-48.805250	40.451390	554.703400	.008436
-47.572090	40.237360	554.710200	.002273
-46.352010	40.011290	554.712000	.000622
-45.145370	39.773560	554.712400	.000259
-43.952510	39.524580	554.712500	.000200
-42.773630	39.264750	554.712700	.000256
-91.793200	40.348750	641.639700	34.769310
-90.591710	40.609810	733.830600	48.903810
-89.375760	40.859430	931.522900	71.917780
-88.145600	41.097210	915.745500	74.717310
-86.901570	41.322720	566.163900	197.271500
-85.644150	41.535550	840.573100	196.014100

-84.373860 41.735290 1707.706000 76.734160
-83.091290 41.921560 2345.421000 192.478300
-81.797160 42.093980 2829.017000 272.485300
-80.492230 42.252200 3253.719000 319.641600
-79.177290 42.395880 3446.479000 327.829900
-77.853240 42.524730 3233.927000 288.856800
-76.521000 42.638450 2500.629000 195.621700
-75.181550 42.736820 1636.432000 87.426830
-73.835900 42.819590 937.081000 75.093860
-72.485100 42.886590 626.895600 113.331900
-71.130210 42.937670 966.670800 187.650200
-69.772340 42.972710 895.008400 127.804000
-68.412590 42.991630 771.581500 88.245770
-67.052090 42.994400 356.830800 153.152200
-65.691960 42.980990 346.206000 118.340500
-64.333320 42.951450 606.260900 36.652450
-62.977290 42.905830 664.562900 38.733730
-61.624970 42.844260 603.071100 21.052430
-60.277440 42.766850 548.865400 15.909790
-58.935750 42.673790 521.100200 17.167830
-57.600940 42.565280 524.196500 13.881450
-56.273990 42.441570 541.548400 6.365238
-54.955840 42.302930 548.381800 4.399672
-53.647390 42.149640 552.453600 1.635357
-52.349470 41.982030 554.194100 .474678
-51.062890 41.800450 554.637800 .163617
-49.788320 41.605260 554.701800 .077611
-48.526440 41.396860 554.711200 .031099
-47.277780 41.175640 554.712400 .010912
-46.042850 40.942000 554.712500 .003141
-44.822010 40.696380 554.712500 .000791
-43.615560 40.439180 554.712400 .000166
-42.423690 40.170830 554.712700 .000234
-92.150770 41.257820 620.305100 26.624250
-90.935390 41.527410 683.987500 38.751630
-89.704910 41.785250 822.851200 60.966870
-88.459540 42.030900 810.304000 91.022380
-87.199620 42.263930 526.365300 102.911800
-85.925610 42.483890 726.379400 128.251300
-84.638040 42.690380 1623.769000 89.848820
-83.337520 42.882970 2395.242000 216.955100
-82.024790 43.061280 3059.854000 313.591000
-80.700610 43.224930 3709.177000 364.961100
-79.365870 43.373580 3928.959000 387.209400
-78.021450 43.506890 3651.096000 336.553700
-76.668370 43.624570 2925.597000 228.137500
-75.307630 43.726360 2043.760000 107.869400
-73.940300 43.812040 1276.915000 138.523000
-72.567500 43.881390 370.315700 153.562600
-71.190370 43.934270 688.142300 110.818600
-69.810050 43.970550 1109.590000 119.442900
-68.427750 43.990140 1204.094000 95.269630
-67.044650 43.993000 678.366000 101.461300
-65.661930 43.979120 589.635800 57.043820
-64.280810 43.948540 726.502000 66.781800
-62.902460 43.901310 591.291600 39.898590
-61.528050 43.837570 499.738000 32.337220
-60.158730 43.757450 485.414400 31.041880
-58.795620 43.661140 512.313300 19.920040
-57.439790 43.548850 547.429400 15.751510
-56.092310 43.420850 553.066000 10.859440
-54.754150 43.277410 555.038300 4.906162
-53.426270 43.118850 555.759700 1.905011
-52.109530 42.945500 555.470800 1.173093
-50.804760 42.757750 555.042400 .639540
-49.512690 42.555960 554.825300 .291242
-48.233980 42.340550 554.745500 .113937

-46.969190	42.111940	554.721100	.038451
-45.718810	41.870550	554.714500	.011398
-44.483210	41.616830	554.712900	.002936
-43.262650	41.351200	554.712500	.000660
-42.057300	41.074100	554.712700	.000244
-92.525320	42.164070	582.813800	13.347540
-91.295520	42.442420	590.040400	16.421700
-90.049940	42.708690	584.588000	31.737600
-88.788760	42.962440	499.862900	73.132360
-87.512310	43.203190	406.825900	78.331630
-86.221000	43.430500	590.625800	90.784850
-84.915410	43.643920	1497.696000	105.590300
-83.596150	43.843030	2413.943000	238.403800
-82.263960	44.027400	3186.332000	343.360200
-80.919660	44.196660	3983.364000	381.689500
-79.564150	44.350410	4161.547000	383.952500
-78.198390	44.488330	3724.634000	324.833800
-76.823410	44.610100	2786.456000	220.420800
-75.440290	44.715440	1867.772000	113.021600
-74.050180	44.804110	1215.136000	222.773300
-72.654240	44.875890	134.738700	183.178900
-71.253690	44.930630	720.818400	181.048500
-69.849760	44.968180	1597.722000	143.557300
-68.443720	44.988460	2012.174000	256.188800
-67.036810	44.991420	1254.704000	88.991140
-65.630330	44.977050	864.974300	61.492940
-64.225530	44.945390	684.149000	50.754500
-62.823690	44.896510	339.375700	106.541800
-61.426040	44.830540	388.700000	56.954790
-60.033810	44.747610	503.316100	34.893610
-58.648170	44.647940	576.888400	30.022500
-57.270290	44.531750	582.663800	22.129780
-55.901260	44.399320	570.322000	15.624070
-54.542130	44.250930	566.190200	8.039647
-53.193900	44.086940	561.877500	5.547261
-51.857480	43.907680	558.135600	3.372640
-50.533710	43.713560	556.056800	1.718853
-49.223370	43.504980	555.149100	.757023
-47.927120	43.282370	554.834200	.290220
-46.645540	43.046150	554.742700	.097176
-45.379100	42.796800	554.719300	.028794
-44.128160	42.534760	554.713900	.007545
-42.892960	42.260480	554.712700	.001751
-41.673610	41.974410	554.712800	.000540
-92.917770	43.067330	558.272900	6.418229
-91.673000	43.354680	545.294900	8.788669
-90.411740	43.629630	493.983900	25.369920
-89.134110	43.891690	337.063900	76.578780
-87.840450	44.140390	334.337200	139.450700
-86.531140	44.375260	100.107200	159.353400
-85.206750	44.595830	1136.500000	152.343200
-83.867900	44.801650	2362.048000	220.444100
-82.515380	44.992280	2982.925000	292.221700
-81.150000	45.167310	3325.692000	317.960400
-79.772730	45.326340	3369.078000	295.609900
-78.384560	45.469020	3067.298000	242.240200
-76.986600	45.595010	2388.294000	150.896000
-75.579970	45.704020	1580.051000	71.680000
-74.165890	45.795780	862.609700	142.653700
-72.745610	45.870080	140.212800	197.749800
-71.320400	45.926740	450.835800	241.283500
-69.891600	45.965610	1681.787000	147.880100
-68.460530	45.986610	2160.325000	280.725500
-67.028560	45.989670	1244.098000	164.290000
-65.597030	45.974800	800.391700	69.866380
-64.167300	45.942020	639.096800	48.602990
-62.740720	45.891430	417.891200	54.549080

-61.318600 45.823140 407.071700 51.800260
-59.902260 45.737310 553.631900 39.705060
-58.492950 45.634170 602.376600 22.307990
-57.091900 45.513950 584.331500 22.308160
-55.700260 45.376930 578.590200 20.087170
-54.319140 45.223450 585.902300 17.001290
-52.949590 45.053840 576.801300 12.810830
-51.592580 44.868490 564.885500 7.530468
-50.248970 44.667810 558.527400 3.680458
-48.919560 44.452230 555.921700 1.571952
-47.605040 44.222190 555.038900 .590894
-46.305980 43.978160 554.790200 .196893
-45.022840 43.720610 554.729300 .057975
-43.755960 43.450020 554.715800 .015233
-42.505550 43.166850 554.713100 .003615
-41.271660 42.871580 554.712900 .001046
-93.329110 43.967430 553.004500 5.813464
-92.068780 44.264030 549.324900 10.066010
-90.791210 44.547890 514.779000 27.602150
-89.496510 44.818510 392.527000 71.263790
-88.184940 45.075390 386.380400 150.372000
-86.856880 45.318050 118.143200 131.050900
-85.512890 45.545990 997.024300 93.616400
-84.153590 45.758730 2038.797000 179.818400
-82.779790 45.955820 2347.454000 201.883100
-81.392350 46.136820 2225.567000 196.969400
-79.992260 46.301310 1990.739000 167.216800
-78.580580 46.448910 1758.196000 93.304470
-77.158460 46.579270 1503.893000 78.938420
-75.727110 46.692070 1410.393000 75.228060
-74.287820 46.787040 1445.294000 178.341400
-72.841890 46.863950 1012.213000 119.853500
-71.390720 46.922590 1017.388000 120.575700
-69.935700 46.962830 1951.435000 273.948500
-68.478260 46.984570 1847.864000 142.701600
-67.019850 46.987740 1113.554000 74.001810
-65.561920 46.972340 695.354400 96.325360
-64.105920 46.938410 674.811100 37.743810
-62.653260 46.886040 705.126200 60.774790
-61.205390 46.815350 597.470600 36.991250
-59.763680 46.726520 589.688200 29.044370
-58.329470 46.619790 630.532800 24.619730
-56.904060 46.495390 640.527300 32.693560
-55.488670 46.353640 653.075000 37.798690
-54.084500 46.194880 659.547900 36.382690
-52.692630 46.019470 620.238600 26.953700
-51.314070 45.827840 581.158900 14.927960
-49.949730 45.620390 563.447200 6.741539
-48.600430 45.397590 557.336000 2.716566
-47.266860 45.159900 555.402700 .991689
-45.949600 44.907820 554.871700 .323932
-44.649100 44.641840 554.745700 .094957
-43.365670 44.362450 554.718900 .024857
-42.099450 44.070140 554.713700 .005917
-40.850440 43.765410 554.713000 .001699
-93.760410 44.864160 559.080500 8.847371
-92.483920 45.170280 561.599400 11.757310
-91.189420 45.463320 545.539000 19.695510
-89.876960 45.742750 413.254100 28.648560
-88.546760 46.008070 204.390500 76.424600
-87.199170 46.258760 321.962500 100.646200
-85.834720 46.494300 913.446400 60.906150
-84.454070 46.714200 1524.549000 102.021400
-83.058010 46.917960 1641.858000 109.161100
-81.647480 47.105130 1346.028000 129.363900
-80.223460 47.275260 990.144800 96.895500
-78.787090 47.427960 471.960800 143.357100

-77.339580	47.562840	239.454000	238.201000
-75.882230	47.679570	905.722600	98.397930
-74.416370	47.777870	1522.390000	122.845600
-72.943440	47.857470	1615.221000	154.416700
-71.464880	47.918190	1568.307000	104.146600
-69.982220	47.959850	1637.273000	99.014080
-68.496960	47.982350	1286.069000	102.107000
-67.010670	47.985630	836.425500	95.082290
-65.524890	47.969690	672.280000	94.998100
-64.041170	47.934570	661.342600	101.154000
-62.561040	47.880340	818.297900	56.602040
-61.086020	47.807170	836.817600	47.937350
-59.617590	47.715230	760.373000	64.614850
-58.157170	47.604770	788.239300	48.077210
-56.706150	47.476050	797.331800	57.313530
-55.265830	47.329400	817.211400	61.990150
-53.837470	47.165180	810.497600	55.830020
-52.422200	46.983780	704.547700	40.765560
-51.021090	46.785640	609.198400	23.188000
-49.635090	46.571200	570.491900	10.040480
-48.265020	46.340950	558.936300	3.818613
-46.911610	46.095370	555.779100	1.338073
-45.575410	45.834990	554.954000	.428600
-44.256860	45.560320	554.761700	.123596
-42.956210	45.271870	554.721700	.032198
-41.673580	44.970160	554.714100	.007617
-40.408870	44.655690	554.713100	.002182
-94.212860	45.757310	573.944700	16.084140
-92.919560	46.073230	585.540000	17.936480
-91.607470	46.375730	581.502800	20.233840
-90.276550	46.664250	487.844800	20.278920
-88.926960	46.938270	413.897500	52.190650
-87.559010	47.197250	578.425800	57.910350
-86.173240	47.440650	805.515100	35.655780
-84.770290	47.667940	989.629500	76.532470
-83.350960	47.878600	1161.770000	117.641400
-81.916200	48.072160	1227.489000	66.028310
-80.467090	48.248140	937.970900	51.916370
-79.004780	48.406110	301.761100	214.463700
-77.530590	48.545680	500.648300	100.118600
-76.045850	48.666500	1057.134000	111.901500
-74.552020	48.768250	1380.424000	104.096700
-73.050600	48.850660	1372.977000	138.151400
-71.543170	48.913520	1388.239000	111.976300
-70.031330	48.956650	1397.429000	87.668160
-68.516710	48.979950	1043.290000	65.501820
-67.000980	48.983350	669.498300	116.624000
-65.485790	48.966840	738.799100	82.368930
-63.972820	48.930470	839.560300	90.946360
-62.463700	48.874330	1059.798000	111.958100
-60.960050	48.798580	1133.574000	84.761280
-59.463460	48.703410	1080.041000	74.172510
-57.975440	48.589080	991.177500	70.388670
-56.497490	48.455880	931.250900	77.580490
-55.030970	48.304140	926.251900	74.698620
-53.577210	48.134270	896.961800	74.146610
-52.137430	47.946660	738.365700	45.337760
-50.712720	47.741790	619.411100	25.335780
-49.304080	47.520120	572.753300	10.840150
-47.912350	47.282170	559.304900	4.077207
-46.538250	47.028450	555.813500	1.404501
-45.182340	46.759510	554.955700	.441923
-43.845020	46.475880	554.761700	.126118
-42.526500	46.178100	554.721600	.032420
-41.226800	45.866700	554.714100	.007595
-39.945740	45.542200	554.713100	.002160
-94.687740	46.646660	600.229200	28.696290

-93.376940	46.972690	624.641600	30.815380
-92.046550	47.284930	640.900300	31.943260
-90.696440	47.582840	615.355200	23.977920
-89.326680	47.865840	582.156700	26.485300
-87.937550	48.133390	644.228800	35.815990
-86.529520	48.384910	694.474100	89.866870
-85.103260	48.619850	875.971300	103.156100
-83.659580	48.837660	1314.464000	82.193900
-82.199460	49.037830	1555.887000	135.258400
-80.724000	49.219880	1538.864000	170.069700
-79.234450	49.383340	1202.568000	144.468400
-77.732160	49.527770	1054.325000	82.040480
-76.218590	49.652830	1044.431000	116.602600
-74.695260	49.758160	1204.555000	102.846200
-73.163800	49.843490	1202.935000	120.220400
-71.625870	49.908580	1232.737000	112.602900
-70.083210	49.953250	1263.236000	92.236720
-68.537570	49.977380	1113.886000	63.930490
-66.990740	49.980900	858.202300	56.704850
-65.444490	49.963800	648.562600	77.762230
-63.900620	49.926140	509.026100	112.306300
-62.360880	49.868010	710.787000	56.793940
-60.827010	49.789570	1014.893000	70.935510
-59.300730	49.691040	1136.681000	84.880620
-57.783640	49.572690	1076.246000	78.990390
-56.277320	49.434830	939.897100	78.569990
-54.783270	49.277820	851.803600	70.293480
-53.302860	49.102080	746.681200	61.090510
-51.837360	48.908040	636.598700	43.002750
-50.387950	48.696200	581.473300	20.004110
-48.955640	48.467040	563.403700	8.228582
-47.541310	48.221130	557.378400	3.174992
-46.145660	47.959000	555.395300	1.122988
-44.769230	47.681210	554.862900	.355470
-43.412380	47.388330	554.742800	.100383
-42.075270	47.080920	554.718400	.025535
-40.757810	46.759540	554.713600	.005882
-39.459720	46.424710	554.713000	.001645
-95.186440	47.531940	643.912700	46.459890
-93.857410	47.868410	686.296400	50.187800
-92.508000	48.190720	729.458500	57.463890
-91.137910	48.498310	765.535400	60.075790
-89.747150	48.790600	825.311100	55.751200
-88.335950	49.067010	924.580600	50.610450
-86.904710	49.326930	998.626100	50.789890
-85.454100	49.569790	1121.894000	64.087260
-83.984930	49.795020	1348.901000	107.940000
-82.498220	50.002060	1424.110000	128.188100
-80.995120	50.190400	1351.826000	127.389300
-79.476920	50.359550	1134.950000	129.815600
-77.945060	50.509060	1096.373000	84.871600
-76.401090	50.638520	1136.349000	83.985100
-74.846650	50.747580	1288.210000	101.039600
-73.283450	50.835950	1243.568000	105.197500
-71.713320	50.903350	1226.282000	96.254030
-70.138080	50.949630	1230.710000	85.967980
-68.559640	50.974620	1130.957000	78.978110
-66.979900	50.978270	1029.389000	61.361630
-65.400800	50.960560	924.543600	90.912280
-63.824260	50.921550	556.455000	74.874920
-62.252170	50.861340	391.414500	148.169800
-60.686400	50.780110	711.978200	58.046330
-59.128770	50.678080	938.759000	68.314900
-57.581030	50.555550	944.539200	75.441380
-56.044860	50.412850	845.007100	70.017660
-54.521820	50.250360	733.113300	61.275880
-53.013420	50.068520	647.449000	44.725650

-51.520960	49.867810	585.406200	28.526610
-50.045680	49.648740	564.512500	13.266400
-48.588630	49.411830	558.345000	5.517478
-47.150700	49.157670	555.907000	2.156639
-45.732590	48.886830	555.019800	.765096
-44.334810	48.599900	554.780100	.239571
-42.957660	48.297460	554.726600	.066944
-41.601190	47.980120	554.715200	.016668
-40.265250	47.648430	554.713000	.003714
-38.949390	47.302930	554.712700	.001001
-95.710510	48.412890	718.582600	67.513230
-94.362470	48.760130	804.464500	72.307450
-92.993230	49.092850	892.364400	82.351920
-91.602360	49.410460	972.834000	81.373120
-90.189750	49.712350	1054.121000	80.990960
-88.755540	49.997940	1082.067000	83.853260
-87.300090	50.266570	1044.597000	74.665300
-85.824040	50.517650	1000.318000	80.529120
-84.328210	50.750570	1015.452000	104.980500
-82.813610	50.964740	1047.617000	124.030100
-81.281460	51.159630	1094.957000	112.700300
-79.733130	51.334700	1037.236000	105.585100
-78.170130	51.489480	1053.921000	88.675220
-76.594090	51.623530	1143.087000	88.731740
-75.006800	51.736490	1262.268000	106.560400
-73.410070	51.828010	1177.828000	113.035800
-71.805870	51.897850	1148.817000	97.471030
-70.196170	51.945790	1167.247000	74.312470
-68.582990	51.971680	1065.144000	62.341690
-66.968440	51.975460	922.769300	66.771790
-65.354560	51.957120	937.479100	56.076190
-63.743440	51.916690	863.932600	59.107430
-62.137130	51.854320	716.043500	46.215920
-60.537630	51.770170	769.021100	58.313060
-58.946900	51.664510	853.316200	67.780300
-57.366820	51.537620	836.070600	68.509230
-55.799180	51.389870	764.473300	58.167600
-54.245650	51.221680	695.712600	43.010740
-52.707820	51.033510	640.701200	28.750410
-51.187080	50.825860	595.602100	16.836200
-49.684720	50.599280	568.848400	8.530934
-48.201800	50.354330	558.669100	3.762172
-46.739240	50.091630	555.655300	1.452238
-45.297720	49.811770	554.921900	.495345
-43.877700	49.515370	554.757900	.151695
-42.479410	49.203060	554.721700	.041370
-41.102810	48.875440	554.714200	.010092
-39.747590	48.533100	554.712800	.002174
-38.413150	48.176570	554.712800	.000592
-96.261670	49.289190	824.553300	79.001050
-94.893750	49.647600	981.644700	75.908660
-93.503820	49.991080	1090.320000	90.877000
-92.091320	50.319060	1091.147000	90.921280
-90.655950	50.630910	1015.078000	80.396350
-89.197760	50.925990	919.871700	81.475520
-87.717060	51.203660	844.191600	76.024100
-86.214420	51.463270	791.855000	71.632920
-84.690660	51.704190	782.182200	76.404930
-83.146820	51.925780	842.152700	84.960680
-81.584160	52.127480	996.437200	80.262920
-80.004110	52.308710	1020.952000	79.317110
-78.408280	52.468990	975.795500	93.107540
-76.798410	52.607840	1053.510000	96.118370
-75.176390	52.724850	1185.223000	102.456900
-73.544200	52.819690	1116.401000	97.028460
-71.903920	52.892050	1082.040000	82.938940
-70.257710	52.941730	1110.037000	62.232270

-68.607750	52.968570	1020.301000	42.369590
-66.956280	52.972490	810.765100	57.782670
-65.305560	52.953480	839.055500	50.939450
-63.657820	52.911580	919.774700	59.612150
-62.015270	52.846950	891.729000	63.761010
-60.380080	52.759750	847.738900	65.218050
-58.754360	52.650280	816.399000	62.042540
-57.140120	52.518850	751.956500	56.032990
-55.539290	52.365840	688.625600	44.955880
-53.953660	52.191710	638.961400	32.521070
-52.384890	51.996940	602.666500	21.340850
-50.834480	51.782070	577.210200	12.032290
-49.303730	51.547700	562.758100	5.822144
-47.793770	51.294400	557.031900	2.438467
-46.305490	51.022820	555.319000	.907783
-44.839570	50.733620	554.860800	.306016
-43.396390	50.427420	554.745500	.092156
-41.976090	50.104880	554.719000	.025020
-40.578500	49.766630	554.713700	.006035
-39.203150	49.413260	554.712700	.001272
-37.849210	49.045330	554.712800	.000365
-96.841810	50.160530	839.380600	69.063080
-95.453030	50.530490	978.242200	82.251030
-94.041510	50.885130	964.978800	77.051450
-92.606450	51.223850	889.431600	83.495000
-91.147380	51.546020	806.938400	77.129300
-89.664200	51.850960	734.468900	67.719240
-88.157130	52.138020	686.284900	56.799390
-86.626690	52.406500	657.104900	48.106120
-85.073680	52.655720	649.033300	44.494370
-83.499180	52.885050	683.036600	53.615270
-81.904440	53.093850	780.276800	67.511860
-80.290990	53.281520	881.187700	70.536270
-78.660530	53.447530	869.427900	78.313700
-77.014920	53.591380	969.915500	83.020550
-75.356160	53.712650	1114.505000	95.172890
-73.686420	53.810940	1043.822000	91.085720
-72.007930	53.885960	995.564900	76.030140
-70.322990	53.937460	1014.764000	50.863970
-68.634010	53.965280	923.657700	37.876130
-66.943400	53.969350	730.563700	72.887230
-65.253590	53.949630	779.416900	38.428720
-63.567000	53.906200	899.921700	56.427310
-61.886040	53.839190	887.240700	65.154790
-60.213040	53.748820	799.086500	57.968490
-58.550290	53.635360	720.148400	50.284030
-56.899960	53.499180	663.825900	41.504240
-55.264110	53.340690	625.446700	32.587940
-53.644650	53.160350	600.263500	21.797060
-52.043340	52.958710	578.212400	13.555410
-50.461760	52.736320	564.857400	7.176598
-48.901280	52.493820	558.410900	3.338749
-47.363030	52.231850	555.903400	1.382471
-45.847900	51.951060	555.053200	.518845
-44.356530	51.652150	554.798200	.174216
-42.889220	51.335790	554.731600	.052890
-41.445980	51.002650	554.716300	.014326
-40.026490	50.653380	554.713100	.003431
-38.630060	50.288590	554.712600	.000717
-37.255620	49.908830	554.712700	.000248
-97.453060	51.026530	709.848800	57.479590
-96.042340	51.408490	756.226000	57.835090
-94.608220	51.774700	753.021700	61.694030
-93.149600	52.124570	729.519300	59.111030
-91.665800	52.457450	697.473400	54.328200
-90.156560	52.772640	660.969500	48.089240
-88.621980	53.069450	629.737400	39.821020

-87.062480	53.347160	599.145000	30.172770
-85.478850	53.605050	550.722000	27.502200
-83.872130	53.842430	446.476800	49.843070
-82.243660	54.058630	320.863300	122.006000
-80.595020	54.253030	506.406200	59.896490
-78.928000	54.425040	710.807700	51.801360
-77.244600	54.574130	891.157300	65.220750
-75.546970	54.699840	1056.244000	88.255320
-73.837420	54.801750	992.375200	82.927060
-72.118360	54.879540	922.090400	65.141490
-70.392330	54.932960	883.944000	38.154670
-68.661900	54.961820	762.775300	42.994300
-66.929700	54.966030	675.684800	78.928340
-65.198370	54.945580	719.214400	48.031540
-63.470540	54.900540	758.354700	57.461490
-61.748820	54.831050	747.095400	53.304370
-60.035740	54.737340	671.757000	42.803960
-58.333780	54.619720	617.857500	28.164640
-56.645260	54.478570	600.124000	19.025890
-54.972420	54.314330	593.837500	17.285970
-53.317280	54.127510	584.444000	13.071910
-51.681740	53.918690	570.755800	8.573438
-50.067420	53.688470	560.940900	4.332482
-48.475770	53.437500	556.853500	1.867952
-46.907930	53.166490	555.397500	.750298
-45.364770	52.876130	554.905800	.274994
-43.846840	52.567130	554.760000	.092005
-42.354370	52.240220	554.722900	.027555
-40.887210	51.896080	554.714500	.007346
-39.444820	51.535370	554.712800	.001747
-38.026250	51.158730	554.712600	.000391
-36.630120	50.766700	554.712700	.000202
-98.097820	51.886770	617.349900	33.951910
-96.663910	52.281200	627.256300	34.980410
-95.206040	52.659450	627.753800	36.138340
-93.722790	53.020900	628.358000	36.045820
-92.213200	53.364910	633.014200	35.875290
-90.676770	53.690770	638.750600	36.727780
-89.113440	53.997730	632.177100	35.055300
-87.523560	54.285050	607.673600	27.546500
-85.907830	54.551970	558.771200	20.517450
-84.267300	54.797760	443.413200	41.376480
-82.603340	55.021710	222.509400	87.424400
-80.917590	55.223140	98.975310	110.835300
-79.211960	55.401440	431.683100	68.522710
-77.488560	55.556020	796.898700	51.568420
-75.749730	55.686380	999.237200	78.621170
-73.997920	55.792100	945.803800	76.086370
-72.235800	55.872810	860.024800	49.069520
-70.466090	55.928230	746.864000	36.899190
-68.691570	55.958180	595.637900	91.931450
-66.915130	55.962550	697.418000	53.658440
-65.139640	55.941330	735.114200	49.082900
-63.367970	55.894590	669.773200	56.099530
-61.602940	55.822490	638.962600	40.542270
-59.847310	55.725280	592.201500	27.739660
-58.103760	55.603290	560.070100	17.627720
-56.374820	55.456930	548.347600	12.684500
-54.662870	55.286670	554.261800	8.100394
-52.970100	55.093080	567.329400	6.125754
-51.298500	54.876750	568.079000	6.270138
-49.649800	54.638340	560.526900	3.460192
-48.025460	54.378550	556.591100	1.373092
-46.426660	54.098120	555.292100	.493895
-44.854210	53.797780	554.874800	.168212
-43.308580	53.478290	554.751200	.052341
-41.789850	53.140400	554.720500	.014600

-40.297680	52.784830	554.713900	.003602
-38.831280	52.412250	554.712600	.000808
-37.389370	52.023280	554.712500	.000216
-35.970190	51.618480	554.712700	.000195
-98.778840	52.740810	577.686300	16.944700
-97.320270	53.148240	578.065900	16.987220
-95.837370	53.539010	578.573700	17.474750
-94.328290	53.912530	579.785300	18.427300
-92.791720	54.268120	584.482200	19.985260
-91.226900	54.605080	593.476700	22.435000
-89.633560	54.922620	604.816100	24.944930
-88.011890	55.219970	615.093800	27.704610
-86.362530	55.496330	605.404400	28.580730
-84.686490	55.750910	549.593600	30.533590
-82.985150	55.982970	402.134400	60.201520
-81.260250	56.191770	113.265400	106.896600
-79.513790	56.376650	185.118800	120.441300
-77.748020	56.536990	711.697600	55.267450
-75.965460	56.672260	939.382500	73.869080
-74.168790	56.781970	903.923300	66.191140
-72.360860	56.865740	812.840600	38.380950
-70.544630	56.923280	666.364700	49.369740
-68.723180	56.954370	600.862500	74.292880
-66.899620	56.958910	767.736500	54.174410
-65.077100	56.936880	728.982200	48.285980
-63.258730	56.888360	630.733200	42.446580
-61.447630	56.813510	566.580800	36.107090
-59.646790	56.712620	567.554600	18.605030
-57.859090	56.586040	555.253600	8.735538
-56.087300	56.434210	548.563100	5.787195
-54.333970	56.257640	552.203700	3.344295
-52.601470	56.056940	561.927700	4.168852
-50.891880	55.832740	568.129300	6.713400
-49.207030	55.585770	561.663000	4.248298
-47.548420	55.316760	557.077100	1.706614
-45.917200	55.026500	555.440700	.576780
-44.314130	54.715760	554.926000	.186755
-42.739580	54.385350	554.765100	.055181
-41.193420	54.036030	554.723300	.014416
-39.675070	53.668560	554.714400	.003203
-38.183400	53.283620	554.712700	.000590
-36.716770	52.881820	554.712500	.000199
-35.272900	52.463700	554.712700	.000196
-99.499240	53.588130	562.172100	7.606680
-98.014280	54.009130	561.732400	7.326969
-96.504860	54.412960	561.827800	7.528794
-94.968610	54.799050	562.272200	8.076630
-93.403790	55.166730	563.924300	9.159599
-91.809290	55.515250	567.515600	10.988170
-90.184560	55.843850	573.961700	13.558080
-88.529620	56.151670	583.614900	16.742700
-86.844990	56.437890	592.533800	20.200690
-85.131650	56.701690	596.943800	26.777060
-83.390960	56.942230	566.526400	46.391590
-81.624720	57.158780	411.167500	61.163160
-79.835050	57.350580	317.162600	98.179790
-78.024370	57.516980	708.611800	71.294620
-76.195360	57.657400	870.512000	77.348820
-74.350940	57.771310	866.836800	55.718820
-72.494220	57.858320	781.680800	42.782050
-70.628410	57.918090	666.042600	56.663570
-68.756890	57.950390	689.000900	43.819560
-66.883060	57.955100	784.299600	47.211810
-65.010370	57.932220	704.539000	46.506280
-63.142240	57.881810	617.990800	31.854560
-61.282040	57.804080	562.095900	19.096870
-59.433060	57.699310	546.697200	11.014940

-57.598450 57.567890 542.036900 7.823366
-55.781200 57.410300 544.943400 5.287215
-53.984060 57.227100 551.225500 2.154281
-52.209560 57.018940 558.768900 2.502764
-50.459930 56.786500 567.036400 6.413631
-48.737060 56.530560 564.845200 5.544647
-47.042490 56.251900 558.667100 2.571728
-45.377330 55.951360 555.887800 .877443
-43.742270 55.629770 555.042800 .274718
-42.137470 55.287960 554.795700 .080332
-40.562610 54.926740 554.730200 .021292
-39.016750 54.546860 554.715600 .004915
-37.498410 54.149020 554.713000 .000942
-36.005400 53.733830 554.712600 .000258
-34.534880 53.301770 554.712700 .000202
-100.262700 54.428120 556.814500 3.051019
-98.749250 54.863350 556.577500 2.852366
-97.211560 55.280850 556.612900 2.907212
-95.646590 55.680060 556.835100 3.192077
-94.052090 56.060360 557.449200 3.760653
-92.426510 56.420970 558.670700 4.711029
-90.768940 56.761100 560.879600 6.146031
-89.079190 57.079890 564.488200 8.271054
-87.357570 57.376450 569.752700 11.346510
-85.605000 57.649890 578.616100 16.761850
-83.822840 57.899360 585.744500 25.783740
-82.012920 58.124030 550.225200 35.919030
-80.177480 58.323120 543.356400 50.917340
-78.319110 58.495910 751.491300 80.429750
-76.440710 58.641750 800.181600 77.155830
-74.545430 58.760110 821.060900 58.361650
-72.636660 58.850530 765.658800 51.248600
-70.717930 58.912650 726.854100 41.216490
-68.792920 58.946230 743.501200 40.406600
-66.865380 58.951130 765.591900 46.472710
-64.939080 58.927340 678.352600 42.483900
-63.017780 58.874950 604.623100 26.452330
-61.105210 58.794160 563.674400 12.306940
-59.204930 58.685300 547.850900 6.682552
-57.320400 58.548780 540.444000 7.261620
-55.454840 58.385130 539.700700 7.485654
-53.611270 58.194950 546.005100 4.853263
-51.792370 57.978940 553.250800 .808676
-50.000500 57.737840 562.841900 4.337296
-48.237630 57.472490 567.725300 6.764489
-46.505310 57.183730 560.395300 3.436294
-44.804610 56.872430 556.368300 1.185887
-43.136050 56.539500 555.162600 .361576
-41.499610 56.185780 554.829700 .106122
-39.894630 55.812120 554.738500 .028671
-38.319800 55.419290 554.717200 .006894
-36.773090 55.007980 554.713300 .001418
-35.251730 54.578750 554.712600 .000312
-33.752120 54.132060 554.712700 .000195
-101.073700 55.260130 555.237500 1.097342
-99.529010 55.710320 555.158300 .989912
-97.960900 56.142140 555.169800 1.010452
-96.365430 56.555100 555.258100 1.144585
-94.739640 56.948590 555.483000 1.424225
-93.081440 57.321860 555.933000 1.911166
-91.389490 57.674070 556.756800 2.695558
-89.663250 58.004340 558.166800 3.943091
-87.902820 58.311730 560.719800 6.183526
-86.109000 58.595320 566.253300 10.902870
-84.283100 58.854170 572.771400 19.131490
-82.427000 59.087400 562.232100 27.383630
-80.543050 59.294160 615.686800 43.359200

-78.634010	59.473690	755.761100	65.450970
-76.703000	59.625280	673.779300	68.696340
-74.753460	59.748340	694.908900	62.942650
-72.789070	59.842370	743.067300	48.418080
-70.813740	59.906970	747.187100	38.197110
-68.831480	59.941910	772.029800	42.794120
-66.846440	59.947010	751.650400	46.649490
-64.862770	59.922250	661.794400	38.350110
-62.884610	59.867760	595.069100	22.344940
-60.916040	59.783740	563.738200	9.806715
-58.961010	59.670550	551.040300	4.008391
-57.023270	59.528640	546.249800	4.475256
-55.106350	59.358580	543.708700	5.901969
-53.213500	59.161040	541.541700	6.820132
-51.347600	58.936760	545.686500	4.706273
-49.511150	58.686560	555.074800	.342735
-47.706190	58.411320	565.241300	5.588522
-45.934240	58.111950	560.383400	3.434055
-44.196280	57.789400	556.479200	1.280891
-42.492650	57.444580	555.206200	.392139
-40.823030	57.078400	554.850200	.119842
-39.186360	56.691730	554.745200	.033398
-37.580830	56.285340	554.718900	.008299
-36.003750	55.859910	554.713400	.001785
-34.451600	55.415950	554.712600	.000364
-32.919830	54.953830	554.712700	.000192
-101.937200	56.083360	554.833600	.352922
-100.358100	56.549350	554.811300	.308264
-98.756940	56.996240	554.814900	.316005
-97.128790	57.423640	554.843500	.375201
-95.469860	57.830970	554.916700	.506246
-93.777320	58.217490	555.079300	.756277
-92.049300	58.582370	555.428600	1.211973
-90.284800	58.924680	556.172700	2.062833
-88.483640	59.243460	557.948700	3.915129
-86.646400	59.537720	562.382300	8.602348
-84.774360	59.806450	566.085800	17.393880
-82.869400	60.048700	551.962000	24.411680
-80.933970	60.263580	614.393600	32.503310
-78.971000	60.450230	695.690200	57.405340
-76.983890	60.607900	473.832000	113.876800
-74.976370	60.735940	494.665600	71.151560
-72.952470	60.833800	580.015400	60.691300
-70.916480	60.901050	697.057800	40.542970
-68.872850	60.937420	777.859900	43.982890
-66.826130	60.942730	742.576200	46.595800
-64.780930	60.916960	645.534500	35.354040
-62.741830	60.860230	588.612700	19.556500
-60.713310	60.772790	565.647400	8.826102
-58.699720	60.654990	555.919100	3.304817
-56.705200	60.507380	552.107500	1.941813
-54.733590	60.330550	550.341700	2.887680
-52.788400	60.125220	547.556500	4.350253
-50.872730	59.892220	544.777600	5.223587
-48.989200	59.632420	547.440900	4.101026
-47.139900	59.346780	558.253700	2.158777
-45.326320	59.036270	559.572900	2.590417
-43.549270	58.701890	556.733000	1.282486
-41.808860	58.344600	555.276900	.421419
-40.104370	57.965370	554.861100	.123261
-38.434220	57.565060	554.749100	.034766
-36.795950	57.144450	554.720000	.008787
-35.186060	56.704150	554.713700	.001952
-33.600030	56.244660	554.712700	.000396
-32.032190	55.766200	554.712700	.000203
-102.859900	56.896880	554.738800	.102452
-101.242000	57.379640	554.733200	.086484

-99.604390	57.842470	554.734400	.089770
-97.940930	58.285070	554.743300	.114558
-96.246660	58.706940	554.768400	.176053
-94.517820	59.107400	554.835400	.311911
-92.751850	59.485580	555.012900	.605149
-90.947170	59.840560	555.470700	1.250396
-89.103220	60.171320	556.772800	2.882215
-87.220290	60.476800	560.156400	7.487103
-85.299550	60.755960	560.970600	16.694110
-83.342830	61.007760	539.173700	24.140380
-81.352710	61.231220	583.287000	18.444560
-79.332320	61.425420	608.350300	40.846380
-77.285290	61.589540	400.357400	81.566870
-75.215710	61.722870	263.039900	136.949500
-73.128000	61.824790	276.757400	146.879100
-71.026890	61.894870	518.591200	77.334210
-68.917300	61.932760	745.214100	52.787990
-66.804310	61.938290	729.452000	44.481550
-64.692990	61.911450	633.235300	31.778810
-62.588420	61.852340	585.452900	16.909490
-60.495590	61.761240	568.163000	8.634607
-58.419300	61.638580	559.444800	3.777105
-56.364090	61.484900	555.147800	1.357241
-54.334160	61.300890	553.576000	1.088088
-52.333320	61.087320	552.063500	1.915470
-50.364880	60.845090	549.685900	2.995041
-48.431600	60.575160	548.624800	3.282515
-46.535590	60.278550	552.051100	2.163588
-44.678250	59.956310	558.057000	2.143703
-42.860180	59.609490	556.563500	1.051677
-41.081100	59.239120	555.273600	.383969
-39.339830	58.846180	554.846300	.108248
-37.634110	58.431540	554.744700	.029281
-35.960610	57.995940	554.719200	.007573
-34.314830	57.539980	554.713600	.001691
-32.690990	57.064010	554.712700	.000337
-31.082000	56.568130	554.712700	.000190
-103.849600	57.699570	554.718000	.026834
-102.187300	58.200280	554.716800	.021939
-100.509000	58.680030	554.717200	.023756
-98.806840	59.138710	554.720500	.034910
-97.074520	59.575940	554.731300	.066698
-95.307120	59.991050	554.767100	.149325
-93.501110	60.383240	554.876600	.355311
-91.654170	60.751550	555.178300	.866787
-89.765210	61.094930	556.027100	2.313412
-87.834170	61.412270	558.019000	6.802910
-85.861950	61.702450	556.829200	15.850980
-83.850420	61.964360	538.696700	21.617200
-81.802150	62.196920	566.692800	12.503520
-79.720490	62.399150	547.535000	36.259570
-77.609360	62.570130	371.137200	98.169420
-75.473210	62.709080	218.743300	143.565700
-73.316960	62.815350	154.589500	137.290000
-71.145790	62.888430	259.249700	131.937200
-68.965200	62.927950	629.439900	72.944540
-66.780790	62.933720	680.766700	37.157280
-64.598260	62.905720	614.640800	24.202110
-62.423240	62.844070	577.144700	12.900260
-60.261290	62.749080	563.183600	6.410390
-58.117710	62.621230	557.549300	2.836292
-55.997520	62.461110	555.254300	1.039083
-53.905320	62.269450	554.439100	.480355
-51.845240	62.047150	553.901100	.671950
-49.820820	61.795150	553.078800	1.150781
-47.834930	61.514500	552.299000	1.526613
-45.889670	61.206310	552.747100	1.096489

-43.986290	60.871700	555.119500	.462031
-42.125070	60.511780	555.206700	.381964
-40.305270	60.127630	554.890600	.146775
-38.525020	59.720250	554.773900	.051384
-36.781200	59.290500	554.729900	.016624
-35.069400	58.839090	554.716300	.004498
-33.383770	58.366500	554.713200	.001068
-31.716990	57.872960	554.712700	.000292
-30.060130	57.358350	554.712700	.000191
-104.916500	58.490070	554.713700	.006307
-103.202300	59.010120	554.713500	.005072
-101.477600	59.507980	554.713700	.006297
-99.732440	59.983750	554.715200	.012284
-97.958720	60.437220	554.721400	.031301
-96.150010	60.867820	554.744600	.086566
-94.301540	61.274790	554.819900	.234658
-92.410060	61.657170	555.028400	.631087
-90.473700	62.013880	555.550800	1.854331
-88.491920	62.343760	556.685400	5.845431
-86.465330	62.645610	557.536400	13.108790
-84.395630	62.918240	556.109200	15.184390
-82.285480	63.160470	582.182700	17.454090
-80.138370	63.371240	547.774400	26.318470
-77.958560	63.549540	447.756000	66.085530
-75.750930	63.694510	344.867100	112.046600
-73.520860	63.805420	271.423700	124.188500
-71.274150	63.881700	298.607800	95.200260
-69.016910	63.922960	506.007700	46.037200
-66.755400	63.928990	587.351400	23.376220
-64.495980	63.899750	576.848000	13.357910
-62.244980	63.835380	564.280100	7.187367
-60.008560	63.736250	558.306300	3.634304
-57.792640	63.602840	555.903600	1.608533
-55.602750	63.435840	555.004300	.620963
-53.443960	63.236060	554.670000	.244173
-51.320750	63.004450	554.468100	.234710
-49.236890	62.742080	554.254300	.360889
-47.195330	62.450080	554.064900	.478983
-45.198120	62.129630	554.033700	.435567
-43.246250	61.781950	554.632800	.087104
-41.339580	61.408210	554.758200	.061567
-39.476710	61.009530	554.760100	.040036
-37.654880	60.586900	554.733600	.019367
-35.869840	60.141170	554.718700	.006748
-34.115770	59.672970	554.713800	.001998
-32.385130	59.182660	554.712700	.000528
-30.668580	58.670220	554.712800	.000281
-28.954870	58.135290	554.712700	.000192
-106.073800	59.266680	554.713000	.001336
-104.297500	59.807790	554.713000	.001173
-102.518800	60.325180	554.713100	.001994
-100.725000	60.819250	554.713900	.005594
-98.905530	61.289990	554.718000	.017720
-97.052100	61.737010	554.733300	.054010
-95.158340	62.159620	554.783100	.153400
-93.219700	62.556880	554.914900	.425512
-91.233350	62.927700	555.217500	1.305841
-89.198040	63.270870	556.240500	4.201210
-87.113920	63.585110	558.581100	9.032493
-84.982480	63.869130	565.054300	7.444720
-82.806390	64.121680	561.440400	11.968950
-80.589290	64.341560	521.841100	18.917390
-78.335770	64.527670	474.939700	38.158660
-76.051160	64.679080	439.714000	60.494790
-73.741460	64.794950	419.103900	73.237460
-71.413090	64.874680	440.328900	55.501050
-69.072890	64.917820	514.577100	23.404370

-66.727910 64.924110 554.432300 8.919875
-64.385280 64.893550 559.501000 4.630349
-62.052090 64.826280 557.524800 2.971786
-59.735260 64.722690 555.931500 1.629778
-57.441380 64.583340 555.147000 .775789
-55.176610 64.408980 554.836500 .316537
-52.946510 64.200520 554.716600 .119250
-50.755950 63.959000 554.647800 .080017
-48.608900 63.685590 554.585500 .110704
-46.508400 63.381530 554.545000 .134032
-44.456330 63.048090 554.551100 .123423
-42.453320 62.686580 554.652800 .041557
-40.498620 62.298210 554.714100 .008531
-38.589950 61.884150 554.722700 .009718
-36.723370 61.445380 554.717500 .005368
-34.893150 60.982680 554.714100 .002217
-33.091610 60.496560 554.712900 .000724
-31.309040 59.987160 554.712600 .000281
-29.533510 59.454220 554.712700 .000185
-27.750760 58.896990 554.712700 .000183
-107.339200 60.027230 554.712800 .000342
-105.487000 60.591610 554.712800 .000373
-103.643800 61.130270 554.712900 .000929
-101.793500 61.644070 554.713400 .003187
-99.922550 62.133320 554.715700 .010704
-98.020040 62.597820 554.724700 .032762
-96.077510 63.037030 554.750800 .088335
-94.088710 63.450070 554.809900 .223382
-92.049460 63.835860 554.914300 .608692
-89.957580 64.193130 554.866800 1.676894
-87.812570 64.520550 553.750500 2.353317
-85.615560 64.816710 549.670100 5.835642
-83.369120 65.080260 535.757000 11.666330
-81.077050 65.309890 521.371500 15.371040
-78.744270 65.504390 513.534900 23.258080
-76.376630 65.662700 508.934500 30.919060
-73.980770 65.783920 508.378800 33.645420
-71.563890 65.867360 518.069300 26.711550
-69.133670 65.912500 538.087100 12.822640
-66.698060 65.919100 551.434200 4.330954
-64.265090 65.887100 554.901100 1.442801
-61.842790 65.816700 555.299700 .940283
-59.438900 65.708320 555.045800 .628398
-57.060850 65.562580 554.847600 .329290
-54.715470 65.380330 554.756000 .143571
-52.408880 65.162570 554.718800 .054566
-50.146370 64.910470 554.698600 .027332
-47.932120 64.625290 554.680800 .032388
-45.769150 64.308400 554.668900 .038953
-43.659080 63.961160 554.673000 .033187
-41.602020 63.584970 554.694500 .015632
-39.596350 63.181100 554.710000 .002383
-37.638600 62.750720 554.713500 .001381
-35.723270 62.294770 554.713300 .001242
-33.842640 61.813940 554.712900 .000578
-31.986660 61.308540 554.712800 .000273
-30.142740 60.778430 554.712700 .000232
-28.295590 60.222970 554.712700 .000183
-26.427080 59.640870 554.712700 .000183
-108.736700 60.768890 554.712700 .000194
-106.789100 61.359350 554.712800 .000290
-104.866900 61.921510 554.712800 .000750
-102.949500 62.456830 554.713300 .002524
-101.019200 62.966030 554.714900 .008142
-99.061880 63.449270 554.720300 .022792
-97.066180 63.906190 554.730700 .055508
-95.023590 64.336020 554.734700 .121437

-92.928180 64.737730 554.650100 .257424
-90.776390 65.110020 554.247300 .638304
-88.566850 65.451470 552.463800 1.480826
-86.300130 65.760610 547.613800 4.584908
-83.978550 66.035940 539.614100 8.125272
-81.606060 66.276030 535.164400 11.456240
-79.187900 66.479550 534.755500 15.591180
-76.730480 66.645290 534.705000 18.795690
-74.241170 66.772270 535.781900 18.800710
-71.728080 66.859700 540.096300 14.617310
-69.199870 66.907030 546.843600 8.132667
-66.665550 66.913940 551.950400 3.362727
-64.134220 66.880400 554.155000 .968589
-61.614990 66.806620 554.741300 .321373
-59.116610 66.693070 554.797800 .256673
-56.647430 66.540450 554.759700 .162569
-54.215080 66.349720 554.731000 .078434
-51.826330 66.121990 554.717200 .031793
-49.486860 65.858530 554.709500 .013290
-47.201050 65.560780 554.703400 .012153
-44.971820 65.230190 554.699000 .014159
-42.800360 64.868270 554.699800 .012475
-40.685980 64.476460 554.705600 .006663
-38.625890 64.056100 554.710400 .002128
-36.614990 63.608330 554.712400 .000404
-34.645670 63.134020 554.712700 .000300
-32.707610 62.633670 554.712800 .000233
-30.787590 62.107330 554.712800 .000240
-28.869240 61.554470 554.712700 .000186
-26.932890 60.973880 554.712700 .000183
-24.955330 60.363580 554.712700 .000183

Q.26a
-89.453400 32.991660 584.373800 .040179
-88.345930 33.191160 584.384200 .055790
-87.228100 33.381660 584.425400 .118966
-86.100230 33.562890 584.508400 .237017
-84.962690 33.734530 584.650600 .420396
-83.815900 33.896310 584.860500 .662250
-82.660300 34.047950 585.105200 .918330
-81.496410 34.189190 585.294300 1.113698
-80.324730 34.319790 585.340300 1.178192
-79.145840 34.439500 585.229000 1.064637
-77.960310 34.548100 584.937300 .743211
-76.768760 34.645400 584.397600 .288519

-75.571840	34.731200	583.698600	.499689
-74.370180	34.805370	583.411100	.736572
-73.164480	34.867740	583.658000	.646879
-71.955430	34.918200	584.024700	.373758
-70.743730	34.956650	584.266000	.131147
-69.530080	34.983020	584.387200	.032800
-68.315220	34.997260	584.428300	.083279
-67.099880	34.999340	584.420400	.080574
-65.884770	34.989250	584.393500	.052134
-64.670640	34.967020	584.371900	.025017
-63.458190	34.932690	584.361700	.009100
-62.248150	34.886320	584.358300	.002413
-61.041230	34.828000	584.357400	.000658
-59.838120	34.757850	584.357200	.001038
-58.639500	34.676010	584.357100	.001185
-57.446040	34.582610	584.357200	.001069
-56.258390	34.477850	584.357400	.000814
-55.077160	34.361910	584.357600	.000538
-53.902950	34.235010	584.357700	.000313
-52.736340	34.097380	584.357500	.000237
-51.577860	33.949260	584.357500	.000192
-50.428010	33.790910	584.357500	.000181
-49.287250	33.622600	584.357500	.000183
-48.156040	33.444620	584.357500	.000183
-47.034740	33.257250	584.357500	.000183
-45.923690	33.060790	584.357500	.000183
-44.823220	32.855560	584.357500	.000183
-89.701210	33.918400	584.437600	.142936
-88.583520	34.125020	584.491300	.203778
-87.455040	34.322360	584.675700	.408290
-86.316120	34.510110	585.027700	.772524
-85.167100	34.687960	585.603300	1.317429
-84.008420	34.855620	586.419000	2.013145
-82.840550	35.012790	587.352900	2.744700
-81.663990	35.159200	588.084200	3.313280
-80.479300	35.294580	588.288800	3.521543
-79.287040	35.418700	587.885300	3.238252
-78.087840	35.531310	586.815400	2.353982
-76.882320	35.632210	584.815700	1.059209
-75.671150	35.721200	582.126900	1.321734
-74.455030	35.798110	581.039700	1.979145
-73.234660	35.862810	581.948700	1.642244
-72.010770	35.915150	583.202200	.956093
-70.784100	35.955040	584.061300	.315991
-69.555380	35.982390	584.513700	.149952
-68.325390	35.997170	584.667200	.282105
-67.094890	35.999320	584.627800	.262568
-65.864640	35.988860	584.511700	.168377
-64.635410	35.965800	584.420800	.082028
-63.407950	35.930180	584.377300	.032136
-62.183030	35.882080	584.361900	.010158
-60.961370	35.821590	584.357400	.003348
-59.743720	35.748840	584.356000	.003779
-58.530780	35.663950	584.355500	.004215
-57.323260	35.567100	584.356000	.003791
-56.121820	35.458470	584.357100	.002754
-54.927120	35.338260	584.357800	.001845
-53.739770	35.206700	584.357900	.000963
-52.560380	35.064020	584.357700	.000453
-51.389480	34.910500	584.357500	.000250
-50.227610	34.746390	584.357500	.000181
-49.075240	34.571990	584.357500	.000181
-47.932830	34.387580	584.357500	.000183
-46.800740	34.193490	584.357500	.000183
-45.679330	33.990000	584.357500	.000183
-44.568900	33.777440	584.357500	.000183
-89.960660	34.843320	584.789300	.567377

-88.832330	35.057210	585.076500	.812642
-87.692770	35.261540	585.965000	1.542624
-86.542320	35.455950	587.581400	2.794020
-85.381340	35.640140	590.111400	4.590544
-84.210270	35.813810	593.577600	6.840743
-83.029580	35.976620	597.487400	9.180140
-81.839790	36.128310	600.609000	11.042750
-80.641480	36.268600	601.587300	11.797330
-79.435230	36.397220	600.056800	10.982470
-78.221690	36.513940	595.622400	8.205383
-77.001530	36.618520	587.698200	4.108229
-75.775430	36.710780	575.895400	4.466413
-74.544130	36.790520	571.703300	5.559715
-73.308360	36.857590	575.346900	4.678342
-72.068890	36.911860	580.119100	2.654072
-70.826490	36.953220	583.392300	.857356
-69.581950	36.981590	585.244300	.741359
-68.336070	36.996900	585.865000	1.122976
-67.089650	36.999130	585.644500	.997791
-65.843500	36.988290	585.085000	.623287
-64.598400	36.964370	584.663300	.307145
-63.355190	36.927440	584.459900	.126510
-62.114630	36.877570	584.384900	.046312
-60.877510	36.814860	584.360200	.018613
-59.644590	36.739430	584.351500	.016988
-58.416640	36.651430	584.348700	.018320
-57.194370	36.551040	584.350500	.016283
-55.978490	36.438440	584.356000	.011707
-54.769680	36.313860	584.359100	.007454
-53.568590	36.177530	584.359100	.003940
-52.375820	36.029710	584.358300	.001584
-51.191970	35.870660	584.357700	.000576
-50.017550	35.700660	584.357500	.000272
-48.853080	35.520030	584.357500	.000181
-47.698980	35.329070	584.357500	.000183
-46.555660	35.128100	584.357500	.000183
-45.423470	34.917440	584.357500	.000183
-44.302690	34.697410	584.357500	.000183
-90.232250	35.766320	586.170700	1.795342
-89.092860	35.987640	587.391800	2.593327
-87.941790	36.199090	590.836900	4.741852
-86.779330	36.400320	596.851100	8.271190
-85.605870	36.591000	606.001100	13.294090
-84.421870	36.770790	618.170200	19.339730
-83.227800	36.939400	632.034400	25.813110
-82.024180	37.096500	643.503200	31.122480
-80.811620	37.241800	647.360000	33.417530
-79.590740	37.375040	642.295900	31.443930
-78.362190	37.495960	627.140300	24.401080
-77.126670	37.604320	599.718400	12.323370
-75.884930	37.699920	552.938600	13.673330
-74.637700	37.782550	544.800600	14.432380
-73.385770	37.852060	559.122800	9.977033
-72.129940	37.908310	572.301000	5.978974
-70.871030	37.951180	582.612600	2.498717
-69.609860	37.980580	588.868900	3.232261
-68.347290	37.996460	590.814000	4.121785
-67.084140	37.998780	589.665600	3.467898
-65.821270	37.987530	587.227900	2.052312
-64.559530	37.962740	585.545300	.966108
-63.299760	37.924460	584.781800	.414941
-62.042780	37.872770	584.491600	.170086
-60.789440	37.807780	584.385700	.082779
-59.540510	37.729610	584.343400	.070705
-58.296810	37.638420	584.327600	.072006
-57.059080	37.534390	584.332000	.060594
-55.828080	37.417740	584.356800	.046476

-54.604500	37.288680	584.367600	.029504
-53.389020	37.147460	584.363600	.012731
-52.182290	36.994370	584.360400	.005055
-50.984880	36.829660	584.358500	.001546
-49.797380	36.653650	584.357700	.000451
-48.620270	36.466650	584.357500	.000237
-47.454020	36.268990	584.357500	.000180
-46.299010	36.060990	584.357500	.000181
-45.155610	35.843010	584.357500	.000183
-44.024070	35.615370	584.357500	.000183
-90.516530	36.687310	590.308800	4.568113
-89.365660	36.916220	594.450800	6.669703
-88.202590	37.134950	605.458100	12.004950
-87.027650	37.343150	624.097500	20.581590
-85.841200	37.540460	652.032000	32.306070
-84.643710	37.726540	688.165000	46.369140
-83.435650	37.901050	730.975800	61.298960
-82.217600	38.063690	769.276800	74.998630
-80.990140	38.214140	781.552200	80.838020
-79.753940	38.352110	766.838500	77.337210
-78.509670	38.477340	727.581100	60.922890
-77.258070	38.589580	633.379000	29.217490
-75.999910	38.688600	465.455500	35.314490
-74.735980	38.774210	471.259300	31.321600
-73.467090	38.846230	542.356100	29.786200
-72.194090	38.904510	563.159900	12.087850
-70.917830	38.948930	585.468800	7.748177
-69.639210	38.979390	605.698300	12.931570
-68.359090	38.995840	610.289900	14.431070
-67.078360	38.998240	604.845100	11.772610
-65.797930	38.986590	594.229200	6.117418
-64.518690	38.960900	588.160900	2.714172
-63.241520	38.921240	585.772000	1.154523
-61.967320	38.867690	584.889000	.538132
-60.696920	38.800350	584.533300	.326671
-59.431210	38.719360	584.375200	.288298
-58.170970	38.624900	584.301400	.266381
-56.917050	38.517150	584.285400	.226135
-55.670200	38.396340	584.405200	.170820
-54.431160	38.262690	584.420700	.114175
-53.200640	38.116470	584.382900	.043612
-51.979310	37.957960	584.365300	.011642
-50.767770	37.787460	584.359700	.003542
-49.566610	37.605300	584.358000	.000812
-48.376340	37.411790	584.357500	.000252
-47.197410	37.207270	584.357500	.000169
-46.030250	36.992090	584.357500	.000216
-44.875180	36.766620	584.357500	.000227
-43.732470	36.531200	584.357500	.000183
-90.814130	37.606180	599.706800	9.429028
-89.651300	37.842850	611.460000	14.370000
-88.475770	38.069030	642.912200	26.887320
-87.287830	38.284350	693.690500	44.731800
-86.087840	38.488450	767.340800	66.611570
-84.876290	38.680970	864.028100	94.527880
-83.653650	38.861550	979.985400	119.886600
-82.420500	39.029860	1096.098000	143.210100
-81.177470	39.185590	1130.818000	158.497400
-79.925230	39.328410	1098.535000	158.837000
-78.664510	39.458070	1015.026000	131.650000
-77.396060	39.574290	780.001100	69.910220
-76.120680	39.676830	339.593900	102.750800
-74.839220	39.765490	262.559400	64.423160
-73.552520	39.840080	546.879200	95.294910
-72.261480	39.900440	575.145000	45.681480
-70.967010	39.946460	608.184000	27.016030
-69.670030	39.978020	669.141800	39.346490

-68.371470	39.995060	680.634900	44.642760
-67.072270	39.997550	657.333300	34.764430
-65.773400	39.985470	614.020900	15.910620
-64.475770	39.958870	594.848700	6.783209
-63.180340	39.917780	588.433600	3.112571
-61.888020	39.862300	586.242600	1.738454
-60.599740	39.792560	585.374800	1.386860
-59.316390	39.708680	584.975700	1.299545
-58.038840	39.610860	584.620500	1.132261
-56.767930	39.499290	584.332800	.892873
-55.504470	39.374200	584.771700	.686195
-54.249260	39.235840	584.681800	.407907
-53.003000	39.084480	584.451200	.119181
-51.766410	38.920440	584.377700	.028577
-50.540120	38.744010	584.361500	.004651
-49.324710	38.555530	584.358200	.001084
-48.120720	38.355340	584.357500	.000215
-46.928620	38.143810	584.357400	.000146
-45.748810	37.921300	584.357500	.000131
-44.581620	37.688170	584.357500	.000173
-43.427300	37.444810	584.357500	.000183
-91.125630	38.522790	615.866000	16.310600
-89.950410	38.767390	646.153600	27.675620
-88.761920	39.001200	728.605500	51.410020
-87.560450	39.223820	841.147300	75.933490
-86.346370	39.434880	986.342100	102.845500
-85.120160	39.633990	1204.291000	139.042300
-83.882300	39.820790	1459.034000	185.780000
-82.633390	39.994930	1712.336000	226.802300
-81.374080	40.156070	1811.281000	254.016400
-80.105060	40.303890	1788.866000	262.325600
-78.827100	40.438090	1693.038000	246.156000
-77.540980	40.558400	1363.559000	174.883300
-76.247540	40.664560	594.899000	155.384100
-74.947690	40.756360	173.122800	214.473200
-73.642300	40.833600	606.291000	114.313900
-72.332310	40.896110	616.494200	90.772770
-71.018690	40.943760	683.289300	66.739650
-69.702430	40.976440	809.612500	85.345210
-68.384490	40.994090	836.631400	95.427700
-67.065880	40.996670	758.323400	68.546040
-65.747600	40.984170	638.999600	27.253220
-64.430650	40.956610	607.925400	14.179920
-63.116020	40.914060	596.772600	9.269782
-61.804690	40.856610	591.956300	6.430300
-60.497620	40.784390	590.032000	5.665074
-59.195770	40.697540	588.688000	5.544519
-57.900040	40.596260	587.050200	4.814513
-56.611330	40.480750	585.759300	3.257483
-55.330490	40.351270	585.186800	1.369194
-54.058330	40.208070	584.888200	.604064
-52.795620	40.051450	584.558400	.214640
-51.543080	39.881710	584.388600	.029189
-50.301390	39.699200	584.362300	.007343
-49.071130	39.504250	584.357900	.001683
-47.852850	39.297230	584.357500	.000392
-46.647030	39.078520	584.357400	.000169
-45.454070	38.848490	584.357500	.000122
-44.274280	38.607530	584.357500	.000155
-43.107920	38.356030	584.357500	.000183
-91.451740	39.437030	635.642000	23.679820
-90.263630	39.689750	696.930200	41.473000
-89.061680	39.931370	840.242700	79.565780
-87.846150	40.161480	968.796700	107.581200
-86.617400	40.379670	1043.000000	102.187400
-85.375900	40.585540	1332.229000	133.660100
-84.122160	40.778730	1829.927000	213.768800

-82.856800	40.958850	2237.858000	286.666600
-81.580460	41.125550	2480.503000	331.249400
-80.293880	41.278500	2590.537000	367.442700
-78.997860	41.417370	2585.056000	380.221200
-77.693230	41.541890	2309.483000	327.616100
-76.380870	41.651780	1563.399000	182.906800
-75.061690	41.746810	897.725200	100.561400
-73.736660	41.826770	816.063000	108.798500
-72.406780	41.891490	699.775000	84.152760
-71.073040	41.940830	831.598100	82.986700
-69.736500	41.974670	917.523200	115.938500
-68.398190	41.992950	905.038000	109.605800
-67.059160	41.995620	707.852400	52.772790
-65.720480	41.982670	565.111100	19.916730
-64.383220	41.954140	624.224000	24.560510
-63.048400	41.910080	629.873000	28.150090
-61.717090	41.850600	613.637100	19.912550
-60.390290	41.775820	599.497800	14.615400
-59.069010	41.685920	588.312700	11.404870
-57.754210	41.581080	584.652700	8.711285
-56.446830	41.461530	586.742900	4.541062
-55.147770	41.327530	585.109500	1.171418
-53.857880	41.179360	584.095900	.716532
-52.577970	41.017320	584.153700	.416405
-51.308780	40.841740	584.331100	.104038
-50.051000	40.652980	584.357200	.026489
-48.805250	40.451390	584.357600	.005952
-47.572090	40.237360	584.357400	.001310
-46.352010	40.011290	584.357500	.000306
-45.145370	39.773560	584.357400	.000160
-43.952510	39.524580	584.357500	.000149
-42.773630	39.264750	584.357500	.000183
-91.793200	40.348750	641.478600	25.897730
-90.591710	40.609810	708.799100	49.131230
-89.375760	40.859430	837.542200	101.842200
-88.145600	41.097210	781.784500	101.164700
-86.901570	41.322720	446.630500	242.655700
-85.644150	41.535550	602.204200	274.867800
-84.373860	41.735290	1431.376000	75.008710
-83.091290	41.921560	2127.321000	221.637700
-81.797160	42.093980	2632.818000	335.846800
-80.492230	42.252200	3071.766000	434.169600
-79.177290	42.395880	3257.536000	481.325300
-77.853240	42.524730	3022.460000	440.076600
-76.521000	42.638450	2316.821000	274.996700
-75.181550	42.736820	1489.197000	127.506700
-73.835900	42.819590	1011.312000	101.921600
-72.485100	42.886590	699.222900	119.471500
-71.130210	42.937670	886.625900	114.632700
-69.772340	42.972710	899.326500	104.534700
-68.412590	42.991630	733.537400	64.372340
-67.052090	42.994400	362.059200	169.129000
-65.691960	42.980990	348.911700	155.987800
-64.333320	42.951450	657.239500	49.019370
-62.977290	42.905830	700.328700	61.229310
-61.624970	42.844260	637.241000	31.401770
-60.277440	42.766850	579.934500	15.953840
-58.935750	42.673790	555.015300	18.266180
-57.600940	42.565280	563.040600	12.886030
-56.273990	42.441570	578.963000	6.055216
-54.955840	42.302930	584.877100	3.278522
-53.647390	42.149640	584.982700	1.410059
-52.349470	41.982030	584.704100	.521292
-51.062890	41.800450	584.492000	.199475
-49.788320	41.605260	584.393900	.065660
-48.526440	41.396860	584.364200	.016851
-47.277780	41.175640	584.358300	.003286

-46.042850	40.942000	584.357500	.000496
-44.822010	40.696380	584.357500	.000134
-43.615560	40.439180	584.357500	.000163
-42.423690	40.170830	584.357500	.000183
-92.150770	41.257820	636.640300	23.433210
-90.935390	41.527410	709.637900	49.190080
-89.704910	41.785250	868.476800	113.871500
-88.459540	42.030900	882.872200	155.376200
-87.199620	42.263930	565.504800	115.467200
-85.925610	42.483890	548.701400	186.732000
-84.638040	42.690380	1306.390000	79.456180
-83.337520	42.882970	2073.232000	233.300100
-82.024790	43.061280	2791.459000	387.337200
-80.700610	43.224930	3530.530000	529.247400
-79.365870	43.373580	3833.104000	601.430400
-78.021450	43.506890	3620.923000	553.659700
-76.668370	43.624570	2950.054000	413.444800
-75.307630	43.726360	2131.231000	248.759600
-73.940300	43.812040	1352.409000	130.042100
-72.567500	43.881390	602.600500	129.321000
-71.190370	43.934270	849.373400	92.316720
-69.810050	43.970550	1096.067000	161.763900
-68.427750	43.990140	1123.111000	126.941400
-67.044650	43.993000	700.721200	62.656090
-65.661930	43.979120	605.982200	49.081460
-64.280810	43.948540	739.149300	71.094460
-62.902460	43.901310	650.917800	40.846840
-61.528050	43.837570	567.765300	25.612430
-60.158730	43.757450	529.227100	35.788400
-58.795620	43.661140	547.071000	22.746020
-57.439790	43.548850	584.547900	8.210423
-56.092310	43.420850	594.789900	8.291066
-54.754150	43.277410	592.068100	5.047530
-53.426270	43.118850	588.264600	2.800588
-52.109530	42.945500	586.019900	1.420228
-50.804760	42.757750	584.939200	.604485
-49.512690	42.555960	584.516200	.209775
-48.233980	42.340550	584.390600	.058139
-46.969190	42.111940	584.362500	.012353
-45.718810	41.870550	584.358000	.001886
-44.483210	41.616830	584.357500	.000202
-43.262650	41.351200	584.357500	.000155
-42.057300	41.074100	584.357500	.000183
-92.525320	42.164070	608.204700	12.032360
-91.295520	42.442420	635.643700	27.058160
-90.049940	42.708690	706.179500	69.298290
-88.788760	42.962440	702.639300	103.501100
-87.512310	43.203190	531.857400	75.554430
-86.221000	43.430500	577.395900	108.195200
-84.915410	43.643920	1314.482000	128.723400
-83.596150	43.843030	2179.631000	293.242400
-82.263960	44.027400	2970.174000	451.073600
-80.919660	44.196660	3760.547000	608.912300
-79.564150	44.350410	4042.192000	650.839300
-78.198390	44.488330	3699.180000	572.401700
-76.823410	44.610100	2935.619000	401.669900
-75.440290	44.715440	2089.973000	248.000900
-74.050180	44.804110	1206.259000	137.505200
-72.654240	44.875890	293.158800	208.163000
-71.253690	44.930630	747.860800	124.313000
-69.849760	44.968180	1530.055000	262.571900
-68.443720	44.988460	1812.224000	304.762800
-67.036810	44.991420	1231.414000	132.728700
-65.630330	44.977050	861.256100	61.417430
-64.225530	44.945390	677.500200	45.146110
-62.823690	44.896510	469.096800	83.859390
-61.426040	44.830540	451.540200	73.464320

-60.033810	44.747610	542.871400	33.661140
-58.648170	44.647940	611.742100	17.306630
-57.270290	44.531750	632.448400	21.452420
-55.901260	44.399320	616.322000	15.060490
-54.542130	44.250930	604.373400	11.355460
-53.193900	44.086940	596.081800	7.245237
-51.857480	43.907680	589.750600	3.797920
-50.533710	43.713560	586.325700	1.655136
-49.223370	43.504980	584.926600	.595808
-47.927120	43.282370	584.486800	.175574
-46.645540	43.046150	584.380100	.041270
-45.379100	42.796800	584.360300	.007384
-44.128160	42.534760	584.357700	.000937
-42.892960	42.260480	584.357500	.000168
-41.673610	41.974410	584.357500	.000183
-92.917770	43.067330	583.214100	5.375177
-91.673000	43.354680	576.262300	12.497410
-90.411740	43.629630	533.395300	28.061980
-89.134110	43.891690	380.755800	43.168120
-87.840450	44.140390	246.365100	106.488700
-86.531140	44.375260	174.846000	169.951500
-85.206750	44.595830	1125.708000	138.625900
-83.867900	44.801650	2140.939000	322.574700
-82.515380	44.992280	2767.283000	438.807100
-81.150000	45.167310	3150.176000	476.318400
-79.772730	45.326340	3150.230000	452.989800
-78.384560	45.469020	2863.877000	389.982800
-76.986600	45.595010	2264.556000	238.917200
-75.579970	45.704020	1626.616000	117.299400
-74.165890	45.795780	966.630300	157.552600
-72.745610	45.870080	190.785600	242.603200
-71.320400	45.926740	668.708400	177.391000
-69.891600	45.965610	1632.414000	272.859600
-68.460530	45.986610	1966.544000	325.026400
-67.028560	45.989670	1365.579000	154.542800
-65.597030	45.974800	934.362500	83.734390
-64.167300	45.942020	779.840900	59.501530
-62.740720	45.891430	611.476000	34.545770
-61.318600	45.823140	538.290000	52.971810
-59.902260	45.737310	619.066200	21.774320
-58.492950	45.634170	689.898700	34.592730
-57.091900	45.513950	679.116300	36.102610
-55.700260	45.376930	652.824600	32.584960
-54.319140	45.223450	642.278600	27.517110
-52.949590	45.053840	620.491000	18.198400
-51.592580	44.868490	600.276400	9.256505
-50.248970	44.667810	590.033000	3.884829
-48.919560	44.452230	586.023700	1.401311
-47.605040	44.222190	584.752000	.423972
-46.305980	43.978160	584.431700	.105581
-45.022840	43.720610	584.368300	.020967
-43.755960	43.450020	584.358600	.003133
-42.505550	43.166850	584.357500	.000354
-41.271660	42.871580	584.357500	.000185
-93.329110	43.967430	571.678200	6.821796
-92.068780	44.264030	549.497600	15.013230
-90.791210	44.547890	457.249100	28.821820
-89.496510	44.818510	246.027000	126.114800
-88.184940	45.075390	327.815700	133.613600
-86.856880	45.318050	281.624000	141.417800
-85.512890	45.545990	971.483600	105.646300
-84.153590	45.758730	1857.550000	265.960300
-82.779790	45.955820	2121.437000	282.396400
-81.392350	46.136820	1882.896000	178.441600
-79.992260	46.301310	1672.695000	123.620800
-78.580580	46.448910	1596.526000	113.904700
-77.158460	46.579270	1396.763000	80.475230

-75.727110 46.692070 1419.656000 68.993530
-74.287820 46.787040 1453.851000 132.998700
-72.841890 46.863950 1212.933000 127.010100
-71.390720 46.922590 1276.166000 162.974300
-69.935700 46.962830 1836.180000 291.228000
-68.478260 46.984570 1818.543000 250.753900
-67.019850 46.987740 1214.592000 108.967200
-65.561920 46.972340 881.222800 44.429870
-64.105920 46.938410 832.424600 38.907950
-62.653260 46.886040 817.713900 58.679810
-61.205390 46.815350 728.341600 35.058120
-59.763680 46.726520 740.834200 37.946450
-58.329470 46.619790 816.202000 64.236670
-56.904060 46.495390 814.128300 73.964440
-55.488670 46.353640 789.697600 73.976150
-54.084500 46.194880 763.495800 63.805490
-52.692630 46.019470 688.169100 42.080430
-51.314070 45.827840 624.958900 20.158490
-49.949730 45.620390 597.482800 7.701143
-48.600430 45.397590 588.079700 2.643004
-47.266860 45.159900 585.246300 .796034
-45.949600 44.907820 584.530100 .202467
-44.649100 44.641840 584.384200 .042243
-43.365670 44.362450 584.360700 .006979
-42.099450 44.070140 584.357700 .000857
-40.850440 43.765410 584.357500 .000197
-93.760410 44.864160 576.506900 5.854529
-92.483920 45.170280 567.185200 13.426010
-91.189420 45.463320 509.817100 28.493670
-89.876960 45.742750 328.358400 42.770110
-88.546760 46.008070 186.281000 139.486100
-87.199170 46.258760 230.097500 146.988900
-85.834720 46.494300 816.005900 65.297180
-84.454070 46.714200 1286.953000 126.696000
-83.058010 46.917960 1205.385000 89.397750
-81.647480 47.105130 835.581400 191.888400
-80.223460 47.275260 687.753400 208.419000
-78.787090 47.427960 670.144700 177.267300
-77.339580 47.562840 714.840400 235.382200
-75.882230 47.679570 1155.552000 57.565650
-74.416370 47.777870 1622.225000 167.305600
-72.943440 47.857470 1703.352000 194.788200
-71.464880 47.918190 1666.793000 198.752600
-69.982220 47.959850 1719.390000 201.905100
-68.496960 47.982350 1434.844000 145.309000
-67.010670 47.985630 1024.915000 56.997430
-65.524890 47.969690 744.226700 104.425400
-64.041170 47.934570 703.534900 84.095980
-62.561040 47.880340 792.073300 33.777500
-61.086020 47.807170 799.482700 37.061760
-59.617590 47.715230 820.700000 43.696310
-58.157170 47.604770 927.970400 87.083490
-56.706150 47.476050 1017.119000 123.792900
-55.265830 47.329400 1047.061000 134.655100
-53.837470 47.165180 995.390300 120.643300
-52.422200 46.983780 813.305900 75.783760
-51.021090 46.785640 666.598900 34.197820
-49.635090 46.571200 607.312100 12.277870
-48.265020 46.340950 590.335700 3.869880
-46.911610 46.095370 585.749600 1.129657
-45.575410 45.834990 584.627700 .284878
-44.256860 45.560320 584.399800 .060181
-42.956210 45.271870 584.362500 .010170
-41.673580 44.970160 584.358000 .001294
-40.408870 44.655690 584.357500 .000222
-94.212860 45.757310 590.621900 7.298376
-92.919560 46.073230 593.282000 13.480530

-91.607470	46.375730	575.888200	21.774710
-90.276550	46.664250	487.728200	27.756410
-88.926960	46.938270	453.009900	28.557490
-87.559010	47.197250	553.474700	47.290810
-86.173240	47.440650	683.000200	43.070030
-84.770290	47.667940	710.153500	76.566730
-83.350960	47.878600	739.156600	148.792500
-81.916200	48.072160	880.129800	70.069990
-80.467090	48.248140	972.139200	60.609410
-79.004780	48.406110	783.164300	178.321300
-77.530590	48.545680	829.219500	124.837500
-76.045850	48.666500	1197.848000	79.136400
-74.552020	48.768250	1451.757000	144.420300
-73.050600	48.850660	1437.588000	144.427700
-71.543170	48.913520	1420.993000	139.472600
-70.031330	48.956650	1412.782000	150.876000
-68.516710	48.979950	1225.659000	98.730470
-67.000980	48.983350	960.782500	48.609540
-65.485790	48.966840	715.904300	93.674770
-63.972820	48.930470	682.733600	80.411400
-62.463700	48.874330	843.880800	46.615280
-60.960050	48.798580	964.118100	69.950260
-59.463460	48.703410	974.243100	73.072830
-57.975440	48.589080	1023.107000	98.030820
-56.497490	48.455880	1117.090000	143.194200
-55.030970	48.304140	1179.222000	166.008100
-53.577210	48.134270	1107.425000	151.774700
-52.137430	47.946660	866.437500	85.871400
-50.712720	47.741790	681.263400	37.175690
-49.304080	47.520120	609.305900	13.722560
-47.912350	47.282170	590.360200	4.070603
-46.538250	47.028450	585.724900	1.144393
-45.182340	46.759510	584.615500	.287984
-43.845020	46.475880	584.395600	.059036
-42.526500	46.178100	584.361700	.009728
-41.226800	45.866700	584.357700	.001158
-39.945740	45.542200	584.357500	.000229
-94.687740	46.646660	613.723700	17.477380
-93.376940	46.972690	629.269800	24.769330
-92.046550	47.284930	640.025900	30.975730
-90.696440	47.582840	616.721500	27.404780
-89.326680	47.865840	601.983300	31.929410
-87.937550	48.133390	657.602600	37.629460
-86.529520	48.384910	675.901800	60.912560
-85.103260	48.619850	766.735800	70.532290
-83.659580	48.837660	1122.084000	111.888700
-82.199460	49.037830	1504.126000	226.244100
-80.724000	49.219880	1561.879000	242.151100
-79.234450	49.383340	1210.353000	114.036600
-77.732160	49.527770	1065.568000	61.094920
-76.218590	49.652830	1099.807000	74.837350
-74.695260	49.758160	1209.828000	104.345000
-73.163800	49.843490	1196.397000	121.821500
-71.625870	49.908580	1218.506000	130.614400
-70.083210	49.953250	1264.388000	132.860900
-68.537570	49.977380	1196.902000	115.852100
-66.990740	49.980900	1049.026000	72.840710
-65.444490	49.963800	798.921700	53.373990
-63.900620	49.926140	629.939400	77.680110
-62.360880	49.868010	692.431800	49.885760
-60.827010	49.789570	909.309000	71.755230
-59.300730	49.691040	1023.124000	101.177800
-57.783640	49.572690	1040.644000	114.982300
-56.277320	49.434830	1057.382000	129.629300
-54.783270	49.277820	1012.648000	129.254000
-53.302860	49.102080	901.202600	98.325820
-51.837360	48.908040	721.468800	57.501540

-50.387950	48.696200	625.818500	26.422750
-48.955640	48.467040	597.512900	8.199508
-47.541310	48.221130	588.045700	2.892061
-46.145660	47.959000	585.169400	.859454
-44.769230	47.681210	584.492600	.205914
-43.412380	47.388330	584.372400	.040772
-42.075270	47.080920	584.358000	.006100
-40.757810	46.759540	584.357400	.000649
-39.459720	46.424710	584.357500	.000187
-95.186440	47.531940	650.689900	33.580310
-93.857410	47.868410	682.916600	44.568210
-92.508000	48.190720	722.006600	59.370370
-91.137910	48.498310	758.904700	64.919720
-89.747150	48.790600	821.747800	72.505070
-88.335950	49.067010	909.844500	90.216830
-86.904710	49.326930	955.601400	89.707420
-85.454100	49.569790	1049.990000	106.533300
-83.984930	49.795020	1303.215000	181.349500
-82.498220	50.002060	1461.534000	218.466400
-80.995120	50.190400	1414.743000	201.718200
-79.476920	50.359550	1176.321000	129.512600
-77.945060	50.509060	1078.770000	97.095470
-76.401090	50.638520	1111.923000	97.498260
-74.846650	50.747580	1239.491000	135.174400
-73.283450	50.835950	1204.873000	137.713100
-71.713320	50.903350	1190.700000	134.182700
-70.138080	50.949630	1213.437000	133.507400
-68.559640	50.974620	1184.403000	125.516700
-66.979900	50.978270	1141.653000	114.853000
-65.400800	50.960560	986.492800	91.984610
-63.824260	50.921550	703.152200	39.710060
-62.252170	50.861340	545.981300	89.138110
-60.686400	50.780110	717.686300	54.632510
-59.128770	50.678080	906.584800	97.758540
-57.581030	50.555550	957.752000	108.013700
-56.044860	50.412850	925.614700	111.930300
-54.521820	50.250360	844.839800	90.544880
-53.013420	50.068520	744.682400	64.542390
-51.520960	49.867810	646.134000	33.513510
-50.045680	49.648740	602.444600	13.373930
-48.588630	49.411830	589.329200	5.237840
-47.150700	49.157670	585.753600	1.690648
-45.732590	48.886830	584.655300	.486837
-44.334810	48.599900	584.392500	.117063
-42.957660	48.297460	584.356600	.021189
-41.601190	47.980120	584.356100	.002975
-40.265250	47.648430	584.357200	.000430
-38.949390	47.302930	584.357500	.000208
-95.710510	48.412890	715.149000	57.432040
-94.362470	48.760130	787.996700	76.110540
-92.993230	49.092850	874.558800	94.140950
-91.602360	49.410460	944.287300	106.609000
-90.189750	49.712350	1002.096000	117.883300
-88.755540	49.997940	1028.881000	122.044400
-87.300090	50.266570	1005.290000	113.954400
-85.824040	50.517650	977.895700	109.777900
-84.328210	50.750570	1041.974000	132.977600
-82.813610	50.964740	1133.184000	158.801400
-81.281460	51.159630	1171.130000	159.328500
-79.733130	51.334700	1095.089000	132.024600
-78.170130	51.489480	1079.379000	116.033900
-76.594090	51.623530	1136.998000	129.417400
-75.006800	51.736490	1236.773000	160.094500
-73.410070	51.828010	1171.002000	150.363100
-71.805870	51.897850	1134.978000	132.950400
-70.196170	51.945790	1149.125000	118.294300
-68.582990	51.971680	1096.387000	98.429150

-66.968440 51.975460 1040.601000 87.849190
-65.354560 51.957120 1015.078000 100.075200
-63.743440 51.916690 896.510600 84.522230
-62.137130 51.854320 752.761400 53.462430
-60.537630 51.770170 790.185900 72.809840
-58.946900 51.664510 871.881700 97.315190
-57.366820 51.537620 876.208700 103.578900
-55.799180 51.389870 830.760400 87.527920
-54.245650 51.221680 759.863300 68.463720
-52.707820 51.033510 700.099500 46.075480
-51.187080 50.825860 641.884500 25.093890
-49.684720 50.599280 605.329100 10.611190
-48.201800 50.354330 590.073000 3.717602
-46.739240 50.091630 585.589600 1.133197
-45.297720 49.811770 584.563400 .292022
-43.877700 49.515370 584.380000 .060306
-42.479410 49.203060 584.357800 .009963
-41.102810 48.875440 584.357100 .001385
-39.747590 48.533100 584.357400 .000297
-38.413150 48.176570 584.357500 .000227
-96.261670 49.289190 808.620300 80.858610
-94.893750 49.647600 938.514800 106.894000
-93.503820 49.991080 1019.724000 125.941100
-92.091320 50.319060 1022.989000 123.005900
-90.655950 50.630910 971.427400 110.307000
-89.197760 50.925990 898.573100 96.305190
-87.717060 51.203660 837.041100 83.229810
-86.214420 51.463270 802.223800 77.679540
-84.690660 51.704190 839.003500 90.470760
-83.146820 51.925780 956.740500 109.332000
-81.584160 52.127480 1062.548000 130.277100
-80.004110 52.308710 1068.989000 129.811400
-78.408280 52.468990 1033.545000 123.783000
-76.798410 52.607840 1082.716000 134.908000
-75.176390 52.724850 1179.859000 158.366300
-73.544200 52.819690 1111.841000 145.074700
-71.903920 52.892050 1065.555000 121.874700
-70.257710 52.941730 1073.479000 101.348700
-68.607750 52.968570 1005.988000 67.624230
-66.956280 52.972490 916.068800 49.394500
-65.305560 52.953480 936.482100 81.409950
-63.657820 52.911580 964.381600 107.739900
-62.015270 52.846950 918.388300 105.710900
-60.380080 52.759750 877.150100 99.138160
-58.754360 52.650280 852.447200 96.413600
-57.140120 52.518850 802.120500 78.908560
-55.539290 52.365840 734.762800 61.674470
-53.953660 52.191710 683.983500 42.742090
-52.384890 51.996940 645.916600 27.958310
-50.834480 51.782070 615.409700 15.233730
-49.303730 51.547700 595.646900 6.507651
-47.793770 51.294400 587.504200 2.202190
-46.305490 51.022820 585.085600 .631902
-44.839570 50.733620 584.499100 .155427
-43.396390 50.427420 584.380100 .032119
-41.976090 50.104880 584.360400 .005288
-40.578500 49.766630 584.357700 .000714
-39.203150 49.413260 584.357500 .000239
-37.849210 49.045330 584.357500 .000183
-96.841810 50.160530 819.644300 76.201430
-95.453030 50.530490 912.687200 102.055200
-94.041510 50.885130 924.142800 101.301900
-92.606450 51.223850 862.717300 88.906150
-91.147380 51.546020 790.230800 73.649190
-89.664200 51.850960 730.499800 59.073940
-88.157130 52.138020 695.087000 48.522300
-86.626690 52.406500 685.814000 44.554240

-85.073680	52.655720	721.635300	48.713940
-83.499180	52.885050	795.320700	66.574620
-81.904440	53.093850	858.534100	82.215310
-80.290990	53.281520	911.051500	97.070430
-78.660530	53.447530	910.595500	104.107300
-77.014920	53.591380	983.404500	119.733300
-75.356160	53.712650	1094.660000	146.810300
-73.686420	53.810940	1034.398000	137.184100
-72.007930	53.885960	980.588600	109.738300
-70.322990	53.937460	977.108900	85.702160
-68.634010	53.965280	895.599200	44.740410
-66.943400	53.969350	817.660000	41.749650
-65.253590	53.949630	888.301700	65.220310
-63.567000	53.906200	959.578100	109.893900
-61.886040	53.839190	933.469700	109.603200
-60.213040	53.748820	843.045400	89.732120
-58.550290	53.635360	761.594600	65.350730
-56.899960	53.499180	695.852900	48.078710
-55.264110	53.340690	653.846700	33.425310
-53.644650	53.160350	629.255200	23.362630
-52.043340	52.958710	609.806400	14.031920
-50.461760	52.736320	596.113500	7.085452
-48.901280	52.493820	588.746500	2.920107
-47.363030	52.231850	585.721100	1.026150
-45.847900	51.951060	584.714900	.314360
-44.356530	51.652150	584.436300	.082570
-42.889220	51.335790	584.372200	.018177
-41.445980	51.002650	584.359800	.003337
-40.026490	50.653380	584.357700	.000550
-38.630060	50.288590	584.357500	.000186
-37.255620	49.908830	584.357500	.000183
-97.453060	51.026530	709.283000	50.849540
-96.042340	51.408490	749.791500	55.158100
-94.608220	51.774700	737.869600	58.814030
-93.149600	52.124570	708.995500	53.536110
-91.665800	52.457450	681.640400	45.263030
-90.156560	52.772640	656.672100	36.902870
-88.621980	53.069450	638.342600	29.508270
-87.062480	53.347160	624.786700	23.037160
-85.478850	53.605050	607.024900	21.527670
-83.872130	53.842430	565.084400	36.664140
-82.243660	54.058630	509.277900	63.589690
-80.595020	54.253030	583.675000	47.748960
-78.928000	54.425040	737.055100	56.193280
-77.244600	54.574130	902.898700	100.301500
-75.546970	54.699840	1033.075000	136.806000
-73.837420	54.801750	976.154700	130.507200
-72.118360	54.879540	907.644800	95.871510
-70.392330	54.932960	858.003600	64.969320
-68.661900	54.961820	737.995800	46.450280
-66.929700	54.966030	727.109100	46.724620
-65.198370	54.945580	822.423000	65.415940
-63.470540	54.900540	865.994200	92.350720
-61.748820	54.831050	825.590900	83.822880
-60.035740	54.737340	731.333500	55.348880
-58.333780	54.619720	660.880600	34.984700
-56.645260	54.478570	629.615300	21.665150
-54.972420	54.314330	614.408600	15.830370
-53.317280	54.127510	606.785800	13.318040
-51.681740	53.918690	597.751200	8.425345
-50.067420	53.688470	589.950000	3.849868
-48.475770	53.437500	586.336800	1.484607
-46.907930	53.166490	584.987800	.528553
-45.364770	52.876130	584.529900	.168310
-43.846840	52.567130	584.396700	.046322
-42.354370	52.240220	584.364700	.010826
-40.887210	51.896080	584.358600	.002123

-39.444820	51.535370	584.357600	.000360
-38.026250	51.158730	584.357500	.000176
-36.630120	50.766700	584.357500	.000183
-98.097820	51.886770	632.066600	23.340140
-96.663910	52.281200	639.492200	25.678980
-95.206040	52.659450	636.653300	25.278240
-93.722790	53.020900	632.291400	24.731820
-92.213200	53.364910	629.867700	25.487770
-90.676770	53.690770	628.941200	26.782740
-89.113440	53.997730	625.667000	26.141650
-87.523560	54.285050	615.746000	21.273280
-85.907830	54.551970	589.034800	12.919420
-84.267300	54.797760	509.335200	38.821580
-82.603340	55.021710	356.636300	98.028630
-80.917590	55.223140	267.613100	166.965600
-79.211960	55.401440	495.352300	68.109930
-77.488560	55.556020	818.250700	73.226590
-75.749730	55.686380	986.427100	128.419500
-73.997920	55.792100	928.955400	120.963800
-72.235800	55.872810	835.538300	77.581640
-70.466090	55.928230	720.647300	55.969450
-68.691570	55.958180	617.001300	74.543030
-66.915130	55.962550	723.820300	55.237420
-65.139640	55.941330	796.953400	74.003750
-63.367970	55.894590	764.601900	69.570250
-61.602940	55.822490	710.290100	51.528590
-59.847310	55.725280	652.543600	31.502430
-58.103760	55.603290	614.651700	17.984650
-56.374820	55.456930	599.998600	9.097421
-54.662870	55.286670	594.631200	5.930392
-52.970100	55.093080	596.088500	7.369102
-51.298500	54.876750	594.889100	7.064353
-49.649800	54.638340	589.063200	3.404805
-48.025460	54.378550	585.845800	1.210890
-46.426660	54.098120	584.810400	.403399
-44.854210	53.797780	584.482900	.129052
-43.308580	53.478290	584.386500	.036781
-41.789850	53.140400	584.362900	.009026
-40.297680	52.784830	584.358300	.001807
-38.831280	52.412250	584.357600	.000312
-37.389370	52.023280	584.357500	.000170
-35.970190	51.618480	584.357500	.000183
-98.778840	52.740810	600.786400	9.430497
-97.320270	53.148240	600.845200	9.475551
-95.837370	53.539010	600.171900	9.220770
-94.328290	53.912530	599.282300	9.349220
-92.791720	54.268120	600.014600	10.359470
-91.226900	54.605080	603.338700	12.904250
-89.633560	54.922620	607.892600	16.297180
-88.011890	55.219970	611.521200	19.445530
-86.362530	55.496330	605.757600	19.383710
-84.686490	55.750910	569.769500	20.375110
-82.985150	55.982970	450.784300	60.206310
-81.260250	56.191770	224.129600	175.247100
-79.513790	56.376650	290.079200	165.855800
-77.748020	56.536990	724.531300	58.200000
-75.965460	56.672260	930.178000	115.829300
-74.168790	56.781970	878.771700	107.873800
-72.360860	56.865740	780.148100	67.874910
-70.544630	56.923280	663.286900	64.631650
-68.723180	56.954370	645.930600	65.106460
-66.899620	56.958910	791.752900	79.678960
-65.077100	56.936880	782.897800	74.270560
-63.258730	56.888360	682.481100	45.829130
-61.447630	56.813510	628.440800	26.465380
-59.646790	56.712620	613.029400	18.464070
-57.859090	56.586040	604.980700	11.370810

-56.087300	56.434210	593.025900	6.048415
-54.333970	56.257640	589.034900	3.015700
-52.601470	56.056940	591.849700	4.870474
-50.891880	55.832740	594.591200	7.097682
-49.207030	55.585770	590.129400	4.141739
-47.548420	55.316760	586.186600	1.536685
-45.917200	55.026500	584.909000	.484352
-44.314130	54.715760	584.518600	.155507
-42.739580	54.385350	584.397500	.045889
-41.193420	54.036030	584.365700	.011797
-39.675070	53.668560	584.358800	.002572
-38.183400	53.283620	584.357600	.000461
-36.716770	52.881820	584.357500	.000203
-35.272900	52.463700	584.357500	.000183
-99.499240	53.588130	589.351000	3.551507
-98.014280	54.009130	588.917300	3.242867
-96.504860	54.412960	588.647700	3.116688
-94.968610	54.799050	588.371100	3.027588
-93.403790	55.166730	588.603500	3.282917
-91.809290	55.515250	589.760600	4.181083
-90.184560	55.843850	592.109200	5.915993
-88.529620	56.151670	596.129700	8.837923
-86.844990	56.437890	599.497700	12.643320
-85.131650	56.701690	599.374600	18.646880
-83.390960	56.942230	568.196200	31.838330
-81.624720	57.158780	421.077000	70.267180
-79.835050	57.350580	364.684400	115.204700
-78.024370	57.516980	688.011500	66.195990
-76.195360	57.657400	860.588700	104.368100
-74.350940	57.771310	841.162900	97.477450
-72.494220	57.858320	770.299000	73.464160
-70.628410	57.918090	706.385500	53.251510
-68.756890	57.950390	728.270800	62.327160
-66.883060	57.955100	811.682400	83.665470
-65.010370	57.932220	739.601300	63.292450
-63.142240	57.881810	650.717600	33.811420
-61.282040	57.804080	602.243800	14.002120
-59.433060	57.699310	593.801500	8.752569
-57.598450	57.567890	590.890400	9.104606
-55.781200	57.410300	585.238200	5.278202
-53.984060	57.227100	585.011900	1.559208
-52.209560	57.018940	588.646400	2.796879
-50.459930	56.786500	593.865100	6.622559
-48.737060	56.530560	592.420600	5.674395
-47.042490	56.251900	587.513000	2.387946
-45.377330	55.951360	585.262500	.753009
-43.742270	55.629770	584.608500	.225862
-42.137470	55.287960	584.421800	.066001
-40.562610	54.926740	584.371300	.017497
-39.016750	54.546860	584.359900	.004010
-37.498410	54.149020	584.357800	.000774
-36.005400	53.733830	584.357500	.000202
-34.534880	53.301770	584.357500	.000183
-100.262700	54.428120	585.637500	1.143627
-98.749250	54.863350	585.437100	.994648
-97.211560	55.280850	585.353500	.915460
-95.646590	55.680060	585.313800	.885234
-94.052090	56.060360	585.422400	.950739
-92.426510	56.420970	585.790100	1.198714
-90.768940	56.761100	586.531100	1.700570
-89.079190	57.079890	587.829000	2.641205
-87.357570	57.376450	589.726500	4.431181
-85.605000	57.649890	594.366600	9.673456
-83.822840	57.899360	593.192100	20.169320
-82.012920	58.124030	540.238800	28.685420
-80.177480	58.323120	518.793700	46.839690
-78.319110	58.495910	715.071700	81.213460

-76.440710 58.641750 791.235300 93.989750
-74.545430 58.760110 803.610600 91.772060
-72.636660 58.850530 795.621500 71.539780
-70.717930 58.912650 763.868400 62.215390
-68.792920 58.946230 781.378300 65.224880
-66.865380 58.951130 776.542200 72.688800
-64.939080 58.927340 693.897600 47.698510
-63.017780 58.874950 631.450700 23.885690
-61.105210 58.794160 598.354100 10.239670
-59.204930 58.685300 589.893900 6.866223
-57.320400 58.548780 587.744000 7.674266
-55.454840 58.385130 580.402000 6.588483
-53.611270 58.194950 579.368400 4.596496
-51.792370 57.978940 583.863100 .720896
-50.000500 57.737840 590.967300 4.561248
-48.237630 57.472490 594.071500 6.834980
-46.505310 57.183730 588.989700 3.325051
-44.804610 56.872430 585.648700 1.042225
-43.136050 56.539500 584.701000 .297689
-41.499610 56.185780 584.447400 .086131
-39.894630 55.812120 584.378100 .023445
-38.319800 55.419290 584.361400 .005638
-36.773090 55.007980 584.358000 .001149
-35.251730 54.578750 584.357600 .000206
-33.752120 54.132060 584.357500 .000186
-101.073700 55.260130 584.628800 .315284
-99.529010 55.710320 584.567300 .252901
-97.960900 56.142140 584.549600 .228704
-96.365430 56.555100 584.553300 .225333
-94.739640 56.948590 584.606700 .259213
-93.081440 57.321860 584.755100 .358679
-91.389490 57.674070 585.070000 .548417
-89.663250 58.004340 585.673100 .900828
-87.902820 58.311730 587.010900 2.064562
-86.109000 58.595320 591.060700 6.250609
-84.283100 58.854170 591.130600 14.582020
-82.427000 59.087400 567.020400 19.900230
-80.543050 59.294160 604.703200 31.131990
-78.634010 59.473690 723.130100 74.640130
-76.703000 59.625280 689.007300 65.742920
-74.753460 59.748340 718.840300 66.269330
-72.789070 59.842370 765.046400 66.623920
-70.813740 59.906970 786.179900 62.583690
-68.831480 59.941910 792.589100 71.535850
-66.846440 59.947010 754.808300 63.899970
-64.862770 59.922250 671.472700 37.947820
-62.884610 59.867760 616.783000 17.477930
-60.916040 59.783740 594.086700 7.150691
-58.961010 59.670550 590.309400 4.733413
-57.023270 59.528640 589.262600 5.397053
-55.106350 59.358580 580.919900 5.397883
-53.213500 59.161040 576.571500 6.354345
-51.347600 58.936760 577.913900 4.776245
-49.511150 58.686560 584.758600 .390477
-47.706190 58.411320 592.066800 5.417725
-45.934240 58.111950 589.067000 3.376252
-44.196280 57.789400 585.738400 1.137337
-42.492650 57.444580 584.735200 .320594
-40.823030 57.078400 584.462300 .096877
-39.186360 56.691730 584.383000 .027090
-37.580830 56.285340 584.362700 .006809
-36.003750 55.859910 584.358300 .001454
-34.451600 55.415950 584.357600 .000280
-32.919830 54.953830 584.357500 .000186
-101.937200 56.083360 584.404100 .071412
-100.358100 56.549350 584.390300 .052861
-98.756940 56.996240 584.387600 .046804

-97.128790	57.423640	584.392000	.049577
-95.469860	57.830970	584.413200	.067733
-93.777320	58.217490	584.478000	.118107
-92.049300	58.582370	584.652200	.240717
-90.284800	58.924680	585.092000	.598244
-88.483640	59.243460	586.395100	1.898071
-86.646400	59.537720	590.373500	6.222580
-84.774360	59.806450	589.349900	14.792640
-82.869400	60.048700	568.020900	20.491770
-80.933970	60.263580	621.776900	26.115960
-78.971000	60.450230	684.907600	50.272860
-76.983890	60.607900	563.099100	56.753480
-74.976370	60.735940	551.878500	50.657970
-72.952470	60.833800	634.887300	41.157970
-70.916480	60.901050	734.195400	55.564880
-68.872850	60.937420	793.686600	72.884190
-66.826130	60.942730	744.358500	60.052650
-64.780930	60.916960	655.893400	31.540260
-62.741830	60.860230	605.660200	12.043910
-60.713310	60.772790	587.714200	3.751303
-58.699720	60.654990	585.590500	1.408853
-56.705200	60.507380	586.889000	2.149581
-54.733590	60.330550	583.341700	2.140902
-52.788400	60.125220	579.180800	4.027517
-50.872730	59.892220	577.079900	5.288917
-48.989200	59.632420	579.555700	3.595024
-47.139900	59.346780	586.559600	1.717599
-45.326320	59.036270	588.217000	2.691154
-43.549270	58.701890	585.979300	1.217508
-41.808860	58.344600	584.796400	.364491
-40.104370	57.965370	584.471300	.100107
-38.434220	57.565060	584.385600	.028230
-36.795950	57.144450	584.363500	.007106
-35.186060	56.704150	584.358600	.001578
-33.600030	56.244660	584.357600	.000307
-32.032190	55.766200	584.357500	.000194
-102.859900	56.896880	584.363900	.013092
-101.242000	57.379640	584.361500	.008644
-99.604390	57.842470	584.361300	.007773
-97.940930	58.285070	584.363200	.009891
-96.246660	58.706940	584.372300	.019356
-94.517820	59.107400	584.406400	.051625
-92.751850	59.485580	584.518700	.159071
-90.947170	59.840560	584.847000	.535868
-89.103220	60.171320	585.929000	1.875147
-87.220290	60.476800	589.124700	6.336111
-85.299550	60.755960	585.836500	15.631050
-83.342830	61.007760	562.033000	23.445760
-81.352710	61.231220	604.930300	14.961750
-79.332320	61.425420	635.553600	28.898980
-77.285290	61.589540	480.928800	69.888850
-75.215710	61.722870	376.939300	151.562900
-73.128000	61.824790	401.064300	161.889700
-71.026890	61.894870	584.278000	67.989010
-68.917300	61.932760	758.813100	65.424710
-66.804310	61.938290	730.260000	55.855510
-64.692990	61.911450	641.076200	25.548110
-62.588420	61.852340	595.646800	8.195330
-60.495590	61.761240	581.833600	4.122517
-58.419300	61.638580	581.363100	2.311661
-56.364090	61.484900	583.860500	.472718
-54.334160	61.300890	583.740800	.685843
-52.333320	61.087320	582.483900	1.609372
-50.364880	60.845090	580.397200	2.905092
-48.431600	60.575160	579.506900	3.447793
-46.535590	60.278550	583.048900	1.360881
-44.678250	59.956310	586.337200	1.628024

-42.860180	59.609490	585.905300	1.082677
-41.081100	59.239120	584.798300	.353598
-39.339830	58.846180	584.461200	.091157
-37.634110	58.431540	584.382300	.023478
-35.960610	57.995940	584.363000	.006143
-34.314830	57.539980	584.358500	.001369
-32.690990	57.064010	584.357600	.000288
-31.082000	56.568130	584.357500	.000191
-103.849600	57.699570	584.358200	.001851
-102.187300	58.200280	584.357800	.001083
-100.509000	58.680030	584.357900	.001095
-98.806840	59.138710	584.358800	.002348
-97.074520	59.575940	584.363800	.008469
-95.307120	59.991050	584.385500	.034223
-93.501110	60.383240	584.460600	.133674
-91.654170	60.751550	584.674200	.499694
-89.765210	61.094930	585.336900	1.770915
-87.834170	61.412270	587.125100	6.275308
-85.861950	61.702450	583.078300	15.902790
-83.850420	61.964360	564.990700	21.049360
-81.802150	62.196920	595.235500	7.658556
-79.720490	62.399150	590.870600	26.034720
-77.609360	62.570130	458.362200	99.957340
-75.473210	62.709080	330.455500	182.523400
-73.316960	62.815350	283.485300	207.813300
-71.145790	62.888430	404.361300	156.822900
-68.965200	62.927950	649.990100	55.364520
-66.780790	62.933720	684.930100	40.579730
-64.598260	62.905720	622.794200	18.338860
-62.423240	62.844070	590.531400	6.026085
-60.261290	62.749080	582.739200	2.898920
-58.117710	62.621230	582.650500	1.474315
-55.997520	62.461110	583.780500	.441961
-53.905320	62.269450	583.998300	.293197
-51.845240	62.047150	583.738200	.527642
-49.820820	61.795150	583.087600	1.011662
-47.834930	61.514500	582.418900	1.465557
-45.889670	61.206310	582.735100	1.164205
-43.986290	60.871700	584.848000	.455939
-42.125070	60.511780	584.734000	.355315
-40.305270	60.127630	584.496500	.122691
-38.525020	59.720250	584.404200	.040753
-36.781200	59.290500	584.371000	.013549
-35.069400	58.839090	584.360500	.003619
-33.383770	58.366500	584.358000	.000869
-31.716990	57.872960	584.357600	.000205
-30.060130	57.358350	584.357500	.000186
-104.916500	58.490070	584.357600	.000254
-103.202300	59.010120	584.357500	.000229
-101.477600	59.507980	584.357600	.000261
-99.732440	59.983750	584.358100	.001061
-97.958720	60.437220	584.361400	.005723
-96.150010	60.867820	584.375700	.027149
-94.301540	61.274790	584.423200	.116574
-92.410060	61.657170	584.547200	.434344
-90.473700	62.013880	584.829300	1.566282
-88.491920	62.343760	585.585800	5.748024
-86.465330	62.645610	585.012000	13.468260
-84.395630	62.918240	583.843600	12.940380
-82.285480	63.160470	606.964500	14.666300
-80.138370	63.371240	587.692300	18.577630
-77.958560	63.549540	511.325600	57.533420
-75.750930	63.694510	430.881400	114.891600
-73.520860	63.805420	369.670900	157.312100
-71.274150	63.881700	405.920500	128.076700
-69.016910	63.922960	563.746700	48.592670
-66.755400	63.928990	610.063700	21.275310

-64.495980 63.899750 597.913700 8.889833
-62.244980 63.835380 587.434600 3.156764
-60.008560 63.736250 584.349400 1.149372
-57.792640 63.602840 583.917500 .496915
-55.602750 63.435840 584.108300 .203781
-53.443960 63.236060 584.197200 .130946
-51.320750 63.004450 584.148000 .182392
-49.236890 62.742080 584.004600 .295293
-47.195330 62.450080 583.851700 .416570
-45.198120 62.129630 583.815200 .419657
-43.246250 61.781950 584.282000 .086220
-41.339580 61.408210 584.393500 .051071
-39.476710 61.009530 584.393200 .030752
-37.654880 60.586900 584.373700 .015859
-35.869840 60.141170 584.362500 .005412
-34.115770 59.672970 584.358600 .001669
-32.385130 59.182660 584.357600 .000432
-30.668580 58.670220 584.357500 .000188
-28.954870 58.135290 584.357500 .000183
-106.073800 59.266680 584.357500 .000186
-104.297500 59.807790 584.357500 .000186
-102.518800 60.325180 584.357500 .000229
-100.725000 60.819250 584.357800 .000707
-98.905530 61.289990 584.359900 .003974
-97.052100 61.737010 584.368700 .020309
-95.158340 62.159620 584.396800 .086945
-93.219700 62.556880 584.455300 .328761
-91.233350 62.927700 584.524900 1.228202
-89.198040 63.270870 585.184600 4.369316
-87.113920 63.585110 587.658700 9.262719
-84.982480 63.869130 590.753800 4.702568
-82.806390 64.121680 592.497100 12.007020
-80.589290 64.341560 564.716600 18.292310
-78.335770 64.527670 529.801000 34.400160
-76.051160 64.679080 504.659300 52.721810
-73.741460 64.794950 490.075200 66.887960
-71.413090 64.874680 504.826500 56.853160
-69.072890 64.917820 559.604900 25.664690
-66.727910 64.924110 586.710000 9.046571
-64.385280 64.893550 587.756300 3.727738
-62.052090 64.826280 585.536700 1.506236
-59.735260 64.722690 584.504900 .526176
-57.441380 64.583340 584.244400 .191568
-55.176610 64.408980 584.265400 .083932
-52.946510 64.200520 584.300200 .052850
-50.755950 63.959000 584.293300 .061729
-48.608900 63.685590 584.258800 .089119
-46.508400 63.381530 584.229600 .108471
-44.456330 63.048090 584.231800 .106848
-42.453320 62.686580 584.311700 .038204
-40.498620 62.298210 584.358200 .006552
-38.589950 61.884150 584.365400 .008090
-36.723370 61.445380 584.361600 .004294
-34.893150 60.982680 584.359000 .001855
-33.091610 60.496560 584.357800 .000562
-31.309040 59.987160 584.357500 .000206
-29.533510 59.454220 584.357500 .000184
-27.750760 58.896990 584.357500 .000183
-107.339200 60.027230 584.357500 .000183
-105.487000 60.591610 584.357500 .000183
-103.643800 61.130270 584.357500 .000202
-101.793500 61.644070 584.357700 .000428
-99.922550 62.133320 584.358800 .002494
-98.020040 62.597820 584.363400 .012485
-96.077510 63.037030 584.376100 .054030
-94.088710 63.450070 584.397500 .187912
-92.049460 63.835860 584.384900 .602426

-89.957580 64.193130 584.257600 1.800914
-87.812570 64.520550 583.385300 1.950062
-85.615560 64.816710 581.314300 5.630672
-83.369120 65.080260 572.397000 12.765710
-81.077050 65.309890 560.286900 16.204990
-78.744270 65.504390 553.896400 19.150970
-76.376630 65.662700 552.352200 21.065940
-73.980770 65.783920 553.726700 21.638530
-71.563890 65.867360 561.287400 17.567640
-69.133670 65.912500 575.220200 8.844966
-66.698060 65.919100 583.397200 3.544233
-64.265090 65.887100 584.838100 1.534025
-61.842790 65.816700 584.634000 .654268
-59.438900 65.708320 584.394600 .239971
-57.060850 65.562580 584.320600 .076295
-54.715470 65.380330 584.327700 .031348
-52.408880 65.162570 584.340600 .019486
-50.146370 64.910470 584.340700 .019656
-47.932120 64.625290 584.332000 .026101
-45.769150 64.308400 584.324400 .031463
-43.659080 63.961160 584.327500 .026615
-41.602020 63.584970 584.343700 .013274
-39.596350 63.181100 584.355700 .001798
-37.638600 62.750720 584.358500 .001125
-35.723270 62.294770 584.358300 .001075
-33.842640 61.813940 584.357700 .000502
-31.986660 61.308540 584.357600 .000210
-30.142740 60.778430 584.357500 .000185
-28.295590 60.222970 584.357500 .000183
-26.427080 59.640870 584.357500 .000183
-108.736700 60.768890 584.357500 .000183
-106.789100 61.359350 584.357500 .000183
-104.866900 61.921510 584.357500 .000189
-102.949500 62.456830 584.357700 .000363
-101.019200 62.966030 584.358300 .001951
-99.061880 63.449270 584.360500 .009385
-97.066180 63.906190 584.364300 .036042
-95.023590 64.336020 584.353900 .118675
-92.928180 64.737730 584.263400 .287076
-90.776390 65.110020 583.866100 .626239
-88.566850 65.451470 582.816300 1.178401
-86.300130 65.760610 579.168300 5.198212
-83.978550 66.035940 573.063400 8.489839
-81.606060 66.276030 569.636600 9.660208
-79.187900 66.479550 570.251000 9.646894
-76.730480 66.645290 571.213200 9.317087
-74.241170 66.772270 572.693700 8.554835
-71.728080 66.859700 575.897900 6.539098
-69.199870 66.907030 580.228800 3.856503
-66.665550 66.913940 583.206100 1.794445
-64.134220 66.880400 584.213400 .817663
-61.614990 66.806620 584.378200 .352940
-59.116610 66.693070 584.354400 .131389
-56.647430 66.540450 584.341700 .041519
-54.215080 66.349720 584.346400 .014809
-51.826330 66.121990 584.351900 .008675
-49.486860 65.858530 584.352600 .007823
-47.201050 65.560780 584.349900 .009794
-44.971820 65.230190 584.347200 .011360
-42.800360 64.868270 584.347900 .010165
-40.685980 64.476460 584.352500 .005268
-38.625890 64.056100 584.356100 .001817
-36.614990 63.608330 584.357500 .000250
-34.645670 63.134020 584.357600 .000244
-32.707610 62.633670 584.357600 .000192
-30.787590 62.107330 584.357500 .000187
-28.869240 61.554470 584.357500 .000183

-26.932890 60.973880 584.357500 .000183
-24.955330 60.363580 584.357500 .000183

Q.27a
-89.453400 32.991660 615.609900 .062121
-88.345930 33.191160 615.621600 .079006
-87.228100 33.381660 615.668500 .147689
-86.100230 33.562890 615.759000 .269859
-84.962690 33.734530 615.909200 .448893
-83.815900 33.896310 616.128500 .676023
-82.660300 34.047950 616.385100 .914922
-81.496410 34.189190 616.584200 1.099265
-80.324730 34.319790 616.628700 1.148181
-79.145840 34.439500 616.467000 .978716
-77.960310 34.548100 616.009600 .548651
-76.768760 34.645400 615.168800 .310409
-75.571840 34.731200 614.195200 .895051
-74.370180 34.805370 613.938700 1.042392
-73.164480 34.867740 614.465900 .827209
-71.955430 34.918200 615.114000 .433829
-70.743730 34.956650 615.482800 .140815
-69.530080 34.983020 615.619900 .035906
-68.315220 34.997260 615.649800 .072882
-67.099880 34.999340 615.638600 .066565
-65.884770 34.989250 615.615200 .042540
-64.670640 34.967020 615.597600 .021329
-63.458190 34.932690 615.589300 .008857
-62.248150 34.886320 615.586500 .003277
-61.041230 34.828000 615.585700 .001254
-59.838120 34.757850 615.585400 .000875
-58.639500 34.676010 615.585300 .000852
-57.446040 34.582610 615.585400 .000797
-56.258390 34.477850 615.585600 .000725
-55.077160 34.361910 615.585600 .000577
-53.902950 34.235010 615.585600 .000402
-52.736340 34.097380 615.585600 .000258
-51.577860 33.949260 615.585600 .000242
-50.428010 33.790910 615.585600 .000244
-49.287250 33.622600 615.585600 .000244
-48.156040 33.444620 615.585600 .000244
-47.034740 33.257250 615.585600 .000241
-45.923690 33.060790 615.585600 .000244
-44.823220 32.855560 615.585600 .000185
-89.701210 33.918400 615.689000 .178343
-88.583520 34.125020 615.746800 .234859

-87.455040	34.322360	615.942400	.423348
-86.316120	34.510110	616.298000	.743207
-85.167100	34.687960	616.864600	1.199651
-84.008420	34.855620	617.665300	1.768515
-82.840550	35.012790	618.596600	2.377310
-81.663990	35.159200	619.341200	2.864899
-80.479300	35.294580	619.541200	3.011510
-79.287040	35.418700	619.002700	2.632011
-78.087840	35.531310	617.419100	1.594542
-76.882320	35.632210	614.589200	.845291
-75.671150	35.721200	611.108800	2.139080
-74.455030	35.798110	610.315900	2.514224
-73.234660	35.862810	612.062000	1.783017
-72.010770	35.915150	614.117200	.918960
-70.784100	35.955040	615.319000	.244718
-69.555380	35.982390	615.773100	.180389
-68.325390	35.997170	615.867400	.254866
-67.094890	35.999320	615.810700	.213657
-65.864640	35.988860	615.710400	.132212
-64.635410	35.965800	615.637100	.065762
-63.407950	35.930180	615.603000	.028868
-62.183030	35.882080	615.591100	.012328
-60.961370	35.821590	615.587300	.006172
-59.743720	35.748840	615.585700	.004317
-58.530780	35.663950	615.585300	.003720
-57.323260	35.567100	615.585300	.003208
-56.121820	35.458470	615.586000	.002706
-54.927120	35.338260	615.586200	.002009
-53.739770	35.206700	615.585900	.001124
-52.560380	35.064020	615.585700	.000541
-51.389480	34.910500	615.585600	.000266
-50.227610	34.746390	615.585400	.000240
-49.075240	34.571990	615.585600	.000247
-47.932830	34.387580	615.585600	.000244
-46.800740	34.193490	615.585600	.000244
-45.679330	33.990000	615.585400	.000244
-44.568900	33.777440	615.585600	.000186
-89.960660	34.843320	616.107900	.613062
-88.832330	35.057210	616.403800	.818735
-87.692770	35.261540	617.295800	1.422496
-86.542320	35.455950	618.837600	2.405972
-85.381340	35.640140	621.192300	3.759822
-84.210270	35.813810	624.405200	5.433366
-83.029580	35.976620	628.151600	7.261201
-81.839790	36.128310	631.258400	8.795509
-80.641480	36.268600	632.202100	9.323259
-79.435230	36.397220	630.218900	8.273510
-78.221690	36.513940	624.206700	5.363859
-77.001530	36.618520	613.348400	2.867437
-75.775430	36.710780	599.663300	6.466864
-74.544130	36.790520	598.668400	6.360783
-73.308360	36.857590	604.032800	4.657130
-72.068890	36.911860	610.966400	2.155387
-70.826490	36.953220	615.027800	.723386
-69.581950	36.981590	616.674800	.927401
-68.336070	36.996900	616.969400	1.037217
-67.089650	36.999130	616.662700	.826320
-65.843500	36.988290	616.171600	.491897
-64.598400	36.964370	615.832600	.244321
-63.355190	36.927440	615.678500	.113301
-62.114630	36.877570	615.620900	.055950
-60.877510	36.814860	615.599000	.032844
-59.644590	36.739430	615.589600	.024056
-58.416640	36.651430	615.585600	.019457
-57.194370	36.551040	615.585400	.015522
-55.978490	36.438440	615.588600	.012862
-54.769680	36.313860	615.589400	.008947

-53.568590	36.177530	615.587800	.004574
-52.375820	36.029710	615.586300	.001848
-51.191970	35.870660	615.585800	.000664
-50.017550	35.700660	615.585500	.000304
-48.853080	35.520030	615.585400	.000241
-47.698980	35.329070	615.585600	.000244
-46.555660	35.128100	615.585600	.000244
-45.423470	34.917440	615.585400	.000244
-44.302690	34.697410	615.585600	.000190
-90.232250	35.766320	617.703900	1.785471
-89.092860	35.987640	618.918000	2.435936
-87.941790	36.199090	622.263300	4.167709
-86.779330	36.400320	627.785900	6.862579
-85.605870	36.591000	635.944400	10.482840
-84.421870	36.770790	646.576000	14.790940
-83.227800	36.939400	659.467700	19.871330
-82.024180	37.096500	670.888300	24.463980
-80.811620	37.241800	674.458400	26.085220
-79.590740	37.375040	668.602400	23.720930
-78.362190	37.495960	649.659200	16.501890
-77.126670	37.604320	612.157300	8.503861
-75.884930	37.699920	565.203900	17.515800
-74.637700	37.782550	570.446500	19.200580
-73.385770	37.852060	592.951200	12.385400
-72.129940	37.908310	606.572000	5.222114
-70.871030	37.951180	616.823200	3.468102
-69.609860	37.980580	621.261800	3.923121
-68.347290	37.996460	621.777000	3.916131
-67.084140	37.998780	620.237500	2.993059
-65.821270	37.987530	617.956200	1.672652
-64.559530	37.962740	616.557600	.797684
-63.299760	37.924460	615.985400	.394419
-62.042780	37.872770	615.765100	.221095
-60.789440	37.807780	615.668300	.149197
-59.540510	37.729610	615.619600	.115387
-58.296810	37.638420	615.595400	.091568
-57.059080	37.534390	615.588300	.070155
-55.828080	37.417740	615.607400	.059886
-54.604500	37.288680	615.607300	.039495
-53.389020	37.147460	615.595300	.017163
-52.182290	36.994370	615.588900	.006037
-50.984880	36.829660	615.586300	.001808
-49.797380	36.653650	615.585600	.000506
-48.620270	36.466650	615.585400	.000265
-47.454020	36.268990	615.585400	.000245
-46.299010	36.060990	615.585600	.000244
-45.155610	35.843010	615.585400	.000244
-44.024070	35.615370	615.585600	.000196
-90.516530	36.687310	622.392100	4.434436
-89.365660	36.916220	626.560200	6.313050
-88.202590	37.134950	637.533700	11.083480
-87.027650	37.343150	654.701200	18.046790
-85.841200	37.540460	678.820900	26.749890
-84.643710	37.726540	708.305100	36.779930
-83.435650	37.901050	746.417700	49.076910
-82.217600	38.063690	784.644000	62.245760
-80.990140	38.214140	796.492700	67.334810
-79.753940	38.352110	783.532700	63.539800
-78.509670	38.477340	736.318700	47.196380
-77.258070	38.589580	621.580600	21.199570
-75.999910	38.688600	463.942000	37.602950
-74.735980	38.774210	493.482000	45.165890
-73.467090	38.846230	595.939600	44.298320
-72.194090	38.904510	623.402600	22.958200
-70.917830	38.948930	632.267800	13.251370
-69.639210	38.979390	641.846200	14.432310
-68.359090	38.995840	641.439800	13.652800

-67.078360	38.998240	634.536900	10.263880
-65.797930	38.986590	624.253700	5.173704
-64.518690	38.960900	618.866000	2.351114
-63.241520	38.921240	617.043500	1.237955
-61.967320	38.867690	616.362500	.806499
-60.696920	38.800350	616.032900	.631443
-59.431210	38.719360	615.849000	.549155
-58.170970	38.624900	615.720400	.450642
-56.917050	38.517150	615.643700	.330657
-55.670200	38.396340	615.727800	.256460
-54.431160	38.262690	615.707400	.170002
-53.200640	38.116470	615.627100	.061499
-51.979310	37.957960	615.596100	.016998
-50.767770	37.787460	615.588000	.004340
-49.566610	37.605300	615.585800	.000973
-48.376340	37.411790	615.585500	.000380
-47.197410	37.207270	615.585400	.000325
-46.030250	36.992090	615.585400	.000244
-44.875180	36.766620	615.585400	.000244
-43.732470	36.531200	615.585600	.000215
-90.814130	37.606180	633.296500	9.689484
-89.651300	37.842850	646.528700	15.211370
-88.475770	38.069030	680.857900	28.457970
-87.287830	38.284350	728.673500	45.378080
-86.087840	38.488450	787.541500	61.650830
-84.876290	38.680970	857.485500	79.126600
-83.653650	38.861550	954.330600	98.282020
-82.420500	39.029860	1065.848000	121.045400
-81.177470	39.185590	1105.177000	141.971200
-79.925230	39.328410	1088.742000	144.148000
-78.664510	39.458070	1005.264000	118.197400
-77.396060	39.574290	744.805700	56.367590
-76.120680	39.676830	343.209800	90.605430
-74.839220	39.765490	326.324300	61.987170
-73.552520	39.840080	632.927900	82.761850
-72.261480	39.900440	699.566300	64.274560
-70.967010	39.946460	697.070100	43.834530
-69.670030	39.978020	708.965100	40.166280
-68.371470	39.995060	706.176300	39.638600
-67.072270	39.997550	680.099000	28.842090
-65.773400	39.985470	641.917900	13.367450
-64.475770	39.958870	625.229300	6.056217
-63.180340	39.917780	620.676800	3.863116
-61.888020	39.862300	618.814100	2.955084
-60.599740	39.792560	617.912800	2.651564
-59.316390	39.708680	617.327800	2.452602
-58.038840	39.610860	616.715900	2.056250
-56.767930	39.499290	616.217900	1.447066
-55.504470	39.374200	616.185600	.907112
-54.249260	39.235840	616.019100	.520107
-53.003000	39.084480	615.742200	.187192
-51.766410	38.920440	615.614900	.039900
-50.540120	38.744010	615.590100	.007443
-49.324710	38.555530	615.585900	.001366
-48.120720	38.355340	615.585400	.000359
-46.928620	38.143810	615.585400	.000360
-45.748810	37.921300	615.585400	.000357
-44.581620	37.688170	615.585400	.000347
-43.427300	37.444810	615.585600	.000231
-91.125630	38.522790	654.512500	19.269830
-89.950410	38.767390	693.728600	34.442860
-88.761920	39.001200	788.236500	63.964710
-87.560450	39.223820	890.030900	92.783260
-86.346370	39.434880	971.366400	99.851470
-85.120160	39.633990	1099.684000	106.015300
-83.882300	39.820790	1309.240000	136.845400
-82.633390	39.994930	1550.438000	166.030000

-81.374080	40.156070	1675.944000	204.202300
-80.105060	40.303890	1700.170000	232.746800
-78.827100	40.438090	1631.615000	219.804500
-77.540980	40.558400	1249.025000	152.511800
-76.247540	40.664560	607.233200	106.235000
-74.947690	40.756360	373.898400	145.597000
-73.642300	40.833600	718.829900	103.545800
-72.332310	40.896110	839.444200	92.042730
-71.018690	40.943760	829.642500	82.221140
-69.702430	40.976440	838.913600	78.635770
-68.384490	40.994090	828.451200	77.087220
-67.065880	40.996670	749.389800	48.852260
-65.747600	40.984170	660.140600	21.305160
-64.430650	40.956610	637.766200	12.980410
-63.116020	40.914060	634.384000	12.320670
-61.804690	40.856610	629.243400	10.508420
-60.497620	40.784390	625.248400	8.876060
-59.195770	40.697540	622.130700	7.732703
-57.900040	40.596260	619.663600	6.292428
-56.611330	40.480750	618.187400	4.090775
-55.330490	40.351270	616.858200	1.754615
-54.058330	40.208070	616.112400	.661934
-52.795620	40.051450	615.790200	.212833
-51.543080	39.881710	615.630900	.048105
-50.301390	39.699200	615.590100	.007225
-49.071130	39.504250	615.585300	.001221
-47.852850	39.297230	615.585100	.000389
-46.647030	39.078520	615.585400	.000421
-45.454070	38.848490	615.585400	.000367
-44.274280	38.607530	615.585400	.000347
-43.107920	38.356030	615.585600	.000235
-91.451740	39.437030	686.443100	33.128460
-90.263630	39.689750	773.928200	61.764160
-89.061680	39.931370	946.856100	110.647800
-87.846150	40.161480	1029.618000	130.062100
-86.617400	40.379670	939.274800	114.736000
-85.375900	40.585540	1054.045000	109.521600
-84.122160	40.778730	1514.981000	122.766500
-82.856800	40.958850	1929.935000	188.482000
-81.580460	41.125550	2216.873000	238.581300
-80.293880	41.278500	2402.918000	299.960200
-78.997860	41.417370	2459.885000	332.888900
-77.693230	41.541890	2173.096000	269.943700
-76.380870	41.651780	1381.898000	140.521500
-75.061690	41.746810	872.855700	61.329460
-73.736660	41.826770	928.775500	75.452020
-72.406780	41.891490	938.877300	74.064360
-71.073040	41.940830	968.304400	88.899400
-69.736500	41.974670	952.010900	114.844600
-68.398190	41.992950	884.412500	87.969310
-67.059160	41.995620	691.807600	39.324650
-65.720480	41.982670	605.288600	19.848830
-64.383220	41.954140	651.128800	22.880340
-63.048400	41.910080	679.090900	33.675540
-61.717090	41.850600	661.456500	27.756980
-60.390290	41.775820	637.511000	17.679890
-59.069010	41.685920	620.713500	11.356090
-57.754210	41.581080	614.831700	7.131239
-56.446830	41.461530	617.474100	3.899034
-55.147770	41.327530	616.827100	1.098390
-53.857880	41.179360	615.655500	.571259
-52.577970	41.017320	615.379400	.415163
-51.308780	40.841740	615.519300	.135435
-50.051000	40.652980	615.577000	.026236
-48.805250	40.451390	615.583900	.005644
-47.572090	40.237360	615.584600	.001206
-46.352010	40.011290	615.585100	.000304

-45.145370 39.773560 615.585400 .000330
-43.952510 39.524580 615.585400 .000343
-42.773630 39.264750 615.585600 .000240
-91.793200 40.348750 707.170700 42.726690
-90.591710 40.609810 823.830600 78.591690
-89.375760 40.859430 995.332300 136.680700
-88.145600 41.097210 888.460000 136.743500
-86.901570 41.322720 514.498700 121.589200
-85.644150 41.535550 549.671000 139.076000
-84.373860 41.735290 1185.189000 78.156810
-83.091290 41.921560 1888.388000 159.812700
-81.797160 42.093980 2423.626000 271.332600
-80.492230 42.252200 2885.809000 381.790800
-79.177290 42.395880 3104.940000 432.311600
-77.853240 42.524730 2862.183000 391.663700
-76.521000 42.638450 2106.844000 192.826700
-75.181550 42.736820 1318.891000 104.145800
-73.835900 42.819590 1033.889000 78.436920
-72.485100 42.886590 872.629200 82.543020
-71.130210 42.937670 959.779800 94.775510
-69.772340 42.972710 1010.890000 115.172000
-68.412590 42.991630 846.091100 84.306540
-67.052090 42.994400 547.397900 74.747570
-65.691960 42.980990 507.344500 60.927230
-64.333320 42.951450 665.766800 34.682510
-62.977290 42.905830 754.800600 62.120090
-61.624970 42.844260 705.418500 43.728260
-60.277440 42.766850 633.821200 16.604490
-58.935750 42.673790 598.756000 13.527320
-57.600940 42.565280 598.998400 12.296070
-56.273990 42.441570 610.654400 6.025017
-54.955840 42.302930 616.061500 3.214706
-53.647390 42.149640 616.052400 1.532170
-52.349470 41.982030 615.838500 .485713
-51.062890 41.800450 615.702900 .149149
-49.788320 41.605260 615.618500 .053832
-48.526440 41.396860 615.591100 .016113
-47.277780 41.175640 615.585800 .003837
-46.042850 40.942000 615.585200 .000685
-44.822010 40.696380 615.585400 .000380
-43.615560 40.439180 615.585400 .000344
-42.423690 40.170830 615.585600 .000242
-92.150770 41.257820 691.177200 36.867430
-90.935390 41.527410 792.554100 72.506450
-89.704910 41.785250 953.307400 126.825700
-88.459540 42.030900 863.707300 122.170200
-87.199620 42.263930 458.067700 88.648810
-85.925610 42.483890 473.394300 113.450300
-84.638040 42.690380 1195.845000 80.956760
-83.337520 42.882970 1938.987000 217.783100
-82.024790 43.061280 2660.726000 370.247300
-80.700610 43.224930 3402.858000 477.265200
-79.365870 43.373580 3730.198000 564.572800
-78.021450 43.506890 3495.520000 502.261400
-76.668370 43.624570 2737.352000 342.795000
-75.307630 43.726360 1879.514000 141.225000
-73.940300 43.812040 1241.339000 66.942110
-72.567500 43.881390 758.833300 96.056590
-71.190370 43.934270 942.715300 74.204900
-69.810050 43.970550 1197.564000 185.408100
-68.427750 43.990140 1177.807000 142.547100
-67.044650 43.993000 774.781600 61.378460
-65.661930 43.979120 628.067000 40.369270
-64.280810 43.948540 728.328500 49.914630
-62.902460 43.901310 787.185200 63.076590
-61.528050 43.837570 716.070800 40.761900
-60.158730 43.757450 627.903900 23.519270

-58.795620	43.661140	599.014000	22.264560
-57.439790	43.548850	612.032000	15.331250
-56.092310	43.420850	626.256200	9.400723
-54.754150	43.277410	625.262600	5.219378
-53.426270	43.118850	620.045800	2.462253
-52.109530	42.945500	617.295500	1.128726
-50.804760	42.757750	616.159200	.493244
-49.512690	42.555960	615.741300	.184387
-48.233980	42.340550	615.617100	.057396
-46.969190	42.111940	615.589900	.014515
-45.718810	41.870550	615.585600	.002883
-44.483210	41.616830	615.585400	.000523
-43.262650	41.351200	615.585400	.000350
-42.057300	41.074100	615.585600	.000251
-92.525320	42.164070	650.733200	20.052510
-91.295520	42.442420	695.994400	39.565650
-90.049940	42.708690	778.283000	73.005150
-88.788760	42.962440	726.349300	81.114970
-87.512310	43.203190	495.574200	59.103660
-86.221000	43.430500	552.204000	77.261060
-84.915410	43.643920	1271.968000	141.564200
-83.596150	43.843030	2124.674000	289.920300
-82.263960	44.027400	2932.235000	437.995200
-80.919660	44.196660	3674.702000	581.815400
-79.564150	44.350410	3998.715000	601.648600
-78.198390	44.488330	3698.867000	548.448400
-76.823410	44.610100	2935.969000	377.908500
-75.440290	44.715440	2052.978000	188.347000
-74.050180	44.804110	1179.533000	115.113800
-72.654240	44.875890	567.530300	109.622300
-71.253690	44.930630	801.148100	150.652400
-69.849760	44.968180	1505.345000	243.286900
-68.443720	44.988460	1683.423000	254.678800
-67.036810	44.991420	1158.404000	123.204400
-65.630330	44.977050	770.419900	44.898840
-64.225530	44.945390	714.264700	40.766700
-62.823690	44.896510	742.886400	44.828350
-61.426040	44.830540	709.121200	32.210150
-60.033810	44.747610	657.461300	29.643160
-58.648170	44.647940	645.737200	27.260630
-57.270290	44.531750	655.188500	21.910790
-55.901260	44.399320	652.751700	16.539320
-54.542130	44.250930	640.380900	11.166310
-53.193900	44.086940	628.109700	6.480241
-51.857480	43.907680	620.706200	3.148170
-50.533710	43.713560	617.392600	1.343606
-49.223370	43.504980	616.112200	.499863
-47.927120	43.282370	615.705400	.159908
-46.645540	43.046150	615.605600	.042743
-45.379100	42.796800	615.587500	.009329
-44.128160	42.534760	615.585300	.001597
-42.892960	42.260480	615.585400	.000374
-41.673610	41.974410	615.585500	.000218
-92.917770	43.067330	619.599800	7.868098
-91.673000	43.354680	620.977600	14.583570
-90.411740	43.629630	605.837000	30.595950
-89.134110	43.891690	487.582500	61.939560
-87.840450	44.140390	326.445600	79.813200
-86.531140	44.375260	361.920800	128.388400
-85.206750	44.595830	1143.799000	149.894000
-83.867900	44.801650	2109.235000	321.934900
-82.515380	44.992280	2780.275000	437.305800
-81.150000	45.167310	3212.016000	461.068900
-79.772730	45.326340	3293.832000	474.031300
-78.384560	45.469020	3041.559000	402.194700
-76.986600	45.595010	2451.910000	259.526700
-75.579970	45.704020	1772.466000	139.193700

-74.165890	45.795780	1123.081000	77.895070
-72.745610	45.870080	503.940200	206.204500
-71.320400	45.926740	801.754000	170.031700
-69.891600	45.965610	1601.306000	258.163400
-68.460530	45.986610	1829.332000	293.788200
-67.028560	45.989670	1293.151000	124.303000
-65.597030	45.974800	802.351100	68.371480
-64.167300	45.942020	714.378700	34.869120
-62.740720	45.891430	758.278700	33.318760
-61.318600	45.823140	725.753200	29.389900
-59.902260	45.737310	695.973800	23.443590
-58.492950	45.634170	692.358300	28.076980
-57.091900	45.513950	687.795800	30.732710
-55.700260	45.376930	678.189700	28.747060
-54.319140	45.223450	669.640100	24.114470
-52.949590	45.053840	649.468300	15.928960
-51.592580	44.868490	629.989100	7.767457
-50.248970	44.667810	620.466100	3.092545
-48.919560	44.452230	617.029500	1.122135
-47.605040	44.222190	615.938700	.358722
-46.305980	43.978160	615.652600	.098973
-45.022840	43.720610	615.594600	.022889
-43.755960	43.450020	615.586000	.004315
-42.505550	43.166850	615.585400	.000684
-41.271660	42.871580	615.585500	.000263
-93.329110	43.967430	608.031700	5.594892
-92.068780	44.264030	593.964100	12.908400
-90.791210	44.547890	530.994000	34.020570
-89.496510	44.818510	439.292400	71.341690
-88.184940	45.075390	290.775400	79.120480
-86.856880	45.318050	327.480300	106.261600
-85.512890	45.545990	1019.122000	132.583200
-84.153590	45.758730	1847.905000	236.175500
-82.779790	45.955820	2177.030000	261.139700
-81.392350	46.136820	2109.798000	233.467100
-79.992260	46.301310	2019.513000	187.841500
-78.580580	46.448910	1882.768000	143.037500
-77.158460	46.579270	1616.765000	101.494800
-75.727110	46.692070	1528.273000	72.907870
-74.287820	46.787040	1454.259000	118.469400
-72.841890	46.863950	1239.842000	116.490900
-71.390720	46.922590	1365.568000	149.672000
-69.935700	46.962830	1767.438000	258.793200
-68.478260	46.984570	1796.353000	211.629200
-67.019850	46.987740	1229.404000	103.681400
-65.561920	46.972340	816.340700	54.643650
-64.105920	46.938410	730.113900	37.425080
-62.653260	46.886040	755.581200	38.292160
-61.205390	46.815350	749.721600	47.376670
-59.763680	46.726520	765.097700	42.090840
-58.329470	46.619790	794.509300	52.987120
-56.904060	46.495390	796.921600	62.184570
-55.488670	46.353640	788.013500	64.359540
-54.084500	46.194880	770.452100	56.626770
-52.692630	46.019470	710.928000	38.127330
-51.314070	45.827840	653.445300	18.041200
-49.949730	45.620390	626.817700	6.426528
-48.600430	45.397590	618.623400	2.076308
-47.266860	45.159900	616.347700	.650639
-45.949600	44.907820	615.743700	.180561
-44.649100	44.641840	615.610800	.042933
-43.365670	44.362450	615.588300	.008571
-42.099450	44.070140	615.585700	.001424
-40.850440	43.765410	615.585500	.000340
-93.760410	44.864160	612.990500	5.846148
-92.483920	45.170280	605.812100	12.410770
-91.189420	45.463320	556.935400	27.404120

-89.876960	45.742750	414.169200	47.704740
-88.546760	46.008070	343.738600	100.604500
-87.199170	46.258760	386.953600	96.215980
-85.834720	46.494300	868.138100	74.020840
-84.454070	46.714200	1358.333000	115.571300
-83.058010	46.917960	1404.927000	97.831360
-81.647480	47.105130	1270.590000	110.237900
-80.223460	47.275260	1152.904000	128.218500
-78.787090	47.427960	1023.284000	139.980200
-77.339580	47.562840	970.762200	177.428800
-75.882230	47.679570	1219.468000	79.338360
-74.416370	47.777870	1570.148000	106.751000
-72.943440	47.857470	1656.849000	143.304300
-71.464880	47.918190	1652.554000	163.002900
-69.982220	47.959850	1737.355000	170.985900
-68.496960	47.982350	1498.662000	141.273200
-67.010670	47.985630	1050.930000	38.977440
-65.524890	47.969690	738.496000	91.145000
-64.041170	47.934570	774.394000	45.356690
-62.561040	47.880340	860.440400	31.037440
-61.086020	47.807170	925.716700	48.879000
-59.617590	47.715230	992.338000	74.891920
-58.157170	47.604770	1019.941000	102.997300
-56.706150	47.476050	1010.355000	119.922900
-55.265830	47.329400	1002.325000	118.345400
-53.837470	47.165180	953.244100	102.890400
-52.422200	46.983780	816.760600	69.388110
-51.021090	46.785640	694.518900	31.665920
-49.635090	46.571200	637.386800	11.204220
-48.265020	46.340950	620.739600	3.331004
-46.911610	46.095370	616.787400	.949739
-45.575410	45.834990	615.842300	.258641
-44.256860	45.560320	615.630700	.061890
-42.956210	45.271870	615.591600	.012519
-41.673580	44.970160	615.586100	.002147
-40.408870	44.655690	615.585600	.000477
-94.212860	45.757310	628.028100	9.553759
-92.919560	46.073230	634.275700	15.706800
-91.607470	46.375730	627.688800	23.412860
-90.276550	46.664250	584.572100	23.502000
-88.926960	46.938270	551.341600	26.303120
-87.559010	47.197250	629.285100	40.646740
-86.173240	47.440650	849.424200	51.704470
-84.770290	47.667940	1037.221000	73.045890
-83.350960	47.878600	1147.682000	73.627210
-81.916200	48.072160	1209.113000	59.276050
-80.467090	48.248140	1249.344000	41.243900
-79.004780	48.406110	1109.824000	66.495280
-77.530590	48.545680	997.906600	100.819500
-76.045850	48.666500	1221.017000	59.353360
-74.552020	48.768250	1518.305000	123.076200
-73.050600	48.850660	1580.136000	157.530600
-71.543170	48.913520	1562.501000	159.319700
-70.031330	48.956650	1508.083000	161.724800
-68.516710	48.979950	1260.351000	101.784500
-67.000980	48.983350	908.176400	74.189380
-65.485790	48.966840	683.820900	113.650400
-63.972820	48.930470	779.680900	48.532750
-62.463700	48.874330	989.709600	60.872790
-60.960050	48.798580	1156.660000	101.030100
-59.463460	48.703410	1234.610000	125.684200
-57.975440	48.589080	1225.286000	139.852900
-56.497490	48.455880	1175.566000	154.928600
-55.030970	48.304140	1135.827000	147.827700
-53.577210	48.134270	1041.154000	129.300300
-52.137430	47.946660	861.672800	78.034680
-50.712720	47.741790	709.616900	36.266030

-49.304080	47.520120	642.594500	12.715050
-47.912350	47.282170	622.426800	3.882312
-46.538250	47.028450	617.165200	1.135366
-45.182340	46.759510	615.899900	.298091
-43.845020	46.475880	615.638700	.068185
-42.526500	46.178100	615.593100	.013620
-41.226800	45.866700	615.586300	.002285
-39.945740	45.542200	615.585600	.000454
-94.687740	46.646660	652.822300	19.311970
-93.376940	46.972690	670.529200	26.768560
-92.046550	47.284930	689.893100	35.183100
-90.696440	47.582840	687.861600	37.211130
-89.326680	47.865840	711.084800	41.554240
-87.937550	48.133390	797.352800	51.037470
-86.529520	48.384910	924.976000	64.565090
-85.103260	48.619850	1095.501000	79.967560
-83.659580	48.837660	1319.661000	110.443800
-82.199460	49.037830	1562.385000	161.663500
-80.724000	49.219880	1572.637000	168.215600
-79.234450	49.383340	1325.341000	93.082100
-77.732160	49.527770	1180.611000	55.932160
-76.218590	49.652830	1273.814000	90.530300
-74.695260	49.758160	1402.706000	140.720400
-73.163800	49.843490	1419.615000	167.281600
-71.625870	49.908580	1420.836000	170.240800
-70.083210	49.953250	1402.870000	155.076000
-68.537570	49.977380	1241.125000	118.921300
-66.990740	49.980900	988.175000	59.139650
-65.444490	49.963800	780.697100	56.756650
-63.900620	49.926140	761.097500	56.500820
-62.360880	49.868010	889.787200	71.261720
-60.827010	49.789570	1098.800000	109.753500
-59.300730	49.691040	1227.749000	137.469500
-57.783640	49.572690	1218.140000	151.685800
-56.277320	49.434830	1136.478000	141.410100
-54.783270	49.277820	1032.941000	132.866200
-53.302860	49.102080	915.394700	95.262780
-51.837360	48.908040	761.624200	59.522330
-50.387950	48.696200	661.683400	25.928890
-48.955640	48.467040	628.182900	8.209707
-47.541310	48.221130	619.404900	2.722614
-46.145660	47.959000	616.687000	.880868
-44.769230	47.681210	615.820300	.239924
-43.412380	47.388330	615.623000	.054651
-42.075270	47.080920	615.590500	.010262
-40.757810	46.759540	615.586100	.001670
-39.459720	46.424710	615.585600	.000323
-95.186440	47.531940	695.358300	36.349490
-93.857410	47.868410	731.037800	48.654880
-92.508000	48.190720	779.559400	67.194440
-91.137910	48.498310	825.343500	77.451570
-89.747150	48.790600	886.479900	83.316310
-88.335950	49.067010	977.152500	96.170780
-86.904710	49.326930	1075.813000	106.568400
-85.454100	49.569790	1196.703000	123.442700
-83.984930	49.795020	1358.998000	162.508000
-82.498220	50.002060	1472.150000	193.303700
-80.995120	50.190400	1436.339000	180.796500
-79.476920	50.359550	1277.873000	137.104400
-77.945060	50.509060	1216.409000	118.603400
-76.401090	50.638520	1287.874000	136.058400
-74.846650	50.747580	1385.693000	162.982800
-73.283450	50.835950	1368.507000	171.145500
-71.713320	50.903350	1349.075000	162.749600
-70.138080	50.949630	1329.997000	153.322300
-68.559640	50.974620	1238.987000	130.178600
-66.979900	50.978270	1108.200000	97.579950

-65.400800	50.960560	934.168200	79.043590
-63.824260	50.921550	799.200800	64.247880
-62.252170	50.861340	766.032700	79.098690
-60.686400	50.780110	904.601300	97.531230
-59.128770	50.678080	1049.442000	128.794500
-57.581030	50.555550	1073.169000	127.877800
-56.044860	50.412850	980.272700	125.428200
-54.521820	50.250360	874.819500	90.603560
-53.013420	50.068520	771.044200	64.058310
-51.520960	49.867810	682.762900	34.230060
-50.045680	49.648740	635.347100	13.597750
-48.588630	49.411830	620.026900	4.883077
-47.150700	49.157670	616.978300	1.606402
-45.732590	48.886830	616.006500	.511367
-44.334810	48.599900	615.668900	.139847
-42.957660	48.297460	615.596800	.030025
-41.601190	47.980120	615.586800	.005438
-40.265250	47.648430	615.585600	.000807
-38.949390	47.302930	615.585600	.000265
-95.710510	48.412890	772.639300	65.336000
-94.362470	48.760130	847.483000	85.288840
-92.993230	49.092850	936.892300	102.921200
-91.602360	49.410460	1001.430000	114.342400
-90.189750	49.712350	1042.735000	120.811100
-88.755540	49.997940	1067.623000	121.475200
-87.300090	50.266570	1070.049000	120.526900
-85.824040	50.517650	1073.935000	125.315600
-84.328210	50.750570	1113.820000	145.353400
-82.813610	50.964740	1179.247000	166.116700
-81.281460	51.159630	1222.190000	159.920600
-79.733130	51.334700	1200.905000	148.391900
-78.170130	51.489480	1220.170000	140.640000
-76.594090	51.623530	1283.628000	159.488700
-75.006800	51.736490	1341.131000	178.678400
-73.410070	51.828010	1289.826000	171.655900
-71.805870	51.897850	1258.569000	153.154700
-70.196170	51.945790	1242.221000	128.815000
-68.582990	51.971680	1134.151000	100.492700
-66.968440	51.975460	1013.926000	76.941780
-65.354560	51.957120	961.908400	73.891140
-63.743440	51.916690	882.772100	78.720920
-62.137130	51.854320	830.371500	75.370370
-60.537630	51.770170	885.161100	93.623630
-58.946900	51.664510	953.507000	111.730600
-57.366820	51.537620	935.162700	116.177100
-55.799180	51.389870	861.795300	88.729130
-54.245650	51.221680	774.402000	65.857290
-52.707820	51.033510	711.315600	40.304210
-51.187080	50.825860	665.257000	22.184160
-49.684720	50.599280	634.982500	9.992168
-48.201800	50.354330	621.293600	3.522423
-46.739240	50.091630	616.970900	1.134417
-45.297720	49.811770	615.857900	.324823
-43.877700	49.515370	615.627000	.076624
-42.479410	49.203060	615.590900	.015744
-41.102810	48.875440	615.586200	.002501
-39.747590	48.533100	615.585600	.000410
-38.413150	48.176570	615.585600	.000246
-96.261670	49.289190	871.826100	91.431230
-94.893750	49.647600	982.879500	106.527300
-93.503820	49.991080	1058.098000	128.983900
-92.091320	50.319060	1062.995000	126.635400
-90.655950	50.630910	1021.872000	114.305100
-89.197760	50.925990	958.304300	102.434900
-87.717060	51.203660	900.895700	91.422060
-86.214420	51.463270	871.371200	89.678920
-84.690660	51.704190	903.447500	103.279300

-83.146820	51.925780	1019.918000	110.887200
-81.584160	52.127480	1130.661000	133.623300
-80.004110	52.308710	1170.585000	137.347600
-78.408280	52.468990	1162.918000	146.516700
-76.798410	52.607840	1198.790000	161.490100
-75.176390	52.724850	1255.298000	168.974100
-73.544200	52.819690	1204.985000	158.972300
-71.903920	52.892050	1172.456000	138.482300
-70.257710	52.941730	1161.685000	109.223100
-68.607750	52.968570	1032.472000	60.800580
-66.956280	52.972490	877.098600	47.344200
-65.305560	52.953480	896.288300	66.226360
-63.657820	52.911580	959.614600	96.783380
-62.015270	52.846950	952.546000	107.427100
-60.380080	52.759750	930.585900	107.864300
-58.754360	52.650280	896.726800	104.599300
-57.140120	52.518850	839.863800	84.265790
-55.539290	52.365840	766.454200	64.949170
-53.953660	52.191710	709.304100	41.630360
-52.384890	51.996940	671.509700	25.949610
-50.834480	51.782070	645.673600	14.965820
-49.303730	51.547700	627.484200	6.752872
-47.793770	51.294400	619.053300	2.345220
-46.305490	51.022820	616.396700	.699292
-44.839570	50.733620	615.747900	.183251
-43.396390	50.427420	615.614700	.043200
-41.976090	50.104880	615.590200	.008337
-40.578500	49.766630	615.586100	.001388
-39.203150	49.413260	615.585500	.000292
-37.849210	49.045330	615.585600	.000235
-96.841810	50.160530	875.079800	82.745130
-95.453030	50.530490	952.347700	107.349400
-94.041510	50.885130	981.436700	107.570400
-92.606450	51.223850	932.026200	101.298500
-91.147380	51.546020	857.433800	87.041130
-89.664200	51.850960	790.571000	70.214890
-88.157130	52.138020	749.662900	56.819450
-86.626690	52.406500	741.935600	51.616240
-85.073680	52.655720	785.227100	52.413310
-83.499180	52.885050	866.143200	71.928090
-81.904440	53.093850	946.255900	86.210690
-80.290990	53.281520	991.704500	100.712800
-78.660530	53.447530	1008.801000	119.071400
-77.014920	53.591380	1078.728000	137.722300
-75.356160	53.712650	1159.258000	153.828500
-73.686420	53.810940	1122.388000	146.723800
-72.007930	53.885960	1084.102000	128.769900
-70.322990	53.937460	1062.761000	96.528600
-68.634010	53.965280	931.754500	43.567870
-66.943400	53.969350	798.745400	45.245640
-65.253590	53.949630	860.618200	52.478920
-63.567000	53.906200	968.664900	105.299600
-61.886040	53.839190	971.046700	116.333700
-60.213040	53.748820	891.824000	100.848500
-58.550290	53.635360	811.836800	75.646450
-56.899960	53.499180	743.456200	56.685540
-55.264110	53.340690	695.442300	38.215600
-53.644650	53.160350	664.599500	24.701210
-52.043340	52.958710	642.744800	14.490210
-50.461760	52.736320	628.293600	7.420684
-48.901280	52.493820	620.323100	3.106323
-47.363030	52.231850	617.028000	1.094286
-45.847900	51.951060	615.963600	.341840
-44.356530	51.652150	615.672900	.095066
-42.889220	51.335790	615.603100	.022630
-41.445980	51.002650	615.588500	.004675
-40.026490	50.653380	615.585900	.000855

-38.630060	50.288590	615.585500	.000302
-37.255620	49.908830	615.585600	.000225
-97.453060	51.026530	765.217300	59.900030
-96.042340	51.408490	804.454600	62.050160
-94.608220	51.774700	802.241800	71.174010
-93.149600	52.124570	773.075900	66.354160
-91.665800	52.457450	741.446500	56.184570
-90.156560	52.772640	712.279800	46.174780
-88.621980	53.069450	689.695600	36.955720
-87.062480	53.347160	674.478900	29.178860
-85.478850	53.605050	664.885900	26.673900
-83.872130	53.842430	643.444300	37.212880
-82.243660	54.058630	609.933800	60.840510
-80.595020	54.253030	634.086800	57.878330
-78.928000	54.425040	777.733600	54.835220
-77.244600	54.574130	957.249300	105.356900
-75.546970	54.699840	1072.396000	136.103300
-73.837420	54.801750	1043.912000	133.302500
-72.118360	54.879540	987.724000	107.689600
-70.392330	54.932960	918.823100	71.404180
-68.661900	54.961820	794.205400	47.697610
-66.929700	54.966030	750.119400	46.846150
-65.198370	54.945580	831.250400	54.730590
-63.470540	54.900540	899.307100	93.008700
-61.748820	54.831050	869.690700	90.424670
-60.035740	54.737340	784.979400	67.290410
-58.333780	54.619720	712.821400	44.689330
-56.645260	54.478570	672.480700	27.268320
-54.972420	54.314330	652.677000	18.345150
-53.317280	54.127510	642.122900	14.425910
-51.681740	53.918690	631.322600	9.342273
-50.067420	53.688470	622.148200	4.302133
-48.475770	53.437500	617.783800	1.560864
-46.907930	53.166490	616.261500	.531795
-45.364770	52.876130	615.773700	.169132
-43.846840	52.567130	615.630400	.048111
-42.354370	52.240220	615.594400	.012017
-40.887210	51.896080	615.586900	.002574
-39.444820	51.535370	615.585700	.000555
-38.026250	51.158730	615.585400	.000256
-36.630120	50.766700	615.585600	.000224
-98.097820	51.886770	674.356600	27.584180
-96.663910	52.281200	683.829200	30.505760
-95.206040	52.659450	684.442000	32.125340
-93.722790	53.020900	682.935600	32.758280
-92.213200	53.364910	682.915300	34.643830
-90.676770	53.690770	682.063000	36.033760
-89.113440	53.997730	677.795800	34.712970
-87.523560	54.285050	666.569200	28.526010
-85.907830	54.551970	635.511200	16.075470
-84.267300	54.797760	548.917400	41.979100
-82.603340	55.021710	403.580000	103.282300
-80.917590	55.223140	353.211500	140.054600
-79.211960	55.401440	514.080000	74.423750
-77.488560	55.556020	836.401700	70.112020
-75.749730	55.686380	996.024700	119.000900
-73.997920	55.792100	968.615800	113.598200
-72.235800	55.872810	883.519800	77.854560
-70.466090	55.928230	763.168000	57.541250
-68.691570	55.958180	694.268700	63.604820
-66.915130	55.962550	749.534100	48.767830
-65.139640	55.941330	820.019600	68.463470
-63.367970	55.894590	808.188400	71.637220
-61.602940	55.822490	750.605000	57.353560
-59.847310	55.725280	693.904500	37.408420
-58.103760	55.603290	654.681600	22.736680
-56.374820	55.456930	633.443500	12.305690

-54.662870	55.286670	626.188700	7.490475
-52.970100	55.093080	627.664200	8.014906
-51.298500	54.876750	626.562700	7.836827
-49.649800	54.638340	621.061800	4.202319
-48.025460	54.378550	617.347300	1.439595
-46.426660	54.098120	616.092000	.426872
-44.854210	53.797780	615.724900	.128545
-43.308580	53.478290	615.618700	.036053
-41.789850	53.140400	615.591700	.009030
-40.297680	52.784830	615.586300	.001938
-38.831280	52.412250	615.585600	.000455
-37.389370	52.023280	615.585400	.000274
-35.970190	51.618480	615.585600	.000232
-98.778840	52.740810	635.271500	10.594410
-97.320270	53.148240	636.052100	11.028590
-95.837370	53.539010	636.402600	11.574770
-94.328290	53.912530	637.924400	13.177060
-92.791720	54.268120	642.704700	16.459810
-91.226900	54.605080	650.913800	21.350910
-89.633560	54.922620	659.524900	26.351820
-88.011890	55.219970	664.026700	29.178070
-86.362530	55.496330	653.982400	25.809000
-84.686490	55.750910	601.353700	24.676560
-82.985150	55.982970	450.937700	80.999270
-81.260250	56.191770	291.213800	149.733400
-79.513790	56.376650	382.707200	124.613900
-77.748020	56.536990	728.462000	56.824390
-75.965460	56.672260	922.427000	102.003400
-74.168790	56.781970	901.614700	96.161960
-72.360860	56.865740	823.887800	65.572410
-70.544630	56.923280	724.373700	56.761110
-68.723180	56.954370	714.001600	54.061880
-66.899620	56.958910	810.450000	68.078680
-65.077100	56.936880	815.971900	70.525730
-63.258730	56.888360	732.199600	53.321270
-61.447630	56.813510	669.789900	31.586550
-59.646790	56.712620	644.758500	20.029560
-57.859090	56.586040	637.531600	14.139790
-56.087300	56.434210	624.760400	7.565304
-54.333970	56.257640	619.262000	3.674148
-52.601470	56.056940	623.106300	5.681867
-50.891880	55.832740	625.580200	7.958280
-49.207030	55.585770	622.113600	5.359091
-47.548420	55.316760	617.853200	2.053090
-45.917200	55.026500	616.217200	.582829
-44.314130	54.715760	615.759800	.167390
-42.739580	54.385350	615.629100	.047232
-41.193420	54.036030	615.594400	.011862
-39.675070	53.668560	615.586800	.002617
-38.183400	53.283620	615.585600	.000592
-36.716770	52.881820	615.585400	.000332
-35.272900	52.463700	615.585600	.000233
-99.499240	53.588130	621.617600	3.931886
-98.014280	54.009130	621.249700	3.735677
-96.504860	54.412960	621.241100	3.863399
-94.968610	54.799050	621.636800	4.350112
-93.403790	55.166730	623.343600	5.656590
-91.809290	55.515250	627.141300	8.238212
-90.184560	55.843850	633.615200	12.291100
-88.529620	56.151670	642.661900	17.517740
-86.844990	56.437890	649.739000	22.349840
-85.131650	56.701690	642.168900	24.199220
-83.390960	56.942230	579.618700	36.485660
-81.624720	57.158780	427.775700	80.898960
-79.835050	57.350580	444.755200	87.678040
-78.024370	57.516980	704.782500	62.910370
-76.195360	57.657400	853.664100	92.610340

-74.350940 57.771310 856.124700 86.542460
-72.494220 57.858320 825.456200 69.222960
-70.628410 57.918090 776.829000 54.289670
-68.756890 57.950390 784.988600 60.467270
-66.883060 57.955100 842.317800 77.353230
-65.010370 57.932220 785.674100 66.379110
-63.142240 57.881810 691.252600 36.990990
-61.282040 57.804080 639.122700 18.046420
-59.433060 57.699310 628.975200 11.754300
-57.598450 57.567890 627.871000 11.564710
-55.781200 57.410300 619.381100 7.351377
-53.984060 57.227100 616.332900 2.647436
-52.209560 57.018940 620.232000 3.367865
-50.459930 56.786500 624.842600 7.541170
-48.737060 56.530560 623.961100 6.924805
-47.042490 56.251900 619.286600 3.236207
-45.377330 55.951360 616.655800 .984541
-43.742270 55.629770 615.862900 .263139
-42.137470 55.287960 615.654800 .070886
-40.562610 54.926740 615.600500 .017932
-39.016750 54.546860 615.588000 .004042
-37.498410 54.149020 615.585800 .000825
-36.005400 53.733830 615.585500 .000332
-34.534880 53.301770 615.585600 .000230
-100.262700 54.428120 617.196300 1.317696
-98.749250 54.863350 616.982500 1.193809
-97.211560 55.280850 616.961400 1.195513
-95.646590 55.680060 617.074900 1.324115
-94.052090 56.060360 617.521900 1.687308
-92.426510 56.420970 618.555300 2.459561
-90.768940 56.761100 620.621700 3.903095
-89.079190 57.079890 624.230800 6.380398
-87.357570 57.376450 629.408200 10.181170
-85.605000 57.649890 635.063400 15.564020
-83.822840 57.899360 621.342700 18.896730
-82.012920 58.124030 561.761000 30.471890
-80.177480 58.323120 560.871600 35.016650
-78.319110 58.495910 738.645900 71.073230
-76.440710 58.641750 801.198100 91.196360
-74.545430 58.760110 826.721300 83.477460
-72.636660 58.850530 853.485800 74.651030
-70.717930 58.912650 828.806600 67.116430
-68.792920 58.946230 827.333600 68.924360
-66.865380 58.951130 818.024200 74.676030
-64.939080 58.927340 743.997100 52.967790
-63.017780 58.874950 672.405500 26.456180
-61.105210 58.794160 634.306300 11.656070
-59.204930 58.685300 626.302100 8.766143
-57.320400 58.548780 624.851000 10.196180
-55.454840 58.385130 615.888900 8.261054
-53.611270 58.194950 611.494000 5.704561
-51.792370 57.978940 615.229700 1.074832
-50.000500 57.737840 622.065900 5.175005
-48.237630 57.472490 624.729900 7.621702
-46.505310 57.183730 620.737200 4.352111
-44.804610 56.872430 617.147600 1.422636
-43.136050 56.539500 615.974100 .364386
-41.499610 56.185780 615.682000 .095027
-39.894630 55.812120 615.607600 .024398
-38.319800 55.419290 615.589500 .005648
-36.773090 55.007980 615.586000 .001177
-35.251730 54.578750 615.585500 .000349
-33.752120 54.132060 615.585600 .000230
-101.073700 55.260130 615.945500 .393493
-99.529010 55.710320 615.874800 .335379
-97.960900 56.142140 615.872700 .330814
-96.365430 56.555100 615.918200 .372205

-94.739640 56.948590 616.056700 .485642
-93.081440 57.321860 616.355700 .713056
-91.389490 57.674070 616.923600 1.123591
-89.663250 58.004340 617.894500 1.866590
-87.902820 58.311730 619.629100 3.492070
-86.109000 58.595320 622.055400 7.264083
-84.283100 58.854170 614.835700 11.898000
-82.427000 59.087400 593.970600 16.412650
-80.543050 59.294160 635.640400 24.442630
-78.634010 59.473690 739.438400 70.563550
-76.703000 59.625280 735.443200 75.742260
-74.753460 59.748340 767.231800 73.717860
-72.789070 59.842370 824.731600 73.746760
-70.813740 59.906970 838.614100 66.636720
-68.831480 59.941910 831.231800 72.683990
-66.846440 59.947010 793.625500 64.769510
-64.862770 59.922250 717.046100 41.785550
-62.884610 59.867760 656.311800 19.491160
-60.916040 59.783740 629.308900 7.893499
-58.961010 59.670550 625.713600 5.994998
-57.023270 59.528640 624.815600 7.420370
-55.106350 59.358580 615.283700 6.950024
-53.213500 59.161040 609.303800 7.311824
-51.347600 58.936760 609.819100 5.318610
-49.511150 58.686560 616.182600 .481179
-47.706190 58.411320 622.251200 5.523380
-45.934240 58.111950 620.740400 4.319508
-44.196280 57.789400 617.321600 1.607400
-42.492650 57.444580 616.024600 .412258
-40.823030 57.078400 615.697800 .108106
-39.186360 56.691730 615.612700 .028646
-37.580830 56.285340 615.590900 .006890
-36.003750 55.859910 615.586300 .001474
-34.451600 55.415950 615.585600 .000422
-32.919830 54.953830 615.585600 .000233
-101.937200 56.083360 615.651700 .101372
-100.358100 56.549350 615.634800 .081469
-98.756940 56.996240 615.635800 .079584
-97.128790 57.423640 615.650900 .093867
-95.469860 57.830970 615.693700 .132074
-93.777320 58.217490 615.789900 .210168
-92.049300 58.582370 615.981700 .355803
-90.284800 58.924680 616.337000 .701276
-88.483640 59.243460 617.124600 1.965356
-86.646400 59.537720 617.697200 5.863836
-84.774360 59.806450 608.033400 12.205750
-82.869400 60.048700 593.679100 17.235920
-80.933970 60.263580 645.693900 19.507610
-78.971000 60.450230 704.175700 51.829400
-76.983890 60.607900 645.274300 54.813960
-74.976370 60.735940 632.451800 55.568040
-72.952470 60.833800 702.917700 51.699190
-70.916480 60.901050 778.394200 55.727070
-68.872850 60.937420 821.884600 66.466560
-66.826130 60.942730 780.638300 58.754490
-64.780930 60.916960 697.239400 33.613160
-62.741830 60.860230 638.895400 12.366130
-60.713310 60.772790 617.414500 3.842017
-58.699720 60.654990 617.365000 1.600557
-56.705200 60.507380 619.993400 3.198690
-54.733590 60.330550 615.585600 3.346311
-52.788400 60.125220 610.448100 5.207848
-50.872730 59.892220 608.670900 6.109964
-48.989200 59.632420 611.668200 3.569155
-47.139900 59.346780 617.394800 1.636249
-45.326320 59.036270 619.272700 3.000574
-43.549270 58.701890 617.451700 1.626665

-41.808860	58.344600	616.119500	.497462
-40.104370	57.965370	615.713700	.121310
-38.434220	57.565060	615.615700	.030724
-36.795950	57.144450	615.591700	.007368
-35.186060	56.704150	615.586500	.001614
-33.600030	56.244660	615.585600	.000405
-32.032190	55.766200	615.585600	.000225
-102.859900	56.896880	615.595500	.022290
-101.242000	57.379640	615.592300	.016648
-99.604390	57.842470	615.592700	.016480
-97.940930	58.285070	615.596700	.021359
-96.246660	58.706940	615.609000	.034704
-94.517820	59.107400	615.641200	.065283
-92.751850	59.485580	615.714500	.147059
-90.947170	59.840560	615.873100	.453172
-89.103220	60.171320	616.276600	1.732985
-87.220290	60.476800	615.771400	5.868732
-85.299550	60.755960	604.484300	13.361530
-83.342830	61.007760	591.205100	18.055390
-81.352710	61.231220	636.868500	11.962180
-79.332320	61.425420	666.207600	27.880230
-77.285290	61.589540	557.109700	49.664940
-75.215710	61.722870	485.925800	106.579700
-73.128000	61.824790	519.699300	103.451400
-71.026890	61.894870	638.178000	63.130350
-68.917300	61.932760	775.580300	55.978840
-66.804310	61.938290	760.609100	51.239130
-64.692990	61.911450	676.073700	25.594200
-62.588420	61.852340	622.994800	8.001216
-60.495590	61.761240	607.284500	5.869918
-58.419300	61.638580	609.204900	3.396154
-56.364090	61.484900	614.376000	.772028
-54.334160	61.300890	614.941800	1.093608
-52.333320	61.087320	613.324300	2.323554
-50.364880	60.845090	611.255500	3.790441
-48.431600	60.575160	611.021700	3.772661
-46.535590	60.278550	614.756300	.984239
-44.678250	59.956310	617.146400	1.486412
-42.860180	59.609490	617.104300	1.219402
-41.081100	59.239120	616.110400	.473541
-39.339830	58.846180	615.708900	.118612
-37.634110	58.431540	615.612600	.026798
-35.960610	57.995940	615.591200	.006471
-34.314830	57.539980	615.586400	.001399
-32.690990	57.064010	615.585600	.000394
-31.082000	56.568130	615.585600	.000217
-103.849600	57.699570	615.586700	.004061
-102.187300	58.200280	615.586300	.002838
-100.509000	58.680030	615.586300	.002953
-98.806840	59.138710	615.587300	.004695
-97.074520	59.575940	615.591000	.010151
-95.307120	59.991050	615.602300	.028698
-93.501110	60.383240	615.627000	.101100
-91.654170	60.751550	615.654400	.397435
-89.765210	61.094930	615.599400	1.631615
-87.834170	61.412270	614.082200	5.749668
-85.861950	61.702450	604.790100	12.730240
-83.850420	61.964360	599.376800	14.679440
-81.802150	62.196920	633.875600	9.892659
-79.720490	62.399150	632.539200	18.622900
-77.609360	62.570130	525.179100	69.398310
-75.473210	62.709080	416.804500	131.758900
-73.316960	62.815350	389.285500	155.719200
-71.145790	62.888430	503.831000	103.502700
-68.965200	62.927950	667.146800	46.983890
-66.780790	62.933720	708.072600	33.099610
-64.598260	62.905720	652.761900	16.599170

-62.423240	62.844070	616.254400	5.653153
-60.261290	62.749080	608.096900	4.559588
-58.117710	62.621230	610.773900	2.696611
-55.997520	62.461110	614.130000	.829163
-53.905320	62.269450	615.066900	.382440
-51.845240	62.047150	614.838600	.722821
-49.820820	61.795150	614.033000	1.431622
-47.834930	61.514500	613.353900	1.965086
-45.889670	61.206310	614.148300	1.154640
-43.986290	60.871700	616.028900	.356125
-42.125070	60.511780	616.084200	.489842
-40.305270	60.127630	615.760700	.175217
-38.525020	59.720250	615.636300	.047702
-36.781200	59.290500	615.599500	.014546
-35.069400	58.839090	615.588600	.003765
-33.383770	58.366500	615.586000	.000909
-31.716990	57.872960	615.585500	.000337
-30.060130	57.358350	615.585600	.000210
-104.916500	58.490070	615.585700	.000651
-103.202300	59.010120	615.585600	.000417
-101.477600	59.507980	615.585600	.000574
-99.732440	59.983750	615.585800	.001307
-97.958720	60.437220	615.586900	.004752
-96.150010	60.867820	615.590900	.019997
-94.301540	61.274790	615.593000	.085015
-92.410060	61.657170	615.539200	.350161
-90.473700	62.013880	615.161300	1.479883
-88.491920	62.343760	613.789400	4.865671
-86.465330	62.645610	611.852100	9.726957
-84.395630	62.918240	619.502900	8.645502
-82.285480	63.160470	640.231000	13.223180
-80.138370	63.371240	623.610000	13.920680
-77.958560	63.549540	545.972900	49.689000
-75.750930	63.694510	465.908900	100.738500
-73.520860	63.805420	422.358400	126.621200
-71.274150	63.881700	467.523300	102.584600
-69.016910	63.922960	596.377100	40.041550
-66.755400	63.928990	640.483100	17.657240
-64.495980	63.899750	626.701800	7.061009
-62.244980	63.835380	615.141000	2.619483
-60.008560	63.736250	613.085700	1.860992
-57.792640	63.602840	614.002700	1.001648
-55.602750	63.435840	614.984100	.357784
-53.443960	63.236060	615.334200	.161649
-51.320750	63.004450	615.341300	.213394
-49.236890	62.742080	615.167700	.388644
-47.195330	62.450080	614.949500	.595653
-45.198120	62.129630	614.963700	.551724
-43.246250	61.781950	615.534900	.081578
-41.339580	61.408210	615.638200	.077865
-39.476710	61.009530	615.622100	.034072
-37.654880	60.586900	615.602100	.016888
-35.869840	60.141170	615.590600	.005691
-34.115770	59.672970	615.586700	.001701
-32.385130	59.182660	615.585700	.000506
-30.668580	58.670220	615.585600	.000318
-28.954870	58.135290	615.585600	.000208
-106.073800	59.266680	615.585600	.000237
-104.297500	59.807790	615.585600	.000244
-102.518800	60.325180	615.585500	.000220
-100.725000	60.819250	615.585600	.000641
-98.905530	61.289990	615.585800	.003025
-97.052100	61.737010	615.586800	.014701
-95.158340	62.159620	615.580600	.064105
-93.219700	62.556880	615.505200	.273510
-91.233350	62.927700	615.165900	1.081586
-89.198040	63.270870	614.863200	3.517095

-87.113920	63.585110	617.309000	5.711234
-84.982480	63.869130	622.561000	4.779827
-82.806390	64.121680	625.325200	7.711082
-80.589290	64.341560	599.027100	13.279650
-78.335770	64.527670	558.965800	31.915300
-76.051160	64.679080	528.872100	52.032390
-73.741460	64.794950	513.347300	66.406640
-71.413090	64.874680	530.757100	56.347750
-69.072890	64.917820	588.634900	24.589010
-66.727910	64.924110	616.127900	8.345132
-64.385280	64.893550	617.515300	2.900292
-62.052090	64.826280	615.570100	1.019032
-59.735260	64.722690	615.039400	.537004
-57.441380	64.583340	615.152300	.289247
-55.176610	64.408980	615.372300	.137157
-52.946510	64.200520	615.487900	.067104
-50.755950	63.959000	615.507600	.067179
-48.608900	63.685590	615.476100	.102257
-46.508400	63.381530	615.436800	.138495
-44.456330	63.048090	615.429000	.148560
-42.453320	62.686580	615.534300	.046532
-40.498620	62.298210	615.583200	.008268
-38.589950	61.884150	615.593000	.008110
-36.723370	61.445380	615.589700	.004487
-34.893150	60.982680	615.586900	.001868
-33.091610	60.496560	615.585800	.000596
-31.309040	59.987160	615.585500	.000320
-29.533510	59.454220	615.585500	.000212
-27.750760	58.896990	615.585600	.000201
-107.339200	60.027230	615.585600	.000240
-105.487000	60.591610	615.585600	.000244
-103.643800	61.130270	615.585600	.000250
-101.793500	61.644070	615.585400	.000378
-99.922550	62.133320	615.585400	.001926
-98.020040	62.597820	615.584800	.009335
-96.077510	63.037030	615.577600	.040278
-94.088710	63.450070	615.530500	.147295
-92.049460	63.835860	615.290300	.556279
-89.957580	64.193130	614.585000	1.458274
-87.812570	64.520550	614.042600	1.500445
-85.615560	64.816710	613.255700	3.537069
-83.369120	65.080260	605.908000	9.036897
-81.077050	65.309890	589.616400	14.897630
-78.744270	65.504390	578.799200	19.986890
-76.376630	65.662700	575.899600	23.268730
-73.980770	65.783920	577.639600	24.907770
-71.563890	65.867360	586.716200	20.665890
-69.133670	65.912500	604.099900	10.272940
-66.698060	65.919100	613.671400	3.923428
-64.265090	65.887100	615.564900	1.211728
-61.842790	65.816700	615.510000	.438108
-59.438900	65.708320	615.424900	.183755
-57.060850	65.562580	615.450500	.102330
-54.715470	65.380330	615.514800	.053766
-52.408880	65.162570	615.554400	.026893
-50.146370	64.910470	615.563800	.020900
-47.932120	64.625290	615.557100	.028036
-45.769150	64.308400	615.549400	.035045
-43.659080	63.961160	615.551000	.032652
-41.602020	63.584970	615.569200	.016193
-39.596350	63.181100	615.582900	.002479
-37.638600	62.750720	615.586300	.001044
-35.723270	62.294770	615.586100	.001031
-33.842640	61.813940	615.585700	.000487
-31.986660	61.308540	615.585600	.000279
-30.142740	60.778430	615.585400	.000240
-28.295590	60.222970	615.585600	.000244

-26.427080 59.640870 615.585600 .000193
-108.736700 60.768890 615.585600 .000241
-106.789100 61.359350 615.585600 .000241
-104.866900 61.921510 615.585600 .000252
-102.949500 62.456830 615.585400 .000346
-101.019200 62.966030 615.585200 .001650
-99.061880 63.449270 615.583200 .007671
-97.066180 63.906190 615.572000 .028978
-95.023590 64.336020 615.518200 .096791
-92.928180 64.737730 615.313500 .258222
-90.776390 65.110020 614.669300 .702292
-88.566850 65.451470 613.142000 1.455493
-86.300130 65.760610 609.810400 4.476010
-83.978550 66.035940 602.391900 8.276533
-81.606060 66.276030 596.713100 10.897930
-79.187900 66.479550 596.571400 11.414090
-76.730480 66.645290 598.413100 11.060460
-74.241170 66.772270 600.888500 10.287580
-71.728080 66.859700 605.065600 8.069163
-69.199870 66.907030 610.485300 4.621439
-66.665550 66.913940 614.006200 1.961161
-64.134220 66.880400 615.181000 .767004
-61.614990 66.806620 615.447800 .276619
-59.116610 66.693070 615.496700 .111240
-56.647430 66.540450 615.528000 .054580
-54.215080 66.349720 615.557600 .028324
-51.826330 66.121990 615.574000 .013727
-49.486860 65.858530 615.578500 .008714
-47.201050 65.560780 615.576500 .010088
-44.971820 65.230190 615.574100 .012029
-42.800360 64.868270 615.574800 .010992
-40.685980 64.476460 615.579700 .005856
-38.625890 64.056100 615.583600 .001904
-36.614990 63.608330 615.585200 .000279
-34.645670 63.134020 615.585600 .000308
-32.707610 62.633670 615.585600 .000267
-30.787590 62.107330 615.585600 .000240
-28.869240 61.554470 615.585600 .000244
-26.932890 60.973880 615.585600 .000241
-24.955330 60.363580 615.585600 .000186

Q.28a
-89.453400 32.991660 648.505500 .048695
-88.345930 33.191160 648.517900 .065804
-87.228100 33.381660 648.565700 .132307

-86.100230	33.562890	648.658000	.247813
-84.962690	33.734530	648.802400	.408988
-83.815900	33.896310	648.996200	.599116
-82.660300	34.047950	649.200000	.779792
-81.496410	34.189190	649.332200	.900153
-80.324730	34.319790	649.325600	.904882
-79.145840	34.439500	649.168400	.725010
-77.960310	34.548100	648.766900	.298018
-76.768760	34.645400	648.011000	.436993
-75.571840	34.731200	647.035900	1.062027
-74.370180	34.805370	646.750000	1.239820
-73.164480	34.867740	647.244500	1.025513
-71.955430	34.918200	647.893900	.611685
-70.743730	34.956650	648.278500	.269976
-69.530080	34.983020	648.453600	.068950
-68.315220	34.997260	648.516700	.028733
-67.099880	34.999340	648.524000	.040735
-65.884770	34.989250	648.509100	.029990
-64.670640	34.967020	648.495700	.016073
-63.458190	34.932690	648.489200	.007242
-62.248150	34.886320	648.487300	.003474
-61.041230	34.828000	648.486800	.002215
-59.838120	34.757850	648.486700	.001836
-58.639500	34.676010	648.486600	.001536
-57.446040	34.582610	648.486400	.001115
-56.258390	34.477850	648.486100	.000677
-55.077160	34.361910	648.486000	.000363
-53.902950	34.235010	648.486000	.000238
-52.736340	34.097380	648.486000	.000239
-51.577860	33.949260	648.486000	.000244
-50.428010	33.790910	648.486000	.000244
-49.287250	33.622600	648.486000	.000244
-48.156040	33.444620	648.486000	.000244
-47.034740	33.257250	648.486000	.000244
-45.923690	33.060790	648.486000	.000244
-44.823220	32.855560	648.486000	.000244
-89.701210	33.918400	648.580200	.159830
-88.583520	34.125020	648.645400	.222764
-87.455040	34.322360	648.865000	.426539
-86.316120	34.510110	649.261900	.762972
-85.167100	34.687960	649.856200	1.216449
-84.008420	34.855620	650.616700	1.732428
-82.840550	35.012790	651.403200	2.222024
-81.663990	35.159200	651.928600	2.556901
-80.479300	35.294580	651.951000	2.588088
-79.287040	35.418700	651.362900	2.137543
-78.087840	35.531310	649.867200	1.086464
-76.882320	35.632210	647.162700	.937067
-75.671150	35.721200	643.657700	2.434716
-74.455030	35.798110	642.622000	2.805263
-73.234660	35.862810	644.335000	2.241871
-72.010770	35.915150	646.462300	1.358685
-70.784100	35.955040	647.793800	.571754
-69.555380	35.982390	648.414100	.126548
-68.325390	35.997170	648.643600	.120320
-67.094890	35.999320	648.660500	.141683
-65.864640	35.988860	648.592200	.099873
-64.635410	35.965800	648.532000	.054005
-63.407950	35.930180	648.503500	.026774
-62.183030	35.882080	648.494000	.014361
-60.961370	35.821590	648.491300	.009680
-59.743720	35.748840	648.490500	.007694
-58.530780	35.663950	648.489700	.006241
-57.323260	35.567100	648.488200	.004410
-56.121820	35.458470	648.487000	.002629
-54.927120	35.338260	648.486400	.001299
-53.739770	35.206700	648.486100	.000560

-52.560380	35.064020	648.486000	.000265
-51.389480	34.910500	648.486000	.000254
-50.227610	34.746390	648.485800	.000244
-49.075240	34.571990	648.485900	.000299
-47.932830	34.387580	648.486000	.000244
-46.800740	34.193490	648.486000	.000244
-45.679330	33.990000	648.485800	.000244
-44.568900	33.777440	648.485900	.000299
-89.960660	34.843320	649.002100	.598133
-88.832330	35.057210	649.359300	.843183
-87.692770	35.261540	650.434600	1.538884
-86.542320	35.455950	652.281900	2.644443
-85.381340	35.640140	654.919100	4.069432
-84.210270	35.813810	658.147000	5.651601
-83.029580	35.976620	661.443100	7.147096
-81.839790	36.128310	663.726300	8.204745
-80.641480	36.268600	663.946900	8.343516
-79.435230	36.397220	661.616300	7.121155
-78.221690	36.513940	655.461700	4.093680
-77.001530	36.618520	645.317100	2.730993
-75.775430	36.710780	631.142300	6.364589
-74.544130	36.790520	628.938700	6.970908
-73.308360	36.857590	633.884800	5.433506
-72.068890	36.911860	641.329700	3.129840
-70.826490	36.953220	646.090600	1.285387
-69.581950	36.981590	648.486500	.313786
-68.336070	36.996900	649.360800	.578972
-67.089650	36.999130	649.364600	.581084
-65.843500	36.988290	649.013200	.387472
-64.598400	36.964370	648.725800	.212255
-63.355190	36.927440	648.588600	.112019
-62.114630	36.877570	648.538600	.066776
-60.877510	36.814860	648.521700	.046585
-59.644590	36.739430	648.514500	.036922
-58.416640	36.651430	648.507600	.028965
-57.194370	36.551040	648.499600	.020128
-55.978490	36.438440	648.493300	.011601
-54.769680	36.313860	648.489200	.005702
-53.568590	36.177530	648.486900	.002313
-52.375820	36.029710	648.486100	.000772
-51.191970	35.870660	648.486000	.000364
-50.017550	35.700660	648.485800	.000350
-48.853080	35.520030	648.485800	.000360
-47.698980	35.329070	648.485900	.000299
-46.555660	35.128100	648.485900	.000299
-45.423470	34.917440	648.485800	.000244
-44.302690	34.697410	648.485900	.000303
-90.232250	35.766320	650.701000	1.816707
-89.092860	35.987640	652.235500	2.597548
-87.941790	36.199090	656.460400	4.622883
-86.779330	36.400320	663.418500	7.705309
-85.605870	36.591000	673.041900	11.571690
-84.421870	36.770790	684.260700	15.712390
-83.227800	36.939400	695.878100	19.679690
-82.024180	37.096500	704.319100	22.583940
-80.811620	37.241800	705.276200	23.177200
-79.590740	37.375040	697.778400	20.294770
-78.362190	37.495960	678.048600	13.419150
-77.126670	37.604320	642.928500	8.427832
-75.884930	37.699920	595.369300	15.381830
-74.637700	37.782550	599.021900	16.172650
-73.385770	37.852060	613.376700	12.579380
-72.129940	37.908310	629.582100	6.113792
-70.871030	37.951180	642.831700	2.337301
-69.609860	37.980580	650.364200	1.705415
-68.347290	37.996460	652.868300	2.445094
-67.084140	37.998780	652.401700	2.147368

-65.821270	37.987530	650.680300	1.330748
-64.559530	37.962740	649.494300	.716397
-63.299760	37.924460	648.969200	.404156
-62.042780	37.872770	648.763900	.259166
-60.789440	37.807780	648.674700	.191796
-59.540510	37.729610	648.628000	.152878
-58.296810	37.638420	648.590700	.119858
-57.059080	37.534390	648.549300	.080321
-55.828080	37.417740	648.522500	.045504
-54.604500	37.288680	648.502100	.021367
-53.389020	37.147460	648.491100	.008113
-52.182290	36.994370	648.487100	.002641
-50.984880	36.829660	648.486100	.000737
-49.797380	36.653650	648.485800	.000361
-48.620270	36.466650	648.485800	.000368
-47.454020	36.268990	648.485800	.000360
-46.299010	36.060990	648.485800	.000360
-45.155610	35.843010	648.485800	.000244
-44.024070	35.615370	648.485800	.000244
-90.516530	36.687310	655.834000	4.502555
-89.365660	36.916220	661.105200	6.637055
-88.202590	37.134950	674.986600	11.827850
-87.027650	37.343150	697.336700	19.533730
-85.841200	37.540460	727.339800	28.818710
-84.643710	37.726540	760.321700	37.999860
-83.435650	37.901050	795.005900	46.347530
-82.217600	38.063690	822.259800	53.095920
-80.990140	38.214140	824.981900	55.039100
-79.753940	38.352110	808.053000	50.859870
-78.509670	38.477340	758.258900	36.255500
-77.258070	38.589580	645.979600	20.579710
-75.999910	38.688600	495.162800	32.355400
-74.735980	38.774210	520.082400	60.158740
-73.467090	38.846230	595.507400	30.825050
-72.194090	38.904510	619.084900	14.099370
-70.917830	38.948930	641.097200	6.487826
-69.639210	38.979390	662.658800	7.860445
-68.359090	38.995840	668.719100	9.125281
-67.078360	38.998240	664.777300	7.429252
-65.797930	38.986590	656.520100	4.081118
-64.518690	38.960900	652.024900	2.107935
-63.241520	38.921240	650.348300	1.267153
-61.967320	38.867690	649.678700	.889343
-60.696920	38.800350	649.329500	.682666
-59.431210	38.719360	649.123600	.561692
-58.170970	38.624900	648.952600	.441273
-56.917050	38.517150	648.749100	.285540
-55.670200	38.396340	648.631600	.160745
-54.431160	38.262690	648.547100	.073048
-53.200640	38.116470	648.505600	.025775
-51.979310	37.957960	648.490300	.006613
-50.767770	37.787460	648.486500	.001687
-49.566610	37.605300	648.485700	.000396
-48.376340	37.411790	648.485800	.000412
-47.197410	37.207270	648.485800	.000380
-46.030250	36.992090	648.485800	.000364
-44.875180	36.766620	648.485800	.000244
-43.732470	36.531200	648.485800	.000244
-90.814130	37.606180	667.465100	9.351631
-89.651300	37.842850	683.167900	14.637290
-88.475770	38.069030	725.144600	27.522040
-87.287830	38.284350	789.981100	45.049580
-86.087840	38.488450	871.040700	63.510530
-84.876290	38.680970	954.273500	79.462040
-83.653650	38.861550	1039.197000	89.405430
-82.420500	39.029860	1114.231000	100.313300
-81.177470	39.185590	1125.184000	107.083200

-79.925230 39.328410 1103.467000 105.131300
-78.664510 39.458070 1017.755000 85.442250
-77.396060 39.574290 753.253700 46.011770
-76.120680 39.676830 310.960700 95.239370
-74.839220 39.765490 334.885000 57.910140
-73.552520 39.840080 585.834700 97.673050
-72.261480 39.900440 634.251800 33.390630
-70.967010 39.946460 658.495800 23.843690
-69.670030 39.978020 710.416300 24.316730
-68.371470 39.995060 725.414400 27.671030
-67.072270 39.997550 706.349100 21.486350
-65.773400 39.985470 673.628200 10.781380
-64.475770 39.958870 659.273900 5.524499
-63.180340 39.917780 654.851600 3.622146
-61.888020 39.862300 652.979000 2.766512
-60.599740 39.792560 651.946400 2.318781
-59.316390 39.708680 651.281600 2.050899
-58.038840 39.610860 650.575600 1.683338
-56.767930 39.499290 649.604400 1.093363
-55.504470 39.374200 648.965100 .444571
-54.249260 39.235840 648.651300 .186701
-53.003000 39.084480 648.533800 .064759
-51.766410 38.920440 648.496800 .015543
-50.540120 38.744010 648.487100 .002366
-49.324710 38.555530 648.485700 .000472
-48.120720 38.355340 648.485400 .000104
-46.928620 38.143810 648.485700 .000294
-45.748810 37.921300 648.485800 .000370
-44.581620 37.688170 648.485800 .000247
-43.427300 37.444810 648.485800 .000244
-91.125630 38.522790 687.914300 16.602740
-89.950410 38.767390 730.537700 29.029320
-88.761920 39.001200 841.818000 55.147180
-87.560450 39.223820 989.903000 88.398510
-86.346370 39.434880 1138.049000 118.816500
-85.120160 39.633990 1298.421000 134.756100
-83.882300 39.820790 1467.392000 140.353800
-82.633390 39.994930 1629.851000 155.628500
-81.374080 40.156070 1687.158000 163.363100
-80.105060 40.303890 1699.411000 175.266800
-78.827100 40.438090 1648.970000 172.127900
-77.540980 40.558400 1263.111000 126.065200
-76.247540 40.664560 528.118800 163.755900
-74.947690 40.756360 255.666900 189.116900
-73.642300 40.833600 673.630900 95.046590
-72.332310 40.896110 747.926600 75.330150
-71.018690 40.943760 752.725300 51.392050
-69.702430 40.976440 822.698300 54.008140
-68.384490 40.994090 841.796000 61.947310
-67.065880 40.996670 777.597300 45.552430
-65.747600 40.984170 698.156400 19.295040
-64.430650 40.956610 676.000200 12.569330
-63.116020 40.914060 670.308000 10.581430
-61.804690 40.856610 665.031400 8.710172
-60.497620 40.784390 661.100700 7.511763
-59.195770 40.697540 658.298100 6.681722
-57.900040 40.596260 655.399900 5.453578
-56.611330 40.480750 652.163300 3.152583
-55.330490 40.351270 649.546100 .949830
-54.058330 40.208070 648.722100 .218300
-52.795620 40.051450 648.534400 .067993
-51.543080 39.881710 648.494400 .015768
-50.301390 39.699200 648.486600 .004254
-49.071130 39.504250 648.485100 .000912
-47.852850 39.297230 648.485000 .000311
-46.647030 39.078520 648.485500 .000188
-45.454070 38.848490 648.485800 .000379

-44.274280	38.607530	648.485800	.000256
-43.107920	38.356030	648.485800	.000244
-91.451740	39.437030	713.345500	24.706650
-90.263630	39.689750	798.028100	45.318300
-89.061680	39.931370	985.954300	95.171830
-87.846150	40.161480	1132.774000	147.864100
-86.617400	40.379670	1161.804000	185.447100
-85.375900	40.585540	1319.115000	203.605700
-84.122160	40.778730	1689.435000	177.300600
-82.856800	40.958850	2005.913000	196.327800
-81.580460	41.125550	2206.485000	209.643500
-80.293880	41.278500	2369.369000	238.865100
-78.997860	41.417370	2454.752000	274.355200
-77.693230	41.541890	2213.861000	262.836500
-76.380870	41.651780	1433.512000	136.191900
-75.061690	41.746810	877.146800	89.153080
-73.736660	41.826770	1032.114000	133.632100
-72.406780	41.891490	1011.891000	82.776540
-71.073040	41.940830	986.097000	70.875950
-69.736500	41.974670	961.747400	84.886820
-68.398190	41.992950	904.116900	81.162980
-67.059160	41.995620	734.653200	38.435660
-65.720480	41.982670	663.601000	11.193760
-64.383220	41.954140	704.534500	23.079650
-63.048400	41.910080	720.228300	29.114830
-61.717090	41.850600	702.080600	23.991650
-60.390290	41.775820	676.968800	16.885740
-59.069010	41.685920	660.969500	13.614420
-57.754210	41.581080	653.190600	9.533483
-56.446830	41.461530	651.705600	4.393834
-55.147770	41.327530	649.165600	.489578
-53.857880	41.179360	648.360900	.332469
-52.577970	41.017320	648.423500	.163952
-51.308780	40.841740	648.472700	.058162
-50.051000	40.652980	648.482200	.014625
-48.805250	40.451390	648.484200	.003183
-47.572090	40.237360	648.484900	.000648
-46.352010	40.011290	648.485200	.000221
-45.145370	39.773560	648.485800	.000331
-43.952510	39.524580	648.485800	.000266
-42.773630	39.264750	648.485800	.000244
-91.793200	40.348750	719.608000	28.007970
-90.591710	40.609810	809.607900	52.253150
-89.375760	40.859430	949.757300	98.036380
-88.145600	41.097210	837.926900	82.908060
-86.901570	41.322720	404.725600	205.951300
-85.644150	41.535550	446.954100	257.988900
-84.373860	41.735290	1144.935000	45.430030
-83.091290	41.921560	1766.142000	93.766220
-81.797160	42.093980	2264.487000	170.009400
-80.492230	42.252200	2753.504000	264.026200
-79.177290	42.395880	3023.372000	326.701200
-77.853240	42.524730	2815.006000	301.178400
-76.521000	42.638450	2015.503000	127.142200
-75.181550	42.736820	1179.788000	216.383700
-73.835900	42.819590	1035.912000	103.210800
-72.485100	42.886590	984.866700	76.044950
-71.130210	42.937670	1046.948000	98.869190
-69.772340	42.972710	1003.879000	81.759200
-68.412590	42.991630	825.596500	54.890450
-67.052090	42.994400	525.863800	146.283200
-65.691960	42.980990	565.390000	71.315280
-64.333320	42.951450	747.446700	44.146490
-62.977290	42.905830	810.084000	67.278220
-61.624970	42.844260	750.399300	44.511790
-60.277440	42.766850	656.703300	15.171890
-58.935750	42.673790	618.490700	20.125100

-57.600940 42.565280 621.954300 17.032550
-56.273990 42.441570 637.380200 8.260495
-54.955840 42.302930 645.455500 3.974406
-53.647390 42.149640 648.026200 1.360820
-52.349470 41.982030 648.535100 .373427
-51.062890 41.800450 648.533100 .108810
-49.788320 41.605260 648.498500 .032462
-48.526440 41.396860 648.487100 .008016
-47.277780 41.175640 648.485300 .001531
-46.042850 40.942000 648.485300 .000232
-44.822010 40.696380 648.485700 .000290
-43.615560 40.439180 648.485800 .000269
-42.423690 40.170830 648.485800 .000247
-92.150770 41.257820 706.168000 23.238660
-90.935390 41.527410 785.864700 45.659850
-89.704910 41.785250 917.944300 100.648400
-88.459540 42.030900 809.253400 102.523300
-87.199620 42.263930 373.211600 97.447880
-85.925610 42.483890 380.030400 114.993400
-84.638040 42.690380 1079.951000 64.512700
-83.337520 42.882970 1751.896000 124.111000
-82.024790 43.061280 2448.200000 228.272800
-80.700610 43.224930 3230.087000 353.650300
-79.365870 43.373580 3612.427000 425.675400
-78.021450 43.506890 3403.346000 396.521700
-76.668370 43.624570 2599.608000 246.218500
-75.307630 43.726360 1671.759000 79.536120
-73.940300 43.812040 1153.642000 71.552780
-72.567500 43.881390 776.668200 77.351050
-71.190370 43.934270 966.903300 81.589470
-69.810050 43.970550 1156.935000 112.276700
-68.427750 43.990140 1108.553000 87.490480
-67.044650 43.993000 712.468500 89.253790
-65.661930 43.979120 670.697300 39.172260
-64.280810 43.948540 796.455000 70.103680
-62.902460 43.901310 813.705900 77.837930
-61.528050 43.837570 704.801600 30.896350
-60.158730 43.757450 581.967200 60.411010
-58.795620 43.661140 574.124100 52.012160
-57.439790 43.548850 621.147800 20.413250
-56.092310 43.420850 647.567700 10.229320
-54.754150 43.277410 652.363300 5.309308
-53.426270 43.118850 650.954100 1.951162
-52.109530 42.945500 649.577300 .796298
-50.804760 42.757750 648.859600 .326216
-49.512690 42.555960 648.581100 .114137
-48.233980 42.340550 648.502600 .031902
-46.969190 42.111940 648.487200 .006867
-45.718810 41.870550 648.485300 .001053
-44.483210 41.616830 648.485700 .000330
-43.262650 41.351200 648.485800 .000301
-42.057300 41.074100 648.485800 .000250
-92.525320 42.164070 675.343200 12.917340
-91.295520 42.442420 717.470800 27.142780
-90.049940 42.708690 803.781300 62.565380
-88.788760 42.962440 746.587300 98.755300
-87.512310 43.203190 421.014400 75.320440
-86.221000 43.430500 363.041400 135.440400
-84.915410 43.643920 1155.065000 111.288400
-83.596150 43.843030 1964.095000 202.163800
-82.263960 44.027400 2759.041000 313.773100
-80.919660 44.196660 3600.411000 475.109400
-79.564150 44.350410 3960.014000 530.166500
-78.198390 44.488330 3636.454000 459.836000
-76.823410 44.610100 2745.833000 266.721300
-75.440290 44.715440 1755.547000 90.327510
-74.050180 44.804110 964.291900 65.718570

-72.654240	44.875890	445.754100	143.767800
-71.253690	44.930630	791.963300	83.265270
-69.849760	44.968180	1437.461000	194.375100
-68.443720	44.988460	1590.571000	233.387600
-67.036810	44.991420	1040.058000	54.259700
-65.630330	44.977050	745.237700	41.537990
-64.225530	44.945390	718.164400	37.050040
-62.823690	44.896510	689.186300	33.909110
-61.426040	44.830540	575.432400	41.545440
-60.033810	44.747610	564.414600	44.865110
-58.648170	44.647940	634.107200	17.798080
-57.270290	44.531750	681.400800	23.326980
-55.901260	44.399320	678.437700	15.919860
-54.542130	44.250930	665.563400	8.551624
-53.193900	44.086940	657.377200	4.371751
-51.857480	43.907680	652.401400	2.180342
-50.533710	43.713560	649.874500	.947390
-49.223370	43.504980	648.869400	.343821
-47.927120	43.282370	648.564300	.102506
-46.645540	43.046150	648.496500	.024403
-45.379100	42.796800	648.486300	.004487
-44.128160	42.534760	648.485300	.000596
-42.892960	42.260480	648.485700	.000240
-41.673610	41.974410	648.485800	.000264
-92.917770	43.067330	649.375200	4.144023
-91.673000	43.354680	655.079800	9.614091
-90.411740	43.629630	661.318500	30.062850
-89.134110	43.891690	550.742800	65.405140
-87.840450	44.140390	313.602600	67.619190
-86.531140	44.375260	258.413900	184.538000
-85.206750	44.595830	1140.395000	121.629300
-83.867900	44.801650	2112.156000	276.661500
-82.515380	44.992280	2756.013000	349.096700
-81.150000	45.167310	3219.756000	404.352100
-79.772730	45.326340	3319.224000	402.068100
-78.384560	45.469020	3012.588000	338.401500
-76.986600	45.595010	2303.185000	169.920100
-75.579970	45.704020	1510.574000	79.080530
-74.165890	45.795780	857.581300	145.742000
-72.745610	45.870080	336.121400	246.159500
-71.320400	45.926740	696.936600	168.715800
-69.891600	45.965610	1495.639000	166.734000
-68.460530	45.986610	1703.840000	221.130500
-67.028560	45.989670	1135.400000	66.525960
-65.597030	45.974800	748.977400	88.426930
-64.167300	45.942020	747.995400	38.214700
-62.740720	45.891430	691.971600	36.188470
-61.318600	45.823140	512.377300	110.746100
-59.902260	45.737310	569.046400	48.026850
-58.492950	45.634170	693.437300	30.056930
-57.091900	45.513950	720.016700	27.852740
-55.700260	45.376930	701.246500	21.861280
-54.319140	45.223450	690.031700	16.008350
-52.949590	45.053840	674.753900	10.377730
-51.592580	44.868490	660.103900	5.340698
-50.248970	44.667810	652.530900	2.264401
-48.919560	44.452230	649.653000	.827718
-47.605040	44.222190	648.750900	.254352
-46.305980	43.978160	648.530200	.064435
-45.022840	43.720610	648.490400	.013086
-43.755960	43.450020	648.485600	.002040
-42.505550	43.166850	648.485500	.000265
-41.271660	42.871580	648.485800	.000292
-93.329110	43.967430	636.558300	4.975330
-92.068780	44.264030	619.460400	11.103340
-90.791210	44.547890	552.307100	28.043510
-89.496510	44.818510	418.906800	78.741840

-88.184940	45.075390	323.977800	70.957080
-86.856880	45.318050	370.978900	111.116500
-85.512890	45.545990	1111.573000	105.839200
-84.153590	45.758730	1915.987000	241.888700
-82.779790	45.955820	2196.028000	239.314700
-81.392350	46.136820	2121.863000	169.791400
-79.992260	46.301310	2009.901000	148.055400
-78.580580	46.448910	1900.971000	123.102800
-77.158460	46.579270	1689.530000	92.189380
-75.727110	46.692070	1584.190000	77.545760
-74.287820	46.787040	1507.731000	142.160600
-72.841890	46.863950	1286.016000	103.949500
-71.390720	46.922590	1339.087000	107.606200
-69.935700	46.962830	1736.044000	213.976800
-68.478260	46.984570	1737.047000	186.116200
-67.019850	46.987740	1220.876000	72.461330
-65.561920	46.972340	887.865600	65.727070
-64.105920	46.938410	912.767900	57.712550
-62.653260	46.886040	854.584000	46.446210
-61.205390	46.815350	653.601700	114.887700
-59.763680	46.726520	683.329800	64.854750
-58.329470	46.619790	793.001400	34.273730
-56.904060	46.495390	803.294300	42.888990
-55.488670	46.353640	787.525000	41.435760
-54.084500	46.194880	775.195000	35.337060
-52.692630	46.019470	726.489300	24.578270
-51.314070	45.827840	679.710000	12.198750
-49.949730	45.620390	658.095900	4.652025
-48.600430	45.397590	651.122700	1.595042
-47.266860	45.159900	649.118100	.492757
-45.949600	44.907820	648.605800	.128597
-44.649100	44.641840	648.502500	.027693
-43.365670	44.362450	648.487100	.004781
-42.099450	44.070140	648.485600	.000651
-40.850440	43.765410	648.485800	.000320
-93.760410	44.864160	640.204900	5.110616
-92.483920	45.170280	629.857100	10.931110
-91.189420	45.463320	581.332100	25.663010
-89.876960	45.742750	445.969800	42.458790
-88.546760	46.008070	388.556500	79.545420
-87.199170	46.258760	496.959800	81.321000
-85.834720	46.494300	964.862200	48.612050
-84.454070	46.714200	1384.461000	115.091700
-83.058010	46.917960	1329.211000	72.440080
-81.647480	47.105130	1153.500000	200.901800
-80.223460	47.275260	1132.040000	192.798400
-78.787090	47.427960	1196.296000	117.277200
-77.339580	47.562840	1256.215000	121.438900
-75.882230	47.679570	1508.180000	72.248390
-74.416370	47.777870	1809.800000	157.980300
-72.943440	47.857470	1852.231000	188.883000
-71.464880	47.918190	1789.642000	173.560500
-69.982220	47.959850	1784.318000	155.589600
-68.496960	47.982350	1538.448000	115.101500
-67.010670	47.985630	1135.113000	54.922250
-65.524890	47.969690	900.868600	55.618950
-64.041170	47.934570	1030.915000	56.885900
-62.561040	47.880340	1102.505000	62.849300
-61.086020	47.807170	1013.541000	50.761580
-59.617590	47.715230	989.659700	56.277230
-58.157170	47.604770	1011.731000	69.032280
-56.706150	47.476050	998.382100	79.593250
-55.265830	47.329400	985.724900	79.658360
-53.837470	47.165180	947.415700	74.310300
-52.422200	46.983780	823.691400	46.953410
-51.021090	46.785640	715.547100	21.655390
-49.635090	46.571200	667.176500	8.040059

-48.265020	46.340950	653.057600	2.522084
-46.911610	46.095370	649.542700	.740214
-45.575410	45.834990	648.698600	.194680
-44.256860	45.560320	648.520100	.043048
-42.956210	45.271870	648.489900	.007836
-41.673580	44.970160	648.486100	.001170
-40.408870	44.655690	648.485800	.000322
-94.212860	45.757310	652.959000	5.088649
-92.919560	46.073230	652.990900	9.832242
-91.607470	46.375730	633.133700	17.267380
-90.276550	46.664250	560.695200	26.145210
-88.926960	46.938270	536.748800	36.741050
-87.559010	47.197250	634.123000	21.218950
-86.173240	47.440650	820.376800	31.973600
-84.770290	47.667940	905.826500	75.287480
-83.350960	47.878600	931.200100	162.219100
-81.916200	48.072160	1086.383000	85.172280
-80.467090	48.248140	1277.789000	49.483650
-79.004780	48.406110	1312.235000	51.407540
-77.530590	48.545680	1348.969000	58.572240
-76.045850	48.666500	1545.933000	107.333300
-74.552020	48.768250	1735.046000	147.621300
-73.050600	48.850660	1746.552000	149.573400
-71.543170	48.913520	1705.089000	147.196800
-70.031330	48.956650	1620.061000	144.701500
-68.516710	48.979950	1338.211000	95.905760
-67.000980	48.983350	914.169000	123.499000
-65.485790	48.966840	634.129500	222.363000
-63.972820	48.930470	892.425200	60.886590
-62.463700	48.874330	1225.426000	114.026500
-60.960050	48.798580	1358.182000	132.209100
-59.463460	48.703410	1340.443000	114.368200
-57.975440	48.589080	1268.370000	107.999900
-56.497490	48.455880	1179.146000	110.973300
-55.030970	48.304140	1136.421000	115.129600
-53.577210	48.134270	1052.531000	107.829600
-52.137430	47.946660	873.687400	60.562850
-50.712720	47.741790	732.576500	26.445460
-49.304080	47.520120	672.659900	9.654212
-47.912350	47.282170	654.577900	3.058764
-46.538250	47.028450	649.851900	.897768
-45.182340	46.759510	648.747700	.228126
-43.845020	46.475880	648.528600	.049659
-42.526500	46.178100	648.491600	.009222
-41.226800	45.866700	648.486400	.001434
-39.945740	45.542200	648.486000	.000358
-94.687740	46.646660	675.297500	11.767680
-93.376940	46.972690	686.225800	15.936160
-92.046550	47.284930	695.777400	19.802640
-90.696440	47.582840	683.666600	16.462300
-89.326680	47.865840	708.502500	23.600720
-87.937550	48.133390	787.179100	29.287270
-86.529520	48.384910	851.952600	47.178650
-85.103260	48.619850	939.996600	74.431370
-83.659580	48.837660	1163.296000	64.523750
-82.199460	49.037830	1489.801000	152.432700
-80.724000	49.219880	1599.451000	177.411000
-79.234450	49.383340	1473.803000	114.214500
-77.732160	49.527770	1408.198000	93.895070
-76.218590	49.652830	1468.584000	108.615400
-74.695260	49.758160	1551.426000	134.155100
-73.163800	49.843490	1536.050000	142.314400
-71.625870	49.908580	1532.833000	143.397600
-70.083210	49.953250	1525.332000	142.505400
-68.537570	49.977380	1375.037000	121.887800
-66.990740	49.980900	1046.771000	73.697850
-65.444490	49.963800	684.421300	132.064700

-63.900620	49.926140	689.266100	115.914200
-62.360880	49.868010	966.186400	58.277210
-60.827010	49.789570	1230.131000	108.590100
-59.300730	49.691040	1339.601000	132.590100
-57.783640	49.572690	1290.922000	126.130900
-56.277320	49.434830	1171.796000	110.089700
-54.783270	49.277820	1061.137000	99.852060
-53.302860	49.102080	938.439200	73.856790
-51.837360	48.908040	781.182000	43.516670
-50.387950	48.696200	690.093000	20.452050
-48.955640	48.467040	660.616100	7.062880
-47.541310	48.221130	652.127100	2.425383
-46.145660	47.959000	649.437700	.744369
-44.769230	47.681210	648.672800	.194164
-43.412380	47.388330	648.514700	.041283
-42.075270	47.080920	648.489700	.007444
-40.757810	46.759540	648.486100	.001157
-39.459720	46.424710	648.486000	.000349
-95.186440	47.531940	712.999500	24.349520
-93.857410	47.868410	740.628900	31.494690
-92.508000	48.190720	780.836200	40.818550
-91.137910	48.498310	830.288000	48.505380
-89.747150	48.790600	911.515100	61.034560
-88.335950	49.067010	1006.751000	79.846870
-86.904710	49.326930	1066.370000	83.440630
-85.454100	49.569790	1132.194000	86.861740
-83.984930	49.795020	1296.567000	126.003500
-82.498220	50.002060	1436.158000	151.576000
-80.995120	50.190400	1433.630000	134.767800
-79.476920	50.359550	1333.055000	108.953200
-77.945060	50.509060	1339.511000	113.011900
-76.401090	50.638520	1410.914000	127.234900
-74.846650	50.747580	1485.597000	147.864800
-73.283450	50.835950	1444.051000	141.506800
-71.713320	50.903350	1421.172000	132.584700
-70.138080	50.949630	1422.722000	127.787900
-68.559640	50.974620	1355.544000	115.761200
-66.979900	50.978270	1229.129000	114.875500
-65.400800	50.960560	999.709300	97.442510
-63.824260	50.921550	818.838900	51.939840
-62.252170	50.861340	820.729400	70.135220
-60.686400	50.780110	968.137100	85.269560
-59.128770	50.678080	1115.851000	108.147000
-57.581030	50.555550	1133.372000	108.221200
-56.044860	50.412850	1035.690000	96.118930
-54.521820	50.250360	917.802900	69.417080
-53.013420	50.068520	803.856100	45.928970
-51.520960	49.867810	709.497800	25.582200
-50.045680	49.648740	665.861900	11.195310
-48.588630	49.411830	653.168400	4.224523
-47.150700	49.157670	649.943500	1.481912
-45.732590	48.886830	648.847600	.453855
-44.334810	48.599900	648.547900	.113341
-42.957660	48.297460	648.494000	.023622
-41.601190	47.980120	648.486800	.004251
-40.265250	47.648430	648.485800	.000636
-38.949390	47.302930	648.485900	.000298
-95.710510	48.412890	782.378400	43.634600
-94.362470	48.760130	849.057700	54.662710
-92.993230	49.092850	933.707800	69.091860
-91.602360	49.410460	1007.250000	83.358120
-90.189750	49.712350	1066.463000	98.704320
-88.755540	49.997940	1093.918000	103.312000
-87.300090	50.266570	1079.896000	95.810370
-85.824040	50.517650	1059.829000	88.385220
-84.328210	50.750570	1085.182000	97.989140
-82.813610	50.964740	1145.349000	109.013600

-81.281460	51.159630	1195.503000	108.296800
-79.733130	51.334700	1203.941000	106.944600
-78.170130	51.489480	1255.686000	117.535900
-76.594090	51.623530	1325.985000	129.933000
-75.006800	51.736490	1388.483000	142.006900
-73.410070	51.828010	1331.323000	130.256300
-71.805870	51.897850	1299.120000	118.548200
-70.196170	51.945790	1299.675000	105.557700
-68.582990	51.971680	1222.891000	86.267790
-66.968440	51.975460	1131.322000	77.033900
-65.354560	51.957120	1068.996000	85.105260
-63.743440	51.916690	955.171700	80.421090
-62.137130	51.854320	881.919700	69.952940
-60.537630	51.770170	920.387200	81.661220
-58.946900	51.664510	995.946400	93.925650
-57.366820	51.537620	992.220500	92.734950
-55.799180	51.389870	911.729300	71.016600
-54.245650	51.221680	814.253700	48.716530
-52.707820	51.033510	743.054200	29.811770
-51.187080	50.825860	694.036000	16.675170
-49.684720	50.599280	665.480300	7.259001
-48.201800	50.354330	653.381200	2.696875
-46.739240	50.091630	649.604700	.871802
-45.297720	49.811770	648.690600	.244279
-43.877700	49.515370	648.515700	.057698
-42.479410	49.203060	648.489600	.011608
-41.102810	48.875440	648.486300	.001938
-39.747590	48.533100	648.486000	.000406
-38.413150	48.176570	648.485800	.000243
-96.261670	49.289190	877.196900	60.977550
-94.893750	49.647600	989.539600	84.331020
-93.503820	49.991080	1070.786000	103.914700
-92.091320	50.319060	1080.870000	104.294000
-90.655950	50.630910	1039.084000	91.998880
-89.197760	50.925990	973.619400	78.583540
-87.717060	51.203660	914.992100	68.424700
-86.214420	51.463270	878.152700	63.012290
-84.690660	51.704190	888.742500	66.166370
-83.146820	51.925780	981.859100	75.907260
-81.584160	52.127480	1098.662000	99.642710
-80.004110	52.308710	1147.101000	106.142000
-78.408280	52.468990	1146.524000	106.155000
-76.798410	52.607840	1203.610000	116.313000
-75.176390	52.724850	1277.402000	129.507800
-73.544200	52.819690	1225.610000	116.817800
-71.903920	52.892050	1195.248000	104.367900
-70.257710	52.941730	1189.232000	87.439470
-68.607750	52.968570	1094.913000	56.386200
-66.956280	52.972490	983.193400	53.233790
-65.305560	52.953480	985.587800	68.694890
-63.657820	52.911580	995.109400	84.966430
-62.015270	52.846950	961.652300	90.104790
-60.380080	52.759750	951.725500	88.326930
-58.754360	52.650280	941.554900	84.993180
-57.140120	52.518850	886.990500	67.773580
-55.539290	52.365840	805.518400	48.781060
-53.953660	52.191710	742.352500	31.718440
-52.384890	51.996940	701.355200	18.903640
-50.834480	51.782070	674.896500	10.173000
-49.303730	51.547700	658.427000	4.454331
-47.793770	51.294400	651.293100	1.572479
-46.305490	51.022820	649.133600	.483962
-44.839570	50.733620	648.614300	.129679
-43.396390	50.427420	648.508400	.029565
-41.976090	50.104880	648.489400	.005613
-40.578500	49.766630	648.486300	.000963
-39.203150	49.413260	648.485900	.000309

-37.849210	49.045330	648.485800	.000237
-96.841810	50.160530	887.908000	62.429210
-95.453030	50.530490	973.348400	86.602910
-94.041510	50.885130	996.190900	85.881130
-92.606450	51.223850	946.798100	75.399640
-91.147380	51.546020	875.910500	63.505830
-89.664200	51.850960	812.450000	51.431240
-88.157130	52.138020	772.179500	41.963460
-86.626690	52.406500	757.326000	36.828120
-85.073680	52.655720	781.548500	39.130720
-83.499180	52.885050	848.457600	55.485150
-81.904440	53.093850	935.735300	81.804840
-80.290990	53.281520	990.123800	84.463920
-78.660530	53.447530	1002.955000	81.700600
-77.014920	53.591380	1080.217000	96.643710
-75.356160	53.712650	1175.091000	114.557900
-73.686420	53.810940	1134.591000	105.207300
-72.007930	53.885960	1099.915000	91.494080
-70.322990	53.937460	1067.167000	68.687140
-68.634010	53.965280	951.986300	35.977910
-66.943400	53.969350	843.519000	63.254800
-65.253590	53.949630	911.786600	46.908500
-63.567000	53.906200	996.541500	89.341920
-61.886040	53.839190	996.586400	96.698260
-60.213040	53.748820	934.373800	83.648900
-58.550290	53.635360	861.959700	62.762800
-56.899960	53.499180	784.794600	44.052820
-55.264110	53.340690	726.501400	29.390330
-53.644650	53.160350	696.059000	19.857000
-52.043340	52.958710	674.835700	11.516690
-50.461760	52.736320	660.353700	5.590377
-48.901280	52.493820	652.765300	2.278140
-47.363030	52.231850	649.771400	.815491
-45.847900	51.951060	648.818200	.256775
-44.356530	51.652150	648.559900	.069445
-42.889220	51.335790	648.500100	.016028
-41.445980	51.002650	648.488000	.003244
-40.026490	50.653380	648.486100	.000636
-38.630060	50.288590	648.485900	.000298
-37.255620	49.908830	648.485800	.000244
-97.453060	51.026530	784.730000	41.384110
-96.042340	51.408490	824.368500	47.836420
-94.608220	51.774700	823.907300	51.261290
-93.149600	52.124570	799.697300	49.636590
-91.665800	52.457450	769.982600	44.145050
-90.156560	52.772640	740.431600	36.498460
-88.621980	53.069450	716.478000	28.763800
-87.062480	53.347160	696.632200	20.856260
-85.478850	53.605050	672.897100	19.553920
-83.872130	53.842430	632.096500	44.875520
-82.243660	54.058630	589.593500	83.478230
-80.595020	54.253030	667.232400	54.859790
-78.928000	54.425040	812.887600	46.473340
-77.244600	54.574130	973.818300	73.715770
-75.546970	54.699840	1088.517000	98.873570
-73.837420	54.801750	1063.217000	92.984380
-72.118360	54.879540	1009.945000	77.073670
-70.392330	54.932960	908.807700	46.294170
-68.661900	54.961820	771.444700	53.330700
-66.929700	54.966030	745.457000	68.417710
-65.198370	54.945580	847.992700	44.901250
-63.470540	54.900540	941.726800	76.929410
-61.748820	54.831050	933.168500	81.157710
-60.035740	54.737340	850.158700	60.201500
-58.333780	54.619720	771.168700	41.403740
-56.645260	54.478570	718.005200	25.977720
-54.972420	54.314330	687.591800	16.645040

-53.317280	54.127510	675.749800	13.808250
-51.681740	53.918690	665.414900	9.097223
-50.067420	53.688470	655.691400	4.064742
-48.475770	53.437500	650.919200	1.493508
-46.907930	53.166490	649.227700	.517851
-45.364770	52.876130	648.684400	.162957
-43.846840	52.567130	648.530200	.045006
-42.354370	52.240220	648.493800	.010894
-40.887210	51.896080	648.486800	.002264
-39.444820	51.535370	648.486000	.000531
-38.026250	51.158730	648.485800	.000245
-36.630120	50.766700	648.485800	.000244
-98.097820	51.886770	702.407400	20.451650
-96.663910	52.281200	711.416400	22.276460
-95.206040	52.659450	712.653900	23.835430
-93.722790	53.020900	712.025200	25.310810
-92.213200	53.364910	713.119800	28.117610
-90.676770	53.690770	713.943400	31.337000
-89.113440	53.997730	709.182700	30.520380
-87.523560	54.285050	694.081500	23.640710
-85.907830	54.551970	654.558400	13.842100
-84.267300	54.797760	548.714000	51.832580
-82.603340	55.021710	381.568700	143.233200
-80.917590	55.223140	347.990400	162.478600
-79.211960	55.401440	577.157200	65.469110
-77.488560	55.556020	875.542100	60.494050
-75.749730	55.686380	1010.912000	82.934520
-73.997920	55.792100	984.398300	77.546360
-72.235800	55.872810	895.874800	53.660860
-70.466090	55.928230	730.052400	56.626930
-68.691570	55.958180	651.119100	90.007850
-66.915130	55.962550	762.802700	48.880980
-65.139640	55.941330	855.086200	61.041850
-63.367970	55.894590	861.426300	60.092670
-61.602940	55.822490	820.715600	52.103950
-59.847310	55.725280	758.706200	37.790600
-58.103760	55.603290	711.351600	25.791790
-56.374820	55.456930	682.052700	15.031120
-54.662870	55.286670	667.623400	9.169308
-52.970100	55.093080	666.163000	9.493864
-51.298500	54.876750	663.975200	9.287645
-49.649800	54.638340	655.976800	4.578273
-48.025460	54.378550	650.940600	1.614797
-46.426660	54.098120	649.211100	.529100
-44.854210	53.797780	648.683600	.166990
-43.308580	53.478290	648.530600	.047176
-41.789850	53.140400	648.493800	.011698
-40.297680	52.784830	648.486800	.002471
-38.831280	52.412250	648.486000	.000550
-37.389370	52.023280	648.485800	.000242
-35.970190	51.618480	648.485800	.000244
-98.778840	52.740810	666.832600	8.400757
-97.320270	53.148240	667.548800	8.563158
-95.837370	53.539010	667.948000	9.014696
-94.328290	53.912530	669.073200	10.054970
-92.791720	54.268120	673.201000	12.338860
-91.226900	54.605080	680.860000	16.188980
-89.633560	54.922620	689.318300	20.799220
-88.011890	55.219970	694.365800	24.949260
-86.362530	55.496330	682.190600	24.532880
-84.686490	55.750910	621.140800	30.046060
-82.985150	55.982970	458.327400	74.745710
-81.260250	56.191770	274.297100	168.992700
-79.513790	56.376650	397.492800	132.151300
-77.748020	56.536990	781.348400	65.038860
-75.965460	56.672260	940.209600	72.244770
-74.168790	56.781970	898.814500	61.518930

-72.360860 56.865740 810.447000 45.007890
-70.544630 56.923280 680.161900 67.539810
-68.723180 56.954370 700.461700 62.940360
-66.899620 56.958910 859.800000 78.042740
-65.077100 56.936880 869.352700 69.505670
-63.258730 56.888360 783.647400 43.634980
-61.447630 56.813510 720.009000 27.859540
-59.646790 56.712620 692.420800 19.643270
-57.859090 56.586040 682.414600 16.398010
-56.087300 56.434210 665.671100 9.235317
-54.333970 56.257640 658.175500 5.064606
-52.601470 56.056940 660.798900 6.938896
-50.891880 55.832740 664.154900 9.892342
-49.207030 55.585770 658.148100 5.949353
-47.548420 55.316760 651.896900 2.276086
-45.917200 55.026500 649.504000 .723622
-44.314130 54.715760 648.774800 .232382
-42.739580 54.385350 648.556700 .067780
-41.193420 54.036030 648.499600 .017326
-39.675070 53.668560 648.487800 .003819
-38.183400 53.283620 648.486000 .000743
-36.716770 52.881820 648.485900 .000301
-35.272900 52.463700 648.485800 .000242
-99.499240 53.588130 654.051000 3.143827
-98.014280 54.009130 653.715300 2.994525
-96.504860 54.412960 653.760800 3.070029
-94.968610 54.799050 654.097500 3.354694
-93.403790 55.166730 655.511700 4.177807
-91.809290 55.515250 658.662700 5.868427
-90.184560 55.843850 664.164800 8.574727
-88.529620 56.151670 672.007000 12.401020
-86.844990 56.437890 677.126200 16.588200
-85.131650 56.701690 667.534700 25.144330
-83.390960 56.942230 603.318300 51.564620
-81.624720 57.158780 436.676100 76.732240
-79.835050 57.350580 425.441500 120.369100
-78.024370 57.516980 739.605700 67.868930
-76.195360 57.657400 863.838500 60.458480
-74.350940 57.771310 833.066700 55.432950
-72.494220 57.858320 799.649400 48.797520
-70.628410 57.918090 756.141200 43.601090
-68.756890 57.950390 811.205900 57.272000
-66.883060 57.955100 899.080600 79.989110
-65.010370 57.932220 836.329500 57.871770
-63.142240 57.881810 735.768100 32.820450
-61.282040 57.804080 671.344500 16.637350
-59.433060 57.699310 659.089600 9.368741
-57.598450 57.567890 658.654800 9.655093
-55.781200 57.410300 649.818200 6.149897
-53.984060 57.227100 649.162800 2.197174
-52.209560 57.018940 655.221600 4.103134
-50.459930 56.786500 663.046300 9.384917
-48.737060 56.530560 661.392900 8.085403
-47.042490 56.251900 654.090100 3.561642
-45.377330 55.951360 650.184100 1.149892
-43.742270 55.629770 648.951800 .347054
-42.137470 55.287960 648.603000 .101015
-40.562610 54.926740 648.510300 .026379
-39.016750 54.546860 648.489600 .006054
-37.498410 54.149020 648.486300 .001197
-36.005400 53.733830 648.485900 .000307
-34.534880 53.301770 648.485800 .000240
-100.262700 54.428120 649.908300 1.035341
-98.749250 54.863350 649.720800 .931366
-97.211560 55.280850 649.727700 .931677
-95.646590 55.680060 649.857400 1.024111
-94.052090 56.060360 650.286800 1.271098

-92.426510	56.420970	651.215800	1.771813
-90.768940	56.761100	652.941700	2.678078
-89.079190	57.079890	655.643800	4.185392
-87.357570	57.376450	658.801300	6.527682
-85.605000	57.649890	661.214800	11.862840
-83.822840	57.899360	645.508900	26.436430
-82.012920	58.124030	577.317700	37.685480
-80.177480	58.323120	561.549000	48.870570
-78.319110	58.495910	755.547200	68.053940
-76.440710	58.641750	797.927400	51.608240
-74.545430	58.760110	797.809000	51.544180
-72.636660	58.850530	837.361800	53.795850
-70.717930	58.912650	837.111800	50.226730
-68.792920	58.946230	869.550000	62.611640
-66.865380	58.951130	869.020100	65.317020
-64.939080	58.927340	785.913800	43.126900
-63.017780	58.874950	702.738000	22.207720
-61.105210	58.794160	657.604000	10.015810
-59.204930	58.685300	650.276200	5.969584
-57.320400	58.548780	650.923000	8.253655
-55.454840	58.385130	640.059000	8.745825
-53.611270	58.194950	638.494200	6.798335
-51.792370	57.978940	646.371500	2.222794
-50.000500	57.737840	658.037500	6.587711
-48.237630	57.472490	663.049400	9.731302
-46.505310	57.183730	656.255100	4.864242
-44.804610	56.872430	650.890600	1.596196
-43.136050	56.539500	649.128100	.459584
-41.499610	56.185780	648.650700	.133691
-39.894630	55.812120	648.522500	.035863
-38.319800	55.419290	648.492100	.008464
-36.773090	55.007980	648.486500	.001743
-35.251730	54.578750	648.486000	.000399
-33.752120	54.132060	648.485900	.000294
-101.073700	55.260130	648.782400	.293441
-99.529010	55.710320	648.724100	.246493
-97.960900	56.142140	648.729300	.244395
-96.365430	56.555100	648.779500	.276196
-94.739640	56.948590	648.920700	.362441
-93.081440	57.321860	649.212800	.529871
-91.389490	57.674070	649.732900	.807400
-89.663250	58.004340	650.481900	1.260056
-87.902820	58.311730	651.365700	2.455625
-86.109000	58.595320	652.076200	6.562122
-84.283100	58.854170	644.429000	15.425270
-82.427000	59.087400	618.602600	22.509030
-80.543050	59.294160	649.519000	26.500840
-78.634010	59.473690	755.244600	63.543610
-76.703000	59.625280	725.982800	42.361890
-74.753460	59.748340	754.999500	48.809780
-72.789070	59.842370	832.220100	57.794700
-70.813740	59.906970	861.864100	55.373890
-68.831480	59.941910	873.702600	61.736020
-66.846440	59.947010	836.600500	53.782870
-64.862770	59.922250	749.818000	33.599290
-62.884610	59.867760	684.402000	15.594490
-60.916040	59.783740	653.210800	6.601444
-58.961010	59.670550	650.804900	3.712229
-57.023270	59.528640	652.338500	6.659100
-55.106350	59.358580	640.437100	7.082535
-53.213500	59.161040	633.339500	10.037710
-51.347600	58.936760	636.011900	7.978187
-49.511150	58.686560	647.504700	2.914088
-47.706190	58.411320	658.929400	7.935169
-45.934240	58.111950	656.073500	4.889118
-44.196280	57.789400	651.075000	1.755728
-42.492650	57.444580	649.197000	.504457

-40.823030	57.078400	648.677200	.151106
-39.186360	56.691730	648.531700	.042134
-37.580830	56.285340	648.494500	.010367
-36.003750	55.859910	648.486900	.002198
-34.451600	55.415950	648.486000	.000447
-32.919830	54.953830	648.485900	.000293
-101.937200	56.083360	648.535500	.069429
-100.358100	56.549350	648.522700	.054757
-98.756940	56.996240	648.524400	.053923
-97.128790	57.423640	648.539200	.065291
-95.469860	57.830970	648.579700	.094862
-93.777320	58.217490	648.671800	.155762
-92.049300	58.582370	648.850800	.275758
-90.284800	58.924680	649.135000	.600359
-88.483640	59.243460	649.556700	1.816646
-86.646400	59.537720	649.723100	6.021908
-84.774360	59.806450	641.972200	14.186840
-82.869400	60.048700	624.810800	21.242330
-80.933970	60.263580	672.784200	21.967160
-78.971000	60.450230	731.652300	47.274510
-76.983890	60.607900	653.756900	45.457360
-74.976370	60.735940	658.344200	42.862530
-72.952470	60.833800	744.467500	46.287260
-70.916480	60.901050	812.866000	53.295580
-68.872850	60.937420	859.631900	60.006790
-66.826130	60.942730	816.478800	48.982490
-64.780930	60.916960	728.738600	27.087910
-62.741830	60.860230	673.119400	11.466580
-60.713310	60.772790	650.751800	4.295817
-58.699720	60.654990	648.413500	1.552873
-56.705200	60.507380	651.192700	3.092790
-54.733590	60.330550	645.458800	3.191004
-52.788400	60.125220	638.281000	6.157568
-50.872730	59.892220	634.759000	8.410863
-48.989200	59.632420	639.024800	6.677639
-47.139900	59.346780	649.991800	3.717719
-45.326320	59.036270	653.823600	4.016826
-43.549270	58.701890	651.199500	1.832633
-41.808860	58.344600	649.301700	.572534
-40.104370	57.965370	648.695700	.158564
-38.434220	57.565060	648.536700	.043967
-36.795950	57.144450	648.496200	.011019
-35.186060	56.704150	648.487300	.002427
-33.600030	56.244660	648.486100	.000529
-32.032190	55.766200	648.485900	.000295
-102.859900	56.896880	648.492500	.013620
-101.242000	57.379640	648.490100	.009779
-99.604390	57.842470	648.490700	.009930
-97.940930	58.285070	648.493900	.013597
-96.246660	58.706940	648.504600	.024005
-94.517820	59.107400	648.533400	.052936
-92.751850	59.485580	648.597400	.150567
-90.947170	59.840560	648.707800	.500351
-89.103220	60.171320	648.889100	1.770779
-87.220290	60.476800	648.547500	6.092809
-85.299550	60.755960	640.029700	14.167770
-83.342830	61.007760	624.407300	20.964520
-81.352710	61.231220	669.802500	14.146360
-79.332320	61.425420	706.534300	33.952210
-77.285290	61.589540	607.044300	40.584820
-75.215710	61.722870	558.126500	57.303810
-73.128000	61.824790	597.380600	53.694000
-71.026890	61.894870	700.211400	51.355410
-68.917300	61.932760	816.793500	61.115480
-66.804310	61.938290	796.796900	46.430250
-64.692990	61.911450	714.101600	22.527640
-62.588420	61.852340	665.426500	9.650883

-60.495590 61.761240 649.117000 5.100541
-58.419300 61.638580 647.218600 3.133269
-56.364090 61.484900 649.533800 2.421765
-54.334160 61.300890 648.406000 2.290640
-52.333320 61.087320 645.740500 2.934299
-50.364880 60.845090 642.022500 4.306334
-48.431600 60.575160 640.452900 5.013146
-46.535590 60.278550 645.417800 2.929045
-44.678250 59.956310 650.650600 2.602075
-42.860180 59.609490 650.700400 1.590486
-41.081100 59.239120 649.252700 .537346
-39.339830 58.846180 648.679600 .142995
-37.634110 58.431540 648.530600 .037280
-35.960610 57.995940 648.495200 .009517
-34.314830 57.539980 648.487100 .002114
-32.690990 57.064010 648.486000 .000468
-31.082000 56.568130 648.485900 .000293
-103.849600 57.699570 648.486500 .002118
-102.187300 58.200280 648.486300 .001413
-100.509000 58.680030 648.486300 .001516
-98.806840 59.138710 648.486900 .002741
-97.074520 59.575940 648.489800 .008175
-95.307120 59.991050 648.499400 .032073
-93.501110 60.383240 648.517900 .129249
-91.654170 60.751550 648.518700 .478582
-89.765210 61.094930 648.361000 1.754806
-87.834170 61.412270 647.192800 6.008299
-85.861950 61.702450 639.975500 13.360960
-83.850420 61.964360 632.051000 18.199950
-81.802150 62.196920 669.568700 12.227150
-79.720490 62.399150 674.676500 24.609770
-77.609360 62.570130 580.956100 49.693950
-75.473210 62.709080 495.767400 85.293200
-73.316960 62.815350 479.969100 105.281700
-71.145790 62.888430 571.054500 82.441090
-68.965200 62.927950 726.375900 55.103890
-66.780790 62.933720 751.329000 39.116590
-64.598260 62.905720 694.985600 17.635880
-62.423240 62.844070 661.186600 8.849522
-60.261290 62.749080 651.296000 5.953138
-58.117710 62.621230 650.123800 5.175066
-55.997520 62.461110 650.903400 5.016721
-53.905320 62.269450 650.737700 4.817120
-51.845240 62.047150 650.256800 5.132130
-49.820820 61.795150 649.180100 5.572931
-47.834930 61.514500 647.569100 4.869003
-45.889670 61.206310 647.200600 2.530352
-43.986290 60.871700 649.429600 .709647
-42.125070 60.511780 649.227500 .525302
-40.305270 60.127630 648.758500 .199125
-38.525020 59.720250 648.570600 .064619
-36.781200 59.290500 648.509700 .020821
-35.069400 58.839090 648.491000 .005661
-33.383770 58.366500 648.486400 .001328
-31.716990 57.872960 648.486000 .000375
-30.060130 57.358350 648.485900 .000271
-104.916500 58.490070 648.486000 .000399
-103.202300 59.010120 648.485900 .000301
-101.477600 59.507980 648.485900 .000427
-99.732440 59.983750 648.485900 .001041
-97.958720 60.437220 648.486700 .005530
-96.150010 60.867820 648.489700 .026877
-94.301540 61.274790 648.489700 .114339
-92.410060 61.657170 648.432300 .430820
-90.473700 62.013880 648.053600 1.601556
-88.491920 62.343760 646.892900 5.378073
-86.465330 62.645610 645.405100 11.404450

-84.395630	62.918240	653.204900	10.437960
-82.285480	63.160470	678.396900	19.361560
-80.138370	63.371240	661.795200	18.411270
-77.958560	63.549540	594.055500	32.069480
-75.750930	63.694510	532.936600	56.415550
-73.520860	63.805420	499.941000	75.704300
-71.274150	63.881700	533.629000	72.047050
-69.016910	63.922960	644.394500	34.551040
-66.755400	63.928990	682.666800	20.184630
-64.495980	63.899750	668.611000	10.454430
-62.244980	63.835380	656.204700	6.531466
-60.008560	63.736250	651.797300	5.059041
-57.792640	63.602840	650.722900	4.428049
-55.602750	63.435840	650.546400	3.997979
-53.443960	63.236060	650.125700	3.286249
-51.320750	63.004450	649.749500	2.889454
-49.236890	62.742080	649.466700	2.882499
-47.195330	62.450080	648.901200	2.457236
-45.198120	62.129630	648.305500	1.463364
-43.246250	61.781950	648.579300	.408391
-41.339580	61.408210	648.595200	.117511
-39.476710	61.009530	648.552600	.049265
-37.654880	60.586900	648.515400	.023570
-35.869840	60.141170	648.494600	.008428
-34.115770	59.672970	648.487500	.002494
-32.385130	59.182660	648.486100	.000632
-30.668580	58.670220	648.486000	.000337
-28.954870	58.135290	648.486000	.000252
-106.073800	59.266680	648.486000	.000243
-104.297500	59.807790	648.486000	.000247
-102.518800	60.325180	648.485900	.000285
-100.725000	60.819250	648.485800	.000743
-98.905530	61.289990	648.486100	.004224
-97.052100	61.737010	648.486700	.020753
-95.158340	62.159620	648.480300	.087778
-93.219700	62.556880	648.412100	.329660
-91.233350	62.927700	648.105800	1.238642
-89.198040	63.270870	647.806800	4.022635
-87.113920	63.585110	650.756300	7.183628
-84.982480	63.869130	657.859900	7.384671
-82.806390	64.121680	659.667400	11.384710
-80.589290	64.341560	629.433000	19.553590
-78.335770	64.527670	592.483600	34.364780
-76.051160	64.679080	571.115600	41.425830
-73.741460	64.794950	565.887100	40.784370
-71.413090	64.874680	583.463700	31.688600
-69.072890	64.917820	631.258500	16.608040
-66.727910	64.924110	654.780500	7.861048
-64.385280	64.893550	654.483200	4.155557
-62.052090	64.826280	651.466900	2.758327
-59.735260	64.722690	649.897300	2.124542
-57.441380	64.583340	649.364400	1.808354
-55.176610	64.408980	649.229900	1.599528
-52.946510	64.200520	649.016000	1.236466
-50.755950	63.959000	648.818100	.973990
-48.608900	63.685590	648.723600	.907173
-46.508400	63.381530	648.580800	.732787
-44.456330	63.048090	648.437700	.461823
-42.453320	62.686580	648.457000	.163531
-40.498620	62.298210	648.495200	.045880
-38.589950	61.884150	648.501600	.014540
-36.723370	61.445380	648.493400	.006765
-34.893150	60.982680	648.488000	.002707
-33.091610	60.496560	648.486300	.000865
-31.309040	59.987160	648.486000	.000386
-29.533510	59.454220	648.486000	.000280
-27.750760	58.896990	648.486000	.000244

-107.339200 60.027230 648.486000 .000244
-105.487000 60.591610 648.486000 .000244
-103.643800 61.130270 648.486000 .000249
-101.793500 61.644070 648.485900 .000506
-99.922550 62.133320 648.485800 .002837
-98.020040 62.597820 648.485200 .013607
-96.077510 63.037030 648.478100 .054937
-94.088710 63.450070 648.434900 .188401
-92.049460 63.835860 648.229900 .604420
-89.957580 64.193130 647.684600 1.574168
-87.812570 64.520550 646.807700 1.702830
-85.615560 64.816710 645.716000 5.250355
-83.369120 65.080260 636.100100 13.937000
-81.077050 65.309890 621.080400 19.312120
-78.744270 65.504390 613.343800 20.718650
-76.376630 65.662700 613.038100 19.384710
-73.980770 65.783920 617.090800 16.187420
-71.563890 65.867360 626.425000 11.092750
-69.133670 65.912500 640.768300 5.682397
-66.698060 65.919100 648.628700 2.562325
-64.265090 65.887100 649.717400 1.368108
-61.842790 65.816700 649.243800 .868897
-59.438900 65.708320 648.835200 .651731
-57.060850 65.562580 648.679900 .549290
-54.715470 65.380330 648.646400 .479145
-52.408880 65.162570 648.599200 .377363
-50.146370 64.910470 648.549700 .298083
-47.932120 64.625290 648.521400 .264458
-45.769150 64.308400 648.487100 .209183
-43.659080 63.961160 648.465900 .134815
-41.602020 63.584970 648.472200 .060823
-39.596350 63.181100 648.485400 .019504
-37.638600 62.750720 648.488200 .005389
-35.723270 62.294770 648.487100 .001850
-33.842640 61.813940 648.486100 .000688
-31.986660 61.308540 648.486000 .000322
-30.142740 60.778430 648.485900 .000300
-28.295590 60.222970 648.486000 .000244
-26.427080 59.640870 648.486000 .000244
-108.736700 60.768890 648.486000 .000244
-106.789100 61.359350 648.486000 .000243
-104.866900 61.921510 648.486000 .000246
-102.949500 62.456830 648.486000 .000501
-101.019200 62.966030 648.485600 .002404
-99.061880 63.449270 648.483800 .010751
-97.066180 63.906190 648.472700 .039061
-95.023590 64.336020 648.420700 .117455
-92.928180 64.737730 648.230800 .284603
-90.776390 65.110020 647.637300 .614770
-88.566850 65.451470 646.250800 1.343027
-86.300130 65.760610 642.606100 5.675253
-83.978550 66.035940 635.169000 10.026560
-81.606060 66.276030 630.592200 11.467000
-79.187900 66.479550 631.261700 10.545560
-76.730480 66.645290 633.203600 9.007554
-74.241170 66.772270 635.821500 7.105186
-71.728080 66.859700 639.853500 4.745537
-69.199870 66.907030 644.691000 2.497475
-66.665550 66.913940 647.710800 1.142878
-64.134220 66.880400 648.604100 .567181
-61.614990 66.806620 648.663400 .333209
-59.116610 66.693070 648.578900 .251209
-56.647430 66.540450 648.536300 .214779
-54.215080 66.349720 648.526200 .185121
-51.826330 66.121990 648.516700 .150749
-49.486860 65.858530 648.504800 .122029
-47.201050 65.560780 648.494300 .103141

-44.971820 65.230190 648.483800 .081397
-42.800360 64.868270 648.478300 .053879
-40.685980 64.476460 648.480600 .026864
-38.625890 64.056100 648.484400 .010248
-36.614990 63.608330 648.485700 .003066
-34.645670 63.134020 648.486100 .000822
-32.707610 62.633670 648.486000 .000299
-30.787590 62.107330 648.486000 .000243
-28.869240 61.554470 648.486000 .000244
-26.932890 60.973880 648.486000 .000244
-24.955330 60.363580 648.486000 .000244

Q.29a
-89.453400 32.991660 683.268500 .331302
-88.345930 33.191160 683.303300 .378451
-87.228100 33.381660 683.444500 .579275
-86.100230 33.562890 683.684900 .898805
-84.962690 33.734530 684.016400 1.292948
-83.815900 33.896310 684.399200 1.696965
-82.660300 34.047950 684.740200 2.027031
-81.496410 34.189190 684.916300 2.194279
-80.324730 34.319790 684.858000 2.111381
-79.145840 34.439500 684.530800 1.672940
-77.960310 34.548100 683.757600 .830388
-76.768760 34.645400 682.341700 .668199
-75.571840 34.731200 680.658800 1.725770
-74.370180 34.805370 679.870300 2.207171
-73.164480 34.867740 680.492400 2.005908
-71.955430 34.918200 681.642900 1.351756
-70.743730 34.956650 682.574200 .620012
-69.530080 34.983020 683.065600 .148103
-68.315220 34.997260 683.241100 .157746
-67.099880 34.999340 683.259000 .186093
-65.884770 34.989250 683.219800 .141447
-64.670640 34.967020 683.181300 .086258
-63.458190 34.932690 683.158900 .047404
-62.248150 34.886320 683.149200 .026012
-61.041230 34.828000 683.145400 .015420
-59.838120 34.757850 683.143900 .010041
-58.639500 34.676010 683.142900 .006811
-57.446040 34.582610 683.142100 .004487
-56.258390 34.477850 683.141500 .002792
-55.077160 34.361910 683.141200 .001608
-53.902950 34.235010 683.140900 .000820
-52.736340 34.097380 683.140800 .000412

-51.577860	33.949260	683.140800	.000224
-50.428010	33.790910	683.140800	.000183
-49.287250	33.622600	683.140800	.000182
-48.156040	33.444620	683.140800	.000182
-47.034740	33.257250	683.140800	.000183
-45.923690	33.060790	683.140800	.000183
-44.823220	32.855560	683.140800	.000183
-89.701210	33.918400	683.536300	.723480
-88.583520	34.125020	683.679400	.865181
-87.455040	34.322360	684.151600	1.330763
-86.316120	34.510110	684.924200	2.042374
-85.167100	34.687960	685.961000	2.898103
-84.008420	34.855620	687.144300	3.757812
-82.840550	35.012790	688.192000	4.450247
-81.663990	35.159200	688.740500	4.810431
-80.479300	35.294580	688.619100	4.685706
-79.287040	35.418700	687.745500	3.887011
-78.087840	35.531310	685.608600	2.268264
-76.882320	35.632210	681.746800	1.167822
-75.671150	35.721200	676.675800	2.807488
-74.455030	35.798110	674.577200	3.542389
-73.234660	35.862810	676.271000	3.265035
-72.010770	35.915150	679.433800	2.202758
-70.784100	35.955040	681.842900	.977852
-69.555380	35.982390	683.122400	.341217
-68.325390	35.997170	683.565800	.512707
-67.094890	35.999320	683.564500	.506188
-65.864640	35.988860	683.408400	.358680
-64.635410	35.965800	683.273500	.212156
-63.407950	35.930180	683.201800	.118122
-62.183030	35.882080	683.171100	.068264
-60.961370	35.821590	683.159000	.043051
-59.743720	35.748840	683.153100	.029120
-58.530780	35.663950	683.149300	.019872
-57.323260	35.567100	683.145800	.012949
-56.121820	35.458470	683.143600	.007896
-54.927120	35.338260	683.142300	.004435
-53.739770	35.206700	683.141400	.002206
-52.560380	35.064020	683.140900	.000918
-51.389480	34.910500	683.140800	.000365
-50.227610	34.746390	683.140800	.000156
-49.075240	34.571990	683.140800	.000124
-47.932830	34.387580	683.140800	.000122
-46.800740	34.193490	683.140800	.000122
-45.679330	33.990000	683.140800	.000122
-44.568900	33.777440	683.140800	.000122
-89.960660	34.843320	684.686300	2.000966
-88.832330	35.057210	685.332600	2.467653
-87.692770	35.261540	687.211800	3.773807
-86.542320	35.455950	690.159400	5.679064
-85.381340	35.640140	693.998900	7.885592
-84.210270	35.813810	698.258100	10.023860
-83.029580	35.976620	702.021200	11.681040
-81.839790	36.128310	703.989700	12.523150
-80.641480	36.268600	703.681600	12.310400
-79.435230	36.397220	700.999300	10.660930
-78.221690	36.513940	694.176500	7.223507
-77.001530	36.618520	682.098400	3.892663
-75.775430	36.710780	664.715100	4.834212
-74.544130	36.790520	657.994900	6.018011
-73.308360	36.857590	663.330700	5.140043
-72.068890	36.911860	672.882300	3.718012
-70.826490	36.953220	680.101900	1.807592
-69.581950	36.981590	683.926100	1.504671
-68.336070	36.996900	685.181800	1.906361
-67.089650	36.999130	684.974100	1.678935
-65.843500	36.988290	684.245900	1.131910

-64.598400	36.964370	683.679600	.656255
-63.355190	36.927440	683.398900	.374954
-62.114630	36.877570	683.281200	.230219
-60.877510	36.814860	683.230000	.153515
-59.644590	36.739430	683.202800	.106947
-58.416640	36.651430	683.182600	.073096
-57.194370	36.551040	683.164900	.046624
-55.978490	36.438440	683.154500	.028219
-54.769680	36.313860	683.147800	.015511
-53.568590	36.177530	683.143400	.007443
-52.375820	36.029710	683.141700	.002978
-51.191970	35.870660	683.140900	.000990
-50.017550	35.700660	683.140800	.000301
-48.853080	35.520030	683.140800	.000138
-47.698980	35.329070	683.140800	.000122
-46.555660	35.128100	683.140800	.000122
-45.423470	34.917440	683.140800	.000122
-44.302690	34.697410	683.140800	.000122
-90.232250	35.766320	688.412400	5.005271
-89.092860	35.987640	690.910800	6.345519
-87.941790	36.199090	697.478600	9.650458
-86.779330	36.400320	707.331700	14.211140
-85.605870	36.591000	719.699400	19.221340
-84.421870	36.770790	733.138500	23.807600
-83.227800	36.939400	744.946200	27.151920
-82.024180	37.096500	751.023100	28.675920
-80.811620	37.241800	750.262300	28.383060
-79.590740	37.375040	743.528700	25.745000
-78.362190	37.495960	725.419600	20.036410
-77.126670	37.604320	691.981800	14.451520
-75.884930	37.699920	636.133100	13.852600
-74.637700	37.782550	627.845500	14.058920
-73.385770	37.852060	637.041100	10.395580
-72.129940	37.908310	661.351600	5.926351
-70.871030	37.951180	679.441200	5.102607
-69.609860	37.980580	689.158500	5.804645
-68.347290	37.996460	691.863800	6.138484
-67.084140	37.998780	690.297200	5.065457
-65.821270	37.987530	687.201800	3.245603
-64.559530	37.962740	685.090100	1.860253
-63.299760	37.924460	684.132300	1.102684
-62.042780	37.872770	683.735400	.725182
-60.789440	37.807780	683.541200	.510239
-59.540510	37.729610	683.423300	.362288
-58.296810	37.638420	683.329500	.246183
-57.059080	37.534390	683.246600	.154773
-55.828080	37.417740	683.201700	.092031
-54.604500	37.288680	683.170800	.050746
-53.389020	37.147460	683.152400	.022239
-52.182290	36.994370	683.144200	.008504
-50.984880	36.829660	683.141700	.002686
-49.797380	36.653650	683.140900	.000682
-48.620270	36.466650	683.140700	.000239
-47.454020	36.268990	683.140800	.000121
-46.299010	36.060990	683.140800	.000121
-45.155610	35.843010	683.140800	.000121
-44.024070	35.615370	683.140800	.000122
-90.516530	36.687310	698.414400	10.953150
-89.365660	36.916220	706.632100	14.276640
-88.202590	37.134950	726.659100	21.640930
-87.027650	37.343150	755.144200	30.967900
-85.841200	37.540460	790.088800	40.573650
-84.643710	37.726540	826.285300	48.503470
-83.435650	37.901050	859.866100	53.866400
-82.217600	38.063690	874.898200	55.387000
-80.990140	38.214140	870.842500	54.857380
-79.753940	38.352110	861.145300	53.380940

-78.509670	38.477340	822.533300	46.631340
-77.258070	38.589580	734.967700	38.871590
-75.999910	38.688600	575.834600	34.963530
-74.735980	38.774210	572.750100	57.367540
-73.467090	38.846230	619.126500	39.632680
-72.194090	38.904510	655.106800	19.209400
-70.917830	38.948930	690.553800	15.966260
-69.639210	38.979390	712.894600	17.367960
-68.359090	38.995840	716.499800	16.812850
-67.078360	38.998240	708.723600	13.186070
-65.797930	38.986590	696.237500	8.026637
-64.518690	38.960900	689.203400	4.565984
-63.241520	38.921240	686.556600	2.939766
-61.967320	38.867690	685.416000	2.094752
-60.696920	38.800350	684.749100	1.533614
-59.431210	38.719360	684.288000	1.114740
-58.170970	38.624900	683.903200	.767937
-56.917050	38.517150	683.546500	.464650
-55.670200	38.396340	683.393800	.289574
-54.431160	38.262690	683.262600	.155978
-53.200640	38.116470	683.179600	.059792
-51.979310	37.957960	683.151600	.018998
-50.767770	37.787460	683.143200	.005592
-49.566610	37.605300	683.141200	.001379
-48.376340	37.411790	683.140800	.000271
-47.197410	37.207270	683.140700	.000187
-46.030250	36.992090	683.140800	.000118
-44.875180	36.766620	683.140800	.000121
-43.732470	36.531200	683.140800	.000122
-90.814130	37.606180	720.229300	20.861400
-89.651300	37.842850	743.499100	27.980450
-88.475770	38.069030	799.439000	42.322190
-87.287830	38.284350	873.716700	58.670610
-86.087840	38.488450	960.807200	74.123630
-84.876290	38.680970	1051.956000	87.954960
-83.653650	38.861550	1127.805000	89.732920
-82.420500	39.029860	1158.167000	88.679430
-81.177470	39.185590	1139.220000	87.716030
-79.925230	39.328410	1146.926000	92.712890
-78.664510	39.458070	1114.322000	93.484230
-77.396060	39.574290	902.032700	79.656550
-76.120680	39.676830	403.398900	111.023200
-74.839220	39.765490	370.979700	105.307600
-73.552520	39.840080	629.806000	96.221150
-72.261480	39.900440	702.617100	52.537620
-70.967010	39.946460	742.834600	38.164410
-69.670030	39.978020	793.061600	40.730010
-68.371470	39.995060	795.604700	39.120120
-67.072270	39.997550	765.708700	30.253500
-65.773400	39.985470	719.558800	16.203330
-64.475770	39.958870	699.208900	9.421310
-63.180340	39.917780	693.696900	6.961889
-61.888020	39.862300	691.049600	5.491818
-60.599740	39.792560	689.055600	4.206779
-59.316390	39.708680	687.571900	3.149622
-58.038840	39.610860	686.179400	2.217563
-56.767930	39.499290	684.568000	1.404473
-55.504470	39.374200	684.044900	.843675
-54.249260	39.235840	683.545800	.459248
-53.003000	39.084480	683.258700	.146176
-51.766410	38.920440	683.164800	.033478
-50.540120	38.744010	683.145900	.007661
-49.324710	38.555530	683.141700	.001664
-48.120720	38.355340	683.140800	.000316
-46.928620	38.143810	683.140700	.000156
-45.748810	37.921300	683.140700	.000219
-44.581620	37.688170	683.140800	.000120

-43.427300 37.444810 683.140800 .000122
-91.125630 38.522790 757.357100 34.094940
-89.950410 38.767390 817.307000 47.440960
-88.761920 39.001200 963.244100 73.737400
-87.560450 39.223820 1133.472000 101.344200
-86.346370 39.434880 1306.272000 121.940300
-85.120160 39.633990 1484.690000 142.028400
-83.882300 39.820790 1643.046000 133.073900
-82.633390 39.994930 1686.809000 114.828100
-81.374080 40.156070 1626.012000 117.306500
-80.105060 40.303890 1713.448000 138.399700
-78.827100 40.438090 1805.720000 159.490800
-77.540980 40.558400 1580.610000 142.608400
-76.247540 40.664560 550.875900 157.164500
-74.947690 40.756360 29.349890 106.861600
-73.642300 40.833600 681.594800 129.433900
-72.332310 40.896110 846.518300 84.275920
-71.018690 40.943760 882.227800 73.002990
-69.702430 40.976440 973.747300 66.977560
-68.384490 40.994090 969.099800 68.095950
-67.065880 40.996670 876.125600 49.287850
-65.747600 40.984170 753.107400 22.729780
-64.430650 40.956610 717.939200 15.219630
-63.116020 40.914060 714.482600 14.859470
-61.804690 40.856610 709.051700 13.101500
-60.497620 40.784390 703.505000 10.640600
-59.195770 40.697540 698.516500 8.362274
-57.900040 40.596260 693.370600 6.252786
-56.611330 40.480750 687.709600 3.925499
-55.330490 40.351270 685.328500 1.770007
-54.058330 40.208070 683.983000 .803622
-52.795620 40.051450 683.323000 .183788
-51.543080 39.881710 683.179600 .043363
-50.301390 39.699200 683.146700 .011781
-49.071130 39.504250 683.142000 .004043
-47.852850 39.297230 683.140900 .001044
-46.647030 39.078520 683.140700 .000208
-45.454070 38.848490 683.140700 .000187
-44.274280 38.607530 683.140800 .000119
-43.107920 38.356030 683.140800 .000121
-91.451740 39.437030 802.577800 46.819980
-90.263630 39.689750 928.347500 69.688830
-89.061680 39.931370 1241.169000 109.978500
-87.846150 40.161480 1489.600000 140.734400
-86.617400 40.379670 1586.061000 152.081100
-85.375900 40.585540 1798.558000 199.553700
-84.122160 40.778730 2024.105000 164.813300
-82.856800 40.958850 2208.035000 142.985700
-81.580460 41.125550 2219.138000 116.414200
-80.293880 41.278500 2406.760000 173.035700
-78.997860 41.417370 2667.551000 236.674500
-77.693230 41.541890 2540.202000 229.396400
-76.380870 41.651780 1680.683000 108.464800
-75.061690 41.746810 583.624600 208.814500
-73.736660 41.826770 1220.266000 150.740500
-72.406780 41.891490 1180.789000 96.764880
-71.073040 41.940830 1149.042000 98.644040
-69.736500 41.974670 1143.286000 84.934350
-68.398190 41.992950 1059.060000 73.513610
-67.059160 41.995620 829.046600 50.469700
-65.720480 41.982670 674.327600 27.121210
-64.383220 41.954140 744.098100 21.875570
-63.048400 41.910080 777.767300 31.020550
-61.717090 41.850600 761.568900 28.703420
-60.390290 41.775820 735.512300 20.521460
-59.069010 41.685920 714.543000 15.165250
-57.754210 41.581080 700.505100 12.649710

-56.446830	41.461530	690.623000	6.371176
-55.147770	41.327530	683.935600	1.054265
-53.857880	41.179360	682.761400	1.046049
-52.577970	41.017320	683.044600	.478247
-51.308780	40.841740	683.140400	.156995
-50.051000	40.652980	683.149900	.048902
-48.805250	40.451390	683.143800	.015219
-47.572090	40.237360	683.141400	.004232
-46.352010	40.011290	683.140900	.000946
-45.145370	39.773560	683.140700	.000211
-43.952510	39.524580	683.140700	.000225
-42.773630	39.264750	683.140800	.000121
-91.793200	40.348750	819.753200	52.634140
-90.591710	40.609810	975.644200	82.673380
-89.375760	40.859430	1333.704000	127.172000
-88.145600	41.097210	1341.563000	128.325400
-86.901570	41.322720	564.657700	141.418200
-85.644150	41.535550	173.950500	215.069100
-84.373860	41.735290	1121.130000	96.113430
-83.091290	41.921560	1649.816000	68.613110
-81.797160	42.093980	2087.131000	95.960240
-80.492230	42.252200	2829.427000	199.527300
-79.177290	42.395880	3338.025000	291.849900
-77.853240	42.524730	3241.378000	285.755300
-76.521000	42.638450	2255.305000	129.349200
-75.181550	42.736820	1188.566000	169.923500
-73.835900	42.819590	1213.423000	86.802470
-72.485100	42.886590	1067.767000	110.963500
-71.130210	42.937670	1274.081000	110.326300
-69.772340	42.972710	1080.875000	109.595200
-68.412590	42.991630	870.710600	66.703780
-67.052090	42.994400	299.590500	156.903600
-65.691960	42.980990	427.178200	134.561200
-64.333320	42.951450	849.976000	38.685650
-62.977290	42.905830	939.295200	56.819300
-61.624970	42.844260	854.449700	43.134710
-60.277440	42.766850	732.214400	23.054250
-58.935750	42.673790	656.462600	20.689580
-57.600940	42.565280	638.789000	19.387920
-56.273990	42.441570	669.923300	8.217322
-54.955840	42.302930	681.618000	4.622084
-53.647390	42.149640	683.500900	1.903123
-52.349470	41.982030	683.538700	.757094
-51.062890	41.800450	683.346300	.325486
-49.788320	41.605260	683.215600	.133218
-48.526440	41.396860	683.161800	.048430
-47.277780	41.175640	683.145600	.014915
-46.042850	40.942000	683.141500	.003820
-44.822010	40.696380	683.140900	.000762
-43.615560	40.439180	683.140700	.000220
-42.423690	40.170830	683.140800	.000121
-92.150770	41.257820	787.776300	45.764290
-90.935390	41.527410	905.848500	73.057300
-89.704910	41.785250	1180.672000	117.963700
-88.459540	42.030900	1159.601000	130.599400
-87.199620	42.263930	470.951300	143.172200
-85.925610	42.483890	186.619600	128.609700
-84.638040	42.690380	704.117800	140.502500
-83.337520	42.882970	1332.982000	128.330200
-82.024790	43.061280	2259.211000	142.765000
-80.700610	43.224930	3404.639000	301.224400
-79.365870	43.373580	4033.606000	404.189800
-78.021450	43.506890	3983.035000	382.789200
-76.668370	43.624570	3182.852000	253.321700
-75.307630	43.726360	2173.172000	136.926800
-73.940300	43.812040	1495.901000	119.307000
-72.567500	43.881390	766.088400	136.370000

-71.190370 43.934270 1018.324000 98.211810
-69.810050 43.970550 1329.825000 119.157000
-68.427750 43.990140 1273.963000 93.902910
-67.044650 43.993000 601.839400 116.529300
-65.661930 43.979120 736.333700 56.018840
-64.280810 43.948540 1011.264000 72.985960
-62.902460 43.901310 1038.224000 70.791840
-61.528050 43.837570 851.450900 37.419120
-60.158730 43.757450 615.819500 56.439960
-58.795620 43.661140 616.594700 37.173920
-57.439790 43.548850 679.181700 17.901820
-56.092310 43.420850 695.360100 12.272530
-54.754150 43.277410 690.861100 6.383012
-53.426270 43.118850 687.454800 3.173984
-52.109530 42.945500 685.288800 1.761251
-50.804760 42.757750 684.054100 .886750
-49.512690 42.555960 683.455100 .384805
-48.233980 42.340550 683.229900 .143156
-46.969190 42.111940 683.161600 .045882
-45.718810 41.870550 683.144700 .012577
-44.483210 41.616830 683.141400 .002869
-43.262650 41.351200 683.140900 .000520
-42.057300 41.074100 683.140800 .000153
-92.525320 42.164070 734.377300 29.932640
-91.295520 42.442420 783.668900 45.176820
-90.049940 42.708690 889.343700 69.631100
-88.788760 42.962440 821.302700 80.872200
-87.512310 43.203190 376.183800 119.705400
-86.221000 43.430500 349.401800 143.064500
-84.915410 43.643920 1101.789000 123.396600
-83.596150 43.843030 1704.494000 133.995400
-82.263960 44.027400 2710.607000 259.997400
-80.919660 44.196660 4233.615000 410.235400
-79.564150 44.350410 4729.694000 483.286700
-78.198390 44.488330 4328.265000 416.102500
-76.823410 44.610100 3135.466000 243.143700
-75.440290 44.715440 1909.133000 84.208230
-74.050180 44.804110 1006.331000 102.565100
-72.654240 44.875890 103.301600 142.397500
-71.253690 44.930630 970.372600 172.270500
-69.849760 44.968180 1745.841000 181.835600
-68.443720 44.988460 2047.829000 211.824500
-67.036810 44.991420 1251.492000 65.021740
-65.630330 44.977050 865.341100 61.419950
-64.225530 44.945390 797.128500 76.802730
-62.823690 44.896510 917.322000 54.927700
-61.426040 44.830540 832.407100 51.971240
-60.033810 44.747610 689.610400 50.250350
-58.648170 44.647940 733.404800 33.442200
-57.270290 44.531750 734.787400 25.533130
-55.901260 44.399320 695.795500 17.634040
-54.542130 44.250930 698.581700 9.813596
-53.193900 44.086940 695.908100 6.939190
-51.857480 43.907680 690.291100 4.404339
-50.533710 43.713560 686.170700 2.265592
-49.223370 43.504980 684.179100 .974520
-47.927120 43.282370 683.436400 .362776
-46.645540 43.046150 683.211500 .117689
-45.379100 42.796800 683.155000 .033112
-44.128160 42.534760 683.143200 .007996
-42.892960 42.260480 683.141000 .001621
-41.673610 41.974410 683.140800 .000392
-92.917770 43.067330 694.487700 14.390330
-91.673000 43.354680 701.139900 20.926670
-90.411740 43.629630 701.159100 41.283730
-89.134110 43.891690 593.979200 90.533000
-87.840450 44.140390 455.497500 176.945000

-86.531140 44.375260 48.060080 119.941900
-85.206750 44.595830 1024.875000 130.118400
-83.867900 44.801650 2297.645000 197.621900
-82.515380 44.992280 3067.067000 294.685700
-81.150000 45.167310 3764.293000 377.276400
-79.772730 45.326340 3960.886000 368.385300
-78.384560 45.469020 3553.215000 304.740900
-76.986600 45.595010 2528.880000 144.027100
-75.579970 45.704020 1436.484000 99.425790
-74.165890 45.795780 644.803500 146.965500
-72.745610 45.870080 72.264380 139.260600
-71.320400 45.926740 412.083400 214.999000
-69.891600 45.965610 1587.611000 140.366500
-68.460530 45.986610 2094.437000 186.075300
-67.028560 45.989670 1270.250000 81.458080
-65.597030 45.974800 697.182600 132.696200
-64.167300 45.942020 818.251600 57.436550
-62.740720 45.891430 1053.507000 71.960950
-61.318600 45.823140 806.117800 58.452560
-59.902260 45.737310 695.422100 55.559890
-58.492950 45.634170 670.374500 51.803090
-57.091900 45.513950 660.400400 47.209400
-55.700260 45.376930 700.880100 25.933650
-54.319140 45.223450 737.347100 18.368750
-52.949590 45.053840 726.068400 16.353410
-51.592580 44.868490 704.499800 10.108340
-50.248970 44.667810 691.626300 5.033465
-48.919560 44.452230 685.965900 2.117090
-47.605040 44.222190 683.936800 .773229
-46.305980 43.978160 683.332300 .249156
-45.022840 43.720610 683.180300 .070835
-43.755960 43.450020 683.147700 .017572
-42.505550 43.166850 683.141700 .003768
-41.271660 42.871580 683.140900 .000924
-93.329110 43.967430 676.790200 6.244719
-92.068780 44.264030 660.864900 11.916460
-90.791210 44.547890 592.875300 38.039950
-89.496510 44.818510 356.342300 109.819700
-88.184940 45.075390 406.533900 124.910700
-86.856880 45.318050 302.555100 165.098900
-85.512890 45.545990 1042.320000 93.254320
-84.153590 45.758730 2254.829000 211.166600
-82.779790 45.955820 2551.027000 225.137800
-81.392350 46.136820 2346.014000 131.048900
-79.992260 46.301310 2091.956000 115.244800
-78.580580 46.448910 2003.537000 115.689400
-77.158460 46.579270 1814.369000 60.848930
-75.727110 46.692070 1720.930000 65.614390
-74.287820 46.787040 1791.359000 143.670000
-72.841890 46.863950 1444.197000 115.027700
-71.390720 46.922590 1275.696000 104.755800
-69.935700 46.962830 1997.004000 176.428700
-68.478260 46.984570 1990.574000 163.366400
-67.019850 46.987740 1455.730000 76.423730
-65.561920 46.972340 878.859100 82.106770
-64.105920 46.938410 947.690200 59.547070
-62.653260 46.886040 1069.423000 59.533060
-61.205390 46.815350 763.990300 84.582700
-59.763680 46.726520 738.533900 68.369140
-58.329470 46.619790 815.113000 41.718470
-56.904060 46.495390 825.558300 34.925880
-55.488670 46.353640 840.508500 37.396240
-54.084500 46.194880 862.415800 41.768660
-52.692630 46.019470 804.045700 33.878680
-51.314070 45.827840 736.483800 19.718840
-49.949730 45.620390 702.178600 9.356482
-48.600430 45.397590 689.210800 3.829279

-47.266860	45.159900	684.826200	1.369553
-45.949600	44.907820	683.545500	.434371
-44.649100	44.641840	683.225300	.122924
-43.365670	44.362450	683.156000	.030882
-42.099450	44.070140	683.143100	.006830
-40.850440	43.765410	683.141200	.001769
-93.760410	44.864160	680.788800	6.464308
-92.483920	45.170280	674.343000	11.975660
-91.189420	45.463320	632.947500	31.591180
-89.876960	45.742750	470.254200	46.612030
-88.546760	46.008070	393.019100	74.623500
-87.199170	46.258760	204.168500	117.667300
-85.834720	46.494300	881.859700	54.954400
-84.454070	46.714200	1523.238000	120.102900
-83.058010	46.917960	1320.674000	60.980110
-81.647480	47.105130	714.761500	248.820500
-80.223460	47.275260	802.689700	210.358900
-78.787090	47.427960	1054.334000	161.874100
-77.339580	47.562840	989.071000	218.976300
-75.882230	47.679570	1374.219000	110.692200
-74.416370	47.777870	1965.893000	98.513190
-72.943440	47.857470	2157.947000	149.244000
-71.464880	47.918190	2068.416000	139.293400
-69.982220	47.959850	1940.342000	152.997300
-68.496960	47.982350	1699.780000	118.288300
-67.010670	47.985630	1344.935000	82.651180
-65.524890	47.969690	951.566700	73.948340
-64.041170	47.934570	1154.068000	76.961750
-62.561040	47.880340	1249.866000	80.443890
-61.086020	47.807170	1168.985000	53.510750
-59.617590	47.715230	1057.556000	62.872780
-58.157170	47.604770	1159.548000	64.216790
-56.706150	47.476050	1167.798000	80.979920
-55.265830	47.329400	1155.549000	84.621590
-53.837470	47.165180	1123.357000	79.114200
-52.422200	46.983780	953.386900	56.892900
-51.021090	46.785640	788.223900	31.690660
-49.635090	46.571200	716.681600	14.144080
-48.265020	46.340950	693.083500	5.587956
-46.911610	46.095370	685.840400	1.965731
-45.575410	45.834990	683.788000	.616143
-44.256860	45.560320	683.276300	.172759
-42.956210	45.271870	683.165700	.043274
-41.673580	44.970160	683.144800	.009708
-40.408870	44.655690	683.141500	.002555
-94.212860	45.757310	697.498400	10.837800
-92.919560	46.073230	697.920400	15.760780
-91.607470	46.375730	663.307400	30.603690
-90.276550	46.664250	486.304100	43.829060
-88.926960	46.938270	389.141400	65.571280
-87.559010	47.197250	568.419000	33.274550
-86.173240	47.440650	765.344800	45.407400
-84.770290	47.667940	686.316700	77.912770
-83.350960	47.878600	436.744200	222.011300
-81.916200	48.072160	992.758900	58.081400
-80.467090	48.248140	1315.689000	54.157060
-79.004780	48.406110	1255.643000	73.877400
-77.530590	48.545680	1288.826000	126.724000
-76.045850	48.666500	1660.122000	92.908770
-74.552020	48.768250	1921.940000	113.343700
-73.050600	48.850660	1941.198000	150.166100
-71.543170	48.913520	1936.291000	149.352500
-70.031330	48.956650	1861.122000	137.870600
-68.516710	48.979950	1453.834000	83.053540
-67.000980	48.983350	684.038500	149.851000
-65.485790	48.966840	150.858400	193.167600
-63.972820	48.930470	789.744900	80.003710

-62.463700	48.874330	1310.744000	89.338260
-60.960050	48.798580	1559.547000	117.693200
-59.463460	48.703410	1542.882000	106.764500
-57.975440	48.589080	1451.596000	108.291000
-56.497490	48.455880	1384.705000	115.924100
-55.030970	48.304140	1369.950000	117.949100
-53.577210	48.134270	1315.762000	108.599200
-52.137430	47.946660	1042.926000	73.515340
-50.712720	47.741790	819.969300	38.829230
-49.304080	47.520120	725.835000	16.720360
-47.912350	47.282170	695.383300	6.538520
-46.538250	47.028450	686.359000	2.253770
-45.182340	46.759510	683.892600	.694207
-43.845020	46.475880	683.298200	.192876
-42.526500	46.178100	683.169900	.048131
-41.226800	45.866700	683.145600	.010774
-39.945740	45.542200	683.141700	.002847
-94.687740	46.646660	728.484100	23.010530
-93.376940	46.972690	746.976400	27.389920
-92.046550	47.284930	750.620200	33.747180
-90.696440	47.582840	704.658800	29.317370
-89.326680	47.865840	700.301000	30.780000
-87.937550	48.133390	814.642900	41.046400
-86.529520	48.384910	745.420400	63.166070
-85.103260	48.619850	697.311300	124.451800
-83.659580	48.837660	1299.212000	108.718700
-82.199460	49.037830	1733.146000	166.612100
-80.724000	49.219880	1873.318000	162.499600
-79.234450	49.383340	1563.482000	92.553210
-77.732160	49.527770	1473.054000	73.798360
-76.218590	49.652830	1610.461000	97.327140
-74.695260	49.758160	1800.906000	128.172200
-73.163800	49.843490	1789.475000	148.156800
-71.625870	49.908580	1772.173000	152.663800
-70.083210	49.953250	1737.387000	142.738200
-68.537570	49.977380	1554.220000	107.065200
-66.990740	49.980900	1121.175000	58.353610
-65.444490	49.963800	367.911200	162.002500
-63.900620	49.926140	454.922000	129.626600
-62.360880	49.868010	857.828700	68.329020
-60.827010	49.789570	1243.653000	82.103030
-59.300730	49.691040	1426.551000	104.546900
-57.783640	49.572690	1415.195000	103.870500
-56.277320	49.434830	1291.646000	110.866500
-54.783270	49.277820	1220.391000	99.697410
-53.302860	49.102080	1077.774000	86.478990
-51.837360	48.908040	891.890900	60.628680
-50.387950	48.696200	765.655200	31.469010
-48.955640	48.467040	711.312900	14.186720
-47.541310	48.221130	692.231800	5.606210
-46.145660	47.959000	685.633600	1.952585
-44.769230	47.681210	683.732400	.603392
-43.412380	47.388330	683.263700	.167181
-42.075270	47.080920	683.164100	.041468
-40.757810	46.759540	683.144700	.009247
-39.459720	46.424710	683.141500	.002447
-95.186440	47.531940	778.164300	40.889250
-93.857410	47.868410	817.708400	47.336470
-92.508000	48.190720	857.353500	56.403370
-91.137910	48.498310	893.385600	62.040640
-89.747150	48.790600	985.108500	72.482610
-88.335950	49.067010	1131.543000	89.904320
-86.904710	49.326930	1211.827000	88.890700
-85.454100	49.569790	1288.743000	94.187220
-83.984930	49.795020	1550.352000	148.063100
-82.498220	50.002060	1694.922000	153.499400
-80.995120	50.190400	1599.603000	136.051500

-79.476920	50.359550	1422.760000	117.308100
-77.945060	50.509060	1575.231000	102.292200
-76.401090	50.638520	1733.718000	130.769800
-74.846650	50.747580	1877.921000	155.365000
-73.283450	50.835950	1753.401000	154.639200
-71.713320	50.903350	1627.588000	143.433400
-70.138080	50.949630	1586.757000	122.314600
-68.559640	50.974620	1481.118000	110.132300
-66.979900	50.978270	1380.863000	97.797380
-65.400800	50.960560	1168.894000	92.871350
-63.824260	50.921550	871.921500	66.209750
-62.252170	50.861340	713.991100	104.414000
-60.686400	50.780110	981.670300	75.293820
-59.128770	50.678080	1210.875000	93.586070
-57.581030	50.555550	1249.553000	106.431700
-56.044860	50.412850	1196.016000	96.188210
-54.521820	50.250360	1031.837000	81.168760
-53.013420	50.068520	877.656700	59.682030
-51.520960	49.867810	778.778800	38.523990
-50.045680	49.648740	723.671300	21.318020
-48.588630	49.411830	698.043000	9.653519
-47.150700	49.157670	688.019300	3.815069
-45.732590	48.886830	684.503400	1.333555
-44.334810	48.599900	683.465600	.414323
-42.957660	48.297460	683.209800	.115130
-41.601190	47.980120	683.153700	.028504
-40.265250	47.648430	683.142900	.006248
-38.949390	47.302930	683.141200	.001635
-95.710510	48.412890	859.457900	61.765500
-94.362470	48.760130	943.972700	73.112310
-92.993230	49.092850	1029.039000	87.770110
-91.602360	49.410460	1116.848000	96.223430
-90.189750	49.712350	1221.357000	102.904400
-88.755540	49.997940	1277.053000	108.909500
-87.300090	50.266570	1262.556000	106.767300
-85.824040	50.517650	1228.898000	108.043400
-84.328210	50.750570	1272.562000	123.631500
-82.813610	50.964740	1350.857000	129.635900
-81.281460	51.159630	1396.573000	131.834100
-79.733130	51.334700	1470.772000	118.666100
-78.170130	51.489480	1600.107000	131.473100
-76.594090	51.623530	1654.133000	149.529800
-75.006800	51.736490	1718.042000	157.243700
-73.410070	51.828010	1599.140000	149.005700
-71.805870	51.897850	1502.835000	130.103800
-70.196170	51.945790	1424.649000	103.945300
-68.582990	51.971680	1299.519000	76.760420
-66.968440	51.975460	1222.166000	72.756100
-65.354560	51.957120	1231.964000	85.456370
-63.743440	51.916690	1122.309000	71.209700
-62.137130	51.854320	973.803800	63.227850
-60.537630	51.770170	1009.674000	78.915310
-58.946900	51.664510	1122.877000	93.920070
-57.366820	51.537620	1161.484000	93.430660
-55.799180	51.389870	1053.566000	84.247340
-54.245650	51.221680	916.389800	62.377750
-52.707820	51.033510	816.013600	40.729000
-51.187080	50.825860	754.665800	25.604100
-49.684720	50.599280	712.101100	13.595020
-48.201800	50.354330	692.718800	6.051408
-46.739240	50.091630	685.921900	2.356171
-45.297720	49.811770	683.864000	.814526
-43.877700	49.515370	683.309400	.251442
-42.479410	49.203060	683.175700	.068962
-41.102810	48.875440	683.147200	.016661
-39.747590	48.533100	683.141800	.003582
-38.413150	48.176570	683.140900	.000938

-96.261670	49.289190	972.379000	81.518450
-94.893750	49.647600	1134.386000	95.379550
-93.503820	49.991080	1257.050000	108.808800
-92.091320	50.319060	1268.394000	109.633400
-90.655950	50.630910	1199.942000	104.616100
-89.197760	50.925990	1114.694000	99.713890
-87.717060	51.203660	1051.562000	93.253650
-86.214420	51.463270	1019.772000	89.304510
-84.690660	51.704190	1060.496000	95.125200
-83.146820	51.925780	1194.096000	111.476600
-81.584160	52.127480	1432.320000	117.570700
-80.004110	52.308710	1489.309000	127.510500
-78.408280	52.468990	1422.284000	130.402500
-76.798410	52.607840	1455.444000	133.197100
-75.176390	52.724850	1542.943000	138.095100
-73.544200	52.819690	1443.628000	133.473900
-71.903920	52.892050	1406.602000	115.158100
-70.257710	52.941730	1359.879000	85.767930
-68.607750	52.968570	1149.790000	49.798780
-66.956280	52.972490	1016.691000	47.403920
-65.305560	52.953480	1163.848000	64.339980
-63.657820	52.911580	1160.826000	84.984630
-62.015270	52.846950	1102.528000	83.056440
-60.380080	52.759750	1099.198000	87.528910
-58.754360	52.650280	1114.511000	88.282010
-57.140120	52.518850	1030.715000	81.236550
-55.539290	52.365840	911.697300	63.417490
-53.953660	52.191710	818.689600	44.958680
-52.384890	51.996940	756.737500	28.680290
-50.834480	51.782070	719.928600	16.622160
-49.303730	51.547700	697.912200	8.281844
-47.793770	51.294400	688.018800	3.576540
-46.305490	51.022820	684.569700	1.369541
-44.839570	50.733620	683.513300	.466590
-43.396390	50.427420	683.226500	.140689
-41.976090	50.104880	683.158500	.037670
-40.578500	49.766630	683.144000	.008948
-39.203150	49.413260	683.141200	.001869
-37.849210	49.045330	683.140900	.000465
-96.841810	50.160530	995.129500	79.276950
-95.453030	50.530490	1157.192000	96.016000
-94.041510	50.885130	1147.781000	100.884200
-92.606450	51.223850	1073.370000	99.829480
-91.147380	51.546020	989.926800	89.761540
-89.664200	51.850960	915.552700	78.473430
-88.157130	52.138020	870.801600	69.232300
-86.626690	52.406500	864.395100	65.277240
-85.073680	52.655720	926.074500	69.400960
-83.499180	52.885050	1091.630000	82.889700
-81.904440	53.093850	1188.311000	97.077920
-80.290990	53.281520	1227.141000	103.396400
-78.660530	53.447530	1173.759000	102.725700
-77.014920	53.591380	1252.593000	108.464500
-75.356160	53.712650	1378.838000	120.457500
-73.686420	53.810940	1288.049000	119.954500
-72.007930	53.885960	1270.561000	101.683100
-70.322990	53.937460	1250.101000	75.149310
-68.634010	53.965280	1063.559000	51.874190
-66.943400	53.969350	821.580900	88.427500
-65.253590	53.949630	1033.638000	41.000540
-63.567000	53.906200	1188.130000	79.318950
-61.886040	53.839190	1165.601000	93.744030
-60.213040	53.748820	1106.567000	87.005950
-58.550290	53.635360	1000.180000	77.985130
-56.899960	53.499180	882.442900	61.294500
-55.264110	53.340690	795.630200	44.291070
-53.644650	53.160350	756.074400	31.377650

-52.043340 52.958710 721.672600 19.271780
-50.461760 52.736320 700.608300 10.207040
-48.901280 52.493820 689.950100 4.843776
-47.363030 52.231850 685.479400 2.049890
-45.847900 51.951060 683.843800 .770734
-44.356530 51.652150 683.325800 .256827
-42.889220 51.335790 683.183900 .076671
-41.445980 51.002650 683.149600 .020455
-40.026490 50.653380 683.142400 .004791
-38.630060 50.288590 683.141000 .000982
-37.255620 49.908830 683.140800 .000290
-97.453060 51.026530 858.642300 58.666160
-96.042340 51.408490 911.851100 66.029120
-94.608220 51.774700 923.686700 72.314200
-93.149600 52.124570 912.292100 73.446930
-91.665800 52.457450 883.058000 71.294010
-90.156560 52.772640 840.802700 65.122650
-88.621980 53.069450 806.061700 56.657630
-87.062480 53.347160 780.941700 47.511650
-85.478850 53.605050 761.096700 42.099580
-83.872130 53.842430 697.291300 51.369560
-82.243660 54.058630 558.709500 88.889430
-80.595020 54.253030 715.364000 55.722500
-78.928000 54.425040 908.861600 69.533260
-77.244600 54.574130 1090.380000 85.491410
-75.546970 54.699840 1260.573000 101.112200
-73.837420 54.801750 1176.375000 104.403900
-72.118360 54.879540 1147.770000 80.889810
-70.392330 54.932960 1053.571000 60.332690
-68.661900 54.961820 845.436300 75.848530
-66.929700 54.966030 648.229300 141.287200
-65.198370 54.945580 887.077900 57.807090
-63.470540 54.900540 1082.064000 69.252500
-61.748820 54.831050 1106.415000 85.416090
-60.035740 54.737340 981.687600 74.856510
-58.333780 54.619720 868.624500 56.302150
-56.645260 54.478570 792.485300 39.223210
-54.972420 54.314330 742.250600 28.187610
-53.317280 54.127510 726.888500 22.414660
-51.681740 53.918690 708.881200 14.839250
-50.067420 53.688470 694.073300 7.317336
-48.475770 53.437500 687.179100 3.246418
-46.907930 53.166490 684.504000 1.318247
-45.364770 52.876130 683.550500 .480443
-43.846840 52.567130 683.249500 .159271
-42.354370 52.240220 683.166500 .047961
-40.887210 51.896080 683.146100 .012897
-39.444820 51.535370 683.141800 .003057
-38.026250 51.158730 683.140900 .000645
-36.630120 50.766700 683.140800 .000205
-98.097820 51.886770 756.764600 32.548890
-96.663910 52.281200 769.435300 35.592840
-95.206040 52.659450 775.584000 39.262800
-93.722790 53.020900 782.080000 43.929040
-92.213200 53.364910 794.997500 48.710980
-90.676770 53.690770 811.601200 53.210400
-89.113440 53.997730 811.197300 53.891710
-87.523560 54.285050 787.325600 47.450430
-85.907830 54.551970 730.717400 31.817860
-84.267300 54.797760 588.296100 36.675090
-82.603340 55.021710 251.321300 110.657900
-80.917590 55.223140 80.664750 116.657600
-79.211960 55.401440 530.759900 86.816320
-77.488560 55.556020 953.959000 72.705440
-75.749730 55.686380 1137.223000 86.147000
-73.997920 55.792100 1070.271000 83.922040
-72.235800 55.872810 992.858600 59.788160

-70.466090	55.928230	784.095600	71.989850
-68.691570	55.958180	568.495700	132.022700
-66.915130	55.962550	762.548300	84.197070
-65.139640	55.941330	914.294100	61.284160
-63.367970	55.894590	956.789200	66.324530
-61.602940	55.822490	966.666400	63.166350
-59.847310	55.725280	851.992400	54.416530
-58.103760	55.603290	776.019900	37.271530
-56.374820	55.456930	731.236300	23.569870
-54.662870	55.286670	712.761900	15.460160
-52.970100	55.093080	710.681800	15.348790
-51.298500	54.876750	707.704800	14.165830
-49.649800	54.638340	693.933400	7.096510
-48.025460	54.378550	686.950100	2.952157
-46.426660	54.098120	684.406900	1.162040
-44.854210	53.797780	683.525600	.418330
-43.308580	53.478290	683.245000	.138195
-41.789850	53.140400	683.165800	.042128
-40.297680	52.784830	683.146000	.011678
-38.831280	52.412250	683.141800	.002869
-37.389370	52.023280	683.140900	.000620
-35.970190	51.618480	683.140800	.000218
-98.778840	52.740810	710.821400	15.827200
-97.320270	53.148240	711.830400	16.208570
-95.837370	53.539010	714.104700	17.881540
-94.328290	53.912530	717.671000	20.506030
-92.791720	54.268120	726.103500	24.706220
-91.226900	54.605080	740.589800	30.814940
-89.633560	54.922620	759.654800	37.434470
-88.011890	55.219970	779.387800	42.848280
-86.362530	55.496330	772.650600	42.381380
-84.686490	55.750910	699.339500	34.786380
-82.985150	55.982970	495.172200	48.058000
-81.260250	56.191770	49.412160	99.739630
-79.513790	56.376650	146.556900	125.530900
-77.748020	56.536990	883.935500	70.593910
-75.965460	56.672260	1032.951000	78.747470
-74.168790	56.781970	938.641800	71.639420
-72.360860	56.865740	843.357700	66.241670
-70.544630	56.923280	676.654500	78.249660
-68.723180	56.954370	637.297800	102.670400
-66.899620	56.958910	964.149700	71.286770
-65.077100	56.936880	981.469700	66.296140
-63.258730	56.888360	872.196100	54.723490
-61.447630	56.813510	777.927200	45.202400
-59.646790	56.712620	762.063500	29.881200
-57.859090	56.586040	734.437100	24.007410
-56.087300	56.434210	705.812400	13.430120
-54.333970	56.257640	696.549300	7.800390
-52.601470	56.056940	700.459400	9.994272
-50.891880	55.832740	708.710600	14.565280
-49.207030	55.585770	696.169900	8.355110
-47.548420	55.316760	688.025200	3.483399
-45.917200	55.026500	684.788100	1.371784
-44.314130	54.715760	683.663000	.499284
-42.739580	54.385350	683.286600	.165299
-41.193420	54.036030	683.176900	.050768
-39.675070	53.668560	683.148600	.014430
-38.183400	53.283620	683.142300	.003706
-36.716770	52.881820	683.140900	.000850
-35.272900	52.463700	683.140800	.000283
-99.499240	53.588130	692.308000	6.630859
-98.014280	54.009130	692.003700	6.514535
-96.504860	54.412960	692.545400	7.090480
-94.968610	54.799050	693.747500	8.258293
-93.403790	55.166730	696.700600	10.335570
-91.809290	55.515250	702.454500	13.646470

-90.184560 55.843850 711.965500 18.280670
-88.529620 56.151670 725.336500 24.167480
-86.844990 56.437890 737.423500 29.952760
-85.131650 56.701690 741.729300 36.942490
-83.390960 56.942230 684.205600 53.637900
-81.624720 57.158780 438.669400 64.545720
-79.835050 57.350580 248.480700 109.046700
-78.024370 57.516980 871.583700 75.425040
-76.195360 57.657400 952.936400 78.672200
-74.350940 57.771310 812.631500 85.577160
-72.494220 57.858320 807.477100 63.965500
-70.628410 57.918090 777.935700 49.076210
-68.756890 57.950390 820.176400 67.744530
-66.883060 57.955100 997.942600 67.538340
-65.010370 57.932220 949.929200 65.010440
-63.142240 57.881810 817.414300 46.571540
-61.282040 57.804080 717.772900 24.585760
-59.433060 57.699310 691.166900 18.282940
-57.598450 57.567890 687.785500 19.618610
-55.781200 57.410300 678.204300 11.918660
-53.984060 57.227100 681.730300 4.485716
-52.209560 57.018940 691.353300 4.987551
-50.459930 56.786500 705.977700 13.073620
-48.737060 56.530560 701.780300 11.117030
-47.042490 56.251900 690.800500 4.952031
-45.377330 55.951360 685.653800 1.855374
-43.742270 55.629770 683.914400 .663477
-42.137470 55.287960 683.359000 .218185
-40.562610 54.926740 683.195800 .066996
-39.016750 54.546860 683.153200 .019238
-37.498410 54.149020 683.143300 .005050
-36.005400 53.733830 683.141200 .001198
-34.534880 53.301770 683.140900 .000360
-100.262700 54.428120 685.810900 2.467038
-98.749250 54.863350 685.565100 2.321684
-97.211560 55.280850 685.702000 2.480803
-95.646590 55.680060 686.133900 2.935545
-94.052090 56.060360 687.127700 3.821085
-92.426510 56.420970 688.978600 5.278880
-90.768940 56.761100 692.095900 7.414990
-89.079190 57.079890 696.598900 10.289010
-87.357570 57.376450 701.638900 13.810620
-85.605000 57.649890 705.807400 19.820150
-83.822840 57.899360 696.499000 31.760970
-82.012920 58.124030 612.096900 42.777180
-80.177480 58.323120 565.410800 53.431610
-78.319110 58.495910 886.177800 88.781430
-76.440710 58.641750 892.997800 80.408450
-74.545430 58.760110 809.049200 77.944300
-72.636660 58.850530 861.833100 60.045710
-70.717930 58.912650 886.314800 39.943980
-68.792920 58.946230 900.586900 47.734210
-66.865380 58.951130 966.696200 59.132490
-64.939080 58.927340 862.924900 52.975930
-63.017780 58.874950 758.690700 30.629590
-61.105210 58.794160 691.979900 16.088690
-59.204930 58.685300 682.833400 9.915109
-57.320400 58.548780 675.090100 18.285600
-55.454840 58.385130 654.252100 21.065820
-53.611270 58.194950 663.346900 13.693840
-51.792370 57.978940 676.914600 5.221770
-50.000500 57.737840 696.035000 8.208678
-48.237630 57.472490 706.620300 13.548750
-46.505310 57.183730 693.493200 6.444181
-44.804610 56.872430 686.480500 2.288034
-43.136050 56.539500 684.144200 .803974
-41.499610 56.185780 683.428800 .267391

-39.894630 55.812120 683.215000 .082104
-38.319800 55.419290 683.158100 .023796
-36.773090 55.007980 683.144200 .006386
-35.251730 54.578750 683.141400 .001570
-33.752120 54.132060 683.140900 .000471
-101.073700 55.260130 683.824500 .828246
-99.529010 55.710320 683.729700 .746291
-97.960900 56.142140 683.760800 .777624
-96.365430 56.555100 683.900100 .925273
-94.739640 56.948590 684.220800 1.239858
-93.081440 57.321860 684.823300 1.786675
-91.389490 57.674070 685.792400 2.626434
-89.663250 58.004340 686.997100 3.795961
-87.902820 58.311730 687.911600 5.546503
-86.109000 58.595320 687.818700 10.133190
-84.283100 58.854170 678.918200 20.765380
-82.427000 59.087400 628.741000 27.633300
-80.543050 59.294160 676.586500 38.271040
-78.634010 59.473690 891.590900 73.070550
-76.703000 59.625280 823.531700 74.036670
-74.753460 59.748340 807.747900 75.175580
-72.789070 59.842370 900.897400 56.481640
-70.813740 59.906970 897.741900 39.694430
-68.831480 59.941910 944.454600 44.030460
-66.846440 59.947010 911.131100 55.831970
-64.862770 59.922250 805.936100 39.725450
-62.884610 59.867760 728.634800 19.741450
-60.916040 59.783740 686.303000 10.234310
-58.961010 59.670550 680.445600 5.920304
-57.023270 59.528640 682.786600 11.039280
-55.106350 59.358580 662.684000 15.484560
-53.213500 59.161040 647.288500 21.523830
-51.347600 58.936760 656.403100 16.247090
-49.511150 58.686560 677.965300 5.668211
-47.706190 58.411320 700.272600 10.845050
-45.934240 58.111950 692.755100 6.174651
-44.196280 57.789400 686.487400 2.241682
-42.492650 57.444580 684.193800 .809773
-40.823030 57.078400 683.460300 .283361
-39.186360 56.691730 683.227100 .089464
-37.580830 56.285340 683.161300 .026140
-36.003750 55.859910 683.145100 .007135
-34.451600 55.415950 683.141700 .001810
-32.919830 54.953830 683.140900 .000577
-101.937200 56.083360 683.296100 .250782
-100.358100 56.549350 683.267000 .215867
-98.756940 56.996240 683.275000 .221026
-97.128790 57.423640 683.315500 .264843
-95.469860 57.830970 683.408300 .360643
-93.777320 58.217490 683.583800 .528181
-92.049300 58.582370 683.852700 .791962
-90.284800 58.924680 684.089000 1.267142
-88.483640 59.243460 683.912400 2.709943
-86.646400 59.537720 682.711900 8.341322
-84.774360 59.806450 671.824800 20.355340
-82.869400 60.048700 622.689600 28.852110
-80.933970 60.263580 699.374500 24.643050
-78.971000 60.450230 846.637600 47.829720
-76.983890 60.607900 692.010900 62.165410
-74.976370 60.735940 714.839800 70.690380
-72.952470 60.833800 801.866400 54.850230
-70.916480 60.901050 869.926900 44.914810
-68.872850 60.937420 935.726600 48.365140
-66.826130 60.942730 878.275300 48.552960
-64.780930 60.916960 773.514600 30.535330
-62.741830 60.860230 716.199800 15.151240
-60.713310 60.772790 689.734200 6.516590

-58.699720	60.654990	682.459200	2.237419
-56.705200	60.507380	684.808800	3.988878
-54.733590	60.330550	676.147900	5.918812
-52.788400	60.125220	665.819200	11.268770
-50.872730	59.892220	655.380000	16.255000
-48.989200	59.632420	658.675200	14.436460
-47.139900	59.346780	684.476000	6.000152
-45.326320	59.036270	690.212200	5.149041
-43.549270	58.701890	686.571700	2.284327
-41.808860	58.344600	684.222700	.777003
-40.104370	57.965370	683.464900	.267133
-38.434220	57.565060	683.229900	.086509
-36.795950	57.144450	683.162700	.025690
-35.186060	56.704150	683.145600	.007069
-33.600030	56.244660	683.141700	.001808
-32.032190	55.766200	683.140900	.000603
-102.859900	56.896880	683.172100	.067903
-101.242000	57.379640	683.164800	.055730
-99.604390	57.842470	683.166800	.056364
-97.940930	58.285070	683.177100	.068766
-96.246660	58.706940	683.200800	.094864
-94.517820	59.107400	683.241100	.138320
-92.751850	59.485580	683.273300	.244535
-90.947170	59.840560	683.132000	.704058
-89.103220	60.171320	682.426100	2.437663
-87.220290	60.476800	680.299700	8.390467
-85.299550	60.755960	667.391800	20.864030
-83.342830	61.007760	615.605300	33.345620
-81.352710	61.231220	693.492700	12.967860
-79.332320	61.425420	777.283500	32.355620
-77.285290	61.589540	621.254300	84.104770
-75.215710	61.722870	505.650600	133.017500
-73.128000	61.824790	569.777400	103.566500
-71.026890	61.894870	738.248800	70.886430
-68.917300	61.932760	891.434100	51.466020
-66.804310	61.938290	858.577600	42.950090
-64.692990	61.911450	759.628400	25.846320
-62.588420	61.852340	712.680400	15.339630
-60.495590	61.761240	694.646800	10.254370
-58.419300	61.638580	688.393400	5.867230
-56.364090	61.484900	687.576900	4.161129
-54.334160	61.300890	684.784800	3.208728
-52.333320	61.087320	680.741600	3.516119
-50.364880	60.845090	674.224300	5.919648
-48.431600	60.575160	669.255900	8.233609
-46.535590	60.278550	671.552100	7.504037
-44.678250	59.956310	687.545800	4.155191
-42.860180	59.609490	685.909400	2.060468
-41.081100	59.239120	684.141500	.698113
-39.339830	58.846180	683.414400	.217774
-37.634110	58.431540	683.217000	.070122
-35.960610	57.995940	683.159700	.021232
-34.314830	57.539980	683.145000	.005921
-32.690990	57.064010	683.141700	.001526
-31.082000	56.568130	683.140900	.000530
-103.849600	57.699570	683.146500	.016301
-102.187300	58.200280	683.144800	.012632
-100.509000	58.680030	683.145300	.012530
-98.806840	59.138710	683.147300	.015235
-97.074520	59.575940	683.150400	.020784
-95.307120	59.991050	683.146800	.046106
-93.501110	60.383240	683.085300	.187454
-91.654170	60.751550	682.754400	.719787
-89.765210	61.094930	681.585200	2.502459
-87.834170	61.412270	678.233900	8.249608
-85.861950	61.702450	664.161400	20.414280
-83.850420	61.964360	625.908800	31.394810

-81.802150 62.196920 696.278300 13.647750
-79.720490 62.399150 719.486300 39.976410
-77.609360 62.570130 561.080400 105.692000
-75.473210 62.709080 431.444700 160.255800
-73.316960 62.815350 416.089300 151.504000
-71.145790 62.888430 522.193400 126.902800
-68.965200 62.927950 798.564900 56.203530
-66.780790 62.933720 811.445900 36.699720
-64.598260 62.905720 744.722000 23.270440
-62.423240 62.844070 711.165200 15.573640
-60.261290 62.749080 698.931300 11.991910
-58.117710 62.621230 695.062700 10.603150
-55.997520 62.461110 693.924000 9.979173
-53.905320 62.269450 692.688200 9.277426
-51.845240 62.047150 692.403400 9.652359
-49.820820 61.795150 691.548800 10.100420
-47.834930 61.514500 688.119000 8.306219
-45.889670 61.206310 684.177700 4.174192
-43.986290 60.871700 684.504400 1.382113
-42.125070 60.511780 684.184500 .729915
-40.305270 60.127630 683.512600 .295272
-38.525020 59.720250 683.282700 .119557
-36.781200 59.290500 683.183800 .043048
-35.069400 58.839090 683.152200 .013526
-33.383770 58.366500 683.143300 .003902
-31.716990 57.872960 683.141200 .001054
-30.060130 57.358350 683.140900 .000389
-104.916500 58.490070 683.141700 .003421
-103.202300 59.010120 683.141400 .002446
-101.477600 59.507980 683.141400 .002217
-99.732440 59.983750 683.141200 .002480
-97.958720 60.437220 683.138400 .008506
-96.150010 60.867820 683.119900 .044502
-94.301540 61.274790 683.024400 .188264
-92.410060 61.657170 682.621800 .685985
-90.473700 62.013880 681.272400 2.265942
-88.491920 62.343760 677.416300 7.285781
-86.465330 62.645610 668.443100 16.919420
-84.395630 62.918240 663.399000 19.357900
-82.285480 63.160470 732.866800 21.420670
-80.138370 63.371240 706.684800 27.846950
-77.958560 63.549540 616.190200 58.419040
-75.750930 63.694510 536.439000 91.236080
-73.520860 63.805420 491.743700 108.036800
-71.274150 63.881700 521.360100 96.332120
-69.016910 63.922960 670.201400 50.608950
-66.755400 63.928990 729.366100 22.437340
-64.495980 63.899750 714.902300 14.301990
-62.244980 63.835380 702.648400 12.178730
-60.008560 63.736250 695.857200 10.186120
-57.792640 63.602840 692.973100 8.913456
-55.602750 63.435840 691.684400 8.024560
-53.443960 63.236060 689.677800 6.370981
-51.320750 63.004450 688.269300 5.304240
-49.236890 62.742080 687.844700 5.140291
-47.195330 62.450080 686.474800 4.148537
-45.198120 62.129630 684.415800 2.294034
-43.246250 61.781950 683.719900 .742678
-41.339580 61.408210 683.374900 .260532
-39.476710 61.009530 683.283000 .123458
-37.654880 60.586900 683.196900 .055927
-35.869840 60.141170 683.159400 .020477
-34.115770 59.672970 683.145900 .006898
-32.385130 59.182660 683.142000 .002098
-30.668580 58.670220 683.140900 .000607
-28.954870 58.135290 683.140800 .000269
-106.073800 59.266680 683.140900 .000635

-104.297500 59.807790 683.140900 .000413
-102.518800 60.325180 683.140800 .000275
-100.725000 60.819250 683.140300 .001442
-98.905530 61.289990 683.136200 .008456
-97.052100 61.737010 683.116400 .039177
-95.158340 62.159620 683.025500 .156343
-93.219700 62.556880 682.683800 .531422
-91.233350 62.927700 681.520000 1.744653
-89.198040 63.270870 678.999500 5.149466
-87.113920 63.585110 676.008100 11.848460
-84.982480 63.869130 695.798000 9.147088
-82.806390 64.121680 709.274200 14.721690
-80.589290 64.341560 667.223800 19.699830
-78.335770 64.527670 620.687800 35.165140
-76.051160 64.679080 594.891500 48.372800
-73.741460 64.794950 589.393600 52.624730
-71.413090 64.874680 610.300500 45.199620
-69.072890 64.917820 664.602400 24.303810
-66.727910 64.924110 692.244900 9.697508
-64.385280 64.893550 693.524500 5.818678
-62.052090 64.826280 690.460900 4.972410
-59.735260 64.722690 688.115100 4.250205
-57.441380 64.583340 686.927400 3.674919
-55.176610 64.408980 686.321100 3.222981
-52.946510 64.200520 685.493800 2.492123
-50.755950 63.959000 684.843800 1.923201
-48.608900 63.685590 684.574500 1.690057
-46.508400 63.381530 684.129500 1.291336
-44.456330 63.048090 683.620200 .758133
-42.453320 62.686580 683.286300 .295340
-40.498620 62.298210 683.231400 .113361
-38.589950 61.884150 683.185300 .052565
-36.723370 61.445380 683.159600 .021322
-34.893150 60.982680 683.147100 .008470
-33.091610 60.496560 683.142600 .003028
-31.309040 59.987160 683.141100 .000952
-29.533510 59.454220 683.140900 .000291
-27.750760 58.896990 683.140800 .000166
-107.339200 60.027230 683.140800 .000227
-105.487000 60.591610 683.140800 .000181
-103.643800 61.130270 683.140700 .000311
-101.793500 61.644070 683.140100 .001370
-99.922550 62.133320 683.136800 .006741
-98.020040 62.597820 683.121300 .028806
-96.077510 63.037030 683.057700 .105541
-94.088710 63.450070 682.837600 .332550
-92.049460 63.835860 682.230800 .895424
-89.957580 64.193130 680.853300 2.198347
-87.812570 64.520550 679.621700 3.069214
-85.615560 64.816710 684.730400 7.607483
-83.369120 65.080260 673.971800 14.347880
-81.077050 65.309890 659.091400 16.976980
-78.744270 65.504390 651.348900 18.875760
-76.376630 65.662700 648.154400 21.674610
-73.980770 65.783920 650.199800 21.735850
-71.563890 65.867360 659.368600 17.333430
-69.133670 65.912500 674.429400 10.052420
-66.698060 65.919100 683.793900 4.461517
-64.265090 65.887100 685.538100 2.011232
-61.842790 65.816700 685.224400 1.577992
-59.438900 65.708320 684.627200 1.422290
-57.060850 65.562580 684.269200 1.251269
-54.715470 65.380330 684.072500 1.081842
-52.408880 65.162570 683.838800 .855179
-50.146370 64.910470 683.645800 .665806
-47.932120 64.625290 683.542700 .549790
-45.769150 64.308400 683.405900 .403278

-43.659080	63.961160	683.275700	.245667
-41.602020	63.584970	683.199200	.111553
-39.596350	63.181100	683.167900	.048016
-37.638600	62.750720	683.153800	.018872
-35.723270	62.294770	683.146000	.008094
-33.842640	61.813940	683.142600	.003325
-31.986660	61.308540	683.141400	.001210
-30.142740	60.778430	683.140900	.000407
-28.295590	60.222970	683.140800	.000186
-26.427080	59.640870	683.140800	.000139
-108.736700	60.768890	683.140800	.000186
-106.789100	61.359350	683.140800	.000124
-104.866900	61.921510	683.140700	.000314
-102.949500	62.456830	683.140100	.001456
-101.019200	62.966030	683.136800	.006372
-99.061880	63.449270	683.123000	.024308
-97.066180	63.906190	683.072000	.081000
-95.023590	64.336020	682.914600	.225934
-92.928180	64.737730	682.530100	.496510
-90.776390	65.110020	681.821900	.851898
-88.566850	65.451470	681.062700	1.223317
-86.300130	65.760610	680.821200	5.155974
-83.978550	66.035940	674.703400	8.701771
-81.606060	66.276030	669.483800	9.655389
-79.187900	66.479550	668.155900	9.897879
-76.730480	66.645290	667.895600	10.813730
-74.241170	66.772270	669.244100	10.825270
-71.728080	66.859700	673.124100	8.839250
-69.199870	66.907030	678.508500	5.548625
-66.665550	66.913940	682.256500	2.668442
-64.134220	66.880400	683.619300	1.056855
-61.614990	66.806620	683.805200	.613303
-59.116610	66.693070	683.689500	.588722
-56.647430	66.540450	683.571800	.543162
-54.215080	66.349720	683.489400	.470084
-51.826330	66.121990	683.410500	.378522
-49.486860	65.858530	683.343200	.296738
-47.201050	65.560780	683.293900	.235271
-44.971820	65.230190	683.243100	.170800
-42.800360	64.868270	683.195900	.105077
-40.685980	64.476460	683.165200	.053208
-38.625890	64.056100	683.151700	.022841
-36.614990	63.608330	683.145400	.009700
-34.645670	63.134020	683.142800	.004158
-32.707610	62.633670	683.141500	.001689
-30.787590	62.107330	683.140900	.000674
-28.869240	61.554470	683.140800	.000293
-26.932890	60.973880	683.140800	.000207
-24.955330	60.363580	683.140800	.000186

Q.30a

-89.453400	32.991660	719.684600	.090847
-88.345930	33.191160	719.698300	.116006
-87.228100	33.381660	719.752900	.216206
-86.100230	33.562890	719.850700	.382925
-84.962690	33.734530	719.992900	.599559
-83.815900	33.896310	720.163700	.829089
-82.660300	34.047950	720.315400	1.014173
-81.496410	34.189190	720.379500	1.103111
-80.324730	34.319790	720.334000	1.047836
-79.145840	34.439500	720.173900	.756205
-77.960310	34.548100	719.779100	.132307
-76.768760	34.645400	718.937300	.931776
-75.571840	34.731200	717.777200	1.905419
-74.370180	34.805370	717.236900	2.297181
-73.164480	34.867740	717.826900	2.030166
-71.955430	34.918200	718.710700	1.336027
-70.743730	34.956650	719.301600	.633709
-69.530080	34.983020	719.582000	.178703
-68.315220	34.997260	719.685700	.042731
-67.099880	34.999340	719.700100	.078675
-65.884770	34.989250	719.682300	.060545
-64.670640	34.967020	719.664400	.033188
-63.458190	34.932690	719.654800	.014919
-62.248150	34.886320	719.650900	.005875
-61.041230	34.828000	719.649400	.001901
-59.838120	34.757850	719.648500	.001022
-58.639500	34.676010	719.647900	.001734
-57.446040	34.582610	719.647500	.002016
-56.258390	34.477850	719.647600	.001645
-55.077160	34.361910	719.647900	.001005
-53.902950	34.235010	719.648400	.000476
-52.736340	34.097380	719.648400	.000225
-51.577860	33.949260	719.648500	.000121
-50.428010	33.790910	719.648500	.000118
-49.287250	33.622600	719.648500	.000122
-48.156040	33.444620	719.648500	.000122
-47.034740	33.257250	719.648500	.000122
-45.923690	33.060790	719.648500	.000122
-44.823220	32.855560	719.648500	.000122
-89.701210	33.918400	719.777100	.269495
-88.583520	34.125020	719.841200	.358092
-87.455040	34.322360	720.055200	.642542
-86.316120	34.510110	720.429100	1.092983
-85.167100	34.687960	720.963100	1.657552
-84.008420	34.855620	721.593100	2.229758
-82.840550	35.012790	722.145900	2.684556
-81.663990	35.159200	722.379500	2.900565
-80.479300	35.294580	722.257800	2.796360
-79.287040	35.418700	721.792500	2.172869
-78.087840	35.531310	720.510600	.793947
-76.882320	35.632210	717.778300	1.430828
-75.671150	35.721200	713.724900	3.425210
-74.455030	35.798110	711.940900	4.122415
-73.234660	35.862810	713.904100	3.756588
-72.010770	35.915150	716.813700	2.533933
-70.784100	35.955040	718.683800	1.185942
-69.555380	35.982390	719.554100	.265390
-68.325390	35.997170	719.852200	.191382
-67.094890	35.999320	719.869800	.257481
-65.864640	35.988860	719.783400	.184554
-64.635410	35.965800	719.709500	.100146

-63.407950	35.930180	719.672900	.047365
-62.183030	35.882080	719.658500	.021094
-60.961370	35.821590	719.652800	.008801
-59.743720	35.748840	719.649500	.003953
-58.530780	35.663950	719.646300	.004863
-57.323260	35.567100	719.644200	.005609
-56.121820	35.458470	719.644600	.004592
-54.927120	35.338260	719.646500	.002447
-53.739770	35.206700	719.647900	.001095
-52.560380	35.064020	719.648400	.000380
-51.389480	34.910500	719.648400	.000124
-50.227610	34.746390	719.648400	.000017
-49.075240	34.571990	719.648500	.000000
-47.932830	34.387580	719.648500	.000000
-46.800740	34.193490	719.648500	.000000
-45.679330	33.990000	719.648500	.000000
-44.568900	33.777440	719.648500	.000122
-89.960660	34.843320	720.226900	.938637
-88.832330	35.057210	720.562600	1.266718
-87.692770	35.261540	721.570000	2.184540
-86.542320	35.455950	723.262100	3.567878
-85.381340	35.640140	725.601600	5.216003
-84.210270	35.813810	728.274900	6.822165
-83.029580	35.976620	730.571700	8.028852
-81.839790	36.128310	731.543900	8.568706
-80.641480	36.268600	731.174000	8.317418
-79.435230	36.397220	729.513400	6.897394
-78.221690	36.513940	724.663800	3.861823
-77.001530	36.618520	714.556900	2.223228
-75.775430	36.710780	698.785500	5.918835
-74.544130	36.790520	692.842500	7.143127
-73.308360	36.857590	699.705600	6.588274
-72.068890	36.911860	709.887100	4.787027
-70.826490	36.953220	716.577600	2.176058
-69.581950	36.981590	719.747500	.371598
-68.336070	36.996900	720.794900	.940665
-67.089650	36.999130	720.746400	1.000937
-65.843500	36.988290	720.291900	.674102
-64.598400	36.964370	719.937400	.363823
-63.355190	36.927440	719.769000	.180877
-62.114630	36.877570	719.703500	.090990
-60.877510	36.814860	719.674900	.045429
-59.644590	36.739430	719.656700	.022765
-58.416640	36.651430	719.640800	.018296
-57.194370	36.551040	719.630200	.019902
-55.978490	36.438440	719.632200	.014406
-54.769680	36.313860	719.640600	.007692
-53.568590	36.177530	719.646100	.002881
-52.375820	36.029710	719.647900	.001011
-51.191970	35.870660	719.648400	.000275
-50.017550	35.700660	719.648400	.000087
-48.853080	35.520030	719.648400	.000017
-47.698980	35.329070	719.648500	.000000
-46.555660	35.128100	719.648500	.000000
-45.423470	34.917440	719.648500	.000000
-44.302690	34.697410	719.648500	.000122
-90.232250	35.766320	721.935500	2.719818
-89.092860	35.987640	723.411100	3.730735
-87.941790	36.199090	727.456500	6.258921
-86.779330	36.400320	733.977400	9.866281
-85.605870	36.591000	742.768800	13.990710
-84.421870	36.770790	752.311100	17.707070
-83.227800	36.939400	760.725500	20.376290
-82.024180	37.096500	763.845100	21.203360
-80.811620	37.241800	762.503200	20.592830
-79.590740	37.375040	758.083700	18.562230
-78.362190	37.495960	743.605400	12.828880

-77.126670	37.604320	710.124300	7.760697
-75.884930	37.699920	657.320100	10.739930
-74.637700	37.782550	647.069400	11.860280
-73.385770	37.852060	666.844500	8.971682
-72.129940	37.908310	694.066600	5.678221
-70.871030	37.951180	712.688500	2.605947
-69.609860	37.980580	722.363200	2.497417
-68.347290	37.996460	725.259700	3.902791
-67.084140	37.998780	724.501200	3.521505
-65.821270	37.987530	722.298600	2.206587
-64.559530	37.962740	720.825000	1.160983
-63.299760	37.924460	720.181100	.612029
-62.042780	37.872770	719.921000	.345669
-60.789440	37.807780	719.791500	.203864
-59.540510	37.729610	719.703400	.121094
-58.296810	37.638420	719.628500	.079627
-57.059080	37.534390	719.579800	.065411
-55.828080	37.417740	719.582100	.049505
-54.604500	37.288680	719.626700	.019065
-53.389020	37.147460	719.642500	.008244
-52.182290	36.994370	719.647300	.002570
-50.984880	36.829660	719.648400	.000849
-49.797380	36.653650	719.648400	.000253
-48.620270	36.466650	719.648400	.000043
-47.454020	36.268990	719.648400	.000000
-46.299010	36.060990	719.648500	.000000
-45.155610	35.843010	719.648500	.000000
-44.024070	35.615370	719.648500	.000122
-90.516530	36.687310	727.211300	6.529175
-89.365660	36.916220	732.520600	9.158968
-88.202590	37.134950	746.364300	15.238790
-87.027650	37.343150	767.912700	23.422600
-85.841200	37.540460	796.297200	32.053270
-84.643710	37.726540	826.041500	39.704100
-83.435650	37.901050	852.158000	43.312110
-82.217600	38.063690	858.540300	43.014370
-80.990140	38.214140	852.410900	41.929760
-79.753940	38.352110	847.711700	40.504360
-78.509670	38.477340	816.968800	35.691470
-77.258070	38.589580	724.168200	23.175000
-75.999910	38.688600	568.700400	21.852090
-74.735980	38.774210	518.456400	35.315560
-73.467090	38.846230	629.131800	30.119230
-72.194090	38.904510	677.424900	12.135960
-70.917830	38.948930	713.542500	6.829487
-69.639210	38.979390	738.682000	11.092340
-68.359090	38.995840	744.717700	13.177290
-67.078360	38.998240	739.546100	10.955430
-65.797930	38.986590	729.237200	6.307357
-64.518690	38.960900	723.694200	3.223020
-63.241520	38.921240	721.671900	1.831912
-61.967320	38.867690	720.849800	1.188774
-60.696920	38.800350	720.355800	.814604
-59.431210	38.719360	720.003700	.557444
-58.170970	38.624900	719.682700	.359842
-56.917050	38.517150	719.389200	.246159
-55.670200	38.396340	719.426300	.157283
-54.431160	38.262690	719.602700	.094010
-53.200640	38.116470	719.649600	.024203
-51.979310	37.957960	719.650900	.009481
-50.767770	37.787460	719.649700	.003030
-49.566610	37.605300	719.648700	.000893
-48.376340	37.411790	719.648400	.000240
-47.197410	37.207270	719.648400	.000049
-46.030250	36.992090	719.648400	.000000
-44.875180	36.766620	719.648500	.000000
-43.732470	36.531200	719.648500	.000122

-90.814130	37.606180	739.822300	13.142040
-89.651300	37.842850	756.306600	19.346880
-88.475770	38.069030	799.216600	32.711440
-87.287830	38.284350	862.091800	48.899150
-86.087840	38.488450	940.587700	65.152340
-84.876290	38.680970	1022.376000	76.896560
-83.653650	38.861550	1085.965000	81.099070
-82.420500	39.029860	1091.302000	71.776040
-81.177470	39.185590	1070.294000	65.994420
-79.925230	39.328410	1084.320000	76.122960
-78.664510	39.458070	1062.756000	75.335460
-77.396060	39.574290	868.960100	56.855510
-76.120680	39.676830	440.016800	74.556470
-74.839220	39.765490	303.054800	84.218520
-73.552520	39.840080	569.549300	66.607860
-72.261480	39.900440	706.275200	36.611770
-70.967010	39.946460	744.741000	25.379260
-69.670030	39.978020	798.959500	31.929510
-68.371470	39.995060	811.256100	35.839380
-67.072270	39.997550	787.870200	28.467660
-65.773400	39.985470	748.811800	15.037150
-64.475770	39.958870	731.639800	7.751341
-63.180340	39.917780	726.575900	4.982261
-61.888020	39.862300	724.490500	3.802773
-60.599740	39.792560	723.049700	2.991670
-59.316390	39.708680	721.835400	2.351223
-58.038840	39.610860	720.371600	1.704352
-56.767930	39.499290	719.003300	1.092851
-55.504470	39.374200	719.160800	.581765
-54.249260	39.235840	719.632300	.247989
-53.003000	39.084480	719.712900	.126465
-51.766410	38.920440	719.674400	.029475
-50.540120	38.744010	719.655700	.009124
-49.324710	38.555530	719.650100	.002687
-48.120720	38.355340	719.648700	.000673
-46.928620	38.143810	719.648400	.000131
-45.748810	37.921300	719.648400	.000000
-44.581620	37.688170	719.648500	.000000
-43.427300	37.444810	719.648500	.000122
-91.125630	38.522790	763.026500	22.633890
-89.950410	38.767390	808.721100	35.677500
-88.761920	39.001200	922.581800	63.104420
-87.560450	39.223820	1063.999000	90.924240
-86.346370	39.434880	1211.560000	115.985100
-85.120160	39.633990	1385.589000	132.964600
-83.882300	39.820790	1521.375000	135.198100
-82.633390	39.994930	1523.465000	104.531600
-81.374080	40.156070	1479.647000	87.052260
-80.105060	40.303890	1554.276000	115.502800
-78.827100	40.438090	1626.505000	152.821200
-77.540980	40.558400	1386.494000	142.371100
-76.247540	40.664560	685.290200	132.481000
-74.947690	40.756360	259.069600	191.374000
-73.642300	40.833600	671.652900	96.864810
-72.332310	40.896110	810.753100	64.946910
-71.018690	40.943760	852.407300	47.242230
-69.702430	40.976440	919.496900	64.547810
-68.384490	40.994090	935.725800	71.337340
-67.065880	40.996670	862.277000	53.669690
-65.747600	40.984170	772.684100	22.746940
-64.430650	40.956610	747.944100	14.905630
-63.116020	40.914060	743.473700	12.972360
-61.804690	40.856610	738.476800	11.311220
-60.497620	40.784390	732.830900	9.438282
-59.195770	40.697540	727.474600	7.862077
-57.900040	40.596260	722.061800	6.039775
-56.611330	40.480750	719.149500	3.559729

-55.330490 40.351270 719.973000 1.281307
-54.058330 40.208070 720.132300 .610884
-52.795620 40.051450 719.910200 .225590
-51.543080 39.881710 719.737800 .074335
-50.301390 39.699200 719.671300 .020988
-49.071130 39.504250 719.653500 .005649
-47.852850 39.297230 719.649200 .001416
-46.647030 39.078520 719.648400 .000306
-45.454070 38.848490 719.648400 .000057
-44.274280 38.607530 719.648500 .000000
-43.107920 38.356030 719.648500 .000122
-91.451740 39.437030 792.632600 32.436040
-90.263630 39.689750 884.633900 56.053670
-89.061680 39.931370 1083.187000 107.693500
-87.846150 40.161480 1232.760000 150.838300
-86.617400 40.379670 1272.347000 160.393000
-85.375900 40.585540 1495.153000 183.131600
-84.122160 40.778730 1816.540000 194.019600
-82.856800 40.958850 1895.633000 148.898300
-81.580460 41.125550 1886.870000 99.894120
-80.293880 41.278500 2084.147000 150.945300
-78.997860 41.417370 2317.877000 235.799500
-77.693230 41.541890 2206.153000 249.444400
-76.380870 41.651780 1462.853000 124.768600
-75.061690 41.746810 759.688400 157.257400
-73.736660 41.826770 948.507000 119.071400
-72.406780 41.891490 1012.818000 78.388480
-71.073040 41.940830 1020.781000 68.948480
-69.736500 41.974670 1031.099000 84.626300
-68.398190 41.992950 983.641700 84.975190
-67.059160 41.995620 794.489100 42.552150
-65.720480 41.982670 711.141000 18.659980
-64.383220 41.954140 768.118500 20.899710
-63.048400 41.910080 795.641700 29.887490
-61.717090 41.850600 780.369700 27.640500
-60.390290 41.775820 746.427000 19.743760
-59.069010 41.685920 723.224500 14.612700
-57.754210 41.581080 713.262900 9.910540
-56.446830 41.461530 718.559200 4.741510
-55.147770 41.327530 722.360200 2.127281
-53.857880 41.179360 721.758100 1.287413
-52.577970 41.017320 720.324600 .432072
-51.308780 40.841740 719.805800 .144819
-50.051000 40.652980 719.686300 .038160
-48.805250 40.451390 719.657000 .010401
-47.572090 40.237360 719.649900 .002368
-46.352010 40.011290 719.648500 .000469
-45.145370 39.773560 719.648400 .000066
-43.952510 39.524580 719.648500 .000017
-42.773630 39.264750 719.648500 .000122
-91.793200 40.348750 799.846700 36.171660
-90.591710 40.609810 901.407200 67.826500
-89.375760 40.859430 1070.421000 133.409700
-88.145600 41.097210 980.373900 121.100400
-86.901570 41.322720 601.418100 154.960400
-85.644150 41.535550 688.594500 190.789100
-84.373860 41.735290 1260.020000 60.767840
-83.091290 41.921560 1603.227000 51.327770
-81.797160 42.093980 1870.970000 76.308970
-80.492230 42.252200 2384.596000 156.318000
-79.177290 42.395880 2802.966000 277.260400
-77.853240 42.524730 2753.629000 282.236700
-76.521000 42.638450 2011.236000 92.830250
-75.181550 42.736820 1138.928000 217.373900
-73.835900 42.819590 995.823700 92.394650
-72.485100 42.886590 949.143200 76.431280
-71.130210 42.937670 1070.960000 101.462400

-69.772340	42.972710	1069.168000	92.954860
-68.412590	42.991630	904.574500	48.919110
-67.052090	42.994400	513.060100	157.282300
-65.691960	42.980990	548.375800	114.225800
-64.333320	42.951450	800.080600	40.510650
-62.977290	42.905830	894.630200	56.894790
-61.624970	42.844260	840.101100	43.580090
-60.277440	42.766850	726.582600	18.171320
-58.935750	42.673790	680.585700	25.569290
-57.600940	42.565280	686.495400	24.785090
-56.273990	42.441570	713.344100	7.502865
-54.955840	42.302930	723.383900	3.672737
-53.647390	42.149640	722.079300	1.924156
-52.349470	41.982030	720.385600	.717122
-51.062890	41.800450	719.828700	.260920
-49.788320	41.605260	719.691100	.087826
-48.526440	41.396860	719.657800	.024647
-47.277780	41.175640	719.650000	.005680
-46.042850	40.942000	719.648600	.001033
-44.822010	40.696380	719.648400	.000156
-43.615560	40.439180	719.648500	.000039
-42.423690	40.170830	719.648500	.000122
-92.150770	41.257820	774.756000	29.991460
-90.935390	41.527410	848.324200	57.686930
-89.704910	41.785250	963.987700	115.143000
-88.459540	42.030900	827.733200	126.848100
-87.199620	42.263930	360.081000	96.156910
-85.925610	42.483890	392.845200	132.857900
-84.638040	42.690380	883.657800	93.963170
-83.337520	42.882970	1345.016000	102.810800
-82.024790	43.061280	2002.478000	94.381630
-80.700610	43.224930	2896.650000	267.364700
-79.365870	43.373580	3422.307000	388.821500
-78.021450	43.506890	3347.719000	393.939700
-76.668370	43.624570	2625.423000	252.727300
-75.307630	43.726360	1743.786000	107.179800
-73.940300	43.812040	1213.272000	107.169700
-72.567500	43.881390	778.121800	78.082260
-71.190370	43.934270	979.758400	77.893500
-69.810050	43.970550	1220.311000	128.062000
-68.427750	43.990140	1148.551000	107.572800
-67.044650	43.993000	620.956800	168.526200
-65.661930	43.979120	631.011700	61.560640
-64.280810	43.948540	862.476000	64.357810
-62.902460	43.901310	945.927900	67.951000
-61.528050	43.837570	845.828200	37.497030
-60.158730	43.757450	693.172800	46.968320
-58.795620	43.661140	690.089500	27.057140
-57.439790	43.548850	733.143700	11.273700
-56.092310	43.420850	750.048000	12.560360
-54.754150	43.277410	733.709600	6.805096
-53.426270	43.118850	723.435700	3.451552
-52.109530	42.945500	720.546000	1.540051
-50.804760	42.757750	719.846300	.651212
-49.512690	42.555960	719.691100	.236005
-48.233980	42.340550	719.658300	.070784
-46.969190	42.111940	719.650600	.017637
-45.718810	41.870550	719.648900	.003557
-44.483210	41.616830	719.648400	.000583
-43.262650	41.351200	719.648400	.000107
-42.057300	41.074100	719.648500	.000121
-92.525320	42.164070	738.452000	16.781630
-91.295520	42.442420	759.874100	31.869170
-90.049940	42.708690	782.817600	65.163410
-88.788760	42.962440	642.084600	64.111960
-87.512310	43.203190	400.902900	112.622700
-86.221000	43.430500	527.564600	91.815230

-84.915410 43.643920 1021.195000 79.558370
-83.596150 43.843030 1570.050000 75.316670
-82.263960 44.027400 2414.433000 194.765000
-80.919660 44.196660 3464.950000 462.341900
-79.564150 44.350410 3910.448000 532.924700
-78.198390 44.488330 3584.007000 446.502200
-76.823410 44.610100 2633.790000 209.846500
-75.440290 44.715440 1672.919000 64.098930
-74.050180 44.804110 950.094900 59.412750
-72.654240 44.875890 387.238200 193.265900
-71.253690 44.930630 805.486300 88.161550
-69.849760 44.968180 1450.321000 191.285500
-68.443720 44.988460 1631.031000 237.126300
-67.036810 44.991420 1024.869000 46.954190
-65.630330 44.977050 746.778900 63.479380
-64.225530 44.945390 787.224000 44.156770
-62.823690 44.896510 875.215500 33.737770
-61.426040 44.830540 802.430900 29.544420
-60.033810 44.747610 718.881900 42.042110
-58.648170 44.647940 764.443400 31.266710
-57.270290 44.531750 791.924700 31.649980
-55.901260 44.399320 769.633100 20.452920
-54.542130 44.250930 729.966200 9.567172
-53.193900 44.086940 720.322000 7.491931
-51.857480 43.907680 720.510900 3.811777
-50.533710 43.713560 720.208100 1.650266
-49.223370 43.504980 719.823400 .623312
-47.927120 43.282370 719.693200 .199617
-46.645540 43.046150 719.659100 .052842
-45.379100 42.796800 719.650700 .011615
-44.128160 42.534760 719.648800 .002059
-42.892960 42.260480 719.648500 .000278
-41.673610 41.974410 719.648500 .000121
-92.917770 43.067330 714.715900 6.897161
-91.673000 43.354680 704.359600 17.056910
-90.411740 43.629630 652.893400 30.667540
-89.134110 43.891690 500.800500 46.946110
-87.840450 44.140390 343.295500 57.091880
-86.531140 44.375260 312.046500 160.248700
-85.206750 44.595830 907.335900 64.130590
-83.867900 44.801650 1782.623000 165.492700
-82.515380 44.992280 2533.706000 280.409900
-81.150000 45.167310 3182.895000 396.266100
-79.772730 45.326340 3365.455000 413.517900
-78.384560 45.469020 3000.852000 319.302300
-76.986600 45.595010 2199.018000 110.409400
-75.579970 45.704020 1444.996000 105.016600
-74.165890 45.795780 859.543600 162.828500
-72.745610 45.870080 372.839400 265.670500
-71.320400 45.926740 762.873500 136.134600
-69.891600 45.965610 1517.806000 148.199400
-68.460530 45.986610 1780.593000 221.017300
-67.028560 45.989670 1204.897000 66.700770
-65.597030 45.974800 803.870800 98.245380
-64.167300 45.942020 792.154600 55.328650
-62.740720 45.891430 904.235000 51.976590
-61.318600 45.823140 789.042900 49.125940
-59.902260 45.737310 721.184800 60.023010
-58.492950 45.634170 770.502300 42.034210
-57.091900 45.513950 779.986900 40.847990
-55.700260 45.376930 763.235500 21.732620
-54.319140 45.223450 734.014200 24.413390
-52.949590 45.053840 726.012200 18.798260
-51.592580 44.868490 724.634600 9.343311
-50.248970 44.667810 722.304400 3.902310
-48.919560 44.452230 720.573300 1.483740
-47.605040 44.222190 719.883700 .481116

-46.305980 43.978160 719.699200 .133062
-45.022840 43.720610 719.658200 .030836
-43.755960 43.450020 719.650100 .005946
-42.505550 43.166850 719.648700 .000910
-41.271660 42.871580 719.648500 .000191
-93.329110 43.967430 709.649000 4.339647
-92.068780 44.264030 692.438400 10.034080
-90.791210 44.547890 628.551000 27.279620
-89.496510 44.818510 505.985700 63.184430
-88.184940 45.075390 418.806200 98.353630
-86.856880 45.318050 348.921000 174.461500
-85.512890 45.545990 847.574500 90.788600
-84.153590 45.758730 1702.566000 178.119600
-82.779790 45.955820 2110.572000 210.255300
-81.392350 46.136820 2198.068000 163.458200
-79.992260 46.301310 2142.698000 132.054900
-78.580580 46.448910 1969.923000 92.628460
-77.158460 46.579270 1687.409000 55.092660
-75.727110 46.692070 1612.571000 61.729010
-74.287820 46.787040 1589.750000 137.396900
-72.841890 46.863950 1385.705000 117.010400
-71.390720 46.922590 1447.417000 108.170200
-69.935700 46.962830 1832.930000 214.035000
-68.478260 46.984570 1872.956000 205.842400
-67.019850 46.987740 1369.857000 77.179730
-65.561920 46.972340 974.031500 43.551870
-64.105920 46.938410 931.475000 45.240760
-62.653260 46.886040 1006.926000 53.056890
-61.205390 46.815350 854.431900 89.028060
-59.763680 46.726520 776.839100 98.282850
-58.329470 46.619790 839.268700 44.213190
-56.904060 46.495390 865.611500 39.136810
-55.488670 46.353640 865.995100 41.277460
-54.084500 46.194880 838.739100 46.197320
-52.692630 46.019470 789.076900 35.693570
-51.314070 45.827840 748.627500 18.402480
-49.949730 45.620390 729.513400 7.850922
-48.600430 45.397590 722.583100 2.903480
-47.266860 45.159900 720.387300 .937323
-45.949600 44.907820 719.804700 .261215
-44.649100 44.641840 719.677100 .062397
-43.365670 44.362450 719.653100 .012589
-42.099450 44.070140 719.649200 .002082
-40.850440 43.765410 719.648500 .000407
-93.760410 44.864160 716.889800 3.720094
-92.483920 45.170280 708.373800 7.983105
-91.189420 45.463320 654.970500 20.771500
-89.876960 45.742750 511.487700 43.457590
-88.546760 46.008070 426.433300 107.663500
-87.199170 46.258760 448.750200 85.433750
-85.834720 46.494300 863.861400 61.640690
-84.454070 46.714200 1358.595000 100.922600
-83.058010 46.917960 1449.870000 58.443430
-81.647480 47.105130 1359.766000 146.305000
-80.223460 47.275260 1332.254000 146.082100
-78.787090 47.427960 1287.282000 148.478700
-77.339580 47.562840 1255.663000 228.701500
-75.882230 47.679570 1542.846000 82.995930
-74.416370 47.777870 1897.264000 134.508400
-72.943440 47.857470 1976.126000 187.772200
-71.464880 47.918190 1935.245000 190.859900
-69.982220 47.959850 1936.698000 186.720600
-68.496960 47.982350 1729.698000 141.377700
-67.010670 47.985630 1285.266000 59.118240
-65.524890 47.969690 992.508000 62.931710
-64.041170 47.934570 1077.451000 59.512390
-62.561040 47.880340 1235.017000 84.097380

-61.086020	47.807170	1179.732000	53.294080
-59.617590	47.715230	1091.303000	51.364720
-58.157170	47.604770	1101.538000	63.143160
-56.706150	47.476050	1109.182000	90.606110
-55.265830	47.329400	1113.836000	100.409000
-53.837470	47.165180	1065.852000	96.633230
-52.422200	46.983780	923.396300	63.901660
-51.021090	46.785640	799.214800	30.999140
-49.635090	46.571200	743.160600	12.500200
-48.265020	46.340950	725.745200	4.468580
-46.911610	46.095370	721.091200	1.411264
-45.575410	45.834990	719.949700	.389380
-44.256860	45.560320	719.703600	.093267
-42.956210	45.271870	719.657300	.019079
-41.673580	44.970160	719.649700	.003296
-40.408870	44.655690	719.648700	.000588
-94.212860	45.757310	730.321200	7.967534
-92.919560	46.073230	731.414100	10.619480
-91.607470	46.375730	702.671000	14.338250
-90.276550	46.664250	604.941800	32.077440
-88.926960	46.938270	528.037400	68.362300
-87.559010	47.197250	648.721100	54.500810
-86.173240	47.440650	863.997500	32.643110
-84.770290	47.667940	1075.465000	42.757210
-83.350960	47.878600	1190.882000	93.339210
-81.916200	48.072160	1349.782000	49.667230
-80.467090	48.248140	1461.390000	50.466800
-79.004780	48.406110	1407.171000	56.175500
-77.530590	48.545680	1431.502000	69.724240
-76.045850	48.666500	1664.214000	90.933460
-74.552020	48.768250	1892.066000	154.270100
-73.050600	48.850660	1928.053000	170.248000
-71.543170	48.913520	1879.133000	172.875700
-70.031330	48.956650	1777.524000	160.727200
-68.516710	48.979950	1477.079000	77.347570
-67.000980	48.983350	962.112500	141.308300
-65.485790	48.966840	639.143500	213.160400
-63.972820	48.930470	893.942600	56.728200
-62.463700	48.874330	1285.823000	114.530100
-60.960050	48.798580	1477.825000	148.481700
-59.463460	48.703410	1471.034000	133.909500
-57.975440	48.589080	1404.816000	130.405800
-56.497490	48.455880	1341.726000	140.911400
-55.030970	48.304140	1312.784000	152.339200
-53.577210	48.134270	1220.775000	144.843500
-52.137430	47.946660	1003.288000	83.175030
-50.712720	47.741790	827.540500	37.035580
-49.304080	47.520120	750.663300	14.409340
-47.912350	47.282170	727.386400	5.035396
-46.538250	47.028450	721.420200	1.562140
-45.182340	46.759510	720.008100	.426631
-43.845020	46.475880	719.712900	.100873
-42.526500	46.178100	719.658900	.020456
-41.226800	45.866700	719.649900	.003474
-39.945740	45.542200	719.648700	.000623
-94.687740	46.646660	752.506700	18.153280
-93.376940	46.972690	766.083100	22.506210
-92.046550	47.284930	773.897800	25.740380
-90.696440	47.582840	763.410500	21.455660
-89.326680	47.865840	781.570500	20.294700
-87.937550	48.133390	874.397100	35.234060
-86.529520	48.384910	974.262600	40.726900
-85.103260	48.619850	1115.866000	55.079310
-83.659580	48.837660	1385.747000	89.557200
-82.199460	49.037830	1663.818000	166.827300
-80.724000	49.219880	1732.159000	184.462200
-79.234450	49.383340	1597.209000	113.663900

-77.732160	49.527770	1573.595000	95.051610
-76.218590	49.652830	1669.935000	121.005500
-74.695260	49.758160	1766.966000	157.063600
-73.163800	49.843490	1746.514000	165.607800
-71.625870	49.908580	1707.810000	163.207300
-70.083210	49.953250	1661.396000	154.814100
-68.537570	49.977380	1499.991000	120.331000
-66.990740	49.980900	1093.501000	44.029530
-65.444490	49.963800	641.631800	166.027600
-63.900620	49.926140	642.906500	139.335900
-62.360880	49.868010	971.316300	53.485240
-60.827010	49.789570	1309.543000	113.296400
-59.300730	49.691040	1459.698000	149.190300
-57.783640	49.572690	1435.070000	151.827400
-56.277320	49.434830	1329.116000	140.225700
-54.783270	49.277820	1219.943000	128.573500
-53.302860	49.102080	1075.642000	97.995550
-51.837360	48.908040	892.698300	57.300840
-50.387950	48.696200	778.501200	27.019430
-48.955640	48.467040	736.722100	10.875260
-47.541310	48.221130	724.433300	3.897339
-46.145660	47.959000	720.822000	1.236124
-44.769230	47.681210	719.887100	.333117
-43.412380	47.388330	719.691100	.077605
-42.075270	47.080920	719.655100	.015095
-40.757810	46.759540	719.649400	.002447
-39.459720	46.424710	719.648600	.000420
-95.186440	47.531940	790.138100	32.939860
-93.857410	47.868410	821.419200	40.770510
-92.508000	48.190720	863.323500	50.472390
-91.137910	48.498310	916.465700	58.313670
-89.747150	48.790600	1004.188000	71.299700
-88.335950	49.067010	1105.394000	89.545070
-86.904710	49.326930	1165.096000	89.628800
-85.454100	49.569790	1249.157000	94.728960
-83.984930	49.795020	1425.037000	139.052700
-82.498220	50.002060	1549.534000	164.312400
-80.995120	50.190400	1536.851000	145.871200
-79.476920	50.359550	1470.117000	120.624100
-77.945060	50.509060	1529.807000	129.018600
-76.401090	50.638520	1625.291000	152.688500
-74.846650	50.747580	1697.621000	178.397300
-73.283450	50.835950	1641.157000	171.437900
-71.713320	50.903350	1572.073000	151.880400
-70.138080	50.949630	1538.157000	136.660800
-68.559640	50.974620	1496.996000	128.488900
-66.979900	50.978270	1336.452000	111.173200
-65.400800	50.960560	1035.213000	81.278400
-63.824260	50.921550	827.014800	52.379050
-62.252170	50.861340	864.493000	71.910440
-60.686400	50.780110	1081.461000	91.179150
-59.128770	50.678080	1252.281000	126.261600
-57.581030	50.555550	1280.588000	133.163400
-56.044860	50.412850	1196.691000	126.044700
-54.521820	50.250360	1059.098000	97.895910
-53.013420	50.068520	917.942000	65.793170
-51.520960	49.867810	800.820400	37.210530
-50.045680	49.648740	745.170400	17.195190
-48.588630	49.411830	727.128900	6.943325
-47.150700	49.157670	721.764800	2.483064
-45.732590	48.886830	720.159700	.766859
-44.334810	48.599900	719.749500	.203963
-42.957660	48.297460	719.665700	.044988
-41.601190	47.980120	719.651200	.008195
-40.265250	47.648430	719.648800	.001179
-38.949390	47.302930	719.648500	.000226
-95.710510	48.412890	860.234400	52.802730

-94.362470	48.760130	930.468600	65.031940
-92.993230	49.092850	1013.401000	81.104270
-91.602360	49.410460	1086.043000	93.261730
-90.189750	49.712350	1150.190000	107.490000
-88.755540	49.997940	1180.329000	110.944900
-87.300090	50.266570	1165.618000	102.348300
-85.824040	50.517650	1152.349000	97.474570
-84.328210	50.750570	1192.561000	109.192700
-82.813610	50.964740	1255.283000	119.728100
-81.281460	51.159630	1314.545000	122.016800
-79.733130	51.334700	1361.012000	124.227100
-78.170130	51.489480	1435.601000	141.680500
-76.594090	51.623530	1505.422000	154.613400
-75.006800	51.736490	1560.811000	169.673200
-73.410070	51.828010	1499.322000	160.797600
-71.805870	51.897850	1438.383000	140.248800
-70.196170	51.945790	1405.562000	113.767500
-68.582990	51.971680	1372.559000	96.542630
-66.968440	51.975460	1313.304000	91.094490
-65.354560	51.957120	1198.793000	94.626690
-63.743440	51.916690	1048.226000	84.065890
-62.137130	51.854320	982.147600	78.727330
-60.537630	51.770170	1052.244000	98.094060
-58.946900	51.664510	1134.120000	114.790900
-57.366820	51.537620	1137.913000	117.373700
-55.799180	51.389870	1053.706000	98.292470
-54.245650	51.221680	937.720700	71.318030
-52.707820	51.033510	840.921300	45.732190
-51.187080	50.825860	775.310300	24.943850
-49.684720	50.599280	740.056200	11.535280
-48.201800	50.354330	725.625700	4.476768
-46.739240	50.091630	721.068800	1.521312
-45.297720	49.811770	719.935900	.446319
-43.877700	49.515370	719.700600	.110205
-42.479410	49.203060	719.657300	.022481
-41.102810	48.875440	719.649700	.003626
-39.747590	48.533100	719.648600	.000453
-38.413150	48.176570	719.648500	.000135
-96.261670	49.289190	954.457800	70.454000
-94.893750	49.647600	1065.964000	94.286910
-93.503820	49.991080	1148.055000	112.429500
-92.091320	50.319060	1156.031000	111.671000
-90.655950	50.630910	1113.859000	99.114530
-89.197760	50.925990	1050.233000	86.135910
-87.717060	51.203660	994.467100	76.380030
-86.214420	51.463270	967.939800	73.004230
-84.690660	51.704190	1006.391000	78.017720
-83.146820	51.925780	1123.162000	93.352200
-81.584160	52.127480	1260.934000	123.523200
-80.004110	52.308710	1305.153000	131.617400
-78.408280	52.468990	1284.837000	124.568800
-76.798410	52.607840	1324.625000	131.343900
-75.176390	52.724850	1400.039000	149.730800
-73.544200	52.819690	1360.229000	144.597600
-71.903920	52.892050	1324.714000	128.112600
-70.257710	52.941730	1299.582000	99.153620
-68.607750	52.968570	1245.115000	59.987450
-66.956280	52.972490	1198.258000	52.420250
-65.305560	52.953480	1193.414000	85.462060
-63.657820	52.911580	1153.928000	102.260000
-62.015270	52.846950	1099.526000	104.591600
-60.380080	52.759750	1087.700000	108.041800
-58.754360	52.650280	1074.986000	107.561500
-57.140120	52.518850	1011.674000	90.205490
-55.539290	52.365840	920.309100	68.016560
-53.953660	52.191710	841.965100	47.241200
-52.384890	51.996940	786.378900	28.956390

-50.834480	51.782070	751.021000	15.646270
-49.303730	51.547700	731.215100	7.027594
-47.793770	51.294400	722.926900	2.660237
-46.305490	51.022820	720.432700	.862061
-44.839570	50.733620	719.817200	.240742
-43.396390	50.427420	719.681400	.056602
-41.976090	50.104880	719.653900	.010865
-40.578500	49.766630	719.649000	.001649
-39.203150	49.413260	719.648500	.000182
-37.849210	49.045330	719.648500	.000124
-96.841810	50.160530	960.395300	71.404270
-95.453030	50.530490	1048.992000	94.792400
-94.041510	50.885130	1067.650000	93.524260
-92.606450	51.223850	1018.322000	83.546710
-91.147380	51.546020	947.726600	70.880570
-89.664200	51.850960	884.808400	58.462650
-88.157130	52.138020	848.147100	50.179580
-86.626690	52.406500	847.527900	47.410350
-85.073680	52.655720	904.979900	54.565080
-83.499180	52.885050	1017.971000	77.780570
-81.904440	53.093850	1105.767000	95.487300
-80.290990	53.281520	1110.491000	93.714490
-78.660530	53.447530	1067.086000	86.755260
-77.014920	53.591380	1128.484000	96.368200
-75.356160	53.712650	1243.061000	125.087500
-73.686420	53.810940	1225.662000	125.579100
-72.007930	53.885960	1198.143000	110.540700
-70.322990	53.937460	1164.263000	81.627800
-68.634010	53.965280	1095.453000	42.429200
-66.943400	53.969350	1055.114000	43.357320
-65.253590	53.949630	1118.530000	63.332970
-63.567000	53.906200	1168.494000	110.559400
-61.886040	53.839190	1137.125000	115.178800
-60.213040	53.748820	1066.159000	103.794700
-58.550290	53.635360	977.344100	81.163140
-56.899960	53.499180	884.882500	59.085480
-55.264110	53.340690	816.024700	42.159080
-53.644650	53.160350	781.608200	30.331870
-52.043340	52.958710	754.466600	18.455930
-50.461760	52.736320	734.825100	9.206405
-48.901280	52.493820	724.926300	3.941669
-47.363030	52.231850	721.220300	1.456661
-45.847900	51.951060	720.061000	.470825
-44.356530	51.652150	719.743300	.130676
-42.889220	51.335790	719.667100	.030732
-41.445980	51.002650	719.651300	.005960
-40.026490	50.653380	719.648700	.000945
-38.630060	50.288590	719.648500	.000117
-37.255620	49.908830	719.648500	.000121
-97.453060	51.026530	856.801100	46.693640
-96.042340	51.408490	894.187000	55.043160
-94.608220	51.774700	895.205100	57.998830
-93.149600	52.124570	870.028400	56.979240
-91.665800	52.457450	839.153300	51.896840
-90.156560	52.772640	809.896000	44.622960
-88.621980	53.069450	789.167600	37.351090
-87.062480	53.347160	780.664100	32.068680
-85.478850	53.605050	789.621700	28.875430
-83.872130	53.842430	799.744300	36.412020
-82.243660	54.058630	786.277900	56.423100
-80.595020	54.253030	808.242100	46.936900
-78.928000	54.425040	861.365200	49.287530
-77.244600	54.574130	994.479700	69.188910
-75.546970	54.699840	1132.110000	107.857700
-73.837420	54.801750	1127.363000	112.129300
-72.118360	54.879540	1069.187000	90.309410
-70.392330	54.932960	961.126400	53.038750

-68.661900	54.961820	864.936100	44.623710
-66.929700	54.966030	889.874800	58.947980
-65.198370	54.945580	991.619100	50.562210
-63.470540	54.900540	1070.008000	92.323710
-61.748820	54.831050	1055.387000	99.887230
-60.035740	54.737340	963.528100	77.668240
-58.333780	54.619720	869.352200	53.247510
-56.645260	54.478570	801.982500	34.932490
-54.972420	54.314330	765.642600	24.781960
-53.317280	54.127510	756.239600	20.931030
-51.681740	53.918690	743.985500	14.144460
-50.067420	53.688470	730.073500	6.748274
-48.475770	53.437500	723.086500	2.692769
-46.907930	53.166490	720.674900	.968237
-45.364770	52.876130	719.921300	.312531
-43.846840	52.567130	719.710900	.089099
-42.354370	52.240220	719.660600	.021928
-40.887210	51.896080	719.650500	.004608
-39.444820	51.535370	719.648700	.000785
-38.026250	51.158730	719.648500	.000098
-36.630120	50.766700	719.648500	.000122
-98.097820	51.886770	772.486600	24.426390
-96.663910	52.281200	781.756300	25.775950
-95.206040	52.659450	782.365100	28.376000
-93.722790	53.020900	781.013100	30.884420
-92.213200	53.364910	782.055200	34.323430
-90.676770	53.690770	783.227100	37.436370
-89.113440	53.997730	779.920800	36.815070
-87.523560	54.285050	770.772300	31.571330
-85.907830	54.551970	750.117200	22.102710
-84.267300	54.797760	694.284900	32.702770
-82.603340	55.021710	592.251300	97.694850
-80.917590	55.223140	557.215700	127.378300
-79.211960	55.401440	676.456800	82.471060
-77.488560	55.556020	906.366500	50.462970
-75.749730	55.686380	1047.348000	90.944730
-73.997920	55.792100	1033.008000	97.324440
-72.235800	55.872810	921.638100	63.139170
-70.466090	55.928230	735.270800	48.734010
-68.691570	55.958180	685.574000	88.891800
-66.915130	55.962550	852.407300	48.367060
-65.139640	55.941330	957.042800	65.165700
-63.367970	55.894590	961.300200	71.494440
-61.602940	55.822490	922.064200	68.160280
-59.847310	55.725280	851.059400	50.248300
-58.103760	55.603290	795.089000	32.746660
-56.374820	55.456930	761.330900	19.953190
-54.662870	55.286670	746.058300	13.858250
-52.970100	55.093080	746.284900	14.449480
-51.298500	54.876750	743.957800	13.341340
-49.649800	54.638340	731.428600	6.855013
-48.025460	54.378550	723.484300	2.680519
-46.426660	54.098120	720.767800	.948289
-44.854210	53.797780	719.951400	.311646
-43.308580	53.478290	719.719300	.092931
-41.789850	53.140400	719.662700	.024540
-40.297680	52.784830	719.650900	.005547
-38.831280	52.412250	719.648700	.001048
-37.389370	52.023280	719.648500	.000133
-35.970190	51.618480	719.648500	.000122
-98.778840	52.740810	737.020400	10.530030
-97.320270	53.148240	737.733500	10.768580
-95.837370	53.539010	738.020300	11.377720
-94.328290	53.912530	739.101000	12.698080
-92.791720	54.268120	743.229200	15.617030
-91.226900	54.605080	750.869700	20.479040
-89.633560	54.922620	759.903000	25.923610

-88.011890	55.219970	767.089900	30.459140
-86.362530	55.496330	760.897300	28.449450
-84.686490	55.750910	716.903400	26.129450
-82.985150	55.982970	603.558700	73.476260
-81.260250	56.191770	466.148600	183.194800
-79.513790	56.376650	551.190600	153.226700
-77.748020	56.536990	854.984400	49.972980
-75.965460	56.672260	973.650800	77.816410
-74.168790	56.781970	913.280000	73.805680
-72.360860	56.865740	784.881800	41.783020
-70.544630	56.923280	654.949000	68.172650
-68.723180	56.954370	718.982100	61.160830
-66.899620	56.958910	943.216500	83.430430
-65.077100	56.936880	968.010600	75.820260
-63.258730	56.888360	878.536300	54.173250
-61.447630	56.813510	803.271000	35.589680
-59.646790	56.712620	767.173600	22.959330
-57.859090	56.586040	751.973100	18.286120
-56.087300	56.434210	737.948700	10.118270
-54.333970	56.257640	732.810200	6.929390
-52.601470	56.056940	738.628400	9.943069
-50.891880	55.832740	744.918700	13.464120
-49.207030	55.585770	735.132100	8.276220
-47.548420	55.316760	725.168000	3.412292
-45.917200	55.026500	721.270100	1.233966
-44.314130	54.715760	720.106100	.412138
-42.739580	54.385350	719.761900	.127391
-41.193420	54.036030	719.672200	.035268
-39.675070	53.668560	719.652700	.008497
-38.183400	53.283620	719.649200	.001725
-36.716770	52.881820	719.648500	.000269
-35.272900	52.463700	719.648500	.000130
-99.499240	53.588130	724.779600	4.087889
-98.014280	54.009130	724.450000	3.904410
-96.504860	54.412960	724.475700	4.060221
-94.968610	54.799050	724.778300	4.534320
-93.403790	55.166730	726.211000	5.659919
-91.809290	55.515250	729.472400	7.739404
-90.184560	55.843850	735.316700	11.274680
-88.529620	56.151670	743.740700	16.238010
-86.844990	56.437890	750.566200	20.671920
-85.131650	56.701690	744.204800	24.512990
-83.390960	56.942230	684.192000	44.333750
-81.624720	57.158780	557.169100	103.109700
-79.835050	57.350580	572.088000	132.455600
-78.024370	57.516980	845.702500	53.153580
-76.195360	57.657400	909.825400	72.756050
-74.350940	57.771310	812.869400	59.089830
-72.494220	57.858320	747.180500	49.043750
-70.628410	57.918090	738.870500	46.025370
-68.756890	57.950390	849.389500	60.366440
-66.883060	57.955100	978.855800	85.964900
-65.010370	57.932220	931.088000	67.642500
-63.142240	57.881810	822.384500	37.009490
-61.282040	57.804080	747.412700	16.288200
-59.433060	57.699310	722.391500	11.345390
-57.598450	57.567890	719.384400	8.794940
-55.781200	57.410300	713.231800	6.148330
-53.984060	57.227100	717.733900	1.866213
-52.209560	57.018940	729.481600	5.211600
-50.459930	56.786500	743.110400	12.210710
-48.737060	56.530560	740.504200	10.671900
-47.042490	56.251900	728.721400	4.989082
-45.377330	55.951360	722.384200	1.794125
-43.742270	55.629770	720.390600	.587874
-42.137470	55.287960	719.834400	.181494
-40.562610	54.926740	719.688900	.051284

-39.016750 54.546860 719.656300 .012853
-37.498410 54.149020 719.649700 .002759
-36.005400 53.733830 719.648600 .000480
-34.534880 53.301770 719.648500 .000153
-100.262700 54.428120 720.948700 1.390928
-98.749250 54.863350 720.779700 1.259834
-97.211560 55.280850 720.773700 1.272247
-95.646590 55.680060 720.882800 1.428749
-94.052090 56.060360 721.290100 1.823299
-92.426510 56.420970 722.206800 2.593100
-90.768940 56.761100 724.034100 3.899216
-89.079190 57.079890 727.140100 5.853196
-87.357570 57.376450 731.401800 8.551019
-85.605000 57.649890 734.713000 13.066250
-83.822840 57.899360 716.941600 24.883320
-82.012920 58.124030 647.710400 48.160170
-80.177480 58.323120 659.071000 55.416170
-78.319110 58.495910 854.358900 60.195680
-76.440710 58.641750 874.132200 73.297290
-74.545430 58.760110 791.030900 61.196260
-72.636660 58.850530 795.856600 54.714820
-70.717930 58.912650 845.161900 61.842850
-68.792920 58.946230 915.162700 71.137800
-66.865380 58.951130 944.228200 75.598920
-64.939080 58.927340 864.250400 49.789330
-63.017780 58.874950 776.333900 22.422370
-61.105210 58.794160 724.376500 10.074070
-59.204930 58.685300 712.590700 6.012060
-57.320400 58.548780 709.396100 9.154248
-55.454840 58.385130 697.397000 14.120810
-53.611270 58.194950 700.047700 10.530260
-51.792370 57.978940 714.540300 3.195967
-50.000500 57.737840 734.432000 7.854104
-48.237630 57.472490 743.320600 12.486570
-46.505310 57.183730 732.156100 6.477303
-44.804610 56.872430 723.529500 2.328456
-43.136050 56.539500 720.673800 .752387
-41.499610 56.185780 719.909100 .230808
-39.894630 55.812120 719.707500 .066598
-38.319800 55.419290 719.659900 .017242
-36.773090 55.007980 719.650300 .003892
-35.251730 54.578750 719.648800 .000725
-33.752120 54.132060 719.648500 .000206
-101.073700 55.260130 719.932700 .411837
-99.529010 55.710320 719.879700 .350396
-97.960900 56.142140 719.878700 .348910
-96.365430 56.555100 719.917500 .398608
-94.739640 56.948590 720.040000 .531869
-93.081440 57.321860 720.309300 .799178
-91.389490 57.674070 720.838100 1.266212
-89.663250 58.004340 721.746700 2.015266
-87.902820 58.311730 723.234600 3.462809
-86.109000 58.595320 724.716600 7.481659
-84.283100 58.854170 715.150600 16.384560
-82.427000 59.087400 679.345700 28.821520
-80.543050 59.294160 707.330900 31.742880
-78.634010 59.473690 844.878600 59.195920
-76.703000 59.625280 840.946100 68.615910
-74.753460 59.748340 801.077200 60.182630
-72.789070 59.842370 832.873600 64.330180
-70.813740 59.906970 875.197100 66.067430
-68.831480 59.941910 915.079600 70.041010
-66.846440 59.947010 899.843400 62.692920
-64.862770 59.922250 817.112700 36.583120
-62.884610 59.867760 751.234200 15.173500
-60.916040 59.783740 718.767900 6.507794
-58.961010 59.670550 714.963300 4.536424

-57.023270	59.528640	715.012500	6.171132
-55.106350	59.358580	700.070600	11.460110
-53.213500	59.161040	691.070500	16.291130
-51.347600	58.936760	697.111600	12.608700
-49.511150	58.686560	716.786900	3.360594
-47.706190	58.411320	736.237200	9.826340
-45.934240	58.111950	731.749700	6.226158
-44.196280	57.789400	723.818600	2.423775
-42.492650	57.444580	720.775900	.805754
-40.823030	57.078400	719.951000	.254062
-39.186360	56.691730	719.721300	.074743
-37.580830	56.285340	719.663500	.020113
-36.003750	55.859910	719.651000	.004732
-34.451600	55.415950	719.648900	.000935
-32.919830	54.953830	719.648500	.000257
-101.937200	56.083360	719.703300	.103971
-100.358100	56.549350	719.690400	.083415
-98.756940	56.996240	719.690500	.082508
-97.128790	57.423640	719.701400	.099463
-95.469860	57.830970	719.735400	.144081
-93.777320	58.217490	719.817600	.236252
-92.049300	58.582370	720.003100	.420530
-90.284800	58.924680	720.386800	.866004
-88.483640	59.243460	721.186700	2.292261
-86.646400	59.537720	721.821500	6.901164
-84.774360	59.806450	712.780900	15.706510
-82.869400	60.048700	687.025600	23.010300
-80.933970	60.263580	724.941200	21.015220
-78.971000	60.450230	811.658300	47.466300
-76.983890	60.607900	778.616000	47.722530
-74.976370	60.735940	758.120500	48.265930
-72.952470	60.833800	790.231300	47.133010
-70.916480	60.901050	840.714800	55.444790
-68.872850	60.937420	892.022400	62.119870
-66.826130	60.942730	869.124600	52.411060
-64.780930	60.916960	791.883400	29.691790
-62.741830	60.860230	742.469800	12.508530
-60.713310	60.772790	721.481000	3.475605
-58.699720	60.654990	717.982700	1.552427
-56.705200	60.507380	719.668600	2.366255
-54.733590	60.330550	711.801600	4.783234
-52.788400	60.125220	701.349000	9.839123
-50.872730	59.892220	695.512900	13.154130
-48.989200	59.632420	702.486600	10.458090
-47.139900	59.346780	721.550800	3.561986
-45.326320	59.036270	727.940600	4.887475
-43.549270	58.701890	723.983200	2.395876
-41.808860	58.344600	720.945900	.828873
-40.104370	57.965370	719.979100	.256145
-38.434220	57.565060	719.729000	.076014
-36.795950	57.144450	719.665500	.020463
-35.186060	56.704150	719.651600	.004978
-33.600030	56.244660	719.648900	.001018
-32.032190	55.766200	719.648500	.000291
-102.859900	56.896880	719.658000	.022201
-101.242000	57.379640	719.655300	.016546
-99.604390	57.842470	719.655300	.016751
-97.940930	58.285070	719.658100	.022543
-96.246660	58.706940	719.668000	.038443
-94.517820	59.107400	719.696800	.078604
-92.751850	59.485580	719.778300	.199612
-90.947170	59.840560	719.985500	.618017
-89.103220	60.171320	720.494600	2.077387
-87.220290	60.476800	720.506100	6.791744
-85.299550	60.755960	711.141000	15.661530
-83.342830	61.007760	690.182000	21.389720
-81.352710	61.231220	729.633700	11.092970

-79.332320	61.425420	778.575100	29.159280
-77.285290	61.589540	720.312200	22.654500
-75.215710	61.722870	674.825400	37.565260
-73.128000	61.824790	686.966200	40.109870
-71.026890	61.894870	760.354100	36.721540
-68.917300	61.932760	853.714300	51.558480
-66.804310	61.938290	846.358300	46.330150
-64.692990	61.911450	780.206400	26.061690
-62.588420	61.852340	740.564000	13.021340
-60.495590	61.761240	726.078400	7.233949
-58.419300	61.638580	722.160800	3.743389
-56.364090	61.484900	722.301300	2.624734
-54.334160	61.300890	719.715200	2.100994
-52.333320	61.087320	715.179600	3.366257
-50.364880	60.845090	708.911900	5.958735
-48.431600	60.575160	706.183200	7.523184
-46.535590	60.278550	713.815900	4.612179
-44.678250	59.956310	723.249200	3.056163
-42.860180	59.609490	723.089700	2.055719
-41.081100	59.239120	720.881200	.728655
-39.339830	58.846180	719.954800	.221653
-37.634110	58.431540	719.719400	.063129
-35.960610	57.995940	719.663800	.017433
-34.314830	57.539980	719.651300	.004243
-32.690990	57.064010	719.648900	.000904
-31.082000	56.568130	719.648500	.000272
-103.849600	57.699570	719.650000	.003879
-102.187300	58.200280	719.649500	.002679
-100.509000	58.680030	719.649400	.002940
-98.806840	59.138710	719.650100	.005102
-97.074520	59.575940	719.653500	.013010
-95.307120	59.991050	719.666300	.042370
-93.501110	60.383240	719.705400	.154369
-91.654170	60.751550	719.799100	.561220
-89.765210	61.094930	719.937100	2.009121
-87.834170	61.412270	719.055500	6.636924
-85.861950	61.702450	711.124200	14.499000
-83.850420	61.964360	700.039900	17.019230
-81.802150	62.196920	734.873100	8.049372
-79.720490	62.399150	750.546300	19.540220
-77.609360	62.570130	684.700600	35.796480
-75.473210	62.709080	624.022100	69.060930
-73.316960	62.815350	609.649800	87.965630
-71.145790	62.888430	672.653600	71.506420
-68.965200	62.927950	788.332200	38.516880
-66.780790	62.933720	809.369400	36.763470
-64.598260	62.905720	766.510900	21.864800
-62.423240	62.844070	738.575300	12.671980
-60.261290	62.749080	728.755700	8.058360
-58.117710	62.621230	725.974200	5.744470
-55.997520	62.461110	725.487200	4.866853
-53.905320	62.269450	724.595200	4.307723
-51.845240	62.047150	723.648900	4.178560
-49.820820	61.795150	721.716600	4.176960
-47.834930	61.514500	718.729700	3.512964
-45.889670	61.206310	717.881500	1.902813
-43.986290	60.871700	721.146200	1.176121
-42.125070	60.511780	720.906900	.751790
-40.305270	60.127630	720.084800	.319168
-38.525020	59.720250	719.783600	.109169
-36.781200	59.290500	719.687000	.036566
-35.069400	58.839090	719.657400	.010534
-33.383770	58.366500	719.650100	.002740
-31.716990	57.872960	719.648700	.000581
-30.060130	57.358350	719.648500	.000198
-104.916500	58.490070	719.648700	.000552
-103.202300	59.010120	719.648500	.000372

-101.477600 59.507980 719.648600 .000520
-99.732440 59.983750 719.648700 .001851
-97.958720 60.437220 719.650100 .007919
-96.150010 60.867820 719.655500 .033178
-94.301540 61.274790 719.671700 .131229
-92.410060 61.657170 719.686400 .499049
-90.473700 62.013880 719.497000 1.839323
-88.491920 62.343760 718.399500 5.761797
-86.465330 62.645610 716.083900 10.904100
-84.395630 62.918240 719.838300 9.460026
-82.285480 63.160470 745.902400 15.392230
-80.138370 63.371240 735.889800 13.843970
-77.958560 63.549540 684.370100 26.626780
-75.750930 63.694510 639.885400 50.616970
-73.520860 63.805420 619.207800 68.870340
-71.274150 63.881700 646.039400 64.330440
-69.016910 63.922960 724.568600 31.914890
-66.755400 63.928990 755.227100 19.048990
-64.495980 63.899750 742.859100 13.384300
-62.244980 63.835380 731.765900 8.692947
-60.008560 63.736250 726.955400 6.013258
-57.792640 63.602840 725.199000 4.689319
-55.602750 63.435840 724.426400 3.964346
-53.443960 63.236060 723.423200 3.234154
-51.320750 63.004450 722.669600 2.832885
-49.236890 62.742080 722.125200 2.677130
-47.195330 62.450080 721.005600 2.189938
-45.198120 62.129630 719.709200 1.313795
-43.246250 61.781950 719.966400 .473673
-41.339580 61.408210 719.855200 .223052
-39.476710 61.009530 719.761900 .095349
-37.654880 60.586900 719.697200 .043346
-35.869840 60.141170 719.663000 .015675
-34.115770 59.672970 719.652000 .005054
-32.385130 59.182660 719.649000 .001306
-30.668580 58.670220 719.648500 .000315
-28.954870 58.135290 719.648500 .000143
-106.073800 59.266680 719.648500 .000143
-104.297500 59.807790 719.648500 .000127
-102.518800 60.325180 719.648500 .000242
-100.725000 60.819250 719.648500 .001206
-98.905530 61.289990 719.648700 .005797
-97.052100 61.737010 719.650700 .024930
-95.158340 62.159620 719.654500 .100325
-93.219700 62.556880 719.627100 .382957
-91.233350 62.927700 719.380600 1.391265
-89.198040 63.270870 719.035700 3.921071
-87.113920 63.585110 720.640800 6.878955
-84.982480 63.869130 727.043500 5.454497
-82.806390 64.121680 730.986000 9.497496
-80.589290 64.341560 707.524800 15.855220
-78.335770 64.527670 677.656000 26.488540
-76.051160 64.679080 661.946500 34.334830
-73.741460 64.794950 659.884500 36.307700
-71.413090 64.874680 674.808100 29.885080
-69.072890 64.917820 710.550600 15.257370
-66.727910 64.924110 727.914100 7.934326
-64.385280 64.893550 727.226900 5.381056
-62.052090 64.826280 724.401300 3.821201
-59.735260 64.722690 722.689100 2.695035
-57.441380 64.583340 721.931400 2.086596
-55.176610 64.408980 721.560500 1.722813
-52.946510 64.200520 721.043300 1.302601
-50.755950 63.959000 720.619400 1.007313
-48.608900 63.685590 720.414400 .898430
-46.508400 63.381530 720.105400 .717981
-44.456330 63.048090 719.744700 .422322

-42.453320	62.686580	719.668300	.163032
-40.498620	62.298210	719.681600	.048992
-38.589950	61.884150	719.678600	.028048
-36.723370	61.445380	719.661300	.013641
-34.893150	60.982680	719.652600	.005841
-33.091610	60.496560	719.649400	.001816
-31.309040	59.987160	719.648500	.000508
-29.533510	59.454220	719.648500	.000156
-27.750760	58.896990	719.648500	.000125
-107.339200	60.027230	719.648500	.000122
-105.487000	60.591610	719.648500	.000120
-103.643800	61.130270	719.648500	.000185
-101.793500	61.644070	719.648400	.000853
-99.922550	62.133320	719.648400	.003986
-98.020040	62.597820	719.648400	.016665
-96.077510	63.037030	719.645900	.062480
-94.088710	63.450070	719.623900	.216657
-92.049460	63.835860	719.457600	.697712
-89.957580	64.193130	718.892000	1.774415
-87.812570	64.520550	718.325400	1.509247
-85.615560	64.816710	717.737700	4.242661
-83.369120	65.080260	710.844500	11.526750
-81.077050	65.309890	698.688900	14.649980
-78.744270	65.504390	692.317900	16.083550
-76.376630	65.662700	692.282000	16.005780
-73.980770	65.783920	695.889500	14.387700
-71.563890	65.867360	703.330400	10.202240
-69.133670	65.912500	714.503100	5.087386
-66.698060	65.919100	720.660800	2.282013
-64.265090	65.887100	721.400400	1.658198
-61.842790	65.816700	720.928600	1.220274
-59.438900	65.708320	720.487100	.862658
-57.060850	65.562580	720.262500	.643500
-54.715470	65.380330	720.153600	.513430
-52.408880	65.162570	720.010900	.375795
-50.146370	64.910470	719.888100	.270606
-47.932120	64.625290	719.822800	.222053
-45.769150	64.308400	719.740200	.166269
-43.659080	63.961160	719.671900	.100592
-41.602020	63.584970	719.648600	.037762
-39.596350	63.181100	719.655700	.013734
-37.638600	62.750720	719.653700	.006673
-35.723270	62.294770	719.650900	.004112
-33.842640	61.813940	719.649200	.001643
-31.986660	61.308540	719.648700	.000558
-30.142740	60.778430	719.648400	.000159
-28.295590	60.222970	719.648500	.000122
-26.427080	59.640870	719.648500	.000122
-108.736700	60.768890	719.648500	.000122
-106.789100	61.359350	719.648500	.000122
-104.866900	61.921510	719.648500	.000184
-102.949500	62.456830	719.648400	.000800
-101.019200	62.966030	719.648100	.003457
-99.061880	63.449270	719.646800	.013167
-97.066180	63.906190	719.640000	.045199
-95.023590	64.336020	719.603800	.133754
-92.928180	64.737730	719.454900	.345261
-90.776390	65.110020	718.929200	.708442
-88.566850	65.451470	717.818100	.910295
-86.300130	65.760610	715.239400	4.531422
-83.978550	66.035940	709.440200	7.652571
-81.606060	66.276030	705.293900	8.533956
-79.187900	66.479550	705.745000	8.126396
-76.730480	66.645290	707.502300	7.584404
-74.241170	66.772270	709.805000	6.481284
-71.728080	66.859700	713.124600	4.555823
-69.199870	66.907030	716.959800	2.303351

-66.665550	66.913940	719.341500	.900104
-64.134220	66.880400	720.006200	.556422
-61.614990	66.806620	720.023100	.445263
-59.116610	66.693070	719.915200	.322055
-56.647430	66.540450	719.844400	.235591
-54.215080	66.349720	719.804400	.181056
-51.826330	66.121990	719.763200	.132017
-49.486860	65.858530	719.727100	.093224
-47.201050	65.560780	719.702300	.070363
-44.971820	65.230190	719.675400	.049249
-42.800360	64.868270	719.655000	.029047
-40.685980	64.476460	719.648800	.013424
-38.625890	64.056100	719.649000	.004347
-36.614990	63.608330	719.649200	.002316
-34.645670	63.134020	719.648900	.001167
-32.707610	62.633670	719.648700	.000559
-30.787590	62.107330	719.648500	.000226
-28.869240	61.554470	719.648500	.000125
-26.932890	60.973880	719.648500	.000122
-24.955330	60.363580	719.648500	.000122

Q.31a

-89.453400	32.991660	758.109700	.003216
-88.345930	33.191160	758.111100	.006357
-87.228100	33.381660	758.117200	.019796
-86.100230	33.562890	758.132000	.050111
-84.962690	33.734530	758.160900	.103447
-83.815900	33.896310	758.201700	.173212
-82.660300	34.047950	758.240400	.232973
-81.496410	34.189190	758.253400	.256138
-80.324730	34.319790	758.240400	.244657
-79.145840	34.439500	758.226900	.215649
-77.960310	34.548100	758.186200	.126626
-76.768760	34.645400	758.044400	.130496
-75.571840	34.731200	757.790200	.489014
-74.370180	34.805370	757.643400	.672711
-73.164480	34.867740	757.800200	.495903
-71.955430	34.918200	757.980800	.232544
-70.743730	34.956650	758.075600	.070504
-69.530080	34.983020	758.107900	.008183
-68.315220	34.997260	758.116700	.014912
-67.099880	34.999340	758.116100	.014395
-65.884770	34.989250	758.112500	.008144
-64.670640	34.967020	758.110000	.003169
-63.458190	34.932690	758.109200	.001035

-62.248150	34.886320	758.109100	.000518
-61.041230	34.828000	758.109000	.000425
-59.838120	34.757850	758.109000	.000419
-58.639500	34.676010	758.109000	.000417
-57.446040	34.582610	758.108800	.000336
-56.258390	34.477850	758.108600	.000310
-55.077160	34.361910	758.108800	.000353
-53.902950	34.235010	758.108800	.000299
-52.736340	34.097380	758.109000	.000430
-51.577860	33.949260	758.109000	.000427
-50.428010	33.790910	758.109000	.000427
-49.287250	33.622600	758.109000	.000427
-48.156040	33.444620	758.109000	.000427
-47.034740	33.257250	758.109000	.000427
-45.923690	33.060790	758.109000	.000427
-44.823220	32.855560	758.109000	.000427
-89.701210	33.918400	758.116700	.019686
-88.583520	34.125020	758.126700	.038026
-87.455040	34.322360	758.165400	.104889
-86.316120	34.510110	758.253400	.246024
-85.167100	34.687960	758.409400	.479637
-84.008420	34.855620	758.619500	.769525
-82.840550	35.012790	758.808300	1.016000
-81.663990	35.159200	758.874000	1.114881
-80.479300	35.294580	758.832800	1.086456
-79.287040	35.418700	758.758400	.972057
-78.087840	35.531310	758.555200	.624845
-76.882320	35.632210	757.919400	.415221
-75.671150	35.721200	756.699200	1.796339
-74.455030	35.798110	756.092000	2.484508
-73.234660	35.862810	756.700700	1.856993
-72.010770	35.915150	757.503800	.897369
-70.784100	35.955040	757.933800	.287177
-69.555380	35.982390	758.105800	.035357
-68.325390	35.997170	758.155600	.069883
-67.094890	35.999320	758.152600	.067781
-65.864640	35.988860	758.131700	.039082
-64.635410	35.965800	758.117000	.016391
-63.407950	35.930180	758.111300	.005946
-62.183030	35.882080	758.109900	.002704
-60.961370	35.821590	758.109700	.002083
-59.743720	35.748840	758.110000	.002235
-58.530780	35.663950	758.109700	.001903
-57.323260	35.567100	758.109100	.001064
-56.121820	35.458470	758.108300	.000773
-54.927120	35.338260	758.108500	.000679
-53.739770	35.206700	758.108600	.000380
-52.560380	35.064020	758.108800	.000303
-51.389480	34.910500	758.109000	.000430
-50.227610	34.746390	758.109000	.000488
-49.075240	34.571990	758.109000	.000427
-47.932830	34.387580	758.109000	.000427
-46.800740	34.193490	758.109000	.000427
-45.679330	33.990000	758.109000	.000488
-44.568900	33.777440	758.109000	.000427
-89.960660	34.843320	758.171900	.120887
-88.832330	35.057210	758.243000	.225513
-87.692770	35.261540	758.487500	.570683
-86.542320	35.455950	759.002700	1.255246
-85.381340	35.640140	759.876500	2.337626
-84.210270	35.813810	760.980500	3.632194
-83.029580	35.976620	761.963600	4.711538
-81.839790	36.128310	762.337900	5.168817
-80.641480	36.268600	762.146500	5.083437
-79.435230	36.397220	761.826500	4.662830
-78.221690	36.513940	760.793200	3.190961
-77.001530	36.618520	757.625600	1.440560

-75.775430	36.710780	751.812700	6.871489
-74.544130	36.790520	748.981800	9.425593
-73.308360	36.857590	751.749800	7.174370
-72.068890	36.911860	755.247300	3.536464
-70.826490	36.953220	757.250900	1.165020
-69.581950	36.981590	758.124900	.144982
-68.336070	36.996900	758.394700	.368208
-67.089650	36.999130	758.372900	.346780
-65.843500	36.988290	758.252300	.202049
-64.598400	36.964370	758.165000	.090350
-63.355190	36.927440	758.129900	.038656
-62.114630	36.877570	758.119500	.020569
-60.877510	36.814860	758.118400	.016262
-59.644590	36.739430	758.118900	.015849
-58.416640	36.651430	758.117700	.013939
-57.194370	36.551040	758.113200	.008468
-55.978490	36.438440	758.108300	.003803
-54.769680	36.313860	758.107800	.002022
-53.568590	36.177530	758.108300	.001075
-52.375820	36.029710	758.108600	.000425
-51.191970	35.870660	758.108800	.000310
-50.017550	35.700660	758.109000	.000496
-48.853080	35.520030	758.109000	.000488
-47.698980	35.329070	758.109000	.000427
-46.555660	35.128100	758.109000	.000427
-45.423470	34.917440	758.109000	.000488
-44.302690	34.697410	758.109000	.000427
-90.232250	35.766320	758.461700	.544725
-89.092860	35.987640	758.830800	1.002537
-87.941790	36.199090	759.994000	2.378970
-86.779330	36.400320	762.347800	5.007626
-85.605870	36.591000	766.263300	9.084850
-84.421870	36.770790	771.077600	13.748800
-83.227800	36.939400	775.544000	17.552280
-82.024180	37.096500	777.126200	19.033630
-80.811620	37.241800	776.125200	18.894090
-79.590740	37.375040	775.159800	17.883730
-78.362190	37.495960	771.008100	13.091490
-77.126670	37.604320	758.912400	3.977493
-75.884930	37.699920	734.436500	21.421770
-74.637700	37.782550	721.454300	25.774930
-73.385770	37.852060	736.103800	20.029900
-72.129940	37.908310	748.234700	10.223140
-70.871030	37.951180	755.267900	3.140718
-69.609860	37.980580	758.635100	.779578
-68.347290	37.996460	759.651500	1.742811
-67.084140	37.998780	759.457800	1.542301
-65.821270	37.987530	758.811600	.858286
-64.559530	37.962740	758.403100	.397335
-63.299760	37.924460	758.239700	.197450
-62.042780	37.872770	758.189200	.122421
-60.789440	37.807780	758.176400	.096721
-59.540510	37.729610	758.176800	.090631
-58.296810	37.638420	758.168000	.078469
-57.059080	37.534390	758.141800	.050063
-55.828080	37.417740	758.112400	.021350
-54.604500	37.288680	758.109300	.008327
-53.389020	37.147460	758.108200	.002857
-52.182290	36.994370	758.108500	.001099
-50.984880	36.829660	758.108800	.000375
-49.797380	36.653650	758.108900	.000412
-48.620270	36.466650	758.109000	.000491
-47.454020	36.268990	758.109000	.000488
-46.299010	36.060990	758.109000	.000427
-45.155610	35.843010	758.109000	.000488
-44.024070	35.615370	758.109000	.000427
-90.516530	36.687310	759.516800	1.825799

-89.365660	36.916220	760.973000	3.365628
-88.202590	37.134950	765.416000	7.725573
-87.027650	37.343150	774.310100	15.788890
-85.841200	37.540460	789.593200	28.408200
-84.643710	37.726540	808.170300	41.659640
-83.435650	37.901050	825.802400	52.038950
-82.217600	38.063690	830.654700	54.790540
-80.990140	38.214140	825.149500	53.607720
-79.753940	38.352110	823.926100	53.914810
-78.509670	38.477340	815.471700	43.238670
-77.258070	38.589580	775.029600	14.818000
-75.999910	38.688600	674.794900	40.176550
-74.735980	38.774210	627.098000	58.449380
-73.467090	38.846230	695.322000	35.044740
-72.194090	38.904510	733.784800	18.241910
-70.917830	38.948930	752.316800	4.906314
-69.639210	38.979390	763.297400	6.065040
-68.359090	38.995840	766.089200	8.040417
-67.078360	38.998240	764.521900	6.512456
-65.797930	38.986590	761.072400	3.205328
-64.518690	38.960900	759.320800	1.439167
-63.241520	38.921240	758.752800	.819761
-61.967320	38.867690	758.557200	.580836
-60.696920	38.800350	758.496300	.478791
-59.431210	38.719360	758.484000	.439272
-58.170970	38.624900	758.435800	.377232
-56.917050	38.517150	758.284400	.231080
-55.670200	38.396340	758.119700	.108799
-54.431160	38.262690	758.123200	.040040
-53.200640	38.116470	758.113000	.010334
-51.979310	37.957960	758.109200	.002499
-50.767770	37.787460	758.108800	.000780
-49.566610	37.605300	758.108900	.000401
-48.376340	37.411790	758.108900	.000384
-47.197410	37.207270	758.109000	.000488
-46.030250	36.992090	758.109000	.000488
-44.875180	36.766620	758.109000	.000488
-43.732470	36.531200	758.109000	.000427
-90.814130	37.606180	762.215700	4.584784
-89.651300	37.842850	767.049400	8.720012
-88.475770	38.069030	782.034100	20.189060
-87.287830	38.284350	811.246200	39.508780
-86.087840	38.488450	864.454300	68.434670
-84.876290	38.680970	928.967900	99.770970
-83.653650	38.861550	990.137900	112.492700
-82.420500	39.029860	1001.096000	111.976500
-81.177470	39.185590	974.044300	116.408300
-79.925230	39.328410	981.484700	118.544200
-78.664510	39.458070	983.339800	112.868500
-77.396060	39.574290	898.657300	61.352590
-76.120680	39.676830	612.807400	49.226260
-74.839220	39.765490	424.887500	73.771190
-73.552520	39.840080	611.587600	40.181060
-72.261480	39.900440	718.333900	20.144890
-70.967010	39.946460	754.884300	14.958440
-69.670030	39.978020	786.974500	25.511940
-68.371470	39.995060	793.849500	29.594700
-67.072270	39.997550	784.786400	22.418890
-65.773400	39.985470	768.915400	10.045420
-64.475770	39.958870	762.411700	4.591343
-63.180340	39.917780	760.774300	2.962468
-61.888020	39.862300	760.227100	2.380959
-60.599740	39.792560	760.038900	2.119660
-59.316390	39.708680	760.023900	2.020454
-58.038840	39.610860	759.790000	1.722900
-56.767930	39.499290	758.994400	1.005162
-55.504470	39.374200	758.200700	.410887

-54.249260	39.235840	758.158000	.130134
-53.003000	39.084480	758.131600	.043225
-51.766410	38.920440	758.111000	.006511
-50.540120	38.744010	758.108900	.001617
-49.324710	38.555530	758.108800	.000445
-48.120720	38.355340	758.108900	.000372
-46.928620	38.143810	758.109000	.000499
-45.748810	37.921300	758.109000	.000488
-44.581620	37.688170	758.109000	.000488
-43.427300	37.444810	758.109000	.000427
-91.125630	38.522790	767.374100	8.695329
-89.950410	38.767390	781.501200	18.365530
-88.761920	39.001200	824.669000	38.858400
-87.560450	39.223820	899.838000	66.188890
-86.346370	39.434880	1034.688000	99.988370
-85.120160	39.633990	1218.660000	138.806300
-83.882300	39.820790	1364.893000	161.572100
-82.633390	39.994930	1393.963000	147.587100
-81.374080	40.156070	1351.310000	144.086700
-80.105060	40.303890	1357.206000	179.130600
-78.827100	40.438090	1419.157000	183.292100
-77.540980	40.558400	1295.144000	141.565300
-76.247540	40.664560	812.601600	65.014140
-74.947690	40.756360	324.758000	152.238100
-73.642300	40.833600	596.351200	87.785030
-72.332310	40.896110	746.640300	86.299750
-71.018690	40.943760	800.758600	57.210770
-69.702430	40.976440	847.985500	60.746610
-68.384490	40.994090	862.388100	64.713570
-67.065880	40.996670	828.164600	39.824410
-65.747600	40.984170	782.756000	20.473630
-64.430650	40.956610	771.113900	12.117240
-63.116020	40.914060	768.883500	10.570130
-61.804690	40.856610	767.248800	8.980947
-60.497620	40.784390	766.287900	7.616371
-59.195770	40.697540	765.876600	6.302316
-57.900040	40.596260	764.986600	5.365860
-56.611330	40.480750	762.185300	3.555689
-55.330490	40.351270	759.126800	.863828
-54.058330	40.208070	758.190200	.304379
-52.795620	40.051450	758.131300	.067694
-51.543080	39.881710	758.104200	.020651
-50.301390	39.699200	758.105500	.006511
-49.071130	39.504250	758.108200	.001509
-47.852850	39.297230	758.108800	.000465
-46.647030	39.078520	758.108900	.000394
-45.454070	38.848490	758.109000	.000491
-44.274280	38.607530	758.109000	.000488
-43.107920	38.356030	758.109000	.000427
-91.451740	39.437030	773.834200	12.602440
-90.263630	39.689750	803.606900	26.853110
-89.061680	39.931370	874.028900	47.130340
-87.846150	40.161480	949.748000	50.677060
-86.617400	40.379670	1083.625000	58.551360
-85.375900	40.585540	1392.209000	109.256200
-84.122160	40.778730	1708.462000	131.519300
-82.856800	40.958850	1783.849000	115.101900
-81.580460	41.125550	1736.862000	112.985600
-80.293880	41.278500	1836.659000	153.043800
-78.997860	41.417370	1952.467000	229.824200
-77.693230	41.541890	1877.170000	193.281300
-76.380870	41.651780	1239.687000	110.916400
-75.061690	41.746810	619.218500	69.215650
-73.736660	41.826770	744.440900	102.517300
-72.406780	41.891490	888.059400	86.498320
-71.073040	41.940830	939.215600	84.553830
-69.736500	41.974670	928.707300	99.339260

-68.398190 41.992950 915.669900 69.973130
-67.059160 41.995620 812.849500 26.290810
-65.720480 41.982670 774.381600 14.056870
-64.383220 41.954140 790.853800 23.971300
-63.048400 41.910080 799.760600 30.430190
-61.717090 41.850600 791.202900 25.593590
-60.390290 41.775820 779.375800 15.133310
-59.069010 41.685920 772.719100 11.250580
-57.754210 41.581080 770.338600 8.174275
-56.446830 41.461530 765.446700 3.695678
-55.147770 41.327530 758.683200 1.211493
-53.857880 41.179360 757.898600 .852830
-52.577970 41.017320 757.912400 .275220
-51.308780 40.841740 758.029900 .100517
-50.051000 40.652980 758.088300 .027686
-48.805250 40.451390 758.104900 .006009
-47.572090 40.237360 758.108200 .001066
-46.352010 40.011290 758.108800 .000367
-45.145370 39.773560 758.109000 .000499
-43.952510 39.524580 758.109000 .000488
-42.773630 39.264750 758.109000 .000427
-91.793200 40.348750 772.914400 12.904590
-90.591710 40.609810 798.948500 29.262950
-89.375760 40.859430 816.938100 49.301000
-88.145600 41.097210 721.709800 44.587380
-86.901570 41.322720 649.939100 95.398250
-85.644150 41.535550 909.904000 71.898850
-84.373860 41.735290 1282.807000 77.364220
-83.091290 41.921560 1444.347000 59.321430
-81.797160 42.093980 1647.647000 83.330150
-80.492230 42.252200 1981.637000 172.602400
-79.177290 42.395880 2285.964000 253.526700
-77.853240 42.524730 2225.607000 255.441500
-76.521000 42.638450 1580.508000 102.033100
-75.181550 42.736820 816.032200 116.552000
-73.835900 42.819590 770.017500 65.496090
-72.485100 42.886590 917.236800 71.294110
-71.130210 42.937670 1003.567000 96.947300
-69.772340 42.972710 997.254900 108.694300
-68.412590 42.991630 888.672500 77.284490
-67.052090 42.994400 701.672600 70.696590
-65.691960 42.980990 720.102400 49.709310
-64.333320 42.951450 829.711300 34.792440
-62.977290 42.905830 866.857100 57.736670
-61.624970 42.844260 833.872700 42.346330
-60.277440 42.766850 780.090400 19.753350
-58.935750 42.673790 766.654000 11.783680
-57.600940 42.565280 763.200600 7.416976
-56.273990 42.441570 755.420200 2.912246
-54.955840 42.302930 751.867500 4.763278
-53.647390 42.149640 753.923800 2.447499
-52.349470 41.982030 756.964500 1.075546
-51.062890 41.800450 757.856200 .260822
-49.788320 41.605260 758.055400 .065243
-48.526440 41.396860 758.098900 .014517
-47.277780 41.175640 758.107200 .002688
-46.042850 40.942000 758.108500 .000461
-44.822010 40.696380 758.108800 .000348
-43.615560 40.439180 758.109000 .000488
-42.423690 40.170830 758.109000 .000427
-92.150770 41.257820 766.336800 9.381363
-90.935390 41.527410 783.543000 30.078020
-89.704910 41.785250 789.025000 59.764670
-88.459540 42.030900 644.053800 58.016820
-87.199620 42.263930 430.033500 53.163620
-85.925610 42.483890 498.011200 53.887740
-84.638040 42.690380 748.974500 55.106250

-83.337520	42.882970	941.142000	124.660300
-82.024790	43.061280	1426.164000	115.467100
-80.700610	43.224930	2278.213000	236.775400
-79.365870	43.373580	2730.286000	382.019700
-78.021450	43.506890	2740.129000	337.098400
-76.668370	43.624570	2158.192000	196.703500
-75.307630	43.726360	1393.932000	61.945140
-73.940300	43.812040	1034.950000	45.242640
-72.567500	43.881390	815.807700	71.974000
-71.190370	43.934270	1001.880000	98.041180
-69.810050	43.970550	1095.328000	156.521600
-68.427750	43.990140	1080.194000	102.106000
-67.044650	43.993000	795.697500	84.160130
-65.661930	43.979120	762.706700	40.092460
-64.280810	43.948540	882.115200	54.678260
-62.902460	43.901310	932.165000	72.574030
-61.528050	43.837570	860.358500	52.510060
-60.158730	43.757450	769.709500	32.624180
-58.795620	43.661140	760.500500	18.806160
-57.439790	43.548850	770.508300	11.738870
-56.092310	43.420850	754.221400	7.895751
-54.754150	43.277410	747.708800	6.442475
-53.426270	43.118850	753.617800	3.536844
-52.109530	42.945500	756.927400	1.057437
-50.804760	42.757750	757.811800	.315746
-49.512690	42.555960	758.034700	.088995
-48.233980	42.340550	758.092900	.022663
-46.969190	42.111940	758.106000	.004929
-45.718810	41.870550	758.108300	.000883
-44.483210	41.616830	758.108800	.000335
-43.262650	41.351200	758.108900	.000378
-42.057300	41.074100	758.109000	.000427
-92.525320	42.164070	755.893700	3.438094
-91.295520	42.442420	757.077100	11.414600
-90.049940	42.708690	738.547100	22.991940
-88.788760	42.962440	611.278300	38.687970
-87.512310	43.203190	492.580200	65.136340
-86.221000	43.430500	491.618100	59.124860
-84.915410	43.643920	675.561000	61.730330
-83.596150	43.843030	986.843200	124.600900
-82.263960	44.027400	1739.657000	189.557200
-80.919660	44.196660	2714.109000	373.594900
-79.564150	44.350410	3232.717000	416.321300
-78.198390	44.488330	3040.859000	382.833500
-76.823410	44.610100	2344.302000	214.850300
-75.440290	44.715440	1574.673000	50.843560
-74.050180	44.804110	896.305500	42.909290
-72.654240	44.875890	489.274900	119.788600
-71.253690	44.930630	724.046500	126.886700
-69.849760	44.968180	1263.285000	160.819200
-68.443720	44.988460	1461.049000	146.207600
-67.036810	44.991420	1075.074000	75.641490
-65.630330	44.977050	877.182400	43.773660
-64.225530	44.945390	894.999200	53.291450
-62.823690	44.896510	943.155600	62.581100
-61.426040	44.830540	883.233900	47.392110
-60.033810	44.747610	787.865500	43.534610
-58.648170	44.647940	791.657300	28.298480
-57.270290	44.531750	786.407900	22.546110
-55.901260	44.399320	756.818700	10.109090
-54.542130	44.250930	745.112700	6.159492
-53.193900	44.086940	752.973000	3.796351
-51.857480	43.907680	757.077100	1.198137
-50.533710	43.713560	757.932600	.391331
-49.223370	43.504980	758.061400	.106768
-47.927120	43.282370	758.092100	.024937
-46.645540	43.046150	758.104100	.006303

-45.379100 42.796800 758.107700 .001597
-44.128160 42.534760 758.108600 .000426
-42.892960 42.260480 758.108800 .000340
-41.673610 41.974410 758.109000 .000427
-92.917770 43.067330 745.611000 12.231160
-91.673000 43.354680 720.988800 25.851910
-90.411740 43.629630 625.438300 53.150650
-89.134110 43.891690 465.392600 74.223690
-87.840450 44.140390 419.485000 72.580500
-86.531140 44.375260 422.856600 60.891000
-85.206750 44.595830 779.721200 76.908490
-83.867900 44.801650 1363.444000 125.663300
-82.515380 44.992280 1974.923000 282.732900
-81.150000 45.167310 2642.856000 359.187900
-79.772730 45.326340 2827.307000 404.728900
-78.384560 45.469020 2566.599000 309.866500
-76.986600 45.595010 1961.527000 152.651700
-75.579970 45.704020 1414.746000 55.861260
-74.165890 45.795780 963.263300 112.278100
-72.745610 45.870080 454.724800 162.012500
-71.320400 45.926740 683.089400 179.443800
-69.891600 45.965610 1342.357000 189.239500
-68.460530 45.986610 1597.190000 187.509700
-67.028560 45.989670 1254.742000 101.270800
-65.597030 45.974800 934.026900 53.874000
-64.167300 45.942020 902.030400 42.830500
-62.740720 45.891430 961.461800 44.658570
-61.318600 45.823140 913.058000 47.921610
-59.902260 45.737310 842.158300 46.974320
-58.492950 45.634170 820.348400 39.847450
-57.091900 45.513950 779.003400 24.040610
-55.700260 45.376930 740.765100 12.646880
-54.319140 45.223450 744.779100 12.719170
-52.949590 45.053840 755.567000 8.673629
-51.592580 44.868490 759.295500 3.641064
-50.248970 44.667810 758.834400 1.478885
-48.919560 44.452230 758.314700 .455256
-47.605040 44.222190 758.137500 .099984
-46.305980 43.978160 758.107400 .015651
-45.022840 43.720610 758.107200 .002355
-43.755960 43.450020 758.108300 .000634
-42.505550 43.166850 758.108800 .000357
-41.271660 42.871580 758.109000 .000432
-93.329110 43.967430 743.789400 13.953070
-92.068780 44.264030 713.566200 31.762890
-90.791210 44.547890 594.337600 62.397590
-89.496510 44.818510 436.360100 77.402850
-88.184940 45.075390 377.997000 55.214910
-86.856880 45.318050 314.699200 124.298600
-85.512890 45.545990 793.670500 65.776630
-84.153590 45.758730 1490.255000 138.510200
-82.779790 45.955820 1854.254000 221.051500
-81.392350 46.136820 2001.368000 284.376100
-79.992260 46.301310 1998.445000 260.710000
-78.580580 46.448910 1810.276000 186.791600
-77.158460 46.579270 1520.930000 111.436000
-75.727110 46.692070 1477.970000 49.510910
-74.287820 46.787040 1531.377000 85.647880
-72.841890 46.863950 1358.921000 105.964300
-71.390720 46.922590 1398.401000 143.583300
-69.935700 46.962830 1646.454000 198.586900
-68.478260 46.984570 1754.940000 177.867500
-67.019850 46.987740 1381.553000 108.797800
-65.561920 46.972340 1087.853000 44.824990
-64.105920 46.938410 1021.330000 46.308110
-62.653260 46.886040 1039.371000 70.217020
-61.205390 46.815350 997.833500 75.644130

-59.763680	46.726520	944.894300	70.902100
-58.329470	46.619790	893.361900	66.565020
-56.904060	46.495390	817.952400	53.118230
-55.488670	46.353640	795.471200	38.719620
-54.084500	46.194880	811.547600	35.888080
-52.692630	46.019470	796.385900	28.434310
-51.314070	45.827840	772.197600	14.592910
-49.949730	45.620390	762.117500	4.810228
-48.600430	45.397590	759.083500	1.401621
-47.266860	45.159900	758.296300	.333436
-45.949600	44.907820	758.131200	.060192
-44.649100	44.641840	758.108900	.007982
-43.365670	44.362450	758.108200	.000969
-42.099450	44.070140	758.108600	.000356
-40.850440	43.765410	758.108900	.000328
-93.760410	44.864160	750.386000	8.307707
-92.483920	45.170280	736.538600	17.999370
-91.189420	45.463320	682.635800	42.788400
-89.876960	45.742750	551.608300	50.735590
-88.546760	46.008070	512.161100	43.253100
-87.199170	46.258760	493.503200	34.486950
-85.834720	46.494300	864.835800	45.571210
-84.454070	46.714200	1307.969000	90.988290
-83.058010	46.917960	1483.157000	143.634900
-81.647480	47.105130	1510.819000	156.280700
-80.223460	47.275260	1457.004000	141.348200
-78.787090	47.427960	1341.853000	100.747600
-77.339580	47.562840	1265.421000	105.153400
-75.882230	47.679570	1420.902000	100.143200
-74.416370	47.777870	1716.213000	124.243600
-72.943440	47.857470	1826.017000	142.988600
-71.464880	47.918190	1779.610000	183.046900
-69.982220	47.959850	1868.011000	189.085200
-68.496960	47.982350	1745.603000	182.525600
-67.010670	47.985630	1416.721000	75.734610
-65.524890	47.969690	1160.876000	53.701830
-64.041170	47.934570	1172.462000	33.206060
-62.561040	47.880340	1260.430000	80.378650
-61.086020	47.807170	1254.691000	116.117600
-59.617590	47.715230	1204.057000	115.851400
-58.157170	47.604770	1108.344000	114.770900
-56.706150	47.476050	1003.656000	108.097800
-55.265830	47.329400	998.896500	94.286890
-53.837470	47.165180	991.434200	83.133630
-52.422200	46.983780	891.482400	65.095240
-51.021090	46.785640	804.595200	31.720340
-49.635090	46.571200	768.960100	10.882300
-48.265020	46.340950	760.366800	2.815401
-46.911610	46.095370	758.546600	.648309
-45.575410	45.834990	758.175800	.125587
-44.256860	45.560320	758.115100	.018748
-42.956210	45.271870	758.108800	.002087
-41.673580	44.970160	758.108600	.000319
-40.408870	44.655690	758.108900	.000331
-94.212860	45.757310	757.596600	1.260004
-92.919560	46.073230	754.053000	4.182627
-91.607470	46.375730	735.176300	14.456240
-90.276550	46.664250	684.712600	32.217330
-88.926960	46.938270	635.124000	30.045360
-87.559010	47.197250	675.419600	32.089370
-86.173240	47.440650	831.926100	25.519640
-84.770290	47.667940	1052.332000	54.126870
-83.350960	47.878600	1251.439000	84.116890
-81.916200	48.072160	1393.346000	109.550100
-80.467090	48.248140	1459.683000	104.697000
-79.004780	48.406110	1410.904000	85.198110
-77.530590	48.545680	1391.581000	72.967800

-76.045850	48.666500	1511.479000	107.445200
-74.552020	48.768250	1662.480000	172.318800
-73.050600	48.850660	1697.205000	216.787500
-71.543170	48.913520	1715.185000	221.821400
-70.031330	48.956650	1722.887000	229.468100
-68.516710	48.979950	1596.160000	177.978500
-67.000980	48.983350	1250.622000	84.965580
-65.485790	48.966840	926.307600	111.075700
-63.972820	48.930470	1028.384000	67.309300
-62.463700	48.874330	1304.211000	68.154140
-60.960050	48.798580	1457.653000	119.061100
-59.463460	48.703410	1469.214000	132.646600
-57.975440	48.589080	1357.315000	137.000600
-56.497490	48.455880	1213.989000	152.339700
-55.030970	48.304140	1178.400000	136.876600
-53.577210	48.134270	1116.071000	118.764200
-52.137430	47.946660	961.398300	80.112140
-50.712720	47.741790	827.542200	41.933090
-49.304080	47.520120	774.858900	14.215660
-47.912350	47.282170	761.524000	3.731056
-46.538250	47.028450	758.746600	.846631
-45.182340	46.759510	758.210400	.159529
-43.845020	46.475880	758.120900	.024469
-42.526500	46.178100	758.109700	.002926
-41.226800	45.866700	758.108600	.000303
-39.945740	45.542200	758.109000	.000431
-94.687740	46.646660	766.880300	9.909515
-93.376940	46.972690	771.481100	14.236140
-92.046550	47.284930	777.796000	19.454450
-90.696440	47.582840	780.331700	21.166010
-89.326680	47.865840	791.388900	19.275010
-87.937550	48.133390	829.602900	22.771630
-86.529520	48.384910	897.488900	34.816140
-85.103260	48.619850	1019.051000	51.290860
-83.659580	48.837660	1247.241000	71.468100
-82.199460	49.037830	1487.468000	118.962900
-80.724000	49.219880	1542.955000	146.302900
-79.234450	49.383340	1472.032000	135.788000
-77.732160	49.527770	1447.305000	133.226700
-76.218590	49.652830	1483.902000	170.657800
-74.695260	49.758160	1540.040000	205.069800
-73.163800	49.843490	1529.219000	226.684300
-71.625870	49.908580	1528.475000	232.222100
-70.083210	49.953250	1563.832000	224.198000
-68.537570	49.977380	1513.757000	173.852400
-66.990740	49.980900	1203.903000	88.310440
-65.444490	49.963800	857.458200	84.607930
-63.900620	49.926140	802.844200	65.223330
-62.360880	49.868010	1010.212000	65.133060
-60.827010	49.789570	1239.830000	118.322200
-59.300730	49.691040	1356.150000	139.383200
-57.783640	49.572690	1313.363000	149.932700
-56.277320	49.434830	1188.395000	152.039900
-54.783270	49.277820	1082.796000	141.126200
-53.302860	49.102080	992.804700	104.122300
-51.837360	48.908040	864.692100	69.748940
-50.387950	48.696200	791.435900	30.226310
-48.955640	48.467040	767.210600	9.618979
-47.541310	48.221130	760.493300	2.657648
-46.145660	47.959000	758.641200	.643873
-44.769230	47.681210	758.197000	.126989
-43.412380	47.388330	758.119800	.018670
-42.075270	47.080920	758.109900	.002209
-40.757810	46.759540	758.109000	.000452
-39.459720	46.424710	758.109000	.000425
-95.186440	47.531940	784.400000	26.357450
-93.857410	47.868410	800.394200	37.302310

-92.508000 48.190720 827.282400 55.649300
-91.137910 48.498310 869.122500 70.261790
-89.747150 48.790600 935.058900 79.820600
-88.335950 49.067010 1003.703000 80.067310
-86.904710 49.326930 1038.424000 69.641790
-85.454100 49.569790 1088.320000 68.411150
-83.984930 49.795020 1218.888000 110.493300
-82.498220 50.002060 1322.283000 161.219500
-80.995120 50.190400 1343.154000 180.588500
-79.476920 50.359550 1320.290000 175.477600
-77.945060 50.509060 1357.695000 168.416100
-76.401090 50.638520 1417.786000 184.568100
-74.846650 50.747580 1467.928000 200.724100
-73.283450 50.835950 1434.150000 209.090700
-71.713320 50.903350 1405.270000 200.750700
-70.138080 50.949630 1400.618000 197.752500
-68.559640 50.974620 1393.063000 176.190600
-66.979900 50.978270 1273.183000 112.141700
-65.400800 50.960560 1011.009000 73.107550
-63.824260 50.921550 852.989900 58.339140
-62.252170 50.861340 855.927700 87.449820
-60.686400 50.780110 975.123400 121.470300
-59.128770 50.678080 1101.545000 145.401900
-57.581030 50.555550 1142.359000 141.188500
-56.044860 50.412850 1049.678000 137.815400
-54.521820 50.250360 954.681400 101.702400
-53.013420 50.068520 859.129800 70.470410
-51.520960 49.867810 795.831400 36.101120
-50.045680 49.648740 768.767600 14.412240
-48.588630 49.411830 761.261200 4.656420
-47.150700 49.157670 759.065000 1.350803
-45.732590 48.886830 758.314600 .322787
-44.334810 48.599900 758.140900 .056855
-42.957660 48.297460 758.112800 .007970
-41.601190 47.980120 758.109400 .001029
-40.265250 47.648430 758.109000 .000446
-38.949390 47.302930 758.109000 .000425
-95.710510 48.412890 823.605100 53.188990
-94.362470 48.760130 869.795400 73.026900
-92.993230 49.092850 937.166600 89.663000
-91.602360 49.410460 994.919000 98.888310
-90.189750 49.712350 1039.626000 103.406800
-88.755540 49.997940 1063.167000 103.143500
-87.300090 50.266570 1048.851000 97.420620
-85.824040 50.517650 1025.614000 103.060200
-84.328210 50.750570 1047.609000 133.719600
-82.813610 50.964740 1107.580000 164.351900
-81.281460 51.159630 1178.177000 173.373900
-79.733130 51.334700 1218.936000 168.356400
-78.170130 51.489480 1266.837000 165.106700
-76.594090 51.623530 1303.265000 182.317200
-75.006800 51.736490 1335.818000 194.128600
-73.410070 51.828010 1288.079000 197.550500
-71.805870 51.897850 1263.458000 182.680600
-70.196170 51.945790 1272.161000 159.207400
-68.582990 51.971680 1256.605000 144.742600
-66.968440 51.975460 1208.564000 122.122000
-65.354560 51.957120 1114.448000 104.786200
-63.743440 51.916690 978.757000 96.946100
-62.137130 51.854320 910.503900 90.662480
-60.537630 51.770170 932.066700 111.034400
-58.946900 51.664510 1000.082000 123.120000
-57.366820 51.537620 1007.794000 126.008400
-55.799180 51.389870 956.708200 100.129900
-54.245650 51.221680 875.593200 73.591740
-52.707820 51.033510 819.251800 41.040440
-51.187080 50.825860 786.330700 20.248650

-49.684720 50.599280 768.839100 8.543102
-48.201800 50.354330 760.936400 3.004209
-46.739240 50.091630 758.680300 .728610
-45.297720 49.811770 758.207600 .143155
-43.877700 49.515370 758.123600 .023324
-42.479410 49.203060 758.111000 .003355
-41.102810 48.875440 758.109100 .000570
-39.747590 48.533100 758.109000 .000465
-38.413150 48.176570 758.109000 .000427
-96.261670 49.289190 890.419300 79.523370
-94.893750 49.647600 983.044900 88.267680
-93.503820 49.991080 1044.825000 102.629300
-92.091320 50.319060 1053.624000 102.543400
-90.655950 50.630910 1022.332000 99.329550
-89.197760 50.925990 970.772400 95.978200
-87.717060 51.203660 928.084800 87.799630
-86.214420 51.463270 906.097400 87.776730
-84.690660 51.704190 937.243200 108.351200
-83.146820 51.925780 1064.890000 118.121600
-81.584160 52.127480 1164.585000 135.543800
-80.004110 52.308710 1190.092000 140.273700
-78.408280 52.468990 1153.630000 152.984500
-76.798410 52.607840 1160.590000 162.635400
-75.176390 52.724850 1206.157000 171.376600
-73.544200 52.819690 1177.286000 172.946900
-71.903920 52.892050 1172.089000 156.789200
-70.257710 52.941730 1183.342000 132.855900
-68.607750 52.968570 1168.899000 103.053500
-66.956280 52.972490 1126.938000 85.471880
-65.305560 52.953480 1079.254000 115.614100
-63.657820 52.911580 1042.001000 118.178800
-62.015270 52.846950 979.492000 117.094900
-60.380080 52.759750 966.485400 108.261600
-58.754360 52.650280 961.467800 107.021500
-57.140120 52.518850 932.293600 89.350860
-55.539290 52.365840 868.387300 70.535190
-53.953660 52.191710 822.007200 44.794940
-52.384890 51.996940 792.752800 25.695880
-50.834480 51.782070 774.658600 12.843150
-49.303730 51.547700 763.625200 5.346342
-47.793770 51.294400 759.465400 1.481273
-46.305490 51.022820 758.395100 .348205
-44.839570 50.733620 758.161900 .071300
-43.396390 50.427420 758.117900 .012761
-41.976090 50.104880 758.110200 .002125
-40.578500 49.766630 758.109000 .000438
-39.203150 49.413260 758.109000 .000483
-37.849210 49.045330 758.109000 .000427
-96.841810 50.160530 907.185800 72.118320
-95.453030 50.530490 969.494700 87.547210
-94.041510 50.885130 993.848500 94.015170
-92.606450 51.223850 953.367500 94.633670
-91.147380 51.546020 900.112700 83.750540
-89.664200 51.850960 857.974300 68.941320
-88.157130 52.138020 834.428800 58.499690
-86.626690 52.406500 836.028400 59.278540
-85.073680 52.655720 895.742700 65.050470
-83.499180 52.885050 993.033400 84.249520
-81.904440 53.093850 1065.721000 92.360150
-80.290990 53.281520 1047.189000 107.492400
-78.660530 53.447530 994.869500 120.639700
-77.014920 53.591380 1031.620000 130.041600
-75.356160 53.712650 1109.562000 142.987400
-73.686420 53.810940 1093.933000 152.718100
-72.007930 53.885960 1091.863000 139.521000
-70.322990 53.937460 1100.969000 108.380400
-68.634010 53.965280 1074.268000 65.748500

-66.943400	53.969350	1039.980000	55.721320
-65.253590	53.949630	1030.433000	89.634280
-63.567000	53.906200	1033.400000	120.699500
-61.886040	53.839190	1011.462000	114.484000
-60.213040	53.748820	955.938700	101.172200
-58.550290	53.635360	905.849300	79.124940
-56.899960	53.499180	848.058600	60.305580
-55.264110	53.340690	805.073700	39.374830
-53.644650	53.160350	787.327000	24.420410
-52.043340	52.958710	774.624800	14.217520
-50.461760	52.736320	764.920400	6.688725
-48.901280	52.493820	760.267400	2.305246
-47.363030	52.231850	758.688700	.682590
-45.847900	51.951060	758.243300	.174476
-44.356530	51.652150	758.135600	.037602
-42.889220	51.335790	758.113100	.006875
-41.445980	51.002650	758.109400	.001089
-40.026490	50.653380	758.109000	.000460
-38.630060	50.288590	758.109000	.000488
-37.255620	49.908830	758.109000	.000427
-97.453060	51.026530	832.234100	52.992940
-96.042340	51.408490	866.929300	56.280670
-94.608220	51.774700	862.104600	65.863140
-93.149600	52.124570	845.102400	62.105820
-91.665800	52.457450	829.815500	54.293090
-90.156560	52.772640	815.895800	46.932690
-88.621980	53.069450	805.804300	40.835430
-87.062480	53.347160	802.193800	38.543870
-85.478850	53.605050	811.732400	45.401530
-83.872130	53.842430	838.757600	44.996230
-82.243660	54.058630	828.978500	54.623740
-80.595020	54.253030	829.173800	49.893050
-78.928000	54.425040	857.620900	63.901530
-77.244600	54.574130	943.529200	94.665700
-75.546970	54.699840	1035.703000	118.754800
-73.837420	54.801750	1035.650000	126.422100
-72.118360	54.879540	1009.737000	109.121700
-70.392330	54.932960	960.883900	72.238890
-68.661900	54.961820	907.151200	49.190420
-66.929700	54.966030	924.915700	46.083640
-65.198370	54.945580	940.634700	82.838010
-63.470540	54.900540	968.182000	102.141700
-61.748820	54.831050	953.639900	95.400940
-60.035740	54.737340	890.865400	74.408580
-58.333780	54.619720	833.557100	50.470110
-56.645260	54.478570	798.511200	30.998420
-54.972420	54.314330	779.817800	19.576500
-53.317280	54.127510	774.464200	14.607010
-51.681740	53.918690	769.646300	9.689571
-50.067420	53.688470	762.694200	4.445747
-48.475770	53.437500	759.456400	1.491422
-46.907930	53.166490	758.466600	.443314
-45.364770	52.876130	758.190900	.113576
-43.846840	52.567130	758.123500	.023396
-42.354370	52.240220	758.110800	.003690
-40.887210	51.896080	758.109100	.000572
-39.444820	51.535370	758.109000	.000484
-38.026250	51.158730	758.109000	.000488
[truncated: 629,577 more chars]
